# Supplementary material for: Genomic Assessment of the Contribution of the Wolbachia Endosymbiont of Eurosta solidaginis to Gall Induction
Source: Int J Mol Sci. 2023 Jun 1;24(11):9613. doi: 10.3390/ijms24119613 (PMC10253641; doi:10.3390/ijms24119613)

Supplemental Figure S1. Methyl erythritol phosphate (MEP) pathway enzymes and intermediates. Pathway and enzymes abbreviations follow KEGG pathway map 00900.

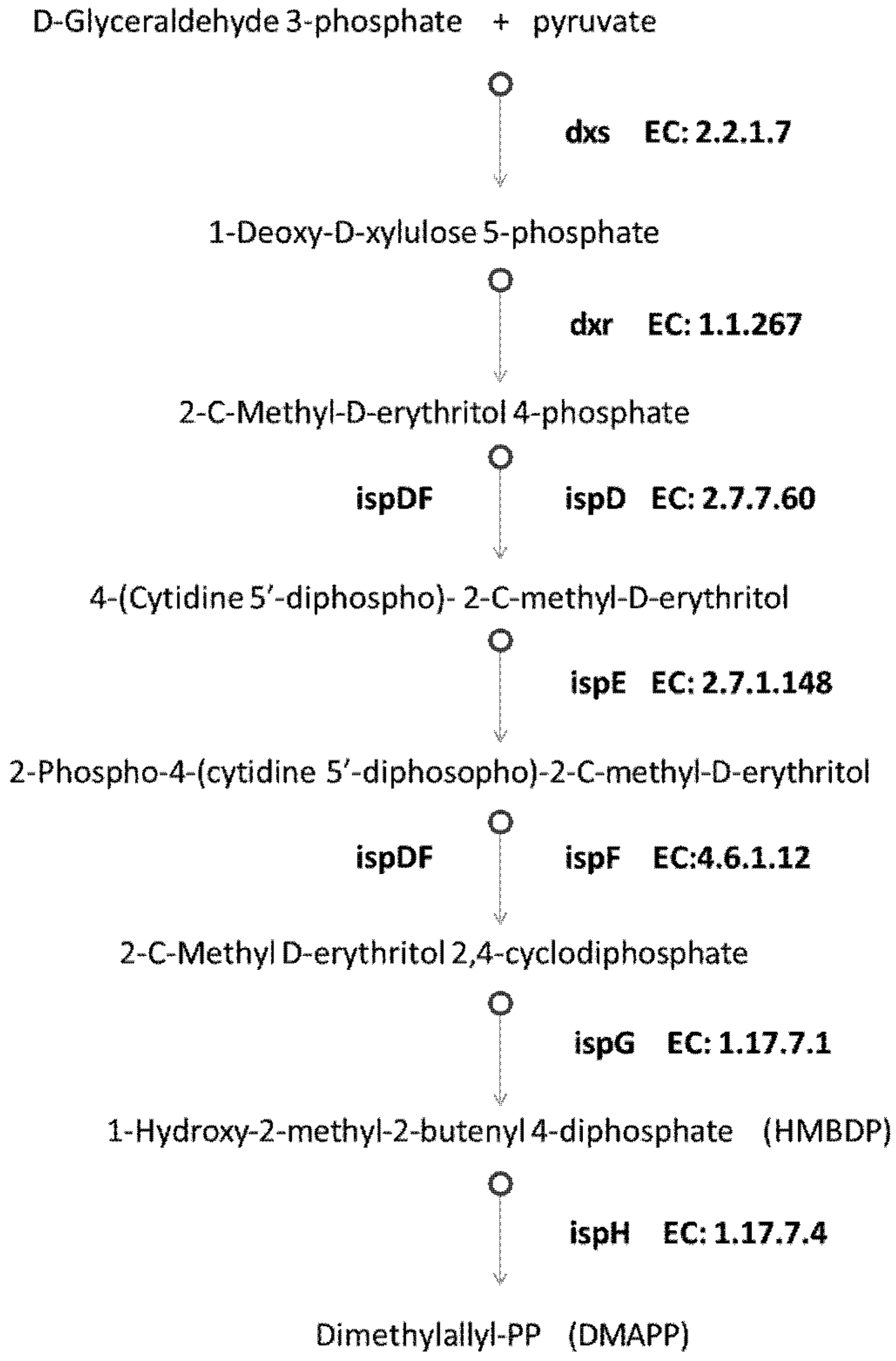

Supplemental Figure S2. S-Adenosyl-L-methionine (SAM) pathway enzymes and intermediates. KEGG EC numbers represent enzymes. Figure based on KEGG pathway may 00270. Red text indicates enzymes missing from *w*Esol genome.

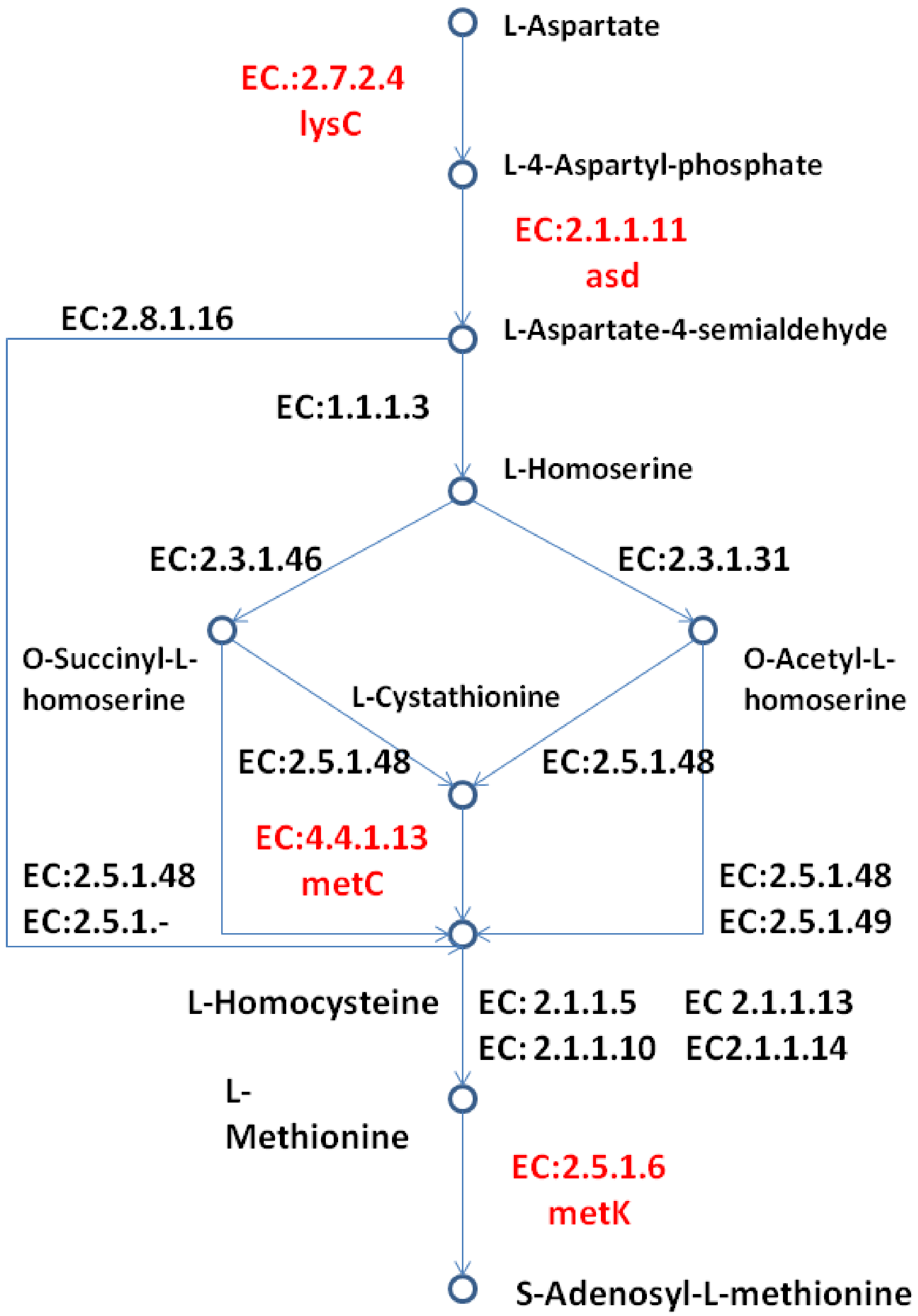

Supplement: Supplementary file 1 [file ijms-24-09613-s001.zip › Supplemental Figures S1 & S2.pdf]
