# Supplementary material for: Genomic Assessment of the Contribution of the Wolbachia Endosymbiont of Eurosta solidaginis to Gall Induction
Source: Int J Mol Sci. 2023 Jun 1;24(11):9613. doi: 10.3390/ijms24119613 (PMC10253641; doi:10.3390/ijms24119613)
Supplement: Supplementary file 1 [file ijms-24-09613-s001.zip › Supplemental File S1 Annotated genome of wEsol.pdf]

```
LOCUS       JAQZAU010000001           5193 bp    dna     linear   ENV 17-MAR-2023
DEFINITION  MAG: Wolbachia pipientis isolate wEsol contig_10007, whole genome
            shotgun sequence.
ACCESSION   JAQZAU010000001 JAQZAU010000000
VERSION     JAQZAU010000001.1
DBLINK      BioProject: PRJNA929258
            BioSample: SAMN33142973
KEYWORDS    WGS; ENV; Metagenome Assembled Genome; MAG.
SOURCE      Wolbachia pipientis (insect metagenome)
  ORGANISM  Wolbachia pipientis
            Bacteria; Pseudomonadota; Alphaproteobacteria; Rickettsiales;
            Anaplasmataceae; Wolbachieae; Wolbachia.
REFERENCE   1  (bases 1 to 5193)
  AUTHORS   Fiutek,N., Couger,B.M., Roy,S., de la Torre,J.R. and Connor,E.F.
  TITLE     Genomic assessment of the contribution of the Wolbachia
            endosymbiont of Eurosta solidaginis to gall-induction
  JOURNAL   International Journal Molecular Sciences
REFERENCE   2  (bases 1 to 5193)
  AUTHORS   Fiutek,N., Couger,B.M., Roy,S., de la Torre,J.R. and Connor,E.F.
  TITLE     Direct Submission
  JOURNAL   Submitted (10-FEB-2023) Biology, San Francisco State University,
            1600 Holloway Ave, San Francisco, CA 94132, USA
COMMENT     ##Genome-Assembly-Data-START##
            Assembly Date          :: JUN-2019
            Assembly Method        :: FLYE v. 06-2019
            Genome Representation  :: Full
            Expected Final Version :: Yes
            Genome Coverage        :: 100.0x
            Sequencing Technology  :: Illumina HiSeq; PacBio Sequel
            ##Genome-Assembly-Data-END##
FEATURES             Location/Qualifiers
     source          1..5193
                     /organism="Wolbachia pipientis"
                     /mol_type="genomic DNA"
                     /submitter_seqid="contig_10007"
                     /isolate="wEsol"
                     /isolation_source="galls on Solidago altissima"
                     /host="Eurosta solidaginis MN-2018-EFC"
                     /db_xref="taxon:955"
                     /environmental_sample
                     /country="USA: Cowling Arboretum, Carleton College,
                     Northfield, MN"
                     /lat_lon="44.48 N 93.13 W"
                     /altitude="280 m"
                     /collection_date="2018-11"
                     /metagenome_source="insect metagenome"
                     /note="metagenomic"
     gene            <1..136
                     /locus_tag="wEsol_00001"
     CDS             <1..136
                     /locus_tag="wEsol_00001"
                     /codon_start=1
                     /transl_table=11
                     /product="hypothetical protein"
                     /translation="XLKYWRFLCFKRLTSEIQLLLIATNLRCKCLRNLLSRKKDKESPS"
     gene            complement(212..391)
                     /locus_tag="wEsol_00002"
     CDS             complement(212..391)
                     /locus_tag="wEsol_00002"
                     /codon_start=1
```

```
                     /transl_table=11
                     /product="hypothetical protein"

/translation="MTNSASPLIITVKLLYLLSPICRLKGKDYLMYRRLIFNFLQYIDTVCLYKTSSTSRFLSK"
     gene            complement(430..993)
                     /locus_tag="wEsol_00003"
     CDS             complement(430..993)
                     /locus_tag="wEsol_00003"
                     /codon_start=1
                     /transl_table=11
                     /fullproduct="qcoverage 100 , hcoverage 97.3958333333333
                     , similarity 100 , identity 99.5 , evalue 9.54e-129 ,
                     alnlength 187 , Bacteria , Proteobacteria ,
                     WP_015588787.1 MULTISPECIES: RlmE family RNA
                     methyltransferase [Wolbachia]"
                     /cog="FtsJ COG0293 205 23S rRNA methylase"
                     /pfam="FtsJ Methyltransf_23 Methyltransf_31
                     Methyltransf_18 Methyltransf_11 NSP13"
                     /tigr="TIGR00438 rrmJ 188 ribosomal RNA large subunit
                     methyltransferase J"
                     /product="RlmE family RNA methyltransferase"
                     /translation="MQKTSKDGYRSRSAYKLVEMDNKFKLFQEGQKIIDLGASPGGWS
                     QVASQKGANVVALDIKPMNAINGVEFIQCDIINEFEILREKFKDQKFDVILSDMAPES
                     CGLKSLDHIRIMLLCEAALNFAKHFLSHGGTFVVKIFQGESDKDFCNELKKMFKTVKY
                     FKPKSSRSESTEMYLVSLGFIGSRLSI"
                     /besthit="qcoverage 100 , hcoverage 97.3958333333333 ,
                     similarity 100 , identity 99.5 , evalue 9.54e-129 ,
                     alnlength 187 , Bacteria , Proteobacteria ,
                     WP_015588787.1 MULTISPECIES: RlmE family RNA
                     methyltransferase [Wolbachia]"
     gene            complement(1085..1660)
                     /locus_tag="wEsol_00004"
     CDS             complement(1085..1660)
                     /locus_tag="wEsol_00004"
                     /codon_start=1
                     /transl_table=11
                     /fullproduct="qcoverage 100 , hcoverage 100 , similarity
                     100 , identity 100 , evalue 5.23e-111 , alnlength 191 ,
                     Bacteria , Proteobacteria , WP_010082598.1 MULTISPECIES:
                     DUF2497 domain-containing protein [Wolbachia]"
                     /cog="COG3827 COG3827 231 Uncharacterized protein
                     conserved in bacteria"
                     /pfam="DUF2497"
                     /product="DUF2497 domain-containing protein"
                     /translation="MHDEQGNQSVKDILEDIKKAISGKNASNDKAEIKDENDDVLYLE
                     EEYPEEIEEDGKKENNEKEDIDNDDQSEEMACNNNQFNGHSYNSEEKKDIYLYDNIQM
                     SNNKASNSGLQAQNNDHLILKENMEEIKALLGKMQNELQHKQQKKANLTVEELVTSLL
                     KPQLSEWLNKYLHALVKEVVEKELKDIINNK"
                     /besthit="qcoverage 100 , hcoverage 100 , similarity 100
                     , identity 100 , evalue 5.23e-111 , alnlength 191 ,
                     Bacteria , Proteobacteria , WP_010082598.1 MULTISPECIES:
                     DUF2497 domain-containing protein [Wolbachia]"
     gene            complement(1680..2903)
                     /locus_tag="wEsol_00005"
     CDS             complement(1680..2903)
                     /locus_tag="wEsol_00005"
                     /codon_start=1
                     /transl_table=11
                     /fullproduct="qcoverage 100 , hcoverage 100 , similarity
                     100 , identity 100 , evalue 2.14e-275 , alnlength 407 ,
```

```
                     Bacteria , Proteobacteria , WP_006279873.1 MULTISPECIES:
                     TolC family protein [Wolbachia]"
                     /cog="TolC COG1538 457 Outer membrane protein"
                     /pfam="OEP OEP"
                     /tigr="TIGR01844 type_I_sec_TolC 414 type I secretion
                     outer membrane protein, TolC family"
                     /product="TolC family protein"
                     /translation="MFRLIITLAIIFNTISCYATDVGEVISKAIKNSSKIKSQFYQYK
                     SAEKHYKSSGLAGFLPDINLQYNFDSNFNLDTSEKKLTLRQKLIDGGGTFATFSRSSH
                     LLKAEKMRFQQLKQELALNAVKAYVNVLQKTEILKLREHKERVSLEHLSAMKKRFSLG
                     EVTNAEVLLAKAKFSSSISERVDAEGKLKLVNIAYYHLIGEDADDLSEANDKLPSVPE
                     LNECLQLAKTNNLSLKAAVYQKRAAGMEVIAESSKWLPSLNLSASKNFGEDGMKVDKL
                     LENVHVVFTLDVPIFKRGVNAFGVSKAKMDAKKSTYDYYETVKNIEQAVVNAWNNVLT
                     AKAIIKASQEAEKAAALALEGVEQEVNLNLKSTSDLLDTEDALFKARSDLVEAKSNYV
                     ISVYNLLFMINSIDL"
                     /besthit="qcoverage 100 , hcoverage 100 , similarity 100
                     , identity 100 , evalue 2.14e-275 , alnlength 407 ,
                     Bacteria , Proteobacteria , WP_006279873.1 MULTISPECIES:
                     TolC family protein [Wolbachia]"
     gene            3033..3491
                     /locus_tag="wEsol_00006"
     CDS             3033..3491
                     /locus_tag="wEsol_00006"
                     /codon_start=1
                     /transl_table=11
                     /fullproduct="qcoverage 100 , hcoverage 100 , similarity
                     100 , identity 100 , evalue 2.96e-107 , alnlength 152 ,
                     Bacteria , Proteobacteria , WP_010082596.1 MULTISPECIES:
                     50S ribosomal protein L13 [Wolbachia]"
                     /cog="RplM COG0102 148 Ribosomal protein L13"
                     /pfam="Ribosomal_L13"
                     /tigr="TIGR01066 rplM_bact 141 ribosomal protein L13"
                     /product="50S ribosomal protein L13"
                     /translation="MKTFFLKEKQINKKWFVIDAEGLVVGRLAAFVATLLRGKHKPEY
                     TPHMDCGDNVIIINAEKVHFTGKKLKDKIYYKHTGYSGGLKKTTPDNILNGKFPERVI
                     EMAVKRMLDDGPMARRRFENLYVYSGSEHKHQGQQPEKIDFASLNRKNKK"
                     /besthit="qcoverage 100 , hcoverage 100 , similarity 100
                     , identity 100 , evalue 2.96e-107 , alnlength 152 ,
                     Bacteria , Proteobacteria , WP_010082596.1 MULTISPECIES:
                     50S ribosomal protein L13 [Wolbachia]"
     gene            3502..3957
                     /locus_tag="wEsol_00007"
     CDS             3502..3957
                     /locus_tag="wEsol_00007"
                     /codon_start=1
                     /transl_table=11
                     /fullproduct="qcoverage 100 , hcoverage 100 , similarity
                     100 , identity 100 , evalue 1.36e-98 , alnlength 151 ,
                     Bacteria , Proteobacteria , WP_007548415.1 MULTISPECIES:
                     30S ribosomal protein S9 [Wolbachia]"
                     /cog="RpsI COG0103 130 Ribosomal protein S9"
                     /pfam="Ribosomal_S9"
                     /tigr="TIGR03627 arch_S9P 130 archaeal ribosomal protein
                     S9P"
                     /product="30S ribosomal protein S9"
                     /translation="MKSSTINNNDWSEKTIKSAVDSLGRLYATGRRKESVARVWIKPG
                     SGKFSVNKKGDLISYFKRESVCQTVKMPFVATSMLDKYDVFATVKGGGLSGQAGALAH
                     GISRALSSVSQDLHSILRNGEFLTRDSRVVERKKYGQHKARKKCQFSKR"
                     /besthit="qcoverage 100 , hcoverage 100 , similarity 100
                     , identity 100 , evalue 1.36e-98 , alnlength 151 ,
```

```
                     Bacteria , Proteobacteria , WP_007548415.1 MULTISPECIES:
                     30S ribosomal protein S9 [Wolbachia]"
     gene            4104..4415
                     /locus_tag="wEsol_00008"
     CDS             4104..4415
                     /locus_tag="wEsol_00008"
                     /codon_start=1
                     /transl_table=11
                     /fullproduct="qcoverage 100 , hcoverage 100 , similarity
                     100 , identity 100 , evalue 4.46e-63 , alnlength 103 ,
                     Bacteria , Proteobacteria , WP_006279876.1 MULTISPECIES:
                     HU family DNA-binding protein [Wolbachia]"
                     /cog="HimA COG0776 94 Bacterial nucleoid DNA-binding
                     protein"
                     /pfam="Bac_DNA_binding"
                     /tigr="TIGR00988 hip 94 integration host factor, beta
                     subunit"
                     /product="HU family DNA-binding protein"
                     /translation="MSKEDIIKQLKEDCSSQNIDITKSDLSKIHDAFIKIIKNELHGK
                     GEIRLHGIGTFSTAISQEKQCRNPQSGEIMTVPKKTRVKFKASQTLLSILNEKEKAAV
                     S"
                     /besthit="qcoverage 100 , hcoverage 100 , similarity 100
                     , identity 100 , evalue 4.46e-63 , alnlength 103 ,
                     Bacteria , Proteobacteria , WP_006279876.1 MULTISPECIES:
                     HU family DNA-binding protein [Wolbachia]"
     gene            complement(4408..5115)
                     /locus_tag="wEsol_00009"
     CDS             complement(4408..5115)
                     /locus_tag="wEsol_00009"
                     /codon_start=1
                     /transl_table=11
                     /fullproduct="qcoverage 100 , hcoverage 100 , similarity
                     100 , identity 100 , evalue 6.90e-165 , alnlength 235 ,
                     Bacteria , Proteobacteria , WP_141456804.1 MULTISPECIES:
                     pyridoxine 5'-phosphate synthase [unclassified
                     Wolbachia]"
                     /cog="PdxJ COG0854 243 Pyridoxal phosphate biosynthesis
                     protein"
                     /pfam="PdxJ Pfam-B_19671"
                     /tigr="TIGR00559 pdxJ 239 pyridoxine 5'-phosphate
                     synthase"
                     /product="pyridoxine 5'-phosphate synthase"
                     /translation="MKLGVNIDHVATLRNARGTSYPDPLKAAEIAIDAGADFITVHLR
                     EDRRHIRDEDVFNLKKSISTELNLEIAATEEMLEIAKKVKPYSISIVPEKREELTTEG
                     GLDIVNMHSKLSSIIEEMHSSSIKVSLFIDPNIDQLTYLEKLGTKPDIIEIHTGDYCD
                     NPSEKKLQLITNAAEYVNKLGIECHAGHGITYKHAKKMTRIPHISALNIGHYLISEAV
                     FYGLHSVVKTMKMTMSN"
                     /besthit="qcoverage 100 , hcoverage 100 , similarity 100
                     , identity 100 , evalue 6.90e-165 , alnlength 235 ,
                     Bacteria , Proteobacteria , WP_141456804.1 MULTISPECIES:
                     pyridoxine 5'-phosphate synthase [unclassified
                     Wolbachia]"
ORIGIN
        1 tttaaaatat tggcgttttt tatgttttaa acgcttgaca agcgaaattc agctgctttt
       61 aattgcaact aacctacgct gcaaatgttt aagaaattta ctaagcagaa aaaaagacaa
      121 agaatccccg agttagctag tcttttacta tctctgtcga gtattggcat tttttgatgt
      181 cttgtaacgc tttataagcg cgttcagctt atttagataa aaatctagat gtagatgaag
      241 ttttgtaaag acatacagta tctatatact gcaaaaaatt gaatataaga cgccgataca
      301 ttaagtaatc cttacctttt aatctgcaga ttggcgaaag caaatacaac agcttcactg
      361 ttatgataag ggggctggcg gagtttgtca aggaagtttt ttcgtttctg tgggctacct
```

421 ggattaatat tatatagaaa gtctgctgcc aataaagcct aaacttacca aatacatttc
481 tgtagattca gatctacttg actttggttt aaagtatttt actgttttaa acattttttt
541 tagctcgtta caaaaatctt tatcagattc tccttggaaa atttttacta caaacgtgcc
601 accatggctc aagaaatgct ttgcaaaatt gagcgctgcc tcacataaaa gcataattct
661 gatatgatcc aacgatttta aaccacaaga ttcgggcgcc atgtcagata aaattacatc
721 aaatttttga tccttgaatt tttctcttaa aatttcaaac tcattgataa tatcacattg
781 tataaactct actccattaa ttgcgttcat tggttttatg tcaagagcaa ccacatttgc
841 acctttctga gatgcaactt gtgaccatcc accaggagaa gcaccaagat caataatttt
901 ttgcccttct tggaataatt taaatttgtt atccatttct accagcttat atgctgagcg
961 tgacctataa ccatccttgc tagtttttttg cacatactga tcattcaaat gacgatgtag
1021 ccaccttgtt gaagatagtt ttctgcctct agccgtcttt actctagttt ttattgctaa
1081 taccctactt attattgatt atatctttaa gctcttttttc aactacttct tttaccagtg
1141 catgtagata tttattcagc cattctgaga gctgaggttt taaaagagat gtaactaatt
1201 cttcaacagt aaggttcgct tttttttgtt gtttatgctg taattcgttt tgcatttttc
1261 caagcaacgc tttgatctct tccatattct cttttaaaat tagatgatcg ttattttgcg
1321 cttgtaagcc gctattactc gctttgttat tactcatttg gatattatcg tacaaataaa
1381 tatctttttt ttcttctgaa ttgtagctat ggccattaaa ttggttattg ttgcaagcca
1441 tttcctcact ttggtcatca ttatcaatgt cctctttttc attattttct tttttgccat
1501 cttcttctat ttcttctgga tactcttcct caaggtatag cacatcatca ttttcatcct
1561 ttatttctgc tttatcgtta ctcgcgtttt tacccgatat agcttttttt atatcttcta
1621 ggatatcttt tacagattga ttgccttgtt catcatgcat agtgctaata ccattaaatt
1681 tagaggtcta tactatttat cataaaaagc aagttgtaaa cactaatcac ataattgctt
1741 tttgcttcaa ctaagtctga acgtgcttta aataatgcat cttcagtgtc caaaaggtca
1801 gatgtactct ttaaatttaa gtttacttct tgctcaactc cttccaatgc tagagctgct
1861 gctttttccg cttcttgact tgctttgata atggcctttg ctgtcaacac attattccaa
1921 gcatttacaa ctgcttgttc tatatttttt accgtttcat aataatcgta ggtagatttt
1981 tttgcatcca ttttagcttt actgacacca aaagcattaa ctcctctctt aaagattgga
2041 acatcaagag taaaaactac atgaacattt tctaatagtt tgtctacctt cataccatcc
2101 tccccaaaat ttttgcttgc acttagattt aaagaaggaa gccacttgga gctttcagca
2161 atcacttcca ttccggctgc tctttttttga taaactgctg cttttagaga taaattattg
2221 gtttttgcta actgtaaaca ttcatttagc tctggaacag aaggtagttt atcattagct
2281 tcagaaaggt catcagcatc ttcgccaatc aaatgataat aagcaatatt taccaacttt
2341 aatttacctt cagcatcaac tctttcggat atagaagacg aaaattttgc ttttgctaac
2401 aaaacttcag cattggttac ctctccaagg gaaaagcgtt ttttcatagc tgacaaatgt
2461 tccaaagaaa cgcgttcttt atgttctctg agctttaata tttctgtctt ttgtaaaaca
2521 ttaacatatg ctttcacagc attaagtgca agctcttgtt ttaattgctg aaatcgcatc
2581 ttttctgctt tgagaagatg gcttgatcga ctgaatgtgg caaaggtacc accgccatcg
2641 attaatttct ggcgcaatgt aagttttttt tcagatgtat caagattaaa gttactatcg
2701 aaattgtatt gcaaattaat gtcaggtaaa aatccagcta acccagaaga tttatagtgt
2761 ttttctgcgc ttttatattg gtaaaattga gactttattt ttgagctatt tttgatggct
2821 ttgcttatca cttctcccac atcggttgca tagcaactta tagtattaaa aatgatcgca
2881 agtgtgataa tcaatcgaaa cattctaatt cctatgcata ttggttgcgc agctcaatta
2941 ctaacataaa ttactttaat aatcaataac taatattgtt gacactattg agaaattaag
3001 tatatcatag cttactattg ttggatcaaa taatgaagac tttttttctta aaggagaaac
3061 aaatcaataa aaagtggttt gtcatagatg cagaaggatt agtggtaggg aggcttgcag
3121 catttgtagc gacactatta cgtggaaagc ataaacctga gtatacacct catatggatt
3181 gtggcgataa tgtaattatt atcaacgcag aaaaggtgca tttcaccgga aagaagctta
3241 aagataaaat ttactataag catacaggtt attctggtgg tttgaagaaa actactccag
3301 ataatatttt aaatggcaaa tttcctgagc gtgtaataga aatggcagtg aaaaggatgc
3361 ttgatgatgg gcctatggca cgtagacgct ttgaaaattt gtatgtttat tctggttcag
3421 agcataagca tcaaggacag caacctgaaa agatagattt tgcctctttg aatcgtaaaa
3481 ataaaaagta atggagtaaa aatgaagagt tctacaataa ataacaatga ttggtcagaa
3541 aagacaatta aatcagcagt tgattcgctt ggtcgtttat atgctacagg ccgaagaaaa
3601 gaatctgtag caagagtatg gataaagccg gggagtggaa aatttagtgt taacaaaaaa
3661 ggcgatctaa tttcttattt taaaagagag tcagtgtgcc aaacagtaaa aatgccattt
3721 gttgcaacct ctatgttaga taaatatgat gtatttgcaa ctgtaaaagg tggagggcta
3781 tctggtcaag caggtgctct agctcatgga ataagcagag ctttaagtag cgtaagccaa
3841 gatttgcatt ctatattacg taatggtgag ttcttaacta gagattcacg tgttgttgaa
3901 cgtaagaaat atggtcaaca taaagctcgg aaaaagtgtc aattttctaa gagatagtta
3961 ttttatagtg gtgttttttt gtaagtatta tacaaatatt tgcaaaaatg tatgatatcc

```
     4021 tctatttttg tgatgaattt agttgactat cggtgtcaac ataatatgct atttatgtga
     4081 ttttttcaat attaaggtaa tttatgagta aagaagatat aataaaacaa ttaaaggaag
     4141 attgttctag tcagaatata gatataacaa aatctgattt gagcaagatt catgatgcgt
     4201 ttattaaaat aataaagaat gagctgcatg ggaaaggtga aatacgtttg catggaatag
     4261 gaacattttc tactgctatc agtcaggaaa aacaatgtcg taaccccaa agtggtgaga
     4321 tcatgactgt tcctaaaaag acaagggtaa aatttaaagc aagccaaact cttttaagta
     4381 ttttaaatga gaaggaaaaa gcagcagtta gttagacatt gtcattttca ttgtttttac
     4441 tacgctgtgt aagccgtaaa atacagcttc gctaattaaa tagtggccta tgttaagagc
     4501 tgagatgtga ggtattcttg tcatcttctt agcgtgttta taagttatgc catgtcctgc
     4561 gtggcattct attcctaatt tattgacgta ctctgcagca ttagtaatta actgtaactt
     4621 tttttctgat ggattatcac agtaatcccc tgtgtgaatt tctattatgt caggttttgt
     4681 ccctagcttt tcaagatatg ttagttgatc aatatttgga tcgataaaca gtgagacttt
     4741 tatgctagag ctatgcattt cctctattat actagaaagt ttactgtgca tattcacgat
     4801 atccaggcca ccttcggttg ttaattcttc tctttttct ggtactatac taatcgaata
     4861 gggttttacc ttttttgcta tttcaagcat ttcttccgtg gctgcaattt caagatttaa
     4921 ctcagtgcta atgctttttt ttaggttaaa tacgtcttca tctctaatat gccttctatc
     4981 ttctcgtaag tgtacagtaa taaagtctgc tccagcgtca atggctattt ctgctgcttt
     5041 caatggatct ggataagaag ttccgcgtgc gttgcggagg gttgcaacgt gatcgatatt
     5101 aacacctagt ttcacaatct acctcagaaa ttatttatag attatacatg ttattagttt
     5161 ttttgcatag tcattgtttg accttaccca gaa
//
LOCUS       JAQZAU010000002            7388 bp    dna     linear   ENV 17-MAR-2023
DEFINITION  MAG: Wolbachia pipientis isolate wEsol contig_10300, whole genome
            shotgun sequence.
ACCESSION   JAQZAU010000002 JAQZAU010000000
VERSION     JAQZAU010000002.1
DBLINK      BioProject: PRJNA929258
            BioSample: SAMN33142973
KEYWORDS    WGS; ENV; Metagenome Assembled Genome; MAG.
SOURCE      Wolbachia pipientis (insect metagenome)
  ORGANISM  Wolbachia pipientis
            Bacteria; Pseudomonadota; Alphaproteobacteria; Rickettsiales;
            Anaplasmataceae; Wolbachieae; Wolbachia.
REFERENCE   1  (bases 1 to 7388)
  AUTHORS   Fiutek,N., Couger,B.M., Roy,S., de la Torre,J.R. and Connor,E.F.
  TITLE     Genomic assessment of the contribution of the Wolbachia
            endosymbiont of Eurosta solidaginis to gall-induction
  JOURNAL   Unpublished
REFERENCE   2  (bases 1 to 7388)
  AUTHORS   Fiutek,N., Couger,B.M., Roy,S., de la Torre,J.R. and Connor,E.F.
  TITLE     Direct Submission
  JOURNAL   Submitted (10-FEB-2023) Biology, San Francisco State University,
            1600 Holloway Ave, San Francisco, CA 94132, USA
COMMENT     ##Genome-Assembly-Data-START##
            Assembly Date          :: JUN-2019
            Assembly Method        :: FLYE v. 06-2019
            Genome Representation  :: Full
            Expected Final Version :: Yes
            Genome Coverage        :: 100.0x
            Sequencing Technology  :: Illumina HiSeq; PacBio Sequel
            ##Genome-Assembly-Data-END##
FEATURES             Location/Qualifiersw
     source          1..7388
                     /organism="Wolbachia pipientis"
                     /mol_type="genomic DNA"
                     /submitter_seqid="contig_10300"
                     /isolate="wEsol"
                     /isolation_source="galls on Solidago altissima"
                     /host="Eurosta solidaginis MN-2018-EFC"
                     /db_xref="taxon:955"
```

```
                     /environmental_sample
                     /country="USA: Cowling Arboretum, Carleton College,
                     Northfield, MN"
                     /lat_lon="44.48 N 93.13 W"
                     /altitude="280 m"
                     /collection_date="2018-11"
                     /metagenome_source="insect metagenome"
                     /note="metagenomic"
     gene            complement(<1..171)
                     /locus_tag="wEsol_00010"
     CDS             complement(<1..171)
                     /locus_tag="wEsol_00010"
                     /codon_start=1
                     /transl_table=11
                     /product="putative transposase"
                     /pfam="Transposase_20"
                     /translation="MKEIRQDIEDAIAELKGKNELLISYKGIGSKTASVLLTEVPELG
                     RLENREIACLVGV"
                     /besthit="qcoverage 100 , hcoverage 55.3398058252427 ,
                     similarity 100 , identity 100 , evalue 4.67e-30 ,
                     alnlength 57 , Bacteria , Proteobacteria , WP_213862765.1
                     transposase [Wolbachia endosymbiont of Rhagoletis
                     cerasi]"
     gene            complement(495..662)
                     /locus_tag="wEsol_00011"
     CDS             complement(495..662)
                     /locus_tag="wEsol_00011"
                     /codon_start=1
                     /transl_table=11
                     /fullproduct="qcoverage 100 , hcoverage 100 , similarity
                     100 , identity 100 , evalue 2.77e-31 , alnlength 55 ,
                     Bacteria , Proteobacteria , WP_155968863.1 MULTISPECIES:
                     hypothetical protein [Wolbachia]"
                     /translation="MARIIFMGVDVSKEALDISINGKHKRIANVSDAISKFIETNIVD
                     LPIKLCVSWWL"
                     /product="hypothetical protein"
                     /besthit="qcoverage 100 , hcoverage 100 , similarity 100
                     , identity 100 , evalue 2.77e-31 , alnlength 55 ,
                     Bacteria , Proteobacteria , WP_155968863.1 MULTISPECIES:
                     hypothetical protein [Wolbachia]"
     gene            complement(1378..1719)
                     /locus_tag="wEsol_00012"
     CDS             complement(1378..1719)
                     /locus_tag="wEsol_00012"
                     /codon_start=1
                     /transl_table=11
                     /fullproduct="qcoverage 100 , hcoverage 100 , similarity
                     100 , identity 100 , evalue 1.53e-79 , alnlength 113 ,
                     Bacteria , Proteobacteria , AAS13810.1 transposase, IS5
                     family, OrfB [Wolbachia endosymbiont of Drosophila
                     melanogaster]"
                     /cog="COG3293 COG3293 124 Transposase and inactivated
                     derivatives"
                     /pfam="DDE_Tnp_1_2 DDE_Tnp_1"
                     /product="transposase, IS5 family, OrfB"
                     /translation="MRTKYTKVKKMWADMGYQGRNLKNHIKEEYDIDIEIVKRPPCRF
                     WVHKDTPPELLPTREQGFKVQPRRWVVERTFAWVNRNRRLSKEYDLLTTSTENFIYLA
                     MSRVMLKREYA"
                     /besthit="qcoverage 100 , hcoverage 100 , similarity 100
                     , identity 100 , evalue 1.53e-79 , alnlength 113 ,
```

```
                     Bacteria , Proteobacteria , AAS13810.1 transposase, IS5
                     family, OrfB [Wolbachia endosymbiont of Drosophila
                     melanogaster]"
     gene            complement(1830..2210)
                     /locus_tag="wEsol_00013"
     CDS             complement(1830..2210)
                     /locus_tag="wEsol_00013"
                     /codon_start=1
                     /transl_table=11
                     /fullproduct="qcoverage 100 , hcoverage 100 , similarity
                     100 , identity 100 , evalue 1.16e-87 , alnlength 126 ,
                     Bacteria , Proteobacteria , AOV87106.1 transposase
                     [Wolbachia endosymbiont of Drosophila incompta]"
                     /cog="COG3293 COG3293 124 Transposase and inactivated
                     derivatives"
                     /pfam="DUF4096"
                     /product="transposase"
                     /translation="MRKKYPTDLSEREWARIEKHFRVSYKKGGRPPKYSKREILEAIF
                     YVLRTGCQWRYLPNDFPLWKTVYEQFRQWKKQGIFEKMNYEITKYSRRKIGRNEQPSA
                     CIVDSQSVKTTEKGGSKAMMEVKK"
                     /besthit="qcoverage 100 , hcoverage 100 , similarity 100
                     , identity 100 , evalue 1.16e-87 , alnlength 126 ,
                     Bacteria , Proteobacteria , AOV87106.1 transposase
                     [Wolbachia endosymbiont of Drosophila incompta]"
     gene            2282..2692
                     /locus_tag="wEsol_00014"
     CDS             2282..2692
                     /locus_tag="wEsol_00014"
                     /codon_start=1
                     /transl_table=11
                     /product="hypothetical protein"
                     /translation="MEKYTNAWFNKKDNHDCANDGTCGQSIKDFRIGNSDISFFDSNK
                     NLIAKVPKLPKYSSDKYGHQLYKEAESKQFNYFSTNIHDVLSNSFTVKYGYINCTIKY
                     DNLSEQEQNQIETDIKTAYEAFKEKFCFKDSSIS"
                     /besthit="qcoverage 99.2647058823529 , hcoverage
                     63.0841121495327 , similarity 98.5 , identity 97.8 ,
                     evalue 4.57e-91 , alnlength 135 , Bacteria ,
                     Proteobacteria , AAX14461.1 hypothetical protein
                     WwMoj0018, partial [Wolbachia endosymbiont of Drosophila
                     mojavensis]"
     gene            2837..3049
                     /locus_tag="wEsol_00015"
     CDS             2837..3049
                     /locus_tag="wEsol_00015"
                     /codon_start=1
                     /transl_table=11
                     /product="hypothetical protein"
                     /translation="MDFVLGHELGHIFQLCFSPSATVQTLSILDTELIANVVGREVEE
                     KNYKAICKQMGVDEYKYDGWSFILNT"
                     /besthit="qcoverage 95.7142857142857 , hcoverage
                     13.7577002053388 , similarity 100 , identity 100 , evalue
                     3.07e-38 , alnlength 67 , Bacteria , Proteobacteria ,
                     WP_197907219.1 hypothetical protein [Wolbachia
                     endosymbiont of Kradibia gibbosae]"
     gene            3159..4370
                     /locus_tag="wEsol_00016"
     CDS             3159..4370
                     /locus_tag="wEsol_00016"
                     /codon_start=1
                     /transl_table=11
```

```
                     /product="hypothetical protein"
                     /translation="MFKFKYKDTTGSIYLKDLSEDEKFQIIQRVKNSGLDEYEDHGWI
                     FSFKYKGTTYNVRYKDFSEDEKFKAIQNVKNIHELVDEYKDHGWIFSFKYKGTTYNVR
                     CKYFSEDEKFQFIKDIKNSHESLVSECDGLGNENTVLSIHVDENKEISRMYFRNSDKN
                     IFKQLDKTLISRSKSEEEVNENRTNPEPEEEQKGDDYTEVTGMLQFKYKGTNYAIQHR
                     GYLLVEEEKLRLIQDIKNSHESLVSERYRLNEGSLSQNATVVIFVDENKDIEKMYFHN
                     PDKNMSKWLDKTLISRSKPEQDVDDTAGKEPEPESAEEDTQQPSNFLSNIFSAIKSII
                     DSISSLFSWLFGSKEKESNQQSDDNPSLFKLDANELDHEVKPDANELGHDVRDHLSDN
                     YNHYNSSDGLM"
                     /besthit="qcoverage 99.7518610421836 , hcoverage
                     61.6564417177914 , similarity 95.8 , identity 95.0 ,
                     evalue 1.53e-173 , alnlength 402 , Bacteria ,
                     Proteobacteria , WP_141456949.1 hypothetical protein
                     [Wolbachia endosymbiont of Carposina sasakii]"
     gene            4638..4967
                     /locus_tag="wEsol_00017"
     CDS             4638..4967
                     /locus_tag="wEsol_00017"
                     /codon_start=1
                     /transl_table=11
                     /pfam="DDE_Tnp_1_5"
                     /fullproduct="qcoverage 100 , hcoverage 100 , similarity
                     100 , identity 100 , evalue 4.31e-75 , alnlength 109 ,
                     Bacteria , Proteobacteria , WP_141456678.1 MULTISPECIES:
                     transposase [unclassified Wolbachia]"
                     /translation="MPQKMRVSNCHEYNKFLQERGSIFCYINDAIENWYENCPKMQGG
                     NYIYSDKVVILVHIIVSFFRIGLRQTVGFIKGYLQQIGRDLAVISYSQASRRFKKLNI
                     KINDCRK"
                     /product="transposase"
                     /besthit="qcoverage 100 , hcoverage 100 , similarity 100
                     , identity 100 , evalue 4.31e-75 , alnlength 109 ,
                     Bacteria , Proteobacteria , WP_141456678.1 MULTISPECIES:
                     transposase [unclassified Wolbachia]"
     gene            4951..5151
                     /locus_tag="wEsol_00018"
     CDS             4951..5151
                     /locus_tag="wEsol_00018"
                     /codon_start=1
                     /transl_table=11
                     /product="hypothetical protein"
                     /translation="MIAENNMEDIEIAIDSTGISIYNNTPGHSKENSTDRKYRGYEQT
                     RKLHVMLNINNKKAIAVKYSNG"
                     /besthit="qcoverage 100 , hcoverage 31.2796208530806 ,
                     similarity 100 , identity 100 , evalue 1.52e-35 ,
                     alnlength 66 , Bacteria , Proteobacteria , WP_081600764.1
                     MULTISPECIES: transposase [unclassified Wolbachia]"
     gene            5171..5578
                     /locus_tag="wEsol_00019"
     CDS             5171..5578
                     /locus_tag="wEsol_00019"
                     /codon_start=1
                     /transl_table=11
                     /pfam="DDE_Tnp_1_2"
                     /fullproduct="qcoverage 100 , hcoverage 100 , similarity
                     100 , identity 100 , evalue 9.23e-96 , alnlength 135 ,
                     Bacteria , Proteobacteria , WP_174516160.1 MULTISPECIES:
                     transposase [unclassified Wolbachia]"
                     /translation="MLKEVDFQHAIKALYADRACDRHKFYKLCNEYDIKTKIPPINNA
                     AEHPEIDYMSDRNAAIRLIKLYGEDGMKEWKKEVNYGKRSYIEGFFSRLKQIFGFSFR
                     NKSEINREKELLIKCYLLNKFTDIGMAKFEIVT"
```

```
                     /product="transposase"
                     /besthit="qcoverage 100 , hcoverage 100 , similarity 100
                     , identity 100 , evalue 9.23e-96 , alnlength 135 ,
                     Bacteria , Proteobacteria , WP_174516160.1 MULTISPECIES:
                     transposase [unclassified Wolbachia]"
     gene            5883..6386
                     /locus_tag="wEsol_00020"
     CDS             5883..6386
                     /locus_tag="wEsol_00020"
                     /codon_start=1
                     /transl_table=11
                     /pfam="DsbC"
                     /fullproduct="qcoverage 100 , hcoverage 100 , similarity
                     100 , identity 100 , evalue 3.64e-122 , alnlength 167 ,
                     Bacteria , Proteobacteria , WP_155968372.1 MULTISPECIES:
                     hypothetical protein [Wolbachia]"
                     /translation="MKIHIYLILFLFFSCTQLYADEEIAKFDLLIGEINEASFTLKGA
                     IKVTVEPGWHIYYKDPGDFGLPTSISYKGNASDINIHWPIPKEHKDKVGRETFVSNVY
                     EGMVLFPFKMNIFLNQGYIDLNFHINYAICKDRCIPKNVEIKIRQPLKDFIDSDINKL
                     INEWYEK"
                     /product="hypothetical protein"
                     /besthit="qcoverage 100 , hcoverage 100 , similarity 100
                     , identity 100 , evalue 3.64e-122 , alnlength 167 ,
                     Bacteria , Proteobacteria , WP_155968372.1 MULTISPECIES:
                     hypothetical protein [Wolbachia]"
     gene            6824..7024
                     /locus_tag="wEsol_00021"
     CDS             6824..7024
                     /locus_tag="wEsol_00021"
                     /codon_start=1
                     /transl_table=11
                     /product="hypothetical protein"
                     /translation="MVSHKKFRSQCHALTVRTLRYKSNLYQEKRCHPSAQTLGWLCCI
                     ASYDGLKIKKIGNISSLLFWYL"
ORIGIN
        1 aactccaact aagcatgcaa tttctctatt ctctaatcta ccaagctcag gcacctctgt
       61 cagtagaacg ctcgccgtct ttgatccaat acctttatag ctgattaaaa gctcattttt
      121 tcctttcaat tcagctattg cgtcttctat gtcttgccta atttctttca attgtttttc
      181 agcaaaccct atctgtcttt ctatgtattc tctagctttt cccgtcgcac tttgcaggcg
      241 acacttgttt gcatgaattg catcatttaa attacgctca actgccccca gctcctttaa
      301 ttcctcttgt ttttgtgaaa ttatacatcc ttttacttca ccgtctttac tgacaaattc
      361 agcgtatttt tccagtaatt tagcatctaa cttgtcagtt tttgcaaaat gattgcaagc
      421 tttggcaaat gcatataccc gattcgggtg agctttatgg acagttatct tgcaacaatt
      481 ttataatgag cttttcatag ccaccagctt acacataact tgatgggtaa atcgactata
      541 tttgtttcta taaacttaga tatagcatca ctcacgtttg ctatgcgctt atgcttgcca
      601 ttaattgaga tgtccaatgc ttccttggaa acatctactc ccatgaaaat tattctagcc
      661 atttttgtac ctataaatat aatcaaacaa taatgcaaaa tgctgtatac aggattgtaa
      721 ttcgatcttt tagatccacc taccgttcgt actacatttt gctttaagcc ttactattac
      781 ccacaaataa aaacatagta ttgtatttac tcaaataatt taattttatt aacctgaaag
      841 cttattagat taaaattatt aaactaagag ttataatttt agcaactaaa agctgtaaat
      901 tgcaccatat aaacagttat ttcaagcaaa agttatgcca ttaagagcta tgcaacaaac
      961 atttatattt gtgggtaata gtaagatcta aatccgatag agcattgttg gcacaccatc
     1021 aaaagttgcc tcagacctct aatgcatcaa tgtacagact tacacctttt agttggtaat
     1081 accattttag acgtttatca ttcgttttag aaaatactat agtagcggaa tgacgaattt
     1141 ttgtttttca aattgtcagt aaatctaagt cagtttagct atataaaaca tagtataaaa
     1201 agagctattt tagcaaaatt ttaaacaaat aaagttaatt acttcacaat tgatacctta
     1261 cgagaaaagc acatttgata cagatttaaa atacaagtta acgaattatt atgattttta
     1321 ccaagataat aatatagtaa ggtttggtag tgagaggttg tccggaaact agtaaattca
     1381 agcatattcc ctctttaaca taaccctact catagctagg tatatgaaat tctcagtgga
     1441 tgttgtgagt aaatcatact ccttcgatag ccttctattc ctattaaccc aagcaaaagt
```

1501 cctttctaca acccatcttc ttggctgtac tttaaaccct tgttctcttg ttggtagtag
1561 ctcaggtggc gtatctttgt gcacccaaaa tctacatgga ggccttttaa caatttcaat
1621 atctatgtca tattcttcct ttatgtgatt ctttaaattt cttccttggt atcccatgtc
1681 agcccacatt tttttaactt tagtatattt tgttctcata ttgtttaatg ctattttaat
1741 accatctcta tcattttcgt tagcagcgcc tacgtaacaa cctagtataa aaccctgagt
1801 gtctgtaatt atatgccttt ttctaccctt tacttttttta cttccatcat agcctttgat
1861 ccccccttttt ctgtagtctt tacagattga ctatctacta tacaggcact cggctgctca
1921 ttccttccta tttttcttct actatatttt gtaatttcat aattcatttt ctcaaaaatt
1981 ccctgcttct tccattgcct gaactgctca tacacagtct tccatagcgg aaaatcattt
2041 ggtaaatacc gccattgaca ccctgtacgc aatacataga aaattgcttc taatatttct
2101 cttttgctat actttggcgg ccttcctcct ttcttgtatg atactctgaa gtgtttttct
2161 attcttgccc attccctttc gcttagatct gttggatact tttttctcat ctttaccccg
2221 tactaaaatt tcagatatta tagcctttta cttatttccg gacaacctct gaggtatcaa
2281 aatggaaaaa tacaccaacg catggtttaa taagaaagat aatcatgatt gtgcaaatga
2341 tggcacttgc gggcaatcta taaaggattt taggattggt aattctgata tttctttttt
2401 tgatagtaac aaaaacttaa ttgccaaagt acctaaacta ccaaagtatt ctagtgataa
2461 atatgggcat caattgtata aagaagccga gtcaaagcag ttcaattatt tttccacaaa
2521 tatacatgat gttttgtcta acagctttac cgttaaatac gggtatataa attgtactat
2581 aaaatacgac aacttatctg agcaggaaca aaaccaaata gaaacagaca ttaaaactgc
2641 ctatgaggca tttaaagaaa agttttgctt caaagatagt agcataagtt aaaatataac
2701 ggtttacatt ttcaataata gaagcgatta tacaaagtat aacaatcttc tcggcataga
2761 tgaagatggt ggtccaggat atattacacg aggagtaaca gattgtcaga atactctgac
2821 atataagcaa agtgccatgg attttgttct gggtcatgag ctaggccaca tatttcaatt
2881 gtgtttttca ccatcggcaa cagtccaaac tttgagtatt ctggatactg aactcatagc
2941 aaatgttgtt ggacgagaag tagaagaaaa aaattataaa gctatttgca agcagatggg
3001 tgtagatgaa tataaatacg atggatggag ttttatttta aatacatagg tacaactgta
3061 gtgtataccg cctaaacctt tcagaagaag agaaatttca aattatacaa cgtgtcaaga
3121 gttctggact agtagatgaa tatgaagacc atggatggat gtttaaattt aaatataaag
3181 atacaactgg tagtatatac ctcaaggacc tttcagaaga tgaaaaattt caaattatac
3241 aacgtgtcaa gaattctgga ctagatgaat atgaagacca tggatggata tttagtttta
3301 aatacaaagg caccacttat aacgtaagat acaaggactt ttcagaagat gaaaaattta
3361 aagctataca aaatgtcaag aatattcatg aactagtaga tgaatataaa gaccatggat
3421 ggatatttag ttttaaatac aaaggcacca cttataacgt aagatgcaag tacttttcag
3481 aagatgaaaa atttcaattt atcaaagaca ttaagaactc tcatgaatcg ctcgttagtg
3541 aatgtgatgg attggggaat gagaatacag ttttatcgat tcacgttgat gaaaataagg
3601 agataagtcg aatgtatttt cgaaactccg acaaaaatat attcaaacag ctagataaga
3661 cacttataag cagaagtaag tctgaagaag aggtaaatga aaaccgcacg aatccagagc
3721 cagaagaaga acaaaaaggt gatgactaca cagaggttac aggaatgttg caatttaagt
3781 acaaagggac caattatgct atacaacaca gaggttacct tttagtagaa gaagaaaaac
3841 ttcgattgat tcaagatata aagaactctc atgaatcgct cgttagtgaa cgttatagat
3901 taaatgaagg gtcattaagt cagaatgcaa ctgtagtaat ttttgttgat gaaaacaagg
3961 acatagagaa aatgtatttt cataatccag acaagaatat gtccaaatgg ttagataaga
4021 cactcataag cagaagtaag cctgaacaag atgtggatga cacagcagga aaagagcctg
4081 aaccagaatc agccgaagag gatactcagc aacccagtaa tttcttatcc aatatttttt
4141 cagcaattaa atccattatt gattctatct catcactctt ttcatggtta tttgggtcga
4201 aagagaaaga gtctaatcaa caatctgatg ataatccttc tttgtttaag ctcgatgcta
4261 atgagttaga tcacgaagtt aagcctgatg ccaatgagtt aggtcacgat gttagagatc
4321 acttatcaga caattataac cattacaact catctgatgg gctaatgtaa tatagctaat
4381 ttacagtatc tgttcagatg catggctaat gtacaaatat tgaaacaaaa attccagtgc
4441 ccctgtgatg tcattccagt gcttgacact ggatccgctt tccatatcat caaaacttgt
4501 ttttacaaaa aacagctata gctgccaact taataaaatt ctggatccca gtgggctttg
4561 ttgcatcgcc atgaaaagaa ctggcagtta ctgacaaaat tcattataaa tagccattta
4621 actgttgaag aaaaaatatg ccacaaaaaa tgagagtcag taactgccat gaatataaca
4681 aatttctcca agaaagagga agcatttttt gttatatcaa tgacgccata gaaaattggt
4741 acgagaattg tccaaaaatg cagggcggca actatattta tagtgataaa gttgtgattt
4801 tggtgcatat aatcgtcagt ttctttagaa ttggtttaag acaaacagtc gggtttataa
4861 aagggtattt gcaacaaata ggaagagatt tggcagttat cagctattca caagcatcaa
4921 gaaggtttaa gaaacttaat attaagataa atgattgcag aaaataatat ggaagatatc
4981 gaaattgcta tagacagtac aggaattagt atatacaaca acactcctgg tcacagcaag
5041 gaaaatagca ctgacagaaa atatcgtggc tatgagcaaa cgagaaaatt gcacgtaatg

```
     5101 ttgaatataa acaacaaaaa agccatagct gtaaaataca gtaacggctg atcactatgg
     5161 agcttgtgat ttgctaaaag aagttgattt tcagcatgcc ataaaagcac tatatgcaga
     5221 tagagcatgt gataggcaca agttttataa gttgtgtaac gaatatgata taaagacaaa
     5281 aattccacca ataaacaatg cggcagaaca tccagaaata gattatatgt ctgacaggaa
     5341 tgctgctatt aggttaataa aattatacgg tgaagatggc atgaaagaat ggaaaaaaga
     5401 agtaaattat gggaaaagat cttatattga agggtttttc tcaagattaa agcaaatatt
     5461 cggatttagt tttaggaata aatctgagat aaatcgagaa aaagaactgc taatcaaatg
     5521 ctatttgctt aataaattca ctgatattgg tatggctaaa tttgagatag ttacatgaat
     5581 ttaccataaa tcaccatctc ataaagtgtg atgcaacaaa gccatcccag tgtcaagcac
     5641 tgggatgaca ccctccttat gggccaaaat cacaatgttc gtacagttgt ggattcattc
     5701 gcggtatctc ggcatagatt ccgctaacaa gtagcggaat gacgaatttg tcgtttttca
     5761 gattgtcgta agtcagttta gctatagcat aatgctatat taataaataa tctatataat
     5821 actattaaat ataataatta aaatctaaat tactttctct ataaaaggag aaatttacct
     5881 taatgaaaat acatatatat ttaatattgt ttttattttt ttcttgcact caactatatg
     5941 ctgacgagga aattgcaaaa tttgatttac ttattggcga gataaatgaa gcaagcttca
     6001 ctcttaaagg tgcaataaag gttacagtag aaccaggctg gcacatatat tataaagatc
     6061 ctggagattt tggccttccc acgtctatta gttacaaagg taatgcatca gatataaata
     6121 ttcactggcc tattccaaag gaacataaag ataaagtagg gagagagacg tttgtaagta
     6181 acgtatatga gggtatggta ttatttccct ttaagatgaa tatattttta aatcaagggt
     6241 atattgacct taacttccat ataaattatg cgatatgtaa agataggtgc atccctaaaa
     6301 atgttgagat aaaaatcagg caaccattaa aggattttat agattctgat atcaataaac
     6361 ttataaatga gtggtatgaa aaatagagtt ctcatggggt aggtgtcatt ccagcgcgtg
     6421 acactggaat ggctttgttg catcgctact tatgaaaggc taactataat taggattata
     6481 agcaacttat tgaaaatctt gtttttttgc aatcaatctg atcaaattta agaatagtaa
     6541 tttgttattt atattaataa atttaattct attgaaaata gctaaagcat tgaaattctt
     6601 gaattttagc cggattagtg agtggtagtg aaatttcttt tactcaaatt taggtattca
     6661 ctgaccgttc ttgttataaa taccagaata ataagctact gatattccct atcttttta
     6721 tctttaatcc atcatagcta gcgatgcaac aaagcccact ggaatctcat ttactttttt
     6781 gttcactatg tgaggaactt gggcctacaa gtatataaac ataatggttt cgcataaaaa
     6841 atttagatcc cagtgtcacg cactaactgt acgaacattg agatataaga gtaatttgta
     6901 ccaagaaaaa agatgtcatc ccagtgccca gacactggga tggctttgtt gcatcgctag
     6961 ctatgatgga ttaaagataa aaaagatagg gaatatcagt agcttattat tctggtattt
     7021 ataacaagaa cggtcagtga atacctaaat ttgagtaaaa gaaatttcac taccactcac
     7081 taatccggct aaaattcaag aatttcaatg ctttagctat tttcaataga attaaattta
     7141 ttaatataaa taacaaatta ctattcttaa atttgatcag attgattgca aaaaaacaag
     7201 attttcaata ggttgcttat aatcctaact atagttagcc tttcataagt agcgatgcaa
     7261 caaagcccac tgggatccag attggacact aagttggtga gtataaaagt agctaacctt
     7321 atgttaaaac acaatatttt tggtgagggg ttgcattaaa agctggattc cagtgtcaag
     7381 cactggaa
//
LOCUS       JAQZAU010000003           54148 bp    dna     linear   ENV 17-MAR-2023
DEFINITION  MAG: Wolbachia pipientis isolate wEsol contig_10723, whole genome
            shotgun sequence.
ACCESSION   JAQZAU010000003  JAQZAU010000000
VERSION     JAQZAU010000003.1
DBLINK      BioProject: PRJNA929258
            BioSample: SAMN33142973
KEYWORDS    WGS; ENV; Metagenome Assembled Genome; MAG.
SOURCE      Wolbachia pipientis (insect metagenome)
  ORGANISM  Wolbachia pipientis
            Bacteria; Pseudomonadota; Alphaproteobacteria; Rickettsiales;
            Anaplasmataceae; Wolbachieae; Wolbachia.
REFERENCE   1  (bases 1 to 54148)
  AUTHORS   Fiutek,N., Couger,B.M., Roy,S., de la Torre,J.R. and Connor,E.F.
  TITLE     Genomic assessment of the contribution of the Wolbachia
            endosymbiont of Eurosta solidaginis to gall-induction
  JOURNAL   International Journal Molecular Sciences
REFERENCE   2  (bases 1 to 54148)
  AUTHORS   Fiutek,N., Couger,B.M., Roy,S., de la Torre,J.R. and Connor,E.F.
  TITLE     Direct Submission
```

```
  JOURNAL   Submitted (10-FEB-2023) Biology, San Francisco State University,
            1600 Holloway Ave, San Francisco, CA 94132, USA
COMMENT     ##Genome-Assembly-Data-START##
            Assembly Date          :: JUN-2019
            Assembly Method        :: FLYE v. 06-2019
            Genome Representation  :: Full
            Expected Final Version :: Yes
            Genome Coverage        :: 100.0x
            Sequencing Technology  :: Illumina HiSeq; PacBio Sequel
            ##Genome-Assembly-Data-END##
FEATURES             Location/Qualifiers
     source          1..54148
                     /organism="Wolbachia pipientis"
                     /mol_type="genomic DNA"
                     /submitter_seqid="contig_10723"
                     /isolate="wEsol"
                     /isolation_source="galls on Solidago altissima"
                     /host="Eurosta solidaginis MN-2018-EFC"
                     /db_xref="taxon:955"
                     /environmental_sample
                     /country="USA: Cowling Arboretum, Carleton College,
                     Northfield, MN"
                     /lat_lon="44.48 N 93.13 W"
                     /altitude="280 m"
                     /collection_date="2018-11"
                     /metagenome_source="insect metagenome"
                     /note="metagenomic"
     gene            <1..214
                     /locus_tag="wEsol_00022"
     CDS             <1..214
                     /locus_tag="wEsol_00022"
                     /codon_start=1
                     /transl_table=11
                     /product="hypothetical protein"
                     /translation="X LWLLTLIFCGIFFLSRLNGYFIMNFVSSHQIFSVAMQQSHPS
                     VKHWNDITGALEFLFQYLYISHASEQIL"
     gene            complement(225..2177)
                     /locus_tag="wEsol_00023"
     CDS             complement(225..2177)
                     /locus_tag="wEsol_00023"
                     /codon_start=1
                     /transl_table=11
                     /fullproduct="qcoverage 100 , hcoverage 100 , similarity
                     99.7 , identity 99.7 , evalue 0.0 , alnlength 650 ,
                     Bacteria , Proteobacteria , WP_209452344.1 hypothetical
                     protein [Wolbachia endosymbiont of Ceratosolen solmsi]"
                     /translation="MEKYTNAWFNKKDNHDCANDGTCGQSIKDFRIGNSDISFFDSNK
                     NLIAKVPKLPKYSSDKYGHQLYKEAESKQFNYFSTNIHDVLSNSFTVKYGYINCTIKY
                     DNLSEQEQNQIETDIKTAYEAFKEKFCFKDSSISKNITVYIFNNRSDYTKYNNLLGID
                     EDGSPGYITRGVTDCQNTLTYKQSAMDFVLGHELGHIFQLCFSPSATVQTLSILDTEL
                     IANVVGREVEEKNYKAICKQMGVDEYKYDGWSFIFKYIGTTGSVYRLNLSEEEKFQII
                     QRVKSSGLDEYEDHGWMFKFKYKDTTGSIYLKDLSEDEKFQIIQRVKNSGLDEYEDHG
                     WIFSFKYKGTTYNVRCKYFSEEEKFQFIKDIKNSHESLVSECDGLGNENTVLSIHVDE
                     NKEISRMYFRNSDKNIFKQLDKTLISRSKSEEEVNENRTNPEPEEEQKGDDYTEVTGM
                     LQFKYKGTNYAIQHRGYLLVEEEKLRLIQDIKNSHESLVSERYRLNEGSLSQNATVVI
                     FVDENKDIEKMYFHNPDKNMSKWLDKTLISRSKPEQDVDDTAGKEPEPESAEEDTQQP
                     SNFLSNIFSAIKSIIDSISSLFSWLFGSKEKESNQQSDDNPSLFKLDANELDHEVKPD
                     ANELGHDVRDHLSDNYNHYNSSDGLM"
                     /product="hypothetical protein"
                     /besthit="qcoverage 100 , hcoverage 100 , similarity 99.7
```

```
                     , identity 99.7 , evalue 0.0 , alnlength 650 , Bacteria ,
                     Proteobacteria , WP_209452344.1 hypothetical protein
                     [Wolbachia endosymbiont of Ceratosolen solmsi]"
     gene            2878..3045
                     /locus_tag="wEsol_00024"
     CDS             2878..3045
                     /locus_tag="wEsol_00024"
                     /codon_start=1
                     /transl_table=11
                     /fullproduct="qcoverage 100 , hcoverage 100 , similarity
                     100 , identity 100 , evalue 2.77e-31 , alnlength 55 ,
                     Bacteria , Proteobacteria , WP_153296805.1 MULTISPECIES:
                     hypothetical protein [Wolbachia]"
                     /translation="MARIIFMGVDVSKEALDISINGKHKRIANVSDAISKFIETNIVD
                     LPIKLYVNWWL"
                     /product="hypothetical protein"
                     /besthit="qcoverage 100 , hcoverage 100 , similarity 100
                     , identity 100 , evalue 2.77e-31 , alnlength 55 ,
                     Bacteria , Proteobacteria , WP_153296805.1 MULTISPECIES:
                     hypothetical protein [Wolbachia]"
     gene            3494..3799
                     /locus_tag="wEsol_00025"
     CDS             3494..3799
                     /locus_tag="wEsol_00025"
                     /codon_start=1
                     /transl_table=11
                     /fullproduct="qcoverage 100 , hcoverage 100 , similarity
                     100 , identity 100 , evalue 2.23e-62 , alnlength 101 ,
                     Bacteria , Proteobacteria , WP_051062205.1 MULTISPECIES:
                     transposase [unclassified Wolbachia]"
                     /cog="COG3547 COG3547 303 Transposase and inactivated
                     derivatives"
                     /pfam="Transposase_20"
                     /product="transposase"
                     /translation="MPELGRLENREIACLVGVAPKTSESGRKVGKAQIKGGRFYARKA
                     LYMSALVAMRHNRKMKAFYQRLVDSGKAAKVALVAVMRKIIVCLNAMIKNNNFYLDS"
                     /besthit="qcoverage 100 , hcoverage 100 , similarity 100
                     , identity 100 , evalue 2.23e-62 , alnlength 101 ,
                     Bacteria , Proteobacteria , WP_051062205.1 MULTISPECIES:
                     transposase [unclassified Wolbachia]"
     gene            3875..5248
                     /locus_tag="wEsol_00026"
     CDS             3875..5248
                     /locus_tag="wEsol_00026"
                     /codon_start=1
                     /transl_table=11
                     /fullproduct="qcoverage 100 , hcoverage 100 , similarity
                     100 , identity 100 , evalue 0.0 , alnlength 457 ,
                     Bacteria , Proteobacteria , WP_015589121.1 MULTISPECIES:
                     dihydrolipoyl dehydrogenase [unclassified Wolbachia]"
                     /cog="Lpd COG1249 454 Pyruvate/2-oxoglutarate
                     dehydrogenase complex, dihydrolipoamide dehydrogenase
                     (E3) component, and related enzymes"
                     /pfam="FAD_binding_3 Thi4 HI0933_like Pyr_redox_2 GIDA
                     DAO FAD_oxidored FAD_binding_2 Pyr_redox_3 NAD_binding_8
                     K_oxygenase 2-Hacid_dh_C Pyr_redox FAD_binding_3 3HCDH_N
                     NAD_binding_8 HI0933_like Pyr_redox_3 DAO NAD_binding_9
                     Pyr_redox_dim"
                     /tigr="TIGR01350 lipoamide_DH 461 dihydrolipoyl
                     dehydrogenase"
```

```
                     /product="dihydrolipoyl dehydrogenase"
                     /translation="MNEYDITVIGSGPGGYIAAIRAAQLGFKTAIVEKEKNLGGICLN
                     WGCIPTKSLLRASEVYRLIKRSKEFGIEVKGANFDIQSIVKYSRNVVDKLSSGVAYLM
                     KKNNIKVHQGFGKLAGNGTIKVVSDKEEQEIVSKHIILATGVRARNLPGIEADGDLIW
                     NAQHAMTPERLPKSLLIIGSGAIGIEFASFYSTLGVDVTIIEIKSTILPLEDKDISDL
                     AQEIFTKQGIKIYTNSSVKALTKSKDSAQVLLSSGESKEFDKVIVAVGIQANIENIGL
                     ENTKIKLSPSGFIETNEWYETSESNVYAIGDVAGPPCLAHKASHEAVICIEKIAGKNA
                     HALKKECIPNCTYSHPQIASIGLTEEQAIKGGYDIKIGKFHSNFNGKSVALSETEGLV
                     KTIIDKKTGEILGSHMIGAEVTELISNFALAKQLEGTDFDIKSTIFPHPTISEMIHES
                     VLAADVK"
                     /besthit="qcoverage 100 , hcoverage 100 , similarity 100
                     , identity 100 , evalue 0.0 , alnlength 457 , Bacteria ,
                     Proteobacteria , WP_015589121.1 MULTISPECIES:
                     dihydrolipoyl dehydrogenase [unclassified Wolbachia]"
     gene            complement(5254..5646)
                     /locus_tag="wEsol_00027"
     CDS             complement(5254..5646)
                     /locus_tag="wEsol_00027"
                     /codon_start=1
                     /transl_table=11
                     /fullproduct="qcoverage 100 , hcoverage 100 , similarity
                     100 , identity 100 , evalue 1.14e-80 , alnlength 130 ,
                     Bacteria , Proteobacteria , WP_006279962.1 MULTISPECIES:
                     hypothetical protein [Wolbachia]"
                     /translation="MIVELKMPKSNSKKEPNEKNKPQEQREGRSGGFLELLKNIIKIL
                     FNSVVDLPEHEQHNKMKSTNLQQGLDTKGKGPEDLHPKAKLEVKEAAKGLKEELQNSD
                     AGGNQSIETKVPNQTTTQDIDNSKTMER"
                     /product="hypothetical protein"
                     /besthit="qcoverage 100 , hcoverage 100 , similarity 100
                     , identity 100 , evalue 1.14e-80 , alnlength 130 ,
                     Bacteria , Proteobacteria , WP_006279962.1 MULTISPECIES:
                     hypothetical protein [Wolbachia]"
     gene            5716..6747
                     /locus_tag="wEsol_00028"
     CDS             5716..6747
                     /locus_tag="wEsol_00028"
                     /codon_start=1
                     /transl_table=11
                     /fullproduct="qcoverage 100 , hcoverage 100 , similarity
                     99.7 , identity 99.7 , evalue 3.75e-239 , alnlength 343 ,
                     Bacteria , Proteobacteria , WP_015589119.1 MULTISPECIES:
                     undecaprenyldiphospho-muramoylpentapeptide
                     beta-N-acetylglucosaminyltransferase [Wolbachia]"
                     /cog="MurG COG0707 357 UDP-N-acetylglucosamine:LPS
                     N-acetylglucosamine transferase"
                     /pfam="Glyco_transf_28 Glyco_trans_4_4 Glyco_trans_1_3
                     Glyco_tran_28_C"
                     /tigr="TIGR01133 murG 353
                     undecaprenyldiphospho-muramoylpentapeptide
                     beta-N-acetylglucosaminyltransferase"
                     /product="undecaprenyldiphospho-muramoylpentapeptide
                     beta-N-acetylglucosaminyltransferase"
                     /translation="MDIILATGGTGGHIFPAIALAKALKTQGYNCILFTDKKTNKNTD
                     IESYTLPLRRPSSNKFKFFLFLIYSSMLALYQVRKLKPKSVIGFGSYASFPTLLAARV
                     LSIPIILHEQNTVLGRVNRFFFKSAKLIATSFPETKYAEGNKCIFTGNFVDIKAQSHS
                     STEKILNILVIAGSQGANFFDDVVSRVICDLPIKMKKKIRVTQQCTKKNVNKVKSLYK
                     SEKIDCELSEFFDDMENRLANAHLVISRAGATSIAEITLARRSAIYIPYPYSKDNHQF
                     YNAKYIEDSRAAIIVEQNSEAKKNLTEVLFDLLNNSQKLRDMTNSTEKTGIKNGTTEF
                     VKVIVHRFS"
                     /besthit="qcoverage 100 , hcoverage 100 , similarity 99.7
```

, identity 99.7 , evalue 3.75e-239 , alnlength 343 ,
Bacteria , Proteobacteria , WP_015589119.1 MULTISPECIES:
undecaprenyldiphospho-muramoylpentapeptide
beta-N-acetylglucosaminyltransferase [Wolbachia]"
gene complement(6732..7661)
/locus_tag="wEsol_00029"
CDS complement(6732..7661)
/locus_tag="wEsol_00029"
/codon_start=1
/transl_table=11
/fullproduct="qcoverage 100 , hcoverage 100 , similarity
100 , identity 100 , evalue 2.21e-223 , alnlength 309 ,
Bacteria , Proteobacteria , WP_015589118.1 MULTISPECIES:
glutathione synthase [Wolbachia]"
/cog="RimK COG0189 318 Glutathione synthase/Ribosomal
protein S6 modification enzyme (glutaminyl transferase)"
/pfam="GSH-S_N GSH-S_ATP ATPgrasp_YheCD RimK"
/tigr="TIGR01380 glut_syn 312 glutathione synthase"
/product="glutathione synthase"
/translation="MKIAFQMDENINFETDTTFVLIKEAQRRKHEVFVYAPNNLALKL
NHPIALAQKVSVDDFSFISKEDVAINLNEMDIIFIRQDPPFDMRYITTTYILEKTSAL
VINNPTEIRNCPEKLITSLFPELIPPTLITENISMIRDFYYNYQDIILKPLYSYGGND
VIRIQDENSIQVVVDLMIAKYECPVIAQSFCKNIDKDKRILLLYGQPIGVMKRIPKIS
GEIRTNMRLGASFEPAQMNDRNNEICNKIGPELKKRGLIFVGIDIIDDFLLEINTTSP
TGVVYINKLYNISLEKDLWDVFEEKASSHQLNL"
/besthit="qcoverage 100 , hcoverage 100 , similarity 100
, identity 100 , evalue 2.21e-223 , alnlength 309 ,
Bacteria , Proteobacteria , WP_015589118.1 MULTISPECIES:
glutathione synthase [Wolbachia]"
gene complement(7722..8318)
/locus_tag="wEsol_00030"
CDS complement(7722..8318)
/locus_tag="wEsol_00030"
/codon_start=1
/transl_table=11
/fullproduct="qcoverage 100 , hcoverage 100 , similarity
100 , identity 100 , evalue 1.02e-125 , alnlength 198 ,
Bacteria , Proteobacteria , WP_006279461.1 MULTISPECIES:
BON domain-containing protein [Wolbachia]"
/cog="OsmY COG2823 196 Predicted periplasmic or secreted
lipoprotein"
/pfam="BON BON"
/product="BON domain-containing protein"
/translation="MRLITSFLLAIFLITQSGCTTLIIGGVVATATATAITMQDKSLG
NIIDDTTMVIRINKGLLKHGLFSSIKVKVSEGRVLLIGSVDTPEKQLTAEKIAWQQKE
IKEVINEIRVIPIQMASMLDTTVDGMITAEIRTRLLGKRNIKSINYSVNTVDRVVYLM
GIAQNKAELKAVIAIARKVKGVKQVVSYVRYRYSKLRH"
/besthit="qcoverage 100 , hcoverage 100 , similarity 100
, identity 100 , evalue 1.02e-125 , alnlength 198 ,
Bacteria , Proteobacteria , WP_006279461.1 MULTISPECIES:
BON domain-containing protein [Wolbachia]"
gene 8465..8623
/locus_tag="wEsol_00031"
CDS 8465..8623
/locus_tag="wEsol_00031"
/codon_start=1
/transl_table=11
/product="hypothetical protein"
/translation="MAELVDVPGLGPGELARGGSSPSIRTLKSCNLGFKGKFIITKYT
FVCVKTCS"

```
gene            8696..10033
                /locus_tag="wEsol_00032"
CDS             8696..10033
                /locus_tag="wEsol_00032"
                /codon_start=1
                /transl_table=11
                /fullproduct="qcoverage 99.7752808988764 , hcoverage 100
                , similarity 100 , identity 100 , evalue 7.48e-311 ,
                alnlength 444 , Bacteria , Proteobacteria ,
                WP_015589117.1 MULTISPECIES: trigger factor [unclassified
                Wolbachia]"
                /cog="Tig COG0544 441 FKBP-type peptidyl-prolyl cis-trans
                isomerase (trigger factor)"
                /pfam="Trigger_N FKBP_C Trigger_C"
                /tigr="TIGR00115 tig 410 trigger factor"
                /product="trigger factor"
                /translation="MMSNNIPQNTVEVSSVYTYKELSIDKLKYEYEITVGSDYIKQKV
                NSRLQEIAENAKSPGFRAGKMPYDLVVANYKNEALEYVINNTIDYCSSDLMKKIEVKS
                HIYPKVDVISLPDLGKENEEGNFVYKLSFESMPEVPMIDLDKINLKKIEAKIEEEDIK
                EFIDSIKTKFPNFASVDDASYQAKDGDKLIIDFEGRIRNKLFQGGSSKDFAVNLGSGT
                FINGFEDQLTGMKKGETKNFKLKFPENYQAISLAGQEADFSVRVNEIQIAKDFENDDE
                IAKSIGFKDYSLLINHAKKMIGDQCTEMRNLLIKKELFDYLDANYSFDLPTDIVKQEQ
                QRMEGELGAQNDSRKEAEKRVKLAMLFMKFSAEHKISLTQNDVLSVIVNQYVSKDVQF
                DRVIKHFESNKQFQELVRGQALEYKVTDYIIEKVSKEEQIVSVKELKELFDNI"
                /besthit="qcoverage 99.7752808988764 , hcoverage 100 ,
                similarity 100 , identity 100 , evalue 7.48e-311 ,
                alnlength 444 , Bacteria , Proteobacteria ,
                WP_015589117.1 MULTISPECIES: trigger factor [unclassified
                Wolbachia]"
gene            10045..10671
                /locus_tag="wEsol_00033"
CDS             10045..10671
                /locus_tag="wEsol_00033"
                /codon_start=1
                /transl_table=11
                /fullproduct="qcoverage 100 , hcoverage 100 , similarity
                100 , identity 100 , evalue 9.15e-143 , alnlength 208 ,
                Bacteria , Proteobacteria , WP_006280254.1 MULTISPECIES:
                ATP-dependent Clp endopeptidase proteolytic subunit ClpP
                [Wolbachia]"
                /cog="ClpP COG0740 200 Protease subunit of ATP-dependent
                Clp proteases"
                /pfam="CLP_protease Pfam-B_306"
                /tigr="TIGR00493 clpP 192 ATP-dependent Clp
                endopeptidase, proteolytic subunit ClpP"
                /product="ATP-dependent Clp
                endopeptidase proteolytic subunit ClpP"
                /translation="MTLIPIVVEQTSRGERAYDIYSRLVKERIIFVTGPIEDNMASVI
                VAQLLFLESENPDKDICMYINSPGGVVTAGLSIYDTMQYINPDVSTLCIGQAASMGSL
                LLAAGTKGKRYSLPHSRIMIHQPSGGYHGQATDIEIHANEILRVKKKLNQIYEKHTGN
                SLKKIEGMMERDKFMDPEEARKIGLIDRVIAERTDIEIENIKVKQKVG"
                /besthit="qcoverage 100 , hcoverage 100 , similarity 100
                , identity 100 , evalue 9.15e-143 , alnlength 208 ,
                Bacteria , Proteobacteria , WP_006280254.1 MULTISPECIES:
                ATP-dependent Clp endopeptidase proteolytic subunit ClpP
                [Wolbachia]"
gene            10671..11948
                /locus_tag="wEsol_00034"
CDS             10671..11948
                /locus_tag="wEsol_00034"
```

/codon_start=1
/transl_table=11
/fullproduct="qcoverage 100 , hcoverage 100 , similarity 100 , identity 100 , evalue 1.86e-301 , alnlength 425 , Bacteria , Proteobacteria , WP_006279985.1 MULTISPECIES: ATP-dependent Clp protease ATP-binding subunit ClpX [Wolbachia]"
/cog="ClpX COG1219 408 ATP-dependent protease Clp, ATPase subunit"
/pfam="zf-C4_ClpX MCM PhoH DEAD Sigma54_activat T2SE RuvB_N AAA_2 IstB_IS21 AAA_22 Mg_chelatase AAA_14 AAA_5 AAA AAA_17 ClpB_D2-small"
/tigr="TIGR00382 clpX 414 ATP-dependent Clp protease, ATP-binding subunit ClpX"
/product="ATP-dependent Clp protease ATP-binding subunit ClpX"
/translation="MDNNNDLHYCSFCNKAQDEVDKLITNSSDGLKVFICNECIELSH
KAISQKKDRSFNSDRISDMKLLLKKPEDIKNFLSKHVVGQEHAQHVLSVAMYNHCQSM
VQFHAISDIEIEKSNIMLIGPTGSGKTLLAKTLAKVSDVPFAMADATTLTEAGYVGDD
VESVLSRLLQAANYDVVKAQRGIVFIDEIDKITRKSESTSITRDVSGEGVQQALLKIM
EGTVAYVPPQGGRKHPQQEFIQVDTSNILFICGGAFEGLDKIIEARKKGTSVGFGADI
SQSKEQKKKNALHDVQPEDLIKFGLIPEFVGRVPITAVLDELDHEDLIHVLTEPRNAL
IKQYKALLAFSKVNLEFSDEAISAIAKKAISYKTGARMLRAILESLLLDIMYTSGNGG
FEGSTIVITKKMVELGKATVNHNNNGNVITVND"
/besthit="qcoverage 100 , hcoverage 100 , similarity 100 , identity 100 , evalue 1.86e-301 , alnlength 425 , Bacteria , Proteobacteria , WP_006279985.1 MULTISPECIES: ATP-dependent Clp protease ATP-binding subunit ClpX [Wolbachia]"

gene 11964..14417
/locus_tag="wEsol_00035"

CDS 11964..14417
/locus_tag="wEsol_00035"
/codon_start=1
/transl_table=11
/fullproduct="qcoverage 100 , hcoverage 100 , similarity 100 , identity 100 , evalue 0.0 , alnlength 817 , Bacteria , Proteobacteria , WP_141456951.1 MULTISPECIES: endopeptidase La [unclassified Wolbachia]"
/cog="Lon COG0466 782 ATP-dependent Lon protease, bacterial type"
/pfam="LON IstB_IS21 AAA_PrkA RuvB_N AAA_16 AAA_25 AAA_14 AAA_22 AAA_17 AAA AAA_5 RNA_helicase AAA_24 AAA_3 AAA_2 Lon_C ChlI"
/tigr="TIGR00763 lon 795 ATP-dependent protease La"
/product="endopeptidase La"
/translation="MNIERAVSSSFTALPVLPLRDVVIFPNIMVPLFIGREKSVNALE
YAISSSNHQNEIFLVAQKDGSVDNPEPEDLYEVGVLASIVQPLIKLPDNAVKVIIRGI
RRGRVVEYISSHTLLQARVELDNHYKEDEDNIDLEALRRSVVDAFDSWCKLNKKNQPE
VAINSIDQIKEVDQLVDTVASHLNIKVSDKQSILEAYDPEERLKKAFAFIEREMSILN
AQNRLYKTIKSQVESTQKVYYLNEQLKAIQKELGEFENGDEGNILNEFEKKINETKLS
QEAREKAITDLKRYKKMNPISPEATVISSYLHWLLELPWGKYKDAKINLNAAKKILDE
NHYGIEKVKDRIIEFLAVLKRVKEIKGPILCLVGPPGVGKTSLAKSMARAVGRDFVRI
ALGGVRDESEIRGHRKTYIGSMPGKIIQHMKKANSCNPLFLLDEIDKMGSDSRGDPAS
ALLEVLDTEHNKHFTDHYLEVEFDLSSVMFVATANSLNLPHPLRDRMEIIQLSGYTED
EKISIATHHLIPKLKKEHGLYQKEWDITNEALYELIRLYTRESGVRSMERELAKLMRK
AVKEILTDKNKKISVGVDNLQDYLGVRKYTFGIAENESLVGIVTGLAYTETGGDILMI
ESVLIPGKGEIKYTGKLGEVMQESIKAAYSYIRSNCLFFGIKPEKFQNNDIHLHVPEG
AVPKDGPSAGGAVCTSIVSLMTNIPVNKNVAMTGEVTLRGRVLAIGGLREKLLAALRG
SIKTVIIPSENEKDMQEIPASIKEGINVVFAKNIDEVMKVALMHPTTPIDGDDNQNSV

```
                     PSSIESKDDTFPMSETLKH"
                     /besthit="qcoverage 100 , hcoverage 100 , similarity 100
                     , identity 100 , evalue 0.0 , alnlength 817 , Bacteria ,
                     Proteobacteria , WP_141456951.1 MULTISPECIES:
                     endopeptidase La [unclassified Wolbachia]"
     gene            complement(14414..14521)
                     /locus_tag="wEsol_00036"
     CDS             complement(14414..14521)
                     /locus_tag="wEsol_00036"
                     /codon_start=1
                     /transl_table=11
                     /product="hypothetical protein"
                     /translation="MNDITTKQVISLWDAQVGGFLPDKREMEIDNIFLI"
                     /besthit="qcoverage 80 , hcoverage 71.7948717948718 ,
                     similarity 96.4 , identity 89.3 , evalue 5.63e-10 ,
                     alnlength 28 , Bacteria , Proteobacteria , RLT59764.1 ABC
                     transporter ATP-binding domain protein [Wolbachia
                     endosymbiont of Drosophila ananassae]"
     gene            14701..15996
                     /locus_tag="wEsol_00037"
     CDS             14701..15996
                     /locus_tag="wEsol_00037"
                     /codon_start=1
                     /transl_table=11
                     /fullproduct="qcoverage 100 , hcoverage 100 , similarity
                     99.8 , identity 99.8 , evalue 3.12e-287 , alnlength 431 ,
                     Bacteria , Proteobacteria , WP_141456953.1 MULTISPECIES:
                     sodium:proton antiporter [unclassified Wolbachia]"
                     /cog="NhaC COG1757 485 Na+/H+ antiporter"
                     /pfam="CitMHS Na_H_antiporter DUF1686 Na_H_antiporter"
                     /tigr="TIGR00931 antiport_nhaC 454 Na+/H+ antiporter
                     NhaC"
                     /product="sodium:proton antiporter"
                     /translation="MFYFTNFLPILLFLFLFVGSGIYFTVLGVSNAFYQLSPLVAIVP
                     AIALGWVLYKGNTEEKMYAFLDGVRDRNIITMCIIFLLAGAFSEVTKAIGSADATVNL
                     ALSFIPTQFLLIGLFFVAAFISTAIGTSMGTIATIAPIAVELTQAGISPSLGVATVVG
                     GAMFGDNLSIISDTTIASVMSQKADIKKKLKLNAKVALIASIFTIIILFYSSSNSAAI
                     SVKEYSLLLVTPYIILILLANFGINVFTALVLSITFAGIIGFINNNNYGLLFLSQNIT
                     KGFMSMHEIMLLSLMVGGLSGLVGKNSKELADKLSSWVMIQRGGQKTAQLFIAKIVSI
                     FDILLANNTIAIIFSGEIARDIAKKYEIPPHYSVAWLGIFSCVFQGLIPYGAQLLLAS
                     TIAGVSPLAVVPHVYYCYVLGIIAVLYIVLNKIEKKDCA"
                     /besthit="qcoverage 100 , hcoverage 100 , similarity 99.8
                     , identity 99.8 , evalue 3.12e-287 , alnlength 431 ,
                     Bacteria , Proteobacteria , WP_141456953.1 MULTISPECIES:
                     sodium:proton antiporter [unclassified Wolbachia]"
     gene            15989..17281
                     /locus_tag="wEsol_00038"
     CDS             15989..17281
                     /locus_tag="wEsol_00038"
                     /codon_start=1
                     /transl_table=11
                     /pfam="Pfam-B_19638 COX1 Pfam-B_19638 Pfam-B_19638"
                     /fullproduct="qcoverage 100 , hcoverage 100 , similarity
                     100 , identity 100 , evalue 1.66e-302 , alnlength 430 ,
                     Bacteria , Proteobacteria , WP_015589113.1 cbb3-type
                     cytochrome c oxidase subunit I [Wolbachia endosymbiont of
                     Drosophila simulans]"
                     /translation="MLNIQDNAPLCRKWMFLAVCALACSGLLSIIVIFLRLPFISSLV
                     PSAQYIFDNALVIHVNLSILVWMCSIISLLFIINLQNTNSWFNFSWVLSTLSMLLMFI
                     SAFVPNTEVIKSNYIPVLQNKLFLFGLSLFIASILVNTMLAYTSKKEVSFLSVGQIGL
```

```
                     VIILVSSFLCFVLAYNNMPPGLYHSDNNLFYEYLFWGGGHLLQFAFTQGMFLVYLMIS
                     DVSLASSNKITILPLFINTILAVGAPFVYFVYPVDSAKLIQFFTWHMRIAGAVLPCFL
                     IILVCCNIRTFINDKSNYLLHSFLLFTYGDVLGVLTIEGNVTIPAHYHGSVVGITIAF
                     MNFIYWLLPKLNFKEIKGSMVRLQIYAYSIGHFLHITGLVWLGGYGALRKVADLPSIS
                     SMLARTCFIIGGAISVVGGMLFVIIVLLHLLKGKARTN"
                     /product="cbb3-type cytochrome c oxidase subunit I"
                     /besthit="qcoverage 100 , hcoverage 100 , similarity 100
                     , identity 100 , evalue 1.66e-302 , alnlength 430 ,
                     Bacteria , Proteobacteria , WP_015589113.1 cbb3-type
                     cytochrome c oxidase subunit I [Wolbachia endosymbiont of
                     Drosophila simulans]"
     gene            18567..20306
                     /locus_tag="wEsol_00039"
     CDS             18567..20306
                     /locus_tag="wEsol_00039"
                     /codon_start=1
                     /transl_table=11
                     /fullproduct="qcoverage 100 , hcoverage 100 , similarity
                     100 , identity 100 , evalue 0.0 , alnlength 579 ,
                     Bacteria , Proteobacteria , WP_010962506.1 MULTISPECIES:
                     single-stranded-DNA-specific exonuclease RecJ
                     [Wolbachia]"
                     /cog="RecJ COG0608 491 Single-stranded DNA-specific
                     exonuclease"
                     /pfam="DHH DHHA1"
                     /tigr="TIGR00644 recJ 546 single-stranded-DNA-specific
                     exonuclease RecJ"
                     /product="single-stranded-DNA-specific exonuclease RecJ"
                     /translation="MLLEIEKRSITNALWKLQEADQRQILTLTQRFELPEILARILVT
                     RGVNIENASDFLYPLIRSLLPDPFHLLDMDKAVSRIIRAIKNDENLAIFGDYDVDGAT
                     SSALINRYLRAIDIHSIIYIPDRVDEGYGLNTDALLQLKKNGIDLCISVDCGTLAYQP
                     IEDAKAFGLDIIVVDHHLGTEKLPSAVAVVNPNRLDESSPYNNLAAVGVSFLLIVALN
                     KSLREQGFFTNKKEPDLFDLLDLVALGTVCDVMQITGLNRALVSQGLKVMSTRKNVGL
                     RVLFDALGILEKPSVSRLGFSIGPCINAGGRIGEASLGARLLSTDDDEEAHSIALKLI
                     DLNNARKVLENEALLEATAQAEKSAQSGANFIMISGNWHQGIIGIIASRLKEQFHLPT
                     VVISLNNGIGKASCRSILGVDIGAAVLSAKFINLIIEGGGHSMAAGFSIKEDKINDLH
                     DFFTERFSNSINEKTLKADSIVTAKAINLSLWNQLQRLGPFGVGNPEPRFIIQGAKIR
                     KPEVIGVDHIKCFIADDNVMVRAIAFRSANTHLGSAIMQGNVKAILGKISMNYWNGNE
                     FVQFLIEDVLTIS"
                     /besthit="qcoverage 100 , hcoverage 100 , similarity 100
                     , identity 100 , evalue 0.0 , alnlength 579 , Bacteria ,
                     Proteobacteria , WP_010962506.1 MULTISPECIES:
                     single-stranded-DNA-specific exonuclease RecJ
                     [Wolbachia]"
     gene            complement(21133..21939)
                     /locus_tag="wEsol_00040"
     CDS             complement(21133..21939)
                     /locus_tag="wEsol_00040"
                     /codon_start=1
                     /transl_table=11
                     /fullproduct="qcoverage 100 , hcoverage 100 , similarity
                     100 , identity 100 , evalue 9.12e-191 , alnlength 268 ,
                     Bacteria , Proteobacteria , WP_015589111.1 MULTISPECIES:
                     folate-binding protein YgfZ [unclassified Wolbachia]"
                     /cog="COG0354 COG0354 305 Predicted
                     aminomethyltransferase related to GcvT"
                     /pfam="GCV_T GCV_T_C"
                     /product="folate-binding protein YgfZ"
                     /translation="MSYIPFLSRGVIVLYGPDTRDFLQGIITNDINKLDSQKAIYSLL
                     LSPQGKYLYDFFLIEYGKYTLLECENMHLQQIIEKLDLLKTYLRVKIKDVSALYKVGV
```

```
                     LFNTKLAECSGKSQVIFQDPRHKLLGMRIIHKDEIKEPVGDFTQYEKVRIQNLVPDGA
                     KDMVQNSSFPLQFLIDKVNGISFNKGCYIGQEVVNRMSRQEIFRRKLYLVEGDNALPD
                     IGTKVTNENNEEIGELRSSVDNIGFALLNTGKSHANLYADGVRCVKTLKS"
                     /besthit="qcoverage 100 , hcoverage 100 , similarity 100
                     , identity 100 , evalue 9.12e-191 , alnlength 268 ,
                     Bacteria , Proteobacteria , WP_015589111.1 MULTISPECIES:
                     folate-binding protein YgfZ [unclassified Wolbachia]"
     gene            22117..22407
                     /locus_tag="wEsol_00041"
     CDS             22117..22407
                     /locus_tag="wEsol_00041"
                     /codon_start=1
                     /transl_table=11
                     /fullproduct="qcoverage 100 , hcoverage 100 , similarity
                     100 , identity 100 , evalue 7.12e-59 , alnlength 96 ,
                     Bacteria , Proteobacteria , WP_007549140.1 MULTISPECIES:
                     co-chaperone GroES [Wolbachia]"
                     /cog="GroS COG0234 96 Co-chaperonin GroES (HSP10)"
                     /pfam="Cpn10"
                     /product="co-chaperone GroES"
                     /translation="MSSISLNVLDDSVLIKPISEEKQGGIVLPSSAEKKPTKGEVIAI
                     GEGSRNSSGERVTLTVKAGDKVFYRQWAGTEIEHNDEKLIVMKESDILAVIK"
                     /besthit="qcoverage 100 , hcoverage 100 , similarity 100
                     , identity 100 , evalue 7.12e-59 , alnlength 96 ,
                     Bacteria , Proteobacteria , WP_007549140.1 MULTISPECIES:
                     co-chaperone GroES [Wolbachia]"
     gene            22508..24157
                     /locus_tag="wEsol_00042"
     CDS             22508..24157
                     /locus_tag="wEsol_00042"
                     /codon_start=1
                     /transl_table=11
                     /fullproduct="qcoverage 100 , hcoverage 100 , similarity
                     100 , identity 100 , evalue 0.0 , alnlength 549 ,
                     Bacteria , Proteobacteria , WP_141456977.1 MULTISPECIES:
                     chaperonin GroEL [unclassified Wolbachia]"
                     /cog="GroL COG0459 524 Chaperonin GroEL (HSP60 family)"
                     /pfam="Cpn60_TCP1"
                     /tigr="TIGR02348 GroEL 526 chaperonin GroL"
                     /product="chaperonin GroEL"
                     /translation="MTNVVVSGEQLQEAFREVAAIVDSTVAITAGPRGKTVGINKPYG
                     APEITKDGYKVMKGIKPEKPLNAVIASIFAQSCSQCNDKVGDGTTTCSILTSNMIMEA
                     SKSIAAGNDRVGIKNGIQKAKDVILKEITSMSRTISLEKIDEVAQVAIISANGDKDIG
                     NSIADSVKKVGKEGVITVEESKGSKELEVELTTGMQFDRGYLSPYFITNNEKMIVELD
                     NPYLLITEKKLNIIQPLLPILEAIVKSGKPLVIIAEDIEGEALSTLVINKLRGGLKVA
                     AVKAPGFGDRRKEMLEDIATLTGAKYVIKDELGIKMEDLTLDDLGTAKNVKITKDNTT
                     VVSENSDSDSVKARIEQIKSQIETSTSDYDKEKLRERLAKLSGGVAVLKVGGATEVEV
                     KERRDRVEDALHATRAAIEEGIVPGGGVALLYASSVLDKLKGASDEEQIGINIIKKVL
                     SAPIRRLVKNAGLESAVIIDYLIKQNDKELIYNVEAMNYANAFTAGVIDPAKVVRIAF
                     ETAVSVASVLITTESMIVDVPSKENASSPMGAGEMSGMGGF"
                     /besthit="qcoverage 100 , hcoverage 100 , similarity 100
                     , identity 100 , evalue 0.0 , alnlength 549 , Bacteria ,
                     Proteobacteria , WP_141456977.1 MULTISPECIES: chaperonin
                     GroEL [unclassified Wolbachia]"
     gene            complement(24711..27836)
                     /locus_tag="wEsol_00043"
     CDS             complement(24711..27836)
                     /locus_tag="wEsol_00043"
                     /codon_start=1
                     /transl_table=11
```

```
                     /pfam="AsmA_2 DUF3971 AsmA_2 AsmA_1"
                     /fullproduct="qcoverage 100 , hcoverage 100 , similarity
                     99.9 , identity 99.9 , evalue 0.0 , alnlength 1041 ,
                     Bacteria , Proteobacteria , WP_015589123.1 MULTISPECIES:
                     AsmA-like C-terminal domain-containing protein
                     [unclassified Wolbachia]"
                     /translation="MLKKITVLFSVALLFILCFFIFLKPLEININYVNFYVKHKISKI
                     FVGSSVNVENISVVWQKGSKDPYLVITDLAIANPNFTIKVPELFVHFKLSSLFKGNIK
                     FSQVLADNVRVYIKREEINVNPQNSLDLLDNHYLSNNRLPSVSFQRVTLESRKKETSS
                     QATWMTGKKDIGMTGKKDTGMTGKNFKMTESSAKNLLKTIREFFFDLNADSKIEFTNI
                     AINKGTEDEFFIDKLYIGKGEDFNVLDIHVNTKDEKGFLDDLSITIKNRNNLLNVYGT
                     FYDLKLGLLSEFSTLVKSYNLDKNIGFKGSFSVKINKKDEIVDGNIYVLNTENYLNKN
                     LALTNVNINLTYSNGIISVKNFHFKLNDTYLSLIGKMNFGTSHALLRINISKFAAKDL
                     CTYIPDSVVNSKFKSWYCDNIDGDVLNTIVSFNGKINNLVDDDLSDIVIVADIENGSV
                     KFDEDFEQVKELKGDLIIKNNDLEITVNSAKFQNFTVNGGDIEMKSLNKEDSVLTING
                     KAVSDAYGLYEPIRFKLDDVVKVERDKVSGMAKSVFNFRIFNLNADDKKVDFSANFHS
                     EIDNLAVYNASLGKYDIKLSFGSDFIDLNGSGMVNNTQLLFDLKSSNRNESFAWNLTG
                     DLPAQIFNFDSGYVSANLESVINQDKTGYVNGDIDLSEFESHSSYLGWKNRFEDHNKI
                     LFSTRLKGAGELLVDKLDIVGNDLDIKFSGRVENGNLYLNSSSFKLPDNDFSIEIESG
                     KEKNAITIYGEEINLSDILGLLGKNSNGLNKDIEISMNVDNVIMKEGIVIKNAKLNVT
                     CTKGNCNGSQFTGQFLEDSSNILAEYSGIGLEIYADNSGMLLRSLGISKSIKNGKLSF
                     YLSPQRESGEHYGTLSISNFYIKDAPLLTTLLSMSSLPGIVNAIKNEGVYFYKCNAPF
                     SYKDGTIEIEESWLEGAELGISTGGKLDIRNYKFQVAGQVIPAYSINKSLLKIPIIGK
                     LLTGGKSRGIVSIDYKASGDDKNNNVSVDLISSLTPNLLKRLLGVFDRIMTKTNESGL
                     KGGVKKKFGSI"
                     /product="AsmA-like C-terminal domain-containing protein"
                     /besthit="qcoverage 100 , hcoverage 100 , similarity 99.9
                     , identity 99.9 , evalue 0.0 , alnlength 1041 , Bacteria
                     , Proteobacteria , WP_015589123.1 MULTISPECIES: AsmA-like
                     C-terminal domain-containing protein [unclassified
                     Wolbachia]"
     gene            complement(27829..28266)
                     /locus_tag="wEsol_00044"
     CDS             complement(27829..28266)
                     /locus_tag="wEsol_00044"
                     /codon_start=1
                     /transl_table=11
                     /fullproduct="qcoverage 100 , hcoverage 100 , similarity
                     100 , identity 100 , evalue 9.71e-105 , alnlength 145 ,
                     Bacteria , Proteobacteria , WP_015589124.1 MULTISPECIES:
                     ribonuclease HI [unclassified Wolbachia]"
                     /cog="RnhA COG0328 154 Ribonuclease HI"
                     /pfam="RNase_H"
                     /product="ribonuclease HI"
                     /translation="MSKKEVTIYTDGACSGNPGAGGWAAIILFQDYRKDIYGREENTT
                     NNKMELTAVINGLKVLKFSCNINLYTDSLYVKHGITEWINKWKMNGWKTSNKKSVKNM
                     ELWKELDNVALQHEIDWKWVKAHSGDKYNEEADSLARKAIINA"
                     /besthit="qcoverage 100 , hcoverage 100 , similarity 100
                     , identity 100 , evalue 9.71e-105 , alnlength 145 ,
                     Bacteria , Proteobacteria , WP_015589124.1 MULTISPECIES:
                     ribonuclease HI [unclassified Wolbachia]"
     gene            complement(28443..29330)
                     /locus_tag="wEsol_00045"
     CDS             complement(28443..29330)
                     /locus_tag="wEsol_00045"
                     /codon_start=1
                     /transl_table=11
                     /fullproduct="qcoverage 100 , hcoverage 100 , similarity
                     100 , identity 100 , evalue 2.00e-203 , alnlength 295 ,
                     Bacteria , Proteobacteria , WP_015589125.1 MULTISPECIES:
```

```
                     heme o synthase [unclassified Wolbachia]"
                     /cog="CyoE COG0109 304 Polyprenyltransferase (cytochrome
                     oxidase assembly factor)"
                     /pfam="UbiA"
                     /tigr="TIGR01473 cyoE_ctaB 281 protoheme IX
                     farnesyltransferase"
                     /product="heme o synthase"
                     /translation="MYTSVLLNVESTILDFWRLLKPRIMYLVVFTAVAGMVAAPGGMH
                     PFLALISLICVALGSGSAGAINMWYDRDIDLLMERTKNRPIPSGRVSAESALEFGITL
                     GILSVFIMAIAVNYISAALLAISILFYVFVYTIWLKRRTPQNIVIGGAAGAFPPMIGW
                     AAVTNSVSWESFILFLVIFMWTPPHFWALSLNKSEDYVKASIPMFNIVYGPKKTRKYI
                     LIYSVLLVLTSLLPALFLKKALLYLSMATFEGCVFIWYAISILRFKNHSSQKKMFSYS
                     ISYLFSLFASIIFCSIDLF"
                     /besthit="qcoverage 100 , hcoverage 100 , similarity 100
                     , identity 100 , evalue 2.00e-203 , alnlength 295 ,
                     Bacteria , Proteobacteria , WP_015589125.1 MULTISPECIES:
                     heme o synthase [unclassified Wolbachia]"
     gene            complement(29332..30882)
                     /locus_tag="wEsol_00046"
     CDS             complement(29332..30882)
                     /locus_tag="wEsol_00046"
                     /codon_start=1
                     /transl_table=11
                     /fullproduct="qcoverage 100 , hcoverage 100 , similarity
                     100 , identity 100 , evalue 0.0 , alnlength 516 ,
                     Bacteria , Proteobacteria , WP_015589126.1 MULTISPECIES:
                     cytochrome c oxidase subunit I [unclassified Wolbachia]"
                     /cog="CyoB COG0843 566 Heme/copper-type cytochrome/quinol
                     oxidases, subunit 1"
                     /pfam="Pfam-B_15059 COX1 Pfam-B_523"
                     /tigr="TIGR02891 CtaD_CoxA 504 cytochrome c oxidase,
                     subunit I"
                     /product="cytochrome c oxidase subunit I"
                     /translation="MSDVPKGIKRWLFSTNHKDIGTLYIIFSILAGIIGGLLSVIIRT
                     QLMHINILNNNYQLYNVMVTGHALIMVFFMIMPALMGGFGNWFVPLMIGAPDMAFPRM
                     NNLSFWLLVSSFILLILSVFIGEGPGTGWTLYPPLSQVMSHPSAGVDIAILALHVAGM
                     SSIVGAINFIVTIFNMRAKGMSLTKMPLFVWSVLLTAFMLIVALPVLAGAITMLLTDR
                     NIGTSFFDPAGGGDPVLFQHLFWFFGHPEVYVIIFPAFGIISQVVSTFSHRPVFGYIG
                     MVYAMIGIAVFGFMVWAHHMFTVGLSADAAAFFSTTTIFIGVITGVKVFSWIATMWGG
                     AIEFKTPMLFALGFIFMFVGGGITGIILSHGGIDKLLHDTYYVVAHFHYVMSLAALFG
                     AFAGFYYWIGKMSGKQYNERLGQIHFWLTFISTNITFLPQHFLGLAGMPRRIPDYPDA
                     FIPWNYISSIGSYMSFVSVMFFVFIVIHLFKWGKKAGDNPWKGDTLEWTVSSPPPFHT
                     FEKPPVVK"
                     /besthit="qcoverage 100 , hcoverage 100 , similarity 100
                     , identity 100 , evalue 0.0 , alnlength 516 , Bacteria ,
                     Proteobacteria , WP_015589126.1 MULTISPECIES: cytochrome
                     c oxidase subunit I [unclassified Wolbachia]"
     gene            complement(30898..31662)
                     /locus_tag="wEsol_00047"
     CDS             complement(30898..31662)
                     /locus_tag="wEsol_00047"
                     /codon_start=1
                     /transl_table=11
                     /fullproduct="qcoverage 100 , hcoverage 100 , similarity
                     100 , identity 100 , evalue 4.16e-180 , alnlength 254 ,
                     Bacteria , Proteobacteria , WP_015589127.1 MULTISPECIES:
                     cytochrome c oxidase subunit II [unclassified Wolbachia]"
                     /cog="CyoA COG1622 247 Heme/copper-type cytochrome/quinol
                     oxidases, subunit 2"
                     /pfam="COX2_TM Polysacc_synt COX2"
```

```
                     /tigr="TIGR02866 CoxB 202 cytochrome c oxidase, subunit
                     II"
                     /product="cytochrome c oxidase subunit II"
                     /translation="MVKLFALLIIFYSNISVASAPTSWQFGFPAPATEVMEAVVRSHS
                     FVMLVMVTIMLFVWALLAYIAFRFRKSKVTNISKTTHSVPLEIIWFVIPTIIVGILAF
                     ENAKLLKLQEEIPKADITLKVIGHQWYWSYQYPEYQGVSFDSYIKGKEDFIEGDLKLF
                     SVDNNIVLPVNANVRLQVTAGDVIHSWGVPAFGVKIDAIPGRLNEAWFNIKKPGVYYG
                     QCYELCGQGHGFMPIVVEAVSKEDFNKWIENRKLVS"
                     /besthit="qcoverage 100 , hcoverage 100 , similarity 100
                     , identity 100 , evalue 4.16e-180 , alnlength 254 ,
                     Bacteria , Proteobacteria , WP_015589127.1 MULTISPECIES:
                     cytochrome c oxidase subunit II [unclassified Wolbachia]"
     gene            complement(33189..33353)
                     /locus_tag="wEsol_00048"
     CDS             complement(33189..33353)
                     /locus_tag="wEsol_00048"
                     /codon_start=1
                     /transl_table=11
                     /fullproduct="qcoverage 100 , hcoverage 100 , similarity
                     100 , identity 100 , evalue 2.06e-28 , alnlength 54 ,
                     Bacteria , Proteobacteria , WP_167482801.1 hypothetical
                     protein [Wolbachia endosymbiont of Carposina sasakii]"
                     /cog="Tra5 COG2801 232 Transposase and inactivated
                     derivatives"
                     /pfam="rve"
                     /product="hypothetical protein"
                     /translation="MSSSINKQLVMDSLLMDVNKRKPAKNLLLHSDQGSQYTLQGYQY
                     LLSIKNIDES"
                     /besthit="qcoverage 100 , hcoverage 100 , similarity 100
                     , identity 100 , evalue 2.06e-28 , alnlength 54 ,
                     Bacteria , Proteobacteria , WP_167482801.1 hypothetical
                     protein [Wolbachia endosymbiont of Carposina sasakii]"
     gene            complement(33894..34076)
                     /locus_tag="wEsol_00049"
     CDS             complement(33894..34076)
                     /locus_tag="wEsol_00049"
                     /codon_start=1
                     /transl_table=11
                     /fullproduct="qcoverage 85 , hcoverage 89.4736842105263 ,
                     similarity 100 , identity 100 , evalue 7.96e-26 ,
                     alnlength 51 , Bacteria , Proteobacteria , WP_210446103.1
                     transposase, partial [Wolbachia endosymbiont of
                     Drosophila melanogaster]"
                     /cog="COG2963 COG2963 116 Transposase and inactivated
                     derivatives"
                     /pfam="HTH_Tnp_1 HTH_23 HTH_28"
                     /product="transposase, partial"
                     /translation="MAREYTAEFKLEAVKLANEQRKVGQPVAKVARDLGIRDSVLGKW
                     MKKYNEKSQRQMHFQM"
                     /besthit="qcoverage 85 , hcoverage 89.4736842105263 ,
                     similarity 100 , identity 100 , evalue 7.96e-26 ,
                     alnlength 51 , Bacteria , Proteobacteria , WP_210446103.1
                     transposase, partial [Wolbachia endosymbiont of
                     Drosophila melanogaster]"
     gene            complement(34095..34202)
                     /locus_tag="wEsol_00050"
     CDS             complement(34095..34202)
                     /locus_tag="wEsol_00050"
                     /codon_start=1
                     /transl_table=11
```

```
                     /fullproduct="qcoverage 100 , hcoverage 100 , similarity
                     100 , identity 100 , evalue 1.13e-13 , alnlength 35 ,
                     Bacteria , Proteobacteria , AAS14033.1 hypothetical
                     protein WD_0297 [Wolbachia endosymbiont of Drosophila
                     melanogaster]"
                     /translation="MNNKNKSKDRKKEIEFRIDLTQKKWRERKEFFRVK"
                     /product="hypothetical protein"
                     /besthit="qcoverage 100 , hcoverage 100 , similarity 100
                     , identity 100 , evalue 1.13e-13 , alnlength 35 ,
                     Bacteria , Proteobacteria , AAS14033.1 hypothetical
                     protein WD_0297 [Wolbachia endosymbiont of Drosophila
                     melanogaster]"
     gene            complement(34399..34566)
                     /locus_tag="wEsol_00051"
     CDS             complement(34399..34566)
                     /locus_tag="wEsol_00051"
                     /codon_start=1
                     /transl_table=11
                     /product="hypothetical protein"
                     /translation="MKKMSPIDGFRMLLRTLDVLFTSFINPSIKRLKRLANGCYEFGA
                     VGDIKDTLFGV"
     gene            35188..36435
                     /locus_tag="wEsol_00052"
     CDS             35188..36435
                     /locus_tag="wEsol_00052"
                     /codon_start=1
                     /transl_table=11
                     /pfam="Pox_D5 RVT_1 GIIM"
                     /fullproduct="qcoverage 100 , hcoverage 100 , similarity
                     100 , identity 100 , evalue 7.62e-314 , alnlength 415 ,
                     Bacteria , Proteobacteria , WP_155968744.1 group II
                     intron reverse transcriptase/maturase [Wolbachia
                     pipientis]"
                     /translation="MNKTKSFDIPKQLIWKAYKQVSKNRGAAGVDEVSITKFEENLKD
                     NLYKLWNRMSSGSYFPESVKAVAIPKDTGGQRTLCVPSVFDRIGQTAATMYLEPLVEP
                     EFHEDSYGYRPNKSALDAVSTACKRCWRSDWTIDLDISGFFDNLDHVLALQTIKKHTD
                     CKWVILYVERWMKAPIQLADGSKVVRNKGVPQGGSVSPIISNIFMHHAFDMWMRQNYP
                     TIPFERYVDDAMVHCKTQKQAKFIKDKIEERLAEFKLKLHPEKTQIVYCKDDNRRDEF
                     PIQSFDFLGYTFRPRLAKNNKIGNYFVSFLPAISNKAKKKINQTIRSWKIRRQTYTTL
                     EKISKKINPIVRGWYQYYGRFYKSEMYPSLRNVEWHLVGWVRAKYKKLRNHRRLAKQF
                     LAKVRKRSPNIFYHWTLGLGSKG"
                     /product="group II intron reverse transcriptase/maturase"
                     /besthit="qcoverage 100 , hcoverage 100 , similarity 100
                     , identity 100 , evalue 7.62e-314 , alnlength 415 ,
                     Bacteria , Proteobacteria , WP_155968744.1 group II
                     intron reverse transcriptase/maturase [Wolbachia
                     pipientis]"
     gene            36455..36649
                     /locus_tag="wEsol_00053"
     CDS             36455..36649
                     /locus_tag="wEsol_00053"
                     /codon_start=1
                     /transl_table=11
                     /fullproduct="qcoverage 100 , hcoverage 100 , similarity
                     100 , identity 100 , evalue 1.64e-36 , alnlength 64 ,
                     Bacteria , Proteobacteria , WP_213863496.1 hypothetical
                     protein [Wolbachia endosymbiont of Ceratitis capitata]"
                     /translation="MRGLRTVLRETGGEAPPVYSPSGFPIVLSKFLGARNDYSLKRIL
                     ATEKNEDIFIRKKRGKNREN"
                     /product="hypothetical protein"
```

```
                     /besthit="qcoverage 100 , hcoverage 100 , similarity 100
                     , identity 100 , evalue 1.64e-36 , alnlength 64 ,
                     Bacteria , Proteobacteria , WP_213863496.1 hypothetical
                     protein [Wolbachia endosymbiont of Ceratitis capitata]"
     gene            36828..37892
                     /locus_tag="wEsol_00054"
     CDS             36828..37892
                     /locus_tag="wEsol_00054"
                     /codon_start=1
                     /transl_table=11
                     /product="hypothetical protein"
                     /pfam="Ank_2 Ank_4 Ank_5 Ank Ank_3 Ank_2 Ank Ank_3 Ank_5
                     Ank_4 Pfam-B_1873 Ank_5 Ank Ank_3 Ank_4 Ank_2 Ank_5 Ank
                     Ank_4 Ank_3 Ank_5 Ank Ank_3 Ank_2 Ank_5 Ank Ank_4 Ank_3
                     Pfam-B_1714 Ank Ank_3 Ank_5 Ank_4 Pfam-B_10896 Ank_5
                     Ank_4 Ank Ank_3 Ank_2 Ank Ank_3 Ank_5"
                     /translation="MFTNRISNNLDQLQFFVEFLNENYEIQKHLSLTPLHLAAGNGQL
                     DLVNTLLGEGLDINSEIKYDGFTPLYFSIAKNRLEMVNFLIAHGADVNHKTILGFTPL
                     SFASQQGYLDIVNTLIANGADLSTKTDKLNTPLHLAAENGHLDIVNVFIEKGLDVNAV
                     NNDRARPLHSAVQNGNLEVVKALISQGSNINAGSSGIGNHKVDANITPLHLGTQTGRL
                     DIVKVLLEAGANVNAKTDDKITPLHLASQNGFLELVDILLKAKSNVNAKDYENLTPLH
                     LAAERNHFGVVKSLLLVRGIDVNAKDHDNSTALHIGSQNGHLEVVKLLIEKKANVNAK
                     KKRRFYTFASSNSAKSFRGE"
                     /besthit="qcoverage 93.2203389830508 , hcoverage
                     69.7674418604651 , similarity 99.7 , identity 99.7 ,
                     evalue 1.59e-39 , alnlength 330 , Bacteria ,
                     Proteobacteria , WP_179947094.1 ankyrin repeat
                     domain-containing protein, partial [Wolbachia
                     endosymbiont of Bemisia tabaci]"
     gene            37852..39429
                     /locus_tag="wEsol_00055"
     CDS             37852..39429
                     /locus_tag="wEsol_00055"
                     /codon_start=1
                     /transl_table=11
                     /product="hypothetical protein"
                     /pfam="Ank_3 Ank_2 Ank Ank_4 Ank_5 Pfam-B_1873 Ank_5
                     Ank_3 Ank Ank_2 Ank_4 Ank_5 Pfam-B_10896 Ank Ank_3 Ank_4
                     Ank_5 Pfam-B_1873 Ank Ank_4 Ank_3 Ank_2 Ank_5
                     Pfam-B_10896 Ank_4 Ank Ank_3 Ank_5 Ank_4 Ank_3 Ank Ank_5
                     Ank Ank_4 Ank_3 Ank_2 Ank Ank_4 Ank_2 Ank_5 Ank Ank_3
                     Ank_4 Shigella_OspC Pfam-B_1714 Ank_5 Ank Ank_3 Ank_2
                     Ank_5 Ank Ank_3 Ank_4 Ank_2"
                     /translation="MHLAIQQSHFEVSDFLIKNGANINTVDDQNWTPLHNAAYNGFSL
                     KIVESLIAKGANINAKMDDGRRALHLAAEHNRLEIMNFLIENGADINALDNRSWTPLH
                     CAAYDGNLEVAKSLLDKGADINAKTVKSTTPLHFAVDHDHLEVVELLLEKEADINALD
                     HTNWTPLHFAAEKGYDQIATVLLKHGADVNVKENQNKGTALHLAAQYGHPKVVKTLII
                     NGADVNAKMDKNATPLHLGAQIGNLDIVRSLLMSGAYFNARAEGGRYVLPLHFAERRG
                     NPEVIKLLKLVEKLFKAIEDNNYLGIESSIRDGAIIDSKNVDGRTPLHYAVNNGHIKV
                     VNILLTNGANVSQVTNKGNTPLHTATSKCYKEIVEVLLQHISRDKLNDFVNAKTTSSG
                     TTSLHVAAKGGSLEVVKSLLKHGAIYNIKNKEGKAPLDLSRDQNITNLLKLVEELFEN
                     AKNGNVEIISKLKAIKPDERVAVTNARDDQGKSLVQVAVINKHSNLASRLLEILKSPD
                     QSLQDVSVENRVKSLKL"
                     /besthit="qcoverage 63.8095238095238 , hcoverage
                     97.3837209302326 , similarity 86.9 , identity 85.4 ,
                     evalue 2.41e-126 , alnlength 335 , Bacteria ,
                     Proteobacteria , WP_179947054.1 ankyrin repeat
                     domain-containing protein, partial [Wolbachia
                     endosymbiont of Bemisia tabaci]"
     gene            39965..40726
```

```
                     /locus_tag="wEsol_00056"
     CDS             39965..40726
                     /locus_tag="wEsol_00056"
                     /codon_start=1
                     /transl_table=11
                     /fullproduct="qcoverage 100 , hcoverage 100 , similarity
                     95.3 , identity 92.5 , evalue 8.45e-159 , alnlength 253 ,
                     Bacteria , Proteobacteria , WP_197907410.1 NTP
                     transferase domain-containing protein [Wolbachia
                     endosymbiont of Kradibia gibbosae]"
                     /cog="GlmU COG1207 460 N-acetylglucosamine-1-phosphate
                     uridyltransferase (contains nucleotidyltransferase and
                     I-patch acetyltransferase domains)"
                     /pfam="IspD NTP_transf_3 NTP_transferase"
                     /tigr="TIGR01173 glmU 452 UDP-N-acetylglucosamine
                     diphosphorylase/glucosamine-1-phosphate
                     N-acetyltransferase"
                     /product="NTP transferase domain-containing protein"
                     /translation="MDNTLIILAAGKSSRMNSEYPKALHQVGNLTLLEHIISNAKLLS
                     LKSLSIVVNNSLLKDLEKFDTLKSIIDQYNIKLIIQEDITGTGTAVKIALENLEELSD
                     QDIVLIQYGDTPFISSDTVMKMTDCLKCNNKNLVLLGFNSQDKQYGRLVIDDNDNVQK
                     VLKSEDEILLANSGIMVSYAKDLYTLIKEVKFNDSTNECCLNDIIPIAASNNLRAGYV
                     VADEKEAMGVNNKEDLVKAERYFQANRRRQPTLLS"
                     /besthit="qcoverage 100 , hcoverage 100 , similarity 95.3
                     , identity 92.5 , evalue 8.45e-159 , alnlength 253 ,
                     Bacteria , Proteobacteria , WP_197907410.1 NTP
                     transferase domain-containing protein [Wolbachia
                     endosymbiont of Kradibia gibbosae]"
     gene            40858..41817
                     /locus_tag="wEsol_00057"
     CDS             40858..41817
                     /locus_tag="wEsol_00057"
                     /codon_start=1
                     /transl_table=11
                     /fullproduct="qcoverage 100 , hcoverage 100 , similarity
                     99.7 , identity 99.4 , evalue 1.40e-231 , alnlength 319 ,
                     Bacteria , Proteobacteria , WP_015588901.1 MULTISPECIES:
                     NAD(P)-dependent oxidoreductase [unclassified Wolbachia]"
                     /cog="WcaG COG0451 314 Nucleoside-diphosphate-sugar
                     epimerases"
                     /pfam="RmlD_sub_bind Epimerase NAD_binding_10
                     Polysacc_synt_2 3Beta_HSD NAD_binding_4 NAD_binding_4
                     Polysacc_synt_2 Epimerase_Csub"
                     /tigr="TIGR01179 galE 332 UDP-glucose 4-epimerase"
                     /product="NAD(P)-dependent oxidoreductase"
                     /translation="MGILITGAAGLIGSALVEKLEKQGYEVISCDIRFRDNPLSFFSE
                     DIMPLLAKCTGVIHLAAISRVIHGELYPKLCQKVNVDGTIQFLEFCKSLPNKPWFIYA
                     SSREVYGEQKELPVAESASINPINNYAKGKAFIEEQVINLKGSNFNVAILRFSNVYGG
                     LLDHSSRVIPALCINALKGDPIRIEGKECVFDFTYLDDVIEGIYLTVKYLQNEKSSLP
                     AIHLTTNSPCTLENLAKTILKVTESDSRIDFYPPRNFDVTKFHGDFTRAKELLGWSPK
                     HSLEVGLSKFIKSLQNNTQEYPNNIDMVIYENIKSYSWLPSLL"
                     /besthit="qcoverage 100 , hcoverage 100 , similarity 99.7
                     , identity 99.4 , evalue 1.40e-231 , alnlength 319 ,
                     Bacteria , Proteobacteria , WP_015588901.1 MULTISPECIES:
                     NAD(P)-dependent oxidoreductase [unclassified Wolbachia]"
     gene            41774..44101
                     /locus_tag="wEsol_00058"
     CDS             41774..44101
                     /locus_tag="wEsol_00058"
                     /codon_start=1
```

```
                     /transl_table=11
                     /pfam="Glyco_transf_4 Glyco_trans_4_4 Glyco_trans_4_2
                     Pfam-B_15682 Glycos_transf_1 Glyco_trans_1_4 Radical_SAM
                     Fer4_14 DUF4008"
                     /fullproduct="qcoverage 100 , hcoverage 100 , similarity
                     99.5 , identity 97.4 , evalue 0.0 , alnlength 775 ,
                     Bacteria , Proteobacteria , WP_226079611.1
                     glycosyltransferase [Wolbachia endosymbiont of Tribolium
                     confusum]"
                     /translation="MKILKVIHGYPPYYSAGSEVYSQTLAHELANNNEVQVFTRYENS
                     FLPDFYYTTVLDSSDSRILLHLINIPTAKYRYKFINEEVDIQFKRVIDNFQPDLIHFG
                     HLNHLSITLPEIAFKENIPTIFTLHDFWLMCPRGRFIQRNSEDLLRLCDGQKDQKCAT
                     QCYKGYFTGDEEFLNSDLNYWEQWVATRMKQTKKVVDYIDYFIAPSKFLMDKFTQDFN
                     VPINKISYLDYGFDLNRLKNRNRVQEKEFIFGYIGTHTPEKGVDLLLKAFSRLSSKAK
                     LRIWGAAREETKALKAIADQFSPVVKERIEWMGSYDNKNIVTDVFNKVDAIVVPSIWG
                     ENSPLVIHEAQQLRIPVITADYGGMAEYVKDGLLFKHRDASSLSEKMQVLSTNQELYN
                     KLTQKGYPYTENGNIPSISEHTEKLNKIYRNAIEKKGKSVAVKPGPWRITFDTNPDYC
                     NFSCIMCECFSPYSKVKEEKKAKGIKPKILSIETIRKVIKEAAGTPLREIIPSTMGEP
                     LMYKSFDEIINLCHEFGLKLNLTTNGSFPIKGARKWAELLVPILSDVKISWNGATKET
                     HERIMKGSKWEVVTENLKTFLEVRDKYFSDTGERCTVTLQLTFLESNLHELYDIVKMA
                     IKNGIDRVKGHHLWAHFEEIKDLSMRRDELAISRWNTEVRRLYELRDNILLPNGKKIK
                     LENFTILSQEGIKDLAPGGQCPFLGKEAWINNEGKFSPCCAPDELRKTLGNFGNVNEV
                     KLEEIWQSSEYLNLQKNYLNYELCKTCNMRKPLVN"
                     /product="glycosyltransferase"
                     /besthit="qcoverage 100 , hcoverage 100 , similarity 99.5
                     , identity 97.4 , evalue 0.0 , alnlength 775 , Bacteria ,
                     Proteobacteria , WP_226079611.1 glycosyltransferase
                     [Wolbachia endosymbiont of Tribolium confusum]"
     gene            44062..45756
                     /locus_tag="wEsol_00059"
     CDS             44062..45756
                     /locus_tag="wEsol_00059"
                     /codon_start=1
                     /transl_table=11
                     /pfam="Pfam-B_323"
                     /fullproduct="qcoverage 100 , hcoverage 100 , similarity
                     100 , identity 100 , evalue 1.36e-288 , alnlength 564 ,
                     Bacteria , Proteobacteria , WP_006280066.1 MULTISPECIES:
                     WG repeat-containing protein [Wolbachia]"
                     /translation="MQNMQHEKTISQLMKDSEYLKSIRVSPCERFHQLSENPLYKNRF
                     IRVDKFHEPGLAPVYDETGAYHIDAMGEAIYRDRFLKTFGFYCNRAAVEDDTGYYHID
                     PSGCRVYKHSYQWIGNYQEDICVIRKHDKFFHIDLNGNRVYEQEYNYVGDFKDGIAVV
                     HKDGKATHINNHGKLVHNKWYKKLNVFHKGFAIAEDKHGWFHIDINGNPVYRQRFKMV
                     EAFYNGMAKVETFEGVLGQIDITGNVKFSIFDLDKESQVHKISAELSAFWKTYLANVA
                     IELDLLNILPATMPVLSQKLNIIVPNLERLLRALWEIGFIDYDKNKDLWQLSSKGKCF
                     KEIPFLPKAAAMWARVAAEKNWLKIADILRQESISSFESFKEKETSEDRKIAFYQALL
                     GYSRFDTKEFNSRVNIDGAKNILLFGVHSLFLAYSDIHNKGSIGLYYYNEHKVPRQAV
                     EDLKIKLITQEELSATNYELGVFCRFLQHYDDNKVLSYLKLVKNKGIPRVLVIETILD
                     YYSPVGGSVDINVMVETGGKLRTLSDWKRILKQVKGLKIFSIVPLTDYLSVIDIRC"
                     /product="WG repeat-containing protein"
                     /besthit="qcoverage 100 , hcoverage 100 , similarity 100
                     , identity 100 , evalue 1.36e-288 , alnlength 564 ,
                     Bacteria , Proteobacteria , WP_006280066.1 MULTISPECIES:
                     WG repeat-containing protein [Wolbachia]"
     gene            45760..46749
                     /locus_tag="wEsol_00060"
     CDS             45760..46749
                     /locus_tag="wEsol_00060"
                     /codon_start=1
                     /transl_table=11
```

```
                     /pfam="PhyH Pfam-B_17183 2OG-FeII_Oxy_3 2OG-FeII_Oxy_5"
                     /fullproduct="qcoverage 100 , hcoverage 100 , similarity
                     100 , identity 100 , evalue 1.26e-239 , alnlength 329 ,
                     Bacteria , Proteobacteria , WP_006280067.1 MULTISPECIES:
                     phytanoyl-CoA dioxygenase family protein [Wolbachia]"
                     /translation="MKALQKNGFFIIKNLVPLELITQSLNDIISKISKLSQELGVSTS
                     DYLNCTGRWGTSSQVTRIVSQVLDKIIKNYLEKSLQCQILQKKSNVICKTADLIDAVP
                     FHQDISYSFNDPYHFSVWLALNNVNKTSGALQVIEDSHNWKIQPLVDFWHPYFLDQYS
                     DNRENYKIKSLPISAGDAIVFDSRLWHGSDKNIDAKDRFAYVTRWVVKDKSLPCVPKP
                     QPSVFGILNCGALTEFILREYLSLFRRQESIKTKNMEELIKSWLAFITDTTTNISEVN
                     TAEAKRDLYKLLILNQASALHDAGDISGKIYKNLWFSLLVFLNKKVNVVELAL"
                     /product="phytanoyl-CoA dioxygenase family protein"
                     /besthit="qcoverage 100 , hcoverage 100 , similarity 100
                     , identity 100 , evalue 1.26e-239 , alnlength 329 ,
                     Bacteria , Proteobacteria , WP_006280067.1 MULTISPECIES:
                     phytanoyl-CoA dioxygenase family protein [Wolbachia]"
     gene            46750..48555
                     /locus_tag="wEsol_00061"
     CDS             46750..48555
                     /locus_tag="wEsol_00061"
                     /codon_start=1
                     /transl_table=11
                     /fullproduct="qcoverage 100 , hcoverage 100 , similarity
                     99.7 , identity 99.3 , evalue 0.0 , alnlength 601 ,
                     Bacteria , Proteobacteria , WP_174516732.1 MULTISPECIES:
                     ABC transporter ATP-binding protein/permease
                     [unclassified Wolbachia]"
                     /cog="MdlB COG1132 567 ABC-type multidrug transport
                     system, ATPase and permease components"
                     /pfam="ABC_membrane ABC_tran SMC_N Pfam-B_16450
                     Pfam-B_6534 Pfam-B_5913"
                     /tigr="TIGR02204 MsbA_rel 576 ABC transporter,
                     permease/ATP-binding protein"
                     /product="ABC transporter ATP-binding protein/permease"
                     /translation="MNKSRVGSFKYILVFILNILSKFKLNVLVMFLVALVVAVDLSFR
                     KYLVKNILDTAVKYQEGNVIENLLLPVSAYVCMALLITTAFRFYGYFVDIRMFTSMRQ
                     KIADMSFYRLLQQDHSYYQNNLSGSLVYKVSNLMDSVIELIRLLIDCFFSYSIALILA
                     IYTLSLVNIKFAIATFIWVNIFILISVFSFRVLTGLADDHSKQSSQVIAGIADSILNV
                     ISVRLFSRQAHERHKFFQVCKKKTISERKLQWAYFWLWFIYGYSFDILQAVNLYFLIH
                     DYQLNKIAIGDIALVLGINISIIEFLNHLTRNLTQFSTHFGKVSDALPILTTVPEIQD
                     KENAKKLNLLSGRITFNNVSFSYEGQKSLFQDFSVTINPCEKVGLVGYSGGGKSTFIN
                     LIIRLFDVKKGNIQIDNQVVSEVTQSSLRQQISVIPQEPLLFHDTILANIIYGKLQST
                     IEEIIRAAKLAGIYDFIMTLPDQYETVVGEKGIKLSGGERQRIIIARAFLKNAPILFL
                     DEPTSQLDSITEKTIQMSLFKLMENKTTITIAHRISTLLHMDRILVFDKGKIVQDGKH
                     SELVCKKGLYKELWDAQIGCLNIKKENGKNYRPEK"
                     /besthit="qcoverage 100 , hcoverage 100 , similarity 99.7
                     , identity 99.3 , evalue 0.0 , alnlength 601 , Bacteria ,
                     Proteobacteria , WP_174516732.1 MULTISPECIES: ABC
                     transporter ATP-binding protein/permease [unclassified
                     Wolbachia]"
     gene            48527..49576
                     /locus_tag="wEsol_00062"
     CDS             48527..49576
                     /locus_tag="wEsol_00062"
                     /codon_start=1
                     /transl_table=11
                     /fullproduct="qcoverage 100 , hcoverage 100 , similarity
                     100 , identity 99.7 , evalue 7.09e-247 , alnlength 349 ,
                     Bacteria , Proteobacteria , WP_174516731.1 threonine
                     aldolase family protein [Wolbachia endosymbiont of
```

```
                     Cardiocondyla obscurior]"
                     /cog="GLY1 COG2008 342 Threonine aldolase"
                     /pfam="Beta_elim_lyase DegT_DnrJ_EryC1 Aminotran_1_2
                     Aminotran_5 SelA"
                     /product="threonine aldolase family protein"
                     /translation="MEKTIDLRSDTTTLQSSNVMHAISSAIVGDFAYGEDKSCNNLSE
                     YCKQLFKVEEALFVTSGMLANRLAIASQTSPGDELITHYNYHVNFFDSAGNAKVNSIV
                     FNCIRNNTGILDIDEVEYAINSKPRYKIFAQVSLVSIENSINGFNGKIYPFKKQVELY
                     NFLKAHNISLHLDGARIFNAHAETNVALADYAKYVDTMSFSFTKGLGAPFGSMLMGKR
                     EIIEKAKKLQVWLGSGYHQIGYCANAAKYVLQNNISRLKEDNKLAKLFADKIKEIPHV
                     KLILPYPETNIVSFSIKELNVTNEFFLSKCQAYGLLLFPWLESHIRAVVHLGIEEADI
                     INAAEIIKEVVLNLV"
                     /besthit="qcoverage 100 , hcoverage 100 , similarity 100
                     , identity 99.7 , evalue 7.09e-247 , alnlength 349 ,
                     Bacteria , Proteobacteria , WP_174516731.1 threonine
                     aldolase family protein [Wolbachia endosymbiont of
                     Cardiocondyla obscurior]"
     gene            49573..50616
                     /locus_tag="wEsol_00063"
     CDS             49573..50616
                     /locus_tag="wEsol_00063"
                     /codon_start=1
                     /transl_table=11
                     /fullproduct="qcoverage 100 , hcoverage 100 , similarity
                     100 , identity 100 , evalue 4.65e-251 , alnlength 347 ,
                     Bacteria , Proteobacteria , WP_174516730.1 MULTISPECIES:
                     threonine aldolase family protein [unclassified
                     Wolbachia]"
                     /cog="GLY1 COG2008 342 Threonine aldolase"
                     /pfam="Beta_elim_lyase"
                     /product="threonine aldolase family protein"
                     /translation="MRVKIDFFSDSNTNPSSEMREVMAKAKVGNEAACEDPTVNELIE
                     TTCKILGKPAGIFLISGTMCNVIAYKVHIQRPGDYLLLDETSHPLIVQSGLIAAQAHA
                     TPLPIKGTRGIFTGEQIVEFITRPSLRNVPKVGLVSVEQTTNFGGGAVWPLEYIQEIS
                     SLCKKNDVSSHLDGARLFNACAYAKISPKEYANYFDSVFIDFSKGLGAPMGAVLAGSE
                     EFIEKAWYYKFQIGGGMHQAGIISAACLYALNNNMNSLIEDHKKMQHLIKGLSSIDQV
                     IINIDLYKTNIAYFSLRTDCISAQELINILMTNYGIRMANIRGKIRVITHRDISCEDI
                     NTTISTIRKILNR"
                     /besthit="qcoverage 100 , hcoverage 100 , similarity 100
                     , identity 100 , evalue 4.65e-251 , alnlength 347 ,
                     Bacteria , Proteobacteria , WP_174516730.1 MULTISPECIES:
                     threonine aldolase family protein [unclassified
                     Wolbachia]"
     gene            50912..51124
                     /locus_tag="wEsol_00064"
     CDS             50912..51124
                     /locus_tag="wEsol_00064"
                     /codon_start=1
                     /transl_table=11
                     /product="hypothetical protein"
                     /translation="MDSKNNPLFMFGLCNIATGLLTIGIALSYNNLTLLLLTLILLAW
                     TQGLGWPAITKFMVTNSSISSIASSW"
                     /cog="UhpC COG2271 448 Sugar phosphate permease"
                     /besthit="qcoverage 100 , hcoverage 20.8955223880597 ,
                     similarity 98.6 , identity 98.6 , evalue 2.52e-37 ,
                     alnlength 70 , Bacteria , Proteobacteria , CCE77508.1
                     putative Major facilitator family transporter [Wolbachia
                     pipientis wAlbB]"
     gene            51128..51919
                     /locus_tag="wEsol_00065"
```

```
CDS             51128..51919
                /locus_tag="wEsol_00065"
                /codon_start=1
                /transl_table=11
                /product="hypothetical protein"
                /pfam="MFS_1 MFS_1 Sugar_tr"
                /translation="MSASQQLGSCITLIVLPSIIKIYDAKSAFLYSGLLCLLFGIYII
                LKAYYEESANFTTYYKVQKSQIGIKVKSSIWFLCCATFCAYIIKMGIFFWFPIILKNK
                LQISLVQSSLITGVYDLGGILGTLITGRISDMYFFQNRSLLASLYMLGIALTFIAMNF
                GQNIILMNLGAILSGFFIFGVQVLTGVMAVEIAPQNNVCAIVSLTGLFGYIASSIFSC
                VLLGVLVKHCGNSIMFLFFSICSVVALVCFSILSSKDLKKLSKAV"
                /cog="UhpC COG2271 448 Sugar phosphate permease"
                /besthit="qcoverage 100 , hcoverage 69.0288713910761 ,
                similarity 99.2 , identity 98.1 , evalue 9.49e-168 ,
                alnlength 263 , Bacteria , Proteobacteria , OAM03919.1
                MFS transporter [Wolbachia endosymbiont of Dactylopius
                coccus]"
gene            complement(51937..53268)
                /locus_tag="wEsol_00066"
CDS             complement(51937..53268)
                /locus_tag="wEsol_00066"
                /codon_start=1
                /transl_table=11
                /fullproduct="qcoverage 100 , hcoverage 100 , similarity
                98.0 , identity 96.6 , evalue 1.05e-307 , alnlength 443 ,
                Bacteria , Proteobacteria , CCE77509.1 UDP-glucose
                6-dehydrogenase [Wolbachia pipientis wAlbB]"
                /cog="Ugd COG1004 414 Predicted UDP-glucose
                6-dehydrogenase"
                /pfam="UDPG_MGDP_dh_N NAD_binding_2 3HCDH_N Pyr_redox
                AlaDh_PNT_C ApbA UDPG_MGDP_dh UDPG_MGDP_dh_C"
                /tigr="TIGR03026 NDP-sugDHase 413 nucleotide sugar
                dehydrogenase"
                /product="UDP-glucose 6-dehydrogenase"
                /translation="MLSYIFKVMHITIIGVGYVGLVSCAMLSEQGHKVDCIDINTEKI
                ELLKSGKIPLYEPGLADYLENNIKSQRIRFFDSYFQINPNTEVVFITVDTPSDSLGNA
                NLKNVYNAVSEVSERINQDCLIVIKSTIPPGATKNIYNYLSNKGYNFDLGVNPEFLKQ
                GSAVSDFLYPDRIIIGVKTKRARVKLEAIYRSFTDRNIPLIVTDTVTAEMIKYASNAF
                LATKVAFINEMAGLCELLGADIELLANSMGMDHRIGKEFLKAGPGFGGSCFPKDLSAL
                IRLAEDHNVKLQILNSVKESNYNHITNIAKKVEYVLNGVQNKKIAIWGLTFKSGTDDV
                RNSPAIDIAQLLVNNKAKITAYDPMGMDNARQVLKDIEYADNAIGAARDAEALLLLTE
                WKEFKNQDFASLKSIMAAPNVFDFRNLLDSELLLRYGYHVYSLGKKSIYKV"
                /besthit="qcoverage 100 , hcoverage 100 , similarity 98.0
                , identity 96.6 , evalue 1.05e-307 , alnlength 443 ,
                Bacteria , Proteobacteria , CCE77509.1 UDP-glucose
                6-dehydrogenase [Wolbachia pipientis wAlbB]"
gene            complement(53284..>54148)
                /locus_tag="wEsol_00067"
CDS             complement(53284..>54148)
                /locus_tag="wEsol_00067"
                /codon_start=1
                /transl_table=11
                /fullproduct="qcoverage 100.348432055749 , hcoverage
                95.6810631229236 , similarity 98.3 , identity 97.2 ,
                evalue 1.07e-179 , alnlength 288 , Bacteria ,
                Proteobacteria , WP_010082202.1 MULTISPECIES: DMT family
                transporter [Wolbachia]"
                /cog="RhaT COG0697 292 Permeases of the drug/metabolite
                transporter (DMT) superfamily"
                /pfam="EamA EmrE EamA"
```

```
                     /tigr="TIGR00950 2A78 264 carboxylate/amino acid/amine
                     transporter"
                     /product="DMT family transporter"
                     /translation="IWFILSLFSSVANDTISKYLSLHLQSFEVIFFRFLFSTITLVPF
                     MLYYGREAFKTSQISIQITRGVLLFVGITLWTYGLAIFPIVTATIISFSIPLFVILLA
                     IPLLNENIIWQRWSVTVIGFVGIAITTKAYSEDFNPKILIFIVSTLIFAILDILNKKL
                     AIKESVISMLFYSALTTTIFSTVPLLFYWHLPSLLELILLLILGISSNLILFFMLKAF
                     ALTDATALAPYRYIELIISAIVAYVIFNELPDKSALYGTLILIPSTLFIAYSEGKAIK
                     KNNVTTKVYNC"
                     /besthit="qcoverage 100.348432055749 , hcoverage
                     95.6810631229236 , similarity 98.3 , identity 97.2 ,
                     evalue 1.07e-179 , alnlength 288 , Bacteria ,
                     Proteobacteria , WP_010082202.1 MULTISPECIES: DMT family
                     transporter [Wolbachia]"
     gene            8459..8540
                     /locus_tag="wEsol_tRNA-01"
     tRNA            8459..8540
                     /locus_tag="wEsol_tRNA-01"
                     /gene="tRNA-Leu"
                     /intronpos="0..0"
                     /program="tRNAscan-SE"
                     /cove_score="62.7"
                     /evidence=predicted
                     /type="Leu"
                     /anticodon=TAG
                     /product="tRNA-Leu (TAG)"
ORIGIN
        1 gctatggtta ctgaccctca ttttttgtgg catatttttt ctttcaaggt taaatggcta
       61 ttttataatg aattttgtca gtagccacca gatttttttca gttgctatgc aacaaagcca
      121 tcccagtgtc aagcactgga atgacatcac aggggcactg gaatttttgt ttcaatattt
      181 gtacattagc catgcatctg aacagatact gtaaattagc tatattacat tagcccatca
      241 gatgagttgt aatggttata attgtctgat aagtgatctc taacatcgtg acctaactca
      301 ttggcatcag gcttaacttc gtgatctaac tcattagcat cgagcttaaa caaagaagga
      361 ttatcatcag attgttgatt agactctttc tctttcgacc caaataacca tgaaaagagt
      421 gatgagatag aatcaataat ggatttaatt gctgaaaaaa tattggataa gaaattactg
      481 ggttgctgag tatcctcttc ggctgattct ggttcaggct cttttcctgc tgtgtcatcc
      541 acatcttgtt caggcttact tctgcttatg agtgtcttat ctaaccattt ggacatattc
      601 ttgtctggat tatgaaaata cattttctct atgtccttgt tttcatcaac aaaaaattact
      661 acagttgcat tctgacttaa tgacccttca tttaatctat aacgttcact aacgagcgat
      721 tcatgagagt tctttatatc ttgaatcaat cgaagttttt cttcttctac taaaaggtaa
      781 cctctgtgtt gtatagcata attggtccct ttgtacttaa attgcaacat tcctgtaacc
      841 tctgtgtagt catcaccttt ttgttcttct tctggctctg gattcgtgcg gttttcattt
      901 acctcttctt cagacttact tctgcttata agtgtcttat ctagctgttt gaatatattt
      961 ttgtcggagt ttcgaaaata cattcgactt atctccttat tttcatcaac gtgaatcgat
     1021 aaaactgtat tctcattccc caatccatca cattcactaa cgagcgattc atgagagttc
     1081 ttaatgtctt tgataaattg aaatttttct tcttctgaaa agtacttgca tcttacgtta
     1141 taagtggtgc ctttgtattt aaaactaaat atccatccat ggtcttcata ttcatctagt
     1201 ccagaattct tgacacgttg tataatttga aattttttcat cttctgaaag gtccttgagg
     1261 tatatactac cagttgtatc tttatattta aatttaaaca tccatccatg gtcttcatat
     1321 tcatctagtc cagaactctt gacacgttgt ataatttgaa atttctcttc ttctgaaagg
     1381 tttaggcggt atacactacc agttgtacct atgtatttaa aaataaaact ccatccatcg
     1441 tatttatatt catctacacc catctgcttg caaatagctt tataattttt ttcttctact
     1501 tctcgtccaa caacatttgc tatgagttca gtatccagaa tactcaaagt ttggactgtt
     1561 gccgatggtg aaaaacacaa ttgaaatatg tggcctagct catgacccag aacaaaatcc
     1621 atggcacttt gcttatatgt cagagtattc tgacaatctg ttactcctcg tgtaatatat
     1681 cctggactac catcttcatc tatgccgaga agattgttat actttgtata atcgcttcta
     1741 ttattgaaaa tgtaaaccgt tatattttta cttatgctac tatctttgaa gcaaaacttt
     1801 tctttaaatg cctcataggc agttttaatg tctgtttcta tttggttttg ttcctgctca
     1861 gataagttgt cgtattttat agtacaattt atatacccgt atttaacggt aaagctgtta
     1921 gacaaaacat catgtatatt tgtggaaaaa taattgaact gctttgactc ggcttcttta
```

1981 tacaattgat gcccatattt atcactagaa tactttggta gtttaggtac tttggcaatt
2041 aagtttttgt tactatcaaa aaaagaaata tcagaattac caatcctaaa atcctttata
2101 gattgcccgc aagtgccatc atttgcacaa tcatgattat ctttcttatt aaaccatgcg
2161 ttggtgtatt tttccatttt gatacctcac taccaaacct tactatatta ttatcttggt
2221 aaaaatcata ataattcgtt aacttgtatt ttaaatctgt atcaaatgtg cttttctcgt
2281 aaggtatcaa ttgtgaagta attaacttta tttgtttaaa attttgctaa aatagctctt
2341 tttatactat gttttatata gctaaactga cttagattta ctgacaattt gaaaaacaaa
2401 aattcgtcat tccgctacta tagtattttc taaaacgaat gataaacgtc taaaatggta
2461 ttaccaacta aaaggtgtaa gtttgtacat tgatgcatta gaggtctgag gcgacttttg
2521 atggtgtgcc aacaatgctc tatcggattt agatcttact attacccaca aatataaatg
2581 tttgttgcat agttcttaat ggcataactt ttgcttgaaa taactgttta tatggtgcaa
2641 tttacagctt ttagttgcta aaattataac tcttagttta ataattttag tctaataagc
2701 tttcaggtta ataaaattaa attatttgag taaatacaat attatgtttt tatttgtggg
2761 taatagtaag gcttaaagca aaatgtagta cgaacggtag gtggatctaa aagatcgaat
2821 tacaatcctg tatacagcat tttgcattat tgtttgatta tatttatagg tacaaaaatg
2881 gctagaataa ttttcatggg agtagatgtt tccaaggaag cattggacat ctcaattaat
2941 ggcaagcata agcgcatagc aaacgtgagt gatgctatat ctaagtttat agaaacaaat
3001 atagtcgatt tacccatcaa gttatatgta aactggtggc tatgaaaagc tcattataaa
3061 attgttgcaa gataactgtc cataaagctc acccgaatcg ggtatatgca tttgccaaag
3121 cttgcaatca ttttgcaaaa actgacaagt tagatgctaa attactggaa aaatacgctg
3181 aatttgtcag taaagacggt gaagtaaaag gatgtataat ttcacaaaag caagaggaat
3241 taaaggagct gggggcagtt gagcgtaatt taaatgatgc aattcatgca aacaagtgtc
3301 gcctgcaaag tgcgacggga aaagctagag aatacataga aagacagata gggtttgctg
3361 aaaaacaatt gaaagaaatt aggcaagaca tagaagacac aatagattct gaccctgaat
3421 tgaaaggaaa aaatgagctt ttaatcagct ataaaggtat tggtcaaaga cggcgagcgt
3481 tctactgaca gaggtgcctg agcttggtag attagagaat agagaaattg catgcttagt
3541 tggagttgca cctaaaacta gtgaaagtgg aagaaaagtt ggcaaagctc aaatcaaagg
3601 aggcaggttt tatgcaagga aagcactata catgtcagct cttgttgcta tgcgccacaa
3661 cagaaaaatg aaagcgtttt atcagcgttt ggttgattct ggcaaagctg ctaaggttgc
3721 attagttgct gtcatgagaa aaattatcgt ttgtttaaat gccatgataa aaaataacaa
3781 cttttatctt gactcttaag acggtagatg acagaccatt gattgtatgg gttccatctg
3841 ttatatataa tctttttgaa tacgaagaaa actgatgaat gagtatgata ttacggttat
3901 aggtagcggt cctggtggtt atatagcggc aattagggcg gcacagcttg ggtttaaaac
3961 tgcaattgtg gaaaaagaaa aaaatttagg cggtatatgc ttaaattggg gatgtatacc
4021 aacaaaatca ctgcttagag catctgaggt ttataggcta ataaaaagat caaaagagtt
4081 tggtatagaa gtaaagggag cgaattttga tatacaatca atagtgaaat actcaagaaa
4141 cgttgttgat aaattgtcaa gcggtgttgc atatttgatg aaaaaaaata atatcaaagt
4201 tcatcaaggt tttggtaaac ttgcaggcaa tggtactata aaagtcgtta gcgataagga
4261 agaacaagaa attgtttcca agcatatcat tttagcaaca ggcgtgaggg cacggaatct
4321 gcctggaata gaggcagatg gagatttaat atggaatgcg cagcacgcta tgactccaga
4381 gagattacca aaatcgctac taattatagg gtctggtgca attggaatag aatttgcaag
4441 tttttatagt actttggggg ttgatgtaac aatcatagag ataaagagca ctattttgcc
4501 gctggaggat aaggacattt cagatttagc acaagaaata ttcacaaaac aggggataaa
4561 aatatataca aacagtagcg taaaagctct tactaaaagt aaagactctg ctcaagtgct
4621 actaagtagt ggtgaaagca aagaatttga taaggtgatt gttgcggttg gaattcaagc
4681 aaatattgaa aatataggtt tagaaaatac aaaaattaaa ttaagccctt ctggctttat
4741 tgaaacgaat gaatggtatg aaactagtga atcaaatgtg tatgcaatag gtgatgtagc
4801 tggcccacca tgtttagcac ataaagcgag ccatgaagcc gtgatctgca ttgaaaagat
4861 tgctggtaaa aatgctcatg cgttaaaaaa agaatgcata ccaaattgca cttattctca
4921 tccacaaata gcgagcatcg gccttactga ggaacaggca ataaaaggtg ggtatgatat
4981 aaaaatagga aaatttcact ctaattttaa tggtaagtct gttgcactca gtgaaactga
5041 agggttagtg aaaacgatta tagataaaaa aaccggagaa attctgggta gccacatgat
5101 aggtgcagaa gtaacggagt taattagcaa ttttgctctt gcaaagcaac tagaaggaac
5161 agacttcgac ataaaatcta cgattttttcc tcatccaacc atttcagaga tgatacatga
5221 atcagtgctt gctgcagatg ttaaatagtt gaactaacgt tccatcgttt tagagttgtc
5281 aatgtcttga gtagtagttt gattaggcac tttagtttca atagattgat taccaccagc
5341 atcagaattc tgtaactctt ctttcaatcc cttagctgct tctttaactt caagctttgc
5401 ctttggatga agatcttctg ggccttttcc tttagtatcc aaaccttgtt gtagattagt
5461 gcttttcatt ttattatgct gctcgtgctc aggtaggtca acgaccgaat tgaaaagaat
5521 ttttattata ttttttagca gttctaaaaa acctccgctt ctgccttctc tttgttcttg

5581 aggtttattt ttctcattag gttcttttt actattactt ttaggcattt ttaactcaac
5641 tatcataacg cttaataaaa ttctacgtgc aaattgttaa ataacagtta acaatctagg
5701 atttattatt tacagatgga tattatttta gcaacaggtg gcacaggtgg gcatatcttt
5761 ccggccatag ctttagcaaa agcactaaag acacaaggat acaattgcat attattcact
5821 gataaaaaaa caaataaaaa taccgacata gaaagctaca ccttaccatt acgtagacca
5881 agcagcaaca aatttaagtt tttccttttc ttgatataca gctctatgtt agcactgtat
5941 caagtaagaa aattaaaacc aaaatcggtc attggttttg gtagctatgc ttcttttcca
6001 actcttcttg cagcaagagt tctttctata cctataattt tacatgaaca aaacacagtt
6061 ttagggagag taaatagatt ctttttcaag agtgcaaaat taattgcaac cagctttccg
6121 gagactaaat atgcagaggg caataaatgt atttttacag gaaattttgt tgatataaaa
6181 gcacagagcc attctagcac tgagaaaatc ctaaatatat tggtcatagc aggcagccaa
6241 ggtgcaaatt tttttgatga tgtagtaagc agagtaattt gtgatttacc tattaaaatg
6301 aaaaagaaaa ttagagtgac gcagcaatgt acgaagaaaa atgtaaacaa ggtcaaaagt
6361 ctatacaaaa gtgaaaagat cgattgtgaa ttgagtgaat tttttgatga tatggagaat
6421 agactggcca atgctcactt ggtaattagc agagcaggag caacttcaat agcagagatc
6481 actcttgcta ggcgttctgc tatatatatt ccttaccctt actcaaaaga taatcaccaa
6541 ttttataacg caaaatatat tgaagactcg agagcagcta taatagttga gcagaatagt
6601 gaagcaaaaa aaaatctaac agaggtacta tttgatctat taaataattc tcaaaaattg
6661 cgtgatatga ccaatagtac agaaaaaaca gggataaaga atgggactac tgagtttgtt
6721 aaggtgattg ttcacaggtt tagttgatgg ctgcttgctt tttcttcaaa cacgtcccaa
6781 agatcctttt ctagcgatat attatacaat ttattgatat aaactactcc cgtgggtgaa
6841 gttgtgttaa tttcgagcag gaagtcatct ataatatcaa taccaacaaa tattagccct
6901 ctttcttta attcaggacc aattttgtta catatttcat tgtttctatc gttcattga
6961 gcgggctcaa aacttgctcc aagtcgcatg tttgttctga tctctccact gatttttgga
7021 attcgtttca ttacaccaat tggttggcca taaagtagca atattctttt atctttatct
7081 atgtttttac aaaatgattg tgcaatcaca gggcattcgt attttgctat catgagatcc
7141 actactactt gaatactatt ctcatcttgt attcttatca catcgtttcc tccataacta
7201 tacaatggct tcagaataat atcttgatag ttgtagtaga aatctctaat cattgatata
7261 ttttcagtaa tcaaagttgg cggaattagc tctggaaata atgaagttat caatttttca
7321 ggacaatttc ttatttctgt tggattgtta attaccaatg cgctggtttt ttctaagata
7381 tatgttgttg taatataacg catatcaaag ggtggatcct gtctgataaa tatgatgtcc
7441 atttcattca agttgattgc tacatcctct ttagagataa aactaaaatc atcaacgctg
7501 actttctgag ccagagcaat tggatggttt agctttagtg ctaaattgtt aggagcatag
7561 acaaaaactt cgtgcttcct cctttgtgct tcctttatta gcacgaatgt agtgtcagtt
7621 tcaaaattta tattttcatc catctgaaag gcaattttca tttatgttct ctacctattg
7681 tcacaactgt acaaatgtta taatttgaaa gcgatttttg cctaatggcg taacttactg
7741 tacctgtatc gtacataact tacaacttgt tttactccct ttactttctt tgcaattgct
7801 attacagctt ttaattccgc tttattttgg gctataccca tcaaataaac aactctatca
7861 accgtattaa cgctataatt aattgactta atatttcttt ttcccaggag tcttgttctt
7921 atttccgctg ttatcatgcc atctacagtg gtgtctagca tggaagccat ttgaattggt
7981 attactctta tttcatttat cacttcttta atttcttttt gctgccaagc tattttttct
8041 gctgtaagtt gtttttcagg agtgtcaaca cttccaatga gcaatactct tccttcactt
8101 actttaacct ttatggatga aaatagccca tgttttaaga gccccttatt aattctgatg
8161 accatagtcg tatcatcaat gatatttccc aaggatttgt cctgcattgt tattgctgtt
8221 gctgtcgctg tagccaccac accacctatt ataagggttg tgcaaccact ttgtgtaatc
8281 aaaaaaattg cgagtaagaa gcttgttatt aatctcatta ttgattttca tggtttagag
8341 ctttcattaa gaaatgttaa taagcttttt tgattgtgta aataccataa attaagtaag
8401 actttaaacc ttgaatattt ttaatggaat acctataata gggtactgaa aatttcttgc
8461 ggatatggcg gaattggtag acgtgccagg tttaggtcct ggtgagcttg ctcgtggggg
8521 ttcaagtccc tctatccgca ctttaaagtc atgtaactta ggttttaagg gaaaatttat
8581 aatcactaaa tacactttttg tttgtgtaaa aacctgttct taaggattgt gtttaactta
8641 aattctggta aaatgactct attaagatat gaattaaaat gttaataaat gaattatgat
8701 gtctaataat atacctcaaa atacagtcga agtaagtagc gtatatacct acaaagagct
8761 tagtatagat aaactaaagt atgagtatga aatcacagtt ggtagtgatt atataaaaca
8821 aaaggtaaat tccaggttgc aagagatagc agaaaatgca aaatcacctg gatttagggc
8881 tggaaagatg ccctatgacc ttgttgttgc aaattataaa aacgaggctt tggaatatgt
8941 gataaataat acaattgatt attgctcaag tgatttgatg aaaaaaattg aagtcaagtc
9001 tcacatttat cctaaggttg atgttatatc attaccagat ctgggcaaag aaaatgagga
9061 aggcaatttt gtatacaagc tatcttttga gtcgatgcct gaagtaccga tgatagatct
9121 tgacaaaata aacttaaaga aaattgaagc aaagattgaa gaggaagata taaaagaatt

9181 tattgattct atcaaaacaa agttccccaa ttttgcctct gttgatgatg cttcttatca
9241 agcgaaagat ggggataaat tgataattga ttttgaaggg cggattagga ataagctctt
9301 tcaaggtgga agtagcaaag attttgccgt taatttaggg tctggcacat ttattaatgg
9361 ttttgaagat caattaactg gtatgaaaaa gggagaaaca aagaacttta agttgaaatt
9421 tcctgaaaat taccaagcca tttcccttgc aggacaggaa gctgattttt ctgtgcgagt
9481 taatgagatt caaattgcca aagattttga aaatgatgat gaaatagcta agagtattgg
9541 gtttaaagat tattctttgc taataaatca tgcaaaaaaa atgattggtg atcagtgcac
9601 cgaaatgaga aatctcttaa ttaaaaagga gctgtttgat tatttggatg ctaattatag
9661 ttttgattta cccacagaca tagtaaaaca ggagcaacaa agaatggaag gagaattggg
9721 tgctcaaaat gattctcgta aagaagctga aaaacgtgta aagcttgcta tgttatttat
9781 gaaatttagt gcagaacata aaatatcatt aactcaaaat gatgttttga gtgttattgt
9841 aaatcaatat gtcagtaaag atgtgcaatt tgacagagta attaagcatt ttgaatcaaa
9901 taaacaattt caagaattag ttagagggca agcgcttgag tataaagtga cagattatat
9961 aatagaaaaa gttagtaaag aagagcagat tgtttctgtg aaagaattaa aggaattatt
10021 tgataatatt taataggaaa aggtatgact cttataccaa ttgtagttga acaaactagt
10081 cgcggtgagc gcgcttatga catatattca agactagtga aagaaagaat aatttttgta
10141 acaggtccta ttgaagacaa tatggccagt gtaatagtag cacagctttt atttttagaa
10201 tcggaaaatc ccgataaaga catttgtatg tacatcaact caccaggtgg tgttgtaact
10261 gctggtttgt cgatctacga tacaatgcag tatataaacc cagacgtttc aactttatgt
10321 ataggtcaag ctgcgtctat gggttcttta ttgcttgcag ctggtacaaa aggtaagcgc
10381 tactctctac ctcattcaag aattatgata catcagccat ctggtggtta tcatggtcaa
10441 gcaactgata tagaaataca tgctaatgaa attttgcgag ttaagaaaaa actaaatcaa
10501 atttatgaaa aacatactgg aaattcactg aagaaaattg aaggaatgat ggaaagagat
10561 aaattcatgg atcctgaaga agcaaggaaa attggcttaa ttgacagggt gatagctgag
10621 cgtacagata ttgaaattga aaatattaaa gttaaacaaa aggtgggtta atggataaca
10681 ataatgattt acactactgt tctttttgca acaaggcgca agatgaagta gataaattga
10741 ttactaactc ttcggatggt ttaaaggtgt ttatttgtaa tgagtgcata gaattatccc
10801 ataaagcaat aagtcaaaag aaagatagat cttttaattc agatcgtata tctgatatga
10861 agctattact aaagaaacct gaggatataa aaaactttct cagcaagcat gttgtaggtc
10921 aagaacacgc gcaacacgtt ttatctgtag ctatgtataa ccattgtcaa tccatggtac
10981 aatttcatgc tataagtgat attgaaatcg agaagtcaaa tataatgctt attggtccca
11041 ctggttctgg taagactttg ctagccaaaa cacttgctaa agtttcagat gtgccatttg
11101 ctatggctga tgcgacaact ttaactgagg caggttatgt gggtgatgat gtagaaagcg
11161 tgttgtcgcg tttattacaa gctgcaaatt atgacgttgt aaaagcacag cgtggtatag
11221 tgtttataga tgaaatagac aagattacaa gaaaatctga aagcacttca ataacccgtg
11281 acgtttcagg tgaaggggtc cagcaagctc tgcttaagat tatggagggt acagttgctt
11341 atgttccgcc acaaggaggg cgaaaacacc cgcaacaaga gtttatacaa gtggatacta
11401 gtaacatact gtttatttgt ggaggagctt ttgaaggcct cgataaaatt attgaagcga
11461 gaaaaaaggg aacgtcagtt ggctttggag cagatataag tcagtctaaa gagcaaaaga
11521 aaaagaatgc tttacatgat gttcaacctg aagatttaat caaattcggt ttaatacccg
11581 aatttgtagg gcgggttcca ataactgctg ttttagatga gctggatcat gaagatttaa
11641 tacatgtgtt gacagagcca aggaatgcac taataaaaca gtacaaagca ttgcttgcgt
11701 ttagcaaagt aaaccttgag ttttcagatg aagcaatatc agctattgct aaaaaggcta
11761 taagctacaa aacaggtgca agaatgttac gtgctatctt ggagtcactt ttgcttgaca
11821 ttatgtatac atctggaaac ggaggctttg aaggtagcac tatagtaatc actaagaaaa
11881 tggtagaatt aggaaaagca acggttaatc ataataacaa tggcaatgtt ataacggtta
11941 atgattaatt tgaggcatat attatgaaca ttgaacgtgc tgtaagctct agttttactg
12001 cattgccagt attaccttta agagatgtag taatttttcc aaatataatg gtgcctttat
12061 tcataggcag agaaaaatct gttaatgcac tagagtatgc aataagtagt agtaatcacc
12121 aaaatgagat ttttcttgta gcacagaaag atggctctgt tgataatcca gagcctgaag
12181 atctttatga agtaggtgtg ttagcaagca ttgtacaacc gttaataaaa ttacctgaca
12241 atgcagtaaa agttataatc cggggataa gaaggggaag agttgtagaa tatattagct
12301 ctcatacttt attacaagcc agggtagagt tagacaatca ttataaagaa gatgaagaca
12361 atattgattt agaagcttta aggcggtctg ttgtagacgc gttcgatagc tggtgtaagc
12421 taaacaagaa aaatcaaccc gaggttgcca ttaactccat tgatcaaatc aaagaagttg
12481 atcaacttgt agacacggta gcttcacatt taaatataaa agtatcagat aaacaaagta
12541 tacttgaggc ttatgaccca gaagagcgtt taaaaaaagc ttttgctttt attgagagag
12601 aaatgagtat tttgaacgca caaaaccgtt tatataagac aattaaatcg caagttgaaa
12661 gcactcagaa agtttactac cttaacgagc aattgaaagc tatacagaaa gaattaggcg
12721 aatttgagaa tggtgatgaa gggaatatac tcaatgaatt tgaaaaaaag ataaatgaaa

12781 caaagctttc tcaggaagca agagagaaag ctataactga tttgaagaga tacaagaaaa
12841 tgaatcccat ttctcctgaa gctacagtta tatctagtta cttgcattgg ttgcttgagt
12901 tgccatgggg aaagtacaaa gatgcaaaaa ttaacttaaa tgcagctaag aaaatcttag
12961 atgaaaatca ttatggcata gagaaagtaa aagatagaat aatagaattt ttagcggtat
13021 taaaaagagt aaaagagata aaaggtccta tactttgctt agttggaccg ccaggtgttg
13081 gtaaaacttc tttagctaag tctatggcaa gagcagtagg aagagatttt gttcgcatag
13141 ctcttggtgg tgtgcgtgat gagtctgaga tacgagggca taggaaaact tatattggct
13201 caatgcctgg taaaatcatt caacacatga aaaaagctaa ttcatgcaac ccgctttttc
13261 tgcttgatga aatagataag atgggttctg attcgcgtgg cgatcctgca tctgcattac
13321 ttgaggtttt agatactgag cacaataaac actttacgga tcattacttg gaagttgagt
13381 ttgatctttc aagtgtaatg tttgtagcta cggcaaacag cttaaattta ccacatcctt
13441 tgcgtgatag gatggaaatt atacaattgt ccgggtatac tgaagatgaa aaaatcagta
13501 ttgctacaca ccaccttatt ccgaagttaa agaaggagca tggtttgtat cagaaggaat
13561 gggacataac taatgaggcg ttatacgagt tgatacgttt atatacacgt gaaagtggtg
13621 tgcggagtat ggaaagggag cttgcaaagc tcatgagaaa ggcagttaaa gaaatattga
13681 ctgataaaaa taagaaaata tccgtagggg ttgacaattt acaagattat ttaggggtac
13741 gtaaatatac ctttggcatt gcggaaaatg agagtttagt aggaatagta actgggctcg
13801 cctatactga aacaggtggt gatattttga tgatagaatc agtcctaatt ccgggcaagg
13861 gagaaataaa atacacagga aagcttggag aggttatgca agaatctata aaagctgcgt
13921 atagttatat tcgatcaaat tgtttgtttt ttggtataaa gcccgaaaaa ttccaaaata
13981 atgatataca tttacatgta cctgagggtg cagtaccaaa agatggccct tctgctggcg
14041 gcgcggtatg tacgtctatt gtttctctta tgacgaatat accagttaac aaaaatgttg
14101 ctatgacagg cgaagtgaca ttgcgtggta gagttttagc tattgggggc ttaagagaaa
14161 aattgcttgc agcgctcaga ggatcaatca aaactgtgat tataccaagt gaaaatgaga
14221 aagatatgca agagattccg gcaagcatca aagaggggat caacgtagtt tttgctaaga
14281 atattgatga agttatgaaa gttgctttaa tgcaccctac tactccaatt gatggtgatg
14341 ataatcaaaa tagtgtgcct tcctctatag aaagtaaaga tgatactttt cctatgtcgg
14401 aaacattgaa gcattagatt aaaaaaatat tatcaatctc catttctctt ttatctggca
14461 agaatccacc aacttgtgca tcccataagg atatgacttg ctttgttgtt atgtcattca
14521 tattttttac ttattctatc tcccagttta attcaagtag atgatatatt ataatagtcc
14581 ttttcatgga aaccttttct ggtggattgc tttctagaaa aacattggtc aaggtgccaa
14641 ctgtggttta taataatata aattatatca tagttgattt gcgtttatca gagctgaggt
14701 atgttttatt ttacaaattt tttgcctatt ttactgttcc tatttctttt tgtaggaagc
14761 ggcatatatt ttactgttct tggcgtgtca aatgcttttt atcagttatc tccactagta
14821 gccattgttc ctgccatagc tctgggttgg gttttataca agggcaatac tgaagaaaaa
14881 atgtatgcct ttctagatgg ggtaagagat cgtaatatta tcactatgtg catcattttt
14941 ttacttgcag gtgcattcag tgaagtaact aaagcaatcg gtagtgctga tgccacagtt
15001 aatttggctc tttcatttat accaacacaa tttttactca taggattatt ttttgtagca
15061 gctttcatct ccactgctat tggtacatca atgggcacca ttgcaacaat tgctcctatt
15121 gcagttgaac tgacacaagc tgggatttct ccttccttag gagttgcaac cgttgtagga
15181 ggagcaatgt ttggcgacaa tttatctatt atctctgata ctacaattgc atctgtgatg
15241 tctcaaaagg ctgatataaa gaaaaaatta aaactgaatg caaaagtggc actaattgct
15301 tctatattca caataattat attattttat agctcaagca atagtgctgc tatttcagtt
15361 aaggagtatt ctttattgct tgtgactcct tacataatac ttatattgct tgcaaacttt
15421 ggaataaatg tatttacggc attggtttta agcataactt ttgctggaat cattggattt
15481 ataaataaca acaattatgg tttattgttt ttaagccaaa acatcactaa ggggtttatg
15541 agtatgcatg aaattatgct actgtcattg atggttggcg ggctttcagg actggtaggt
15601 aaaaattcta aggagcttgc agataaactt tcatcttggg ttatgataca acgtggtgga
15661 caaaaaactg cgcaattatt tattgcaaaa atagtcagca tatttgatat tttacttgca
15721 aataacacca tcgctataat ttttagtggt gaaattgcta gagatattgc taagaaatat
15781 gaaataccgc ctcactatag tgtagcatgg ctaggcatat tttcttgtgt atttcaagga
15841 ttaataccgt atggcgctca attgcttttg gctagcacta ttgctggagt ttcacctttg
15901 gcagtcgtgc cacacgtata ctattgctat gttttaggta ttattgcagt gttatatatt
15961 gtattgaaca aaattgagaa aaaagattgt gcttaatata caagacaacg ctccgctctg
16021 tagaaaatgg atgttcttgg cagtatgtgc tcttgcttgc tctgggttac tgtcaataat
16081 tgttattttt ctacgattgc cttttatctc ctctcttgtt ccttctgcac aatatatttt
16141 tgataatgcg ctggtaatac acgtgaactt gtcaatttta gtgtggatgt gctctataat
16201 atctctgctc tttatcataa acctacaaaa taccaatagt tggtttaact tttcatgggt
16261 tctatcaacc ctttcgatgc tgctcatgtt tatatccgca ttcgttccaa acactgaagt
16321 tatcaaaagt aattatattc ccgtgttaca aaacaaatta tttttgtttg gactcagttt

16381 atttattgcc agtatattag taaatacaat gctagcatac acatcaaaaa aagaggtttc
16441 atttctctct gttgggcaaa ttgggctagt tattatactt gtatcgtcat ttctttgctt
16501 tgttttagca tataataata tgcctccagg tctatatcac tcagataata atttatttta
16561 cgaatatctc ttttggggag gcggacattt attgcagttt gcttttactc aaggaatgtt
16621 tttagtctat ttaatgatca gtgatgttag tttagcttca agcaataaaa ttactatttt
16681 accactattt ataaacacga ttttagctgt aggcgcaccg tttgtatatt ttgtttatcc
16741 agttgatagt gccaagttaa tacagttttt tacttggcat atgaggattg ctggagcagt
16801 tttgccgtgc tttttgataa tattagtgtg ctgcaacatc agaactttta ttaatgataa
16861 aagcaactat ttattacatt catttttgtt gttcacttat ggagatgtgc tcggagtgtt
16921 gactattgaa ggaaatgtca ccattcccgc tcattatcac ggttctgtag ttgggatcac
16981 tatagctttt atgaatttca tttattggct gttacccaaa ttaaacttta aagagataaa
17041 aggttccatg gtaagattac agatttatgc ctacagtata gggcattttc tccatatcac
17101 tggtcttgtg tggcttggtg gatatggtgc tttaaggaag gttgcagatc tgccaagtat
17161 ttcatcaatg ttagcacgca cgtgcttcat tataggtggc gctatatcag ttgttggtgg
17221 aatgttgttc gtaataattg tgcttttaca tctgttaaaa ggcaaagcgc gtaccaactg
17281 acccatagaa acaaaaaaaa cttccttgac aaactccgcc agccccctta tcatgaaact
17341 gaagctattt atttatcttc tctgtacaga ttaaatgaca aaaaaactca cgtatctggc
17401 gtcccatgtt taattttttg cactatgtgc accttatgtc tttataaaat ttctaggttt
17461 ttacctaata taagctgaaa tgcgcttata aagcatttaa aacatcaaaa aacgccgatt
17521 taaaaaatgg atagtgaata actagctacc ctagggtttc ttttgccttt ttttctgttt
17581 agtaaatttc ttaatgttta taatttagat tagttgcggc ttaaaagcaa ctaaatcgcg
17641 gttattaagc gtttagaata aaaaaacgcc atacttgaaa gtataatgta agtaattagc
17701 caaccacggg gcttcttttg cctttttttt catttggtaa atttttaat atttatacgg
17761 aaggtagcgc gtgacgctgg aaccccaaaa aaaggatgat gtcatcccag tgctcctttt
17821 tttgtcatcc gagtagctga cactgtcctc ctttgtcatc ccagtgccca aacattggga
17881 tccagcattt cacgcaatcc catcaagaac gttgtatttt acataacact tttatgctta
17941 ccaacttaat aaaattcctg gatcccagtg ggctttgttg catcgctact tatgaaaggc
18001 taactataat taggattata agcaacttat tgaaaatctt gtttttttg caatcaatct
18061 gatcaaattt aagaatagta acttgttatt tatattaata aatttaattc tattgaaaat
18121 agctaaagca ttgaaattct tgaattttag ccggattagt gagtggtagt gaaatttctt
18181 ttactcaaat ttaggtattc actgaccgtt cttgttataa ataccagaat aataagctac
18241 tgatattctc cacctttttt atctttaatc catcatagct agcgatgcaa caaagccatc
18301 atagtcaagg cactggaatg acaaaaaaag gaggcactgg aacgacaaag tggagtgtct
18361 gcttctgtca tcctatgata gctaaaaaga cctggtcacg cgctggaatg acaccgaagg
18421 ggctactgcc ctcttggatg gagaccagtg tcagctactt agatgatacc ttttttctac
18481 ttagtttagg ttatgcagaa gtctaataca attatagaac aagcttaaaa attctggtaa
18541 agtagtttta tttacaaaga aagaatatgc tacttgaaat tgaaaagcgc agcataacaa
18601 atgcattgtg gaagttgcaa gaagcagatc aaaggcaaat cttaactctt acacagagat
18661 ttgagttacc agaaatatta gcaagaatat tggttactcg tggtgttaac atagaaaatg
18721 caagcgactt cttatatcca ctaatcagat cgctattacc agatcccttt caccttcttg
18781 atatggataa agctgtttct cgtataatac gggcaataaa aaatgatgaa aaccttgcaa
18841 tatttggtga ctatgatgtt gatggtgcaa cgtcgtcagc attgattaac aggtacctga
18901 gagcaatcga tatacactct ataatctaca ttccagatcg tgttgatgag ggttatggat
18961 taaacacaga tgctttatta caacttaaaa agaatggaat tgatttatgt atttccgttg
19021 attgtggtac gcttgcatat caaccaatag aagatgcaaa ggcttttggt cttgatatta
19081 tagttgttga ccatcacctt ggtacggaaa aattaccaag cgccgtagca gttgtgaatc
19141 caaaccgcct tgatgagagc tctccttata ataacctagc agcagttgga gtgtcatttc
19201 tgctaattgt tgctcttaat aaaagcctac gtgagcaagg attttttacc aacaaaaaag
19261 aaccagattt atttgatctg ttggatttgg ttgccctggg aactgtctgt gatgttatgc
19321 agatcacagg cctcaataga gcacttgtct cacaaggatt aaaagttatg tcaacaagaa
19381 aaaacgttgg cttgcgtgtt ttatttgatg ctttgggaat ccttgaaaaa ccaagtgttt
19441 cgcggttggg gtttagcatt ggaccgtgca taaatgctgg aggaagaatt ggagaagcat
19501 cgcttggtgc aaggttgctt tccaccgatg atgatgaaga ggcacattca atcgcgctaa
19561 aattaataga tttgaataat gcaagaaaag tgttagaaaa tgaggctctc ttggaagcta
19621 cagcacaagc ggaaaagtct gcccaatcag gtgcgaattt tataatgata agtggcaatt
19681 ggcaccaggg aataattggt ataattgcat caaggctaaa ggagcagttt cacttgccaa
19741 cagtagtgat atctttaaat aatggaatag gaaaagcaag ctgtagatca atttgggag
19801 ttgatatcgg tgctgcagtc ctttccgcaa agtttataaa tttgattatt gaaggtggtg
19861 gccatagtat ggcggcagga ttttcaatta aagaagacaa aataaatgat ttgcatgatt
19921 tttttactga aagattttca aactctataa atgagaaaac tttaaaagct gatagtatag

19981 taactgctaa agcaataaat ttgtctttat ggaatcaatt gcaacgcttg ggaccatttg
20041 gagttggaaa tcctgaacca aggtttatta tccaaggagc aaagataaga aagcctgaag
20101 ttataggagt tgaccatata aaatgtttta ttgctgatga taatgttatg gtaagagcta
20161 ttgcatttcg ttctgcaaat actcatcttg gctctgctat catgcagggt aatgttaaag
20221 ctattttggg taaaatctct atgaattact ggaatggcaa tgaatttgta caatttttaa
20281 tagaagatgt attgaccatt agttgagtgt atctgttcag cagagtggca aaataggtag
20341 acaagataaa ccaaaaaatc gagaaaaagt gagtttacaa caactggtgt catgaaatta
20401 tctgacactg atttccatcc aagagggtgt catcccagtg ctcctaaatg tcatcccagt
20461 gcgtgacact gggatgacac cctacttaac cgtcataccg cgattcattc gcggtatctc
20521 ttagccgcta acaagtagcg ggatgacgag cttatcgtca tgccactgcg aaccgtcata
20581 ccgcgattca ttcgcggtat ctctagatcc cgctaacaag cagcgggatg acggctgtcg
20641 cttagccata aacgttaaaa aatttaccaa acgaaaaaaa aggcaaaaga agccccgtgg
20701 ttattatccg ctttaaaata ttggcgtttt ttatgtttta aacgcttgac aagcgagatt
20761 cagccgcttt taattgcaac taacttacgc tgcaaatgtt taagaaattt actaagcaga
20821 aaaaaagaca aagaatcccc gagttagcta gtcttttact atctctgtcg agtattggca
20881 ttttttgatg tcttgtaacg ctttataagc gcgttcagct tatttagata aaaatctaga
20941 tgtagatgaa gttttgtaaa gacatacagt atctatatac tgcaaaaaat tgaacataag
21001 acgccgatac attaagtaat ccttaccttt taatctgcag attggcgaaa gcaaatacaa
21061 tagcttcact atcatgataa gggcgctggc gaagggtgtc aagtaagttt tttcgtttct
21121 atatgcgcta ccttaagact tcaacgtctt aacgcaccta actccatctg catataaatt
21181 tgcatggctc tttccagtat taagcaatgc aaatccaatg ttgtctacac tagagcgcag
21241 ttcccctatt tcctcattgt tttcatttgt cactttagtg ccaatatctg gaagtgcgtt
21301 atccccctca acaaggtaaa gttttcttct gaatatttct tgtctgctca ttcgatttac
21361 aacttcctga cctatataac accccttatt aaagcttata ccgtttacct tatcgattaa
21421 aaattgcagc ggaaacgatg aattctgcac catatctttc gctccatctg gaacgagatt
21481 ttgaattcta actttttcat actgagtaaa atccccaacc ggctctttta tttcatcttt
21541 atgtatgatc ctcattccta gcagcttgtg ccttggatct tgaaaaataa cttgtgactt
21601 accactacac tccgccaact tagtattaaa caaaactcca accttatata gtgcgctaac
21661 gtctttaatc ttcactctca agtaagtttt aagcaaatcc agtttctcga ttatttgctg
21721 caagtgcatg ttttcgcact ctaagagggt gtatttacca tattcaataa gaaaaaaatc
21781 atatagatat tttccctgag ggctaagcaa taaagagtaa atggcttttt ggctatccaa
21841 cttattaata tcattggtta taataccctg cagaaaatct ctagtgtcag gcccatatag
21901 cactatcaca ccacgactta aaaatggtat ataactcatg aaatctctat aaataaatct
21961 aaattttatt atataacatt aggctctttt agaaactgct tttggcgtgc atattttata
22021 atggcgtgca tattttacaa gctccagccc ttgaaatcta acttacgagt atatatatat
22081 aaacttataa gttaatttgt tgatgaggaa aaacaaatgt caagtataag tttaaatgta
22141 ttagatgata gcgtgctgat caagcctatt agcgaagaaa agcaaggtgg aattgtgctt
22201 ccatcaagtg ctgaaaagaa acctactaaa ggtgaagtta tagcaattgg tgaaggttca
22261 cgcaattcaa gtggcgaacg cgtaacttta actgtgaaag ctggtgataa agtgttctat
22321 cgtcaatggg ctggtacaga aatagaacat aatgatgaaa agcttatcgt gatgaaagag
22381 tccgatatac ttgctgtcat taaatagcag tctttatttt actaaactta aacaaagcgc
22441 aacatgacgc attgtgaatg attttattat agttttctaa atttttttatt aacaagaggt
22501 gataaaaatg actaatgtag tagtatcagg tgagcagttg caagaagcct ttcgtgaagt
22561 cgcagcaata gtagactcaa cggtggcaat aactgcggga cctagaggaa aaacagtagg
22621 gattaataag ccctatggag caccagaaat tacaaaagat ggttataagg tgatgaaggg
22681 tatcaagcct gaaaaaccat taaacgctgt aatagcaagc atctttgcac aaagttgttc
22741 tcaatgtaac gataaagttg gtgatggtac aacaacgtgc tcaatactaa ctagcaacat
22801 gataatggaa gcttcaaaat caattgctgc tggaaacgat cgtgttggta ttaaaaacgg
22861 aatacagaag gcaaaagatg taatattaaa ggaaattacg tcaatgtctc gtacaatttc
22921 tctagagaaa atagacgaag tggcacaagt tgcaataata tctgcaaatg gtgataagga
22981 tataggtaac agtattgctg attccgtgaa aaaagttgga aaagagggtg taataactgt
23041 tgaagagagt aaaggttcaa aagagttaga agttgagctg actactggca tgcaatttga
23101 tcgcggttat ctctctccgt attttattac aaataatgaa aaaatgatcg tggagcttga
23161 taatccttat ctattaatta cagagaaaaa attaaatatt attcaacctt tacttcctat
23221 tcttgaagct attgttaaat ctggtaaacc tttggttatt attgcagagg atatcgaagg
23281 tgaagcatta agcactttag ttatcaataa attgcgtggt ggtttaaaag ttgctgcagt
23341 aaaagctcca ggttttggtg acagaagaaa ggagatgctc gaagacatag caactttaac
23401 tggtgctaag tacgtcataa aagatgaact tggaatcaag atggaagatc tcacgcttga
23461 tgatcttggt acggctaaaa atgttaaaat tactaaagat aataccacag ttgtcagcga
23521 aaatagcgat tctgacagtg tgaaagctag aatcgagcag attaaatctc aaattgaaac

23581 ttcaacttct gattatgata aagaaaagct aagagaacgt ttagcgaaat tatcaggtgg
23641 cgttgctgtg ctcaaagttg gtggagcaac tgaagtggaa gttaaagaac gtagagatag
23701 agtcgaagat gcgctgcatg caacaagagc tgcaattgaa gaaggtatag ttccaggtgg
23761 tggggttgcg cttctttatg cttcatctgt tctcgacaaa ttaaaaggtg caagtgacga
23821 ggaacaaata ggcataaaca ttatcaagaa agttctcagt gctccaatta gaaggttagt
23881 caaaaatgct ggtcttgaat ctgctgttat aattgactat ttgattaagc agaatgataa
23941 agagcttata tacaacgttg aggctatgaa ttacgctaat gcatttacag ctggtgtgat
24001 tgatccagca aaagtggtgc gtattgcttt tgaaacagcg gtatctgttg cgagtgtgct
24061 gataactact gaatctatga tagttgatgt accaagcaaa gaaaatgctt catctcctat
24121 gggtgcagga gaaatgagtg gcatgggtgg attctaagta gaatgaaacc gtggagcaat
24181 tgctccacgg tagttccaaa aaatctcaca ttttactatt cgttaaaggt aatacgtttg
24241 gtgcagaaat gcactactgt ttgcatccgt ttcatgcctt tatattgtgg ttgtctaata
24301 acaaaaaggc agcataaaag aactataatc cctagtatat ttatactata gctaacacgt
24361 cataccgcga ttcattccac aactgtacga acattacaat atggcacata gtaaacgatg
24421 tcatgaaagt agctgacact ggaatgacac ccttttggat ggaaaccagt gtcacgcact
24481 ggcatgacac ccatttgttc ctatagttgt cttttctcgt ctaccttatt ttgccactct
24541 gctgaacaga tacaatttac aaccaccccc ctaccctttt tagttgttca gcaaatatag
24601 agaggttaaa gggagtggta ttacttaaac aactaaataa aaaaccaggt agatcattct
24661 gaaattcttg catttttgtc cataattttg ttaaataact tccctttttgg tcatatcgaa
24721 ccaaatttct ttttcacacc accttttaag ccactttcgt tagtttttgt cataatgcga
24781 tcaaacactc ccaataacct ctttaatagg tttggagtta gcgaagagat aagatcaaca
24841 gatacgttat tgtttttgtc atcaccactt gctttatagt ctattgaaac gattccccta
24901 cttttcccac cagtaagaag ttttccaatt ataggaattt ttaacaaaga tttattaatt
24961 gaatatgctg gtattacttg tcctgcaacc tggaatttat aatttctaat atcgagctta
25021 ccaccagtgc taatacctaa ctccgcccct tcaagccaag attcttcaat ttcaatagtg
25081 ccatctttgt atgaaaaagg tgcattacat ttgtaaaagt atacaccttc atttttatg
25141 gcatttacaa tgccaggcaa tgaagacatc gacaataaag tagtaagtag tggagcatct
25201 ttgatgtaga aattgctaat ggataacgta ccataatgtt ctccactttc tctctgagga
25261 gagagataaa aagacaattt gccgtttttta attgatttac taatgcctaa ggaacgcaaa
25321 agcatacctg aattatccgc atatatttcg agccctattc cactgtattc tgctagtatg
25381 ttgctgctat cttccagaaa ctgccctgta aattgacttc cattgcaatt acctttagta
25441 caggttacat tcagcttagc atttttgata acgatgcctt ctttcataat tacattgtca
25501 acattcatac taatctctat atctttattt aatccattac tgtttttgcc aagcaagcct
25561 aaaatatcac ttaagttaat ttcctcacca taaatagtta tggcattttt ctccttacct
25621 gattcaattt ctatactaaa atcattatca ggcaatttaa agctactaga atttaaatat
25681 aaatttccat tttctactct tccgctgaat tttatatcta ggtcatttcc tacaatgtct
25741 aatttatcta ctaacaattc acctgctcct tttaaccttg tagaaaacaa aattttattg
25801 tgatcttcaa agcgattttt ccaccctaag tagcttgaat gtgattcaaa ttctgataaa
25861 tctatatcac cattaacata ccccgtctta tcttgattta tcactgactc taaatttgca
25921 ctgacatagc cactatcaaa attaaatatt tgagcgggta aatctccggt caaattccag
25981 gcgaaacttt cattcctatt gctacttttc aggtcaaata atagctgtgt gttattcacc
26041 ataccactac catttaaatc tataaagtca ctgccaaaac taagcttaat atcatattta
26101 ccaagacttg cattatagac ggccaaattg tcaatttcag aatgaaaatt tgctgagaag
26161 tctacttttt tgtcatcagc atttagatta aaaatgcgaa aattaaatac ggattttgcc
26221 attccactta ctttatccct ttcaaccttc accacatcat ccagcttaaa tcttataggc
26281 tcatataaac catacgcatc acttacagct ttgccattaa tggttagaac tgaatcttcc
26341 ttatttagag atttcatttc aatatcacca ccattaacag tgaaattctg aaacttagcg
26401 ctatttacag taatttcaag atcgttattc ttgatgatca aatcaccctt taattccttt
26461 acttgctcaa aatcttcatc aaattttacg ctaccgtttt ctatatcagc aacaattaca
26521 atgtctgaca gatcgtcatc aaccaaattg ttaattttgc cattgaaact tacaatggta
26581 tttaaaacat ccccatcaat attatcacaa taccagcttt taaatttact atttactaca
26641 ctatctggta tataagtaca taaatccttt gcagcaaact tactaatatt aattctaagc
26701 aaagcatgac ttgtaccaaa gttcattttg cctattaaag agagatacgt gtcatttaac
26761 ttaaaatgga agtttttttac actaataatt ccattactat atgttaaatt tatatttaca
26821 ttagttaagg ccaaattttt gttcaaataa ttttctgtat tcaatacgta tatatttcca
26881 tctacaattt catccttttt attaattttc actgagaagc ttcctttgaa tcctatattt
26941 ttgtccaaat tgtagctttt aactaatgta gaaaactcgc ttaatagtcc caattttaga
27001 tcataaaatg tcccgtacac atttagcaaa ttgttgcgat tttttatcgt aattgataaa
27061 tcatccaaaa atccctttttc atcttttgta ttcacatgaa tatctaaaac attaaaatct
27121 tccccccttcc ctatatacaa tttatcaata aaaaattcat cttccgtgcc tttattaata

27181 gcgatgttag taaattcaat ttttgaatcc gcatttaagt caaaaaaaaa ttcccttatt
27241 gtttttaata gattttttgc actgctttct gtcatcttga aattcttccc tgtcatccca
27301 gtatcttttt ttcctgtcat cccaatatct tttttttcctg tcatccaagt agcttgacta
27361 cttgtttctt tttttctaga ttccagcgtc acgcgctgga atgacaccga aggaaggcgg
27421 ttattactta aataatgatt atccaaaagg tctaatgagt tttgcggatt aacattaatt
27481 tcttctctct tgatatatac acgcacatta tcagctaaaa cttgagaaaa ttttatgtta
27541 cctttaaata aggaacttaa cttaaaatgt acaaaaagct caggaacttt tatagtaaaa
27601 ttagggtttg ctattgccaa atctgtaatg actaaatacg gatctttgct acctttctgc
27661 caaacaactg agatattctc cacattaacg cttgagccaa caaatatttt tgatatttta
27721 tgtttaacat aaaaattaac ataattaata ttgatttcta aaggctttaa aaaaataaag
27781 aagcacaata tgaacaataa agctacggaa aataatacag taattttttt aagcattgat
27841 tattgctttt cttgctaaac tatcagcctc ctcattatac ttatcgccac tgtgtgcttt
27901 aacccacttc caatcaattt cgtgttgtaa agcaacgttg tctaactctt tccacaattc
27961 catatttttt actgattttt tattacttgt tttccaacca ttcattttcc acttatttat
28021 ccactctgtt atgccatgtt taacatagag actatccgta tataaattaa tattacaaga
28081 aaatttcaat actttcagcc cattgatcac cgctgttaac tccattttat tgtttgtggt
28141 atttcttctt ctaccataaa tgtcttttct ataatcttga aataatatga tagctgccca
28201 tcctcccgct ccaggattac cagaacacgc cccatccgta tatattgtca cttccttttt
28261 gctcatcttt aactgtaatg aattaataaa gtatatagtt gcttctatga aagcacaatt
28321 tctaaaggta gtttaagctg cacctttaaa ttttattata gaaacaaaaa aaagtgtgac
28381 aacgagcatt accaaaagga aaagtaaaaa atagtttttta tgcttctgcc gttctttcat
28441 aattaaaata aatcaataga acaaaaaata atactggcga atagggaaaa tagataagaa
28501 attgaataag aaaacatttt tttctgtgag ctgtggttct tgaatctgag aattgatata
28561 gcgtaccaaa taaaaacgca cccttcaaag gttgccatgc ttagataaag taaagccttt
28621 ttcaagaata gagctggaag caagctagtt agcaccagca aaacactata aattaatata
28681 tattttcttg ttttcttcgg accataaaca atattgaaca ttggaatcga tgcctttaca
28741 tagtcttcag acttatttaa agataggggcc caaaagtgtg gtggagtcca cataaagatt
28801 actaaaaata aaatgaaact ttcccaactt acagaattag tcacagctgc ccagccaatc
28861 attggaggaa aagcacctgc tgcaccaccg ataacgatat tctgcggagt gcgtcttttg
28921 agccagatcg tatatacaaa aacgtaaaac aatatgctga ttgcaagtaa agcagcagaa
28981 atatagttta ctgctattgc catgataaat actgataata ttccaagagt gataccaaat
29041 tcaagcgcgc tttctgcaga aactctacct gagggtatag ggcggttttt tgtcctttcc
29101 atgagcagat ctatatctct gtcataccac atatttatag cacctgcaga tcctgagcca
29161 agagcaacac atataagaga tattaatgct agaaaaggat gcataccacc tggtgcagca
29221 accataccgg caactgcagt aaataccaca agatacatta tccttggctt cagcaaacgc
29281 caaaaatcca gtattgttga ttcaacattt agcaaaacac ttgtgtacat tctattttac
29341 cactggtggc ttttcgaaag tatgaaaagg cggtggtgaa gataccgtcc attccaaggt
29401 gtcacctttc caaggattat ctccagcttt cttgccccat ttgaagagat gtataactat
29461 aaacacaaaa aacataactg aaacaaagga catatatgag ccaattgagg atatataatt
29521 ccaagggata aacgcatcag gataatcagg tatacgtctt ggcataccag ctaatcccaa
29581 gaaatgttga ggtaaaaaag tgatattggt gctaataaaa gtaagccaaa agtggatttg
29641 acctaagcgc tcattatatt gtttacccga cattttacca atccaataat aaaagccagc
29701 aaaagctcca aataatgcag caagtgacat gacataatgg aagtgagcaa caacataata
29761 ggtgtcgtgc aagagcttat ctattccacc atgagaaaga attattcccg ttatgccacc
29821 gccaacaaac atgaaaataa aacctagtgc aaatagcata ggggtcttaa actcaattgc
29881 tccaccccac atagttgcaa tccagctaaa gacttttaca ccagttataa caccgataaa
29941 aattgtggta gtgctaaaaa atgcagcagc gtcagcacta agcccaacag tgaacatatg
30001 gtgagcccaa accataaagc caaatactgc tatacctatc attgcataaa ccatccctat
30061 gtaaccaaat acaggtctgt gagaaaaagt tgatacaacc tgacttatga tgccaaatgc
30121 aggaaaaata attacgtaaa cttctggatg accaaaaaac caaaatagat gttgaaataa
30181 cacaggatcg ccgccaccgg caggatcaaa aaaggaagta ccaatattgc gatcagtaag
30241 aagcatagtt atagcaccgg caagcactgg taaggcaaca atcaacataa atgctgttaa
30301 caagacagac caaacaaaca gtggcatctt agttaatgac atgccttttg cgcgcatgtt
30361 aaatatagta actataaagt tgatcgcccc aacaattgac gacataccag caacgtggag
30421 tgcaagtata gcaatgtcaa ctcctgcact tggatgggac attacctgtg ataaaggtgg
30481 atataaagtc caacctgtac ctggaccttc accaataaac actgagagaa tgagcaaaat
30541 aaaagatgac actaataacc agaaactcaa attattcata cgaggaaacg ccatatctgg
30601 tgcgccaatc atgagaggta caaaccagtt accaaatcct cccatgaggg ctggcattat
30661 cataaaaaac accattatca acgcatgccc tgtaaccatt acgttatata attgatagtt
30721 attgttaagt atattaatgt gcattagctg agtgcgaata atcaccgata ataatccacc

30781 aataattcca gctaatatgg aaaaaataat gtacagtgtc cctatatctt tatgattggt
30841 ggaaaacaac caacgcttta tgccctttgg tacgtcactc atatctatac tccaaattta
30901 actcactaat tttctatttt caatccactt attaaaatct tctttgctta ctgcttcaac
30961 aacaattggc ataaatccat ggccttgacc acacaattcg tagcactgcc cataataaac
31021 accaggcttt ttgatattaa accacgcctc attcagccta cctggtatcg catcaatttt
31081 tacaccaaaa gctggtactc cccagctatg tataacatct cctgctgtaa cttgtaaacg
31141 aacgttagca ttaacgggta aaacgatgtt gttatcaaca gagaacagct ttaaatctcc
31201 ttcgataaag tcttcttttc ccttaatata actatcaaat gacacacctt gatattctgg
31261 atattggtaa ctccaatacc attgatggcc aataactttc agtgttatat cagctttcgg
31321 tatttcttcc tgtagcttca gtaatttagc gttttcaaag gctaatatac caacaatgat
31381 agttggtata acaaaccaaa taatttctaa aggaacacta tgggtagttt tacttatgtt
31441 tgttactttg cttttacgaa agcgaaacgc tatataagca agcagcgccc acacaaaaag
31501 cattattgta accatcacaa gcatcacaaa cgaatgtgac ctaactacag cctccattac
31561 ttcagttgca ggagcaggaa atccaaattg ccaggaagta ggagcagagg caacagatat
31621 atttgagtaa aatattatca atagtgcaaa taacttcacc atatcttttg tttgtttgct
31681 ataaaactaa gtgatatact gtataatagt atatataaac ttactatgca aatagagatt
31741 agtgatggtt aataattttt tagcattcaa caagtataat agtacaatac taattgagga
31801 aaaaagcatt tacataaatc tattgtaagt aaaaatatta taatatttta aattaagtat
31861 ttactttaag taaattttat aataagttta atagtgtctg ttactgctaa taaccctcta
31921 gcctcacacc taaaagcagt aacagacact attaaacttt atgacaagtt tataatctgt
31981 aagatcaaag tcttacaagt ttctttattt aattatacat tgcagtttgt taattaactt
32041 ttaatgccta tggtggcatt tacagcttgg accaaaggtt tatgtattat agaaaataat
32101 tataaaaaaa acacactatt tgtataccat acaaactttt tttattctgc atagctgagt
32161 tactagattc tgtaaacgaa atctttagag aagccaaatg ggcaaagttt catgaaggtc
32221 tttggcatct cttagcatag atcccttact taaccatcat accgcgattc attcgcagta
32281 tctcaaagca tagatcccgc taacacgtag cgggatgacg attgtcaggc tagctgtcat
32341 cccagtgtca agcactggga tgacaaggta taaaccttat cgtcatgccg ccacaaaccg
32401 tcataccgca attcattcgc ggtatctcta gatcccgcta acaagtagcg ggatgacgat
32461 tgtcgtttag ccataaacat taagaaattt accaaataaa aaaaaggcaa aagaagccct
32521 ggggttatta tccgctttaa aatattggcg ttttttatgt tttaaacgct tgacaagcga
32581 aattcagctg cttttaattg caactaacct acgctgcaaa tgtttaagaa atttactaag
32641 cagaaaaaaa gacaaagaat ccccgagtta gctagtcttt tactatctct gtcgagtatt
32701 ggcatttttt gatgtcttgt aacgctttat aagcgcgttt cggcttatat aggtaaaaac
32761 ccagaaattt ttaaagacat gcgatgcacg tagtgcgaaa aattaaacat gagacgccaa
32821 ataccctaag tttgactcgc gacataatgt tgaacatttt tttttgcatt tccagagcaa
32881 gttcttgaaa cctaaacatt atatgtacat catcatgatc aattctaatg catctaacta
32941 atgctatgat gaccttaccc agaaaaagtg gagagaaagt gtggaatgtt ttttacaaaa
33001 agaatgatgc atcaaattgt tctggaatgc aatagttaat agtgacgttg tttgttataa
33061 aaaatttcta tatattcaaa tatagcagtt ttgatatgct gtttagagtg ccgagaggtg
33121 tctattggta actccctttt agagaactga agaagctttc cacaacagaa ttgtcgtaac
33181 aacaaccttt atgactcatc tatgtttttt atggataaga gatattgata accttgcaaa
33241 gtatattgtg aaccttgatc actatgtaat agtagatttt tggcaggttt acgcttgtta
33301 acgtccatta ataaagagtc cataaccaat tgtttattta ttgaactgct cattaactac
33361 catgcgtgaa tatagatcga ttattgttgc caaatatagc catccttctt tggtttttat
33421 ataagtaatc ccatacttta tttggttgat cgacaataaa gttttggtct aatatattgg
33481 gagctactgt ccgtttgttg tttcttaatt ttaaattttc ttctcaatat agcctgaata
33541 ccatttttct gcataatact ttgcactgtt tttaagttgc aacttttacc caaagccttt
33601 aattcagcat gaattttagg agctccatat ctacatttag aagcttgata tattttttga
33661 atatctgcta ataactcttt atttgctgat tctctactgc ttatttttct tgtggtccac
33721 ttatagtagc cactagcaga aaatcctaca taattcctgt actttatagc agttactata
33781 taaaaaaata ttttactctt tttgactggc cagggctttt tttaaaatgt ctctttctct
33841 tgttactttt gctaactctt tctgtaaatc aaatctctct ttgtcataag gtgctacatc
33901 tggaaatgca tttgccgctg actttttttcg ttatatttct tcatccattt tcctaataca
33961 ctatccctta ttcctagatc tcttgctact tttgcaactg gttgacctac ctttctttgt
34021 tcatttgcca gcttaacagc ttccaatttg aattctgccg tatattctct tgccatttct
34081 aatcctccaa aattttattt tacccgaaaa aactcctttc tttctctcca ctttttctgg
34141 gtaaggtcaa ttcggaactc tatttctttt ttacgatctt tacttttatt cttattgttc
34201 atataatata cccttcacta ataataaatt atttttaaat gtttactgtt attttcttag
34261 cagtattggc ataaagccat agcttttggg tgaagtcaaa tcaaattttc aatttttttt
34321 gtttattttt ttatgaaaag gtagaagata ttaatagctg ctaatttctt taaacgatgt

34381 tgtcgtagat tcgtattttt atacaccaaa aagggtatct ttgatatctc ctacagctcc
34441 aaactcatag catccatttg ccagcctttt aaggcgcttt atagaagggt taataaaaga
34501 tgtaaaaagg acgtctaagg tgcgtagtag cattctaaag ccatctatgg gagacatttt
34561 tttcatcaac gcattcaaaa cttaaaaaaa taaaaaactt gaacatttct tcagaattgt
34621 gtataattat taatgctttt tgtgtgccgt agagatacaa ggaatgttca ggaaagagca
34681 aactaagtgc gaaagctgca caatagatga gggtggaccc ctcggagccg gtttacaaga
34741 gcaggcttca aacaaccata agctgctttg gtaaagagcc aaggtagtga gcgttatgga
34801 aaaggcgtaa ttatgcgtca tgtgtgaaat agagagatga acgcaagtga accactgatt
34861 acacgtcgaa aggtaatgcg atgatgtcaa aaccagggga gaaaaagtaa cttgggataa
34921 gcctatcggg aacttgtcta ctgggtaggt ggcatccggc atataggtgg catgaaccta
34981 tttcaggcta ttgtgtggaa ctacgggaac ctgtcgtttc gatgataagg gagaagtcca
35041 aggagctgaa ctctaaggaa gagagtactg atgcgaaaaa caggggcgga ttagcttgta
35101 gtagtgaaga agtttttgta atggaaatgg agcgaagggg ctgagttatt tagttttaat
35161 tatttatcaa ccgaaaggga agagttaatg aataaaacaa aatcttttga cataccaaag
35221 caactgattt ggaaagctta taaacaagta tccaaaaaca gaggtgcggc aggtgtagat
35281 gaggtctcga taacaaagtt tgaagaaaat ctaaaagata atctatacaa actgtggaat
35341 cggatgtcat ccggaagtta ttttccagaa tcagtaaaag ctgttgcgat accaaaagat
35401 acaggaggac aaagaacttt atgtgttcct tcagtattcg acaggatagg gcaaacagca
35461 gctactatgt atctcgaacc gttagtagag ccggaatttc acgaagattc atatggttat
35521 agaccaaaca agtctgcact ggatgcggta agtacagcgt gtaagagatg ttggagaagt
35581 gattggacga tagatcttga tatttctgga tttttcgaca atctggatca tgtgttagca
35641 ctgcaaacta tcaagaagca tacagattgc aagtgggtca tactgtacgt ggaaaggtgg
35701 atgaaagccc cgattcaact agcagatggc agtaaggtag ttaggaataa gggagttcca
35761 caaggaggtt ctgtaagccc aatcatttct aacatattta tgcatcatgc atttgatatg
35821 tggatgaggc aaaactaccc aacaataccg tttgagagat atgtagatga tgcaatggtg
35881 cactgtaaaa cccagaaaca ggcaaaattc ataaaagaca agatcgaaga aagattggct
35941 gagtttaagc taaaattaca tcctgaaaag acacaaatag tgtactgtaa ggatgacaat
36001 aggagagatg agtttccaat acagagcttt gactttctgg gttacacgtt cagacctaga
36061 ctggcaaaga acaataaaat aggaaactat tttgtctcat ttctacctgc aattagcaac
36121 aaagccaaga agaagatcaa ccaaaccata aggtcatgga aaatacgtag gcaaacatat
36181 acaacactag agaaaatatc aaagaaaata aatcctatag tccgaggctg gtatcagtac
36241 tatggcaggt tttataaatc agagatgtat ccatctctca gaaatgtaga gtggcaccta
36301 gtaggatggg tcagagccaa atataagaaa cttcgaaatc atagaagact agcaaagcaa
36361 tttctagcaa aagtgagaaa gaggtctcca aatattttct atcactggac actaggattg
36421 ggatcaaaag gctgaataac gggagctgta taaattgaga ggtttacgta cagttctgcg
36481 agagactggt ggggaagctc cgccggtcta ctctccaagt ggatttccta ttgttctttc
36541 taagttcctt ggtgcacgta acgattattc tttaaaaaga atattggcta ctgagaagaa
36601 tgaagacatc ttcatcagaa aaaaaagggg caaaaacaga gaaaattgac gtagctactt
36661 aaggcaggtg tctctattgc agctgagaca actgatctac tcatgatcaa atgaaaaagt
36721 tataagcata cagattggat tacagtatct tttttaagaa gaagatttat cttgcttaaa
36781 aacttgtaga tactatattt tacatacatt cttcatgcga ggcctttatg tttactaata
36841 gaatatctaa taatctggat cagctgcaat tttttgtaga gtttttaaat gaaaattatg
36901 aaattcagaa acatcttagt cttacaccac tacatttagc agcaggaaat ggtcagttgg
36961 atttagtcaa cactttatta ggagaaggtt tagatattaa ttcagagatt aagtacgatg
37021 gctttacgcc actgtatttt tcaattgcaa aaaatcgctt agagatggtt aactttctta
37081 ttgctcatgg agcagatgta aaccataaga ccattctagg ctttacacct ttaagttttg
37141 catcccagca aggatattta gatatagtca acaccttgat tgcaaatgga gcagatctta
37201 gtactaaaac agataaactt aatacacctt tacacctagc agcagagaat ggtcatctag
37261 atatagtaaa tgtttttatt gaaaagggat tagatgttaa tgctgtaaat aatgatcgag
37321 caagaccttt gcattctgca gtacaaaatg gtaacctcga agtagttaaa gctcttattt
37381 cacaagggtc taatattaac gctggatctt caggcatagg taatcataaa gttgatgcaa
37441 acattacacc cttacatctg ggaacacaaa ctggtaggtt agatatagtt aaagttctgc
37501 ttgaagcggg agcaaacgtt aatgcaaaaa cagatgataa gattacacct ctacatttag
37561 cgtctcaaaa tggcttttta gagttagtag acattttact aaaagcaaaa tctaatgtta
37621 atgctaagga ttatgagaat cttacacctc tacatttagc agcagaacgt aatcactttg
37681 gagtagttaa gtctcttctt ctcgtaaggg gaattgatgt taatgctaag gaccacgata
37741 attccacagc tttacatata ggatctcaga atggtcactt agaagtagtg aagctactaa
37801 tagagaaaaa agccaatgtt aatgctaaaa aaaaacgaag gttttacacc tttgcatcta
37861 gcaattcagc aaagtcattt cgaggtgagt gatttttttaa ttaaaaatgg agcaaacatt
37921 aacaccgtgg atgatcagaa ttggactcct ttacataatg cagcatataa tggtttttct

37981 ttaaaaatag tagagagctt aattgcaaaa ggagcaaata taaatgcaaa gatggatgat
38041 ggtagaagag ctctacattt ggcagcagaa cataatcgct tggaaataat gaatttcttg
38101 attgaaaatg gagcagatat taatgctcta gataacagaa gttggactcc tctacattgt
38161 gcagcatatg atggtaattt agaagttgct aaatctttgc ttgataaagg agcagacatt
38221 aatgctaaaa cagttaaaag tactacacct ctacactttg cagtagacca tgatcattta
38281 gaggtggttg aattgctctt agaaaaggaa gcagatatta atgctttaga tcacacaaat
38341 tggactcctt tacattttgc agcagaaaaa ggttatgatc agatagcaac cgttctattg
38401 aaacatggtg ctgatgtaaa tgtaaaagaa aatcaaaata aaggcacagc cctacatctt
38461 gcagctcaat atggtcatcc taaggtagtt aaaactctta ttataaatgg agcggatgtt
38521 aatgcaaaaa tggataaaaa tgctacgcct ttacacttag gggcacaaat tggtaattta
38581 gatatagtta ggtctctact tatgagcgga gcatacttta atgctagagc tgaaggaggt
38641 agatatgttt taccattaca ctttgcggaa agaagaggaa acccagaagt aataaaatta
38701 ctaaaattgg ttgagaagtt atttaaggct atagaagata ataattattt aggaattgag
38761 agttccatta gagatggagc aatcattgat tcgaagaacg tggatggaag aacgccatta
38821 cattatgctg ttaataatgg gcacataaaa gttgtaaata tcttactaac aaatggagct
38881 aatgttagtc aagttactaa taaaggtaat acaccattac acactgctac ttccaaatgt
38941 tacaaagaaa ttgttgaggt tctgttacaa catatcagtc gtgataaatt aaatgacttt
39001 gttaatgcta aaactacttc tagtggtact acatcacttc atgttgcagc taaaggtggc
39061 tccttggaag ttgtaaaatc tttattaaaa catggtgcaa tttataacat caagaataaa
39121 gaaggtaaag caccacttga tctttctcga gaccaaaata ttactaacct attgaaatta
39181 gtagaagagc tgtttgagaa tgcaaaaaat ggtaatgttg aaattatcag taagctgaaa
39241 gcaataaaac ccgatgagcg cgtagctgta acaaatgctc gtgatgatca aggcaagtcg
39301 ttagtccagg ttgcagtaat caataaacac tcaaaccttg caagtagatt attagaaata
39361 ttaaaaagtc cagatcagag tttacaagat gtcagcgtag aaaatcgagt aaagagtttg
39421 aagttatagc tctcttaaat gaaactctaa tgagggtgcc aagatctaga tcatttagaa
39481 aattatagtg ggctttacat cttattaatt ataaattaac ttactatcta atcaatccct
39541 aagtcatgta tgagtaacat tcggcgtcaa gactcttgcc agcatagaaa atatcagttc
39601 ctatataaat atccttatgg gctaaaagaa acttctattg gttgatagaa aacagaaaaa
39661 aactatttaa aaagtatact caacaatata acaaggaagc ataagttagg gaaagtagta
39721 ggccgttgat aaggatttgc ctattttctc gatcaactag cttttgtata agtactgatg
39781 ggtcaatata aatgagtagt tctgggaagc ttactttatc ggctaatcta tattagcgtc
39841 atagaggatt tatatatttc aagcatgtta attatttttt ctggatcatt taggaaacta
39901 tagtagactt tacaactcat taattatcac ttaacttacc tcctagtaag ttcttgagtt
39961 atgtatggat aacacgttga taatattagc agcaggaaag agcagtagaa tgaattctga
40021 atatccaaaa gcgttgcacc aagtgggaaa tcttactctt ttagaacata taatttctaa
40081 tgcaaaattg ctgagtctaa aaagcttatc aattgttgtt aacaactccc ttctaaaaga
40141 tttagaaaag tttgataccc taaaaagtat aatcgatcaa tacaatataa aactaataat
40201 tcaagaagat attacaggta ctggcactgc agttaaaatt gctctagaaa atctggagga
40261 gttatctgat caagacatag ttttaataca gtatggagac actcctttta tatctagtga
40321 tacagttatg aaaatgactg attgtttaaa gtgtaataat aaaaatttag ttcttcttgg
40381 atttaatagt caagataagc aatatggaag attagtaatt gatgataatg acaatgttca
40441 aaaagtcttg aaaagtgaag atgaaattct tcttgcaaac tctggaataa tggtttcata
40501 cgctaaagat ctttataccc taataaaaga agtaaaattt aatgattcga ccaacgaatg
40561 ttgtcttaat gatataatac ccattgcagc aagcaataac ttacgtgcag gttatgtagt
40621 cgctgacgaa aaggaagcaa tgggggtaaa taacaaagag gatttggtca aagcagaacg
40681 ttattttcag gcaaatagaa gaagacaacc aacccttctt tcataactat atttttttat
40741 tcattgatcc ttaatttaag ttgacatttg ttttatacta gttttataat gctttttaag
40801 tcttacttgt aagatgatag gtgtgaaaga ttttcctggt tgtagtgaga gggacacatg
40861 ggaatattaa taacaggtgc tgcaggtttg ataggatcgg ctttagtaga aaagttagaa
40921 aagcagggtt atgaagtaat aagctgcgat attaggtttc gcgacaaccc actcagtttt
40981 ttttcagaag atataatgcc gctactcgct aagtgtacgg gggttattca tctggctgca
41041 atttctagag ttatacatgg tgagctttat cctaaattgt gccaaaaagt taatgtcgat
41101 ggtacaatcc aattcttaga attctgcaag tcgcttccga ataaaccatg gtttatatat
41161 gcaagcagta gagaagtata tggagaacaa aaggaactac cagttgcaga gtctgctagt
41221 attaacccaa taaataatta cgccaaaggt aaagcgttta ttgaagaaca agtaataaat
41281 ttaaaaggtt ctaactttaa tgtagcaata ttacgcttct caaatgtata tggtggttta
41341 ctagatcata gtagtagagt aattcctgca ctctgtatca atgcattgaa aggcgatcca
41401 attaggatag aaggtaaaga atgtgttttt gattttacct atttggatga tgttatagag
41461 ggtatatact taactgttaa atatttacaa aacgagaaat cttctcttcc tgctattcat
41521 cttactacta atagcccatg tactttagaa aacttagcaa aaacaatatt aaaagtcacg

41581 gaaagtgatt ctagaattga tttttatcct ccaagaaatt ttgacgtaac taagttccat
41641 ggtgatttta ctagagctaa agagctactt ggttggtctc caaaacattc attagaagta
41701 ggattaagta aatttataaa aagtctacaa aacaatacac aagaatatcc taacaatata
41761 gatatggtaa tatatgaaaa tattaaaagt tattcatggt taccctcctt attatagtgc
41821 tggttcagag gtttatagcc aaactcttgc tcatgaacta gcaaataaca atgaggtaca
41881 agtatttact aggtatgaaa atagcttttt acctgatttt tattacacta cagtcctaga
41941 tagtagtgac tctcgaatct tactgcattt aattaatata cccacagcta aatatcgtta
42001 caaatttatc aatgaagaag tagatataca atttaaaaga gtaatagata actttcagcc
42061 agatcttata cattttggtc atcttaatca tctatcaatt actttaccag aaattgcttt
42121 taaagaaaat atacctacaa tttttacgct acatgacttt tggttaatgt gtccaagagg
42181 aaggtttatt caacgcaatt ctgaagattt gttacggctt tgtgatggac agaaggatca
42241 aaaatgtgca acccaatgtt acaaggggta ttttacagga gatgaagaat tcttgaattc
42301 agacttaaat tattgggaac aatgggttgc aactagaatg aagcaaacaa agaaagtagt
42361 ggactatata gattacttca ttgctccgtc aaaattctta atggataaat ttactcaaga
42421 ctttaatgtt ccgataaaca aaatttctta tcttgattat ggttttgatc taaatcgtct
42481 taagaataga aatagggtgc aagaaaaaga gtttattttc ggttatatag gtactcatac
42541 tcccgagaaa ggtgttgacc tattattgaa ggctttttct cgcttatcat ctaaagcaaa
42601 acttagaatt tggggagcag caagagaaga aactaaagct ttaaaagcga ttgccgatca
42661 gttttcacct gttgttaaag aaagaataga gtggatggga agttatgata ataagaatat
42721 agttactgac gtatttaata aagttgatgc aatagtcgtt ccttcaattt ggggtgaaaa
42781 ttcaccatta gtaatacatg aagcacagca acttagaata ccagttataa cagccgatta
42841 tggtggtatg gcagaatatg taaaagatgg actattattt aagcacagag atgcaagtag
42901 tttgtcagaa aaaatgcaag tcctatctac aaatcaggag ttatacaata aacttaccca
42961 aaaaggctat ccttatactg aaaatggaaa tataccttct ataagtgagc atactgaaaa
43021 acttaacaag atttaccgta atgctattga aaagaaaggc aaatcagtag ctgttaaacc
43081 cggtccttgg agaattactt ttgatactaa tccagattac tgcaattttt cttgtataat
43141 gtgcgaatgt ttttcaccat acagtaaagt taaagaagag aagaaagcta aaggaataaa
43201 acctaagata ctatcaatag aaactatcag aaaagtaatt aaagaagcag ctggtacacc
43261 tttaagggaa atcattcctt ctactatggg tgagccttta atgtataaaa gctttgatga
43321 aataatcaat ttgtgccatg agttcggcct gaagcttaac ctcacgacca atggttcttt
43381 tcctattaaa ggagctagaa aatgggctga actattggta ccaattttat ctgatgttaa
43441 gatttcctgg aatgggggcaa ctaaagaaac tcatgaaaga atcatgaaag gttcaaaatg
43501 ggaagtagtg acagaaaatt taaaaacttt ccttgaagtt agagataaat actttagcga
43561 tacaggtgaa aggtgtaccg ttactttaca attaacattt ttggaaagta atttacatga
43621 actctatgac atagttaaga tggccataaa aaacggcata gatagagtta aaggacatca
43681 tctttgggca cattttgagg aaattaagga tctatctatg agaagagatg agttagcaat
43741 tagtagatgg aacacggagg tgagaaggtt atacgaactt agagataata ttctattacc
43801 aaatggtaaa aagataaaat tagaaaattt cacaatcctt tctcaagaag gtattaagga
43861 tttggctcca ggtggtcaat gcccattttt aggtaaagaa gcatggataa acaatgaagg
43921 aaagtttagt ccttgctgtg ctccagatga actccgtaag acattaggaa attttggtaa
43981 tgttaatgag gttaaactcg aggaaatatg gcaaagtagc gagtacctaa acttacaaaa
44041 gaattattta aactatgaat tatgcaaaac atgcaacatg agaaaaccat tagtcaatta
44101 atgaaagata gtgagtacct taaatctatt agggtttccc cctgtgaaag gtttcaccaa
44161 ttgtcagaaa atcctttgta caaaaataga tttattagag ttgataaatt tcacgagcct
44221 ggccttgcac cagtttatga tgagacaggc gcttatcaca tcgatgcaat gggagaagca
44281 atttatcgcg acagattttt aaaaactttt ggattttact gcaatagagc agccgtagaa
44341 gatgacacag ggtattacca tattgatcca tctggatgta gagtatacaa acattcgtat
44401 caatggattg gaaattacca agaagatatt tgtgtgatta gaaagcatga taagtttttt
44461 catattgatt tgaacggcaa tagagtttat gagcaagaat acaattatgt tggggatttt
44521 aaggatggta tcgctgtagt ccataaagat ggtaaagcta cacatattaa taatcatgga
44581 aagttagtac ataataaatg gtataaaaag cttaatgttt ttcataaagg atttgctatt
44641 gcagaagata agcatggctg gtttcatata gatattaatg gcaatcctgt ttaccggcaa
44701 agatttaaaa tggtagaagc attctataat ggcatggcaa aagttgaaac ttttgaaggt
44761 gtattaggac agatcgatat taccggaaac gtaaagttta gtatttttga tttagataaa
44821 gaatctcaag tgcataagat ttctgcagaa ctttcagcct tttggaaaac ttacctcgca
44881 aatgttgcta ttgaacttga tttgttaaat atcttacctg caactatgcc agttttatct
44941 caaaagttaa atattatcgt tccaaatcta gaaaggctgt taagggcgtt gtgggaaata
45001 gggtttattg attacgataa gaacaaggac ttatggcaac tatcatcaaa aggtaagtgt
45061 tttaaggaaa taccattttt gcctaaagca gcagcaatgt gggcaagggt tgccgctgaa
45121 aagaattggt taaaaattgc cgatatatta aggcaggagt caatatcttc atttgaatct

45181 tttaaggaga aagaaacatc agaagacagg aaaatagcgt tttaccaagc attgctagga
45241 tattccaggt ttgacactaa agagtttaat tctagggtta atatagatgg tgctaaaaat
45301 atattgctat ttggcgtaca ctctttattt cttgcttatt ctgatataca caataaaggt
45361 tctataggct tatattatta caacgaacat aaagtgccta ggcaggcagt agaagattta
45421 aaaattaaac tcataactca agaagaatta tcagctacaa attatgaatt aggtgtcttt
45481 tgccgctttc tgcagcatta tgatgataat aaggtattgt cttatttaaa attggtaaaa
45541 aataaaggaa taccacgcgt tttagtgata gaaactatcc tagattacta ctccccagtg
45601 ggaggttctg tagatattaa tgtaatggtt gaaacagggg gtaaactaag gacattgagc
45661 gattggaaaa gaatactaaa acaagtaaaa gggcttaaaa tttttagtat tgtaccttta
45721 acagattatt tatcagttat tgacattagg tgttaatata tgaaagcatt gcaaaaaaat
45781 ggttttttta ttatcaaaaa tctagttcct ttagagttaa taactcagtc tttaaatgat
45841 ataataagca agatatcaaa gttatctcaa gaattaggtg tttcaacttc tgactattta
45901 aactgcactg gaagatgggg tacatcatct caggtaacta gaattgtatc tcaagtatta
45961 gataaaatta ttaaaaatta tcttgaaaag tcacttcaat gccagatact gcagaaaaaa
46021 tcaaatgtta tatgtaaaac tgctgattta atagatgcag ttcccttcca tcaggacatc
46081 tcttatagct ttaatgaccc ttatcatttt tctgtttggc tagctttgaa taatgtcaat
46141 aaaacttcag gtgcactaca agttatagaa gatagtcaca actggaaaat acagcctcta
46201 gttgattttt ggcatccata ctttttggat caatactcgg ataaccggga aaattataaa
46261 attaagtcac tacctatttc agcaggagat gcgatagttt ttgattcacg gttatggcat
46321 ggtagtgata agaatataga tgctaaagat aggtttgctt atgttacgag atgggttgta
46381 aaagataaaa gtcttccttg tgtgccaaaa cctcagcctt cagtttttgg tatattaaac
46441 tgtggtgcat taacagaatt tatactgaga gaatacttat cattatttcg tcgccaggaa
46501 agtataaaaa caaaaaatat ggaagaactt attaagagct ggttagcctt tattacagac
46561 acaactacaa atatttctga ggtcaacact gctgaagcta agcgagattt atataagcta
46621 cttattctga atcaagcgtc agcactacat gatgctggag acatttcagg aaaaatatac
46681 aaaaatttat ggttttcact gcttgtattt ctcaataaaa aagtcaatgt ggtggagcta
46741 gcattataaa tgaacaaaag tagagttggt agtttcaagt atatattggt gttcattttg
46801 aatatcttat ccaagtttaa gcttaatgtg cttgtaatgt tcctggttgc cctagtagtg
46861 gctgttgatt tatcttttag aaagtattta gtaaaaaata tcttagatac agctgtaaaa
46921 tatcaagaag gtaatgtaat tgaaaatctt ttattacctg taagcgctta tgtttgtatg
46981 gcattactta ttactactgc ttttagattc tatggctatt ttgttgacat tcgaatgttt
47041 acctcaatgc gtcaaaaaat agctgatatg tctttttaca ggctgcttca gcaagaccat
47101 tcctattatc aaaacaacct atccggaagt ttagtatata aggtcagtaa cttaatggat
47161 agtgtgatag agctaataag actgcttata gactgtttct ttagttatag tatagcatta
47221 atcttggcta tttatacttt atcactagta aatataaagt ttgcaatagc tacattcatt
47281 tgggtgaaca tttttatttt gatttcggtt ttcagttttc gggtgcttac cggattagcc
47341 gatgatcatt ctaaacaaag ttcacaagta atagcgggta tagccgacag tattttaaat
47401 gttatatcag taaggttatt ttctcggcaa gcgcatgaga ggcataaatt ttttcaagta
47461 tgcaagaaaa aaactatttc agaaagaaaa ttacaatggg catacttttg gctttggttt
47521 atatatggct attcatttga tatactccaa gcagtaaact tgtatttttt aattcatgat
47581 tatcaattga acaagattgc aataggagat attgctcttg tacttggaat taatatatcg
47641 attatagagt ttcttaacca tctaactagg aatcttaccc aattttctac tcatttcggc
47701 aaagtttcag atgcattgcc aatcttgact accgtaccag aaattcaaga taaggaaaat
47761 gctaaaaaat taaatttatt aagtggaaga ataacgttta ataatgtttc tttttcttat
47821 gagggtcaga agtcattatt tcaagatttt tcagttacca ttaatccttg cgaaaaggta
47881 ggtctagttg gttattctgg aggtggtaaa tctactttta taaacttaat cataaggcta
47941 tttgatgtta aaaaaggtaa tatacagatt gataatcaag tagtatcgga agttacacag
48001 agttctttga ggcaacaaat atctgtaata cctcaagaac cattgttgtt tcatgatact
48061 attttagcaa atattatata tggtaaactt caatctacta tcgaagaaat aataagagct
48121 gcaaagctgg ctggtattta cgatttcatt atgactctac ccgatcaata tgaaactgta
48181 gtcggagaaa aaggcatcaa gctatctgga ggagaaagac aaagaataat aatagcaagg
48241 gcatttttaa aaaacgctcc aatattattt cttgatgagc caactagtca gttagattct
48301 ataacagaga aaacaattca aatgagttta tttaagttga tggaaaataa aaccaccatt
48361 actatagcac accgtatttc tacactttta catatggacc gaattctagt atttgacaaa
48421 ggaaaaaattg tccaagatgg taaacattct gaactagttt gcaagaaagg tctttataaa
48481 gaactttggg atgctcaaat tggctgtcta aatatcaaaa aagaaaatgg aaaaaactat
48541 cgacctgaga agtgacacta caactttaca aagctccaac gttatgcacg caataagtag
48601 tgcaatcgtt ggagattttg cttacggtga agacaaaagc tgtaacaact tatcggaata
48661 ttgtaaacaa ttattcaaag tagaagaagc attgttcgta accagcggta tgcttgcaaa
48721 taggttagct attgccagtc aaacaagtcc tggtgatgaa ctaattactc attataatta

48781 tcatgttaat tttttcgata gtgcaggcaa tgcaaaagta aatagtatag tgtttaactg
48841 tattaggaat aataccggaa tcttagatat tgatgaagta gaatatgcta ttaactctaa
48901 accgagatat aaaatttttg ctcaggtgag tctagtttct atagaaaata gtataaacgg
48961 gtttaacggt aaaatttatc cttttaagaa acaagtagag ttatataatt tcttaaaagc
49021 tcacaatata agtcttcatt tagatggtgc gagaatattt aatgctcatg ctgaaacaaa
49081 tgttgcttta gctgactatg ctaaatatgt agatacaatg agcttttctt ttactaaagg
49141 acttggagct ccttttggtt caatgcttat gggtaaaaga gagataattg aaaaggctaa
49201 gaagttacag gtttggttgg ggagtggtta tcatcaaatt gggtactgcg ctaatgcagc
49261 aaaatatgta ctacagaata atatctctag gcttaaagaa gataataagc ttgctaaatt
49321 gtttgcagac aagattaaag aaattccaca tgtaaagctg atactacctt atccagaaac
49381 taacatagta agctttagta taaaagaact aaatgtgact aatgagtttt ttttgagtaa
49441 atgccaagct tatggtttat tgctctttcc ttggctcgaa tctcatataa gagcagttgt
49501 tcatcttggc atagaggaag cagacataat aaatgctgct gaaattataa aggaggttgt
49561 attaaattta gtatgagggt aaaaatagat ttctttagcg attcaaacac taatccttca
49621 tctgaaatga gggaagtaat ggctaaagca aaagttggaa atgaagctgc atgtgaagat
49681 ccaactgtaa acgaattaat agaaaccaca tgtaaaatat taggtaagcc agcaggaata
49741 tttttgattt cgggaacgat gtgtaatgta attgcttata aagtgcacat tcaaagacct
49801 ggtgattatt tgttactcga tgaaacttcg catccattaa tagtgcaatc tggattaatt
49861 gcagcccagg cacatgccac tcctttacct attaaaggaa caagaggaat atttactgga
49921 gagcaaatcg ttgagtttat tactcgtcct agcttaagga atgttccaaa agttgggctt
49981 gtttctgtcg aacaaacaac gaattttggt ggtggagctg tgtggccttt agagtatatt
50041 caagagatct caagtctctg caaaaagaac gacgtatcct ctcatttaga tggagcaagg
50101 ctgttcaatg cttgtgctta tgctaaaata tctccaaaag aatatgcaaa ttattttgat
50161 tcggtgttta ttgattttag taagggatta ggcgctccaa tgggtgcagt attggctgga
50221 agtgaagagt ttattgaaaa agcttggtac tataagtttc aaattggcgg aggtatgcac
50281 caagctggca ttatttcagc tgcatgttta tatgctctca acaacaacat gaattcttta
50341 atagaagatc ataaaaaaat gcagcacctt ataaaaggtc ttagctctat agatcaagtt
50401 ataatcaata ttgacctata caaaactaat atagcgtatt tctcattacg tactgattgt
50461 atatctgctc aagaattaat aaatatacta atgacaaatt atggaattag gatggctaac
50521 ataagaggta aaatcagagt tataacacat agagatataa gctgtgaaga tattaatact
50581 actatatcta ctataagaaa gatactgaat aggtaatttc gattgttatc aattttctac
50641 attaatggtt tgcaaacttc ttgtaaaaca atattatacc aacatagtat tatgctagta
50701 tttgcatggg actgtttggg gaataggtgt gaactttgtt ttctttcaat atttactatt
50761 ctttttttgga tatgtgctac tttacttctc aaggcagagt ataccaattg ctctaccact
50821 gttaatagat actgaccata taaaattaag ctattttttt gcctgctttt cttttttttta
50881 tggagtatca aaattcgtta atggaatagt catggactcc aaaaataatc cattatttat
50941 gtttgggcta tgcaatattg caactggatt attaaccatt ggtatagctt tatcatataa
51001 taatctaact ttattactgt taacattaat attattagct tggacacaag ggttaggttg
51061 gccagctatt accaagttta tggttacaaa ctctagtatc tcttcaattg catcatcatg
51121 gtgagttatg agcgcatctc agcaacttgg ttcttgtatt actttaattg tattaccaag
51181 tataatcaaa atatatgatg caaaaagtgc atttttgtac tctggtctct tatgcttact
51241 ttttggaatt tatataatat taaaagcata ttacgaagaa agtgccaact ttactaccta
51301 ttataaggtg cagaaatctc agataggtat caaagtgaaa agttccatat ggtttctatg
51361 ttgtgctact ttttgtgctt atattataaa aatgggaatc ttcttttggt ttcctataat
51421 tttaaagaat aaattacaaa tttctcttgt acaatcctca ctaataacag gcgtatatga
51481 tttaggtgga atccttggaa ctttaatcac aggaagaatt agtgatatgt acttctttca
51541 aaatagaagc ttgctggcat cactttatat gttgggtata gcactcactt ttattgcaat
51601 gaactttggt caaaacataa ttcttatgaa ccttggtgca atcttatcag gattctttat
51661 atttggagtg caagtactga caggtgttat ggctgtagaa attgctccac aaaataatgt
51721 atgtgcaatt gtaagcttaa caggattatt tgggtatatt gcaagttcaa tattttcatg
51781 cgtgctctta ggagtacttg ttaagcactg tggtaacagt attatgtttt tatttttttc
51841 catttgctct gttgttgcac tcgtttgctt ttccatatta tcaagcaaag atcttaaaaa
51901 actaagtaaa gcggtgtaga aaacatttct caacagttaa actttgtaga tagatttttt
51961 acccaaggaa taaacatggt aaccatatct tagtaatagc tcgctatcta ataaattacg
52021 gaaatcaaaa acattaggtg cagccataat actctttaaa cttgcaaaat cttgattttt
52081 aaattctttc cactccgtta acaataacaa ggcttccgcg tcccttgcag cccctatagc
52141 attgtccgcg tattctatat cctttagaac ttgcctagca ttatccatcc ccattgggtc
52201 atatgcagta attttagcct tgttattcac taacaattgt gcaatatcta tagctgggct
52261 atttcttaca tcatcagtac cagacttaaa agttagcccc catattgcta tttttttatt
52321 ttggactcca tttaatacat attctacctt tttagcaatg tttgttatat gattatagtt

```
     52381 agattcctta actgaattta aaatttgtag cttaacattg tggtcttcag caagccttat
     52441 taaagctgac aaatctttag gaaaacatga tcctccaaat cctggaccag ctttgagaaa
     52501 ttccttacct attctatggt ccattcccat actgttagct aaaagttcaa tgtctgctcc
     52561 taatagctca cataaacctg ccatttcatt gataaaagca acctttgttg ctagaaaagc
     52621 attagaagca tatttaatca tttcagctgt aactgtatca gtgactatta acggaatatt
     52681 cctatctgta aatgatctat atatagcttc tagttttact ctagctcgct tagtttttac
     52741 tcctattatt attctatctg gatataaaaa atctgatact gcagagcctt gcttgagaaa
     52801 ctccggatta actcccaaat caaagttgta gcccttatta gataaataat tatatatatt
     52861 ttttgtagca ccaggaggaa tagttgattt tataactatt aagcaatctt gattaattct
     52921 ctcagaaact tcacttactg cattatatac attctttaaa tttgcatttc ctagggagtc
     52981 tgatggagta tctacagtta taaatactac ttctgtattg ggatttattt gaaaatacga
     53041 atcgaaaaat cttatccttt gtgactttat attattctct aaataatctg ctaatccagg
     53101 ttcatataag ggaattttcc ctgatttcaa aagttcaatc ttctcagtat ttatgtcaat
     53161 gcaatcaact ttatgaccct gctctgataa cattgcacaa gatactaaac ctacataccc
     53221 tactcctata atagttatgt gcattacctt aaaaatatag cttagcatct aaaataaata
     53281 ccattaacaa ttataaactt ttgttgttac attatttttc ttaattgcct taccttccga
     53341 ataagctata aataaggttg atggtattaa tatcaaagta ccatataaag cacttttatc
     53401 aggtaattca ttaaatataa catatgctac tattgcagaa attattagct ctatgtatct
     53461 ataaggtgca agtgcagtag catcagtaag agcaaaagct tttaacatga agaacagaat
     53521 taggtttgaa cttattccta aaatcagcag taatattaat tctaacaagg aaggcaaatg
     53581 ccaataaaac agcaaaggca cagtggaaaa aatagttgtt gttagagctg aataaaataa
     53641 catacttatt acggactctt ttattgcgag tttcttatta agtatgtcta atatagcaaa
     53701 gattaatgta gatacaataa aaataagaat tttaggatta aagtcttcac tataagcttt
     53761 ggtagtaata gcaataccaa caaagccaat aacagttact gaccatcttt gccaaattat
     53821 attttcattt agtaaaggga ttgcaagaag tataacaaat aatggtatag aaaaacttat
     53881 aattgttgca gttactatgg gaaaaatagc taacccatag gtccacaagg ttattccaac
     53941 aaataacaat actcctctag ttatctgaat agatatttga cttgttttaa aagcttctct
     54001 tccatagtaa agcataaaag gtacaagggt aatagtgcta aataaaaagc ggaaaaagat
     54061 tacttcaaaa ctttggagat gtaaacttag atattttgat atagtatcat tagctacact
     54121 actaaataaa ctaagtataa accagata
//
LOCUS       JAQZAU010000004             3526 bp    dna     linear   ENV 17-MAR-2023
DEFINITION  MAG: Wolbachia pipientis isolate wEsol contig_11120, whole genome
            shotgun sequence.
ACCESSION   JAQZAU010000004  JAQZAU010000000
VERSION     JAQZAU010000004.1
DBLINK      BioProject: PRJNA929258
            BioSample: SAMN33142973
KEYWORDS    WGS; ENV; Metagenome Assembled Genome; MAG.
SOURCE      Wolbachia pipientis (insect metagenome)
  ORGANISM  Wolbachia pipientis
            Bacteria; Pseudomonadota; Alphaproteobacteria; Rickettsiales;
            Anaplasmataceae; Wolbachieae; Wolbachia.
REFERENCE   1  (bases 1 to 3526)
  AUTHORS   Fiutek,N., Couger,B.M., Roy,S., de la Torre,J.R. and Connor,E.F.
  TITLE     Genomic assessment of the contribution of the Wolbachia
            endosymbiont of Eurosta solidaginis to gall-induction
  JOURNAL   International Journal Molecular Sciences
REFERENCE   2  (bases 1 to 3526)
  AUTHORS   Fiutek,N., Couger,B.M., Roy,S., de la Torre,J.R. and Connor,E.F.
  TITLE     Direct Submission
  JOURNAL   Submitted (10-FEB-2023) Biology, San Francisco State University,
            1600 Holloway Ave, San Francisco, CA 94132, USA
COMMENT     ##Genome-Assembly-Data-START##
            Assembly Date          :: JUN-2019
            Assembly Method        :: FLYE v. 06-2019
            Genome Representation  :: Full
            Expected Final Version :: Yes
            Genome Coverage        :: 100.0x
            Sequencing Technology  :: Illumina HiSeq; PacBio Sequel
```

```
            ##Genome-Assembly-Data-END##
FEATURES             Location/Qualifiers
     source          1..3526
                     /organism="Wolbachia pipientis"
                     /mol_type="genomic DNA"
                     /submitter_seqid="contig_11120"
                     /isolate="wEsol"
                     /isolation_source="galls on Solidago altissima"
                     /host="Eurosta solidaginis MN-2018-EFC"
                     /db_xref="taxon:955"
                     /environmental_sample
                     /country="USA: Cowling Arboretum, Carleton College,
                     Northfield, MN"
                     /lat_lon="44.48 N 93.13 W"
                     /altitude="280 m"
                     /collection_date="2018-11"
                     /metagenome_source="insect metagenome"
                     /note="metagenomic"
     gene            complement(34..216)
                     /locus_tag="wEsol_00068"
     CDS             complement(34..216)
                     /locus_tag="wEsol_00068"
                     /codon_start=1
                     /transl_table=11
                     /product="hypothetical protein"
                     /translation="MLYSLWLLTLIFCGIFFLSRLNGYFIMNFVSSHQIFSVAMQQSH
                     PSVKHWDDTLLVETLL"
     gene            173..502
                     /locus_tag="wEsol_00069"
     CDS             173..502
                     /locus_tag="wEsol_00069"
                     /codon_start=1
                     /transl_table=11
                     /product="transposase"
                     /pfam="DDE_Tnp_1_5"
                     /fullproduct="qcoverage 100 , hcoverage 100 , similarity
                     100 , identity 100 , evalue 1.19e-72 , alnlength 109 ,
                     Bacteria , Proteobacteria , WP_007548762.1 MULTISPECIES:
                     transposase [Wolbachia]"
                     /translation="MPQKMRVSNHSEYNKFLEKRGNIFHYINEAIEKWYENGPKIPGG
                     NNIYSDKVVILVHIITCLFRIGLRQTVGFIAGYLEQIGKNLQVISYSQSSRRFKKLNI
                     KINDCRK"
                     /besthit="qcoverage 100 , hcoverage 100 , similarity 100
                     , identity 100 , evalue 1.19e-72 , alnlength 109 ,
                     Bacteria , Proteobacteria , WP_007548762.1 MULTISPECIES:
                     transposase [Wolbachia]"
     gene            486..686
                     /locus_tag="wEsol_00070"
     CDS             486..686
                     /locus_tag="wEsol_00070"
                     /codon_start=1
                     /transl_table=11
                     /fullproduct="qcoverage 100 , hcoverage 100 , similarity
                     100 , identity 100 , evalue 3.80e-36 , alnlength 66 ,
                     Bacteria , Proteobacteria , WP_010082383.1 MULTISPECIES:
                     hypothetical protein [Wolbachia]"
                     /translation="MIAENNMEDIEIAIDSTGISIYNNTPGHSKENSEDRKYRSYEQT
                     RKLHVMLNTNSKKAIAVKYSNG"
                     /product="hypothetical protein"
                     /besthit="qcoverage 100 , hcoverage 100 , similarity 100
```

```
                     , identity 100 , evalue 3.80e-36 , alnlength 66 ,
                     Bacteria , Proteobacteria , WP_010082383.1 MULTISPECIES:
                     hypothetical protein [Wolbachia]"
     gene            706..1113
                     /locus_tag="wEsol_00071"
     CDS             706..1113
                     /locus_tag="wEsol_00071"
                     /codon_start=1
                     /transl_table=11
                     /pfam="DDE_Tnp_1 DDE_Tnp_1_2"
                     /fullproduct="qcoverage 100 , hcoverage 100 , similarity
                     100 , identity 100 , evalue 8.54e-92 , alnlength 135 ,
                     Bacteria , Proteobacteria , WP_007548764.1 MULTISPECIES:
                     transposase [Wolbachia]"
                     /translation="MLKEVDFQYVIKVLYADRAYDREKLYKLCNQYGIKTKIPPKNNA
                     AEHPKLDYMAERNSAVKLIKSYDEDGIKKWKKEMSYGKRSYIESFFSRLKQTFGFSFR
                     NKSETNREKELLVKCYLLNKFTDIGMAKFEIAS"
                     /product="transposase"
                     /besthit="qcoverage 100 , hcoverage 100 , similarity 100
                     , identity 100 , evalue 8.54e-92 , alnlength 135 ,
                     Bacteria , Proteobacteria , WP_007548764.1 MULTISPECIES:
                     transposase [Wolbachia]"
     gene            complement(1116..1400)
                     /locus_tag="wEsol_00072"
     CDS             complement(1116..1400)
                     /locus_tag="wEsol_00072"
                     /codon_start=1
                     /transl_table=11
                     /fullproduct="qcoverage 100 , hcoverage 100 , similarity
                     100 , identity 100 , evalue 1.29e-60 , alnlength 94 ,
                     Bacteria , Proteobacteria , WP_041573534.1 hypothetical
                     protein [Wolbachia endosymbiont of Drosophila simulans]"
                     /translation="MGNSKINNSANFSMICGSGVYVRSIARDLGIALNCFGHITKLRR
                     TMVGDFRENESVTIEQLTKKNTTTYSPMSSQCLTLGWLCCIALRIVGNAQ"
                     /product="hypothetical protein"
                     /besthit="qcoverage 100 , hcoverage 100 , similarity 100
                     , identity 100 , evalue 1.29e-60 , alnlength 94 ,
                     Bacteria , Proteobacteria , WP_041573534.1 hypothetical
                     protein [Wolbachia endosymbiont of Drosophila simulans]"
     gene            1467..1634
                     /locus_tag="wEsol_00073"
     CDS             1467..1634
                     /locus_tag="wEsol_00073"
                     /codon_start=1
                     /transl_table=11
                     /fullproduct="qcoverage 100 , hcoverage 100 , similarity
                     100 , identity 100 , evalue 4.62e-30 , alnlength 55 ,
                     Bacteria , Proteobacteria , CDR78442.1 hypothetical
                     protein WPAU_0043 [Wolbachia endosymbiont of Drosophila
                     simulans wAu]"
                     /translation="MANESNEWAKNEFGDAVLGDKRLTDRLVKIADSLISLPESSINE
                     ACGSWSEAKAA"
                     /product="hypothetical protein"
                     /besthit="qcoverage 100 , hcoverage 100 , similarity 100
                     , identity 100 , evalue 4.62e-30 , alnlength 55 ,
                     Bacteria , Proteobacteria , CDR78442.1 hypothetical
                     protein WPAU_0043 [Wolbachia endosymbiont of Drosophila
                     simulans wAu]"
     gene            1943..2266
                     /locus_tag="wEsol_00074"
```

```
CDS             1943..2266
                /locus_tag="wEsol_00074"
                /codon_start=1
                /transl_table=11
                /fullproduct="qcoverage 100 , hcoverage 100 , similarity
                100 , identity 100 , evalue 5.96e-72 , alnlength 107 ,
                Bacteria , Proteobacteria , CDR78441.1 Transposase for
                transposon Tn5 [Wolbachia endosymbiont of Drosophila
                simulans wAu]"
                /translation="MKWLESLKKTNNIIDLAQTEAITVCDREADMHDFFELAHNLNSA
                ILVRARHNRNVNKKFMHTRNKQKLWSFIQGLPCAGKVEVEIPARDNKQKRTAFLEIRF
                GKFIT"
                /product="Transposase for transposon Tn5"
                /besthit="qcoverage 100 , hcoverage 100 , similarity 100
                , identity 100 , evalue 5.96e-72 , alnlength 107 ,
                Bacteria , Proteobacteria , CDR78441.1 Transposase for
                transposon Tn5 [Wolbachia endosymbiont of Drosophila
                simulans wAu]"
gene            2441..2716
                /locus_tag="wEsol_00075"
CDS             2441..2716
                /locus_tag="wEsol_00075"
                /codon_start=1
                /transl_table=11
                /fullproduct="qcoverage 100 , hcoverage 100 , similarity
                97.8 , identity 97.8 , evalue 6.78e-57 , alnlength 91 ,
                Bacteria , Proteobacteria , CDR78440.1 hypothetical
                protein WPAU_0041 [Wolbachia endosymbiont of Drosophila
                simulans wAu]"
                /translation="MRWKIEILHKILKSGLKVEECRLGTAERLMRYLTVMSIIAWRIF
                FITSIARTNPTLPCTALLAEEEWKVLYVKIHRKPCSNIAPTIKEAVS"
                /product="hypothetical protein"
                /besthit="qcoverage 100 , hcoverage 100 , similarity 97.8
                , identity 97.8 , evalue 6.78e-57 , alnlength 91 ,
                Bacteria , Proteobacteria , CDR78440.1 hypothetical
                protein WPAU_0041 [Wolbachia endosymbiont of Drosophila
                simulans wAu]"
gene            complement(2764..3054)
                /locus_tag="wEsol_00076"
CDS             complement(2764..3054)
                /locus_tag="wEsol_00076"
                /codon_start=1
                /transl_table=11
                /fullproduct="qcoverage 100 , hcoverage 100 , similarity
                97.9 , identity 95.8 , evalue 3.40e-57 , alnlength 96 ,
                Bacteria , Proteobacteria , RLT63249.1 putative
                transposase [Wolbachia endosymbiont of Drosophila
                ananassae]"
                /translation="MEHGRCWRCRKRKSSKLPEGVTPDTFGPRVKSIIAALSGFYKNS
                KREVASIINDIFNLNISVGSISNLTITHKYEVREQVAILLLDQRNVSTLSKE"
                /product="putative transposase"
                /besthit="qcoverage 100 , hcoverage 100 , similarity 97.9
                , identity 95.8 , evalue 3.40e-57 , alnlength 96 ,
                Bacteria , Proteobacteria , RLT63249.1 putative
                transposase [Wolbachia endosymbiont of Drosophila
                ananassae]"
gene            complement(3011..3439)
                /locus_tag="wEsol_00077"
CDS             complement(3011..3439)
                /locus_tag="wEsol_00077"
```

```
                     /codon_start=1
                     /transl_table=11
                     /pfam="Pfam-B_3816 DUF972 Pfam-B_4504 Pfam-B_3314 DivIC
                     HALZ Pfam-B_1653 Pfam-B_3883 Pfam-B_17409"
                     /fullproduct="qcoverage 100 , hcoverage 100 , similarity
                     99.3 , identity 98.6 , evalue 6.00e-75 , alnlength 142 ,
                     Bacteria , Proteobacteria , QEF50519.1 putative
                     transposase [Wolbachia endosymbiont of Drosophila
                     ananassae]"
                     /translation="MVNLLELCKNLQQKIEKLKAEIENLKAENKALKIENAELKERLG
                     LNSKNSSLPSSKELYKTKKDKPKSERNVGDQVGYKGNFHATMEADKVVKIELPNICEC
                     GGELVICEKPYVHQKVDLPEIRPYVVIGTWSLLEMQKKKK"
                     /product="putative transposase"
                     /besthit="qcoverage 100 , hcoverage 100 , similarity 99.3
                     , identity 98.6 , evalue 6.00e-75 , alnlength 142 ,
                     Bacteria , Proteobacteria , QEF50519.1 putative
                     transposase [Wolbachia endosymbiont of Drosophila
                     ananassae]"
ORIGIN
        1 tgtttatggc taaagattac acgaatgttg caattacaaa agcgtttcta ctaggagggt
       61 gtcatcccag tgcttgacac tgggatggct ttgttgcata gcaactgaaa aaatctggtg
      121 gctactgaca aaattcatta taaaatagcc atttaacctt gaaagaaaaa atatgccaca
      181 aaaaatgagg gtcagtaacc atagcgaata taacaaattt ctagaaaaaa gaggaaatat
      241 ttttcattat atcaacgaag ccatagaaaa gtggtacgag aatggtccaa aaataccagg
      301 tggcaacaat atttatagcg ataaagttgt aattttggtg cacataataa cttgtttatt
      361 tagaataggc ctgaggcaaa cagttgggtt tatagcagga taccttgagc aaattgggaa
      421 aaatttgcaa gttatcagct attcacaatc atcaagaagg tttaagaaac tcaatattaa
      481 gatcaatgat tgcagaaaat aatatggaag acatcgaaat tgctatagac agtacaggaa
      541 ttagtatata caacaacact cctggtcata gcaaagaaaa tagcgaagat agaaagtatc
      601 gcagctatga acagacaaga aaattgcatg taatgttgaa tacaaacagc aaaaaagcca
      661 tagctgtaaa atacagtaac ggctgatcac tatggagctt gtgatttgct aaaagaagtc
      721 gattttcagt atgtcataaa agtactatat gcagataggg catatgacag agaaaagctc
      781 tataagctgt gtaatcaata tggtataaaa acgaaaattc ctccgaaaaa caacgcagca
      841 gagcatccaa agttagacta tatggctgag agaaattctg cagtcaagct cataaagtca
      901 tatgatgaag atggtataaa gaagtggaaa aaagagatga gttatggaaa aagatcttat
      961 atagaaagtt ttttctcgcg actgaaacaa acattcgggt ttagttttag gaataaatct
     1021 gagacaaata gagagaagga actacttgtt aaatgctatt tgctcaataa gttcactgat
     1081 attggtatgg ctaaatttga gatagcttca taaatttatt gtgcgttacc tactatccga
     1141 agagcgatgc aacaaagcca tcccagtgtc aagcactggg atgacatcgg agagtaagtt
     1201 gttgtgtttt ttttagtaag ttgctcaatt gtcactgatt catttctct aaagtcacct
     1261 accatagttc ttcttaattt tgtaatgtgt ccaaaacaat ttaatgcaat tccaagatca
     1321 cgagcaattg atcttacata tacaccacta ccgcatatca tagaaaaatt tgcactatta
     1381 tttatcttac tattacccac aaatataaat gtacttataa ataaagtatt acaatattaa
     1441 agtttgcaaa tattgagggt ataaaaatgg caaacgagag taatgaatgg gctaaaaatg
     1501 aatttggaga tgctgtactt ggagataaaa gattaactga ccgattagta aagattgctg
     1561 atagtttgat aagtttacct gaaagctcaa ttaatgaggc atgcggaagt tggtcagaag
     1621 caaaagcagc ataacgtttt tttcaaaatg agaacgtaaa agaagtcgat attctagctt
     1681 cacacattga taaaacagtt gaaagaacaa aagctcataa aagaattctt gtgattcaag
     1741 atacaactta tatttcatac tcaagccata agcggattgg gaactattgc tggaaaagga
     1801 gcagtaatgc atacagccct tgctgttagt acggaagctg ggaatactgg atcaaaagat
     1861 ttactcgagg caactcattt ctgaagaaga aaaaagacta aagagccatc gtaacaacgt
     1921 tcatattgag gataaagaaa gtatgaaatg gctagaaagt ttaaagaaaa caaataatat
     1981 tatagatctg gcccaaaccg aagctataac tgtatgtgac agagaagcag atatgcatga
     2041 ttttttttgaa cttgcacata atcttaactc agcaatttta gtaagagctc gccacaacag
     2101 aaatgtaaat aaaaaattca tgcataccag gaacaagcaa aaattatggt catttatcca
     2161 aggcctccct tgcgctggaa aagtagaagt tgaaatccct gctagagata acaagcaaaa
     2221 aaggacagcg tttttagaaa ttagattcgg aaaatttatc acatgaaggc catataaaat
     2281 gtaaagaagg tcacataaaa tataaaccga cagagtctac aacttcacgc aattcatgtt
     2341 gttgaaaaaa actctcctcc cgaagcaagt cctctagagt ggatgctttt ttccggtcag
     2401 tacttttgaa gaagctgttg aaaaagttag ttggtattgt ttgagatgga aaatagagat
```

```
     2461 attgcataag attttaaaat ctggtctcaa agttgaggaa tgtagacttg gaacagcaga
     2521 aagattaatg aggtatttaa cagtcatgag cattattgct tggagaattt tctttattac
     2581 atcaattgca agaactaacc caacattacc atgtactgcc ttattagctg aggaagaatg
     2641 gaaagtttta tatgttaaaa tacacaggaa accatgttca aatatagccc ctactataaa
     2701 agaagccgtt tcgtgaactt ggaggtcatt tagcaagaaa aagcgaccca aaaccaggac
     2761 caattactct ttggaaaggg tggagacgtt tctttgatct agaagaagga tggcgacttg
     2821 ctcacgaacc tcatatttgt gggtaatagt aagatttgat atgctgccaa cacttatgtt
     2881 caaattaaaa atgtcattta tgatactagc cacttcacgc tttgagtttt tgtaaaatcc
     2941 gctgagtgct gcaattatag acttaaccct tggaccaaat gtatctggcg taacaccttc
     3001 tggtaacttg ctactttttc tttttctgca tctccaacaa cgaccatgtt ccaattacta
     3061 cataaggcct aatttccgga agatcaactt tttggtgaac ataaggtttt tcgcatatta
     3121 caagctctcc tccgcattcg caaatattag gcaactctat ttttactact ttatccgctt
     3181 ccattgtggc atgaaaattt cctttatacc caacctgatc gccaacattc ctttcgcttt
     3241 ttggcttgtc ctttttttgtt ttgtacaatt ctttagagct cggtagagat gaatttttcg
     3301 agtttaaacc aagcctttcc tttaattcag cgttctctat ctttaaagct ttattttctg
     3361 cttttaagtt ttctatctct gctttcagtt tttctatttt ttgctgtaaa tttttgcaaa
     3421 gttctaaaag gtttaccaca ttacctcact tttgctctat gattatcttt ttagatcact
     3481 ttgtctacct tattctacca ctccgctgaa cggatacaat ttacta
//
LOCUS       JAQZAU010000005            1489 bp    dna     linear   ENV 17-MAR-2023
DEFINITION  MAG: Wolbachia pipientis isolate wEsol contig_11580, whole genome
            shotgun sequence.
ACCESSION   JAQZAU010000005  JAQZAU010000000
VERSION     JAQZAU010000005.1
DBLINK      BioProject: PRJNA929258
            BioSample: SAMN33142973
KEYWORDS    WGS; ENV; Metagenome Assembled Genome; MAG.
SOURCE      Wolbachia pipientis (insect metagenome)
  ORGANISM  Wolbachia pipientis
            Bacteria; Pseudomonadota; Alphaproteobacteria; Rickettsiales;
            Anaplasmataceae; Wolbachieae; Wolbachia.
REFERENCE   1  (bases 1 to 1489)
  AUTHORS   Fiutek,N., Couger,B.M., Roy,S., de la Torre,J.R. and Connor,E.F.
  TITLE     Genomic assessment of the contribution of the Wolbachia
            endosymbiont of Eurosta solidaginis to gall-induction
  JOURNAL   International Journal Molecular Sciences
REFERENCE   2  (bases 1 to 1489)
  AUTHORS   Fiutek,N., Couger,B.M., Roy,S., de la Torre,J.R. and Connor,E.F.
  TITLE     Direct Submission
  JOURNAL   Submitted (10-FEB-2023) Biology, San Francisco State University,
            1600 Holloway Ave, San Francisco, CA 94132, USA
COMMENT     ##Genome-Assembly-Data-START##
            Assembly Date          :: JUN-2019
            Assembly Method        :: FLYE v. 06-2019
            Genome Representation  :: Full
            Expected Final Version :: Yes
            Genome Coverage        :: 100.0x
            Sequencing Technology  :: Illumina HiSeq; PacBio Sequel
            ##Genome-Assembly-Data-END##
FEATURES             Location/Qualifiers
     source          1..1489
                     /organism="Wolbachia pipientis"
                     /mol_type="genomic DNA"
                     /submitter_seqid="contig_11580"
                     /isolate="wEsol"
                     /isolation_source="galls on Solidago altissima"
                     /host="Eurosta solidaginis MN-2018-EFC"
                     /db_xref="taxon:955"
                     /environmental_sample
                     /country="USA: Cowling Arboretum, Carleton College,
```

```
                     Northfield, MN"
                     /lat_lon="44.48 N 93.13 W"
                     /altitude="280 m"
                     /collection_date="2018-11"
                     /metagenome_source="insect metagenome"
                     /note="metagenomic"
     gene            complement(23..364)
                     /locus_tag="wEsol_00078"
     CDS             complement(23..364)
                     /locus_tag="wEsol_00078"
                     /codon_start=1
                     /transl_table=11
                     /fullproduct="qcoverage 100 , hcoverage 100 , similarity
                     100 , identity 100 , evalue 1.53e-79 , alnlength 113 ,
                     Bacteria , Proteobacteria , AAS13810.1 transposase, IS5
                     family, OrfB [Wolbachia endosymbiont of Drosophila
                     melanogaster]"
                     /cog="COG3293 COG3293 124 Transposase and inactivated
                     derivatives"
                     /pfam="DDE_Tnp_1_2 DDE_Tnp_1"
                     /product="transposase, IS5 family, OrfB"
                     /translation="MRTKYTKVKKMWADMGYQGRNLKNHIKEEYDIDIEIVKRPPCRF
                     WVHKDTPPELLPTREQGFKVQPRRWVVERTFAWVNRNRRLSKEYDLLTTSTENFIYLA
                     MSRVMLKREYA"
                     /besthit="qcoverage 100 , hcoverage 100 , similarity 100
                     , identity 100 , evalue 1.53e-79 , alnlength 113 ,
                     Bacteria , Proteobacteria , AAS13810.1 transposase, IS5
                     family, OrfB [Wolbachia endosymbiont of Drosophila
                     melanogaster]"
     gene            complement(475..855)
                     /locus_tag="wEsol_00079"
     CDS             complement(475..855)
                     /locus_tag="wEsol_00079"
                     /codon_start=1
                     /transl_table=11
                     /fullproduct="qcoverage 100 , hcoverage 100 , similarity
                     99.2 , identity 99.2 , evalue 3.88e-86 , alnlength 126 ,
                     Bacteria , Proteobacteria , AOV87106.1 transposase
                     [Wolbachia endosymbiont of Drosophila incompta]"
                     /cog="COG3293 COG3293 124 Transposase and inactivated
                     derivatives"
                     /pfam="DUF4096"
                     /product="transposase"
                     /translation="MRKKYPTDLSEREWARIEKHFRVSYKKGGRPPKYSKREILEAIF
                     CVLRTGCQWRYLPNDFPLWKTVYEQFRQWKKQGIFEKMNYEITKYSRRKIGRNEQPSA
                     CIVDSQSVKTTEKGGSKAMMEVKK"
                     /besthit="qcoverage 100 , hcoverage 100 , similarity 99.2
                     , identity 99.2 , evalue 3.88e-86 , alnlength 126 ,
                     Bacteria , Proteobacteria , AOV87106.1 transposase
                     [Wolbachia endosymbiont of Drosophila incompta]"
     gene            962..>1489
                     /locus_tag="wEsol_00080"
     CDS             962..>1489
                     /locus_tag="wEsol_00080"
                     /codon_start=1
                     /transl_table=11
                     /product="hypothetical protein"
                     /pfam="Ank_2 PAZ_siRNAbind PAZ_siRNAbind PAZ_siRNAbind"
                     /translation="MQQAELSNLLKSVLSAKIISDDSLIYTPLTYAILCKNSKAIEEI
                     LKVSKGNDILKDILNATTTIKYLDGRRATVTPLACAILCGDSIAIEEILKVSKEKDIL
```

```
                     NTTATIKYPDSQKVTYTPLTYAMFWKDSKAIKEILVAAKGNSTLLKDILNTTTTMKYL
                     DREVTYTPLAYVILRR"
                     /besthit="qcoverage 93.1818181818182 , hcoverage 25 ,
                     similarity 69.5 , identity 68.3 , evalue 7.05e-07 ,
                     alnlength 164 , Bacteria , Proteobacteria ,
                     WP_182366402.1 hypothetical protein [Wolbachia
                     pipientis]"
ORIGIN
        1 ggttgtccgg aaactagtaa attcaagcat attccctctt taacataacc ctactcatag
       61 ctaggtatat gaaattctca gtggatgttg tgagtaaatc atactccttc gatagccttc
      121 tattcctatt aacccaagca aaagtccttt ctacaaccca tcttcttggc tgtactttaa
      181 acccttgttc tcttgttggt agtagctcag gtggcgtatc tttgtgcacc caaaatctac
      241 atggaggcct tttaacaatt tcaatatcta tgtcatattc ttcctttatg tgattcttta
      301 aatttcttcc ttggtatccc atgtcagccc acattttttt aactttagta tattttgttc
      361 tcatattgtt taatgctatt ttaataccat ctctatcatt ttcgttagca gcgcctacgt
      421 aacaacctag tataaaaccc tgagtgtctg taattatatg ccttttttcta ccctttactt
      481 ttttacttcc atcatagcct ttgatccccc cttttctgta gtctttacag attgactatc
      541 tactatacag gcactcggct gctcattcct tcctattttt cttctactat attttgtaat
      601 ttcataattc attttctcaa aaattccctg cttcttccat tgcctgaact gctcatacac
      661 agtcttccat agcggaaaat catttggtaa ataccgccat tgacaccctg tacgcaatac
      721 acagaaaatt gcttctaata tttctctttt gctatacttt ggcggccttc ctcctttctt
      781 gtatgatact ctgaagtgtt tttctattct tgcccattcc ctttcgctta gatctgttgg
      841 atactttttt ctcatcttta ccccgtacta aaatttcaga tattatagcc ttttacttat
      901 ttccggacaa cctcttactc atgctatatt ttgcgagaat agtcaagctg taaaggatat
      961 tttgcaacaa gccgaattaa gcaacctttt aaagagtgtt cttagtgcaa aaataattag
     1021 tgatgattca ttaatttaca caccacttac ttatgccata ttatgtaaaa atagcaaagc
     1081 tattgaagaa attttaaaag tctctaaagg aaacgatatt ctgaaagata ttcttaacgc
     1141 aacaacaact ataaaatatc tagatggtcg gagagcaact gttacaccac ttgcttgtgc
     1201 tatactatgt ggagatagta tagctattga agaaatttta aaagtctcta aggagaaaga
     1261 tattcttaac acaacagcaa ctataaaata tccagatagc cagaaagtga cttacacacc
     1321 acttacttat gctatgttct ggaaggatag caaagctatt aaagaaattc tagtagcagc
     1381 taaagggaac agtactcttc tgaaagatat tcttaacaca acaacaacta tgaaatatct
     1441 agaccgggaa gtgacttaca caccacttgc ttatgttata ctacgtaga
//
LOCUS       JAQZAU010000006            911 bp    dna     linear   ENV 17-MAR-2023
DEFINITION  MAG: Wolbachia pipientis isolate wEsol contig_12340, whole genome
            shotgun sequence.
ACCESSION   JAQZAU010000006 JAQZAU010000000
VERSION     JAQZAU010000006.1
DBLINK      BioProject: PRJNA929258
            BioSample: SAMN33142973
KEYWORDS    WGS; ENV; Metagenome Assembled Genome; MAG.
SOURCE      Wolbachia pipientis (insect metagenome)
  ORGANISM  Wolbachia pipientis
            Bacteria; Pseudomonadota; Alphaproteobacteria; Rickettsiales;
            Anaplasmataceae; Wolbachieae; Wolbachia.
REFERENCE   1  (bases 1 to 911)
  AUTHORS   Fiutek,N., Couger,B.M., Roy,S., de la Torre,J.R. and Connor,E.F.
  TITLE     Genomic assessment of the contribution of the Wolbachia
            endosymbiont of Eurosta solidaginis to gall-induction
  JOURNAL   International Journal Molecular Sciences
REFERENCE   2  (bases 1 to 911)
  AUTHORS   Fiutek,N., Couger,B.M., Roy,S., de la Torre,J.R. and Connor,E.F.
  TITLE     Direct Submission
  JOURNAL   Submitted (10-FEB-2023) Biology, San Francisco State University,
            1600 Holloway Ave, San Francisco, CA 94132, USA
COMMENT     ##Genome-Assembly-Data-START##
            Assembly Date          :: JUN-2019
            Assembly Method        :: FLYE v. 06-2019
            Genome Representation  :: Full
```

```
          Expected Final Version :: Yes
          Genome Coverage        :: 100.0x
          Sequencing Technology  :: Illumina HiSeq; PacBio Sequel
          ##Genome-Assembly-Data-END##
FEATURES             Location/Qualifiers
     source          1..911
                     /organism="Wolbachia pipientis"
                     /mol_type="genomic DNA"
                     /submitter_seqid="contig_12340"
                     /isolate="wEsol"
                     /isolation_source="galls on Solidago altissima"
                     /host="Eurosta solidaginis MN-2018-EFC"
                     /db_xref="taxon:955"
                     /environmental_sample
                     /country="USA: Cowling Arboretum, Carleton College,
                     Northfield, MN"
                     /lat_lon="44.48 N 93.13 W"
                     /altitude="280 m"
                     /collection_date="2018-11"
                     /metagenome_source="insect metagenome"
                     /note="metagenomic"
     gene            complement(74..256)
                     /locus_tag="wEsol_00081"
     CDS             complement(74..256)
                     /locus_tag="wEsol_00081"
                     /codon_start=1
                     /transl_table=11
                     /product="putative transposase"
                     /translation="MLKGLHVDFGCTKIRTPELLPTREQGFKVQPRRWVVERTFAWVN
                     RNRRLSKEYDLLHNIH"
     gene            complement(204..362)
                     /locus_tag="wEsol_00082"
     CDS             complement(204..362)
                     /locus_tag="wEsol_00082"
                     /codon_start=1
                     /transl_table=11
                     /product="hypothetical protein"
                     /translation="MRTKYTKVKKMWADMGYQGRNLKNHIKEEYDIDIEIVKRPPCRF
                     WVHKDTHT"
                     /besthit="qcoverage 96.1538461538462 , hcoverage
                     73.5294117647059 , similarity 100 , identity 100 , evalue
                     1.69e-31 , alnlength 50 , Bacteria , Proteobacteria ,
                     EAL57896.1 transposase, IS5 family, OrfB, partial
                     [Wolbachia endosymbiont of Drosophila ananassae]"
     gene            complement(473..610)
                     /locus_tag="wEsol_00083"
     CDS             complement(473..610)
                     /locus_tag="wEsol_00083"
                     /codon_start=1
                     /transl_table=11
                     /fullproduct="qcoverage 100 , hcoverage 81.8181818181818
                     , similarity 100 , identity 100 , evalue 3.71e-22 ,
                     alnlength 45 , Bacteria , Proteobacteria , EAL58108.1
                     transposase, IS5 family, OrfA, partial [Wolbachia
                     endosymbiont of Drosophila ananassae]"
                     /translation="MNYEITKYSRRKIGRNEQPSACIVDSQSVKTTEKGGSKAMMEVK
                     K"
                     /product="transposase, IS5 family, OrfA, partial"
                     /besthit="qcoverage 100 , hcoverage 81.8181818181818 ,
                     similarity 100 , identity 100 , evalue 3.71e-22 ,
```

```
                     alnlength 45 , Bacteria , Proteobacteria , EAL58108.1
                     transposase, IS5 family, OrfA, partial [Wolbachia
                     endosymbiont of Drosophila ananassae]"
     gene            complement(607..852)
                     /locus_tag="wEsol_00084"
     CDS             complement(607..852)
                     /locus_tag="wEsol_00084"
                     /codon_start=1
                     /transl_table=11
                     /product="hypothetical protein"
                     /pfam="DUF4096"
                     /translation="MRKKYPTDLSEREWARIEKHFRVSYKKGGRPPKYSKREILEQFS
                     MYCVQGVNGGIYQMIFRYGRLCMSSSGNGRSREFLRK"
                     /besthit="qcoverage 50.6172839506173 , hcoverage
                     80.3921568627451 , similarity 100 , identity 100 , evalue
                     1.76e-20 , alnlength 41 , Bacteria , Proteobacteria ,
                     WP_038198919.1 transposase, partial [Wolbachia
                     pipientis]"
     gene            834..>911
                     /locus_tag="wEsol_00085"
     CDS             834..>911
                     /locus_tag="wEsol_00085"
                     /codon_start=1
                     /transl_table=11
                     /product="hypothetical protein"
                     /translation="MDTFFSSLPRTKISDIIAFYLFPDNS"
ORIGIN
        1 tccggaaact agtaaattca agcatattcc ctctttaaca taaccctaac tcatagctag
       61 gtatatgaaa ttctcagtgg atgttgtgaa gtaaatcata ctccttcgat agccttctat
      121 tcctattaac ccaagcaaaa gtcctttcta caacccatct tcttggctgt actttaaacc
      181 cttgttctct tgttggtagt agctcaggtg tgcgtatctt tgtgcaccca aaatctacat
      241 ggaggccttt taacaatttc aatatctatg tcatattctt cctttatgtg attctttaaa
      301 tttcttcctt ggtatcccat gtcagcccac attttttaa ctttagtata ttttgttctc
      361 atattgttta atgctatttt aataccatct ctatcatttt cgttagcagc gcctacgtaa
      421 caacctagta taaaaccctg agtgtctgta attatatgcc tttttctacc ctttactttt
      481 ttacttccat catagccttt gatccccccct tttctgtagt ctttacagat tgactatcta
      541 ctatacaggc actcggctgc tcattccttc ctatttttct tctactatat tttgtaattt
      601 cataattcat tttctcaaaa attccctgct tcttccattg cctgaactgc tcatacacag
      661 tcttccatag cggaaaatca tttggtaaat accgccattg acaccctgta cgcaatacat
      721 agaaaattgc tctaatattt ctcttttgct atactttggc ggccttcctc ctttcttgta
      781 tgatactctg aagtgttttt ctattcttgc ccattccctt tcgcttagat ctgttggata
      841 ctttttctc atctttaccc cgtactaaaa tttcagatat tatagccttt tacttatttc
      901 cggacaactc t
//
LOCUS       JAQZAU010000007            1277 bp    dna     linear   ENV 17-MAR-2023
DEFINITION  MAG: Wolbachia pipientis isolate wEsol contig_12505, whole genome
            shotgun sequence.
ACCESSION   JAQZAU010000007 JAQZAU010000000
VERSION     JAQZAU010000007.1
DBLINK      BioProject: PRJNA929258
            BioSample: SAMN33142973
KEYWORDS    WGS; ENV; Metagenome Assembled Genome; MAG.
SOURCE      Wolbachia pipientis (insect metagenome)
  ORGANISM  Wolbachia pipientis
            Bacteria; Pseudomonadota; Alphaproteobacteria; Rickettsiales;
            Anaplasmataceae; Wolbachieae; Wolbachia.
REFERENCE   1  (bases 1 to 1277)
  AUTHORS   Fiutek,N., Couger,B.M., Roy,S., de la Torre,J.R. and Connor,E.F.
  TITLE     Genomic assessment of the contribution of the Wolbachia
            endosymbiont of Eurosta solidaginis to gall-induction
```

```
  JOURNAL   International Journal Molecular Sciences
REFERENCE   2  (bases 1 to 1277)
  AUTHORS   Fiutek,N., Couger,B.M., Roy,S., de la Torre,J.R. and Connor,E.F.
  TITLE     Direct Submission
  JOURNAL   Submitted (10-FEB-2023) Biology, San Francisco State University,
            1600 Holloway Ave, San Francisco, CA 94132, USA
COMMENT     ##Genome-Assembly-Data-START##
            Assembly Date          :: JUN-2019
            Assembly Method        :: FLYE v. 06-2019
            Genome Representation  :: Full
            Expected Final Version :: Yes
            Genome Coverage        :: 100.0x
            Sequencing Technology  :: Illumina HiSeq; PacBio Sequel
            ##Genome-Assembly-Data-END##
FEATURES             Location/Qualifiers
     source          1..1277
                     /organism="Wolbachia pipientis"
                     /mol_type="genomic DNA"
                     /submitter_seqid="contig_12505"
                     /isolate="wEsol"
                     /isolation_source="galls on Solidago altissima"
                     /host="Eurosta solidaginis MN-2018-EFC"
                     /db_xref="taxon:955"
                     /environmental_sample
                     /country="USA: Cowling Arboretum, Carleton College,
                     Northfield, MN"
                     /lat_lon="44.48 N 93.13 W"
                     /altitude="280 m"
                     /collection_date="2018-11"
                     /metagenome_source="insect metagenome"
                     /note="metagenomic"
     gene            <1..110
                     /locus_tag="wEsol_00086"
     CDS             <1..110
                     /locus_tag="wEsol_00086"
                     /codon_start=1
                     /transl_table=11
                     /product="hypothetical protein"
                     /translation="XRSGQLSIDLTQQKSGEKVWNVFYKKNDASNCSGMQ"
     gene            complement(111..275)
                     /locus_tag="wEsol_00087"
     CDS             complement(111..275)
                     /locus_tag="wEsol_00087"
                     /codon_start=1
                     /transl_table=11
                     /fullproduct="qcoverage 100 , hcoverage 100 , similarity
                     100 , identity 100 , evalue 2.14e-30 , alnlength 54 ,
                     Bacteria , Proteobacteria , WP_010082072.1 MULTISPECIES:
                     IS3 family transposase [Wolbachia]"
                     /cog="Tra5 COG2801 232 Transposase and inactivated
                     derivatives"
                     /pfam="rve_3 rve_2"
                     /product="IS3 family transposase"
                     /translation="MSNKGCCYDNSVAESFFSSLKREILIDTSQHSAQQTRTAIFEYI
                     EIFYNKQRHY"
                     /besthit="qcoverage 100 , hcoverage 100 , similarity 100
                     , identity 100 , evalue 2.14e-30 , alnlength 54 ,
                     Bacteria , Proteobacteria , WP_010082072.1 MULTISPECIES:
                     IS3 family transposase [Wolbachia]"
     gene            complement(272..442)
```

```
                     /locus_tag="wEsol_00088"
     CDS             complement(272..442)
                     /locus_tag="wEsol_00088"
                     /codon_start=1
                     /transl_table=11
                     /fullproduct="qcoverage 87.5 , hcoverage 96.078431372549
                     , similarity 89.8 , identity 83.7 , evalue 3.55e-18 ,
                     alnlength 49 , Bacteria , Proteobacteria , MBA8756266.1
                     hypothetical protein [Wolbachia pipientis]"
                     /translation="MVVMTYDAINKQLVMNSLLMDVNKRKPAKNLLLHSDQGSQYTLL
                     RVSISLSCKEYR"
                     /product="hypothetical protein"
                     /besthit="qcoverage 87.5 , hcoverage 96.078431372549 ,
                     similarity 89.8 , identity 83.7 , evalue 3.55e-18 ,
                     alnlength 49 , Bacteria , Proteobacteria , MBA8756266.1
                     hypothetical protein [Wolbachia pipientis]"
     gene            complement(873..1052)
                     /locus_tag="wEsol_00089"
     CDS             complement(873..1052)
                     /locus_tag="wEsol_00089"
                     /codon_start=1
                     /transl_table=11
                     /product="hypothetical protein"
                     /translation="MHFQVDGNMAPYDKERLQFNKQEITRERDIYKRKPSASTKKELK
                     YFYYILVTAISTGIM"
     gene            complement(1182..>1277)
                     /locus_tag="wEsol_00090"
     CDS             complement(1182..>1277)
                     /locus_tag="wEsol_00090"
                     /codon_start=1
                     /transl_table=11
                     /product="hypothetical protein"
                     /translation="EERKEFFLGKINFGGLEMVREYTRRIQIGSC"
ORIGIN
        1 agagaagtgg acagttgtca attgacctta cccagcaaaa aagtggagag aaagtgtgga
       61 atgtttttta caaaaagaat gatgcatcaa attgttctgg aatgcaatag ttaatagtga
      121 cgttgtttgt tataaaaaat ttctatgtat tcaaatattg cagttctagt ttgttgagca
      181 gagtgttgtg aagtatcaat aagtatttct cttttcaatg agctaaagaa gctttctgca
      241 acagaattgt cgtaacaaca gcctttatta ctcatctata ttctttacag ctaagagata
      301 ttgataccct tagcaaagta tattgtgaac cttgatcact atgtaatagt agatttttgg
      361 caggtttacg cttgttaacg tccattaata aagagttcat aaccaattgt ttatttattg
      421 catcatacgt cattactacc atacgtggag catatattga ctaaagccaa atcatagcca
      481 tcaagaaaaa ccctccttag gttttagatg tagataataa aacccatact ttatttattt
      541 ggttgatcga aacaataaag tatttgtgat ctagtagatt gggagtctac agacatctat
      601 tatctgtttg ttgtttttta aaattttaaa cttttcttct cagtatagtc cctgatgttc
      661 atttctgca tataatactt tgcaccgttt tcaaatttgc atgcaacttt tacccaaagc
      721 tcaattcagc atgaatttta gggagctcca tatctacatt taaatagctt ggatatattt
      781 tttgaatatc tgctaatata cgtcttttat ttgctgattc ctactgcttt atttttctgt
      841 ggtccactta gagtagccac tagcagaaaa tcctacataa ttcctgtact tatagcagtt
      901 actaatatat aataaaaata ttttaactct ttttttgtac tggccgaggg ctttctttta
      961 taaatgtctc tttcccttgt tatctcttgc ttgttaaatt gtaacctctc tttgtcgtaa
     1021 ggagccatgt taccatctac ctggaaatgc atctgctgcc gactttttt tccgttgtgt
     1081 ttctttatcc attttcctaa taaaccaccc tttattccta aatcttcttg ctattttttgt
     1141 aattgtttgt gacctgcctt ttctttgtca tgttgtccag tttaacagct tccaatttga
     1201 attctgcgcg tatattctct taccatttct aatcctccaa aatttatttt acccaaaaaa
     1261 aactcttttc tttcctc
//
LOCUS       JAQZAU010000008         5990 bp    dna     linear   ENV 17-MAR-2023
DEFINITION  MAG: Wolbachia pipientis isolate wEsol contig_12536, whole genome
            shotgun sequence.
```

```
ACCESSION   JAQZAU010000008 JAQZAU010000000
VERSION     JAQZAU010000008.1
DBLINK      BioProject: PRJNA929258
            BioSample: SAMN33142973
KEYWORDS    WGS; ENV; Metagenome Assembled Genome; MAG.
SOURCE      Wolbachia pipientis (insect metagenome)
  ORGANISM  Wolbachia pipientis
            Bacteria; Pseudomonadota; Alphaproteobacteria; Rickettsiales;
            Anaplasmataceae; Wolbachieae; Wolbachia.
REFERENCE   1  (bases 1 to 5990)
  AUTHORS   Fiutek,N., Couger,B.M., Roy,S., de la Torre,J.R. and Connor,E.F.
  TITLE     Genomic assessment of the contribution of the Wolbachia
            endosymbiont of Eurosta solidaginis to gall-induction
  JOURNAL   International Journal Molecular Sciences
REFERENCE   2  (bases 1 to 5990)
  AUTHORS   Fiutek,N., Couger,B.M., Roy,S., de la Torre,J.R. and Connor,E.F.
  TITLE     Direct Submission
  JOURNAL   Submitted (10-FEB-2023) Biology, San Francisco State University,
            1600 Holloway Ave, San Francisco, CA 94132, USA
COMMENT     ##Genome-Assembly-Data-START##
            Assembly Date          :: JUN-2019
            Assembly Method        :: FLYE v. 06-2019
            Genome Representation  :: Full
            Expected Final Version :: Yes
            Genome Coverage        :: 100.0x
            Sequencing Technology  :: Illumina HiSeq; PacBio Sequel
            ##Genome-Assembly-Data-END##
FEATURES             Location/Qualifiers
     source          1..5990
                     /organism="Wolbachia pipientis"
                     /mol_type="genomic DNA"
                     /submitter_seqid="contig_12536"
                     /isolate="wEsol"
                     /isolation_source="galls on Solidago altissima"
                     /host="Eurosta solidaginis MN-2018-EFC"
                     /db_xref="taxon:955"
                     /environmental_sample
                     /country="USA: Cowling Arboretum, Carleton College,
                     Northfield, MN"
                     /lat_lon="44.48 N 93.13 W"
                     /altitude="280 m"
                     /collection_date="2018-11"
                     /metagenome_source="insect metagenome"
                     /note="metagenomic"
     gene            complement(<1..256)
                     /locus_tag="wEsol_00091"
     CDS             complement(<1..256)
                     /locus_tag="wEsol_00091"
                     /codon_start=1
                     /transl_table=11
                     /fullproduct="qcoverage 100 , hcoverage 100 , similarity
                     100 , identity 100 , evalue 2.25e-59 , alnlength 85 ,
                     Bacteria , Proteobacteria , WP_044471356.1 IS5 family
                     transposase, partial [Wolbachia endosymbiont of
                     Drosophila suzukii]"
                     /cog="COG3293 COG3293 124 Transposase and inactivated
                     derivatives"
                     /pfam="DUF4096"
                     /product="IS5 family transposase, partial"
                     /translation="MRKKYPTDLSEREWARIEKHFRVSYKKGGRPPKYSKREILEAIF
```

YVLRTGCQWRYLPNDFPLWKTVYEQFRQWKKQGIFEKMNYE"
/besthit="qcoverage 100 , hcoverage 100 , similarity 100 , identity 100 , evalue 2.25e-59 , alnlength 85 , Bacteria , Proteobacteria , WP_044471356.1 IS5 family transposase, partial [Wolbachia endosymbiont of Drosophila suzukii]"

gene 339..1859
/locus_tag="wEsol_00092"

CDS 339..1859
/locus_tag="wEsol_00092"
/codon_start=1
/transl_table=11
/fullproduct="qcoverage 100 , hcoverage 100 , similarity 100 , identity 100 , evalue 0.0 , alnlength 506 , Bacteria , Proteobacteria , WP_174516815.1 MULTISPECIES: HlyD family type I secretion periplasmic adaptor subunit [Wolbachia]"
/cog="AcrA COG0845 372 Membrane-fusion protein"
/pfam="HlyD_2 HlyD Biotin_lipoyl_2 Biotin_lipoyl Pfam-B_12304 HlyD_3"
/tigr="TIGR01843 type_I_hlyD 422 type I secretion membrane fusion protein, HlyD family"
/product="HlyD family type I secretion periplasmic adaptor subunit"
/translation="MISKLKKLSAGFSLTGLIGHGDNINMQGAKNKKKKYPKFLDKTF
ALIDAVINFILKRESNNVNEVLKVTWGPLFFGLVVILIFFGIGGIWSAIAPIDGAVHA
SGEVIVSSNRKIVQHLGGGIISKILVKEGQAVKKDEPLVLLSDVNEKANLSIIKEKLL
SFLATEARLLAIRVDLDTLEFPDEVKKLSHDELVNKAIKNQVKLFNSQRKSILGKTDI
LQQRIKQLNDELAGLNFQLNAAHKQYDLITEELETKRQLLDSGHISKPHILALEKQFA
EIEGRVGHYRSAISQVQQKIGENELEIINVRNDSQERANAELKEVSTSIADLKERLMV
AEDSLARTIIKSPQDGIVTDIRYHTEGGVIQSGVPIMSVVPSDDDLIIDAKIQTRNIE
EILSAQKKDSNIVSIDGLEGLKVKVRLSAYSARRLSLINGIVSNISPDALDDPRLGRY
YSVRVVIPKPELAQFKNVYLYPGMPAEVYIVTQSRTLLSFLFTPIIATVDRSFIER"
/besthit="qcoverage 100 , hcoverage 100 , similarity 100 , identity 100 , evalue 0.0 , alnlength 506 , Bacteria , Proteobacteria , WP_174516815.1 MULTISPECIES: HlyD family type I secretion periplasmic adaptor subunit [Wolbachia]"

gene 2002..2244
/locus_tag="wEsol_00093"

CDS 2002..2244
/locus_tag="wEsol_00093"
/codon_start=1
/transl_table=11
/fullproduct="qcoverage 100 , hcoverage 100 , similarity 100 , identity 100 , evalue 4.41e-48 , alnlength 80 , Bacteria , Proteobacteria , WP_155968338.1 transposase [Wolbachia pipientis]"
/cog="COG2963 COG2963 116 Transposase and inactivated derivatives"
/pfam="HTH_Tnp_1 HTH_23 HTH_28"
/product="transposase"
/translation="MAREYTAEFKLEAVKLANEQRKAGQTITKIAKDLGIKGGLLGKW
IKKHNEKKSAANAFPDVAPYDKERFDLQKELEVVRK"
/besthit="qcoverage 100 , hcoverage 100 , similarity 100 , identity 100 , evalue 4.41e-48 , alnlength 80 , Bacteria , Proteobacteria , WP_155968338.1 transposase [Wolbachia pipientis]"

gene 2318..2698
/locus_tag="wEsol_00094"

CDS 2318..2698

```
                     /locus_tag="wEsol_00094"
                     /codon_start=1
                     /transl_table=11
                     /fullproduct="qcoverage 100 , hcoverage 100 , similarity
                     100 , identity 100 , evalue 1.16e-87 , alnlength 126 ,
                     Bacteria , Proteobacteria , AOV87106.1 transposase
                     [Wolbachia endosymbiont of Drosophila incompta]"
                     /cog="COG3293 COG3293 124 Transposase and inactivated
                     derivatives"
                     /pfam="DUF4096"
                     /product="transposase"
                     /translation="MRKKYPTDLSEREWARIEKHFRVSYKKGGRPPKYSKREILEAIF
                     YVLRTGCQWRYLPNDFPLWKTVYEQFRQWKKQGIFEKMNYEITKYSRRKIGRNEQPSA
                     CIVDSQSVKTTEKGGSKAMMEVKK"
                     /besthit="qcoverage 100 , hcoverage 100 , similarity 100
                     , identity 100 , evalue 1.16e-87 , alnlength 126 ,
                     Bacteria , Proteobacteria , AOV87106.1 transposase
                     [Wolbachia endosymbiont of Drosophila incompta]"
     gene            2809..3150
                     /locus_tag="wEsol_00095"
     CDS             2809..3150
                     /locus_tag="wEsol_00095"
                     /codon_start=1
                     /transl_table=11
                     /fullproduct="qcoverage 100 , hcoverage 100 , similarity
                     100 , identity 100 , evalue 1.53e-79 , alnlength 113 ,
                     Bacteria , Proteobacteria , AAS13810.1 transposase, IS5
                     family, OrfB [Wolbachia endosymbiont of Drosophila
                     melanogaster]"
                     /cog="COG3293 COG3293 124 Transposase and inactivated
                     derivatives"
                     /pfam="DDE_Tnp_1_2 DDE_Tnp_1"
                     /product="transposase, IS5 family, OrfB"
                     /translation="MRTKYTKVKKMWADMGYQGRNLKNHIKEEYDIDIEIVKRPPCRF
                     WVHKDTPPELLPTREQGFKVQPRRWVVERTFAWVNRNRRLSKEYDLLTTSTENFIYLA
                     MSRVMLKREYA"
                     /besthit="qcoverage 100 , hcoverage 100 , similarity 100
                     , identity 100 , evalue 1.53e-79 , alnlength 113 ,
                     Bacteria , Proteobacteria , AAS13810.1 transposase, IS5
                     family, OrfB [Wolbachia endosymbiont of Drosophila
                     melanogaster]"
     gene            3197..3703
                     /locus_tag="wEsol_00096"
     CDS             3197..3703
                     /locus_tag="wEsol_00096"
                     /codon_start=1
                     /transl_table=11
                     /product="hypothetical protein"
                     /pfam="DnaJ"
                     /translation="MARNVLNENEYHALKQLFLTFNIQKEDVVNSDIKNIKKKLSQQY
                     RKLSLKYHSDKNPNNKEAEEKFKVIALNKPFFEEFIIEPLEKGLNKRNNLIKELRGKL
                     WSRSYEELQKYLDDKCRNKLYNKQQSINNNVTAYVKYSSYSNLLGVLLVSFTSIAFLI
                     INGINYVF"
                     /besthit="qcoverage 98.2142857142857 , hcoverage
                     47.5504322766571 , similarity 100 , identity 99.4 ,
                     evalue 2.50e-103 , alnlength 165 , Bacteria ,
                     Proteobacteria , QVU15912.1 DnaJ domain protein
                     [Wolbachia endosymbiont of Drosophila yakuba]"
     gene            complement(3887..4408)
                     /locus_tag="wEsol_00097"
```

```
     CDS             complement(3887..4408)
                     /locus_tag="wEsol_00097"
                     /codon_start=1
                     /transl_table=11
                     /fullproduct="qcoverage 100 , hcoverage 100 , similarity
                     100 , identity 100 , evalue 4.69e-112 , alnlength 173 ,
                     Bacteria , Proteobacteria , RLT59977.1 transposase
                     IS116/IS110/IS902 family protein [Wolbachia endosymbiont
                     of Drosophila ananassae]"
                     /cog="COG3547 COG3547 303 Transposase and inactivated
                     derivatives"
                     /pfam="Transposase_20"
                     /product="transposase IS116/IS110/IS902 family protein"
                     /translation="MRTQEKCRLEAPENDHIKESCQKTIEFFDSQINELNDTIQKIID
                     ESRELQQRQKILKTVPGIGKKLSQDFLCLMPELGYLNRKEVASLAGVAPHPKESGKAV
                     GYRRITGGRSNVRSKLFTAAMAATRSKSILGAFYSKLVESGKKKMVAITALMRKIIVI
                     ANTRLKEAINLHT"
                     /besthit="qcoverage 100 , hcoverage 100 , similarity 100
                     , identity 100 , evalue 4.69e-112 , alnlength 173 ,
                     Bacteria , Proteobacteria , RLT59977.1 transposase
                     IS116/IS110/IS902 family protein [Wolbachia endosymbiont
                     of Drosophila ananassae]"
     gene            4738..4845
                     /locus_tag="wEsol_00098"
     CDS             4738..4845
                     /locus_tag="wEsol_00098"
                     /codon_start=1
                     /transl_table=11
                     /product="hypothetical protein"
                     /pfam="HTH_Tnp_IS630"
                     /translation="MAYSVDLRERAVSMIEKGKSKVEVAELLEIGIATL"
                     /besthit="qcoverage 100 , hcoverage 52.2388059701493 ,
                     similarity 100 , identity 100 , evalue 3.26e-13 ,
                     alnlength 35 , Bacteria , Proteobacteria , WP_213863574.1
                     IS630 transposase-related protein, partial [Wolbachia
                     endosymbiont of Ceratitis capitata]"
     gene            5285..5596
                     /locus_tag="wEsol_00099"
     CDS             5285..5596
                     /locus_tag="wEsol_00099"
                     /codon_start=1
                     /transl_table=11
                     /fullproduct="qcoverage 100 , hcoverage 100 , similarity
                     100 , identity 100 , evalue 1.60e-74 , alnlength 103 ,
                     Bacteria , Proteobacteria , WP_155968470.1 transposase
                     [Wolbachia pipientis]"
                     /cog="COG3335 COG3335 132 Transposase and inactivated
                     derivatives"
                     /pfam="DDE_3 DDE_1"
                     /product="transposase"
                     /translation="MTFKGGCDKEVFNTWLEKMLLPKLPHGTTIVMDNATFHKTPKTK
                     ELIDNARCRLLYLPTYSPDLNPIEHCWNTIKSRLRPLMHKYTDLQLLVGNTIMEIYHS
                     F"
                     /besthit="qcoverage 100 , hcoverage 100 , similarity 100
                     , identity 100 , evalue 1.60e-74 , alnlength 103 ,
                     Bacteria , Proteobacteria , WP_155968470.1 transposase
                     [Wolbachia pipientis]"
     gene            complement(5517..5747)
                     /locus_tag="wEsol_00100"
     CDS             complement(5517..5747)
```

```
                     /locus_tag="wEsol_00100"
                     /codon_start=1
                     /transl_table=11
                     /product="hypothetical protein"
                     /pfam="DEDD_Tnp_IS110 Pfam-B_8792"
                     /translation="MVTSYQNFIGIDIGKFKNVVAIHKQKNAVEFDNNASGWQQLFQE
                     FSDIVFSKMNDKFPLWYYQPKVVSLYIYALKV"
                     /besthit="qcoverage 64.4736842105263 , hcoverage
                     59.0361445783133 , similarity 100 , identity 98.0 ,
                     evalue 7.61e-26 , alnlength 49 , Bacteria ,
                     Proteobacteria , WP_213863621.1 transposase, partial
                     [Wolbachia endosymbiont of Ceratitis capitata]"
ORIGIN
        1 tttcataatt cattttctca aaaattccct gcttcttcca ttgcctgaac tgctcataca
       61 cagtcttcca tagcggaaaa tcatttggta aataccgcca ttgacaccct gtacgcaata
      121 catagaaaat tgcttctaat atttctcttt tgctatactt tggcggcctt cctcctttct
      181 tgtatgatac tctgaagtgt ttttctattc ttgcccattc cctttcgctt agatctgttg
      241 gatacttttt tctcatcttt accccgtact aaaatttcag atattatagc cttttactta
      301 tttccggaca acctctaaca caaattcaca gtagtattat gataagtaag ttaaaaaagc
      361 ttagcgctgg attttctttg acaggcttga ttggccatgg tgataatata aatatgcaag
      421 gagcaaaaaa taaaaagaag aagtacccaa aatttttgga taagacgttt gcattgattg
      481 atgcagtgat aaatttttatt ttaaaacgcg aaagtaataa cgtaaatgaa gtcctgaaag
      541 taacatgggg accactattt tttggtctgg tagtaatact catctttttt gggataggtg
      601 gtatttggtc ggctatagct ccaattgacg gtgcggtgca tgcaagtgga gaagttattg
      661 tatcttcaaa tagaaaaata gttcaacact tgggcggagg aataataagc aaaattttag
      721 taaaagaggg tcaagcagtt aaaaaagatg agcctctagt tttgttgagt gatgttaacg
      781 agaaggcgaa tttaagtatc atcaaagaga agctcttatc atttttagca acagaagcaa
      841 ggcttcttgc aatcagagta gatttggaca cgctcgagtt tcctgatgag gttaaaaaat
      901 tatctcacga tgaacttgta aataaagcga taaagaatca agtaaagttg ttcaactctc
      961 agagaaagag catattggga aaaacagaca tactgcaaca acgtataaag cagttaaatg
     1021 atgaattagc ggggctaaac tttcaactaa atgcagctca taagcaatat gacttgataa
     1081 ctgaggagtt ggaaacaaaa agacaattac ttgatagtgg ccatataagc aaaccacaca
     1141 ttttagcttt agaaaagcag tttgctgaaa ttgaaggcag agttggacat taccgttctg
     1201 caatatctca agtgcagcaa aaaattggag agaacgaatt agaaattata aacgtgagaa
     1261 atgattctca agaaagagca aacgctgaac ttaaggaagt tagtacgtct attgcagact
     1321 tgaaagagag actgatggtt gctgaagatt cattagcacg tacaataatt aaatcgcctc
     1381 aagatgggat agtcacagat ataagatacc atactgaagg tggtgttata caatctggag
     1441 ttccgatcat gagcgtagtg ccatcggatg atgacctaat aatagatgct aaaattcaga
     1501 ccagaaacat agaagagata ctgtcagcac aaaagaaaga tagcaacata gtctctattg
     1561 atgggctaga agggctaaag gttaaagttc gtttaagtgc ttacagtgca cgtcgtttaa
     1621 gtttaattaa tggtatagta agcaatattt ctcccgatgc ccttgatgat ccaaggctag
     1681 ggcgttatta ttcagtacgt gtagtgatac caaaaccaga gcttgctcag tttaaaaacg
     1741 tatacctata ccctggtatg ccagcagaag tgtacatagt tactcaatct cgtactcttt
     1801 tatcattctt gtttacgcct ataattgcaa cagtcgatag gtcttttata gaaaggtagc
     1861 tcactcgtta aagtagtagt gaaagtgccg taataaaatt taggtacata attagacaat
     1921 aggcttcttg accttaccca gaaaaagtgg agagaaagaa aggagttttt tcgggtaaaa
     1981 taaaattttg gaggattaga aatggcaaga gaatatacgg cagaattcaa attggaagct
     2041 gttaagctgg caaatgaaca aagaaaggca ggtcaaacaa ttacaaaaat agcaaaggat
     2101 ttaggaataa agggtggttt attaggaaaa tggataaaga aacacaacga aaaaaagtca
     2161 gcggcaaatg catttccaga tgtagcacct tatgacaaag agagatttga tttacagaaa
     2221 gagttagagg ttgtccggaa ataagtaaaa ggctataaga ggttgtccgg aaataagtaa
     2281 aaggctataa tatctgaaat tttagtacgg ggtaaagatg agaaaaaagt atccaacaga
     2341 tctaagcgaa agggaatggg caagaataga aaaacacttc agagtatcat acaagaaagg
     2401 aggaaggccg ccaaagtata gcaaaagaga aatattagaa gcaattttct atgtattgcg
     2461 tacagggtgt caatggcggt atttaccaaa tgattttccg ctatggaaga ctgtgtatga
     2521 gcagttcagg caatggaaga agcagggaat ttttgagaaa atgaattatg aaattacaaa
     2581 atatagtaga agaaaaatag gaaggaatga gcagccgagt gcctgtatag tagatagtca
     2641 atctgtaaag actacagaaa agggggggatc aaaggctatg atggaagtaa aaaagtaaag
     2701 ggtagaaaaa ggcatataat tacagacact cagggtttta tactaggttg ttacgtaggc
     2761 gctgctaacg aaaatgatag agatggtatt aaaatagcat taaacaatat gagaacaaaa
```

```
     2821 tatactaaag ttaaaaaaat gtgggctgac atgggatacc aaggaagaaa tttaaagaat
     2881 cacataaagg aagaatatga catagatatt gaaattgtta aaaggcctcc atgtagattt
     2941 tgggtgcaca aagatacgcc acctgagcta ctaccaacaa gagaacaagg gtttaaagta
     3001 cagccaagaa gatgggttgt agaaaggact tttgcttggg ttaataggaa tagaaggcta
     3061 tcgaaggagt atgatttact cacaacatcc actgagaatt tcatatacct agctatgagt
     3121 agggttatgt taaagaggga atatgcttga atttactagt ttccggacaa cctcttagtt
     3181 aatttgtagg tgtaatgtgg ctagaaatgt tttgaatgaa aatgagtacc acgctttaaa
     3241 gcagttgttt ttaacattta atattcaaaa ggaggatgtg gtaaattctg acattaagaa
     3301 tattaagaaa aagcttagtc aacaataccg taaattatcg ttaaaatatc actcagataa
     3361 aaacccaaac aataaagaag cggaggagaa atttaaggtt attgcattaa ataagccatt
     3421 ttttgaagaa tttataatag agccattgga gaaaggtctc aataaaagaa ataatttaat
     3481 aaaagaatta cgcggtaagt tgtggagtcg cagttatgaa gaattgcaaa aatacttaga
     3541 tgataagtgc agaaataaac tctataataa gcagcagtca attaacaata atgtaactgc
     3601 ttatgtaaaa tattcttctt attcaaatct cttaggagtt cttttagtat ccttcacatc
     3661 aattgcattt ttaataatta atggtatcaa ctatgttttt taaaaatttt tttatttgtg
     3721 taattttgca aattatatgg gcaataaaaa cccgtttgtc atagaaatag aattttatat
     3781 caaacagcta ccacagcaat ttctgcttaa gctaatgaat atgtgcttta agtctgtgtg
     3841 gacgcacata ttcattagcc aatttcagtt accgttgtga agatttttaa gtatgcaaat
     3901 taattgcttc tttaagtctt gtattagcaa ttactataat tttacgcatt agggctgtta
     3961 tagccaccat cttcttctta ccactttcaa caagcttaga ataaaaggcg ccaagtatag
     4021 atttcgacct tgtagcagcc atagcggctg taaagagctt tgaacgaacg ttacttctac
     4081 cacctgtaat ccttcggtaa ccaacagctt taccactttc ttttggatgc ggtgcaactc
     4141 cggcaagact tgctacttct tttctgttta agtaaccaag ctctggcatt aaacacaaaa
     4201 aatcttgtga taacttttta cctattccag gaactgtttt aagaattttt tggcgttgtt
     4261 gtaattcgcg gctttcatca ataatctttt gtatagtatc attgagttcg tttatttgac
     4321 tatcgaaaaa ctcaatggtc ttttgacagc tttcttttat atgatcattt tccggtgctt
     4381 caagtctgca tttttcttga gttctcattt gcgtaatatc atcacgacgt tgacaaagtg
     4441 cagccaaagt tgattgttct gtagaggtag gtacaaatag agagatagtt ctatggcgtt
     4501 caaatccata ttgagcaaga gctcttgcat ctagactttg ctaaagttcc gtgagataaa
     4561 ataaagcttt ttactttacg agtattagct cgatgcacgg caatattttt gtcggtaaga
     4621 aaatgtgata agccaagctc atatttccct gtattttcta aagtcactaa agcattaggt
     4681 agaatatagt acttcccaaa gtaaatgata aaggtataat aagctatttg agttataatg
     4741 gcgtatagtg tggatttaag ggaaagggca gtatcgatga tagaaaaagg gaagtcaaag
     4801 gttgaggtgg cagagctttt ggagatagga atagcaactc tgtagcgctg gttaagaaag
     4861 aaagccgctg gtgaaagcct aaggccatcg aaaaacggca gctttattcg aaaaatagac
     4921 ccaaaaatac tcgaagaata tgttaaaaag aatccagatc atacgctggc agagatgaaa
     4981 caaaatcttg gatttggaat aaattcaatt tggtataggc tgaaacagct aagaattact
     5041 ttaaaaaaaa gttacactat atcaagagcg caatcaagaa gataggcagc gatttaccga
     5101 taaaatctca aaaatagacc attccagcat cttatatata gatgaagcag gagttgataa
     5161 taggttatac cgagagtatg gacgtgctcc aataggaaag aaaatttatg cagatattcc
     5221 aggaagaaaa cgagagagaa tcagtataat aggcgggtgg attggaaaga gatttattgc
     5281 accaatgact ttcaaaggtg ggtgtgacaa agaggtattc aatacatggt tagagaagat
     5341 gttattacct aaattgccac atggtactac aatagttatg gacaatgcca cattccataa
     5401 aactcccaaa acaaaggagt taatagataa tgctagatgc cgtttgctct atctaccaac
     5461 atattcacca gatttgaacc ctatagagca ttgttggaat accatcaaaa gccgcctcag
     5521 acctttaatg cataaatata cagacttaca acttttggtt ggtaatacca taatggaaat
     5581 ttatcattca ttttagaaaa tactatatct gaaaactctt gaaataattg ttgccaacca
     5641 gaagcattat tatcaaattc gacagcattc ttctgtttgt gaattgcaac gacattttta
     5701 aattttccga tgtcaatgcc aataaaattt tgataagatg taaccataat aaacctcgat
     5761 agttttagtt aatttaagat tgtaaacggg tgcatatata tgtcccaagc aactattcaa
     5821 acgtatcgag agataggggct agcgtacctt gataccaacg gttgtaatac caatttcctt
     5881 tcggtcgcgc acgcccgcta ttcctagtga gcagggttat ctttctctct tatctctttt
     5941 atatcacgtt tttaacttac aatttcttct aatgtaccaa tagcagtagg
//
LOCUS       JAQZAU010000009           1297 bp    dna     linear   ENV 17-MAR-2023
DEFINITION  MAG: Wolbachia pipientis isolate wEsol contig_13076, whole genome
            shotgun sequence.
ACCESSION   JAQZAU010000009  JAQZAU010000000
VERSION     JAQZAU010000009.1
DBLINK      BioProject: PRJNA929258
```

```
            BioSample: SAMN33142973
KEYWORDS    WGS; ENV; Metagenome Assembled Genome; MAG.
SOURCE      Wolbachia pipientis (insect metagenome)
  ORGANISM  Wolbachia pipientis
            Bacteria; Pseudomonadota; Alphaproteobacteria; Rickettsiales;
            Anaplasmataceae; Wolbachieae; Wolbachia.
REFERENCE   1  (bases 1 to 1297)
  AUTHORS   Fiutek,N., Couger,B.M., Roy,S., de la Torre,J.R. and Connor,E.F.
  TITLE     Genomic assessment of the contribution of the Wolbachia
            endosymbiont of Eurosta solidaginis to gall-induction
  JOURNAL   International Journal Molecular Sciences
REFERENCE   2  (bases 1 to 1297)
  AUTHORS   Fiutek,N., Couger,B.M., Roy,S., de la Torre,J.R. and Connor,E.F.
  TITLE     Direct Submission
  JOURNAL   Submitted (10-FEB-2023) Biology, San Francisco State University,
            1600 Holloway Ave, San Francisco, CA 94132, USA
COMMENT     ##Genome-Assembly-Data-START##
            Assembly Date          :: JUN-2019
            Assembly Method        :: FLYE v. 06-2019
            Genome Representation  :: Full
            Expected Final Version :: Yes
            Genome Coverage        :: 100.0x
            Sequencing Technology  :: Illumina HiSeq; PacBio Sequel
            ##Genome-Assembly-Data-END##
FEATURES             Location/Qualifiers
     source          1.1297
                     /organism="Wolbachia pipientis"
                     /mol_type="genomic DNA"
                     /submitter_seqid="contig_13076"
                     /isolate="wEsol"
                     /isolation_source="galls on Solidago altissima"
                     /host="Eurosta solidaginis MN-2018-EFC"
                     /db_xref="taxon:955"
                     /environmental_sample
                     /country="USA: Cowling Arboretum, Carleton College,
                     Northfield, MN"
                     /lat_lon="44.48 N 93.13 W"
                     /altitude="280 m"
                     /collection_date="2018-11"
                     /metagenome_source="insect metagenome"
                     /note="metagenomic"
     gene            complement(95..337)
                     /locus_tag="wEsol_00101"
     CDS             complement(95..337)
                     /locus_tag="wEsol_00101"
                     /codon_start=1
                     /transl_table=11
                     /product="hypothetical protein"
                     /translation="MPHGTTIVMDNATFHKTPKTKELIDNARCRLLYLPTYSPDLNPI
                     EHCWNTIKSRLRPLMHKYTDLQLLVGNTIMEIYHSF"
     gene            complement(374..715)
                     /locus_tag="wEsol_00102"
     CDS             complement(374..715)
                     /locus_tag="wEsol_00102"
                     /codon_start=1
                     /transl_table=11
                     /fullproduct="qcoverage 100 , hcoverage 100 , similarity
                     100 , identity 100 , evalue 1.53e-79 , alnlength 113 ,
                     Bacteria , Proteobacteria , AAS13810.1 transposase, IS5
                     family, OrfB [Wolbachia endosymbiont of Drosophila
```

```
                     melanogaster]"
                     /cog="COG3293 COG3293 124 Transposase and inactivated
                     derivatives"
                     /pfam="DDE_Tnp_1_2 DDE_Tnp_1"
                     /product="transposase, IS5 family, OrfB"
                     /translation="MRTKYTKVKKMWADMGYQGRNLKNHIKEEYDIDIEIVKRPPCRF
                     WVHKDTPPELLPTREQGFKVQPRRWVVERTFAWVNRNRRLSKEYDLLTTSTENFIYLA
                     MSRVMLKREYA"
                     /besthit="qcoverage 100 , hcoverage 100 , similarity 100
                     , identity 100 , evalue 1.53e-79 , alnlength 113 ,
                     Bacteria , Proteobacteria , AAS13810.1 transposase, IS5
                     family, OrfB [Wolbachia endosymbiont of Drosophila
                     melanogaster]"
     gene            complement(826..1206)
                     /locus_tag="wEsol_00103"
     CDS             complement(826..1206)
                     /locus_tag="wEsol_00103"
                     /codon_start=1
                     /transl_table=11
                     /fullproduct="qcoverage 100 , hcoverage 100 , similarity
                     99.2 , identity 99.2 , evalue 3.88e-86 , alnlength 126 ,
                     Bacteria , Proteobacteria , AOV87106.1 transposase
                     [Wolbachia endosymbiont of Drosophila incompta]"
                     /cog="COG3293 COG3293 124 Transposase and inactivated
                     derivatives"
                     /pfam="DUF4096"
                     /product="transposase"
                     /translation="MRKKYPTDLSEREWARIEKHFRVSYKKGGRPPKYSKREILEAIF
                     CVLRTGCQWRYLPNDFPLWKTVYEQFRQWKKQGIFEKMNYEITKYSRRKIGRNEQPSA
                     CIVDSQSVKTTEKGGSKAMMEVKK"
                     /besthit="qcoverage 100 , hcoverage 100 , similarity 99.2
                     , identity 99.2 , evalue 3.88e-86 , alnlength 126 ,
                     Bacteria , Proteobacteria , AOV87106.1 transposase
                     [Wolbachia endosymbiont of Drosophila incompta]"
ORIGIN
        1 tgaccttacc cagaaaaagt ggagagaaag tttggaatgt ttttcacaaa gagaatgatg
       61 aatcaaaatg ctcaggaatg caatatagta ttttctaaaa tgaatgataa atttccatta
      121 tggtattacc aaccaaaagt tgtaagtctg tatatttatg cattaaaggt ctgaggcggc
      181 ttttgatggt attccaacaa tgctctatag ggttcaaatc tggtgaatat gttggtagat
      241 agagcaaacg gcatctagca ttatctatta actcctttgt tttgggagtt ttatggaatg
      301 tggcattgtc cataactatt gtagtaccat gtggcaattt aggtaataag aggttgtccg
      361 gaaactagta aattcaagca tattccctct ttaacataac cctactcata gctaggtata
      421 tgaaattctc agtggatgtt gtgagtaaat catactcctt cgatagcctt ctattcctat
      481 taacccaagc aaaagtcctt tctacaaccc atcttcttgg ctgtacttta aacccttgtt
      541 ctcttgttgg tagtagctca ggtggcgtat ctttgtgcac ccaaaatcta catggaggcc
      601 ttttaacaat ttcaatatct atgtcatatt cttcctttat gtgattcttt aaatttcttc
      661 cttggtatcc catgtcagcc cacatttttt taactttagt atattttgtt ctcatattgt
      721 ttaatgctat tttaatacca tctctatcat tttcgttagc agcgcctacg taacaaccta
      781 gtataaaacc ctgagtgtct gtaattatat gccttttct accctttact tttttacttc
      841 catcatagcc tttgatcccc ccttttctgt agtctttaca gattgactat ctactataca
      901 ggcactcggc tgctcattcc ttcctatttt tcttctacta tattttgtaa tttcataatt
      961 cattttctca aaaattccct gcttcttcca ttgcctgaac tgctcataca cagtcttcca
     1021 tagcggaaaa tcatttggta aataccgcca ttgacaccct gtacgcaata cacagaaaat
     1081 tgcttctaat atttctcttt tgctatactt tggcggcctt cctcctttct tgtatgatac
     1141 tctgaagtgt ttttctattc ttgcccattc cctttcgctt agatctgttg gatacttttt
     1201 tctcatcttt accccgtact aaaatttcag atattatagc cttttactta tttccggaca
     1261 acctcttata gccttttact tatttccgga caatctc
//
LOCUS       JAQZAU010000010            1378 bp    dna     linear   ENV 17-MAR-2023
DEFINITION  MAG: Wolbachia pipientis isolate wEsol contig_14176, whole genome
```

```
            shotgun sequence.
ACCESSION   JAQZAU010000010 JAQZAU010000000
VERSION     JAQZAU010000010.1
DBLINK      BioProject: PRJNA929258
            BioSample: SAMN33142973
KEYWORDS    WGS; ENV; Metagenome Assembled Genome; MAG.
SOURCE      Wolbachia pipientis (insect metagenome)
  ORGANISM  Wolbachia pipientis
            Bacteria; Pseudomonadota; Alphaproteobacteria; Rickettsiales;
            Anaplasmataceae; Wolbachieae; Wolbachia.
REFERENCE   1  (bases 1 to 1378)
  AUTHORS   Fiutek,N., Couger,B.M., Roy,S., de la Torre,J.R. and Connor,E.F.
  TITLE     Genomic assessment of the contribution of the Wolbachia
            endosymbiont of Eurosta solidaginis to gall-induction
  JOURNAL   International Journal Molecular Sciences
REFERENCE   2  (bases 1 to 1378)
  AUTHORS   Fiutek,N., Couger,B.M., Roy,S., de la Torre,J.R. and Connor,E.F.
  TITLE     Direct Submission
  JOURNAL   Submitted (10-FEB-2023) Biology, San Francisco State University,
            1600 Holloway Ave, San Francisco, CA 94132, USA
COMMENT     ##Genome-Assembly-Data-START##
            Assembly Date          :: JUN-2019
            Assembly Method        :: FLYE v. 06-2019
            Genome Representation  :: Full
            Expected Final Version :: Yes
            Genome Coverage        :: 100.0x
            Sequencing Technology  :: Illumina HiSeq; PacBio Sequel
            ##Genome-Assembly-Data-END##
FEATURES             Location/Qualifiers
     source          1..1378
                     /organism="Wolbachia pipientis"
                     /mol_type="genomic DNA"
                     /submitter_seqid="contig_14176"
                     /isolate="wEsol"
                     /isolation_source="galls on Solidago altissima"
                     /host="Eurosta solidaginis MN-2018-EFC"
                     /db_xref="taxon:955"
                     /environmental_sample
                     /country="USA: Cowling Arboretum, Carleton College,
                     Northfield, MN"
                     /lat_lon="44.48 N 93.13 W"
                     /altitude="280 m"
                     /collection_date="2018-11"
                     /metagenome_source="insect metagenome"
                     /note="metagenomic"
     gene            59..439
                     /locus_tag="wEsol_00104"
     CDS             59..439
                     /locus_tag="wEsol_00104"
                     /codon_start=1
                     /transl_table=11
                     /fullproduct="qcoverage 100 , hcoverage 100 , similarity
                     100 , identity 100 , evalue 1.16e-87 , alnlength 126 ,
                     Bacteria , Proteobacteria , AOV87106.1 transposase
                     [Wolbachia endosymbiont of Drosophila incompta]"
                     /cog="COG3293 COG3293 124 Transposase and inactivated
                     derivatives"
                     /pfam="DUF4096"
                     /product="transposase"
                     /translation="MRKKYPTDLSEREWARIEKHFRVSYKKGGRPPKYSKREILEAIF
```

```
                     YVLRTGCQWRYLPNDFPLWKTVYEQFRQWKKQGIFEKMNYEITKYSRRKIGRNEQPSA
                     CIVDSQSVKTTEKGGSKAMMEVKK"
                     /besthit="qcoverage 100 , hcoverage 100 , similarity 100
                     , identity 100 , evalue 1.16e-87 , alnlength 126 ,
                     Bacteria , Proteobacteria , AOV87106.1 transposase
                     [Wolbachia endosymbiont of Drosophila incompta]"
     gene            550..891
                     /locus_tag="wEsol_00105"
     CDS             550..891
                     /locus_tag="wEsol_00105"
                     /codon_start=1
                     /transl_table=11
                     /fullproduct="qcoverage 100 , hcoverage 100 , similarity
                     100 , identity 100 , evalue 1.53e-79 , alnlength 113 ,
                     Bacteria , Proteobacteria , AAS13810.1 transposase, IS5
                     family, OrfB [Wolbachia endosymbiont of Drosophila
                     melanogaster]"
                     /cog="COG3293 COG3293 124 Transposase and inactivated
                     derivatives"
                     /pfam="DDE_Tnp_1_2 DDE_Tnp_1"
                     /product="transposase, IS5 family, OrfB"
                     /translation="MRTKYTKVKKMWADMGYQGRNLKNHIKEEYDIDIEIVKRPPCRF
                     WVHKDTPPELLPTREQGFKVQPRRWVVERTFAWVNRNRRLSKEYDLLTTSTENFIYLA
                     MSRVMLKREYA"
                     /besthit="qcoverage 100 , hcoverage 100 , similarity 100
                     , identity 100 , evalue 1.53e-79 , alnlength 113 ,
                     Bacteria , Proteobacteria , AAS13810.1 transposase, IS5
                     family, OrfB [Wolbachia endosymbiont of Drosophila
                     melanogaster]"
     gene            complement(1133..>1378)
                     /locus_tag="wEsol_00106"
     CDS             complement(1133..>1378)
                     /locus_tag="wEsol_00106"
                     /codon_start=1
                     /transl_table=11
                     /product="hypothetical protein"
                     /translation="YFLRLENVLLSVQGGMSEQAAIDQLSPPLFFKQLQSFKSHLKSL
                     QLSELKKILEKLISLEVTCKKTDLDHKMIFQHALTGS"
                     /cog="HolA COG1466 334 DNA polymerase III, delta subunit"
                     /besthit="qcoverage 100 , hcoverage 24.5454545454545 ,
                     similarity 100 , identity 100 , evalue 2.43e-44 ,
                     alnlength 81 , Bacteria , Proteobacteria , WP_010082540.1
                     MULTISPECIES: DNA polymerase III subunit delta
                     [Wolbachia]"
ORIGIN
        1 aggttgtccg gaaataagta aaaggctata atatctgaaa ttttagtacg gggtaaagat
       61 gagaaaaaag tatccaacag atctaagcga aagggaatgg gcaagaatag aaaaacactt
      121 cagagtatca tacaagaaag gaggaaggcc gccaaagtat agcaaaagag aaatattaga
      181 agcaattttc tatgtattgc gtacagggtg tcaatggcgg tatttaccaa atgattttcc
      241 gctatggaag actgtgtatg agcagttcag gcaatggaag aagcagggaa tttttgagaa
      301 aatgaattat gaaattacaa aatatagtag aagaaaaata ggaaggaatg agcagccgag
      361 tgcctgtata gtagatagtc aatctgtaaa gactacagaa aagggggat caaaggctat
      421 gatggaagta aaaaagtaaa gggtagaaaa aggcatataa ttacagacac tcagggtttt
      481 atactaggtt gttacgtagg cgctgctaac gaaaatgata gagatggtat taaaatagca
      541 ttaaacaata tgagaacaaa atatactaaa gttaaaaaaa tgtgggctga catgggatac
      601 caaggaagaa atttaaagaa tcacataaag gaagaatatg acatagatat tgaaattgtt
      661 aaaaggcctc catgtagatt ttgggtgcac aaagatacgc cacctgagct actaccaaca
      721 agagaacaag ggtttaaagt acagccaaga agatgggttg tagaaaggac ttttgcttgg
      781 gttaatagga atagaaggct atcgaaggag tatgatttac tcacaacatc cactgagaat
      841 ttcatatacc tagctatgag tagggttatg ttaaagaggg aatatgcttg aatttactag
```

```
      901 tttccggaca acctctaatt ttaacatttc aggtgttatt acaattttttg aaatgtcaaa
      961 catatctgta tttttatcta gttatattca attatattcg aaaatatcct attagcaaag
     1021 aatacttgtg agacacacac taaagaatga attttggtag gcataaaagc agctatttta
     1081 tgttaaaata caacattttg atgaaattat aagaaaggct ggatccgagt agtcagctac
     1141 ccgtcagtgc atgttgaaaa atcattttat gatctaaatc agttttttta caggtaactt
     1201 ctaagcttat caatttttcc aagatcttct taagttctga aagttgcaaa cttttcaaat
     1261 gagatttaaa actctgcaat tgtttaaaaa acaatggagg gcttagctga tcaatcgcag
     1321 cctgctcact cattccacct tgcactgaca gcaaaacgtt ttcaaggcgt aggaaata
//
LOCUS       JAQZAU010000011             4022 bp    dna     linear   ENV 17-MAR-2023
DEFINITION  MAG: Wolbachia pipientis isolate wEsol contig_14296, whole genome
            shotgun sequence.
ACCESSION   JAQZAU010000011  JAQZAU010000000
VERSION     JAQZAU010000011.1
DBLINK      BioProject: PRJNA929258
            BioSample: SAMN33142973
KEYWORDS    WGS; ENV; Metagenome Assembled Genome; MAG.
SOURCE      Wolbachia pipientis (insect metagenome)
  ORGANISM  Wolbachia pipientis
            Bacteria; Pseudomonadota; Alphaproteobacteria; Rickettsiales;
            Anaplasmataceae; Wolbachieae; Wolbachia.
REFERENCE   1  (bases 1 to 4022)
  AUTHORS   Fiutek,N., Couger,B.M., Roy,S., de la Torre,J.R. and Connor,E.F.
  TITLE     Genomic assessment of the contribution of the Wolbachia
            endosymbiont of Eurosta solidaginis to gall-induction
  JOURNAL   International Journal Molecular Sciences
REFERENCE   2  (bases 1 to 4022)
  AUTHORS   Fiutek,N., Couger,B.M., Roy,S., de la Torre,J.R. and Connor,E.F.
  TITLE     Direct Submission
  JOURNAL   Submitted (10-FEB-2023) Biology, San Francisco State University,
            1600 Holloway Ave, San Francisco, CA 94132, USA
COMMENT     ##Genome-Assembly-Data-START##
            Assembly Date          :: JUN-2019
            Assembly Method        :: FLYE v. 06-2019
            Genome Representation  :: Full
            Expected Final Version :: Yes
            Genome Coverage        :: 100.0x
            Sequencing Technology  :: Illumina HiSeq; PacBio Sequel
            ##Genome-Assembly-Data-END##
FEATURES             Location/Qualifiers
     source          1..4022
                     /organism="Wolbachia pipientis"
                     /mol_type="genomic DNA"
                     /submitter_seqid="contig_14296"
                     /isolate="wEsol"
                     /isolation_source="galls on Solidago altissima"
                     /host="Eurosta solidaginis MN-2018-EFC"
                     /db_xref="taxon:955"
                     /environmental_sample
                     /country="USA: Cowling Arboretum, Carleton College,
                     Northfield, MN"
                     /lat_lon="44.48 N 93.13 W"
                     /altitude="280 m"
                     /collection_date="2018-11"
                     /metagenome_source="insect metagenome"
                     /note="metagenomic"
     gene            complement(<1..737)
                     /locus_tag="wEsol_00107"
     CDS             complement(<1..737)
                     /locus_tag="wEsol_00107"
```

```
                     /codon_start=1
                     /transl_table=11
                     /fullproduct="qcoverage 100 , hcoverage 83.0508474576271
                     , similarity 99.2 , identity 98.4 , evalue 6.72e-166 ,
                     alnlength 245 , Eukaryota , Arthropoda , GFY80019.1
                     uncharacterized protein TNIN_56691 [Trichonephila
                     inaurata madagascariensis]"
                     /pfam="PDDEXK_2"
                     /tigr="TIGR01784 T_den_put_tspse 273 conserved
                     hypothetical protein"
                     /product="uncharacterized protein"
                     /translation="MALSKFLDPKNDISFKRIFGTEKNKDILIHFLNDILGFTGKSTI
                     QDIEFLSTIQDPDIASKKQSIVDVLCRDENGLQVIVEMQVAKTKGFEKRAQYYAAKAY
                     SRQSDKGDQYHDLKEIIFIAIADCILFPDKSEYKSKHTIRDEDTNEHDLKDFYFIFIE
                     LPKFPKTKEDQLSSIVEKWVYFFKYADETSEEELEKIIGSDLIIKKAYEELNRFNWSE
                     KEFIAYEQEIKRILDEQAVLAQKLDDA"
                     /besthit="qcoverage 100 , hcoverage 83.0508474576271 ,
                     similarity 99.2 , identity 98.4 , evalue 6.72e-166 ,
                     alnlength 245 , Eukaryota , Arthropoda , GFY80019.1
                     uncharacterized protein TNIN_56691 [Trichonephila
                     inaurata madagascariensis]"
     gene            858..1397
                     /locus_tag="wEsol_00108"
     CDS             858..1397
                     /locus_tag="wEsol_00108"
                     /codon_start=1
                     /transl_table=11
                     /pfam="HTH_37 HTH_31 HTH_19 HTH_3 HTH_26"
                     /fullproduct="qcoverage 96.6480446927374 , hcoverage
                     97.1910112359551 , similarity 97.7 , identity 91.9 ,
                     evalue 7.03e-103 , alnlength 173 , Bacteria ,
                     Proteobacteria , WP_146038214.1 MULTISPECIES:
                     helix-turn-helix domain-containing protein, partial
                     [unclassified Wolbachia]"
                     /translation="MANISIRYQIAQKVRNWRLKRGYTQKDLAKKVSVTYQVVLQYEK
                     GTRKISIEKLYAIAEILSIGIVDLIPVSNEKICLEDEGEEILNLVRKYKTINDQELRK
                     VFYLLTKFTRVGEKSSKKAEKVKIAKGMVKAGISVDIVSQAIGLSANECVEEKTGSIY
                     YQIGKKIKEWKRVYSKGFG"
                     /product="helix-turn-helix domain-containing protein,
                     partial"
                     /besthit="qcoverage 96.6480446927374 , hcoverage
                     97.1910112359551 , similarity 97.7 , identity 91.9 ,
                     evalue 7.03e-103 , alnlength 173 , Bacteria ,
                     Proteobacteria , WP_146038214.1 MULTISPECIES:
                     helix-turn-helix domain-containing protein, partial
                     [unclassified Wolbachia]"
     gene            1402..1752
                     /locus_tag="wEsol_00109"
     CDS             1402..1752
                     /locus_tag="wEsol_00109"
                     /codon_start=1
                     /transl_table=11
                     /fullproduct="qcoverage 102.586206896552 , hcoverage
                     90.8396946564885 , similarity 97.5 , identity 95.8 ,
                     evalue 2.04e-66 , alnlength 119 , Viruses , Uroviricota ,
                     QHJ75536.1 putative transcriptional regulator [Wolbachia
                     phage WO]"
                     /cog="HipB COG1396 120 Predicted transcriptional
                     regulators"
                     /pfam="HTH_19 HTH_26 HTH_3"
```

```
                     /product="putative transcriptional regulator"
                     /translation="MDTTRDKISNYEQGRVAIPLEKLYAIAETLSISITDLLIEEDEI
                     VESELPDLIKEYKKIESQELRYALIKSLFESIQICEEKVKRAEKIKDLVKGGISTDII
                     LQITGLSLGEIQQI"
                     /besthit="qcoverage 102.586206896552 , hcoverage
                     90.8396946564885 , similarity 97.5 , identity 95.8 ,
                     evalue 2.04e-66 , alnlength 119 , Viruses , Uroviricota ,
                     QHJ75536.1 putative transcriptional regulator [Wolbachia
                     phage WO]"
     gene            1922..2578
                     /locus_tag="wEsol_00110"
     CDS             1922..2578
                     /locus_tag="wEsol_00110"
                     /codon_start=1
                     /transl_table=11
                     /fullproduct="qcoverage 100 , hcoverage 100 , similarity
                     100 , identity 100 , evalue 1.83e-147 , alnlength 218 ,
                     Bacteria;Viruses , Proteobacteria;Uroviricota ,
                     WP_006280787.1 MULTISPECIES: DNA repair protein RadC
                     [Wolbachia]"
                     /cog="RadC COG2003 224 DNA repair proteins"
                     /pfam="Pfam-B_8535 RadC Prok-JAB"
                     /tigr="TIGR00608 radc 218 DNA repair protein RadC"
                     /product="DNA repair protein RadC"
                     /translation="MNKSKEEIEFRILESKGKALLDREIMETFLSAVHESPQAQEIAK
                     NLVNTYTGVGRILGREMDDLKVIEGVTDSAVAMIMCVKETLERVLKEKLKSEPIMDLQ
                     GIVEYLNVSIGHSERECVKILYLNKRRQLIGEESYIGEMEKAPVYIKEITRKALMKNA
                     TSIIMSHNHPGGSLEPSEEDQAVTKSLAAACSTVSVKLFDHIIITSGGYFSFRENGLL
                     "
                     /besthit="qcoverage 100 , hcoverage 100 , similarity 100
                     , identity 100 , evalue 1.83e-147 , alnlength 218 ,
                     Bacteria;Viruses , Proteobacteria;Uroviricota ,
                     WP_006280787.1 MULTISPECIES: DNA repair protein RadC
                     [Wolbachia]"
     gene            2679..3863
                     /locus_tag="wEsol_00111"
     CDS             2679..3863
                     /locus_tag="wEsol_00111"
                     /codon_start=1
                     /transl_table=11
                     /pfam="Pfam-B_5362"
                     /fullproduct="qcoverage 95.6852791878173 , hcoverage 100
                     , similarity 99.7 , identity 99.7 , evalue 5.72e-251 ,
                     alnlength 377 , Bacteria , Proteobacteria ,
                     WP_007549714.1 MULTISPECIES: hypothetical protein
                     [Wolbachia]"
                     /translation="MTSKDRNKKTNVNHKKIFGALEGVDIKQSKFVSKINNQKEINRE
                     ASPSRNIRENKINYGKSLDYVYGTKPLNDQAEDLNPFASNLQKIKPSASESEKLSWFK
                     SAVVETKNVNALHKVIEQVLASGARVNACNDGEWSFAEYMILGTHFHRLEKSDRKKIM
                     RKLMLSGAEFHDTLLQNKLIGEIYNELQPEVQPQIDERLEELEKAGESAVQEGELIDV
                     EIDNTTSYIEFSGNSKVEVAKILRELGSNILKMGNNAVEIRREEGGARNYVDVSDGSS
                     VVLELPTSLGKLNIVLYQDVKNCSQVQVKVADKEMWAELQKRGEEIGKNCLFGGVKLK
                     KVVERGNFTRCGIWSEKQKEVSEKLFSWADKVRENSKETCRALLFINLKVPESGYYIW
                     SL"
                     /product="hypothetical protein"
                     /besthit="qcoverage 95.6852791878173 , hcoverage 100 ,
                     similarity 99.7 , identity 99.7 , evalue 5.72e-251 ,
                     alnlength 377 , Bacteria , Proteobacteria ,
                     WP_007549714.1 MULTISPECIES: hypothetical protein
                     [Wolbachia]"
```

```
     gene            3888..>4022
                     /locus_tag="wEsol_00112"
     CDS             3888..>4022
                     /locus_tag="wEsol_00112"
                     /codon_start=1
                     /transl_table=11
                     /product="hypothetical protein"
                     /pfam="Pfam-B_12671 HTH_31 HTH_19 HTH_3"
                     /translation="MVLFVEKSLDYKVGEKLKSWRLERGYTQKDLAEKLGVKYWVILQ
                     Y"
                     /besthit="qcoverage 100 , hcoverage 37.8151260504202 ,
                     similarity 100 , identity 100 , evalue 6.27e-23 ,
                     alnlength 45 , Bacteria , Proteobacteria , WP_007549715.1
                     helix-turn-helix domain-containing protein, partial
                     [Wolbachia endosymbiont of Drosophila ananassae]"
ORIGIN
        1 gtagcatcat caagtttttg agcaaggaca gcctgttcat caagaatacg ttttatttcc
       61 tgttcatagg caataaattc tttttctgac cagttgaacc tatttagttc ttcataggcc
      121 tttttaatta ttagatcact acctattatt ttttccagct cttcttcact agtctcatct
      181 gcatatttaa agaagtagac ccatttctca acaatacttg aaagctgatc ttctttggtt
      241 tttggaaatt taggcaactc aataaatata aagtaaaaat cttttagatc atgctcatta
      301 gtatcttcat cgcgaatagt gtgctttgat ttgtactcag acttatcagg aaataaaata
      361 cagtctgcta tagcaataaa gataatttcc ttaaggtcat ggtattgatc acctttatca
      421 gactgtcttg agtaagcttt agcagcatag tattgtgcac gtttttcaaa gcccttagtt
      481 ttagcgacct gcatttcaac tatgacttgc agcccatttt catctctaca aagaacatca
      541 acaatgcttt gtttttttaga agcaatatca gggtcttgaa tagtacttaa gaactctata
      601 tcctgtattg tacttttgcc agtgaagcca aggatatcat taaggaagtg aataagaatg
      661 tccttatttt tttcagtacc aaagatgcgc ttgaacgata tatcattctt gggatcgaga
      721 aacttcgaaa gagccatagg aaatccactt aaaaagcatt aataattata cataattgtg
      781 aagaaatatt caactaaaat gcaaacatag aagtttttgt atatctttgt ttggtagtat
      841 taacatgtat aatatttatg gcaaacatct caataaggta tcagatagca caaaaagtaa
      901 ggaactggag gttaaagcga ggatatactc aaaaagattt agcgaagaaa gtcagtgtaa
      961 cgtatcaagt agtactacaa tatgaaaaag gaacacgtaa aatttcgatt gaaaagttgt
     1021 atgctatagc agagatattg tcgattggta ttgtagacct aattcctgta tcaaatgaaa
     1081 aaatctgtct tgaagatgag ggagaagaaa tattaaatct agtaagaaaa tataaaacga
     1141 ttaatgatca agagttgcgc aaggtgtttt acttgctaac aaaatttacc cgagttggtg
     1201 agaaaagtag taaaaaagca gagaaggtaa aaattgcaaa gggtatggtt aaagcaggaa
     1261 tttctgttga tattgtttca caagcaattg gcctctctgc taatgagtgt gttgaagaaa
     1321 aaacaggttc tatctactac caaataggaa aaaagataaa agaatggaag agagtatact
     1381 caaaaggatt tggctgagaa aatggataca acacgtgata aaataagcaa ctatgagcaa
     1441 ggacgtgtgg ccattccact ggaaaaatta tatgcaatag cagaaacatt atcaattagc
     1501 attacagatc tgctcataga ggaagatgag atagtagaaa gtgagctacc tgatttaata
     1561 aaagaataca aaaaaattga gagtcaagaa ttacgctatg cgctaataaa atctctgttt
     1621 gaaagcatac aaatttgcga ggaaaaagtg aaaagagcag aaaagataaa ggatttagtg
     1681 aaaggaggaa tttctaccga tattattttg caaataacag gcctctcttt aggcgaaatt
     1741 caacagattt aaaataaaaa atcagtatat attaatatct ttactcttag atgaaaaaat
     1801 aagtaaaaaa gattgagaaa ttgatttgac ttcactcgaa agctatggct ttatgccaat
     1861 gctgataaaa aacagcagta aatatctaaa aaaagtttgt tgttagtgaa aggtaaatta
     1921 tatgaataaa agtaaagaag aaatcgaatt cagaatatta gaaagcaaag gcaaagcact
     1981 acttgatcgt gaaataatgg aaacgtttct aagtgcagta catgaaagcc cacaggctca
     2041 agaaattgct aaaaatttgg tgaatactta tacaggagta ggaaggattt taggtagaga
     2101 aatggatgac ctgaaagtta tagaaggagt aactgattct gcagtagcaa tgattatgtg
     2161 tgttaaggaa acactagaaa gggtactgaa agaaaagctt aagagtgagc caatcatgga
     2221 cttacaaggg atagtagagt acttaaacgt aagtataggc cactcagaaa gggaatgtgt
     2281 aaaaatactg tacttgaata aaaggcgtca actaattgga gaagaatcct atattggtga
     2341 aatggaaaaa gcaccagtat acataaagga aattacaaga aaggcattaa tgaaaaatgc
     2401 aacatcaata ataatgtcac ataaccatcc tggagggagc ttagagccat cagaagaaga
     2461 tcaagcagta acgaagagct tagcagcagc atgtagtact gtaagcgtaa aattgtttga
     2521 ccatattatc atcacaagtg gaggctattt cagctttcga gaaaatggat tgttatagta
     2581 gaaagtattt gcaaatctac tcattataat ttataatgag tagtaagatg tataaagttt
```

```
     2641 attgtacgct taaaaagtat ttttaatatg aggtttatat gactagtaaa gatcggaata
     2701 aaaagactaa tgttaatcac aagaaaatat ttggtgctct tgaaggcgtt gatataaagc
     2761 agtctaaatt tgtgagtaaa attaataacc agaaggagat aaaccgggag gcctccccta
     2821 gcagaaacat tcgcgaaaac aaaatcaact acggaaaaag ccttgactat gtttatggca
     2881 caaaaccttt aaatgatcaa gcagaagatt taaatccatt tgcttcaaat ttgcaaaaaa
     2941 taaaaccaag tgcaagcgaa agtgaaaagt tgagctggtt taagagtgct gtagtagaga
     3001 caaaaaaacgt aaatgcattg cataaagtta tagagcaagt attagcctct ggagcaagag
     3061 taaatgcatg taatgatggg gagtggagct tcgcagaata catgatattg ggcacacact
     3121 ttcacagatt agaaaaaagt gatcgaaaaa agataatgcg taagctaatg ctaagtggtg
     3181 cagagtttca tgatactcta ctacagaata aactgatagg tgaaatctat aatgagctac
     3241 aaccagaagt tcagccacag atagatgaga gactagaaga actagagaaa gctggcgaaa
     3301 gtgctgtgca agaaggagaa ttaatagatg ttgagataga taatacaaca tcgtacatag
     3361 agttttctgg gaatagcaag gtagaggtag ccaaaatact gagagagtta gggagtaaca
     3421 ttttaaaaat gggtaataat gcagttgaga tcagaaggga ggaaggaggt gcaagaaatt
     3481 acgttgatgt atcagatggt agctccgttg tgttagaatt gcctacaagc cttggtaagc
     3541 taaatattgt attgtaccag gacgtaaaaa actgcagtca ggtacaagta aaagtagcgg
     3601 ataaggaaat gtgggctgaa ttacaaaaaa gaggagaaga aattggaaaa aattgccttt
     3661 ttgggggagt gaaacttaag aaagtggtag aaagaggtaa tttcactagg tgcggcatat
     3721 ggagtgaaaa gcagaaggaa gtaagtgaaa aattgttttc atgggcagac aaagtacgtg
     3781 aaaatagtaa agaaacttgt agggcacttt tattcatcaa tttaaaggtt ccagagtctg
     3841 gctattatat ctggagcctt tgagctatag tataaaatat tatatgtatg gttctttttg
     3901 tggaaaaaag tctagattat aaagtgggag aaaaattaaa aagttggagg ttagagagag
     3961 ggtatactca gaaagattta gcagagaaac ttggtgtaaa gtactgggta atacttcaat
     4021 at
//
LOCUS       JAQZAU010000012          11379 bp    dna     linear   ENV 17-MAR-2023
DEFINITION  MAG: Wolbachia pipientis isolate wEsol contig_14687, whole genome
            shotgun sequence.
ACCESSION   JAQZAU010000012  JAQZAU010000000
VERSION     JAQZAU010000012.1
DBLINK      BioProject: PRJNA929258
            BioSample: SAMN33142973
KEYWORDS    WGS; ENV; Metagenome Assembled Genome; MAG.
SOURCE      Wolbachia pipientis (insect metagenome)
  ORGANISM  Wolbachia pipientis
            Bacteria; Pseudomonadota; Alphaproteobacteria; Rickettsiales;
            Anaplasmataceae; Wolbachieae; Wolbachia.
REFERENCE   1  (bases 1 to 11379)
  AUTHORS   Fiutek,N., Couger,B.M., Roy,S., de la Torre,J.R. and Connor,E.F.
  TITLE     Genomic assessment of the contribution of the Wolbachia
            endosymbiont of Eurosta solidaginis to gall-induction
  JOURNAL   International Journal Molecular Sciences
REFERENCE   2  (bases 1 to 11379)
  AUTHORS   Fiutek,N., Couger,B.M., Roy,S., de la Torre,J.R. and Connor,E.F.
  TITLE     Direct Submission
  JOURNAL   Submitted (10-FEB-2023) Biology, San Francisco State University,
            1600 Holloway Ave, San Francisco, CA 94132, USA
COMMENT     ##Genome-Assembly-Data-START##
            Assembly Date          :: JUN-2019
            Assembly Method        :: FLYE v. 06-2019
            Genome Representation  :: Full
            Expected Final Version :: Yes
            Genome Coverage        :: 100.0x
            Sequencing Technology  :: Illumina HiSeq; PacBio Sequel
            ##Genome-Assembly-Data-END##
FEATURES             Location/Qualifiers
     source          1..11379
                     /organism="Wolbachia pipientis"
                     /mol_type="genomic DNA"
                     /submitter_seqid="contig_14687"
```

```
                     /isolate="wEsol"
                     /isolation_source="galls on Solidago altissima"
                     /host="Eurosta solidaginis MN-2018-EFC"
                     /db_xref="taxon:955"
                     /environmental_sample
                     /country="USA: Cowling Arboretum, Carleton College,
                     Northfield, MN"
                     /lat_lon="44.48 N 93.13 W"
                     /altitude="280 m"
                     /collection_date="2018-11"
                     /metagenome_source="insect metagenome"
                     /note="metagenomic"
     gene            438..1910
                     /locus_tag="wEsol_00113"
     CDS             438..1910
                     /locus_tag="wEsol_00113"
                     /codon_start=1
                     /transl_table=11
                     /fullproduct="qcoverage 100 , hcoverage 100 , similarity
                     99.6 , identity 99.6 , evalue 0.0 , alnlength 490 ,
                     Bacteria , Proteobacteria , WP_149168725.1 hypothetical
                     protein [Wolbachia endosymbiont of Chrysomya
                     megacephala]"
                     /translation="MPKSKTKRGTEDLKGNAGPSKRSRLSSDPKKNKEIISSKVISKL
                     KDVVKGDRTSAIEEWVKSHPVTVEGLTVEQSDLLCNAFRDESCWSGATLDVAKLVGEL
                     AKSGVLNPFAIYKIACIECVESEIKQLFDKALESFRSDLSHKGACEEDRNLACSDKLA
                     RVELLSFMGRRDPVFNFWIDQESGNLRENIEAEDGFNKAVDLKWSKGVEHFYNRLCSE
                     EKLVKEEREKLLVSAIAKLSPLQSSYKLASTLNSLLGKVISAKVDHKSLLGLPNKKDR
                     GVIYRPLSYLVEHGFLCTTKYVIQYLSEGCSRSEVEKMLSPRGYAHLLSSLSFVVVSK
                     DYDLDNRNEARSAISSLWESSVFNQNKINVVDPFKDRIAFVAMENAISNLIVDQENSK
                     DTQSAGDGEKVDLVLSILKFAKDCCSDKSFKSLKERIANSLDKTRNSKMIDATSSCNL
                     IEELCKSARNLNLFSASTEGPQSTLVGTNVSISPAAVVNK"
                     /product="hypothetical protein"
                     /besthit="qcoverage 100 , hcoverage 100 , similarity 99.6
                     , identity 99.6 , evalue 0.0 , alnlength 490 , Bacteria ,
                     Proteobacteria , WP_149168725.1 hypothetical protein
                     [Wolbachia endosymbiont of Chrysomya megacephala]"
     gene            1919..4009
                     /locus_tag="wEsol_00114"
     CDS             1919..4009
                     /locus_tag="wEsol_00114"
                     /codon_start=1
                     /transl_table=11
                     /pfam="DUF1703 DUF1703"
                     /fullproduct="qcoverage 100 , hcoverage 100 , similarity
                     99.9 , identity 99.9 , evalue 0.0 , alnlength 696 ,
                     Bacteria , Proteobacteria , WP_006014164.1 MULTISPECIES:
                     hypothetical protein [Wolbachia]"
                     /translation="MHGNNEDRELVRALLSGGCDEFSRQFVGFLNNCPSFLHSANKPG
                     FFPTFFFGMFSTAHDAGILVEDERVYFRFDNYGNLKVAVLTNKENRRIVRCYTVADNE
                     NSPGSRFSAEEKQQVEENLPQELQEDEDLDWEEYKIFRFGEECRFIHEIDRFPQRDEP
                     GAPIFHEINPIREQGELLDLMSELANDDTGEVSTNVKRILEYVIDIHDEHEDSLVFRA
                     ESDYHGFLCGFLVNFRYRAVADFYPELLIGKGYADVVLLVRGVDQTNDSVPIIIELKV
                     GDEEGLEQAKDYAKSCSVSSLPIHTSSPSAVCVALNFQLRGGAGLRTSVQAFSEGGLS
                     LIPGLLHPHGNGVRGNVKRFLQPIASEFTQSPHCNTFSCTSSFVFGNVLSTRRDLETN
                     DGREVRVTKYLFNHSQGEKMKRTGGRGDAADIVSHALTLALFLSNIGFFVLHIFRRLK
                     WQTLPDKALNLSLLPQATDDAKVRQVLCEVDVQGHLEVASAKKFESLRAYSRSHSEGY
                     FEGRFSEQMGNVRNLHQLADQLMSAEPNFGNDSNVNGEYRARYEVLFNEISRLLSPLL
                     NGNRLLVNNEAKFQALLRGIFQSCDNPAKVIIEFQLQRGRKIDLVLSKSAENDDTHPI
                     GIELKYANTAEQVERKRVEANRQLSEYEFCGGCKRITGGDAMVLLYAILNAVGQEQDL
```

```
                     ILIGGLRRASGFSR"
                     /product="hypothetical protein"
                     /besthit="qcoverage 100 , hcoverage 100 , similarity 99.9
                     , identity 99.9 , evalue 0.0 , alnlength 696 , Bacteria ,
                     Proteobacteria , WP_006014164.1 MULTISPECIES:
                     hypothetical protein [Wolbachia]"
     gene            complement(4677..5024)
                     /locus_tag="wEsol_00115"
     CDS             complement(4677..5024)
                     /locus_tag="wEsol_00115"
                     /codon_start=1
                     /transl_table=11
                     /product="hypothetical protein"
                     /pfam="rve rve_3 rve_2"
                     /translation="MLEFCLYTLKEALKQGKPEIFNSDQGVQFTSYNFIMELERANIK
                     ISMDHKGRCFDNIFVERLWRTLKQEAIYYYRPNSIRDLNLIINDFVAWYNYRRRHQTL
                     HYKVPADLYYHKQ"
                     /cog="Tra5 COG2801 232 Transposase and inactivated
                     derivatives"
                     /besthit="qcoverage 97.3913043478261 , hcoverage
                     59.5744680851064 , similarity 100 , identity 99.1 ,
                     evalue 5.82e-78 , alnlength 112 , Bacteria ,
                     Proteobacteria , KLT21577.1 integrase, catalytic region
                     [Wolbachia endosymbiont of Armadillidium vulgare str.
                     wVulC]"
     gene            complement(4975..5373)
                     /locus_tag="wEsol_00116"
     CDS             complement(4975..5373)
                     /locus_tag="wEsol_00116"
                     /codon_start=1
                     /transl_table=11
                     /product="hypothetical protein"
                     /pfam="HTH_21 rve DDE_Tnp_IS240"
                     /translation="MIQEVYLASDCRYGYRKITAEIIASGVVVNHKKILRIMKKMKIS
                     GLYCRKRCNTSIKEKKHKIYPYLLKDLIICRVNQVWATDITYIMVEGKFVYFVAIMDL
                     YSRYIIAHSLSPYLDAGVLPLYSQRSSKTR"
                     /cog="Tra5 COG2801 232 Transposase and inactivated
                     derivatives"
                     /besthit="qcoverage 90.1515151515152 , hcoverage
                     51.5151515151515 , similarity 100 , identity 100 , evalue
                     6.88e-77 , alnlength 119 , Bacteria , Proteobacteria ,
                     KLT22412.1 integrase, catalytic region [Wolbachia
                     endosymbiont of Armadillidium vulgare str. wVulC]"
     gene            complement(5501..5785)
                     /locus_tag="wEsol_00117"
     CDS             complement(5501..5785)
                     /locus_tag="wEsol_00117"
                     /codon_start=1
                     /transl_table=11
                     /fullproduct="qcoverage 100 , hcoverage 100 , similarity
                     100 , identity 98.9 , evalue 4.02e-55 , alnlength 94 ,
                     Bacteria , Proteobacteria , OAM05084.1 hypothetical
                     protein TV41_03535 [Wolbachia endosymbiont of Dactylopius
                     coccus]"
                     /cog="COG2963 COG2963 116 Transposase and inactivated
                     derivatives"
                     /pfam="HTH_Tnp_1 HTH_28"
                     /product="hypothetical protein"
                     /translation="MATKKYEPELKAKIALEAIKNQRSTAEICSEYKIPSTNLYDWRD
                     RVLARLKDLFVEESESARKQRILAQEIESLHKVIGELTVENSYLKKKLLK"
```

```
                     /besthit="qcoverage 100 , hcoverage 100 , similarity 100
                     , identity 98.9 , evalue 4.02e-55 , alnlength 94 ,
                     Bacteria , Proteobacteria , OAM05084.1 hypothetical
                     protein TV41_03535 [Wolbachia endosymbiont of Dactylopius
                     coccus]"
     gene            complement(6085..6216)
                     /locus_tag="wEsol_00118"
     CDS             complement(6085..6216)
                     /locus_tag="wEsol_00118"
                     /codon_start=1
                     /transl_table=11
                     /pfam="Ank_2 Ank_4"
                     /fullproduct="qcoverage 100 , hcoverage 100 , similarity
                     100 , identity 100 , evalue 6.35e-23 , alnlength 43 ,
                     Bacteria , Proteobacteria , EAL57869.1 hypothetical
                     protein WwAna0747 [Wolbachia endosymbiont of Drosophila
                     ananassae]"
                     /translation="MAVHHPAKFDSLEVTKFLVNNKGVSFNAKTNDENKPIHIAADF"
                     /product="hypothetical protein"
                     /besthit="qcoverage 100 , hcoverage 100 , similarity 100
                     , identity 100 , evalue 6.35e-23 , alnlength 43 ,
                     Bacteria , Proteobacteria , EAL57869.1 hypothetical
                     protein WwAna0747 [Wolbachia endosymbiont of Drosophila
                     ananassae]"
     gene            complement(6513..6887)
                     /locus_tag="wEsol_00119"
     CDS             complement(6513..6887)
                     /locus_tag="wEsol_00119"
                     /codon_start=1
                     /transl_table=11
                     /pfam="HTH_31 HTH_19 Pfam-B_8788 HTH_3"
                     /fullproduct="qcoverage 100 , hcoverage 100 , similarity
                     100 , identity 100 , evalue 3.61e-81 , alnlength 124 ,
                     Bacteria;Eukaryota , Proteobacteria;Arthropoda ,
                     WP_007549999.1 MULTISPECIES: helix-turn-helix
                     domain-containing protein [Wolbachia]"
                     /translation="MTIQHPIDKQIGERIRKRRLMCGFSQRDLGKKLEISFQHVQGYE
                     SGEIRLVVDRLYKLAEVLSVDMSYFFTKASEDLHDKAFHSDVGSEEISRLVREYRKIE
                     DETLRDIVHLVIKALANTGFKA"
                     /product="helix-turn-helix domain-containing protein"
                     /besthit="qcoverage 100 , hcoverage 100 , similarity 100
                     , identity 100 , evalue 3.61e-81 , alnlength 124 ,
                     Bacteria;Eukaryota , Proteobacteria;Arthropoda ,
                     WP_007549999.1 MULTISPECIES: helix-turn-helix
                     domain-containing protein [Wolbachia]"
     gene            complement(6910..7239)
                     /locus_tag="wEsol_00120"
     CDS             complement(6910..7239)
                     /locus_tag="wEsol_00120"
                     /codon_start=1
                     /transl_table=11
                     /pfam="HTH_31 HTH_19 HTH_26 HTH_3"
                     /fullproduct="qcoverage 100 , hcoverage 100 , similarity
                     100 , identity 100 , evalue 4.50e-68 , alnlength 109 ,
                     Bacteria , Proteobacteria , WP_007549995.1 MULTISPECIES:
                     helix-turn-helix domain-containing protein [Wolbachia]"
                     /translation="MQLGKKIKQLRLDRALTQTELGKRIGVSYRQIQKYENGSNCVLA
                     SRLYDLAKALSIDVANFFTDMHTDSHEAYDEEILKLVKGYNEIKSKRLRSAVYILVKS
                     FSQSDNT"
                     /product="helix-turn-helix domain-containing protein"
```

```
                     /besthit="qcoverage 100 , hcoverage 100 , similarity 100
                     , identity 100 , evalue 4.50e-68 , alnlength 109 ,
                     Bacteria , Proteobacteria , WP_007549995.1 MULTISPECIES:
                     helix-turn-helix domain-containing protein [Wolbachia]"
     gene            complement(7291..8223)
                     /locus_tag="wEsol_00121"
     CDS             complement(7291..8223)
                     /locus_tag="wEsol_00121"
                     /codon_start=1
                     /transl_table=11
                     /fullproduct="qcoverage 100 , hcoverage 100.977198697068
                     , similarity 97.4 , identity 96.1 , evalue 2.37e-195 ,
                     alnlength 310 , Bacteria , Proteobacteria ,
                     WP_012673225.1 MULTISPECIES: hypothetical protein
                     [Wolbachia]"
                     /translation="MDILSYSTEKLKKHCQLLDDEEKIVLYEQLLDKAKDILENSRDD
                     IAKLKEVSKAVVAIEETTDKQLLEKFNDDHPLREVDILIYSPQGNTEVANYLFSIDNS
                     SELYDLKKDKEKALYNAVKSNDVELVKKLLMILLPTEVGDFDVEYLEELKILLSGIHK
                     ELQLSQDMKNYLEKTIKFYSFLCSNFNLLVANPTDVKAMIDLFAAQPNIDYQIDKLLL
                     SFIVRDVEEKKLNSEISHMIELLEQHERFAELEYKVRRLRSEFASGKSRYSAEVIRNS
                     IAEREKEMREIEKKYVRPNDLISERQKLLKQLLC"
                     /product="hypothetical protein"
                     /besthit="qcoverage 100 , hcoverage 100.977198697068 ,
                     similarity 97.4 , identity 96.1 , evalue 2.37e-195 ,
                     alnlength 310 , Bacteria , Proteobacteria ,
                     WP_012673225.1 MULTISPECIES: hypothetical protein
                     [Wolbachia]"
     gene            8422..9504
                     /locus_tag="wEsol_00122"
     CDS             8422..9504
                     /locus_tag="wEsol_00122"
                     /codon_start=1
                     /transl_table=11
                     /pfam="Pfam-B_5362"
                     /fullproduct="qcoverage 100 , hcoverage 100 , similarity
                     99.7 , identity 99.7 , evalue 1.23e-248 , alnlength 360 ,
                     Bacteria , Proteobacteria , WP_012673224.1 MULTISPECIES:
                     hypothetical protein [Wolbachia]"
                     /translation="MKKINGKKLSSKMLKAVERIAKEGLLPNFIKAAPTANKKISKLP
                     VLTYPEELNPFRSALCKIRLSEYENVKLNEFLEKAIAAENLDKLSKIVDEALDLGIRV
                     NARNEDGFSFTNVTMFKMYRDEFKENKQEDIIRKLALHGADFNVQVDKKIAEMYQKVQ
                     KEIEPQIYSRLKKLREVGEDATIEGTVEDVEIDNKTFYIEFSHSCIVDVAKVVEGAKN
                     LGLSKGDLNLGGSIIKIGDGEVEVKTGKDGKRNYVDISDNSAFTVTLCTSLGELNLMV
                     YHDTEDYHQVQVEVEDEEMWNELQEIGEIVGKGCLFGGMSVKEVVEKGSFTRCGRWGD
                     QNSKSSETFSWGSKLKDTKTTSVGRS"
                     /product="hypothetical protein"
                     /besthit="qcoverage 100 , hcoverage 100 , similarity 99.7
                     , identity 99.7 , evalue 1.23e-248 , alnlength 360 ,
                     Bacteria , Proteobacteria , WP_012673224.1 MULTISPECIES:
                     hypothetical protein [Wolbachia]"
     gene            complement(9641..10012)
                     /locus_tag="wEsol_00123"
     CDS             complement(9641..10012)
                     /locus_tag="wEsol_00123"
                     /codon_start=1
                     /transl_table=11
                     /pfam="HTH_31 Pfam-B_17975 HTH_19 HTH_3 HTH_26"
                     /fullproduct="qcoverage 100 , hcoverage 100 , similarity
                     100 , identity 100 , evalue 1.60e-79 , alnlength 123 ,
                     Bacteria , Proteobacteria , WP_141457079.1
```

helix-turn-helix domain-containing protein [Wolbachia endosymbiont of Carposina sasakii]"
/translation="MIVQDEQHPIDKQIGERVRKRRLICGFSQRDLGKKLGISFQHIQ
GYETGEVRLVIDRLYNLAEALSVDMSYFFTKASEDLHDKAFCSDVGSEEISRLVREYR
KIKDETLRDIVHSVIKALANK"
/product="helix-turn-helix domain-containing protein"
/besthit="qcoverage 100 , hcoverage 100 , similarity 100
, identity 100 , evalue 1.60e-79 , alnlength 123 ,
Bacteria , Proteobacteria , WP_141457079.1
helix-turn-helix domain-containing protein [Wolbachia
endosymbiont of Carposina sasakii]"

gene complement(10047..10379)
/locus_tag="wEsol_00124"

CDS complement(10047..10379)
/locus_tag="wEsol_00124"
/codon_start=1
/transl_table=11
/fullproduct="qcoverage 100 , hcoverage 100 , similarity
100 , identity 100 , evalue 1.02e-69 , alnlength 110 ,
Bacteria , Proteobacteria , WP_041582615.1 MULTISPECIES:
helix-turn-helix domain-containing protein [Wolbachia]"
/cog="HipB COG1396 120 Predicted transcriptional
regulators"
/pfam="HTH_31 HTH_19 Pfam-B_9358 HTH_3 HTH_23 HTH_26"
/product="helix-turn-helix domain-containing protein"
/translation="MELGKKIKELRLYCGLTQTELGKKIGVSYRQIQRYENSSNQILA
SRLYDLAKALSINVADFFTDVHVDSHETYDEEILKLVKGYNEIKSKRLRSVVYILVKS
FSQSYNES"
/besthit="qcoverage 100 , hcoverage 100 , similarity 100
, identity 100 , evalue 1.02e-69 , alnlength 110 ,
Bacteria , Proteobacteria , WP_041582615.1 MULTISPECIES:
helix-turn-helix domain-containing protein [Wolbachia]"

gene complement(10483..11142)
/locus_tag="wEsol_00125"

CDS complement(10483..11142)
/locus_tag="wEsol_00125"
/codon_start=1
/transl_table=11
/fullproduct="qcoverage 100 , hcoverage 100 , similarity
100 , identity 100 , evalue 1.45e-149 , alnlength 219 ,
Bacteria , Proteobacteria , WP_012673223.1 MULTISPECIES:
DNA repair protein RadC [Wolbachia]"
/cog="RadC COG2003 224 DNA repair proteins"
/pfam="RadC"
/tigr="TIGR00608 radc 218 DNA repair protein RadC"
/product="DNA repair protein RadC"
/translation="MNNSERKEKLETRILSSRGRSLLDHELLERTLSECEEGREVAKK
LIELFSSLGRVINADFHELKNVTGMNDGAIASIFCLKEIFDRILKGKLKKLSILDNRE
ELLKYFKTAIGQSRKESLRVIYLDRSGHLIYEYIQDCGTIDRVPLYVREIIKQGLLID
ASSIAISHNHPSGDTEPSKEDEDNTLTLAATCENVGMKLIDHIIVTQKSHFSFYDSGL
L"
/besthit="qcoverage 100 , hcoverage 100 , similarity 100
, identity 100 , evalue 1.45e-149 , alnlength 219 ,
Bacteria , Proteobacteria , WP_012673223.1 MULTISPECIES:
DNA repair protein RadC [Wolbachia]"

gene complement(11227..11349)
/locus_tag="wEsol_00126"

CDS complement(11227..11349)
/locus_tag="wEsol_00126"
/codon_start=1

```
                     /transl_table=11
                     /fullproduct="qcoverage 100 , hcoverage 100 , similarity
                     100 , identity 100 , evalue 8.18e-18 , alnlength 40 ,
                     Bacteria , Proteobacteria , ONI56244.1 hypothetical
                     protein N500_0678 [Wolbachia pipientis wUni]"
                     /translation="MIANINTVALQGISTVNVNAQIHMANGIPAFKATRTTLQI"
                     /product="hypothetical protein"
                     /besthit="qcoverage 100 , hcoverage 100 , similarity 100
                     , identity 100 , evalue 8.18e-18 , alnlength 40 ,
                     Bacteria , Proteobacteria , ONI56244.1 hypothetical
                     protein N500_0678 [Wolbachia pipientis wUni]"
ORIGIN
        1 aacaaataag ctctcaatct gtaatccata aagcttctga tttaatagat acgatgtact
       61 catataattt tactacaaaa ctaataatat aaaagttttc tgatgtacgt ccccagaatg
      121 tgatcgtaag ggttcattat aaataattcg gtaaatttcg tctctttaga catattaaga
      181 gctatgtaac gaacgtttat atttgtgagt aatagtaagg ctcaaggtaa ggaaaatgct
      241 agtgaggtaa agtagaataa cgttaatttg ataatttaag ctcattccct cttgctacat
      301 ctctctttaa tatctcctct tttgcagcag gtctataatt actgcttaaa tttaggtgca
      361 cactaatttt tcgatttata tattattcta atgcttgtat aataccttca ttgaatattt
      421 gattttgagg taattagatg ccaaaaagta aaactaaacg tggaacggaa gatttgaagg
      481 gtaatgcagg cccaagcaaa agatctcgtc tcagttctga tcctaaaaaa aataaagaga
      541 ttatctctag caaagtaata agtaagctga aggatgttgt taaaggtgat agaacttcag
      601 ctattgagga atgggtcaag tctcaccctg taacagtaga gggtctaacc gttgagcaat
      661 cggacctctt atgtaatgcg tttcgtgatg aatcttgttg gtcaggtgcg acactagatg
      721 ttgctaaatt ggtaggagaa ttagctaaat caggtgtgtt gaatccattt gctatatata
      781 aaatagcatg tattgagtgt gtagagagtg aaattaagca attatttgac aaggcgttag
      841 agtcttttag atctgactta tctcataaag gtgcatgtga ggaagatagg aatttagctt
      901 gcagtgataa gcttgcaaga gttgaattgt taagtttcat gggaagacgt gatcctgttt
      961 tcaatttctg gattgatcaa gaatcaggta accttagaga aaatatagaa gcagaagatg
     1021 gatttaataa ggctgtagat ttgaagtgga gtaagggagt ggaacacttc tataatcgtc
     1081 tgtgttctga agaaaaatta gtgaaagaag agagagaaaa attgctagtt tctgctattg
     1141 caaaattatc tccattgcaa tctagctata aacttgcttc taccttaaat tcccttctag
     1201 gtaaagtcat aagcgcaaaa gtagatcata agtcactact tgggctaccg aataagaaag
     1261 atagggggtgt gatctatcgt cctcttagtt acttagtaga gcacggtttt ctttgcacaa
     1321 ctaagtatgt tatccagtac ttgagcgagg gatgttcaag atctgaagta gagaaaatgc
     1381 tttcacctag aggatatgca catcttctct catcgctttc atttgttgta gtttctaaag
     1441 attatgactt ggataacagg aatgaagcaa ggtcagctat tagcagtctt tgggaatcta
     1501 gtgtatttaa ccaaaataaa ataaatgttg tcgatccttt taaagatagg attgcttttg
     1561 ttgcaatgga aaatgcaatt tcaaatttga ttgtagatca ggagaacagc aaggatactc
     1621 aaagtgctgg cgatggtgaa aaagttgatt tggtcttgag tattttaaag tttgctaaag
     1681 attgttgttc agacaaaagc tttaaatcat taaaagagag gatagcaaat agtttagata
     1741 aaacaaggaa ttctaagatg atagatgcaa ctagctcctg caatttaata gaagagttgt
     1801 gtaagtcagc gagaaatttg aatttattct ctgctagcac tgaaggtcct caatctacgt
     1861 tagtgggtac taatgttagt atttcgcctg ctgcagttgt taacaaatag gagtatttat
     1921 gcatggtaat aatgaagatc gtgaattagt tagggcttta ttaagtggag gttgtgatga
     1981 gtttagtaga caatttgtag gtttttttaaa caactgtcca tcttttttgc attcggctaa
     2041 taagcctggc tttttttccta cattcttttt tggtatgttt tctactgcac atgatgcagg
     2101 tatattagtt gaagatgaaa gagtctattt tcgttttgac aattatggaa atctaaaagt
     2161 tgctgttctc actaataaag aaaatagaag aatagtcagg tgttatactg ttgctgataa
     2221 tgagaacagc cctgggtcaa ggtttagtgc agaagagaag cagcaggtag aagagaatct
     2281 tccacaagaa ttacaggaag atgaggatct ggattgggaa gagtataaaa tatttcggtt
     2341 tggagaagaa tgtaggttta ttcatgaaat agatagattt cctcaacgtg atgaacctgg
     2401 agctccaatt tttcatgaaa ttaacccaat cagagaacaa ggtgaattgt tagacctgat
     2461 gagtgagttg gcaaatgacg atacaggaga agtgagtact aatgttaaaa gaattttgga
     2521 atatgttatt gatatccatg atgaacatga agatagctta gtgtttcgtg cagagtctga
     2581 ttaccacggt tttctgtgtg ggtttttagt aaattttaga taccgagctg tggctgattt
     2641 ctacccagag ctacttatag gaaaaggtta tgcagatgtt gttttgcttg ttcgtggtgt
     2701 tgatcagaca aatgattcgg ttccaattat aattgagttg aaggttggtg atgaggaagg
     2761 attagagcaa gctaaagatt atgctaaaag ttgttctgtt tcgtctttgc ctattcatac
     2821 ctcatcacca agtgctgttt gtgtagcgtt aaattttcaa ttacgtggag gtgctggtct
```

2881 ccgaacttct gtgcaggcct tttcagaagg tggtctttcc ttaataccgg gtttactaca
2941 tcctcatgga aatggagtta ggggaaatgt aaaacgtttt ttacaaccca tagcatcaga
3001 gttcactcaa tcgcctcatt gtaacacttt ttcctgtacg tcatcgtttg tttttggaaa
3061 tgttttatct acaaggaggg acttagaaac aaatgatggg cgggaggtaa gggttaccaa
3121 gtatctattt aaccactctc agggagagaa aatgaaacgt acaggtggta ggggagatgc
3181 agcagatatt gtaagccatg cgttaacttt agctctattt ttatcaaata ttggtttttt
3241 tgtgcttcac atttttcgtc gtttaaagtg gcagacttta ccagacaagg cattgaacct
3301 gtcgttactg cctcaagcca cagatgatgc taaggtgcgt caagtacttt gtgaagtaga
3361 tgtccagggt catctggaag tggcttctgc aaagaaattc gaatcactac gtgcttactc
3421 acgttctcat agtgaaggtt atttcgaggg aaggttttca gaacaaatgg gtaatgttag
3481 gaatttacat caacttgcag atcagttgat gagtgctgag cctaattttg gtaatgatag
3541 taatgttaat ggtgagtaca gggctaggta tgaagtttta tttaatgaga tttctcgtct
3601 gttgtctccg ttattaaatg gaaaccgtct actcgtgaac aatgaagcta aatttcaggc
3661 tttgttgcgt gggatatttc aaagttgcga taatcctgcc aaggtaatta ttgagttcca
3721 gctacagaga ggaaggaaaa tagacctagt attatcaaaa tctgcggaaa atgatgatac
3781 tcatccaatt ggaatagagt tgaagtatgc taacaccgca gaacaagttg aacgaaaaag
3841 ggtggaggca aatcgacagt taagtgaata cgaattttgt ggaggatgca agcgtattac
3901 tgggggagat gcgatggttt tgttatacgc tatattaaat gctgtaggac aagagcagga
3961 tctgatattg attggtgggc ttcgtagagc atctgggttt tctagatgac ttgtaactaa
4021 taaaagtacc aaagataagg ttgtttatgg gtaaaattat gcattaagct agagtacgtt
4081 tgagtaaggt tggagaaaaa gcaaggtctt tgagcaagaa aatttgattt atgttttctt
4141 aagttaaagt taacctgatg aagtttgtat aatttattgt tggagttttt ttgttctatt
4201 tttactataa gttcaatctg ctctatcttt ggtctttttt tcttagtttt gcggcaagtc
4261 taatgttttt actacttaat ggaataactt gcagaaagtg gagatataat gctaaggcag
4321 aggagctcag acagttatct taaacaataa agaaatacta aaggcaaaag atacgcttgc
4381 gtagttcgaa agctttctat aggataagcc tcacctgatt tacaaataga attgttatgt
4441 tagatgtttg ctatttattc ttaagctcat tttaacagca ttttgctttt tttagtatgt
4501 aggggttttc taggaattat cctactgaat tctatgatat tattttttgt aacattgtta
4561 tacattataa tgcacccatt tgtctagacc attttttttca aagtgatccg atttttaaaa
4621 tgaatatgtt gataaatacg tcgacacata tttaaagtat ttatgaatat attcatttac
4681 tgtttatgat aatagagatc agcaggaact ttataatgta gagtctgatg tcgccttcta
4741 tagttatacc aagcaacaaa atcgtttatt ataagattca aatctctgat actatttggt
4801 ctataataat atatagcttc ttgctttaaa gttctccata agcgctcaac aaatatattg
4861 tcgaagcaac gtcctttatg gtccatactg attttaatat tagcacgctc taattccata
4921 ataaagttgt agctagtaaa ctgtacccct tgatcactat taaaaatctc aggtttacct
4981 tgttttagag cttctttgag agtataaagg caaaactcca gcatcgagat atggtgataa
5041 tgaatgagca ataatatagc gactatacaa gtccattatt gccacaaaat agacaaactt
5101 accttctacc ataatatatg ttatatcagt agcccatacc tgattaactc tacaaataat
5161 caaatctttg agtaaataag gatatatttt atgctttttt tctttaatac ttgtattaca
5221 tctttttcta caatacagcc cactaatctt catttttttc ataattctta agattttttt
5281 gtgattgact actactccac tcgctatgat ctcagcagta attttacgat atccataacg
5341 gcaatcagaa gccaaatata cttcttgaat caaatttcac tttcgttatt aattataggc
5401 ctataatata ggctagatct gcaaatcccc agtaaatcag cctatttcct aattgacaga
5461 tcagaatctt tttctataaa tcttactcta tctttttttgc ttatttcagt aatttttttt
5521 tcaaataact attttccact gtcaattctc ctattacttt atgtaaactt tctatttctt
5581 gcgctaagat tctttgtttt ctcgcacttt cactttcttc aacaaatagg tcttttaacc
5641 ttgccaatac tctatcacgc caatcatata gatttgttga tggtatttta tattcactac
5701 atatctcagc tgtgcttctt tgatttttta ttgcttccaa agctatcttt gcttttaact
5761 ctggttcata tttttttgtt gccatacttt taccccttta ctttcctact cggatcaacc
5821 atttttttttg gtctagttta tggggtgcat tatattctat gagaaattct acagatggta
5881 aatggccttc cttggcagca tagtagactg atctgtagtt ttagaattat taataaatga
5941 ttcttgttgt tgcttttcta ttagaagctt tacaattcct agatatcctt tattggtagc
6001 ataatgcaaa ggagtccaat tgtttttacc ttttgtacca acttctactc ccttattgag
6061 agaaaatttt acaatatcga tatttcaaaa atcagctgca atatgaatag gcttgttttc
6121 gtcattagtt ttagcgttaa aacttacacc tttattattt actaaaaatt ttgttacttc
6181 taagctatca aacttagctg gatggtgcac agccatcttg accaaaaaat catcccccta
6241 tcgcgttctt atcattatgc cttataccct gtattacacc ttcagatata gtcatttcta
6301 acctccaaca tccttgctaa tagaccctca ctaaaactgc cgaaaagact ttataagtta
6361 atattctaaa tctcagtatt ttatggtgca ggttttgatt tttaaaacat tatactttgt
6421 gtagggtata actacacgca tcaaagtgaa agaaaagcaa aatgatgaag aaaatgaaag

6481 tttaagtttt cttcaacgac tcaaggagag aactatgctt taaagcctgt attagcaaga
6541 gccttaatca ctaaatgaac aatgtcacgc agagtttcat cctcaatttt tctatattcc
6601 ctcactagcc ttgatatttc ttcgctgcct acatcgctat ggaaggcctt atcatgtaag
6661 tcttcagagg ctttggtaaa aaaatacgac atatcaaccg atagtacttc tgctaattta
6721 tataacctat caactacaag cctaatctct ccgctttcat atccttgtac atgctgaaat
6781 gaaatttcaa gctttttttcc taagtctctt tgactaaaac cacacattag ccttcttttc
6841 cttattcttt cacctatttg tttatcgata ggatgttgta tagtcatttt acgtatcagg
6901 taatattaac tatgtattat cactttgaga aaaagatttt accaatatat aaactgcact
6961 acgtaacctt ttgctcttaa tttcgttata tcctttgact aactttagta tttcttcatc
7021 ataagcttca tgtgagtcag tgtgcatatc agtaaaaaag ttagcaacat ctatcgatag
7081 tgcttttgct aaatcatata gtctactggc caaaacacaa tttgaaccat tttcgtactt
7141 ttgtatctgt ctatacgaaa cacctattct ctttcccaat tcggtctgtg tcaaagcacg
7201 atctaaccta agttgtttta ttttttttacc taattgcact tttcacctct tattcaaatt
7261 tacaacaaat tttatgaagt ggaaaaaaag ctagcaaagt aattgcttta ataatttctg
7321 cctctcgcta attaaatcgt ttggccttac gtattttttc tcaatttccc tcatctcttt
7381 ttctcgttct gcaatgctat ttcgtattac ttcagccgag tatctgcttt tgccacttgc
7441 aaactctgat cttaatcttc taaccttgta ttcgagctca gcaaatcttt cgtgttgctc
7501 aagaagttct atcatatgac taatttcaga gtttagcttt ttttcctcaa catctcttac
7561 aataaaagaa agtagaagct tatctatttg gtaatcaata tttggttgag cagcaaatag
7621 atcaatcatg gcttttacgt cagtgggatt tgcaactagt aagttaaaat tactacataa
7681 aaaactatag aattttattg tcttctcaag ataatttttc atatcttgtg ataattgcaa
7741 ttctttgtga attcctgaca gcaatatttt taattcttcc aagtattcta catcaaaatc
7801 acctacttct gtaggtaaaa ggatcattag cagctttttt acaagctcaa catcatttga
7861 cttaactgca ttataaagag ctttttcttt atcttttttt aaatcataaa gctctgatga
7921 gttatctatg ctgaacaggt aatttgccac ttcagtgttt ccttgaggag agtagattaa
7981 aatgtccact tctcttagcg ggtgatcatc attaaacttt tctagtaact gcttatcggt
8041 agtttcctct attgctacaa ctgctttgct tacctctttc aactttgcaa tatcatctct
8101 tgagttttca agtatatctt tagccttgtc tagcagctgc tcatatagaa ctatttttttc
8161 ttcatcatca agtagctgac aatgcttttt cagcttttca gtagagtaag ataggatatc
8221 catttttttc aattttacga ttacgatacc ccatttttgt tgaaactgaa ataattttta
8281 taggatgtgt ttttctagtg tagatgcccg caaaacttgt tattataata tacagtaaat
8341 caataagtcg tataatatta acaattaaat aatatttatc acgatatgat cgaatagtaa
8401 tgaaattaag tgagggttac aatgaaaaaa ataaatggta aaaaattgtc tagtaaaatg
8461 ttaaaagcgg tagagcgaat agcaaaagaa gggttattac ctaattttat aaaagctgct
8521 ccaacagcaa ataaaaagat tagtaaatta cctgtcttaa catatcctga ggagctcaat
8581 ccatttcgat cagctttatg caagataagg ctaagtgaat atgaaaatgt gaaattaaat
8641 gaattcttgg aaaaagctat tgcagctgaa aatctagaca aattaagtaa aatcgtcgat
8701 gaagctttgg atttaggaat aagagtaaac gctcgcaacg aagatggctt tagcttcact
8761 aacgttacaa tgtttaagat gtatcgtgat gaattcaaag aaaataaaca agaagatata
8821 atcagaaagt tagccttaca tggagcagat tttaatgtac aagttgataa gaaaatagct
8881 gaaatgtatc aaaaagtgca aaaagaaatt gagccacaaa tatatagtag gctcaagaaa
8941 cttagggaag ttggagaaga cgctactata gaaggaactg ttgaagatgt agaaatcgat
9001 aataagacct tctatattga attttcacat agctgcatag ttgatgttgc caaagttgta
9061 gaaggtgcaa aaaatttggg cttaagtaaa ggagacttaa accttggcgg tagtatcata
9121 aaaataggtg atggtgaagt tgaggtcaaa actgggaaag acggtaaaag aaattacgta
9181 gatatatccg ataacagcgc ttttacagta acactttgca ctagcctagg agagctaaat
9241 ttaatggtat atcatgacac agaagactat catcaagtac aagttgaggt agaagatgaa
9301 gaaatgtgga acgagttgca agaaatagga gaaatcgtag gaaagggttg tttatttggt
9361 ggaatgtctg ttaaagaagt agtggaaaaa ggcagcttta ctagatgtgg aaggtggggc
9421 gaccaaaatt ctaaaagtag tgagaccttt tcttgggggga gcaaattaaa agataccaaa
9481 acgacatctg taggacgttc ttgattggtg gctatgtttt tacatagcca taattattgc
9541 aaagtaaagt agttcttgaa atttaatcaa aattaccata aaaaatttac cttaactagc
9601 attgcttgta gaagtggatt gaagatatat tcaggcaata ttatttattg gcaagcgctt
9661 ttatcactga atgcacaata tcacgcaacg tttcatcctt aattttcctg tattccctca
9721 ctagccttga tatttcttca ctacccacat cgctacaaaa ggccttatcg tgtaagtctt
9781 cagaagcttt ggtaaaaaaa tatgacatat caacggacaa agcttctgcc aaattataca
9841 atctatcaat tacaagcctt acctctccag tttcatatcc ttgtatatgc tgaaatgaaa
9901 ttccgagctt ttttcctaag tctctttggc taaaaccgca tattaacctt ctcttcctta
9961 ctctttcacc tatttgttta tcaataggat gttgttcatc ttgtacaatc atttattaat
10021 atattaactg aggtattgaa gcaatactaa ctctcattgt aactttgaga aaaagatttt

```
    10081 accaatatat aaactacgct acgtaacctt ttgcttttaa tttcattata tcctttgact
    10141 aattttagta tttcttcatc ataagtttca tgtgagtcga catgcacatc agtaaaaaaa
    10201 tcagccacat taatcgatag tgcttttgct aagtcgtata gcctactggc caaaatttgg
    10261 tttgaactat tttcgtacct ttgtatttgt ctatacgaaa cgcctatttt ctttcccaac
    10321 tcagtttgtg ttaaaccaca gtataatcta agttctttta tttttttgcc taattccacc
    10381 tttcattact aattttagct gctcaagctt atattgaatt ttttaattta tgaagtagaa
    10441 aaaaagaagg aaagttggag aactttcctt ccggtctaca gattacaata aaccactatc
    10501 ataaaaacta aagtggctct tctgtgttac gattatgtga tctattaatt tcattcctac
    10561 attttcgcag gttgcagcta atgtaagtgt gttatcctca tcctcctttg atggttctgt
    10621 atctccactt ggatgattat gtgaaattgc aatagatgat gcatcgatca gtaatccctg
    10681 tttgattatt tctctcacat atagaggtac tctgtctata gttccacagt cttgaatata
    10741 ctcgtatatc aaatgaccac ttcgatctaa gtatattact cgcaagcttt cctttcgtga
    10801 ctgccctatt gctgttttaa agtattttag tagctcttct ctgttatcaa gaatcgacag
    10861 cttttttaac ttacctttca gtatcctatc gaaaatctct ttcagacaaa agatacttgc
    10921 tattgctcca tcattcattc ctgtaacatt ttttagctca tgaaagtcag cgttaattac
    10981 ccttcctaaa ctactaaaga gctctattag ctttttggcc acttctcttc cttcttcaca
    11041 ttcagataga gtacgttcca gtagctcatg gtcaagtaaa gacctgcctc tacttgataa
    11101 aatgcgagtt tccaattttt cttttctctc actattattc ataaaaacct ccaaggtcaa
    11161 cattatatac aaattcaacg ccagctttct gacgcagtag ttctcgagga ataacccttt
    11221 tgaactctaa atttgcaatg ttgtccttgt tgctttaaaa gctggaatac catttgccat
    11281 atgaatttgt gcattgacat ttactgtgct aattccctga agcgcaacgg tatttatatt
    11341 tgcaatcata atatattata taaaattgtt aatataata
//
LOCUS       JAQZAU010000013            5775 bp    dna     linear   ENV 17-MAR-2023
DEFINITION  MAG: Wolbachia pipientis isolate wEsol contig_14972, whole genome
            shotgun sequence.
ACCESSION   JAQZAU010000013  JAQZAU010000000
VERSION     JAQZAU010000013.1
DBLINK      BioProject: PRJNA929258
            BioSample: SAMN33142973
KEYWORDS    WGS; ENV; Metagenome Assembled Genome; MAG.
SOURCE      Wolbachia pipientis (insect metagenome)
  ORGANISM  Wolbachia pipientis
            Bacteria; Pseudomonadota; Alphaproteobacteria; Rickettsiales;
            Anaplasmataceae; Wolbachieae; Wolbachia.
REFERENCE   1  (bases 1 to 5775)
  AUTHORS   Fiutek,N., Couger,B.M., Roy,S., de la Torre,J.R. and Connor,E.F.
  TITLE     Genomic assessment of the contribution of the Wolbachia
            endosymbiont of Eurosta solidaginis to gall-induction
  JOURNAL   International Journal Molecular Sciences
REFERENCE   2  (bases 1 to 5775)
  AUTHORS   Fiutek,N., Couger,B.M., Roy,S., de la Torre,J.R. and Connor,E.F.
  TITLE     Direct Submission
  JOURNAL   Submitted (10-FEB-2023) Biology, San Francisco State University,
            1600 Holloway Ave, San Francisco, CA 94132, USA
COMMENT     ##Genome-Assembly-Data-START##
            Assembly Date          :: JUN-2019
            Assembly Method        :: FLYE v. 06-2019
            Genome Representation  :: Full
            Expected Final Version :: Yes
            Genome Coverage        :: 100.0x
            Sequencing Technology  :: Illumina HiSeq; PacBio Sequel
            ##Genome-Assembly-Data-END##
FEATURES             Location/Qualifiers
     source          1..5775
                     /organism="Wolbachia pipientis"
                     /mol_type="genomic DNA"
                     /submitter_seqid="contig_14972"
                     /isolate="wEsol"
                     /isolation_source="galls on Solidago altissima"
```

```
                     /host="Eurosta solidaginis MN-2018-EFC"
                     /db_xref="taxon:955"
                     /environmental_sample
                     /country="USA: Cowling Arboretum, Carleton College,
                     Northfield, MN"
                     /lat_lon="44.48 N 93.13 W"
                     /altitude="280 m"
                     /collection_date="2018-11"
                     /metagenome_source="insect metagenome"
                     /note="metagenomic"
     gene            complement(<1..362)
                     /locus_tag="wEsol_00127"
     CDS             complement(<1..362)
                     /locus_tag="wEsol_00127"
                     /codon_start=1
                     /transl_table=11
                     /product="hypothetical protein"
                     /pfam="Ank Ank_4 Ank_2 Ank_5 Ank_2 Ank Ank_4 Ank_5 Ank
                     Ank_3"
                     /translation="MMDLVRYLVDKGADVYTQDKGGKTLLDIATDQKHNNIVEYLKRV
                     QLDKELLIAAQYGNLSKVEDLVSQRASLSAKYSDGRTLMHCAAYGGELNVVKYLVVGE
                     KSSLKIKDNSGRIPIQYA"
                     /cog="Arp COG0666 235 FOG: Ankyrin repeat"
                     /besthit="qcoverage 53.3333333333333 , hcoverage
                     1.67451596023025 , similarity 98.4 , identity 96.9 ,
                     evalue 2.95e-29 , alnlength 64 , Bacteria ,
                     Proteobacteria , WP_208571570.1 ankyrin repeat
                     domain-containing protein [Wolbachia pipientis]"
     gene            complement(331..726)
                     /locus_tag="wEsol_00128"
     CDS             complement(331..726)
                     /locus_tag="wEsol_00128"
                     /codon_start=1
                     /transl_table=11
                     /product="hypothetical protein"
                     /pfam="Ank_2 Pfam-B_10896 Ank_5 Ank_4 Ank Ank_3 Ank_4
                     Ank_5 Ank Ank_3"
                     /translation="MLTDGRTDGRTDMAQSNFFSILIIILIYGSLYLSRFLYICTTNRY
                     PIKINILCELCSTEYKNGWTPLHFAAWRGLLEVANFLIEKGADINAENIFGRIPIHIA
                     AENNNKDIIDFFLSKGMSIDDGFSKVPRR"
                     /besthit="qcoverage 23.6641221374046 , hcoverage 25 ,
                     similarity 87.1 , identity 71.0 , evalue 4.99e-07 ,
                     alnlength 31 , Eukaryota , Arthropoda , XP_011706058.1
                     PREDICTED: receptor-interacting serine/threonine-protein
                     kinase 4-like, partial [Wasmannia auropunctata]"
     gene            3112..3564
                     /locus_tag="wEsol_00129"
     CDS             3112..3564
                     /locus_tag="wEsol_00129"
                     /codon_start=1
                     /transl_table=11
                     /product="hypothetical protein"
                     /translation="MICFQKGGLLFDEFDMLFHSLYEETDTYQNIIRAIAKKPKGISR
                     KELIKVTKITEGGRLTDKLRSLEEAEFIGGFLPLNKGKRGVYYRVMDEYVLFYLTWIE
                     PFKEVTKRAIISYAHYWELAIKKPAWQTWTGQTFEAVCFKHANVIRKN"
                     /besthit="qcoverage 99.3333333333333 , hcoverage
                     46.417445482866 , similarity 96.0 , identity 95.3 ,
                     evalue 4.69e-95 , alnlength 149 , Bacteria ,
                     Proteobacteria , MBV2146633.1 winged helix-turn-helix
                     domain-containing protein [Wolbachia endosymbiont of
```

Pissodes strobi]"
gene 3678..3887
/locus_tag="wEsol_00130"
CDS 3678..3887
/locus_tag="wEsol_00130"
/codon_start=1
/transl_table=11
/product="hypothetical protein"
/translation="MLSEIKYSDKPYTVTKEFVEQLKRKEAIYREKTKSKKQIFWVLI
AANGVSENQYLKDTIHHVITLEDLF"
/besthit="qcoverage 100 , hcoverage 21.4953271028037 ,
similarity 98.6 , identity 98.6 , evalue 7.80e-36 ,
alnlength 69 , Bacteria , Proteobacteria , MBV2146633.1
winged helix-turn-helix domain-containing protein
[Wolbachia endosymbiont of Pissodes strobi]"
gene 3995..4579
/locus_tag="wEsol_00131"
CDS 3995..4579
/locus_tag="wEsol_00131"
/codon_start=1
/transl_table=11
/product="hypothetical protein"
/pfam="UbiD"
/tigr="TIGR03701 mena_SCO4490 433 menaquinone
biosynthesis decarboxylase, SCO4490 family"
/translation="MTEIHRRVLSNKGPAIIFENVVTENGKNSIPVLVNLFRTIKRIA
LGLNINPGELRDLGKILAFLRSPEPPKNFKDAVKMFPLLKVVLSMRSKVVSKAPCLEV
VLTGDEVDLSLLPIQTCWPNEPAPLITWPIVVTKGPTEDKEDNFNLGIYRMQVIDKKN
DSNALACTSWWCRSPQTMEREGTKYEISCCCCDR"
/cog="UbiD COG0043 477 3-polyprenyl-4-hydroxybenzoate
decarboxylase and related decarboxylases"
/besthit="qcoverage 81.9587628865979 , hcoverage
72.6027397260274 , similarity 98.1 , identity 94.3 ,
evalue 1.07e-101 , alnlength 159 , Bacteria ,
Proteobacteria , POG51429.1 hypothetical protein
BJU59_05630, partial [Wolbachia sp. wRi_2]"
gene 4548..4859
/locus_tag="wEsol_00132"
CDS 4548..4859
/locus_tag="wEsol_00132"
/codon_start=1
/transl_table=11
/product="hypothetical protein"
/pfam="UbiD"
/tigr="TIGR00148 TIGR00148 438 UbiD family decarboxylase"
/translation="MKFPAAAVIGSDPAKIIAAVTPVPETLSEYQFAGLLRKKPLELV
NCKTIPLQVPAHAEIVLEGYVSLDRYQDEGPYGDHTGYYNSVEQFPEFNITAITTNRI
I"
/cog="UbiD COG0043 477 3-polyprenyl-4-hydroxybenzoate
decarboxylase and related decarboxylases"
/besthit="qcoverage 98.0582524271845 , hcoverage
61.9631901840491 , similarity 97.0 , identity 96.0 ,
evalue 1.54e-62 , alnlength 101 , Bacteria ,
Proteobacteria , EAL58158.1
3-octaprenyl-4-hydroxybenzoate carboxy-lyase, partial
[Wolbachia endosymbiont of Drosophila ananassae]"
gene complement(5313..5666)
/locus_tag="wEsol_00133"
CDS complement(5313..5666)
/locus_tag="wEsol_00133"

```
                     /codon_start=1
                     /transl_table=11
                     /product="hypothetical protein"
                     /translation="MDPVLFLSDAARYSSSAFASYVRTNIKWNDLALIRISTRHNKSF
                     PRWSGLVPERTGSAFGNIDNTPQQQQQPQSQHRGSELSFPSNDNCDRRRGTMGRYGQK
                     VEVLVTGTCRIDVYP"
ORIGIN
        1 gcagcatact gaatgggaat tctaccacta ttatctttaa tttttaaact actttttttca
       61 cctaccacaa gatattttac tacatttagc tcgcccccat aagcagcaca atgcataaga
      121 gttcggccat cgctatactt agcacttaag ctagctcttt gacttacaag atcttcaact
      181 ttgctaagat ttccatattg tgctgcaatc aataattctt tatctaattg cactcgtttt
      241 aaatactcta caatattatt gtgtttttga tcagtagcaa tatccagcaa tgttttaccg
      301 cctttgtctt gagtgtaaac gtcagccccc ttatctacga ggtaccttac taaatccatc
      361 atcaatgctc attcctttgc tgagaaaaaa atctataatg tctttgttat tgttttcagc
      421 ggcaatgtgt ataggaattc tgccaaaaat gttctcagcg ttaatatcag cacccttttc
      481 tattaaaaag ttagcaactt ctaacaagcc tctccatgca gcaaaatgta gcggtgtcca
      541 accattttta tactcagttg agcagagctc acagagtata ttaattttga ttggataacg
      601 gttggttgta catatataaa ggaatcgaga tagatataga cttccatata tcaaaataat
      661 caggatcgaa aaaaaatttg attgagccat gtccgtccgt ccgtccgtcc gtccgtccgt
      721 taacacgata acttgagtaa attttgaggt atcttgatga aatttggtat gtaggttcct
      781 gagtactcat ctcagatcgc tatttaaaat gaacgatatc ggactataac cacgcccact
      841 ttttcgatat cgaaaatttc gaaaaaccga aaaagtgcga taattcatta caaaagacag
      901 ataaagcgac gaaacttggt agatgggttg acgttatgac gcagaataga aaattagtaa
      961 gattttggac aatgggcgtg gcaccgccca cttttacaag aaggtaattt aaaagttttg
     1021 caagctgtaa tttggcagtc gttgaagata tcatgatgaa atttggcagg aacgttacta
     1081 ctattactct atatgtgcta aataaaaatt agcaaaactg gatgaagaac acgcccactt
     1141 tttaaaaaaa atttttttaa attcaaattt taacaaaaaa tttaatatct ttactgtata
     1201 taagtaaatt aagtcaaaat tcaactccag taatgatatg atgcaacaaa atacaaaaat
     1261 aaaagaaaat ttcaaaatgg gcgtggctcc gcccatttc atttagtttg tctagaatac
     1321 ttttattgcc ataagtcgaa caaaaattta ccaatccttc tcaaatttgg taggagcata
     1381 gattctatga caataactgt tctctgtgaa aatgggcgaa atcggtggaa gccacgccca
     1441 gtttttatac acagtccacc gtctgtcctt ccgctcggcc gttaacacaa taacttgagc
     1501 aaaatccgat atatctttac taaacttagc ccacgtactt acctgaactc actttttctt
     1561 ggtataaaaa atgggcgaaa tctgaccata accacgccca ctttatcgat atcgaaaatt
     1621 acgaaaaatg aaaaaaatgc cataattcta taccaaatac gaaaaaaggg atgaaacatg
     1681 gtaactggat tggtttattg acgcaaaata taactttgga aaaaactttg taaaatgggt
     1741 gtgacaccta ccatgttaag tagaagaaaa tgaaaaagtt ctacaaggcg caatcaacag
     1801 cccttggaat cttggcagga atactgttag tggtattgca tatataaata tattagcagt
     1861 acccgacaga tgattttctg gatcacctgg tccacatttt ggtcgatatc gcgagaacgc
     1921 cttcacatat acatctaagg gccactcgct tttaaaaccc tcattaatac ctttaatttg
     1981 atatccatat cgtacaaaaa cataccagag tcacccctgt cccaccctaa tggcgatatc
     2041 tcgaaaaggc gtccacctat agacctaatg cccactccct cttaaaatgc tcagtaacac
     2101 ctttcgtttg atacccatat cgtacaaaca ttctagagtc acccctggcc caccctaatg
     2161 gcgatatctc gaaaaggcgt ccacctatag acctagtgtc cactccctct taaaatgctc
     2221 agtaacacct ttcgtttgat acccttatcg tacaaacatt ctagagtcac ccctggccca
     2281 tcctaatggc gatatctcga aaaggcgtcc acctatagac ctaatgtcca ctccctctta
     2341 aaatcctcag taacagcttt cgtttgatac ccatatcgta caaacattct agagtcaccc
     2401 ctggcccatc ctaatggcga tatctcgaaa aggcgtccac ctgtagacct aatgcccact
     2461 ccctcttaaa atgctcagta acacctttcg tttgataccc atatcgtaca aacattctag
     2521 agtcacacct ggcccaccct aatggcgata tttcgaaaag gcgtccacct atagaactaa
     2581 ggattactcc cttttaaaat actcattacc acctttcatt tgatacccat atcgtacaaa
     2641 cacattctag agtcaccctg gcccacccta atggcctgaa gtgtggctgg atgcattaca
     2701 acaattaaca cttgctattg ataacacaca tgagaatgaa cacattgtct tattttttga
     2761 tgaacttcca tggttagcta caagaaagtt tcgttttcta ggaacgttag aatatttttg
     2821 gaatatacat tggacaaacc gaaagaagct tatcttggtt gtatgtggtt cagcagcttc
     2881 ttggatgttg gaaaatataa tttataccaa aagggggggtt gcataaccgc attaccgcaa
     2941 ggataacttt gcagcctttt aacttacgtg aaactaaaga gtatctaaga tatttaggaa
     3001 tcaaccttaa tcaccagcag atattacaga tctatatggc tattggagga atgaatacca
     3061 cattacctta aggaagtaag taaaggatta tcagcagcac aaaacattaa tatgatctgt
     3121 tttcagaagg gtggtttgtt atttgatgaa tttgatatgt tgtttcactc cttgtatgaa
```

```
     3181 gagacagaca catatcaaaa tatcattcgt gcgattgcaa aaaagccaaa agggataagc
     3241 agaaaagaac ttataaaagt cactaaaatt actgagggtg gtcgcttaac tgataaatta
     3301 aggagtttag aagaagcaga atttattggt ggttttttac cattgaacaa gggaaagaga
     3361 ggagtatatt acagggtaat ggacgaatat gtattatttt atctaacgtg gattgagccg
     3421 tttaaggaag taacaaaaag ggcaataata agttatgctc actattggga gttagcaata
     3481 aagaaaccag cttggcaaac atggacaggg caaacatttg aagcagtgtg ttttaaacat
     3541 gccaatgtaa ttagaaaaaa ttagatatag agcatatagc agttatatgt ggagattggc
     3601 aatatcaacc agaaaaatac agtaaagagt ctggtgctca aatagatcta gttttcgatc
     3661 gagaagatgg atgtgtaatg ctcagtgaga taaaatatag tgataaacct tatacagtaa
     3721 caaaggaatt tgtcgaacaa ttaaaaagaa aagaagcaat ttatagagaa aaaacgaaat
     3781 caaagaagca aatattttgg gtgttaattg ctgcaaatgg agtttctgag aaccaatatc
     3841 taaaagatac gatacatcat gtgattacac tagaagactt gttttaagtt attttaaatt
     3901 ttaatatgta caccaactta cgcgacttca ttaaagcctt agaagaagaa aagatctgat
     3961 taggattaag gaggaagttt caacagttct tgaaatgaca gaaattcacc gtagggtttt
     4021 gtcaaataaa ggaccagcta tcatctttga aaatgttgtc acggaaaacg gtaaaaattc
     4081 gattccagtt ttggtgaatt tatttcgtac tattaagagg attgcattgg ggttgaacat
     4141 aaaccctggt gaactaagag atcttggtaa aattttagca tttttgcgat cacctgagcc
     4201 accaaaaaac ttcaaagatg ccgtgaaaat gtttccttta ctaaaagttg tattgtcgat
     4261 gcgaagtaaa gttgtgagca aggctccatg tctagaagtg gtgctaactg ggatgaggt
     4321 ggatcttagt ttgctaccta ttcaaacatg ctggccaaat gaacctgcac cacttatcac
     4381 ctggccaatt gtggtgacaa aaggcccaac ggaagacaag gaagataatt tcaatcttgg
     4441 aatatatcgt atgcaggtta tagataaaaa aaacgactct aatgcgctgg cttgcacatc
     4501 gtggtggtgc aggtcaccac aaacgatgga aagagaagga acaaaatatg aaatttcctg
     4561 ctgctgctgt gatcggtagt gatcctgcaa aaattattgc tgcggtaact ccagtgccag
     4621 aaacattgtc agaataccaa tttgctgggt tgctgcgcaa aaaaccgctt gagcttgtaa
     4681 attgtaagac tattccgctt caagtgccag ctcatgcaga gattgttctg gaaggctatg
     4741 taagtttgga tagatatcaa gatgaagggc catacggtga tcacaccggc tactataatt
     4801 ctgttgagca atttccagaa tttaatatta ctgcaattac tacgaaccga ataatttaaa
     4861 attttttag ataaataaga taaaaaaggc acatcccttt aagcttgtaa tacagtagac
     4921 gttcctatca ggttggccgg gttgaaagaa ggcgggcgtt gcacatggac agaaaagaga
     4981 tagaaccaag catgaagtgc aaaaagtcaa agttcatgcg caggtcttac aaccttagac
     5041 aaggtgctat ttgtctcaat atatcgattc tttttcgatg tgtgcctccc gagacgctgg
     5101 gaagtgcatg gccaaaaaag aggcatagcc acatccgata ggtaaaaggt ctcggatgag
     5161 atttctttat tctaaaaaca accacgacaa acgctttaag gatagattct gccttggcag
     5221 aatgacaata gggcattacg ccgtcaatgt tgttgtaggg ataaagactc ttgccgaagt
     5281 ttgtcggggt gttatcgatg ttgatggtct tttcatggat atacatcgat ccggcacgtt
     5341 ccggtaacaa gcacctcaac cttctggcca taccgcccca ttgttccacg acgtctatcg
     5401 caattatcat tagaagggaa gctcaattca gagccacgat gttggctttg ggttgttgt
     5461 tgttgttggg gagtgttatc gatgttgccg aatgcagatc cggtacgttc cggtaccaag
     5521 cccgaccatc tcggaaacga tttgttatgt ctcgttgaga tacgtatcaa ggcgaggtca
     5581 ttccatttaa tattggttcg cacatagctc gcaaaagccg atgaggaata tcgcgctgca
     5641 tctgataaga acaacactgg atccatttga gcaacagagt tgactgcact aatttttttt
     5701 tgatttcaga ttatagttag aactggaccc ataggattca agggattttt aagcgcaata
     5761 cgaaaactta tagct
//
LOCUS       JAQZAU010000014           1413 bp    dna     linear   ENV 17-MAR-2023
DEFINITION  MAG: Wolbachia pipientis isolate wEsol contig_15719, whole genome
            shotgun sequence.
ACCESSION   JAQZAU010000014  JAQZAU010000000
VERSION     JAQZAU010000014.1
DBLINK      BioProject: PRJNA929258
            BioSample: SAMN33142973
KEYWORDS    WGS; ENV; Metagenome Assembled Genome; MAG.
SOURCE      Wolbachia pipientis (insect metagenome)
  ORGANISM  Wolbachia pipientis
            Bacteria; Pseudomonadota; Alphaproteobacteria; Rickettsiales;
            Anaplasmataceae; Wolbachieae; Wolbachia.
REFERENCE   1  (bases 1 to 1413)
  AUTHORS   Fiutek,N., Couger,B.M., Roy,S., de la Torre,J.R. and Connor,E.F.
  TITLE     Genomic assessment of the contribution of the Wolbachia
```

```
            endosymbiont of Eurosta solidaginis to gall-induction
  JOURNAL   International Journal Molecular Sciences
REFERENCE   2  (bases 1 to 1413)
  AUTHORS   Fiutek,N., Couger,B.M., Roy,S., de la Torre,J.R. and Connor,E.F.
  TITLE     Direct Submission
  JOURNAL   Submitted (10-FEB-2023) Biology, San Francisco State University,
            1600 Holloway Ave, San Francisco, CA 94132, USA
COMMENT     ##Genome-Assembly-Data-START##
            Assembly Date          :: JUN-2019
            Assembly Method        :: FLYE v. 06-2019
            Genome Representation  :: Full
            Expected Final Version :: Yes
            Genome Coverage        :: 100.0x
            Sequencing Technology  :: Illumina HiSeq; PacBio Sequel
            ##Genome-Assembly-Data-END##
FEATURES             Location/Qualifiers
     source          1..1413
                     /organism="Wolbachia pipientis"
                     /mol_type="genomic DNA"
                     /submitter_seqid="contig_15719"
                     /isolate="wEsol"
                     /isolation_source="galls on Solidago altissima"
                     /host="Eurosta solidaginis MN-2018-EFC"
                     /db_xref="taxon:955"
                     /environmental_sample
                     /country="USA: Cowling Arboretum, Carleton College,
                     Northfield, MN"
                     /lat_lon="44.48 N 93.13 W"
                     /altitude="280 m"
                     /collection_date="2018-11"
                     /metagenome_source="insect metagenome"
                     /note="metagenomic"
     gene            <1..582
                     /locus_tag="wEsol_00134"
     CDS             <1..582
                     /locus_tag="wEsol_00134"
                     /codon_start=1
                     /transl_table=11
                     /fullproduct="qcoverage 100 , hcoverage 90.1869158878505
                     , similarity 100 , identity 100 , evalue 2.12e-134 ,
                     alnlength 193 , Bacteria , Proteobacteria ,
                     WP_141457257.1 MULTISPECIES: hypothetical protein
                     [unclassified Wolbachia]"
                     /translation="EDCTLVPESSFYKSVHYVDNVLGPVKDVICGVESKDDYNKYYKN
                     NDNCEYVGYRAEKVYSLCALSAILAANIILQPLFLIVACLSYFPVKLLSKITGDYCFK
                     TDTKSFVNYSNIFLEMGQDCANAVARVFNKIVSYACAAIIWAAASIITPLVWACDKVQ
                     SKLSEVQKPEKHQFLSKDNSKTTSVNGIPQTEL"
                     /product="hypothetical protein"
                     /besthit="qcoverage 100 , hcoverage 90.1869158878505 ,
                     similarity 100 , identity 100 , evalue 2.12e-134 ,
                     alnlength 193 , Bacteria , Proteobacteria ,
                     WP_141457257.1 MULTISPECIES: hypothetical protein
                     [unclassified Wolbachia]"
     gene            698..1408
                     /locus_tag="wEsol_00135"
     CDS             698..1408
                     /locus_tag="wEsol_00135"
                     /codon_start=1
                     /transl_table=11
                     /fullproduct="qcoverage 100 , hcoverage 100 , similarity
```

```
                     100 , identity 100 , evalue 2.17e-148 , alnlength 236 ,
                     Bacteria , Proteobacteria , WP_015589276.1 MULTISPECIES:
                     ComF family protein [unclassified Wolbachia]"
                     /cog="ComFC COG1040 225 Predicted
                     amidophosphoribosyltransferases"
                     /pfam="FYVE DUF1258 Pribosyltran"
                     /tigr="TIGR00201 comF 190 comF family protein"
                     /product="ComF family protein"
                     /translation="MNLLLLKKATNLIFPSVCVSCECIIDENLNLCSECNKKINFLTK
                     HYCNVCGVVISDNIYTCGKCIINPPPFKVLRSVFAYDQHSKNMIINFKFFDNLNYVKI
                     YAKWIHQANQDTFQNAEVVIPIPLHKMRLFKRKYNQAALLAKELTKLSNLSYTPFAIK
                     RPRHTIPQAGLSLKQREKNLKKAFSISNKKIIENKIVILVDDVVTTGATVRSCSQEIL
                     NSGAREVRVLSLARTVAC"
                     /besthit="qcoverage 100 , hcoverage 100 , similarity 100
                     , identity 100 , evalue 2.17e-148 , alnlength 236 ,
                     Bacteria , Proteobacteria , WP_015589276.1 MULTISPECIES:
                     ComF family protein [unclassified Wolbachia]"
ORIGIN
        1 gaagattgca cactagttcc agaaagtagt ttctataagt ccgtacatta cgttgataat
       61 gtgctaggac ctgtgaaaga tgtaatttgt ggagttgaga gtaaagatga ttataacaag
      121 tattataaaa acaatgataa ttgtgaatac gtgggttaca gagcagagaa agtgtattca
      181 ttgtgtgcac tgagtgctat tttagcagcc aacataatat tgcaaccttt atttttgata
      241 gtggcatgtt tgtcatactt tccagtcaag ttattgtcaa aaataacagg ggactattgt
      301 tttaaaacgg atacaaaatc attcgttaat tattcaaaca tatttttaga aatggggcaa
      361 gattgtgcta atgcagtagc tagagtattt aacaagattg taagttatgc ttgcgctgct
      421 atcatttggg ctgccgcgtc aatcataacg ccacttgtgt gggcttgtga caaagtgcaa
      481 agtaagttga gtgaagtaca gaagccagaa aaacatcaat ttttatctaa ggataattca
      541 aagactactt cagtcaatgg tataccacaa actgagttat aagttctgct tttcagcagc
      601 atgaactata ttgaaatgga ataaaaagtg aaagagtgta agcttaagtt actttagcta
      661 tactatataa attattttat aatatattta atgagttgtg aaccttctct tactaaaaaa
      721 agctacaaat ctcatatttc caagcgtatg cgtaagttgc gaatgtatca ttgatgaaaa
      781 tcttaatctg tgtagtgaat gcaacaaaaa aatcaatttt ctaactaagc attactgcaa
      841 tgtttgtggc gtagtaatct cagacaatat ttatacgtgt ggtaagtgca tcatcaatcc
      901 tccaccgttt aaagtattaa gatcagtttt tgcttatgat caacatagta aaaacatgat
      961 tataaatttc aaattttttg ataatttgaa ttacgtaaaa atctatgcaa agtggataca
     1021 ccaagctaat caggatacgt ttcagaacgc agaggtcgta attcctatac cgttacataa
     1081 aatgcgtttg tttaaacgta aatataatca agcagcactg cttgcaaaag agttgactaa
     1141 gttatccaat ttatcctata cgccatttgc aataaaacgt cctcgccata ctatccctca
     1201 ggctggtctt tcacttaagc agcgtgaaaa aaatttaaag aaggctttta gcataagcaa
     1261 caagaaaatt atcgaaaata aaatcgtgat attagttgac gacgtagtaa caactggagc
     1321 aactgtaagg tcctgctctc aagaaatttt aaattctggt gcaagagaag tgagagtgct
     1381 atcgcttgca agaacggtag catgttaaaa cgt
//
LOCUS       JAQZAU010000015            2282 bp    dna     linear   ENV 17-MAR-2023
DEFINITION  MAG: Wolbachia pipientis isolate wEsol contig_15818, whole genome
            shotgun sequence.
ACCESSION   JAQZAU010000015  JAQZAU010000000
VERSION     JAQZAU010000015.1
DBLINK      BioProject: PRJNA929258
            BioSample: SAMN33142973
KEYWORDS    WGS; ENV; Metagenome Assembled Genome; MAG.
SOURCE      Wolbachia pipientis (insect metagenome)
  ORGANISM  Wolbachia pipientis
            Bacteria; Pseudomonadota; Alphaproteobacteria; Rickettsiales;
            Anaplasmataceae; Wolbachieae; Wolbachia.
REFERENCE   1  (bases 1 to 2282)
  AUTHORS   Fiutek,N., Couger,B.M., Roy,S., de la Torre,J.R. and Connor,E.F.
  TITLE     Genomic assessment of the contribution of the Wolbachia
            endosymbiont of Eurosta solidaginis to gall-induction
  JOURNAL   International Journal Molecular Sciences
```

```
REFERENCE   2  (bases 1 to 2282)
  AUTHORS   Fiutek,N., Couger,B.M., Roy,S., de la Torre,J.R. and Connor,E.F.
  TITLE     Direct Submission
  JOURNAL   Submitted (10-FEB-2023) Biology, San Francisco State University,
            1600 Holloway Ave, San Francisco, CA 94132, USA
COMMENT     ##Genome-Assembly-Data-START##
            Assembly Date          :: JUN-2019
            Assembly Method        :: FLYE v. 06-2019
            Genome Representation  :: Full
            Expected Final Version :: Yes
            Genome Coverage        :: 100.0x
            Sequencing Technology  :: Illumina HiSeq; PacBio Sequel
            ##Genome-Assembly-Data-END##
FEATURES             Location/Qualifiers
     source          1..2282
                     /organism="Wolbachia pipientis"
                     /mol_type="genomic DNA"
                     /submitter_seqid="contig_15818"
                     /isolate="wEsol"
                     /isolation_source="galls on Solidago altissima"
                     /host="Eurosta solidaginis MN-2018-EFC"
                     /db_xref="taxon:955"
                     /environmental_sample
                     /country="USA: Cowling Arboretum, Carleton College,
                     Northfield, MN"
                     /lat_lon="44.48 N 93.13 W"
                     /altitude="280 m"
                     /collection_date="2018-11"
                     /metagenome_source="insect metagenome"
                     /note="metagenomic"
     gene            38..403
                     /locus_tag="wEsol_00136"
     CDS             38..403
                     /locus_tag="wEsol_00136"
                     /codon_start=1
                     /transl_table=11
                     /pfam="DDE_Tnp_1_5"
                     /fullproduct="qcoverage 100 , hcoverage 100 , similarity
                     100 , identity 100 , evalue 1.05e-83 , alnlength 121 ,
                     Bacteria , Proteobacteria , WP_155969141.1 MULTISPECIES:
                     transposase [Wolbachia]"
                     /translation="MPQKMRVSNCHEYNKFLQERGSIFCYINDAIENWYENCPKMQGG
                     NYIYSDKVVILVHIIVSFFRIGLRQTVGFIKGYLQQIGRDLAVISYSQASRRFKKLNI
                     KINDCRIDKNIALSIKVRM"
                     /product="transposase"
                     /besthit="qcoverage 100 , hcoverage 100 , similarity 100
                     , identity 100 , evalue 1.05e-83 , alnlength 121 ,
                     Bacteria , Proteobacteria , WP_155969141.1 MULTISPECIES:
                     transposase [Wolbachia]"
     gene            437..817
                     /locus_tag="wEsol_00137"
     CDS             437..817
                     /locus_tag="wEsol_00137"
                     /codon_start=1
                     /transl_table=11
                     /fullproduct="qcoverage 100 , hcoverage 100 , similarity
                     100 , identity 100 , evalue 3.88e-86 , alnlength 126 ,
                     Bacteria , Proteobacteria , AOV87628.1 transposase
                     [Wolbachia endosymbiont of Drosophila incompta]"
                     /cog="COG3415 COG3415 138 Transposase and inactivated
```

```
                     derivatives"
                     /pfam="HTH_Tnp_IS630 HTH_23 HTH_28 HTH_29 CENP-B_N HTH_7
                     HTH_23"
                     /product="transposase"
                     /translation="MPAAYSYDLRKKAMEALDEGESRETVAARFKIGRTTLWEWQQRR
                     KETGDFQSKKLGNGGYNHKITDWDTFAKFAREHGGKTLSEMAKLWNNVSIQTIYRALK
                     KIGFTRKKRHMDTRKETKKNVLNF"
                     /besthit="qcoverage 100 , hcoverage 100 , similarity 100
                     , identity 100 , evalue 3.88e-86 , alnlength 126 ,
                     Bacteria , Proteobacteria , AOV87628.1 transposase
                     [Wolbachia endosymbiont of Drosophila incompta]"
     gene            814..1143
                     /locus_tag="wEsol_00138"
     CDS             814..1143
                     /locus_tag="wEsol_00138"
                     /codon_start=1
                     /transl_table=11
                     /product="hypothetical protein"
                     /pfam="DDE_3"
                     /translation="MKIIATKEPKNLVYIDESGIDNTEDYPYGYCQKGQRFYALKSGK
                     KTQRISMVAALSGRKIVVPLTFEGHCNMDVFNGWVEQFLTPILEPGQTDLTQKKWRER
                     KEFFWVK"
                     /besthit="qcoverage 84.4036697247707 , hcoverage
                     69.1729323308271 , similarity 98.9 , identity 98.9 ,
                     evalue 1.75e-61 , alnlength 92 , Bacteria ,
                     Proteobacteria , EAL58695.1 putative transposase
                     [Wolbachia endosymbiont of Drosophila ananassae]"
     gene            1162..1344
                     /locus_tag="wEsol_00139"
     CDS             1162..1344
                     /locus_tag="wEsol_00139"
                     /codon_start=1
                     /transl_table=11
                     /product="hypothetical protein"
                     /pfam="HTH_Tnp_1 HTH_23 HTH_28"
                     /translation="MAREYTAEFKLEAVKLTNEQRKVGQPVAKVARDLGIRDSVLGKW
                     MKKYNEKSQRQMHFQM"
                     /cog="COG2963 COG2963 116 Transposase and inactivated
                     derivatives"
                     /besthit="qcoverage 85 , hcoverage 70.8333333333333 ,
                     similarity 100 , identity 100 , evalue 8.30e-26 ,
                     alnlength 51 , Bacteria , Proteobacteria , WP_094189595.1
                     transposase, partial [Wolbachia endosymbiont of
                     Drosophila ananassae]"
     gene            1877..2041
                     /locus_tag="wEsol_00140"
     CDS             1877..2041
                     /locus_tag="wEsol_00140"
                     /codon_start=1
                     /transl_table=11
                     /fullproduct="qcoverage 100 , hcoverage 100 , similarity
                     100 , identity 100 , evalue 4.17e-28 , alnlength 54 ,
                     Bacteria , Proteobacteria , WP_156768661.1 MULTISPECIES:
                     hypothetical protein [Wolbachia]"
                     /cog="Tra5 COG2801 232 Transposase and inactivated
                     derivatives"
                     /pfam="rve"
                     /product="hypothetical protein"
                     /translation="MSSSINKQLVMDSLLMAVNKRKPAKNLLLHSDQGSQYTSQGYQY
                     LLSIKNIDES"
```

```
                     /besthit="qcoverage 100 , hcoverage 100 , similarity 100
                     , identity 100 , evalue 4.17e-28 , alnlength 54 ,
                     Bacteria , Proteobacteria , WP_156768661.1 MULTISPECIES:
                     hypothetical protein [Wolbachia]"
     gene            2031..2195
                     /locus_tag="wEsol_00141"
     CDS             2031..2195
                     /locus_tag="wEsol_00141"
                     /codon_start=1
                     /transl_table=11
                     /fullproduct="qcoverage 100 , hcoverage 100 , similarity
                     100 , identity 100 , evalue 5.23e-31 , alnlength 54 ,
                     Bacteria , Proteobacteria , WP_080717614.1 MULTISPECIES:
                     IS3 family transposase [Wolbachia]"
                     /cog="Tra5 COG2801 232 Transposase and inactivated
                     derivatives"
                     /pfam="rve_3 rve rve_2"
                     /product="IS3 family transposase"
                     /translation="MSHKGCCYDNSVVESFFSSLKREILIDTSQHSAQQTRTAIFEYI
                     EIFYNKQRHY"
                     /besthit="qcoverage 100 , hcoverage 100 , similarity 100
                     , identity 100 , evalue 5.23e-31 , alnlength 54 ,
                     Bacteria , Proteobacteria , WP_080717614.1 MULTISPECIES:
                     IS3 family transposase [Wolbachia]"
     gene            complement(2196..>2282)
                     /locus_tag="wEsol_00142"
     CDS             complement(2196..>2282)
                     /locus_tag="wEsol_00142"
                     /codon_start=1
                     /transl_table=11
                     /product="hypothetical protein"
                     /translation="LTLPRKSGEKVWNVFHKENDESKCSGMQ"
ORIGIN
        1 tcattataaa tagccattta actgttgaag aaaaaatatg ccacaaaaaa tgagagtcag
       61 taactgccat gaatataaca aatttctcca agaaagagga agcatttttt gttatatcaa
      121 tgacgccata gaaaattggt acgagaattg tccaaaaatg cagggcggca actatattta
      181 tagtgataaa gttgtgattt tggtgcatat aatcgtcagt ttctttagaa ttggtttaag
      241 acaaacagtc gggtttataa aagggtattt gcaacaaata ggaagagatt tggcagttat
      301 cagctattca caagcatcaa gaaggtttaa gaaacttaat attaagataa atgattgcag
      361 aattgataaa aatatagctc tttccataaa agtccggatg tgataggata agggtagatg
      421 atgataagga ggttgaatgc cagcagcata tagttatgat ttaagaaaaa aagcaatgga
      481 agcgctagac gaaggagaaa gcagagaaac agtggcagca agatttaaga ttggacgaac
      541 tactttatgg gagtggcagc aaagaagaaa agaaacagga gactttcaat caaaaaaact
      601 tggaaatggg ggttacaatc acaaaattac cgactgggat acctttgcca aatttgccag
      661 agaacacgga ggaaagactc tatcggaaat ggctaaactt tggaacaacg ttagtatcca
      721 aacgatctat cgagccctta aaaaaattgg atttacgcgc aaaaaaagac atatggatac
      781 aaggaaagaa acgaagaaaa acgtgctgaa tttttgaaaa ttatagcgac aaaagaacct
      841 aaaaacttag tgtatataga tgagtctggc attgataaca ccgaagacta cccctacgga
      901 tattgccaga aaggacagcg gttttatgcc ttaaaatctg gaaagaaaac tcaacgaatc
      961 agtatggttg cagccttaag tggaagaaag atagttgttc cgttaacctt tgaaggccac
     1021 tgtaatatgg atgtttttaa tggatgggtt gagcaatttt tgacaccgat cttggaacct
     1081 ggacagactg accttaccca gaaaaagtgg agagaaagaa aggagttttt ttgggtaaaa
     1141 taaaattttg gaggattaga aatggcaaga gaatatacgg cagaattcaa attggaagct
     1201 gttaagctga caaatgaaca aagaaaggta ggtcaaccag ttgcaaaagt agcaagagat
     1261 ctaggaataa gggatagtgt attaggaaaa tggatgaaga aatataacga aaaaagtcag
     1321 cggcaaatgc atttccagat gtagcacctt atgacaaaga gagatttgat ttacaaagca
     1381 aaagtaacaa gagaaagaga cattttaaaa aaagccctgg ccagtcaaaa agagtaaaat
     1441 attttttat atagtaactg ctataaagta caggaattat gtaggatttt ctgctagtgg
     1501 ctactataag tggaccacaa gaaaaataag cagtagagaa tcagcaaata aagagctact
     1561 cgcagctatt caaaaaatat atcaagtttc taaatgtaga tatggagctc ctaaaattca
```

```
     1621 tgctgaattg aaggctttgg gtaaaagttg caacttaaaa acagtgcaaa gtattatgca
     1681 gaaaaatggt attcaggcta tattgagaag aaaatttaaa attaagaaac aacaaacgga
     1741 cagtagctcc caatatatta gaccaaaact ttattgtcga tcaaccaaat aaagtatggg
     1801 attacttata taaaaaccaa agaaggatgg ctatatttgg caacaataat cgatctatat
     1861 tcacgcatgg tagttaatga gcagttcaat aaataaacaa ttggttatgg actctttatt
     1921 aatggccgtt aacaagcgta aacctgccaa aaatctacta ttacatagtg atcaaggttc
     1981 acaatatact tcgcaaggtt atcaatatct cttatccata aaaaacatag atgagtcata
     2041 aaggttgttg ttacgacaat tctgttgtgg aaagcttctt tagctcattg aaaagagaaa
     2101 tacttattga tacttcacaa cactctgctc aacaaactag aactgcaata tttgaataca
     2161 tagaaatttt ttataacaaa caacgtcact attaactatt gcattcctga gcattttgat
     2221 tcatcattct ctttgtgaaa aacattccaa actttctctc cactttttct gggtaaggtc
     2281 ag
//
LOCUS       JAQZAU010000016            1465 bp    dna     linear   ENV 17-MAR-2023
DEFINITION  MAG: Wolbachia pipientis isolate wEsol contig_16892, whole genome
            shotgun sequence.
ACCESSION   JAQZAU010000016  JAQZAU010000000
VERSION     JAQZAU010000016.1
DBLINK      BioProject: PRJNA929258
            BioSample: SAMN33142973
KEYWORDS    WGS; ENV; Metagenome Assembled Genome; MAG.
SOURCE      Wolbachia pipientis (insect metagenome)
  ORGANISM  Wolbachia pipientis
            Bacteria; Pseudomonadota; Alphaproteobacteria; Rickettsiales;
            Anaplasmataceae; Wolbachieae; Wolbachia.
REFERENCE   1  (bases 1 to 1465)
  AUTHORS   Fiutek,N., Couger,B.M., Roy,S., de la Torre,J.R. and Connor,E.F.
  TITLE     Genomic assessment of the contribution of the Wolbachia
            endosymbiont of Eurosta solidaginis to gall-induction
  JOURNAL   International Journal Molecular Sciences
REFERENCE   2  (bases 1 to 1465)
  AUTHORS   Fiutek,N., Couger,B.M., Roy,S., de la Torre,J.R. and Connor,E.F.
  TITLE     Direct Submission
  JOURNAL   Submitted (10-FEB-2023) Biology, San Francisco State University,
            1600 Holloway Ave, San Francisco, CA 94132, USA
COMMENT     ##Genome-Assembly-Data-START##
            Assembly Date          :: JUN-2019
            Assembly Method        :: FLYE v. 06-2019
            Genome Representation  :: Full
            Expected Final Version :: Yes
            Genome Coverage        :: 100.0x
            Sequencing Technology  :: Illumina HiSeq; PacBio Sequel
            ##Genome-Assembly-Data-END##
FEATURES             Location/Qualifiers
     source          1..1465
                     /organism="Wolbachia pipientis"
                     /mol_type="genomic DNA"
                     /submitter_seqid="contig_16892"
                     /isolate="wEsol"
                     /isolation_source="galls on Solidago altissima"
                     /host="Eurosta solidaginis MN-2018-EFC"
                     /db_xref="taxon:955"
                     /environmental_sample
                     /country="USA: Cowling Arboretum, Carleton College,
                     Northfield, MN"
                     /lat_lon="44.48 N 93.13 W"
                     /altitude="280 m"
                     /collection_date="2018-11"
                     /metagenome_source="insect metagenome"
                     /note="metagenomic"
```

```
     gene            <1..108
                     /locus_tag="wEsol_00143"
     CDS             <1..108
                     /locus_tag="wEsol_00143"
                     /codon_start=1
                     /transl_table=11
                     /product="hypothetical protein"
                     /translation="TNILSSIREGRPLTGRDGALTPFIKKLLEVSLEGE"
                     /besthit="qcoverage 100 , hcoverage 43.2098765432099 ,
                     similarity 100 , identity 100 , evalue 3.42e-15 ,
                     alnlength 35 , Eukaryota , Arthropoda , GFQ64109.1
                     mutator family transposase [Trichonephila clavata]"
     gene            101..439
                     /locus_tag="wEsol_00144"
     CDS             101..439
                     /locus_tag="wEsol_00144"
                     /codon_start=1
                     /transl_table=11
                     /product="hypothetical protein"
                     /pfam="Transposase_mut Pfam-B_11109"
                     /translation="MNDRRNGRNAKTLRTSAGSFELLTPRDREGSFEPQIVKKRQTNL
                     HPELETKILSTFASGMGYRDIVSHVEEIYDHKISAAEISSITDKLLPVINEWRSRPLQ
                     SVYPIVFMVF"
                     /cog="COG3328 COG3328 379 Transposase and inactivated
                     derivatives"
                     /besthit="qcoverage 99.1071428571429 , hcoverage
                     63.0681818181818 , similarity 98.2 , identity 94.6 ,
                     evalue 5.55e-68 , alnlength 111 , Eukaryota , Arthropoda
                     , GFQ83888.1 mutator family transposase [Trichonephila
                     clavata]"
     gene            474..1073
                     /locus_tag="wEsol_00145"
     CDS             474..1073
                     /locus_tag="wEsol_00145"
                     /codon_start=1
                     /transl_table=11
                     /fullproduct="qcoverage 96.9849246231156 , hcoverage
                     87.3303167420815 , similarity 99.5 , identity 99.0 ,
                     evalue 2.98e-135 , alnlength 193 , Eukaryota , Arthropoda
                     , CAD7417219.1 unnamed protein product [Timema
                     poppensis]"
                     /cog="COG3328 COG3328 379 Transposase and inactivated
                     derivatives"
                     /pfam="Transposase_mut MULE DDE_Tnp_IS240 DDE_Tnp_ISL3
                     DDE_Tnp_ISL3 DDE_Tnp_IS240"
                     /product="IS256 family transposase"
                     /translation="MYNILGIDQNGKKEVLGFYLAESEGANFWLGVLNDLKERGVEDI
                     LIACVDGLKSFPAAINSVFPNAEVQMCIVHQIRNSLKYMASKDAKSFLSDLKKVYQAS
                     SKEIAENYLLELDEKWGTKYPMVIKSWQNNWENLSGYFKYSGPVRRIIYTTNPIEGLH
                     RQIRKFTKTKGSFTSINALYKQVYCAIKKAEGKVDDACT"
                     /besthit="qcoverage 96.9849246231156 , hcoverage
                     87.3303167420815 , similarity 99.5 , identity 99.0 ,
                     evalue 2.98e-135 , alnlength 193 , Eukaryota , Arthropoda
                     , CAD7417219.1 unnamed protein product [Timema
                     poppensis]"
     gene            complement(1190..>1465)
                     /locus_tag="wEsol_00146"
     CDS             complement(1190..>1465)
                     /locus_tag="wEsol_00146"
                     /codon_start=1
```

```
                     /transl_table=11
                     /product="hypothetical protein"
                     /translation="ATGGNNTDKETHFSHQLEQKTRQEGINASASATVYTDREIPTSN
                     QSEQKVQQEIINTSTSAPVLQIHIDKEIPSSSARTKNTTKRNTKKIN"
ORIGIN
        1 acaaatatcc tgtcgtctat acgagaagga agaccgttga caggaagaga tggagcatta
       61 acaccgttta taaaaaagtt gctggaggta agcctggaag gtgaatgatc gaagaaatgg
      121 gaggaatgca aagactttac gtacaagcgc agggtcattt gagcttttga cgccaagaga
      181 tagggaagga agtttcgaac cacaaatagt caaaaaaagg caaacaaact tacatccaga
      241 gcttgaaacg aagattttga gcacatttgc cagtggtatg ggctatagag atatagtgtc
      301 acatgttgag gaaatttatg atcacaaaat atcggcggca gagatatcaa gtattactga
      361 caaattgcta ccagtaatca acgaatggcg tagtcgcccg ctgcagtctg tatatccgat
      421 agttttcatg gtttttaaa gtcaaagagg acgggcattg cgtaagtaaa tgcatgtata
      481 acatattagg catagatcaa aatggcaaaa aagaagtatt aggtttttat ttggctgaaa
      541 gtgaaggagc taacttctgg ttgggagtgc taaatgacct caaagaaaga ggagtagaag
      601 atatccttat tgcctgcgtt gatgggctga aaagctttcc tgcagctata aacagtgtct
      661 ttcctaatgc ggaagtgcag atgtgtatag tgcatcagat acgcaactca ctgaagtata
      721 tggccagtaa agatgcgaag agttttttga gcgatttaaa gaaggtatat caagcttcaa
      781 gcaaggaaat tgccgagaat tatctgttgg aattggatga aaaatggggc acaaagtacc
      841 ctatggttat aaaatcttgg cagaataatt gggaaaactt gtctggttat tttaagtatt
      901 ctggtccagt taggaggata atctacacca ccaatcccat agaggggcta cataggcaaa
      961 ttaggaaatt cactaagacc aaaggctcat ttaccagcat aaatgccttg tataagcagg
     1021 tatattgtgc tataaagaag gcagagggaa aagtggacga tgcctgtaca tgattgggca
     1081 ttaactatct ctcaacttga tgttttcttt cctggcagat tgaaaattga gttgaactaa
     1141 aaatgctatt tgacacagtt tatttaacac tccctctttc ttgtaattat taattaattt
     1201 tttttgtatt tctttttgtt gtgtttttttg ttcttgctga tgaggaaggg atttctttat
     1261 ctatgtgaat ttgtaaaacc ggcgcactag tactggtatt aattatttct tgttgtactt
     1321 tttgctctga ttgattagaa gtaggaattt ctctatccgt ataaacagtc gcactagcgc
     1381 tggcattaat tccttcttgt cgtgtttttt gctctagttg atgagaaaaa tgggtttctt
     1441 tatccgtatt gttacccccg gttgc
//
LOCUS       JAQZAU010000017        10077 bp    dna     linear   ENV 17-MAR-2023
DEFINITION  MAG: Wolbachia pipientis isolate wEsol contig_17836, whole genome
            shotgun sequence.
ACCESSION   JAQZAU010000017  JAQZAU010000000
VERSION     JAQZAU010000017.1
DBLINK      BioProject: PRJNA929258
            BioSample: SAMN33142973
KEYWORDS    WGS; ENV; Metagenome Assembled Genome; MAG.
SOURCE      Wolbachia pipientis (insect metagenome)
  ORGANISM  Wolbachia pipientis
            Bacteria; Pseudomonadota; Alphaproteobacteria; Rickettsiales;
            Anaplasmataceae; Wolbachieae; Wolbachia.
REFERENCE   1  (bases 1 to 10077)
  AUTHORS   Fiutek,N., Couger,B.M., Roy,S., de la Torre,J.R. and Connor,E.F.
  TITLE     Genomic assessment of the contribution of the Wolbachia
            endosymbiont of Eurosta solidaginis to gall-induction
  JOURNAL   International Journal Molecular Sciences
REFERENCE   2  (bases 1 to 10077)
  AUTHORS   Fiutek,N., Couger,B.M., Roy,S., de la Torre,J.R. and Connor,E.F.
  TITLE     Direct Submission
  JOURNAL   Submitted (10-FEB-2023) Biology, San Francisco State University,
            1600 Holloway Ave, San Francisco, CA 94132, USA
COMMENT     ##Genome-Assembly-Data-START##
            Assembly Date          :: JUN-2019
            Assembly Method        :: FLYE v. 06-2019
            Genome Representation  :: Full
            Expected Final Version :: Yes
            Genome Coverage        :: 100.0x
            Sequencing Technology  :: Illumina HiSeq; PacBio Sequel
```

```
                ##Genome-Assembly-Data-END##
FEATURES             Location/Qualifiers
     source          1..10077
                     /organism="Wolbachia pipientis"
                     /mol_type="genomic DNA"
                     /submitter_seqid="contig_17836"
                     /isolate="wEsol"
                     /isolation_source="galls on Solidago altissima"
                     /host="Eurosta solidaginis MN-2018-EFC"
                     /db_xref="taxon:955"
                     /environmental_sample
                     /country="USA: Cowling Arboretum, Carleton College,
                     Northfield, MN"
                     /lat_lon="44.48 N 93.13 W"
                     /altitude="280 m"
                     /collection_date="2018-11"
                     /metagenome_source="insect metagenome"
                     /note="metagenomic"
     gene            complement(404..1003)
                     /locus_tag="wEsol_00147"
     CDS             complement(404..1003)
                     /locus_tag="wEsol_00147"
                     /codon_start=1
                     /transl_table=11
                     /pfam="OMP_b-brl"
                     /fullproduct="qcoverage 100 , hcoverage 99.0049751243781
                     , similarity 100 , identity 100 , evalue 1.52e-137 ,
                     alnlength 199 , Bacteria , Proteobacteria ,
                     WP_015588785.1 MULTISPECIES: OmpA-like protein
                     [unclassified Wolbachia]"
                     /translation="MKRLCALAVLLTSSLAGTANNSASKSEDQYYAGLNFGAGWGGGL
                     KIKPSVVFGYHHDKNSKFELEVLTDISDMFGKEEKKMGASLLANYRYYPNLDIDPVKL
                     YVSGGLGGYLQVIPFGFGNSGTSEPASKDIEAAGEKASVVDKILGSISYKLKVGVDYE
                     IAPQIVGTVGITAGGQLSGFKPPVQIPDAILEVGIRYNF"
                     /product="OmpA-like protein"
                     /besthit="qcoverage 100 , hcoverage 99.0049751243781 ,
                     similarity 100 , identity 100 , evalue 1.52e-137 ,
                     alnlength 199 , Bacteria , Proteobacteria ,
                     WP_015588785.1 MULTISPECIES: OmpA-like protein
                     [unclassified Wolbachia]"
     gene            complement(1128..3578)
                     /locus_tag="wEsol_00148"
     CDS             complement(1128..3578)
                     /locus_tag="wEsol_00148"
                     /codon_start=1
                     /transl_table=11
                     /fullproduct="qcoverage 100 , hcoverage 100 , similarity
                     100 , identity 100 , evalue 0.0 , alnlength 816 ,
                     Bacteria , Proteobacteria , WP_141456805.1 MULTISPECIES:
                     leucine--tRNA ligase [unclassified Wolbachia]"
                     /cog="LeuS COG0495 814 Leucyl-tRNA synthetase"
                     /pfam="tRNA-synt_1d tRNA-synt_1 tRNA-synt_1f tRNA-synt_1g
                     tRNA-synt_1e tRNA-synt_1 tRNA-synt_1_2 tRNA-synt_1
                     tRNA-synt_1 tRNA-synt_1 tRNA-synt_1g Anticodon_1"
                     /tigr="TIGR00396 leuS_bact 843 leucine--tRNA ligase"
                     /product="leucine--tRNA ligase"
                     /translation="MKYDFKSVEKFYQDKWDFSVSKSGKQEKCYVLEMFPYPSGKIHM
                     GHLRNYAIGDVIARYKRANGFEVLHPIGWDAFGLPAENAARDNNISPETWTKENIDNM
                     RTQLKSIGLSYNWERELSTCEPDYYKHEQKFFLDFLKHGLAYRKESWVNWDPVDQTVL
                     ANEQVVDGKGWRSGAVVEKRKLSQWFLKITDFAEDLLKCLQSLKNWPEKVKTMQERWI
```

```
                     GKSEGVTIDFEIVGLNKKLKIFTTSPHTWFGASFFAVAAEHPIVQDLKDKEIRDFIDG
                     MKAKEENDEKIGVYTGLNVKHPFLDKELPLYIANFVLMEYGEGAIFGCPAHDQRDFEF
                     AQKYDLPVIPVICEKGAQAIVTLEEAYTGGEIMCNSEFLNGLTVSEAKEVIIKKLKEK
                     GIGEKTTNYRLHDWGISRQRYWGCPIPIIYCRDCGTVPVPEKDLPVVLPTDVEFTSGG
                     NPLDKHPTWKFVDCPKCGKQAERETDTFDTFFESSWYFAAFCSEDKSIDKDACNRFMP
                     VDYYIGGIEHAILHLLYSRFFCRALTKCGYFDIKEPFSTLITQGMVCHATYKDENGKW
                     LFPAEAKELIAQGAKVQVGKVEKMSKSKKNTVDPNFIIEKYGADTARLFVLSDTPPEK
                     DMEWSDDGVEGCSRYVNKLWRMVMQLKPVNMHYDNKSVTGGLLEYRKKIHKLLHGLTD
                     DLENCRLNCVVAKFREMTNLIAEIDVAAGKSLINEGICILIRVIEPFMPHLAESLWQE
                     IGGQPWPKADESLLVDDTVTIAVQINGKLRATIEVAINLPQEELKKIAIDSVSSKIDQ
                     NKVRTVYAVPNKIVNIVI"
                     /besthit="qcoverage 100 , hcoverage 100 , similarity 100
                     , identity 100 , evalue 0.0 , alnlength 816 , Bacteria ,
                     Proteobacteria , WP_141456805.1 MULTISPECIES:
                     leucine--tRNA ligase [unclassified Wolbachia]"
     gene            complement(3591..4187)
                     /locus_tag="wEsol_00149"
     CDS             complement(3591..4187)
                     /locus_tag="wEsol_00149"
                     /codon_start=1
                     /transl_table=11
                     /pfam="Peptidase_M23 RnfC_N"
                     /fullproduct="qcoverage 100 , hcoverage 100 , similarity
                     100 , identity 100 , evalue 2.02e-141 , alnlength 198 ,
                     Bacteria , Proteobacteria , WP_006279879.1 MULTISPECIES:
                     M23 family metallopeptidase [Wolbachia]"
                     /translation="MWHIASFLMLLAILIGCGLQKPAPVLLKGEEFYGKRDLEYTREY
                     HLIREPSVQKESRKAIINRIYKDNNNTQNVGVNCKFVMPVKGSATSSDEMCKDGIKIA
                     AQNGTNVVSSAPGKVIYVGKGLRWYGNLIIVEHKDNYMTVYSYLKNIHVEIGDKVKQG
                     QVIGSAGKSSTQDKDPQMCFTIRHNGQAVDPLMHMNCN"
                     /product="M23 family metallopeptidase"
                     /besthit="qcoverage 100 , hcoverage 100 , similarity 100
                     , identity 100 , evalue 2.02e-141 , alnlength 198 ,
                     Bacteria , Proteobacteria , WP_006279879.1 MULTISPECIES:
                     M23 family metallopeptidase [Wolbachia]"
     gene            complement(4233..4559)
                     /locus_tag="wEsol_00150"
     CDS             complement(4233..4559)
                     /locus_tag="wEsol_00150"
                     /codon_start=1
                     /transl_table=11
                     /pfam="HTH_17 MerR_1 MerR"
                     /fullproduct="qcoverage 100 , hcoverage 100 , similarity
                     100 , identity 100 , evalue 2.52e-69 , alnlength 108 ,
                     Bacteria , Proteobacteria , WP_006279880.1 MULTISPECIES:
                     MerR family transcriptional regulator [Wolbachia]"
                     /translation="MNKEKLFYTIGEVAEELHLEQHVLRFWEGQFHQIKPTKRKGRRL
                     YDRKCIEAIKKVKYMLYDKGYTIKGVQKEFGNDIKVTKNLLQELTDLRDYLTSKINNE
                     ESNKQA"
                     /product="MerR family transcriptional regulator"
                     /besthit="qcoverage 100 , hcoverage 100 , similarity 100
                     , identity 100 , evalue 2.52e-69 , alnlength 108 ,
                     Bacteria , Proteobacteria , WP_006279880.1 MULTISPECIES:
                     MerR family transcriptional regulator [Wolbachia]"
     gene            complement(4559..4864)
                     /locus_tag="wEsol_00151"
     CDS             complement(4559..4864)
                     /locus_tag="wEsol_00151"
                     /codon_start=1
                     /transl_table=11
```

```
                     /fullproduct="qcoverage 100 , hcoverage 100 , similarity
                     100 , identity 100 , evalue 2.51e-59 , alnlength 101 ,
                     Bacteria , Proteobacteria , WP_006279881.1 MULTISPECIES:
                     integration host factor subunit alpha [Wolbachia]"
                     /cog="HimA COG0776 94 Bacterial nucleoid DNA-binding
                     protein"
                     /pfam="Pfam-B_95 Bac_DNA_binding"
                     /tigr="TIGR00987 himA 96 integration host factor, alpha
                     subunit"
                     /product="integration host factor subunit alpha"
                     /translation="MDHVTTKDTTVTKATIAGDINQEIGLSKEDSVSIIDDILDEIKT
                     SLVKDGIVKISSFGTFLVKKKKERPGNIPNTSEKVMIQARNSVSFRPSKIIKKSINN"
                     /besthit="qcoverage 100 , hcoverage 100 , similarity 100
                     , identity 100 , evalue 2.51e-59 , alnlength 101 ,
                     Bacteria , Proteobacteria , WP_006279881.1 MULTISPECIES:
                     integration host factor subunit alpha [Wolbachia]"
     gene            complement(4925..6103)
                     /locus_tag="wEsol_00152"
     CDS             complement(4925..6103)
                     /locus_tag="wEsol_00152"
                     /codon_start=1
                     /transl_table=11
                     /fullproduct="qcoverage 100 , hcoverage 100 , similarity
                     99.7 , identity 99.7 , evalue 6.97e-262 , alnlength 392 ,
                     Bacteria , Proteobacteria , WP_155968857.1 MULTISPECIES:
                     MFS transporter [Wolbachia]"
                     /pfam="MFS_1 MFS_1_like"
                     /tigr="TIGR00886 2A0108 368 nitrite transporter"
                     /product="MFS transporter"
                     /translation="MESRAKAWFIWLISNLVVIFSNMQIIYTFISVNLEKELGLTIAQ
                     VALANSAYTWTFAILQFFSGATFNVFSSKKIYFFSLSTMILGFFVLINSDNFSHLILS
                     QVLIATGASFGFIGAAHTSSICFSAAQFGLMFSLVQTISSLSALVIQILFSNLLAEDA
                     YWKNLIVCIILFGVLIFVLTLFCPNTLPESSSEKRTIKSSMKTVMCSILTVLKLRDIW
                     ITSVVGAITFGTFLALNTLWAPRLLGNSELNAMESGIATAILWLGLAVGAPIADRISN
                     LFKNRKHVISAFALLQGVSIIIILLYSHLTVHVVYFCMLMFGFFAGGHMLNFTVGSEIV
                     KRKYISTSSSIINGFMFIGSGVIVSMLALFTDHKMALFAILIVAGILNYATKETYSKK
                     "
                     /besthit="qcoverage 100 , hcoverage 100 , similarity 99.7
                     , identity 99.7 , evalue 6.97e-262 , alnlength 392 ,
                     Bacteria , Proteobacteria , WP_155968857.1 MULTISPECIES:
                     MFS transporter [Wolbachia]"
     gene            complement(6475..8013)
                     /locus_tag="wEsol_00153"
     CDS             complement(6475..8013)
                     /locus_tag="wEsol_00153"
                     /codon_start=1
                     /transl_table=11
                     /fullproduct="qcoverage 100 , hcoverage 100 , similarity
                     100 , identity 100 , evalue 0.0 , alnlength 512 ,
                     Bacteria , Proteobacteria , AGJ99536.1 Putative cytosol
                     aminopeptidase [Wolbachia endosymbiont of Drosophila
                     simulans wHa]"
                     /cog="PepB COG0260 485 Leucyl aminopeptidase"
                     /pfam="Peptidase_M17_N Peptidase_M17"
                     /product="Putative cytosol aminopeptidase"
                     /translation="MKNILLMFLLCFMYSLQLFATEIPAMKITISKISPDFKTIVMGL
                     FEDNETVNDGGVLQGKQVIDNIKQFSDFNGSFGEFFSTALPEEKNVIVVGLGKKDEWN
                     ENKELNIGGKIYCELSRLKIKKAAVLIEGSAANVAYGAFLRSFKFDKYKTKKDEKITE
                     VEEITVLVKDEQLSNAERSFEHLRQEGESIFLARSFITEPPNILYPESYADHIKKELT
                     KLGLEIEVLDKKQMEEKKMGALLGVAQGSSKEPKLVVIKWNGASKEQKPIAFVGKGIT
```

```
                     FDTGGVSLKPSRGMESMKYDMAGSATVVGVMHALAGRKAKVNAIGVVALAENAVGGNA
                     QRPSDVVTSMSGQTIEVLNTDAEGRLILADALWYTQDRFSPKFMIDLATLTGAIVVAL
                     GNNEYAGLFSNNDELANRLIDAGNEVNEKLWRFPMNETYDKIIDSPIADVQNIAPAGS
                     GGDSIMAAQFLQRFVNETCWAHLDIAGTAWHEKGTDICPRGAVGFGVRLLNKLVEKYY
                     EAND"
                     /besthit="qcoverage 100 , hcoverage 100 , similarity 100
                     , identity 100 , evalue 0.0 , alnlength 512 , Bacteria ,
                     Proteobacteria , AGJ99536.1 Putative cytosol
                     aminopeptidase [Wolbachia endosymbiont of Drosophila
                     simulans wHa]"
     gene            complement(8074..8715)
                     /locus_tag="wEsol_00154"
     CDS             complement(8074..8715)
                     /locus_tag="wEsol_00154"
                     /codon_start=1
                     /transl_table=11
                     /fullproduct="qcoverage 100 , hcoverage 100 , similarity
                     100 , identity 100 , evalue 5.98e-155 , alnlength 213 ,
                     Bacteria , Proteobacteria , WP_015588781.1 MULTISPECIES:
                     protocatechuate 3,4-dioxygenase [unclassified Wolbachia]"
                     /cog="PcaH COG3485 226 Protocatechuate 3,4-dioxygenase
                     beta subunit"
                     /pfam="Dioxygenase_C"
                     /product="protocatechuate 3,4-dioxygenase"
                     /translation="MNVKNILLAFLVQMVFSLPLFAADPVLLNCIETPEIYDLDARPK
                     NFNSSNNLRRKPGSPNSAIGELIHIVGRITDINCLPIQNAVVSIWHANSRGVNHYDKN
                     IEDDLNFAGSGRFVVNNLGYYNFITIAPGKIGDRAPHINFLVQHPDFPEFTTQMFFAD
                     HNCDNCADPVLEDFVSNGLASLLITPFTYSDQVIKTYTFNITLGGYNKFSNKR"
                     /besthit="qcoverage 100 , hcoverage 100 , similarity 100
                     , identity 100 , evalue 5.98e-155 , alnlength 213 ,
                     Bacteria , Proteobacteria , WP_015588781.1 MULTISPECIES:
                     protocatechuate 3,4-dioxygenase [unclassified Wolbachia]"
     gene            9146..9574
                     /locus_tag="wEsol_00155"
     CDS             9146..9574
                     /locus_tag="wEsol_00155"
                     /codon_start=1
                     /transl_table=11
                     /pfam="Pfam-B_3816 DUF972 Pfam-B_4504 Pfam-B_3314 DivIC
                     HALZ Pfam-B_1653 Pfam-B_3883 Pfam-B_17409"
                     /fullproduct="qcoverage 100 , hcoverage 100 , similarity
                     99.3 , identity 98.6 , evalue 6.00e-75 , alnlength 142 ,
                     Bacteria , Proteobacteria , QEF50519.1 putative
                     transposase [Wolbachia endosymbiont of Drosophila
                     ananassae]"
                     /translation="MVNLLELCKNLQQKIEKLKAEIENLKAENKALKIENAELKERLG
                     LNSKNSSLPSSKELYKTKKDKPKSERNVGDQVGYKGNFHATMEADKVVKIELPNICEC
                     GGELVICEKPYVHQKVDLPEIRPYVVIGTWSLLEMQKKKK"
                     /product="putative transposase"
                     /besthit="qcoverage 100 , hcoverage 100 , similarity 99.3
                     , identity 98.6 , evalue 6.00e-75 , alnlength 142 ,
                     Bacteria , Proteobacteria , QEF50519.1 putative
                     transposase [Wolbachia endosymbiont of Drosophila
                     ananassae]"
     gene            9531..9821
                     /locus_tag="wEsol_00156"
     CDS             9531..9821
                     /locus_tag="wEsol_00156"
                     /codon_start=1
                     /transl_table=11
```

```
                     /fullproduct="qcoverage 100 , hcoverage 100 , similarity
                     97.9 , identity 95.8 , evalue 3.40e-57 , alnlength 96 ,
                     Bacteria , Proteobacteria , RLT63249.1 putative
                     transposase [Wolbachia endosymbiont of Drosophila
                     ananassae]"
                     /translation="MEHGRCWRCRKRKSSKLPEGVTPDTFGPRVKSIIAALSGFYKNS
                     KREVASIINDIFNLNISVGSISNLTITHKYEVREQVAILLLDQRNVSTLSKE"
                     /product="putative transposase"
                     /besthit="qcoverage 100 , hcoverage 100 , similarity 97.9
                     , identity 95.8 , evalue 3.40e-57 , alnlength 96 ,
                     Bacteria , Proteobacteria , RLT63249.1 putative
                     transposase [Wolbachia endosymbiont of Drosophila
                     ananassae]"
     gene            complement(9869..>10077)
                     /locus_tag="wEsol_00157"
     CDS             complement(9869..>10077)
                     /locus_tag="wEsol_00157"
                     /codon_start=1
                     /transl_table=11
                     /product="hypothetical protein"
                     /translation="GTAERLMRYLTVMSIIAWRIFFITSIARTNPTLPCTALLAEEEW
                     KVLYVKIHRKPCSNIAPTIKEAVS"
                     /besthit="qcoverage 100 , hcoverage 74.7252747252747 ,
                     similarity 97.1 , identity 97.1 , evalue 5.28e-39 ,
                     alnlength 68 , Bacteria , Proteobacteria , CDR78440.1
                     hypothetical protein WPAU_0041 [Wolbachia endosymbiont of
                     Drosophila simulans wAu]"
ORIGIN
        1 ggattcatat tttttgcctt gaccaagaaa aaattcagct attgccttac tttctcttgc
       61 aacaacttct tccttaacaa tttttccttt ctcatcaacg atacttataa aagtttcttt
      121 tagtgagatc gctataatat tccatgagac tgctcctact aataaaagtt taaattattt
      181 ttgaagaact tattctactg aattcgttac ttcgtgctaa aataatggac tccatcattc
      241 aacagcaatt acagtatgtt taccctttt cttgttattt ttctaatcta tagtcagcgt
      301 tcaaaaccat attcgaaaag tatctcatat atttccagat ttttttaatg ctgtttattt
      361 catcctcaac attgcctcta cctttatgaa ctaagtagca gcattaaaaa ttataacgta
      421 ttcctacttc tagaatcgcg tccggtattt gtactggtgg tttaaagcca cttagttgcc
      481 cgcctgcggt aataccaact gtaccaacta tctgtggagc aatttcataa tcaacaccga
      541 cctttagttt ataggaaata gaacccagta ttttatctac tacagatgcc ttctctccag
      601 cagcttcaat atctttgctt gcaggttcac ttgtaccact gttaccaaag ccaaaaggta
      661 ttacttgtag gtatcctcct aaacctccgc taacatatag cttaacggga tcaatatcaa
      721 ggttagggta ataacggtaa tttgctaata agctagcccc cattttcttt tcttctttac
      781 caaacatatc acttatgtca gtgagaacct caagttcaaa cttagagttt ttgtcatgat
      841 gataaccaaa aacaacgcta ggttttattt ttaagccacc accccatcca gcaccaaagt
      901 ttaaacctgc ataatactga tcttctgact tacttgcact attattagct gtacctgcta
      961 gagatgaagt gagcaacaca gctaaagcac ataatctttt cattttcata taactcctca
     1021 tacaaaaaaa tatataatgc ataagtatat atcctttata ttaaaaaaga tataatcgca
     1081 tgttaaaaag agtataacca tgaagtcaaa gatgcgctac ggattttttta tataactata
     1141 tttactatct tatttggtac agcatataca gtgcgaactt tgttttgatc gatcttactg
     1201 gacacagagt ctatcgctat tttcttcaat tcttcttgag gtaagttaat tgccacttcg
     1261 attgtcgcac gcaactttcc attgatctgc actgctatag ttaccgtatc gtcaactaat
     1321 aatgattcat cagcttttgg ccaaggttga cctcctattt cttgccatag gctttcagct
     1381 aaatgcggca taaatggttc gattactctg attagtatgc atataccttc attaataaga
     1441 gattttccag ctgctacgtc tatttcagct attaaattcg tcatttcacg aaattttgcc
     1501 actacacagt ttaatctgca gttttccaag tcatctgtaa gtccatgtaa gagtttatga
     1561 atttttttc tgtactctag aagtccaccc gtaacacttt tgttatcata gtgcatattc
     1621 acaggcttaa gctgcatgac catacgccac aatttattta cataacgaga acaaccttcc
     1681 acaccatcat cggaccattc catatctttc tctggaggag tgtcagacaa cacaaacaag
     1741 cgggcagtat cagcaccata cttttctatg ataaaatttg gatcaactgt gttcttttc
     1801 gatttgctca ttttctcgac ttttccaact tgaaccttag ctccttgtgc aatcaactct
     1861 tttgcttctg cgggaaatag ccacttacca ttttcgtctt tataagttgc atggcagacc
```

1921 attccttgag tgattaaagt agagaaaggc tctttaatat caaaatagcc acatttggtt
1981 aaagcacggc agaaaaatcg tgagtaaagc aaatgcagaa ttgcatgctc tatcccacct
2041 atataataat caacaggcat aaaacgatta catgcatctt tgtctattga tttatcttca
2101 ctacagaatg cagcaaaata ccaggaagac tcaaaaaagg tgtcgaatgt atcagtttca
2161 cgctctgctt gctttccaca ttttgggcaa tcaacgaact tccaagttgg gtgtttatca
2221 agcggattac caccacttgt aaattccaca tctgttggta gaactacagg aaggtctttt
2281 tctggtactg gaacagttcc gcagtctctg caatatatga taggtatagg gcatccccaa
2341 taacgttgtc tcgaaattcc ccaatcatgt aaacgatagt ttgttgtttt ctcacctatc
2401 ccttttttctt tgagcttttt aatgattact tcctttgctt cactaaccgt caatccattt
2461 aagaattcag agttgcacat tatttcgccg ccagtgtagg cttcttctag agtcactatg
2521 gcttgtgcac cctttttcaca aataacagga ataaccggca aatcgtattt ctgtgcaaat
2581 tcaaaatcac gctgatcatg tgcagggcac ccaaaaatcg cgccttcccc atattccatc
2641 aacacaaaat tcgctatgta aagcggcaat tccttatcaa gaaatggatg cttgacgttt
2701 aatccggtgt aaactcctat tttttcgtca ttttcctcct ttgctttcat gccatcgata
2761 aagtctcgta tttctttgtc cttaagatcc tgcacaatag ggtgttctgc tgctaccgca
2821 aaaaaagaag ctccaaacca agtatgagga gaagttgtaa aaatctttaa tttcttattt
2881 aagccaacta tttcaaaatc tatagttacc ccttcagatt ttcctatcca acgctcctgc
2941 attgttttga ctttttctgg ccaatttttc aaactttgca gacacttgag taaatcttca
3001 gcgaaatcag taatctttaa aaaccactgg gataacttac gtttttcaac aacggcgcct
3061 gaccgccatc cttttccatc aaccacctgt tcatttgcaa gcactgtttg gtccactgga
3121 tcccagttaa cccatgattc ttttcgatag gcaagcccat gctttaaaaa atccaggaaa
3181 aatttttgtt cgtgtttgta ataatcaggc tcacatgtgg agagttcacg ctcccaatta
3241 taagaaagac ctatagattt caactgtgta cgcatattat ctatattctc ttttgtccat
3301 gtttcggggc taatattgtt atcccttgct gcattctcag ctggtaatcc aaacgcatcc
3361 cagccaattg gatgcaaaac ctcaaatcca ttagctctct tgtaacgcgc tatcacatcc
3421 cctattgcat agttacgtaa gtgtcccata tgaattttac cagatggata tggaaacatt
3481 tccaacacgt aacacttttc ttgtttacca ctcttgctta cagaaaaatc ccatttatct
3541 tgataaaatt tttcaacact tttaaaatca tatttcatat aaatccaaat tcaattacag
3601 ttcatatgca tcaaaggatc aaccgcttgg ccattatgtc gtattgtgaa acacatctgt
3661 ggatctttat cttgtgtgct tgatttacct gcagatccaa ttacttgacc ttgctttact
3721 ttatcgccaa tttcaacatg tatgtttttt aaataggagt acacagtcat gtaattatct
3781 ttgtgttcca ctataattaa attcccatac catctcagcc ctttgcctac atatatcact
3841 ttgcctggtg cagaggaaac tacgttcgtt ccattttgag cagcaatttt tatgccatcc
3901 ttacacattt catcagaaga ggtagctgaa cctttaactg gcattacaaa tttacaattt
3961 accccccacat tttgtgtatt attgttatct ttgtatatcc tatttatgat ggcttttcta
4021 ctttcttttt gcactgaagg ctccctaatt aaatggtact ctcttgtata ttccagatct
4081 ctttttccgt aaaattcttc gcctttaagc agcacaggtg caggtttttg caggccacag
4141 cctattaata ttgctaataa cataagaaat gaggctatat gccacatttt aaactctcac
4201 aaaactccaa aattttagcg agacgtagtg tttcacgcct gcttgttact ttcctcgtta
4261 tttattttac tagttaaata gtccctcaaa tcggtgagtt cttgcaacaa attctttgtg
4321 acttttatat cattaccaaa ttctttttgc actcctttta ttgtatatcc tttatcatat
4381 agcatgtatt ttactttctt tatggcctct atacacttgc gatcatatag ccttcttcct
4441 ttacgctttg taggcttaat ttgatgaaat tgaccttccc aaaatctcaa aacatgctgt
4501 tccaaatgta gctcttcagc tacttcccca atcgtataaa ataattttttc cttgttcatt
4561 aattattgat tgattttttt ataattttttg aaggcctaaa agaaactgaa tttcttgcct
4621 gaatcatcac tttctccgat gtatttggta tatttcctgg tctttccttc tttttcttaa
4681 ccaagaatgt tccaaatgac gatattttta ctattccatc ctttaccaag cttgtcttta
4741 tttcgtccaa tatatcatct attatcgaaa cagagtcttc ctttgataat cctatttctt
4801 ggtttatatc tccagctatc gttgccttag ttactgttgt gtcttttgtt gtaacgtgat
4861 ccattattga taaatattac aatacgtaaa tataatatag caatcgactc gagattcagc
4921 tactttattt tttagagtat gtctcttttg ttgcataatt taaaataccg gcaactatca
4981 atattgcaaa aagtgccatt ttatgatctg taaaaagtgc taacattgac actataactc
5041 cactgccaat aaacataaat ccattgataa tggatgatga tgtgctgatg tattttcgtt
5101 tcacaatctc gctgccgaca gtaaaattaa gcatatgtcc tcccgcaaaa aacccgaaca
5161 ttaacataca aaagtacaca acgtggactg ttaaatggct gtatagtaaa ataataattg
5221 agacgccttg caataaagca aaagcagaaa ttacatgttt cctattttta aataagtttg
5281 agattcgatc tgcaattgga gcaccaactg caagaccaag ccatagtatt gcagttgcta
5341 tacctgactc cattgcattg agttctgagt tgcccagtaa ccttggagcc cacaaggtat
5401 taagtgctaa aaatgttcca aaagtaatag cacccaccac tgaagttatc cagatatctc
5461 ttaattttag cactgttaat atagagcaca ttactgtctt catagagctt tttattgtgc

5521 gcttttctga gctgctttct ggaagtgtgt ttggacaaaa aagcgtaagt acaaatatca
5581 acacaccaaa tagtattatg catacaatca gatttttcca atatgcatct tcggcaagca
5641 agttagagaa cagtatttga attaccaaag cagaaaggct tgaaattgtc tgcactagtg
5701 aaaacattag tccaaattgg gcagcggaaa aacatatgct acttgtgtga gcagcaccaa
5761 taaaaccaaa tgatgctcca gttgcaatca acacttgaga caaaatcaaa tgggagaaat
5821 tgtcactatt aataaggaca aaaaaacccca agatcatagt tgataacgag aaaaaataaa
5881 ttttttttact ggaaaaaaca ttaaacgttg ctccactaaa aaattgtaag atggcgaaag
5941 tccaagtata tgctgaatta gccagtgcaa cttgcgcgat tgtaagtcca agttcctttt
6001 cgagatttac acttataaag gtataaatta tctgcatgtt gctgaatata acaaccaagt
6061 tactaatcag ccaaatgaac caagccttag ctctgctttc cataagattt atttttataa
6121 gtaatctatt agaaatttaa cactaagtct ctttttaagc tattgtttcc tgactactgt
6181 tatagctaac ccaaaaaacg gtttccctac gtcatgccgc cgcggtatct ctagatcccg
6241 ctaacaagca gcgggatgac gagcttatcg tcacgccgcc acgaaccgtc ataccgcgat
6301 tcattcgcgg tatctcagcc gctaacaagt agcgggatga cggttgtcag ggtgtcatga
6361 aagtagctga cactggaatc cagaaaaaaa gaaatatgta tcagctactt tcatttgctg
6421 cgcctcgaaa ataaacgttc atgtagctaa gccataaatc cagtataaag acctttaatc
6481 attggcttcg tagtacttct caaccaactt attaagtaac cttacaccaa aacctactgc
6541 tcctcttgga caaatgtcag tacctttctc gtgccaagca gtgcctgcga tatctaaatg
6601 tgcccagcaa gtttcattca caaaacgctg taaaaactgt gcagccatta tgctatctcc
6661 accagagcct gcaggagcga tgttttgaac atcagcaatc ggtgaatcaa taattttgtc
6721 ataagtttca ttcataggaa aacgccataa cttctcattt acttcatttc ctgcatcaat
6781 cagacggttt gctaattcat cattatttga aaaaagacca gcatattcgt tatttccaag
6841 tgcaaccact atagcaccag ttaaagttgc aagatcaatc ataaattttg gtgagaatct
6901 atcttgcgta taccataaag catctgcaag tatgagcctt ccttctgcat cggtgttcaa
6961 cacttctatt gtttgtccag acattgaagt tactacatca cttggtcttt gagcattacc
7021 acccactgca ttttctgcaa gtgcgaccac gccaattgca tttacttttg cttttcgtcc
7081 tgctaaagca tgcatcaccc caaccacagt agcagagcct gccatgtcat atttcatcga
7141 ctccatacca cgtgaaggtt tgagtgatac tccaccagtg tcaaacgtta tacctttacc
7201 aacaaaagct ataggctttt gttctttaga agctccattc cattttatca ctactaattt
7261 tggttcttta ctacttcctt gtgcaactcc aagcaaggct cccatttttt tctcttccat
7321 ctgcttttta tcaagcacct cgatttcaag gccaagctta gtaagttctt tttttatatg
7381 atcagcatag gattctggat atagaatgtt aggaggctct gttataaaag agcgcgcaag
7441 aaatatactc tcaccttctt gccttaagtg ctcaaatgat ctttcagcat tacttaattg
7501 ttcatctttt actaatacgg taatctcctc tacctctgta attttctcat ccttttagt
7561 tttatactta tcaaacttaa aactacgcag aaacgcacca tatgcaacat ttgctgcact
7621 accttcgatt aaaaccgctg ctttttttaat ttttaatctg cttagctcac aatatatttt
7681 accaccaata tttaattctt tattttcatt ccattcatcc ttcttaccca gtccaacaac
7741 tataacattt ttttcttctg gcaaagcagt agagaaaaat tcaccaaaac ttccattgaa
7801 atcgctaaat tgctttatat tatctatgac ctgttttcct tgcaaaactc cgccatcatt
7861 tacggtttcg ttgtcttcaa ataaacccat tactattgtt ttaaaatcgg gcgaaatttt
7921 agaaattgtt attttcattg ctggtatctc cgttgcaaat aattgtaaac tgtacataaa
7981 acatagcaaa aacatcagta agatattttt catatacatc ctaagtacaa taacacttaa
8041 gtttactatg gactccctta catgcaaggt tttttatctt ttattagaga acttattata
8101 tccgcccaag gtaatgttaa acgtataggt ttttatgacc tgatcactgt aagtaaatgg
8161 tgttatgaga aggcttgcaa gcccattact aacaaaatct tcaagaacag gatcagcgca
8221 attatcgcaa ttatggtcag caaaaaacat ttgcgttgtg aattctggaa aatccggatg
8281 ttgaactaga aagttgatat gtggagctct atcaccaatt ttaccaggtg ctatcgtaat
8341 aaaattataa tagccaagat tattcactac aaaccttcct gatccagcaa aattcagatc
8401 atcctctata ttcttatcgt aatggttcac gccgcgtgaa ttcgcgtgcc atatagaaac
8461 tacagcattt tgtattggta agcagtttat atcagtaatt ctacccacta tgtgtattaa
8521 ttcccctatt gcactatttg gagaaccagg ttttcttctt aaattgtttg aagagttaaa
8581 attttttggc cttgcatcaa ggtcatatat ttctggagtt tcaatgcagt tgagcaaaac
8641 aggatcagct gcaaatagcg gcaaactgaa taccatttgt actaaaaacg ctaataagat
8701 atttttcaca ttcatttcat ttataataga cttcttgcat aaccatataa caaactttta
8761 ttgggaatag aaacgaaaaa attactttac aaactccgcc agccctctta tcataaaact
8821 gaaggtattc agttatcttc atctgtgcag attaaacagc aagaaaacaa cgtagttggc
8881 gtcttatttt taattttttg cactatgtgc accttatgtc ttcacaatat ttctaggttt
8941 ttacctacac aagctgaaac gcgcttataa gtcgttcaag acagtatagt acgccaattt
9001 gcaggattag agagtgacaa ctagctaaca cggggtttct tttgcctttt tttctgttta
9061 gtaaattgta tccgttcagc ggagtggtag aataaggtag acaaagtgat ctaaaaagat

```
     9121 aatcatagag caaaagtgag gtaatgtggt aaacctttta gaactttgca aaaatttaca
     9181 gcaaaaaata gaaaaactga aagcagagat agaaaactta aaagcagaaa ataaagcttt
     9241 aaagatagag aacgctgaat taaaggaaag gcttggttta aactcgaaaa attcatctct
     9301 accgagctct aaagaattgt acaaaacaaa aaaggacaag ccaaaaagcg aaaggaatgt
     9361 tggcgatcag gttgggtata aaggaaattt tcatgccaca atggaagcgg ataaagtagt
     9421 aaaaatagag ttgcctaata tttgcgaatg cggaggagag cttgtaatat gcgaaaaacc
     9481 ttatgttcac caaaaagttg atcttccgga aattaggcct tatgtagtaa ttggaacatg
     9541 gtcgttgttg gagatgcaga aaaagaaaaa gtagcaagtt accagaaggt gttacgccag
     9601 atacatttgg tccaagggtt aagtctataa ttgcagcact cagcggattt tacaaaaact
     9661 caaagcgtga agtggctagt atcataaatg acatttttaa tttgaacata agtgttggca
     9721 gcatatcaaa tcttactatt acccacaaat atgaggttcg tgagcaagtc gccatccttc
     9781 ttctagatca aagaaacgtc tccacccttt ccaaagagta attggtcctg gttttgggtc
     9841 gctttttctt gctaaatgac ctccaagttc acgaaacggc ttcttttata gtaggggcta
     9901 tatttgaaca tggtttcctg tgtattttaa catataaaac tttccattct tcctcagcta
     9961 ataaggcagt acatggtaat gttgggttag ttcttgcaat tgatgtaata aagaaaattc
    10021 tccaagcaat aatgctcatg actgttaaat acctcattaa tctttctgct gttccaa
//
LOCUS       JAQZAU010000018             2900 bp    dna     linear   ENV 17-MAR-2023
DEFINITION  MAG: Wolbachia pipientis isolate wEsol contig_18195, whole genome
            shotgun sequence.
ACCESSION   JAQZAU010000018  JAQZAU010000000
VERSION     JAQZAU010000018.1
DBLINK      BioProject: PRJNA929258
            BioSample: SAMN33142973
KEYWORDS    WGS; ENV; Metagenome Assembled Genome; MAG.
SOURCE      Wolbachia pipientis (insect metagenome)
  ORGANISM  Wolbachia pipientis
            Bacteria; Pseudomonadota; Alphaproteobacteria; Rickettsiales;
            Anaplasmataceae; Wolbachieae; Wolbachia.
REFERENCE   1  (bases 1 to 2900)
  AUTHORS   Fiutek,N., Couger,B.M., Roy,S., de la Torre,J.R. and Connor,E.F.
  TITLE     Genomic assessment of the contribution of the Wolbachia
            endosymbiont of Eurosta solidaginis to gall-induction
  JOURNAL   International Journal Molecular Sciences
REFERENCE   2  (bases 1 to 2900)
  AUTHORS   Fiutek,N., Couger,B.M., Roy,S., de la Torre,J.R. and Connor,E.F.
  TITLE     Direct Submission
  JOURNAL   Submitted (10-FEB-2023) Biology, San Francisco State University,
            1600 Holloway Ave, San Francisco, CA 94132, USA
COMMENT     ##Genome-Assembly-Data-START##
            Assembly Date          :: JUN-2019
            Assembly Method        :: FLYE v. 06-2019
            Genome Representation  :: Full
            Expected Final Version :: Yes
            Genome Coverage        :: 100.0x
            Sequencing Technology  :: Illumina HiSeq; PacBio Sequel
            ##Genome-Assembly-Data-END##
FEATURES             Location/Qualifiers
     source          1..2900
                     /organism="Wolbachia pipientis"
                     /mol_type="genomic DNA"
                     /submitter_seqid="contig_18195"
                     /isolate="wEsol"
                     /isolation_source="galls on Solidago altissima"
                     /host="Eurosta solidaginis MN-2018-EFC"
                     /db_xref="taxon:955"
                     /environmental_sample
                     /country="USA: Cowling Arboretum, Carleton College,
                     Northfield, MN"
                     /lat_lon="44.48 N 93.13 W"
```

```
                     /altitude="280 m"
                     /collection_date="2018-11"
                     /metagenome_source="insect metagenome"
                     /note="metagenomic"
     gene            <1..1984
                     /locus_tag="wEsol_00158"
     CDS             <1..1984
                     /locus_tag="wEsol_00158"
                     /codon_start=1
                     /transl_table=11
                     /product="hypothetical protein"
                     /pfam="Pfam-B_2381 Pfam-B_13184 AAA_16 NB-ARC AAA_22
                     AAA_14 Ank_2 Ank_4 Ank_5 Pfam-B_1873 Ank Ank_4 Ank_3
                     Pfam-B_10896 Ank_5 Pfam-B_1714 Ank Ank_3 Ank_2 Ank_4
                     Ank_5 Ank Ank_3 Ank_4 Ank_2 Pfam-B_1873 Ank_5
                     Pfam-B_10896 Ank_3 Ank Ank_4 Ank_5 Ank Ank_3 Ank_5 Ank_2
                     Ank Ank_3 Ank_4"
                     /translation="WFGIIEPVASFTGRGRVLTALHNMLQRSAKKQAVISQVASISGL
                     GGVGKSELARKYTYKYGKDYYVNAIWIDAESTETMKNSFLDLANNRLGIPTKDRHERD
                     KTIENIVREVYAFFARRGRRSLFIFDNAEGYEDIKRFLPSSLHPRHKKPYILITSRNK
                     DWRIAEDEEKIKTIQLGVFKKTEAIRFVKRALNIKDNLQDEEIKKLIEELQYFPLALG
                     QAIAYINESNIVLSRRGEERVGVSDYLKRYEKEAEKLLDFESKYKSDRYTKTTFIAWK
                     ITIDAIAKRECGPEALKILEVMAYLAPDKIHIEEVFSKLIAEDKEKLWKAVELLDRYS
                     IIDLKKGVANIHRLVQKVTELNLQKAVREEEVLRKALELINSGDIAISHIVSIWEYAS
                     KYGKLIDDFYFNSSCIHRKPFFIKKSTPLHLLAASGDFKAIKAILTHISTHFPGKLIM
                     AVNVENNSGHAPLHFAVYNGRLDVVKYLVSKGADISAKSKDGSTLLHYAAQGGSLNVV
                     EYLIDEKGTDINIKDNDGTTPLHSVAYLGYLAVVKKFIEKGADINSRDIYYKTPLHLA
                     ASNSDLDVVEHLVNKGANVNAMDKDGLTPLHCAVFRENLEIVEYLAEKGVNTKNKDDD
                     TPLHFAAVMGKVAVAKILLKHNADVNAKNNEGKTAL"
                     /besthit="qcoverage 100 , hcoverage 71.0441334768568 ,
                     similarity 99.5 , identity 99.5 , evalue 0.0 , alnlength
                     660 , Bacteria , Proteobacteria , WP_155968791.1
                     MULTISPECIES: ankyrin repeat domain-containing protein
                     [Wolbachia]"
     gene            2043..2423
                     /locus_tag="wEsol_00159"
     CDS             2043..2423
                     /locus_tag="wEsol_00159"
                     /codon_start=1
                     /transl_table=11
                     /fullproduct="qcoverage 100 , hcoverage 100 , similarity
                     100 , identity 100 , evalue 1.16e-87 , alnlength 126 ,
                     Bacteria , Proteobacteria , AOV87106.1 transposase
                     [Wolbachia endosymbiont of Drosophila incompta]"
                     /cog="COG3293 COG3293 124 Transposase and inactivated
                     derivatives"
                     /pfam="DUF4096"
                     /product="transposase"
                     /translation="MRKKYPTDLSEREWARIEKHFRVSYKKGGRPPKYSKREILEAIF
                     YVLRTGCQWRYLPNDFPLWKTVYEQFRQWKKQGIFEKMNYEITKYSRRKIGRNEQPSA
                     CIVDSQSVKTTEKGGSKAMMEVKK"
                     /besthit="qcoverage 100 , hcoverage 100 , similarity 100
                     , identity 100 , evalue 1.16e-87 , alnlength 126 ,
                     Bacteria , Proteobacteria , AOV87106.1 transposase
                     [Wolbachia endosymbiont of Drosophila incompta]"
     gene            2534..2875
                     /locus_tag="wEsol_00160"
     CDS             2534..2875
                     /locus_tag="wEsol_00160"
                     /codon_start=1
```

```
                     /transl_table=11
                     /fullproduct="qcoverage 100 , hcoverage 100 , similarity
                     100 , identity 100 , evalue 1.53e-79 , alnlength 113 ,
                     Bacteria , Proteobacteria , AAS13810.1 transposase, IS5
                     family, OrfB [Wolbachia endosymbiont of Drosophila
                     melanogaster]"
                     /cog="COG3293 COG3293 124 Transposase and inactivated
                     derivatives"
                     /pfam="DDE_Tnp_1_2 DDE_Tnp_1"
                     /product="transposase, IS5 family, OrfB"
                     /translation="MRTKYTKVKKMWADMGYQGRNLKNHIKEEYDIDIEIVKRPPCRF
                     WVHKDTPPELLPTREQGFKVQPRRWVVERTFAWVNRNRRLSKEYDLLTTSTENFIYLA
                     MSRVMLKREYA"
                     /besthit="qcoverage 100 , hcoverage 100 , similarity 100
                     , identity 100 , evalue 1.53e-79 , alnlength 113 ,
                     Bacteria , Proteobacteria , AAS13810.1 transposase, IS5
                     family, OrfB [Wolbachia endosymbiont of Drosophila
                     melanogaster]"
ORIGIN
        1 atggtttggc ataatagagc cagtggcatc ttttactggt agaggcagag tgttaacagc
       61 tctgcataat atgctacaaa gaagtgcaaa aaaacaagcg gtgatatcgc aggttgcctc
      121 tattagcggt cttggaggag taggtaaaag tgagttggct aggaaatata cttacaaata
      181 tggaaaggat tattatgtta atgctatctg gatagatgct gaaagcactg aaactatgaa
      241 aaattctttc ctggatttag ctaataatag attaggcatt cctaccaaag ataggcacga
      301 aagggacaag acgatagaaa acattgttag agaagtatat gctttttttg caagaagggg
      361 aaggagaagc ctgttcattt ttgataatgc tgagggatat gaagacatta aaaggttttt
      421 accttcatct ttacacccta gacataaaaa accttacatt ttaattactt ctcgtaataa
      481 agattggaga atagcagaag acgaggaaaa aataaaaaca atacagctag gtgtattcaa
      541 aaaaacggag gctataaggt ttgtcaagag ggctttaaat ataaaagata atttacaaga
      601 tgaagaaata aaaaaactaa tagaggagtt acaatatttc ccactagctt tggggcaagc
      661 aattgcatat ataaatgaaa gcaacattgt attaagtcgc agaggtgaag aaagagttgg
      721 ggttagtgac tacctaaaaa ggtatgaaaa agaagcagag aaactgctcg actttgagtc
      781 taaatataaa agtgaccgtt atactaaaac aacattcata gcttggaaaa ttacgattga
      841 tgctatagca aaaagagaat gtgggccaga agcattgaag attctagaag taatggctta
      901 tcttgctcct gacaagattc atatagaaga agtcttttca aagttaatag cagaggataa
      961 ggaaaagtta tggaaggctg ttgagctact cgatagatat tcaataattg acttaaaaaa
     1021 aggagtggca aacattcaca gactagtgca gaaagtaact gagttgaatt tacaaaaggc
     1081 agtccgggaa gaagaagttc ttagaaaagc tctggaattg ataaatagtg gtgatatagc
     1141 aatcagtcac attgtgtcta tttgggaata tgcaagtaaa tatggtaagt tgattgatga
     1201 tttttacttt aactcatctt gtatacatag gaaaccattt tttataaaga aaagtactcc
     1261 tttgcacttg cttgctgcaa gtggtgattt taaagcaatt aaggctatac tcacacatat
     1321 atcaacacat ttcccaggta aattgataat ggctgttaat gttgaaaata attctggtca
     1381 cgctccatta cactttgctg tttataatgg aagattagat gtagtaaagt accttgttag
     1441 taaaggtgct gacattagtg ctaagagcaa agacggcagt actctgttgc actatgctgc
     1501 tcagggtgga agcttgaatg tagtagaata cttgatagat gaaaagggta cagacattaa
     1561 cataaaagat aatgacggta caactcctct acactcggtt gcttatctag gctacttagc
     1621 tgtagtgaaa aagtttatag agaaaggagc tgatattaat tctagggata tttattataa
     1681 aacacctctg catttagctg ctagtaatag tgatttagat gtagtagaac atcttgtaaa
     1741 caaaggcgct aatgtcaatg ctatggataa agatggcttg actccattac actgtgctgt
     1801 ttttcgtgaa aatttggaaa tagtagaata ccttgccgaa aaaggtgtta ataccaagaa
     1861 caaagatgac gatacgccac tacactttgc tgccgtaatg ggaaaagttg ctgtggcaaa
     1921 aatactgcta aaacacaacg ccgatgttaa cgctaaaaat aatgaaggaa aaacagcatt
     1981 gtagaggttg tccggaaata agtaaaaggc tataatatct gaaattttag tacggggtaa
     2041 agatgagaaa aaagtatcca acagatctaa gcgaaaggga atgggcaaga atagaaaaac
     2101 acttcagagt atcatacaag aaaggaggaa ggccgccaaa gtatagcaaa agagaaatat
     2161 tagaagcaat tttctatgta ttgcgtacag ggtgtcaatg gcggtattta ccaaatgatt
     2221 ttccgctatg gaagactgtg tatgagcagt tcaggcaatg gaagaagcag ggaatttttg
     2281 agaaaatgaa ttatgaaatt acaaaatata gtagaagaaa aataggaagg aatgagcagc
     2341 cgagtgcctg tatagtagat agtcaatctg taaagactac agaaaagggg ggatcaaagg
     2401 ctatgatgga agtaaaaaag taaagggtag aaaaaggcat ataattacag acactcaggg
```

```
     2461 ttttatacta ggttgttacg taggcgctgc taacgaaaat gatagagatg gtattaaaat
     2521 agcattaaac aatatgagaa caaaatatac taaagttaaa aaaatgtggg ctgacatggg
     2581 ataccaagga agaaatttaa agaatcacat aaaggaagaa tatgacatag atattgaaat
     2641 tgttaaaagg cctccatgta gattttgggt gcacaaagat acgccacctg agctactacc
     2701 aacaagagaa caagggttta aagtacagcc aagaagatgg gttgtagaaa ggacttttgc
     2761 ttgggttaat aggaatagaa ggctatcgaa ggagtatgat ttactcacaa catccactga
     2821 gaatttcata tacctagcta tgagtagggt tatgttaaag agggaatatg cttgaattta
     2881 ctagtttccg gacaacctct
//
LOCUS       JAQZAU010000019         1932 bp    dna     linear   ENV 17-MAR-2023
DEFINITION  MAG: Wolbachia pipientis isolate wEsol contig_18291, whole genome
            shotgun sequence.
ACCESSION   JAQZAU010000019  JAQZAU010000000
VERSION     JAQZAU010000019.1
DBLINK      BioProject: PRJNA929258
            BioSample: SAMN33142973
KEYWORDS    WGS; ENV; Metagenome Assembled Genome; MAG.
SOURCE      Wolbachia pipientis (insect metagenome)
  ORGANISM  Wolbachia pipientis
            Bacteria; Pseudomonadota; Alphaproteobacteria; Rickettsiales;
            Anaplasmataceae; Wolbachieae; Wolbachia.
REFERENCE   1  (bases 1 to 1932)
  AUTHORS   Fiutek,N., Couger,B.M., Roy,S., de la Torre,J.R. and Connor,E.F.
  TITLE     Genomic assessment of the contribution of the Wolbachia
            endosymbiont of Eurosta solidaginis to gall-induction
  JOURNAL   International Journal Molecular Sciences
REFERENCE   2  (bases 1 to 1932)
  AUTHORS   Fiutek,N., Couger,B.M., Roy,S., de la Torre,J.R. and Connor,E.F.
  TITLE     Direct Submission
  JOURNAL   Submitted (10-FEB-2023) Biology, San Francisco State University,
            1600 Holloway Ave, San Francisco, CA 94132, USA
COMMENT     ##Genome-Assembly-Data-START##
            Assembly Date          :: JUN-2019
            Assembly Method        :: FLYE v. 06-2019
            Genome Representation  :: Full
            Expected Final Version :: Yes
            Genome Coverage        :: 100.0x
            Sequencing Technology  :: Illumina HiSeq; PacBio Sequel
            ##Genome-Assembly-Data-END##
FEATURES             Location/Qualifiers
     source          1..1932
                     /organism="Wolbachia pipientis"
                     /mol_type="genomic DNA"
                     /submitter_seqid="contig_18291"
                     /isolate="wEsol"
                     /isolation_source="galls on Solidago altissima"
                     /host="Eurosta solidaginis MN-2018-EFC"
                     /db_xref="taxon:955"
                     /environmental_sample
                     /country="USA: Cowling Arboretum, Carleton College,
                     Northfield, MN"
                     /lat_lon="44.48 N 93.13 W"
                     /altitude="280 m"
                     /collection_date="2018-11"
                     /metagenome_source="insect metagenome"
                     /note="metagenomic"
     gene            complement(58..222)
                     /locus_tag="wEsol_00161"
     CDS             complement(58..222)
                     /locus_tag="wEsol_00161"
```

```
                     /codon_start=1
                     /transl_table=11
                     /product="hypothetical protein"

/translation="MSNKGCCYDNSVAESFFSSLKRELPIDTSRHSKQHIKTAIFEYIEIFYNKQRHY"
     gene            complement(215..376)
                     /locus_tag="wEsol_00162"
     CDS             complement(215..376)
                     /locus_tag="wEsol_00162"
                     /codon_start=1
                     /transl_table=11
                     /fullproduct="qcoverage 100 , hcoverage 100 , similarity
                     98.1 , identity 98.1 , evalue 4.57e-27 , alnlength 53 ,
                     Bacteria , Proteobacteria , WP_153801315.1 MULTISPECIES:
                     hypothetical protein [unclassified Wolbachia]"
                     /cog="Tra5 COG2801 232 Transposase and inactivated
                     derivatives"
                     /pfam="rve"
                     /product="hypothetical protein"
                     /translation="MSSSINKQLVMDSLLMAVNKRKPAKNLLLHSDQGSQYTAQGYQY
                     LLAVKNIDE"
                     /besthit="qcoverage 100 , hcoverage 100 , similarity 98.1
                     , identity 98.1 , evalue 4.57e-27 , alnlength 53 ,
                     Bacteria , Proteobacteria , WP_153801315.1 MULTISPECIES:
                     hypothetical protein [unclassified Wolbachia]"
     gene            complement(816..1100)
                     /locus_tag="wEsol_00163"
     CDS             complement(816..1100)
                     /locus_tag="wEsol_00163"
                     /codon_start=1
                     /transl_table=11
                     /fullproduct="qcoverage 100 , hcoverage 100 , similarity
                     100 , identity 100 , evalue 9.86e-56 , alnlength 94 ,
                     Bacteria , Proteobacteria , WP_039964423.1 transposase
                     [Wolbachia endosymbiont of Drosophila simulans]"
                     /cog="COG2963 COG2963 116 Transposase and inactivated
                     derivatives"
                     /pfam="HTH_Tnp_1 HTH_23 HTH_28 Pfam-B_9422"
                     /product="transposase"
                     /translation="MAREYTAEFKLEAVKLTNEQRKVGQPVAKVARDLGIRDSVLGKW
                     MKKYNEKKSAANAFPDVAPYDKERFDLQKELAKVTRERDILKKALASQKE"
                     /besthit="qcoverage 100 , hcoverage 100 , similarity 100
                     , identity 100 , evalue 9.86e-56 , alnlength 94 ,
                     Bacteria , Proteobacteria , WP_039964423.1 transposase
                     [Wolbachia endosymbiont of Drosophila simulans]"
     gene            complement(1119..1289)
                     /locus_tag="wEsol_00164"
     CDS             complement(1119..1289)
                     /locus_tag="wEsol_00164"
                     /codon_start=1
                     /transl_table=11
                     /fullproduct="qcoverage 100 , hcoverage 100 , similarity
                     100 , identity 100 , evalue 2.53e-32 , alnlength 56 ,
                     Bacteria , Proteobacteria , WP_197907316.1 hypothetical
                     protein [Wolbachia endosymbiont of Kradibia gibbosae]"
                     /translation="MSQKVVNRTTGLVDYKELEANILSSIREGRPLTGRDGAFDLTQK
                     KWRERKEFFWVK"
                     /product="hypothetical protein"
                     /besthit="qcoverage 100 , hcoverage 100 , similarity 100
                     , identity 100 , evalue 2.53e-32 , alnlength 56 ,
```

```
                     Bacteria , Proteobacteria , WP_197907316.1 hypothetical
                     protein [Wolbachia endosymbiont of Kradibia gibbosae]"
     gene            complement(1369..1701)
                     /locus_tag="wEsol_00165"
     CDS             complement(1369..1701)
                     /locus_tag="wEsol_00165"
                     /codon_start=1
                     /transl_table=11
                     /fullproduct="qcoverage 100 , hcoverage 100 , similarity
                     100 , identity 100 , evalue 8.36e-69 , alnlength 110 ,
                     Bacteria , Proteobacteria , POG52374.1 hypothetical
                     protein BJU59_00535 [Wolbachia sp. wRi_2]"
                     /translation="MYKSHIRLDKLKGKPCRVLCELYAKLCAILIFHGIVGCTEVKKN
                     TELSLTKAFIELKRRVRELFLVLTKINSLKAFLKKLTITWSRFSLKDKRRRARVSTLT
                     SLNLLTIS"
                     /product="hypothetical protein"
                     /besthit="qcoverage 100 , hcoverage 100 , similarity 100
                     , identity 100 , evalue 8.36e-69 , alnlength 110 ,
                     Bacteria , Proteobacteria , POG52374.1 hypothetical
                     protein BJU59_00535 [Wolbachia sp. wRi_2]"
     gene            complement(1836..>1932)
                     /locus_tag="wEsol_00166"
     CDS             complement(1836..>1932)
                     /locus_tag="wEsol_00166"
                     /codon_start=1
                     /transl_table=11
                     /fullproduct="qcoverage 100 , hcoverage 81.5789473684211
                     , similarity 83.9 , identity 80.6 , evalue 2.59e-07 ,
                     alnlength 31 , Bacteria , Proteobacteria , KLT23187.1
                     transposase, IS4 family [Wolbachia endosymbiont of
                     Armadillidium vulgare str. wVulC]"
                     /translation="LENEVLLGKEAKIRVRIICQKLTEYGQKKES"
                     /product="transposase, IS4 family"
                     /besthit="qcoverage 100 , hcoverage 81.5789473684211 ,
                     similarity 83.9 , identity 80.6 , evalue 2.59e-07 ,
                     alnlength 31 , Bacteria , Proteobacteria , KLT23187.1
                     transposase, IS4 family [Wolbachia endosymbiont of
                     Armadillidium vulgare str. wVulC]"
ORIGIN
        1 gtgtggaatg tttttacaa aaagaatgat gcatcaaatt gttctggaat gcaatagtta
       61 atagtgacgt tgtttgttat aaaaaatttc tatatattca aatatagcag ttttgatatg
      121 ctgtttagag tgccgagagg tgtctattgg taactccctt tttagagaac tgaagaagct
      181 ttctgcaaca gaattgtcgt aacaacagcc tttattactc atctatattc tttacagcta
      241 agagatattg atacccttgc gcagtatatt gtgaaccttg atcactatgt aatagtagat
      301 ttttggcagg tttacgcttg ttaacggcca ttaataaaga gtccataacc aattgtttat
      361 ttattgaact gctcattaac taccatgcgt gaatatagat cgattattgt tgccaaatat
      421 agccatcctt ctttggtttt tatataagta atcccatact ttatttggtt gatcgacaat
      481 aaagttttgg tctaatatat tgggagctac tgtccgtttg ttgtttctta attttaaatt
      541 ttcttctcaa tatagcctga ataccatttt tctgcataat actttgcact gtttttaagt
      601 tgcaactttt acccaaagcc tttaattcag catgaatttt aggagctcca tatctacatt
      661 tagaagcttg atatattttt tgaatatctg ctaataactc tttatttgct gattctctac
      721 tgcttatttt tcttgtggtc cacttatagt agccactagc agaaaatcct acataattcc
      781 tgtactttat agcagttact atataaaaaa atattttact ctttttgact ggccagggct
      841 ttttttaaaa tgtctctttc tcttgttact tttgctaact ctttctgtaa atcaaatctc
      901 tctttgtcat aaggtgctac atctggaaat gcatttgccg ctgacttttt ttcgttatat
      961 ttcttcatcc attttcctaa tacactatcc cttattccta gatctcttgc tacttttgca
     1021 actggttgac ctacctttct ttgttcattt gtcagcttaa cagcttccaa tttgaattct
     1081 gccgtatatt ctcttgccat ttctaatcct ccaaaatttt attttaccca aaaaaactcc
     1141 tttctttctc tccacttttt ctgggtaagg tcaaatgctc catcccttcc tgttaatggt
     1201 ctgccttccc ttattgatga cagtatattt gcttccagtt ctttgtaatc taccaaaccg
```

```
     1261 gtagttctgt ttactacttt ctgactcatt gtcaaacctc catttttta tatcaattta
     1321 ttactttttt ttcggtttga cacacttttt tgaaccatac gcgtcaagtt aagagattgt
     1381 gagcaaattt agagaggtta aagtggatac tctagccctt ctacgtttgt cttttaatga
     1441 aaatcgcgac caagttatag tgagtttttt aagaaatgct tttaaactat taattttggt
     1501 taatactaaa aataactctc taacccgcct ttttagctca ataaatgcct ttgttaagct
     1561 aagttctgta ttctttttca cttctgtgca accaactatg ccgtgaaata taagaattgc
     1621 gcacaattta gcatatagtt cacataatac tctgcatggc tttcctttta gcttatcaag
     1681 cctgatatgg cttttataca atttaaataa tttgccacct tactctgtaa atcgttaata
     1741 cttgctcagc actaatttta ttttctggaa cattagttat accagttcaa caatttttga
     1801 tttcttttag aggatcctgc gatcttgcta atctattagc tttccttctt ctggccatat
     1861 tcagttagtt tctgacatat aattcttact ctaattttg cttcttttcc taacaatacc
     1921 tcgttttcta aa
//
LOCUS       JAQZAU010000020            972 bp    dna     linear   ENV 17-MAR-2023
DEFINITION  MAG: Wolbachia pipientis isolate wEsol contig_18651, whole genome
            shotgun sequence.
ACCESSION   JAQZAU010000020  JAQZAU010000000
VERSION     JAQZAU010000020.1
DBLINK      BioProject: PRJNA929258
            BioSample: SAMN33142973
KEYWORDS    WGS; ENV; Metagenome Assembled Genome; MAG.
SOURCE      Wolbachia pipientis (insect metagenome)
  ORGANISM  Wolbachia pipientis
            Bacteria; Pseudomonadota; Alphaproteobacteria; Rickettsiales;
            Anaplasmataceae; Wolbachieae; Wolbachia.
REFERENCE   1  (bases 1 to 972)
  AUTHORS   Fiutek,N., Couger,B.M., Roy,S., de la Torre,J.R. and Connor,E.F.
  TITLE     Genomic assessment of the contribution of the Wolbachia
            endosymbiont of Eurosta solidaginis to gall-induction
  JOURNAL   International Journal Molecular Sciences
REFERENCE   2  (bases 1 to 972)
  AUTHORS   Fiutek,N., Couger,B.M., Roy,S., de la Torre,J.R. and Connor,E.F.
  TITLE     Direct Submission
  JOURNAL   Submitted (10-FEB-2023) Biology, San Francisco State University,
            1600 Holloway Ave, San Francisco, CA 94132, USA
COMMENT     ##Genome-Assembly-Data-START##
            Assembly Date          :: JUN-2019
            Assembly Method        :: FLYE v. 06-2019
            Genome Representation  :: Full
            Expected Final Version :: Yes
            Genome Coverage        :: 100.0x
            Sequencing Technology  :: Illumina HiSeq; PacBio Sequel
            ##Genome-Assembly-Data-END##
FEATURES             Location/Qualifiers
     source          1..972
                     /organism="Wolbachia pipientis"
                     /mol_type="genomic DNA"
                     /submitter_seqid="contig_18651"
                     /isolate="wEsol"
                     /isolation_source="galls on Solidago altissima"
                     /host="Eurosta solidaginis MN-2018-EFC"
                     /db_xref="taxon:955"
                     /environmental_sample
                     /country="USA: Cowling Arboretum, Carleton College,
                     Northfield, MN"
                     /lat_lon="44.48 N 93.13 W"
                     /altitude="280 m"
                     /collection_date="2018-11"
                     /metagenome_source="insect metagenome"
                     /note="metagenomic"
```

```
     gene            <1..102
                     /locus_tag="wEsol_00167"
     CDS             <1..102
                     /locus_tag="wEsol_00167"
                     /codon_start=1
                     /transl_table=11
                     /product="hypothetical protein"
                     /translation="EYNKFRKRGKFFIISPEDIEKWYENGPKIPGEQ"
ORIGIN
        1 gaatataaca aattcagaaa aagaggaaaa tttttcatta tatcacccga agacatagaa
       61 aagtggtacg agaatggtcc aaaaatacca ggggaacaat aatttatatc atcaagtttt
      121 aatttttgtg tgcacatact aactgtgtat agactcggct gaggcaaaac agagttgagt
      181 ttaatagcag gataccttga gcacatgcgg aaaaatttta gcagttaatc agctattaca
      241 atcatcaaga agtttaagaa actccaatat agatcaatga ttgcagaaaa taatatggaa
      301 gacatcaaat tagctataga aagtacagcg aattagtatt atacaacaca ctcctggtca
      361 tagcaaagaa aatagcgaga taaagatcgc agcttgaaga gaacaaaaat tgcatgaatg
      421 ttgaatacaa acagcaaaaa agcccagcgg taaaatccag taaacggcta tgatcactat
      481 ggagcttgtg atttgctaaa agaagtcgat tttcagtata ggtatcataa agattatcta
      541 tatgcagata ggacaataat ggacagagag aaaagctcta taagctgatg ataatcacta
      601 tggtattaaa aacgaaaaat tacctccgaa aacaacgcag cagagcagac acaagttagc
      661 acataaatgg ctatgacaaa ttctgcaagt caagctcata aagtcatatg atgagatagg
      721 tataaagaag tggaaaaaca aaagagatga gttatggaaa aagatttaat agaaggttta
      781 attcttgcac gatttcaaaa aagaatttga tttagttttt taggaacaaa tctgaaatga
      841 atcgtgagag ggaatgctac tcaaatgtga tttgcttaac caaattttac ggaaataggc
      901 atggactaat ttggatagac ttcataatgt attgtgcgtt acctactatc gaagagagat
      961 gacacaaagc ca
//
LOCUS       JAQZAU010000021         2594 bp    dna     linear   ENV 17-MAR-2023
DEFINITION  MAG: Wolbachia pipientis isolate wEsol contig_19356, whole genome
            shotgun sequence.
ACCESSION   JAQZAU010000021 JAQZAU010000000
VERSION     JAQZAU010000021.1
DBLINK      BioProject: PRJNA929258
            BioSample: SAMN33142973
KEYWORDS    WGS; ENV; Metagenome Assembled Genome; MAG.
SOURCE      Wolbachia pipientis (insect metagenome)
  ORGANISM  Wolbachia pipientis
            Bacteria; Pseudomonadota; Alphaproteobacteria; Rickettsiales;
            Anaplasmataceae; Wolbachieae; Wolbachia.
REFERENCE   1  (bases 1 to 2594)
  AUTHORS   Fiutek,N., Couger,B.M., Roy,S., de la Torre,J.R. and Connor,E.F.
  TITLE     Genomic assessment of the contribution of the Wolbachia
            endosymbiont of Eurosta solidaginis to gall-induction
  JOURNAL   International Journal Molecular Sciences
REFERENCE   2  (bases 1 to 2594)
  AUTHORS   Fiutek,N., Couger,B.M., Roy,S., de la Torre,J.R. and Connor,E.F.
  TITLE     Direct Submission
  JOURNAL   Submitted (10-FEB-2023) Biology, San Francisco State University,
            1600 Holloway Ave, San Francisco, CA 94132, USA
COMMENT     ##Genome-Assembly-Data-START##
            Assembly Date          :: JUN-2019
            Assembly Method        :: FLYE v. 06-2019
            Genome Representation  :: Full
            Expected Final Version :: Yes
            Genome Coverage        :: 100.0x
            Sequencing Technology  :: Illumina HiSeq; PacBio Sequel
            ##Genome-Assembly-Data-END##
FEATURES             Location/Qualifiers
     source          1..2594
                     /organism="Wolbachia pipientis"
```

```
                     /mol_type="genomic DNA"
                     /submitter_seqid="contig_19356"
                     /isolate="wEsol"
                     /isolation_source="galls on Solidago altissima"
                     /host="Eurosta solidaginis MN-2018-EFC"
                     /db_xref="taxon:955"
                     /environmental_sample
                     /country="USA: Cowling Arboretum, Carleton College,
                     Northfield, MN"
                     /lat_lon="44.48 N 93.13 W"
                     /altitude="280 m"
                     /collection_date="2018-11"
                     /metagenome_source="insect metagenome"
                     /note="metagenomic"
     gene            <1..261
                     /locus_tag="wEsol_00168"
     CDS             <1..261
                     /locus_tag="wEsol_00168"
                     /codon_start=1
                     /transl_table=11
                     /product="hypothetical protein"
                     /translation="THTGSTGVYKQRHRLPEVFHSMGRDRLERMVQELLQARPPKLVK
                     GMSTGSKEDKWLDSPTGPFACGIGKFTPGAEDFSYRQNLMLG"
                     /besthit="qcoverage 100 , hcoverage 11.8294360385144 ,
                     similarity 100 , identity 100 , evalue 5.72e-51 ,
                     alnlength 86 , Bacteria , Proteobacteria , WP_174516735.1
                     AAA family ATPase [Wolbachia endosymbiont of
                     Cardiocondyla obscurior]"
     gene            2042..>2594
                     /locus_tag="wEsol_00169"
     CDS             2042..>2594
                     /locus_tag="wEsol_00169"
                     /codon_start=1
                     /transl_table=11
                     /product="hypothetical protein"
                     /translation="MEIQRLLSKTPEQLREYCESLGYEDKQNLYKQVVDEAKGKRLRE
                     LKQLSKLATAIEKTTDKKLLKPFRGDDNPLNGVVILSFFGKLSRRLLLIMHSSVYDKS
                     LKQLDELDELLPQMYGELRPKLTSSMRNYAERELRFSNFLRDNIDTFKLLEKAEHGSI
                     EERKKAAIALFIRQPELNFQGDVF"
                     /besthit="qcoverage 100 , hcoverage 69.4339622641509 ,
                     similarity 100 , identity 100 , evalue 3.24e-120 ,
                     alnlength 184 , Bacteria , Proteobacteria ,
                     WP_174516736.1 MULTISPECIES: hypothetical protein
                     [unclassified Wolbachia]"
ORIGIN
        1 actcacacag gaagcacagg agtatataag caacggcata gactacctga agtttttcac
       61 agcatgggaa gggatagact agagcgtatg gtacaggaac ttttacaagc aagaccacca
      121 aaattagtaa agggaatgtc tacaggttcg aaagaagata aatggcttga cagtccaact
      181 ggaccatttg cttgtggtat aggaaaattc actcctggag ctgaagattt tagctatagg
      241 caaaatttaa tgttaggata agtctttttt tatgggtaaa aatattttag agtttaccca
      301 gtttcttcta ttgtttttttc aacttgtagc tactatcaat tcatatctac ttaactaaaa
      361 cgctaatttt agaactcaat ataattttca attaaaggta gatagaatat ataagaaata
      421 acaccaccaa caattgaatt acattcccaa ttcccaggtt cccaaatcag taattggaaa
      481 ttaggaatct gaatcctgcg tgagagctag cttttccacc tgcgttccca aaagcgttcc
      541 tagaaatcat gtgtctaaag tcagccagta tagcggattc tagcttccca aaacccttat
      601 atattatata tatataatag taataaataa taatagataa aataatataa gctaataata
      661 aaacatacaa atctaaatgc attcaacttt taactaagtc tgacccaaaa aatagtgtta
      721 aaatgttcat aaaacagaac gtagaaaagc aaatagcctt caatgtcgtt tttaagcgat
      781 tctagtatct taaggtaaaa taataccaac tacacctaag taacgcttgt aacacccttt
      841 aaaagccgat taaaaggtat atctaagtca tcacccacct caaattttat ctttgcagaa
```

```
      901 gaaactctat tttagtttat cacgtttttt attcgttgta aaatattttt tcaaaaaaag
      961 tgaacagttt tcataatttc ggtacaacaa gagctggtta ttttggtata ttggaggtct
     1021 tatggaaata taacgacttt tatctaaaac tcctaaggga atattgtgag tcttttgatg
     1081 aaaaaatctc tataaacagg ttatagatga aggtcaagga aaaaagtttg aagttactgc
     1141 catagaagaa gaaacagaag ctcgtaaaat ttgattgctg cttaaaatgg agacaaagag
     1201 caaaatttat tgtctccaac agaaaaactt cttttgtagt agtcaaaaaa agcagagata
     1261 tagggctttc tgaagggagg ggacaatttt tcacccccct tgaaattaaa accaaaaaga
     1321 tacagtttta catagcacat tttcattgta aaaaagagct atcagcccat ttcaattgtc
     1381 tccattgcaa caccattctt aaaaacgcct tagaaggcta ttatagcgtt actgatgatc
     1441 tagataggga gatactgttt tatcaaaaac tcatgtaacc tcttctagat tcaaataatc
     1501 agttgtttcc aaaatggaaa gatctcaaat agcgaagaag ctgttgtaaa agacatagct
     1561 ggttttaggc tctttttaat ttcttagctg ccttaaaatt attttaactt ttttttagaa
     1621 aatcccggaa aaagtggtga gaaatccgga gtataggggc attgagtgta gataacacaa
     1681 aagtcggcaa acaaacagag tagagaagag aagattgaag aaagcaaaag aaatttaggc
     1741 ttttggtaag ttacgtaatt gagaatttgt attttttttag atgaggtaaa aactttgttt
     1801 taaggctaga tgcaacttta gggtacaaaa atactaccat taccaaagaa cgcatataat
     1861 gccccttaga acttgaataa atggcattct tagcaattct attactgtat agtaaactca
     1921 ctagtgagtt tagcacgttt tttacgcttt gcaaagaaaa attaaaaaaa agtgaacagt
     1981 ttttgtaaaa tcggtgaata tatataacaa aagctggtta agttggtata ttggaggtct
     2041 tatggaaata caacgacttt tatctaaaac tcctgaacaa ttgagggaat attgtgagtc
     2101 tttaggttat gaagataagc aaaatctcta taaacaggtt gtagatgaag ctaaaggaaa
     2161 acgtttaaga gagctgaaac aattaagtaa attagctact gcaatagaaa aaacaacgga
     2221 taagaagtta ttgaaacctt ttcgcggtga tgataatccg cttaatggag tagtaatttt
     2281 aagttttttt ggaaaattaa gtaggcgcct tcttttaata atgcactctt cagtttacga
     2341 taaaagttta aaacagttgg atgagttaga tgaattatta ccacagatgt atggagaact
     2401 aagacctaaa ttgacaagta gcatgagaaa ttacgcagaa agagagctaa gattcagcaa
     2461 cttcttgcgc gataacattg atactttcaa gcttctagaa aaagctgaac atggttctat
     2521 agaagaaaga aaaaaagcag caatcgcgtt atttatacgc cagccagaat taaatttcca
     2581 aggggatgta tttt
//
LOCUS       JAQZAU010000022         1088 bp    dna     linear   ENV 17-MAR-2023
DEFINITION  MAG: Wolbachia pipientis isolate wEsol contig_19940, whole genome
            shotgun sequence.
ACCESSION   JAQZAU010000022 JAQZAU010000000
VERSION     JAQZAU010000022.1
DBLINK      BioProject: PRJNA929258
            BioSample: SAMN33142973
KEYWORDS    WGS; ENV; Metagenome Assembled Genome; MAG.
SOURCE      Wolbachia pipientis (insect metagenome)
  ORGANISM  Wolbachia pipientis
            Bacteria; Pseudomonadota; Alphaproteobacteria; Rickettsiales;
            Anaplasmataceae; Wolbachieae; Wolbachia.
REFERENCE   1  (bases 1 to 1088)
  AUTHORS   Fiutek,N., Couger,B.M., Roy,S., de la Torre,J.R. and Connor,E.F.
  TITLE     Genomic assessment of the contribution of the Wolbachia
            endosymbiont of Eurosta solidaginis to gall-induction
  JOURNAL   International Journal Molecular Sciences
REFERENCE   2  (bases 1 to 1088)
  AUTHORS   Fiutek,N., Couger,B.M., Roy,S., de la Torre,J.R. and Connor,E.F.
  TITLE     Direct Submission
  JOURNAL   Submitted (10-FEB-2023) Biology, San Francisco State University,
            1600 Holloway Ave, San Francisco, CA 94132, USA
COMMENT     ##Genome-Assembly-Data-START##
            Assembly Date          :: JUN-2019
            Assembly Method        :: FLYE v. 06-2019
            Genome Representation  :: Full
            Expected Final Version :: Yes
            Genome Coverage        :: 100.0x
            Sequencing Technology  :: Illumina HiSeq; PacBio Sequel
            ##Genome-Assembly-Data-END##
```

```
FEATURES             Location/Qualifiers
     source          1..1088
                     /organism="Wolbachia pipientis"
                     /mol_type="genomic DNA"
                     /submitter_seqid="contig_19940"
                     /isolate="wEsol"
                     /isolation_source="galls on Solidago altissima"
                     /host="Eurosta solidaginis MN-2018-EFC"
                     /db_xref="taxon:955"
                     /environmental_sample
                     /country="USA: Cowling Arboretum, Carleton College,
                     Northfield, MN"
                     /lat_lon="44.48 N 93.13 W"
                     /altitude="280 m"
                     /collection_date="2018-11"
                     /metagenome_source="insect metagenome"
                     /note="metagenomic"
     gene            complement(<1..89)
                     /locus_tag="wEsol_00170"
     CDS             complement(<1..89)
                     /locus_tag="wEsol_00170"
                     /codon_start=1
                     /transl_table=11
                     /product="hypothetical protein"
                     /translation="MPFKPRYFTYFSMAFFLLLSIRYLCSGNW"
     gene            83..265
                     /locus_tag="wEsol_00171"
     CDS             83..265
                     /locus_tag="wEsol_00171"
                     /codon_start=1
                     /transl_table=11
                     /fullproduct="qcoverage 100 , hcoverage 100 , similarity
                     100 , identity 100 , evalue 1.53e-28 , alnlength 60 ,
                     Bacteria , Proteobacteria , EAL57766.1 hypothetical
                     protein WwAna0375 [Wolbachia endosymbiont of Drosophila
                     ananassae]"
                     /translation="MALRSKLLDEKVVESAKEMLKKVRNNAYVAKKLNAVIAAKKHSI
                     TAVAKICCISRKALTA"
                     /product="hypothetical protein"
                     /besthit="qcoverage 100 , hcoverage 100 , similarity 100
                     , identity 100 , evalue 1.53e-28 , alnlength 60 ,
                     Bacteria , Proteobacteria , EAL57766.1 hypothetical
                     protein WwAna0375 [Wolbachia endosymbiont of Drosophila
                     ananassae]"
     gene            327..551
                     /locus_tag="wEsol_00172"
     CDS             327..551
                     /locus_tag="wEsol_00172"
                     /codon_start=1
                     /transl_table=11
                     /fullproduct="qcoverage 100 , hcoverage 100 , similarity
                     100 , identity 100 , evalue 6.33e-43 , alnlength 74 ,
                     Bacteria , Proteobacteria , EAL59033.1 conserved
                     hypothetical protein [Wolbachia endosymbiont of
                     Drosophila ananassae]"
                     /cog="COG3415 COG3415 138 Transposase and inactivated
                     derivatives"
                     /pfam="HTH_32 Pfam-B_19535 HTH_Tnp_Tc3_2 HTH_33 HTH_24"
                     /product="conserved hypothetical protein"
                     /translation="MNQSQREQVEVWIEENPNITIKEMKIRIQEKFGLNISKSTVHRN
```

```
                     MQRMKFSYITPRPVHNGQDKSKQEEFKKKS"
                     /besthit="qcoverage 100 , hcoverage 100 , similarity 100
                     , identity 100 , evalue 6.33e-43 , alnlength 74 ,
                     Bacteria , Proteobacteria , EAL59033.1 conserved
                     hypothetical protein [Wolbachia endosymbiont of
                     Drosophila ananassae]"
     gene            565..1071
                     /locus_tag="wEsol_00173"
     CDS             565..1071
                     /locus_tag="wEsol_00173"
                     /codon_start=1
                     /transl_table=11
                     /pfam="DDE_3 DDE_1"
                     /fullproduct="qcoverage 100 , hcoverage 100 , similarity
                     100 , identity 100 , evalue 3.77e-120 , alnlength 168 ,
                     Bacteria , Proteobacteria , EAL59034.1 transposase family
                     protein [Wolbachia endosymbiont of Drosophila ananassae]"
                     /translation="MYPEKELFFFDESRFGTHSKIGHGWFKKGIRTQVKVKLGRQNFY
                     LYSAVNPRNGESSSSNVNTDCMNIFLEQMSQYLGTREAFLIMDCASWHKSKNLKVPKN
                     IDIIYLPPYSPDLNPVERFWLYIKQNILRNKIYDTIALLESALCKFITSLSHSTIKKL
                     CNAAYLIH"
                     /product="transposase family protein"
                     /besthit="qcoverage 100 , hcoverage 100 , similarity 100
                     , identity 100 , evalue 3.77e-120 , alnlength 168 ,
                     Bacteria , Proteobacteria , EAL59034.1 transposase family
                     protein [Wolbachia endosymbiont of Drosophila ananassae]"
ORIGIN
        1 taccaattcc cgctacacaa gtaacggata gacaataata gaaaaaaagc catactgaag
       61 taagtaaagt agcgaggttt aaatggcatt aagatcaaaa ttattggatg aaaaagtggt
      121 ggaatcagca aaagaaatgc tgaagaaagt aagaaataac gcatatgttg caaaaaaact
      181 aaatgctgta attgcagcaa aaaagcacag tataacagcc gtagcaaaga tatgctgcat
      241 ttcaagaaag gcacttactg catgacctaa aatttggcag agaagaaaaa ttatttgctc
      301 cgcctcaacg ccgtagaaaa actatattga atcaaagtca acgtgaacaa gttgaggtgt
      361 ggatagagga aaaccccaat attactataa aagaaatgaa aataagaatt caagaaaagt
      421 ttggtttgaa tatcagcaaa tctacagtac accgtaatat gcaaagaatg aaattctcat
      481 atatcacacc aagaccagtg cataacggac aagataaaag taaacaagag gaatttaaaa
      541 aaaaatctta atgaaactat tgtcatgtat cctgaaaaag agctattttt cttcgatgaa
      601 tcgcggtttg gcacacattc aaagattgga cacgggtggt ttaaaaaagg cattaggaca
      661 caggttaagg taaaactagg taggcaaaat ttttatctct atagtgcagt taatcccaga
      721 aatggagaaa gttctagctc aaacgtcaat actgattgta tgaatatatt ccttgagcag
      781 atgtcacaat atttaggaac aagggaggca tttcttatca tggattgtgc tagttggcat
      841 aagtcaaaaa atttaaaagt acctaaaaat atcgacatta tatacctacc accatactca
      901 cctgacctca atcctgttga gaggttttgg ttatatataa aacagaacat tttgcgtaac
      961 aaaatctacg acacaattgc tttgcttgag agcgctttgt gcaaatttat tacctctctt
     1021 tctcactcca caattaaaaa actctgcaat gccgcttatt tgattcacta gtaatgagaa
     1081 ttggtatt
//
LOCUS       JAQZAU010000023            937 bp    dna     linear   ENV 17-MAR-2023
DEFINITION  MAG: Wolbachia pipientis isolate wEsol contig_20599, whole genome
            shotgun sequence.
ACCESSION   JAQZAU010000023 JAQZAU010000000
VERSION     JAQZAU010000023.1
DBLINK      BioProject: PRJNA929258
            BioSample: SAMN33142973
KEYWORDS    WGS; ENV; Metagenome Assembled Genome; MAG.
SOURCE      Wolbachia pipientis (insect metagenome)
  ORGANISM  Wolbachia pipientis
            Bacteria; Pseudomonadota; Alphaproteobacteria; Rickettsiales;
            Anaplasmataceae; Wolbachieae; Wolbachia.
REFERENCE   1  (bases 1 to 937)
```

```
  AUTHORS   Fiutek,N., Couger,B.M., Roy,S., de la Torre,J.R. and Connor,E.F.
  TITLE     Genomic assessment of the contribution of the Wolbachia
            endosymbiont of Eurosta solidaginis to gall-induction
  JOURNAL   International Journal Molecular Sciences
REFERENCE   2  (bases 1 to 937)
  AUTHORS   Fiutek,N., Couger,B.M., Roy,S., de la Torre,J.R. and Connor,E.F.
  TITLE     Direct Submission
  JOURNAL   Submitted (10-FEB-2023) Biology, San Francisco State University,
            1600 Holloway Ave, San Francisco, CA 94132, USA
COMMENT     ##Genome-Assembly-Data-START##
            Assembly Date          :: JUN-2019
            Assembly Method        :: FLYE v. 06-2019
            Genome Representation  :: Full
            Expected Final Version :: Yes
            Genome Coverage        :: 100.0x
            Sequencing Technology  :: Illumina HiSeq; PacBio Sequel
            ##Genome-Assembly-Data-END##
FEATURES             Location/Qualifiers
     source          1..937
                     /organism="Wolbachia pipientis"
                     /mol_type="genomic DNA"
                     /submitter_seqid="contig_20599"
                     /isolate="wEsol"
                     /isolation_source="galls on Solidago altissima"
                     /host="Eurosta solidaginis MN-2018-EFC"
                     /db_xref="taxon:955"
                     /environmental_sample
                     /country="USA: Cowling Arboretum, Carleton College,
                     Northfield, MN"
                     /lat_lon="44.48 N 93.13 W"
                     /altitude="280 m"
                     /collection_date="2018-11"
                     /metagenome_source="insect metagenome"
                     /note="metagenomic"
     gene            complement(<1..460)
                     /locus_tag="wEsol_00174"
     CDS             complement(<1..460)
                     /locus_tag="wEsol_00174"
                     /codon_start=1
                     /transl_table=11
                     /fullproduct="qcoverage 100 , hcoverage 99.3506493506494
                     , similarity 99.3 , identity 99.3 , evalue 1.02e-101 ,
                     alnlength 153 , Bacteria , Proteobacteria ,
                     WP_197907400.1 MULTISPECIES: phage baseplate assembly
                     protein V [unclassified Wolbachia]"
                     /cog="gpV COG4540 184 Phage P2 baseplate assembly protein
                     gpV"
                     /pfam="Phage_base_V"
                     /tigr="TIGR01644 phage_P2_V 192 phage baseplate assembly
                     protein V"
                     /product="phage baseplate assembly protein V"
                     /translation="MLEHNFAISELNRKLANIIRIGLVKEVDYEKARVRVKIGEFLTD
                     WLPWVTARAGEDRSWFAPNIDEQVIVLSPYGELSLGVVLPAIYQEKYPPPENKKEINS
                     AKFQDGTKLSYDKDKHHLEIDVIDKITLKVGESSIEMTKKGIKLKAKRINL"
                     /besthit="qcoverage 100 , hcoverage 99.3506493506494 ,
                     similarity 99.3 , identity 99.3 , evalue 1.02e-101 ,
                     alnlength 153 , Bacteria , Proteobacteria ,
                     WP_197907400.1 MULTISPECIES: phage baseplate assembly
                     protein V [unclassified Wolbachia]"
     gene            complement(454..912)
```

```
                     /locus_tag="wEsol_00175"
     CDS             complement(454..912)
                     /locus_tag="wEsol_00175"
                     /codon_start=1
                     /transl_table=11
                     /pfam="Pfam-B_4030"
                     /fullproduct="qcoverage 100 , hcoverage 100 , similarity
                     100 , identity 100 , evalue 9.93e-106 , alnlength 152 ,
                     Bacteria , Proteobacteria , WP_209452513.1 hypothetical
                     protein [Wolbachia endosymbiont of Ceratosolen solmsi]"
                     /translation="MILTELHKAICRMLKKEIPAIQTCEVYPTIRREIVAPAVFVELS
                     SLEQGKDPGTEELALRARFEARIVVDGTVEDSSVVVRELAAEVARVVNKNTWDVENVS
                     AGEFISAGGDDFRPELDAYLVWMVEWVHEIHVWKSVWSETGTYNKCWRKC"
                     /product="hypothetical protein"
                     /besthit="qcoverage 100 , hcoverage 100 , similarity 100
                     , identity 100 , evalue 9.93e-106 , alnlength 152 ,
                     Bacteria , Proteobacteria , WP_209452513.1 hypothetical
                     protein [Wolbachia endosymbiont of Ceratosolen solmsi]"
ORIGIN
        1 ttagatttat tctttttgct ttgagtttta ttccctttt tgtcatttct atacttgatt
       61 ccccaacttt tagcgttatt ttatctatta catctatctc taaatgatgt ttatctttat
      121 cgtacgataa ctttgtccca tcttgaaatt ttgcactatt tatttccttt ttattctccg
      181 gagggggata tttctcctga tatattgctg gtaatactac ccctaatgac aactctccat
      241 atggcgataa tactattact tgctcatcaa tattcggcgc aaaccaactt ctatcttctc
      301 ctgctcttgc cgttacccac ggaagccaat ccgttaaaaa ttctcctatt tttacccgca
      361 ctcttgcttt ttcatagtct acttctttaa ctagacctat acgaataata ttcgctaact
      421 tcctattcag ctctgaaatt gcaaaattat gctctaacat tttcgccaac acttattgta
      481 tgtgccagtt tctgaccaca ctgacttcca tacatggatt tcatgcaccc attcaaccat
      541 ccacactaga tatgcatcca actctggcct aaaatcatct cctcctgctg atataaattc
      601 acctgctgaa acattttcca cgtcccaagt attcttattc actactctcg caacttctgc
      661 tgctaattct ctcacaacta ccgaggaatc ctcgactgtt ccatcaacta caattcttgc
      721 ttcaaatctt gctctcagcg ccaattcctc tgttcctgga tcttttcctt gctctaaact
      781 tgacaattct acaaataccg ctggtgctac tatttccctt cttatcgttg gatatacttc
      841 acatgtctgt atcgctggta tttctttctt caacatcctg caaattgctt tgtgcaactc
      901 agttaaaatc atacttttac aatatagttt aattcac
//
LOCUS       JAQZAU010000024            8269 bp    dna     linear   ENV 17-MAR-2023
DEFINITION  MAG: Wolbachia pipientis isolate wEsol contig_20751, whole genome
            shotgun sequence.
ACCESSION   JAQZAU010000024  JAQZAU010000000
VERSION     JAQZAU010000024.1
DBLINK      BioProject: PRJNA929258
            BioSample: SAMN33142973
KEYWORDS    WGS; ENV; Metagenome Assembled Genome; MAG.
SOURCE      Wolbachia pipientis (insect metagenome)
  ORGANISM  Wolbachia pipientis
            Bacteria; Pseudomonadota; Alphaproteobacteria; Rickettsiales;
            Anaplasmataceae; Wolbachieae; Wolbachia.
REFERENCE   1  (bases 1 to 8269)
  AUTHORS   Fiutek,N., Couger,B.M., Roy,S., de la Torre,J.R. and Connor,E.F.
  TITLE     Genomic assessment of the contribution of the Wolbachia
            endosymbiont of Eurosta solidaginis to gall-induction
  JOURNAL   International Journal Molecular Sciences
REFERENCE   2  (bases 1 to 8269)
  AUTHORS   Fiutek,N., Couger,B.M., Roy,S., de la Torre,J.R. and Connor,E.F.
  TITLE     Direct Submission
  JOURNAL   Submitted (10-FEB-2023) Biology, San Francisco State University,
            1600 Holloway Ave, San Francisco, CA 94132, USA
COMMENT     ##Genome-Assembly-Data-START##
            Assembly Date          :: JUN-2019
```

```
          Assembly Method        :: FLYE v. 06-2019
          Genome Representation  :: Full
          Expected Final Version :: Yes
          Genome Coverage        :: 100.0x
          Sequencing Technology  :: Illumina HiSeq; PacBio Sequel
          ##Genome-Assembly-Data-END##
FEATURES             Location/Qualifiers
     source          1..8269
                     /organism="Wolbachia pipientis"
                     /mol_type="genomic DNA"
                     /submitter_seqid="contig_20751"
                     /isolate="wEsol"
                     /isolation_source="galls on Solidago altissima"
                     /host="Eurosta solidaginis MN-2018-EFC"
                     /db_xref="taxon:955"
                     /environmental_sample
                     /country="USA: Cowling Arboretum, Carleton College,
                     Northfield, MN"
                     /lat_lon="44.48 N 93.13 W"
                     /altitude="280 m"
                     /collection_date="2018-11"
                     /metagenome_source="insect metagenome"
                     /note="metagenomic"
     gene            <1..526
                     /locus_tag="wEsol_00176"
     CDS             <1..526
                     /locus_tag="wEsol_00176"
                     /codon_start=1
                     /transl_table=11
                     /fullproduct="qcoverage 100 , hcoverage 94.0540540540541
                     , similarity 100 , identity 100 , evalue 1.83e-112 ,
                     alnlength 174 , Bacteria , Proteobacteria , EEH12441.1
                     DnaJ domain protein, partial [Wolbachia endosymbiont of
                     Muscidifurax uniraptor]"
                     /translation="FLAPLLAGSILSLAVKSIYTRNGKEFNHQTNILLLFNFDQECEN
                     FSKSDRTKLKWLKNSVILSNTICTSLMFFGVGLDLVANGFGITNSMMLSGLLLSLPFT
                     LLIVTSPILNAASNYILKKTAINSIEEQFKGDDQFIEVDNEKPSSTMSNQSYFPIGQL
                     ISSKLAILHSEVSS"
                     /product="DnaJ domain protein, partial"
                     /besthit="qcoverage 100 , hcoverage 94.0540540540541 ,
                     similarity 100 , identity 100 , evalue 1.83e-112 ,
                     alnlength 174 , Bacteria , Proteobacteria , EEH12441.1
                     DnaJ domain protein, partial [Wolbachia endosymbiont of
                     Muscidifurax uniraptor]"
     gene            complement(494..1774)
                     /locus_tag="wEsol_00177"
     CDS             complement(494..1774)
                     /locus_tag="wEsol_00177"
                     /codon_start=1
                     /transl_table=11
                     /fullproduct="qcoverage 100 , hcoverage 99.3006993006993
                     , similarity 99.8 , identity 99.5 , evalue 7.18e-314 ,
                     alnlength 426 , Bacteria , Proteobacteria ,
                     WP_197907432.1 MULTISPECIES: bifunctional
                     folylpolyglutamate synthase/dihydrofolate synthase
                     [unclassified Wolbachia]"
                     /cog="FolC COG0285 427 Folylpolyglutamate synthase"
                     /pfam="Mur_ligase_M"
                     /tigr="TIGR01499 folC 400 bifunctional protein FolC"
                     /product="bifunctional
```

```
                     folylpolyglutamate synthase/dihydrofolate synthase"
                     /translation="MPHWPKPLGSRPNDFSLDRIKSFLSKLDNPEKKVPPVIHIAGTN
                     GKGSTLAFIRYIMQAAGYKVHAYTSPHLVNFNERIVIAGSYIDDNELYSLLEECRAVI
                     AEQPITLFEAATIAAFLAFSRHKADITLIEVGMGGRLDTTNVIDSPILTIITSIALDH
                     TEYLGPTIEIIAGEKAGIMKPNVPCVIAPQEKSIMNTLEYHAINKKSPLYRGGFEWNC
                     KKKNNKMVFQSAIQSIEFPLPSLKGDHQIINAGNAIAACSILSGKYGFDIGEEDITSG
                     LQCTYWPAQLERIKEGNLISLLPKDWQLFLDGAHNNDGARVLFQWVKDNFAEGIYIIF
                     GVTRNRNVEEFLEHLKPYIKLLCAVCVKSEPKATNTNLIREKANNIGIKAIECESIGD
                     AISNHILKASIQNVKTILICGSLFLARDFAMENS"
                     /besthit="qcoverage 100 , hcoverage 99.3006993006993 ,
                     similarity 99.8 , identity 99.5 , evalue 7.18e-314 ,
                     alnlength 426 , Bacteria , Proteobacteria ,
                     WP_197907432.1 MULTISPECIES: bifunctional
                     folylpolyglutamate synthase/dihydrofolate synthase
                     [unclassified Wolbachia]"
     gene            complement(1780..2370)
                     /locus_tag="wEsol_00178"
     CDS             complement(1780..2370)
                     /locus_tag="wEsol_00178"
                     /codon_start=1
                     /transl_table=11
                     /fullproduct="qcoverage 100 , hcoverage 100 , similarity
                     100 , identity 100 , evalue 4.23e-133 , alnlength 196 ,
                     Bacteria , Proteobacteria , WP_015589354.1 MULTISPECIES:
                     ABC transporter substrate-binding protein [unclassified
                     Wolbachia]"
                     /cog="Ttg2D COG2854 202 ABC-type transport system
                     involved in resistance to organic solvents, auxiliary
                     component"
                     /pfam="Pfam-B_9033 Tol_Tol_Ttg2"
                     /tigr="TIGR03481 HpnM 200 hopanoid biosynthesis
                     associated membrane protein HpnM"
                     /product="ABC transporter substrate-binding protein"
                     /translation="MNITKVLIIIVFIVISSSAYTNCTDHKIFVHTMKQQVDSISTKG
                     NRKNLEHVHEKLQEVIQNNVNLKEISRFVMGKYWVLTTRKEREDFLREYEVYLTRLCV
                     KILSKYMSNSEMIIMSTKAVDDETCLIGTRFSYGDEEFTNIDFKVTKNHSSFLISDVV
                     MSGISIAINQRSQFSEKIDTHGIASVIGELKCNNNL"
                     /besthit="qcoverage 100 , hcoverage 100 , similarity 100
                     , identity 100 , evalue 4.23e-133 , alnlength 196 ,
                     Bacteria , Proteobacteria , WP_015589354.1 MULTISPECIES:
                     ABC transporter substrate-binding protein [unclassified
                     Wolbachia]"
     gene            2572..3114
                     /locus_tag="wEsol_00179"
     CDS             2572..3114
                     /locus_tag="wEsol_00179"
                     /codon_start=1
                     /transl_table=11
                     /fullproduct="qcoverage 100 , hcoverage 100 , similarity
                     100 , identity 100 , evalue 2.23e-127 , alnlength 180 ,
                     Bacteria , Proteobacteria , ACN95788.1 cyclase/dehydrase
                     [Wolbachia sp. wRi]"
                     /cog="COG2867 COG2867 146 Oligoketide cyclase/lipid
                     transport protein"
                     /pfam="Polyketide_cyc"
                     /product="cyclase/dehydrase"
                     /translation="MPTLTSGLRNLLRLTVDTDPFLIGTKFMLHQYREQGTFLCSPNE
                     VFQIVIDVERYPDFVPWCKAVYIKEKIDNQMVVDLLAAFHGIKGRYTSEVTFLSPSRT
                     NEGWIKAVSSNGIFKHLCNKWQFIPIDENKTMVKFYIEFKFKSNSFSTLLNSVYKYTQ
                     SKIIAAFKDRAESLAKQKLP"
```

```
                     /besthit="qcoverage 100 , hcoverage 100 , similarity 100
                     , identity 100 , evalue 2.23e-127 , alnlength 180 ,
                     Bacteria , Proteobacteria , ACN95788.1 cyclase/dehydrase
                     [Wolbachia sp. wRi]"
     gene            3168..3854
                     /locus_tag="wEsol_00180"
     CDS             3168..3854
                     /locus_tag="wEsol_00180"
                     /codon_start=1
                     /transl_table=11
                     /pfam="Thioredoxin_4 Thioredoxin_2 DSBA"
                     /fullproduct="qcoverage 100 , hcoverage 100 , similarity
                     100 , identity 100 , evalue 3.18e-162 , alnlength 228 ,
                     Bacteria , Proteobacteria , WP_015589356.1 MULTISPECIES:
                     DsbA family protein [unclassified Wolbachia]"
                     /translation="MIFRLLLLSIFISVNSYAVVNQHIQKETNEITSKELLSLLPDDK
                     LLGDPKAPILMIEYASLTCYHCSLFHKNVFPKIKEKYIDTGKMLYIFRHFPLDYRGLK
                     AAMLSHCYEKQEDYFNFNKAVFNSIDSWNYYNLSDLTLLQRIAALSNLKQDAFNQCIN
                     DKKIMDKIINDKSLAINKLGITATPIFFIKLNDGKSYIEHNKVKHEGYKELKYLTDVI
                     DKLYGKAIVK"
                     /product="DsbA family protein"
                     /besthit="qcoverage 100 , hcoverage 100 , similarity 100
                     , identity 100 , evalue 3.18e-162 , alnlength 228 ,
                     Bacteria , Proteobacteria , WP_015589356.1 MULTISPECIES:
                     DsbA family protein [unclassified Wolbachia]"
     gene            complement(3894..4163)
                     /locus_tag="wEsol_00181"
     CDS             complement(3894..4163)
                     /locus_tag="wEsol_00181"
                     /codon_start=1
                     /transl_table=11
                     /pfam="CotH DUF2671"
                     /fullproduct="qcoverage 97.752808988764 , hcoverage
                     94.5652173913043 , similarity 100 , identity 100 , evalue
                     2.43e-52 , alnlength 87 , Bacteria , Proteobacteria ,
                     WP_007551133.1 MULTISPECIES: DUF2671 domain-containing
                     protein [Wolbachia]"
                     /translation="MSYTIASEVETLNKKSNKNEGVMLLNNPAYREKYKERFDEAQRK
                     VMDMVQFYDGTIVLIENKIAMYYYAWHSKKREFERKKQQTVVKKQ"
                     /product="DUF2671 domain-containing protein"
                     /besthit="qcoverage 97.752808988764 , hcoverage
                     94.5652173913043 , similarity 100 , identity 100 , evalue
                     2.43e-52 , alnlength 87 , Bacteria , Proteobacteria ,
                     WP_007551133.1 MULTISPECIES: DUF2671 domain-containing
                     protein [Wolbachia]"
     gene            4415..5062
                     /locus_tag="wEsol_00182"
     CDS             4415..5062
                     /locus_tag="wEsol_00182"
                     /codon_start=1
                     /transl_table=11
                     /fullproduct="qcoverage 100 , hcoverage 100 , similarity
                     99.5 , identity 99.5 , evalue 7.82e-152 , alnlength 215 ,
                     Bacteria , Proteobacteria , WP_015589357.1 MULTISPECIES:
                     O-methyltransferase [unclassified Wolbachia]"
                     /cog="COG4122 COG4122 219 Predicted O-methyltransferase"
                     /pfam="Methyltransf_3 Pfam-B_2663 PCMT GCD14
                     Methyltransf_31 Methyltransf_18 Methyltransf_26
                     Methyltransf_24 Methyltransf_15 Cons_hypoth95"
                     /product="O-methyltransferase"
```

```
                     /translation="MRNNFSIKLSYIRNLFAKEFKTIEEYCILEKKQHIQIGHEEGKL
                     LSLFIKIHKIKSIVEIGTLYGYSSICMAKALLEDGHIYTIENNPQYSRIAKKNFSAFN
                     LSDKITLIEGNALEKLNELSAKAPFDMIFIDVDKGGYPKYLDWAESYIKQDGLIVADN
                     TLLFNTVFLESPPKEVSEKSWHAMREFNNRLSDEKKYFSILIPTDEGMTVALKLT"
                     /besthit="qcoverage 100 , hcoverage 100 , similarity 99.5
                     , identity 99.5 , evalue 7.82e-152 , alnlength 215 ,
                     Bacteria , Proteobacteria , WP_015589357.1 MULTISPECIES:
                     O-methyltransferase [unclassified Wolbachia]"
     gene            complement(5064..5717)
                     /locus_tag="wEsol_00183"
     CDS             complement(5064..5717)
                     /locus_tag="wEsol_00183"
                     /codon_start=1
                     /transl_table=11
                     /fullproduct="qcoverage 100 , hcoverage 100 , similarity
                     100 , identity 100 , evalue 5.32e-160 , alnlength 217 ,
                     Bacteria , Proteobacteria , WP_141457290.1 MULTISPECIES:
                     glutathione S-transferase family protein [unclassified
                     Wolbachia]"
                     /cog="Gst COG0625 211 Glutathione S-transferase"
                     /pfam="GST_N Glutaredoxin GST_N_3 GST_N_2 GST_C GST_C_2"
                     /product="glutathione S-transferase family protein"
                     /translation="MILYHFPLCPFSRKVRALLKEKKLGCDLVYENPWEKRNEFMEIN
                     PTGQVPVLIDNNFVIADSNAICEYIEETYNSDVKLFGSSTIIKSKIRALINWFDNKFY
                     NEVTKYIMNEKVITNRSPDSRFIYAAQHNLLCHIEYVEHLINKNIWLATDKFTLADIT
                     LASHISVIDYVSSFPWEKSKILKEWYSIVKSKPCFREILLERISGLIPPKHYADLDF"
                     /besthit="qcoverage 100 , hcoverage 100 , similarity 100
                     , identity 100 , evalue 5.32e-160 , alnlength 217 ,
                     Bacteria , Proteobacteria , WP_141457290.1 MULTISPECIES:
                     glutathione S-transferase family protein [unclassified
                     Wolbachia]"
     gene            6008..7651
                     /locus_tag="wEsol_00184"
     CDS             6008..7651
                     /locus_tag="wEsol_00184"
                     /codon_start=1
                     /transl_table=11
                     /fullproduct="qcoverage 100 , hcoverage 100 , similarity
                     99.3 , identity 98.2 , evalue 0.0 , alnlength 547 ,
                     Bacteria , Proteobacteria , ERN55306.1 DNA polymerase III
                     subunits gamma and tau [Wolbachia pipientis wMelPop]"
                     /cog="DnaX COG2812 515 DNA polymerase III, gamma/tau
                     subunits"
                     /pfam="RuvB_N AAA_16 Pfam-B_18840 DNA_pol3_delta2 AAA_22
                     AAA AAA_17 AAA_18 TIP49 Pfam-B_5933 DNA_pol3_gamma3
                     DUF3646"
                     /product="DNA polymerase III subunits gamma and tau"
                     /translation="MGCIPTSLFVLSEAVNIALKHRPSSFKDLVGQDILVRVLENAFI
                     LNKIPQSILLSGSSGVGKTTTARIIALCLNCSLGPIFEPCGSCENCLAIKNSSHPDVI
                     EIDAASHTSIDDIKVILGDICYSPISSKFKVYIIDEVHMLSSSAFNALLKTLEEPPSS
                     VKFILATTEIKKIPITIIARCQRFDLHNIPVAKIVERLNDVAQKESYSIEKGALELIA
                     RHSGNSMRNALFLMNQAVLYSKDGAISTKNVTDILGLVDKDIIFDLLGAILEGDLQKA
                     LSVFDKAIKTANPLSIFEGLLQTIQLVCRFLITKEIDSAVTEYEKNRIKDLSIKRSLI
                     FFSRLWKVLLKGIQDIKTSTCNDVAAEMMLISLCYLSDLPSPEQVIKKVLSQNVEQGN
                     RQGRPVAPSPSVIQVADYSDPKKYVESKQQPHSVIPVRDTGIHVENKQQNYDFDKILQ
                     LLRRSNQIYLYKQLCNNLQLIDCKPGYLKLKAVSKLDSNFCNDLKNYLNQATQQEWVI
                     TVDTGYINREVSNLNYAPAVKDILDMFKGAKVVNIENKE"
                     /besthit="qcoverage 100 , hcoverage 100 , similarity 99.3
                     , identity 98.2 , evalue 0.0 , alnlength 547 , Bacteria ,
                     Proteobacteria , ERN55306.1 DNA polymerase III subunits
```

```
                     gamma and tau [Wolbachia pipientis wMelPop]"
     gene            7655..7987
                     /locus_tag="wEsol_00185"
     CDS             7655..7987
                     /locus_tag="wEsol_00185"
                     /codon_start=1
                     /transl_table=11
                     /fullproduct="qcoverage 100 , hcoverage 100 , similarity
                     100 , identity 100 , evalue 1.39e-67 , alnlength 110 ,
                     Bacteria , Proteobacteria , WP_015589360.1 YbaB/EbfC
                     family nucleoid-associated protein [Wolbachia
                     endosymbiont of Drosophila simulans]"
                     /cog="COG0718 COG0718 105 Uncharacterized protein
                     conserved in bacteria"
                     /pfam="YbaB_DNA_bd"
                     /tigr="TIGR00103 DNA_YbaB_EbfC 104 DNA-binding protein,
                     YbaB/EbfC family"
                     /product="YbaB/EbfC family nucleoid-associated protein"
                     /translation="MDFSQIMKQAQEMQKKLAEAQEKYIGKEFQGISGGGKVSVLVEV
                     IKIGSYKAKKVNIDLELMRNEEKDIVEDLVTAAFNDAVKKAEEDMANATSDLAGMMGL
                     PPGFKLPF"
                     /besthit="qcoverage 100 , hcoverage 100 , similarity 100
                     , identity 100 , evalue 1.39e-67 , alnlength 110 ,
                     Bacteria , Proteobacteria , WP_015589360.1 YbaB/EbfC
                     family nucleoid-associated protein [Wolbachia
                     endosymbiont of Drosophila simulans]"
ORIGIN
        1 ttttcttgct cccttgttag caggatctat tttgagtcta gctgttaaga gcatttatac
       61 tagaaatggg aaagagttta atcatcagac taatatttta ttattgttta actttgacca
      121 agaatgcgaa aatttttcaa agagtgatag gactaaatta aagtggctaa aaaattcagt
      181 tattttgagc aacacaatct gtacatcttt aatgtttttc ggtgtgggat tagatttagt
      241 tgccaacggc tttggtataa ctaactctat gatgttatcc ggcttattac tttcattacc
      301 attcacgctt ttaattgtta cttcacctat tttaaatgct gcaagtaatt atattctcaa
      361 aaagacagct ataaattcaa ttgaggagca atttaaaggt gatgatcagt ttatagaagt
      421 cgataacgaa aagccaagca gtactatgag caatcaaagt tatttcccca tcggtcagtt
      481 aatatcatca aagttagcta ttctccatag cgaagtctct agctaggaac aatgagccac
      541 agattagtat ggttttgacg ttttggatag aggcttttaa tatgtgattt gatattgcat
      601 cgccaattga ctcacactca attgctttta ttcctatgtt attggctttt tccctaatca
      661 agtttgtatt tgttgctttg ggctcagact taacacaaac cgcacatagt agcttgatat
      721 atggctttaa gtgctctaag aactcttcca catttctgtt acgagtaaca ccaaaaatta
      781 tatagatacc ttcagcaaaa ttatctttta cccactgaaa tagtactcta gcaccatcat
      841 tattgtgcgc accatctaaa aataattgcc aatcttttgg tagtaaagaa attaaattgc
      901 cctcttttat acgctccagt tgtgcaggcc aataagtgca ctgtaaacct gaagtgatat
      961 cctcttctcc aatatcaaat ccatattttc cactcaaaat gctacatgct gcaatagcat
     1021 tgcctgcatt gattatttgg tgatcaccct ttaaagacgg caaaggaaat tctattgatt
     1081 gaatagctga ttggaaaacc attttgttgt tcttttttt gcaattccat tcaaaacctc
     1141 ctctatataa gggagatttc ttatttattg cgtggtattc tagcgtgttc attatagatt
     1201 tttcttgtgg tgctatcacg caaggaacat taggtttcat tattccagcc ttttcacctg
     1261 ctataatttc tatagtaggg ccaagatatt ccgtatgatc aagtgcaatg gaagtaatta
     1321 ttgttaaaat cggactatct ataacatttg ttgtatcaag tcttccaccc atacccactt
     1381 caattaaagt tatatcggct ttgtgacgag aaaaagctaa aaaagctgca atagttgcag
     1441 cttcaaacag agtgataggt tgttctgcaa ttacggcacg gcattcttct aacaagctat
     1501 ataattcatt atcgtcgata taactgcctg caataactat tctttcatta aaattcacca
     1561 aatgtggaga agtgtatgcg tgtaccttat accctgctgc ttgcattata tatcttataa
     1621 atgctagtgt tgagcctttc ccattagttc cagctatatg aattacggga ggcacttttt
     1681 tctcgggatt atctagttta ctaagaaagc tttttatgcg atcaagagaa aaatcattcg
     1741 gcctgctacc aagtggctta ggccaatgag gcatatatat catagattat tgttacattt
     1801 taattcacct atcacacttg ctatgccatg cgtgtcaatc ttctcactaa attgagaacg
     1861 ttgattaata gcaatactta ttccactcat tacaacatca cttatcaaga aagaactgtg
     1921 atttttagta actttaaaat caatatttgt aaattcttca tcaccgtaag agaaccttgt
```

1981 gccaattaag caagtttcat cgtcaactgc ttttgtactc attatgatca tttcactatt
2041 actcatgtat ttagataaaa ttttaacgca caaacgtgta agatacactt catactctct
2101 taagaaatct tccctttcct tccgggtagt caaaacccaa tattttccca tgacaaatcg
2161 agatatttct ttgaggttaa cattattctg aatgacctcc tgaagctttt cgtgtacatg
2221 ctctaggttt tttctatttc cttttgtaga tatgctgtct acttgttgtt tcatagtatg
2281 cacaaaaatt ttgtgatctg tacaattagt gtaagcactt gaagaaatta caataaaaac
2341 aataatgatt aaaactttag taatattcat aagatatact taaaatgcaa agtttaataa
2401 agattaaata acaattactg actttaatat acctatagac atgccctgtg taaatcatct
2461 ataattcaag taggggtctg tgctatgcgt tatgagttaa gttaaatttc ccgagcgata
2521 atttctagag aagggaattg cctctgctgg tttcgttgaa actaatatgc gatgcccacc
2581 ttaacttcag gtctcaggaa tctactaagg ctgacggtag atacggatcc ttttttgatt
2641 ggtactaaat ttatgcttca tcaatacaga gaacaaggta ccttcctatg ttcgcctaat
2701 gaagtattcc aaattgtaat tgatgttgaa aggtatcctg attttgttcc ttggtgcaaa
2761 gccgtttata taaaagaaaa aattgacaat caaatggttg tggaccttct agcagctttt
2821 catggaatta aaggaagata tacatcagag gtaacctttc tttctccaag tagaacaaac
2881 gaaggttgga taaaagctgt atcatcaaat ggaatattta aacatctatg caataaatgg
2941 caattcattc ccatagatga aaataaaact atggtaaagt tttatataga gtttaagttt
3001 aagtccaact cattttcaac cctattaaac tcagtatata aatatacaca aagtaaaata
3061 attgctgcat ttaaagacag ggctgaaagc cttgctaaac aaaagttacc ttgacactat
3121 ttatataaat atataatttt tttagtaagt taagtattta tatcgtaatg atttttagac
3181 tattgctttt atcaattttt ataagtgtta attcttatgc agttgttaac caacatattc
3241 aaaaagaaac taatgaaata acatcaaagg agttactatc actattaccc gatgataaat
3301 tattaggaga tccaaaagca ccaattctaa tgatagaata tgcttcatta acttgctatc
3361 actgctctct ttttcataag aatgtttttc ctaaaataaa agagaaatat atagacacag
3421 gtaagatgtt atatatattt cgtcattttc ctttggatta cagagggcta aaggctgcaa
3481 tgctaagtca ttgctacgaa aaacaagaag actactttaa ttttaacaaa gctgttttta
3541 actcaataga ttcgtggaat tactacaatt taagtgattt gactttacta caaagaattg
3601 ctgcactaag caacttaaag caagatgcat ttaaccaatg cattaatgat aagaaaatta
3661 tggataaaat cataaatgat aaatcactgg caattaataa acttggtatc acagctaccc
3721 caatattttt catcaagctt aacgatggta agtcatatat agagcataat aaagttaaac
3781 atgaagggta taaagagctg aaatacctca ctgatgtaat agataaatta tatggaaaag
3841 ctatagtgaa gtaataccac tgtagcttac catataaaag actctatgct gagtcattgt
3901 tttttcacga ctgtttgttg tttttttacgc tcaaactctc tttttttact gtgccaagcg
3961 tagtaataca tagcaatttt attctctatt aatactattg ttccatcata aaactgaacc
4021 atatccataa cttttctctg agcttcatca aaacgttctt tatacttttc acgataagct
4081 ggattattta gtaacataac cccttcattc ttgttagatt ttttgtttaa cgtttcaact
4141 tcgctagcaa tagtataaga catataaccc cctctaattg attataaaaa ttatatttta
4201 ataaagagaa atgcgcataa acaatctctt taattccagc acaataatcg actgcttatt
4261 atactagtta actatagtac aatatataat aattcttgtc acttaaaatt ttatactatg
4321 aaataataac ttaaaataaa tgtttttatt atttcaaact atattattat atttataaat
4381 tgtgtatgaa ttaagcttcc ctaatttata atctatgcgt aataatttta gtataaaatt
4441 gtcatacata agaaatctat ttgcaaaaga atttaaaaca atagaagaat attgtattct
4501 tgaaaaaaag caacatattc agatcggcca tgaagaagga aagctattaa gtttatttat
4561 aaaaatccat aaaatcaaaa gtattgttga gattggcacg ttatacggtt attcgtcgat
4621 ttgcatggca aaagctctac tggaagatgg tcacatatat acaatagaaa ataaccctca
4681 atactcaaga atagcaaaaa aaaattttag tgcttttaat ctgagtgata aaattactct
4741 aatagaaggc aatgcattgg aaaaacttaa tgaattatca gcaaaagcac catttgacat
4801 gatatttatc gatgttgata aaggtggtta tcctaaatat ttagattggg cagagtcata
4861 cattaaacaa gatgggctaa tcgttgcaga taacactcta ttgtttaaca cagtattttt
4921 agaatcgcct ccaaaagaag tatcagaaaa atcatggcat gctatgaggg agtttaataa
4981 tagattatca gatgaaaaaa aatatttctc tatattgatt ccaactgatg aaggaatgac
5041 tgtagcttta aagctaacat aagttaaaaa tcaaggtcag cataatgttt aggagggatt
5101 aaaccagaaa tcctctctaa taatatctca cgaaaacaag gctttgattt aacaatggag
5161 taccattctt ttaaaatttt actttttttcc catggaaaac tacttacata gtctattaca
5221 gaaatatgcg atgctaaagt aatatcagcc aaagtgaact tatcggttgc aagccaaatg
5281 ttcttgttaa ttaagtgttc aacatattct atatggcaaa gcagattatg ctgagctgca
5341 tagataaacc tagaatcagg gctgcggtta gtaattactt tttcattcat aatatactta
5401 gtaacttcat tgtaaaattt gttatcgaac caattgatta gagcgcgtat tttagactta
5461 ataatagtgg atgaaccaaa taatttgacg tcgctattgt aagtttcttc tatatattca
5521 caaatggcat tactatccgc tattacaaag ttattatcta ttaatactgg tacttgcccg

```
     5581 gttggattga tctccataaa ttcattccgt ttttcccacg gattttcata cactagatca
     5641 caacccaact ttttttcttt gagaagggct ctaacttttc gtgaaaatgg acaaagagga
     5701 aaatgatata aaatcataat attcctcttt tgttatccca gtacctatag gttgttattc
     5761 cagtgtgtga cactgaaatc cacatctttt ttctctggat ccaagttggg atgacaagag
     5821 aagagtattt taggtaacag ttgtttactc caaagtaatg ttgcaagagg tctaacatat
     5881 aaattataag actaatatag gatatacctt gctaaataaa aaaaaagtat aatattttat
     5941 aaggtatgca ggcaaaaact ctgtaatttg gtcagatcag aaatgaagca gccatatcag
     6001 aacctttttg ggttgcatac ctacttcatt atttgtttta tccgaagctg tgaatatagc
     6061 attaaaacat cgtcctagta gctttaaaga tctagtaggt caagatatat tagtgcgtgt
     6121 attagaaaat gcttttattt taaacaaaat cccacaatct atactgctct ctggtagtag
     6181 tggagttggt aaaaccacca ctgcaaggat aatagcttta tgtttgaatt gttccctggg
     6241 gccaattttt gaaccttgcg gatcatgtga aaattgtcta gcaataaaaa attcaagcca
     6301 tccagatgtg attgaaatcg atgcagcaag tcacactagc attgacgata ttaaagtaat
     6361 cctgggagat atttgctatt cacctataag ctctaaattc aaagtctaca ttatagatga
     6421 agtacatatg ttatccagca gtgcatttaa cgcattactt aaaaccctag aagagccacc
     6481 atctagtgta aaattcattt tagcaactac ggaaataaaa aagataccga taactattat
     6541 tgcacgttgt caaagatttg atttacacaa cattcctgta gctaaaatag tggaacgttt
     6601 aaatgatgtt gcacaaaaag agagttattc tattgaaaag ggagcattag aattaattgc
     6661 acgacactct ggaaattcaa tgcgtaacgc cttgtttttg atgaatcaag cagtgctata
     6721 tagcaaagat ggtgcaatat ccactaaaaa tgtgacagac atacttggcc tagtggataa
     6781 agatattata tttgatctat taggggcaat attagaaggt gatttacaga aagctctatc
     6841 agtgtttgac aaggcgataa aaacagcaaa cccacttagt atttttgaag gtctattgca
     6901 aacaattcag ttagtatgcc gtttttttaat tacgaaagag attgatagtg cagttacaga
     6961 gtatgaaaag aataggataa aagatttaag tataaagaga tctttaatat ttttttcaag
     7021 attgtggaag gtgttgctta aaggtattca agatataaaa acttcaacat gcaacgatgt
     7081 tgctgctgaa atgatgctaa ttagtctttg ctatctctct gatctacctt ctccagaaca
     7141 ggtgatcaaa aaagttcttt cacaaaatgt agagcaagga aatagacagg gcagacctgt
     7201 tgcaccttcc ccctctgtca tccaagtagc tgactactca gatccaaaaa aatatgtgga
     7261 aagtaaacaa caaccccact ctgtcatccc agtgcgtgac actgggatcc atgtggaaaa
     7321 taaacaacaa aattacgatt ttgataagat tttgcaacta ttaaggcgga gtaaccaaat
     7381 ttatctttac aaacaactgt gtaataatct acaattaata gattgtaaac ctgggtattt
     7441 aaaattaaaa gctgtatcta agttggacag taatttttgc aatgatttaa aaaattactt
     7501 aaaccaggca actcagcagg agtgggttat tacagttgat acgggttata taaacaggga
     7561 agtgagtaat ttaaattatg cacctgcagt caaggatata cttgacatgt ttaaaggtgc
     7621 aaaagtagtt aatatagaaa ataaggagta agttgtggat tttagtcaaa taatgaaaca
     7681 agcgcaagag atgcaaaaaa agcttgcaga agctcaagaa aaatatattg gaaaagagtt
     7741 tcaaggtatt tctggtggtg gcaaagtttc tgtattagtg gaagtcataa aaataggtag
     7801 ctataaagct aaaaaggtga acatagactt agaacttatg agaaatgagg aaaaagatat
     7861 agtagaggat ttagtaacag cagcgtttaa tgatgccgtt aaaaaagcag aagaagatat
     7921 ggcaaatgca acttctgatc tcgcaggtat gatgggttta ccacctggat ttaaacttcc
     7981 tttttgaatt tactactgaa gtttctttta gtgtaagtat tttttttcta atactagact
     8041 tctttatata atgcgcctat agctagcaca gccctatgtc ataccgcaat tcattccaca
     8101 actgtacgaa cattgcaata tggcacatag taaacgatgt cattccagtc tggaatccag
     8161 ccttgccgta atctcatcaa gaacgttgtg ttttaacata aaacggctac ttttatgctt
     8221 accaacttaa taaaattcct ggatcccaga ctgggatgac aagaagatg
//
LOCUS       JAQZAU010000025           6079 bp    dna     linear   ENV 17-MAR-2023
DEFINITION  MAG: Wolbachia pipientis isolate wEsol contig_21001, whole genome
            shotgun sequence.
ACCESSION   JAQZAU010000025  JAQZAU010000000
VERSION     JAQZAU010000025.1
DBLINK      BioProject: PRJNA929258
            BioSample: SAMN33142973
KEYWORDS    WGS; ENV; Metagenome Assembled Genome; MAG.
SOURCE      Wolbachia pipientis (insect metagenome)
  ORGANISM  Wolbachia pipientis
            Bacteria; Pseudomonadota; Alphaproteobacteria; Rickettsiales;
            Anaplasmataceae; Wolbachieae; Wolbachia.
REFERENCE   1  (bases 1 to 6079)
  AUTHORS   Fiutek,N., Couger,B.M., Roy,S., de la Torre,J.R. and Connor,E.F.
```

```
  TITLE     Genomic assessment of the contribution of the Wolbachia
            endosymbiont of Eurosta solidaginis to gall-induction
  JOURNAL   International Journal Molecular Sciences
REFERENCE   2  (bases 1 to 6079)
  AUTHORS   Fiutek,N., Couger,B.M., Roy,S., de la Torre,J.R. and Connor,E.F.
  TITLE     Direct Submission
  JOURNAL   Submitted (10-FEB-2023) Biology, San Francisco State University,
            1600 Holloway Ave, San Francisco, CA 94132, USA
COMMENT     ##Genome-Assembly-Data-START##
            Assembly Date          :: JUN-2019
            Assembly Method        :: FLYE v. 06-2019
            Genome Representation  :: Full
            Expected Final Version :: Yes
            Genome Coverage        :: 100.0x
            Sequencing Technology  :: Illumina HiSeq; PacBio Sequel
            ##Genome-Assembly-Data-END##
FEATURES             Location/Qualifiers
     source          1..6079
                     /organism="Wolbachia pipientis"
                     /mol_type="genomic DNA"
                     /submitter_seqid="contig_21001"
                     /isolate="wEsol"
                     /isolation_source="galls on Solidago altissima"
                     /host="Eurosta solidaginis MN-2018-EFC"
                     /db_xref="taxon:955"
                     /environmental_sample
                     /country="USA: Cowling Arboretum, Carleton College,
                     Northfield, MN"
                     /lat_lon="44.48 N 93.13 W"
                     /altitude="280 m"
                     /collection_date="2018-11"
                     /metagenome_source="insect metagenome"
                     /note="metagenomic"
     gene            complement(251..1711)
                     /locus_tag="wEsol_00186"
     CDS             complement(251..1711)
                     /locus_tag="wEsol_00186"
                     /codon_start=1
                     /transl_table=11
                     /fullproduct="qcoverage 100 , hcoverage 100 , similarity
                     100 , identity 100 , evalue 0.0 , alnlength 486 ,
                     Bacteria , Proteobacteria , WP_015589085.1 MULTISPECIES:
                     TrkH family potassium uptake protein [unclassified
                     Wolbachia]"
                     /cog="TrkG COG0168 499 Trk-type K+ transport systems,
                     membrane components"
                     /pfam="Pfam-B_14546 Pfam-B_11327 TrkH"
                     /tigr="TIGR00933 2a38 391 potassium uptake protein, TrkH
                     family"
                     /product="TrkH family potassium uptake protein"
                     /translation="MKNHQRLQTLRSIAFIVGIFLLLFSLAMLIPAITNKYLSYEWKN
                     FFAGFIITCTSGAIFILLGKLSRLHGMPAIFAITSCTWIALSLFAAIPFYFDNLSYVD
                     ALFETISGITTTGATIFNDIEKQSPGILLWRAMLHGMGGFGVITLGIAVFPMFKVLSL
                     NNLLYSEYSDATKRKLPNTRSVVIHVTAIYYGLILLCIFSYYLAGMPLFDAICHGMSA
                     VSTGGFANYNDSIGHYNNPILEAITIVFMILGSLPFLSYLKIIRRLDICYDEQVSYFV
                     KIATISSLFACFWLYKNVDLGVSLSFRCSTFTITSLITSTGYAICNHIDWSFISVLAF
                     FLTFVGGCSGSSSGGVKIFRLIVLLRSIRNYFYLLLNPDADNGVKFNGKILENDEVHS
                     VFIFFAIFMLTFTISSIVMSYLSNADFITSISSVSATLTNSGPGFSNLIGPSGNYSSF
                     SNEVKLFLSFLMLLGRLEILPIYFCIGNLFLFNREK"
                     /besthit="qcoverage 100 , hcoverage 100 , similarity 100
```

, identity 100 , evalue 0.0 , alnlength 486 , Bacteria , Proteobacteria , WP_015589085.1 MULTISPECIES: TrkH family potassium uptake protein [unclassified Wolbachia]"
gene 1853..2023
/locus_tag="wEsol_00187"
CDS 1853..2023
/locus_tag="wEsol_00187"
/codon_start=1
/transl_table=11
/fullproduct="qcoverage 100 , hcoverage 100 , similarity 100 , identity 100 , evalue 1.17e-28 , alnlength 56 , Bacteria , Proteobacteria , WP_167482803.1 MULTISPECIES: hypothetical protein [unclassified Wolbachia]"
/translation="MVELTDAQEEILGSLRQAVRNDNFQEVQNVLNNKEHNDIQAVLS
HEGTLTSDKGTI"
/product="hypothetical protein"
/besthit="qcoverage 100 , hcoverage 100 , similarity 100 , identity 100 , evalue 1.17e-28 , alnlength 56 , Bacteria , Proteobacteria , WP_167482803.1 MULTISPECIES: hypothetical protein [unclassified Wolbachia]"
gene 2119..3105
/locus_tag="wEsol_00188"
CDS 2119..3105
/locus_tag="wEsol_00188"
/codon_start=1
/transl_table=11
/fullproduct="qcoverage 100 , hcoverage 100 , similarity 100 , identity 100 , evalue 6.65e-230 , alnlength 328 , Bacteria , Proteobacteria , WP_015589084.1 MULTISPECIES: hypothetical protein [unclassified Wolbachia]"
/translation="MCDYKVTKSFEDQPYFTDTIDDLKRFQGKFDPHSTEYFALVQVV
QKIEDHKWVMDLDSNLQEAIENLDIDQVKAALENCGEDVERVLEREVYWSSGRVDALL
VYPLKVEYSEELNQEDVEKIKESFKLMFDAASPGLLGFEPNGNNYVMDKLTCAQKRFA
KIPGVGEQCFQDLIQEFEEKLSKNELDNAKINDQIAWDKEYATRFSQEINQDPSENDD
LNFIDDTDLDQNTVNSAPTADNKTPEKNDTNFWSEHKGKIALGVVELCVVGAIAAYVL
AYPVVALVLEVLAVSALAVLAYIGGVCEKSESPNTKSSDPVVSGVLQQRRTV"
/product="hypothetical protein"
/besthit="qcoverage 100 , hcoverage 100 , similarity 100 , identity 100 , evalue 6.65e-230 , alnlength 328 , Bacteria , Proteobacteria , WP_015589084.1 MULTISPECIES: hypothetical protein [unclassified Wolbachia]"
gene complement(3185..4201)
/locus_tag="wEsol_00189"
CDS complement(3185..4201)
/locus_tag="wEsol_00189"
/codon_start=1
/transl_table=11
/fullproduct="qcoverage 100 , hcoverage 100 , similarity 100 , identity 100 , evalue 7.92e-243 , alnlength 338 , Bacteria , Proteobacteria , WP_015589083.1 MULTISPECIES: substrate-binding domain-containing protein [unclassified Wolbachia]"
/cog="PstS COG0226 318 ABC-type phosphate transport system, periplasmic component"
/pfam="PBP_like_2 PBP_like"
/tigr="TIGR02136 ptsS_2 295 phosphate binding protein"
/product="substrate-binding domain-containing protein"
/translation="MLRNFLLIFVFVLFTPPSNADARKYIRIVGSSTVFPFISFIAEE
FNRVFPFKTPVVESIGSGSGFKMFCSGIGEDTPDITTSSRPMKEVERELCKRNKINEV
IEIIIGYDGIVIANSNQSHRFDFTKKDLFETLSAYSQENDKLVKNNKKFWSDVNQALP

```
                     KTEIEIYGPHQNTGTYETLVNSIMLDQYSCMNSRIFKENYKDQEERKKACGNIRDDGR
                     YIEVGINENIIIQKLKSNKNALGIFSFSFLMRNQDKIQGSTIAGIEPTYENISSGKYI
                     LARPLYLYIKKEHLDTVDGLREFIKEIIDSIGIEDGYLSRLGLIPLSSEDIKKASAKV
                     YDIT"
                     /besthit="qcoverage 100 , hcoverage 100 , similarity 100
                     , identity 100 , evalue 7.92e-243 , alnlength 338 ,
                     Bacteria , Proteobacteria , WP_015589083.1 MULTISPECIES:
                     substrate-binding domain-containing protein [unclassified
                     Wolbachia]"
     gene            4343..4900
                     /locus_tag="wEsol_00190"
     CDS             4343..4900
                     /locus_tag="wEsol_00190"
                     /codon_start=1
                     /transl_table=11
                     /fullproduct="qcoverage 100 , hcoverage 100 , similarity
                     100 , identity 100 , evalue 6.36e-134 , alnlength 185 ,
                     Bacteria;Eukaryota , Proteobacteria;Arthropoda ,
                     WP_006280485.1 MULTISPECIES: dCTP deaminase [Wolbachia]"
                     /cog="Dcd COG0717 183 Deoxycytidine deaminase"
                     /pfam="dUTPase"
                     /tigr="TIGR02274 dCTP_deam 180 deoxycytidine triphosphate
                     deaminase"
                     /product="dCTP deaminase"
                     /translation="MAVMPDKWIREKAENFGMIEPFVNHKSSKGVISFGLSSYGYDAR
                     VDNKFKIFTNVNSAIVDPKNFSENSFIDKETDVCIIPPNSFVLASTVEYFRIPRNVLV
                     ICVGKSTYARCGIIVNVTPLEPGWEGHVTLEFSNTTPLPAKIYANEGACQFVFLSGES
                     ECEKSYDDMKGKYMNQHGITLPLVK"
                     /besthit="qcoverage 100 , hcoverage 100 , similarity 100
                     , identity 100 , evalue 6.36e-134 , alnlength 185 ,
                     Bacteria;Eukaryota , Proteobacteria;Arthropoda ,
                     WP_006280485.1 MULTISPECIES: dCTP deaminase [Wolbachia]"
     gene            complement(4906..5928)
                     /locus_tag="wEsol_00191"
     CDS             complement(4906..5928)
                     /locus_tag="wEsol_00191"
                     /codon_start=1
                     /transl_table=11
                     /fullproduct="qcoverage 100 , hcoverage 100 , similarity
                     100 , identity 100 , evalue 2.73e-235 , alnlength 340 ,
                     Bacteria;Eukaryota , Proteobacteria;Arthropoda ,
                     WP_141456986.1 MULTISPECIES: hypothetical protein
                     [unclassified Wolbachia]"
                     /translation="MPAVVNTPDNIQRPSKLKKAKEILLAPVRWLIIGLSVVLLIIFV
                     ISFLVFLSIANVLSKLYLFAGGKAPEWLEQRAEAFMEAARIPQTTHDTDVNDAININV
                     QKLKENYGEPDIDDKFIEEIEGFIDQLECIANGTQKAADKDQLTEDEKKHALECLKRI
                     KNNPGTHSGSGLTLKQVLNLVWKACNDQTRANNIAINTRISQRKYQLVKHLVESQTEY
                     GVRGGRGSSACFTGTFNSIVSSLYTFEEFSFLQKADKNTIKQEFENELQKKGEELFNQ
                     LSNDKEKKEVAEALSNPSTNFFSSYVESFKNHSARAVLGDKECDKLVKTHFSNLQYNK
                     TISRFT"
                     /product="hypothetical protein"
                     /besthit="qcoverage 100 , hcoverage 100 , similarity 100
                     , identity 100 , evalue 2.73e-235 , alnlength 340 ,
                     Bacteria;Eukaryota , Proteobacteria;Arthropoda ,
                     WP_141456986.1 MULTISPECIES: hypothetical protein
                     [unclassified Wolbachia]"
ORIGIN
        1 tctatagcta taccgccgcg aaccgtcatg ccgcgattca ttcgcggtat ctcttagcat
       61 agatcccgct aacaagtagc gggatgacga gaaaggggct acttggatga cattcttcct
      121 ggtgtgccca aatcacaatg ttcgtacatc tgtggattta ttcgcggtat agatatcgct
```

181 aacgagtagc ggaatgacgg tttttcaaat tgtcgtaagt cagtttagct ataatatcaa
241 gtttcaccaa ctatttctct ctattaaata agaataaatt acctatacaa aaatagattg
301 gcaatatttc aagtctgcca agcagcatta aaaacgatag aaataatttt acttcattac
361 tgaaagagga ataattaccc gaaggaccta ttaaattact aaatcccggg ccagagtttg
421 taagcgttgc agagactgaa ctgatgctag ttataaagtc tgcattgctt aagtaagaca
481 tcactattga tgaaatggta aacgttaaca tgaaaattgc aaaaaatata aagacagagt
541 gaacttcatc attctccaga atttttccat tgaattttac tccattgtct gcatcagggt
601 ttaataaaag gtaaaagtaa tttcttatag accttaaaag aacaatcaag cggaagattt
661 tgacaccacc acttgaagaa ccgctacatc caccaacaaa ggttagaaaa aaagctaaga
721 ctgaaatgaa actccaatct atatgattac agattgcata accagttgac gtaatcaagg
781 aagtgatggt aaatgtgctg catctaaatg ataatgacac tcctaaatca acatttttat
841 acaaccaaaa acaagcaaat aaagatgaga tagtagctat cttaacaaag taagagacct
901 gttcatcata gcaaatgtct aaccgtctta taatttttaa atagctaaga aaaggcagag
961 aacccaaaat cataaaaacg atcgttatgg cttctagtat gggattatta tagtgcccta
1021 tagaatcatt atagttagca aatccaccag ttgatacagc agacattccg tgacatattg
1081 catcaaatag tggcatacca gctagataat aggaaaatat gcataacaaa attaagccat
1141 aatatattgc cgtaacatga atcactacgc ttcgcgtgtt cggcaacttc cttttggtag
1201 catctgaata ttcagaataa agtaaattat ttaggctgag aaccttaaac ataggaaaaa
1261 ctgcaattcc taaagtgatt acgccaaaac cccccatgcc gtgtagcatt gctctccaca
1321 gcagtattcc tggagattgt ttttcaatat cattaaatat agttgcccct gtggttgtga
1381 tgccagatat cgtttcaaac aatgcatcaa catagctcag attatcaaaa tagaaaggaa
1441 tagctgcaaa cagagacaag gcaatccagg tgcaactagt aatcgcaaaa attgctggca
1501 ttccatgcaa tctacttaat ttaccgagta aaataaaaat tgcgccagat gtgcaggtaa
1561 ttatgaatcc agcgaaaaaa tttttccatt cgtagctgag atacttattg gtaatagcag
1621 gaataagcat tgctaagcta aatagcaata ggaatattcc cacaataaat gcaattgaac
1681 gtaaagtttg taaacgttga tgatttttca tagagttact tcctcgctat acaaagccat
1741 aaaagctact ctaacgtcaa gcattttgtt ggcctttatt aacagttatt aacaatatac
1801 ttgatttact tgtaaatatg tatgataatt aaatttttat tagaggtaag atatggttga
1861 attaactgat gcacaagaag aaattctagg ttcattaaga caggcagtca ggaatgacaa
1921 ttttcaagaa gtccagaacg tactcaacaa taaggaacat aatgatatac aagcagtttt
1981 gagccacgag gggactctga ctagtgacaa aggtaccatt taaatgatgt tattggctgt
2041 actaaaacta gcgttaacac taaagtaggg atcattgaac taatttggaa ggaagctgat
2101 caaaaaactc gtgatttttt gtgtgattac aaggtaacaa aatcttttga agatcaacca
2161 tattttacgg atacaataga tgatcttaaa cgctttcaag gaaaatttga ccctcatagc
2221 acagaatatt ttgctcttgt acaagttgtt caaaaaatag aagatcacaa gtgggtaatg
2281 gatcttgatt ccaacttaca agaagcaata gaaaaccttg atattgatca agttaaagca
2341 gcattagaga attgtggtga agatgtagaa agggttttag agcgagaggt atattggagt
2401 agtggaagag tagatgccct tttagtttat cctctgaaag ttgaatatag tgaagaattg
2461 aaccaagaag atgtggaaaa gataaaagaa agctttaaat taatgtttga tgctgcttct
2521 ccagggctcc ttgggttcga gcccaatgga aataactatg taatggataa acttacatgt
2581 gcacaaaaac gttttgctaa aatacctgga gtaggtgagc aatgctttca agatttgatt
2641 caagaatttg aggaaaaact tagtaaaaat gaactagata atgccaagat taatgatcag
2701 atagcatggg ataaagagta tgcgactcga ttttcacaag aaatcaatca ggatccgtct
2761 gagaatgatg atcttaactt tatagatgac actgatctag atcaaaacac cgtaaactct
2821 gcaccaactg cagacaacaa aaccccagaa aaaaacgata caaacttctg gagtgaacac
2881 aaagggaaga tcgctctggg cgttgttgaa ctatgcgtag ttggtgctat tgcagcctat
2941 gtgctggcat atcctgtagt tgcattagtt ttagaagttt tagcagtttc agcattggct
3001 gtactagctt atattggagg agtttgtgag aaatcagaaa gcccaaatac gaaaagctcg
3061 gacccggttg tttctggggt gttgcaacaa cgcagaactg tctaattagt aatccattgt
3121 catcccagcg cgccgcgctg ggtctgacta cgctagaaca aagaatataa aagaaacgtt
3181 gccgtcacgt tatatcataa acctttgctg aagctttctt tatatcttca cttgaaagtg
3241 gaattagacc aagcctagat aggtacccat cttcaatgcc gatcgaatct ataatttctt
3301 ttatgaattc tcttaatcca tcaacagtat ccaggtgttc tttctttata taaaggtata
3361 gaggtcttgc taatatatat tttcccgatg atatattttc gtaagttggc tcaattcctg
3421 caattgtgct cccttgtatt ttatcttgat tcctcattaa aaagctaaaa ctaaatatcc
3481 ctaaagcgtt cttgttactt ttcaattttt gtattattat gttttcatta attccaactt
3541 ctatatacct tccgtcatcc cttatattac cacatgcttt ctttctttct tcctgatctt
3601 tataattctc tttgaaaatt ctcgagttca tgcatgaata ttgatcaagc ataatagaat
3661 taaccaaagt ttcgtatgtg cctgtatttt gatgtggacc ataaatttca atttctgttt
3721 ttggtaaggc ttgatttaca tcagaccaaa acttcttatt atttttact aatttatcat

```
     3781 tttcttgaga atatgcagat aaagtttcaa ataagtcctt ctttgtgaaa tcaaatctat
     3841 ggctttgatt tgaatttgca atgacaattc catcatagcc aatgatgatc tctatcactt
     3901 cattaatttt atttctttta catagttctc tttctacttc cttcataggg cgagatgaag
     3961 tggtgatgtc tggcgtgtct tcccctattc ctgagcaaaa cattttgaac cctgatccac
     4021 ttcctattga ttccacaact ggagtcttaa aagggaatac acgattgaac tcctcggcta
     4081 taaacgagat aaaaggaaaa acagttgaag atccaacaat tctgatgtat ttcctggcat
     4141 cagcatttga cggcggcgtg aataatacga atacaaaaat caggagaaag tttctaagca
     4201 ttatttttag ttaagaacat aaatctaatt attgtaacaa aacatcaaac tgaaaagctt
     4261 taattgcatg acaaaagcta cagtttacat ttaaatttta ttttatatta ttgtaagtat
     4321 tacacaactt taaggaacag ttatggcagt tatgccagat aaatggataa gagaaaaagc
     4381 tgaaaacttt ggaatgatag agccttttgt gaatcacaaa agcagcaaag gtgttatatc
     4441 tttcggacta tcatcttacg gttacgatgc aagagtggat aataaattta aaatttttac
     4501 taacgttaat tcagccatcg tggaccctaa aaatttttct gagaatagtt ttatagataa
     4561 agaaacagac gtatgcataa ttccaccaaa tagctttgtg cttgcaagta cagtggaata
     4621 ttttcgtata ccaaggaatg tactggtcat ttgtgttggt aaatcaactt acgcaagatg
     4681 tggaattata gtaaacgtta ctcccttaga acctggatgg gaaggtcatg tcacactcga
     4741 attctcaaac accactccac ttcctgcaaa aatttacgct aatgaaggcg catgccaatt
     4801 cgtattttta agcggcgaaa gtgaatgcga gaaatcatat gatgatatga aaggaaaata
     4861 catgaatcag catggtatca ctttgccgtt agttaagtga ttactttagg taaagcgtga
     4921 tattgtttta ttgtactgaa gattactaaa atgagtttta actaatttgt cgcattcttt
     4981 atcacctaaa actgcacgtg cagagtggtt tttaaatgac tctacatagc ttgaaaagaa
     5041 atttgtagaa ggattactta aagcttctgc aacttctttt ttttctttat cattggaaag
     5101 ctgattaaat agttcttctc cctttttttg cagctcattt tcaaattcct gctttatagt
     5161 attcttatct gctttttgca agaaggaaaa ctcttcaaaa gtgtacaagg aagagacaat
     5221 actgttaaat gtgccagtaa aacatgcact tgaaccacga ccaccacgca ctccgtattc
     5281 tgtttgcgat tctactaaat gtttaaccag ttgatatttt ctctgtgata tgcgggtatt
     5341 aatagctata ttatttgctc tagtttgatc gttgcaagcc ttccaaacta aatttaaaac
     5401 ttgcttcaat gttaacccgc ttccactatg tgtaccaggg ttattttta tacgcttcaa
     5461 gcactcaagt gcatgttttt tctcatcttc tgtaagttga tctttatctg cagcttttg
     5521 tgtgccattt gctatgcact ctaactgatc aataaatcct tctatttcct ctataaactt
     5581 atcatcaata tcaggctcac cgtagttttc ttttagcttt tgaacgttaa tattaattgc
     5641 atcatttaca tcagtatcat gcgttgtttg tggaatacgt gctgcctcca taaatgcttc
     5701 agctctttgc tctaaccatt ctggtgcttt gcctcctgca aaaagatata gcttactcaa
     5761 tacattggcg attgagagaa acaccaggaa agaaataaca aatattatca aaagtactac
     5821 agataaacct atgattagcc accttacagg agctagcaga atttcttttg cttttttaa
     5881 ttttgacggt ctttgtatgt tgtctggtgt atttactact gctggcataa ttagtttaag
     5941 ttaatatcaa tattattaat atactatatt ttttcttatt ttgtcaactc aaatttctac
     6001 ttccgcactt tcttattgtt gcttacaatg attatagcta caccatgtgc ttttcttaat
     6061 tacaaacttc aactattca
//
LOCUS       JAQZAU010000026          741 bp    dna     linear   ENV 17-MAR-2023
DEFINITION  MAG: Wolbachia pipientis isolate wEsol contig_22294, whole genome
            shotgun sequence.
ACCESSION   JAQZAU010000026 JAQZAU010000000
VERSION     JAQZAU010000026.1
DBLINK      BioProject: PRJNA929258
            BioSample: SAMN33142973
KEYWORDS    WGS; ENV; Metagenome Assembled Genome; MAG.
SOURCE      Wolbachia pipientis (insect metagenome)
  ORGANISM  Wolbachia pipientis
            Bacteria; Pseudomonadota; Alphaproteobacteria; Rickettsiales;
            Anaplasmataceae; Wolbachieae; Wolbachia.
REFERENCE   1  (bases 1 to 741)
  AUTHORS   Fiutek,N., Couger,B.M., Roy,S., de la Torre,J.R. and Connor,E.F.
  TITLE     Genomic assessment of the contribution of the Wolbachia
            endosymbiont of Eurosta solidaginis to gall-induction
  JOURNAL   International Journal Molecular Sciences
REFERENCE   2  (bases 1 to 741)
  AUTHORS   Fiutek,N., Couger,B.M., Roy,S., de la Torre,J.R. and Connor,E.F.
  TITLE     Direct Submission
```

```
  JOURNAL   Submitted (10-FEB-2023) Biology, San Francisco State University,
            1600 Holloway Ave, San Francisco, CA 94132, USA
COMMENT     ##Genome-Assembly-Data-START##
            Assembly Date          :: JUN-2019
            Assembly Method        :: FLYE v. 06-2019
            Genome Representation  :: Full
            Expected Final Version :: Yes
            Genome Coverage        :: 100.0x
            Sequencing Technology  :: Illumina HiSeq; PacBio Sequel
            ##Genome-Assembly-Data-END##
FEATURES             Location/Qualifiers
     source          1..741
                     /organism="Wolbachia pipientis"
                     /mol_type="genomic DNA"
                     /submitter_seqid="contig_22294"
                     /isolate="wEsol"
                     /isolation_source="galls on Solidago altissima"
                     /host="Eurosta solidaginis MN-2018-EFC"
                     /db_xref="taxon:955"
                     /environmental_sample
                     /country="USA: Cowling Arboretum, Carleton College,
                     Northfield, MN"
                     /lat_lon="44.48 N 93.13 W"
                     /altitude="280 m"
                     /collection_date="2018-11"
                     /metagenome_source="insect metagenome"
                     /note="metagenomic"
     gene            complement(<1..215)
                     /locus_tag="wEsol_00192"
     CDS             complement(<1..215)
                     /locus_tag="wEsol_00192"
                     /codon_start=1
                     /transl_table=11
                     /product="hypothetical protein"
                     /translation="MTHQVNDSKLKEKDEKNILKLASKIKGKKSSIAEELEKISKKEK
                     QRILTTEVSKHRGKEPTIISLAYAILR"
                     /besthit="qcoverage 100 , hcoverage 75.531914893617 ,
                     similarity 100 , identity 98.6 , evalue 4.26e-35 ,
                     alnlength 71 , Bacteria , Proteobacteria , RLT59986.1
                     hypothetical protein WANA31_0645, partial [Wolbachia
                     endosymbiont of Drosophila ananassae]"
     gene            665..>741
                     /locus_tag="wEsol_00193"
     CDS             665..>741
                     /locus_tag="wEsol_00193"
                     /codon_start=1
                     /transl_table=11
                     /product="hypothetical protein"
                     /translation="MTEETKDGYASLYVAIQEGNIEAAE"
                     /besthit="qcoverage 100 , hcoverage 13.3689839572193 ,
                     similarity 100 , identity 100 , evalue 1.14e-06 ,
                     alnlength 25 , Bacteria , Proteobacteria , WP_179949445.1
                     ankyrin repeat domain-containing protein, partial
                     [Wolbachia endosymbiont of Drosophila santomea]"
ORIGIN
        1 ttacgtagta tagcataagc aagtgatata atagttggtt ctttaccccg atgtttgctt
       61 acttccgtag taagaatcct ttgtttttcc tttttagata ttttttctag ttcttcagca
      121 atgctacttt ttttgccttt tatttttgat gctaatttta aaatattttt ttcatctttc
      181 tctttcaatt tcgaatcgtt tacctgatga gtcatttttt ttctcaaggt ctttcttatt
      241 aaactaagta taacatttcc tctcttcgtg gttataatat ttgcttttat gaatcagttg
```

```
      301 tgttgataat gtattaattg catagttcag acattgtttt ataaagcaaa attttagtag
      361 accaccttac aagaacgttt cttgctaatt ctgaatccga aaaaaggacg cagtaaagtt
      421 atttgtaagg gaagaatgaa acttgtagct atagctaaac tgacttacga taatttgaaa
      481 aaccgtcatc ccgctgcttg ttagcgggat ctagagatac cgcggcggta tgacggttcg
      541 cagtggtatg acggttcgcg gcgggatgac gataagctcg tcatcccgct acattaatat
      601 tgtattaata aaaacttaat aataatcctt aataatatat gaactaacat gttcgcaatt
      661 tcgtatgaca gaggaaacaa aagatggata tgcttcctta tatgttgcaa ttcaggaagg
      721 taatattgaa gctgcagaac t
//
LOCUS       JAQZAU010000027          529 bp    dna     linear   ENV 17-MAR-2023
DEFINITION  MAG: Wolbachia pipientis isolate wEsol contig_23028, whole genome
            shotgun sequence.
ACCESSION   JAQZAU010000027 JAQZAU010000000
VERSION     JAQZAU010000027.1
DBLINK      BioProject: PRJNA929258
            BioSample: SAMN33142973
KEYWORDS    WGS; ENV; Metagenome Assembled Genome; MAG.
SOURCE      Wolbachia pipientis (insect metagenome)
  ORGANISM  Wolbachia pipientis
            Bacteria; Pseudomonadota; Alphaproteobacteria; Rickettsiales;
            Anaplasmataceae; Wolbachieae; Wolbachia.
REFERENCE   1  (bases 1 to 529)
  AUTHORS   Fiutek,N., Couger,B.M., Roy,S., de la Torre,J.R. and Connor,E.F.
  TITLE     Genomic assessment of the contribution of the Wolbachia
            endosymbiont of Eurosta solidaginis to gall-induction
  JOURNAL   International Journal Molecular Sciences
REFERENCE   2  (bases 1 to 529)
  AUTHORS   Fiutek,N., Couger,B.M., Roy,S., de la Torre,J.R. and Connor,E.F.
  TITLE     Direct Submission
  JOURNAL   Submitted (10-FEB-2023) Biology, San Francisco State University,
            1600 Holloway Ave, San Francisco, CA 94132, USA
COMMENT     ##Genome-Assembly-Data-START##
            Assembly Date          :: JUN-2019
            Assembly Method        :: FLYE v. 06-2019
            Genome Representation  :: Full
            Expected Final Version :: Yes
            Genome Coverage        :: 100.0x
            Sequencing Technology  :: Illumina HiSeq; PacBio Sequel
            ##Genome-Assembly-Data-END##
FEATURES             Location/Qualifiers
     source          1..529
                     /organism="Wolbachia pipientis"
                     /mol_type="genomic DNA"
                     /submitter_seqid="contig_23028"
                     /isolate="wEsol"
                     /isolation_source="galls on Solidago altissima"
                     /host="Eurosta solidaginis MN-2018-EFC"
                     /db_xref="taxon:955"
                     /environmental_sample
                     /country="USA: Cowling Arboretum, Carleton College,
                     Northfield, MN"
                     /lat_lon="44.48 N 93.13 W"
                     /altitude="280 m"
                     /collection_date="2018-11"
                     /metagenome_source="insect metagenome"
                     /note="metagenomic"
     gene            complement(<1..>529)
                     /locus_tag="wEsol_00194"
     CDS             complement(<1..>529)
                     /locus_tag="wEsol_00194"
```

```
                     /codon_start=1
                     /transl_table=11
                     /product="putative DNA primase (bacterial type)"
                     /translation="ISICMDGDSAGRHAATRVAELALPILEPGYTLKFVTLPSNKDPY
                     DICNELAYKKEDVLTALDHSTKLHSEYLWHHIIDSNLQNYEKLAPEKYSILEHKFMEY
                     VNTISNSSIRRYYRDYFYNKASELRSSFKKQIFNSKTGATKGEYLHNKSPELIEAEQN
                     QAVILRIAVEFPEILN"
                     /cog="DnaG COG0358 568 DNA primase (bacterial type)"
                     /besthit="qcoverage 100 , hcoverage 59.6610169491525 ,
                     similarity 98.9 , identity 97.7 , evalue 1.47e-116 ,
                     alnlength 176 , Bacteria , Proteobacteria ,
                     WP_006279250.1 toprim domain-containing protein, partial
                     [Wolbachia endosymbiont of Muscidifurax uniraptor]"
ORIGIN
        1 gttcaaaatt tcaggaaatt ctacggctat acgcaaaatt acggcctgat tttgctctgc
       61 ttcaattagc tctggagatt tgttatgaag atactcacct tttgttgctc cagttttact
      121 attaaaaatc tgtttttga agctacttct tagttcacta gccttattgt aaaaataatc
      181 tctgtaatac cttctaatac tgctgttgct aatagtattt acatattcca tgaacttgtg
      241 ctcaagaatt gaatattttt ccggagcaag tttttcataa ttttgcaaat tgctatcgat
      301 tatgtgatgc cataaatatt cagaatgcag ttttgttgaa tggtcaagag cagtcaacac
      361 gtcttctttc ttgtatgcca gttcgttaca tatatcgtat ggatctttat tacttggcaa
      421 agtcacaaac tttaacgtgt atccaggctc taatattggc agagcaagtt ctgcaactcg
      481 agtagcagca tgacgtccag cactatcgcc atccatacag atggagatt
//
LOCUS       JAQZAU010000028          698 bp    dna     linear   ENV 17-MAR-2023
DEFINITION  MAG: Wolbachia pipientis isolate wEsol contig_23078, whole genome
            shotgun sequence.
ACCESSION   JAQZAU010000028  JAQZAU010000000
VERSION     JAQZAU010000028.1
DBLINK      BioProject: PRJNA929258
            BioSample: SAMN33142973
KEYWORDS    WGS; ENV; Metagenome Assembled Genome; MAG.
SOURCE      Wolbachia pipientis (insect metagenome)
  ORGANISM  Wolbachia pipientis
            Bacteria; Pseudomonadota; Alphaproteobacteria; Rickettsiales;
            Anaplasmataceae; Wolbachieae; Wolbachia.
REFERENCE   1  (bases 1 to 698)
  AUTHORS   Fiutek,N., Couger,B.M., Roy,S., de la Torre,J.R. and Connor,E.F.
  TITLE     Genomic assessment of the contribution of the Wolbachia
            endosymbiont of Eurosta solidaginis to gall-induction
  JOURNAL   International Journal Molecular Sciences
REFERENCE   2  (bases 1 to 698)
  AUTHORS   Fiutek,N., Couger,B.M., Roy,S., de la Torre,J.R. and Connor,E.F.
  TITLE     Direct Submission
  JOURNAL   Submitted (10-FEB-2023) Biology, San Francisco State University,
            1600 Holloway Ave, San Francisco, CA 94132, USA
COMMENT     ##Genome-Assembly-Data-START##
            Assembly Date          :: JUN-2019
            Assembly Method        :: FLYE v. 06-2019
            Genome Representation  :: Full
            Expected Final Version :: Yes
            Genome Coverage        :: 100.0x
            Sequencing Technology  :: Illumina HiSeq; PacBio Sequel
            ##Genome-Assembly-Data-END##
FEATURES             Location/Qualifiers
     source          1..698
                     /organism="Wolbachia pipientis"
                     /mol_type="genomic DNA"
                     /submitter_seqid="contig_23078"
                     /isolate="wEsol"
```

```
                     /isolation_source="galls on Solidago altissima"
                     /host="Eurosta solidaginis MN-2018-EFC"
                     /db_xref="taxon:955"
                     /environmental_sample
                     /country="USA: Cowling Arboretum, Carleton College,
                     Northfield, MN"
                     /lat_lon="44.48 N 93.13 W"
                     /altitude="280 m"
                     /collection_date="2018-11"
                     /metagenome_source="insect metagenome"
                     /note="metagenomic"
     gene            <1..128
                     /locus_tag="wEsol_00195"
     CDS             <1..128
                     /locus_tag="wEsol_00195"
                     /codon_start=1
                     /transl_table=11
                     /product="hypothetical protein"
                     /translation="PNDLCTDNAIMVGWTGIERLQKNYIDPLNFAPRPKWELESY"
                     /cog="QRI7 COG0533 342 Metal-dependent proteases with
                     possible chaperone activity"
                     /besthit="qcoverage 100 , hcoverage 51.25 , similarity
                     100 , identity 100 , evalue 2.17e-24 , alnlength 41 ,
                     Bacteria , Proteobacteria , EAL57873.1 peptidase, M22
                     family protein, partial [Wolbachia endosymbiont of
                     Drosophila ananassae]"
     gene            complement(186..395)
                     /locus_tag="wEsol_00196"
     CDS             complement(186..395)
                     /locus_tag="wEsol_00196"
                     /codon_start=1
                     /transl_table=11
                     /fullproduct="qcoverage 100 , hcoverage 87.3417721518987
                     , similarity 100 , identity 97.1 , evalue 3.43e-42 ,
                     alnlength 69 , Bacteria , Proteobacteria , WP_210437498.1
                     hypothetical protein, partial [Wolbachia endosymbiont of
                     Drosophila auraria]"
                     /translation="MPMEGTSKNLVRLRGKYVAVHFRLTSIMRLDKFIVISDRGENHM
                     LLLTYVKLPRIMEKICCFDLCENYC"
                     /product="hypothetical protein, partial"
                     /besthit="qcoverage 100 , hcoverage 87.3417721518987 ,
                     similarity 100 , identity 97.1 , evalue 3.43e-42 ,
                     alnlength 69 , Bacteria , Proteobacteria , WP_210437498.1
                     hypothetical protein, partial [Wolbachia endosymbiont of
                     Drosophila auraria]"
     gene            complement(481..>698)
                     /locus_tag="wEsol_00197"
     CDS             complement(481..>698)
                     /locus_tag="wEsol_00197"
                     /codon_start=1
                     /transl_table=11
                     /pfam="Transposase_20"
                     /fullproduct="qcoverage 100 , hcoverage 98.6111111111111
                     , similarity 100 , identity 100 , evalue 2.23e-44 ,
                     alnlength 71 , Bacteria , Proteobacteria , WP_179943635.1
                     transposase [Wolbachia endosymbiont of Drosophila
                     simulans]"
                     /translation="TPRQYSSGEVDRHGSISKMGPPECRSMLYEAAQTMLTVTKRTFK
                     LKNWGCEEKRNEKSNCSSSKKISCNYA"
                     /product="transposase"
```

```
                     /besthit="qcoverage 100 , hcoverage 98.6111111111111 ,
                     similarity 100 , identity 100 , evalue 2.23e-44 ,
                     alnlength 71 , Bacteria , Proteobacteria , WP_179943635.1
                     transposase [Wolbachia endosymbiont of Drosophila
                     simulans]"
ORIGIN
        1 cccctaacga cctatgtaca gacaatgcaa ttatggttgg atggacagga attgaaaggt
       61 tacagaaaaa ttatatagat ccacttaatt ttgcaccaag accaaagtgg gaattggaaa
      121 gttattaaaa gctctcaatt ctgaattata gctaaagtga cttaggttct ctaattgcac
      181 ataagtcaac aataattttc acacaaatca aaacagcata tcttctccat aatccttggt
      241 aattttacat aagttaaaag tagcatatgg ttctctccgc ggtctgagat gacaataaat
      301 ttgtcaagcc gcattatact tgttaggcgg aagtggacgg caacatattt accacgaagt
      361 ctaactaagt tcttgcttgt cccttccatc ggcattgcta tctttatttt caataaaatt
      421 attgtcctat gctcttttga tgtttattga taataaaatt ctgttttatc aaccaacatc
      481 ctatgcataa ttacagctaa ttttcttgct actgctacaa ttgctttttt cattcctttt
      541 ttcttcgcaa ccccagtttt ttaatttaaa tgtcctcttg gtaactgtaa gcattgtttg
      601 tgcagcttca tataacatac ttctacattc cggtggtccc atctttgata tgctgccatg
      661 ccgatcaact tctccagaag aatattgcct tggtgtca
//
LOCUS       JAQZAU010000029              646 bp    dna     linear   ENV 17-MAR-2023
DEFINITION  MAG: Wolbachia pipientis isolate wEsol contig_23080, whole genome
            shotgun sequence.
ACCESSION   JAQZAU010000029  JAQZAU010000000
VERSION     JAQZAU010000029.1
DBLINK      BioProject: PRJNA929258
            BioSample: SAMN33142973
KEYWORDS    WGS; ENV; Metagenome Assembled Genome; MAG.
SOURCE      Wolbachia pipientis (insect metagenome)
  ORGANISM  Wolbachia pipientis
            Bacteria; Pseudomonadota; Alphaproteobacteria; Rickettsiales;
            Anaplasmataceae; Wolbachieae; Wolbachia.
REFERENCE   1  (bases 1 to 646)
  AUTHORS   Fiutek,N., Couger,B.M., Roy,S., de la Torre,J.R. and Connor,E.F.
  TITLE     Genomic assessment of the contribution of the Wolbachia
            endosymbiont of Eurosta solidaginis to gall-induction
  JOURNAL   International Journal Molecular Sciences
REFERENCE   2  (bases 1 to 646)
  AUTHORS   Fiutek,N., Couger,B.M., Roy,S., de la Torre,J.R. and Connor,E.F.
  TITLE     Direct Submission
  JOURNAL   Submitted (10-FEB-2023) Biology, San Francisco State University,
            1600 Holloway Ave, San Francisco, CA 94132, USA
COMMENT     ##Genome-Assembly-Data-START##
            Assembly Date          :: JUN-2019
            Assembly Method        :: FLYE v. 06-2019
            Genome Representation  :: Full
            Expected Final Version :: Yes
            Genome Coverage        :: 100.0x
            Sequencing Technology  :: Illumina HiSeq; PacBio Sequel
            ##Genome-Assembly-Data-END##
FEATURES             Location/Qualifiers
     source          1..646
                     /organism="Wolbachia pipientis"
                     /mol_type="genomic DNA"
                     /submitter_seqid="contig_23080"
                     /isolate="wEsol"
                     /isolation_source="galls on Solidago altissima"
                     /host="Eurosta solidaginis MN-2018-EFC"
                     /db_xref="taxon:955"
                     /environmental_sample
                     /country="USA: Cowling Arboretum, Carleton College,
```

```
                     Northfield, MN"
                     /lat_lon="44.48 N 93.13 W"
                     /altitude="280 m"
                     /collection_date="2018-11"
                     /metagenome_source="insect metagenome"
                     /note="metagenomic"
     gene            <1..222
                     /locus_tag="wEsol_00198"
     CDS             <1..222
                     /locus_tag="wEsol_00198"
                     /codon_start=1
                     /transl_table=11
                     /product="hypothetical protein"
                     /translation="EKLIKDLHQNNNQKEQRYTRNQDMTVIEGAISSQLGLKIKINDN
                     NSKGKVMIRYDNPNELDLILKILNRKLEA"
                     /besthit="qcoverage 100 , hcoverage 25.7950530035336 ,
                     similarity 100 , identity 100 , evalue 1.97e-39 ,
                     alnlength 73 , Bacteria , Proteobacteria , WP_006279814.1
                     MULTISPECIES: ParB/RepB/Spo0J family partition protein
                     [Wolbachia]"
     gene            complement(335..511)
                     /locus_tag="wEsol_00199"
     CDS             complement(335..511)
                     /locus_tag="wEsol_00199"
                     /codon_start=1
                     /transl_table=11
                     /product="hypothetical protein"
                     /translation="MAKDCTNVAITKTISTRVSRTGMTPFFFLDPSSLGTGMTATLTI
                     VTPLLVSGYRGGMT"
     gene            complement(527..>646)
                     /locus_tag="wEsol_00200"
     CDS             complement(527..>646)
                     /locus_tag="wEsol_00200"
                     /codon_start=1
                     /transl_table=11
                     /product="hypothetical protein"
                     /translation="LVAIKSSLILLVKRLKHKKRQYFKADNNPRVSFAFFFIW"

ORIGIN
        1 gaaaaactga taaaggacct acatcaaaac aacaatcaaa aagagcagag atatactaga
       61 aatcaagata tgacagtaat agaaggcgct atatcttctc aacttggttt aaagatcaaa
      121 attaatgaca acaattctaa gggtaaagtt atgatacggt atgataatcc aaatgaattg
      181 gatttgatat tgaaaatttt gaataggaaa cttgaggcat agctagtagt tggctagcaa
      241 agcaacaaac caatatcaac tgttggtgtc atgaaagtag ctgacactgg aatccaggaa
      301 ttttattaag ttggtaagca taaaagtata acttctacgt cataccgccg cggtatccgc
      361 taacaagcag cggggtgacg attgtcaggg tagctgtcat cccagtgccc agactacttg
      421 gatccaggaa aaagaatggt gtcatcccag tgcgtgatac cctagtagaa atcgtttttg
      481 taattgcaac attcgtgcaa tctttagcca taaatattta aaaaatttac caaatgaaaa
      541 aaaaggcaaa agaaaccctg ggttattat ccgctttaaa atattggcgt tttttatgtt
      601 ttaaacgctt gacaagcaag attaagctgc ttttaattgc aactaa
//
LOCUS       JAQZAU010000030         7725 bp    dna     linear   ENV 17-MAR-2023
DEFINITION  MAG: Wolbachia pipientis isolate wEsol contig_25003, whole genome
            shotgun sequence.
ACCESSION   JAQZAU010000030 JAQZAU010000000
VERSION     JAQZAU010000030.1
DBLINK      BioProject: PRJNA929258
            BioSample: SAMN33142973
KEYWORDS    WGS; ENV; Metagenome Assembled Genome; MAG.
SOURCE      Wolbachia pipientis (insect metagenome)
```

```
  ORGANISM  Wolbachia pipientis
            Bacteria; Pseudomonadota; Alphaproteobacteria; Rickettsiales;
            Anaplasmataceae; Wolbachieae; Wolbachia.
REFERENCE   1  (bases 1 to 7725)
  AUTHORS   Fiutek,N., Couger,B.M., Roy,S., de la Torre,J.R. and Connor,E.F.
  TITLE     Genomic assessment of the contribution of the Wolbachia
            endosymbiont of Eurosta solidaginis to gall-induction
  JOURNAL   International Journal Molecular Sciences
REFERENCE   2  (bases 1 to 7725)
  AUTHORS   Fiutek,N., Couger,B.M., Roy,S., de la Torre,J.R. and Connor,E.F.
  TITLE     Direct Submission
  JOURNAL   Submitted (10-FEB-2023) Biology, San Francisco State University,
            1600 Holloway Ave, San Francisco, CA 94132, USA
COMMENT     ##Genome-Assembly-Data-START##
            Assembly Date          :: JUN-2019
            Assembly Method        :: FLYE v. 06-2019
            Genome Representation  :: Full
            Expected Final Version :: Yes
            Genome Coverage        :: 100.0x
            Sequencing Technology  :: Illumina HiSeq; PacBio Sequel
            ##Genome-Assembly-Data-END##
FEATURES             Location/Qualifiers
     source          1..7725
                     /organism="Wolbachia pipientis"
                     /mol_type="genomic DNA"
                     /submitter_seqid="contig_25003"
                     /isolate="wEsol"
                     /isolation_source="galls on Solidago altissima"
                     /host="Eurosta solidaginis MN-2018-EFC"
                     /db_xref="taxon:955"
                     /environmental_sample
                     /country="USA: Cowling Arboretum, Carleton College,
                     Northfield, MN"
                     /lat_lon="44.48 N 93.13 W"
                     /altitude="280 m"
                     /collection_date="2018-11"
                     /metagenome_source="insect metagenome"
                     /note="metagenomic"
     gene            complement(<1..1705)
                     /locus_tag="wEsol_00201"
     CDS             complement(<1..1705)
                     /locus_tag="wEsol_00201"
                     /codon_start=1
                     /transl_table=11
                     /pfam="Toprim_4 Toprim_3 AAA_25"
                     /fullproduct="qcoverage 100 , hcoverage 83.2844574780059
                     , similarity 99.3 , identity 98.8 , evalue 0.0 ,
                     alnlength 568 , Bacteria , Proteobacteria ,
                     WP_226079819.1 AAA family ATPase [Wolbachia endosymbiont
                     of Tribolium confusum]"
                     /translation="MDIYCSQRNGHYKLLGKKEQLRTQLLSNIRSCLSYLLPRGTFRG
                     DKFYVGDLQGSKGKSMIVELTGSKAGLWHDFSTGEGGDIFDLWAAVTGKNQFTDTIED
                     IAKWIGYSEKNNTLGQPTASWNYYDESDQVIATVYRYNTDSGKRYLPFDVKRSSFTPP
                     ETRPLYNIPGIMKSDKVILVEGEKCADALIEQGMTATTAMLGANAPIEKTDWSPLKGK
                     HVIIWPDNDEPGKQYAEKVVKKLTFLGVLSLTLLEIPDNKPKGWDSADGIEEKMNIPE
                     FIENNSKKIIVKQLLNIQEWSVERFIGPVPEQKFLVEGLFPLGVTSILAAMGDTGKGM
                     LLLDLALKVASRKDQPCGFGPLVTEHGSVVIFSAEDDMSEIHRRLERLDSQCERLKYK
                     DRLFIVPLPNVSGSLTIIKSVSGKVIETSPEFESITKQLNKIKDLKLIVFDPLASFVH
                     ADLNTDPAVGDYLMSLLSDLAGSTGASIITAHHMRKPKSEKPISTVEQARDAIRGTSA
                     LVNGVRCSYAFWSVEDTAKLTIFKSIGESVRQNALFYGAIVKANGLADRTLRTYLRNE
```

```
                     ET"
                     /product="AAA family ATPase"
                     /besthit="qcoverage 100 , hcoverage 83.2844574780059 ,
                     similarity 99.3 , identity 98.8 , evalue 0.0 , alnlength
                     568 , Bacteria , Proteobacteria , WP_226079819.1 AAA
                     family ATPase [Wolbachia endosymbiont of Tribolium
                     confusum]"
     gene            complement(1723..2961)
                     /locus_tag="wEsol_00202"
     CDS             complement(1723..2961)
                     /locus_tag="wEsol_00202"
                     /codon_start=1
                     /transl_table=11
                     /pfam="AAA_25 DnaB_C AAA_31"
                     /fullproduct="qcoverage 100 , hcoverage 100 , similarity
                     99.3 , identity 98.5 , evalue 1.07e-298 , alnlength 412 ,
                     Bacteria , Proteobacteria , WP_226079818.1 AAA family
                     ATPase [Wolbachia endosymbiont of Tribolium confusum]"
                     /translation="MKYDEGKLWAAVITRAIQDAAGKNPKLKKEACQWIRSRSFETVC
                     ELANLDFKRLKNMLNKFGINKENFMDFSSKNALKIKSLLINNIKDCLSYLLPEGKSYQ
                     NKFYIGSRNGNKIIVEMEGENAGNWYNLIEEKKGNIIDLWTLVKGNIDSAETWLNGKD
                     ERSSILQEKQNEEKAFSVNHYLNDKSPMPKDIIGPRILTPGGLLVIGGTPKIGKSHFL
                     LSLLVHLAAGRSFLGMKPARPLKIFYLQNEMEYDYIRERIQQLNKLPHLATENLVVTN
                     KMNLTLNEEGIEKIKDMIAEKFDLVVIDSPYNSVNYGLQHAIEKLRSKINPMAGIIIT
                     NHTKKVSTSTLEKNPFQALVGANALRSFYTSGIVIFQPNKRTNILQVVYELRNGRSIP
                     TKFINKINGCWKTANVIART"
                     /product="AAA family ATPase"
                     /besthit="qcoverage 100 , hcoverage 100 , similarity 99.3
                     , identity 98.5 , evalue 1.07e-298 , alnlength 412 ,
                     Bacteria , Proteobacteria , WP_226079818.1 AAA family
                     ATPase [Wolbachia endosymbiont of Tribolium confusum]"
     gene            complement(2958..3458)
                     /locus_tag="wEsol_00203"
     CDS             complement(2958..3458)
                     /locus_tag="wEsol_00203"
                     /codon_start=1
                     /transl_table=11
                     /pfam="Pfam-B_8806"
                     /fullproduct="qcoverage 100 , hcoverage 100 , similarity
                     100 , identity 100 , evalue 4.97e-115 , alnlength 166 ,
                     Bacteria , Proteobacteria , WP_182364992.1 MULTISPECIES:
                     hypothetical protein [Wolbachia]"
                     /translation="MLQDFLTDFNNAKLQSSLIPKGTIVKVKMAIKPGGYENWFTKNY
                     TTGSIYLNTEFTVTEGPYAKRKIFQIIGIKNGKAEGEEDIWGESGRSMLRSILESSRN
                     IHANDTSEKAVTARKINSIADFNDLEFTAKVGVETNNTYGDRNRITTVITPELQKSFQ
                     DDWLPF"
                     /product="hypothetical protein"
                     /besthit="qcoverage 100 , hcoverage 100 , similarity 100
                     , identity 100 , evalue 4.97e-115 , alnlength 166 ,
                     Bacteria , Proteobacteria , WP_182364992.1 MULTISPECIES:
                     hypothetical protein [Wolbachia]"
     gene            complement(3496..4284)
                     /locus_tag="wEsol_00204"
     CDS             complement(3496..4284)
                     /locus_tag="wEsol_00204"
                     /codon_start=1
                     /transl_table=11
                     /pfam="AAA_25"
                     /fullproduct="qcoverage 100 , hcoverage 100 , similarity
                     99.2 , identity 98.5 , evalue 8.75e-186 , alnlength 262 ,
```

```
                     Bacteria , Proteobacteria , WP_226079816.1 helicase RepA
                     family protein [Wolbachia endosymbiont of Tribolium
                     confusum]"
                     /translation="MLQNFLTIGQKIPFFSVKEYLDDQSPIPEDIISPRILTKRGLLV
                     FGGPPKIGKSDFLISWLVYMAAGRSFLGMTPNRPLKIFYMQTEIEYEYMKERLQQLDN
                     ELLNVAANNLIITPKVHLSFNRDEISEIKEIVNERFKPDVLAIDPLRNIFSSEYGNEN
                     DNSAMLFFLQKTLEKLRSAINPDACIVLTHHTKKLSKKMLEEDPFQGLSGAGSLRGFY
                     STGMVMFAHDEESSARQIVFELRNGERVQNKLVDKINGHWQLME"
                     /product="helicase RepA family protein"
                     /besthit="qcoverage 100 , hcoverage 100 , similarity 99.2
                     , identity 98.5 , evalue 8.75e-186 , alnlength 262 ,
                     Bacteria , Proteobacteria , WP_226079816.1 helicase RepA
                     family protein [Wolbachia endosymbiont of Tribolium
                     confusum]"
     gene            complement(4296..5117)
                     /locus_tag="wEsol_00205"
     CDS             complement(4296..5117)
                     /locus_tag="wEsol_00205"
                     /codon_start=1
                     /transl_table=11
                     /pfam="AAA_24"
                     /fullproduct="qcoverage 100 , hcoverage 100 , similarity
                     100 , identity 100 , evalue 7.01e-202 , alnlength 273 ,
                     Bacteria , Proteobacteria , WP_213863537.1 AAA family
                     ATPase [Wolbachia endosymbiont of Ceratitis capitata]"
                     /translation="MKIMNSNERLKVQTGIKMVIFGPYGIGKTSLLKTINEPTLCLDF
                     EAGLLAVQDWQGDSVSIRTWNEARDIACLIGGPNPALKSDSAYSQRHYEHVSSKYKEL
                     ASEFLKYRCVFIDSITVASRLCLLWSKMQPEAFSERSGKQDMRAAYGLLAQEMMAWLN
                     QFQHIPNKDIITVGTLGQYLDDFNRSTWLPQCEGAKTASEIPGIVDEVISMVGIKKDD
                     GTEVRSFVCQTINSWGYPAKDRSGCLNMVEEPHLGKLLTKIKTKAFAIQPITHNI"
                     /product="AAA family ATPase"
                     /besthit="qcoverage 100 , hcoverage 100 , similarity 100
                     , identity 100 , evalue 7.01e-202 , alnlength 273 ,
                     Bacteria , Proteobacteria , WP_213863537.1 AAA family
                     ATPase [Wolbachia endosymbiont of Ceratitis capitata]"
     gene            complement(5211..5705)
                     /locus_tag="wEsol_00206"
     CDS             complement(5211..5705)
                     /locus_tag="wEsol_00206"
                     /codon_start=1
                     /transl_table=11
                     /fullproduct="qcoverage 100 , hcoverage 100 , similarity
                     100 , identity 100 , evalue 1.32e-109 , alnlength 164 ,
                     Bacteria , Proteobacteria , WP_019236463.1 MULTISPECIES:
                     sigma-70 family RNA polymerase sigma factor [Wolbachia]"
                     /cog="RpoE COG1595 182 DNA-directed RNA polymerase
                     specialized sigma subunit, sigma24 homolog"
                     /pfam="Sigma70_r2 TrmB"
                     /tigr="TIGR02937 sigma70-ECF 165 RNA polymerase sigma
                     factor, sigma-70 family"
                     /product="sigma-70 family RNA polymerase sigma factor"
                     /translation="MKNSYQGIDSTIVKHVRFQASRLKSKSCFINESLEDIEQELFCQ
                     VWQYLNQHDEKRSSFSTFVARLANYCARNMLRNQLCLKRNITFDDINDDIPDHRNSEI
                     EAIIRTDINNIISTLSEKDSNLCQLLKVFTITEVSVITKIPKTTIYRTLERIRDKFSV
                     LEKL"
                     /besthit="qcoverage 100 , hcoverage 100 , similarity 100
                     , identity 100 , evalue 1.32e-109 , alnlength 164 ,
                     Bacteria , Proteobacteria , WP_019236463.1 MULTISPECIES:
                     sigma-70 family RNA polymerase sigma factor [Wolbachia]"
     gene            6397..6684
```

```
                     /locus_tag="wEsol_00207"
     CDS             6397..6684
                     /locus_tag="wEsol_00207"
                     /codon_start=1
                     /transl_table=11
                     /product="hypothetical protein"
                     /translation="MCFLSFHNWLNSLKHEFTAVYFEEVRRHLGTDAAHCYGGFLAVL
                     SAWCEEHHVPYKGVPVKTIKRFIAGKGNASKSEVIEAVKKKGFFTSRRQRI"
                     /besthit="qcoverage 91.5789473684211 , hcoverage
                     76.9911504424779 , similarity 98.9 , identity 95.4 ,
                     evalue 7.59e-55 , alnlength 87 , Bacteria ,
                     Proteobacteria , ONI57848.1 holliday junction resolvasome
                     [Wolbachia pipientis wVitA]"
     gene            6855..7622
                     /locus_tag="wEsol_00208"
     CDS             6855..7622
                     /locus_tag="wEsol_00208"
                     /codon_start=1
                     /transl_table=11
                     /product="hypothetical protein"
                     /pfam="ParBc Pfam-B_13714 Pfam-B_3908"
                     /translation="MNLAIHYYPTQNLVEYERNPRKNDDVVNRMCASIREFGFRIPIV
                     AKSDGTVVDGHLRLKAARKLGMESIPVVLSDNLNEPQTKAFRLLANQSANWAKWDDDL
                     LKVEIQELEDLQFDLKMTGFELEKVQHFLDDLDGEKEDFSDLVGDEKRVEITKPGDLW
                     ILGDHRIYCGDSSVVESYKVLLDDKMADITVCDPPYNVDYGSSQEREDKKILNDNQGE
                     KYELFLYDICSQYFSIYERCNLHLHIIIRIFNVAKSI"
                     /besthit="qcoverage 89.8039215686275 , hcoverage
                     68.3582089552239 , similarity 99.1 , identity 98.3 ,
                     evalue 9.06e-160 , alnlength 229 , Eukaryota , Arthropoda
                     , KAG8301083.1 hypothetical protein J6590_060860
                     [Homalodisca vitripennis]"
ORIGIN
        1 ctgtttcttc atttcttaaa taggtgcgta aagtgcgatc agccaaaccg tttgctttaa
       61 caattgctcc ataaaataat gcgttctgtc ttacagattc tcctattgat ttaaaaattg
      121 tcagtttagc tgtatcttcc acagaccaaa aagcgtatga acacctaaca ccattaacaa
      181 gagcagatgt acctctaatg gcatcacgtg cttgctcaac agtcgatata ggtttttcac
      241 tttttggctt tctcatatgg tgggcagtaa ttattgatgc tccagtgcta cctgctaaat
      301 cagataataa agacatcaaa taatcgccta cggctgggtc agtatttaaa tcagcatgca
      361 caaatgatgc aagtggatca aatacgatta attttaagtc ttttatttta ttaagttgtt
      421 ttgttatcga ttcaaattca ggagaagtct ctataacttt acctgaaaca ctctttatga
      481 ttgtaagtga tccactaaca tttggtaatg gtacaataaa gagcctatcc ttatatttta
      541 atctttcgca ttgcgaatca agacgctcta aacgacgatg tatttcactc atatcatctt
      601 ctgctgagaa aattaccaca gatccatgtt cagtaactaa aggaccaaaa ccacatggct
      661 gatcttttct actcgcaact ttcagtgcta aatcaagcag aagcatacct ttaccagtat
      721 ctcccatcgc agctagaata gatgttactc ctaaaggaaa taacccttca acaagaaact
      781 tttgctctgg tactggacct ataaaacgtt ctacgctcca ttcttgaata ttaagcaatt
      841 gcttaacgat gatttttttc gagttatttt caatgaattc aggaatattc atcttttctt
      901 ctataccatc agctgaatcc catccttttg gtttattgtc aggtatttca agaagagtaa
      961 gtgataaaac accaagaaaa gtaagctttt tcacaacttt ctcagcatat tgtttacctg
     1021 gttcatcatt gtctggccaa ataataacat gcttaccctt aagtggtgac cagtctgttt
     1081 tttctattgg tgcatttgct cctaacattg cagttgtggc agtcatacct tgctctatca
     1141 gagcatctgc acacttttca ccttcaacta aaatcacctt atcagatttc atgatacctg
     1201 gaatattata taaaggcctg gtctctggtg gagtaaaact agaccttttt acatcaaaag
     1261 gaagatatct ttttcctgaa tcagtgttat aacgataaac tgtagcaatc acttgatcac
     1321 tttcatcgta atagttccat gatgccgttg gttgccccag agtgttattt ttttccgaat
     1381 aaccaatcca cttagcaata tcttctatcg tgtcagtaaa ttgatttttt ccagtaacag
     1441 cagcccaaag atcaaaaata tctcctcctt ctccggtaga aaaatcgtgc cataatccgg
     1501 ctttactgcc agttaactca actatcatac ttttcccttt actgccttgc agatcaccta
     1561 catagaactt atcaccacga aaagttcctc ttggcagtaa ataagaaaga catgacctta
     1621 tatttgatag gagttgagtt cttaactgtt ctttcttccc taataattta taatgtccat
```

1681 ttctttgtga acaataaata tccataccta cctgaagaat ttttatgttc tagctataac
1741 gttagcagtt ttccaacatc cattaatctt attgatgaat tttgttggta tagatctacc
1801 gtttctaagt tcatatacta cttgtaagat atttgtacgt ttattgggtt gaaatattac
1861 tattccagat gtataaaaac ttcttaaagc gttagcacca acgagagctt gaaatggatt
1921 cttttccaat gtagaggtag atactttttt ggtgtgattt gtgatgatta ttccagccat
1981 agggttaatc ttagagcgta gtttttctat tgcatgctgc agaccatagt taacggaatt
2041 ataaggagaa tcaatcacaa ctaagtcaaa cttttcagct atcatatctt ttattttttc
2101 tataccttct tcatttaagg tcaaattcat cttgttcgta acaactaagt tttctgttgc
2161 taaatgagga agtttgttga gttgttgtat acgctctcta atgtaatcat attccatttc
2221 attttgtaga tagaatattt ttagcggtct tgcaggcttc atgccaagaa atgatcttcc
2281 tgctgccaga tgtactaata gagaaaggag aaaatgactt ttgccaatct ttggagtacc
2341 acctatgacc aaaagaccac ctggtgtcaa aattcttggg cctattatat cttttggcat
2401 tggtgattta tcgtttaagt aatggtttac agaaaatgct ttttcttcgt tttgtttttc
2461 ttgtaatatg gaacttcttt catctttacc gttcaaccat gtttcagcag aatctatatt
2521 acctttaact aaagtccaaa ggtctattat atttcctttc ttttcttcga tcaggttgta
2581 ccaatttcct gcattttctc cttccatttc aactattatc ttatttccat ttctactgcc
2641 aatatagaat ttattttggt aagatttgcc ttcaggtaac aaataagata aacaatcttt
2701 gatattatta attaataatg attttatttt taaagcattt ttagatgaaa aatccataaa
2761 attctccttg tttattccaa atttgttcaa catatttttt agacgtttaa agtcaagatt
2821 agccagctca caaacagttt caaaagatcg tgagcgaatc cattgacaag cctctttttt
2881 aagtttaggg tttttttcctg ctgcatcctg aatagctcta gtaataacag cagcccacaa
2941 ctttccttcg tcatacttca aaatggcaac caatcatctt gaaaactttt ttgtaattct
3001 ggagtaataa ctgtagtaat cctatttcta tctccgtacg tattattagt ttcaacgcct
3061 actttcgctg taaactctaa atcgttgaaa tcagctatag agttgatttt tctagcagta
3121 actgctttct ctgaagtgtc atttgcatga atatttctgg atgattccaa aatactacgc
3181 agcatcgaac gtccagattc tccccagatg tcttcttcac cttctgcttt gccatttttg
3241 atacctataa tctgaaaaat cttgcgtttt gcatatggac cttcagtgac agtaaattca
3301 gtgtttaaat agatgctgcc agtagtgtag tttttagtga accaattttc ataacctcca
3361 ggctttattg ccatttttac cttgactatt gtacctttcg gtattaaact gctctgtagt
3421 tttgcattat taaaatcagt taaaaaatct tgtagcatac gctctcctat aagttgattt
3481 taaagtgaaa attagctatt ccattagctg ccaatgacca tttatcttat cgacaagttt
3541 attttgcacg cgttcaccat tgcgaagctc aaatactatc tggcgtgcac tactttcttc
3601 atcatgagca aacatcacca taccagtact gtaaaatcca cgtaaagatc cagcaccact
3661 taggccttga aatggatctt cttcgagcat ctttttttgat aatttttttg tatgatgagt
3721 aagtacaata caagcatctg gattgatagc actgcgtagc ttttcaagag tcttttgcag
3781 aaaaaacagc atagcactat tgtcattttc atttccatat tcactgctaa agatgttacg
3841 taatggatct atggccaaaa catcaggttt aaagcgttca tttacaatct ccttaatttc
3901 acttatttca tcacgattaa atgataagtg cacttttggc gtaatgatta agttgttagc
3961 agctacattc aaaagttcat tatcaagttg ttgcaaacgt tctttcatat attcatattc
4021 aatttcagtt tgcatgtaga agattttcaa aggtctattt ggtgtcatac caagaaatga
4081 tctaccagca gccatgtaaa caagccaaga gatcaaaaag tcgcttttgc caatcttagg
4141 agggccacca aatactaaca aacctctttt agtcagaatt cttggtgaga ttatatcctc
4201 tggtattggc gattgatcat ccaagtattc tttcacactg aaaaaaggaa ttttttgacc
4261 aatagttaaa aagttttgta gcatataata acctcctata tgttgtgcgt gataggttga
4321 attgcaaaag ctttggtttt aatctttgta agaagttttc ctagatgcgg ttcttcaacc
4381 atattcaggc atccacttcg atctttagca ggatatcccc aagaattaat tgtctgacag
4441 acaaatgaac gaacctctgt accatcatct ttcttgattc caaccatact gattacttcg
4501 tcaactatcc caggaatttc acttgcagtt ttagctcctt cacattgagg tagccaagtt
4561 gagcgattga aatcatcaag gtattgccca agagttccaa ctgtaatgat gtctttattt
4621 ggaatatgtt gaaactgatt gagccaagcc atcatctctt gagcaagtaa cccgtaagca
4681 gctctcatat cttgttttcc tgatctttca gaaaaagctt caggttgcat ttttgaccat
4741 aaaagacata aacgcgaggc tactgtaata ctatcgataa aaacacatcg atacttgaga
4801 aactccgaag caagctcttt gtatttactg gatacatgct catagtgtct ttggctatat
4861 gctgagtcag attttaacgc aggattagga ccacctatga gacatgcaat atctcttgcc
4921 tcattccaag tacgaatact tacagaatcc ccttgccaat cttgaacagc aagtaaacct
4981 gcctcaaaat caagacaaag tgttggttca tttatggtct ttaagagact agttttacct
5041 ataccataag gaccaaagat gaccattttt atacctgttt gaacttttag ccgttcattg
5101 ctgttcatta ttttcatttt tctcccagta cctaaatata tatgtacgat gacctaggca
5161 attcgtccgc aatttgcaaa aaaaaatttt gagcaaatat aatcgagagc ttataacttc
5221 tctaaaactg agaatttatc gcgtattcgt tctagtgtgc gatagatagt tgtctttggt

```
     5281 attttggtta ttacagaaac ttcagtaata gtaaatacct taagtaattg acataaatta
     5341 ctatcttttt cagacaaagt tgaaattata ttatttatat cagtgcgaat tatcgcttcg
     5401 atttccgagt tcctatgatc tggaatatcg tcgtttatat catcaaaagt tatattacgt
     5461 tttagacata actgattgcg taacatattg cgagcacaat aattagctaa tctagcaacg
     5521 aaagtactaa aactacttct tttctcatcg tgctggttaa gatattgcca aacctgacaa
     5581 aaaagctctt gttctatatc ttcgagactt tcgttaataa aacagctttt ggatttaagg
     5641 cgcgaagctt gaaatctcac atgtttaaca attgtagaat cgattccttg ataagaattc
     5701 ttcataaaaa ctccaaaagt ttcatgaata gagattttta ccaagacttt gtatgactaa
     5761 tttattataa aaaagtagta ataatttttt tatctaaaag aataaaatct ttaaaaatta
     5821 aaataagatt taaacgtttt gatacactac tgaagctact ttttcatagg taaaaaacta
     5881 ccaatttcgt acctaatttc tgagttacta attagtattt cagaaagcag tgatcgttaa
     5941 gttattgata atattgtctt ttttatgttc tattcctaaa agccgtttct aaagttaagt
     6001 ggctaaaatc ggccagtaaa gagggtttaa ggattactga agtcttatat atattatata
     6061 tatatttaaa tacaggtaaa atataatata taataatata aatattggcg taatataagt
     6121 tatgtaaaat agttattaag ataagtaaag atgaaaaaaa ctcaaatggc aggatatgaa
     6181 attttttta atttttttc aaaattgcgg acgaatttgc gagtcatcgt acatatatat
     6241 tatacagggc attgaacttt aattacacaa aggagttttt gtacgtcaat cttaacgcta
     6301 gatctcggca agcaaacagg ctgggcaatt cttacagatg gagtaattga aagtggaagc
     6361 aaaagttttc atggtagccg tttcagtgga ggtggaatgt gtttttaag ttttcacaat
     6421 tggcttaatt ctttgaaaca tgagttcact gcagtgtatt ttgaagaagt aagaagacat
     6481 ctaggaactg atgcagcgca ttgttatggt ggtttttgg ctgttctatc tgcttggtgt
     6541 gaagaacatc atgtgccgta caaaggtgtt cctgtaaaga ctataaaacg ctttatagca
     6601 ggcaaaggca acgcaagtaa gagtgaagtt attgaagctg taaagaaaaa aggtttttc
     6661 acctcgagac gacaacgaat ctgatgcttt agcttaatgt tctatattag taaagatatt
     6721 aatgtattaa aaataccata aaaagtgggt cctttcggct ataatggcgg gtttggtggt
     6781 gctgacccca ggccttttct agtgttagac atattttgaa tattaacttt ttaattatta
     6841 agaatttaaa ttatatgaat ttagcaatcc actactatcc tactcaaaat ctggtcgaat
     6901 atgagcgtaa tcctcgtaaa aatgacgatg tggtcaacag aatgtgtgct tcaatcaggg
     6961 aattcggctt tcgtattcca atagttgcaa aaagcgatgg aactgtggtt gatggccatt
     7021 taagacttaa agcagcaaga aaacttggta tggagagtat tccagtggtt ttaagtgata
     7081 atttaaatga accacaaact aaagcttttc gattactcgc aaatcaatca gctaattggg
     7141 caaagtggga tgatgatctt ttaaaggtgg aaattcaaga gttagaagat ttacagtttg
     7201 atcttaaaat gacaggattt gaattggaaa aagttcaaca tttccttgat gacttagatg
     7261 gtgaaaaaga agatttttct gacttggttg gtgatgaaaa aagggtagaa ataacaaaac
     7321 caggtgatct ttggatttta ggtgatcatc gaatctattg tggtgatagc tctgtagttg
     7381 aatcatataa agtgctgtta gatgataaaa tggcagacat tactgtttgt gatcctccat
     7441 ataacgttga ctatggtagt agccaagaga gagaagataa aaaaatatta aacgataatc
     7501 aaggtgaaaa gtatgagctt tttctctatg acatctgttc ccaatatttt agcatatacg
     7561 aaaggtgcaa tttacatctg catatcatca tcagaatttt caacgttgca aaaagcattt
     7621 gaagaggcag gagggaaatg gtcaacattt atcatttggg caaagaatca ttttacacta
     7681 ggaagatctg attatcaaag acaatacgaa gcaatgctct atgga
//
LOCUS       JAQZAU010000031            11993 bp    dna     linear   ENV 17-MAR-2023
DEFINITION  MAG: Wolbachia pipientis isolate wEsol contig_2547, whole genome
            shotgun sequence.
ACCESSION   JAQZAU010000031 JAQZAU010000000
VERSION     JAQZAU010000031.1
DBLINK      BioProject: PRJNA929258
            BioSample: SAMN33142973
KEYWORDS    WGS; ENV; Metagenome Assembled Genome; MAG.
SOURCE      Wolbachia pipientis (insect metagenome)
  ORGANISM  Wolbachia pipientis
            Bacteria; Pseudomonadota; Alphaproteobacteria; Rickettsiales;
            Anaplasmataceae; Wolbachieae; Wolbachia.
REFERENCE   1  (bases 1 to 11993
  AUTHORS   Fiutek,N., Couger,B.M., Roy,S., de la Torre,J.R. and Connor,E.F.
  TITLE     Genomic assessment of the contribution of the Wolbachia
            endosymbiont of Eurosta solidaginis to gall-induction
  JOURNAL   International Journal Molecular Sciences
REFERENCE   2  (bases 1 to 11993)
```

```
  AUTHORS   Fiutek,N., Couger,B.M., Roy,S., de la Torre,J.R. and Connor,E.F.
  TITLE     Direct Submission
  JOURNAL   Submitted (10-FEB-2023) Biology, San Francisco State University,
            1600 Holloway Ave, San Francisco, CA 94132, USA
COMMENT     ##Genome-Assembly-Data-START##
            Assembly Date          :: JUN-2019
            Assembly Method        :: FLYE v. 06-2019
            Genome Representation  :: Full
            Expected Final Version :: Yes
            Genome Coverage        :: 100.0x
            Sequencing Technology  :: Illumina HiSeq; PacBio Sequel
            ##Genome-Assembly-Data-END##
FEATURES             Location/Qualifiers
     source          1..11993
                     /organism="Wolbachia pipientis"
                     /mol_type="genomic DNA"
                     /submitter_seqid="contig_2547"
                     /isolate="wEsol"
                     /isolation_source="galls on Solidago altissima"
                     /host="Eurosta solidaginis MN-2018-EFC"
                     /db_xref="taxon:955"
                     /environmental_sample
                     /country="USA: Cowling Arboretum, Carleton College,
                     Northfield, MN"
                     /lat_lon="44.48 N 93.13 W"
                     /altitude="280 m"
                     /collection_date="2018-11"
                     /metagenome_source="insect metagenome"
                     /note="metagenomic"
     gene            complement(<1..282)
                     /locus_tag="wEsol_00209"
     CDS             complement(<1..282)
                     /locus_tag="wEsol_00209"
                     /codon_start=1
                     /transl_table=11
                     /fullproduct="qcoverage 100 , hcoverage 84.6846846846847
                     , similarity 98.9 , identity 98.9 , evalue 5.85e-56 ,
                     alnlength 94 , Bacteria , Proteobacteria , WP_213863527.1
                     GPW/gp25 family protein [Wolbachia endosymbiont of
                     Ceratitis capitata]"
                     /cog="COG3628 COG3628 116 Phage baseplate assembly
                     protein W"
                     /pfam="DUF2634 GPW_gp25"
                     /product="GPW/gp25 family protein"
                     /translation="MKGMDSKTGKELEGIEHLKQSIIDILTTPINSRVMRRDYGSRLF
                     ELVDKPINRDLTLEIYSAVAEALQSWEERFKLEKVKMTEVKEGKVTLDLE"
                     /besthit="qcoverage 100 , hcoverage 84.6846846846847 ,
                     similarity 98.9 , identity 98.9 , evalue 5.85e-56 ,
                     alnlength 94 , Bacteria , Proteobacteria , WP_213863527.1
                     GPW/gp25 family protein [Wolbachia endosymbiont of
                     Ceratitis capitata]"
     gene            complement(285..539)
                     /locus_tag="wEsol_00210"
     CDS             complement(285..539)
                     /locus_tag="wEsol_00210"
                     /codon_start=1
                     /transl_table=11
                     /product="hypothetical protein"
                     /pfam="PAAR_motif DUF4150 Pfam-B_14026 PAAR_motif"
                     /translation="MNKSVVRVGDYCVEVTPHFCISGSNNVFVNGKPMCRQGESFSEG
```

```
                     EVMVQGSKTVFANGFGAGRVGDIVSCGFKVIKGSENVFAG"
                     /besthit="qcoverage 58.3333333333333 , hcoverage
                     58.3333333333333 , similarity 100 , identity 100 , evalue
                     3.94e-28 , alnlength 49 , Bacteria , Proteobacteria ,
                     WP_174516944.1 hypothetical protein [Wolbachia
                     endosymbiont of Cardiocondyla obscurior]"
     gene            complement(540..698)
                     /locus_tag="wEsol_00211"
     CDS             complement(540..698)
                     /locus_tag="wEsol_00211"
                     /codon_start=1
                     /transl_table=11
                     /product="hypothetical protein"
                     /pfam="Pfam-B_2532"
                     /translation="MKFQDGTRLLYDKEKHHLEIEVVDKITLKAGESSIEMTKSEIKL
                     KADRINLN"
                     /cog="gpV COG4540 184 Phage P2 baseplate assembly protein
                     gpV"
                     /besthit="qcoverage 100 , hcoverage 33.7662337662338 ,
                     similarity 100 , identity 98.1 , evalue 1.16e-24 ,
                     alnlength 52 , Bacteria , Proteobacteria , WP_065095149.1
                     MULTISPECIES: phage baseplate assembly protein V
                     [unclassified Wolbachia]"
     gene            complement(712..1023)
                     /locus_tag="wEsol_00212"
     CDS             complement(712..1023)
                     /locus_tag="wEsol_00212"
                     /codon_start=1
                     /transl_table=11
                     /fullproduct="qcoverage 100 , hcoverage 100 , similarity
                     100 , identity 100 , evalue 1.60e-74 , alnlength 103 ,
                     Bacteria , Proteobacteria , WP_155968470.1 transposase
                     [Wolbachia pipientis]"
                     /cog="COG3335 COG3335 132 Transposase and inactivated
                     derivatives"
                     /pfam="DDE_3 DDE_1"
                     /product="transposase"
                     /translation="MTFKGGCDKEVFNTWLEKMLLPKLPHGTTIVMDNATFHKTPKTK
                     ELIDNARCRLLYLPTYSPDLNPIEHCWNTIKSRLRPLMHKYTDLQLLVGNTIMEIYHS
                     F"
                     /besthit="qcoverage 100 , hcoverage 100 , similarity 100
                     , identity 100 , evalue 1.60e-74 , alnlength 103 ,
                     Bacteria , Proteobacteria , WP_155968470.1 transposase
                     [Wolbachia pipientis]"
     gene            complement(1223..1570)
                     /locus_tag="wEsol_00213"
     CDS             complement(1223..1570)
                     /locus_tag="wEsol_00213"
                     /codon_start=1
                     /transl_table=11
                     /fullproduct="qcoverage 100 , hcoverage 100 , similarity
                     100 , identity 100 , evalue 2.74e-74 , alnlength 115 ,
                     Bacteria;Eukaryota , Proteobacteria;Arthropoda ,
                     WP_155968342.1 MULTISPECIES: IS630 transposase-related
                     protein [Wolbachia]"
                     /cog="COG3415 COG3415 138 Transposase and inactivated
                     derivatives"
                     /pfam="HTH_Tnp_IS630 HTH_Tnp_1 HTH_23 HTH_28 HTH_29 HTH_7
                     CENP-B_N Pfam-B_14738 HTH_8 HTH_17 HTH_24 Pfam-B_2106"
                     /product="IS630 transposase-related protein"
```

```
                     /translation="MAYSVDLRERAVSMIEKGKSKVEVAELLEIGIATLYRWLRKKAA
                     GESLRPSKNGSFIRKIDPKILEEYVKKNPDHTLAEMKQNLGFGINSIWYRLKQLRITL
                     KKSYTISRAQSRR"
                     /besthit="qcoverage 100 , hcoverage 100 , similarity 100
                     , identity 100 , evalue 2.74e-74 , alnlength 115 ,
                     Bacteria;Eukaryota , Proteobacteria;Arthropoda ,
                     WP_155968342.1 MULTISPECIES: IS630 transposase-related
                     protein [Wolbachia]"
     gene            complement(1560..1925)
                     /locus_tag="wEsol_00214"
     CDS             complement(1560..1925)
                     /locus_tag="wEsol_00214"
                     /codon_start=1
                     /transl_table=11
                     /product="hypothetical protein"
                     /pfam="Phage_base_V"
                     /tigr="TIGR01644 phage_P2_V 192 phage baseplate assembly
                     protein V"
                     /translation="MLESNFAISELQRKLANIVRIGVVKEVDYEKAKVRVKIGELLTD
                     FLPWITSRAGEERSWLPPSINEQVVILSPLGELSLGVVLAGIYQQKYAAPENKKEINI
                     VLPKVNDKGIISYLSYNGV"
                     /cog="gpV COG4540 184 Phage P2 baseplate assembly protein
                     gpV"
                     /besthit="qcoverage 85.1239669421488 , hcoverage
                     66.8831168831169 , similarity 99.0 , identity 99.0 ,
                     evalue 2.06e-63 , alnlength 103 , Bacteria ,
                     Proteobacteria , WP_208571542.1 MULTISPECIES: phage
                     baseplate assembly protein V [Wolbachia]"
     gene            complement(1912..2385)
                     /locus_tag="wEsol_00215"
     CDS             complement(1912..2385)
                     /locus_tag="wEsol_00215"
                     /codon_start=1
                     /transl_table=11
                     /pfam="Pfam-B_4030"
                     /fullproduct="qcoverage 100 , hcoverage 100 , similarity
                     100 , identity 100 , evalue 1.06e-107 , alnlength 157 ,
                     Bacteria , Proteobacteria , WP_213863529.1 hypothetical
                     protein [Wolbachia endosymbiont of Ceratitis capitata]"
                     /translation="MNFKDLHNAIFTTLKKEIPAIQTCEIYPSIRKELLAPALFVELV
                     SLESGKDPGTEELALKARFEARIVIDSTIENAAIIVRTLAAEVAKVVNKNTWNIKNVS
                     PGEFISAEIDGFRPELDAYLVWMVDWSHQLHLGKSIWTENKIKPHTIIIEENVRE"
                     /product="hypothetical protein"
                     /besthit="qcoverage 100 , hcoverage 100 , similarity 100
                     , identity 100 , evalue 1.06e-107 , alnlength 157 ,
                     Bacteria , Proteobacteria , WP_213863529.1 hypothetical
                     protein [Wolbachia endosymbiont of Ceratitis capitata]"
     gene            complement(2382..2894)
                     /locus_tag="wEsol_00216"
     CDS             complement(2382..2894)
                     /locus_tag="wEsol_00216"
                     /codon_start=1
                     /transl_table=11
                     /pfam="Pfam-B_14565 Pfam-B_12992 Minor_tail_Z"
                     /fullproduct="qcoverage 100 , hcoverage 100 , similarity
                     100 , identity 100 , evalue 9.88e-108 , alnlength 170 ,
                     Bacteria , Proteobacteria , WP_213863530.1 phage tail
                     protein [Wolbachia endosymbiont of Ceratitis capitata]"
                     /translation="MFDIKISNNINEIVYNIDEKEQKVKLAAIKALNRTALWLKAQAA
                     KEISEEKKIKLSLIRKRLRIFKAKTSRLEVLIRANLYDIRASTIGKIQKTRRGSKVGK
```

```
                     HEFIGGFAAVMPKGNSGMFKREGRAALPIKEVKLSLEPEASRIIGNLVNYEVEEVFEK
                     FFERDITRNI"
                     /product="phage tail protein"
                     /besthit="qcoverage 100 , hcoverage 100 , similarity 100
                     , identity 100 , evalue 9.88e-108 , alnlength 170 ,
                     Bacteria , Proteobacteria , WP_213863530.1 phage tail
                     protein [Wolbachia endosymbiont of Ceratitis capitata]"
     gene            complement(2887..3198)
                     /locus_tag="wEsol_00217"
     CDS             complement(2887..3198)
                     /locus_tag="wEsol_00217"
                     /codon_start=1
                     /transl_table=11
                     /fullproduct="qcoverage 100 , hcoverage 100 , similarity
                     100 , identity 100 , evalue 5.85e-68 , alnlength 103 ,
                     Bacteria , Proteobacteria , WP_141457308.1 MULTISPECIES:
                     hypothetical protein [unclassified Wolbachia]"
                     /translation="MNIGKLLEDCFEHLGVEATYCNKNKEGIRRIKVLIKRPETTYSL
                     GADGALTQQIASIEIRDQDVTSFSIGDYIKIEKRFYKIFEPPLKDSSSEIWKIEAIEN
                     V"
                     /product="hypothetical protein"
                     /besthit="qcoverage 100 , hcoverage 100 , similarity 100
                     , identity 100 , evalue 5.85e-68 , alnlength 103 ,
                     Bacteria , Proteobacteria , WP_141457308.1 MULTISPECIES:
                     hypothetical protein [unclassified Wolbachia]"
     gene            complement(3198..4202)
                     /locus_tag="wEsol_00218"
     CDS             complement(3198..4202)
                     /locus_tag="wEsol_00218"
                     /codon_start=1
                     /transl_table=11
                     /pfam="Phage_cap_E"
                     /fullproduct="qcoverage 100 , hcoverage 100 , similarity
                     100 , identity 100 , evalue 5.18e-246 , alnlength 334 ,
                     Bacteria , Proteobacteria , WP_213863531.1 major capsid
                     protein [Wolbachia endosymbiont of Ceratitis capitata]"
                     /translation="MQNPFTNTAFSMTALTNAMNILPINYGRVENLNLFPSRSVRFRH
                     ITIEEHNGVLSLLPTQVPGAPATVGKRGKRKVRTFTIPHIPHDDVVLPEEVQGIRAFG
                     SESELKALADVITDHLQLMRNKHAITLEHLRMGALKGIILDADGSGLLNLYNEFEITP
                     KVVNFALGTATTDVKRKCLEVLRHIEDNLSGEYMTGIHALVSPEFFDALTSHAKVKEA
                     YERWQEGAALRNDMRSGFTFCGITFEEYRGQATDPEGTVRRFIEKDTGHCFPVGTAST
                     FTTYFAPADFNETVNTLGQPLYAKQEPRRFDRGTDLHTQSNPLPMCHRPGVLVKISSS
                     "
                     /product="major capsid protein"
                     /besthit="qcoverage 100 , hcoverage 100 , similarity 100
                     , identity 100 , evalue 5.18e-246 , alnlength 334 ,
                     Bacteria , Proteobacteria , WP_213863531.1 major capsid
                     protein [Wolbachia endosymbiont of Ceratitis capitata]"
     gene            complement(4228..4596)
                     /locus_tag="wEsol_00219"
     CDS             complement(4228..4596)
                     /locus_tag="wEsol_00219"
                     /codon_start=1
                     /transl_table=11
                     /pfam="HDPD"
                     /fullproduct="qcoverage 100 , hcoverage 100 , similarity
                     100 , identity 100 , evalue 1.95e-75 , alnlength 122 ,
                     Bacteria , Proteobacteria , WP_077188124.1 MULTISPECIES:
                     head decoration protein [Wolbachia]"
                     /translation="MSCIIEQNNLGDLLKYEASSLYSRDQITVAKGQNLKLGTVVGSD
```

```
                     KDNMIKIINPTATDGTQTAIGAIVSDVNATENAKAVIITRIAMLADHAIVWPTGITEE
                     QKAAAIKQLEGRGIIVRKGV"
                     /product="head decoration protein"
                     /besthit="qcoverage 100 , hcoverage 100 , similarity 100
                     , identity 100 , evalue 1.95e-75 , alnlength 122 ,
                     Bacteria , Proteobacteria , WP_077188124.1 MULTISPECIES:
                     head decoration protein [Wolbachia]"
     gene            complement(4674..5720)
                     /locus_tag="wEsol_00220"
     CDS             complement(4674..5720)
                     /locus_tag="wEsol_00220"
                     /codon_start=1
                     /transl_table=11
                     /pfam="CLP_protease Peptidase_S49"
                     /fullproduct="qcoverage 100 , hcoverage 100 , similarity
                     100 , identity 100 , evalue 3.57e-235 , alnlength 348 ,
                     Bacteria , Proteobacteria , WP_213863532.1 S49 family
                     peptidase [Wolbachia endosymbiont of Ceratitis capitata]"
                     /translation="MKQQWLNRCVMVEPRSFELLSLQTGKQPIFKNIKHAVRNSERGI
                     IPIHGILTKKPGAFDEMLGMTSYEQIEEQITQALADSSIETIILEIDSPGGEVNGIFD
                     LADFIYESRAKKRIIAIANDDAYSAAYAIASSAEKVFVSRTSGVGSIGVIASHIDQSG
                     FNEKCGIKYTTVFAGSRKNDLNPHEPMTSESLESLQKEVDRLYEMFVQLIARNRGLSI
                     EKIRSTEAGLYFGERAIEIGLADGITILSSINKNRSITMNEQTTTDLETDNLTKYRTE
                     VLELIRLCNLSRMPEKIEEFIEQGVSIEQAREVLMELLAERTKKTEILSAIPQNSGEE
                     LMMEVARTRAQSSI"
                     /product="S49 family peptidase"
                     /besthit="qcoverage 100 , hcoverage 100 , similarity 100
                     , identity 100 , evalue 3.57e-235 , alnlength 348 ,
                     Bacteria , Proteobacteria , WP_213863532.1 S49 family
                     peptidase [Wolbachia endosymbiont of Ceratitis capitata]"
     gene            complement(5717..7135)
                     /locus_tag="wEsol_00221"
     CDS             complement(5717..7135)
                     /locus_tag="wEsol_00221"
                     /codon_start=1
                     /transl_table=11
                     /fullproduct="qcoverage 100 , hcoverage 100 , similarity
                     100 , identity 100 , evalue 0.0 , alnlength 472 ,
                     Bacteria , Proteobacteria , WP_213863533.1 phage portal
                     protein [Wolbachia endosymbiont of Ceratitis capitata]"
                     /cog="COG5511 COG5511 492 Bacteriophage capsid protein"
                     /pfam="Phage_portal_2"
                     /tigr="TIGR01539 portal_lambda 457 phage portal protein,
                     lambda family"
                     /product="phage portal protein"
                     /translation="MMLKNFKQLFSKPKIKSSAWDAAGSGRRVMYWQGETGSINNLLS
                     QSLETLRSRSRDMVRKNPYAANIIDTIVSNSIGTGIKPQSKARDGEFRKKVQELWLRW
                     TDEADSCGISDFYGLQALVCRSMIEGGECFVRLRTRKLEDRFSVPLQLQVLESEHLDN
                     KTNQTLGNGNVIRNGIEFNKLGQREAYYLFREHPGEGSFGESVRVPANDVLHIYRPLR
                     PGQIRGEPWLSSILLKLYELDQYDDAELVRKKTAAMFAGFITRLDPEANILGEGEANE
                     QGVALSGLEPGTMQLLDPGEDIKFSEPSDVGGSYEAFIRQQLRAIAIGTGITYEQLTG
                     DLTGVNYSSIRAGLIEFRRRCAMLQHNVIVFQFCRPIWNRWLELAVLCGELSIDEKVV
                     KAAKEEVKWIPQGFDWVDPLKDQQAQQMAVRNGFKSRSEVVSEMGYDVEEIDQEIAED
                     QRRASELGLSFDSEVTANQEVI"
                     /besthit="qcoverage 100 , hcoverage 100 , similarity 100
                     , identity 100 , evalue 0.0 , alnlength 472 , Bacteria ,
                     Proteobacteria , WP_213863533.1 phage portal protein
                     [Wolbachia endosymbiont of Ceratitis capitata]"
     gene            complement(7132..7836)
                     /locus_tag="wEsol_00222"
```

```
CDS             complement(7132..7836)
                /locus_tag="wEsol_00222"
                /codon_start=1
                /transl_table=11
                /fullproduct="qcoverage 100 , hcoverage 100 , similarity
                100 , identity 100 , evalue 1.07e-172 , alnlength 234 ,
                Bacteria , Proteobacteria , WP_213863534.1 glycoside
                hydrolase family 25 protein [Wolbachia endosymbiont of
                Ceratitis capitata]"
                /cog="Acm COG3757 269 Lyzozyme M1
                (1,4-beta-N-acetylmuramidase)"
                /pfam="Glyco_hydro_25"
                /product="glycoside hydrolase family 25 protein"
                /translation="MRENFLRKVVKEEYSEFLEQLKTFIKLWENRESPVNGIIDLSHW
                QENVNFKLDKENGIVGVIHKATQGLKHVDSKYAKRRKKAEEEGLLWGAYHFGTEGNGK
                NQADHFLKTVGESKNTILALDIEENKSGKNIAVKQAEEFVKRVEEVTGRLPLIYGSSY
                FLKDFATPTLTKCPLWIASWRKELTLPKGWNDWILWQYTDGKTNPEPHEVEGIGKCDR
                NKFNGTLKELREFWLV"
                /besthit="qcoverage 100 , hcoverage 100 , similarity 100
                , identity 100 , evalue 1.07e-172 , alnlength 234 ,
                Bacteria , Proteobacteria , WP_213863534.1 glycoside
                hydrolase family 25 protein [Wolbachia endosymbiont of
                Ceratitis capitata]"
gene            complement(7833..8057)
                /locus_tag="wEsol_00223"
CDS             complement(7833..8057)
                /locus_tag="wEsol_00223"
                /codon_start=1
                /transl_table=11
                /pfam="gpW"
                /fullproduct="qcoverage 100 , hcoverage 100 , similarity
                100 , identity 98.6 , evalue 3.67e-42 , alnlength 74 ,
                Bacteria , Proteobacteria , WP_017531716.1 hypothetical
                protein [Wolbachia endosymbiont of Diaphorina citri]"
                /translation="MYNEDYLIQVEEAIKKLQNGERVVSIAYGDHVVRYAEVQINDLL
                SLRQRIKGELKIAGMKPKRKIIFATNKGVI"
                /product="hypothetical protein"
                /besthit="qcoverage 100 , hcoverage 100 , similarity 100
                , identity 98.6 , evalue 3.67e-42 , alnlength 74 ,
                Bacteria , Proteobacteria , WP_017531716.1 hypothetical
                protein [Wolbachia endosymbiont of Diaphorina citri]"
gene            complement(8061..9884)
                /locus_tag="wEsol_00224"
CDS             complement(8061..9884)
                /locus_tag="wEsol_00224"
                /codon_start=1
                /transl_table=11
                /fullproduct="qcoverage 100 , hcoverage 100 , similarity
                99.3 , identity 99.3 , evalue 0.0 , alnlength 607 ,
                Bacteria , Proteobacteria , WP_012673198.1 MULTISPECIES:
                phage terminase large subunit family protein [Wolbachia]"
                /cog="COG5525 COG5525 611 Phage terminase, large subunit
                GpA"
                /pfam="Terminase_GpA DUF3268"
                /product="phage terminase large subunit family protein"
                /translation="MIYATSFSEGLKPDPELKVSEWANEYRVLAPTAASEPGKWRTER
                TPYLKEIMDSLSPSSPAEKVVFMKGAQIGGTEAGNNWIGYIIYQTPGPMLVVQPTVEM
                GKRWSKGRFAPLIESTPCLKSKVKDPRSRDSGNTVQSKEFPGGIVVITGANSSVGLRS
                MPVKYLFLDEIDAYPGDSGGEGDPVLLSIARTNTSARRKIFLVSTPTIHGISRIEKEF
                EATDKQYFFVPCPYCNYYQVLKWSQIKWENNDSRTAHYVCTECSGKIENHQKTEMLER
```

GEWRPTNRVKGEKKGFHLSSLYSPVGWYSWTQAVEDFLHAKESEQLLKVWINTTLGET
WVDKGEVPDWKQLFNRREFFPVGTVPRREVVLTAGVDVQKDRLEVEVVAWGKSRESWS
IEYRVFEGDTGRGEVWGKLSELLNHHFIGENGLEYMISMMAVDAGYATQEVYNWVRGH
QGSGRVMAVKGVNKALVPLSSPSRVDITVGGQKLKRGIKLWPVGVSILKSELFQLLNV
LKDGEEAPAGYCHFPEYAPEYFKQLTAEQLVSKVVKGYTKQEWQKVRERNEVLDCRIY
ARAASIALGIDRWPESKWNSLSEKPESKKSKKIVKSKWISE"
/besthit="qcoverage 100 , hcoverage 100 , similarity 99.3
, identity 99.3 , evalue 0.0 , alnlength 607 , Bacteria ,
Proteobacteria , WP_012673198.1 MULTISPECIES: phage
terminase large subunit family protein [Wolbachia]"
gene complement(9881..11365)
/locus_tag="wEsol_00225"
CDS complement(9881..11365)
/locus_tag="wEsol_00225"
/codon_start=1
/transl_table=11
/pfam="Ank_2 Ank_4 Pfam-B_1881 Ank_5 Pfam-B_10896 Ank
Ank_3 Ank_5 Ank_3 Ank Ank_4 Ank_2 Pfam-B_10896 Ank Ank_3
Ank_5 Ank_2 Pfam-B_11730 Ank_3 Ank Ank_4 Ank_2
Pfam-B_10896 Ank_5 Ank_4 Ank_5 Ank Ank_4 Ank_3 Ank_2
Ank_5 Pfam-B_1873 Ank Ank_3 Ank_5 Pfam-B_4408"
/fullproduct="qcoverage 99.7975708502024 , hcoverage
100.612244897959 , similarity 98.8 , identity 98.6 ,
evalue 4.61e-297 , alnlength 493 , Bacteria ,
Proteobacteria , WP_114517457.1 ankyrin repeat
domain-containing protein [Wolbachia endosymbiont of
Cylisticus convexus]"
/translation="MADNLSLELIKCLINQPGLDVNVRGLNGKTPLHCAIEFDELSMV
DLLLTKKNINPFVEDNEGKTSLDYAKEGKKAEILQALINHKYGSEQDSLLHLAAMIGE
VNAVRYLIRKGIDVNVRNALHHTPLHLASGIGHENIVKILVEEGNAEIDVFDARNQTP
MHYAVNNKKLEIVKLLLKLGADVNSARMGQNSMKLSPVHIAVSNTNYDERDLCLDILK
CLIKEPNAQVNLQDYENKTPLHYAERLKTIEVLLTREDIDPLIRDDNGKTPFDYAKPE
IKKALMSNKYGSEKNSLLHLAAQRGEIELVESILKEEIDIDIVNNKGLSPVYLAAEKG
HLHVVKLLLKKGANYTPVLHLAIKSNNLELLKVLFTEKNGALLCRDTVINFPTLHNKY
IAQREIADKRMKKHNNIICICITVSAVAMAAYIGLTATTISSAIIFATTTGIFALIIA
LMISEMSKRYIEKEFQKKMFMELEECSSTVNDVEIEPIMSRCRQ"
/product="ankyrin repeat domain-containing protein"
/besthit="qcoverage 99.7975708502024 , hcoverage
100.612244897959 , similarity 98.8 , identity 98.6 ,
evalue 4.61e-297 , alnlength 493 , Bacteria ,
Proteobacteria , WP_114517457.1 ankyrin repeat
domain-containing protein [Wolbachia endosymbiont of
Cylisticus convexus]"
gene complement(11385..11870)
/locus_tag="wEsol_00226"
CDS complement(11385..11870)
/locus_tag="wEsol_00226"
/codon_start=1
/transl_table=11
/fullproduct="qcoverage 100 , hcoverage 100 , similarity
99.4 , identity 99.4 , evalue 3.65e-101 , alnlength 161 ,
Bacteria , Proteobacteria , WP_077188119.1 hypothetical
protein [Wolbachia pipientis]"
/translation="MEKITQTEWAREIGVSKQYVYYLVKKGIVELEDGLISREQANEA
VAAIRDPSQPLRRKNPENENTNNLSTMLLKTRIKNEMERGKLLEAKAKAGIGELVAVE
EVKRDAFNVARVVRNNLLNIPNRVSALLASLSDTEKIHKTLTEEITNSLQELSNTKFQ
I"
/product="hypothetical protein"
/besthit="qcoverage 100 , hcoverage 100 , similarity 99.4
, identity 99.4 , evalue 3.65e-101 , alnlength 161 ,

```
                     Bacteria , Proteobacteria , WP_077188119.1 hypothetical
                     protein [Wolbachia pipientis]"
     gene            complement(11887..>11993)
                     /locus_tag="wEsol_00227"
     CDS             complement(11887..>11993)
                     /locus_tag="wEsol_00227"
                     /codon_start=1
                     /transl_table=11
                     /product="hypothetical protein"
                     /translation="FVDVTIKRWQIYTGRDAILAGTGKTFSEIQEENL"
                     /besthit="qcoverage 100 , hcoverage 8.43672456575682 ,
                     similarity 100 , identity 100 , evalue 1.04e-13 ,
                     alnlength 34 , Bacteria , Proteobacteria , WP_208571699.1
                     DNA modification methylase [Wolbachia pipientis]"
ORIGIN
        1 ttctagatca agcgttactt ttccttcttt cacctctgtc atttttactt tttctagctt
       61 aaatctttcc tcccaacttt gtagagcttc tgctactgct gaatagattt ccaaagttaa
      121 atctctattt attggcttat ctactaattc aaatagtctt gacccataat ctctcctcat
      181 tactctactg ttaataggag tggtcagtat atcaattatt gattgtttca gatgttctat
      241 tccttctaat tcttttcctg ttttggagtc catccctttc atttttagcc tgcaaacacg
      301 ttttcactgc cttttattac tttaaaccca catgaaacta tatctcctac tcttcccgct
      361 ccaaacccat ttgcaaacac tgtttttgat ccttgaacca ttacttctcc ttcactaaaa
      421 ctttctcctt gtctgcacat tggcttacca tttacaaaaa cattgttact accactaatg
      481 caaaaatgcg gtgttacttc tacacaataa tctcctactc gtacaaccga tttattcatt
      541 taattaaggt ttattctatc tgctttcagt tttatttcgc tcttcgtcat ctctatgctc
      601 gattccccag ctttcagtgt tattttgtct actacttcaa tctctaaatg atgcttctct
      661 ttatcatata acaatcttgt tccatcctga aacttcacac tatagtattt tctaaaatga
      721 atgataaatt tccattatgg tattaccaac caaaagttgt aagtctgtat atttatgcat
      781 taaaggtctg aggcggcttt tgatggtatt ccaacaatgc tctatagggt tcaaatctgg
      841 tgaatatgtt ggtagataga gcaaacggca tctagcatta tctattaact cctttgtttt
      901 gggagtttta tggaatgtgg cattgtccat aactattgta gtaccatgtg gcaatttagg
      961 taataacatc ttctctaacc atgtattgaa tacctctttg tcacacccac ctttgaaagt
     1021 cattggtgca ataaatctct ttccaatcca cccgcctatt atactgattc tctctcgttt
     1081 tcttcctgga atatctgcat aaattttctt tcctattgga gcacgtccat actctcggta
     1141 taacctatta tcaactcctg cttcatctat atataagatg ctggaatggt ctatttttga
     1201 gattttatcg gtaaatcgct gcctatcttc ttgattgcgc tcttgatata gtgtaacttt
     1261 tttttaaagt aattcttagc tgtttcagcc tataccaaat tgaatttatt ccaaatccaa
     1321 gattttgttt catctctgcc agcgtatgat ctggattctt tttaacatat tcttcgagta
     1381 tttttgggtc tatttttcga ataaagctgc cgtttttcga tggccttagg ctttcaccag
     1441 cggctttctt tcttaaccag cggtacagag ttgctattcc tatctccaaa agctctgcca
     1501 cctcaacctt tgacttccct ttttctatca tcgatactgc cctttccctt aaatccacac
     1561 tatacgccat tataactcaa atagcttatt atacctttat catttacttt gggaagtact
     1621 atatttattt ctttttttatt ctctggtgca gcatacttct gttgatatat tccagctagt
     1681 actactccta atgataactc tcccaatggt gacaatatta ctacctgttc atttatgctt
     1741 ggtggtagcc agcttctttc ctctcctgct ctagaagtta tccatggaag aaaatctgtt
     1801 aaaagttctc ctatcttcac tcttactttt gctttctcat aatctacttc ttttacaact
     1861 cctattcgta caatgtttgc taactttctt tgcagctctg aaatagcaaa attactctct
     1921 aacattttct tctattataa tcgtatgtgg cttaatctta ttttctgtcc atatcgattt
     1981 acctaaatga agctgatgac tccaatcaac catccacacc aaatatgcat ctaattcggg
     2041 tctaaatccg tcaatttctg cagagataaa ttctcctggt gaaacatttt ttatattcca
     2101 tgtatttttg tttacaactt ttgccacctc agcagctaat gttctgacaa taatagctgc
     2161 atttctattt gtgctatcaa tcacaattcg tgcttcaaat cttgctttca gtgctaattc
     2221 ttctgttcct ggatcttttc ccgattctaa actcacaagc tctacaaata acgctggcgc
     2281 taataattct ttccttatcg atggataaat ttcacaagtt tgaattgctg gtatttcttt
     2341 cttcagcgta gtgaagattg cgttatgcaa atctttaaaa ttcatatatt tcttgtaata
     2401 tcacgttcaa agaacttttc aaacacttcc tcaacctcat aattaacaag attccctatt
     2461 atccttgaag cctcaggttc gagtgacaat ttaacttcct ttattggcaa tgctgctctt
     2521 ccttcacgtt taaacatacc gctatttcct tttggcataa ctgctgcaaa tcctcctata
     2581 aactcatgct ttcctacttt cgatcctctt cttgtttttt gtattttgcc aattgtcgat
     2641 gctctaatgt cgtagagatt tgctctaatt aacacttcta atctgcttgt tttgctttta
```

2701 aaaattctta atctctttct tatcaaactt aattttatct tcttttcctc actgatttcc
2761 ttagctgctt gcgcttttaa ccatagtgct gttctattta gtgcttttat agctgccagt
2821 tttactttct gctctttttc gtcaatattg tagactattt cattaatatt gttactaatt
2881 ttaatatcaa acattttcta ttgcctcaat tttccatatt tcactcgaag aatcttttaa
2941 cggtggttca aaaatcttgt agaacctttt ttcaatcttg atataatcac caatgctaaa
3001 ggaagtaacg tcctgatcac gtatttctat agaagcaatt tgctgagtta atgctccatc
3061 tgcacctaga gagtacgtag tctcaggacg tttaattagc acttttattc tacgaatgcc
3121 ctccttattt ttattacaat atgtggcttc cactcctaaa tgctcaaaac aatcttctaa
3181 taattttcct atattcatca actagaacta atttttacca aaactcccgg acgatggcac
3241 attggcaatg gattcgactg cgtatgtaaa tcagttcctc tatcaaatct tcttggctct
3301 tgttttgcat agagtggctg tccaagagta ttcactgttt cattaaaatc tgctggtgca
3361 aaatatgttg taaatgtgct tgctgttcct actggaaaac agtgccctgt atctttctca
3421 ataaatcttc ttacggttcc ttcagggtcg gttgcttgcc ctctatattc ctcaaatgtt
3481 atgccacaga acgtaaatcc tgacctcata tcatttcgaa gtgctgctcc ttcttgccat
3541 ctttcatatg cttcttttac tttagcatga gaagttaatg catcaaaaaa ctcagggctt
3601 accaaggcat gaattccagt catatattca ccacttagat tatcttctat gtgccggagt
3661 acttccagac acttacgttt tacgtcagtt gttgctgttc caagggcaaa atttactact
3721 tttggtgtta tttcaaattc gttgtacaga tttaataatc ctgacccatc agcatctaaa
3781 attattcctt tgagcgctcc cattcgcaaa tgctctaatg ttattgcatg tttgtttctc
3841 attagctgca aatgatcagt tattacatct gcaagcgctt taagttcact ctctgatcca
3901 aatgccctta ttccttgtac ttcttctggc agcactacat catcatgcgg aatgtgcgga
3961 atcgtaaatg ttcttacctt tctttttcct ctttttccta ctgttgctgg tgctccgggt
4021 acttgcgttg gtagtaaact taacactccg ttgtgttctt ctatcgtaat atgtctaaat
4081 cttactgacc tacttggaaa caaatttaaa ttttcaacac gtccataatt tatcggcaat
4141 atattcatcg catttgttag tgccgtcatg ctaaatgctg tattcgtaaa tggattttgc
4201 attctttttc ccccttatta atttaagtta cactcccttg cggacaatga tccctcgtcc
4261 ttcaagttgc tttattgctg cagctttttg ctcttcagtg atacctgttg gccacacaat
4321 tgcatgatct gctagcattg ctatacgtgt aatgattact gctttggcat ttctgtcgc
4381 atttacatca cttactatcg cacctattgc tgtttgtgta ccatctgttg cagttggatt
4441 tattatctta atcatgttat ccttatcact accaacaact gtaccaagtt taagattttg
4501 tcccttggct acagttattt ggtctcttga atatagactt gatgcctcat actttaataa
4561 gtcacctaga ttattttgtt cgattataca actcataaaa tttcctcatt tattttcccc
4621 tttagtttta ccgccatgtt tgtaattgtt gtacgcggcg gttataaaaa aacttaaata
4681 cttgattgcg ctctagtcct tgctacctcc atcatcaact cttctcctga attctgcggt
4741 attgcactca gtatctctgt ctttttcgtt ctctctgcaa gtaattccat taaaacttcc
4801 ctggcttgct caatacttac gccttgctct ataaattctt ctattttctc tggcattcgt
4861 gataagttac ataaacgtat taattcaaga acttcagtac gatacttagt taaattatca
4921 gtttctaggt cagttgtagt ttgttcattc atagtaatac tcctgttttt attaatagat
4981 gaaagaattg taattccatc tgcaagacct atttctattg ctctctcgcc aaaatatagc
5041 cctgcctccg ttgatcgaat cttttcaatt gaaagacctc tgttcctcgc tattagctgc
5101 acaaacattt catatagtcg gtctacttct ttttgtaagc tttccagact ttcagacgtc
5161 attggctcat gtggatttaa atcatttttt ctacttcctg caaatactgt ggtatatttt
5221 attccacatt tttcattaaa tccactttga tctatatgac ttgcaattac ccctatgctt
5281 cctactcctg aggttctgct cacaaatacc ttttcagcgc tagaggctat agcgtacgca
5341 gcagaatatg catcatcatt tgctatcgct attatcctct tttttgctct tgattcataa
5401 ataaagtcag ctaggtcaaa tataccgttt acctctcctc cggggctatc tatttccagt
5461 ataattgtct ctatgctact atctgctaat gcttgtgtaa tttgttcttc tatttgctca
5521 tatgatgtca ttccgagcat ttcatcaaat gcaccaggtt ttttagttaa aatgccatgt
5581 atcggtatta ttcctctttc actatttctt actgcatgtt ttatattttt aaagataggc
5641 tgctttcctg tttgtagtga cagtaactca aaactccttg gttctaccat tacacatctg
5701 tttagccatt gttgtttcat atcacctctt gatttgcagt aacctcagaa tcaaaactta
5761 agcccagttc gcttgcacgt ctttgatctt ctgctatttc ttggtcaatt tcttcaacat
5821 cgtaacccat ttctgatact acttccgatc gactcttaaa tccatttctt actgccattt
5881 gttgtgcttg ctgatctttc agtggatcca cccaatcaaa tccctgtggt atccatttta
5941 cttcttcttt cgctgctttt actacttttt catctatact tagttctcca caaagcactg
6001 ctaattctag ccatctattc caaataggtc tacaaaactg gaacactatc acgttatgct
6061 gtaacattgc acaccttctg cgaaactcta tcagccctgc tcgaatggat gaataattaa
6121 cgccggttaa atctcctgtt agttgctcgt atgttattcc tgttcctatt gctattgccc
6181 tcagttgctg tcttataaac gcttcataac ttcctccaac atctgacggt tctgaaaatt
6241 ttatgtcttc tcctgggtct aaaagctgca ttgttcctgg ctctaaacct gataatgcta

6301 ctccttgctc attcgcttcg ccttctccta aaatatttgc ctcaggatca agtcttgtaa
6361 taaaccctgc aaacattgct gctgtctttt ttctcactaa ttctgcatca tcgtattgat
6421 caagctcata aagtttcaga agtatactcg aaagccaagg ctcccctcta atttgcccag
6481 gtcttagtgg tctatagata tgtaaaacat cgtttgctgg tactctcact gactctccaa
6541 acgaaccttc tcctggatgt tctctaaata agtaatatgc ctctctttgc ccaagcttgt
6601 taaactcaat cccgtttctt attacattac catttcctaa agtttgattt gttttattat
6661 ctaaatgttc agactcaagt acttgcagtt gtaatggcac agaaaatcta tcttccagct
6721 ttctcgttct taaacgtaca aaacattctc ctccctctat catacttctg catactagag
6781 cttgtaatcc ataaaaatca cttattccac agctatctgc ttcatctgtc catcttagcc
6841 ataattcttg caccttcttt cgaaattctc catctcttgc ctttgattgt ggttttattc
6901 ctgttccaat cgagttactt actattgtat caattatatt tgctgcatat ggatttttc
6961 ttaccatatc acgtgatcta ctacgcaaag tttcaaggct ttgagagagt aaattgttta
7021 tacttcctgt ttccccctgc caatacatta ctcttcttcc tgagcctgct gcatcccaag
7081 cagaactttt gatttttggc ttactaaata gttgtttgaa atttttcagc atcatactaa
7141 ccaaaattct cttaattctt tcaaagttcc gttaaattta tttctgtcac attttccgat
7201 tccttctact tcatgtggct ctggatttgt tttaccgtca gtatattgcc ataaaatcca
7261 atcattccag ccttttggta atgtgagctc ttttctccag cttgctatcc ataatggaca
7321 ttttgtcaaa gttggcgtgg caaaatcctt aagaaaatag ctactgccat aaattagggg
7381 taatcttcct gttacttctt caactctctt tacaaattcc tcagcttgct taactgcaat
7441 atttttgcca cttttgtttt cttcaatatc cagcgcaagt atggtatttt tgctttcgcc
7501 taccgttttt agaaaatgat cagcttgatt cttgccgttt ccttctgtgc caaaatgata
7561 tgctccccag agtaatcctt cttcttcagc tttctttctt ctcttcgcat actttgaatc
7621 cacatgtttt agcccttgtg ttgccttgtg aattactcca actataccat tctctttgtc
7681 tagcttaaaa ttcacatttt cttgccaatg tgacaaatct attattccat ttactggact
7741 ctctctattc tcccatagtt ttataaaagt ttttaattgc tctaaaaatt cagaatattc
7801 ctcttttaca acttttctca gaaaatttc cctcatataa ctcctttatt tgttgcaaaa
7861 ataatctttc gtttaggctt cattccagca atctttagtt cacctttgat gcgttgtctg
7921 aggctgagca aatcatttat ctgaacttca gcatatctca ccacatggtc accatatgca
7981 attgatacta ctcgctctcc gttttgtagt ttctttatcg cttcttcgac ttgaattaga
8041 taatcttcgt tatacattta ctactcgctg atccatttgc ttttcactat tttctttgat
8101 tttttacttt ctggtttttc acttaaacta ttccatttac tctctggcca tctatctatt
8161 cccagtgcta tcgatgctgc tcttgcataa atcctacagt ctaatacttc atttctttct
8221 cttacctttt gccactcttg tttggtataa ccttttacta ctttgctgac taattgctct
8281 gccgttagct gcttaaaata ttcaggtgca tactcgggaa aatgacagta tcctgctgga
8341 gcttcttcac cgtcctttaa aacattaagt aattgaaaaa gttctgactt taaaatcgat
8401 actcccactg gccagagctt tattcctctt tttagctttt gaccaccaac tgttatatct
8461 actctacttg ggctgctaag tggtactaga gctttattta cacctttcac tgccattact
8521 cttccagagc cttgatgacc tcttacccaa ttgtatactt cttgtgtcgc atacccagca
8581 tcaaccgcca tcatacttat catgtattca agaccatttt caccgataaa atgatgattt
8641 aagagctcag agagttttcc ccatacttct cctcttccag tatcaccttc aaatactcgg
8701 tattctattg accaactttc gcggcttttt ccccatgcta caacttctac ttctaaacga
8761 tctttttgga catctactcc tgcagtaagc accacttctc tcctaggtac tgtgcctacg
8821 ggaaaaaatt ctctcctgtt aaataattgc ttccaatctg gtacttctcc tttgtctacc
8881 caggtttctc caagcgtagt atttatccat acttttagta attgttcact ttcttttgca
8941 tgaagaaaat cttctactgc ttgagtccag ctataccagc ctactggact ataaagactt
9001 gaaagatgaa atcctttttt ctcacctttt actctattag taggtctcca ttctccacgc
9061 tcaagcatct ctgtcttttg atgattttct attttgccgc tacattcagt gcagacataa
9121 tgtgctgttc ttgagtcgtt gttttcccat ttaatttgtg accattttag aacttgatag
9181 taattacaat acggacatgg tacaaaaaag tactgcttat ctgttgcttc aaattctttc
9241 tcaattctgc ttattccatg aatcgttggt gttgatacta aaaaaatctt tcgccgtgca
9301 gatgtattag ttcgagcaat actgagcagc actggatctc cttcaccacc tgaatctcct
9361 ggataggcgt ctatttcatc aagaaagaga tattttactg gcatagacct cagtcctaca
9421 ctgctgtttg ctccagttat tactactatt ccacctggaa attccttact ttgtacagta
9481 ttgcctgagt ctcttgacct tgggtctttt actttactct ttaaacatgg cgtactctct
9541 attaacggcg caaatcttcc ttttgaccaa cgctttccca tttcaactgt tggctgcaca
9601 actaacattg gaccaggagt ctgatatata atatagccta tccaattgtt accagcttct
9661 gttcctccaa tctgcgctcc tttcatgaat actacttttt ctgctggtga ggacggagaa
9721 agtgaatcca tgatttcttt taaataagga gttctttctg ttctccattt acctggctct
9781 gatgctgcag ttggcgctaa aactcgatac tcatttgccc actctgatac tttaagctct
9841 ggatctggtt ttaaaccttc agagaaagat gtggcgtata tcattgtctg catctactca

```
     9901 taattggttc tatctcaaca tcattaacag tagaactaca ctcttccaat tccataaaca
     9961 tctttttctg aaattccttt tctatatatc ttttgctcat ctcacttatc attagtgcta
    10021 taataagcgc aaatatccct gttgttgttg caaaaatgat tgcactgctt attgttgttg
    10081 ctgttaaacc tatatatgct gccattgcta ctgcgctaac tgtaatacag atgcagataa
    10141 tattattatg tttttttcatc cttttatctg ctatttctct ctgtgctata tatttattat
    10201 gaagggttgg aaaattaata acggtatctc tgcagagcaa tgcaccattt ttttcagtaa
    10261 ataaaacttt aagtaactct aaattgtttg atttaattgc taaatgtaaa acaggtgtat
    10321 aatttgctcc ttttttcagc aataacttta ctacatgtaa atgccctttt tccgcagcaa
    10381 ggtaaaccgg tgataaacct ttattattta caatatcaat atcgatttct tcttttaaga
    10441 tagactctac aagctcaatt tctcctcttt gtgcagctaa atgtagtaga ctattctttt
    10501 cagaaccgta tttattactc atcaaagcct tctttatctc aggtttagcg taatcaaatg
    10561 gtgtcttacc attatcatcc ctgattaaag gatctatatc ctctcgagtt agtaaaacct
    10621 ctattgtttt aagtctttca gcgtaatgta gtggcgtttt attttcgtag tcttgcaaat
    10681 ttacttgagc attaggctcc tttattaagc atttgagaat atcaagacac aaatctcttt
    10741 catcgtaatt agtattactt acagctatat gaacaggcga caatttcatt gagttttgtc
    10801 ccatgcgtgc actatttaca tctgctccaa gtttcagcag taactttact atctctaact
    10861 ttttgttatt aactgcatag tgcattggtg tctgatttcg tgcatcaaag acatctattt
    10921 cagcatttcc ttcttccact aaaattttga caatattctc atgtcctatt cctgatgcta
    10981 gatgtaatgg tgtatgatgc agagcatttc gtacattaac gtcaatacct tttctgatca
    11041 aatatctcac tgcattaact tcacctatca ttgcagctaa atgaagtaag ctatcttgtt
    11101 ctgatccgta tttgtgattg attaatgctt gtaatatttc tgcttttttta ccttctttgg
    11161 cataatctaa ggaggttttg ccttcattgt cctcaacaaa aggattaatg ttcttcttcg
    11221 tgagtaataa atccaccatg cttaactcgt caaactctat agcgcaatgt agtggtgtct
    11281 ttccatttaa tcctctgaca ttaacatcta atccaggttg attgatgaga cattttatta
    11341 actccaaact taagttatca gccattttat attcaaaatc tttttatat ttgaaattta
    11401 gtattagata attcttgtaa tgagtttgta atctcttcgg ttagcgtctt atgaatcttt
    11461 tcagtgtcgc tcagtgatgc aagcagtgct gaaactctat ttggaatatt aagcaaatta
    11521 ttacggacaa ctcttgctac attaaatgca tcgcgcttta cttcctctac tgctacaagt
    11581 tcaccaatcc cagccttagc tttcgcctca agcagtttac cacgttccat ctcatttttt
    11641 attcgcgttt ttagcaacat tgtagaaagg ttatttgtat tttcgttttc tggatttttc
    11701 ctcctaagtg gctgacttgg atctcttatt gctgccaccg cttcatttgc ttgttctcta
    11761 ctgatcaaac catcctccaa ctcaactatt cctttcttta ctagataata gacatattgc
    11821 tttgatactc ctatctctct tgcccattcc gtttgtgtga tcttttccac tttttttgct
    11881 tctttttac aaatttcttt cttgaatctc tgaaaaggtt ttgccagtac cggctaaaat
    11941 ggcatctctt ccggtatata tttgccaacg ttttatagtt acatctacaa att
//
LOCUS       JAQZAU010000032            3012 bp    dna     linear   ENV 17-MAR-2023
DEFINITION  MAG: Wolbachia pipientis isolate wEsol contig_26490, whole genome
            shotgun sequence.
ACCESSION   JAQZAU010000032 JAQZAU010000000
VERSION     JAQZAU010000032.1
DBLINK      BioProject: PRJNA929258
            BioSample: SAMN33142973
KEYWORDS    WGS; ENV; Metagenome Assembled Genome; MAG.
SOURCE      Wolbachia pipientis (insect metagenome)
  ORGANISM  Wolbachia pipientis
            Bacteria; Pseudomonadota; Alphaproteobacteria; Rickettsiales;
            Anaplasmataceae; Wolbachieae; Wolbachia.
REFERENCE   1  (bases 1 to 3012)
  AUTHORS   Fiutek,N., Couger,B.M., Roy,S., de la Torre,J.R. and Connor,E.F.
  TITLE     Genomic assessment of the contribution of the Wolbachia
            endosymbiont of Eurosta solidaginis to gall-induction
  JOURNAL   International Journal Molecular Sciences
REFERENCE   2  (bases 1 to 3012)
  AUTHORS   Fiutek,N., Couger,B.M., Roy,S., de la Torre,J.R. and Connor,E.F.
  TITLE     Direct Submission
  JOURNAL   Submitted (10-FEB-2023) Biology, San Francisco State University,
            1600 Holloway Ave, San Francisco, CA 94132, USA
COMMENT     ##Genome-Assembly-Data-START##
            Assembly Date          :: JUN-2019
```

```
            Assembly Method        :: FLYE v. 06-2019
            Genome Representation  :: Full
            Expected Final Version :: Yes
            Genome Coverage        :: 100.0x
            Sequencing Technology  :: Illumina HiSeq; PacBio Sequel
            ##Genome-Assembly-Data-END##
FEATURES             Location/Qualifiers
     source          1..3012
                     /organism="Wolbachia pipientis"
                     /mol_type="genomic DNA"
                     /submitter_seqid="contig_26490"
                     /isolate="wEsol"
                     /isolation_source="galls on Solidago altissima"
                     /host="Eurosta solidaginis MN-2018-EFC"
                     /db_xref="taxon:955"
                     /environmental_sample
                     /country="USA: Cowling Arboretum, Carleton College,
                     Northfield, MN"
                     /lat_lon="44.48 N 93.13 W"
                     /altitude="280 m"
                     /collection_date="2018-11"
                     /metagenome_source="insect metagenome"
                     /note="metagenomic"
     gene            281..1627
                     /locus_tag="wEsol_00228"
     CDS             281..1627
                     /locus_tag="wEsol_00228"
                     /codon_start=1
                     /transl_table=11
                     /pfam="DEDD_Tnp_IS110 Transposase_20"
                     /fullproduct="qcoverage 100 , hcoverage 100 , similarity
                     100 , identity 100 , evalue 0.0 , alnlength 448 ,
                     Bacteria , Proteobacteria , WP_155968747.1 MULTISPECIES:
                     IS110 family transposase [Wolbachia]"
                     /translation="MIKAKNKLEIVNPDTTGIDIGSAVHYVCVPEGRDEQRVKKFGCF
                     TVDLHNLAGWLKKCKVRTVAMESTGVYWIPLFQILESYGFEVKLVNARYIKNVPGRKS
                     DVQDCQWLQQLHSYGLLHGSFRPDDQMCVLRNYVRQRSNLIESASTHILRMQKALIQM
                     NVQLHKVISDITGVTGMQIIKAIIEGERNPEKLSELRDVRIRNDRSTMAKALKGDYRK
                     ELLFVLKQEFELYNIYQEKIAECDKSIEQYYKTFETKSDENKRCSKIKNRFSKNRPDF
                     PLHEELYRVTGIDFTKVPGLDVLTIQTIISEVGLNHDKWPSEKHFTSWLGLSPANKVT
                     GEKVFGTRTRKVIARAANAFRMAAQCVSRSNGGIGAYCRRLKKRLGAPKAITATARKL
                     ACIFYSMLKYGQEYVEKGIDYYEKLYKEKIVKTLSKKASEFGYVLIKKELLAERVS"
                     /product="IS110 family transposase"
                     /besthit="qcoverage 100 , hcoverage 100 , similarity 100
                     , identity 100 , evalue 0.0 , alnlength 448 , Bacteria ,
                     Proteobacteria , WP_155968747.1 MULTISPECIES: IS110
                     family transposase [Wolbachia]"
     gene            1810..2118
                     /locus_tag="wEsol_00229"
     CDS             1810..2118
                     /locus_tag="wEsol_00229"
                     /codon_start=1
                     /transl_table=11
                     /product="hypothetical protein"
                     /pfam="Pfam-B_5362"
                     /translation="MDVSKGGIEMSFTTEVGEISIYLSPSEKGSNKIKVEIDGENRAR
                     FNELKNRGKSLGENCLLGGKSVEEAIEHESFERYGSVPNTAMKEPSLGCVAKRLSGNK
                     "
                     /besthit="qcoverage 98.0392156862745 , hcoverage
                     44.8430493273543 , similarity 95.0 , identity 95.0 ,
```

```
                     evalue 7.70e-59 , alnlength 100 , Bacteria ,
                     Proteobacteria , ONI56661.1 protein, WF-1 [Wolbachia
                     pipientis wUni]"
     gene            2156..2536
                     /locus_tag="wEsol_00230"
     CDS             2156..2536
                     /locus_tag="wEsol_00230"
                     /codon_start=1
                     /transl_table=11
                     /fullproduct="qcoverage 100 , hcoverage 100 , similarity
                     100 , identity 100 , evalue 1.16e-87 , alnlength 126 ,
                     Bacteria , Proteobacteria , AOV87106.1 transposase
                     [Wolbachia endosymbiont of Drosophila incompta]"
                     /cog="COG3293 COG3293 124 Transposase and inactivated
                     derivatives"
                     /pfam="DUF4096"
                     /product="transposase"
                     /translation="MRKKYPTDLSEREWARIEKHFRVSYKKGGRPPKYSKREILEAIF
                     YVLRTGCQWRYLPNDFPLWKTVYEQFRQWKKQGIFEKMNYEITKYSRRKIGRNEQPSA
                     CIVDSQSVKTTEKGGSKAMMEVKK"
                     /besthit="qcoverage 100 , hcoverage 100 , similarity 100
                     , identity 100 , evalue 1.16e-87 , alnlength 126 ,
                     Bacteria , Proteobacteria , AOV87106.1 transposase
                     [Wolbachia endosymbiont of Drosophila incompta]"
     gene            2647..2988
                     /locus_tag="wEsol_00231"
     CDS             2647..2988
                     /locus_tag="wEsol_00231"
                     /codon_start=1
                     /transl_table=11
                     /fullproduct="qcoverage 100 , hcoverage 100 , similarity
                     100 , identity 100 , evalue 1.53e-79 , alnlength 113 ,
                     Bacteria , Proteobacteria , AAS13810.1 transposase, IS5
                     family, OrfB [Wolbachia endosymbiont of Drosophila
                     melanogaster]"
                     /cog="COG3293 COG3293 124 Transposase and inactivated
                     derivatives"
                     /pfam="DDE_Tnp_1_2 DDE_Tnp_1"
                     /product="transposase, IS5 family, OrfB"
                     /translation="MRTKYTKVKKMWADMGYQGRNLKNHIKEEYDIDIEIVKRPPCRF
                     WVHKDTPPELLPTREQGFKVQPRRWVVERTFAWVNRNRRLSKEYDLLTTSTENFIYLA
                     MSRVMLKREYA"
                     /besthit="qcoverage 100 , hcoverage 100 , similarity 100
                     , identity 100 , evalue 1.53e-79 , alnlength 113 ,
                     Bacteria , Proteobacteria , AAS13810.1 transposase, IS5
                     family, OrfB [Wolbachia endosymbiont of Drosophila
                     melanogaster]"
ORIGIN
        1 tgtattagga gcgtcgaaca agaaaaaaat cttgttaaac cggaaagaaa ctatttcttc
       61 acttccatag caatttttat atataattta tcaagaagta cttctgcagt tataaccgac
      121 tgatcaatgg tgcttaagtg cccgtttagg agcgtggttt tggaggaacg gttaaaccac
      181 tgatggagtc tattatgact acctcgatta gaagattgta taatctaacc gtcccaagct
      241 taagtgcttc tccattataa gttatagtag gataattacc atgataaagg caaaaaataa
      301 actagaaata gtgaatcctg atacaacagg aattgatatc ggctcagctg tacattatgt
      361 atgtgtacct gaaggaagag atgaacaacg tgtcaaaaaa tttggatgct ttacagttga
      421 tcttcataac ttagcagggt ggttaaagaa atgtaaagtt agaacagtag ctatggaatc
      481 aacaggagta tattggattc ctttgtttca aatacttgag tcatatggat ttgaggtaaa
      541 attggtaaat gcacgatata ttaagaacgt gcctggtaga aaatccgacg ttcaagactg
      601 tcaatggtta caacaattac acagctatgg attactccat gggtcgttta gacctgatga
      661 tcaaatgtgt gtgttgcgta attacgttag gcaacgtagc aacctgattg aaagtgcatc
```

```
      721 cacacatatt ctacgtatgc aaaaagcgtt aattcaaatg aacgttcaat tacacaaagt
      781 cataagtgat attactggag tgaccggtat gcaaattatc aaagcaataa ttgaaggtga
      841 aaggaatcct gaaaaattat cagaattgag agatgtacgg ataagaaatg atagatctac
      901 catggcaaaa gcgctaaaag gcgactacag aaaggaactc ttatttgtgt taaagcaaga
      961 atttgaattg tataatattt atcaggaaaa aatagcagaa tgcgacaaaa gcatcgaaca
     1021 atattacaaa acatttgaaa caaaatctga cgagaataag cgatgcagca aaataaagaa
     1081 taggttttca aaaaacagac cagactttcc tttacacgaa gaactgtata gagtaaccgg
     1141 tattgatttt actaaagttc ctgggcttga tgtgctaact atacaaacaa ttatctcaga
     1201 agttggtttg aaccacgata aatggccatc agagaagcac tttacttctt ggttggggct
     1261 aagtcctgct aataaagtta caggggaaaa agtatttggg acgagaactc gcaaagttat
     1321 tgctcgtgct gcaaatgctt ttaggatggc tgcccagtgt gtgtcaagaa gcaacggtgg
     1381 gattggtgca tattgcagaa ggttaaaaaa acgactagga gcaccgaaag cgattaccgc
     1441 tacggctagg aaattggcgt gcattttta tagtatgcta aagtatggac aggaatatgt
     1501 agagaagggc attgattact atgaaaagtt atataaggaa aaaatagtga agactctaag
     1561 caagaaagct agtgaatttg gttatgtctt gattaaaaaa gagctactag cagaaagagt
     1621 ttcttagaag tagacaagga taatagatat ttttgtatag agtactcgca agatagtatt
     1681 gttgaatttg cagaaatcct aaacaatgaa aaagccaaag acttaaattt aagagttggc
     1741 atactcaaaa ttggagaaag tatagtaaga gttgaaaatg tagaagaaat agggggaaaa
     1801 agaaattaca tggatgtctc aaaaggtggt attgaaatgt cttttactac tgaggtagga
     1861 gagataagta tttatctaag tcctagtgag aaaggcagca ataaaataaa agtggagatt
     1921 gatggggaaa acagagcaag gtttaatgag ttaaaaaata ggggaaaaag tttaggggaa
     1981 aactgtcttt taggaggaaa aagtgttgag gaagctatag agcatgaaag ttttgaaaga
     2041 tatggcagtg tacctaacac tgctatgaag gaacctagct taggttgcgt tgctaagagg
     2101 ttgtccggaa ataagtaaaa ggctataata tctgaaattt tagtacgggg taaagatgag
     2161 aaaaaagtat ccaacagatc taagcgaaag ggaatgggca agaatagaaa aacacttcag
     2221 agtatcatac aagaaaggag gaaggccgcc aaagtatagc aaaagagaaa tattagaagc
     2281 aattttctat gtattgcgta cagggtgtca atggcggtat ttaccaaatg attttccgct
     2341 atggaagact gtgtatgagc agttcaggca atggaagaag cagggaattt ttgagaaaat
     2401 gaattatgaa attacaaaat atagtagaag aaaaatagga aggaatgagc agccgagtgc
     2461 ctgtatagta gatagtcaat ctgtaaagac tacagaaaag gggggatcaa aggctatgat
     2521 ggaagtaaaa aagtaaaggg tagaaaaagg catataatta cagacactca gggttttata
     2581 ctaggttgtt acgtaggcgc tgctaacgaa aatgatagag atggtattaa aatagcatta
     2641 aacaatatga gaacaaaata tactaaagtt aaaaaaatgt gggctgacat gggataccaa
     2701 ggaagaaatt taaagaatca cataaaggaa gaatatgaca tagatattga aattgttaaa
     2761 aggcctccat gtagattttg ggtgcacaaa gatacgccac ctgagctact accaacaaga
     2821 gaacaagggt ttaaagtaca gccaagaaga tgggttgtag aaaggacttt tgcttgggtt
     2881 aataggaata gaaggctatc gaaggagtat gatttactca caacatccac tgagaatttc
     2941 atatacctag ctatgagtag ggttatgtta aagagggaat atgcttgaat ttactagttt
     3001 ccggacaacc tc
//
LOCUS       JAQZAU010000033            2559 bp    dna     linear   ENV 17-MAR-2023
DEFINITION  MAG: Wolbachia pipientis isolate wEsol contig_26953, whole genome
            shotgun sequence.
ACCESSION   JAQZAU010000033  JAQZAU010000000
VERSION     JAQZAU010000033.1
DBLINK      BioProject: PRJNA929258
            BioSample: SAMN33142973
KEYWORDS    WGS; ENV; Metagenome Assembled Genome; MAG.
SOURCE      Wolbachia pipientis (insect metagenome)
  ORGANISM  Wolbachia pipientis
            Bacteria; Pseudomonadota; Alphaproteobacteria; Rickettsiales;
            Anaplasmataceae; Wolbachieae; Wolbachia.
REFERENCE   1  (bases 1 to 2559)
  AUTHORS   Fiutek,N., Couger,B.M., Roy,S., de la Torre,J.R. and Connor,E.F.
  TITLE     Genomic assessment of the contribution of the Wolbachia
            endosymbiont of Eurosta solidaginis to gall-induction
  JOURNAL   International Journal Molecular Sciences
REFERENCE   2  (bases 1 to 2559)
  AUTHORS   Fiutek,N., Couger,B.M., Roy,S., de la Torre,J.R. and Connor,E.F.
  TITLE     Direct Submission
```

```
  JOURNAL   Submitted (10-FEB-2023) Biology, San Francisco State University,
            1600 Holloway Ave, San Francisco, CA 94132, USA
COMMENT     ##Genome-Assembly-Data-START##
            Assembly Date          :: JUN-2019
            Assembly Method        :: FLYE v. 06-2019
            Genome Representation  :: Full
            Expected Final Version :: Yes
            Genome Coverage        :: 100.0x
            Sequencing Technology  :: Illumina HiSeq; PacBio Sequel
            ##Genome-Assembly-Data-END##
FEATURES             Location/Qualifiers
     source          1..2559
                     /organism="Wolbachia pipientis"
                     /mol_type="genomic DNA"
                     /submitter_seqid="contig_26953"
                     /isolate="wEsol"
                     /isolation_source="galls on Solidago altissima"
                     /host="Eurosta solidaginis MN-2018-EFC"
                     /db_xref="taxon:955"
                     /environmental_sample
                     /country="USA: Cowling Arboretum, Carleton College,
                     Northfield, MN"
                     /lat_lon="44.48 N 93.13 W"
                     /altitude="280 m"
                     /collection_date="2018-11"
                     /metagenome_source="insect metagenome"
                     /note="metagenomic"
     gene            45..227
                     /locus_tag="wEsol_00232"
     CDS             45..227
                     /locus_tag="wEsol_00232"
                     /codon_start=1
                     /transl_table=11
                     /product="hypothetical protein"
                     /pfam="HTH_Tnp_1 HTH_23 HTH_28"
                     /translation="MAREYTAEFKLEAVKLTNEQRKVGQPVAKVARDLGIRDSVLGKW
                     MKKYNEKSQRQMHFQM"
                     /cog="COG2963 COG2963 116 Transposase and inactivated
                     derivatives"
                     /besthit="qcoverage 85 , hcoverage 70.8333333333333 ,
                     similarity 100 , identity 100 , evalue 8.30e-26 ,
                     alnlength 51 , Bacteria , Proteobacteria , WP_094189595.1
                     transposase, partial [Wolbachia endosymbiont of
                     Drosophila ananassae]"
     gene            760..924
                     /locus_tag="wEsol_00233"
     CDS             760..924
                     /locus_tag="wEsol_00233"
                     /codon_start=1
                     /transl_table=11
                     /fullproduct="qcoverage 100 , hcoverage 100 , similarity
                     100 , identity 100 , evalue 2.93e-28 , alnlength 54 ,
                     Bacteria , Proteobacteria , WP_156768648.1 MULTISPECIES:
                     hypothetical protein [Wolbachia]"
                     /cog="Tra5 COG2801 232 Transposase and inactivated
                     derivatives"
                     /pfam="rve"
                     /product="hypothetical protein"
                     /translation="MSSSINKQLVMDSLLMAVNKRKPAKNLLLHSDQGSQYTSQGYQY
                     LLSIKNIDEL"
```

```
                     /besthit="qcoverage 100 , hcoverage 100 , similarity 100
                     , identity 100 , evalue 2.93e-28 , alnlength 54 ,
                     Bacteria , Proteobacteria , WP_156768648.1 MULTISPECIES:
                     hypothetical protein [Wolbachia]"
     gene            914..1078
                     /locus_tag="wEsol_00234"
     CDS             914..1078
                     /locus_tag="wEsol_00234"
                     /codon_start=1
                     /transl_table=11
                     /pfam="rve_3 rve_2"
                     /fullproduct="qcoverage 100 , hcoverage 100 , similarity
                     100 , identity 100 , evalue 1.56e-32 , alnlength 54 ,
                     Bacteria , Proteobacteria , WP_080717620.1 MULTISPECIES:
                     IS3 family transposase [Wolbachia]"
                     /translation="MSYKGCCYDNSVVESFFSSLKRELPIDTSRHSKQHIKTAIFEYI
                     EIFYNKQRHY"
                     /product="IS3 family transposase"
                     /besthit="qcoverage 100 , hcoverage 100 , similarity 100
                     , identity 100 , evalue 1.56e-32 , alnlength 54 ,
                     Bacteria , Proteobacteria , WP_080717620.1 MULTISPECIES:
                     IS3 family transposase [Wolbachia]"
     gene            1133..1480
                     /locus_tag="wEsol_00235"
     CDS             1133..1480
                     /locus_tag="wEsol_00235"
                     /codon_start=1
                     /transl_table=11
                     /fullproduct="qcoverage 100 , hcoverage 100 , similarity
                     100 , identity 100 , evalue 2.74e-74 , alnlength 115 ,
                     Bacteria;Eukaryota , Proteobacteria;Arthropoda ,
                     WP_155968342.1 MULTISPECIES: IS630 transposase-related
                     protein [Wolbachia]"
                     /cog="COG3415 COG3415 138 Transposase and inactivated
                     derivatives"
                     /pfam="HTH_Tnp_IS630 HTH_Tnp_1 HTH_23 HTH_28 HTH_29 HTH_7
                     CENP-B_N Pfam-B_14738 HTH_8 HTH_17 HTH_24 Pfam-B_2106"
                     /product="IS630 transposase-related protein"
                     /translation="MAYSVDLRERAVSMIEKGKSKVEVAELLEIGIATLYRWLRKKAA
                     GESLRPSKNGSFIRKIDPKILEEYVKKNPDHTLAEMKQNLGFGINSIWYRLKQLRITL
                     KKSYTISRAQSRR"
                     /besthit="qcoverage 100 , hcoverage 100 , similarity 100
                     , identity 100 , evalue 2.74e-74 , alnlength 115 ,
                     Bacteria;Eukaryota , Proteobacteria;Arthropoda ,
                     WP_155968342.1 MULTISPECIES: IS630 transposase-related
                     protein [Wolbachia]"
     gene            1704..2084
                     /locus_tag="wEsol_00236"
     CDS             1704..2084
                     /locus_tag="wEsol_00236"
                     /codon_start=1
                     /transl_table=11
                     /fullproduct="qcoverage 100 , hcoverage 100 , similarity
                     100 , identity 100 , evalue 1.16e-87 , alnlength 126 ,
                     Bacteria , Proteobacteria , AOV87106.1 transposase
                     [Wolbachia endosymbiont of Drosophila incompta]"
                     /cog="COG3293 COG3293 124 Transposase and inactivated
                     derivatives"
                     /pfam="DUF4096"
                     /product="transposase"
```

```
                     /translation="MRKKYPTDLSEREWARIEKHFRVSYKKGGRPPKYSKREILEAIF
                     YVLRTGCQWRYLPNDFPLWKTVYEQFRQWKKQGIFEKMNYEITKYSRRKIGRNEQPSA
                     CIVDSQSVKTTEKGGSKAMMEVKK"
                     /besthit="qcoverage 100 , hcoverage 100 , similarity 100
                     , identity 100 , evalue 1.16e-87 , alnlength 126 ,
                     Bacteria , Proteobacteria , AOV87106.1 transposase
                     [Wolbachia endosymbiont of Drosophila incompta]"
     gene            2195..2536
                     /locus_tag="wEsol_00237"
     CDS             2195..2536
                     /locus_tag="wEsol_00237"
                     /codon_start=1
                     /transl_table=11
                     /fullproduct="qcoverage 100 , hcoverage 100 , similarity
                     100 , identity 100 , evalue 1.53e-79 , alnlength 113 ,
                     Bacteria , Proteobacteria , AAS13810.1 transposase, IS5
                     family, OrfB [Wolbachia endosymbiont of Drosophila
                     melanogaster]"
                     /cog="COG3293 COG3293 124 Transposase and inactivated
                     derivatives"
                     /pfam="DDE_Tnp_1_2 DDE_Tnp_1"
                     /product="transposase, IS5 family, OrfB"
                     /translation="MRTKYTKVKKMWADMGYQGRNLKNHIKEEYDIDIEIVKRPPCRF
                     WVHKDTPPELLPTREQGFKVQPRRWVVERTFAWVNRNRRLSKEYDLLTTSTENFIYLA
                     MSRVMLKREYA"
                     /besthit="qcoverage 100 , hcoverage 100 , similarity 100
                     , identity 100 , evalue 1.53e-79 , alnlength 113 ,
                     Bacteria , Proteobacteria , AAS13810.1 transposase, IS5
                     family, OrfB [Wolbachia endosymbiont of Drosophila
                     melanogaster]"
ORIGIN
        1 gaaaggagtt tttttgggta aaataaaatt ttggaggatt agaaatggca agagaatata
       61 cggcagaatt caaattggaa gctgttaagc tgacaaatga acaaagaaag gtaggtcaac
      121 cagttgcaaa agtagcaaga gatctaggaa taagggatag tgtattagga aaatggatga
      181 agaaatataa cgaaaaaagt cagcggcaaa tgcatttcca gatgtagcac cttatgacaa
      241 agagagattt gatttacaaa gcaaaagtaa caagagaaag agacatttta aaaaaagccc
      301 tggccagtca aaaagagtaa aatattttt tatatagtaa ctgctataaa gtacaggaat
      361 tatgtaggat tttctgctag tggctactat aagtggacca caagaaaaat aagcagtaga
      421 gaatcagcaa ataaagagtt attagcagat attcaaaaaa tatatcaagc ttctaaatgt
      481 agatatggag ctcctaaaat tcatgctgaa ttaaaggctt tgggtaaaag ttgcaactta
      541 aaaacagtgc aaagtattat gcagaaaaat ggtattcagg ctatattgag aagaaaattt
      601 aaaattaaga aacaacaaac ggacagtagc tcccaatata ttagaccaaa actttattgt
      661 cgatcaacca aataaagtat gggattactt atataaaaac caaagaagga tggctatatt
      721 tggcaacaat aatcgatcta tattcacgca tggtagttaa tgagcagttc aataaataaa
      781 caattggtta tggactcttt attaatggcc gttaacaagc gtaaacctgc caaaaatcta
      841 ctattacata gtgatcaagg ttcacaatat acttcgcaag gttatcaata tctcttatcc
      901 ataaaaaaca tagatgagtt ataaaggttg ttgttacgac aattctgttg tggaaagctt
      961 cttcagttct ctaaaaaggg agttaccaat agacacctct cgacactcta aacagcatat
     1021 caaaactgct atatttgaat atatagaaat tttttataac aaacaacgtc actattaact
     1081 atagtacttc ccaaagtaaa tgataaaggt ataataagct atttgagtta taatggcgta
     1141 tagtgtggat ttaagggaaa gggcagtatc gatgatagaa aaagggaagt caaaggttga
     1201 ggtggcagag cttttggaga taggaatagc aactctgtac cgctggttaa gaaagaaagc
     1261 cgctggtgaa agcctaaggc catcgaaaaa cggcagcttt attcgaaaaa tagacccaaa
     1321 aatactcgaa gaatatgtta aaaagaatcc agatcatacg ctggcagaga tgaaacaaaa
     1381 tcttggattt ggaataaatt caatttggta taggctgaaa cagctaagaa ttactttaaa
     1441 aaaaagttac actatatcaa gagcgcaatc aagaagatag gcagcgattt accgataaaa
     1501 tctcaaaaat agaccattcc agcatcttat atatagatga agcaggagtt gataataggt
     1561 tataccgaga gtatggacgt gctccaatag gaaagaaaat ttatgcagat attccaggaa
     1621 gaaaacgaga gagaatcagt ataagaggtt gtccggaaat aagtaaaagg ctataatatc
     1681 tgaaatttta gtacggggta aagatgagaa aaaagtatcc aacagatcta agcgaaaggg
```

```
     1741 aatgggcaag aatagaaaaa cacttcagag tatcatacaa gaaaggagga aggccgccaa
     1801 agtatagcaa aagagaaata ttagaagcaa ttttctatgt attgcgtaca gggtgtcaat
     1861 ggcggtattt accaaatgat tttccgctat ggaagactgt gtatgagcag ttcaggcaat
     1921 ggaagaagca gggaattttt gagaaaatga attatgaaat tacaaaatat agtagaagaa
     1981 aaataggaag gaatgagcag ccgagtgcct gtatagtaga tagtcaatct gtaaagacta
     2041 cagaaaaggg gggatcaaag gctatgatgg aagtaaaaaa gtaaagggta gaaaaaggca
     2101 tataattaca gacactcagg gttttatact aggttgttac gtaggcgctg ctaacgaaaa
     2161 tgatagagat ggtattaaaa tagcattaaa caatatgaga acaaaatata ctaaagttaa
     2221 aaaaatgtgg gctgacatgg gataccaagg aagaaattta aagaatcaca taaaggaaga
     2281 atatgacata gatattgaaa ttgttaaaag gcctccatgt agattttggg tgcacaaaga
     2341 tacgccacct gagctactac caacaagaga acaagggttt aaagtacagc caagaagatg
     2401 ggttgtagaa aggacttttg cttgggttaa taggaataga aggctatcga aggagtatga
     2461 tttactcaca acatccactg agaatttcat atacctagct atgagtaggg ttatgttaaa
     2521 gagggaatat gcttgaattt actagtttcc ggacaacct
//
LOCUS       JAQZAU010000034          30551 bp    dna     linear   ENV 17-MAR-2023
DEFINITION  MAG: Wolbachia pipientis isolate wEsol contig_28785, whole genome
            shotgun sequence.
ACCESSION   JAQZAU010000034 JAQZAU010000000
VERSION     JAQZAU010000034.1
DBLINK      BioProject: PRJNA929258
            BioSample: SAMN33142973
KEYWORDS    WGS; ENV; Metagenome Assembled Genome; MAG.
SOURCE      Wolbachia pipientis (insect metagenome)
  ORGANISM  Wolbachia pipientis
            Bacteria; Pseudomonadota; Alphaproteobacteria; Rickettsiales;
            Anaplasmataceae; Wolbachieae; Wolbachia.
REFERENCE   1  (bases 1 to 30551)
  AUTHORS   Fiutek,N., Couger,B.M., Roy,S., de la Torre,J.R. and Connor,E.F.
  TITLE     Genomic assessment of the contribution of the Wolbachia
            endosymbiont of Eurosta solidaginis to gall-induction
  JOURNAL   International Journal Molecular Sciences
REFERENCE   2  (bases 1 to 30551)
  AUTHORS   Fiutek,N., Couger,B.M., Roy,S., de la Torre,J.R. and Connor,E.F.
  TITLE     Direct Submission
  JOURNAL   Submitted (10-FEB-2023) Biology, San Francisco State University,
            1600 Holloway Ave, San Francisco, CA 94132, USA
COMMENT     ##Genome-Assembly-Data-START##
            Assembly Date          :: JUN-2019
            Assembly Method        :: FLYE v. 06-2019
            Genome Representation  :: Full
            Expected Final Version :: Yes
            Genome Coverage        :: 100.0x
            Sequencing Technology  :: Illumina HiSeq; PacBio Sequel
            ##Genome-Assembly-Data-END##
FEATURES             Location/Qualifiers
     source          1..30551
                     /organism="Wolbachia pipientis"
                     /mol_type="genomic DNA"
                     /submitter_seqid="contig_28785"
                     /isolate="wEsol"
                     /isolation_source="galls on Solidago altissima"
                     /host="Eurosta solidaginis MN-2018-EFC"
                     /db_xref="taxon:955"
                     /environmental_sample
                     /country="USA: Cowling Arboretum, Carleton College,
                     Northfield, MN"
                     /lat_lon="44.48 N 93.13 W"
                     /altitude="280 m"
                     /collection_date="2018-11"
```

```
                     /metagenome_source="insect metagenome"
                     /note="metagenomic"
     gene            complement(1704..1847)
                     /locus_tag="wEsol_00238"
     CDS             complement(1704..1847)
                     /locus_tag="wEsol_00238"
                     /codon_start=1
                     /transl_table=11
                     /product="hypothetical protein"
                     /translation="MSGKHADERHEQPATQVEGIFRRYKSQPIKRVIHDLNPICERVG
                     KLL"
     gene            complement(4313..5269)
                     /locus_tag="wEsol_00239"
     CDS             complement(4313..5269)
                     /locus_tag="wEsol_00239"
                     /codon_start=1
                     /transl_table=11
                     /product="hypothetical protein"
                     /pfam="Ank_4 Ank_2 Ank_5 Ank_2 Ank Ank_4 Pfam-B_10896
                     Ank_5 Ank Ank_3 Pfam-B_10896 Ank_5 Ank_4 Ank_2 Ank Ank_3
                     Ank_4 Ank_2 Ank Pfam-B_10896 Ank_2 Ank_5 Ank_4 Ank Ank_4
                     Ank_3 Pfam-B_1714 Ank_2 Pfam-B_10896 Ank_3 Ank Ank_4
                     Ank_5 Ank Ank_3"
                     /translation="MMDLVRYLVDKGADVYTQDKGGKTLLDIATDQKHNNIVEYLKRV
                     QLDKELLIAAQYGNLSKVEDLVSQRASLSAKYSDGRTLMHCAAYGGELNVVKYLVVGE
                     KSSLKIKDNSGRIPLQYAAYNGKLDIVKYFIDEEKIDVNIKDHGGWAPLYYASCGGYS
                     NVINYLGKIHNTDLISNLQGNSTSLEIPNSNNIEVQDELGRTPLHLAAEAGDLSKINL
                     LCDDYNANIEAQDNFGYTPIFFATQSGKLNVVSLLLYYGANINAQDMYGSTPLHCAAQ
                     EGNLDMIQLLLSRCARISSQPLRSINLLSEIGVTKLGGNLNR"
                     /besthit="qcoverage 67.6100628930818 , hcoverage
                     6.33844339622642 , similarity 94.0 , identity 91.6 ,
                     evalue 6.65e-51 , alnlength 215 , Bacteria ,
                     Proteobacteria , WP_208571528.1 ankyrin repeat
                     domain-containing protein [Wolbachia pipientis]"
     gene            complement(5238..6095)
                     /locus_tag="wEsol_00240"
     CDS             complement(5238..6095)
                     /locus_tag="wEsol_00240"
                     /codon_start=1
                     /transl_table=11
                     /product="hypothetical protein"
                     /pfam="Pfam-B_11762 Pfam-B_11762 Ank_2 Ank_4 Ank_5 Ank
                     Ank_3 Ank_2 Ank_4 Ank_5 Ank Ank_3 Pfam-B_1873 Ank_5
                     Pfam-B_10896 Ank Ank_3 Ank_4 Ank_5 Ank_3 Ank"
                     /translation="MNVAFNETLYAASNYFNYNDDRRMVFTWIPGGRVVRDLWTVKPD
                     GNSCSIMNVELNEYLYAADYAKYDNDRRRVFFWIPGGQVSQGMWMIEDCGSTLRKVRN
                     ADASSNLIVSRQVQLDYALLNATKIDNISEVTGLVNQGANVNTEDRDGNTPARNAVLK
                     GHFGIIKYLVEKGVSLKAEDHYCGPPTCNAAYSGHLNILKYLISKCVDINDTDKNGWT
                     PLHFAAWRGLLEVANFLIEKGADINAENIFGRNPIHIAAENNNKDIIDFFLSKGMSID
                     DGFSKVPRR"
                     /besthit="qcoverage 60 , hcoverage 4.47409733124019 ,
                     similarity 76.0 , identity 71.3 , evalue 1.47e-46 ,
                     alnlength 171 , Bacteria , Proteobacteria ,
                     WP_208571570.1 ankyrin repeat domain-containing protein
                     [Wolbachia pipientis]"
     gene            complement(6095..6532)
                     /locus_tag="wEsol_00241"
     CDS             complement(6095..6532)
                     /locus_tag="wEsol_00241"
                     /codon_start=1
```

/transl_table=11
/product="hypothetical protein"
/pfam="Pfam-B_11762"
/translation="MYGRVDTKETLSYVRSHHNAGQRISGYVAVFDAMQRNDDLNNSA
IFKLAYYVKEVMEMKNYSDVHQEDRSNLEKLKSRLPESVRNAVFSSKVCIKNVYQGEY
LYAANRCFDDYGDKRRTVFTWIPKDEKMISLGGRLNLMVTTFI"
/besthit="qcoverage 88.9655172413793 , hcoverage
3.27079107505071 , similarity 87.6 , identity 76.7 ,
evalue 7.66e-57 , alnlength 129 , Bacteria ,
Proteobacteria , WP_211908378.1 ankyrin repeat
domain-containing protein [Wolbachia endosymbiont of
Spodoptera picta]"
gene complement(6545..7009)
/locus_tag="wEsol_00242"
CDS complement(6545..7009)
/locus_tag="wEsol_00242"
/codon_start=1
/transl_table=11
/product="hypothetical protein"
/pfam="Ank_4 Ank_2 Ank Ank_5 Ank Ank_3"
/translation="MLKIKTSMVVTLCTRLLSKANLNIPKFLVDKGADIGAKDNDGRT
PLHVAAYSGDLDMVKFFLDKNASIEAKSSDPYKMMGVVEGVKNEIINQADTALNVKRW
AEFFVEKLRYSIKSVAKEKLKDGMLHNRYSSVNELANEIYKSDGKLFDDIIK"
/besthit="qcoverage 83.7662337662338 , hcoverage
3.37519623233909 , similarity 90.7 , identity 88.4 ,
evalue 5.90e-50 , alnlength 129 , Bacteria ,
Proteobacteria , WP_208571570.1 ankyrin repeat
domain-containing protein [Wolbachia pipientis]"
gene complement(6966..7157)
/locus_tag="wEsol_00243"
CDS complement(6966..7157)
/locus_tag="wEsol_00243"
/codon_start=1
/transl_table=11
/product="hypothetical protein"
/pfam="Ank_2 Ank_5 Ank_4 Ank_3 Ank"
/translation="MQSDISEAEDLTRKIETIDIEGKRLTPLYIAIQKGRLDIVEILF
DRKHFSVKDKDIYGCNPLH"
/besthit="qcoverage 92.0634920634921 , hcoverage
1.59165751920966 , similarity 89.7 , identity 72.4 ,
evalue 6.65e-17 , alnlength 58 , Bacteria ,
Proteobacteria , WP_141456901.1 ankyrin repeat
domain-containing protein [Wolbachia endosymbiont of
Carposina sasakii]"
gene complement(7244..8335)
/locus_tag="wEsol_00244"
CDS complement(7244..8335)
/locus_tag="wEsol_00244"
/codon_start=1
/transl_table=11
/product="hypothetical protein"
/translation="MSLSDTIEDIRYQKLRNSGSDTDREIIQNSEKKLKAFIQASILD
QELLDTYQIAKGIADDNNFDIPISQIEVTKNHMGIPAEKVIIAGIYSDQVIVDFSYNN
SDVTQKYRNNGRSYDYDHYMTLCNYYQDATVEGGEGQHQYIIKLPDTLNHEISSFPIR
LNLKIKSKAVPYQNIPYNIIDFAELNVTDVDSISIQEGKRSYINECYNQSISDLTEDS
LEIKDITVLGSEGTKWSLSIGLVDYFQSPEHQQIVLRMNNELYKIDSTNLRLEHVEMN
PNSFRYYQPEEQGLQIYHNQPISKNDIGLVDFRDKSVLGFDMEIANGSLVLSYGNSTI
AKVENWSNYQPAREMMFAFNDTMVLIQSA"
/besthit="qcoverage 99.1735537190083 , hcoverage
9.87925356750823 , similarity 90.8 , identity 83.1 ,

```
                evalue 1.28e-189 , alnlength 360 , Bacteria ,
                Proteobacteria , WP_141456901.1 ankyrin repeat
                domain-containing protein [Wolbachia endosymbiont of
                Carposina sasakii]"
gene            complement(8314..8634)
                /locus_tag="wEsol_00245"
CDS             complement(8314..8634)
                /locus_tag="wEsol_00245"
                /codon_start=1
                /transl_table=11
                /product="hypothetical protein"
                /translation="MHHRGLKQTIFIVNEDSDNARIISDLGSTGKMKGNIDVIRVQVA
                NITQWGVSKDTEKDSYSLDLLANDTQSIASSTTIDDFKNLVIQVNCQGITESVTIQCL
                CLIQ"
                /besthit="qcoverage 94.3396226415094 , hcoverage
                2.74423710208562 , similarity 92.0 , identity 81.0 ,
                evalue 1.11e-45 , alnlength 100 , Bacteria ,
                Proteobacteria , WP_141456901.1 ankyrin repeat
                domain-containing protein [Wolbachia endosymbiont of
                Carposina sasakii]"
gene            complement(8681..9001)
                /locus_tag="wEsol_00246"
CDS             complement(8681..9001)
                /locus_tag="wEsol_00246"
                /codon_start=1
                /transl_table=11
                /product="hypothetical protein"
                /translation="MHIPEGSNIRYIGSKNNETIFIINNYMSGTLKGGAGKENTLVMN
                VKANNIAANLHSGTIRYGNSNNIRLVNTYNYVSNSDNKQNITTHCETRLINVKKRESL
                AEFI"
                /besthit="qcoverage 94.3396226415094 , hcoverage
                2.74423710208562 , similarity 97.0 , identity 97.0 ,
                evalue 5.84e-53 , alnlength 100 , Bacteria ,
                Proteobacteria , WP_141456901.1 ankyrin repeat
                domain-containing protein [Wolbachia endosymbiont of
                Carposina sasakii]"
gene            complement(9059..9607)
                /locus_tag="wEsol_00247"
CDS             complement(9059..9607)
                /locus_tag="wEsol_00247"
                /codon_start=1
                /transl_table=11
                /product="hypothetical protein"
                /translation="MPSPHGPGCATQMKWTCDLEKKYKDISFDKINIASSVKRDLSSI
                DISKILTMTPYTLVMSEGWERFICGIKNSHQCYQEIEEKKVYVVNTDAKHIPHLTKYE
                YENLGLRVVDAPLTNPIQKSQCSKKINTKHTGDDAFAPCNSGKIYRNCQESFTLSGEP
                FIFTNPKRKDPGNTKKQTFPRG"
                /besthit="qcoverage 100 , hcoverage 5.36556603773585 ,
                similarity 98.4 , identity 97.3 , evalue 2.50e-116 ,
                alnlength 182 , Bacteria , Proteobacteria ,
                WP_208571528.1 ankyrin repeat domain-containing protein
                [Wolbachia pipientis]"
gene            complement(9737..10276)
                /locus_tag="wEsol_00248"
CDS             complement(9737..10276)
                /locus_tag="wEsol_00248"
                /codon_start=1
                /transl_table=11
                /product="hypothetical protein"
                /translation="MLGVSISPSSKVGKLIGKIPGPARQAFVKVISNPVVQSITFATI
```

```
                     AYQFGYSINEIAQGNNHPLNYYWTASSGIKLASMSIRPISAGVSFTIKSVSATTKILR
                     GLSAAGKVLGRAAVVTMVADVLITMGVKIHERMEYTKAIAEQVTLLPGGEQAEVFFAG
                     VIKFFTGRDVEKNMKIQLE"
                     /besthit="qcoverage 94.413407821229 , hcoverage
                     5.52829571475303 , similarity 98.8 , identity 97.6 ,
                     evalue 5.35e-95 , alnlength 169 , Bacteria ,
                     Proteobacteria , WP_065095480.1 ankyrin repeat
                     domain-containing protein [Wolbachia endosymbiont of
                     Nomada leucophthalma]"
     gene            complement(10251..10394)
                     /locus_tag="wEsol_00249"
     CDS             complement(10251..10394)
                     /locus_tag="wEsol_00249"
                     /codon_start=1
                     /transl_table=11
                     /product="hypothetical protein"
                     /translation="MMAIYDLLDSIGDTATLGRHNNDALKQVFGINGILDAMDDVRCK
                     YFS"
                     /besthit="qcoverage 89.3617021276596 , hcoverage
                     1.06571936056838 , similarity 100 , identity 97.6 ,
                     evalue 1.28e-17 , alnlength 42 , Bacteria ,
                     Proteobacteria , WP_063631194.1 ankyrin repeat
                     domain-containing protein [Wolbachia endosymbiont of
                     Laodelphax striatellus]"
     gene            complement(10889..11644)
                     /locus_tag="wEsol_00250"
     CDS             complement(10889..11644)
                     /locus_tag="wEsol_00250"
                     /codon_start=1
                     /transl_table=11
                     /product="hypothetical protein"
                     /translation="MFSFRDFINEEANFQGVLHGAFSYYSDLKLEESPRALVLTEFQT
                     GRGKRIDMLVNGIKFAEGASNAKEYDPVALELKGPREGKTADALVKEANDQINKEYVK
                     GVTYKTLTDGRKVAFMGVVFDKGASNADSLILPSKNEFTPVKVVHSSIFSFSQCATRK
                     RRDIGIPCIDSRDEEKVTKEEKKQRVKELFDIDNAKDLTNEIVDIGREQVFDMIGNAD
                     RTDVYVKVKDNNGNDKKLIIGDIIEMKNAERYL"
                     /besthit="qcoverage 98.00796812749 , hcoverage
                     6.75082327113063 , similarity 92.7 , identity 86.6 ,
                     evalue 1.71e-129 , alnlength 246 , Bacteria ,
                     Proteobacteria , WP_141456901.1 ankyrin repeat
                     domain-containing protein [Wolbachia endosymbiont of
                     Carposina sasakii]"
     gene            complement(12094..12918)
                     /locus_tag="wEsol_00251"
     CDS             complement(12094..12918)
                     /locus_tag="wEsol_00251"
                     /codon_start=1
                     /transl_table=11
                     /product="hypothetical protein"
                     /pfam="DUF1703"
                     /translation="MLKHKIKKYLTLLLVIIFYEIKKEEEIWKDENDNTKSKPEDYIA
                     ELTNSDASKVEESSKKVIDRITKVHSKYKGSLTYADKAREAAHHGFIAGALVNFRYRH
                     NLRVYLEQFAGRGYADIVLVPRGKDRSLNAVPIIIELKAATKEELKELEKGGKVKDGS
                     QTTSAIALEQAKHYAKSFQPNTMRVLTISDNVLCEGLNLDSTEGEKFSMHISPREDRE
                     IALPTMQALLKEASDWDGRQDTVTNVKEKSSSHWSVFITHFLVRQRKEVITLVDFS"
                     /besthit="qcoverage 77.007299270073 , hcoverage
                     25.9852216748768 , similarity 87.2 , identity 75.4 ,
                     evalue 1.93e-96 , alnlength 211 , Bacteria ,
                     Proteobacteria , WP_155968920.1 hypothetical protein
                     [Wolbachia pipientis]"
```

```
gene            complement(12912..13046)
                /locus_tag="wEsol_00252"
CDS             complement(12912..13046)
                /locus_tag="wEsol_00252"
                /codon_start=1
                /transl_table=11
                /product="hypothetical protein"
                /translation="MLKENLHPDTQQKIGTARGIKDRIKGRVKSKLVQINKNEENIFC
                "
                /besthit="qcoverage 100 , hcoverage 1.20680197476687 ,
                similarity 84.1 , identity 72.7 , evalue 5.32e-09 ,
                alnlength 44 , Eukaryota , Arthropoda , KAG8290545.1
                hypothetical protein J6590_079680 [Homalodisca
                vitripennis]"
gene            complement(13110..13472)
                /locus_tag="wEsol_00253"
CDS             complement(13110..13472)
                /locus_tag="wEsol_00253"
                /codon_start=1
                /transl_table=11
                /product="hypothetical protein"
                /translation="MLSQEKPQDFELIELLIRETAGVSQPGPSTSTQQKSDFESFKAR
                FQSYIDQVPSYLHSVGKEGFFSHFFLGSFSILPDTQIAERLGDKKVYFRFATAKTLKV
                VVVKNSQIKSDVDAIEGI"
                /besthit="qcoverage 97.5 , hcoverage 3.43713278495887 ,
                similarity 89.7 , identity 88.9 , evalue 5.11e-57 ,
                alnlength 117 , Bacteria , Proteobacteria , NGZ20114.1
                hypothetical protein [Wolbachia pipientis]"
gene            complement(13501..14022)
                /locus_tag="wEsol_00254"
CDS             complement(13501..14022)
                /locus_tag="wEsol_00254"
                /codon_start=1
                /transl_table=11
                /product="hypothetical protein"
                /translation="MFNFDQFQKLFDCLKPSDVVLGSYYCTWLRNIKLGGCSGQYLNV
                GAKLLMHMLMKDGFDSHRTLALNGEVMNNSTFQGSLLVPLVEKGCMEQVWAILDKTNS
                KQIEEFMSSMRAGYICSILEQGDHASLNKFLSYSGSVDVDLDQKSIPGPSGDLSDVKL
                NKMHDKSHIRLGE"
                /besthit="qcoverage 100 , hcoverage 39.5881006864989 ,
                similarity 90.8 , identity 79.2 , evalue 5.59e-91 ,
                alnlength 173 , Bacteria , Proteobacteria , OAM06474.1
                hypothetical protein TV41_04045 [Wolbachia endosymbiont
                of Dactylopius coccus]"
gene            complement(14037..14177)
                /locus_tag="wEsol_00255"
CDS             complement(14037..14177)
                /locus_tag="wEsol_00255"
                /codon_start=1
                /transl_table=11
                /product="hypothetical protein"
                /translation="MKIAVHAAHDNGYPDVFEFCLSQINPDRYPELLKRDLEKNGYYG
                SL"
                /besthit="qcoverage 100 , hcoverage 10.6728538283063 ,
                similarity 97.8 , identity 93.5 , evalue 2.46e-22 ,
                alnlength 46 , Bacteria , Proteobacteria , WP_172758421.1
                hypothetical protein [Wolbachia endosymbiont of
                Diaphorina citri]"
gene            complement(14233..14706)
                /locus_tag="wEsol_00256"
```

```
CDS             complement(14233..14706)
                /locus_tag="wEsol_00256"
                /codon_start=1
                /transl_table=11
                /product="hypothetical protein"
                /translation="METINTGNLPFFLDILSDEECWEKTDTIHGIKLNRRIVAKKMIE
                PKSWKGTNNPLDDFDRYQIACWCCLEEDIITLFEHFKREDEIKDGDSDALKKLVKSVS
                GSWYTDAMMQFWSYFVSGYIAELDLKDQHPYVLGLHRAAISMRRKRVRSSRVLLE"
                /besthit="qcoverage 94.9044585987261 , hcoverage
                34.5707656612529 , similarity 97.3 , identity 88.6 ,
                evalue 9.40e-94 , alnlength 149 , Bacteria ,
                Proteobacteria , WP_168456002.1 hypothetical protein
                [Wolbachia endosymbiont of Ctenocephalides felis wCfeJ]"
gene            15485..16027
                /locus_tag="wEsol_00257"
CDS             15485..16027
                /locus_tag="wEsol_00257"
                /codon_start=1
                /transl_table=11
                /product="hypothetical protein"
                /pfam="Pfam-B_5326 LRR_6 LRR_4 LRR_6 LRR_4 LRR_6 LRR_4
                LRR_6 LRR_4"
                /translation="MDFKQYVKGDTLLLSSDAINISDLVSFLERNQSITRLILSFSKI
                GNEGAKILASGNLPNLTSLHLFCNNISAEGAEALAKGNLLNLASLHLLFNKIGDEGVK
                ALANGNLTKLTSLRLVANKIGNEGAKALANSNLPNLKLLYLGNHLDTKSVKALLATNN
                VRNLLSPELAITLVIKVQKL"
                /besthit="qcoverage 36.1111111111111 , hcoverage
                39.1566265060241 , similarity 72.3 , identity 60.0 ,
                evalue 1.12e-11 , alnlength 65 , Bacteria ,
                Proteobacteria , WP_226080053.1 hypothetical protein
                [Wolbachia endosymbiont of Tribolium confusum]"
gene            16102..16884
                /locus_tag="wEsol_00258"
CDS             16102..16884
                /locus_tag="wEsol_00258"
                /codon_start=1
                /transl_table=11
                /pfam="LRR_8 LRR_6 LRR_4"
                /product="hypothetical protein"
                /translation="MAKDNLPNLTSLGLSCNNIGNKGAEALANVNLTKFTKLDLFGNN
                IDFEHIEVLARSQCSGIKLGDRDQKVFYDFLLKKCSNNQVVDEESIKRMIFSVARSNH
                SGDLVRYILTQPDKYPFLINSKDEQGHTLSHFYNHSPEIQEFLFKHGLVSEKDSTLRN
                ITNDKQSVHAGLAVKKTKFITKNLVESIKASEGQLEQAVNSYVENIGLLKQYQNDPIR
                LRLLSLTNNEKKVRWKKHYVRLILRQVMKNLLRLLLIKPSKH"
                /besthit="qcoverage 63.8461538461538 , hcoverage
                34.8008385744235 , similarity 72.3 , identity 55.4 ,
                evalue 2.35e-47 , alnlength 166 , Bacteria ,
                Proteobacteria , WP_175937404.1 leucine-rich repeat
                domain-containing protein [Wolbachia endosymbiont of
                Madathamugadia hiepei]"
gene            16921..17295
                /locus_tag="wEsol_00259"
CDS             16921..17295
                /locus_tag="wEsol_00259"
                /codon_start=1
                /transl_table=11
                /product="hypothetical protein"
                /translation="MTKGISTNRMQYDYTRGSERFITIPESIGYIKLLIDDFSVPLKE
                KKELLVTLMEQNPDLVKNKLSKVKEKLGNSVILDKEQFNKTELHGLLDGIDDGKKVDE
                LFKEISCLNIEEVWREQKNLFS"
```

```
                /besthit="qcoverage 95.1612903225807 , hcoverage
                15.7965194109772 , similarity 92.4 , identity 84.7 ,
                evalue 3.47e-54 , alnlength 118 , Bacteria ,
                Proteobacteria , WP_153295431.1 hypothetical protein
                [Wolbachia endosymbiont of Armadillidium vulgare]"
gene            17244..17627
                /locus_tag="wEsol_00260"
CDS             17244..17627
                /locus_tag="wEsol_00260"
                /codon_start=1
                /transl_table=11
                /product="hypothetical protein"
                /translation="MFEYRRGLERAKEFILLKQIYIAATTYGENSSACIQGTWSQIIG
                SINEISSEVVAQYDRYLEEEQRREMQKDAITEENINPFLEGLANKLIQHVELNPELKE
                TLEDFVVCTVDIDKPEEITFNSKKF"
                /besthit="qcoverage 90.5511811023622 , hcoverage
                25.5555555555556 , similarity 91.3 , identity 79.1 ,
                evalue 1.57e-53 , alnlength 115 , Bacteria ,
                Proteobacteria , WP_191111036.1 hypothetical protein
                [Candidatus Wolbachia massiliensis]"
gene            17747..18100
                /locus_tag="wEsol_00261"
CDS             17747..18100
                /locus_tag="wEsol_00261"
                /codon_start=1
                /transl_table=11
                /product="hypothetical protein"
                /translation="MQSFVQPGQEPFNLQENITGENVPSIPGKLQPSKPSEKRSKLPI
                VTASALAIAGVVSGIAIAVYLEMLAVGIAVGACCLVAAAIIYYCNRPSSSLGNSNVQG
                SCKEVQEPHSEGKKK"
                /besthit="qcoverage 69.2307692307692 , hcoverage
                94.1860465116279 , similarity 84.0 , identity 76.5 ,
                evalue 6.41e-29 , alnlength 81 , Bacteria ,
                Proteobacteria , MBA8758310.1 hypothetical protein
                [Wolbachia pipientis]"
gene            complement(18181..19170)
                /locus_tag="wEsol_00262"
CDS             complement(18181..19170)
                /locus_tag="wEsol_00262"
                /codon_start=1
                /transl_table=11
                /pfam="HTH_31 HTH_19 Pfam-B_3762 HTH_3 Pfam-B_12708
                HTH_31 HTH_19 HTH_3"
                /fullproduct="qcoverage 93.0091185410334 , hcoverage
                98.7096774193548 , similarity 95.8 , identity 90.5 ,
                evalue 2.72e-186 , alnlength 306 , Eukaryota , Arthropoda
                , GFS56378.1 putative transcriptional regulator [Nephila
                pilipes]"
                /translation="MTNDTKLTDYEVGKKLKHEREKKRYTQAGLGSKVGVLEKQMSRY
                ERGENPISIIIFCMIVVELMIDMAKLLPESTISEKIEGKTLNLVRECKKFRNQELCKM
                ASALNKSIQMGEEEGKKAGKIKVAKNLLKAGISLDIILQTTGLSKDELEKHNKTEIVN
                FIAYKTGENMKDWRLARGRSEEDLGNEVGLTGGQIRRYEQGTHIMTIGKLCNTADELS
                THAKTLLAKISELIEKGNVEIERSNEDREGENGVLDLVREYNKIDDQEIRDKFYSLIM
                SVSKRIQIDKEKGKKIGRTEMAKNLLEVGVPINVVHQATGLKNLKASDMLEGP"
                /product="putative transcriptional regulator"
                /besthit="qcoverage 93.0091185410334 , hcoverage
                98.7096774193548 , similarity 95.8 , identity 90.5 ,
                evalue 2.72e-186 , alnlength 306 , Eukaryota , Arthropoda
                , GFS56378.1 putative transcriptional regulator [Nephila
                pilipes]"
```

```
     gene            complement(19242..19541)
                     /locus_tag="wEsol_00263"
     CDS             complement(19242..19541)
                     /locus_tag="wEsol_00263"
                     /codon_start=1
                     /transl_table=11
                     /product="hypothetical protein"
                     /pfam="RadC"
                     /translation="MEQGVRIEASSMVVSHNYPEREAPTEGDISIVRKLVIACPSVGI
                     DLVDYKTVTEYGCFSFNGLIREGCKILWKDEVVWKDDKEYKNAIREDNYKVEKLH"
                     /besthit="qcoverage 61.6161616161616 , hcoverage
                     26.9911504424779 , similarity 73.8 , identity 45.9 ,
                     evalue 8.45e-07 , alnlength 61 , Bacteria ,
                     Proteobacteria , WP_168464812.1 DNA repair protein RadC
                     [Wolbachia endosymbiont of Ctenocephalides felis wCfeT]"
     gene            complement(19526..19663)
                     /locus_tag="wEsol_00264"
     CDS             complement(19526..19663)
                     /locus_tag="wEsol_00264"
                     /codon_start=1
                     /transl_table=11
                     /product="hypothetical protein"
                     /translation="MEQSDKEHLRVFYMDEKHYLLVEEVYDVFKIDKKRYIRESLWSK
                     G"
     gene            complement(22340..22720)
                     /locus_tag="wEsol_00265"
     CDS             complement(22340..22720)
                     /locus_tag="wEsol_00265"
                     /codon_start=1
                     /transl_table=11
                     /product="hypothetical protein"
                     /pfam="RadC"
                     /translation="MTINKKGIQVVYMDAECKTITENTYGYECIETVQSVMEQILMKE
                     AITSIKITHYLTTSEPLKDDMEIAKDLVLILPNIGVELKDYIMVTQSGYVSLIEQGLT
                     KQVKKVNFGCNRTLHTQLRAMVTT"
     gene            complement(22775..23728)
                     /locus_tag="wEsol_00266"
     CDS             complement(22775..23728)
                     /locus_tag="wEsol_00266"
                     /codon_start=1
                     /transl_table=11
                     /pfam="Pfam-B_12980 Ank_2 Ank_4 Ank_5 Ank Ank_3 Ank_4
                     Ank_5 NMT1_2 Ank Ank_4 Ank_2 Pfam-B_10896 Ank_5
                     Pfam-B_11730 Pfam-B_1873 Ank Ank_3 DUF3447 Ank_4 Ank_4
                     Pfam-B_1873 DUF3447 Ank_2 Shigella_OspC Ank_4 Ank_5 Ank_3
                     Ank Pfam-B_10896 Ank_5 Ank Ank_3 Ank_4 Ank_5 Ank_4 Ank_2
                     Ank Ank_3"
                     /fullproduct="qcoverage 100 , hcoverage 111.619718309859
                     , similarity 80.1 , identity 78.9 , evalue 1.14e-74 ,
                     alnlength 317 , Eukaryota , Arthropoda , GFQ64082.1
                     ANK_REP_REGION domain-containing protein [Trichonephila
                     clavata]"
                     /translation="MCNIIEKYLVDKKDVNVKNDKGNTPLHLAVEEGNLEAVKHLIEK
                     GADSNAIDQYRCTPLCIAAKKGNLYLTAYILIEGADLNAQDEGSWTPLYYAVYSGNID
                     IVRFLINQGANYNITDNESTPVYYAFQYGHVRIVKYFVEEKSIDPMAPINEYGSTLFG
                     EAIVGGDLDTIEYLMSRKDVTYDCNDLLEIAILNGHLDVVEYLVEEKGVDVNFVGENG
                     WTPLLNAVERDNVEVVEYLIEKGAYVNATTEDGTIGLHIAAEHCNFYMVKCLVINGAN
                     INARGKRDNTPLYLATQDSEYQDKDIKEDVKEYLISRGAHI"
                     /product="ANK_REP_REGION domain-containing protein"
                     /besthit="qcoverage 100 , hcoverage 111.619718309859 ,
```

similarity 80.1 , identity 78.9 , evalue 1.14e-74 ,
alnlength 317 , Eukaryota , Arthropoda , GFQ64082.1
ANK_REP_REGION domain-containing protein [Trichonephila
clavata]"

```
gene            complement(23890..24114)
                /locus_tag="wEsol_00267"
CDS             complement(23890..24114)
                /locus_tag="wEsol_00267"
                /codon_start=1
                /transl_table=11
                /fullproduct="qcoverage 105.405405405405 , hcoverage
                98.7341772151899 , similarity 87.2 , identity 78.2 ,
                evalue 2.28e-28 , alnlength 78 , Eukaryota , Arthropoda ,
                GFQ64080.1 ANK_REP_REGION domain-containing protein
                [Trichonephila clavata]"
                /translation="MSNNNNNRESVLARLLSKTPEQLKLYCKLLDNEKRQDLYQKVLK
                EMRKNQEINQLKKLNEVAAVIEEAIEEGRK"
                /product="ANK_REP_REGION domain-containing protein"
                /besthit="qcoverage 105.405405405405 , hcoverage
                98.7341772151899 , similarity 87.2 , identity 78.2 ,
                evalue 2.28e-28 , alnlength 78 , Eukaryota , Arthropoda ,
                GFQ64080.1 ANK_REP_REGION domain-containing protein
                [Trichonephila clavata]"
gene            complement(24229..24471)
                /locus_tag="wEsol_00268"
CDS             complement(24229..24471)
                /locus_tag="wEsol_00268"
                /codon_start=1
                /transl_table=11
                /product="hypothetical protein"
                /translation="MGVALLVSSVVAYILKMYAIAVIGGIVGLACVSYALYNALKPGT
                KLEKVEDVERIDEKPLPSLCRKCSIEANIFTIINNK"
                /besthit="qcoverage 72.5 , hcoverage 42.3357664233577 ,
                similarity 93.1 , identity 84.5 , evalue 2.34e-19 ,
                alnlength 58 , Eukaryota , Arthropoda , GFT52245.1
                uncharacterized protein TNCV_4275591 [Trichonephila
                clavipes]"
gene            complement(24453..24767)
                /locus_tag="wEsol_00269"
CDS             complement(24453..24767)
                /locus_tag="wEsol_00269"
                /codon_start=1
                /transl_table=11
                /product="hypothetical protein"
                /translation="MPANACEIVTEVCSFDVEHKPVIDKATTDTGLCEDVNKSSEGLQ
                PICENSEKVQQTSVKTDEQESNASNDLSPKPRFDDNATDKREKPLTTQAVISGSVWVW
                RY"
                /besthit="qcoverage 96.1538461538462 , hcoverage
                40.3225806451613 , similarity 58.0 , identity 38.0 ,
                evalue 4.31e-06 , alnlength 100 , Bacteria ,
                Proteobacteria , WP_174134220.1 hypothetical protein
                [Wolbachia endosymbiont of Atemnus politus]"
gene            complement(24791..25285)
                /locus_tag="wEsol_00270"
CDS             complement(24791..25285)
                /locus_tag="wEsol_00270"
                /codon_start=1
                /transl_table=11
                /product="hypothetical protein"
                /pfam="PAZ_siRNAbind PAZ_siRNAbind PAZ_siRNAbind"
```

/translation="MPAVKDLLEEAKKQKLLKEVLNERNDYKTFRWSEETHTILTDAI
SRRDNDIMRVVLKISEENGILKDILKKRIIIKHSDGEEMIYTPFTYAEECENSEAVEE
ILKFSRELGDVLNKRTTIEQQAPFLTVVTETATNASTTTAPCDEDSLSLMQQQLQNIK
ILLL"
/besthit="qcoverage 61.5853658536585 , hcoverage
34.1216216216216 , similarity 79.2 , identity 74.3 ,
evalue 9.05e-35 , alnlength 101 , Eukaryota , Arthropoda
, GFQ79632.1 uncharacterized protein TNCT_539301
[Trichonephila clavata]"
gene complement(26741..27127)
/locus_tag="wEsol_00271"
CDS complement(26741..27127)
/locus_tag="wEsol_00271"
/codon_start=1
/transl_table=11
/fullproduct="qcoverage 99.21875 , hcoverage
91.3669064748201 , similarity 99.2 , identity 98.4 ,
evalue 3.77e-85 , alnlength 127 , Bacteria ,
Proteobacteria , WP_182183014.1 nucleoside deaminase
[Wolbachia pipientis]"
/cog="CumB COG0590 152 Cytosine/adenosine deaminases"
/pfam="dCMP_cyt_deam_1 APOBEC_N MafB19-deam"
/tigr="TIGR00326 eubact_ribD 343 riboflavin biosynthesis
protein RibD"
/product="nucleoside deaminase"
/translation="MELAIEQAKLAQKNDEVPICAVIISGSNVVSSAHNISNDPTAHA
EMLAIKQACELLSTSTLYDSDIYVTLEPCPMCAQAISFARIKRLYFGAYNPKGGGIEN
GAKIFQFCSHIPEVYGGILETECSFC"
/besthit="qcoverage 99.21875 , hcoverage 91.3669064748201
, similarity 99.2 , identity 98.4 , evalue 3.77e-85 ,
alnlength 127 , Bacteria , Proteobacteria ,
WP_182183014.1 nucleoside deaminase [Wolbachia
pipientis]"
gene complement(27170..28774)
/locus_tag="wEsol_00272"
CDS complement(27170..28774)
/locus_tag="wEsol_00272"
/codon_start=1
/transl_table=11
/fullproduct="qcoverage 100 , hcoverage 100 , similarity
98.7 , identity 97.6 , evalue 0.0 , alnlength 534 ,
Bacteria , Proteobacteria , MBV2146595.1 CTP synthase
[Wolbachia endosymbiont of Pissodes strobi]"
/cog="PyrG COG0504 533 CTP synthase (UTP-ammonia lyase)"
/pfam="CTP_synth_N GATase Peptidase_C26"
/tigr="TIGR00337 PyrG 526 CTP synthase"
/product="CTP synthase"
/translation="MKEAKFIFVTGGVVSSLGKGLVASSVGALLQAYGFKIRIRKLDP
YLNIDPGTMNPTQHGEVFVTEDGGETDLDLGHYKRFTGIKATKDDNITTGKIYHELLK
KERRGDYLGKTVQVIPHVTALIKLFIFNSTEGLDFIICEIGATVGEIESQPFLEAIRQ
VNYTLGKQRVILIHLTLIPYLTAAQELKTKPTQHSVRELNSAGLQPDIILCRSEKEIF
DNQREKIAKLCNVSLSSVIPAPDVSHIYELPMLYSQCGLDTQILEHFHLSEPKPSLIE
WDQIVHSTRHPTQEVTVSIVGKYTEFPDAYKSLVEALNNGAISNKVKVKINWVNSREK
EEKPINEKLIGEKLQNSHAILVPGGFGHDGVESKILAINYARTNNIPFFGICLGMQLA
IIEFARNVVKLEDAHSEEFHNCKHPIVKLAGDQDDDLGGTMRLGAYKCNINASSKMMD
AYSNTTISERHRHRYIINSDYKDDLEKNGLLCSGISEDGTCIEAVELESHPWFIGVQF
HPEFQSKPFSPHPLFISFIKAAVNKI"
/besthit="qcoverage 100 , hcoverage 100 , similarity 98.7
, identity 97.6 , evalue 0.0 , alnlength 534 , Bacteria ,
Proteobacteria , MBV2146595.1 CTP synthase [Wolbachia

```
                     endosymbiont of Pissodes strobi]"
     gene            complement(28774..29139)
                     /locus_tag="wEsol_00273"
     CDS             complement(28774..29139)
                     /locus_tag="wEsol_00273"
                     /codon_start=1
                     /transl_table=11
                     /fullproduct="qcoverage 86.7768595041322 , hcoverage
                     99.0566037735849 , similarity 96.2 , identity 96.2 ,
                     evalue 3.25e-50 , alnlength 105 , Bacteria ,
                     Proteobacteria , WP_010962615.1 MULTISPECIES: preprotein
                     translocase subunit SecG [Wolbachia]"
                     /cog="SecG COG1314 86 Preprotein translocase subunit
                     SecG"
                     /pfam="SecG"
                     /product="preprotein
                     translocase subunit SecG"
                     /translation="MYFMLIKFLFSSLSIISVAVLSIFQVILVVVLVILVLLQPPGSS
                     SLSGFSNTQQGVNSMIPVKSSENPLSRITAIVDGLFIINTLLLSGLYSKDVHKKSIAE
                     KIILEKKQESEPTSVPFEN"
                     /besthit="qcoverage 86.7768595041322 , hcoverage
                     99.0566037735849 , similarity 96.2 , identity 96.2 ,
                     evalue 3.25e-50 , alnlength 105 , Bacteria ,
                     Proteobacteria , WP_010962615.1 MULTISPECIES: preprotein
                     translocase subunit SecG [Wolbachia]"
     gene            29239..29646
                     /locus_tag="wEsol_00274"
     CDS             29239..29646
                     /locus_tag="wEsol_00274"
                     /codon_start=1
                     /transl_table=11
                     /product="hypothetical protein"
                     /pfam="Hexapep Pfam-B_7219 Pfam-B_411 Hexapep Hexapep
                     Hexapep"
                     /tigr="TIGR02287 PaaY 192 phenylacetic acid degradation
                     protein PaaY"
                     /translation="MYHILKYKDYEPKIGERAFIAGGSHIIGKVEIGKDASIWFNCVV
                     RGDVGSIKIGDGTNIQDGTVIHVDRNPGGDTTIGNMVTVGHFCMLHACMVHDKAFIGM
                     GSTVMDHAVVEPEAMVAAGSLVTHGKVIKSGEI"
                     /cog="PaaY COG0663 176 Carbonic
                     anhydrases/acetyltransferases, isoleucine patch
                     superfamily"
                     /besthit="qcoverage 100 , hcoverage 78.9473684210526 ,
                     similarity 98.5 , identity 98.5 , evalue 1.01e-91 ,
                     alnlength 135 , Bacteria , Proteobacteria , MBV2146597.1
                     gamma carbonic anhydrase family protein [Wolbachia
                     endosymbiont of Pissodes strobi]"
     gene            complement(30043..>30551)
                     /locus_tag="wEsol_00275"
     CDS             complement(30043..>30551)
                     /locus_tag="wEsol_00275"
                     /codon_start=1
                     /transl_table=11
                     /product="hypothetical protein"
                     /pfam="tRNA-synt_1b MazG"
                     /tigr="TIGR00233 trpS 328 tryptophan--tRNA ligase"
                     /translation="NNHYKLDYFIVPEILTLDCTSRIMSLRDSTSKMSKSDPSEYSCI
                     NLDDTDDLIVKKIEKAKTDSIIGFDFATLECRPEISNLINIYSVLSNVNAEKVCEEVN
                     KHDMKHFKKELADLIISVISPIRERMNDLLKDQFHLREILKKGTKKAAEIATHNIKEM
                     KDIIGFAQ"
```

```
                     /cog="TrpS COG0180 314 Tryptophanyl-tRNA synthetase"
                     /besthit="qcoverage 100 , hcoverage 50.6024096385542 ,
                     similarity 99.4 , identity 98.2 , evalue 3.66e-109 ,
                     alnlength 168 , Bacteria , Proteobacteria , MBV2146598.1
                     tryptophan--tRNA ligase [Wolbachia endosymbiont of
                     Pissodes strobi]"
ORIGIN
        1 tatattccaa ttctacaaac tttccaggtg tttcttttca gcgtctctat catcacctga
       61 ttatttgtga gcgctatagt aatctcctcc tcttactaag ccttactgct gattcttaat
      121 atagcaataa ctcatctatt tttatcactc tactccagag tgaactcaat aatagcgttc
      181 atcacgtgct tatagatcaa caggctgctc gtcatattaa taccctgtca acatatatac
      241 tagacacaaa acgtttctaa acttatattc tttttctatg ataattttcc tcatatcctg
      301 ccgtaatatt tttccttaat tctctcagat attccggact atgtctgcca tattcgtctc
      361 taattcctta atttaaaaat tcaactctct atattcacct gactcgtgaa ggatcatcag
      421 caaatgtttc actaccactc tctatcgttg gcattgatcg cctgactatt atattgcact
      481 ttattctcta tcactcatct caaatcatac agctcacgca tcagattagc actgaacgct
      541 aaataagtag ttcttatgct ttcttcagga gcatagatgc agatatctaa cttccgctta
      601 atgtggcttt tttaatgacc actcgtttca caagcatttt ctactaagct acttactata
      661 tcaatagttt cttctatcgc cctaacagcc atccgttact caactcttta gctgatctat
      721 atccgcctct atgaatttct aggtgtattt gttaacttca ttatcacctc gctataaaga
      781 ctttgtttga tcctcgctac taatactgca cagcaatgct cctttaggtt gcttggaagt
      841 tttagatcaa gttgtactaa catacgcgcg tgtgtttgtg ccaaaatgct acacgcttta
      901 atcattatag cagattttaa gtgaaagtaa actgagatag cctgcatatc tccaatatat
      961 aggtcagtct actcgagact gctataatcc atggcatcca tttcactagc cttttaagat
     1021 ctactttaca gatagggctt gatacaaaaa gcggacgtcg tctaacgtag cagaatgcct
     1081 tctatactct aagatgcatc tcatgacagc cgttttctaa cgcattacaa aaaactaaaa
     1141 aaacttgaac attgcttcag aattgcgagt ccataatcta taatgcactt tctactcggt
     1201 tagttactgt tattcggctc tgtcttccta agtttctcga tccgcgaatg agatatgatg
     1261 tcgtcgcctt ttcgcctcgt ttatctagct agggtcgtcg atgaactgtc gttcagccta
     1321 tttattctag cagctttcgc gtaggactga tcctcttgtg gtgtctcaca ataggcctac
     1381 aatccataat tccatgccat aaacgttcca cgctcactcg cctcgattca aaaccgcagc
     1441 actccgcctc tacgctattc ctagcagtat tacgttacca aagttctaat tataacatat
     1501 tctactgagt gatcgccttt ccggtttgta tacctattct gtcgtctgca tatagaccca
     1561 gcgttttgtg atatccatcg ctactccact tcttccaccc aaacccttac tttgccttcc
     1621 tggctcgcat taagttgtac tgtcttacct tcttttccac ccaatgttgt tacgtattga
     1681 actttcgacc agagttccct atcctaaagt agtttaccca ccctctcaca tatcggattg
     1741 aggtcatgta tcactctttt tatcggttgc gatttatagc gccgaaatat tccttcaact
     1801 tgcgtagcag gttgttcgtg tctttcatct gcgtgttttc ctgacattcc actttccttg
     1861 tcttgtgatc tcttgaaaat caaacctagg taagctaaat gttctccttt ctttaggtga
     1921 tgacttttgt cttttcttca tttacgctca cttctagttt tgctaattct tctccaacct
     1981 tctgtatact gctctttcta gccattccgt ttagggtgtt cactaatcag tatcactaaa
     2041 tcgtctgccc atctggcata ttccatacgt tggtactttc cttctttggt cacctctttt
     2101 gctttctcca atattttatc tacatcattt aggtatatat tgcttagtag tggcggaaag
     2161 tggtgatcct tgtgctattc ctcgttttcc tcctgtttga ggattagctt aatcatgcgc
     2221 atgtttttctt tgtcatttat tcttttggca atcatttctc catcagaatt tcctccagat
     2281 ttggtatttc ttcctccggt tcctatttgg gctataggtc ttggttatta gttcgtgtct
     2341 tatttgttga agatactttt ctaagccttc tgcctctatc gattcgaatg tcatccatct
     2401 attcctggtg caccattact cttctttgtt agcttatatg cttcttccag tgtctcccat
     2461 cttgcagacg tgaacataga ttccccagaa tcgccattcc ggttctgctt tcgctttgat
     2521 ttcttctcag gtcttgtaat tttattgtcg tttttttatat cattttttacc tttaccttcc
     2581 ttttgttaaa agatttctac tagcagggtg tctttgctcc atgagcgtta cctcacttct
     2641 tcactactac gcacccatcc gccaccctct catctcggtt cacttcctgt tttccagtta
     2701 tagaacctat ctccaaggta tttctccttg ggatgaggga gggcttctcc agttgctttg
     2761 tatgtctcat tgccatgccg ttactaacac cccgctgaag tagaccaatc gtttcattcg
     2821 tttttcgtct ggtctattct gtcttcgcaa gtaccaagat ggctcgacct tcagaatatt
     2881 tctttcgagg caatctgtgt tattcgcttt cgctacggcc tggtaattcg ccattttcct
     2941 acgataatgt tgtcggtagg ctttaacatt ttggtttccc tctatgctgc tacccaagct
     3001 acaggattcc gacttttatc ctgcgtaggt ctatatccta ctgaacaata ctcacttctc
     3061 tggacgcact tggtactgaa aagaataaag acatcctcat tcacttcctc aatgatattc
     3121 tgggttttac tggcaaaaat gcaatacagg atatagagtt tttaagtact attcaagacc
```

3181 ctgatattgc ctctaaaaag caaagtattg ttgatgtcct ctgtagagat tccactgggg
3241 ccagatacgt gattgagatg caacttgctc gtgataaagg cttcgaaaaa acgagctcaa
3301 ttatatgctg ctaaggctta ctcaaggcag gctgataaag gtggttaagt aacattgatt
3361 taaagaaggt tttctttatt gctatttcca attgtaaatt atttcctgat agcctgatta
3421 tatatctagc cataccataa gagatgaaaa actaacgagc atgacttaaa agattttcaa
3481 tttatcttca ttgagttgcc aaaatttcct aaaaatagag aagagcagct agaaaatgta
3541 gtagatcgtt ggctattttt tttcaaatac gcagaagaaa acaactgatg gaagatttga
3601 gaaaaatagc agaaaaaatc accaataata aagctagcat atgatgaatt agataatgtt
3661 cattggaagg agaaggacct agtggcttat gaagaaagag ttaatggatt tacagaaaga
3721 agctgctatc ttagaacaaa aaactcgatg atgctaaact agaaaggtag acaagaaggt
3781 atccaaattg gccgtgaaaa aggtagagct gaaggtaagg aagagggtgg aaaaacagac
3841 taaaatagct gttgccaaaa actcactcaa ggccggtgtc tgtattgatg ttatcgctga
3901 aattaccggt ctctctcatt ctgaaatttt gcaactcaaa gaaaaaacat aatattccta
3961 cttattcact ttaacattta taaaaacctt ttctaagtta gacatgtccc ctaattaaga
4021 atatagtgaa ctgtctaaaa cagctatgaa gattgcacac agaattatat aaattactat
4081 cataacgtta cccaaacttc tatatcaaag ctacgtgctg tatgtttaat atggatagta
4141 tattaaccat ttattcccca actctagtct tgtattccta cttcattcat gttagtatta
4201 gaaccatcaa acttgctact ggtataccta attattttgt ggctaatatt gccaccttca
4261 ctttaaattt tccactactg gcatattata gctccacctc ttatattata acctacctat
4321 tgagattacc tccaagcttg gttactccta tctcagaaag caagttgata cttctgagtg
4381 gctgtgaact gatcctagca catctactaa gaagaagctg aatcatatct aagttacctt
4441 cttgagcagc acagtgtaaa ggcgtagaac catacatatc ttgagcatta atattagcac
4501 cataataaag aaggagactc actacattca attttcctga ttgagtagcg aaaaaaattg
4561 gtgtataacc aaaattgtct tgagcttcaa tattagcatt ataatcatca caaagaaggt
4621 ttattttgct cagatcacct gcttcggcag caagatgtaa gggtgttcta ccaagttcgt
4681 cttgaacctc aatgttatta ctatttggga tttctaaaga cgtagaattt ccttgtaagt
4741 tacttatcaa atctgtatta tgtattttac caaggtagtt tattacatta gaataaccac
4801 cacaagaagc ataatataga ggcgcccagc caccatgatc tttaatatta acatcaattt
4861 tttcttcgtc tataaaatat tttactatat ctagtttacc attgtaagca gcatactgaa
4921 ggggaattct accactatta tctttaattt ttaaactact tttttcacct accacaagat
4981 atttactac atttagctcg cccccataag cagcacaatg cataagagtt cggccatcgc
5041 tatacttagc acttaagcta gctctttgac ttacaagatc ttcaactttg ctaagatttc
5101 catattgtgc tgcaatcaat aattctttat ctaattgcac tcgttttaaa tactctacaa
5161 tattattgtg tttttgatca gtagcaatat ccagcaatgt tttaccgcct ttgtcttgag
5221 tgtaaacgtc agccccctta tctacgaggt accttactaa atccatcatc aatgctcatt
5281 cctttgctga gaaaaaaatc tataatgtct ttgttattgt tttcagcggc aatgtgtata
5341 ggatttctgc caaaaatgtt ctcagcgtta atatcagcac ccttttctat taaaaagtta
5401 gcaacttcta acaagcctct ccatgcagca aaatgtagcg gtgtccaacc atttttatca
5461 gtatcattaa tatcaacaca tttgctaata aggtacttta gtatatttaa atgaccacta
5521 taagcggcat tacatgtagg gggaccacaa taatgatctt cagcctttaa actaacaccc
5581 ttttcaacaa ggtacttaat tatgccaaag tgacctttta atacagcatt gcgtgcagga
5641 gtattaccat ctctgtcttc agtattaaca ttagctcctt gattaacaag gcctgttact
5701 tcactaatat tgtcaatttt tgtggcattc aacaatgcgt aatccaattg tacttgcctt
5761 gaaactatta aattagaact agcatctgca tttctcactt ttcttaaagt agatccacaa
5821 tcttcaatca tccacatccc ttgtgaaacc tgacctcctg gaatccaaaa aaatactcta
5881 cgcctatcat tgtcatattt agcataatca gcagcatata aatattcatt aagctcaaca
5941 ttcattatag aacagctgtt accatcaggt ttaaccgtcc acaaatctcg tacaactctt
6001 cctcctggaa tccaagtaaa taccattcgc ctatcatcat tataattaaa atagttactg
6061 gcagcataca aagtttcatt aaatgcaaca ttcactatat aaaagttgtc accatcaggt
6121 ttaatcgtcc acctaaactt atcatttttt catcctttgg aatccaagta aataccgttc
6181 ttcttttgtc accataatca tcgaaacatc ggttagcagc atataaatat tcaccttgat
6241 aaacattctt aatacatact tttgaagaaa acactgcatt tctcactgat tctggcaatc
6301 tgctttttag tttctcaagg ttagatctat cttcttgatg gacatcagaa tagttcttca
6361 tttccattac ttctttgaca taataagcta acttaaatat tgcactatta tttaaatcat
6421 catttctttg cattgcatca aacacagcta cataacctga tatacgctga ccagcattat
6481 gatgactgcg tacataactt aatgtttctt tcgtatctac tctcccatac acatcattta
6541 taactcattt aattatatca tcaaacagct ttccatctga tttataaatt tcattagcaa
6601 gttcattaac tgaactatac ctattatgca acatgccatc ctttagcttc tcttttgcta
6661 cactttttat tgaatatctt aacttttcta caaaaaactc tgcccatctt tttacattaa
6721 gtgcagtatc tgcttggttg ataatttcgt tctttacgcc ctcaactact cccatcatct

6781 tatatgggtc gctactttta gcttcaatac tagcattctt atcaagaaaa aatttgacca
6841 tatctaggtc accactataa gcggctacat gcagaggtgt tctaccatca ttatctttag
6901 ctccaatatc agcaccttta tccacaagaa acttaggtat attcaaattt gccttgctga
6961 gcagtctagt gcagagggtt acaaccatag atgtctttat ctttaacact aaaatgcttc
7021 ctatcaaaaa gaatttctac tatgtccaac ctaccttttt ggatagcaat gtataaaggt
7081 gttaaacgtt taccttcaat atcaatagtt tcaatttttc tagttaaatc ttcagcttca
7141 ctgatatcac tttgcactat agcattaaat acctgctctt ttaataaatt tactttctcc
7201 ttgttaaagt cctctataat atcctctgaa ttacaaacag agactatgca ctttggatta
7261 gaaccattgt atcattaaaa gcgaacatca tttctcttgc aggttgataa ttactccaat
7321 tctccacttt tgcaatggtg ctatttccat atgatagcac taaactacca ttagcaatct
7381 ccatatcaaa acccaataca gacttatctc taaaatcaac taaaccaatg tcattcttgc
7441 tgataggttg attatgataa atctgtaacc cttgttcttc tggttgataa tatctgaaag
7501 agttagggtt catttctaca tgctcaagcc ttaagtttgt actatcaatc ttataaagct
7561 cattattcat tcgtaataca atttgttgat gttctgggct ttgaaaataa tcaactaaac
7621 caatggataa agaccattta gtaccttcac taccaagtac tgtaatatct tttatctcta
7681 agctatcctc tgtaaggtcg gaaattgatt gattataaca ttcatttata taactccttt
7741 taccctcttg tatgcttata ctatcaacat ctgttacatt caattcagca aaatcaataa
7801 tattgtaagg tatgttctga taaggcactg ctttgctttt tatttttaaa ttaagtctta
7861 tggggaatga cgagatttcg tggtttaatg tatctggtag tttgataata tactgatgtt
7921 gtccttctcc cccttctact gtggcgtctt gatagtaatt acatagtgtc atatagtgat
7981 cataatcata gctccttcca ttattacggt atttctgagt aacgtcagaa ttattatagc
8041 taaaatcaac aattacctga tctgagtata tgccagcaat tatcaccttt tcagcaggta
8101 ttcccatatg atttttagtg acttctattt gagatattgg tatgtcaaaa ttattgtcat
8161 cagcaatacc ttttgcaatt tgataagtat caagtaactc ttgatctaga atacttgctt
8221 gtataaatgc ttttaacttt ttttcactat tttgtatgat ttctctgtca gtatcacttc
8281 ctgaatttct caacttttga tacctaatat cttctattgt atcagacaaa gacattgtat
8341 tgtaacagac tctgttattc cctggcaatt tacttgaatt actagatttt taaaatcatc
8401 gattgtagta cttgaagcaa tactttgtgt atcattagct aaaagatcta agctatagct
8461 gtctttttct gtatctttac ttactcccca ctgtgtaata ttagcaactt gaactcttat
8521 tacgtcaata tttcctttca tttttcctgt actgcctaaa tcacttatta tcctagcatt
8581 atcactgtct tcattaacga taaatattgt ttgctttaat cccctgtgat gcacgttttc
8641 tttgctgact actctaactt cataatcttt atctgtgcag ttagatgaat tctgccaaac
8701 tttcgcgttt ttttacatta attaatctcg tttcacagtg agtagtaata ttttgcttgt
8761 tgtcagagtt agatacatag ttatatgtgt taaccaatct tatgttatta ctattaccat
8821 aacgtatagt tccactatgc aaatttgcag cgatattatt agctttaaca ttcattacta
8881 gagtattttc ttttcctgct ccacctttga gggtaccaga catataatta ttaataataa
8941 aaatagtttc attgttttta gagccaatat aacgtatatt actaccctct ggaatatgca
9001 ttactgctgg ataatttgct gctgctgtta acgtttttgg ccgtgaaata tataatatct
9061 atcctctagg aaatgtttgc tttttttgtat tgccaggatc tttccttttc ggattagtaa
9121 aaataaaagg ttcgcctgag agagtaaagc tctcttgaca gtttctataa atttgccac
9181 tgttacatgg agcgaacgca tcgtcacctg tgtgctttgt attgattttt ttgctgcatt
9241 gagatttttg tatcggattt gttaatggtg catctacaac tcttaaacca agattttcat
9301 attcatattt tgttagatga gggatatgtt ttgcatccgt attaacaaca taaactttct
9361 tttcttcaat ctcttgatag cattgatgac tattctttat accacagata aacctttccc
9421 aaccttcact cataaccaaa gtataaggtg tcatagtcaa tatcttagag atatcaatag
9481 aagacaaatc tctttttaca ctagaagcta tattaatttt gtcaaaagag atatctttat
9541 attttttttc taaatcacat gtccacttca tctgcgtcgc acaaccagga ccatgcggtg
9601 atggcataat acatattttt aatcacccca ctatatttct cttcaattga gattacgtat
9661 tgtacaaccg catctatgtc ataattgtca tttaacagtt taatagctgc ttcttttacg
9721 taagttaaat accctttttat tctaattgta tcttcatatt cttttctaca tctcttcctg
9781 taaaaaactt aatcacccct gcaaaaaata cttccgcttg ttcaccacca ggtaatagag
9841 tcacttgctc tgctatcgct ttagtatact ccattctttc atgaattttt acgcccattg
9901 ttatcaatac atcagcaacc atagtaacta ctgctgccct acctaaaact ttgcctgcag
9961 cagataaccc ccttaaaatc ttagtagtag cacttacact ttttatcgta aaactaaccc
10021 ctgcacttat aggccttatg ctcatgcttg ctagttttat accactacta gctgtccaat
10081 aataatttaa tggatgatta ttaccttgag ctatttcgtt tatactgtat ccaaattgat
10141 aagcaatggt tgcaaatgta atactttgaa ctacaggatt actgataacc ttaacaaaag
10201 cttgacgtgc aggccctggt attttaccaa tcagttttcc tactttacta ctaggagaaa
10261 tacttacacc taacatcatc catagcatct agtataccat tgataccaaa tacttgcttt
10321 aaagcatcat tattatgtcg tccaagtgtt gctgtatcac ctatactatc aagcaagtca

10381 tatatagcca tcatggaaaa aaactgtcca ccttttgctc cttttatttt cgctgaatca
10441 aatttatcac gatcaaatgc aattcctttt tctttaaggc tgctttctac attacaaata
10501 taatctgcaa aaactttttc accaagatca tcaaaatgac ttttaaacct gtcgaaagta
10561 tcttccctaa ctccttttgc taataaattc tatttaattt cctttacaat atcattgtaa
10621 tcttgattag ttccaacata tcgaacatca catatgtctt caatgttttt tttattctcc
10681 tacaactgct tatcatttga tagttgatgt aatttattat gaattttctc tctttctgca
10741 tctgtataaa atttttaat tatatcatcg aattttcttc atagccatcg atgcttaaat
10801 aatactcaat attttgtcct tttattttt cttttataac gcgatcctcc tacttttact
10861 aagatctcaa taccatcact ataatcatct acaaatatct ttctgcattc ttcatttcta
10921 taatatcacc tattatcaat tttttatcat taccattgtt atcttttact tttacatata
10981 catcagtcct atcagcattg cctatcatat caaacacttg ctcacgacct atatctacaa
11041 tttcattagt taaatcctta gcattatcaa tatcaaatag ctctttaaca cgttgctttt
11101 tttcttcctt tgtaaccttt tcttcatcac gtgaatctat gcagggtata cctatatcac
11161 gtcttttcct tgttgcacat tggctaaaac taaaaataga actatgaact accttaacag
11221 gagtaaactc atttttgctt ggtagaataa gagaatctgc attactcgct cctttgtcaa
11281 agacaacacc cataaaagcc actttcctgc catctgtaag cgttttataa gtaacacctt
11341 ttacatactc cttatttatc tgatcatttg cctcttttac taatgcatca gctgttttac
11401 cttcccttgg tcccttaagc tctaatgcta ctggatcata ctccttagca ttactagctc
11461 cttctgcaaa cttaatacca ttaaccagca tatcaatacg ttttcccctg ccagtttgaa
11521 actctgttaa tactaatgct ctaggagatt cttctaattt caaatcacta taataactaa
11581 atgccccatg taacactcct tgaaaattag cctcttcatt aataaaatct ctaaaagaaa
11641 acattccttc tccaactttc tttagtagtg aattatattt atctactggt agagactggc
11701 ctgctgcagg agtaacttga gacttaagtg cctcatcaaa tgcctctctt agtccattgg
11761 gttaaggaac atcttaagga attatatcta ttaacctcct ctatgttaaa aaatctgatt
11821 tcttttccgc atgtaagctt atgtatacct ctgtgatttt gctttgctcc ggtagatcac
11881 caggtatagg tatcttttta ctgctacgat cccctcttcc cccttctcta acatgaaaaa
11941 taaatacctc tttactacga ccttctctaa tcaacgcaaa agttgttaca ggctttccgg
12001 atcttgtggt agaagcaaga gaatattcac cgtatgaaaa tataaatttc tttagatccg
12061 ctcttttaaa tttatcagca agaagaagtt gccctaagag aaatctacta aagtaattac
12121 ctcctttctc tggcgtacca ggaaatgtgt gataaatacg ctccagtggc tgcttgattt
12181 ttctttaaca ttagtgaccg tatcttgtct tccatcccaa tctgaagctt cctttaagag
12241 tgcttgcatt gtaggtagag caatttctct gtcttcacgg ggtgatatat gcattgaaaa
12301 tttttcacct tcagttgaat ccaaatttaa cccttcacac agtacattat ctgaaatagt
12361 caaaacacgc attgtatttg gctgaaagga ttttgcataa tgttttgctt gctctaatgc
12421 aatagctgat gtagtttgac tcccatcttt cactttacct cctttctcta actcttttaa
12481 ctcttcttta gtggcagctt tcagttctat aataatagga acagcattta atgatctatc
12541 cttaccacga ggcactagaa caatatcagc gtaacctctt cctgcaaatt gctctaagta
12601 aaccctgaga ttatgtctat agcgaaaatt tactaaagca cctgctataa acccatgatg
12661 tgctgcttct cgtgctttat cagcataagt aagcgaaccc ttgtattttg agtgaacttt
12721 agtgattcta tcaatgactt tcttggaaga ttcttcaact ttacttgcat cagaattagt
12781 taactcagct atataatcct cgggcttgct ttttgtatta tcattttcat ctttccatat
12841 ttcctcttct tttttaattt cgtaaaaaat tatgactaat agcagagtca agtatttctt
12901 tatcttgtgt ttcaacaaaa aatattctct tcatttttgt tgatttgcac taacttagat
12961 ttaaccctac ctttaattct atctttaata cctcgtgccg taccgatctt ttgttgagtg
13021 tcaggatgca aattttcttt taacatatat tccagttcac cagaagtaaa ttgatgacca
13081 gaattaccat cttctgaaat agaaaatagt tatataccttt caatagcatc tacatcactt
13141 ttaatttgac tatttttaac aacaactacc tttaatgttt ttgcagtggc aaaacgaaaa
13201 taaacttttt tgtcacctaa tctttccgca atttgtgtat ctggtaaaat agagaagctt
13261 cctaaaaaga aatgcgaaaa aaaccccttct ttacctaccg aatgtaaata tgatggaacc
13321 tggtcaatgt atgattgaaa ccttgctttg aaagattcaa aatcactttt ttgttgggtt
13381 gaagtacttg ggccaggttg actaactccc gctgtctctc gaattaacag ctctattaat
13441 tcaaaatctt gtggtttttc ttgacttaac atatagcccc ctaaattata taataacttt
13501 ttattcacct aaccttatat gagatttatc atgcattttg ttaagcttta catcagataa
13561 atcaccactt ggaccaggta tactcttttg gtcaagatct acatcaacag aaccactata
13621 agataaaaac ttatttaatg aagcgtgatc tccttgctct aagatggaac atatgtaacc
13681 tgctcgcata gaactcataa attcttcaat ctgtttagaa ttagttttgt ccaatattgc
13741 ccacacttgc tccatacaac ctttctcaac caaaggtact aataaacttc cttgaaaagt
13801 agaattgttc atcacttctc catttagtgc taaggtacga tgactatcga atccatcttt
13861 cattaacata tgcatcaaaa gcttcgcacc tacatttaag tattgcccag aacaaccccc
13921 aagttttata tttcttaacc aggtacaata ataactaccc aacaccacat cacttggctt

13981 taagcagtcg aataactttt gaaattgatc aaagttaaac atttctaaca tcctacctag
14041 agagaaccat aataaccatt tttctccaaa tctcttttca aaagctctgg atatctatca
14101 ggatttattt ggcttaaaca aaattcaaac acatcaggat atccattatc gtgtgctgca
14161 tgcactgcaa ttttcataaa aaccttatct ttttcctgag cacttaactc actttcaggt
14221 aatgatttta tcttattcca aaagaactct actgcttctt acacgttttc tacgcatact
14281 tattgctgca cggtgtaaac caagcacata gggatgctga tcttttagat ctaactctgc
14341 aatgtatcca ctaacgaaat acgaccaaaa ttgcatcatc gcatctgtat accaacttcc
14401 acttactgat ttgactaact tttttaatgc atctgaatct ccatccttta tttcatcttc
14461 ccgtttaaaa tgctcaaata gggtaatgat gtcttcttct aagcaacacc aacatgcaat
14521 ttggtaacga tcaaaatcat ctagcggatt atttgtacct ttccaactct tgggttcaat
14581 catcttttta gccactattc ttctattgag tttgatacca tgtatcgtat cggtttttc
14641 ccaacattct tcatctgaca atatatcaag gaaaaaagga aggtttccgg tattaatagt
14701 ttccaagtac ccacgccccc aattccttat ttgaagttgt ttagattctt tataaggatc
14761 gtcatcctta ataatagaga aaagctgcct tggttaggtc atctaagtcc ataattatta
14821 cctccacaaa caaaagcgta ttaagatata gaactatgat aatttataca ttctatatca
14881 ttttcaatat cgtactacat ctttgtaacc tgtcaatcac tattttagaa aaactttcta
14941 acccaaagta attttcaatg aaagcctttt aataggctga atatatgata ttttatttat
15001 ttttaaattc tgtttttat aagtttatct taattcttcc acaaacaaat tattattaac
15061 ttagcaagat atattattag gatctcaagt aaagcaacca tttccagcac caaaaaacaa
15121 gggcggtgac aacatttcgt tttgggatcg tgtggtctac caagtgtacc gttcgaactt
15181 ttccttataa taattttacc atatcacaaa atcagctata ttagcctact ttaactaaat
15241 ttatatacga attaatttgc ctagcttcgt tgcaaaaatt ttgcgttttt attttcacaa
15301 tactattttt acgcaacgtg ttctcgcaaa ggtaggtact atttttatgt ttttaaagaa
15361 agtaaataat caaaattttt gtgatgctat agagtctggt atttttata agcttttaca
15421 tgattgacaa tcactgaaat accaactaaa atatcaatgt ggtaatgtta agcgagaaga
15481 aaaagtggat tttaaacaat atgtcaaagg ggatactctt ttactttcat ctgacgccat
15541 aaatataagc gacttagtaa gcttcctaga aaggaaccaa agcataacaa ggcttatctt
15601 gagttttagc aaaattggta atgaaggtgc aaaaatttta gctagtggca atcttccaaa
15661 tcttacttca cttcatttat tttgtaacaa cattagcgct gaaggtgcag aagctttggc
15721 taagggcaat cttctaaacc ttgcttcact tcatttactt tttaacaaga ttggtgatga
15781 aggtgtaaaa gctttagcta acggtaatct tacaaagctt acctcacttc ggttagtagc
15841 taataagatt ggtaatgaag gtgcaaaagc tttagctaat agcaatcttc caaatcttaa
15901 attgctttat ttagggaacc atcttgatac aaaaagtgta aaagctttac tcgctaccaa
15961 taatgttaga aatcttctta gccctgaatt agcaataaca ttggtgataa aggtgcagaa
16021 gctctagcta agggcaatct tctaaacctt gcttcacttg aattatctaa taacaacatt
16081 agcgctgaag gtgcagaagc tttggctaag gacaaccttc caaatcttac ttcacttggt
16141 ttaagttgta acaacattgg taataagggt gcagaagctt tagctaatgt taaccttaca
16201 aagtttacta aacttgatct atttggtaat aatattgatt ttgagcatat agaggttcta
16261 gccagatctc aatgttctgg aatcaagtta ggtgatcgag atcagaaggt cttttatgat
16321 ttcctattga aaaaatgttc taacaatcaa gtcgtagatg aagagagcat aaaaaggatg
16381 atttttagtg tagctagaag taatcatagt ggagatttgg ttaggtacat attgacacaa
16441 cctgataaat acccttttt aatcaattca aaagatgagc aagggcacac actatctcat
16501 ttttataatc actcacctga gatacaagag tttttgttca agcatggttt agtttctgaa
16561 aaagatagta cactacgaaa tattactaat gataaacaat ctgtgcacgc aggtttagca
16621 gttaagaaaa cgaagtttat cactaaaaac ttagtagaat ctataaaagc tagtgaaggt
16681 caattagagc aagcagtaaa ttcttatgtg gaaaatatag gactgcttaa acaatatcag
16741 aatgatccaa taagactaag gcttcttagt ttaactaata atgaaaaaaa agtgcgatgg
16801 aaaaaacatt acgtgagact gatcctacgc caggtgatga aaaatttatt gaggctgtta
16861 ttgataaagc cgagcaagca ctaaaacagc agtatttaaa aaagaacttc cgcggtgaat
16921 atgaccaagg gtatctctac aaataggatg cagtatgatt acacaagggg tagcgaaagg
16981 tttattacta tacctgaatc tataggttat ataaaactac ttattgatga tttttccgtt
17041 ccactgaaag aaaaaaagga gttgctagtc actcttatgg aacaaaatcc agatttggta
17101 aagaacaaat tatctaaggt aaaagaaaaa cttggaaata gcgtaatcct cgataaagaa
17161 cagtttaata aaacagaact acatggatta ttagatggta tagatgacgg taaaaaagta
17221 gatgagttgt tcaaagaaat cagttgtttg aatatagaag aggtctggag agagcaaaag
17281 aatttattct cctaaaacaa atatatatag cagcaacaac atatggtgaa aatagcagtg
17341 cttgcatcca aggcacatgg agtcaaatta ttggtagtat aaacgaaatt agctccgaag
17401 tcgtggcaca atatgatcgc tatttagaag aagaacaaag gcgggaaatg caaaaagatg
17461 ctattacaga agaaaatatt aatccatttt tggaaggttt ggctaataag ctaattcaac
17521 acgtagaact taatccagaa ttaaaagaaa ccttagaaga ttttgttgta tgcactgtgg

17581 atattgataa acctgaagaa attaccttta acagcaaaaa attttagcag agataaataa
17641 atgctttagt gaaaatatta atgacatttt acagaattat aatagaaaca taccaagtag
17701 aggcgaatgc ggccttgtca tcgagggatt gtcagaagtg aaagtaatgc aaagctttgt
17761 acaaccaggt caagagccat ttaatcttca ggagaatatc acaggggaaa acgtaccgtc
17821 aattccagga aagttacaac caagtaaacc aagtgaaaag agaagtaaac tgcctattgt
17881 aactgcctct gcactggcaa tagctggagt tgtttcggga atagctattg cagtttactt
17941 agaaatgtta gcagtcggaa tagcagttgg agcttgctgc ctagttgctg ctgctatcat
18001 atattactgt aacagacctt caagttcact tggaaatagc aatgttcaag gatcttgcaa
18061 agaagttcaa gaaccccatt ctgaaggaaa gaaaaaatag tgagagatgt tctttaaata
18121 ttaagtgata accataataa tttagagatt atcttgtttt tttttaagtt tacttgacga
18181 ttatggtcct tctagcatat cactagcttt aagatttttt aggccagttg cctgatgtac
18241 tacattaata ggaacaccta cctcaagtag attctttgcc atttctgttc tccctatttt
18301 tttacccttt tctttatcaa tctgtattct tttagatact gacataatta atgaataaaa
18361 tttatcacgt atttcttgat catcaatttt attatactct ctcactaaat ctagtacccc
18421 gttttctccc tctctatcct cattacttct ttcaatttca acattgcctt tttctattag
18481 ttcactaatt tttgccagta gagttttggc gtgcgttgat aattcgtctg ctgtgttaca
18541 tagttttcct attgtcatga tatgggttcc ttgctcatag cgtcgtattt gaccacctgt
18601 taaaccgact tcatttccta aatcctcctc agaacgtccc cttgctagtc tccagtcttt
18661 catattttcc cctgttttat aggctataaa attaactatc tctgttttat tatgtttctc
18721 aagttcatct ttagacaagc ctgttgtttg gagaatgata tcaagggaaa tacccgcttt
18781 aagtagattc tttgctactt taatctttcc tgccttttta ccttcttctt cgcccatctg
18841 gatagatttg tttaatgcag aggccatctt acataattcc tgattcctaa atttttttaca
18901 ttctctaact agatttaatg ttttaccttc aattttctct gatatcgtcg attcaggcag
18961 tagttttgcc atatcaatca ttagttccac tactatcata cagaatataa tgattgaaat
19021 cggattttcc cctcgctcat atctggacat ttgcttctct aatactccga ctttacttcc
19081 tagacctgcc tgagtatatc ttttcttttc cctctcatgt tttaattttt tccctacttc
19141 gtagtctgtt aacttagtgt cattagtcat gtctctttaa tataccttga gacacatata
19201 tactctttaa tctaagattc tattaaattc actttcagag cttagtgtag tttttcaact
19261 ttataattat cctccctaat tgcgttttta tactctttat catctttcca cactacttca
19321 tctttccaaa gaattttaca accctcccgt attaagccat tgaaactaaa acagccatac
19381 tctgttactg ttttataatc cactagatct attcctacac ttggacatgc tatcaccagc
19441 tttcttacaa ttgatatgtc tccttctgtt ggtgcttccc tttccgggta gttatgtgat
19501 actaccatag aacttgcttc tattcttacc ccttgctcca taagctttct cgtatatatc
19561 gctttttatc tattttaaaa acatcatata cttcttctac caataaataa tgcttttcat
19621 ccatgtaaaa tactcttaag tgttctttat ccgattgttc catcaaatat tcaactaact
19681 ttttcgagtc gttagtaatt ggtaaacgtt tatctaagtg tttttgcgtt ccattttgta
19741 cctcctagcg aagccaaata accataagtt tttatactca ttgagcagag ctcacagagt
19801 atattaagtt tgattggata acggttggtt gtacaggtat aaaggaatcg agatagatat
19861 agacttccat atatcaaaat catcaggatc gaaaaaaaat ttgattgagc catgtccgtc
19921 cgtccgtccg tccgttaaca cgataacttg agtaaatttt gagatatctt gatgaaattt
19981 ggtatgtagg ttcctgagca ctcatctcag atcgctattt aaaatggacg atatctgact
20041 ataaccacgc ccactttttc gatatcgaaa atttcgaaaa accgaaaaag tgcgataatt
20101 cattaccaaa gacagataaa gcgatgaaac ttcgtaggtg ggttgaactc atgacgcaga
20161 atataaaatt agtaaaattt tggacaatgg gcgtggcacc gcccactttt aaaagaaggt
20221 aatttaaaat tttgcaagct gtaatttggc agtcgttgaa gatatcatga tgaaatttgg
20281 caggaacgtt actcctatta ctatatgtac gcctaataaa aattagcaaa atcggagaag
20341 gaccacgccc actttaaaaa aaaattttta aagaaaattt taacaaaaaa tttaatatct
20401 ttacagtata taagtaaatt atgtcaacat tcaactccag taatgatatg gtgcaacaaa
20461 atacaaaaat aaaagaaaat ttcaaaatgg gcgtggctcc gcccttttc atttaatttg
20521 tctaggatac ttttaatgtc ataagtcgaa caaaaattta catcttttga aatttgtagg
20581 ggcatagatt ttatgacgct aactcttttc tgtgaaaatg ggcgaaatcg gttaaagcca
20641 cgcccagttt ttatacacag tggtccgtct gtccttccgc atggccctta acacgataac
20701 ttgagcaaaa atcgacatat ctttactgaa cttagttcac gtacttatct gaactcactt
20761 tatcttggta taaaaaataa acgaaatccg actatgacca cgcctacttt tgcgatatcg
20821 aaaattacga aaaatgccat aattctatac caaatacgaa aaaagggatg aaacatggca
20881 attggattgg tttattgacg cgaaatataa ctttagaaaa aactttgtaa aatggttgtg
20941 atacctacca tattaagtac aagaaaatga aaaagttctg cagtgcgaaa taaaaaaccc
21001 ttgaaatctt ggcagatatt acatatataa ataaattagc ggtatccaac agatgatgtt
21061 ctgggtcacc ctggtccaca ttttggtcga tatctggaaa acgccttcac atatacaact
21121 accaccactc cctttttaaaa ctctcattaa tacctttaat ttgatacaaa tatcgtacaa

21181 acacattcta gagtcacccc tggtcctcac ctttatggcg atatctcgaa aaggcgtcca
21241 cctatagaac taagccccac tccctttaa aatactcatt aacacctttc atttgatacc
21301 catatcgtac aaacatattc tagagtcacc cctggtccac ctttatggcg atatctcgag
21361 aaagcgacca cctatagaac gaagacccac tccctttaa aaatactcat taacaccttt
21421 catttgatac ccatatcgta caaacaaagt ctaagttgcg atacctcgaa aaggcgtccc
21481 tcacctatag aactaaggcg gcccactccc ttttaaaata ctcattaaca cctttctttt
21541 gatacccata ttatacaaac gcattctaga gtcacccctg gtccaccttt atggcgatat
21601 ctcgatcacc actccctttt aaaaccctca ttaatacctt taattttata accttatcgt
21661 acaaacacat tctagagtca cccctggtcc acctttatgg cgatatttcg aaacggcttc
21721 cacctataga actaaggccc actccctttt aaaatactca ttaacacctt tcgtttgata
21781 cccatattgt acaaacgcat tctagagtca cccctggtcc acctttatgg cgatatctct
21841 gaaaaggcga ccacctatac aactaccacc actacatttt aaaaccctca ttaatacctt
21901 taatttgata cccatatcgt acaaacaaat tctagggtca cccctggtcc acctttatgg
21961 cgatatctcg aaacggcgtc cacctatgga actaagggcc tccctttaa tactcattac
22021 acctttcttt tgatacccat ttgtaaaaca tttagtcacc cctgtccacc tttatggcga
22081 tatctcaaac gcgtccccta tgactaagga ctcctttaa aaactcatta acctttcatt
22141 tgatacccat atcgtgcaaa caaattctag agtcaaccct gatccacctt tatggcgata
22201 tccctaaatg gcgtccacct atagaactat ggcccactcc ctcataaaat actctttagt
22261 gcctttcgtt tgatagacat gtcatacaaa cacattccag ggtttccctc ggttcatttt
22321 cgtacatggt tattttccct tatgttgtca ccatagctct caactgagta tgtaatgttc
22381 ggttacaccc gaaattaacc ttctttactt gttttgttaa accttgttca atcagactta
22441 catagccact ttgagtaacc atgatataat cttttagttc taccccctata tttggtagta
22501 ttaagacaag gtccttagca atttccatat catcttttaa tggctctgat gtagtgagat
22561 aatgggtaat ttttattgaa gttattgctt ctttcatcaa tatttgttcc ataacacttt
22621 gtacagtttc tatacattca taaccgtagg tattttctgt aattgtttta cactctgcat
22681 ccatgtaaac cacttgtatg cctttttat taattgtcat attttctcct cagaacaaaa
22741 aaattccggt agtataaaga ggtttctgct ttctctaaat atgtgcacca cgactgataa
22801 ggtactcctt cacgtcctct ttaatatctt tatcttgata ttcactatct tgagtagcca
22861 aatatagcgg tgtattatct ctcttgcctc tggcatttat atttgcccca tttattacaa
22921 gacatttgac catgtaaaag ttacaatgtt cagcagctat atgtaatcca atggtgccat
22981 cttctgttgt agcattgaca taagctcctt tctctattag gtattctact acttcaacat
23041 tatctcgctc aacagcatta agtaaaggtg tccagccgtt ttctcccaca aaatttacat
23101 caactccttt ctcctctacc aaatattcaa caacgtccaa gtggccattt aatatcgcta
23161 tttccaatag atcattgcag tcataagtga catcttttcg gctcatcaaa tattctatag
23221 tatccaaatc accacctaca atagcttcac caaataaagt actgccatat tcattaattg
23281 gtgccatagg atcaatactc ttttcttcta caaaatattt gactattcta acatggccat
23341 attgaaatgc ataatatacg ggagtacttt cattgtcagt aatattgtaa ttggcaccct
23401 gatttattaa aaatcttact atatcaatat ttcctgaata gacagcgtaa tacaacggag
23461 tccaactacc ttcatcctga gcgtttaaat cagctccttc aattagaata tatgctgtta
23521 gatacaaatt acctttttt gcagcaatac acaaaggagt acatctgtac tgatcaatag
23581 cattactatc agcccctttc tctataagat gtttaactgc ttcaagatta ccttcttcca
23641 cagctaaatg caaaggcgta tttcctttat cattcttaac attaacatct ttcttatcta
23701 ctaaatactt ttctatgata ttacacatac caaccccact aaattaataa aactattcca
23761 ttctacagac acaaacatta tttttaatt aactgcaaaa cccttataaa ttcctatatg
23821 cactgctcat tctctctaat gagggcattc ataattatgc taatatctta tatatattaa
23881 ttaacaacct cactttcttc cctcttcaat ggcctcttct atgacggcag ctacttcgtt
23941 cagcttcttc aactgattga tttcctggtt tttacgcatt tccttcagca ccttttgata
24001 aagatcttgt cttttctcat tatcgagcag tttgcagtat agttttagct gctcaggggt
24061 ctttgatagt agtcgtgcta acactgattc tcggttattg ttattattac tcatataaaa
24121 atcctcacta attaacgctt caatttttt taatgtttac tactgttttc ttagtagtat
24181 tggcataaag ccatctattt tggcggaagt caagaaattt ttaaatattt atttgttatt
24241 aataatggta aatatattgg cttcgataga gcattttcta cacaaactag gaagaggttt
24301 ttcatctatt cgttctacat cttccacttt ttctagcttt gtgccaggct ttagagcatt
24361 atacaacgca taactcacac aagctaatcc aacaattccc ccaatcaccg ctatggcata
24421 cattttcaat atataagcta caacactact gactagtaac gccacaccca cacactacca
24481 gatatgactg cttgcgttgt taatggtttt tctcttttat ctgtagcatt atcatcaaaa
24541 cgaggtttgg gagataggtc attggaagca tttgactctt gttcatcagt cttcactgaa
24601 gtttgctgga ctttttcgct gttttcacat ataggctgaa gaccttcact acttttattg
24661 acatcttcgc ataatccggt gtcagtggta gctttatcaa ttacaggttt atgctccaca
24721 tcaaaactac atacttcagt tactatttca catgcgttag ctggcatatc agtagctctg

24781 gtactatcaa ttacagaagc agtatcttta tgttctgtag ttgttgttgc attagactca
24841 aactatcttc atcgcatggt gcagttgtag tactagcatt ggtggcagtc tctgttacaa
24901 cagtaagaaa tggtgcttgt tgttctatag ttgttctctt attaagcaca tctcccagtt
24961 ctctagaaaa ttttaagatt tcttctacag cttcactatt ttcacactct tcagcatagg
25021 tgaatggggt gtaaatcatt tcctcgccat ctgaatgttt tataattatt ctcttttta
25081 gaatatcttt caaaatacca ttttcctcag agatttttaa cactactctc ataatgtcat
25141 tgtctctacg tgatatagca tctgtaagta ttgtatgagt ttcttccgac catctgaatg
25201 ttttgtagtc atttctttca ttcagaactt cctttaatag tttttgtttt ttggcttctt
25261 ctaaaagatc ctttacagca ggcatgctta accaacgaac cgaatatcat ttcgttacga
25321 tattaacgtt aataaacgaa acaaagacat ttcgtttcgt ttattaacgt taagaacgtc
25381 aaattaacga aatgatttg tttcgttttt ctgccatatc aaaacgaaat caaatcgttt
25441 atgttgatta ttaatttcaa actatgctgc aaagctgatt gatggcggag tcattaaggt
25501 gaaagcatta gccaagctga ttgcaacttt tcctcatgaa ttttgacagt acgaacattt
25561 ctcagagtag ttttgacatt accaccagga ttacacaaac aaattacatg aagtttgtgt
25621 gtatgttcgc tcgctgttag caccttacgg cttcaccatg gctatagcat tgccagcaca
25681 atcaaacata taaagtatca cgtttttgt taacgttttt aaggttaacg aaatctttcg
25741 ttcggttaaa aacgagatat atttatcttg ataattttt tatcgataat attcgaaagt
25801 gtttcaacgg aaaacggtgg gaaatttttg atacgagtag cttaacgtta agatgcatcc
25861 ctgactttac agcttgatta tttttatgac ttatagctaa tgatagagaa gataatgtct
25921 cctctctacc tgatattgta agttattttc tgctaagaat cgtgcttgcg tatagctgat
25981 ttttgaatat tctatttctg aacactccag caatactttc cctcctctat tctgttgcta
26041 gtgtttgata ttcttttctt cgtatctgtt aactctatat atcacatact atgtcaggtt
26101 tgacgttttc ataatgtttt cccctattaa gatttattga tttagtggta gtagttgcag
26161 ttgctcaccc cataaagttg gtgctttctt tcaatcaaag gtattctact gttttaaatt
26221 ccctgtaaag atttagctta tcttttacat ctaaacagca ttaacacgat ttggcttttt
26281 gtagatgagt aattctctag ggattatcct attgaattct ataatatttt ttgtcacatt
26341 gtctaaagtg cccagacact gggatcagaa ttttgttatg cctgagctga tgagctaaag
26401 gtagttgtct tacgctaaaa caaacgtttt ttaattaagt tgcatagaag ctggatccca
26461 gtgtctgggc actgggatga caccatttgc tgtgcaattt accttcaaaa atgaatgttc
26521 gtacagctat gtgtcagcta cttgcatgac accctgctgg tgggacaatg tttgtatagt
26581 tgtgcgtcac gcgctggaat gacgaattac ttaaccgtca taccgtcacg gtatctctta
26641 ggatagatcc cgctaacacg tagcgggatg acgaatttgt atcggaaaac aagtagcatg
26701 acggtttgtc aaatgtctct cagtttctca aaaaaatcct ttaacaaaaa gagcattctg
26761 tttctaatat cccaccataa acttcaggta tatggctgca aaattgaaat atcttagcac
26821 cattttcaat tcctccacct tttggattat aagctccaaa gtacaatcgt ttaatcctgg
26881 caaaggaaat agcttgagca cacatcggac atggttctaa tgttacgtat atgtcagaat
26941 cataaagcgt tgaagttgag agcaactcac atgcctgctt gatcgctaac atctctgcat
27001 gtgcagttgg atcgttggat atgttgtgtg cagaagaaac gacattgctt ccactaatta
27061 ttacggcaca tatgggaacc tcatcatttt tttgagcaag tttggcttgc tctatggcaa
27121 gctccatata ttgatacttg aacactttcc tttgctaatc ttcttttttt taaattttat
27181 taactgctgc cttaataaac gatataaaaa gaggatgagg agaaaacggc tttgattgaa
27241 actctggatg aaactgcaca ccaataaacc acggatgact ctctaactcc actgcctcta
27301 tacatgttcc atcttctgat atgccactgc atagcagccc attttttcc aaatcatctt
27361 tataatctga attgattatg tatctatgtc tgtgtctttc tgaaatagta gtgttactat
27421 atgcatccat cattttagag cttgcattta tattacattt gtatgcccca agtctcatgg
27481 ttccaccaag atcgtcatct tgatcgccag ctaacttaac gattggatgt ttacagttat
27541 gaaattcttc agagtgtgca tcttcaagct taacaacatt acgagcaaac tcaataattg
27601 caagctgcat accaaggcaa attccgaaaa atgggatatt atttgtacgg gcataattta
27661 ttgctaatat tttactctct actccgtcat gaccaaaccc ccctggacca agaattgcat
27721 gagaattctg taacttctcc cctataagct tttcatttat aggtttttct tccttctctc
27781 ttgagttaac ccaatttatc tttactttga ctttattact aatcgcacca ttatttaatg
27841 cttcaactag tgatttatac gcatcaggga attcggtgta ttttcccact atagacacag
27901 taacttcctg tgttgggtgc cttgtggaat gcactatttg atcccattca atcaagcttg
27961 gttttggctc acttaaatga aagtgctcca aaatttgcgt atcaagccca cattggctat
28021 acaacatcgg taactcatat atatgactca catcaggagc aggtatcaca ctggacaaag
28081 aaacattgca aagcttagct attttctctc tctgattatc gaaaatttct ttctcactgc
28141 ggcataatat aatatctggt tgtaaccccg cggaatttaa ctctcgaact gaatgctgcg
28201 ttggttttgt ctttaattct tgcgctgcgg taagatatgg tattaaagtt aagtgaataa
28261 ggataactct ttgttttcct aatgtgtagt taacttggcg tatagcctcc aaaaatggtt
28321 ggctttcaat ttcacctaca gttgcgccta tttcacatat tataaaatct aaaccttccg

```
    28381 tactattaaa aatgaacaat ttgattaaag ctgtcacatg aggaatgact tgcacagttt
    28441 tgcccagata atcaccacgc ctctctttct tcaacaactc atgatatatc ttaccagttg
    28501 ttatattgtc atcttttgtt gctttaattc cagtaaaacg cttataatgc ccaagatcca
    28561 aatcagtttc accaccatcc tcggtaacaa acacctctcc gtgctgagtt gggttcattg
    28621 tcccaggatc aatgttgaga tacgggtcaa gttttctgat acggatctta aaaccgtaag
    28681 cttgaagaag cgcaccaacg cttgaagcaa ctaagccttt accaagcgat gacacaactc
    28741 cacctgtgac aaagataaat ttagcttcct tcattaattt tcaaatggaa cagaagtagg
    28801 ttcactttct tgcttttttt ctaatataat tttctctgca atcgattttt tatgtacgtc
    28861 ttttgaatat aatcctgaca atagcaatgt gtttataata aacaatccat caacaatagc
    28921 cgttattctg ctgagcgggt tttcagaaga ttttaccgga atcatcgaat tgactccctg
    28981 ttgtgtatta ctaaaaccac ttaacgaact actcccaggc ggctgcaata gcactaaaat
    29041 tactaacaca acaaccaata ttacttgaaa aatacttaat accgctactg atattataga
    29101 tagagatgaa aataaaaatt ttatgagcat aaagtacata agtcaagaac atctgtcacc
    29161 caagtagctt ggaatctaag tatgtattta ctgcctacct tattttggtt agcatatgca
    29221 taaaactgtg ggcttaatat gtatcacatt ttaaaataca aagattatga accaaaaata
    29281 ggcgaacgtg ctttcatcgc aggtggttcg catatcatag gcaaggttga aataggaaaa
    29341 gatgcgagca tctggtttaa ttgtgtggtc agaggggacg ttggatcaat aaaaataggt
    29401 gatggaacaa atattcaaga tggaacggta attcatgtgg atagaaaccc aggtggtgat
    29461 acaactattg gcaatatggt aacagtggga cattttttgta tgttgcatgc atgtatggtg
    29521 catgataaag cgtttattgg tatgggctct accgtgatgg accatgcagt tgtggagcct
    29581 gaagctatgg tagctgctgg ctcgctagtg acacatggaa aagtaataaa aagtggggaa
    29641 atataagttc ttcaaaaaaa tgtcgaatga agaaattaaa catattacac aatcagcaca
    29701 aaattatatt atgctaatga aggaatataa aaattaaagt tcgcgtgagg ctaatttata
    29761 ctaccaaccc tattgacgga ctgcatagac agattagaaa attaactaaa actaagggct
    29821 catttattag cttgtacaaa caggtatatt gtgctatgaa aaaggcagac caaaattgga
    29881 ctatcctaat tgggttttaa ctatatctca acttgatatt ttatttcctg gtagattgaa
    29941 aattgagttg agttaaaaat gcggtttgac acactttttt gaacatttcc ttattgcctg
    30001 tttttacaat tttgaattga ttccttaggt tatgcaagag gtctattgag caaatccaat
    30061 aatatccttc atttctttta tattatgggt tgcaatttct gctgctttct ttgtaccttt
    30121 ttttaatatt tcacgtaaat ggaattggtc ttttaaaaga tcattcattc tctcacgtat
    30181 gggtgatata acactgataa ttaaatcagc taactctttt ttgaagtgct tcatgtcatg
    30241 tttgttcact tcttcacata ctttttccgc atttacatta ctaagcactg aataaatgtt
    30301 tatcaaattg cttatttctg gtcggcactc taaagtagca aaatcaaagc ctataattga
    30361 atctgttttt gctttttcta ttttttttgac aattaaatca tccgtatcat caaggttaat
    30421 gcacgaatat tctgaagggt cagatttact catcttgctt gtgctatctc ttaagctcat
    30481 tattcttgat gtgcaatcca aagttaagat ttcaggtacg atgaaataat ccagtttata
    30541 atgattgtta a
//
LOCUS       JAQZAU010000035            872 bp    dna     linear   ENV 17-MAR-2023
DEFINITION  MAG: Wolbachia pipientis isolate wEsol contig_29171, whole genome
            shotgun sequence.
ACCESSION   JAQZAU010000035 JAQZAU010000000
VERSION     JAQZAU010000035.1
DBLINK      BioProject: PRJNA929258
            BioSample: SAMN33142973
KEYWORDS    WGS; ENV; Metagenome Assembled Genome; MAG.
SOURCE      Wolbachia pipientis (insect metagenome)
  ORGANISM  Wolbachia pipientis
            Bacteria; Pseudomonadota; Alphaproteobacteria; Rickettsiales;
            Anaplasmataceae; Wolbachieae; Wolbachia.
REFERENCE   1  (bases 1 to 872)
  AUTHORS   Fiutek,N., Couger,B.M., Roy,S., de la Torre,J.R. and Connor,E.F.
  TITLE     Genomic assessment of the contribution of the Wolbachia
            endosymbiont of Eurosta solidaginis to gall-induction
  JOURNAL   International Journal Molecular Sciences
REFERENCE   2  (bases 1 to 872)
  AUTHORS   Fiutek,N., Couger,B.M., Roy,S., de la Torre,J.R. and Connor,E.F.
  TITLE     Direct Submission
  JOURNAL   Submitted (10-FEB-2023) Biology, San Francisco State University,
            1600 Holloway Ave, San Francisco, CA 94132, USA
```

```
COMMENT     ##Genome-Assembly-Data-START##
            Assembly Date          :: JUN-2019
            Assembly Method        :: FLYE v. 06-2019
            Genome Representation  :: Full
            Expected Final Version :: Yes
            Genome Coverage        :: 100.0x
            Sequencing Technology  :: Illumina HiSeq; PacBio Sequel
            ##Genome-Assembly-Data-END##
FEATURES             Location/Qualifiers
     source          1..872
                     /organism="Wolbachia pipientis"
                     /mol_type="genomic DNA"
                     /submitter_seqid="contig_29171"
                     /isolate="wEsol"
                     /isolation_source="galls on Solidago altissima"
                     /host="Eurosta solidaginis MN-2018-EFC"
                     /db_xref="taxon:955"
                     /environmental_sample
                     /country="USA: Cowling Arboretum, Carleton College,
                     Northfield, MN"
                     /lat_lon="44.48 N 93.13 W"
                     /altitude="280 m"
                     /collection_date="2018-11"
                     /metagenome_source="insect metagenome"
                     /note="metagenomic"
     gene            complement(163..447)
                     /locus_tag="wEsol_00276"
     CDS             complement(163..447)
                     /locus_tag="wEsol_00276"
                     /codon_start=1
                     /transl_table=11
                     /fullproduct="qcoverage 100 , hcoverage 100 , similarity
                     98.9 , identity 98.9 , evalue 2.51e-58 , alnlength 94 ,
                     Bacteria , Proteobacteria , AAS13858.1 hypothetical
                     protein WD_0102 [Wolbachia endosymbiont of Drosophila
                     melanogaster]"
                     /translation="MNLSWRGYRLIAADVSGMRLPSSGEIVSEFEPNGTTGTIGNLFV
                     DLCTSLICSARLAAWNIGEQTLAAEQLPEVITQMRLLNQEKLLFIYDRLQ"
                     /product="hypothetical protein"
                     /besthit="qcoverage 100 , hcoverage 100 , similarity 98.9
                     , identity 98.9 , evalue 2.51e-58 , alnlength 94 ,
                     Bacteria , Proteobacteria , AAS13858.1 hypothetical
                     protein WD_0102 [Wolbachia endosymbiont of Drosophila
                     melanogaster]"
ORIGIN
        1 tataactctt actttctgtc ctttcatttt atttttttagc ttttcatttt ctaatataaa
       61 atcaaaatct aattcgccag cgttcccata gcttactata atttcttttt tgcaagcgaa
      121 aaataaaatc tacttccaaa tcataatgct gctgaatgaa ttttactgaa gacgatcata
      181 gataaataat aatttctctt gatttaataa acgcatttga gtgataactt cgggtaattg
      241 ctctgccgcc aacgtttgtt cacctatatt ccaagctgca agacgagcac tacaaatcag
      301 cgaagtacac aaatcaacaa acaaattgcc aatggtgcct gttgttccat ttggttcaaa
      361 ctcggataca atttccccag agctgggtaa tctcatacca gaaacatctg ctgcaataag
      421 tctatagcct ctccaactca ggttcatcct gataggctgt ttggatgctt aaattcttga
      481 aagcctgtat ggcaaatctt atatcttgct ttagaaaaag cttgctttga gggtacatgt
      541 caatggctcc ataagttcac attctattcc caaacttttt tttaactaac tttaaaatca
      601 tagaaagtac tgtcgaaaaa gcttgttcgt gtaaaactca acacaatgga ttttctgaaa
      661 agttttcgag tatattatat ttttaatttg tagaataagg ttttttcctt tttgcatatt
      721 tcacctaaaa aggacacatt ttagacttct ttgttgaaat aaaacagaaa caaaagtgct
      781 gtcccttgca ctctttggat aatttaatga taaaaaattt agagagaaat tttatacact
      841 atcgctgata tttttcctta agttgatgcc at
```

```
//
LOCUS       JAQZAU010000036            849 bp    dna     linear   ENV 17-MAR-2023
DEFINITION  MAG: Wolbachia pipientis isolate wEsol contig_29338, whole genome
            shotgun sequence.
ACCESSION   JAQZAU010000036  JAQZAU010000000
VERSION     JAQZAU010000036.1
DBLINK      BioProject: PRJNA929258
            BioSample: SAMN33142973
KEYWORDS    WGS; ENV; Metagenome Assembled Genome; MAG.
SOURCE      Wolbachia pipientis (insect metagenome)
  ORGANISM  Wolbachia pipientis
            Bacteria; Pseudomonadota; Alphaproteobacteria; Rickettsiales;
            Anaplasmataceae; Wolbachieae; Wolbachia.
REFERENCE   1  (bases 1 to 849)
  AUTHORS   Fiutek,N., Couger,B.M., Roy,S., de la Torre,J.R. and Connor,E.F.
  TITLE     Genomic assessment of the contribution of the Wolbachia
            endosymbiont of Eurosta solidaginis to gall-induction
  JOURNAL   International Journal Molecular Sciences
REFERENCE   2  (bases 1 to 849)
  AUTHORS   Fiutek,N., Couger,B.M., Roy,S., de la Torre,J.R. and Connor,E.F.
  TITLE     Direct Submission
  JOURNAL   Submitted (10-FEB-2023) Biology, San Francisco State University,
            1600 Holloway Ave, San Francisco, CA 94132, USA
COMMENT     ##Genome-Assembly-Data-START##
            Assembly Date          :: JUN-2019
            Assembly Method        :: FLYE v. 06-2019
            Genome Representation  :: Full
            Expected Final Version :: Yes
            Genome Coverage        :: 100.0x
            Sequencing Technology  :: Illumina HiSeq; PacBio Sequel
            ##Genome-Assembly-Data-END##
FEATURES             Location/Qualifiers
     source          1..849
                     /organism="Wolbachia pipientis"
                     /mol_type="genomic DNA"
                     /submitter_seqid="contig_29338"
                     /isolate="wEsol"
                     /isolation_source="galls on Solidago altissima"
                     /host="Eurosta solidaginis MN-2018-EFC"
                     /db_xref="taxon:955"
                     /environmental_sample
                     /country="USA: Cowling Arboretum, Carleton College,
                     Northfield, MN"
                     /lat_lon="44.48 N 93.13 W"
                     /altitude="280 m"
                     /collection_date="2018-11"
                     /metagenome_source="insect metagenome"
                     /note="metagenomic"
     gene            complement(740..>849)
                     /locus_tag="wEsol_00277"
     CDS             complement(740..>849)
                     /locus_tag="wEsol_00277"
                     /codon_start=1
                     /transl_table=11
                     /product="hypothetical protein"
                     /translation="RKRVKYLLYRNCYKHRNYVGFCTGGMVAKEKAVKN"
ORIGIN
        1 aaactattac tcaaaaatgg tgcagaccca aattgacctt acccagaaaa agtagagaga
       61 aagttaagac gtttttatca tgagaataaa ttgtgcagga atgcaatagt taatagtaga
      121 atgtctacgt tatttgttat aaaagatttc tatgtattca aatatggcag ttctggtttg
```

```
      181 ttgagcgaga agtatcaata agcagctccc ttttaagtga acttttctgc aacagagtta
      241 tcgtaacaac agcccttatg gctcatgctg gaaatgatgt tttttgtatt caatagaaat
      301 tggtaatttt gagaggtata ctgtgaccct tgatcgctat gtaacagcag atttttatcg
      361 ggtttgcgtt tatcaacggc catcaataaa gaatctataa ctaattatca tgttactcat
      421 taaccaccat gcgtgaatat aggtcaatta ttgccgccaa caaccttctt tagtttttat
      481 gtaggtaatc ccaaacttta tttggctgat cagtagtaaa ttttgatcca atgtgttcgg
      541 agccacaact cggttgctta tttgctgttt ttattttgaa ccttcgtcta agcttggttt
      601 atgcttttct tgcataacac tttgtactgt ttttaatttt tgcctaaagc ttttattcag
      661 cgtgaatttt aggggctcca tatctgcatt tagaatgctt gatgtatttt ttgaatatct
      721 gctagaagcc cttcttttgc taattcttta ctgctttttc cttagcaacc ataccaccag
      781 tacaaaaccc tacatagttc ctgtgtttat agcaatttct atataaaaga tactttactc
      841 tttttcttt
//
LOCUS       JAQZAU010000037            3037 bp    dna     linear   ENV 17-MAR-2023
DEFINITION  MAG: Wolbachia pipientis isolate wEsol contig_29641, whole genome
            shotgun sequence.
ACCESSION   JAQZAU010000037  JAQZAU010000000
VERSION     JAQZAU010000037.1
DBLINK      BioProject: PRJNA929258
            BioSample: SAMN33142973
KEYWORDS    WGS; ENV; Metagenome Assembled Genome; MAG.
SOURCE      Wolbachia pipientis (insect metagenome)
  ORGANISM  Wolbachia pipientis
            Bacteria; Pseudomonadota; Alphaproteobacteria; Rickettsiales;
            Anaplasmataceae; Wolbachieae; Wolbachia.
REFERENCE   1  (bases 1 to 3037)
  AUTHORS   Fiutek,N., Couger,B.M., Roy,S., de la Torre,J.R. and Connor,E.F.
  TITLE     Genomic assessment of the contribution of the Wolbachia
            endosymbiont of Eurosta solidaginis to gall-induction
  JOURNAL   International Journal Molecular Sciences
REFERENCE   2  (bases 1 to 3037)
  AUTHORS   Fiutek,N., Couger,B.M., Roy,S., de la Torre,J.R. and Connor,E.F.
  TITLE     Direct Submission
  JOURNAL   Submitted (10-FEB-2023) Biology, San Francisco State University,
            1600 Holloway Ave, San Francisco, CA 94132, USA
COMMENT     ##Genome-Assembly-Data-START##
            Assembly Date          :: JUN-2019
            Assembly Method        :: FLYE v. 06-2019
            Genome Representation  :: Full
            Expected Final Version :: Yes
            Genome Coverage        :: 100.0x
            Sequencing Technology  :: Illumina HiSeq; PacBio Sequel
            ##Genome-Assembly-Data-END##
FEATURES             Location/Qualifiers
     source          1..3037
                     /organism="Wolbachia pipientis"
                     /mol_type="genomic DNA"
                     /submitter_seqid="contig_29641"
                     /isolate="wEsol"
                     /isolation_source="galls on Solidago altissima"
                     /host="Eurosta solidaginis MN-2018-EFC"
                     /db_xref="taxon:955"
                     /environmental_sample
                     /country="USA: Cowling Arboretum, Carleton College,
                     Northfield, MN"
                     /lat_lon="44.48 N 93.13 W"
                     /altitude="280 m"
                     /collection_date="2018-11"
                     /metagenome_source="insect metagenome"
                     /note="metagenomic"
```

```
gene            40..387
                /locus_tag="wEsol_00278"
CDS             40..387
                /locus_tag="wEsol_00278"
                /codon_start=1
                /transl_table=11
                /fullproduct="qcoverage 100 , hcoverage 100 , similarity
                100 , identity 100 , evalue 2.74e-74 , alnlength 115 ,
                Bacteria;Eukaryota , Proteobacteria;Arthropoda ,
                WP_155968342.1 MULTISPECIES: IS630 transposase-related
                protein [Wolbachia]"
                /cog="COG3415 COG3415 138 Transposase and inactivated
                derivatives"
                /pfam="HTH_Tnp_IS630 HTH_Tnp_1 HTH_23 HTH_28 HTH_29 HTH_7
                CENP-B_N Pfam-B_14738 HTH_8 HTH_17 HTH_24 Pfam-B_2106"
                /product="IS630 transposase-related protein"
                /translation="MAYSVDLRERAVSMIEKGKSKVEVAELLEIGIATLYRWLRKKAA
                GESLRPSKNGSFIRKIDPKILEEYVKKNPDHTLAEMKQNLGFGINSIWYRLKQLRITL
                KKSYTISRAQSRR"
                /besthit="qcoverage 100 , hcoverage 100 , similarity 100
                , identity 100 , evalue 2.74e-74 , alnlength 115 ,
                Bacteria;Eukaryota , Proteobacteria;Arthropoda ,
                WP_155968342.1 MULTISPECIES: IS630 transposase-related
                protein [Wolbachia]"
gene            587..898
                /locus_tag="wEsol_00279"
CDS             587..898
                /locus_tag="wEsol_00279"
                /codon_start=1
                /transl_table=11
                /fullproduct="qcoverage 100 , hcoverage 100 , similarity
                100 , identity 100 , evalue 1.60e-74 , alnlength 103 ,
                Bacteria , Proteobacteria , WP_155968470.1 transposase
                [Wolbachia pipientis]"
                /cog="COG3335 COG3335 132 Transposase and inactivated
                derivatives"
                /pfam="DDE_3 DDE_1"
                /product="transposase"
                /translation="MTFKGGCDKEVFNTWLEKMLLPKLPHGTTIVMDNATFHKTPKTK
                ELIDNARCRLLYLPTYSPDLNPIEHCWNTIKSRLRPLMHKYTDLQLLVGNTIMEIYHS
                F"
                /besthit="qcoverage 100 , hcoverage 100 , similarity 100
                , identity 100 , evalue 1.60e-74 , alnlength 103 ,
                Bacteria , Proteobacteria , WP_155968470.1 transposase
                [Wolbachia pipientis]"
gene            complement(935..1276)
                /locus_tag="wEsol_00280"
CDS             complement(935..1276)
                /locus_tag="wEsol_00280"
                /codon_start=1
                /transl_table=11
                /fullproduct="qcoverage 100 , hcoverage 100 , similarity
                100 , identity 100 , evalue 1.53e-79 , alnlength 113 ,
                Bacteria , Proteobacteria , AAS13810.1 transposase, IS5
                family, OrfB [Wolbachia endosymbiont of Drosophila
                melanogaster]"
                /cog="COG3293 COG3293 124 Transposase and inactivated
                derivatives"
                /pfam="DDE_Tnp_1_2 DDE_Tnp_1"
                /product="transposase, IS5 family, OrfB"
```

```
                /translation="MRTKYTKVKKMWADMGYQGRNLKNHIKEEYDIDIEIVKRPPCRF
                WVHKDTPPELLPTREQGFKVQPRRWVVERTFAWVNRNRRLSKEYDLLTTSTENFIYLA
                MSRVMLKREYA"
                /besthit="qcoverage 100 , hcoverage 100 , similarity 100
                , identity 100 , evalue 1.53e-79 , alnlength 113 ,
                Bacteria , Proteobacteria , AAS13810.1 transposase, IS5
                family, OrfB [Wolbachia endosymbiont of Drosophila
                melanogaster]"
gene            complement(1387..1767)
                /locus_tag="wEsol_00281"
CDS             complement(1387..1767)
                /locus_tag="wEsol_00281"
                /codon_start=1
                /transl_table=11
                /fullproduct="qcoverage 100 , hcoverage 100 , similarity
                100 , identity 100 , evalue 1.16e-87 , alnlength 126 ,
                Bacteria , Proteobacteria , AOV87106.1 transposase
                [Wolbachia endosymbiont of Drosophila incompta]"
                /cog="COG3293 COG3293 124 Transposase and inactivated
                derivatives"
                /pfam="DUF4096"
                /product="transposase"
                /translation="MRKKYPTDLSEREWARIEKHFRVSYKKGGRPPKYSKREILEAIF
                YVLRTGCQWRYLPNDFPLWKTVYEQFRQWKKQGIFEKMNYEITKYSRRKIGRNEQPSA
                CIVDSQSVKTTEKGGSKAMMEVKK"
                /besthit="qcoverage 100 , hcoverage 100 , similarity 100
                , identity 100 , evalue 1.16e-87 , alnlength 126 ,
                Bacteria , Proteobacteria , AOV87106.1 transposase
                [Wolbachia endosymbiont of Drosophila incompta]"
gene            complement(1854..2195)
                /locus_tag="wEsol_00282"
CDS             complement(1854..2195)
                /locus_tag="wEsol_00282"
                /codon_start=1
                /transl_table=11
                /fullproduct="qcoverage 100 , hcoverage 100 , similarity
                100 , identity 100 , evalue 1.53e-79 , alnlength 113 ,
                Bacteria , Proteobacteria , AAS13810.1 transposase, IS5
                family, OrfB [Wolbachia endosymbiont of Drosophila
                melanogaster]"
                /cog="COG3293 COG3293 124 Transposase and inactivated
                derivatives"
                /pfam="DDE_Tnp_1_2 DDE_Tnp_1"
                /product="transposase, IS5 family, OrfB"
                /translation="MRTKYTKVKKMWADMGYQGRNLKNHIKEEYDIDIEIVKRPPCRF
                WVHKDTPPELLPTREQGFKVQPRRWVVERTFAWVNRNRRLSKEYDLLTTSTENFIYLA
                MSRVMLKREYA"
                /besthit="qcoverage 100 , hcoverage 100 , similarity 100
                , identity 100 , evalue 1.53e-79 , alnlength 113 ,
                Bacteria , Proteobacteria , AAS13810.1 transposase, IS5
                family, OrfB [Wolbachia endosymbiont of Drosophila
                melanogaster]"
gene            complement(2306..2686)
                /locus_tag="wEsol_00283"
CDS             complement(2306..2686)
                /locus_tag="wEsol_00283"
                /codon_start=1
                /transl_table=11
                /fullproduct="qcoverage 100 , hcoverage 100 , similarity
                100 , identity 100 , evalue 1.16e-87 , alnlength 126 ,
```

Bacteria , Proteobacteria , AOV87106.1 transposase
[Wolbachia endosymbiont of Drosophila incompta]"
/cog="COG3293 COG3293 124 Transposase and inactivated
derivatives"
/pfam="DUF4096"
/product="transposase"
/translation="MRKKYPTDLSEREWARIEKHFRVSYKKGGRPPKYSKREILEAIF
YVLRTGCQWRYLPNDFPLWKTVYEQFRQWKKQGIFEKMNYEITKYSRRKIGRNEQPSA
CIVDSQSVKTTEKGGSKAMMEVKK"
/besthit="qcoverage 100 , hcoverage 100 , similarity 100
, identity 100 , evalue 1.16e-87 , alnlength 126 ,
Bacteria , Proteobacteria , AOV87106.1 transposase
[Wolbachia endosymbiont of Drosophila incompta]"

ORIGIN

```
        1 aagtaaatga taaaggtata ataagctatt tgagttataa tggcgtatag tgtggattta
       61 agggaaaggg cagtatcgat gatagaaaaa gggaagtcaa aggttgaggt ggcagagctt
      121 ttggagatag gaatagcaac tctgtaccgc tggttaagaa agaaagccgc tggtgaaagc
      181 ctaaggccat cgaaaaacgg cagctttatt cgaaaaatag acccaaaaat actcgaagaa
      241 tatgttaaaa agaatccaga tcatacgctg gcagagatga aacaaaatct tggatttgga
      301 ataaattcaa tttggtatag gctgaaacag ctaagaatta ctttaaaaaa aagttacact
      361 atatcaagag cgcaatcaag aagataggca gcgatttacc gataaaatct caaaaataga
      421 ccattccagc atcttatata tagatgaagc aggagttgat aataggttat accgagagta
      481 tggacgtgct ccaataggaa agaaaattta tgcagatatt ccaggaagaa aacgagagag
      541 aatcagtata ataggcgggt ggattggaaa gagatttatt gcaccaatga ctttcaaagg
      601 tgggtgtgac aaagaggtat tcaatacatg gttagagaag atgttattac ctaaattgcc
      661 acatggtact acaatagtta tggacaatgc cacattccat aaaactccca aaacaaagga
      721 gttaatagat aatgctagat gccgtttgct ctatctacca acatattcac cagatttgaa
      781 ccctatagag cattgttgga ataccatcaa aagccgcctc agacctttaa tgcataaata
      841 tacagactta caacttttgg ttggtaatac cataatggaa atttatcatt cattttagaa
      901 aatactataa gaggttgtcc ggaaactagt aaattcaagc atattccctc tttaacataa
      961 ccctactcat agctaggtat atgaaattct cagtggatgt tgtgagtaaa tcatactcct
     1021 tcgatagcct tctattccta ttaacccaag caaaagtcct ttctacaacc catcttcttg
     1081 gctgtacttt aaacccttgt tctcttgttg gtagtagctc aggtggcgta tctttgtgca
     1141 cccaaaatct acatggaggc cttttaacaa tttcaatatc tatgtcatat tcttccttta
     1201 tgtgattctt taaatttctt ccttggtatc ccatgtcagc ccacattttt ttaactttag
     1261 tatattttgt tctcatattg tttaatgcta ttttaatacc atctctatca ttttcgttag
     1321 cagcgcctac gtaacaacct agtataaaac cctgagtgtc tgtaattata tgcctttttc
     1381 taccctttac tttttacttt ccatcatagc ctttgatccc cccttttctg tagtctttac
     1441 agattgacta tctactatac aggcactcgg ctgctcattc cttcctattt ttcttctact
     1501 atattttgta atttcataat tcattttctc aaaaattccc tgcttcttcc attgcctgaa
     1561 ctgctcatac acagtcttcc atagcggaaa atcatttggt aaataccgcc attgacaccc
     1621 tgtacgcaat acatagaaaa ttgcttctaa tatttctctt ttgctatact ttggcggcct
     1681 tcctcctttc ttgtatgata ctctgaagtg tttttctatt cttgcccatt ccctttcgct
     1741 tagatctgtt ggatactttt ttctcatctt taccccgtac taaaatttca gatattatag
     1801 ccttttactt atttccggac aacctctaag aggttgtccg gaaactagta aattcaagca
     1861 tattccctct ttaacataac cctactcata gctaggtata tgaaattctc agtggatgtt
     1921 gtgagtaaat catactcctt cgatagcctt ctattcctat taacccaagc aaaagtcctt
     1981 tctacaaccc atcttcttgg ctgtacttta aacccttgtt ctcttgttgg tagtagctca
     2041 ggtggcgtat ctttgtgcac ccaaaatcta catggaggcc ttttaacaat ttcaatatct
     2101 atgtcatatt cttcctttat gtgattcttt aaatttcttc cttggtatcc catgtcagcc
     2161 cacatttttt taactttagt atattttgtt ctcatattgt ttaatgctat tttaatacca
     2221 tctctatcat tttcgttagc agcgcctacg taacaaccta gtataaaacc ctgagtgtct
     2281 gtaattatat gcctttttct accctttact tttttacttc catcatagcc tttgatcccc
     2341 ccttttctgt agtctttaca gattgactat ctactataca ggcactcggc tgctcattcc
     2401 ttcctatttt tcttctacta tattttgtaa tttcataatt cattttctca aaaattccct
     2461 gcttcttcca ttgcctgaac tgctcataca cagtcttcca tagcggaaaa tcatttggta
     2521 aataccgcca ttgacaccct gtacgcaata catagaaaat tgcttctaat atttctcttt
     2581 tgctatactt tggcggcctt cctcctttct tgtatgatac tctgaagtgt ttttctattc
     2641 ttgcccattc cctttcgctt agatctgttg gatacttttt tctcatcttt accccgtact
     2701 aaaatttcag atattatagc cttttactta tttccggaca acctctaagt ggtggtgcaa
```

```
     2761 ttattggagc agctatagca taccttgcag gagctgccac tttagctcct gctattgttg
     2821 ctgttgcagt atttattgca gccactgttg ttggtgcact ggtgggttac ggtattggaa
     2881 aatcttgtga aaaggtcagt gaagaaaggc aaaaggatcg ccatatgagc ataggtactg
     2941 tgattaagaa tgtccttact cctgagtgtt taaaatcaca atcgcaagtt catccataag
     3001 gtaggcattt agtattgact ttacaacaat agtcact
//
LOCUS       JAQZAU010000038           14144 bp    dna     linear   ENV 17-MAR-2023
DEFINITION  MAG: Wolbachia pipientis isolate wEsol contig_30611, whole genome
            shotgun sequence.
ACCESSION   JAQZAU010000038  JAQZAU010000000
VERSION     JAQZAU010000038.1
DBLINK      BioProject: PRJNA929258
            BioSample: SAMN33142973
KEYWORDS    WGS; ENV; Metagenome Assembled Genome; MAG.
SOURCE      Wolbachia pipientis (insect metagenome)
  ORGANISM  Wolbachia pipientis
            Bacteria; Pseudomonadota; Alphaproteobacteria; Rickettsiales;
            Anaplasmataceae; Wolbachieae; Wolbachia.
REFERENCE   1  (bases 1 to 14144)
  AUTHORS   Fiutek,N., Couger,B.M., Roy,S., de la Torre,J.R. and Connor,E.F.
  TITLE     Genomic assessment of the contribution of the Wolbachia
            endosymbiont of Eurosta solidaginis to gall-induction
  JOURNAL   International Journal Molecular Sciences
REFERENCE   2  (bases 1 to 14144)
  AUTHORS   Fiutek,N., Couger,B.M., Roy,S., de la Torre,J.R. and Connor,E.F.
  TITLE     Direct Submission
  JOURNAL   Submitted (10-FEB-2023) Biology, San Francisco State University,
            1600 Holloway Ave, San Francisco, CA 94132, USA
COMMENT     ##Genome-Assembly-Data-START##
            Assembly Date          :: JUN-2019
            Assembly Method        :: FLYE v. 06-2019
            Genome Representation  :: Full
            Expected Final Version :: Yes
            Genome Coverage        :: 100.0x
            Sequencing Technology  :: Illumina HiSeq; PacBio Sequel
            ##Genome-Assembly-Data-END##
FEATURES             Location/Qualifiers
     source          1..14144
                     /organism="Wolbachia pipientis"
                     /mol_type="genomic DNA"
                     /submitter_seqid="contig_30611"
                     /isolate="wEsol"
                     /isolation_source="galls on Solidago altissima"
                     /host="Eurosta solidaginis MN-2018-EFC"
                     /db_xref="taxon:955"
                     /environmental_sample
                     /country="USA: Cowling Arboretum, Carleton College,
                     Northfield, MN"
                     /lat_lon="44.48 N 93.13 W"
                     /altitude="280 m"
                     /collection_date="2018-11"
                     /metagenome_source="insect metagenome"
                     /note="metagenomic"
     gene            91..279
                     /locus_tag="wEsol_00284"
     CDS             91..279
                     /locus_tag="wEsol_00284"
                     /codon_start=1
                     /transl_table=11
                     /fullproduct="qcoverage 100 , hcoverage 100 , similarity
```

```
                     100 , identity 100 , evalue 1.00e-36 , alnlength 62 ,
                     Bacteria , Proteobacteria , AOV87641.1 hypothetical
                     protein WG67_03920 [Wolbachia endosymbiont of Drosophila
                     incompta]"
                     /translation="MRYLTVMSIIAWRIFFITSIARANPTLPCTTLLAEEEWKVLYVK
                     IHRKPCPSVVPNIKEAVS"
                     /product="hypothetical protein"
                     /besthit="qcoverage 100 , hcoverage 100 , similarity 100
                     , identity 100 , evalue 1.00e-36 , alnlength 62 ,
                     Bacteria , Proteobacteria , AOV87641.1 hypothetical
                     protein WG67_03920 [Wolbachia endosymbiont of Drosophila
                     incompta]"
     gene            567..2180
                     /locus_tag="wEsol_00285"
     CDS             567..2180
                     /locus_tag="wEsol_00285"
                     /codon_start=1
                     /transl_table=11
                     /fullproduct="qcoverage 100 , hcoverage 100 , similarity
                     100 , identity 100 , evalue 2.04e-309 , alnlength 537 ,
                     Bacteria , Proteobacteria , WP_141457261.1 MULTISPECIES:
                     hypothetical protein [unclassified Wolbachia]"
                     /translation="MNNPFKSENYKKNLDSLIKAVKLGNAEKQYNKTLRDLSHALEGF
                     TGEAKGIAEFVIYTDLNNYVEQPLGLFKTLMNRILGKNFPTKNSSISNFVNDELSSFT
                     LETAAKYTRILKKIDLLEQLEKDFRKNNILKDSLNLEQSEKSQGKDRSHEVSLEGRKQ
                     KEGTFELRKEEQPKEKLGGNRMRQIKRPTTAPPAAPDSRKVRFKSEISSDGESKAKST
                     SSLLKGANSFSMPDAKENPSEDSGYESSLDQGSEYEDISNYQEKKQKENPLYEQREDS
                     GYGSSLNEESVYKEISNYQEKKSKILIENSQSSKSPKEEPIYATVPKEHIEAKRERKQ
                     KQQPQSLVPPKPPQVPKKMFTTGQEVRQESKVVIKDKPAVPPKPKNLSRANQKVSTEP
                     KVVGVILKKSAERVPLESENQDNRKSAQKAAGKAIPKVAAATMKGGANREMGSKVKAL
                     KEKFEQNQAKGNANVQLAESKTTPAAQADIGVKKKQSHSFLTENKRSATKHVTAKSDP
                     KSHISKNSLLTHANVKEMVAQIEKGKGGR"
                     /product="hypothetical protein"
                     /besthit="qcoverage 100 , hcoverage 100 , similarity 100
                     , identity 100 , evalue 2.04e-309 , alnlength 537 ,
                     Bacteria , Proteobacteria , WP_141457261.1 MULTISPECIES:
                     hypothetical protein [unclassified Wolbachia]"
     gene            2358..2705
                     /locus_tag="wEsol_00286"
     CDS             2358..2705
                     /locus_tag="wEsol_00286"
                     /codon_start=1
                     /transl_table=11
                     /fullproduct="qcoverage 100 , hcoverage 100 , similarity
                     100 , identity 100 , evalue 7.86e-74 , alnlength 115 ,
                     Bacteria , Proteobacteria , WP_022626382.1 MULTISPECIES:
                     IS630 transposase-related protein [Wolbachia]"
                     /cog="COG3415 COG3415 138 Transposase and inactivated
                     derivatives"
                     /pfam="HTH_Tnp_IS630 HTH_Tnp_1 HTH_23 HTH_28 HTH_29 HTH_7
                     CENP-B_N Pfam-B_14738 HTH_17 HTH_32"
                     /product="IS630 transposase-related protein"
                     /translation="MVYSVDLREKAVSLVEKGKSKVEVAEVFEIGIATLYRWLKKKAT
                     GQGLKAVKSTGFIRKIDPEILKEYVKKHPDHTLAEMKQNLGFGISAIWYRLRQLKITF
                     KKSHILSRAQSRR"
                     /besthit="qcoverage 100 , hcoverage 100 , similarity 100
                     , identity 100 , evalue 7.86e-74 , alnlength 115 ,
                     Bacteria , Proteobacteria , WP_022626382.1 MULTISPECIES:
                     IS630 transposase-related protein [Wolbachia]"
     gene            2873..3208
```

```
                     /locus_tag="wEsol_00287"
CDS                  2873..3208
                     /locus_tag="wEsol_00287"
                     /codon_start=1
                     /transl_table=11
                     /fullproduct="qcoverage 100 , hcoverage 100 , similarity
                     100 , identity 100 , evalue 1.17e-82 , alnlength 111 ,
                     Bacteria , Proteobacteria , WP_049973597.1 MULTISPECIES:
                     transposase [Wolbachia]"
                     /cog="COG3335 COG3335 132 Transposase and inactivated
                     derivatives"
                     /pfam="DDE_3 DDE_1"
                     /product="transposase"
                     /translation="MDEKFIAPMTFTGGCNKDIFNVWLERRLLPQLQRDTTIVMDNAT
                     FHKTPKTKSLIESFGCHLLYLPTYSPDLNPIEHCWHTIKSCLRPLMHQCTDLHLLVGN
                     TILDVYHSF"
                     /besthit="qcoverage 100 , hcoverage 100 , similarity 100
                     , identity 100 , evalue 1.17e-82 , alnlength 111 ,
                     Bacteria , Proteobacteria , WP_049973597.1 MULTISPECIES:
                     transposase [Wolbachia]"
gene                 complement(3625..5055)
                     /locus_tag="wEsol_00288"
CDS                  complement(3625..5055)
                     /locus_tag="wEsol_00288"
                     /codon_start=1
                     /transl_table=11
                     /fullproduct="qcoverage 100 , hcoverage 100 , similarity
                     100 , identity 100 , evalue 0.0 , alnlength 476 ,
                     Bacteria , Proteobacteria , WP_012673305.1 MULTISPECIES:
                     2-polyprenylphenol 6-hydroxylase [Wolbachia]"
                     /cog="AarF COG0661 517 Predicted unusual protein kinase"
                     /pfam="ABC1 RIO1 Pfam-B_14148 APH"
                     /tigr="TIGR01982 UbiB 436 2-polyprenylphenol
                     6-hydroxylase"
                     /product="2-polyprenylphenol 6-hydroxylase"
                     /translation="MIQNILRLLHITTVLTRYNILPYLLPPSKKSINKIQGHKLKCAL
                     EKLGPVFIKFGQSISSRTDVLNEDITNNLLLICDRLPSFSHKIAVKTIESEFNCKLSD
                     IFSSFSEKPIAAASISQVHRAVTTEGKEVAVKVLRPNIEKTFSRDIKMLSWLAEIAEK
                     FSEQSKRLRPIELVKTFAEICRLELDLRFEAAHSSELKENTKNDRGFYVPEIDWNRTA
                     KKVLTLEWIEATPIYEVEKLNNHKQIAINLIESFCNQVYRDCFFHADMHPGNLMVDSN
                     NNIIALDCGIMGRIDRETCYYVIEILKGFLNRDYDHVAKMHFKAGYVSSQHRNFVTAC
                     RAIGEPIIGQPIQKISFASLLAQLLKITGDFDMKVQTQLLLLQKTMILLEGTCRKIYP
                     EINMWKVVEAWISSQHESKIGYREKIRSSYPVKTIQGIFSLIEKLNLIADKKLENIQI
                     KNKSNGKVYFLLWFVIIILIVKLLIS"
                     /besthit="qcoverage 100 , hcoverage 100 , similarity 100
                     , identity 100 , evalue 0.0 , alnlength 476 , Bacteria ,
                     Proteobacteria , WP_012673305.1 MULTISPECIES:
                     2-polyprenylphenol 6-hydroxylase [Wolbachia]"
gene                 complement(5052..5747)
                     /locus_tag="wEsol_00289"
CDS                  complement(5052..5747)
                     /locus_tag="wEsol_00289"
                     /codon_start=1
                     /transl_table=11
                     /fullproduct="qcoverage 100 , hcoverage 93.9024390243902
                     , similarity 100 , identity 99.6 , evalue 1.01e-154 ,
                     alnlength 231 , Bacteria , Proteobacteria ,
                     WP_015589282.1 MULTISPECIES: metal ABC transporter
                     ATP-binding protein [unclassified Wolbachia]"
                     /cog="ZnuC COG1121 254 ABC-type Mn/Zn transport systems,
```

```
                     ATPase component"
                     /pfam="Arch_ATPase AAA_15 IstB_IS21 Pfam-B_16450 SMC_N
                     AAA_21 ABC_tran AAA_21 Pfam-B_17981 Pfam-B_10459 AAA_15"
                     /tigr="TIGR03265 PhnT2 353 putative
                     2-aminoethylphosphonate ABC transporter, ATP-binding
                     protein"
                     /product="metal ABC
                     transporter ATP-binding protein"
                     /translation="MNNVNNRVLKIENLALTYDGKRILDNINMFMERGDIITILGPNG
                     GGKTSLVKAIAGINKSCTGNIVFADNIKIGYMPQNFSISNLMPITVEYFLLNSSLKRL
                     KKNQSIITETIELVSIGNILKNQVLEISAGQTQLLLLARCLIAEPDLIILDEPVSAMD
                     INARAKFYDIINKIAKKRLVSILMTSHDLNSALPSSDYIICINNTIYCQGKPDEIMKN
                     RTLNEIFSSYAAK"
                     /besthit="qcoverage 100 , hcoverage 93.9024390243902 ,
                     similarity 100 , identity 99.6 , evalue 1.01e-154 ,
                     alnlength 231 , Bacteria , Proteobacteria ,
                     WP_015589282.1 MULTISPECIES: metal ABC transporter
                     ATP-binding protein [unclassified Wolbachia]"
     gene            5784..6665
                     /locus_tag="wEsol_00290"
     CDS             5784..6665
                     /locus_tag="wEsol_00290"
                     /codon_start=1
                     /transl_table=11
                     /pfam="SBP_bac_9"
                     /fullproduct="qcoverage 100 , hcoverage 100 , similarity
                     100 , identity 100 , evalue 1.16e-201 , alnlength 293 ,
                     Bacteria , Proteobacteria , WP_012673303.1 MULTISPECIES:
                     zinc ABC transporter substrate-binding protein
                     [Wolbachia]"
                     /translation="MRHLLTFFLLLSFTLYYNIAFSSNLKVVATIKPIHSLVASITEG
                     ILEPELLGYATSAHNYILKPSDASNLESSDVIFYVDDNLETFVQTFAKNNKELVQLSK
                     AINLLPTRPHSFSRKITHIKDEKDLHIWLSPENAKSMILSVSATLSNIDKENSHRYNS
                     NATKAIKKIDQEVKEIMKELNDFKNQKYIVTHDAYQYFEKYFGLNHPSAILSIEEDSY
                     IGMRSLMKLKKIMKEENVKCIFAHSQEDSIKPNALSNDAKMVVLDPIGSDIEPGKDAY
                     LAIINNIAQNFKSCFVE"
                     /product="zinc ABC
                     transporter substrate-binding protein"
                     /besthit="qcoverage 100 , hcoverage 100 , similarity 100
                     , identity 100 , evalue 1.16e-201 , alnlength 293 ,
                     Bacteria , Proteobacteria , WP_012673303.1 MULTISPECIES:
                     zinc ABC transporter substrate-binding protein
                     [Wolbachia]"
     gene            complement(6722..6997)
                     /locus_tag="wEsol_00291"
     CDS             complement(6722..6997)
                     /locus_tag="wEsol_00291"
                     /codon_start=1
                     /transl_table=11
                     /fullproduct="qcoverage 100 , hcoverage 100 , similarity
                     100 , identity 100 , evalue 4.43e-53 , alnlength 91 ,
                     Bacteria , Proteobacteria , WP_010082632.1 MULTISPECIES:
                     XRE family transcriptional regulator [Wolbachia]"
                     /cog="COG5606 COG5606 91 Uncharacterized conserved small
                     protein"
                     /pfam="HTH_37"
                     /product="XRE family
                     transcriptional regulator"
                     /translation="MQNLQLSYPIIVELGDVKDTKLKLIKVIAQLISKKYTTQKKAAN
                     ALKIDQPKVSQINRSKIEGFSLEYLLNLLIVLGQDINVRIKHNSKPA"
```

```
                     /besthit="qcoverage 100 , hcoverage 100 , similarity 100
                     , identity 100 , evalue 4.43e-53 , alnlength 91 ,
                     Bacteria , Proteobacteria , WP_010082632.1 MULTISPECIES:
                     XRE family transcriptional regulator [Wolbachia]"
gene                 complement(7023..7223)
                     /locus_tag="wEsol_00292"
CDS                  complement(7023..7223)
                     /locus_tag="wEsol_00292"
                     /codon_start=1
                     /transl_table=11
                     /fullproduct="qcoverage 100 , hcoverage 100 , similarity
                     100 , identity 100 , evalue 6.32e-35 , alnlength 66 ,
                     Bacteria , Proteobacteria , WP_015589280.1 MULTISPECIES:
                     50S ribosomal protein L33 [Wolbachia]"
                     /cog="RpmG COG0267 50 Ribosomal protein L33"
                     /pfam="Ribosomal_L33"
                     /tigr="TIGR01023 rpmG_bact 56 ribosomal protein L33"
                     /product="50S ribosomal
                     protein L33"
                     /translation="MAKKNASLLVKLVSTAIKTTKTGEEKSTGYFYVKKRNPKKLTKK
                     LEFRKYDPVVRRHVLFKEEKLK"
                     /besthit="qcoverage 100 , hcoverage 100 , similarity 100
                     , identity 100 , evalue 6.32e-35 , alnlength 66 ,
                     Bacteria , Proteobacteria , WP_015589280.1 MULTISPECIES:
                     50S ribosomal protein L33 [Wolbachia]"
gene                 7373..9145
                     /locus_tag="wEsol_00293"
CDS                  7373..9145
                     /locus_tag="wEsol_00293"
                     /codon_start=1
                     /transl_table=11
                     /fullproduct="qcoverage 100 , hcoverage 100 , similarity
                     100 , identity 100 , evalue 0.0 , alnlength 590 ,
                     Bacteria , Proteobacteria , WP_007549119.1 MULTISPECIES:
                     Rne/Rng family ribonuclease [unclassified Wolbachia]"
                     /cog="CafA COG1530 487 Ribonucleases G and E"
                     /pfam="S1 RNase_E_G"
                     /tigr="TIGR00757 RNaseEG 415 ribonuclease, Rne/Rng
                     family"
                     /product="Rne/Rng family
                     ribonuclease"
                     /translation="MVNSGKRLLLIENSTCSNEVRVALSVNNKVVEFEQEFKEKKQLR
                     GNIYVAYIKRIEPSLQAVFIEYGKNKQGFLSFSEISLDYFNIPEKEKETIFESYSNDD
                     YTEKGNDTIANVSSDSAASKNVNESGSGFVREVPLYKKYKLQDVISVNQKVLVQLTKE
                     ERGNKGASFTTYITLVGRYCVFMPNSMSKGGVSRRIEDANVRKQLKEILSSINLPKRS
                     GLIVRTVGSGKNKKEIEQDYNYLSSLWQSIQKNAFSVNVPSLIYNEADVIMRSVRDFC
                     SDGIEVIVSGKEAFEAVRQYANNALKGSKLRYRLYRGYAPIFTYYGIEDQISELYSNR
                     VELPSGGSLIITLTEAFVSIDVNSGKMTGEDSIEETAYRTNMEAVPEISRQVNLRGLS
                     GLIVVDFIDMLKYQYCRAVESAIKQAFKDDKAKVQFSYINDFGLMVFSRQRIKPNIQE
                     INTTECLHCKGTGRVKSNEVIVSSILRDLQHIANKNRNKSFDLVAHSAVIAHIFNNKR
                     NTVSTIEKEFNITLNVSIDNSLDVNTFVLKQGDDVNLNGYKPLQNSGYQIGDSSNEEA
                     DNSNKLRGNFWLTKWLSRLLSSNN"
                     /besthit="qcoverage 100 , hcoverage 100 , similarity 100
                     , identity 100 , evalue 0.0 , alnlength 590 , Bacteria ,
                     Proteobacteria , WP_007549119.1 MULTISPECIES: Rne/Rng
                     family ribonuclease [unclassified Wolbachia]"
gene                 9232..11154
                     /locus_tag="wEsol_00294"
CDS                  9232..11154
                     /locus_tag="wEsol_00294"
```

```
                     /codon_start=1
                     /transl_table=11
                     /fullproduct="qcoverage 100 , hcoverage 100 , similarity
                     100 , identity 100 , evalue 0.0 , alnlength 640 ,
                     Bacteria;Eukaryota , Proteobacteria;Arthropoda ,
                     WP_012673300.1 MULTISPECIES: molecular chaperone DnaK
                     [unclassified Wolbachia]"
                     /cog="DnaK COG0443 579 Molecular chaperone"
                     /pfam="HSP70 MreB_Mbl"
                     /tigr="TIGR02350 prok_dnaK 596 chaperone protein DnaK"
                     /product="molecular
                     chaperone DnaK"
                     /translation="MGRAIGIDLGTTNSCVAIMQGKDTKVIENKEGARTTPSIVALTS
                     SGERLIGAPAKRQATTNASNTFFATKRLIGRQYSDPEMKNLSVPYKVFAAKSGDAWVK
                     TTDNKEYSPSQIGAFILQNMKEAAEAYLGEEVKDAVITVPAYFNDSQRQATKDAGKIA
                     GLNVLRIVNEPTAAALAYGLDKKHGHTIVVYDLGGGTFDVSILEIGDGVFEVKATNGD
                     THLGGEDFDNGVVSYLLDEFKKSNGIDLKNDPMAMQRIKEAAEKAKIELSSAMETEIN
                     LPFITADASGPKHLNMKLTRAKLESLVNDLIERTMAPCKKALEDAGLSASQIGEVVLV
                     GGMTRMPKVIEKVKEFFGKDPHRGVNPDEVVAIGAAIQAGIIQGDVRDVLLLDVTPLS
                     LGIETLGGVFTPLIERNTTIPTKKSQVFSTAEDNQTAVTIKVHQGERKLAVDNKLLGQ
                     FSLEGIPPAPRGRPQIEVTFDIDANGIAHVSAKDKATGKEQKIRIQSSGGLSDDEINR
                     MVKEAEEKAQEDEKRKKFIEVKNQADSLVHSTEKSLTEYGDKVSPEDRSAIENAVNEL
                     KEVSKSDNIDDADSIQQKVTNLSQLSMKLGEAMYQASQQNSAENGFSSEGNPNDKEEK
                     VVDSDYQDIDNKEENK"
                     /besthit="qcoverage 100 , hcoverage 100 , similarity 100
                     , identity 100 , evalue 0.0 , alnlength 640 ,
                     Bacteria;Eukaryota , Proteobacteria;Arthropoda ,
                     WP_012673300.1 MULTISPECIES: molecular chaperone DnaK
                     [unclassified Wolbachia]"
     gene            11225..11509
                     /locus_tag="wEsol_00295"
     CDS             11225..11509
                     /locus_tag="wEsol_00295"
                     /codon_start=1
                     /transl_table=11
                     /fullproduct="qcoverage 100 , hcoverage 100 , similarity
                     100 , identity 100 , evalue 2.94e-57 , alnlength 94 ,
                     Bacteria , Proteobacteria , WP_007549117.1 MULTISPECIES:
                     BolA family transcriptional regulator [Wolbachia]"
                     /cog="BolA COG0271 90 Stress-induced morphogen (activity
                     unknown)"
                     /pfam="BolA"
                     /product="BolA family
                     transcriptional regulator"
                     /translation="MDIIKTIEEKIHDSIDVIDINIIDESAKHADHYFASSSTLPSHI
                     KLILISDSFIGMNTLKRHKLIYELLKSEIELIHAISLHLYTQNEYNLKNK"
                     /besthit="qcoverage 100 , hcoverage 100 , similarity 100
                     , identity 100 , evalue 2.94e-57 , alnlength 94 ,
                     Bacteria , Proteobacteria , WP_007549117.1 MULTISPECIES:
                     BolA family transcriptional regulator [Wolbachia]"
     gene            11517..12758
                     /locus_tag="wEsol_00296"
     CDS             11517..12758
                     /locus_tag="wEsol_00296"
                     /codon_start=1
                     /transl_table=11
                     /fullproduct="qcoverage 100 , hcoverage 100 , similarity
                     100 , identity 100 , evalue 7.91e-306 , alnlength 413 ,
                     Bacteria , Proteobacteria , WP_012673299.1 MULTISPECIES:
                     cystathionine beta-lyase [unclassified Wolbachia]"
```

```
                     /cog="MetC COG0626 396 Cystathionine
                     beta-lyases/cystathionine gamma-synthases"
                     /pfam="Cys_Met_Meta_PP Aminotran_1_2 Aminotran_5"
                     /tigr="TIGR01324 cysta_beta_ly_B 377 cystathionine
                     beta-lyase"
                     /product="cystathionine
                     beta-lyase"
                     /translation="MKEESLLVKAGRKFKDYKGSMNPPVYHSSTILFPTYKDYLNAAN
                     GESIYDVINDDVARDYSYSNVGTPTVHYFSNALAEIEGSGQALIFPSGLFALTFAILT
                     FTKAGSHVLIQDNSYYRLKRFAENELPNRGIEVTFYDPTQDITDLIQSNTSLIMIETP
                     GSVTFEISNIEHIVKVAKERGIVTVCDNSWATPLLFKPLDYGIDVALYAVTKYLAGHS
                     DLLMGTMIAEGEIFKLLYESYKNYGVTIQSHDCYLAHRGLRTLHTRMKKYQNTAMEVA
                     KWLEAQQKVRKVLYPALPSHPQHELWKSYFKGASGVFSIVLGREYSCEELSCMVDHMK
                     IFGIGASWGGCDSLILPIDRRSMSRSVMNSDYGGSFIRIFCGLEDPEDLIFDLNDALK
                     RLPCLDFQNDEVGHETERITA"
                     /besthit="qcoverage 100 , hcoverage 100 , similarity 100
                     , identity 100 , evalue 7.91e-306 , alnlength 413 ,
                     Bacteria , Proteobacteria , WP_012673299.1 MULTISPECIES:
                     cystathionine beta-lyase [unclassified Wolbachia]"
     gene            12733..>14144
                     /locus_tag="wEsol_00297"
     CDS             12733..>14144
                     /locus_tag="wEsol_00297"
                     /codon_start=1
                     /transl_table=11
                     /fullproduct="qcoverage 100 , hcoverage 97.7130977130977
                     , similarity 100 , identity 100 , evalue 0.0 , alnlength
                     470 , Bacteria , Proteobacteria , WP_015589277.1
                     MULTISPECIES:
                     UDP-N-acetylmuramoyl-L-alanyl-D-glutamate--2,6-
                     diaminopimelate ligase [unclassified Wolbachia]"
                     /cog="MurE COG0769 475 UDP-N-acetylmuramyl tripeptide
                     synthase"
                     /pfam="Mur_ligase_M Mur_ligase_C"
                     /tigr="TIGR01085 murE 472 UDP-N-acetylmuramyl-tripeptide
                     synthetase"
                     /product="
                     UDP-N-acetylmuramoyl-L-alanyl-D-glutamate--2,6-
                     diaminopimelate ligase"
                     /translation="MKLRELLHNIIDVNFDIEIKGVTCNPKKIKEGYLFVCVSERDRI
                     YIDQILSCGAKAIIASDCITEASSLGIIPTRDAGICIFHPNPQEIYSEIVSRFYQFKQ
                     PKYVAAVTGTNGKTSVVEFCRQIWQNSGYNAASIGTLGTCINNGRKGNSNSLTTPGAE
                     DLYATLRGISVEHLVLESSSHGIDQYRIHGLKLSAAAFTNFSQDHLDYHKSLGGYLET
                     KKRLFYEVLPEGKTAILNADIDEYGALLKIAEKRSNKVVTYGKKGSDITLLEQIPTPS
                     GQYLTVKVDNQIYNTFFPVLGKFQAYNLLCAIGISGLNYREICVDKLIFPSGRMEKVK
                     PFAFVDYAHTPSALKQALLSLKWHFNKKIVLVFGCGGNRDQTKRAEMGKVAQIYADKV
                     IITDDNPRDENPAEIRRGILLHCPDALEIEDRREAIEKGVDIAYNEGMILLVAGKGHE
                     GYQIVSGQTFEFSDVEVIKN"
                     /besthit="qcoverage 100 , hcoverage 97.7130977130977 ,
                     similarity 100 , identity 100 , evalue 0.0 , alnlength
                     470 , Bacteria , Proteobacteria , WP_015589277.1
                     MULTISPECIES:
                     UDP-N-acetylmuramoyl-L-alanyl-D-glutamate--2,6-
                     diaminopimelate ligase [unclassified Wolbachia]"
ORIGIN
        1 ttgccttaga tggaaaatag agatattaca taaaatttta aattcggtct caaagttgaa
       61 gaatgtaggc ttggaatagc agaaagacta atgagatatc taacagttat gagtattatt
      121 gcttggagga tcttctttat tacatcaatt gcaagagcta acccaacatt accatgtact
      181 accttattag ctgaagaaga atggaaagtt ttatacgtta aaatacacag aaaaccatgt
      241 ccaagtgtag ttcccaacat aaaagaagct gtttcatgaa cttggaggcc atttagcaag
```

301 aaaaaatgac ccaaaaccag gaccaattac tctttggaaa ggatataaac gtctctttga
361 tttagcagaa ggatggcgac ttgctcacga acctcatatt tgtgggtaat agtaagttgt
421 taggggggatc tggagatacc gcggcggtat gacgtaggga aatcttactt attatagcta
481 tagttctaca tgaattacaa aagtaatatt tactagatct taatatttta gatctatatt
541 ttaataaata atgagctgta attagtatga ataatccatt taaaagtgag aactataaaa
601 aaaatctcga ttctctgatc aaagctgtaa agctaggaaa tgcagagaaa caatataaca
661 aaacgttaag ggatttatct catgcattag aaggttttac aggtgaggca aaaggtatag
721 ctgagtttgt tatatatact gacttgaaca attatgtaga acagccatta ggtcttttta
781 aaacattaat gaatagaatt ttgggaaaaa attttccaac caagaacagt agtataagta
841 attttgtaaa tgatgagttg tcaagtttca ctttagaaac agcagctaag tatacaagaa
901 ttttaaaaaa aattgatctc ttggaacaat tagaaaaaga tttcagaaaa aataatattt
961 taaaagattc tttaaattta gaacaaagtg aaaagagtca aggaaaagat agatcacacg
1021 aagtgagttt ggaaggtaga aagcaaaagg aaggtacttt tgagctgcga aaagaagaac
1081 agccgaaaga aaaattaggt ggtaatcgca tgaggcaaat aaagcgacca acaacagcac
1141 ctccggctgc tccagattcg aggaaggtac gctttaaatc agaaatatcc tcagatggag
1201 aaagcaaggc aaagtctaca tcttctttac taaaaggagc taatagtttt agtatgcctg
1261 atgctaagga aaatccttct gaggattcag gctatgaaag ttcattagat caggggtctg
1321 aatatgaaga catatcaaat tatcaagaga aaaagcaaaa ggaaaatcct ttatatgagc
1381 agagagaaga ttcaggctat ggaagttcat taaatgaaga gtctgtatac aaagagatat
1441 caaattatca agagaagaag agtaaaattt taatagaaaa ttcgcagagt tcaaaatcgc
1501 ccaaagaaga gccaatttat gcaacagttc ctaaagaaca tatagaagca aaaagagaac
1561 gtaagcagaa acaacaacct caatccctcg taccaccaaa accaccgcaa gtgccaaaaa
1621 aaatgtttac tactggtcaa gaagttagac aagaatcgaa agtggtaatc aaagataaac
1681 ctgcagtacc gccaaagcct aaaaatctaa gccgtgctaa tcaaaaagta agcacagagc
1741 caaaagtagt aggagtaatt cttaaaaaat cagctgaaag agtaccacta gaatctgaaa
1801 atcaagacaa taggaaatca gcacaaaaag ctgctggtaa ggcgatacca aaagtagcag
1861 cagctactat gaaaggagga gctaatcgcg agatgggcag taaagtaaag gcattaaaag
1921 aaaaatttga gcaaaatcaa gcaaaaggaa atgctaatgt acagcttgct gaaagcaaaa
1981 ccactccagc agcacaagcg gatatcggtg tgaagaaaaa acaatctcat tcatttctaa
2041 cagagaataa aagatctgca acgaaacacg taacagcaaa atctgatcca aaatctcata
2101 tcagcaaaaa ttcccttctc acgcatgcaa atgtaaagga aatggttgca caaattgaga
2161 aaggaaaagg aggaagatag taacatttgg gaaaaaaaca tatgtgtcaa gtcaagagat
2221 tgtgagcaaa tttaggaaat tatagctaaa ctgacttaga tttaccgaca atttgaaaaa
2281 ccgtcattcc gctaactata gtattttcaa aagcgaatga taaaggtata atatgttttt
2341 ttaaagtagg gaaagcgatg gtatatagtg tagatttaag ggaaaaagca gtgtctttgg
2401 tagaaaaagg aaagtcaaag gttgaagttg cagaggtttt tgagataggg atagcaacac
2461 tatatagatg gctaaaaaag aaagctactg gccaaggtct caaagcggta aaaagtactg
2521 gttttattcg aaaaatagat ccagaaatac tcaaagaata tgtcaaaaaa cacccagatc
2581 acacactagc agaaatgaaa caaaaccttg gctttgggat aagtgcaatt tggtatagat
2641 taagacagct aaaaataacc tttaaaaaaa gtcacattct atcaagagcg caatcaagaa
2701 gataggcagc aatttatcga taaagtctca aaaatagacc actgtagcat tttatatata
2761 gatgaagcag gagttgataa tagactatac cgagaatatg cacgagctcc aagaggagaa
2821 aaaatatatg ctgatgttcc aggaaaaaag cgggaaagag tgagtataat aggtggatga
2881 gaagtttatt gcaccaatga ccttcacagg agggtgtaat aaagatatat ttaatgtatg
2941 gttagagcga agattacttc cacaattaca gcgtgataca accatagtta tggataatgc
3001 tacattccat aaaactccca aaacaaagtc attaatagag tcttttggct gtcatttgct
3061 ttatcttcca acatattcac ctgatctaaa tccgatagag cattgttggc acaccatcaa
3121 aagttgcctc agacctctaa tgcatcaatg tacagactta caccttttag ttggtaatac
3181 cattttagac gtttatcatt cgttttagaa aatactatag ttgaaatagc gcagttactc
3241 accccataaa tttggtgctc tctttcaatc aaaggtgtct tgctgcttga aatgttttct
3301 ttgtaaaaat ttggcttatc tttatatcat ctcattcaaa cagcattaac ataacttatg
3361 cctttttttgg atgtagaaat tttctaggaa ttgtcttttt gaattctatg gtattttgtt
3421 taacattgaa acaggaggcc agtgtcacgc actggcatga cacccatttg ttcctatagt
3481 tgtcttttct cgtctacctt attttgccac tctgctgaac agatacataa agaaagtgga
3541 aaattggaca ttctcaactc ggctcctgca gattaaaaat taagttgaat aaaaattgga
3601 tttgacgcag tttttttccca tagtttaaga aattaaaagt ttaacgatta gaattataat
3661 tacaaaccac aataaaaaat agacttttcc atttgattta tttttaattt ggatgttttc
3721 taattttttta tcagctatta ggttcaattt ttctataagg ctaaatatcc cttggattgt
3781 ttttacagga taagaacttc taatttttttc cctataccct attttacttt catgttgact
3841 gcttatccac gcttcaacta ctttccacat attgatttct gggtagatct ttctacatgt

3901 cccttccagt aaaatcatag ttttctgcag caataacaat tgtgtttgaa ctttcatatc
3961 aaaatcacca gttattttta atagctgagc aagtaaacta gcaaatgaaa tcttctgtat
4021 aggctgccca ataatgggtt cacctattgc cctgcaagct gtgacaaaat ttctatgctg
4081 tgatgaaacg taaccagctt taaagtgcat ttttgcaacg tgatcataat cccgatttaa
4141 aaagcctttg agtatctcta taacgtaata gcatgtctca cgatctattc tacccatgat
4201 tccacaatcc agagcaataa tattgttatt actatcaacc attagatttc caggatgcat
4261 atcagcatga aaaaaacaat ccctgtatac ctgattacaa aacgattcta taagattgat
4321 agctatttgc ttatgattat tcagtttttc aacttcgtat attggcgttg cttctatcca
4381 ttctaatgtt aaaacctttt ttgcagttct attccaatct atttcaggta cataaaaacc
4441 tctgtcattt tttgtgtttt cctttagttc cgaagagtga gcagcttcaa aacgtagatc
4501 taactctaat cggcaaattt cagcaaaagt tttaactaat tcaattggcc tcagcctttt
4561 tgattgttcg ctaaattttt ctgcaatttc tgcaagccaa gaaagcattt ttatatctct
4621 tgagaatgtt ttctcaatat ttggccttaa aaccttcaca gcaacttcct taccctcagt
4681 tgtaaccgct ctatgcacct gagaaatcga tgctgctgca attggctttt cagaaaagct
4741 tgaaaaaatg tcgctcaatt tacaattaaa ctcactttct atagttttaa ctgctatttt
4801 atgtgaaaat gatggcaatc tatcacatat caataacaag ttatttgtta tatcttcatt
4861 taaaacatca gtacgtgatg agatagactg cccaaactta atgaacaccg gacctaattt
4921 ttcaagagca cactttagct tgtggccttg tattttattt attgattttt ttgatggtgg
4981 aagtaaataa ggtaatatat tgtaacgcgt cagcaccgta gttatgtgca gaagacgcaa
5041 aatattttga atcatttcgc tgcgtaactg ctaaatattt catttagggt tctatttttc
5101 ataatctcat caggcttgcc ttggcaatag atagtattat ttatacaaat tatgtaatct
5161 gaggatggta gagcagagtt aagatcatga gaagtcataa gaatcgatac caatcgtttt
5221 tttgctattt tatttataat atcataaaat ttagcccgcg cgttaatatc cattgcacta
5281 actggttcat ctagaatgat caagtcaggc tctgcaatta aacaacgtgc aagtagcaat
5341 aattgtgttt gccctgcaga aatttctaac acttgattct tcaaaatgtt accgatacta
5401 accaactcta tcgtttctgt aataatggat tgatttttct ttagtctttt caaggagcta
5461 tttaaaagga aatattcaac tgttattggc atcaaattac taatactaaa atttgcggc
5521 atatagccaa tttttatgtt gtcagcaaat acgatattac cggtacagct tttatttatg
5581 ccagcaattg ctttcactaa agaggtttta cctccaccat ttggaccaag tattgtaatt
5641 atgtctcctc tttccatgaa catattgata ttgtcaagga ttcttttacc atcatatgtg
5701 agagcaagat tttctatttt tagaacacga ttattgacat tattcaattt attgacaaaa
5761 tttaacttct tctcaacgtt tatatgagac atttactgac tttttttcta ttactttcat
5821 ttacactata ctataatatc gccttttcat ccaatttaaa agttgtagct acaatcaaac
5881 ctatccactc acttgtagct tctattacag aagggatttt agaaccggaa ttgcttggct
5941 atgcaacatc tgcgcataat tatatattaa agccatctga tgcgagtaat ttagaatcaa
6001 gtgacgttat attttatgtt gacgataact tagagacgtt tgttcaaact ttcgccaaaa
6061 ataataaaga actagtgcaa ttgtcaaagg caatcaacct acttcctact cggccacatt
6121 cattttctag aaaaattact cacattaaag atgaaaaaga tttacacatt tggctgagtc
6181 ccgaaaatgc aaagagcatg atactttctg taagtgcaac attgtctaac atagataaag
6241 aaaattccca tcgatacaac tctaatgcaa caaaagctat aaaaaaaata gaccaagaag
6301 taaaagaaat tatgaaagaa ctgaatgatt ttaaaaatca aaaatatatc gtcactcacg
6361 atgcttatca atattttgaa aaatattttg gtttaaatca cccaagtgcc attctttcca
6421 tagaagagga ttcttacata ggtatgagga gtttaatgaa gctaaaaaag ataatgaagg
6481 aagaaaacgt taaatgtata tttgctcatt cacaggaaga cagcataaag cctaatgctc
6541 tttctaatga cgcaaaaatg gtagttcttg atcctattgg atcagacata gagcctggaa
6601 aagatgcata tctagctata atcaataaca ttgcacaaaa ttttaagtct tgttttgttg
6661 agtaataagt tcccttttag cttactgttt tattgttctg catttatttg tcctttacat
6721 attatgcagg tttagaatta tgctttattc ttacgtttat gtcttggccc aatacaatca
6781 ataagttaag caagtattct aaggagaacc cctcgatttt tgacctattg atttgagata
6841 ctttaggttg atctatcttt agagcgtttg ctgctttttt ctgtgttgtg tacttcttac
6901 ttattaattg tgcaatgacc tttattaatt tcagttttgt atctttcaca tcccccaact
6961 ctactattat aggataactt aattgtaaat tttgcataat aataaaatcc cttataaaaa
7021 aattacttta gtttttcttc tttaaataac acatgccttc tgactactgg gtcatacttc
7081 ctaaactcca gtttttttggt aagcttttt ggattacgct tttttacata aaaataacct
7141 gttgattttt cttcaccagt ttttgttgtc ttgattgcag tgctaactaa cttaacgagc
7201 aaagaagcat ttttttttcgc catacttata atgatataat aaattctatg cgagtttatt
7261 acgcaaaaca ctaaaagtca accgaataat gcagatcgta aaattcttga caatcaacag
7321 tgcattaagt agcatgaagt taacttcaga atgaaaagca aacggtaaat aagtggtaaa
7381 tagtggtaaa aggttattat tgatagaaaa ttccacctgt tctaatgagg taagagttgc
7441 cttgtcagtt aataataagg ttgtagaatt tgaacaagaa ttcaaggaaa aaaaacaatt

7501 aagaggtaat atatacgttg cttatataaa gcgtatagag ccttctttgc aagccgtatt
7561 tatcgaatat ggaaaaaata agcaaggttt tttgtctttt tccgagatat ctctagatta
7621 cttcaatatt ccagaaaaag aaaaggaaac tatctttgag agctactcaa atgacgatta
7681 tacagaaaaa gggaacgata ccatagcaaa tgtctcttca gattctgcag ctagtaagaa
7741 tgtcaacgag tcaggtagtg gttttgtaag agaggtacct ttatataaaa aatacaaatt
7801 acaagatgtt atttcagtaa atcaaaaagt attggttcag ctaactaaag aggaacgagg
7861 caataaagga gcttcattta caacatacat aactttagtg ggtagatact gtgttttcat
7921 gccaaattcc atgagtaaag gtggagtgtc tcgcagaatt gaagatgcta atgtcagaaa
7981 gcagttaaag gagatactga gttcaataaa cttgccaaaa aggtcaggtt taatagtaag
8041 aactgttggt tcaggaaaaa ataagaaaga aattgagcag gattacaatt acttatcctc
8101 attatggcag agcattcaaa aaaatgcctt ctctgtaaac gttccatcat taatttacaa
8161 tgaagcagat gtaattatga gatctgttcg tgatttttgt agcgatggta tagaagttat
8221 agtgtctgga aaggaagctt ttgaggcagt taggcaatat gctaacaatg cactaaaagg
8281 tagtaagttg cgctatagat tgtatagagg ttatgctccg atctttactt actacggaat
8341 cgaagatcaa atttctgagt tatatagcaa tagagtagaa ttgccatctg gtgggtcttt
8401 gataataact ttaactgagg cgtttgtttc aatagacgta aattcaggaa agatgacagg
8461 agaagatagc atagaagaaa cagcttatag aaccaacatg gaagcagtac ctgaaatatc
8521 aagacaggtg aatcttaggg gcctatcagg attaatagta gtcgatttta ttgatatgtt
8581 gaaatatcag tattgcagag ctgttgagtc tgctataaag caagcgttta aagatgataa
8641 agctaaagtt caatttagct acataaatga ctttggttta atggtatttt caagacaaag
8701 aattaaacca aacatacaag aaattaatac tacagagtgt ttacactgca aggtactgg
8761 aagagtaaaa tcaaacgaag taattgtttc atcaatacta agggatttac aacatattgc
8821 taacaagaat cgaaataagt cttttgattt agtagcacac agtgcagtta tagcgcacat
8881 ttttaataac aaacgcaata cagtttctac aatcgaaaaa gagtttaata ttacattgaa
8941 cgttagtatt gataacagtt tagatgtaaa tacatttgtt ttaaaacaag gagatgatgt
9001 taatttaaac ggttataagc cattacagaa ctctggatac caaattgggg atagcagcaa
9061 tgaagaagct gataattcaa ataaactacg aggcaatttt tggctgacta agtggctttc
9121 gcgcctttta agttctaata actaattaga aacctaaatt gggcttgtaa ttaatgagcc
9181 ctatacttat atattcttat atcagataat atttacaatg gggtactcat tatgggaaga
9241 gcaataggca tagatctcgg aacaacaaat tcttgtgttg caataatgca gggcaaggat
9301 acaaaggtaa tagaaaataa agaaggggca agaactactc catctatagt tgcattgact
9361 tcttcaggag aaaggttgat tggtgctcca gcaaaaaggc aagcaaccac caatgcaagt
9421 aatacctttt ttgctactaa gagattaata ggccgtcaat atagtgatcc tgagatgaaa
9481 aatctaagtg taccatacaa agtatttgca gcaaaaaagtg gtgatgcatg ggttaaaaca
9541 actgataata aagaatattc tcctagtcaa attggtgcat ttatactaca aaacatgaaa
9601 gaggcagcag aagcttatct tggagaggaa gtaaaagatg ctgtgataac agtgccagca
9661 tacttcaacg actctcaacg tcaagcaaca aaagatgcag gaaaaattgc cggattaaat
9721 gtactcagaa tagtaaatga accgactgct gcagcacttg cttatggtct tgataagaaa
9781 catgggcaca caatagtagt atatgacctt ggtggtggta catttgacgt ttcaatactt
9841 gaaataggtg atggagtttt cgaagtgaaa gcaacaaacg gtgataccca tcttggaggt
9901 gaagactttg ataatggagt agtgagttat ttactagatg aattcaagaa aagtaatggc
9961 attgatttga aaaatgatcc aatggctatg caaagaatca aagaagctgc tgaaaaagcg
10021 aaaattgaat tatcaagtgc aatggaaacg gaaataaatc taccgtttat tacagctgat
10081 gcaagtggtc caaaacactt aaatatgaaa ttaacgagag caaaacttga aagcttagtg
10141 aatgatttaa tcgaaagaac tatggctcct tgtaaaaaag ctcttgagga tgctggttta
10201 tctgctagtc aaattggcga agtggttctt gttggtggca tgactcgtat gccaaaagtc
10261 atagagaaag ttaaagaatt ctttggcaaa gatccacata gaggagttaa ccctgatgaa
10321 gttgtagcaa ttggagctgc aatacaggcg gggatcattc aaggtgatgt aagagatgtg
10381 ttactacttg atgtgacccc actttctctt ggtattgaaa ctctaggagg agtattcact
10441 ccacttattg agcgtaatac tacaattcct actaaaaaat ctcaggtgtt ttcaactgca
10501 gaagataacc aaacagctgt tacaattaag gtgcatcagg gtgaaagaaa actggcagtt
10561 gataataagc tactcggtca gtttagtttg gaagggatac ctcctgctcc gcgcggaagg
10621 ccacaaattg aagtgacatt tgatatagat gcaaatggaa tagcacacgt ttctgcaaaa
10681 gataaagcta ctggaaaaga gcaaaaaata cgcattcaat cttcaggcgg tttatctgat
10741 gatgaaataa atcgcatggt aaaagaagct gaagaaaaag cgcaagaaga tgaaaagcgc
10801 aaaaagttta ttgaagtgaa gaatcaagcg gatagcttag tccattctac agaaaagtct
10861 ctgacagagt atggtgataa agtttcacca gaagatagat ctgctattga aaatgcagtt
10921 aacgaattaa aagaagttag taaatctgat aacattgatg acgctgattc aatacagcag
10981 aaagtcacta atctttctca attatctatg aaacttggag aagctatgta tcaagcatct
11041 caacaaaata gtgctgaaaa tggcttttca tcagaaggaa atccaaatga taaagaggaa

```
    11101 aaagtagtag attctgatta tcaagacata gataataaag aagagaataa gtaattgcga
    11161 ttgtcatcca agtagctgat actgggatga caagaggagc atattgttgc ttaaagaaac
    11221 cattatggat attattaaaa cgatagaaga aaagatacac gattcgatag atgtaattga
    11281 tatcaacatt atcgatgaat cagcaaagca tgctgatcat tattttgctt catcttcaac
    11341 actaccttca catatcaagc taatattgat atctgacagc tttatcggaa tgaacactct
    11401 caaaaggcat aaattgatct atgaattatt aaagagtgag atagagctaa tacatgcaat
    11461 ttctcttcac ttgtatacgc aaaacgagta caatttaaaa aataaataat aaaaatgtga
    11521 aggaagagtc tttattagtt aaagcaggaa gaaaatttaa ggactataaa ggttctatga
    11581 acccgccagt ttatcattct tctaccatat tattccctac ttacaaggac tacttaaatg
    11641 cagcaaatgg agaaagtata tacgatgtaa tcaacgatga tgttgcaaga gattacagct
    11701 atagtaatgt tggtactccc actgttcatt atttttcaaa tgcactggct gaaattgaag
    11761 ggagtggaca agcgcttatt tttccttccg gactatttgc acttactttt gctattttga
    11821 ctttcactaa agcaggttct catgttttaa ttcaggataa cagttattac cgactcaaga
    11881 gatttgccga aaatgagtta ccaaacaggg gaatagaagt aactttttat gatccaacac
    11941 aggatataac tgatttaatt cagagtaata cttcattgat aatgatcgag actcctggtt
    12001 ctgtaacgtt tgagatttcg aatatagagc atatagtaaa agttgctaaa gaacgtggaa
    12061 tcgtaaccgt ttgcgacaat tcatgggcca ctcctttgtt atttaagccg cttgattatg
    12121 gaattgatgt tgcactatat gcggtaacaa agtatctagc cggtcactca gatttattga
    12181 tggggactat gattgctgaa ggtgaaattt ttaaattgct ttatgagagc tataaaaatt
    12241 atggagtaac cattcaatcg cacgactgtt atcttgcaca taggggacta agaacactgc
    12301 acacacgtat gaaaaagtac caaaatacag caatggaagt ggcaaagtgg ctagaagcac
    12361 agcaaaaagt tagaaaggtt ttatatccag cacttccctc ccatcctcaa cacgaattat
    12421 ggaaaagtta ctttaaagga gcaagcggcg tattcagtat agtattgggt agagaatatt
    12481 catgtgaaga actaagctgc atggttgatc acatgaaaat ttttggtatc ggtgcttctt
    12541 ggggagggtg cgacagtttg atattaccaa tagatcgtag atctatgtca agatctgtaa
    12601 tgaattcaga ttatggtgga agttttatac ggatattttg tggactggaa gatcctgaag
    12661 atttaatctt tgaccttaat gacgcactaa aaaggttacc atgtttagac tttcaaaacg
    12721 atgaagtagg acatgaaact gagagaatta ctgcataaca ttattgatgt taactttgat
    12781 attgaaatca aaggcgtcac atgtaatccc aagaaaatca aggaaggtta tctttttgtt
    12841 tgtgtgtcag aaagggatag gatttacata gatcagatat tatcttgtgg agctaaagcg
    12901 ataattgcaa gtgattgcat aacagaagct tcaagccttg gtatcattcc aacgcgtgat
    12961 gctggaatct gtatcttcca cccaaacccct caagaaatat acagcgaaat agtcagcagg
    13021 ttttatcaat tcaaacagcc aaaatatgtt gctgctgtaa caggcacaaa tggcaagact
    13081 tcagtagtag aattttgccg ccagatctgg caaaactctg gctataatgc tgcgtctatt
    13141 ggaacacttg gaacatgcat caataatggc agaaaaggta atagcaacag ccttaccact
    13201 ccaggtgcag aagaccttta cgcaacattg cgtggtataa gtgtagaaca cttagtgttg
    13261 gaatcatcaa gtcatgggat tgatcaatac agaatccatg gattaaagtt gagtgctgca
    13321 gcttttacta atttctcgca agatcattta gattaccata aaagtcttgg tggatattta
    13381 gaaactaaaa aaaggttatt ttacgaagta ttaccagaag gaaaaacagc gattttaaat
    13441 gcggatatag atgaatacgg cgcattgctt aaaatagctg aaaaacgcag taataaagtt
    13501 gtgacttatg gaaaaaaagg ctctgatatt actttattag agcaaatacc aactccaagt
    13561 ggtcaatacc ttacagtaaa agttgataac caaatttaca atacattttt cccagtttta
    13621 ggaaaatttc aagcatataa tctgctatgt gcaattggta tatctggatt aaattacaga
    13681 gaaatatgtg tggataaact cattttcccg tcaggaagaa tggagaaagt gaaacctttt
    13741 gcattcgtgg attacgctca tactccaagt gcgcttaaac aagccctatt gtctttaaaa
    13801 tggcacttca ataaaaaaat agttctagtt tttggatgcg gtggcaatcg tgatcaaaca
    13861 aaacgcgcag aaatgggtaa agtagcacaa atatatgcag acaaagtgat aatcacggat
    13921 gacaatccgc gtgatgagaa tcctgcagaa attcgtcgtg gtattttact acattgccct
    13981 gatgcgctag agatagaaga tagaagagaa gctatagaga aaggagtaga tattgcctat
    14041 aatgagggta tgattctgct agttgcagga aaggggcatg aaggatacca aatcgtgagc
    14101 ggccaaactt ttgaattcag tgatgttgag gttattaaaa atca
//
LOCUS       JAQZAU010000039            18036 bp    dna     linear   ENV 17-MAR-2023
DEFINITION  MAG: Wolbachia pipientis isolate wEsol contig_3090, whole genome
            shotgun sequence.
ACCESSION   JAQZAU010000039  JAQZAU010000000
VERSION     JAQZAU010000039.1
DBLINK      BioProject: PRJNA929258
            BioSample: SAMN33142973
KEYWORDS    WGS; ENV; Metagenome Assembled Genome; MAG.
```

```
SOURCE      Wolbachia pipientis (insect metagenome)
  ORGANISM  Wolbachia pipientis
            Bacteria; Pseudomonadota; Alphaproteobacteria; Rickettsiales;
            Anaplasmataceae; Wolbachieae; Wolbachia.
REFERENCE   1  (bases 1 to 18036)
  AUTHORS   Fiutek,N., Couger,B.M., Roy,S., de la Torre,J.R. and Connor,E.F.
  TITLE     Genomic assessment of the contribution of the Wolbachia
            endosymbiont of Eurosta solidaginis to gall-induction
  JOURNAL   International Journal Molecular Sciences
REFERENCE   2  (bases 1 to 18036)
  AUTHORS   Fiutek,N., Couger,B.M., Roy,S., de la Torre,J.R. and Connor,E.F.
  TITLE     Direct Submission
  JOURNAL   Submitted (10-FEB-2023) Biology, San Francisco State University,
            1600 Holloway Ave, San Francisco, CA 94132, USA
COMMENT     ##Genome-Assembly-Data-START##
            Assembly Date          :: JUN-2019
            Assembly Method        :: FLYE v. 06-2019
            Genome Representation  :: Full
            Expected Final Version :: Yes
            Genome Coverage        :: 100.0x
            Sequencing Technology  :: Illumina HiSeq; PacBio Sequel
            ##Genome-Assembly-Data-END##
FEATURES             Location/Qualifiers
     source          1..18036
                     /organism="Wolbachia pipientis"
                     /mol_type="genomic DNA"
                     /submitter_seqid="contig_3090"
                     /isolate="wEsol"
                     /isolation_source="galls on Solidago altissima"
                     /host="Eurosta solidaginis MN-2018-EFC"
                     /db_xref="taxon:955"
                     /environmental_sample
                     /country="USA: Cowling Arboretum, Carleton College,
                     Northfield, MN"
                     /lat_lon="44.48 N 93.13 W"
                     /altitude="280 m"
                     /collection_date="2018-11"
                     /metagenome_source="insect metagenome"
                     /note="metagenomic"
     gene            complement(<1..140)
                     /locus_tag="wEsol_00298"
     CDS             complement(<1..140)
                     /locus_tag="wEsol_00298"
                     /codon_start=1
                     /transl_table=11
                     /product="hypothetical protein"
                     /translation="MSVATRFFQLLCNKAIPVSSTGMTSLLLQYSHISGGKPIAKISM
                     LR"
     gene            182..511
                     /locus_tag="wEsol_00299"
     CDS             182..511
                     /locus_tag="wEsol_00299"
                     /codon_start=1
                     /transl_table=11
                     /pfam="DDE_Tnp_1_5"
                     /fullproduct="qcoverage 100 , hcoverage 100 , similarity
                     100 , identity 100 , evalue 1.19e-72 , alnlength 109 ,
                     Bacteria , Proteobacteria , WP_007548762.1 MULTISPECIES:
                     transposase [Wolbachia]"
                     /translation="MPQKMRVSNHSEYNKFLEKRGNIFHYINEAIEKWYENGPKIPGG
```

```
                     NNIYSDKVVILVHIITCLFRIGLRQTVGFIAGYLEQIGKNLQVISYSQSSRRFKKLNI
                     KINDCRK"
                     /product="transposase"
                     /besthit="qcoverage 100 , hcoverage 100 , similarity 100
                     , identity 100 , evalue 1.19e-72 , alnlength 109 ,
                     Bacteria , Proteobacteria , WP_007548762.1 MULTISPECIES:
                     transposase [Wolbachia]"
     gene            495..695
                     /locus_tag="wEsol_00300"
     CDS             495..695
                     /locus_tag="wEsol_00300"
                     /codon_start=1
                     /transl_table=11
                     /fullproduct="qcoverage 100 , hcoverage 100 , similarity
                     100 , identity 100 , evalue 3.80e-36 , alnlength 66 ,
                     Bacteria , Proteobacteria , WP_010082383.1 MULTISPECIES:
                     hypothetical protein [Wolbachia]"
                     /translation="MIAENNMEDIEIAIDSTGISIYNNTPGHSKENSEDRKYRSYEQT
                     RKLHVMLNTNSKKAIAVKYSNG"
                     /product="hypothetical protein"
                     /besthit="qcoverage 100 , hcoverage 100 , similarity 100
                     , identity 100 , evalue 3.80e-36 , alnlength 66 ,
                     Bacteria , Proteobacteria , WP_010082383.1 MULTISPECIES:
                     hypothetical protein [Wolbachia]"
     gene            715..993
                     /locus_tag="wEsol_00301"
     CDS             715..993
                     /locus_tag="wEsol_00301"
                     /codon_start=1
                     /transl_table=11
                     /pfam="DDE_Tnp_1 DDE_Tnp_1_2"
                     /fullproduct="qcoverage 96.7391304347826 , hcoverage
                     92.7083333333333 , similarity 98.9 , identity 98.9 ,
                     evalue 5.81e-57 , alnlength 89 , Bacteria ,
                     Proteobacteria , WP_210437473.1 transposase, partial
                     [Wolbachia endosymbiont of Drosophila auraria]"
                     /translation="MLKEVDFQYVIKVLYADRAYDREKLYKLCNQYGIKTKIPPKNNA
                     AEHPKLDYMAERNSAVKLIKSYDEDGIKKWKKEMSYGKRSYIEVFSRD"
                     /product="transposase, partial"
                     /besthit="qcoverage 96.7391304347826 , hcoverage
                     92.7083333333333 , similarity 98.9 , identity 98.9 ,
                     evalue 5.81e-57 , alnlength 89 , Bacteria ,
                     Proteobacteria , WP_210437473.1 transposase, partial
                     [Wolbachia endosymbiont of Drosophila auraria]"
     gene            1209..1469
                     /locus_tag="wEsol_00302"
     CDS             1209..1469
                     /locus_tag="wEsol_00302"
                     /codon_start=1
                     /transl_table=11
                     /fullproduct="qcoverage 100 , hcoverage 100 , similarity
                     100 , identity 100 , evalue 3.75e-54 , alnlength 86 ,
                     Bacteria , Proteobacteria , WP_012673289.1 MULTISPECIES:
                     hypothetical protein [Wolbachia]"
                     /translation="MTEDDGVTGSNTKSFVIPIESALKSMFKVEISLEDAGKIRKGQE
                     VVLNNLRNLKNHDICCTVVGSVPIAICSFIYGCVKPIRVFNI"
                     /product="hypothetical protein"
                     /besthit="qcoverage 100 , hcoverage 100 , similarity 100
                     , identity 100 , evalue 3.75e-54 , alnlength 86 ,
                     Bacteria , Proteobacteria , WP_012673289.1 MULTISPECIES:
```

```
                     hypothetical protein [Wolbachia]"
     gene            1482..1754
                     /locus_tag="wEsol_00303"
     CDS             1482..1754
                     /locus_tag="wEsol_00303"
                     /codon_start=1
                     /transl_table=11
                     /fullproduct="qcoverage 100 , hcoverage 100 , similarity
                     100 , identity 100 , evalue 1.05e-55 , alnlength 90 ,
                     Bacteria , Proteobacteria , WP_006279944.1 MULTISPECIES:
                     30S ribosomal protein S15 [Wolbachia]"
                     /cog="RpsO COG0184 89 Ribosomal protein S15P/S13E"
                     /pfam="Ribosomal_S15"
                     /tigr="TIGR00952 S15_bact 86 ribosomal protein S15"
                     /product="30S ribosomal
                     protein S15"
                     /translation="MSITSEKKKSLINIYAIKEDDTGSSFVQCAILTERISNLTEHFK
                     VHKHDHHSKRGLLILIGRRRKHLNYIKRKFGNEAYQELIEKLGIRK"
                     /besthit="qcoverage 100 , hcoverage 100 , similarity 100
                     , identity 100 , evalue 1.05e-55 , alnlength 90 ,
                     Bacteria , Proteobacteria , WP_006279944.1 MULTISPECIES:
                     30S ribosomal protein S15 [Wolbachia]"
     gene            1769..4042
                     /locus_tag="wEsol_00304"
     CDS             1769..4042
                     /locus_tag="wEsol_00304"
                     /codon_start=1
                     /transl_table=11
                     /fullproduct="qcoverage 100 , hcoverage 100 , similarity
                     100 , identity 100 , evalue 0.0 , alnlength 757 ,
                     Bacteria , Proteobacteria , WP_155969351.1 MULTISPECIES:
                     polyribonucleotide nucleotidyltransferase [Wolbachia]"
                     /cog="Pnp COG1185 692 Polyribonucleotide
                     nucleotidyltransferase (polynucleotide phosphorylase)"
                     /pfam="RNase_PH RNase_PH_C Pfam-B_11765 PNPase RNase_PH
                     Cauli_DNA-bind RNase_PH_C KH_2 KH_1 KH_3 S1"
                     /tigr="TIGR03591 polynuc_phos 689 polyribonucleotide
                     nucleotidyltransferase"
                     /product="polyribonucleotide
                     nucleotidyltransferase"
                     /translation="MFKIIKKSIEWGGRALSLETGKIARQAHGSVVVNYGDTSVLVTV
                     VRKKKEESVDFLPLNVQFIAKSYAMGKIPGGFFKREGKPSDRETLISRVIDRSIRPLF
                     PEGFHDEISVVCNLLTYDTVNPPEVPALIGAVAALAISGVPFHFTIAGVMVGCDENNN
                     YILNPSVQEMKASSLDLFLSGDENSILMVESEVKELSEENVFNAIKFGHEHLKPVIKL
                     IKEFADTIGNKPESFAPIDASDITQELEKYGKDFEKAYSQTVKQERVQALEAIRENIL
                     NTLKETGKDEKLITYAVKNFERSLVREIIRKKSVRIDSRKHDEIRQIEVEVDILSKTH
                     GSALFTRGNTQALVVTALGTTQDEQIVDDIEGDRREHFMLHYNFPSFAVGETSAARAP
                     GRREIGHGKLAWKAIHPVLPDKSEFPYTIRVVSEILESDGSSSMATVCGTSLALMDTG
                     VPIKAPVAGIAMGLIKDKDEYVILSDILGDEDYLGDMDFKVAGTSEGVTALQMDMKIS
                     GISFEIVEKSLEQAKAGRLHILEKMNAVISEHSDDVKDHAPRMLSFYIDKDKISAAIG
                     SKGKNIRSVCERSNAKIEIGDDGKVSVFATSGTEAEIAKSMMIDSITELEQGSIVDVK
                     VVRIEKSIVELEFLNGRKGKMHISEVANEHIDSIESVLKQDDTFKALVIDFEKGGCPK
                     LSRRRVDQETGEFFEGELYNEERKDGPNDRDNYYNNSFSRKPGGSHHKRPPRPRSGFS
                     NRNRPKFGNNDSSSGFY"
                     /besthit="qcoverage 100 , hcoverage 100 , similarity 100
                     , identity 100 , evalue 0.0 , alnlength 757 , Bacteria ,
                     Proteobacteria , WP_155969351.1 MULTISPECIES:
                     polyribonucleotide nucleotidyltransferase [Wolbachia]"
     gene            4249..4635
                     /locus_tag="wEsol_00305"
```

```
CDS             4249..4635
                /locus_tag="wEsol_00305"
                /codon_start=1
                /transl_table=11
                /pfam="Pfam-B_3816 DUF972 Pfam-B_4504 Pfam-B_3314 DivIC
                HALZ Pfam-B_1653 Pfam-B_3883 bZIP_1"
                /fullproduct="qcoverage 100 , hcoverage 100 , similarity
                99.2 , identity 99.2 , evalue 1.74e-65 , alnlength 128 ,
                Bacteria , Proteobacteria , AAS14583.1 transposase,
                degenerate [Wolbachia endosymbiont of Drosophila
                melanogaster]"
                /translation="MVNLLELCKNLQQKIEKLEAEIENLKAENKALKIENAELKERLG
                LNSKNSSLPSSKELYKIKKDKPKSERNVGDQVGHKGNFHATMEADKVVKIELPNICEC
                GGELVICEKPYVHQKVDLPEIRPYVV"
                /product="transposase, degenerate"
                /besthit="qcoverage 100 , hcoverage 100 , similarity 99.2
                , identity 99.2 , evalue 1.74e-65 , alnlength 128 ,
                Bacteria , Proteobacteria , AAS14583.1 transposase,
                degenerate [Wolbachia endosymbiont of Drosophila
                melanogaster]"
gene            complement(4661..5143)
                /locus_tag="wEsol_00306"
CDS             complement(4661..5143)
                /locus_tag="wEsol_00306"
                /codon_start=1
                /transl_table=11
                /product="hypothetical protein"
                /pfam="DDE_3 rve"
                /translation="MKIIATKEPKNLVYIDESGIDNTEDYPYGYCQKGQRFYALKSGK
                KTQRISMVAALSGRKIVVPLTFEGHCNMDVFNGWVEQFLTPILEPGQTVILDNATFHK
                SDKIIELAKGVGAEILYLPPYSPDFNKIEHHWFAIKNRARKNIPLFESFRRAVDSAFL
                "
                /besthit="qcoverage 100 , hcoverage 58.1818181818182 ,
                similarity 100 , identity 99.4 , evalue 1.00e-112 ,
                alnlength 160 , Bacteria , Proteobacteria ,
                WP_213863609.1 IS630 family transposase, partial
                [Wolbachia endosymbiont of Ceratitis capitata]"
gene            complement(5140..5556)
                /locus_tag="wEsol_00307"
CDS             complement(5140..5556)
                /locus_tag="wEsol_00307"
                /codon_start=1
                /transl_table=11
                /fullproduct="qcoverage 86.9565217391304 , hcoverage
                95.2380952380952 , similarity 100 , identity 100 , evalue
                4.82e-81 , alnlength 120 , Bacteria , Proteobacteria ,
                AOV87628.1 transposase [Wolbachia endosymbiont of
                Drosophila incompta]"
                /cog="COG3415 COG3415 138 Transposase and inactivated
                derivatives"
                /pfam="HTH_Tnp_IS630 HTH_23 HTH_28 HTH_29 CENP-B_N HTH_7
                HTH_23"
                /product="transposase"
                /translation="MAFFPLLSICLLCSENWYYDLRKKAMEALDEGESRETVAARFKI
                GRTTLWEWQQRRKETGDFQSKKLGNGGYNHKITDWDTFAKFAREHGGKTLSEMAKLWN
                NVSIQTIYRALKKIGFTRKKRHMDTRKETKKNVLNF"
                /besthit="qcoverage 86.9565217391304 , hcoverage
                95.2380952380952 , similarity 100 , identity 100 , evalue
                4.82e-81 , alnlength 120 , Bacteria , Proteobacteria ,
                AOV87628.1 transposase [Wolbachia endosymbiont of
```

```
                     Drosophila incompta]"
     gene            5586..6107
                     /locus_tag="wEsol_00308"
     CDS             5586..6107
                     /locus_tag="wEsol_00308"
                     /codon_start=1
                     /transl_table=11
                     /product="hypothetical protein"
                     /pfam="HTH_23 HTH_28 HTH_1 HTH_29 HTH_Tnp_IS630 HTH_32
                     Pfam-B_19535 HTH_Tnp_Tc3_2 HTH_24 HTH_33"
                     /translation="MALRSKLLDEKVVESAKAMLKKVRNNAYVTKKLNAVIAAKKHSI
                     TAVAKICCISRTALTAWIKHLKFNREEKLFAPPQRRRKTRLNQSQREQVEAWIQENPN
                     ITIKEMRIKIQERFGLDISKSTVHRNMQRMKFSYITPRPVHNGQDKSKQEEFKKKSQR
                     NYWQISRKRAIFL"
                     /cog="COG3415 COG3415 138 Transposase and inactivated
                     derivatives"
                     /besthit="qcoverage 90.1734104046243 , hcoverage
                     47.1299093655589 , similarity 100 , identity 100 , evalue
                     8.70e-97 , alnlength 156 , Bacteria , Proteobacteria ,
                     WP_155968376.1 MULTISPECIES: IS630 family transposase
                     [Wolbachia]"
     gene            complement(6210..6542)
                     /locus_tag="wEsol_00309"
     CDS             complement(6210..6542)
                     /locus_tag="wEsol_00309"
                     /codon_start=1
                     /transl_table=11
                     /product="hypothetical protein"
                     /translation="MEWVREVTNLHKVRSSRVIVSYILLHKMFCFIYNQNLSTGLSSG
                     EYGGRYIISIFLGTFKFFDLCQLAQSITRKVSRIPRYCDICSRNIFMQSVLTFELELS
                     PFLGLTAL"
                     /besthit="qcoverage 51.8181818181818 , hcoverage
                     96.6101694915254 , similarity 86.0 , identity 82.5 ,
                     evalue 5.00e-24 , alnlength 57 , Bacteria ,
                     Proteobacteria , ONI56732.1 hypothetical protein
                     N500_0170 [Wolbachia pipientis wUni]"
     gene            complement(6545..8089)
                     /locus_tag="wEsol_00310"
     CDS             complement(6545..8089)
                     /locus_tag="wEsol_00310"
                     /codon_start=1
                     /transl_table=11
                     /product="hypothetical protein"
                     /translation="MPNIMNQELVRAFNIILCHNEVAITNLIVQSTDQTFLKKEDRIV
                     ISFDKNMDVGQCMKYLHNIKKSIVETFCGSESGANMSFNTQFFPFNLNERQLTKDKEG
                     YKFSVPIDKGVVFNLKCLLAHGLAKDAKEDMGNEFGERCMEGRVYDVRFDRKSYLYCM
                     RFYDKKLQDYFVESFSSRIKNYKNGPKSTNDFLKIKDNCVYIKGSIFNKAKFIKDIVK
                     HKASLECRDFEVTSAEDETVESLKFPVIDLSRTVGIPINSVLHVSYTGSGENSVLIPA
                     AKEIIDNIIYYKILSKEQADDINKVFGMTVLNNVEDCISPKHLPVFGRRGIYGIDFAN
                     QDISYCVLNKIMESSVINKDHELAIDPELPRTLLAEASQTRGCPKRSRGKGLERPPRQ
                     GSEREGGKGGLFQLVPGKIDSSQSTGDQSQLSSGEIDLSQSTGDQSQLSLGAGEGTLT
                     EANIQDQKRSSSIPSLRAWDSSTFLNSEDSLKDTENICNEEPEKQQVIPILIIRRTNR
                     RHCRVV"
                     /besthit="qcoverage 98.2490272373541 , hcoverage
                     70.6293706293706 , similarity 78.4 , identity 68.5 ,
                     evalue 1.22e-223 , alnlength 505 , Bacteria ,
                     Proteobacteria , WP_065106813.1 hypothetical protein
                     [Wolbachia endosymbiont of Nomada ferruginata]"
     gene            complement(8127..8321)
                     /locus_tag="wEsol_00311"
```

```
     CDS             complement(8127..8321)
                     /locus_tag="wEsol_00311"
                     /codon_start=1
                     /transl_table=11
                     /fullproduct="qcoverage 96.875 , hcoverage
                     93.9393939393939 , similarity 83.9 , identity 77.4 ,
                     evalue 1.10e-21 , alnlength 62 , Bacteria ,
                     Proteobacteria , WP_191111146.1 hypothetical protein
                     [Candidatus Wolbachia massiliensis]"
                     /translation="MDSNKGSGYEKRKYGDNTAPGGGRIRADAIDPRRVDDFTERIKR
                     EEERRKQNQSSGQGSGRSGK"
                     /product="hypothetical protein"
                     /besthit="qcoverage 96.875 , hcoverage 93.9393939393939 ,
                     similarity 83.9 , identity 77.4 , evalue 1.10e-21 ,
                     alnlength 62 , Bacteria , Proteobacteria , WP_191111146.1
                     hypothetical protein [Candidatus Wolbachia massiliensis]"
     gene            complement(8742..9839)
                     /locus_tag="wEsol_00312"
     CDS             complement(8742..9839)
                     /locus_tag="wEsol_00312"
                     /codon_start=1
                     /transl_table=11
                     /pfam="SHOCT"
                     /fullproduct="qcoverage 100.27397260274 , hcoverage
                     85.1162790697674 , similarity 83.1 , identity 80.1 ,
                     evalue 3.78e-168 , alnlength 366 , Bacteria ,
                     Proteobacteria , WP_182309949.1 hypothetical protein
                     [Wolbachia pipientis]"
                     /translation="MLRRLECERQASSCIDEISSQAELHIGEGDDEDYFTGEKGRVLG
                     DFWNFFESLFNLFEVDVDDPKIYQMLKEKKEKNWLLSIIKFLRDKLKELIKKIFARDL
                     SFNQRLDKEIKELQEKLGSGELSEEEFARALERLTALQDLKLRLQMAVVGWIVTMFAE
                     MFGVELAAAVEASTEKEDKKAEKTSLKEENKEVCENVEIKLDEREKPKVPVFLFEISP
                     GFVRPVVLSKPELDLLPKPIMPSIFKEVINNENREEIKPKVEEEKKAKPEEPKKEVCP
                     QKVPEKPREEVPEKVQSAGSRTSSKKSRKINRDSFPAHNYPIGDWQEQASNGKSNAST
                     AQPPKRLIDDVKVNNHVDSIGKAPEKAPSRG"
                     /product="hypothetical protein"
                     /besthit="qcoverage 100.27397260274 , hcoverage
                     85.1162790697674 , similarity 83.1 , identity 80.1 ,
                     evalue 3.78e-168 , alnlength 366 , Bacteria ,
                     Proteobacteria , WP_182309949.1 hypothetical protein
                     [Wolbachia pipientis]"
     gene            10113..10226
                     /locus_tag="wEsol_00313"
     CDS             10113..10226
                     /locus_tag="wEsol_00313"
                     /codon_start=1
                     /transl_table=11
                     /fullproduct="qcoverage 100 , hcoverage 100 , similarity
                     100 , identity 100 , evalue 1.09e-14 , alnlength 37 ,
                     Bacteria , Proteobacteria , QEF50483.1 putative
                     excinuclease ABC, B subunit [Wolbachia endosymbiont of
                     Drosophila ananassae]"
                     /translation="MPKSIIKSISNTLQEKVVIEAKANINKDNLRKAHARV"
                     /product="putative excinuclease ABC, B
                     subunit"
                     /besthit="qcoverage 100 , hcoverage 100 , similarity 100
                     , identity 100 , evalue 1.09e-14 , alnlength 37 ,
                     Bacteria , Proteobacteria , QEF50483.1 putative
                     excinuclease ABC, B subunit [Wolbachia endosymbiont of
                     Drosophila ananassae]"
```

```
gene            10386..11675
                /locus_tag="wEsol_00314"
CDS             10386..11675
                /locus_tag="wEsol_00314"
                /codon_start=1
                /transl_table=11
                /pfam="SHOCT"
                /fullproduct="qcoverage 100.2331002331 , hcoverage 100 ,
                similarity 85.1 , identity 82.1 , evalue 4.98e-209 ,
                alnlength 430 , Bacteria , Proteobacteria ,
                WP_182309949.1 hypothetical protein [Wolbachia
                pipientis]"
                /translation="MVESFSGNNRGNNFIEEAKIMENFSDNNGNGSSIFQGKLREQDF
                SAEQKLNELKAAIEELARQDLLRRLECERQASSCIDEISSQAELHIGEGDDEDYFTGE
                KGRVLGDFWNFFESLFNLFEVDVDDPKIYQMLKEKKEKNWLLSIIKFLRDKLKELIKK
                IFARDLSFNQRLDKEIKELQEKLGSGELSEEEFARALERLTALQDLKLRLQMAVVGWI
                VTMFAEMFGVELAAAVEASTEKEDKKAEKTSLKEENKEVCENVEIKLDEREKPKVPVF
                LFEISPGFVRPVVLSKPELDLLPKPIMPSIFKEVINNENREEIKPKVEEEKKAKPEEP
                KKEVCPQKVPEKPREEVPEKVQSAGSRTSSKKSRKINRDSFPAHNYPIGDWQEQASNG
                KSNASTAQPPKRLIDDVKVNNHVDSIGKAPEKAPSRG"
                /product="hypothetical protein"
                /besthit="qcoverage 100.2331002331 , hcoverage 100 ,
                similarity 85.1 , identity 82.1 , evalue 4.98e-209 ,
                alnlength 430 , Bacteria , Proteobacteria ,
                WP_182309949.1 hypothetical protein [Wolbachia
                pipientis]"
gene            12096..12290
                /locus_tag="wEsol_00315"
CDS             12096..12290
                /locus_tag="wEsol_00315"
                /codon_start=1
                /transl_table=11
                /fullproduct="qcoverage 96.875 , hcoverage
                93.9393939393939 , similarity 83.9 , identity 77.4 ,
                evalue 1.10e-21 , alnlength 62 , Bacteria ,
                Proteobacteria , WP_191111146.1 hypothetical protein
                [Candidatus Wolbachia massiliensis]"
                /translation="MDSNKGSGYEKRKYGDNTAPGGGRIRADAIDPRRVDDFTERIKR
                EEERRKQNQSSGQGSGRSGK"
                /product="hypothetical protein"
                /besthit="qcoverage 96.875 , hcoverage 93.9393939393939 ,
                similarity 83.9 , identity 77.4 , evalue 1.10e-21 ,
                alnlength 62 , Bacteria , Proteobacteria , WP_191111146.1
                hypothetical protein [Candidatus Wolbachia massiliensis]"
gene            12328..14520
                /locus_tag="wEsol_00316"
CDS             12328..14520
                /locus_tag="wEsol_00316"
                /codon_start=1
                /transl_table=11
                /fullproduct="qcoverage 100 , hcoverage 102.097902097902
                , similarity 78.1 , identity 68.8 , evalue 0.0 ,
                alnlength 730 , Bacteria , Proteobacteria ,
                WP_065106813.1 hypothetical protein [Wolbachia
                endosymbiont of Nomada ferruginata]"
                /translation="MPNIMNQELVRAFNIILCHNEVAITNLIVQSTDQTFLKKEDRIV
                ISFDKNMDVGQCMKYLHNIKKSIVETFCGSESGANMSFNTQFFPFNLNERQLTKDKEG
                YKFSVPIDKGVVFNLKCLLAHGLAKDAKEDMGNEFGERCMEGRVYDVRFDRKSYLYCM
                RFYDKKLQDYFVESFSSRIKNYKNGPKSTNDFLKIKDNCVYIKGSIFNKAKFIKDIVK
                HKASLECRDFEVTSAEDETVESLKFPVIDLSRTVGIPINSVLHVSYTGSGENSVLIPA
```

AKEIIDNIIYYKILSKEQADDINKVFGMTVLNNVEDCISPKHLPVFGRRGIYGIDFAN
QDISYCVLNKIMESSVINKDHELAIDPELPRTLLAEASQTRGCPKRSRGKGLERPPRQ
GSEREGGKGGLFQLVPGKIDSSQSTGDQSQLSSGEIDLSQSTGDQSQLSLGAGEGTLT
EANIQDQKRSSSIPSLRAWDSSTFLNSEDSLKDTENICNEEPEKQQVTDESDRKSSDE
CSKAKPQDSKSPEKKHGDGLLCSNSSIGSMWSKLKGNVLPSLKRNNKGRGSVYSISSS
VSAPGGSVPEKLPVSESSSSFPGRIKAKSNPPSAVTSPTHEGVPEWQQRLNPDQREEL
RPFLSPEERKKLNSNYFSPGEEWHLYLTRTLSGKRELSRFLCESEESQLRRLAEAQLS
SPDNSSSKKVDRDSGFCSMGSPAVQHKGSVPSEVKECEVEPVSEALRK"
/product="hypothetical protein"
/besthit="qcoverage 100 , hcoverage 102.097902097902 , similarity 78.1 , identity 68.8 , evalue 0.0 , alnlength 730 , Bacteria , Proteobacteria , WP_065106813.1 hypothetical protein [Wolbachia endosymbiont of Nomada ferruginata]"

gene complement(14500..14769)
/locus_tag="wEsol_00317"

CDS complement(14500..14769)
/locus_tag="wEsol_00317"
/codon_start=1
/transl_table=11
/product="hypothetical protein"
/translation="MTNSLKIVPVIESGSFFLPQQAVWLSDFEYEILMFPEARHDDQV
DSTAQYLQWMRKTNTSAFRVRDFGSNPNKTLGWISKNLNLFSQSL"
/besthit="qcoverage 87.6404494382023 , hcoverage 19.4513715710723 , similarity 89.7 , identity 80.8 , evalue 1.85e-37 , alnlength 78 , Eukaryota , Arthropoda , GFS78656.1 terminase [Nephila pilipes]"

gene complement(14726..15493)
/locus_tag="wEsol_00318"

CDS complement(14726..15493)
/locus_tag="wEsol_00318"
/codon_start=1
/transl_table=11
/product="hypothetical protein"
/pfam="Terminase_6"
/translation="MLVMHRLHLEDLTGHLLSKPKNIWHHICLPMISENKEVIYSINN
HTGVIPTPPPVIPVPRHWDPENSTAKDWIPASSAGMTLPARVLYSREENELLYPLEGG
REEVEMIKVELGSYAFAAQYQQNPLPLSSGIIKQEWLKRYKNFPDNPSHVTQSWDTAV
STNDSSDFSICTTWAKIDNKFYLLDVYRAKLEYPKLKEQVLSLAARWTPHAILIEAKT
SGQQLTQELKANSDLPIIEIIPHNDKLTQNCSGDRVW"
/besthit="qcoverage 96.8627450980392 , hcoverage 48.1481481481481 , similarity 99.2 , identity 97.6 , evalue 1.35e-172 , alnlength 247 , Bacteria , Proteobacteria , MBV2146448.1 phage terminase large subunit [Wolbachia endosymbiont of Pissodes strobi]"

gene complement(15496..15864)
/locus_tag="wEsol_00319"

CDS complement(15496..15864)
/locus_tag="wEsol_00319"
/codon_start=1
/transl_table=11
/product="hypothetical protein"
/translation="MLRGPHGYWATARIIVASYSKLLSEKHSLDTRCIMQSDWYKELF
PEVELSKDQNTKYKFQTVQRGYRIATSVGGTLTGEGGDFIIVDDPLNPAQALSETFRK
RATNWFDQTLVTRLNDRKKE"
/cog="COG5410 COG5410 305 Uncharacterized protein conserved in bacteria"
/besthit="qcoverage 99.1803278688525 , hcoverage 62.0512820512821 , similarity 100 , identity 99.2 , evalue 7.58e-82 , alnlength 121 , Bacteria ,

```
                     Proteobacteria , RLT59430.1 hypothetical protein
                     WANA31_0872 [Wolbachia endosymbiont of Drosophila
                     ananassae]"
gene                 complement(15803..16048)
                     /locus_tag="wEsol_00320"
CDS                  complement(15803..16048)
                     /locus_tag="wEsol_00320"
                     /codon_start=1
                     /transl_table=11
                     /fullproduct="qcoverage 100 , hcoverage 100 , similarity
                     100 , identity 98.8 , evalue 9.11e-55 , alnlength 81 ,
                     Bacteria , Proteobacteria , CDR79199.1 phage
                     protein,Transposase and inactivated derivatives
                     [Wolbachia endosymbiont of Drosophila simulans wAu]"
                     /translation="MDNINFLKFIELCFQTVVPGCEYNDYQYIKVIADRLEAANAGEV
                     RRIIFNMPPRSMKSICMSVAWPAWILGNCKNNSCKLF"
                     /product="phage protein,Transposase and
                     inactivated derivatives"
                     /cog="COG5410 COG5410 305 Uncharacterized protein
                     conserved in bacteria"
                     /besthit="qcoverage 100 , hcoverage 100 , similarity 100
                     , identity 98.8 , evalue 9.11e-55 , alnlength 81 ,
                     Bacteria , Proteobacteria , CDR79199.1 phage
                     protein,Transposase and inactivated derivatives
                     [Wolbachia endosymbiont of Drosophila simulans wAu]"
gene                 16120..16500
                     /locus_tag="wEsol_00321"
CDS                  16120..16500
                     /locus_tag="wEsol_00321"
                     /codon_start=1
                     /transl_table=11
                     /fullproduct="qcoverage 100 , hcoverage 100 , similarity
                     100 , identity 100 , evalue 1.16e-87 , alnlength 126 ,
                     Bacteria , Proteobacteria , AOV87106.1 transposase
                     [Wolbachia endosymbiont of Drosophila incompta]"
                     /cog="COG3293 COG3293 124 Transposase and inactivated
                     derivatives"
                     /pfam="DUF4096"
                     /product="transposase"
                     /translation="MRKKYPTDLSEREWARIEKHFRVSYKKGGRPPKYSKREILEAIF
                     YVLRTGCQWRYLPNDFPLWKTVYEQFRQWKKQGIFEKMNYEITKYSRRKIGRNEQPSA
                     CIVDSQSVKTTEKGGSKAMMEVKK"
                     /besthit="qcoverage 100 , hcoverage 100 , similarity 100
                     , identity 100 , evalue 1.16e-87 , alnlength 126 ,
                     Bacteria , Proteobacteria , AOV87106.1 transposase
                     [Wolbachia endosymbiont of Drosophila incompta]"
gene                 16611..16952
                     /locus_tag="wEsol_00322"
CDS                  16611..16952
                     /locus_tag="wEsol_00322"
                     /codon_start=1
                     /transl_table=11
                     /fullproduct="qcoverage 100 , hcoverage 100 , similarity
                     100 , identity 100 , evalue 1.53e-79 , alnlength 113 ,
                     Bacteria , Proteobacteria , AAS13810.1 transposase, IS5
                     family, OrfB [Wolbachia endosymbiont of Drosophila
                     melanogaster]"
                     /cog="COG3293 COG3293 124 Transposase and inactivated
                     derivatives"
                     /pfam="DDE_Tnp_1_2 DDE_Tnp_1"
```

```
                     /product="transposase, IS5 family, OrfB"
                     /translation="MRTKYTKVKKMWADMGYQGRNLKNHIKEEYDIDIEIVKRPPCRF
                     WVHKDTPPELLPTREQGFKVQPRRWVVERTFAWVNRNRRLSKEYDLLTTSTENFIYLA
                     MSRVMLKREYA"
                     /besthit="qcoverage 100 , hcoverage 100 , similarity 100
                     , identity 100 , evalue 1.53e-79 , alnlength 113 ,
                     Bacteria , Proteobacteria , AAS13810.1 transposase, IS5
                     family, OrfB [Wolbachia endosymbiont of Drosophila
                     melanogaster]"
     gene            17031..17378
                     /locus_tag="wEsol_00323"
     CDS             17031..17378
                     /locus_tag="wEsol_00323"
                     /codon_start=1
                     /transl_table=11
                     /fullproduct="qcoverage 100 , hcoverage 100 , similarity
                     100 , identity 100 , evalue 2.74e-74 , alnlength 115 ,
                     Bacteria;Eukaryota , Proteobacteria;Arthropoda ,
                     WP_155968342.1 MULTISPECIES: IS630 transposase-related
                     protein [Wolbachia]"
                     /cog="COG3415 COG3415 138 Transposase and inactivated
                     derivatives"
                     /pfam="HTH_Tnp_IS630 HTH_Tnp_1 HTH_23 HTH_28 HTH_29 HTH_7
                     CENP-B_N Pfam-B_14738 HTH_8 HTH_17 HTH_24 Pfam-B_2106"
                     /product="IS630
                     transposase-related protein"
                     /translation="MAYSVDLRERAVSMIEKGKSKVEVAELLEIGIATLYRWLRKKAA
                     GESLRPSKNGSFIRKIDPKILEEYVKKNPDHTLAEMKQNLGFGINSIWYRLKQLRITL
                     KKSYTISRAQSRR"
                     /besthit="qcoverage 100 , hcoverage 100 , similarity 100
                     , identity 100 , evalue 2.74e-74 , alnlength 115 ,
                     Bacteria;Eukaryota , Proteobacteria;Arthropoda ,
                     WP_155968342.1 MULTISPECIES: IS630 transposase-related
                     protein [Wolbachia]"
     gene            17578..17889
                     /locus_tag="wEsol_00324"
     CDS             17578..17889
                     /locus_tag="wEsol_00324"
                     /codon_start=1
                     /transl_table=11
                     /fullproduct="qcoverage 100 , hcoverage 100 , similarity
                     100 , identity 100 , evalue 1.60e-74 , alnlength 103 ,
                     Bacteria , Proteobacteria , WP_155968470.1 transposase
                     [Wolbachia pipientis]"
                     /cog="COG3335 COG3335 132 Transposase and inactivated
                     derivatives"
                     /pfam="DDE_3 DDE_1"
                     /product="transposase"
                     /translation="MTFKGGCDKEVFNTWLEKMLLPKLPHGTTIVMDNATFHKTPKTK
                     ELIDNARCRLLYLPTYSPDLNPIEHCWNTIKSRLRPLMHKYTDLQLLVGNTIMEIYHS
                     F"
                     /besthit="qcoverage 100 , hcoverage 100 , similarity 100
                     , identity 100 , evalue 1.60e-74 , alnlength 103 ,
                     Bacteria , Proteobacteria , WP_155968470.1 transposase
                     [Wolbachia pipientis]"
ORIGIN
        1 tgccttaaca tactgatttt cgcaatgggc ttacctccgc taatgtggga atattgaagc
       61 aaaagtgatg tcattccagt gcttgacact gggatggctt tgttgcatag caactgaaaa
      121 aatctggtgg ctactgacaa aattcattat aaaatagcca tttaaccttg aaagaaaaaa
      181 tatgccacaa aaaatgaggg tcagtaacca tagcgaatat aacaaatttc tagaaaaaag
```

241 aggaaatatt tttcattata tcaacgaagc catagaaaag tggtacgaga atggtccaaa
301 aataccaggt ggcaacaata tttatagcga taaagttgta attttggtgc acataataac
361 ttgtttattt agaataggcc tgaggcaaac agttgggttt atagcaggat accttgagca
421 aattgggaaa aatttgcaag ttatcagcta ttcacaatca tcaagaaggt ttaagaaact
481 caatattaag atcaatgatt gcagaaaata atatggaaga catcgaaatt gctatagaca
541 gtacaggaat tagtatatac aacaacactc ctggtcatag caaagaaaat agcgaagata
601 gaaagtatcg cagctatgaa cagacaagaa aattgcatgt aatgttgaat acaaacagca
661 aaaaagccat agctgtaaaa tacagtaacg gctgatcact atggagcttg tgatttgcta
721 aaagaagtcg attttcagta tgtcataaaa gtactatatg cagatagggc atatgacaga
781 gaaaagctct ataagctgtg taatcaatat ggtataaaaa cgaaaattcc tccgaaaaac
841 aacgcagcag agcatccaaa gttagactat atggctgaga gaaattctgc agtcaagctc
901 ataaagtcat atgatgaaga tggtataaag aagtggaaaa aagagatgag ttatggaaaa
961 agatcttata tagaagtttt ttctcgcgac tgaaacaaac attcgggatt tagttttagg
1021 aataaatctg agacaaatag tgagaaggga actacttgtt aaatgctatt tgctcaataa
1081 gttcactgat attggtatgg ctaaatttga gatagcttca taaatttatt gtgcgttacc
1141 tactatccga agagcgatgc aacaaagcca tcccagtgtc agctacttgg atgacagagg
1201 ggggtgggat gacagaagat gatggagtga cagggagtaa tacaaaaagt ttcgtcatcc
1261 ccattgaatc ggctttaaaa tcgatgttca aagttgaaat ttccctagaa gatgcgggga
1321 aaatcagaaa aggtcaggaa gttgtattaa ataacttgcg taatttaaag aatcatgata
1381 tttgttgtac ggtggtgggt agtgtaccta ttgcaatctg cagttttatt tatggttgtg
1441 tgaaacctat tcgtgttttt aatatttaaa atgaggttta gatgtcgata acatccgaaa
1501 agaaaaagag tttgataaat atatacgcaa ttaaagaaga tgatacaggc tcatcttttg
1561 tacaatgtgc aattttgacc gagaggatca gtaacttaac tgagcatttt aaagtgcaca
1621 agcatgacca tcactctaag cgtggtttac ttatattgat aggtagaaga cgcaagcact
1681 taaattatat aaagcgtaaa tttggtaatg aagcctatca ggaattaata gagaagttag
1741 gcattagaaa ataatcgagg aatttaatat gtttaaaatt ataaaaaaat ctatagagtg
1801 gggtggtcgt gccttatctt tagaaacagg aaaaatagca cgccaagctc atggttcagt
1861 agttgtaaat tacggtgata cttctgtttt agtaactgtt gtacgtaaaa agaaggaaga
1921 aagcgttgat ttcctacctt taaatgtgca gtttatcgca aaaagctatg ccatgggtaa
1981 gatccctggc gggtttttta aaagagaagg caagccatct gatagagaaa ctttaatctc
2041 aagagtaata gatagaagta taagaccact attcccagaa ggatttcatg atgaaattag
2101 tgtggtgtgc aatctattaa cttatgatac agtcaatcct cctgaagtgc cagcattgat
2161 tggtgctgtt gcagctcttg caatttctgg tgttcccttt cactttacta tagctggagt
2221 gatggttggt tgtgatgaaa ataataacta tatactcaac ccttctgttc aagagatgaa
2281 agcaagcagc ttggatctgt tttgtctgg tgatgaaaat tcaattttaa tggttgaatc
2341 agaagtgaaa gagctctctg aagaaaatgt ttttaatgca ataaaatttg gccatgaaca
2401 ccttaagcct gtcattaagc tcataaaaga gtttgctgat acaattggca ataaacctga
2461 gagctttgcc cctattgatg catcagatat aacacaagag ctcgaaaaat acggtaaaga
2521 ttttgaaaaa gcatattcgc aaacagtaaa acaagagcga gttcaagctc tagaagcgat
2581 cagagagaat atattgaata ctcttaaaga aactggaaaa gacgaaaagt taattacgta
2641 tgcagtaaaa aactttgaaa gatctttagt acgtgaaata attaggaaga aaagtgtaag
2701 gatagacagt cgtaagcatg atgagatacg tcagatagaa gttgaagttg atattctgtc
2761 caaaactcac ggttctgcac tatttacaag aggcaatact caggcactgg ttgttactgc
2821 tcttggcacc actcaagatg agcaaattgt ggatgatatt gaaggggata gacgtgagca
2881 tttcatgttg cattataact ttccctcatt tgctgttgga gaaacttctg ctgcgcgtgc
2941 accaggaaga agagaaatcg gtcatggcaa acttgcttgg aaagcaattc atcctgtttt
3001 acccgataag tctgaatttc cttatacaat aagagtagtg tccgaaattt tggagtctga
3061 tggttcttct tctatggcaa cagtttgtgg gacttctctt gctttaatgg atacgggtgt
3121 gccaataaag gcccctgttg ctggaattgc tatgggtctc atcaaagata aagacgagta
3181 tgtaatactt tccgatatac ttggtgatga agattatctt ggtgatatgg actttaaagt
3241 agcaggaact agtgaagggg ttacggcgct acaaatggac atgaaaattt ctggtataag
3301 ctttgaaatt gttgaaaaat ctttagaaca ggcaaaagct ggaagattac atattttaga
3361 aaaaatgaat gcagtgattt cagaacacag tgatgatgtc aaagaccatg caccaaggat
3421 gttatcattt tacatagata aagacaaaat ttctgctgct ataggttcta aggggaaaaa
3481 tatacgcagt gtgtgtgaaa gaagtaatgc aaaaattgaa ataggagatg atggtaaagt
3541 ttctgttttt gctacgagtg gcactgaagc tgaaattgca aagagtatga tgattgattc
3601 aataacagaa ctagaacaag gttctatagt tgatgtcaag gttgtaagaa tagagaagtc
3661 tattgtagag cttgaatttc ttaatggcag aaaaggaaaa atgcacataa gtgaagtagc
3721 taatgaacat atagattcta tagagagcgt acttaaacaa gatgatactt tcaaagcact
3781 agtaattgac ttcgaaaaag gtgggtgtcc aaagttgtca agacgtcggg ttgaccaaga

3841 gacgggagag ttttttgaag gtgagcttta caacgaggaa aggaaagatg gtccaaatga
3901 cagggataat tattacaata attcatttag tagaaaacct ggaggaagtc accataagag
3961 gcctcctcgt cctcgttctg gtttcagcaa cagaaatagg ccgaaatttg gtaataatga
4021 ttcatcatca ggtttttatt aagagtaaac ttcttgcgtt acctctcgtc attccagtgc
4081 ttaacactgg aatgacaccc tttctggtga aggttgttct caaatcacaa tgttagtaca
4141 attgtggtgt tagctatagc taacccaagg tatccgttca gcggagtggt agaataaggt
4201 agacaaagca agataataaa gataatcata gagcaaaagt gaggtaatgt ggtaaacctt
4261 ttagaacttt gcaaaaattt acagcaaaaa atagaaaaac tggaagcaga gatagaaaac
4321 ttaaaagcag aaaataaagc tttaaagata gagaacgctg aattaaagga aaggctcggt
4381 ttaaattcaa aaaactcatc cctgcccagc tcaaaagagc tatacaaaat aaaaaaggac
4441 aagccaaaaa gcgaaaggaa tgttggcgat caggttgggc ataaaggaaa ttttcatgcc
4501 acaatggaag cggataaagt agtaaaaata gagttgccta atatttgcga atgcggagga
4561 gagcttgtaa tatgcgaaaa accttatgtt caccaaaaag ttgatcttcc ggaaattagg
4621 ccttatgtag tatagcattt ctcataatag tccgaacagg tcataggaag gcagaatcaa
4681 cagcacgacg aaaggattcg aatagaggaa tgtttttcct ggctctgttt tttatagcaa
4741 accaatgatg ctcaattttg ttgaaatctg gagaataagg cggcagatac aaaatttctg
4801 caccaacccc tttagcaagc tcgataatct tatcagactt atgaaaagta gcattgtcaa
4861 gaatgacagt ctgtccaggt tccaagatcg gtgtcaaaaa ttgctcaacc catccattaa
4921 aaacatccat attacagtgg ccttcaaagg ttaacggaac aactatcttt cttccactta
4981 aggctgcaac catactgatt cgttgagttt tctttccaga ttttaaggca taaaaccgct
5041 gtcctttctg gcaatatccg taggggtagt cttcggtgtt atcaatgcca gactcatcta
5101 tatacactaa gtttttaggt tcttttgtcg ctataatttt caaaaattca gcacgttttt
5161 cttcgtttct ttccttgtat ccatatgtct tttttgcgc gtaaatccaa tttttttaag
5221 ggctcgatag atcgtttgga tactaacgtt gttccaaagt ttagccattt ccgatagagt
5281 ctttcctccg tgttctctgg caaatttggc aaaggtatcc cagtcggtaa ttttgtgatt
5341 gtaaccccca tttccaagtt tttttgattg aaagtctcct gtttcttttc ttctttgctg
5401 ccactcccat aaagtagttc gtccaatctt aaatcttgct gccactgttt ctctgctttc
5461 tccttcgtct agcgcttcca ttgctttttt tcttaaatca taataccaat tctcactgca
5521 caacaaacag atagacaata atggaaaaaa agccatacta aactaagtgt aatagcgagg
5581 tataaatggc attaagatca aaattattgg atgaaaaagt agtggaatcg gcaaaagcaa
5641 tgctgaagaa agtaagaaac aacgcatacg ttacaaaaaa actaaatgct gtaattgcag
5701 caaaaaagca cagcataaca gcggtagcaa agatatgttg catttcaaga actgcactga
5761 ctgcgtggat aaagcaccta aaatttaaca gagaggaaaa attgtttgct ccccctcaac
5821 gtcgtagaaa aactagattg aatcaaagtc aacgtgaaca agttgaagca tggatacaag
5881 aaaatcctaa tatcaccatt aaagaaatga gaataaaaat ccaggaaaga tttggtttgg
5941 atatcagtaa gtccacagta caccgtaata tgcaaagaat gaaattctca tatattactc
6001 caagaccagt tcataatggt caagataaaa gcaaacaaga ggagtttaaa aaaaaatctc
6061 aacgaaacta ttggcaaata tccagaaaaa gagctatttt tctttgatga gtctcggttt
6121 ggcacacatt caaaaattgg acatgggtgg tttaaaaaag gcattaggac acaggttaaa
6181 ataaaattag gtaggcaaaa tttctatctc tatagtgcgg ttaatcctag aaatggagag
6241 agttctagct caaatgtcaa cactgattgc atgaatatat ttcttgagca gatgtcgcaa
6301 tatctaggaa tacgagagac ttttctcgtt atggactgtg ctagttggca taggtcaaaa
6361 aatttaaagg tacctaaaaa tatcgatatt atatacctac ctccatactc acctgagctc
6421 aatcctgttg agaggttttg gttatatata aaacagaaca ttttgtgcaa taaaatatac
6481 gatacaatta ccctgcttga gcgcactttg tgtaaatttg ttacctctct tacccactcc
6541 ataattaaac aactctgcaa tgtctcctat ttgtcctcct aataatgaga attggtataa
6601 cttgctgttt ttcaggttcc tcattacaaa tgttctccgt atcttttaga gaatcttcag
6661 aatttaagaa tgttgaacta tcccatgctc taagagaagg aatcgatgat gatcttttct
6721 gatcctgaat attagcctct gttagcgtac cttcaccagc acctaaagat agctgtgatt
6781 gatcaccagt ggattgactt agatctattt ccccggaaga tagctgtgat tgatcaccag
6841 tggattgact tgaatctatt ttccccggca ccaactgaaa cagcccccct tttcctccct
6901 ctctttccga tccttgcctg ggaggcctct ctaacccttt accacgactt cgcttgggac
6961 acccccgtgt ctgtgatgct tcagctaata gcgttcgagg aagctcaggg tcaatggcca
7021 gttcatggtc tttgttgatt acagagcttt ccatgatttt attcagcaca caataagaaa
7081 tgtcttgatt agcaaaatct attccgtata ttcctctcct gccaaatact ggtagatgtt
7141 ttggtgaaat acaatcttca acattattta acacggtcat accaaatact ttattaatat
7201 catccgcttg ttccttgctt aaaattttat aatatataat attatcaata atttccttag
7261 cagcaggtat aagaactgaa ttttcaccac tgcctgtata agaaacatga agtacagaat
7321 taattggtat acctactgta cgagacaaat caatcactgg aaactttaga ctctcaacag
7381 tttcatcctc agcacttgta acctcaaaat cacgacattc caaagatgct ttgtgtttta

7441 ctatgtcctt aatgaactta gccttattaa agatgctccc ctttatatac acacaattgt
7501 ccttgatttt caaaaaatca ttagtgcttt taggaccatt tttataattt tttatccttg
7561 acgagaatga ctctacaaaa taatcttgta actttttatc atagaaacgc atacaatata
7621 gataagattt cctgtcaaaa cgtacatcat atacgcgccc ttccatacat ctctcaccaa
7681 attcgtttcc catatcctct tttgcatctt ttgcaagccc atgggctagc aaacatttta
7741 gattaaaaac tacacctttg tctattggca ctgaaaactt atatccctcc ttatctttag
7801 ttaactgcct ttcattaagg ttaaaaggaa aaaactgtgt attaaaagac atattagcac
7861 cactttcact cccacaaaat gtttctacta tgcttttctt tatattgtgc aagtacttca
7921 tgcattgacc aacatccata tttttatcaa atgagataac aatacgatct tccttcttaa
7981 gaaaagtttg atctgtgctt tgtacgataa gattagtaat agcaacctca ttatgacaaa
8041 gaataatgtt aaatgctctt actaattctt gattcattat attaggcata ataccccctcc
8101 ctattcaaat taccataaat ttactgttac ttaccacttc tgccactacc ttgaccacta
8161 gattgatttt gcttccttct ctcctcctca cgcttaattc tctctgtaaa gtcatcaact
8221 ctccttggat ctatagcatc cgctctaatt cttcctcctc ctggtgcagt attatcgcca
8281 tattttcgtt tttcataacc tgaaccttta ttactatcca tattatcccc cagtaaaaat
8341 attatgtaac taatatacta aaaataaaat taagtgcaag cactttttta tatttttttt
8401 taaaaaattt tattcactct ttttcataaa acccaaattt caagattcca gcgtcacacg
8461 ctggaatgac aagtcattgg caaccctaag ttccctacgt cataccgcga ttcattcgcg
8521 gtatctcaag catagattcc gctaacacgt agcggaatga cgaactgtcg acatctatct
8581 atatgcccct ggattccagc gtcacgcgct ggaatgacac ctgtatttct ctttgccatc
8641 aatccctact ctttaatgtc ataccagtac ctctttttct tgtcatccca gtgtccagac
8701 actgggatcc agtctttttt gtcatctaaa gctaacacta gctatcccct cgatggtgct
8761 ttttctggtg cctttcctat tgaatccaca tgattattaa cttttacatc atcaatcaat
8821 ctttttggtg gttgagcagt actagcatta gactttccat tagaagcttg ctcttgccaa
8881 tcaccaattg gataattgtg tgcaggaaag ctatcccgat tgatttttct tgatttcttg
8941 ctactagttc tagaacctgc actctgtact ttttctggca cctcctccct aggcttctcc
9001 ggaactttct gtggacacac ctctttcttc ggttcctccg gcttcgcttt tttctcttct
9061 tctactttttg gttttatttc ctcgcggttc tcattgttta taacctcttt aaatatagaa
9121 ggcattatag gctttggtaa aagatcaagt tctggtttgc tcagaacaac cggtcttaca
9181 aatcctggtg aaatttcaaa aaggaaaact ggcactttag gtttttctct ttcgtctaac
9241 ttgatttcta cgttttcgca aacttcttta ttttcttcct ttaatgatgt tttttctgct
9301 tttttatctt ctttttctgt agatgcctct accgcagccg ccaactcaac tccaaacatc
9361 tctgcgaaca ttgtaacaat ccatccgaca acggccattt gtaacctgag tttcagatct
9421 tgcaatgcag ttaatctttc taacgccctt gcaaactctt cttctgataa ctcaccactg
9481 cctaattttt cctgcaattc ttttatctct ttgtccaacc tctgattgaa acttaaatcg
9541 cgtgcaaaaa tcttcttgat taactcctta agcttatcac gcaaaaactt aattatcgaa
9601 agaagccaat ttttttcttt cttctccttg agcatttggt agattttcgg atcatctaca
9661 tctacttcaa aaaggttgaa gagagattca aagaaattcc agaaatctcc gagtactctt
9721 ccttttttcac cagtgaagta atcctcatca tcaccttctc cgatatgtag ctctgcttga
9781 gatgatattt catcgataca actagaagcc tgacgctcac actcgagcct cctgagcaaa
9841 tcttgtctgg tgagctcctc tacgcactgc ctctattgct ctgatatagc taaagtgact
9901 tacgataatt tgaaaaacaa aaattcgtca ttccgctact tgttagcgga atctataccg
9961 cgaatgaatc gcggtatgac gtaggactgc tgtcatccca gtgcgtgaca ctgggatctc
10021 gttccttaac tttatagtgt aaattatttc aaattgtgtc tagctgcaag tataataatt
10081 tccatttata gataaagtat gttgcaaatg ttatgccaaa gagtataata aaatctatat
10141 caaatacttt acaagaaaaa gtggtgatag aggcaaaagc aaatattaat aaggataatt
10201 taagaaaagc gcatgctaga gtttgaaaaa acagcaagaa taaaaaatat tattgaaagg
10261 ttaaatggcg tatgatataa tatagtgtaa agatataaat aattttattg acatctttac
10321 attaatactt cttaaatatt tagtactgta gaattaaatt attaagtttt atgaagggag
10381 taaaaatggt ggaaagcttt tcaggtaaca acagaggcaa taattttatt gaggaggcaa
10441 aaataatgga aaatttttca gataataacg gaaacggtag ctcgatcttc caaggaaaac
10501 tcagggagca agatttctct gcagagcaga agttaaatga actaaaagca gccatagagg
10561 agctcgccag acaagatttg ctcagaaggc tcgagtgtga gcgtcaggct tctagttgta
10621 tcgatgaaat atcatctcaa gcagagctac atatcggaga aggtgatgat gaggattact
10681 tcactggtga aaaaggaaga gtactcggag atttctggaa tttctttgaa tctctcttca
10741 acctttttga agtagatgta gatgatccga aaatctacca aatgctcaag gagaagaaag
10801 aaaaaaattg gcttctttcg ataattaagt ttttgcgtga taagcttaag gagttaatca
10861 agaagatttt tgcacgcgat ttaagtttca atcagaggtt ggacaaagag ataaaagaat
10921 tgcaggaaaa attaggcagt ggtgagttat cagaagaaga gtttgcaagg gcgttagaaa
10981 gattaactgc attgcaagat ctgaaactca ggttacaaat ggccgttgtc ggatggattg

11041 ttacaatgtt cgcagagatg tttggagttg agttggcggc tgcggtagag gcatctacag
11101 aaaaagaaga taaaaaagca gaaaaaacat cattaaagga agaaaataaa gaagtttgcg
11161 aaaacgtaga aatcaagtta gacgaaagag aaaaacctaa agtgccagtt ttcctttttg
11221 aaatttcacc aggatttgta agaccggttg ttctgagcaa accagaactt gatcttttac
11281 caaagcctat aatgccttct atatttaaag aggttataaa caatgagaac cgcgaggaaa
11341 taaaaccaaa agtagaagaa gagaaaaaag cgaagccgga ggaaccgaag aaagaggtgt
11401 gtccacagaa agttccggag aagcctaggg aggaggtgcc agaaaaagta cagagtgcag
11461 gttctagaac tagtagcaag aaatcaagaa aaatcaatcg ggatagcttt cctgcacaca
11521 attatccaat tggtgattgg caagagcaag cttctaatgg aaagtctaat gctagtactg
11581 ctcaaccacc aaaaagattg attgatgatg taaaagttaa taatcatgtg gattcaatag
11641 gaaaggcacc agaaaaagca ccatcgaggg gatagctagt gttagcttta gatgacaaaa
11701 aagactggat cccagtgtct ggacactggg atgacaagaa aaagaggtac tggtatgaca
11761 ttaaagagta gggattgatg gcaaagagaa atacaggtgt cattccagcg cgtgacgctg
11821 gaatccaggg gcatatagat agatgtcgac agttcgtcat tccgctacgt gttagcggaa
11881 tctatgcttg agataccgcg aatgaatcgc ggtatgacgt agggaactta gggttgccaa
11941 tgacttgtca ttccagcgtg tgacgctgga atcttgaaat ttgggtttta tgaaaaagag
12001 tgaataaaat tttttaaaaa aaaatataaa aaagtgcttg cacttaattt tatttttagt
12061 atattagtta cataatattt ttactggggg ataatatgga tagtaataaa ggttcaggtt
12121 atgaaaaacg aaaatatggc gataatactg caccaggagg aggaagaatt agagcggatg
12181 ctatagatcc aaggagagtt gatgacttta cagagagaat taagcgtgag gaggagagaa
12241 ggaagcaaaa tcaatctagt ggtcaaggta gtggcagaag tggtaagtaa cagtaaattt
12301 atggtaattt gaatagggag gggtattatg cctaatataa tgaatcaaga attagtaaga
12361 gcatttaaca ttattctttg tcataatgag gttgctatta ctaatcttat cgtacaaagc
12421 acagatcaaa cttttcttaa gaaggaagat cgtattgtta tctcatttga taaaaatatg
12481 gatgttggtc aatgcatgaa gtacttgcac aatataaaga aaagcatagt agaaacattt
12541 tgtgggagtg aaagtggtgc taatatgtct tttaatacac agttttttcc ttttaacctt
12601 aatgaaaggc agttaactaa agataaggag ggatataagt tttcagtgcc aatagacaaa
12661 ggtgtagttt ttaatctaaa atgtttgcta gcccatgggc ttgcaaaaga tgcaaaagag
12721 gatatgggaa acgaatttgg tgagagatgt atggaagggc gcgtatatga tgtacgtttt
12781 gacaggaaat cttatctata ttgtatgcgt ttctatgata aaaagttaca agattatttt
12841 gtagagtcat tctcgtcaag gataaaaaat tataaaaatg gtcctaaaag cactaatgat
12901 tttttgaaaa tcaaggacaa ttgtgtgtat ataaagggga gcatctttaa taaggctaag
12961 ttcattaagg acatagtaaa acacaaagca tctttggaat gtcgtgattt tgaggttaca
13021 agtgctgagg atgaaactgt tgagagtcta aagtttccag tgattgattt gtctcgtaca
13081 gtaggtatac caattaattc tgtacttcat gtttcttata caggcagtgg tgaaaattca
13141 gttcttatac ctgctgctaa ggaaattatt gataatatta tatattataa aattttaagc
13201 aaggaacaag cggatgatat taataaagta tttggtatga ccgtgttaaa taatgttgaa
13261 gattgtattt caccaaaaca tctaccagta tttggcagga gaggaatata cggaatagat
13321 tttgctaatc aagacatttc ttattgtgtg ctgaataaaa tcatggaaag ctctgtaatc
13381 aacaaagacc atgaactggc cattgaccct gagcttcctc gaacgctatt agctgaagca
13441 tcacagacac gggggtgtcc caagcgaagt cgtggtaaag ggttagagag gcctcccagg
13501 caaggatcgg aaagagaggg aggaaaaggg gggctgtttc agttggtgcc ggggaaaata
13561 gattcaagtc aatccactgg tgatcaatca cagctatctt ccggggaaat agatctaagt
13621 caatccactg gtgatcaatc acagctatct ttaggtgctg gtgaaggtac gctaacagag
13681 gctaatattc aggatcagaa aagatcatca tcgattcctt ctcttagagc atgggatagt
13741 tcaacattct taaattctga agattctcta aaagatacgg agaacatttg taatgaggaa
13801 cctgaaaaac agcaagttac tgatgaaagt gatcgaaaaa gtagtgatga gtgttctaaa
13861 gcaaagccac aagatagtaa atctccggag aagaaacatg gggatggtct tttgtgtagt
13921 aattcgtcta taggtagtat gtggtcaaaa ctaaaaggca atgtgctacc ttctcttaag
13981 cgaaataaca aaggacgtgg ttcggtgtat tcgatatctt caagtgtaag tgctcctggg
14041 ggttctgtac ctgaaaaact acctgtttct gaatcatctt cttcattccc aggcagaata
14101 aaagcaaaaa gtaatccacc ctcagcggtg acttctccta cgcatgaagg tgtaccagag
14161 tggcaacaac gtcttaatcc tgatcagaga gaggaactac gcccatttct ttctcctgag
14221 gagcgaaaga agctaaactc caactatttc tcccctgggg aggagtggca tttatatttg
14281 acgcgtactc tttcggggaa aagggagcta agcagatttc tatgtgagag tgaagaatca
14341 cagttacggc gacttgcaga agcacaatta tcgtcacctg ataactctag ttctaaaaaa
14401 gttgatagag atagtggttt ttgttctatg ggttctcctg ctgtgcaaca taagggaagt
14461 gtcccttcag aggttaaaga gtgtgaggta gagcctgtat cagaggcttt gagaaaataa
14521 atttaggttt ttagatatcc acccaagtgt tttattaggg ttggagccaa aatccctgac
14581 cctgaaagca ctggtgttag tttttctcat ccattgcaga tattgcgcgg tactatccac

14641 ttgatcatca tggcgagctt ctgggaacat taaaatttcg tactcgaaat cgctgagcca
14701 tactgcttgc tgtggcaaaa aaaaactacc agactctatc accggaacaa ttttgagtga
14761 gtttgtcatt gtgtggaatt atttcgataa tcggtagatc gctatttgcc tttagctctt
14821 gtgttaattg ttgaccactc gtttttgctt cgatcaaaat tgcgtgtggc gtccatcttg
14881 cagctagtga cagaacttgc tctttaagct ttgggtactc aagctttgct cgatatacat
14941 caagtaaata gaatttatta tctatttttg cccaggtggt gcagatgcta aagtcgctgg
15001 aatcgtttgt tgaaactgca gtgtcccagc tttgagttac atgtgaggga ttatcaggga
15061 aatttttata gcgctttaac cactcttgtt ttattatacc acttgaaagc ggcagggggt
15121 tttgttggta ttgagcagca aaagcgtaac tcccgagttc aacttttatc atctcaactt
15181 cttctcttcc tccctccagg ggatataata actcattctc ttctcttgag tataatactc
15241 ttgcaggtag tgtcattcca gcacttgatg ctggaatcca gtcttttgcg gttgaatttt
15301 ctggatccca gtgtctgggc actgggatga caggaggggg ggttggaatg acaccagtat
15361 ggttattaat tgaataaata acctccttat tttcagaaat cattggcaaa caaatgtgat
15421 gccatatgtt ttttggctta gataagaggt gcccagttaa gtcttccagg tggagcctat
15481 gcatcacaag cacaattact ccttttttct atcgttgagt ctcgttacta aagtctgatc
15541 gaaccagttt gtggcacgtt ttctaaatgt ttcactcaaa gcttgagcag ggttaagcgg
15601 atcatccaca ataataaagt caccaccttc accggttaat gttcctccaa ctgatgttgc
15661 gattctgtat cctctctgca ctgtttggaa tttatattta gtgttctggt ctttggatag
15721 ttctacctct ggaaatagtt ctttatacca atcagattgc attatgcacc tcgtatcaag
15781 cgaatgtttt tcactaagca acttagaata acttgcaact attattcttg cagttgccca
15841 gtatccatgc gggccacgca acactcatac atatggactt cattgaacgc ggaggcatat
15901 tgaatattat gcgccttact tcgccagcat tcgctgcttc cagcctgtct gctatgactt
15961 ttatgtactg ataatcatta tactcacatc ccggcactac tgtttgaaag cacagttcaa
16021 tgaattttag aaaatttata ttatccatat gtgtatttta gaggttgtcc ggaaataagt
16081 aaaaggctat aatatctgaa attttagtac ggggtaaaga tgagaaaaaa gtatccaaca
16141 gatctaagcg aaagggaatg ggcaagaata gaaaaacact tcagagtatc atacaagaaa
16201 ggaggaaggc cgccaaagta tagcaaaaga gaaatattag aagcaatttt ctatgtattg
16261 cgtacagggt gtcaatggcg gtatttacca aatgattttc cgctatggaa gactgtgtat
16321 gagcagttca ggcaatggaa gaagcaggga atttttgaga aaatgaatta tgaaattaca
16381 aaatatagta gaagaaaaat aggaaggaat gagcagccga gtgcctgtat agtagatagt
16441 caatctgtaa agactacaga aaaggggga tcaaaggcta tgatggaagt aaaaaagtaa
16501 agggtagaaa aaggcatata attacagaca ctcagggttt tatactaggt tgttacgtag
16561 gcgctgctaa cgaaaatgat agagatggta ttaaaatagc attaaacaat atgagaacaa
16621 aatatactaa agttaaaaaa atgtgggctg acatgggata ccaaggaaga aatttaaaga
16681 atcacataaa ggaagaatat gacatagata ttgaaattgt taaaaggcct ccatgtagat
16741 tttgggtgca caaagatacg ccacctgagc tactaccaac aagagaacaa gggtttaaag
16801 tacagccaag aagatgggtt gtagaaagga cttttgcttg ggttaatagg aatagaaggc
16861 tatcgaagga gtatgattta ctcacaacat ccactgagaa tttcatatac ctagctatga
16921 gtagggttat gttaaagagg gaatatgctt gaatttacta gtttccggac aacctcatat
16981 agtacttccc aaagtaaatg ataaaggtat aataagctat ttgagttata atggcgtata
17041 gtgtggattt aagggaaagg gcagtatcga tgatagaaaa agggaagtca aaggttgagg
17101 tggcagagct tttggagata ggaatagcaa ctctgtaccg ctggttaaga aagaaagccg
17161 ctggtgaaag cctaaggcca tcgaaaaacg gcagctttat tcgaaaaata gacccaaaaa
17221 tactcgaaga atatgttaaa aagaatccag atcatacgct ggcagagatg aaacaaaatc
17281 ttggatttgg aataaattca atttggtata ggctgaaaca gctaagaatt actttaaaaa
17341 aaagttacac tatatcaaga gcgcaatcaa gaagataggc agcgatttac cgataaaatc
17401 tcaaaaatag accattccag catcttatat atagatgaag caggagttga taataggtta
17461 taccgagagt atggacgtgc tccaatagga aagaaaattt atgcagatat tccaggaaga
17521 aaacgagaga gaatcagtat aataggcggg tggattggaa agagatttat tgcaccaatg
17581 actttcaaag gtgggtgtga caaagaggta ttcaatacat ggttagagaa gatgttatta
17641 cctaaattgc cacatggtac tacaatagtt atggacaatg ccacattcca taaaactccc
17701 aaaacaaagg agttaataga taatgctaga tgccgtttgc tctatctacc aacatattca
17761 ccagatttga accctataga gcattgttgg aataccatca aaagccgcct cagaccttta
17821 atgcataaat atacagactt acaacttttg gttggtaata ccataatgga aatttatcat
17881 tcattttaga aaatactata gtgaatggaa tgtagacttt aatagttttg atttgtcttg
17941 taagattgag gtgttataat gacaggctta caatgctgct aatttagtaa ataacctacg
18001 agtgctattt cttcaacttt atcgaagtaa agaatt
//
LOCUS JAQZAU010000040 2750 bp dna linear ENV 17-MAR-2023
DEFINITION MAG: Wolbachia pipientis isolate wEsol contig_3091, whole genome

```
            shotgun sequence.
ACCESSION   JAQZAU010000040 JAQZAU010000000
VERSION     JAQZAU010000040.1
DBLINK      BioProject: PRJNA929258
            BioSample: SAMN33142973
KEYWORDS    WGS; ENV; Metagenome Assembled Genome; MAG.
SOURCE      Wolbachia pipientis (insect metagenome)
  ORGANISM  Wolbachia pipientis
            Bacteria; Pseudomonadota; Alphaproteobacteria; Rickettsiales;
            Anaplasmataceae; Wolbachieae; Wolbachia.
REFERENCE   1  (bases 1 to 2750)
  AUTHORS   Fiutek,N., Couger,B.M., Roy,S., de la Torre,J.R. and Connor,E.F.
  TITLE     Genomic assessment of the contribution of the Wolbachia
            endosymbiont of Eurosta solidaginis to gall-induction
  JOURNAL   International Journal Molecular Sciences
REFERENCE   2  (bases 1 to 2750)
  AUTHORS   Fiutek,N., Couger,B.M., Roy,S., de la Torre,J.R. and Connor,E.F.
  TITLE     Direct Submission
  JOURNAL   Submitted (10-FEB-2023) Biology, San Francisco State University,
            1600 Holloway Ave, San Francisco, CA 94132, USA
COMMENT     ##Genome-Assembly-Data-START##
            Assembly Date          :: JUN-2019
            Assembly Method        :: FLYE v. 06-2019
            Genome Representation  :: Full
            Expected Final Version :: Yes
            Genome Coverage        :: 100.0x
            Sequencing Technology  :: Illumina HiSeq; PacBio Sequel
            ##Genome-Assembly-Data-END##
FEATURES             Location/Qualifiers
     source          1..2750
                     /organism="Wolbachia pipientis"
                     /mol_type="genomic DNA"
                     /submitter_seqid="contig_3091"
                     /isolate="wEsol"
                     /isolation_source="galls on Solidago altissima"
                     /host="Eurosta solidaginis MN-2018-EFC"
                     /db_xref="taxon:955"
                     /environmental_sample
                     /country="USA: Cowling Arboretum, Carleton College,
                     Northfield, MN"
                     /lat_lon="44.48 N 93.13 W"
                     /altitude="280 m"
                     /collection_date="2018-11"
                     /metagenome_source="insect metagenome"
                     /note="metagenomic"
     gene            complement(<1..455)
                     /locus_tag="wEsol_00325"
     CDS             complement(<1..455)
                     /locus_tag="wEsol_00325"
                     /codon_start=1
                     /transl_table=11
                     /product="hypothetical protein"
                     /pfam="Pfam-B_19525"
                     /translation="MKAIFADKGNNSGLVKNVLSGISSEIDLKMAEKAKSGEIEHTSG
                     WQVQKEFINNLNEKSLHNLLTQRSLPKGSYFIDSVFSEHYDEIKKVIKECRENHLYSS
                     VSNMFDRAEKKANRPSVKSLTRSVSSSFSSLGRKGSEQSNSLSKSNSLN"
                     /besthit="qcoverage 100 , hcoverage 23.59375 , similarity
                     100 , identity 100 , evalue 1.21e-89 , alnlength 151 ,
                     Bacteria , Proteobacteria , AGK00141.1 hypothetical
                     protein wHa_07010 [Wolbachia endosymbiont of Drosophila
```

```
                     simulans wHa]"
     gene            complement(541..1098)
                     /locus_tag="wEsol_00326"
     CDS             complement(541..1098)
                     /locus_tag="wEsol_00326"
                     /codon_start=1
                     /transl_table=11
                     /product="hypothetical protein"
                     /pfam="Pfam-B_6621"
                     /translation="MLHNEKNSNSVGTQQNQPEDSFDLNKTMQNLPNSDIANDPKKLK
                     ERITDLLKGDTEFSKMKQGDKILVVSAASGLFTAVLPLLAVGATLAIPGAIVGLALYF
                     AVKVAVKTVQSGYKGLKWSAEKTVDGAKYTAGKVKDASTYAAGKVRDGAVHVKDKVKE
                     GYEHSVDSLKKGASFVGEGVANKVP"
                     /besthit="qcoverage 98.9189189189189 , hcoverage
                     36.094674556213 , similarity 99.5 , identity 99.5 ,
                     evalue 1.24e-71 , alnlength 183 , Bacteria ,
                     Proteobacteria , WP_155969361.1 MULTISPECIES: actin
                     bundler [Wolbachia]"
     gene            1254..1469
                     /locus_tag="wEsol_00327"
     CDS             1254..1469
                     /locus_tag="wEsol_00327"
                     /codon_start=1
                     /transl_table=11
                     /fullproduct="qcoverage 100 , hcoverage 100 , similarity
                     100 , identity 100 , evalue 2.45e-41 , alnlength 71 ,
                     Bacteria , Proteobacteria , WP_006279848.1 MULTISPECIES:
                     hypothetical protein [Wolbachia]"
                     /translation="MKYRKRTLIFLAVITLIIIALLFRYNSHFHKDVVHLKSDNKEFK
                     KKPENSIESTILHREKEVYDHVFHPDD"
                     /product="hypothetical protein"
                     /besthit="qcoverage 100 , hcoverage 100 , similarity 100
                     , identity 100 , evalue 2.45e-41 , alnlength 71 ,
                     Bacteria , Proteobacteria , WP_006279848.1 MULTISPECIES:
                     hypothetical protein [Wolbachia]"
     gene            1559..2587
                     /locus_tag="wEsol_00328"
     CDS             1559..2587
                     /locus_tag="wEsol_00328"
                     /codon_start=1
                     /transl_table=11
                     /fullproduct="qcoverage 97.6608187134503 , hcoverage
                     97.0930232558139 , similarity 100 , identity 100 , evalue
                     1.43e-227 , alnlength 334 , Bacteria , Proteobacteria ,
                     WP_010962866.1 MULTISPECIES: FtsH protease activity
                     modulator HflK [Wolbachia]"
                     /cog="HflC COG0330 291 Membrane protease subunits,
                     stomatin/prohibitin homologs"
                     /pfam="Band_7 Pfam-B_5708"
                     /tigr="TIGR01933 hflK 261 HflK protein"
                     /product="FtsH protease
                     activity modulator HflK"
                     /translation="MIIILGIWGKKPVGNKTPNNEDILSKAVSDIRFFLNGLTRNRGK
                     KPYFIIFIILLFYACTGFYIVHPSEESIELTFGKYSNTETPGLRYHFPYPIGKVFKVN
                     VKEVNREEIGVSSSYGRDTDRGEGVMLTGDENIVNVNFEVQWRVRDAKDYLFKVRDYK
                     PGFSVKNAAESAMREIIGKNTISFALGQGRPEISRDTRILLQQILDGYQMGIEILSVQ
                     MKKIDPPEKVISSFRDVQSARADKERTINEAYAYNNDIIPRAKGEAIKIKLDAQAYEN
                     EVINEAKGNANRFLSLYEEYRQNPSLVKNRIYLETMENIFSKVDKVVVTDDLKGMFSY
                     LPLTNLGK"
                     /besthit="qcoverage 97.6608187134503 , hcoverage
```

```
                     97.0930232558139 , similarity 100 , identity 100 , evalue
                     1.43e-227 , alnlength 334 , Bacteria , Proteobacteria ,
                     WP_010962866.1 MULTISPECIES: FtsH protease activity
                     modulator HflK [Wolbachia]"
     gene            2590..>2750
                     /locus_tag="wEsol_00329"
     CDS             2590..>2750
                     /locus_tag="wEsol_00329"
                     /codon_start=1
                     /transl_table=11
                     /product="hypothetical protein"
                     /tigr="TIGR01932 hflC 318 HflC protein"
                     /translation="MSSNIKIVFVSVFVVLLIALSNSIFVVQETKQAIVIQLGKVVRD
                     VRESGLYFK"
                     /cog="HflC COG0330 291 Membrane protease subunits,
                     stomatin/prohibitin homologs"
                     /besthit="qcoverage 100 , hcoverage 18.2758620689655 ,
                     similarity 100 , identity 100 , evalue 3.82e-21 ,
                     alnlength 53 , Bacteria , Proteobacteria , WP_015589224.1
                     MULTISPECIES: protease modulator HflC [unclassified
                     Wolbachia]"
ORIGIN
        1 gaatttagtg aattgctttt tgacaagctg ttagattgtt cagagccttt tcttcccagt
       61 gaagagaatg atgatgacac agacctagtt aaacttttga cagaaggcct gttagctttt
      121 ttctctgctc tgtcaaacat attgcttacg cttgagtaca aatgattttc tctacattct
      181 ttaataacct tcttaatttc atcgtaatgt tcagagaaaa cactgtcgat gaaataacta
      241 cctttaggta atgacctttg tgttaacaag ttatgcaagc ttttttcatt taaattatta
      301 atgaattctt tctgtacttg ccatccagaa gtatgttcaa tctcccctga ttttgccttc
      361 tctgccattt ttaaatcaat ttctgacgat attccagaaa gtacattttt cactaaccca
      421 gaattatttc ctttatcagc aaaaattgct ttcatatcat ttcttatttc gtcagatttc
      481 ttaattgcat ctggagtaaa cctttttacc ttatctaaat agtccgtctc accagatgcc
      541 ttatggaact ttatttgcca ctccttcgcc tacaaaacta gcgccttttt tcagtgaatc
      601 tactgaatgt tcataacctt ctttcacctt atctttaaca tgcacagcac catctctaac
      661 ttttccggca gcgtatgttg atgcgtcctt aactttccca gcagtatatt ttgccccatc
      721 aactgttttt tctgctgacc acttgagccc tttatatcca gattgaactg tttttacagc
      781 gacttttaca gcgaaatata aagcaagacc aactatagca cctggtatag caagtgttgc
      841 accaactgcc aaaagtggta gtactgccgt aaataatcca cttgctgcac ttactacaag
      901 aatcttatct ccttgtttca tcttagagaa ttctgtatcc ccttttaaca ggtcagtaat
      961 tctttctttt agcttcttgg gatcattagc aatatctgaa tttggtagat tttgcatcgt
     1021 tttattcaaa tcaaaactat cttctggttg gttttgttga gttccaacag aatttgaatt
     1081 tttttcatta tgtaacattt taaacctcac tattaattaa atatattata attgtattcc
     1141 ttgattctta agttagtgtt aaattgattg ccagtattta tttgtcttca aataatttcg
     1201 cgtgacgtgt tttgtggtaa catagaaaat taggtcaaga agctgcagca tttatgaagt
     1261 atagaaaaag aacactcatc tttttggctg ttataacttt aataataata gctttactat
     1321 ttaggtataa ttcccatttt cacaaagacg tagtgcactt aaagagtgac aacaaagaat
     1381 tcaaaaaaaa gccagaaaat tccatagagt cgaccatatt acatagagaa aaggaagtgt
     1441 acgatcatgt tttccatcct gatgattaat ttgtaaataa aatagcaatt tatgtttgca
     1501 ttttcttatt aaagaagtaa tattagcgta ttaattatag aatcaatcat tatgtttgat
     1561 gataataatc cttggaatct ggggaaagaa accggtaggg aacaaaactc ctaataatga
     1621 agatatttta agtaaagctg tatctgatat aaggttcttt cttaatggat taaccagaaa
     1681 caggggcaaa aaaccttatt tcatcatttt cattattttg ctgttctatg cttgcactgg
     1741 cttttatatt gtccatccta gtgaagaaag tatagaactt acctttggta aatattctaa
     1801 tacggaaaca cctggtttgc gttatcactt cccctaccct attggcaagg tttttaaagt
     1861 gaacgttaag gaagtaaatc gtgaagaaat tggggtaagt agttcttatg ggcgagatac
     1921 agatcgcggt gaaggcgtga tgctaactgg agatgaaaat atagtcaacg ttaacttcga
     1981 ggttcaatgg cgcgttagag atgctaagga ctatttattc aaagtgcggg attacaaacc
     2041 cggtttcagt gttaaaaatg ctgctgaaag tgccatgaga gaaataatag gtaaaaacac
     2101 gatctctttt gcacttggtc aaggcagacc agaaatttct agagatacta gaattctatt
     2161 gcagcagatt cttgatggat accaaatggg tatagagatt ttatctgttc aaatgaaaaa
     2221 aattgatcca ccagaaaaag taatcagttc gtttagagat gtacaaagtg ctcgtgcaga
```

```
     2281 caaagagcgt actataaacg aagcatatgc ttataataat gatattatac ctcgagcaaa
     2341 gggtgaagcg ataaagataa aattagatgc acaagcatat gaaaatgaag taataaatga
     2401 agcaaaaggt aatgcaaatc gctttttatc tctttatgag gaatatagac agaatccttc
     2461 tctcgttaag aatcgtattt atcttgaaac tatggaaaat attttcagta aggtagacaa
     2521 agttgttgta actgatgatc tgaaaggtat gttttcttat ttacctctta caaatttagg
     2581 aaaataacca tgagtagtaa tattaaaatt gtttttgttt ctgtatttgt tgttttattg
     2641 attgctttat ctaattcaat atttgttgtg caagaaacaa agcaagcaat agttatacaa
     2701 ctgggtaaag ttgtaaggga tgttagggaa agtggcttat attttaagtt
//
LOCUS       JAQZAU010000041          930 bp    dna     linear   ENV 17-MAR-2023
DEFINITION  MAG: Wolbachia pipientis isolate wEsol contig_31005, whole genome
            shotgun sequence.
ACCESSION   JAQZAU010000041 JAQZAU010000000
VERSION     JAQZAU010000041.1
DBLINK      BioProject: PRJNA929258
            BioSample: SAMN33142973
KEYWORDS    WGS; ENV; Metagenome Assembled Genome; MAG.
SOURCE      Wolbachia pipientis (insect metagenome)
  ORGANISM  Wolbachia pipientis
            Bacteria; Pseudomonadota; Alphaproteobacteria; Rickettsiales;
            Anaplasmataceae; Wolbachieae; Wolbachia.
REFERENCE   1  (bases 1 to 930)
  AUTHORS   Fiutek,N., Couger,B.M., Roy,S., de la Torre,J.R. and Connor,E.F.
  TITLE     Genomic assessment of the contribution of the Wolbachia
            endosymbiont of Eurosta solidaginis to gall-induction
  JOURNAL   International Journal Molecular Sciences
REFERENCE   2  (bases 1 to 930)
  AUTHORS   Fiutek,N., Couger,B.M., Roy,S., de la Torre,J.R. and Connor,E.F.
  TITLE     Direct Submission
  JOURNAL   Submitted (10-FEB-2023) Biology, San Francisco State University,
            1600 Holloway Ave, San Francisco, CA 94132, USA
COMMENT     ##Genome-Assembly-Data-START##
            Assembly Date          :: JUN-2019
            Assembly Method        :: FLYE v. 06-2019
            Genome Representation  :: Full
            Expected Final Version :: Yes
            Genome Coverage        :: 100.0x
            Sequencing Technology  :: Illumina HiSeq; PacBio Sequel
            ##Genome-Assembly-Data-END##
FEATURES             Location/Qualifiers
     source          1..930
                     /organism="Wolbachia pipientis"
                     /mol_type="genomic DNA"
                     /submitter_seqid="contig_31005"
                     /isolate="wEsol"
                     /isolation_source="galls on Solidago altissima"
                     /host="Eurosta solidaginis MN-2018-EFC"
                     /db_xref="taxon:955"
                     /environmental_sample
                     /country="USA: Cowling Arboretum, Carleton College,
                     Northfield, MN"
                     /lat_lon="44.48 N 93.13 W"
                     /altitude="280 m"
                     /collection_date="2018-11"
                     /metagenome_source="insect metagenome"
                     /note="metagenomic"
     gene            73..453
                     /locus_tag="wEsol_00330"
     CDS             73..453
                     /locus_tag="wEsol_00330"
```

```
                     /codon_start=1
                     /transl_table=11
                     /fullproduct="qcoverage 100 , hcoverage 100 , similarity
                     100 , identity 100 , evalue 1.16e-87 , alnlength 126 ,
                     Bacteria , Proteobacteria , AOV87106.1 transposase
                     [Wolbachia endosymbiont of Drosophila incompta]"
                     /cog="COG3293 COG3293 124 Transposase and inactivated
                     derivatives"
                     /pfam="DUF4096"
                     /product="transposase"
                     /translation="MRKKYPTDLSEREWARIEKHFRVSYKKGGRPPKYSKREILEAIF
                     YVLRTGCQWRYLPNDFPLWKTVYEQFRQWKKQGIFEKMNYEITKYSRRKIGRNEQPSA
                     CIVDSQSVKTTEKGGSKAMMEVKK"
                     /besthit="qcoverage 100 , hcoverage 100 , similarity 100
                     , identity 100 , evalue 1.16e-87 , alnlength 126 ,
                     Bacteria , Proteobacteria , AOV87106.1 transposase
                     [Wolbachia endosymbiont of Drosophila incompta]"
     gene            564..905
                     /locus_tag="wEsol_00331"
     CDS             564..905
                     /locus_tag="wEsol_00331"
                     /codon_start=1
                     /transl_table=11
                     /fullproduct="qcoverage 100 , hcoverage 100 , similarity
                     100 , identity 100 , evalue 1.53e-79 , alnlength 113 ,
                     Bacteria , Proteobacteria , AAS13810.1 transposase, IS5
                     family, OrfB [Wolbachia endosymbiont of Drosophila
                     melanogaster]"
                     /cog="COG3293 COG3293 124 Transposase and inactivated
                     derivatives"
                     /pfam="DDE_Tnp_1_2 DDE_Tnp_1"
                     /product="transposase, IS5 family, OrfB"
                     /translation="MRTKYTKVKKMWADMGYQGRNLKNHIKEEYDIDIEIVKRPPCRF
                     WVHKDTPPELLPTREQGFKVQPRRWVVERTFAWVNRNRRLSKEYDLLTTSTENFIYLA
                     MSRVMLKREYA"
                     /besthit="qcoverage 100 , hcoverage 100 , similarity 100
                     , identity 100 , evalue 1.53e-79 , alnlength 113 ,
                     Bacteria , Proteobacteria , AAS13810.1 transposase, IS5
                     family, OrfB [Wolbachia endosymbiont of Drosophila
                     melanogaster]"
ORIGIN
        1 actttgacac taagaggttg tccggaaata agtaaaaggc tataatatct gaaattttag
       61 tacggggtaa agatgagaaa aaagtatcca acagatctaa gcgaaaggga atgggcaaga
      121 atagaaaaac acttcagagt atcatacaag aaaggaggaa ggccgccaaa gtatagcaaa
      181 agagaaatat tagaagcaat tttctatgta ttgcgtacag ggtgtcaatg gcggtattta
      241 ccaaatgatt ttccgctatg gaagactgtg tatgagcagt tcaggcaatg gaagaagcag
      301 ggaatttttg agaaaatgaa ttatgaaatt acaaaatata gtagaagaaa aataggaagg
      361 aatgagcagc cgagtgcctg tatagtagat agtcaatctg taaagactac agaaaagggg
      421 ggatcaaagg ctatgatgga agtaaaaaag taaagggtag aaaaaggcat ataattacag
      481 acactcaggg ttttatacta ggttgttacg taggcgctgc taacgaaaat gatagagatg
      541 gtattaaaat agcattaaac aatatgagaa caaaatatac taaagttaaa aaaatgtggg
      601 ctgacatggg ataccaagga agaaatttaa agaatcacat aaaggaagaa tatgacatag
      661 atattgaaat tgttaaaagg cctccatgta gattttgggt gcacaaagat acgccacctg
      721 agctactacc aacaagagaa caagggttta aagtacagcc aagaagatgg gttgtagaaa
      781 ggacttttgc ttgggttaat aggaatagaa ggctatcgaa ggagtatgat ttactcacaa
      841 catccactga gaatttcata tacctagcta tgagtagggt tatgttaaag agggaatatg
      901 cttgaattta ctagtttccg gacaacctct
//
LOCUS       JAQZAU010000042            2288 bp    dna     linear   ENV 17-MAR-2023
DEFINITION  MAG: Wolbachia pipientis isolate wEsol contig_31115, whole genome
```

```
            shotgun sequence.
ACCESSION   JAQZAU010000042 JAQZAU010000000
VERSION     JAQZAU010000042.1
DBLINK      BioProject: PRJNA929258
            BioSample: SAMN33142973
KEYWORDS    WGS; ENV; Metagenome Assembled Genome; MAG.
SOURCE      Wolbachia pipientis (insect metagenome)
  ORGANISM  Wolbachia pipientis
            Bacteria; Pseudomonadota; Alphaproteobacteria; Rickettsiales;
            Anaplasmataceae; Wolbachieae; Wolbachia.
REFERENCE   1  (bases 1 to 2288)
  AUTHORS   Fiutek,N., Couger,B.M., Roy,S., de la Torre,J.R. and Connor,E.F.
  TITLE     Genomic assessment of the contribution of the Wolbachia
            endosymbiont of Eurosta solidaginis to gall-induction
  JOURNAL   International Journal Molecular Sciences
REFERENCE   2  (bases 1 to 2288)
  AUTHORS   Fiutek,N., Couger,B.M., Roy,S., de la Torre,J.R. and Connor,E.F.
  TITLE     Direct Submission
  JOURNAL   Submitted (10-FEB-2023) Biology, San Francisco State University,
            1600 Holloway Ave, San Francisco, CA 94132, USA
COMMENT     ##Genome-Assembly-Data-START##
            Assembly Date          :: JUN-2019
            Assembly Method        :: FLYE v. 06-2019
            Genome Representation  :: Full
            Expected Final Version :: Yes
            Genome Coverage        :: 100.0x
            Sequencing Technology  :: Illumina HiSeq; PacBio Sequel
            ##Genome-Assembly-Data-END##
FEATURES             Location/Qualifiers
     source          1..2288
                     /organism="Wolbachia pipientis"
                     /mol_type="genomic DNA"
                     /submitter_seqid="contig_31115"
                     /isolate="wEsol"
                     /isolation_source="galls on Solidago altissima"
                     /host="Eurosta solidaginis MN-2018-EFC"
                     /db_xref="taxon:955"
                     /environmental_sample
                     /country="USA: Cowling Arboretum, Carleton College,
                     Northfield, MN"
                     /lat_lon="44.48 N 93.13 W"
                     /altitude="280 m"
                     /collection_date="2018-11"
                     /metagenome_source="insect metagenome"
                     /note="metagenomic"
     gene            215..445
                     /locus_tag="wEsol_00332"
     CDS             215..445
                     /locus_tag="wEsol_00332"
                     /codon_start=1
                     /transl_table=11
                     /product="hypothetical protein"
                     /pfam="DEDD_Tnp_IS110 Pfam-B_8792"
                     /translation="MVTSYQNFIGIDIGKFKNVVAIHKQKNAVEFDNNASGWQQLFQE
                     FSDIVFSKMNDKFPLWYYQPKVVSLYIYALKV"
                     /besthit="qcoverage 64.4736842105263 , hcoverage
                     59.0361445783133 , similarity 100 , identity 98.0 ,
                     evalue 7.61e-26 , alnlength 49 , Bacteria ,
                     Proteobacteria , WP_213863621.1 transposase, partial
                     [Wolbachia endosymbiont of Ceratitis capitata]"
```

```
gene            complement(366..677)
                /locus_tag="wEsol_00333"
CDS             complement(366..677)
                /locus_tag="wEsol_00333"
                /codon_start=1
                /transl_table=11
                /fullproduct="qcoverage 100 , hcoverage 100 , similarity
                100 , identity 100 , evalue 1.60e-74 , alnlength 103 ,
                Bacteria , Proteobacteria , WP_155968470.1 transposase
                [Wolbachia pipientis]"
                /cog="COG3335 COG3335 132 Transposase and inactivated
                derivatives"
                /pfam="DDE_3 DDE_1"
                /product="transposase"
                /translation="MTFKGGCDKEVFNTWLEKMLLPKLPHGTTIVMDNATFHKTPKTK
                ELIDNARCRLLYLPTYSPDLNPIEHCWNTIKSRLRPLMHKYTDLQLLVGNTIMEIYHS
                F"
                /besthit="qcoverage 100 , hcoverage 100 , similarity 100
                , identity 100 , evalue 1.60e-74 , alnlength 103 ,
                Bacteria , Proteobacteria , WP_155968470.1 transposase
                [Wolbachia pipientis]"
gene            complement(1117..1224)
                /locus_tag="wEsol_00334"
CDS             complement(1117..1224)
                /locus_tag="wEsol_00334"
                /codon_start=1
                /transl_table=11
                /product="hypothetical protein"
                /pfam="HTH_Tnp_IS630"
                /translation="MAYSVDLRERAVSMIEKGKSKVEVAELLEIGIATL"
                /besthit="qcoverage 100 , hcoverage 52.2388059701493 ,
                similarity 100 , identity 100 , evalue 3.26e-13 ,
                alnlength 35 , Bacteria , Proteobacteria , WP_213863574.1
                IS630 transposase-related protein, partial [Wolbachia
                endosymbiont of Ceratitis capitata]"
gene            1554..2075
                /locus_tag="wEsol_00335"
CDS             1554..2075
                /locus_tag="wEsol_00335"
                /codon_start=1
                /transl_table=11
                /fullproduct="qcoverage 100 , hcoverage 100 , similarity
                100 , identity 100 , evalue 4.69e-112 , alnlength 173 ,
                Bacteria , Proteobacteria , RLT59977.1 transposase
                IS116/IS110/IS902 family protein [Wolbachia endosymbiont
                of Drosophila ananassae]"
                /cog="COG3547 COG3547 303 Transposase and inactivated
                derivatives"
                /pfam="Transposase_20"
                /product="transposase IS116/IS110/IS902 family
                protein"
                /translation="MRTQEKCRLEAPENDHIKESCQKTIEFFDSQINELNDTIQKIID
                ESRELQQRQKILKTVPGIGKKLSQDFLCLMPELGYLNRKEVASLAGVAPHPKESGKAV
                GYRRITGGRSNVRSKLFTAAMAATRSKSILGAFYSKLVESGKKKMVAITALMRKIIVI
                ANTRLKEAINLHT"
                /besthit="qcoverage 100 , hcoverage 100 , similarity 100
                , identity 100 , evalue 4.69e-112 , alnlength 173 ,
                Bacteria , Proteobacteria , RLT59977.1 transposase
                IS116/IS110/IS902 family protein [Wolbachia endosymbiont
                of Drosophila ananassae]"
```

```
ORIGIN
        1 tgtaagttaa aaacgtgata taaaagagat aagagagaaa gataaccctg ctcactagga
       61 atagcgggcg tgcgcgaccg aaaggaaatt ggtattacaa ccgttggtat caaggtacgc
      121 tagccctatc tctcgatacg tttgaatagt tgcttgggac atatatatgc acccgtttac
      181 aatcttaaat taactaaaac tatcgaggtt tattatggtt acatcttatc aaaattttat
      241 tggcattgac atcggaaaat ttaaaaatgt cgttgcaatt cacaaacaga agaatgctgt
      301 cgaatttgat aataatgctt ctggttggca acaattattt caagagtttt cagatatagt
      361 attttctaaa atgaatgata aatttccatt atggtattac caaccaaaag ttgtaagtct
      421 gtatatttat gcattaaagg tctgaggcgg cttttgatgg tattccaaca atgctctata
      481 gggttcaaat ctggtgaata tgttggtaga tagagcaaac ggcatctagc attatctatt
      541 aactcctttg ttttgggagt tttatggaat gtggcattgt ccataactat tgtagtacca
      601 tgtggcaatt taggtaataa catcttctct aaccatgtat tgaatacctc tttgtcacac
      661 ccacctttga aagtcattgg tgcaataaat ctctttccaa tccacccgcc tattatactg
      721 attctctctc gttttcttcc tggaatatct gcataaattt tctttcctat tggagcacgt
      781 ccatactctc ggtataacct attatcaact cctgcttcat ctatatataa gatgctggaa
      841 tggtctattt ttgagatttt atcggtaaat cgctgcctat cttcttgatt gcgctcttga
      901 tatagtgtaa ctttttttta aagtaattct tagctgtttc agcctatacc aaattgaatt
      961 tattccaaat ccaagatttt gtttcatctc tgccagcgta tgatctggat tctttttaac
     1021 atattcttcg agtatttttg ggtctatttt tcgaataaag ctgccgtttt tcgatggcct
     1081 taggctttca ccagcggctt tctttcttaa ccagcgctac agagttgcta ttcctatctc
     1141 caaaagctct gccacctcaa cctttgactt cccttttttct atcatcgata ctgccctttc
     1201 ccttaaatcc acactatacg ccattataac tcaaatagct tattatacct ttatcattta
     1261 ctttgggaag tactatattc tacctaatgc tttagtgact ttagaaaata cagggaaata
     1321 tgagcttggc ttatcacatt ttcttaccga caaaaatatt gccgtgcatc gagctaatac
     1381 tcgtaaagta aaaagcttta ttttatctca cggaacttta gcaaagtcta gatgcaagag
     1441 ctcttgctca atatggattt gaacgccata gaactatctc tctatttgta cctacctcta
     1501 cagaacaatc aactttggct gcactttgtc aacgtcgtga tgatattacg caaatgagaa
     1561 ctcaagaaaa atgcagactt gaagcaccgg aaaatgatca tataaaagaa agctgtcaaa
     1621 agaccattga gtttttcgat agtcaaataa acgaactcaa tgatactata caaaagatta
     1681 ttgatgaaag ccgcgaatta caacaacgcc aaaaaattct taaaacagtt cctggaatag
     1741 gtaaaaagtt atcacaagat tttttgtgtt taatgccaga gcttggttac ttaaacagaa
     1801 aagaagtagc aagtcttgcc ggagttgcac cgcatccaaa agaaagtggt aaagctgttg
     1861 gttaccgaag gattacaggt ggtagaagta acgttcgttc aaagctcttt acagccgcta
     1921 tggctgctac aaggtcgaaa tctatacttg gcgcctttta ttctaagctt gttgaaagtg
     1981 gtaagaagaa gatggtggct ataacagccc taatgcgtaa aattatagta attgctaata
     2041 caagacttaa agaagcaatt aatttgcata cttaaaaatc ttcacaacgg taactgaaat
     2101 tggctaatga atatgtgcgt ccacacagac ttaaagcaca tattcattag cttaagcaga
     2161 aattgctgtg gtagctgttt gatataaaat tctatttcta tgacaaacgg gtttttattg
     2221 cccatataat ttgcaaaatt acacaaataa aaaaattttt aaaaaacata gttgatagca
     2281 ctgacaga
//
LOCUS       JAQZAU010000043            1692 bp    dna     linear   ENV 17-MAR-2023
DEFINITION  MAG: Wolbachia pipientis isolate wEsol contig_31598, whole genome
            shotgun sequence.
ACCESSION   JAQZAU010000043 JAQZAU010000000
VERSION     JAQZAU010000043.1
DBLINK      BioProject: PRJNA929258
            BioSample: SAMN33142973
KEYWORDS    WGS; ENV; Metagenome Assembled Genome; MAG.
SOURCE      Wolbachia pipientis (insect metagenome)
  ORGANISM  Wolbachia pipientis
            Bacteria; Pseudomonadota; Alphaproteobacteria; Rickettsiales;
            Anaplasmataceae; Wolbachieae; Wolbachia.
REFERENCE   1  (bases 1 to 1692)
  AUTHORS   Fiutek,N., Couger,B.M., Roy,S., de la Torre,J.R. and Connor,E.F.
  TITLE     Genomic assessment of the contribution of the Wolbachia
            endosymbiont of Eurosta solidaginis to gall-induction
  JOURNAL   International Journal Molecular Sciences
REFERENCE   2  (bases 1 to 1692)
  AUTHORS   Fiutek,N., Couger,B.M., Roy,S., de la Torre,J.R. and Connor,E.F.
```

```
  TITLE     Direct Submission
  JOURNAL   Submitted (10-FEB-2023) Biology, San Francisco State University,
            1600 Holloway Ave, San Francisco, CA 94132, USA
COMMENT     ##Genome-Assembly-Data-START##
            Assembly Date          :: JUN-2019
            Assembly Method        :: FLYE v. 06-2019
            Genome Representation  :: Full
            Expected Final Version :: Yes
            Genome Coverage        :: 100.0x
            Sequencing Technology  :: Illumina HiSeq; PacBio Sequel
            ##Genome-Assembly-Data-END##
FEATURES             Location/Qualifiers
     source          1..1692
                     /organism="Wolbachia pipientis"
                     /mol_type="genomic DNA"
                     /submitter_seqid="contig_31598"
                     /isolate="wEsol"
                     /isolation_source="galls on Solidago altissima"
                     /host="Eurosta solidaginis MN-2018-EFC"
                     /db_xref="taxon:955"
                     /environmental_sample
                     /country="USA: Cowling Arboretum, Carleton College,
                     Northfield, MN"
                     /lat_lon="44.48 N 93.13 W"
                     /altitude="280 m"
                     /collection_date="2018-11"
                     /metagenome_source="insect metagenome"
                     /note="metagenomic"
     gene            <1..128
                     /locus_tag="wEsol_00336"
     CDS             <1..128
                     /locus_tag="wEsol_00336"
                     /codon_start=1
                     /transl_table=11
                     /product="hypothetical protein"
                     /pfam="DEDD_Tnp_IS110"
                     /translation="KTDKSDAAMIARFCIANKPALWKPIAPEMRHLRALHAIAKR"
                     /besthit="qcoverage 87.8048780487805 , hcoverage
                     29.7520661157025 , similarity 97.2 , identity 94.4 ,
                     evalue 1.07e-15 , alnlength 36 , Bacteria ,
                     Proteobacteria , WP_110410155.1 transposase [Wolbachia
                     endosymbiont of Folsomia candida]"
     gene            109..435
                     /locus_tag="wEsol_00337"
     CDS             109..435
                     /locus_tag="wEsol_00337"
                     /codon_start=1
                     /transl_table=11
                     /pfam="Transposase_20"
                     /fullproduct="qcoverage 100 , hcoverage 100 , similarity
                     100 , identity 100 , evalue 2.83e-66 , alnlength 108 ,
                     Bacteria , Proteobacteria , WP_007549647.1 MULTISPECIES:
                     transposase [Wolbachia]"
                     /translation="MQSLKDDKVQQTNRLENKNMHSSCKEAISKVILAIEEQIIVLEK
                     EINEHINNYPHLKNMVENLKTIKGIGYLTAVAVVAEMPSVDNFDNAKQFTAFAARTLS
                     IGIVSK"
                     /product="transposase"
                     /besthit="qcoverage 100 , hcoverage 100 , similarity 100
                     , identity 100 , evalue 2.83e-66 , alnlength 108 ,
                     Bacteria , Proteobacteria , WP_007549647.1 MULTISPECIES:
```

```
                     transposase [Wolbachia]"
     gene            374..664
                     /locus_tag="wEsol_00338"
     CDS             374..664
                     /locus_tag="wEsol_00338"
                     /codon_start=1
                     /transl_table=11
                     /pfam="Transposase_20"
                     /fullproduct="qcoverage 100 , hcoverage 100 , similarity
                     100 , identity 100 , evalue 2.78e-65 , alnlength 96 ,
                     Bacteria , Proteobacteria , WP_081600779.1 MULTISPECIES:
                     transposase [Wolbachia]"
                     /translation="MLNNLQLLLPGHYQSGSSVSKRSCICKIGSERIRKALYMPAIVV
                     KNHNNHFQKFCQRLASKGKCPKVIVLALMRKLMHVFFGILKKNQPFNCNFVG"
                     /product="transposase"
                     /besthit="qcoverage 100 , hcoverage 100 , similarity 100
                     , identity 100 , evalue 2.78e-65 , alnlength 96 ,
                     Bacteria , Proteobacteria , WP_081600779.1 MULTISPECIES:
                     transposase [Wolbachia]"
     gene            complement(878..>1692)
                     /locus_tag="wEsol_00339"
     CDS             complement(878..>1692)
                     /locus_tag="wEsol_00339"
                     /codon_start=1
                     /transl_table=11
                     /product="hypothetical protein"
                     /pfam="AA_permease_2 AA_permease"
                     /tigr="TIGR00905 2A0302 473 transporter, basic amino
                     acid/polyamine antiporter (APA) family"
                     /translation="VLLVIPIAALFFFDRNNFIISEEISNLTTSQILARSTLITLWGF
                     IGVELATAPAGSVDNPARTIPRAVVLGTISVAVAYFINNLAIMGLINGNDLASSRAPY
                     VDAIKIMASGNWHLIISITAFIFCVGTLNAWVLASGQVALGLAKDKLMPQFFAQRNKH
                     GSPFWGITISSVGTSVLLILTSSNNFAKQITSIIDFSVVSFLFVYLACSLAFLKVIVQ
                     EKSCYKFLIGSIATTFCCWVIFETSVNTLLIASLFTASGVPLYLFWYRRVSV"
                     /cog="PotE COG0531 466 Amino acid transporters"
                     /besthit="qcoverage 100 , hcoverage 63.6792452830189 ,
                     similarity 99.6 , identity 99.6 , evalue 1.16e-180 ,
                     alnlength 270 , Bacteria , Proteobacteria ,
                     WP_141456681.1 MULTISPECIES: amino acid permease
                     [unclassified Wolbachia]"
ORIGIN
        1 ataagacaga taaatcagat gcagctatga tagctagatt ttgtatcgcc aacaaacctg
       61 ctctttggaa gcccattgca cctgaaatga ggcatttgag ggcgttgcat gcaatcgcta
      121 aaagatgata aggtgcaaca gacaaatcgc ttggaaaaca aaaatatgca ttccagttgc
      181 aaagaagcta tatctaaagt aattttggca atagaggagc aaattattgt tctcgaaaaa
      241 gaaattaatg agcatataaa taactatcca catctaaaaa atatggtgga aaaccttaag
      301 actataaaag gtataggata tcttactgct gttgctgttg tcgcagaaat gccatcagtt
      361 gataactttg acaatgctaa acaatttaca gcttttgctg ccaggacatt atcaatcggg
      421 atcgtcagta agtaagagaa gttgcatatg caaaatagga tctgagcgta ttcgaaaagc
      481 tctttatatg ccagctatag tagtcaaaaa ccataataat cattttcaaa agttttgtca
      541 gcgtctagca agtaaaggca aatgtccaaa agtcatagtt cttgcgttaa tgcgaaaatt
      601 aatgcatgtc tttttggta ttcttaaaaa gaatcaacca tttaattgta atttgttgg
      661 ataatttgtt tgacagcaga gacagtatct gggatccaga ttgggcacta agttggtaaa
      721 cacgaaagcg attaatcctt tacaacgttt ttgatggaac tgcgcgaaag aactggatgc
      781 cagtgtcacg cactggcatg acacccattt cttcctatag ttgtcttttc tcgtctacct
      841 tattttgcca ctctgctgaa cagatacata acatttgcta tacagaaact ctgcgatacc
      901 aaaataaata aagaggtacg ccacttgcag tgaataagct tgcgatcagc aaggtattca
      961 cagaagtttc aaatattacc cagcagcaaa aggttgttgc tatactgcca attaaaaatt
     1021 tgtaacaact cttttcttgc acaataactt tgagaaaagc aagactgcat gcaaggtaaa
     1081 caaataaaaa tgaaactaca gaaaagtcga taattgatgt gatctgctta gcaaaattgt
```

```
     1141 tgcttgaagt gaggattagt agaactgaag tgccaacgga gctgattgtt atgccccaga
     1201 aaggagaacc gtgcttgttt ctttgagcaa aaaactgtgg catcagttta tcttttgcaa
     1261 ggccaagggc cacttgtcca ctagctagta cccaagcgtt taaagtacca acacaaaaaa
     1321 tgaacgcagt tatagaaata atcaagtgcc aattgccaga ggccataatt tttattgcgt
     1381 caacatatgg tgctcttgaa cttgctaaat cattgccatt tattaatccc ataattgcaa
     1441 gattgttgat aaaatacgca acagcaacag agattgttcc aagtaccaca gctcttggta
     1501 ttgttctagc tggattatca actgaccctg caggtgctgt tgccaattca actccaataa
     1561 aaccccatag agtaataagt gtagaacgtg caagaatctg cgatgttgta agatttgata
     1621 tttcttcact tatgatgaag ttatttctat caaaaaaaaa caatgctgct ataggtatta
     1681 caagcaatac ag
//
LOCUS       JAQZAU010000044            989 bp    dna     linear   ENV 17-MAR-2023
DEFINITION  MAG: Wolbachia pipientis isolate wEsol contig_31868, whole genome
            shotgun sequence.
ACCESSION   JAQZAU010000044  JAQZAU010000000
VERSION     JAQZAU010000044.1
DBLINK      BioProject: PRJNA929258
            BioSample: SAMN33142973
KEYWORDS    WGS; ENV; Metagenome Assembled Genome; MAG.
SOURCE      Wolbachia pipientis (insect metagenome)
  ORGANISM  Wolbachia pipientis
            Bacteria; Pseudomonadota; Alphaproteobacteria; Rickettsiales;
            Anaplasmataceae; Wolbachieae; Wolbachia.
REFERENCE   1  (bases 1 to 989)
  AUTHORS   Fiutek,N., Couger,B.M., Roy,S., de la Torre,J.R. and Connor,E.F.
  TITLE     Genomic assessment of the contribution of the Wolbachia
            endosymbiont of Eurosta solidaginis to gall-induction
  JOURNAL   International Journal Molecular Sciences
REFERENCE   2  (bases 1 to 989)
  AUTHORS   Fiutek,N., Couger,B.M., Roy,S., de la Torre,J.R. and Connor,E.F.
  TITLE     Direct Submission
  JOURNAL   Submitted (10-FEB-2023) Biology, San Francisco State University,
            1600 Holloway Ave, San Francisco, CA 94132, USA
COMMENT     ##Genome-Assembly-Data-START##
            Assembly Date          :: JUN-2019
            Assembly Method        :: FLYE v. 06-2019
            Genome Representation  :: Full
            Expected Final Version :: Yes
            Genome Coverage        :: 100.0x
            Sequencing Technology  :: Illumina HiSeq; PacBio Sequel
            ##Genome-Assembly-Data-END##
FEATURES             Location/Qualifiers
     source          1..989
                     /organism="Wolbachia pipientis"
                     /mol_type="genomic DNA"
                     /submitter_seqid="contig_31868"
                     /isolate="wEsol"
                     /isolation_source="galls on Solidago altissima"
                     /host="Eurosta solidaginis MN-2018-EFC"
                     /db_xref="taxon:955"
                     /environmental_sample
                     /country="USA: Cowling Arboretum, Carleton College,
                     Northfield, MN"
                     /lat_lon="44.48 N 93.13 W"
                     /altitude="280 m"
                     /collection_date="2018-11"
                     /metagenome_source="insect metagenome"
                     /note="metagenomic"
     gene            complement(<1..105)
                     /locus_tag="wEsol_00340"
```

```
     CDS             complement(<1..105)
                     /locus_tag="wEsol_00340"
                     /codon_start=1
                     /transl_table=11
                     /product="hypothetical protein"
                     /translation="MLEFTSFRTTSNAQSSRLDGESYQYTTRDYMTIYF"
                     /besthit="qcoverage 100 , hcoverage 25.1798561151079 ,
                     similarity 100 , identity 100 , evalue 1.03e-14 ,
                     alnlength 35 , Bacteria , Proteobacteria , WP_155969321.1
                     MULTISPECIES: hypothetical protein [Wolbachia]"
     gene            complement(98..439)
                     /locus_tag="wEsol_00341"
     CDS             complement(98..439)
                     /locus_tag="wEsol_00341"
                     /codon_start=1
                     /transl_table=11
                     /fullproduct="qcoverage 100 , hcoverage 100 , similarity
                     100 , identity 100 , evalue 1.53e-79 , alnlength 113 ,
                     Bacteria , Proteobacteria , AAS13810.1 transposase, IS5
                     family, OrfB [Wolbachia endosymbiont of Drosophila
                     melanogaster]"
                     /cog="COG3293 COG3293 124 Transposase and inactivated
                     derivatives"
                     /pfam="DDE_Tnp_1_2 DDE_Tnp_1"
                     /product="transposase, IS5 family, OrfB"
                     /translation="MRTKYTKVKKMWADMGYQGRNLKNHIKEEYDIDIEIVKRPPCRF
                     WVHKDTPPELLPTREQGFKVQPRRWVVERTFAWVNRNRRLSKEYDLLTTSTENFIYLA
                     MSRVMLKREYA"
                     /besthit="qcoverage 100 , hcoverage 100 , similarity 100
                     , identity 100 , evalue 1.53e-79 , alnlength 113 ,
                     Bacteria , Proteobacteria , AAS13810.1 transposase, IS5
                     family, OrfB [Wolbachia endosymbiont of Drosophila
                     melanogaster]"
     gene            complement(550..930)
                     /locus_tag="wEsol_00342"
     CDS             complement(550..930)
                     /locus_tag="wEsol_00342"
                     /codon_start=1
                     /transl_table=11
                     /fullproduct="qcoverage 100 , hcoverage 100 , similarity
                     100 , identity 100 , evalue 1.16e-87 , alnlength 126 ,
                     Bacteria , Proteobacteria , AOV87106.1 transposase
                     [Wolbachia endosymbiont of Drosophila incompta]"
                     /cog="COG3293 COG3293 124 Transposase and inactivated
                     derivatives"
                     /pfam="DUF4096"
                     /product="transposase"
                     /translation="MRKKYPTDLSEREWARIEKHFRVSYKKGGRPPKYSKREILEAIF
                     YVLRTGCQWRYLPNDFPLWKTVYEQFRQWKKQGIFEKMNYEITKYSRRKIGRNEQPSA
                     CIVDSQSVKTTEKGGSKAMMEVKK"
                     /besthit="qcoverage 100 , hcoverage 100 , similarity 100
                     , identity 100 , evalue 1.16e-87 , alnlength 126 ,
                     Bacteria , Proteobacteria , AOV87106.1 transposase
                     [Wolbachia endosymbiont of Drosophila incompta]"
ORIGIN
        1 aaagtaaatt gtcatatagt ctcttgtggt gtattgataa ctttctccat ctagtctgct
       61 actttgtgcg ttagaggttg tccggaaact agtaaattca agcatattcc ctctttaaca
      121 taaccctact catagctagg tatatgaaat tctcagtgga tgttgtgagt aaatcatact
      181 ccttcgatag ccttctattc ctattaaccc aagcaaaagt cctttctaca acccatcttc
      241 ttggctgtac tttaaaccct tgttctcttg ttggtagtag ctcaggtggc gtatctttgt
```

```
      301 gcacccaaaa tctacatgga ggccttttaa caatttcaat atctatgtca tattcttcct
      361 ttatgtgatt ctttaaattt cttccttggt atcccatgtc agcccacatt tttttaactt
      421 tagtatattt tgttctcata ttgtttaatg ctattttaat accatctcta tcattttcgt
      481 tagcagcgcc tacgtaacaa cctagtataa aaccctgagt gtctgtaatt atatgccttt
      541 ttctaccctt tacttttttta cttccatcat agcctttgat cccccctttt ctgtagtctt
      601 tacagattga ctatctacta tacaggcact cggctgctca ttccttccta tttttcttct
      661 actatatttt gtaatttcat aattcatttt ctcaaaaatt ccctgcttct tccattgcct
      721 gaactgctca tacacagtct tccatagcgg aaaatcattt ggtaaatacc gccattgaca
      781 ccctgtacgc aatacataga aaattgcttc taatatttct cttttgctat actttggcgg
      841 ccttcctcct ttcttgtatg atactctgaa gtgtttttct attcttgccc attccctttc
      901 gcttagatct gttggatact tttttctcat ctttaccccg tactaaaatt tcagatatta
      961 tagcctttta cttatttccg gacaacctc
//
LOCUS       JAQZAU010000045          37288 bp    dna     linear   ENV 17-MAR-2023
DEFINITION  MAG: Wolbachia pipientis isolate wEsol contig_32067, whole genome
            shotgun sequence.
ACCESSION   JAQZAU010000045  JAQZAU010000000
VERSION     JAQZAU010000045.1
DBLINK      BioProject: PRJNA929258
            BioSample: SAMN33142973
KEYWORDS    WGS; ENV; Metagenome Assembled Genome; MAG.
SOURCE      Wolbachia pipientis (insect metagenome)
  ORGANISM  Wolbachia pipientis
            Bacteria; Pseudomonadota; Alphaproteobacteria; Rickettsiales;
            Anaplasmataceae; Wolbachieae; Wolbachia.
REFERENCE   1  (bases 1 to 37288)
  AUTHORS   Fiutek,N., Couger,B.M., Roy,S., de la Torre,J.R. and Connor,E.F.
  TITLE     Genomic assessment of the contribution of the Wolbachia
            endosymbiont of Eurosta solidaginis to gall-induction
  JOURNAL   International Journal Molecular Sciences
REFERENCE   2  (bases 1 to 37288)
  AUTHORS   Fiutek,N., Couger,B.M., Roy,S., de la Torre,J.R. and Connor,E.F.
  TITLE     Direct Submission
  JOURNAL   Submitted (10-FEB-2023) Biology, San Francisco State University,
            1600 Holloway Ave, San Francisco, CA 94132, USA
COMMENT     ##Genome-Assembly-Data-START##
            Assembly Date          :: JUN-2019
            Assembly Method        :: FLYE v. 06-2019
            Genome Representation  :: Full
            Expected Final Version :: Yes
            Genome Coverage        :: 100.0x
            Sequencing Technology  :: Illumina HiSeq; PacBio Sequel
            ##Genome-Assembly-Data-END##
FEATURES             Location/Qualifiers
     source          1..37288
                     /organism="Wolbachia pipientis"
                     /mol_type="genomic DNA"
                     /submitter_seqid="contig_32067"
                     /isolate="wEsol"
                     /isolation_source="galls on Solidago altissima"
                     /host="Eurosta solidaginis MN-2018-EFC"
                     /db_xref="taxon:955"
                     /environmental_sample
                     /country="USA: Cowling Arboretum, Carleton College,
                     Northfield, MN"
                     /lat_lon="44.48 N 93.13 W"
                     /altitude="280 m"
                     /collection_date="2018-11"
                     /metagenome_source="insect metagenome"
                     /note="metagenomic"
```

```
gene            73..354
                /locus_tag="wEsol_00343"
CDS             73..354
                /locus_tag="wEsol_00343"
                /codon_start=1
                /transl_table=11
                /fullproduct="qcoverage 100 , hcoverage 100 , similarity
                98.9 , identity 98.9 , evalue 1.51e-63 , alnlength 93 ,
                Bacteria , Proteobacteria , CDR78524.1 hypothetical
                protein WPAU_0128 [Wolbachia endosymbiont of Drosophila
                simulans wAu]"
                /translation="MLRWHIEECYKRLKVGAELENFSGEAVLQEFWANLVMCNILSLH
                MCDAQGPWNPDQITEYRLNFSVLFGVMRQKLYQVLIAPKNFQALFKYTR"
                /product="hypothetical protein"
                /besthit="qcoverage 100 , hcoverage 100 , similarity 98.9
                , identity 98.9 , evalue 1.51e-63 , alnlength 93 ,
                Bacteria , Proteobacteria , CDR78524.1 hypothetical
                protein WPAU_0128 [Wolbachia endosymbiont of Drosophila
                simulans wAu]"
gene            complement(441..650)
                /locus_tag="wEsol_00344"
CDS             complement(441..650)
                /locus_tag="wEsol_00344"
                /codon_start=1
                /transl_table=11
                /fullproduct="qcoverage 100 , hcoverage 100 , similarity
                100 , identity 100 , evalue 8.37e-39 , alnlength 69 ,
                Bacteria , Proteobacteria , WP_015589377.1 MULTISPECIES:
                hypothetical protein [Wolbachia]"
                /translation="MLVSEQIESVDREELKQQNHIHKDTECSEETTIYGHINQHMEKN
                ISNTLHEIYDALMSYNESEQSMTST"
                /product="hypothetical protein"
                /besthit="qcoverage 100 , hcoverage 100 , similarity 100
                , identity 100 , evalue 8.37e-39 , alnlength 69 ,
                Bacteria , Proteobacteria , WP_015589377.1 MULTISPECIES:
                hypothetical protein [Wolbachia]"
gene            782..2107
                /locus_tag="wEsol_00345"
CDS             782..2107
                /locus_tag="wEsol_00345"
                /codon_start=1
                /transl_table=11
                /fullproduct="qcoverage 100 , hcoverage 100 , similarity
                100 , identity 100 , evalue 3.68e-315 , alnlength 441 ,
                Bacteria , Proteobacteria , WP_010963041.1 MULTISPECIES:
                ribosome biogenesis GTPase Der [Wolbachia]"
                /cog="COG1160 COG1160 444 Predicted GTPases"
                /pfam="Arf MMR_HSR1 FeoB_N Miro AIG1 Ras AAA_17 Gtr1_RagA
                Dynamin_N SRPRB AAA_33 Pfam-B_6479 DUF258 GTP_EFTU AIG1
                Septin Pfam-B_3369 MMR_HSR1 FeoB_N Miro SRPRB Dynamin_N
                Ras AAA_18 Pfam-B_9457"
                /tigr="TIGR03594 GTPase_EngA 432 ribosome-associated
                GTPase EngA"
                /product="ribosome
                biogenesis GTPase Der"
                /translation="MLKIAIVGLPNAGKSTLFNRLVGRKAAVVSNIPGVTRDRREGMG
                RISDLEFKVIDTGGWNDQTNFSLQVIEQIEFSLSNSNIIFFLVDAKVQNERNEEFAKW
                LKRKINKPVILVANKCESHKSENVDYLQFFDFLGPVYISAEHNLGMVDLYDALAGVIE
                NFNENTELPNNELSRLRIAIIGRPNVGKSTFLNGLLAENRLITSSEPGTTRDSVDITY
                DHDGELITLIDTAGIRRKANVVDGLESRFVEKSMESIKRSHVVVLMLDSLVGIEQQDL
```

SIGEAAIKGGKGIIVVLNKWDLIGKDDRSRLIKFVKQQEVTRLFLEVPTITISALKGM
RCGDVIDKCLEVSESLNKKISTAKLNKWLIDAVGKHSHPLVKGKAVKMKYIAQIGTKP
PAFSLICNIPESVDESYKRYLINDLRKNFFADGVPVRLLLKKNKNPYVK"
/besthit="qcoverage 100 , hcoverage 100 , similarity 100
, identity 100 , evalue 3.68e-315 , alnlength 441 ,
Bacteria , Proteobacteria , WP_010963041.1 MULTISPECIES:
ribosome biogenesis GTPase Der [Wolbachia]"
gene 2104..2625
/locus_tag="wEsol_00346"
CDS 2104..2625
/locus_tag="wEsol_00346"
/codon_start=1
/transl_table=11
/fullproduct="qcoverage 100 , hcoverage 100 , similarity
100 , identity 100 , evalue 6.45e-119 , alnlength 173 ,
Bacteria , Proteobacteria , WP_015589379.1 MULTISPECIES:
disulfide bond formation protein B [Wolbachia]"
/cog="DsbB COG1495 170 Disulfide bond formation protein
DsbB"
/pfam="DsbB"
/product="disulfide bond
formation protein B"
/translation="MNTLLYTFRDNLLKVMCNNSRIPAIFLLSSAAALIFAYVLEYFF
NMLPCKLCTYERIVYYIAGLLAVACMLKDNKILIYAMFCSYLIGAVISFYHIGLELHL
FHDVLGCTEQASGNVSIEELRNNLLNPNYSPSCDRPHYVLGVSLATWNLIYLIVALFV
SGKVYCGERKKSK"
/besthit="qcoverage 100 , hcoverage 100 , similarity 100
, identity 100 , evalue 6.45e-119 , alnlength 173 ,
Bacteria , Proteobacteria , WP_015589379.1 MULTISPECIES:
disulfide bond formation protein B [Wolbachia]"
gene 2600..3142
/locus_tag="wEsol_00347"
CDS 2600..3142
/locus_tag="wEsol_00347"
/codon_start=1
/transl_table=11
/fullproduct="qcoverage 100 , hcoverage 100 , similarity
100 , identity 99.4 , evalue 3.15e-118 , alnlength 180 ,
Bacteria;Viruses , Proteobacteria;Uroviricota ,
WP_006279362.1 MULTISPECIES: demethoxyubiquinone
hydroxylase family protein [Wolbachia]"
/cog="CAT5 COG2941 204 Ubiquinone biosynthesis protein
COQ7"
/pfam="COQ7"
/product="
demethoxyubiquinone hydroxylase family protein"
/translation="MEKERNLNNLRYSNNQFLQQAIRVDHAGEYGAICIYSGQKFILK
KSSIINEIIEMEEQEKKHFHYFNEKIKEQKVRPTVLLPVWRVLGVSLGVATAIMGKEA
AMACTAAVEEVIGEHYKEQVSHLEDGELRETISKFRDEELEHKDIAIQHNAESAFGYN
ILSSFIKTGCKTAIYLSKLI"
/besthit="qcoverage 100 , hcoverage 100 , similarity 100
, identity 99.4 , evalue 3.15e-118 , alnlength 180 ,
Bacteria;Viruses , Proteobacteria;Uroviricota ,
WP_006279362.1 MULTISPECIES: demethoxyubiquinone
hydroxylase family protein [Wolbachia]"
gene complement(3641..4276)
/locus_tag="wEsol_00348"
CDS complement(3641..4276)
/locus_tag="wEsol_00348"
/codon_start=1

```
                     /transl_table=11
                     /pfam="DDE_Tnp_1 rve DDE_Tnp_1_2"
                     /fullproduct="qcoverage 100 , hcoverage 100 , similarity
                     100 , identity 100 , evalue 2.85e-152 , alnlength 211 ,
                     Bacteria , Proteobacteria , WP_081600764.1 MULTISPECIES:
                     transposase [unclassified Wolbachia]"
                     /translation="MIAENNMEDIEIAIDSTGISIYNNTPGHSKENSTDRKYRGYEQT
                     RKLHVMLNINNKKAIAVKYSNGVYSDHYGACDLLKEVDFQHAIKALYADRACDRHKFY
                     KLCNEYDIKTKIPPINNAAEHPEIDYMSDRNAAIRLIKLYGEDGMKEWKKEVNYGKRS
                     YIEGFFSRLKQIFGFSFRNKSEINREKELLIKCYLLNKFTDIGMAKFEIVT"
                     /product="transposase"
                     /besthit="qcoverage 100 , hcoverage 100 , similarity 100
                     , identity 100 , evalue 2.85e-152 , alnlength 211 ,
                     Bacteria , Proteobacteria , WP_081600764.1 MULTISPECIES:
                     transposase [unclassified Wolbachia]"
     gene            complement(4260..4589)
                     /locus_tag="wEsol_00349"
     CDS             complement(4260..4589)
                     /locus_tag="wEsol_00349"
                     /codon_start=1
                     /transl_table=11
                     /pfam="DDE_Tnp_1_5"
                     /fullproduct="qcoverage 100 , hcoverage 100 , similarity
                     100 , identity 100 , evalue 4.31e-75 , alnlength 109 ,
                     Bacteria , Proteobacteria , WP_141456678.1 MULTISPECIES:
                     transposase [unclassified Wolbachia]"
                     /translation="MPQKMRVSNCHEYNKFLQERGSIFCYINDAIENWYENCPKMQGG
                     NYIYSDKVVILVHIIVSFFRIGLRQTVGFIKGYLQQIGRDLAVISYSQASRRFKKLNI
                     KINDCRK"
                     /product="transposase"
                     /besthit="qcoverage 100 , hcoverage 100 , similarity 100
                     , identity 100 , evalue 4.31e-75 , alnlength 109 ,
                     Bacteria , Proteobacteria , WP_141456678.1 MULTISPECIES:
                     transposase [unclassified Wolbachia]"
     gene            4813..5703
                     /locus_tag="wEsol_00350"
     CDS             4813..5703
                     /locus_tag="wEsol_00350"
                     /codon_start=1
                     /transl_table=11
                     /fullproduct="qcoverage 96.9594594594595 , hcoverage
                     88.0368098159509 , similarity 100 , identity 100 , evalue
                     7.13e-191 , alnlength 287 , Bacteria , Proteobacteria ,
                     WP_007548592.1 MULTISPECIES:
                     phospho-N-acetylmuramoyl-pentapeptide-transferase
                     [Wolbachia]"
                     /cog="Rfe COG0472 319 UDP-N-acetylmuramyl pentapeptide
                     phosphotransferase/UDP-N- acetylglucosamine-1-phosphate
                     transferase"
                     /pfam="Glycos_transf_4 Pfam-B_8659"
                     /tigr="TIGR00445 mraY 321
                     phospho-N-acetylmuramoyl-pentapeptide-transferase"
                     /product="
                     phospho-N-acetylmuramoyl-pentapeptide-transferase"
                     /translation="MILATKVFFTSFVFGFILFPYFIKLLKKISKDGQPIRSCGPESH
                     LITKKNVPPMGGIIILTSSLLPILLWVQLTPEILLLILITLFFALLGFIDDYLKLKTN
                     HYRGLSAKTKILIQFIVALVGVFILKLYSAECFTKTSLFKGVIIDFGYLYVPFAAFVI
                     VGSSNAVNLTDGLDGLAATQVITSFAFLGLIAYITQADMNITLFCIAFIGAILSFLWF
                     NTHPAKIFMGDVGSLSVGAALGLTSVLIKREMLFAVIGIIFVIETLSVIIQISYFKYT
                     KFKYGEGKRVFPYGTNASSL"
```

/besthit="qcoverage 96.9594594594595 , hcoverage 88.0368098159509 , similarity 100 , identity 100 , evalue 7.13e-191 , alnlength 287 , Bacteria , Proteobacteria , WP_007548592.1 MULTISPECIES: phospho-N-acetylmuramoyl-pentapeptide-transferase [Wolbachia]"

```
gene            5801..6397
                /locus_tag="wEsol_00351"
CDS             5801..6397
                /locus_tag="wEsol_00351"
                /codon_start=1
                /transl_table=11
                /fullproduct="qcoverage 100 , hcoverage 100 , similarity
                100 , identity 100 , evalue 3.92e-139 , alnlength 198 ,
                Bacteria , Proteobacteria , WP_015589381.1 MULTISPECIES:
                ribonuclease HII [Wolbachia]"
                /cog="RnhB COG0164 199 Ribonuclease HII"
                /pfam="RNase_HII"
                /tigr="TIGR00729 TIGR00729 207 ribonuclease HII"
                /product="ribonuclease HII"
                /translation="MKYPDFTLENKLSGVIAGVDEVGRGPLAGPVISAAVVFIDRNTI
                IDGINDSKKLTPQCRQVLYEKIISVAKFGIGMASVEEINSYNILQATKLSMKRALIDL
                DLELDYVLVDGNQPPEVKWQVKSIVNGDNLSTSIAAASIVAKVTRDRLMQELHNKHPE
                YNWYKNKGYGTKEHLNAIGLYGITEHHRKNFAPISRAL"
                /besthit="qcoverage 100 , hcoverage 100 , similarity 100
                , identity 100 , evalue 3.92e-139 , alnlength 198 ,
                Bacteria , Proteobacteria , WP_015589381.1 MULTISPECIES:
                ribonuclease HII [Wolbachia]"
gene            complement(6401..7183)
                /locus_tag="wEsol_00352"
CDS             complement(6401..7183)
                /locus_tag="wEsol_00352"
                /codon_start=1
                /transl_table=11
                /fullproduct="qcoverage 100 , hcoverage 100 , similarity
                100 , identity 100 , evalue 2.72e-188 , alnlength 260 ,
                Bacteria , Proteobacteria , WP_015589382.1 MULTISPECIES:
                P-type conjugative transfer protein VirB9 [unclassified
                Wolbachia]"
                /cog="VirB9 COG3504 265 Type IV secretory pathway, VirB9
                components"
                /pfam="CagX"
                /tigr="TIGR02781 VirB9 248 P-type conjugative transfer
                protein VirB9"
                /product="P-type conjugative
                transfer protein VirB9"
                /translation="MIGLLFAVLLFLCGSDALAKQEPRSIAGDNHIKVINYNPQAIHK
                YTGFYGYQSSILFEPGEIIQNLSMGDSTGWQLLPQGNRLFIKPIDDIADTNATIITNK
                RVYYFELHAEEATGLDDPRLAYEVRFLYPLFSSDEIYTTNNGDIFEQASHTIIPDIND
                IEVVKKGLNFNYSISHVKGSQSIIPIKVFDDGKFTYLQFNKINSDFPAVFMVDSAGYE
                SLVNFRTVDNYLIIERVSSVFTLRNGSSTVCLFNETIPFKKG"
                /besthit="qcoverage 100 , hcoverage 100 , similarity 100
                , identity 100 , evalue 2.72e-188 , alnlength 260 ,
                Bacteria , Proteobacteria , WP_015589382.1 MULTISPECIES:
                P-type conjugative transfer protein VirB9 [unclassified
                Wolbachia]"
gene            complement(7369..7824)
                /locus_tag="wEsol_00353"
CDS             complement(7369..7824)
                /locus_tag="wEsol_00353"
```

```
                     /codon_start=1
                     /transl_table=11
                     /fullproduct="qcoverage 100 , hcoverage 100 , similarity
                     100 , identity 100 , evalue 5.53e-98 , alnlength 151 ,
                     Bacteria;Eukaryota , Proteobacteria;Arthropoda ,
                     WP_007548591.1 MULTISPECIES: outer membrane lipid
                     asymmetry maintenance protein MlaD [Wolbachia]"
                     /cog="Ttg2C COG1463 359 ABC-type transport system
                     involved in resistance to organic solvents, periplasmic
                     component"
                     /pfam="MCE"
                     /tigr="TIGR00996 Mtu_fam_mce 291 virulence factor Mce
                     family protein"
                     /product="outer membrane
                     lipid asymmetry maintenance protein MlaD"
                     /translation="MRRSNILEITAGLFVLIFTIFLIFFAIDKLSYIKKNYKDCYKIY
                     GLFANANGIGVGDSVKISGVDVGSITGVSLDKATYVARIDMCISKDIKLPIDSSALIT
                     SSGVVGSKFVNISPGSDIKLILHGGKIEYTQAEANMGGIMDRILGMFTK"
                     /besthit="qcoverage 100 , hcoverage 100 , similarity 100
                     , identity 100 , evalue 5.53e-98 , alnlength 151 ,
                     Bacteria;Eukaryota , Proteobacteria;Arthropoda ,
                     WP_007548591.1 MULTISPECIES: outer membrane lipid
                     asymmetry maintenance protein MlaD [Wolbachia]"
     gene            complement(7827..8141)
                     /locus_tag="wEsol_00354"
     CDS             complement(7827..8141)
                     /locus_tag="wEsol_00354"
                     /codon_start=1
                     /transl_table=11
                     /fullproduct="qcoverage 100 , hcoverage 100 , similarity
                     100 , identity 100 , evalue 1.73e-74 , alnlength 104 ,
                     Bacteria , Proteobacteria , WP_007548590.1 MULTISPECIES:
                     NADH-ubiquinone oxidoreductase subunit NDUFA12 family
                     protein [Wolbachia]"
                     /cog="COG3761 COG3761 118 NADH:ubiquinone oxidoreductase
                     17.2 kD subunit"
                     /pfam="NDUFA12"
                     /product="NADH-ubiquinone
                     oxidoreductase subunit NDUFA12 family protein"
                     /translation="MLSKICNAIKRLLRREDKFVGRDENGNSYYESSKGKRWVMYSSV
                     SEPTTVPPEWHIWLHYTDNVVPVNNKKRKVKHTPNLTGTKDAYYPNQKVKNYYKSWSP
                     DN"
                     /besthit="qcoverage 100 , hcoverage 100 , similarity 100
                     , identity 100 , evalue 1.73e-74 , alnlength 104 ,
                     Bacteria , Proteobacteria , WP_007548590.1 MULTISPECIES:
                     NADH-ubiquinone oxidoreductase subunit NDUFA12 family
                     protein [Wolbachia]"
     gene            8206..9672
                     /locus_tag="wEsol_00355"
     CDS             8206..9672
                     /locus_tag="wEsol_00355"
                     /codon_start=1
                     /transl_table=11
                     /fullproduct="qcoverage 100 , hcoverage 100 , similarity
                     100 , identity 100 , evalue 0.0 , alnlength 488 ,
                     Bacteria , Proteobacteria , WP_015589384.1 MULTISPECIES:
                     NADH-ubiquinone oxidoreductase [unclassified Wolbachia]"
                     /cog="HyfB COG0651 504 Formate hydrogenlyase subunit
                     3/Multisubunit Na+/H+ antiporter, MnhD subunit"
                     /pfam="NADHdeh_related Oxidored_q1"
```

```
                     /tigr="TIGR00944 2a6301s04 463 multicomponent
                     K+:H+antiporter"
                     /product="NADH-ubiquinone
                     oxidoreductase"
                     /translation="MQLPILQVIIPIIASMFCFLAKKHKVSWFISFIATTITLIISSI
                     LLIKTYKGEIITYHLGNWAPPYGIELRIDVLNSLILTLVNFIALISVLYSFYINEKEI
                     SKNKITGFYSLFLLCLSGLLGILVTNDIFNLYVFLEISSLSSYVLVSMGRDKKALVAA
                     FECLISGTIGATFYLFGIGLLYSMTGTLNMSDMAERIVPLYDNNIIKLGTLFIFVGLS
                     IKMALFPLSRWLVNAYSEAPSFISIFFSGTVTKVMIYVFIRIFYTVFHQNFFLFKPLL
                     DNVIIILALCAIVFGSIFAITAKDIKRLLAHSSISQIGYIILALSFNSKTGVFAAILH
                     IVNHSIIKTSLFMAAGCISYKFDTTKIENLSGLKKSMPYTALAFTLFSLALIGMPLTN
                     GFISKWYIMKAIIESHAWISLVTFAAGSFLALIYMWKMVEKMYFENDATSRTGMTPDK
                     INDVPFPMLFCLLFMAALTVVTGIYSTSIRLVVEKLVF"
                     /besthit="qcoverage 100 , hcoverage 100 , similarity 100
                     , identity 100 , evalue 0.0 , alnlength 488 , Bacteria ,
                     Proteobacteria , WP_015589384.1 MULTISPECIES:
                     NADH-ubiquinone oxidoreductase [unclassified Wolbachia]"
     gene            9726..10616
                     /locus_tag="wEsol_00356"
     CDS             9726..10616
                     /locus_tag="wEsol_00356"
                     /codon_start=1
                     /transl_table=11
                     /fullproduct="qcoverage 99.6621621621622 , hcoverage
                     80.3814713896458 , similarity 99.3 , identity 99.0 ,
                     evalue 7.44e-189 , alnlength 295 , Bacteria ,
                     Proteobacteria , MBH5362260.1 rod shape-determining
                     protein RodA [Wolbachia endosymbiont of Kradibia
                     gibbosae]"
                     /cog="FtsW COG0772 381 Bacterial cell division membrane
                     protein"
                     /pfam="FTSW_RODA_SPOVE"
                     /tigr="TIGR02210 rodA_shape 357 rod shape-determining
                     protein RodA"
                     /product="rod shape-determining protein
                     RodA"
                     /translation="MDRLKKIHWLLVINVIALFCVGIVVQYSSAGGKWVPFAIHQLVI
                     FSFFFLFAIAMSFIELDFYLKYAYFFYIAAAISLLAVNFFGSHIMGSTRWIRIGSISL
                     QPSEFAKVGLILALARYFDKQSVYKMMEFKILLKALIIIFLPVFLVLKQPNLGTAVIM
                     LFIGISIIFTAIIKRSHSVICGTLGIFAVPAIWPFLRPYHKQRILSFLDSSVDPLGIG
                     YNAQQSQIAIGSGGLLGKGFVNGSQTQLGFLPEKRTDFAFAVLSEEWGFLGSMALILL
                     YTSLLGIIFSIAYRSKKLFF"
                     /besthit="qcoverage 99.6621621621622 , hcoverage
                     80.3814713896458 , similarity 99.3 , identity 99.0 ,
                     evalue 7.44e-189 , alnlength 295 , Bacteria ,
                     Proteobacteria , MBH5362260.1 rod shape-determining
                     protein RodA [Wolbachia endosymbiont of Kradibia
                     gibbosae]"
     gene            10681..10830
                     /locus_tag="wEsol_00357"
     CDS             10681..10830
                     /locus_tag="wEsol_00357"
                     /codon_start=1
                     /transl_table=11
                     /product="hypothetical protein"
                     /pfam="FTSW_RODA_SPOVE"
                     /tigr="TIGR02210 rodA_shape 357 rod shape-determining
                     protein RodA"
                     /translation="MGLLPVIGDPLPFLSYGGSTTAASLICIGLLLAIKADEQQNACI
                     LPMLK"
```

```
                     /cog="FtsW COG0772 381 Bacterial cell division membrane
                     protein"
                     /besthit="qcoverage 97.9591836734694 , hcoverage
                     26.2295081967213 , similarity 100 , identity 100 , evalue
                     1.21e-23 , alnlength 48 , Bacteria , Proteobacteria ,
                     AOV88385.1 rod shape-determining protein RodA, partial
                     [Wolbachia endosymbiont of Drosophila incompta]"
     gene            10856..12241
                     /locus_tag="wEsol_00358"
     CDS             10856..12241
                     /locus_tag="wEsol_00358"
                     /codon_start=1
                     /transl_table=11
                     /fullproduct="qcoverage 100 , hcoverage 100 , similarity
                     100 , identity 100 , evalue 0.0 , alnlength 461 ,
                     Bacteria , Proteobacteria , WP_015589386.1 MULTISPECIES:
                     amidophosphoribosyltransferase [Wolbachia]"
                     /cog="PurF COG0034 470 Glutamine
                     phosphoribosylpyrophosphate amidotransferase"
                     /pfam="GATase_2 GATase_4 GATase_2 GATase_6 GATase_7
                     GATase_2 Pribosyltran"
                     /tigr="TIGR01134 purF 432 amidophosphoribosyltransferase"
                     /product="
                     amidophosphoribosyltransferase"
                     /translation="MLDEMREECGVFGISCNQSAAFNSILALHALQHRGQESFGVATS
                     NNDKLHSYHFQGQVSSVFDDIDEIKKSLPGDCAIGHVRYSTSGSKFGVQPMFGKSGKF
                     GDFAIAHNGNLINISPIRKQLIKQECVFQSDIDTEVVVHLTASGEKDSFLESFIYALK
                     QIQGAYSFVAINQEVVIGVRDPSGIRPLVLGKLNGSYVLASETCALDIVNAEFVREIE
                     PGELVTIDRNGNLASAFPFPQQKSSFCIFEYVYFSRPDSIMENRSIYDIRKEIGKILA
                     EESPPKNNVDMVVPIPDSGIPAAIGYAKHSGLPMELGIIRNHYIGRTFIQPTAEVRKV
                     RIKLKFNANKHTLKGKNIILIDDSIVRGSTLTNIIVMLKDAGVKEIHLKISSPPIKHS
                     CFYGIDTPECKDLIAANKSVEEIKEVIGVDSLAFLSIDGLYQAVKGEVRNNATPQYCD
                     ACFTGDYPIGK"
                     /besthit="qcoverage 100 , hcoverage 100 , similarity 100
                     , identity 100 , evalue 0.0 , alnlength 461 , Bacteria ,
                     Proteobacteria , WP_015589386.1 MULTISPECIES:
                     amidophosphoribosyltransferase [Wolbachia]"
     gene            complement(12263..12769)
                     /locus_tag="wEsol_00359"
     CDS             complement(12263..12769)
                     /locus_tag="wEsol_00359"
                     /codon_start=1
                     /transl_table=11
                     /fullproduct="qcoverage 100 , hcoverage 100 , similarity
                     100 , identity 100 , evalue 1.13e-121 , alnlength 168 ,
                     Bacteria , Proteobacteria , WP_141456676.1 MULTISPECIES:
                     DNA-3-methyladenine glycosylase [unclassified Wolbachia]"
                     /cog="Mpg COG2094 200 3-methyladenine DNA glycosylase"
                     /pfam="Pur_DNA_glyco"
                     /tigr="TIGR00567 3mg 192 DNA-3-methyladenine glycosylase"
                     /product="
                     DNA-3-methyladenine glycosylase"
                     /translation="MSNIILPRNFYERPTLTVAGELLGKVLKFSNFSGVITEVEAYIG
                     MNDPACHAARGYTNRTSVMFGMPGFSYVYFIYGMYYCLNIVTEAEGFPAAVLIRGLKL
                     IEPLEANLGGPGILCKRLNITKEHNKQDLTISHKFCVYESHLKPDYICTPRIGISKGQ
                     EKFWRFKI"
                     /besthit="qcoverage 100 , hcoverage 100 , similarity 100
                     , identity 100 , evalue 1.13e-121 , alnlength 168 ,
                     Bacteria , Proteobacteria , WP_141456676.1 MULTISPECIES:
                     DNA-3-methyladenine glycosylase [unclassified Wolbachia]"
```

```
gene            complement(12886..13698)
                /locus_tag="wEsol_00360"
CDS             complement(12886..13698)
                /locus_tag="wEsol_00360"
                /codon_start=1
                /transl_table=11
                /fullproduct="qcoverage 100 , hcoverage 100 , similarity
                100 , identity 100 , evalue 1.02e-188 , alnlength 270 ,
                Bacteria , Proteobacteria , WP_015589388.1 hypothetical
                protein [Wolbachia endosymbiont of Drosophila simulans]"
                /translation="MLDSNETVSLIVDQNIAAGSVSGSIPNKDKCRYVTLDDEETWKR
                FFVDQNNPSTNTTNKVIVKPHVHKNSDGMYRYTAEETKYHGREIVGKAAKQIEEEISK
                NIDAKLIKLRETKNIDNSVKPVIRIALNDYIKEVNLHDILSGNMCKKYDVKTVTLLLP
                DQKTRGVRCQIDKDGARIYEVANGSYQMALKWYVDGQECKIKILISDDGTIKPIEGNG
                VTEEQLRANQEVKVGRQYEAKPLHEALASQLPQTQLQQSSEEVKSWSNFQQM"
                /product="hypothetical protein"
                /besthit="qcoverage 100 , hcoverage 100 , similarity 100
                , identity 100 , evalue 1.02e-188 , alnlength 270 ,
                Bacteria , Proteobacteria , WP_015589388.1 hypothetical
                protein [Wolbachia endosymbiont of Drosophila simulans]"
gene            complement(13776..14756)
                /locus_tag="wEsol_00361"
CDS             complement(13776..14756)
                /locus_tag="wEsol_00361"
                /codon_start=1
                /transl_table=11
                /fullproduct="qcoverage 100 , hcoverage 100 , similarity
                100 , identity 100 , evalue 5.45e-228 , alnlength 326 ,
                Bacteria , Proteobacteria , WP_015589389.1 MULTISPECIES:
                Holliday junction branch migration DNA helicase RuvB
                [unclassified Wolbachia]"
                /cog="RuvB COG2255 332 Holliday junction resolvasome,
                helicase subunit"
                /pfam="RuvB_N Pfam-B_736 Rad17 Pfam-B_11659 TIP49 AAA_16
                Bac_DnaA DUF815 NB-ARC Mg_chelatase AAA_5 AAA_22 NTPase_1
                AAA_3 AAA AAA_14 AAA_24 AAA_17 AAA_18 Pfam-B_17810
                RuvB_C"
                /tigr="TIGR00635 ruvB 305 Holliday junction DNA helicase
                RuvB"
                /product="Holliday junction
                branch migration DNA helicase RuvB"
                /translation="MKSISCGKEYTEDVRNINIRPEQLDDFVGQKDLIQNLKVFINAA
                QTRTEALDHVLLYGPPGLGKTTLAQIVSKELRVSFRATSGPLLSKAGDLAAVLTTLNA
                KDVLFIDEIHRLNRSIEEVLYTAMEDFCLDILVGEGPSTRTLRIDLPPFTLIGATTRL
                GLLSAPLRDRFGIPLHLEFYSFEELVDIIKRGARVLSAEIEEGAAREIACRARGTPRI
                ALRLLRRIRDFVEVKDDKKITYEVADSVLLKLGVDKMGLNKLDMNYLRFLFNTSGPVG
                IDTISIALSEDVGNIEETVEPYLIKISFVKRTPRGRVLTDQAKEYLSFQH"
                /besthit="qcoverage 100 , hcoverage 100 , similarity 100
                , identity 100 , evalue 5.45e-228 , alnlength 326 ,
                Bacteria , Proteobacteria , WP_015589389.1 MULTISPECIES:
                Holliday junction branch migration DNA helicase RuvB
                [unclassified Wolbachia]"
gene            complement(14753..15334)
                /locus_tag="wEsol_00362"
CDS             complement(14753..15334)
                /locus_tag="wEsol_00362"
                /codon_start=1
                /transl_table=11
                /fullproduct="qcoverage 100 , hcoverage 100 , similarity
                100 , identity 100 , evalue 4.06e-125 , alnlength 193 ,
```

```
                     Bacteria , Proteobacteria , WP_141456674.1 MULTISPECIES:
                     Holliday junction branch migration protein RuvA
                     [unclassified Wolbachia]"
                     /cog="RuvA COG0632 201 Holliday junction resolvasome,
                     DNA-binding subunit"
                     /pfam="RuvA_N RuvA_C"
                     /tigr="TIGR00084 ruvA 192 Holliday junction DNA helicase
                     RuvA"
                     /product="Holliday junction
                     branch migration protein RuvA"
                     /translation="MIGNLSGIVDEVRSDHIILNVNDVGYMVYLSAKTLSACAIGSRI
                     KLLIETYANNRENTTQLYGFISEEEQQCLRLLVKVSGVSYKTAMSILSKLTPEQLFLA
                     IMNEDKVALKMSGLGLKLINRIITELSGKVSKLEINNNNFNPINEDALSALINLGYEK
                     VKAYDTIKKIQDKSSNLDTKDIIRIALKELSIL"
                     /besthit="qcoverage 100 , hcoverage 100 , similarity 100
                     , identity 100 , evalue 4.06e-125 , alnlength 193 ,
                     Bacteria , Proteobacteria , WP_141456674.1 MULTISPECIES:
                     Holliday junction branch migration protein RuvA
                     [unclassified Wolbachia]"
     gene            complement(15983..16108)
                     /locus_tag="wEsol_00363"
     CDS             complement(15983..16108)
                     /locus_tag="wEsol_00363"
                     /codon_start=1
                     /transl_table=11
                     /product="hypothetical protein"
                     /translation="MSNLFKVNFQNPRIMYKTTGKMVRSGNLWLRRHTAAVSLSR"
     gene            complement(16196..16525)
                     /locus_tag="wEsol_00364"
     CDS             complement(16196..16525)
                     /locus_tag="wEsol_00364"
                     /codon_start=1
                     /transl_table=11
                     /pfam="Pfam-B_1658 Pfam-B_1913 Pfam-B_4169 SWIM
                     Pfam-B_1913"
                     /fullproduct="qcoverage 100 , hcoverage 100 , similarity
                     100 , identity 100 , evalue 1.02e-73 , alnlength 109 ,
                     Bacteria , Proteobacteria , WP_007548583.1 MULTISPECIES:
                     SWIM zinc finger family protein [Wolbachia]"
                     /translation="MRLYYKDITGLIKEPYRSRGRDYFDKRKVQIISVNESQAKSKVV
                     GSNVYRVTLKHDGPLLSGTCSCPAFVYYGPCKHMAATGFALVALDRKEYRSSARPDEQ
                     TLFERFY"
                     /product="SWIM zinc finger
                     family protein"
                     /besthit="qcoverage 100 , hcoverage 100 , similarity 100
                     , identity 100 , evalue 1.02e-73 , alnlength 109 ,
                     Bacteria , Proteobacteria , WP_007548583.1 MULTISPECIES:
                     SWIM zinc finger family protein [Wolbachia]"
     gene            complement(17489..18739)
                     /locus_tag="wEsol_00365"
     CDS             complement(17489..18739)
                     /locus_tag="wEsol_00365"
                     /codon_start=1
                     /transl_table=11
                     /fullproduct="qcoverage 100 , hcoverage 100 , similarity
                     100 , identity 100 , evalue 9.60e-304 , alnlength 416 ,
                     Bacteria , Proteobacteria , WP_007548582.1 MULTISPECIES:
                     tyrosine--tRNA ligase [Wolbachia]"
                     /cog="TyrS COG0162 401 Tyrosyl-tRNA synthetase"
                     /pfam="tRNA-synt_1b S4"
```

```
                     /tigr="TIGR00234 tyrS 406 tyrosine--tRNA ligase"
                     /product="tyrosine--tRNA
                     ligase"
                     /translation="MKHASEFLNFIQERGYLYQCTNIEGLDQLLLQSNYIVAYIGFDC
                     TAPSLHAGHLIQIMMLCHLQKFGYKPIVLLGGGTTKIGDPSFKDKARSILPVENINQN
                     TSSIRRILEKMVSFNDSKTGAMIVNNADWLDNIKYIDFLRDIGAYFSVNRMLSFDSVK
                     TRLDREQTLSFLEFNYMLLQAYDFIELNKKYDCRLQIGGSDQWGNIVNGIELGKKLNL
                     PELFGLTMPLLLNAQGKKMGKTESGAVWLDDNMLKPYDYWQYFRNVDDQDVGRFLRLL
                     TDVPIDEIRKLESLKDQEINEAKKVLATEVTKICHGDKEAELAQFAAISAFENEDSSL
                     LPDYIIKKEQVASGISLVDLLHDTGFEPSKGAAKRLIQGNGCKINDNVVNNIDHVINF
                     ESFKDQPFIKLSAGKKRHIKVVVD"
                     /besthit="qcoverage 100 , hcoverage 100 , similarity 100
                     , identity 100 , evalue 9.60e-304 , alnlength 416 ,
                     Bacteria , Proteobacteria , WP_007548582.1 MULTISPECIES:
                     tyrosine--tRNA ligase [Wolbachia]"
     gene            complement(18741..18980)
                     /locus_tag="wEsol_00366"
     CDS             complement(18741..18980)
                     /locus_tag="wEsol_00366"
                     /codon_start=1
                     /transl_table=11
                     /fullproduct="qcoverage 100 , hcoverage 100 , similarity
                     100 , identity 100 , evalue 5.36e-53 , alnlength 79 ,
                     Bacteria , Proteobacteria , WP_041573543.1 MULTISPECIES:
                     hypothetical protein [unclassified Wolbachia]"
                     /translation="MELIIQIAGTQLHKRCNFEGNSCTGSGVMKVADTGIQFFMQFHR
                     KRCIVCLFIIKFQCLGTGFSIRVTFSINDRIDWGL"
                     /product="hypothetical protein"
                     /besthit="qcoverage 100 , hcoverage 100 , similarity 100
                     , identity 100 , evalue 5.36e-53 , alnlength 79 ,
                     Bacteria , Proteobacteria , WP_041573543.1 MULTISPECIES:
                     hypothetical protein [unclassified Wolbachia]"
     gene            complement(18971..19288)
                     /locus_tag="wEsol_00367"
     CDS             complement(18971..19288)
                     /locus_tag="wEsol_00367"
                     /codon_start=1
                     /transl_table=11
                     /fullproduct="qcoverage 100 , hcoverage 100 , similarity
                     100 , identity 100 , evalue 4.95e-70 , alnlength 105 ,
                     Bacteria , Proteobacteria , WP_015589391.1 MULTISPECIES:
                     ribosome silencing factor [Wolbachia]"
                     /cog="COG0799 COG0799 115 Uncharacterized homolog of
                     plant Iojap protein"
                     /pfam="Oligomerisation"
                     /tigr="TIGR00090 iojap_ybeB 99 iojap-like
                     ribosome-associated protein"
                     /product="ribosome silencing
                     factor"
                     /translation="MLSDMKSIRSTIEEIIDQNKGHDIVTFDVQNKTVIAKYMIIASG
                     DSSRHVKALAEHVIKSLKPHDKIDVEGMDEGNWVVLNFQGIMVHIFRPEVREYYKIEE
                     LWN"
                     /besthit="qcoverage 100 , hcoverage 100 , similarity 100
                     , identity 100 , evalue 4.95e-70 , alnlength 105 ,
                     Bacteria , Proteobacteria , WP_015589391.1 MULTISPECIES:
                     ribosome silencing factor [Wolbachia]"
     gene            19393..20079
                     /locus_tag="wEsol_00368"
     CDS             19393..20079
                     /locus_tag="wEsol_00368"
```

/codon_start=1
/transl_table=11
/pfam="AAA_33 AAA_18 AAA_17 Pfam-B_5209"
/fullproduct="qcoverage 100 , hcoverage 100 , similarity 99.6 , identity 99.1 , evalue 1.05e-151 , alnlength 228 , Bacteria , Proteobacteria , AGK00351.1 hypothetical protein wHa_09360 [Wolbachia endosymbiont of Drosophila simulans wHa]"
/translation="MFLACTMYILLCIIENSFLLVFIIGKTLIYLIGFPGSGKFTTAK
ELCKIIDAVIVSNNLFNNIIFDIVKLQNAEVPDDLWEKIFAVRENVLAILEKHYVKSK
HYIFTNELIEGDPYDQRLYNSVVNLSKKMDMEIFSVVLHCNNEKLVRRVQSEERYQEN
KITDPDFAIKKIEGKRLFIPEGSLEIDNSNLSAKEVAKKIVEEMKKEKNGKLIKTSVT
NHLKTERTRG"
/product="hypothetical protein"
/besthit="qcoverage 100 , hcoverage 100 , similarity 99.6 , identity 99.1 , evalue 1.05e-151 , alnlength 228 , Bacteria , Proteobacteria , AGK00351.1 hypothetical protein wHa_09360 [Wolbachia endosymbiont of Drosophila simulans wHa]"

gene 20072..20560
/locus_tag="wEsol_00369"

CDS 20072..20560
/locus_tag="wEsol_00369"
/codon_start=1
/transl_table=11
/fullproduct="qcoverage 100 , hcoverage 100 , similarity 99.4 , identity 99.4 , evalue 5.42e-117 , alnlength 162 , Bacteria , Proteobacteria , WP_006279569.1 MULTISPECIES: RNA pyrophosphohydrolase [Wolbachia]"
/cog="MutT COG0494 161 NTP pyrophosphohydrolases including oxidative damage repair enzymes"
/pfam="NUDIX"
/product="RNA
pyrophosphohydrolase"
/translation="MVEQKDKYRPCVGIMLFNRQGHAFIGKRFDSDSYWQMPQGGVDD
GEELEQAALRELLEEVGTNKVKVITKSKDWIYYNLPEEVIPICWNGKYSGQKQRWFLM
KFCGEDEDIDINYTVHPEFKEWRWQGIDSLVASAISFKKEVYKTVIEEFSSIIKASTI
SS"
/besthit="qcoverage 100 , hcoverage 100 , similarity 99.4 , identity 99.4 , evalue 5.42e-117 , alnlength 162 , Bacteria , Proteobacteria , WP_006279569.1 MULTISPECIES: RNA pyrophosphohydrolase [Wolbachia]"

gene 20557..21324
/locus_tag="wEsol_00370"

CDS 20557..21324
/locus_tag="wEsol_00370"
/codon_start=1
/transl_table=11
/fullproduct="qcoverage 100 , hcoverage 100 , similarity 100 , identity 100 , evalue 1.34e-181 , alnlength 255 , Bacteria , Proteobacteria , WP_006280340.1 MULTISPECIES: TatD family hydrolase [Wolbachia]"
/cog="TatD COG0084 256 Mg-dependent DNase"
/pfam="TatD_DNase"
/tigr="TIGR00010 TIGR00010 253 hydrolase, TatD family"
/product="TatD family
hydrolase"
/translation="MIVDSHCHLIYFSDDEIPKVISRAEQNGVKVLHNICVSIDDVPK
LLKISSSYDQVYSSVGIHPLDAAIENGECIHVDELVEFTKNQKVISIGETGLDFYKSD
NKDNQKKSFALHIEASRITGLPLVIHTRSADNEMIDMLRSEMKAGAFSGVMHCFASSK

```
                     ELAYQSIDLGLCISFSGIITFKNASLLREIAQNVPRERVLVETDAPYLSPEPYRGKKN
                     EPAMVKYVVDCLAELWNESPEQVAKVTTSNFFRLFEL"
                     /besthit="qcoverage 100 , hcoverage 100 , similarity 100
                     , identity 100 , evalue 1.34e-181 , alnlength 255 ,
                     Bacteria , Proteobacteria , WP_006280340.1 MULTISPECIES:
                     TatD family hydrolase [Wolbachia]"
     gene            complement(21352..22302)
                     /locus_tag="wEsol_00371"
     CDS             complement(21352..22302)
                     /locus_tag="wEsol_00371"
                     /codon_start=1
                     /transl_table=11
                     /fullproduct="qcoverage 100 , hcoverage 100 , similarity
                     100 , identity 100 , evalue 3.51e-219 , alnlength 316 ,
                     Bacteria , Proteobacteria , WP_007548577.1 MULTISPECIES:
                     malate dehydrogenase [Wolbachia]"
                     /cog="Mdh COG0039 313 Malate/lactate dehydrogenases"
                     /pfam="UDPG_MGDP_dh_N Ldh_1_N Glyco_hydro_4 Ldh_1_C"
                     /tigr="TIGR01763 MalateDH_bact 305 malate dehydrogenase,
                     NAD-dependent"
                     /product="malate
                     dehydrogenase"
                     /translation="MTVQRKKISLIGAGNIGGTLTHMIALRELGDVVLLDISDGIPQG
                     KALDIAESSPIDGFNVNITGTNRYEDIKNSDAIIITAGIARKPGMSRDDLLQTNAKVM
                     KEVGENIKKYSPNAFVIVVTNPLDAMVSVVHKFSNLPTNMIVGMAGVLDSSRFRYFLA
                     SELNISVEDISAFVLGGHGDTMVPLINCASVAGVPLTQIIDMGLITQKKVDEIVERTR
                     NGGKEIVDLLKSGSAYYAPASSAICMLESYLKDKRRILPCAAYLNGEYGVEELFIGVP
                     VIIGKNGIEKILEVKMNDSEQEMFNKSVNSVRELVKSLGS"
                     /besthit="qcoverage 100 , hcoverage 100 , similarity 100
                     , identity 100 , evalue 3.51e-219 , alnlength 316 ,
                     Bacteria , Proteobacteria , WP_007548577.1 MULTISPECIES:
                     malate dehydrogenase [Wolbachia]"
     gene            complement(22306..22878)
                     /locus_tag="wEsol_00372"
     CDS             complement(22306..22878)
                     /locus_tag="wEsol_00372"
                     /codon_start=1
                     /transl_table=11
                     /fullproduct="qcoverage 100 , hcoverage 100 , similarity
                     100 , identity 100 , evalue 1.49e-139 , alnlength 190 ,
                     Bacteria , Proteobacteria , WP_007548576.1 MULTISPECIES:
                     NADH-quinone oxidoreductase subunit C [Wolbachia]"
                     /cog="NuoC COG0852 176 NADH:ubiquinone oxidoreductase 27
                     kD subunit"
                     /pfam="Complex1_30kDa"
                     /product="NADH-quinone
                     oxidoreductase subunit C"
                     /translation="MDKTAKYIQKKTKCECIQKDDGIIVIYSTLDEIEKHLFFLRDDE
                     KCRFELLVDIFGVDYPNREKRFELIYNLLSIVHNIRVHIKLQLCESDMPPSVAKIFST
                     ALWFEREVFDMYGIEFSDHPDLRRILTDYGFKGHPMLKDFPLTGYEEVRYDIETKKVV
                     YNPIDLPQDFRMFDSLSPWEGKTIKVNTKE"
                     /besthit="qcoverage 100 , hcoverage 100 , similarity 100
                     , identity 100 , evalue 1.49e-139 , alnlength 190 ,
                     Bacteria , Proteobacteria , WP_007548576.1 MULTISPECIES:
                     NADH-quinone oxidoreductase subunit C [Wolbachia]"
     gene            complement(22871..23377)
                     /locus_tag="wEsol_00373"
     CDS             complement(22871..23377)
                     /locus_tag="wEsol_00373"
                     /codon_start=1
```

```
                     /transl_table=11
                     /fullproduct="qcoverage 100 , hcoverage 100 , similarity
                     100 , identity 100 , evalue 5.80e-124 , alnlength 168 ,
                     Bacteria , Proteobacteria , WP_174516933.1 NADH-quinone
                     oxidoreductase subunit B [Wolbachia endosymbiont of
                     Cardiocondyla obscurior]"
                     /cog="NuoB COG0377 194 NADH:ubiquinone oxidoreductase 20
                     kD subunit and related Fe-S oxidoreductases"
                     /pfam="Oxidored_q6"
                     /tigr="TIGR01957 nuoB_fam 145 NADH-quinone
                     oxidoreductase, B subunit"
                     /product="NADH-quinone oxidoreductase
                     subunit B"
                     /translation="MTNQILSNDDWGRYKKEGFLVTKFGDLTDYVMNWARSGSLWPMT
                     FGLACCAVEMMHTASSRYDLDRYGIMFRASPRQSDVMIVAGTLTNKMAAALRKVYDQM
                     ADPKYVISMGSCANGGGYYHYSYSVVQGCDRIVPVDVYVPGCPPTAEALLYGMLCLQN
                     KIKRTRNG"
                     /besthit="qcoverage 100 , hcoverage 100 , similarity 100
                     , identity 100 , evalue 5.80e-124 , alnlength 168 ,
                     Bacteria , Proteobacteria , WP_174516933.1 NADH-quinone
                     oxidoreductase subunit B [Wolbachia endosymbiont of
                     Cardiocondyla obscurior]"
     gene            complement(23381..23734)
                     /locus_tag="wEsol_00374"
     CDS             complement(23381..23734)
                     /locus_tag="wEsol_00374"
                     /codon_start=1
                     /transl_table=11
                     /fullproduct="qcoverage 100 , hcoverage 100 , similarity
                     100 , identity 100 , evalue 3.18e-74 , alnlength 117 ,
                     Bacteria , Proteobacteria , WP_006279576.1 MULTISPECIES:
                     NADH-quinone oxidoreductase subunit A [Wolbachia]"
                     /cog="NuoA COG0838 123 NADH:ubiquinone oxidoreductase
                     subunit 3 (chain A)"
                     /pfam="Oxidored_q4"
                     /product="NADH-quinone
                     oxidoreductase subunit A"
                     /translation="MSEYLTIVIFICVSIVVSFALGILPMFLAVSKPDGEKLSTYECG
                     FNPLSSARKNFDIKFYLVSILFIIFDLEIAFLFPWAVSLSKISYGGFWSMMVFLAILT
                     IGFIYEWCKGALEWE"
                     /besthit="qcoverage 100 , hcoverage 100 , similarity 100
                     , identity 100 , evalue 3.18e-74 , alnlength 117 ,
                     Bacteria , Proteobacteria , WP_006279576.1 MULTISPECIES:
                     NADH-quinone oxidoreductase subunit A [Wolbachia]"
     gene            complement(23753..24121)
                     /locus_tag="wEsol_00375"
     CDS             complement(23753..24121)
                     /locus_tag="wEsol_00375"
                     /codon_start=1
                     /transl_table=11
                     /fullproduct="qcoverage 100 , hcoverage 100 , similarity
                     100 , identity 100 , evalue 2.37e-85 , alnlength 122 ,
                     Bacteria , Proteobacteria , WP_015589394.1 MULTISPECIES:
                     HIT domain-containing protein [Wolbachia]"
                     /cog="Hit COG0537 138 Diadenosine tetraphosphate (Ap4A)
                     hydrolase and other HIT family hydrolases"
                     /pfam="DcpS_C HIT"
                     /product="HIT
                     domain-containing protein"
                     /translation="MSNEVYDSDNIFAQILRGELPCEKVHENENVLAFHDKYPDAPVH
```

```
                ILVIPKNQYISYDDFILKAPEEEIADFFKTVREIAHKYNLEKTGYRLVTNYGENGEQV
                VPHFHVHILGGKKLGKHVNS"
                /besthit="qcoverage 100 , hcoverage 100 , similarity 100
                , identity 100 , evalue 2.37e-85 , alnlength 122 ,
                Bacteria , Proteobacteria , WP_015589394.1 MULTISPECIES:
                HIT domain-containing protein [Wolbachia]"
gene            complement(24125..24382)
                /locus_tag="wEsol_00376"
CDS             complement(24125..24382)
                /locus_tag="wEsol_00376"
                /codon_start=1
                /transl_table=11
                /fullproduct="qcoverage 100 , hcoverage 100 , similarity
                100 , identity 100 , evalue 1.37e-51 , alnlength 85 ,
                Bacteria , Proteobacteria , WP_006279579.1 MULTISPECIES:
                hypothetical protein [Wolbachia]"
                /translation="MSEAKKIEERHHSLTVPGDMYHLQPGVITGKRVSGIEPFLKEQQ
                DMVKVRELVSSLNLEDASGEAIKPLSTPNVEKNEERGKGRS"
                /product="hypothetical protein"
                /besthit="qcoverage 100 , hcoverage 100 , similarity 100
                , identity 100 , evalue 1.37e-51 , alnlength 85 ,
                Bacteria , Proteobacteria , WP_006279579.1 MULTISPECIES:
                hypothetical protein [Wolbachia]"
gene            complement(24399..25178)
                /locus_tag="wEsol_00377"
CDS             complement(24399..25178)
                /locus_tag="wEsol_00377"
                /codon_start=1
                /transl_table=11
                /fullproduct="qcoverage 100 , hcoverage 100 , similarity
                100 , identity 100 , evalue 8.70e-180 , alnlength 259 ,
                Bacteria , Proteobacteria , WP_015589395.1 MULTISPECIES:
                hypothetical protein [unclassified Wolbachia]"
                /translation="MLTRENEKSTSILAKVGNAASGVGSFLGRGVKVLVKGAIYPIWP
                KNWFKSWDEMRHDIKNGGRSIIGNPHEEYDPPIKTTQASKDLGVQITEQKKGTGKVSF
                LASRTLTGDIPYLSPEQLDKIKKDLPNVTIFREENKIVIEVDVKKIIEDIKTDSKNKG
                KSPEEIKKLALQGIADSINGDLKKLAGITGGEFKIHTDLKLLNGIAEKLYKEYDNQKQ
                SSDKVVKSDVGQPSETIASGEKKWAQEAIKPGKSQSCGMQK"
                /product="hypothetical protein"
                /besthit="qcoverage 100 , hcoverage 100 , similarity 100
                , identity 100 , evalue 8.70e-180 , alnlength 259 ,
                Bacteria , Proteobacteria , WP_015589395.1 MULTISPECIES:
                hypothetical protein [unclassified Wolbachia]"
gene            25362..25775
                /locus_tag="wEsol_00378"
CDS             25362..25775
                /locus_tag="wEsol_00378"
                /codon_start=1
                /transl_table=11
                /fullproduct="qcoverage 83.9416058394161 , hcoverage
                94.2622950819672 , similarity 100 , identity 97.4 ,
                evalue 4.37e-76 , alnlength 115 , Bacteria ,
                Proteobacteria , EAL59246.1
                UDP-N-acetylmuramoylalanyl-D-glutamyl-2,6-diaminopimelate-
                D-alanyl-D-alanyl ligase, partial [Wolbachia endosymbiont
                of Drosophila ananassae]"
                /cog="MurF COG0770 451 UDP-N-acetylmuramyl pentapeptide
                synthase"
                /pfam="Mur_ligase"
                /product="EAL59246.1
```

```
                UDP-N-acetylmuramoylalanyl-D-glutamyl-2,6-diaminopimelate-
                D-alanyl-D-alanyl ligase, partial"
                /translation="MFKWNTNNIIDATGGRDVNGCNWIHSSNISTDTRSIKKGDLFIA
                LKGRNFDGHNFLHEAFLKGAVAAIVSEGKYKNFPLIIVQDTLKALHDMASYYIRNVLI
                DAKVIAVTGSVGKNHYKGYATHCFIAIWSVPCKRR"
                /besthit="qcoverage 83.9416058394161 , hcoverage
                94.2622950819672 , similarity 100 , identity 97.4 ,
                evalue 4.37e-76 , alnlength 115 , Bacteria ,
                Proteobacteria , EAL59246.1
                UDP-N-acetylmuramoylalanyl-D-glutamyl-2,6-diaminopimelate-
                D-alanyl-D-alanyl ligase, partial [Wolbachia endosymbiont
                of Drosophila ananassae]"
gene            25723..26892
                /locus_tag="wEsol_00379"
CDS             25723..26892
                /locus_tag="wEsol_00379"
                /codon_start=1
                /transl_table=11
                /product="hypothetical protein"
                /pfam="Mur_ligase_M Mur_ligase_C"
                /translation="MLHTVLSQYGVSHANEGNLNNNIGLPLTILKAPKNCQYLILEMG
                MNKAGEIKELSKISNPNIAVITNIEPAHTENFSSLFDVAQAKLEILYGMKNNGTLVLS
                RDNKYYDYLSSHANRNVISFGKDKNAEVCLLNLIRNDESSVISALSSVIPVLDYLDPG
                SSIANEHIRRLYNKDWIPASRAGMTSNRVANRLSLKIRLSDNQIINCNLRVQGEHFAY
                SLLAVAAVVQSLGLDLSKLPLALKNFSVAKGRGNIHKIKYNRKYIHLIDDSYNASPAS
                MKTAIKTLSTYSNQRKVALLGDMLELGDESIEFHTDLVKIITENNIDKVYTVGKFMLE
                LHELLPDNIRGTHFNDSNQLKSDLANIIQNNDAILVKGSRGMKMDLIVREFVIKY"
                /besthit="qcoverage 100 , hcoverage 76.4243614931238 ,
                similarity 100 , identity 100 , evalue 1.90e-273 ,
                alnlength 389 , Bacteria , Proteobacteria ,
                WP_015589396.1
                UDP-N-acetylmuramoyl-tripeptide--D-alanyl-D-alanine
                ligase [Wolbachia endosymbiont of Drosophila simulans]"
gene            26943..27893
                /locus_tag="wEsol_00380"
CDS             26943..27893
                /locus_tag="wEsol_00380"
                /codon_start=1
                /transl_table=11
                /fullproduct="qcoverage 100 , hcoverage 100 , similarity
                100 , identity 100 , evalue 7.70e-223 , alnlength 316 ,
                Bacteria , Proteobacteria , WP_015589397.1 MULTISPECIES:
                complex I NDUFA9 subunit family protein [unclassified
                Wolbachia]"
                /cog="COG0702 COG0702 275 Predicted
                nucleoside-diphosphate-sugar epimerases"
                /pfam="RmlD_sub_bind adh_short NAD_binding_10 Epimerase
                NmrA Saccharop_dh 3Beta_HSD F420_oxidored NAD_binding_4
                NAD_binding_4 RmlD_sub_bind"
                /product="complex I NDUFA9
                subunit family protein"
                /translation="MTKRVIIFGGTGFIGKHIVRRLAAEGYLIKIFTRNQEKAACLKL
                CGNLGQISIFKGDFFDEKSVLDGMEECDVAINLVGILYEAKKHDFYAVHVKIAERIAK
                AAKMKNVPMMIHFSAMGIENSKLSKYAKSKLEGEKAVTSAFQEAIIIRPSLVFGKEDN
                FFNKFARLATILPFLPLIGNGTTKFQPICVTDLAEVVYRIISFNKQDKKIYNMGGPKV
                YSFKSLLKFILNVTNRKCLLVNVSFPMAKLIAFFLENKIISMLLKPITGDTNPMLTQD
                QVKVMMNSSIEKSVDLETMKVRPLAIENVVPEYLKIYRKY"
                /besthit="qcoverage 100 , hcoverage 100 , similarity 100
                , identity 100 , evalue 7.70e-223 , alnlength 316 ,
                Bacteria , Proteobacteria , WP_015589397.1 MULTISPECIES:
```

```
                     complex I NDUFA9 subunit family protein [unclassified
                     Wolbachia]"
     gene            27910..28320
                     /locus_tag="wEsol_00381"
     CDS             27910..28320
                     /locus_tag="wEsol_00381"
                     /codon_start=1
                     /transl_table=11
                     /product="hypothetical protein"
                     /translation="MGFEPTTHSLEGCCSIQLSYGHTTYIKCFLVQVKSSTSCEYDVY
                     LATPCHCSCVSRAAKIGCEKSIYFRKYYIETYLTPFASALIMIVKLLYLLSPICRLKG
                     KDYLMYRRLMFNFLQYIDTVCLYKTSSTSRFLSK"
                     /besthit="qcoverage 46.3235294117647 , hcoverage
                     91.304347826087 , similarity 100 , identity 98.4 , evalue
                     6.28e-33 , alnlength 63 , Bacteria , Proteobacteria ,
                     KAB2977954.1 hypothetical protein DEF52_04360 [Wolbachia
                     endosymbiont of Nasonia oneida]"
     gene            29076..30527
                     /locus_tag="wEsol_00382"
     CDS             29076..30527
                     /locus_tag="wEsol_00382"
                     /codon_start=1
                     /transl_table=11
                     /pfam="Terminase_6"
                     /fullproduct="qcoverage 100 , hcoverage 100 , similarity
                     99.6 , identity 99.0 , evalue 0.0 , alnlength 483 ,
                     Bacteria , Proteobacteria , WP_141456669.1 MULTISPECIES:
                     phage terminase large subunit [unclassified Wolbachia]"
                     /translation="MNFLKFIELCFQTVVPGCEYNDYQYIKVIADRLEAASAGEVKRI
                     IFNMPPRSMKSMCISVAWPAWILGNQPTARIIVASYSQRLSEKHSLDTRCVMQSSWYR
                     ALFPEIELSKDQNTKYKFQTVQRGYRIATSVGGTLTGEGGDFIIVDDPLSSAQALSET
                     LRKRAINWFDQTLVTRLNDRKKGVIVLVMHRLHQEDLTGHLLSKPKNIWHHICLPMIS
                     ENKQVIYSIKKPMPPVPVIRVAQHCATRRLYNKPASPVRILYSREESQLLYPLDGGKE
                     EIEMIKVELGSYAFAAQYQQNPLPLSSGIIKPEWLKRYKKFPDNLSHITQSWDTAVST
                     SDMSNFSVCTTWAKIDNKFYLLDVYRAKLEYPKLKEQVLSLAARWTPHAILIEAKTSG
                     QQLVQELKTNSDLPIIEIVPHDDKLTRFHQIVPIIESGKVFLPHQAVWLNDFEYEILM
                     FPETRHDDQVDSTVQYLQWVRNSTSRVAVIRAL"
                     /product="phage terminase
                     large subunit"
                     /besthit="qcoverage 100 , hcoverage 100 , similarity 99.6
                     , identity 99.0 , evalue 0.0 , alnlength 483 , Bacteria ,
                     Proteobacteria , WP_141456669.1 MULTISPECIES: phage
                     terminase large subunit [unclassified Wolbachia]"
     gene            30919..33369
                     /locus_tag="wEsol_00383"
     CDS             30919..33369
                     /locus_tag="wEsol_00383"
                     /codon_start=1
                     /transl_table=11
                     /fullproduct="qcoverage 100 , hcoverage 100 , similarity
                     100 , identity 99.9 , evalue 0.0 , alnlength 816 ,
                     Bacteria , Proteobacteria , WP_141456668.1 MULTISPECIES:
                     type I DNA topoisomerase [unclassified Wolbachia]"
                     /cog="TopA COG0550 570 Topoisomerase IA"
                     /pfam="Toprim Toprim_4 Topoisom_bac zf-C4_Topoisom
                     Toprim_C_rpt Toprim_C_rpt Toprim_C_rpt"
                     /tigr="TIGR01051 topA_bact 632 DNA topoisomerase I"
                     /product="type I DNA
                     topoisomerase"
                     /translation="MALLIVESPAKAKTIGKYLSKEFKVAASFGHVRDLPAKNGSVDP
```

DNDFAIKYETIEKAEKYIKELVKAASKTSDIYLATDPDREGEAMAWHVIETLKEKKAI
SNENNIYRVVFNEITKRAVQEAIKNPREINMDLVRAQQARRALDYLVGFSLSPLLWTK
LSGSKSAGRVQSVALKIICEREDEISKFITQEYWSIKAEMQNSKDEAFFAMLSHYDNK
KLEKFDIKNEEEAKNLVREIESRQYAVSTVERKQVKRNPLPPFITSSLQQDAVNKLYF
NVKSVMRIAQNLYEGIDIGGETVGLITYMRTDGFHIADEAINSIRGSIKSLYGDKYLP
QSPRKYVKKVKNAQEAHEAIRPTDINRTPGSIKDYLTPEQFKLYDLIWKRTIASQMES
AILDQVVVEISSTDQKVILRASGSSIFFDGFYRVYQDNIEAENEGLLPAMKEGEACKL
ISVEPKQHFTQPPPRYSEASIVKKMEEIGIGRPSTYATIISVLQDREYVSLDSKRFIP
SSRGKIVTIFLETFFQRCVEYDFTAQMEEKLDLISNGHADWKKELGHFWVPFFGHVNS
VKQMTHDEVFSGIHDLVIDWFCSEEGKKEMNTKCPACSDGILKLNFGKAGVFLGCSNY
PECNHTKEITGSNDNSEYPKSLGIDDITNQEVMIKKGPFGLYLEFNNELKKKKTVSIP
KDINVNEIDLSTATQLLSLPKVIGEHPETGKEVKIGLGRFGYYIFYDGRYFSLKKSSK
EVLNRELSEVVQIIANSPCKELKSLGVNEKGKEVFICNGRYGFYIKCGKTNVALGKSA
DIESIDLEKALELIKNKK"
/besthit="qcoverage 100 , hcoverage 100 , similarity 100
, identity 99.9 , evalue 0.0 , alnlength 816 , Bacteria ,
Proteobacteria , WP_141456668.1 MULTISPECIES: type I DNA
topoisomerase [unclassified Wolbachia]"
gene 33427..34053
/locus_tag="wEsol_00384"
CDS 33427..34053
/locus_tag="wEsol_00384"
/codon_start=1
/transl_table=11
/pfam="LRR_4 LRR_8 LRR_6 Pfam-B_10570 LRR_4 LRR_6 LRR_4
MASE1"
/fullproduct="qcoverage 100 , hcoverage 100 , similarity
100 , identity 100 , evalue 1.88e-70 , alnlength 208 ,
Bacteria , Proteobacteria , WP_174516853.1 hypothetical
protein [Wolbachia endosymbiont of Cardiocondyla
obscurior]"
/translation="MIFYTQGDTPALCRLDYTDVSKLVGIPNITRLDLTECKISVKDA
KELAKLTNLTSLHLGKCKISVGVAKELTKLTNLTSLNLIGCKISNEVAKELDKLKKNL
PSLNIIGYKISGKGANVKLQDEVSNSQEKLKSSSKYYICGATVGLVIGLVAAYCLGAA
ALTPVGVSVAAFIASAVVGALLGAGVGYAVSTYLESTELNNMQPRRSC"
/product="hypothetical protein"
/besthit="qcoverage 100 , hcoverage 100 , similarity 100
, identity 100 , evalue 1.88e-70 , alnlength 208 ,
Bacteria , Proteobacteria , WP_174516853.1 hypothetical
protein [Wolbachia endosymbiont of Cardiocondyla
obscurior]"
gene complement(34095..34901)
/locus_tag="wEsol_00385"
CDS complement(34095..34901)
/locus_tag="wEsol_00385"
/codon_start=1
/transl_table=11
/fullproduct="qcoverage 100 , hcoverage 100 , similarity
100 , identity 100 , evalue 2.64e-199 , alnlength 268 ,
Bacteria , Proteobacteria , WP_015589401.1 MULTISPECIES:
rhodanese-related sulfurtransferase [unclassified
Wolbachia]"
/cog="COG1054 COG1054 308 Predicted sulfurtransferase"
/pfam="Rhodanese"
/product="rhodanese-related
sulfurtransferase"
/translation="MSFVIATFYHFVELSNYYDMKDEIKATCNNVELKGTILLAEEGV
NATISGERNAIDKIFDFLRSDYRLRDLTWKESAAEYQPFSKMKVKLKREIVNLGVSNL
DISLKGKYVDPEHWDDFTSQPDVLVIDTRNEYEVKLGKFKNAINPHTQCFREFPQWTE
SFSESKDLKVAMYCTGGIRCEKSTAYMKNLGFNDVYHLKGGILSYLEKTHNKNDNWEG

```
                     ECFVFDDRIAVDNSLTPSNTIKCILCSNQVSTDKLKSVPRGQVVCFNCKL"
                     /besthit="qcoverage 100 , hcoverage 100 , similarity 100
                     , identity 100 , evalue 2.64e-199 , alnlength 268 ,
                     Bacteria , Proteobacteria , WP_015589401.1 MULTISPECIES:
                     rhodanese-related sulfurtransferase [unclassified
                     Wolbachia]"
     gene            35031..36629
                     /locus_tag="wEsol_00386"
     CDS             35031..36629
                     /locus_tag="wEsol_00386"
                     /codon_start=1
                     /transl_table=11
                     /fullproduct="qcoverage 100 , hcoverage 100 , similarity
                     100 , identity 100 , evalue 0.0 , alnlength 532 ,
                     Bacteria , Proteobacteria , WP_015589402.1 MULTISPECIES:
                     iron ABC transporter permease [unclassified Wolbachia]"
                     /cog="ThiP COG1178 540 ABC-type Fe3+ transport system,
                     permease component"
                     /pfam="BPD_transp_1 Pfam-B_3338 Pfam-B_3338 BPD_transp_1"
                     /product="iron ABC
                     transporter permease"
                     /translation="MFLRVFKNIFLFLVSILFVCPILSLISILFTDTANSGWVISTLF
                     PEYILSTLILMIGVGGISFIFGVIPAWLTTFFSFPGSKIFEIALFFPISIPGYIVAFV
                     YVNTLEFSGPVQSSLRDILEWSKGDYWFPEIKSLGGGILVMGFSLYPYVYMLVRSSLK
                     NVSNSVTIASTLGFSSLKSLFSVIIPSIRPSIIAGLSLVLMEVITDFGTPQFLAIDTF
                     TTGIYRTWFLLHDKYSTTVLAVAELIFVVILIAIEKKLQKKGISYSSINTNSDYHNKR
                     SISGAIPLIFAYLMCVLPILIGFILPMIPLIYWSIEKGFFIYEARFYNIIANSISLSF
                     ITALISISIAIMIGYTARKNKVINNIARLISLGYAIPNAIIAISIIIFLSRISSFITQ
                     YVVEISLVGTIGALVYSYLFRFFAISFKAVESGLKKTPNEIEWTAYTMGHGPISTCLN
                     VHIPLIKKSILSGFLLVFMDTIKELTATLIIRPFNFETISTRIYELVSDERYREAAPF
                     SLIIVIIGLISTITLFTLDDKDKK"
                     /besthit="qcoverage 100 , hcoverage 100 , similarity 100
                     , identity 100 , evalue 0.0 , alnlength 532 , Bacteria ,
                     Proteobacteria , WP_015589402.1 MULTISPECIES: iron ABC
                     transporter permease [unclassified Wolbachia]"
     gene            complement(36640..>37288)
                     /locus_tag="wEsol_00387"
     CDS             complement(36640..>37288)
                     /locus_tag="wEsol_00387"
                     /codon_start=1
                     /transl_table=11
                     /fullproduct="qcoverage 100 , hcoverage 93.8864628820961
                     , similarity 100 , identity 100 , evalue 9.25e-136 ,
                     alnlength 215 , Bacteria , Proteobacteria ,
                     WP_103412447.1 MULTISPECIES: Rpn family
                     recombination-promoting nuclease/putative transposase,
                     partial [unclassified Wolbachia]"
                     /pfam="PDDEXK_2 Pfam-B_9730 Pfam-B_12519"
                     /tigr="TIGR01784 T_den_put_tspse 273 conserved
                     hypothetical protein"
                     /product="Rpn family
                     recombination-promoting nuclease/putative transposase,
                     partial"
                     /translation="QLARDKGFEKRAQLYAAKAYSRQLDKSGNYIDLKKVFFIAISNC
                     NLLPEEVDYISTHNIRDIKTNGHYLKDFQFVFIELPKFAKNKVEQLESIVERWCFFFK
                     YAEETTEEDLKEIAEKAPIIKLAYDELDKFRWNEKDLVAYEERIMDLRKEEAILEYRL
                     DLAEEKGIQKGIEKGKIEGKIEVAKAMLANNVDVNTIVKFTGLSISEIEELSGNL"
                     /besthit="qcoverage 100 , hcoverage 93.8864628820961 ,
                     similarity 100 , identity 100 , evalue 9.25e-136 ,
                     alnlength 215 , Bacteria , Proteobacteria ,
```

```
                     WP_103412447.1 MULTISPECIES: Rpn family
                     recombination-promoting nuclease/putative transposase,
                     partial [unclassified Wolbachia]"
     gene            complement(27902..27975)
                     /locus_tag="wEsol_tRNA-02"
     tRNA            complement(27902..27975)
                     /locus_tag="wEsol_tRNA-02"
                     /gene="tRNA-Arg"
                     /intronpos="0..0"
                     /program="tRNAscan-SE"
                     /cove_score="80.0"
                     /evidence=predicted
                     /type="Arg"
                     /anticodon=TCT
                     /product="tRNA-Arg (TCT)"
ORIGIN
        1 taagagttat agtgctgaca ttagccaact gagaaacaga gtttttgacc gagactttgg
       61 agcaaagctt atgtgttaag gtggcacata gaggagtgct ataaacgact caaagtagga
      121 gcagagttag agaatttttc tggagaggct gtattacaag aattttgggc aaacttggtc
      181 atgtgcaata tattatcgct tcatatgtgt gatgcacaag ggccttggaa cccagatcaa
      241 attactgagt atcgtttaaa tttttcagtt ttatttggtg taatgaggca gaaactctat
      301 caggtactta ttgcgccaaa aaactttcaa gccctttttta agtatacgcg ctaaagttaa
      361 aatccgaccg ggacagccgc aataaagtag ataagcctaa acgccatcat gtctttagga
      421 gagtttgcta atgaaagttc ttaagttgac gtcattgact gttccgattc gttatatgac
      481 ataagagcat catatatttc atgcaatgta tttgagatgt ttttttccat atgttgatta
      541 atatgcccat atattgtcgt ctcttccgaa cactctgtat ctttatgtat atgattttgt
      601 tgctttaatt cttctctatc tacactctct atttgctctg acactagcat aactacccca
      661 tattttacat aacgaaataa aaaaattcta actgtagtat gcttatataa tctacgaagt
      721 caatagttaa attgaaaagt ttcggttata atttacttca tatagaacat gcccttgaat
      781 tatgctaaaa atcgccatag taggcctacc aaatgctgga aaatcgaccc tattcaacag
      841 attagtagga agaaaagcag cagtagtgag taacattcca ggagtgacaa gggataggcg
      901 agagggaatg ggagagaatta gcgatttgga gtttaaagtt atagatacag gaggatggaa
      961 cgaccaaact aatttttcac tacaagttat tgaacaaatc gaattttctt tatccaattc
     1021 aaacataatt ttctttcttg ttgatgcaaa agtgcaaaat gagcgaaatg aagagtttgc
     1081 aaagtggctg aagagaaaaa taaataaacc tgtaatacta gtagcaaata aatgcgagag
     1141 tcataaatcc gaaaatgttg attatttgca gtttttttgat tttcttggcc cggtgtatat
     1201 ctctgccgag cataatcttg gcatggttga tctttatgat gcattagctg gtgttattga
     1261 aaattttaac gagaacactg agttacctaa caatgaactg agtaggctga ggatcgcgat
     1321 catcggccgt ccgaatgttg gaaaatcaac tttttttaaat ggtttacttg cagaaaacag
     1381 actaataaca agttcagagc caggtaccac acgtgattct gtggatatta catatgatca
     1441 tgatggagag ttaattactc taattgacac tgccggaatc cgcagaaagg cgaatgttgt
     1501 agatggctta gaatcaagat ttgttgagaa aagcatggaa tcaatcaagc gctctcatgt
     1561 ggtagtttta atgctagact ccctagttgg tattgagcag caggatttat caattggtga
     1621 agctgcaatt aagggaggga aagggattat tgttgtttta aataagtggg atttaatagg
     1681 taaggatgac agaagcaggt taataaaatt tgtcaaacaa caggaagtaa ccaggttatt
     1741 cttggaagtg ccaactataa caatttctgc gttaaaaggc atgcgctgcg gtgatgtgat
     1801 agataagtgt cttgaagtga gtgaatcctt gaacaagaag atcagcactg caaaactgaa
     1861 taagtggctc atagatgctg taggaaaaca ttctcaccct cttgtaaaag gcaaagcagt
     1921 taaaatgaag tatatcgctc aaattggtac taaacctcca gctttttctt tgatatgtaa
     1981 catacctgaa agtgttgatg aaagttacaa acgctattta attaacgatc tcagaaaaaa
     2041 tttctttgca gacggtgtgc cagttagatt gcttttgaaa aaaaataaaa atccctatgt
     2101 aaaatgaata ctctgcttta tactttcagg gataatttac ttaaagttat gtgtaataat
     2161 tccagaattc ctgcaatctt tctgctgtcg agtgccgctg ctctaatttt tgcatatgtg
     2221 ctagaatatt ttttcaacat gctgccatgc aagttatgta catacgagcg aatagtttac
     2281 tatatcgccg gattacttgc agtagcgtgc atgcttaaag acaacaaaat tctaatctat
     2341 gcaatgtttt gcagctacct cataggtgca gtaatatctt tttatcatat aggtcttgaa
     2401 ctccacttat ttcatgatgt tttaggttgt acagagcaag caagtggtaa cgttagtata
     2461 gaagagctca gaaataatct actaaatcct aattactctc catcttgtga cagacctcat
     2521 tacgttctag gtgtttcctt agcaacatgg aatctaattt atctcatagt agctctgttc
     2581 gtatcaggta aggtgtattg tggagaaaga aagaaatcta aataatttaa ggtacagcaa
```

2641 caatcagttc ttgcaacagg caattagagt agatcacgct ggagaatatg gagctatttg
2701 catttattct ggtcaaaaat ttattcttaa gaaatcttct ataatcaatg aaataattga
2761 aatggaagag caggaaaaaa agcattttca ctattttaat gagaaaatca aagagcaaaa
2821 agttcgtcct actgttttgt tgcctgtttg gcgtgtttta ggagtgtcgc ttggcgttgc
2881 aactgccatt atgggcaaag aagctgctat ggcttgcact gctgcagttg aagaagttat
2941 cggggagcac tacaaagagc aagtttcaca tttagaagat ggggaattaa gagaaactat
3001 aagtaaattt cgcgacgagg agttagagca caaagatatt gcaattcagc acaatgctga
3061 aagcgcattt ggctacaaca ttttatcatc attcataaaa acaggctgca aaactgctat
3121 ttacttgtct aagttaattt aactaagcag ctttcttaaa gtgttttaaa tgttctgata
3181 acaacagaaa ccaggaagga attcaagtgt aacaattatt ttataactat atttacatat
3241 caccattatt aataaataat tagtatttgt aaattaaata catctaaagt atagactgca
3301 aaggaggcaa aagtgcgttt taatttagaa gagggaaatg ctcttatgta atacctgcca
3361 caggttcaca actgtacgaa cattcatttt tgaaggtaaa ttgcacagca aatggtgtca
3421 ttccagtctg gaatccagaa attttgctta taactgagct gataagctaa aggtaattgt
3481 cttacactaa aacaaacgtt tttaattaag ttgcatagaa ccagtgtctg ggcactggga
3541 tgacacccca ctggtggtcc aaactacaac gttcgtacag ttgtggattc cagacggctt
3601 tgttgcatca cactttatga gatggtgatt tatggtaaat tcatgtaact atctcaaatt
3661 tagccatacc aatatcagtg aatttattaa gcaaatagca tttgattagc agttcttttt
3721 ctcgatttat ctcagattta ttcctaaaac taaatccgaa tatttgcttt aatcttgaga
3781 aaaacccttc aatataagat cttttcccat aatttacttc tttttccat tctttcatgc
3841 catcttcacc gtataatttt attaacctaa tagcagcatt cctgtcagac atataatcta
3901 tttctggatg ttctgccgca ttgtttattg gtggaatttt tgtctttata tcatattcgt
3961 tacacaactt ataaaacttg tgcctatcac atgctctatc tgcatatagt gcttttatgg
4021 catgctgaaa atcaacttct tttagcaaat cacaagctcc atagtgatca gagtagacac
4081 cgttactgta ttttacagct atggcttttt tgttgtttat attcaacatt acgtgcaatt
4141 ttctcgtttg ctcatagcca cgatattttc tgtcagtgct attttccttg ctgtgaccag
4201 gagtgttgtt gtatatacta attcctgtac tgtctatagc aatttcgata tcttccatat
4261 tattttctgc aatcatttat cttaatatta agtttcttaa accttcttga tgcttgtgaa
4321 tagctgataa ctgccaaatc tcttcctatt tgttgcaaat accctttttat aaacccgact
4381 gtttgtctta aaccaattct aaagaaactg acgattatat gcaccaaaat cacaacttta
4441 tcactataaa tatagttgcc gccctgcatt tttggacaat tctcgtacca attttctatg
4501 gcgtcattga tataacaaaa aatgcttcct ctttcttgga gaaatttgtt atattcatgg
4561 cagttactga ctctcatttt ttgtggcata ttttttcttc aacagttaaa tggctattta
4621 taatgaattt tgtcagtaac tgccagttct tttcacttga gcgatgcaac aaagccattc
4681 cagactggga tgacacccta ctgatgggcc caaatcacaa tgttcgtaca gttgtgggtc
4741 aggctatagg atgacagaaa ttgactttat tagctatagg tggtatgaca ctcagtcaat
4801 ttaagtaagc agatgatctt agctacaaaa gtattttttta cctcatttgt ttttgggttt
4861 attctttttc cctattttat aaagcttcta aaaaaaataa gcaaagatgg gcaaccaatt
4921 agatcatgtg gaccagaaag tcatttaata acaaaaaaga atgtaccacc tatgggcgga
4981 ataataatac tgacttcttc tttattacca atttactct gggttcagtt aacaccagaa
5041 attttgctgc tgatattgat aactctattt tttgctctac ttggatttat tgatgattat
5101 ttaaaattga agacaaatca ctatcgaggt ttaagtgcaa aaaccaaaat acttattcag
5161 tttattgttg ctctggttgg tgtgtttata cttaagctat actctgctga atgttttaca
5221 aaaacgtccc tatttaaagg agtaataata gattttggct atctatatgt tccgtttgct
5281 gcgtttgtaa ttgtcggctc ttctaatgct gtgaatctca cagatggttt ggatggcctt
5341 gctgcaactc aagtcatcac ttcctttgct tttttggggc taattgcata tataactcaa
5401 gcagatatga atattacttt attctgcatt gcatttatag gagctattct aagtttcttg
5461 tggtttaaca cacatccagc aaaaatattt atgggtgatg ttggtagctt gtcggtaggc
5521 gcagccttag ggttaactag tgtcctaatt aaaagagaaa tgctttttgc cgttattgga
5581 ataattttg taatagagac tctatctgtt attattcaga tatcatattt taaatataca
5641 aagtttaaat atggagaagg aaaaagagtt tttccttatg gcaccaatgc atcatcactt
5701 tgaaaagaag ggatggtcag aaaatgtaat cgttatgaaa ttttggataa tttctattat
5761 ctgttcagtt tttactataa ctttcttatt ataaaaacct atgaaatacc cagactttac
5821 attagaaaat aaattatcag gagtgatagc aggagtggat gaagttggaa gaggtccgct
5881 agctggtcca gtaatatccg cagccgtagt gtttattgat agaaatacaa ttattgatgg
5941 aatcaacgac tcgaaaaagt tgactcctca atgtaggcaa gtcctatatg aaaaaataat
6001 atccgttgca aaatttggta taggaatggc aagcgtagaa gaaataaatt catacaatat
6061 cttgcaagca acaaaacttt caatgaaacg tgcgttgata gacttggacc tagaattaga
6121 ttatgtgcta gttgatggta atcaaccacc tgaagtgaaa tggcaagtga aatccatagt
6181 aaatggtgat aatttgagta catcaattgc agcagcttca atcgttgcaa aagttacgag

6241 agatcggctt atgcaagaat tgcacaataa gcatcctgaa tataattggt ataaaaataa
6301 aggatatggg acaaaagagc accttaacgc tattggcctt tatggaatta cagaacatca
6361 tagaaaaaat tttgcaccca tatctcgggc attatagcct ttaccctttt ttgaaaggta
6421 tggtttcatt aaataggcaa actgtactcg atccattcct taaggtaaac actgaactta
6481 ccctttcgat tatcaggtag ttatcaactg tacggaaatt tactaaagac tcatatcctg
6541 cagaatcaac cataaaaact gccggaaaat cagagtttat tttattgaat tgtagatatg
6601 taaattttcc gtcatcaaaa accttaattg gtattattga ctgactacct ttgacgtgcg
6661 agatggaata attaaaattt aatcccttct ttacaacttc aatatcattg atatcaggaa
6721 taatggtatg actagcttgt tcgaagatat caccgttatt tgttgtgtaa atctcatcac
6781 tgctaaatag tggataaaga aaccttactt catatgctaa tcttggatca tctagtccag
6841 ttgcttcttc agcatgaagt tcaaagtaat aaactctttt attggtaatt atagttgcat
6901 tagtatcagc aatatcatct atgggcttaa taaacaatct gttaccttga ggaagaagtt
6961 gccaaccagt tgaatcaccc attgaaaggt tttgtattat ctcccctggt tcaaacaata
7021 tactggactg ataaccataa aaacccgtat acttatgtat agcctgtggg ttataattta
7081 ttacctttat gtgattatct ccagctattg aacgtggctc ctgttttgca agtgcatcac
7141 taccacagag gaacaatagt acggcaaaca gtaaaccaat cataatatca aaatgaatta
7201 atcttcattt tatcattaat agacatgcac tgcaactgtt aaggcacttt ctatactata
7261 aaccccaaag caagcggtag aaaaggcaag aaataaggta aactccttaa atactttaga
7321 actacaggag gcttcacctg aacgggtaca cttggaatcc tgccattttc acttcgtaaa
7381 cataccaaga attctgtcca ttattccacc catgtttgct tcagcttgag tatactctat
7441 tttaccacca tgcaagatta gtttaatatc tgatccaggt gatatattaa caaatttact
7501 gccaacaact ccactgctag tgatcagagc tgaactatca attggcaatt ttatgtcttt
7561 gcttatacac atatcgattc gtgctacgta ggtagctttg tcaagtgata caccagttat
7621 gcttccaaca tctacaccag agatcttgac actatcacca actcctatac cgttagcgtt
7681 agcaaaaaga ccatatattt tatagcaatc cttatagttt ttctttatgt aagatagctt
7741 atcaatagca aagaaaatca gaaaaatagt aaaaattaat acaaataatc cagcggttat
7801 ttcaagtata tttgaccttc gcatctttag ttatcaggac tccagctttt atagtaattt
7861 ttcacttttt ggtttggata gtatgcatct tttgtacccg ttaaattagg agtatgtttt
7921 acttttcttt ttttattatt aactggtact acattatcag tatagtgaag ccatatatgc
7981 cattccggag gcactgttgt aggctcagaa acactgctat acataaccca ccttttttcct
8041 ttacttgatt catagtaaga atttccattt tcatctcttc ctacaaattt atcttctctt
8101 cgtaataggc gttttattgc attacaaatt ttagataaca taagggtatt aaaacaaaaa
8161 gtattatata tactaatact ttaacgatat attaacaaca atttaatgca attaccaatc
8221 ttacaagtta ttatcccaat aattgcatca atgttttgct ttcttgccaa gaaacacaag
8281 gtatcttggt ttatttcttt tattgcaaca acaatcactc ttatcatttc atcaatacta
8341 ctcattaaaa catataaagg tgaaattata acttatcacc ttggaaattg ggctcctccg
8401 tatggaatag agttaagaat cgacgtgtta aattccttaa ttctaaccct agtgaatttt
8461 atagcgctga ttagtgtgct ttatagcttt tatattaacg aaaaagagat tagcaaaaac
8521 aaaatcactg gtttttactc tctatttcta ctgtgcttaa gcggactgct cgggattcta
8581 gtaacaaacg acatatttaa cctttacgtt ttcttggaaa tttcatctct ttcttcttat
8641 gtattagttt caatgggaag ggataaaaaa gccttggttg cagcatttga atgtctaatt
8701 agtggaacaa taggtgcaac attttattta tttggcatcg ggctcttgta ttcaatgact
8761 ggaacgctca atatgtctga catggctgaa agaatagttc cattatacga taacaacatc
8821 ataaaacttg gcacgttgtt catttttgtt ggtctctcaa tcaaaatggc actgtttcca
8881 ttaagcaggt ggctggttaa tgcatatagt gaggcgccaa gtttcattag catattcttt
8941 tcaggcacag tgacaaaagt gatgatatat gttttcatca gaatctttta tactgttttt
9001 catcaaaatt ttttcttgtt taagccgcta ctggacaatg tgataattat cctcgcactt
9061 tgcgctattg tatttgggtc gatatttgca ataaccgcaa aagacataaa aagactgctt
9121 gctcactcta gtattagcca aattggttat ataattttag cactgagttt taactcaaaa
9181 acaggtgtat ttgcagcaat tcttcacata gtaaaccaca gcataataaa aacatcttta
9241 tttatggctg caggatgtat ttcttacaaa tttgatacaa caaaaataga gaatttatcg
9301 ggactcaaga aatcaatgcc ttatacagca cttgcgttca ctctattcag tttagcatta
9361 atcggtatgc ctttaacaaa tggctttata agcaagtggt atataatgaa agcaattatc
9421 gaatctcatg cgtggatttc ccttgtaaca tttgctgctg gctctttcct tgcattgata
9481 tatatgtgga agatggtaga gaaaatgtat tttgaaaatg atgcaacgtc acgcactgga
9541 atgacaccgg acaaaattaa tgatgtgcca tttcctatgc tttttgtct attgtttatg
9601 gcagctttaa cagtagtaac tggaatatat agtacttcaa ttcggttagt agttgagaag
9661 ttggtttttt gatttttatc tttgtttcat attttactat actttaaggt tataaggtct
9721 ttattgtgga taggctgaag aagattcatt ggttgttagt cattaacgtg atagcgctgt
9781 tttgcgttgg cattgttgtt caatactctt ctgctggagg aaagtgggtg ccatttgcga

9841 ttcaccaact ggtcatattc tccttctttt tcctattcgc tatagctatg tcattcatag
9901 agctagattt ttacttgaag tacgcctact tttttatat agcagcagcg atttcgctat
9961 tagcagtaaa ttttttggc tcacacataa tgggttcaac aaggtggata agaattggat
10021 caattagttt gcagccatca gaatttgcaa aagtaggctt aatacttgcg cttgctcgct
10081 atttgataa gcagagtgta tataaaatga tggaatttaa aatattgctt aaggcactta
10141 taattatttt cttacccgtg ttcttggtgt taaagcaacc taatttaggc acagctgtga
10201 taatgttatt tatagggata tcaattatat tcacagcaat aataaaaaga tctcattcgg
10261 taatatgtgg aacgcttggc atttttgcag taccggctat ttggcctttt ttacgtcctt
10321 atcacaagca aaggatattg tcgtttttgg attcatcagt ggatccactt gggataggtt
10381 ataatgcgca gcaatctcag atagctattg gttcaggagg tttgcttggt aagggctttg
10441 ttaacggaag tcaaactcaa cttggatttt tgccggaaaa gcgtacagat tttgcttttg
10501 cagtgctgag tgaggagtgg ggctttttag gtagcatggc tttaattttg ctttatacct
10561 cattacttgg cataattttc tctatcgctt ataggtcgaa aaaattattt ttctaagtcg
10621 ttatctatcg gcatttcgc ctttttagc gctcatttt ttataaacat cggaatgaca
10681 atgggcctct tacctgtgat aggcgaccca ttgccatttc tttcctatgg aggaagtaca
10741 actgctgcaa gcttgatatg cataggatta ttgctggcaa taaaagcaga tgaacaacaa
10801 aatgcttgta ttttacctat gctaaagtaa aattaaactt tacttaatag tttttatgct
10861 agatgaaatg cgtgaggagt gtggggtatt tggcataagt tgcaaccaaa gtgctgcttt
10921 taactctata cttgctctcc acgctttgca gcatagaggt caagagtctt ttggcgtagc
10981 aaccagtaac aacgataagc tgcactctta tcactttcaa ggtcaagtga gcagcgtatt
11041 tgatgatata gatgaaataa agaaatcctt acctggagat tgtgcaatag gtcatgttcg
11101 atactcaaca agtggtagta aatttggagt gcagcccatg tttggcaaaa gtggcaaatt
11161 tggggatttt gccatagcac ataatggtaa tttaattaat atttctccga tacgcaaaca
11221 attaattaaa caagagtgcg tttttcagtc agatattgat acagaagtag tagtgcatct
11281 gacagcaagc ggtgaaaaag atagcttctt agagagtttt atatatgcat taaagcaaat
11341 acaaggtgct tattcttttg tggcaatcaa tcaagaagta gttattggag tacgtgatcc
11401 gtctggaatc aggcctcttg ttttaggaaa gttaaacggt tcttacgttc ttgcatctga
11461 aacttgtgct cttgatatag taaatgcaga gtttgttaga gaaatagaac caggtgagtt
11521 agtcaccatt gatcgaaatg gtaatctagc ttcagcattt cctttcccac agcaaaaatc
11581 aagtttttgt atttttgaat acgtttattt ttcaagacca gatagcataa tggagaatag
11641 gtccatatat gatataagaa aagaaatagg aaaaatactt gcagaagaga gcccaccaaa
11701 aaacaatgta gatatggttg taccaatacc tgattctgga atacctgcag ctattggata
11761 tgcaaagcat tcaggattac ctatggaact gggaataatt cgtaatcatt acataggaag
11821 aacttttata caaccaaccg ctgaagtacg taaagttaga ataaaattaa aattcaatgc
11881 taataagcac actttgaaag gtaaaaacat aattttaata gatgatagta tagtgcgtgg
11941 tagtacgtta acaaatataa tagttatgct aaaggatgca ggagtaaaag agattcacct
12001 caaaatttca agcccaccaa ttaagcattc ttgtttttat ggaatagata caccagagtg
12061 caaagattta attgctgcaa ataaatctgt agaggaaatt aaggaagtta taggagtaga
12121 tagcctagcc ttcttgagta ttgatggact atatcaggct gttaaaggag aggtgcgtaa
12181 caatgctaca ccacaatatt gtgatgcttg tttcactggt gattatccga ttggcaaata
12241 atctacgtaa gatcactgga gctcagatct taaaccgcca aaatttctct tggcctttac
12301 taattccgat tctcggggtg caaatataat ctggtttgag gtgagattca taaacacaaa
12361 atttgtggct tatagtaagg tcctgtttgt tatgttcttt tgtaatattt aatcttttac
12421 acaatattcc tggcccacct aaatttgctt caagcggttc aatgagcttt aatcctcgta
12481 ttaacactgc tgctgggaat ccttctgctt ctgttacaat atttagacaa tagtacatcc
12541 catatataaa atatacgtac gaaaatcccg gcataccaaa cattactgaa gttcgattag
12601 tatagcctct tgccgcgtga caagctgggt cattcattcc tatataggct tcaacttcgg
12661 ttattactcc actaaagtta gaaaatttaa ggacttttcc caataactct ccagctacag
12721 ttaaggtcgg ccgctcgtag aaatttcttg gtagtattat gttgctcatt ataagagtat
12781 aagttacttg caggtgcaag caacttatac taattattac ttttgttttt tcttacttac
12841 tcatttgagc agcaggagct tgatgctttt gtgttgtgct ctctgttaca tctgttgaaa
12901 gttgctccaa gattttactt cttcagaact ttgttgtaat tgtgtttgtg gcaattgtga
12961 tgctaatgct tcatgtaaag gctttgcttc atattgcctt cccactttta cttcttggtt
13021 tgcacgtaac tgttcttcag ttacgccatt accctcgatg ggctttatag taccatcatc
13081 actaatgagg attttaattt tgcattcttg tccatcaaca taccacttta gagccatttg
13141 atatgatcca ttagccactt cataaattct tgccccatcc ttatcaattt ggcaacgtac
13201 ccccccttgtt ttttggtcag gtagtagcaa cgtaactgtc tttacatcgt actttttgca
13261 catattacca cttagaatat catgcaagtt tacttctttt atataatcgt tcagcgcaat
13321 tcttatgaca ggcttaacac tgttatctat atttttagtt tctcttaatt ttataagctt
13381 tgcatcaata tttttagata tctcttcttc aatttgttta gcagctttgc ctacaatttc

13441 ccgaccatga tatttggtct cctcagcagt atatctatac ataccatcgc tattcttatg
13501 aacatgaggt ttcactatta ctttattagt agtattcgta gaagggttgt tctgatctac
13561 aaagaacctc ttccatgttt cttcatcatc aagagtgacg tacctacatt tatccttatt
13621 cggtattgac ccagatacgc taccagcagc aatattttga tcaacaatca aagatacagt
13681 ttcattacta tctaacatat atgcctccat tatccttaat acacaaatgt caacatacga
13741 gcattatctc aattagtcaa tataaattat gacgtttaat gttgaaagct caaatactcc
13801 tttgcttgat cggttagaac gcgccctctt ggtgtgcgct ttacaaaact aattttgatt
13861 aaataaggtt ctactgtttc ttcaatattg ccaacatctt cggatagcgc aatagatatg
13921 gtgtcaattc caacagggcc tgaagtgttg aataaaaatc ttagataatt catatcaagt
13981 ttattgagtc ccattttatc tacacctaat tttagtaata cagaatcagc gacttcataa
14041 gtgatttttt tatcatcttt cacttcaaca aaatccctta ttcttctgag taacctcaaa
14101 gcaattcttg gagtaccgcg cgcacggcag gcaatttccc gtgcagcacc ctcttcaatt
14161 tcagcagaaa gaactcttgc gcctcttttt ataatatcga ctagctcctc aaaagaataa
14221 aactcaagat gcaaaggaat gccaaaacga tctctgagtg gcgcagaaag taatccaagc
14281 cgtgtagttg ctccaattag cgtaaatggt ggtagatcta tccttaaagt gcgagtagat
14341 ggaccttcac ctactaatat gtccaagcaa aaatcttcca tagcagtata taaaacttct
14401 tcgatgctgc gatttaatct atggatttcg tcgataaata ggacatcttt tgcatttaaa
14461 gtggtaagca ctgcggccaa atctccagct ttactgagca atggaccaga agttgcgcgg
14521 aagctgaccc ttaattcttt agagacaatt tgtgctaaag ttgttttgcc aagtcctgga
14581 ggaccgtata ataagacgtg atctaaggct tcagttctcg tctgtgcagc gtttataaac
14641 acttttaaat tttgtattaa gtctttttgc ccgacaaaat catcaagttg ctcaggcctg
14701 atgttgatat tgcgcacatc ttcagtatat tctttaccgc acgatattga tttcatagta
14761 ttgaaagctc cttaagcgct atgcgaataa tatccttagt gtccagattt gacgatttgt
14821 cttgtatttt ctttatcgta tcataagctt tcactttttc atatccaaga ttgatcaatg
14881 ctgaaagggc atcttcatta attggattaa agttattgtt atttatttct agtttactca
14941 ctttgccact caattcagtg atgattcgat ttatgagttt caggccaagt ccactcatct
15001 tgagtgctac tttatcctca ttcataattg ccaaaaatag ctgttctgga gttaatttac
15061 tcaaaattga cattgcagtt ttatagctaa cgccgcttac tttgacaagc aacctcaagc
15121 actgctgttc ttcttcgctt ataaaaccat atagctgagt agtgttttct ctgttatttg
15181 cataggtttc gataagtaat ttgattctac ttccaattgc acaagcactt aaagttttgg
15241 ctgaaagata caccatatag ccaacatcat tcacattcag gattatgtga tcactgcgca
15301 cttcatcaac tattccgctt agatttccta tcatttttaa cactatatag agttcagttt
15361 aaatactccc ggatcttagg tcaagcaaac gaacattata acagggaaac gtggctaaaa
15421 aaccacttga caaactccgc cagcccccttt atcatgaaac tgaagctatt tatttatctt
15481 ctctgtacag attaaatgac aaaaaaactc acgtatctgg cgtatcatgt ttaatttttt
15541 gcactatgtg caccttatgt cttataaaat ttctacctac ataagctgaa acgcgcttat
15601 aaagcgttta aaacataaaa aaacgccaac ttaaaacatg gatagtgaat aactagctac
15661 cctagtgttt cttttgcctt tttttctgtt tagtaaattt cttaacgttt ataatttaga
15721 ttagttgcgg tttaaaagta gctgaatcgc ggttattaag tgtttagaat aaaaaaacgc
15781 catacttgaa agtataatgt aagtaattag ccaatcatgg ggcttttttt gccttttttt
15841 tcgtttggta aatttttttaa tgtttgtggc taaacgacaa ccgtcatccc gctgcttgtt
15901 agcgggatct atgctaagag ataccgcgga tgaatcgcgc ggtatgacgg ttcgtggtgg
15961 catctcgtca tcccgctacg tgttagcggc taagagatac cgcggcggta tgacgtctta
16021 gccatagatt acctgatctc accatctttc cggttgtttt atacataatt ctcggatttt
16081 gaaagttcac tttgaataaa ttgctcatat tctttatcct ctagttcttc aaaaatctca
16141 ggataatgtt tactggaacg agtaattatt gaaattagct cttgctttgt ttttcttagt
16201 aaaacctttc aaagagagtc tgttcatctg ggcgcgcaga cgatcgatac tcctttctat
16261 caagtgcaac caaagcaaaa cctgttgcag ccatatgctt acatggacca taatacacaa
16321 atgcaggaca ggaacatgtt ccactcaaaa gaggaccatc gtgctttagt gttactcgat
16381 atacatttga tccaactacc tttgatttag cttgagattc attaacagat ataatctgta
16441 cctttctttt gtcaaaataa tcccttcctc tggagcggta gggttctttg ataagacctg
16501 tgatatcttt ataatacagt ctcattttta cctcctttaa aaccctcaat tttctaatat
16561 atcctttctt ccataaaaat ttattaactc accacaactt ttatatggcc ctgcggacaa
16621 ttttatagtg ctatagctaa ctcaagaaag gtttccctac gtcataccgc gattcattcc
16681 ataactgtat gaacattcat ttttgaaggt aaattgcaca gcaaatggtg tcatgaaagt
16741 agctgacact ggaatccagc cttgccgtaa tctcatcaag aacgttgtgt tttaacataa
16801 aacggctact tttatactta ccaacttaat aaaattcctg gatcccagtg ggctttgttg
16861 catcgctact tatgaaaggc taactataat taggattata agcaacctat tgaaaatctt
16921 gttttttttgc aatcaatctg atcaaattta agaatagtaa tttattattt atattaataa
16981 atttaattct attgaaaata gctaaagcat tgaaattctt gaattttagc cggattagtg

17041 agtggtagtg aaatttcttt tactcaaatt taggtattca ctgaccgttc ttgttataaa
17101 taccagaata ataagctact gatattctcc accttttttta tctttaatcc attatagcta
17161 gcgatgcaac aaagccatcc cagtgcttga cactgggatg acaagaagag ggtacttgga
17221 taagaggcat tgccctcctg gtaggcccaa attgcaatgt ccgtacagtt atggattcat
17281 tcgcggtata gattccgcta atgagtagcg gaatgacgaa tttgttgttt ttcaaattgt
17341 aggtaaacct aagccacttt agctatatat aatagaattt cgctttctac tatctttcca
17401 gttgttttat acataattct cgaattttga aaatttgctc catatctaat ttctcacttg
17461 tataaaattt gttttcttac ttcgagacct aatctaccac aacttttata tggcgtttct
17521 ttcctgcaga taattttata aatggctgat ctttaaagct ttcgaaattt attacatgat
17581 caatattgtt tacaacatta tcattaatct tgcatccgtt gccctgtatt aaacgctttg
17641 cagcgccttt tgaaggttca aaaccggtgt catgtagtag gtctactaag gatatgccgc
17701 ttgcaacttg ttcttttttt atgatgtaat caggaagtag tgagctatct tcattctcaa
17761 aagcagaaat tgcagcaaat tgtgcaagtt ccgcttcttt gtcaccatga catatttttg
17821 tcacttctgt tgccaagacc tttttgcttt catttatttc ttgatccttt aaagactcta
17881 attttctaat ctcatcaatt ggcacatcag tgagcaatct taaaaaacgt ccaacatctt
17941 gatcatcaac gttgcgaaaa tattgccagt agtcgtaagg ctttagcata ttatcatcaa
18001 gccacactgc tccactttca gttttgccca tttttttttcc ttgtgcattt aataaaagag
18061 gcatggtaag gccaaatagc tcaggtaaat tcaacttttt tccaagttca attccgttta
18121 ctatatttcc ccactggtct gaccctccaa tctgcagacg acaatcatat tttttgttta
18181 attcaataaa gtcgtaagct tgcaatagca tgtaattaaa ttccagaaag cttaaggttt
18241 gctctctatc cagcctggtt ttcacactat caaaacttag catacgattt acagaaaaat
18301 aagctcctat atcacgcaaa aaatctatgt attttatgtt atccaaccaa tctgcgttat
18361 ttactatcat tgcaccagtc ttgctgtcat taaaagacac cattttctct aatattctcc
18421 ttatactgga tgtattttga ttgatgttct ctacaggcaa gatgcttctt gctttatctt
18481 tgaaggatgg gtcaccaatt tttgttgtgc cacctccaag taagactatc ggcttataac
18541 caaatttttg taggtgacag agcatcataa tctgaattag gtgaccagca tgaagacttg
18601 gtgctgtgca atcaaaccca atgtatgcaa ctatataatt gctctgcaat aataattgat
18661 ctagtccttc aatgtttgtg cattggtata ggtaccctct ttcttggatg aaatttaaaa
18721 attcagatgc gtgtttcatt ttataaaccc cagtctatcc tgtcattaat gctgaaggta
18781 accctaatag agaagccagt gcccagacac tggaacttaa ttataaacaa gcacactata
18841 caacgttttc gatgaaattg catgaaaaac tggattccag tgtcagctac tttcatgaca
18901 ccactacctg tgcaggagtt gccttcaaaa ttacaacgtt tgtgcaattg tgtaccagct
18961 atctggataa tcaattccat aactcttcta ttttataata ctcccttact tccggcctga
19021 atatgtgaac cattatgcct tgaaaattca aaaccaccca attgccttca tccatacctt
19081 ctacatctat tttatcatgt ggcttgaggc tttttattac gtgctcagct aaagctttaa
19141 cgtgacggct tgaatcacca gatgcaataa tcatatattt tgcaataacg gttttattct
19201 gcacatcaaa agttactata tcatgacctt tattttgatc tattatctct tcaattgtac
19261 ttcttatcga tttcatatcg ctaagcattg tttacaggtt atatgattca tttataatta
19321 tatactcaaa atttctaaat atgaactatt ttcaaccgtt aaaaaggtgt tacctgcatc
19381 tattttatca tcatgttttt agcatgtaca atgtatatac ttttatgtat aattgagaat
19441 agctttttac tagtattcat tatcggcaag actctcatct acttaatcgg ctttccaggt
19501 agtggtaagt ttactactgc aaaggaactg tgtaaaatca ttgatgcggt gatagtaagc
19561 aataatctat tcaataatat tatatttgat attgttaaat tacaaaacgc tgaagttcca
19621 gatgacctat gggaaaaaat ttttgcagtt agggaaaatg tattagcaat actggaaaaa
19681 cactacgtaa aatcaaaaca ttatatattt acaaatgaat tgatagaggg tgatccctat
19741 gaccaaagat tatataactc agtggtaaat ttaagtaaaa aaatggacat ggaaattttt
19801 tctgtagtat tacattgtaa taatgaaaaa ctagtaagac gtgtccaatc agaagagaga
19861 tatcaggaaa ataaaattac tgatccggat tttgctataa agaaaattga agggaaaagg
19921 ctatttatac ctgagggttc gcttgaaatt gataactcga acttgagtgc aaaggaagtt
19981 gcaaagaaaa ttgttgaaga gatgaagaaa gaaaaaaatg gaaaattaat taaaacttct
20041 gtaacaaatc atttaaaaac agagcgtact cgtggttgag cagaaagata agtaccgtcc
20101 ttgtgttggc ataatgctat ttaacagaca aggacatgcc tttattggaa aacgctttga
20161 tagcgattct tactggcaga tgccacaagg aggtgttgat gatggtgagg aactggaaca
20221 agcagcgtta cgtgagttgc tagaggaggt tggtactaat aaagtaaagg ttataactaa
20281 aagcaaggat tggatatact acaacttgcc tgaagaagtt ataccaatat gctggaatgg
20341 aaaatattct ggtcaaaagc aaaggtggtt cttaatgaag ttttgcggag aggatgagga
20401 cattgatatt aattatactg ttcatccaga gttcaaagag tggcgttggc aaggtataga
20461 tagtttagta gccagtgcta tatcatttaa aaaagaagtt tataaaacag tgatagaaga
20521 gttttcttct ataataaaag cctcaacaat aagctcatga tagtagattc tcattgccac
20581 ttgatttatt tttctgacga tgaaatacca aaagtaattt caagggcaga gcaaaatggt

20641 gtaaaggttt tgcataacat atgcgtaagc attgacgatg ttcctaagtt attaaaaatt
20701 tcttcatcct atgatcaggt ctactcctct gttggtatac atccgcttga tgctgctata
20761 gaaaatggtg agtgtataca tgttgatgaa ttagttgaat ttaccaagaa ccaaaaggta
20821 attagtatcg gtgaaaccgg gttagatttt tataaatctg acaacaaaga caatcagaaa
20881 aaaagttttg cattacatat agaagcgtca agaataactg gactaccttt agttattcac
20941 acaagaagcg ctgataatga gatgatcgat atgttaaggt cagaaatgaa agccggtgct
21001 tttagtggag taatgcactg ctttgcttcc tctaaagaac ttgcttatca atctatagat
21061 ttggggttat gtatttcatt ttctggaatt attactttta aaaatgcgag cttactcaga
21121 gaaattgcac aaaatgtacc acgcgagcgt gttttagtcg aaactgatgc accatatcta
21181 tcacctgagc cttatagggg aaaaaagaat gaaccagcaa tggtgaaata cgttgttgat
21241 tgtttggcag agttgtggaa tgagtcacca gaacaagtag cgaaggtaac cacaagcaac
21301 tttttaggc tgtttgagct ataaagaagt tctataaaac cattaatttg cttatgaacc
21361 taaagatttc accagctctc tcaccgagtt tacagactta ttaaacattt cttgttcact
21421 atcattcatt ttaacttcta ggatcttttc aattccgttt tttccaataa ttacaggaac
21481 accaataaac aactcttcta caccatattc accattaagg taagcagcac atggtaatat
21541 gcgcctctta tcttttaaat atgattctaa catgcatata gctgaagatg caggagcgta
21601 gtatgcagat ccagacttaa gcagatcaac tatttcttta ccgccattgc gagtgcgttc
21661 aactatttca tcgacttttt tctgtgtgat aagacccata tcgataattt gagtaagtgg
21721 aactccagca actgaagcac aattgattag tggtaccata gtgtcgccat gtcctccaag
21781 cacaaaagca gatatatctt caactgagat atttaactca cttgcaagaa agtaacgaaa
21841 acgggaagaa tcaagcactc ctgccatgcc aacaatcata ttagttggaa ggtttgaaaa
21901 tttatgcacc actgaaacca tggcatccaa agggttggta accactatta caaacgcgtt
21961 tggagaatat tttttaatat tttcgccaac ctccttcatt actttagcat tggtttgcag
22021 gaggtcatcc ctgctcattc caggttttct agcgatgcca gcggttatta taattgcatc
22081 ggagtttttt atatcttcat acctatttgt gccagtaata ttgacattaa atccatcaat
22141 aggggatgat tctgctatgt cgagtgcttt accttgtggt attccatcac taatatcaag
22201 taaaacaaca tcaccgagtt cccttagtgc aatcatatgg gtgagagttc caccgatatt
22261 tcctgcaccg attaaagata tttttttct ttgtactgtc atttctact cctttgtatt
22321 tacttttatg gttttacctt cccaaggcga taagctatca aacatacgaa aatcttgcgg
22381 taaatctata ggattataca caaccttttt tgtctctata tcatatctta cttcctcata
22441 accagtgaga gggaaatctt ttaacattgg atgacctttg aatccatagt ctgttagaat
22501 cctacgcaaa tcagggtgat cagaaaactc tattccatac atatcaaaca cttcacgctc
22561 aaaccacaaa gccgtactaa atatcttcgc tacacttgga ggcatgtcgc tttcacataa
22621 ctgaagcttt atgtgtactc taatattatg cacaatgctg agcagattgt atattagctc
22681 gaaacgcttc tctctatttg gatagtcaac cccaaaaata tcaactaata gttcaaacct
22741 acacttttca tcatcacgta gaaagaatag gtgcttttca atttcatcta aagttgaata
22801 tattacaata atgccatcat ctttttgaat gcactcgcat ttggtctttt tttgtatata
22861 ctttgcagtt ttatccattc cgagtccgtt ttattttgtt ttgtaggcat agcattccat
22921 atagcaacgc ctcagcggtt ggtgggcatc cagggacata cacgtcaact ggcacaattc
22981 tatcacagcc ttggaccact gaataagagt aatggtaata accaccacca tttgcacaac
23041 ttcccatgga aataacatac ttcgggtctg ccatttgatc ataaaccttg cgcaatgctg
23101 ctgccatttt attagttagt gtgccagcaa caatcatcac atctgattga cgtgggcttg
23161 cacgaaacat tataccatat ctatcaagat cataacggct ggatgcagta tgcatcatct
23221 caactgcgca acatgcaaga ccaaatgtca ttggccacaa tgaaccagac cttgcccaat
23281 tcattacata atctgttaaa tcaccaaatt tagtgacaag aaacccttct tttttataac
23341 gaccccagtc atcattagat agaatttgat ttgtcataaa ctactcccac tctaatgcac
23401 ctttacacca ttcatagata aaacctatag tgagtattgc aagaaatacc atcatagacc
23461 aaaatccacc atagcttatc ttagataaag aaacagccca aggaaaaaga aaagcaattt
23521 ctaagtcaaa tattataaat aatattgaaa ctagataaaa tttaatgtca aaattttttc
23581 ttgcactgga tagtggattg aagccacact catatgtgga aagcttttca ccatctggct
23641 tactcactgc aagaaacata ggcaatatac ctaaagcaaa agatactaca attgatacgc
23701 agataaagat tactatggtt aaatattcgc tcatttaaca aaacatataa cactatgagt
23761 ttacatgctt tcctaacttt ttaccaccta aaatatgcac gtgaaagtgt ggtacaacct
23821 gctctccatt ttcaccgtag ttagtgacta atctatatcc tgttttttct agattatatt
23881 tgtgtgctat ctctcttaca gttttgaaga aatccgctat ttcctcttca ggagctttta
23941 gaatgaaatc atcatatgaa atatattgat tttttggtat aactaaaata tgaactggtg
24001 cgtcagggta cttatcatga aaagctagta cattttcatt ttcatgtacc ttctcacaag
24061 gtaactcacc tcttaatatt tgagcaaaga tgttatcgct atcataaact tcattgctca
24121 tattttaaga gcgacctttc cctctttctt cgtttttctc tacattaggt gtagatagtg
24181 gttttatcgc ttcaccagaa gcatcttcaa gatttaaaga gcttacaagt tcccttactt

24241 ttaccatgtc ctgctgttct ttcaaaaagg gttctattcc acttaccctt tttccagtaa
24301 taactcctgg ttgtaagtga tacatatccc caggaacagt tagactatga tgtctttcct
24361 ctattttttt agcttcactc ataatactcc cctataactt atttttgcat tccacaactt
24421 tgcgacttac ctggctttat agcttcttgc gcccattttt tctcacctga agctatagtt
24481 tctgaaggct gaccaacatc agacttcaca actttgtctg atgactgctt ttgattatca
24541 tattccttat acaatttttc tgcaatacca ttcagcaatt ttaagtctgt atgaatttta
24601 aattctccac cagtaattcc tgccagcttc ttaaggtcgc cattaatgct atcagcaatt
24661 ccttgaagag caagcttttt tatctcctct ggactctttc ctttattttt agagtcagtt
24721 ttaatgtctt caattatttt tttaacatca acttcaatta caattttatt ttcttctcta
24781 aaaatagtta cattcggtaa atcttttttt attttatcta attgctctgg gctcaaataa
24841 ggtatatctc ccgtaagagt ccttgatgct aaaaaactta cctttcctgt ccctttcttt
24901 tgctctgtta tttgtacgcc aagatcctta ctagcttgtg tggtttttat gggtggatca
24961 tattcttcat gtgggttgcc aataatagaa cgcccgccat tttttatgtc gtgccgcatt
25021 tcatcccagg acttaaacca attctttggc catatagggt atattgcacc cttgactagg
25081 acttttacac ctctacccag aaaactccct actcccgaag ctgcattacc aactttagct
25141 agtatagaag tagatttttc attttctctt gttaacatac atacctccaa ggtactttct
25201 ataactaaat acattttaat tatagacgaa aaaatttaaa cttctatatc cattataaga
25261 tagaattata gggattgcaa ccattatttg cacattgtct aaaacatgta gtattctcgc
25321 tacttttatt gttcaattta ttataaagaa tactctaaat tatgtttaaa tggaacacaa
25381 ataatatcat tgatgcaact ggcggaagag acgtaaatgg ttgtaattgg atacatagct
25441 caaatatttc aacggacaca agaagcataa aaaaaggtga tttatttatt gcactcaagg
25501 gaaggaattt tgatggccat aatttttttgc atgaagcgtt tttaaaaggg gctgtcgctg
25561 caatagtaag tgagggtaaa tataaaaatt tccctctcat tattgtacaa gataccctga
25621 aagctttgca cgatatggca tcatactaca ttagaaatgt tcttattgac gctaaagtta
25681 ttgcagttac aggcagtgtc gggaaaaacc actacaaagg atatgctaca cactgtttta
25741 tcgcaatatg gagtgtccca tgcaaacgaa ggtaacttaa ataataatat agggttgcct
25801 ttgacaattc taaaagctcc aaaaaactgc cagtacttaa tccttgaaat gggaatgaat
25861 aaagctggtg aaataaaaga attatcaaag attagtaatc caaatattgc agttattacc
25921 aacatcgaac ctgcacatac tgaaaatttt tcatcactat ttgacgttgc acaagcgaag
25981 ttagaaattc tgtatggtat gaaaaataac ggtactttgg ttttaagcag agataataaa
26041 tattatgatt atctctcatc acacgccaat agaaatgtaa taagcttcgg taaagataaa
26101 aatgctgaag tttgtttact aaacttaata agaaacgatg aatcttctgt catttcagca
26161 ctatcttctg tcatcccagt gcttgactac ttggatccag gaagttcaat tgcaaatgag
26221 catatcagac ggttgtataa taaagactgg attccagcgt cacgcgctgg aatgacatca
26281 aaccgagttg ctaatagatt aagtttaaaa atcaggctga gtgataatca aattataaac
26341 tgtaatttac gtgtacaagg tgagcatttt gcttattcgc tacttgctgt tgcagcagtt
26401 gtgcaaagcc tcggacttga tttatcaaaa ttaccacttg cacttaagaa ttttagtgta
26461 gcgaaaggta gaggcaatat tcataagatc aaatacaata gaaaatatat acatttaatt
26521 gatgactcct ataacgccag ccctgcttcg atgaaaactg caataaaaac tttgagtaca
26581 tattctaatc agagaaaagt agctttgctt ggtgatatgt tggaacttgg tgatgaaagc
26641 atagagtttc atacagattt ggttaagatt atcacagaaa ataatataga taaagtttat
26701 acagttggta aatttatgtt agaattgcat gagcttttgc cagataatat aagaggcacg
26761 cattttaatg attctaatca attgaaaagt gatttagcta acattattca gaataatgac
26821 gcaattctag ttaaaggctc acgaggaatg aaaatggatc ttattgtacg ggaattcgta
26881 ataaaatatt aagcgaaatc attgtattaa tttacagtaa caaattatta ggaactttaa
26941 ttgtgacaaa acgtgtaatt atttttggtg gaacggggtt tatagggaaa cacatcgtaa
27001 gacgcttggc agcagaagga tatttaataa agatattcac tcgtaatcag gaaaaagctg
27061 cttgtttaaa attgtgtggc aatttaggac aaatatcaat atttaaaggc gatttttttg
27121 atgaaaaatc agttctggat ggtatggaag aatgtgatgt agctataaac ttagtgggaa
27181 tattatatga agcaaaaaag cacgattttt acgctgttca cgttaaaata gctgaaagga
27241 tagcaaaagc tgcaaaaatg aaaaatgtac ctatgatgat acatttttct gctatgggaa
27301 tagaaaatag caagttgtca aaatatgcca aaagtaaatt ggaaggtgaa aaagctgtaa
27361 cttcagcatt tcaagaagca ataataatta ggccaagcct tgtatttggt aaagaagaca
27421 acttctttaa taaatttgca agattagcaa ctattcttcc tttcttacca ctaattggca
27481 atggaacaac taaatttcag ccaatatgtg taacagactt agctgaagtg gtatatcgta
27541 ttatcagttt taataagcaa gacaagaaaa tttataacat gggtggacca aaggtttact
27601 ctttcaaaag cttattgaag tttattctga atgttactaa cagaaagtgt ttgttggtca
27661 atgtatcttt tccaatggca aagttaatag ctttcttttt agaaaataaa attatttcta
27721 tgctgctaaa accaataacc ggagatacga accctatgct tactcaagat caggtgaaag
27781 ttatgatgaa tagttcaatc gaaaagtcag ttgaccttga aacaatgaag gttcgaccat

27841 tagcaattga aaatgtggtg ccagagtact tgaagattta tagaaagtat tgataaaaga
27901 gtgtgcccgg tgggattcga acctacgacc cacagcttag aaggctgttg ctctatccag
27961 ctgagctacg ggcacacaac atatataaaa tgcttcttag tacaagtgaa gtcaagtact
28021 tcgtgtgaat atgatgttta tcttgcgaca ccttgtcatt gcagttgtgt gtcgcgcgct
28081 gccaaaattg gatgcgagaa gtctatttat tttagaaaat attatataga aacttacttg
28141 acacccttcg ccagcgccct tatcatgata gtgaagctat tgtatttgct ttcgccaatc
28201 tgcagattaa aaggtaagga ttacttaatg tatcggcgtc ttatgttcaa ttttttgcag
28261 tatatagata ctgtatgtct ttacaaaact tcatctacat ctagattttt atctaaataa
28321 gctgaacgcg cttataaagc gttacaagac atcaaaaaat gccaatactc gacagagata
28381 gtaaaagact agctaactcg gggattcttt gtcttttttt ctgcttagta aatttcttaa
28441 acatttgcag cgtaagttag ttgcaattaa aagcggctga atctcgcttg tcaagcgttt
28501 aaaacataaa aaacgccaat attttaaagc ggataataac cccagggctt cttttgcctt
28561 tttttttattt ggtaaatttc ttaatgttta tggctaaacg acaatcgtca tcccgctact
28621 tgttagcggg atctagagat accgcgaatg aatcgcggta tgacggtttg cggcggcatg
28681 acctcgtcat tcccctacgt gttagcggga tctagagata ccgcggcggt atgacggttc
28741 gcgcggtggc atgacctcgt catcccgctg cttgttagcg gctgagatac cgcggcggta
28801 tgacggtgta gatgatggtt aagtaattcg tcatcccgct gcttgttagc gggatctctt
28861 gttagcgccg cggcggtatg acgtaggatt gttgtcattc cgctacttgt tagcggctga
28921 gataccgcgg cggtatgacg gttcgcggcg gcatagtaat tcatcatccc gctgcttgtt
28981 agcgggatct agagataccg cgacgtctca cctatagcct tcagtgctcc tttttttgtc
29041 atcccagcgt cacgcgttgg tttcaacatt atatcatgaa ctttctaaaa ttcattgaac
29101 tgtgctttca aacggtagtg ccggggtgtg agtataacga ttatcagtat ataaaagtca
29161 tagcagatag gctggaagca gcgagcgctg gtgaagtgaa acgaataata ttcaatatgc
29221 ctccgcgctc gatgaagtcc atgtgcataa gcgttgcgtg gcccgcatgg atactgggaa
29281 accaaccaac tgcgagaata atagttgcaa gttattctca gcggctcagc gaaaagcact
29341 cgctcgacac caggtgcgtg atgcagtcta gttggtatag agcgctattt ccagagatag
29401 aactatccaa agaccagaat acaaaatata aatttcaaac agtgcagaga ggatacagaa
29461 tcgcaacatc agttggtgga acactaactg gtgaaggtgg tgattttatc attgtggacg
29521 atccgctcag ttctgctcaa gctttgagtg aaacgcttag aaagcgtgcc ataaactggt
29581 ttgatcagac tttagtaacc aggctgaacg atagaaaaaa aggagtcatc gttcttgtaa
29641 tgcacaggct acaccaggag gacttaactg gacaccttct ctccaagcca aaaaacatat
29701 ggcatcatat ttgtttgcca atgatttctg aaaataagca ggttatttat tcaatcaaaa
29761 agccaatgcc tcccgttcct gtcatccgag tagctcagca ctgtgcgacc aggcgattat
29821 ataataaacc agcgtcacct gtaaggatat tatattcaag agaagaaagt cagttgctat
29881 atcccctaga tggaggaaaa gaagaaattg agatgataaa ggttgaactt gggagttacg
29941 cttttgctgc tcaatatcaa caaaaccctc tgccgctttc aagtggtata attaaaccag
30001 agtggttgaa acgctataaa aaatttcctg ataatctctc gcatataaca caaagttggg
30061 atactgcggt ttcaacaagc gacatgagca actttagtgt ctgcaccacc tgggcaaaaa
30121 tagataataa attctattta cttgatgtat atcgcgcaaa gcttgagtat ccaaagctta
30181 aagaacaggt tttgtcacta gctgcaagat ggacgccaca cgcaatttta atcgaagcaa
30241 aaacaagtgg tcaacaattg gtgcaagagc taaagacaaa cagtgattta ccgattattg
30301 aaatagtgcc acatgatgac aagctcactc gatttcatca aattgttccg attatagagt
30361 ctggcaaggt tttctgccca catcaagcag tatggctgaa cgattttgag tatgagattt
30421 taatgtttcc tgaaacgcgc cacgacgacc aagtggacag taccgtgcaa tatttgcagt
30481 gggtgagaaa cagtacctcc agggttgcag ttatacgagc attgtgattt gagtctacca
30541 gtactgggat gacagctagg agacaaccgt catcccgcta cttgttagcg ggatctatgc
30601 taaaagatac cacggcggta tgacgtagaa ctgctgcttg ttagctgatg agataccgcg
30661 aatgaatcgc ggtatgacgg ttcgcggcag catgacggta agctcgtcat cccactactt
30721 gttagcggga tctcttgtta gcggatgaga taccgcgaat gaatcgcggt atgacgtagg
30781 actgctgtca ttccagtgtc agctactttc atgacaccgt ctactttgta aaattcacaa
30841 gaaccattaa aatatttttt tacttttcat tgtatatttc tcaacatatt atctttaagt
30901 actaaaataa ttaacaatat ggcattatta atagttgaat cacctgcgaa agcaaagaca
30961 ataggtaaat atttgagtaa agagttcaaa gtagctgcat cctttggaca tgtgagggat
31021 cttccagcaa aaaacggttc tgtcgatccg gataatgatt ttgctataaa gtatgaaacc
31081 attgaaaaag cagaaaagta tataaaagag ctagtaaaag ccgcaagcaa aacatcagat
31141 atatatcttg caacagaccc agacagagaa ggggaagcaa tggcttggca tgtgatagag
31201 acattaaaag aaaagaaagc aatcagtaat gaaaacaaca tttatagagt agtctttaat
31261 gagataacaa aaagagcagt acaagaagca ataaaaaatc cacgtgaaat taatatggat
31321 ttagtgcgtg cacaacaagc acgtagagct ttggattatc tagttggatt tagtttgtca
31381 ccactgctgt ggacaaaatt gtcgggaagc aagtctgcag ggcgagtgca gtctgttgca

31441 ttaaagatta tatgcgaacg agaagatgaa attagtaaat ttataacaca ggagtattgg
31501 agcataaaag cagaaatgca aaatagcaaa gatgaggctt tttttgctat gctaagccac
31561 tatgacaata aaaagctaga aaaatttgat attaagaatg aagaagaagc aaagaactta
31621 gtcagggaga ttgaatcaag gcaatatgct gtaagcacag tagaacgcaa gcaagttaag
31681 agaaacccgc ttcctccgtt tattacttca agtcttcagc aagatgcagt gaataaactg
31741 tattttaatg tgaaaagtgt tatgcgcata gcgcaaaatt tatatgaagg tatcgatatt
31801 ggtggtgaaa ctgtagggtt gataacttac atgcgtacag atgggtttca tattgcagat
31861 gaggctataa actcaattag agggtcaatt aagtcattat atggtgataa atatttaccg
31921 cagtctcctc gtaaatatgt aaaaaaggtc aaaaatgctc aagaagcaca tgaagcaatt
31981 cgtccaactg atatcaatag aacgccgggt agtattaagg attacttaac gccagagcaa
32041 tttaaattgt atgatttaat ctggaaaagg accattgcaa gtcaaatgga gtcggcgatt
32101 cttgatcaag tggtagtcga aattagttct actgaccaga aagtaattct gcgagcaagt
32161 ggatcaagta tattctttga tggtttttat agagtctacc aagataacat agaagccgaa
32221 aatgaaggcc tgctacctgc catgaaggaa ggggaagcgt gtaagctgat ttcagttgag
32281 ccaaaacaac atttcactca accgccacct cgttatagtg aagcaagtat cgtaaaaaaa
32341 atggaagaaa tcggtatagg tcgaccatca acttatgcaa caattatttc ggtattacaa
32401 gatcgtgagt acgtttcatt ggatagcaaa aggtttattc caagcagccg cggtaaaatc
32461 gttactatat ttttagaaac tttttttcag cgttgtgtag agtatgattt cacagcacaa
32521 atggaagaga agcttgattt aatttcaaat ggacatgcag attggaaaaa agaattaggc
32581 cacttttggg tgccattttt tggtcatgta aactctgtca agcaaatgac gcatgatgaa
32641 gttttcagcg gcattcacga tttggtaatc gattggtttt gttcagaaga aggaaaaaaa
32701 gagatgaata caaaatgccc tgcttgctct gatggcatat tgaaattgaa ctttggaaaa
32761 gccggggtgt tccttggatg ttctaattat cctgaatgca accatacaaa agaaattacg
32821 ggcagtaacg acaattcaga atatccaaaa agtttaggta tagatgatat aacaaaccaa
32881 gaagtgatga tcaaaaaggg tccttttgga ctttatctgg agtttaataa cgagttaaaa
32941 aagaaaaaaa cggtttccat accaaaagat ataaatgtta atgagattga tctgagcacc
33001 gctactcagt tactttcctt gccaaaagta atcggagaac accctgaaac cggaaaggaa
33061 gtaaaaatag gccttgggcg atttgggtac tatattttct atgatggtag atacttttct
33121 cttaagaaaa gctccaaaga ggtgctgaat agagaattga gcgaagttgt acaaattatt
33181 gcaaacagtc catgcaaaga gttaaaatct ctcggggtta atgaaaaggg aaaagaagtt
33241 tttatctgca acggtaggta cggattctat ataaaatgcg gtaaaacaaa cgttgctttg
33301 ggcaagagtg cagatattga aagtatagat ctggagaagg ctttagagtt gattaagaat
33361 aagaagtaga cttttattga aatattaggt aaagttaaaa tatactctaa taatgcggtg
33421 ggataaatga tattttacac acagggcgac actccagctt tatgtaggtt agattacacg
33481 gatgtaagta agttagtagg cataccaaat atcacaagac ttgatttaac tgagtgcaag
33541 attagtgtta aagatgcaaa agaattagcc aaacttacaa atcttacttc acttcattta
33601 ggtaagtgca agattagtgt tggagttgca aaagaattaa ccaaacttac aaatcttact
33661 tcactcaatt taattggttg caagattagc aatgaagttg caaaagaatt agataaactc
33721 aaaaaaaatc ttccttcact taatataatt ggttacaaga ttagcggtaa aggtgcaaac
33781 gtaaaattgc aggatgaggt atcaaactcg caagaaaaac ttaaaagcag tagcaaatac
33841 tatatttgtg gagcaacagt tggtttagtt attggtctag ttgcagcata ttgtttaggg
33901 gctgctgctt taactcctgt tggtgtttct gttgcagcgt ttattgcatc tgctgtggtt
33961 ggcgcgttac tcggagctgg ggtgggttat gctgtgagta catatcttga aagcactgaa
34021 ttaaacaata tgcaaccacg aaggagttgt tgaagatccg tgttaaattc tagtacagga
34081 gctttttttct aatgctaaag tttacagttg aaacaaacca cctggccacg tggaactgat
34141 ttcagtttat cagttgaaac ttgatttgag cagagtatgc attttattgt gttgcttgga
34201 gtaagtgagt tatcaacagc aattctatca tcaaaaacaa aacactcacc ttcccaatta
34261 tcatttttat tatgagtttt ttcgaggtaa gaaagaatgc cgcctttcag gtggtacaca
34321 tcgttaaatc caagattttt catgtacgct gttgattttt cgcatctaat tccaccagtg
34381 cagtacatag ctactttcag gtctttactc tcagaaaatg actctgtcca ctgaggaaac
34441 tcacggaaac attgtgtgtg tggattaatt gcattcttaa acttgcctaa tttcacttca
34501 tactcgtttc gtgtatctat tactaaaacg tcaggttgag aagtaaaatc atcccaatgc
34561 tctggatcaa catatttacc cttcagggaa atatcaagat tgcttacacc aagattcaca
34621 atctctcttt ttaattttac cttcatcttg ctaaatggtt gatattctgc tgcactctct
34681 ttccatgtaa ggtcccttag cctataatca gaacgtagaa aatcgaatat tttatcaatt
34741 gcgtttcttt caccagatat tgttgcatta acaccctctt ctgcaaggag tatagtgccc
34801 tttaactcaa cgttgttgca tgtagctttt atttcgtctt tcatgtcata ataattagaa
34861 agttctacaa aatgatagaa agttgcaatg acgaagctca tgattattca cttatcaact
34921 aaaaacatga tatacaaaat tataaagata ttcaatagta cggctgttaa ttactggata
34981 tcttcagttt ttacatataa cattttaata taaaatatag attcaaatta atgtttttaa

```
    35041 gagtatttaa aaatatattc ttatttctag taagtatatt attcgtctgt ccaatattat
    35101 cattaatatc aattctattt acagatacag caaattccgg atgggtaatc agcacacttt
    35161 tccctgaata tatactgagt acgttaattt tgatgatagg agtgggggga atatctttca
    35221 tatttggagt aattccagcg tggctcacca catttttttc atttcctgga agtaaaattt
    35281 ttgagattgc tttgttcttt ccgatatcga ttcctggata tatagtagcg ttcgtctatg
    35341 taaatacact agaattttca ggtccagtgc agagctcatt aagagacatt ttagagtgga
    35401 gcaaaggtga ctattggttt cctgaaataa aatccctagg tggtggaata ttagtaatgg
    35461 gatttagttt atatccatac gtttatatgt tagtccgctc aagtctaaag aatgttagta
    35521 actcagtcac tattgcatca acacttgggt tctcctcatt gaagagcctg ttttctgtca
    35581 tcataccctc catacgccca tcgattatag ctgggttgtc tttggtacta atggaagtaa
    35641 ttacagattt cggcacacca cagtttcttg ctatcgatac ttttacgacg ggaatatatc
    35701 gcacctggtt tttactgcat gacaaatatt caactactgt tttagcagtt gcagaattaa
    35761 ttttcgttgt gatactaata gctatcgaaa aaaaactaca aaaaaaagga atatcttact
    35821 cttcaatcaa taccaattca gattatcaca ataaacggag tataagtggc gctataccgt
    35881 tgatctttgc ttatcttatg tgtgtattgc cgatattaat aggttttatt ttgccaatga
    35941 ttccgctcat atattggagc atagagaagg ggtttttcat atacgaagca agattctata
    36001 acataatagc aaatagcatt agtttatcat ttatcaccgc attaatctca attagcattg
    36061 caataatgat tggatatacg gcgcgtaaaa ataaggtaat caacaatata gcgcgtctca
    36121 tttctttagg ctacgcaatt ccaaatgcaa ttatcgcaat tagtataata atatttttaa
    36181 gcagaatatc ctctttcatt actcaatatg ttgtggaaat tagcttagta ggaactattg
    36241 gcgcattagt ttactcatat ttattccgtt tttttgctat atctttcaag gcggtagagt
    36301 cagggctcaa aaaaacacca aatgaaattg agtggactgc atataccatg ggtcacggac
    36361 ctatttccac atgtttgaat gttcatatcc ctctcatcaa gaagagtata ttatcgggat
    36421 ttttgctcgt atttatggat actatcaaag agctcacagc aacactcatt ataagaccat
    36481 ttaattttga aactatatca actagaatat atgaacttgt aagcgatgag cgctatagag
    36541 aagctgcccc attttcgtta ataatagtta taataggctt aatctctaca ataaccttat
    36601 ttacacttga tgataaggat aaaaaataaa cgtaactgtt cacagatttc cacttaattc
    36661 ttcaatctca ctaatagaaa ggccagtaaa cttaacaata gtgttgacat caacattatt
    36721 agccagcatt gcttttgcga cttcaatttt cccttcaatt ttccctttct ctataccttt
    36781 ttgtatacct ttctcttcag caagatcaag tctgtattca agaatagctt cttctttgcg
    36841 tagatccatt atcctttctt cataggctac taaatctttt tcgttccaac gaaacttgtc
    36901 taattcatca tatgctagct ttattattgg cgctttttct gctatctctt ttaagtcctc
    36961 ttctgttgtt tcttctgcgt atttaaaaaa gaaacaccac ctttctacta tactttctaa
    37021 ctgctctact ttattttttg cgaatttagg caactcaata aagacaaact gaaaatcttt
    37081 taagtaatgg ccgttagttt ttatatcacg tatattgtga gtagaaatat aatcaacttc
    37141 ttcaggaagt aagttacaat ttgatatagc aataaagaag actttcttta aatcaatgta
    37201 gttgccagat ttatctaatt gtcttgagta ggctttagca gcatagagtt gtgcgcgctt
    37261 ttcaaagccc ttgtctctag cgagctgc
//
LOCUS       JAQZAU010000046         8587 bp    dna     linear   ENV 17-MAR-2023
DEFINITION  MAG: Wolbachia pipientis isolate wEsol contig_32109, whole genome
            shotgun sequence.
ACCESSION   JAQZAU010000046  JAQZAU010000000
VERSION     JAQZAU010000046.1
DBLINK      BioProject: PRJNA929258
            BioSample: SAMN33142973
KEYWORDS    WGS; ENV; Metagenome Assembled Genome; MAG.
SOURCE      Wolbachia pipientis (insect metagenome)
  ORGANISM  Wolbachia pipientis
            Bacteria; Pseudomonadota; Alphaproteobacteria; Rickettsiales;
            Anaplasmataceae; Wolbachieae; Wolbachia.
REFERENCE   1  (bases 1 to 8587)
  AUTHORS   Fiutek,N., Couger,B.M., Roy,S., de la Torre,J.R. and Connor,E.F.
  TITLE     Genomic assessment of the contribution of the Wolbachia
            endosymbiont of Eurosta solidaginis to gall-induction
  JOURNAL   International Journal Molecular Sciences
REFERENCE   2  (bases 1 to 8587)
  AUTHORS   Fiutek,N., Couger,B.M., Roy,S., de la Torre,J.R. and Connor,E.F.
  TITLE     Direct Submission
  JOURNAL   Submitted (10-FEB-2023) Biology, San Francisco State University,
```

```
            1600 Holloway Ave, San Francisco, CA 94132, USA
COMMENT     ##Genome-Assembly-Data-START##
            Assembly Date          :: JUN-2019
            Assembly Method        :: FLYE v. 06-2019
            Genome Representation  :: Full
            Expected Final Version :: Yes
            Genome Coverage        :: 100.0x
            Sequencing Technology  :: Illumina HiSeq; PacBio Sequel
            ##Genome-Assembly-Data-END##
FEATURES             Location/Qualifiers
     source          1..8587
                     /organism="Wolbachia pipientis"
                     /mol_type="genomic DNA"
                     /submitter_seqid="contig_32109"
                     /isolate="wEsol"
                     /isolation_source="galls on Solidago altissima"
                     /host="Eurosta solidaginis MN-2018-EFC"
                     /db_xref="taxon:955"
                     /environmental_sample
                     /country="USA: Cowling Arboretum, Carleton College,
                     Northfield, MN"
                     /lat_lon="44.48 N 93.13 W"
                     /altitude="280 m"
                     /collection_date="2018-11"
                     /metagenome_source="insect metagenome"
                     /note="metagenomic"
     gene            <1..994
                     /locus_tag="wEsol_00388"
     CDS             <1..994
                     /locus_tag="wEsol_00388"
                     /codon_start=1
                     /transl_table=11
                     /fullproduct="qcoverage 100 , hcoverage 97.0588235294118
                     , similarity 100 , identity 100 , evalue 1.13e-213 ,
                     alnlength 330 , Bacteria , Proteobacteria ,
                     WP_155969183.1 MULTISPECIES: hypothetical protein
                     [Wolbachia]"
                     /translation="TDESDRKSSDECSEAKPQSESPSSRKGSESNSFSGSSTLSKKSI
                     MPELEKLFILELEKWFLYFQQNGKKLDSTRSTHELSSKKTATSSERKVKSNSPSKVTP
                     VTEVHTENKPTELNDSITELPSQGTVGKLNMPGDRSSKISVDSGLGSLERGQSPSLIE
                     EFSDSLQQTPLIVDKLENIFSKLLSVVHNLLKNPASPTHKDVSGGLKLQQHLDSDQRE
                     KLYKALSSKMKKKLDSELFLPEYEKLLYLLYVDKREVRLLLSPPGEVEQVCSVLPSEE
                     KEQAQSSSSEEVVRLFQEFFSNQKVSDNYLTSSKIDSPEIETCLKKEISQAVCT"
                     /product="hypothetical protein"
                     /besthit="qcoverage 100 , hcoverage 97.0588235294118 ,
                     similarity 100 , identity 100 , evalue 1.13e-213 ,
                     alnlength 330 , Bacteria , Proteobacteria ,
                     WP_155969183.1 MULTISPECIES: hypothetical protein
                     [Wolbachia]"
     gene            complement(1177..3084)
                     /locus_tag="wEsol_00389"
     CDS             complement(1177..3084)
                     /locus_tag="wEsol_00389"
                     /codon_start=1
                     /transl_table=11
                     /fullproduct="qcoverage 100 , hcoverage 100 , similarity
                     100 , identity 100 , evalue 0.0 , alnlength 635 ,
                     Bacteria , Proteobacteria , WP_141457217.1 MULTISPECIES:
                     excinuclease ABC subunit UvrB [unclassified Wolbachia]"
                     /cog="UvrB COG0556 663 Helicase subunit of the DNA
```

```
                     excision repair complex"
                     /pfam="ResIII DEAD AAA_19 Kinesin Pfam-B_6675 Pfam-B_5382
                     Pfam-B_248 Pfam-B_467 Helicase_C UvrB UVR"
                     /tigr="TIGR00631 uvrb 658 excinuclease ABC subunit B"
                     /product="excinuclease ABC
                     subunit UvrB"
                     /translation="MEFQIATHFQPAGDQPQAIDSLIAGLNSNKRDQVLLGVTGSGKT
                     FTMANVIARTNRPALIMAHNKTLAAQLYEEMKGLFPHNAVEYFISYYDYYQPEAYLPQ
                     TDTYIEKDSAINERIEMLRYSTVCSLLERRDTIVVASVSCIYGLGSPESYRSMIIPLS
                     VGNKIRINDFLSNLTDLQYKRSDARFERGYFRVRGDIIDIFPSHYENKAWRLSLFGDE
                     IEEISEVDAITGNVTKGVNKITIFPNSYYITPREALLQAIELVKKELHERLDYYYSQN
                     KIVEAKRLEQRTNFDIEMMRTTGICKGIENYSRYLYGMEAGDPPPTLFEYLPKDVILF
                     VDESHVTVPQVGAMYSGNEARKKKLIDYGFRLPSAFDNRPLKFEEWEGVRPQTVYISA
                     TPGKYELAKTNNVFIEQVIRPTGLTDPICIVKPIESQIDDVIHEAQVTIGKGFCVLIT
                     TLTKKMAENLAEYMSELNMRVSYLHSDIAALERIEIIYKLRSKEIDVLVGVNLLREGL
                     DIPECGLVAILDADKEGFLRSETSLIQTIGRAARNAEGRVILYADKITGSLDRALRET
                     ERRRKKQEEHNILHNIIPKTIIKPISNTLQERAIAEPKVNTNKDDLRKQMIEHAKNLE
                     FEEAARIKKLL"
                     /besthit="qcoverage 100 , hcoverage 100 , similarity 100
                     , identity 100 , evalue 0.0 , alnlength 635 , Bacteria ,
                     Proteobacteria , WP_141457217.1 MULTISPECIES:
                     excinuclease ABC subunit UvrB [unclassified Wolbachia]"
     gene            complement(3480..3776)
                     /locus_tag="wEsol_00390"
     CDS             complement(3480..3776)
                     /locus_tag="wEsol_00390"
                     /codon_start=1
                     /transl_table=11
                     /fullproduct="qcoverage 100 , hcoverage 100 , similarity
                     100 , identity 100 , evalue 1.48e-61 , alnlength 98 ,
                     Bacteria , Proteobacteria , WP_081600786.1 MULTISPECIES:
                     transposase [Wolbachia]"
                     /cog="COG3293 COG3293 124 Transposase and inactivated
                     derivatives"
                     /pfam="DDE_Tnp_1_2 DDE_Tnp_1"
                     /product="transposase"
                     /translation="MVESAKENLSEVKNILVDAGYTGENFATKIKTIIGATVEVIKRS
                     ELHSFAVLPQRWVVERSFAWLEKCRRLWKNCERKLNTSLQMVVLSFISLLLRRF"
                     /besthit="qcoverage 100 , hcoverage 100 , similarity 100
                     , identity 100 , evalue 1.48e-61 , alnlength 98 ,
                     Bacteria , Proteobacteria , WP_081600786.1 MULTISPECIES:
                     transposase [Wolbachia]"
     gene            3886..4059
                     /locus_tag="wEsol_00391"
     CDS             3886..4059
                     /locus_tag="wEsol_00391"
                     /codon_start=1
                     /transl_table=11
                     /fullproduct="qcoverage 100 , hcoverage 100 , similarity
                     100 , identity 100 , evalue 5.67e-34 , alnlength 57 ,
                     Bacteria , Proteobacteria , ERN55436.1 transposase
                     [Wolbachia pipientis wMelPop]"
                     /translation="MKEKVTEIFCIVDDFCNTVDENFAEKLLPSGKKPTRTPEITHSE
                     IFTIILLYQVRQF"
                     /product="transposase"
                     /besthit="qcoverage 100 , hcoverage 100 , similarity 100
                     , identity 100 , evalue 5.67e-34 , alnlength 57 ,
                     Bacteria , Proteobacteria , ERN55436.1 transposase
                     [Wolbachia pipientis wMelPop]"
     gene            4311..4442
```

```
                     /locus_tag="wEsol_00392"
     CDS             4311..4442
                     /locus_tag="wEsol_00392"
                     /codon_start=1
                     /transl_table=11
                     /product="hypothetical protein"
                     /pfam="DDE_Tnp_1_3"
                     /translation="MGGFSVHLVINETGEIQGVTVIKGNVDTCAKFNSEIDWIIVWR"
                     /besthit="qcoverage 95.3488372093023 , hcoverage
                     45.5555555555556 , similarity 97.6 , identity 97.6 ,
                     evalue 4.08e-20 , alnlength 41 , Bacteria ,
                     Proteobacteria , ERN55437.1 hypothetical protein
                     WMELPOP_04488 [Wolbachia pipientis wMelPop]"
     gene            4387..4707
                     /locus_tag="wEsol_00393"
     CDS             4387..4707
                     /locus_tag="wEsol_00393"
                     /codon_start=1
                     /transl_table=11
                     /pfam="DDE_Tnp_1_3 DDE_Tnp_1 DDE_4 DDE_Tnp_1_6"
                     /fullproduct="qcoverage 100 , hcoverage 100 , similarity
                     99.1 , identity 97.2 , evalue 7.02e-66 , alnlength 106 ,
                     Bacteria , Proteobacteria , ONI57662.1 putative protease
                     domain protein [Wolbachia pipientis wVitA]"
                     /translation="MIPVPNLTQKLTGLLFGDKGYIKKDLFHELFDRGLKLVTSIKKG
                     TKNSLMALNEKILLRKRSIIETVFGSLKNKFEIEHTRHRSPINFLVHIFSTLVSYSIQ
                     PLHF"
                     /product="putative protease domain protein"
                     /besthit="qcoverage 100 , hcoverage 100 , similarity 99.1
                     , identity 97.2 , evalue 7.02e-66 , alnlength 106 ,
                     Bacteria , Proteobacteria , ONI57662.1 putative protease
                     domain protein [Wolbachia pipientis wVitA]"
     gene            complement(6374..6760)
                     /locus_tag="wEsol_00394"
     CDS             complement(6374..6760)
                     /locus_tag="wEsol_00394"
                     /codon_start=1
                     /transl_table=11
                     /fullproduct="qcoverage 100 , hcoverage 100 , similarity
                     100 , identity 100 , evalue 1.02e-82 , alnlength 128 ,
                     Bacteria , Proteobacteria , WP_015589263.1 MULTISPECIES:
                     cytochrome c-type biogenesis protein CcmH [Wolbachia]"
                     /cog="CcmH COG3088 153 Uncharacterized protein involved
                     in biosynthesis of c-type cytochromes"
                     /pfam="CcmH"
                     /tigr="TIGR03147 cyt_nit_nrfF 126 cytochrome c nitrite
                     reductase, accessory protein NrfF"
                     /product="cytochrome c-type
                     biogenesis protein CcmH"
                     /translation="MRAVVSLLFILIFHSGVNAFTLDNKLRDKSMEKRATSLFEIIRC
                     PICSGESLSESGSQIAYDMREAIRKKINDGYTDEEIISELKNSYGNSIIIVPPIKSST
                     YILWFIPLTTLLIGCFLIQRYINTTT"
                     /besthit="qcoverage 100 , hcoverage 100 , similarity 100
                     , identity 100 , evalue 1.02e-82 , alnlength 128 ,
                     Bacteria , Proteobacteria , WP_015589263.1 MULTISPECIES:
                     cytochrome c-type biogenesis protein CcmH [Wolbachia]"
     gene            complement(6744..7784)
                     /locus_tag="wEsol_00395"
     CDS             complement(6744..7784)
                     /locus_tag="wEsol_00395"
```

```
                     /codon_start=1
                     /transl_table=11
                     /fullproduct="qcoverage 100 , hcoverage 100 , similarity
                     100 , identity 100 , evalue 2.98e-242 , alnlength 346 ,
                     Bacteria , Proteobacteria , WP_015589262.1 MULTISPECIES:
                     hypothetical protein [unclassified Wolbachia]"
                     /translation="MRKIFIYIIISLLLFIPVAYSGFWYFSACKVKNLLAETILYIND
                     EKFDISHDFSGFPSNLIFRVTNPKFSNEQLTISSDALLIKNRLFDKSVYIYIPSNEVN
                     IAVHDDEKKNIKCHTNDSSHFVVRLNDLPSSLRFNKNSTVIDYIDTLRYEDYGLKCDV
                     LENQQSVITEVNDKSNYIQFHLDKEPSGNTKLGFDFYTYRYKNTASPESYLSIDTKFD
                     YEFVNHISASRINFNIEKFLIQSNNFSLAADGGVRNYNLVTSSFKDKINVAISNYKEL
                     ISFMIGDSKTSDAFEKLMSSLSEKTTDDGIQFSIKYDDDVGSSFIGKLSTTDFMNQLS
                     KITQLTKDESSS"
                     /product="hypothetical protein"
                     /besthit="qcoverage 100 , hcoverage 100 , similarity 100
                     , identity 100 , evalue 2.98e-242 , alnlength 346 ,
                     Bacteria , Proteobacteria , WP_015589262.1 MULTISPECIES:
                     hypothetical protein [unclassified Wolbachia]"
     gene            complement(7792..8139)
                     /locus_tag="wEsol_00396"
     CDS             complement(7792..8139)
                     /locus_tag="wEsol_00396"
                     /codon_start=1
                     /transl_table=11
                     /fullproduct="qcoverage 100 , hcoverage 100 , similarity
                     100 , identity 100 , evalue 1.03e-78 , alnlength 115 ,
                     Bacteria , Proteobacteria , WP_012673282.1 MULTISPECIES:
                     ferredoxin family 2Fe-2S iron-sulfur cluster binding
                     protein [Wolbachia]"
                     /cog="Fdx COG0633 102 Ferredoxin"
                     /pfam="Fer2"
                     /tigr="TIGR02007 fdx_isc 110 ferredoxin, 2Fe-2S type, ISC
                     system"
                     /product="ferredoxin family
                     2Fe-2S iron-sulfur cluster binding protein"
                     /translation="MPSVTFILPDGSKKSYEAAEGETLLNLAHRSDPDLLEGACEGSL
                     ACSTCHVIVDPKFYNAVEIHNPISDEENDMLDLAFGLTETSRLGCQIKITKDIDGLCV
                     TIPRGTRNISLDK"
                     /besthit="qcoverage 100 , hcoverage 100 , similarity 100
                     , identity 100 , evalue 1.03e-78 , alnlength 115 ,
                     Bacteria , Proteobacteria , WP_012673282.1 MULTISPECIES:
                     ferredoxin family 2Fe-2S iron-sulfur cluster binding
                     protein [Wolbachia]"
ORIGIN
        1 tactgatgaa agtgatcgaa aaagtagtga tgagtgttct gaagcaaagc cacagagtga
       61 atctccgagt agccggaaag ggtcagaaag taattcgttt tcaggtagta gtactttatc
      121 aaaaaaatct attatgccag agctagagaa gttgtttata ttagagctag aaaagtggtt
      181 cctatatttt cagcaaaatg gcaaaaaact tgactcgacg cgttcaactc atgagctttc
      241 aagtaaaaaa acagctactt catcagaaag aaaagtaaaa agtaattcac cctcaaaagt
      301 gactcctgtt actgaagtac ataccgaaaa caagcctact gaattaaatg atagtattac
      361 agaactcccg tcacaaggta ccgtaggcaa actaaacatg cccggggacc gtagttctaa
      421 aatttctgta gatagtggtt tagggtcgct tgagcgggga cagtcaccgt cgcttattga
      481 agagtttagt gattcacttc agcagacacc gttaatagtt gataaacttg aaaatatatt
      541 tagtaaatta ttaagtgttg ttcacaatct attaaaaaac ccggcttctc ctacgcataa
      601 agatgtgtcg ggtggactaa agttgcaaca acatcttgat tctgatcaga gagagaaact
      661 atacaaagct ctttcttcta agatgaagaa gaagctagat tccgaacttt ttctccctga
      721 gtacgagaag ttactatacc tactttatgt tgataagagg gaggtgcgtt tgcttctttc
      781 tcctcctggg gaagtagagc aagtgtgctc agttcttcct tctgaggaga aggagcaagc
      841 acaatcaagt tcttctgagg aggtagtacg cttatttcag gagttcttta gtaatcaaaa
      901 agttagtgat aactatctta caagttctaa gatagattct cctgagatcg agacatgctt
```

961 gaaaaaagaa atttctcaag cggtttgtac ctaatccact gctctcagcc accctaagcg
1021 ttttattagc tgaccctgag agcactggtg ttagtttttc tcatccattg tcagatattg
1081 catcgtacta aatatttaaa aagtattaat ataaagatat caataaaatt atttacattt
1141 tcatgctatg cccatagcat catatattac ttaaccttac aataattttt ttattcttgc
1201 tgcttcttca aactccaaat ttttagcatg ctctatcatt tgctttctca aatcatcctt
1261 attagtattt actttcggtt ctgctattgc cctctcctgt aaggtgtttg atataggttt
1321 tattatagtc ttcgggataa tattgtgcaa tatattatgt tcttcttgtt tttttcttct
1381 tctttcggtc tctctcaatg cacgatctaa agaaccagtg attttatctg catacaaaat
1441 tactctacct tcagcattac gtgcagcacg accaatcgtt tgaattaatg aagtttccga
1501 tcgcaaaaat ccttctttat cagcatccaa aattgcaacc aacccacact ctggaatatc
1561 aagaccttcc ctcagcaagt taacacctac taaaacatcg atttctttag atctcaattt
1621 gtatataatt tctattctct ccagcgcagc aatatcagaa tgcaaataac tcactctcat
1681 attcaattca ctcatatatt cagctaaatt ttcagccatc tttttcgtca atgtagtgat
1741 taaaacacaa aatcccttcc ctattgtcac ttgtgcctca tgaattacat catcaatttg
1801 actctctatt ggttttacaa tgcagattgg gtctgtaagc cctgttggac ggataacttg
1861 ctctataaat acattattgg tctttgccaa ctcatacttt cctggagtgg ctgaaatgta
1921 aacagtctgc ggcctaactc cctcccactc ttcaaatttt aatggacgat tatcaaaagc
1981 ggaagggagc ctgaagccat agtcaatcaa tttcttttta cgcgcctcat taccactgta
2041 catcgcacca acctgaggaa cggtgacatg actctcatca acaaataaaa ttacgtcttt
2101 gggtaaatat tcaaacaaag taggcggtgg atccccagct tccattccat agagataacg
2161 cgaataattt tcaatacctt tacatattcc tgttgttctc atcatttcga tatcaaaatt
2221 agtgcgttgc tcgaggcgct ttgcttcaac aatcttattt tgcgagtagt aataatccaa
2281 gcgttcatgc agctcttttt tgactagttc aattgcctgc aacaaagcct cacgcggggt
2341 tatgtaatag ctatttggaa aaatggtgat tttatttacg cctttggtaa cattgcccgt
2401 tatagcatca acttcagaaa tttcttctat ttcatcgcca aataacgata aacgccaagc
2461 tttattttca taatgggaag gaaatatatc aataatatcg ccgcgcactc tgaaatatcc
2521 tctctcaaac ctggcatcag agcgcttgta ttgaagatca gttaagttgc tcaggaaatc
2581 attaatacga attttatttc caacactaag cggtatgatc atgctgcggt agctctcagg
2641 cgaaccaaga ccgtatatgc aggaaacact ggcaactacg atcgtatccc tgcgctccaa
2701 aagggagcag acggtggaat aacgcagcat ctcaattctt tcgttaattg cagaatcttt
2761 ttcgatataa gtatcagttt gtggtaaata ggcttcaggt tgataataat cataataaga
2821 aatgaagtat tcaacagcat tatgggggaaa caatcctttc atttcctcat aaagctgtgc
2881 tgctaaagtt ttattgtgtg ccataattaa tgcaggcctg tttgtccttg caataacatt
2941 tgccatagta aaagttttcc cagaaccagt aactccaagc aaaacctgat ctcttttatt
3001 gctatttagc cctgcaatta agctatcgat tgcctgtggt tgatccccag ccggttgaaa
3061 atgtgtagcg atttgaaatt ccatagactt tacttactat ctttgattca ttgtatagta
3121 aacactatat tgcttttaag ataagttgta tagctatcgt aaagccataa aacattaacc
3181 aacaatgtca tgcaagtagc tgacactgga atcttgtttc aatgttaaac aaaataccat
3241 agaattcaag aagacaattc ctaaaaaatc cctacatcca aaaaaaggca aaatgctgct
3301 tgaataagct aaattttttt agatgcaaaa acaataaaac atagttgata ctaaagttat
3361 agtactccta tgacaacgtt acaaaaatta aggaaatgga tagctctgat caattggaat
3421 agtattttca ggtaccggtg gtcaatatga catttttaaa ataaagattt agaacctgtt
3481 taaaatcttc tcaatagaag agaaatgaag gaaagaacga ccatttgtag gctagtgtta
3541 agttttcgct cgcaattttt ccacaatcgt ctgcattttt ctaaccaagc gaaagatcgc
3601 tctacaaccc atctttgtgg caatacagca aaggaatgta attcacttcg ttttattact
3661 tctacagttg ctccaataat cgtttttatt ttagttgcaa aattttctcc tgtataacct
3721 gcatcgacca gtatattttt aacttccgag aggttttctt ttgcgctttc aaccattctc
3781 acagcactgc taaccccagt tgtaaattag aaaaaaataga aaaggtaagg aaatagggta
3841 aactccgagg tatttgatat tcattcctaa aataggaggt taccaatgaa agagaaagta
3901 acagaaatct tttgtatagt cgatgatttt tgcaatacag tcgatgaaaa ttttgcagaa
3961 aaacttctac caagtggcaa aaaaccaacc aggacgccag agattacgca ttcagagatt
4021 tttaccataa tattgctata tcaagtgcga caattttaaa attttctatg tgcattattt
4081 gaaattatta tacgaatcag cgtttccaaa attgccatcc tatagtaggt ttatcaggtt
4141 aaaatcgcgg attttatggt atttagcgct cttaatgcaa tggttttgcg aacagtcaaa
4201 agtgacaggg atttcgtaca tagatgcaac atctatcgct gtttgccatc caaaaaggac
4261 ctcgagaaac aaagttttca aagggttggc aaagattgga aagactactt atgggtggtt
4321 tttcggtaca tctagtaatc aatgaaactg gcgaaattca aggggttacg gtgataaaag
4381 gcaacgttga tacctgtgcc aaatttaact cagaaattga ctggattatt gtttggagat
4441 aaaggatata tcaaaaaaga tctttttcac gaattattcg atagagggtt aaagcttgta
4501 accagtataa aaaagggtac gaaaaactca ttaatggcgc taaatgagaa gatttttgtta

4561 agaaaaagat cgattattga gacagttttt ggctctctga aaaacaaatt tgagattgag
4621 catactcgcc atagatcacc gataaatttt ttggtacaca tttttctac gcttgtttca
4681 tattccatac agccgctgca tttctaagct ttacttcgtt ggctaatccg caactggggt
4741 atatagggtt gacaccaagg caatattctt ctggagaagt tgatcggcat ggcagcatat
4801 caaagatggg accagcggaa tgtagaagta tgttatatga agctgcacaa acaatgctta
4861 cagttaccaa gaggacattt aaattaaaaa actggggttg cgaagaaaaa aggaatgaaa
4921 aaagcaattg tagcagtagc aagaaaatta gctgtaatta tgcataggat gttggttgat
4981 aaaacagaat tttattatca ataaacatca aaagagcata ggacaataat tttattgaaa
5041 ataaagatag caatgccgat ggaagggaca agcaagaact tagttagact tcgtggtaat
5101 accaatcccg ctacacagta acggatagac cataataaga aaaaaagcca tactgaatta
5161 agtaaagtag caggtttaaa tggcatgaag atcaaaatta tttggataaa aagtggtgga
5221 tcaggcaaaa gaaatgctga gaaagtaaga aatacgctat gttgcaaaaa aactaatgct
5281 gtattgcagc aaaaaagcac agtataaaca gccgtaagca agatatagct gcatttcaag
5341 aaaggcactt actgctgacc taaaatttgg cagagacaaa aattatttgc tccgcctcaa
5401 cgcgtagaaa aactatattg aatcaaagtc acgtgacaag ttgggtgtgg atagaggaaa
5461 acccacaata ttactataaa aaaatgaaaa taagattcaa gaaaagtttg gtttgaatat
5521 cagcaaatct acagtacacc gtaatatgca aagaatgaaa ttctcatata cacaccaaga
5581 ccagtgcata acggacaaga taaaagtaaa caagaggaat ttaaaaaaaa atcttatgaa
5641 actaattgtt cagttcctga aaagagctat ttttcgtcga tgaatcgcgg tttggcacac
5701 attcaaagat tggacacggg tggttttaaa aaaggcatta ggacacaggt ttaggtacac
5761 taggtaggca aaattttttta tctctatagt gagttatccc agaaatggag aagttctagc
5821 tcaaacgtca atactgattg tatgaatata ttccttgagc agatgtcaca ttttaggaac
5881 aagggaggca tttcttatca tggattgtgc tatgttgcag aagtcaaaaa attaaaagta
5941 cctaaaatat cgacattata tacctaccac catactcacc tgacctcatc ctgttgagag
6001 ttttggttat aatataaaac atgaacattt ttgcgtaaaa aatctagaca caattgcttt
6061 gcttgagagc gcttttgtgc aaatttatta cctctctttt cactcccatt aaaaactctg
6121 catgccgctt atttgattca cagtaaatga gattggtaga aacatgttgc cgtccacttc
6181 cgcctaacaa gtataatgcg gcttgacaaa tttattgtca tctcagaccg cggagagaac
6241 catatgctac ttttaactta tgtaaaatta ccaaggatta tggagaagat atgctgtttt
6301 gatttgtgtg aaaattattg ttgacttatg tgcaattaga gaacctaagt cactttagct
6361 atatataaaa ggtttaagtg gttgtattga tgtatctttg tattaaaaaa cacccaataa
6421 gcagagttgt aagtggaata aaccacaaaa tgtaagtgct agattttata ggcggtacga
6481 ttataattga attgccgtaa gaattttttta actctgagat tatctcttca tctgtgtatc
6541 catcattgat ttttttacga attgcttctc gcatatcata cgcgatctga gatccagatt
6601 cagacaatga ttcgccagaa catattggac accttattat ttcaaataaa ctggttgctc
6661 gtttttccat gcttttatct ctgagtttat tatccagagt aaaagcgttt acgcctgagt
6721 ggaatatcaa tatgaaaagt aaactaacta ctgctctcat ccttggttaa ttgggttatt
6781 ttactgagct ggttcataaa atcagtggtg gataactttc caataaagct tgaccctaca
6841 tcatcatcgt actttattga aaattgaatg ccatcatcag ttgtttttttc tgataaggaa
6901 gacattaatt tttcaaacgc atctgaagtt tttgagtcac caatcataaa cgagattaat
6961 tctttataat ttgatatagc cacgttgatt ttgtctttaa atgaagacgt aaccaaatta
7021 taatttcgca ctccaccatc tgcagcaaga gaaaaattat tactttgaat taaaaatttc
7081 tctatgttga aattaattct ggaagctgag atgtggttta caaattcgta atcaaattta
7141 gtgtcgatac taaggtaact ttcaggactt gcagtatttt tataccgata agtataaaaa
7201 tcaaatccta gtttagtatt tccacttggt tctttatcga ggtgaaattg aatgtaattg
7261 cttttatcat tcacttcagt tataacactt tgctgatttt ctaaaacatc acattttaat
7321 ccataatctt cgtaacgaag tgtatctata tagtctatca cggtactatt tttgttgaat
7381 cgcaatgaag atggtaaatc gtttagtcta acaacaaagt ggctgctatc gtttgtgtgg
7441 cattttatat tctttttctc atcatcatga acagcaatgt tgacttcatt gcttggtatg
7501 tagatatata ctgatttatc aaacaatcta tttttttatca ataaagcatc agatgagatg
7561 gttaattgct cattggaaaa ttttgggttt gtcacacgga aaattaggtt agaaggaaat
7621 ccactaaaat catgtgaaat atcaaatttt tcgtcgttaa tgtatagtat tgtttcagct
7681 aaaagatttt ttaccttaca tgcagaaaaa taccaaaaac cactatacgc tacaggaata
7741 aacaaaagta aagagatgat aatatagata aaaatcttac gcatgattcc cctatttatc
7801 taatgatatg tttcttgtac cccttggtat ggttacacac aagccgtcga tatcttttgt
7861 gatttttatt tggcatccaa gcctcgacgt ctctgttaag ccgaaagcta ggtctaacat
7921 atcattttcc tcatcagata tagggttatg tatttctaca gcattataaa actttggatc
7981 aacaattaca tggcatgtag agcaagcaag agaaccttca catgcacctt caagcagatc
8041 tggatcactt ctgtgggcta aattgagcag agtttctccc tctgcagctt catagctttt
8101 cttactccca tcaggtaaaa taaaagtaac agatggcata ttattcctat taaaatcctt

```
     8161 actaatttct ttatatccta caggtaataa ctaatatttg caaactcttt acaattccag
     8221 tacctatatt tcttgttatc caagtagctc tttgttgtca tccgagtagc tgacactggt
     8281 tcctttatga cggcagtgcc ctcttcttgt catcccagtg ccctgattac ttggatccag
     8341 aaaaaagaat ggtgtcatgc aagtagcctc cttctcctgt catccgagta gctgacactg
     8401 ggatgacacc ccacttaacc gtcatactgc cgcggtatct cttagccgct aacaagcagc
     8461 gggataacga gcttatcgtc acgccgtcgc gaaccgtcat accgcgattc attcgcggta
     8521 tctcagccgc taacaagaga tcccgctaac aagcagcggg atgacgattg tcgtttagcc
     8581 ataaata
//
LOCUS       JAQZAU010000047           28030 bp    dna     linear   ENV 17-MAR-2023
DEFINITION  MAG: Wolbachia pipientis isolate wEsol contig_32754, whole genome
            shotgun sequence.
ACCESSION   JAQZAU010000047  JAQZAU010000000
VERSION     JAQZAU010000047.1
DBLINK      BioProject: PRJNA929258
            BioSample: SAMN33142973
KEYWORDS    WGS; ENV; Metagenome Assembled Genome; MAG.
SOURCE      Wolbachia pipientis (insect metagenome)
  ORGANISM  Wolbachia pipientis
            Bacteria; Pseudomonadota; Alphaproteobacteria; Rickettsiales;
            Anaplasmataceae; Wolbachieae; Wolbachia.
REFERENCE   1  (bases 1 to 28030)
  AUTHORS   Fiutek,N., Couger,B.M., Roy,S., de la Torre,J.R. and Connor,E.F.
  TITLE     Genomic assessment of the contribution of the Wolbachia
            endosymbiont of Eurosta solidaginis to gall-induction
  JOURNAL   International Journal Molecular Sciences
REFERENCE   2  (bases 1 to 28030)
  AUTHORS   Fiutek,N., Couger,B.M., Roy,S., de la Torre,J.R. and Connor,E.F.
  TITLE     Direct Submission
  JOURNAL   Submitted (10-FEB-2023) Biology, San Francisco State University,
            1600 Holloway Ave, San Francisco, CA 94132, USA
COMMENT     ##Genome-Assembly-Data-START##
            Assembly Date          :: JUN-2019
            Assembly Method        :: FLYE v. 06-2019
            Genome Representation  :: Full
            Expected Final Version :: Yes
            Genome Coverage        :: 100.0x
            Sequencing Technology  :: Illumina HiSeq; PacBio Sequel
            ##Genome-Assembly-Data-END##
FEATURES             Location/Qualifiers
     source          1..28030
                     /organism="Wolbachia pipientis"
                     /mol_type="genomic DNA"
                     /submitter_seqid="contig_32754"
                     /isolate="wEsol"
                     /isolation_source="galls on Solidago altissima"
                     /host="Eurosta solidaginis MN-2018-EFC"
                     /db_xref="taxon:955"
                     /environmental_sample
                     /country="USA: Cowling Arboretum, Carleton College,
                     Northfield, MN"
                     /lat_lon="44.48 N 93.13 W"
                     /altitude="280 m"
                     /collection_date="2018-11"
                     /metagenome_source="insect metagenome"
                     /note="metagenomic"
     gene            <1..300
                     /locus_tag="wEsol_00397"
     CDS             <1..300
                     /locus_tag="wEsol_00397"
```

```
                     /codon_start=1
                     /transl_table=11
                     /product="hypothetical protein"
                     /pfam="Pfam-B_208 Sigma70_r4"
                     /tigr="TIGR02393 RpoD_Cterm 238 RNA polymerase sigma
                     factor RpoD"
                     /translation="DDSSTFGDCIEDKRVSRPEDAAILADLRGITTNVLATLTPKEER
                     ILRMRFGLGKDGKEHTLEEVGKIFNVTRERIRQIEAKALRKLKHPSRARKLRGFF"
                     /cog="RpoD COG0568 342 DNA-directed RNA polymerase, sigma
                     subunit (sigma70/sigma32)"
                     /besthit="qcoverage 100 , hcoverage 54.0983606557377 ,
                     similarity 100 , identity 100 , evalue 1.94e-59 ,
                     alnlength 99 , Bacteria , Proteobacteria , EAL60191.1 RNA
                     polymerase sigma factor RpoD [Wolbachia endosymbiont of
                     Drosophila simulans]"
     gene            441..1421
                     /locus_tag="wEsol_00398"
     CDS             441..1421
                     /locus_tag="wEsol_00398"
                     /codon_start=1
                     /transl_table=11
                     /fullproduct="qcoverage 100 , hcoverage 100 , similarity
                     100 , identity 100 , evalue 9.56e-238 , alnlength 326 ,
                     Bacteria , Proteobacteria , WP_010082050.1 MULTISPECIES:
                     SGNH/GDSL hydrolase family protein [Wolbachia]"
                     /cog="COG3240 COG3240 370
                     Phospholipase/lecithinase/hemolysin"
                     /pfam="Lipase_GDSL Lipase_GDSL_2"
                     /product="SGNH/GDSL
                     hydrolase family protein"
                     /translation="MTNKNDNSNSKYRNFYVLGDSLSDNGAIIGILNNLSFAKSVKFD
                     DPFYQGRSFSNGPTAVEYIAKYLDLDEFKPGWSYSFFGRCHEQQGQNYAVSYATASEI
                     FDPIFSYFFNKFRLANQLDAVIKHHPDIGKEDLFCIIIGGNDVMVATAYDDTKAEEVL
                     EQAVSEICNALKVLNEHGVKHVVVANAPEVGSIPAFNRDEEARELATKLTESFNAKLA
                     KGLDYMKKSTNLEIKAFDLNSKMRSMFSEYKSKGLNCQDACTSNIADQVGRFKENIKI
                     LLKLIFKGELDVHYNPGCSKETLKDYFFFDYFHPTAELHHEVGNEVYELI"
                     /besthit="qcoverage 100 , hcoverage 100 , similarity 100
                     , identity 100 , evalue 9.56e-238 , alnlength 326 ,
                     Bacteria , Proteobacteria , WP_010082050.1 MULTISPECIES:
                     SGNH/GDSL hydrolase family protein [Wolbachia]"
     gene            complement(1597..1980)
                     /locus_tag="wEsol_00399"
     CDS             complement(1597..1980)
                     /locus_tag="wEsol_00399"
                     /codon_start=1
                     /transl_table=11
                     /pfam="DEDD_Tnp_IS110"
                     /fullproduct="qcoverage 100 , hcoverage 100 , similarity
                     100 , identity 100 , evalue 9.12e-81 , alnlength 127 ,
                     Bacteria , Proteobacteria , WP_227738574.1 hypothetical
                     protein [Wolbachia endosymbiont of Drosophila simulans]"
                     /translation="MVLKCTSFNQGGRDYAKSLGITTKTDKIDCSIISRYINNTDMRV
                     TPLTVSNGNIDCLKQKLSRRNQLVEIAKIQKTQIQQVTDTSIIKQIEELLAILRNQIK
                     TLEDEMITFIDQNQELKRKYISITI"
                     /product="hypothetical protein"
                     /besthit="qcoverage 100 , hcoverage 100 , similarity 100
                     , identity 100 , evalue 9.12e-81 , alnlength 127 ,
                     Bacteria , Proteobacteria , WP_227738574.1 hypothetical
                     protein [Wolbachia endosymbiont of Drosophila simulans]"
     gene            complement(1931..2191)
```

/locus_tag="wEsol_00400"
CDS complement(1931..2191)
/locus_tag="wEsol_00400"
/codon_start=1
/transl_table=11
/fullproduct="qcoverage 100 , hcoverage 100 , similarity 100 , identity 100 , evalue 3.32e-57 , alnlength 86 , Bacteria , Proteobacteria , WP_010082049.1 MULTISPECIES: IS110 family transposase [Wolbachia]"
/cog="COG3547 COG3547 303 Transposase and inactivated derivatives"
/pfam="DEDD_Tnp_IS110"
/product="IS110 family transposase"
/translation="MNSSNIIAGIDVSKSKLDLHIHPLEHYKIFENNVQSIDEMLDFLRLHNVTKIGLEATGGYEKLCAYTLLSNGFEVYVIQPRWERLC"
/besthit="qcoverage 100 , hcoverage 100 , similarity 100 , identity 100 , evalue 3.32e-57 , alnlength 86 , Bacteria , Proteobacteria , WP_010082049.1 MULTISPECIES: IS110 family transposase [Wolbachia]"
gene complement(2188..2403)
/locus_tag="wEsol_00401"
CDS complement(2188..2403)
/locus_tag="wEsol_00401"
/codon_start=1
/transl_table=11
/fullproduct="qcoverage 100 , hcoverage 100 , similarity 100 , identity 100 , evalue 4.94e-41 , alnlength 71 , Bacteria , Proteobacteria , WP_006279709.1 MULTISPECIES: hypothetical protein [Wolbachia]"
/translation="MLRTSLYKYQSSDIKDTQRRGTVTPLDYKNRCSARFSSLVYYTESSEQLIELLGSYIRLENAVYVIITEEL"
/product="hypothetical protein"
/besthit="qcoverage 100 , hcoverage 100 , similarity 100 , identity 100 , evalue 4.94e-41 , alnlength 71 , Bacteria , Proteobacteria , WP_006279709.1 MULTISPECIES: hypothetical protein [Wolbachia]"
gene complement(2568..6023)
/locus_tag="wEsol_00402"
CDS complement(2568..6023)
/locus_tag="wEsol_00402"
/codon_start=1
/transl_table=11
/fullproduct="qcoverage 100 , hcoverage 100 , similarity 100 , identity 99.9 , evalue 0.0 , alnlength 1151 , Bacteria , Proteobacteria , WP_015589495.1 MULTISPECIES: carbamoyl-phosphate synthase large subunit [unclassified Wolbachia]"
/pfam="CPSase_L_chain ATP-grasp_4 CPSase_L_D2 ATP-grasp CPSase_L_D3 CPSase_L_chain ATP-grasp_4 RimK CPSase_L_D2 GARS_A ATP-grasp CPSase_L_D2 MGS"
/tigr="TIGR01369 CPSaseII_lrg 1052 carbamoyl-phosphate synthase, large subunit"
/product="
carbamoyl-phosphate synthase large subunit"
/translation="MPKRTDIESILVIGAGPIVIGQACEFDYSGTQSCKVLKSEGYKVILVNSNPATIMTDPEFSDATYIEPVLPEIIEKIIIKERPDAILPTMGGQTALNCAIKLADDGVLDKYNVELIGVNREAIKKAEDRELFRQSMDKIGLKYPKSIIIKNQEQIKKALDYVGLPAIIRSSFTLGGAGSGIAYNKEEFFNIAESALKISPINEVQIDESIIGWKEYEMEVIRDCKDNCIIVCSIENVDPMGVHTGDSITVAPALTLRDAEYQQMRNASIAVLREID

```
                     VSAGGANVQFAVNPKEDGSLVVIEMNPRVSRSSALASKATGYPIAKVVTKLAIGYSLD
                     EIRNDCAPIIPAAFEPVIDYIVTKIPRFEFEKFKGKNCELSTSMKSVGEVMSIGRTFN
                     ESLQKAFRSLETDFTGLDEVFPENIDIDHIKSQLAKLLPNRLLIAADAMRHGISIEEI
                     NSITGYDLWFLQNIQQIILAEQKIKENGLPETAYEMLEMKKMGFSDARLAKLSNRPTS
                     VIPVPSNVIPVRDTGIQPALHAGDRVKQIEEIRKKFGIKQVYKRVDTCAAEFESSTAY
                     MYGCYEGDVENKTECEANISDREKVVILGSGPNRIGQGIEFDYACVHAASAAKEMGYE
                     TIMINCNPETVSTDYDTADRLYFAPLIAEDVLEILSKEQENGTLVGVIVQIGGQTPLK
                     LAKVLNERGFKILGTSFDSIDLAEDRMRFKNLALQLNLKQPENSICHSVEEALTNAEK
                     VGFPLVVRPSYVLGGQSMSIRHDTQSFKEYVLNQTKIFEHGSLLLDKFLVNAVEVDVD
                     AVCDGEKVFIAAVMEHIEEAGIHSGDSTCSIPTNTLSDEVIKEIKLQTERIALALKVK
                     GLINIQFAVQENRPLAEPVCDEKSLGKVQMSTTEYSNVFEERRQALTTKLPSEIELCK
                     RSSDVYILEVNLRASRTVPFISKVINIPIAKLATQVILGKKLNQENKPFNHFAVKAAV
                     FPFTRFAGVDTLLGPEMKSTGEVMGIDSSFEAALAKVHMAAGYKLPTEGAALVSVKDD
                     DKEYILPVVRMLKELSFEIYATKGTASYLNNNGIAAQAVNKVREGRPHIVDMLKDGKI
                     NLVINTSKGVKSVSDSKDIRRTAILQNIAYSTTASGSKALVLAIQYVKNSKLEVKSLQ
                     QIQNG"
                     /besthit="qcoverage 100 , hcoverage 100 , similarity 100
                     , identity 99.9 , evalue 0.0 , alnlength 1151 , Bacteria
                     , Proteobacteria , WP_015589495.1 MULTISPECIES:
                     carbamoyl-phosphate synthase large subunit [unclassified
                     Wolbachia]"
     gene            complement(6468..7223)
                     /locus_tag="wEsol_00403"
     CDS             complement(6468..7223)
                     /locus_tag="wEsol_00403"
                     /codon_start=1
                     /transl_table=11
                     /fullproduct="qcoverage 100 , hcoverage 100 , similarity
                     100 , identity 100 , evalue 1.19e-173 , alnlength 251 ,
                     Bacteria , Proteobacteria , WP_174516753.1 MULTISPECIES:
                     SDR family oxidoreductase [unclassified Wolbachia]"
                     /cog="FabG COG1028 251 Dehydrogenases with different
                     specificities (related to short-chain alcohol
                     dehydrogenases)"
                     /pfam="ELFV_dehydrog Shikimate_DH Eno-Rase_NADH_b
                     Pfam-B_3397 adh_short KR Epimerase NAD_binding_10 NmrA
                     Polysacc_synt_2 adh_short_C2"
                     /tigr="TIGR01963 PHB_DH 255 3-hydroxybutyrate
                     dehydrogenase"
                     /product="SDR family
                     oxidoreductase"
                     /translation="MDLAKEKEMGKLEGKVALITGASGGIGSAVAKRFIREGACVILI
                     SRSLDNLKPLYNEIEELEEFKEGSVKLIQLDLLDFENVKILTNMIESLKLSESGALDI
                     LVVCAGILGKLNPIHDCEIEELQNVMNTNFTANWYLLKNLDPMLKKSNAGRAIFMTSE
                     VTLSPSSYPYWMPYAASKVALEIMVQIYASETKHTKLCVNAVYTEGPVGSEQAFPGSE
                     LVSPDKLTDKFVELASEDCGISGKILPLSKSPE"
                     /besthit="qcoverage 100 , hcoverage 100 , similarity 100
                     , identity 100 , evalue 1.19e-173 , alnlength 251 ,
                     Bacteria , Proteobacteria , WP_174516753.1 MULTISPECIES:
                     SDR family oxidoreductase [unclassified Wolbachia]"
     gene            complement(7210..8025)
                     /locus_tag="wEsol_00404"
     CDS             complement(7210..8025)
                     /locus_tag="wEsol_00404"
                     /codon_start=1
                     /transl_table=11
                     /fullproduct="qcoverage 100 , hcoverage 100 , similarity
                     100 , identity 100 , evalue 6.56e-181 , alnlength 271 ,
                     Bacteria , Proteobacteria , WP_015589493.1 MULTISPECIES:
                     YihY/virulence factor BrkB family protein [unclassified
```

Wolbachia]"
/cog="Rbn COG1295 303 Ribonuclease BN family enzyme"
/pfam="Virul_fac_BrkB"
/tigr="TIGR00765 yihY_not_rbn 259 YihY family inner membrane protein"
/product="YihY/virulence factor BrkB family protein"
/translation="MLQKFYSIVYCLYRALIDTIHNDGVEHAGYLSFLILLSIFPFLI
VLMAVASTFANFLDQYNIGWVFIIDNMPQDILSSLMPRIREIISGPPQSLLTLAIVGA
VWTASSTIEGLRTILNKAYKVPVSPPYILRRVLSILQFLVITLIITLTIVFSTLVPML
IDFSYQVLSYTRYLLIEFVLFTVVSWLYFMLPNIKQNLSDVFPGSCVAVILWTISASA
FKQYLKASFDQLDLIYGSLGGVVVSLLFFYMLSLIFIYGAKFNFQLKYFNGSR"
/besthit="qcoverage 100 , hcoverage 100 , similarity 100 , identity 100 , evalue 6.56e-181 , alnlength 271 , Bacteria , Proteobacteria , WP_015589493.1 MULTISPECIES: YihY/virulence factor BrkB family protein [unclassified Wolbachia]"

gene 8161..8481
/locus_tag="wEsol_00405"

CDS 8161..8481
/locus_tag="wEsol_00405"
/codon_start=1
/transl_table=11
/pfam="TrbC"
/fullproduct="qcoverage 100 , hcoverage 100 , similarity 100 , identity 100 , evalue 9.97e-66 , alnlength 106 , Bacteria , Proteobacteria , WP_174516754.1 TrbC/VirB2 family protein [Wolbachia endosymbiont of Cardiocondyla obscurior]"
/translation="MKNFLLLVALILSFAFDANAANTFVDTSDTTSSVICKIIGYTHG
IGGPMITIVIIGAALLAIFGRMPWPALFALGAFTAVFFGAPVVVSKITPGGVICKDGA
VADK"
/product="TrbC/VirB2 family protein"
/besthit="qcoverage 100 , hcoverage 100 , similarity 100 , identity 100 , evalue 9.97e-66 , alnlength 106 , Bacteria , Proteobacteria , WP_174516754.1 TrbC/VirB2 family protein [Wolbachia endosymbiont of Cardiocondyla obscurior]"

gene complement(9291..10370)
/locus_tag="wEsol_00406"

CDS complement(9291..10370)
/locus_tag="wEsol_00406"
/codon_start=1
/transl_table=11
/fullproduct="qcoverage 100 , hcoverage 100 , similarity 100 , identity 100 , evalue 3.45e-259 , alnlength 359 , Bacteria , Proteobacteria , WP_015589490.1 MULTISPECIES: DNA replication/repair protein RecF [unclassified Wolbachia]"
/cog="RecF COG1195 363 Recombinational DNA repair ATPase (RecF pathway)"
/pfam="SMC_N AAA_15 AAA_23 AAA_29 Pfam-B_17122 AAA_21 AAA_15"
/tigr="TIGR00611 recf 365 DNA replication and repair protein RecF"
/product="DNA replication/repair protein RecF"
/translation="MATHCYIKKLKLHNFRSHSNFELDSDDSSVVITGENGIGKTNIL
EAISLLAKSNGMKKAKASEIQNRFSNEDWVVHYDFFNGTDFNSIGIAKSFDKKLIQID
GKTQSSYSSLYKISNVIWLIPQMDYVLLNSPSDRLKFLDRIVSLFEENYTCCYMKYRK
AKHERSKLLRENILNKNWFSSLENIMAVNAVSILHMRSSVLKILQDTIDNHSSKLFPK

```
                     ASLKFSSQLTLDDTAEYFQNRLKENREKDSLTGRVTLGVHNDNFRVFCQKRNVPINLC
                     STGEQKLLLLSIILSSVKARCIHYNKAPLLLLDDIMSHLDKHYRKALMEEVLSIQCQT
                     WITDVNQDNFNSYLYSFKFFELSNE"
                     /besthit="qcoverage 100 , hcoverage 100 , similarity 100
                     , identity 100 , evalue 3.45e-259 , alnlength 359 ,
                     Bacteria , Proteobacteria , WP_015589490.1 MULTISPECIES:
                     DNA replication/repair protein RecF [unclassified
                     Wolbachia]"
     gene            complement(10387..10854)
                     /locus_tag="wEsol_00407"
     CDS             complement(10387..10854)
                     /locus_tag="wEsol_00407"
                     /codon_start=1
                     /transl_table=11
                     /fullproduct="qcoverage 100 , hcoverage 100 , similarity
                     100 , identity 100 , evalue 1.30e-107 , alnlength 155 ,
                     Bacteria , Proteobacteria , WP_015589489.1 MULTISPECIES:
                     thioredoxin-dependent thiol peroxidase [unclassified
                     Wolbachia]"
                     /cog="Bcp COG1225 157 Peroxiredoxin"
                     /pfam="AhpC-TSA Redoxin DUF899"
                     /tigr="TIGR03137 AhpC 187 peroxiredoxin"
                     /product="
                     thioredoxin-dependent thiol peroxidase"
                     /translation="MELVVGNNAPDFSLPTDSGENLSLSEFFDKKNVALYFYPKDDTP
                     GCTMEAKGFRDKIDDFSSLDTVIIGVSKDSVKCHTNFKAKYSLPFSLVSDENAEMLGK
                     YGVWVEKSMFGKKYMGIERTTFLIDKKGKIVKIWKNVKVSGHVDEVLEEVKRI"
                     /besthit="qcoverage 100 , hcoverage 100 , similarity 100
                     , identity 100 , evalue 1.30e-107 , alnlength 155 ,
                     Bacteria , Proteobacteria , WP_015589489.1 MULTISPECIES:
                     thioredoxin-dependent thiol peroxidase [unclassified
                     Wolbachia]"
     gene            complement(11039..12382)
                     /locus_tag="wEsol_00408"
     CDS             complement(11039..12382)
                     /locus_tag="wEsol_00408"
                     /codon_start=1
                     /transl_table=11
                     /fullproduct="qcoverage 100 , hcoverage 94.1052631578947
                     , similarity 99.8 , identity 99.8 , evalue 1.24e-308 ,
                     alnlength 447 , Bacteria , Proteobacteria ,
                     WP_015589488.1 MULTISPECIES: HAMP domain-containing
                     histidine kinase [unclassified Wolbachia]"
                     /cog="BaeS COG0642 336 Signal transduction histidine
                     kinase"
                     /pfam="NrfD Pfam-B_6312 HisKA Pfam-B_15665 HATPase_c"
                     /product="HAMP
                     domain-containing histidine kinase"
                     /translation="MILFSVIYCNYLLRSNFLSSYRDSSVNLKSVLENGIIKKYHYLL
                     IEKHHIKYKNFGYINQLVKLRAELLQSVSKVKDFSLILYDQNGRIIFSNFNTQDYDYE
                     QLLTNDEIDKLLSNQEILYTTGNTLTSIFPIFHENSAKPSFFLKILRSYNNSYVIAYG
                     LFLIFLGLLLIILILIMFYLHFSNTKMLAKQHKTNIELQQIKEALEQENANKLKFFAS
                     VTHELRTPLNAIIGFAELIKNETLGSMDNSQYKEYADDIYNAGTHLLALINDVLDFSK
                     AESSSLTVEKVKFNLNKIIDSCVNMLLPKLKEAGINLKKEMTNKQLLVIADSKRMKQV
                     IINLLSNSIKFTPKDGLIRMVIKENIEKNLLTIEFHDNGIGIMQQDIYKVMSVFGQAD
                     SGYRNEGTGIGLPLSKKLVELMGGTFDIKSEAKLGTTITLNFFYEEQTCEDSIDF"
                     /besthit="qcoverage 100 , hcoverage 94.1052631578947 ,
                     similarity 99.8 , identity 99.8 , evalue 1.24e-308 ,
                     alnlength 447 , Bacteria , Proteobacteria ,
                     WP_015589488.1 MULTISPECIES: HAMP domain-containing
```

```
                histidine kinase [unclassified Wolbachia]"
gene            12532..13707
                /locus_tag="wEsol_00409"
CDS             12532..13707
                /locus_tag="wEsol_00409"
                /codon_start=1
                /transl_table=11
                /fullproduct="qcoverage 100 , hcoverage 100 , similarity
                100 , identity 99.7 , evalue 1.26e-284 , alnlength 391 ,
                Bacteria , Proteobacteria , WP_015589487.1 MULTISPECIES:
                aspartate aminotransferase family protein [unclassified
                Wolbachia]"
                /cog="ArgD COG4992 404 Ornithine/acetylornithine
                aminotransferase"
                /pfam="Aminotran_3 Pfam-B_11691"
                /tigr="TIGR00707 argD 381 transaminase,
                acetylornithine/succinylornithine family"
                /product="aspartate
                aminotransferase family protein"
                /translation="MTISPILPVYSPININFSYGKDIYLYDIDGKRYVDFHSGIAVSS
                LGHTNSRLISALKLQGEKLWHISNTYNIPAANNFAEKLINNSFADTVFFSNSGSEAVE
                CGLKIARVYQNGKGNKNRHRILTFHGAFHGRTFLTCAANDKQKFSELLSPCIDWCDNI
                EPNIESVKKAISSDIGVILIEPIQGQGGIKVMNEVFMKELRKLCDENDILLFFDCVQC
                GAGRTGKLFAYEHIGVEPDICALAKGIGGGFPLGACLATENAAKYMAVGMHGSTFGGN
                PLATSVGNAVFEELLSPGFLKNVEIRGKYLKNKLEDLASKFPIIEEVRGKGLMLGIKV
                KADNQKFAEELSHRGLLTVGVTSDNVVRILPPLIITEKEIDKGIEILAQYLSEKSSD"
                /besthit="qcoverage 100 , hcoverage 100 , similarity 100
                , identity 99.7 , evalue 1.26e-284 , alnlength 391 ,
                Bacteria , Proteobacteria , WP_015589487.1 MULTISPECIES:
                aspartate aminotransferase family protein [unclassified
                Wolbachia]"
gene            13708..14229
                /locus_tag="wEsol_00410"
CDS             13708..14229
                /locus_tag="wEsol_00410"
                /codon_start=1
                /transl_table=11
                /fullproduct="qcoverage 97.1098265895954 , hcoverage 100
                , similarity 100 , identity 100 , evalue 7.96e-112 ,
                alnlength 168 , Bacteria , Proteobacteria ,
                WP_015589486.1 MULTISPECIES: ribosome maturation factor
                RimM [unclassified Wolbachia]"
                /cog="RimM COG0806 174 RimM protein, required for 16S
                rRNA processing"
                /pfam="RimM PRC"
                /tigr="TIGR02273 16S_RimM 166 16S rRNA processing protein
                RimM"
                /product="ribosome
                maturation factor RimM"
                /translation="MLDINMNDNLVCLGIITSPHGIKGAVKLKTFTEKPKNISLYGEL
                ISGDENYKIDSVSIIGDNLVIATISGVNSRNEAELLRNKKLYIERSKLPELNDEDEFY
                QSDLVDMEVRLESNELYGNVKSVYNFGSGDILEILVISTKKRIMLSFTKEIFPHINIK
                GRYIVLNIPEFID"
                /besthit="qcoverage 97.1098265895954 , hcoverage 100 ,
                similarity 100 , identity 100 , evalue 7.96e-112 ,
                alnlength 168 , Bacteria , Proteobacteria ,
                WP_015589486.1 MULTISPECIES: ribosome maturation factor
                RimM [unclassified Wolbachia]"
gene            14292..14858
                /locus_tag="wEsol_00411"
```

```
CDS             14292..14858
                /locus_tag="wEsol_00411"
                /codon_start=1
                /transl_table=11
                /fullproduct="qcoverage 100 , hcoverage 100 , similarity
                100 , identity 100 , evalue 7.39e-130 , alnlength 188 ,
                Bacteria , Proteobacteria , WP_012673415.1 MULTISPECIES:
                elongation factor P [Wolbachia]"
                /pfam="EFP_N EFP Elong-fact-P_C"
                /tigr="TIGR00038 efp 184 translation elongation factor P"
                /product="elongation factor
                P"
                /translation="MAERANDIRPGQVLEHNGGLFLVVGIMHTQPGKGGAYIQAEMKN
                IKTGAKHYERFRSDATIRRAILDEEEYVYLFTEGNIVNLMHPSNYEQITINLDLLGEK
                KIYLQDNMKIKVVAYQDKIISAHVPDYVTLAVKETESVIKGQTATASYKPAILENGMR
                VNVPQFIKEEDKIVVYTPDDSYYERVKE"
                /besthit="qcoverage 100 , hcoverage 100 , similarity 100
                , identity 100 , evalue 7.39e-130 , alnlength 188 ,
                Bacteria , Proteobacteria , WP_012673415.1 MULTISPECIES:
                elongation factor P [Wolbachia]"
gene            14867..15565
                /locus_tag="wEsol_00412"
CDS             14867..15565
                /locus_tag="wEsol_00412"
                /codon_start=1
                /transl_table=11
                /fullproduct="qcoverage 100 , hcoverage 100 , similarity
                100 , identity 100 , evalue 3.55e-161 , alnlength 232 ,
                Bacteria , Proteobacteria , WP_015589485.1 MULTISPECIES:
                inositol monophosphatase family protein [unclassified
                Wolbachia]"
                /cog="SuhB COG0483 260 Archaeal
                fructose-1,6-bisphosphatase and related enzymes of
                inositol monophosphatase family"
                /pfam="Inositol_P"
                /tigr="TIGR02067 his_9_HisN 252 histidinol-phosphatase"
                /product="inositol
                monophosphatase family protein"
                /translation="MSISSPRINVMLDSVRSASRQLMRDFNELQISNVKSADFINKTY
                SKSKQTIYDCLHDYKRDYGFISEDDTDQEVKDDTYTWFIMPIEGRENFSSCMVYFAVS
                VCLIHKNKVVAAVIDAPALRETFWAEEKKGAFLEDFRSRHIKMRVKSREGGLIDVSGN
                LLNKLLPGNSSVRSIGSTILGFAYLAAGRHRGIVYSRVNKYKTLLGRLFLQESGGRLT
                EDNGLIIAGDIKLG"
                /besthit="qcoverage 100 , hcoverage 100 , similarity 100
                , identity 100 , evalue 3.55e-161 , alnlength 232 ,
                Bacteria , Proteobacteria , WP_015589485.1 MULTISPECIES:
                inositol monophosphatase family protein [unclassified
                Wolbachia]"
gene            complement(15557..16759)
                /locus_tag="wEsol_00413"
CDS             complement(15557..16759)
                /locus_tag="wEsol_00413"
                /codon_start=1
                /transl_table=11
                /fullproduct="qcoverage 100 , hcoverage 100 , similarity
                100 , identity 100 , evalue 1.47e-283 , alnlength 400 ,
                Bacteria , Proteobacteria , WP_174516755.1
                5-aminolevulinate synthase [Wolbachia endosymbiont of
                Cardiocondyla obscurior]"
                /cog="BioF COG0156 388 7-keto-8-aminopelargonate
```

synthetase and related enzymes"
/pfam="Aminotran_1_2 DegT_DnrJ_EryC1 Beta_elim_lyase Cys_Met_Meta_PP Aminotran_5 Pfam-B_8788"
/tigr="TIGR01821 5aminolev_synth 402 5-aminolevulinic acid synthase"
/product="5-aminolevulinate synthase"
/translation="MVNYEEIFLNKIKDIKDEGRYREFTHFASLPGRLPHIMDYERNR
EVIVWCSNNYLGMSQNESVIAAIQNSSVGAGGTRNISGTTKEVVELEKSLACLHKKEA
ALTFACGYLANQTTLSTLSSVIPGVVIFSDEKNHSSMIEGIKSGKRPKHIFKHNDVNH
LEQLLKSIDKKTPKIIVLESVYSMDGDVAPLKEICDLADQHNAITYLDEVHAVGMYGS
HGGGIAEREGLMDRITVIQGTLSKAFGVMGGYIASSKSLVDVIRSSAPGFIFTTAMSP
VLAAAAKASVEHLKSSNIEREKQKQVVEKVKNSLRNAGINFILTETHIIPIIIGDPEL
SKKASKLLFDEYGIYVQHINYPTVPRGTERFRITPTPYHTDEMIEHLTESLVKVLEKL
SISVACLS"
/besthit="qcoverage 100 , hcoverage 100 , similarity 100 , identity 100 , evalue 1.47e-283 , alnlength 400 , Bacteria , Proteobacteria , WP_174516755.1 5-aminolevulinate synthase [Wolbachia endosymbiont of Cardiocondyla obscurior]"

gene complement(16824..20426)
/locus_tag="wEsol_00414"

CDS complement(16824..20426)
/locus_tag="wEsol_00414"
/codon_start=1
/transl_table=11
/pfam="Pfam-B_18011 Pfam-B_17474 Pfam-B_1200 Pfam-B_13444 Pfam-B_3281 Pfam-B_1200"
/fullproduct="qcoverage 100.083333333333 , hcoverage 103.267411865864 , similarity 96.3 , identity 96.3 , evalue 0.0 , alnlength 1201 , Bacteria , Proteobacteria , WP_197907087.1 hypothetical protein [Wolbachia endosymbiont of Kradibia gibbosae]"
/translation="MFGSNFRDFVSQDTTSQGQSNSERLIKSWHGFEKPDSEDSGIEE
EFEVLDSESQEIPATPCSTSPEDLSLENAILANFPDGCDRSFTVELKDEDSYGIIPKN
LTFAEIINILNQAIQEDNCEELDKVVQNIEDYEGRSITLDNENLKDFCSNQLNLELQN
RSNHQHIYSNVLSQYKFDQEGFKYDYSVKELVLLAIAANDKLVEQYQDFLFAEENLSL
LPLLVGNQKYIEAFIEKFHGNDGVYGRLKGQAIQLVEPNKIDFFSATALRKEQELLNA
VFVEYVLRDFGQVLKTSKPDPLYGVLETASLIKNEEFITIVLDAFERYINGLTKKAST
EELLKKSFATLLETAISQEHISVIEYLCKRYTNSADHAIYDVYKQAFTDDKVITILRQ
QILKNIGARGRKEIYDLILKTALDAGNIAFIEHLCEKCAKCSNDVIGYLNQKFKNNEF
DNIHESILIALSNVEYTNTDVIVEVLFYTKEIHQNVSHNEQVIRPVIQNILKNATHYS
IGQEDYSLVKEVCYLCTEIDAINIIFREECKIFKSSIEKRIEDLEKENKLLQSEALRE
YNSRLPGIVNGLVQTACIVYDEYTKDPKDNKRQKNIDLIFSLMQIRNILQFSIDVQKY
SCHERQDLWDEMCQTPGTFIIRKRSNNVADELEIPVITSDILSENPGLESTGSQQPQE
NVDRRENIMNNEEASIEEAIDNDYRGGKMSRDKKSTKQKMSRNKNSTEQKLLNTIYIP
KTMESYLKRNNIKDEPILKSIIDKVNAEIQKKKEEEKNIINPGPTKKQRCIDQRNTLL
KTRDFLEKKIKSIGTDAKAAVSSVTDVSATTSSHEAPVNIDDEKKQAHAQPAEHVPEE
SAKLKAQPSNTQEQQKVSETPVPEQTDDIESLSTQDALVLQSKSATVLSIDGEDKQTP
VQPVESESKGDQQGSPPLSPQSSASGDVEGTEASANAAVTTPEEARSLVSDGEHDKIV
SKEHQDDALEKDLIDFSSDDGNSPLGDPVPTSTNEIPGQEDGTQTTTRQQEPNPIPTP
TPVPTPNLTPTPNPIPTPTPAPTPGLTPDLTQDPILTPTLVPTPNLTPTPNPVPTPEP
VPSVANNKSEQVNDTQTNGGLPNNSGKNNAIPQNTKSSKLPVIAASALAIAGIISGVA
VAVYLEMLAVGIAVGACCLVAAAVIYCCTPKSQVENNKVDKPFNTKEASTTVSV"
/product="hypothetical protein"
/besthit="qcoverage 100.083333333333 , hcoverage 103.267411865864 , similarity 96.3 , identity 96.3 , evalue 0.0 , alnlength 1201 , Bacteria , Proteobacteria , WP_197907087.1 hypothetical protein [Wolbachia endosymbiont of Kradibia gibbosae]"

gene complement(20465..22360)

```
                     /locus_tag="wEsol_00415"
     CDS             complement(20465..22360)
                     /locus_tag="wEsol_00415"
                     /codon_start=1
                     /transl_table=11
                     /fullproduct="qcoverage 100 , hcoverage 100 , similarity
                     100 , identity 100 , evalue 0.0 , alnlength 631 ,
                     Bacteria , Proteobacteria , WP_141456863.1 MULTISPECIES:
                     molecular chaperone HtpG [unclassified Wolbachia]"
                     /cog="HtpG COG0326 623 Molecular chaperone, HSP90 family"
                     /pfam="HATPase_c_3 HATPase_c HSP90"
                     /product="molecular
                     chaperone HtpG"
                     /translation="MHNVQETENLKFDAEVGKVLNIVIHSLYTNKDIFLRELISNASD
                     ACDKLRYESQLNPNLLDLSDELKITISSNKDKNELYITDNGIGMNRQDLIDNLGTIAS
                     SGTQKFLDAIKNSKDSSQTVELIGKFGVGFYSSFMVASEVIVESRKAGEEESWIWQSK
                     GDGEYSISKSDNQVPRGTKITLIMHPEENEFLDKFRVENIVTTYSDHINFPVEFIDEE
                     GKSEKLNSKAAIWTKPKNDVTQEEHNDFFRSVAHVGGEPWMILHNKNEGAIEYTNLLY
                     VPSIKPFDLFHPDRRCSVKLYVNKVFITEDNVQIIPQYLRFLKGIVDSPDLPLNISRE
                     TLQNNRVVEQIRKSLTKRAISELGKKAKENLEEYTKFWTNFGAVLKEGLCEAMPTDER
                     EALLSICRFNSTGDEKLISIDDYISRMKPEQEHIYYLTGNSLDSVKNSPQLEGFVSKG
                     LEVLLFVDPVDDFWTSVIHEYKDQKIKSVTRADVDLEKFSSEEDKTDEENKSNGEKTE
                     ETILQYFTTVLGDSVKSVKISKKLTDSPVCLAVDEGAMDLRMERFLREQKQLNYRTPK
                     VLEINTKHPVIKSIMKSYAESGENPTLEDMVHLLFYQACIVEGEEMDDASSFAKRLNN
                     LLGKISV"
                     /besthit="qcoverage 100 , hcoverage 100 , similarity 100
                     , identity 100 , evalue 0.0 , alnlength 631 , Bacteria ,
                     Proteobacteria , WP_141456863.1 MULTISPECIES: molecular
                     chaperone HtpG [unclassified Wolbachia]"
     gene            complement(23882..24811)
                     /locus_tag="wEsol_00416"
     CDS             complement(23882..24811)
                     /locus_tag="wEsol_00416"
                     /codon_start=1
                     /transl_table=11
                     /fullproduct="qcoverage 100 , hcoverage 100 , similarity
                     99.7 , identity 99.7 , evalue 6.81e-218 , alnlength 309 ,
                     Bacteria , Proteobacteria , WP_015589482.1 MULTISPECIES:
                     4-hydroxy-3-methylbut-2-enyl diphosphate reductase
                     [unclassified Wolbachia]"
                     /cog="lytB COG0761 294 4-Hydroxy-3-methylbut-2-enyl
                     diphosphate reductase IspH"
                     /pfam="LYTB"
                     /tigr="TIGR00216 ispH_lytB 282
                     4-hydroxy-3-methylbut-2-enyl diphosphate reductase"
                     /product="
                     4-hydroxy-3-methylbut-2-enyl diphosphate reductase"
                     /translation="MEIILAEPRGFCAGVKRAVDILAITLEKYKNERQVYVLHEIVHN
                     KYIVEDFKKQGVVFVSSIEAIEDNEGILIFSAHGVSKNIEDEAKRKGIQVIDATCPLV
                     SKVHKEAKRYENSGKELILIGHENHPEVKGIRGRVNNPITLVQTLQDVYDLKVKDPDN
                     LSYVTQTTLSIDDTHEIIVALKLRFPSITGPDLKDICYATQNRQNAVKKLAEIVDMVL
                     VVGSKNSSNSNRLLDLCTAREKRAYLIDNYRCVNKSWFQGVEKIGITAGASAPDILVD
                     ELMDYLKVNLSVKISVMPGGIIENVQFKIPNLV"
                     /besthit="qcoverage 100 , hcoverage 100 , similarity 99.7
                     , identity 99.7 , evalue 6.81e-218 , alnlength 309 ,
                     Bacteria , Proteobacteria , WP_015589482.1 MULTISPECIES:
                     4-hydroxy-3-methylbut-2-enyl diphosphate reductase
                     [unclassified Wolbachia]"
     gene            24832..26667
                     /locus_tag="wEsol_00417"
```

```
CDS             24832..26667
                /locus_tag="wEsol_00417"
                /codon_start=1
                /transl_table=11
                /fullproduct="qcoverage 100 , hcoverage 100 , similarity
                100 , identity 100 , evalue 0.0 , alnlength 611 ,
                Bacteria , Proteobacteria , WP_141456705.1 MULTISPECIES:
                penicillin-binding protein 2 [unclassified Wolbachia]"
                /cog="FtsI COG0768 599 Cell division protein
                FtsI/penicillin-binding protein 2"
                /pfam="PBP_dimer Transpeptidase"
                /product="penicillin-binding
                protein 2"
                /translation="MQALLKNKLRSLCFIVPLFIFYIIIIFRIFSLTFDQPTTSENFR
                KDNIVHKQPDILDRNGVVIATNVPTTSLYIDATKVKNPDSIAAQLCSTLHDLEYKNLY
                RVLTSEKKFAWIKRHLTPKELLAIKNAGVPGVNFYDDIKRIYPHSNLFSHVLGYTDID
                GNGIAGVEAYISKNKQHSVIQVPDIQLYECCNLKAIPAQQFPTQQIQCVTLGSRYKNT
                DKLRDEQRIPETYVKNNVCDEIAHKTGFQCQCTGMTPDQASAQSSVIRQGWSRSADQY
                VQLSLDTRVQSIVHEELTKAVSRYQALGGVGIVLNVRNSEVISMVSLPDFNPNLQNKA
                EDVQKFNRASLGVYEMGSVLKYFTIAAALDANATKTSDLYDVSTPITIGKYKIQDFHK
                SKIPKITVQDIFVKSSNIGAAKIAVKLGIEKQVEYFKAMKLFSPLKIEIPEKSTPIIP
                DKWSENTLITASYGYGIAVTPIHLAQTAAALINNGIFHNATLMLNKRSIGEQIISRRT
                SREMRKLLRAAVTDGTGRKAKIKAYSIGGKTGSAEKVVDGKYSKDANIASFIGVLTML
                DPRYIVLIAIDEPQGMHHTGGVIAAPIVRNIINRVAPILNVTPEM"
                /besthit="qcoverage 100 , hcoverage 100 , similarity 100
                , identity 100 , evalue 0.0 , alnlength 611 , Bacteria ,
                Proteobacteria , WP_141456705.1 MULTISPECIES:
                penicillin-binding protein 2 [unclassified Wolbachia]"
gene            complement(26670..26978)
                /locus_tag="wEsol_00418"
CDS             complement(26670..26978)
                /locus_tag="wEsol_00418"
                /codon_start=1
                /transl_table=11
                /fullproduct="qcoverage 99.0196078431373 , hcoverage
                99.0196078431373 , similarity 100 , identity 100 , evalue
                6.60e-69 , alnlength 101 , Bacteria , Proteobacteria ,
                WP_039963709.1 MULTISPECIES: hypothetical protein
                [Wolbachia]"
                /translation="MSDLFYIETTCCVDGQCDVYTGEVSFNDVMLGKFSLNSCSGKEA
                MINLDIITFDINIAIDGQNGKTIKKDLKGLEFVTKDDGSWYINKDFGNDKFEIIVKQL
                "
                /product="hypothetical protein"
                /besthit="qcoverage 99.0196078431373 , hcoverage
                99.0196078431373 , similarity 100 , identity 100 , evalue
                6.60e-69 , alnlength 101 , Bacteria , Proteobacteria ,
                WP_039963709.1 MULTISPECIES: hypothetical protein
                [Wolbachia]"
gene            complement(27154..27861)
                /locus_tag="wEsol_00419"
CDS             complement(27154..27861)
                /locus_tag="wEsol_00419"
                /codon_start=1
                /transl_table=11
                /fullproduct="qcoverage 100 , hcoverage 100 , similarity
                100 , identity 100 , evalue 2.59e-160 , alnlength 235 ,
                Bacteria , Proteobacteria , WP_022626165.1 MULTISPECIES:
                isoprenoid biosynthesis glyoxalase ElbB [Wolbachia]"
                /cog="ElbB COG3155 217 Uncharacterized protein involved
                in an early stage of isoprenoid biosynthesis"
```

```
                     /pfam="DJ-1_PfpI"
                     /product="isoprenoid
                     biosynthesis glyoxalase ElbB"
                     /translation="MGEKKLKAAVVLSGCGHLDGAEVREAVLSLLVLDQQEVDVKCFA
                     PDINITQVMNHRTKEATKEKRNVLVEAARIARGEIYDLKEAKAENFDMLVVPGGYGVA
                     KNLSDLAESKDMVTVMPEFERLVSEFFVTKKPIGAICISPAIIVSILSSKIGKEESKV
                     KVTIGDDREQLIERLGGEHIKCDTELSIEDEEHNVFSCSAYMRSDESTYSVYQGIKHM
                     IDSMVKKINKKTKQPFL"
                     /besthit="qcoverage 100 , hcoverage 100 , similarity 100
                     , identity 100 , evalue 2.59e-160 , alnlength 235 ,
                     Bacteria , Proteobacteria , WP_022626165.1 MULTISPECIES:
                     isoprenoid biosynthesis glyoxalase ElbB [Wolbachia]"
ORIGIN
        1 gatgatagta gtacctttgg tgattgtata gaagacaagc gagtctccag gccagaggat
       61 gctgcaatac ttgctgactt gcgtggcatt acaaccaatg ttcttgcaac tttaacacca
      121 aaggaagaaa gaattttaag aatgcgtttt ggccttggta aagatgggaa ggaacatacc
      181 ttagaagaag taggaaaaat ttttaatgta acacgtgaga gaattaggca aatagaggca
      241 aaagcactgc gcaagttgaa acatccaagc cgtgctagaa aacttagagg gttcttttaa
      301 gtatacgtgt caagttaaga agaagctgat tttgcccgga aaaagtggag agcaagttag
      361 gccaaagttg tataatttca ccgctatttt tctttttcta tgaatctaat gaaacaacat
      421 gctaatctac ggtaaatatt atgacaaaca aaaatgacaa ttctaattca aagtatagaa
      481 atttttatgt tctgggtgat agcttatccg ataatggcgc aatcattgga attttaaata
      541 atttatcctt tgcaaaaagc gtaaaatttg atgatccttt ttatcaagga agatctttta
      601 gcaatggccc tactgctgtt gaatatatag caaaatattt agatttagat gaatttaaac
      661 ctgggtggag ttattcattt tttgggagat gccacgaaca gcaaggtcag aattacgcag
      721 tttcatatgc aacagcatct gaaatttttg accctatttt ttcttatttc ttcaataaat
      781 ttcgtttagc taaccagcta gatgcggtaa ttaaacatca tccagatata ggtaaagaag
      841 atttattttg catcataatc ggcggaaatg atgttatggt tgccactgct tatgacgata
      901 ctaaagcgga agaagtgttg gaacaagcag tgagtgaaat atgtaatgca cttaaggttc
      961 taaatgaaca tggtgttaag catgtagttg ttgcaaatgc acctgaagtt ggctcaatac
     1021 cagcttttaa tagagatgaa gaagcaaggg aactagctac aaagctcacg gagagtttca
     1081 atgcaaaact ggctaaaggc ttagattata tgaagaaaag tacaaatctt gaaataaaag
     1141 catttgatct taactctaaa atgagaagta tgtttagcga atacaagagc aaaggtttaa
     1201 attgtcaaga tgcatgcaca tcgaacattg cagaccaagt tgggagattt aaagaaaata
     1261 taaaaatact cttaaagttg atatttaaag gggaacttga cgttcactat aaccctgggt
     1321 gtagcaaaga aacactaaag gattatttct tttttgatta tttccatcca actgcggaac
     1381 ttcatcatga agttggtaat gaagtgtatg agttgatttg acacagtttt tttgaacata
     1441 tccttttaaa tagtaaccag actgcattct gtgattttta gcaaaacacg atctttcatt
     1501 tgagctttct tcaatttaaa ataaatacag aaaattgctg cctatagcta aactgactta
     1561 gatttaccga caatttgaaa aaccgtcatc ccgctactat atagttattg atatatattt
     1621 tcttttaagc tcttggtttt gatcgataaa tgtaatcatt tcatcttcta aagttttaat
     1681 ttgattacgt aaaattgcaa gaagttcctc tatttgtttg attatagatg tatcagttac
     1741 ctgttggatt tgggtttttt gtatttttgc tatttctaca agttggtttc tacgagataa
     1801 tttttgtttc aagcaatcaa tattaccatt actaactgtt aaaggagtaa cacgcatatc
     1861 tgtattatta atataacgtg agatgatact gcagtctatt ttatcagttt ttgtagtaat
     1921 accaaggctt ttagcatagt ctctcccacc ttggttgaat gacgtacact tcaaaaccat
     1981 tgcttagtaa agtataggca cataattttt cgtatccacc agttgcctca agaccaattt
     2041 tggttacatt atgcaaacgt aaaaagtcca gcatttcatc aatggattgt acattgtttt
     2101 caaatatttt ataatgctca agtgggtgaa tgtggagatc taatttactt ttgctaacat
     2161 caatgccagc aataatgttt gatgaattca taattcctcc gtaataataa catatactgc
     2221 attttccagc cttatatacg agcctaaaag ctcaatcagt tgttcggaac tttcagtata
     2281 ataaaccaga gaagagaacc ttgctgaaca acgattctta taatcaaggg gcgtgacggt
     2341 tcctcttctc tgtgtgtctt taatatcact actttgatat ttataaagac ttgttcttaa
     2401 catataaggg aagtgtcctc taggtaagat tgtggagtat tcgttgtagc atttggctgt
     2461 tcaattacgc tagttgattc tttaggtata ctatttcctt caaaatttcc atctttttat
     2521 tatgttctct ttagcatttt gagagtgaat tattctagtt ttgatattta accgttttgt
     2581 atctgctgta atgatttcac ttccaatttg ctatttttta catattgaat tgcaagcact
     2641 aacgctttac tcccagaagc tgtggtactg taagctatat tttgcaaaat agcagttctc
     2701 ctgatatctt tgctatctga aactgatttc acaccttttg aggtattgat cactagattt
     2761 attttccat ctttgagcat atcaactatg tggggtctgc cttctcttac tttatttaca
```

2821 gcttgtgcag caatgccgtt attgttcaga tacgaagctg tgcctttggt ggcatatatt
2881 tcaaaactca gttctttcaa cattcgtaca acaggcaata tatactcttt atcatcatct
2941 tttactgaaa ccagagctgc tccttctgtt ggtaacttgt atcctgcagc catgtgcact
3001 tttgcaagtg cagcttcgaa agatgagtcg attcccatca cttctcctgt tgatttcatt
3061 tcaggaccaa gtaaagtatc cactcctgca aagcgggtaa atggaaaaac agcagcttta
3121 actgcaaaat gattgaaagg cttgttttcc tgattcaatt ttttacccaa aataacctga
3181 gtggcaagct ttgcaatagg aatattgatt accttggaaa taaaaggaac tgtgcgacta
3241 gcccttaaat tcacttcaag tatgtataca tcactagacc tcttgcataa ctcgatttct
3301 gaaggcaatt ttgtcgtcaa agcttgtctg cgctcctcaa atacattcga gtattctgtg
3361 gtgctcatct gcacttttcc taaagatttc tcatcacaaa caggttctgc aagaggtcta
3421 ttctcttgaa cagcaaactg aatgtttatc agacctttca cttttaatgc taatgctatt
3481 ctttcagttt gtagcttaat ctcttttatt acttcgtcac tcaatgtatt tgtcggtatt
3541 gagcatgttg aatcgccaga gtggatacca gcttcttcaa tgtgctccat gaccgctgca
3601 atgaaaactt tttccccatc acatacagca tcaacgtcaa cctcaactgc attgactaaa
3661 aatttatcaa gaagcagcga tccgtgttca aaaattttgg tttgattcaa gacatactct
3721 ttgaagctct gagtatcgtg tctaatcgac atagactgac cgccgaggac atatgatggc
3781 ctgactacta gtggaaaccc cacctttttct gcgttggtta acgcttcttc tactgaatgg
3841 cagatagaat tttcaggttg ttttaaatta agttgcaaag caagattttt aaatctcatg
3901 cgatcttctg caaggtcaat agaatcaaac gaagttccca gaattttgaa acctctctcg
3961 ttaagcactt tggctaactt taaaggtgtt tgaccgccta tttgaactat tacaccaacc
4021 agtgtgccat tttcttgctc tttacttagt atttcaagca catcctctgc aatgagtggt
4081 gcaaaatata aacgatcagc ggtatcgtag tcagttgaga cagtttcggg attacagttg
4141 atcattattg tttcataccc catttctttg gcggcagaag ctgcatgtac gcatgcataa
4201 tcaaactcaa taccttgacc aatgcgattt ggaccacttc ctaaaatgac aacctttttct
4261 cggtcagaaa tattcgcctc acattccgtt ttattttcaa catcaccctc atagcagcca
4321 tacatataag cagtgctcga ttcaaattca gctgcacagg tgtctacgcg tttataaact
4381 tgtttaatgc caaatttttt tcttatttct tcgatttgtt ttactcgatc tccagcgtgt
4441 aacgctggct ggatcccagt gtcacgcact gggatgacat tagagggtac tggaatgaca
4501 gaggtaggtc tattacttaa ctttgctaat cttgcatctg aaaatcccat tttttttcatc
4561 tctaacattt catatgcagt ctcaggaagg ccgttttcct tgattttttg ttcagccaag
4621 atgatttgct gtatattttg cagaaaccat aagtcatatc ctgtaatcga atttatttct
4681 tctatgctaa ttccgtggcg cattgcgtca gcagcaatca gtaatctgtt tggtagcaat
4741 tttgctaatt gagattttat gtgatcaata tcaatgttct caggaaatac ttcatcaagt
4801 cctgtaaaat cagtttcaag tgaacggaaa gctttctgta gtgactcatt aaaagtgcgg
4861 cctatagaca tcacttctcc cactgatttc atggaggttg ataattcaca attcttcccc
4921 ttaaatttct caaactcaaa tcgaggaatt ttagtgacga tataatcaat cactggttcg
4981 aatgctgctg gtattattgg agcgcagtcg ttacgtattt catcgagcga atagccgata
5041 gcaagtttgg ttacaacttt tgcaatagga tagcctgttg ctttcgaagc aagtgcagaa
5101 gagcgagaaa cccttggatt catttcaatt acaacgaggc ttccatcttc tttaggattt
5161 actgcaaact gaacatttgc accgccggca ctaacatcaa tttctctcag cactgctata
5221 gatgcatttc tcatttgttg atattcagca tcacgcagag tcaaagcagg agcaaccgta
5281 atactatctc cggtgtgcac tcccatcgga tcaacatttt ctatcgaaca gactattatg
5341 cagttatctt tacaatcgcg gataacttcc atttcatact ctttccagcc gataattgat
5401 tcatctatct gaacttcatt tattggcgaa attttgagag cactttctgc gatattgaaa
5461 aactcctctt tattatatgc tatgccacta cctgcaccac caagagtgaa tgatgaacga
5521 atgattgctg gtaatccaac gtaatccaaa gcctttttta tttgttcttg atttttttata
5581 atgatacttt tggggtattt cagtcctatt ttatccatgg attggcggaa taactctcta
5641 tcctctgctt tttttattgc ttctctattt accccctatta attctacgtt gtatttgtct
5701 aacactccat catctgcaag ttttattgca caattgagcg ctgtttgccc gcccattgtt
5761 ggcaatattg cgtctggcct ctccttaatt ataattttct ctatgatttc aggcagtacc
5821 ggctcaatat acgtcgcatc agaaaactca ggatctgtca ttatagttgc ggggttggag
5881 ttaaccaaaa tgactttata accttcgctt tttaatactt tacagctttg cgttccggaa
5941 taatcaaact cgcatgcctg acctataact attgggcctg ctcctattac taatatagat
6001 tctatatctg tgcgtttcgg cataattaca ataacggtct ctattcaagt aatgttacca
6061 atctttcctg tatatgtgaa tttttattaa acctattagc taattacttt taggcttgac
6121 gcataactgt acgaacattg caatttgcgt ggcagattcc agtgcttgac acacagctgt
6181 acaaacattc atttttgaag gtaaattgca cagcaaatga tgtcattcaa gtagctgaca
6241 ctggttcctt tatgacggca gtgcccagag gtgtcatccc agtctgggat ccagaaaact
6301 taactttaaa taagtggctg cataataaag actagattcc agtgtcaagc actggaatga
6361 caccatagag gcactgggat gacatccttc ctagtggaga ttactctcaa atcacaatgt

6421 tcatacagtt gtgggcttaa cggtatcttt gcagatacta tggtaattta ttcgggagat
6481 ttgctaagcg gtaagatttt tcctgatatg ccgcagtctt cagaagctaa ttccacaaat
6541 ttatctgtta gtttatcagg tgacaccaat tcagatccag gaaatgcctg ctcactaccc
6601 acaggtcctt ccgtgtatac agcatttaca cataattttg tatgttttgt ctcagatgca
6661 tatatctgca ccattatttc tagtgcaact ttacttgcag catacggcat ccaatatgga
6721 taagaggaag gagaaagtgt cacttctgag gtcatgaata tcgctcttcc agcattagac
6781 ttttttagca ttggatctaa gtttttttagt aagtaccaat tggcagtaaa atttgtattc
6841 attacgttct gtagctcttc aatctcacag tcatgaatgg ggttcagttt gcctaaaatt
6901 ccagcacaca caactaatat atcaagtgct ccagattctg ataatttcag gctttctatc
6961 atgtttgtca gtatctttac attctcaaag tctaaaagat caagctgaat tagtttcaca
7021 gagccttctt taaattcttc aagctcttca atttcattat atagtggctt gagattatca
7081 agagatcttg aaatcagaat cacacatgca ccttctctta taaacctttt tgcaacagca
7141 gagcctattc cacctgaagc tccggtgatt aaagctactt taccttctaa cttacccatt
7201 tctttctcct tagcgagatc cattgaaata ttttagctga aaattgaatt ttgctccata
7261 tataaaaatc aaacttagca tataaaaaaa tagtaatgac actaccacac cacctaaact
7321 tccatatatc aaatccagtt ggtcaaagga agcttttaag tattgcttaa aagctgaagc
7381 agaaatcgtc caaagaataa cagctacaca agatccagga aatacgtctg ataaattttg
7441 ttttatgttt ggtaacataa aatatagcca agagactact gtgaaaagta caaattcaat
7501 aagtaaatac ctagtataac ttagcacctg ataggaaaaa tcaattagca ttggtactag
7561 tgtagaaaac actatagtta gagttataat aagcgtgatt actaaaaatt gtaatatact
7621 tagtactctt cttaatatat aaggcggcga aaccggaact ttataggcct tattgagtat
7681 cgttctaagc ccttcaattg tcgatgaagc ggtccaaaca gcacctacaa ttgctaaagt
7741 tagtaagctt tgcggcgggc ctgatattat ctccctaata cgcggcatca aagatgataa
7801 aatatcctgt ggcatgttat caatgataaa tacccagcca atgttgtact ggtctaaaaa
7861 atttgcgaat gttgacgcca cagccattaa aacgatgaga aaaggaaata ttgataacag
7921 aattaaaaat gataaatacc ctgcatgctc cactccatca ttatgaattg tgtcaatcaa
7981 agcacgataa aggcaataaa cgatgctgta aaatttctgt agcactgtaa tatagacgaa
8041 ccaagaaaag tattctaaat gaaataaagg atacttttaa gcaaaaaatc ctcaccattt
8101 acttaaattt aatcaaagtg aaggataatc cagcattaaa tagaaattat cggaggcgta
8161 atgaaaaatt ttcttctact tgtggcttta attctctcct ttgcatttga tgcaaatgct
8221 gcaaatactt ttgttgatac aagtgataca acatcatcgg tgatatgcaa gataataggc
8281 tatactcacg gcataggcgg accgatgatc acaatagtga taattggtgc agctttgctt
8341 gcgatattcg gcagaatgcc atggccagcg ctttttgcac tcggtgcgtt cactgctgtg
8401 tttttggcg ctcctgtagt cgtttcaaaa ataacaccag gaggtgtgat ttgtaaagac
8461 ggtgcagtag cggataagta atagaaatta ttggaggcac aaacgtagtt ttctttactt
8521 gaaagtggtc aagctgacca ctttcagtgt ctatgaatac ctaataaatt ctctttctgt
8581 aatcatatca gttaaagttt agattccagc gtcacgcgct ggaatgacaa ggcttatcgt
8641 catgccgccg cataccgtca taccgcgatt cattcgcggt atctcatcag ctaacaagca
8701 gcggaataac ggttgtcgtt tagctatgaa tattaagaaa tttaccaaat gaaaaaaaag
8761 gcaaaagaag ccccgtggtt ggctaattac ttacattata ctttcaagta tggcgttttt
8821 ttattctaaa cgcttaataa ccgcgattca gctgctttta agccgcaact aatctaaatt
8881 ataaacatta agaaatttac tgagcgaaaa aaaaggcaaa gaaaccctag ggtggctagc
8941 attcaaattc tcccttgtca atttgacgtt ttttgctatc ttaaacgctt tgtaagcgcg
9001 tttcagctta tataggtaga aacctagaag ttttataaag acatgaggtg cacatagtgc
9061 aaaaattcag catgatttac gccaaataca ttaagttttt tatcattaat ccactgcaga
9121 gattgcgaag ataaataaat agcttcagtt tcatgataag gggactggca aagtttgtaa
9181 agtaactttt gcatttaatt taaagtaaaa aaaacagaaa tgttttggtt tgaggtttaa
9241 ttgccggaat ttacaattgg gagttgcctg tatgagcttt gaaagaggta ctattcattt
9301 gataattcaa aaaacttgaa agaataaaga tagctattga aattatcttg atttacatca
9361 gttatccaag tttgacattg aatacttaac acttcctcca ttaacgcctt tctgtaatgt
9421 ttatccagat gagacattat atcatcaagt agaagaagtg gtgccttatt gtaatgaata
9481 caccttgctt ttacactgga taaaataata gaaagcagta ataacttttg ttctccagtg
9541 gagcacagat ttattggtac gtttcttttt tgacaaaaaa cccggaaatt atcgttatgc
9601 acaccaaggg ttactctacc agtcaatgaa tctttttctc tgttttcctt tagacgattc
9661 tgaaaatatt ctgcagtatc gtccaaagtt agctggctgc tgaatttcaa acttgccttc
9721 ggaaaaagct tactggaatg gttatcaatt gtatcttgta atatttttaa aacagacgat
9781 cgcatatgta aaatactaac tgcattaacg gccattatgt tttcaagact agagaaccag
9841 tttttgttca aaatgttctc cctcaatagt ttacttcgct catgtttagc ttttctatat
9901 ttcatgtagc agcaagtgta attttcttca aacagtgaaa ctatacggtc taaaaatttt
9961 aatctgtcac ttggagaatt aagaagaaca tagtccattt gtggaatcag ccatattaca

10021 ttggatatct tatacagaga tgaataactt gattgcgttt ttccatcaat ttgtattagt
10081 ttcttatcaa aactctttgc gataccaatt gaattaaaat ccgttccatt aaaaaaatca
10141 tagtgcacta cccaatcttc attactgaat ctattttgta tctcactggc ttttgctttt
10201 ttcattccat tgcttttagc aagcaacgag attgcttcaa gtatattagt tttaccaata
10261 ccatttttcgc cagttattac aactgagcta tcatctgaat ccagttcaaa gtttgagtgg
10321 ctacgaaaat tatgtaattt cagctttttt atgtagcaat gggtagccat taacctattc
10381 tttaatttat attctcttta cttcttccag aacctcatca acatgtccac taacttttac
10441 atttttccag atcttcacta ttttaccttt tttatctatt aaaaaagtag tgcgttctat
10501 tcccatatac tttttgccaa acatactttt ctctacccaa acaccatatt ttcccaacat
10561 ttcagcattc tcatcagaaa ctaaagaaaa cggcagagag tattttgctt tgaagttagt
10621 gtggcactta acactatctt ttgatacacc aattatcact gtgtcaaggg aagaaaaatc
10681 atctatttta tctctaaagc cttttgcttc catcgtacaa cccggagtat cgtctttagg
10741 ataaaaataa agagctacat ttttttatc aaaaaattca ctcagtgata aattttcacc
10801 agaatctgtt ggtaaactaa aatcaggtgc attatttcct actactaact ccataccttg
10861 ctccattaat aacgcttcta tctatagtag tatattgtag tcagagtatc aaattttata
10921 tatagctaac acagataaga ttcttgatag gggcttatt gataactgcc ttctcggctt
10981 gccgcttttg ataaagtgtg gttttcgtct gatgtaaggc catatcaaat agatcctatt
11041 aaaaatcaat cgaatcttca catgtttgtt cttcataaaa gaagttcaat gttatcgtag
11101 taccaagttt cgcttcactt ttaatgtcaa aagttccacc cattaactct actaatttct
11161 tgcttagcgg taatccgatg cccgtacctt cgtttctata tcccgaatct gcttggccaa
11221 aaacagacat aactttatat atatcctgct gcattattcc tattccattg tcatgaaact
11281 caatggttaa taagtttttt tctatatttt cttttatcac cattcttatt aaaccatctt
11341 taggagtaaa cttgattgag tttgataata aatttattat aacttgtttc attctcttgg
11401 aatctgcaat gactaataat tgtttattag tcatctcttt ttttaaatta atccctgctt
11461 cttttaattt aggtaatagc atatttacgc atgaatctat tattttgttc aggttaaact
11521 tcactttttc tactgttaaa ctacttgatt cagctttaga aaaatcgagc acatcattaa
11581 ttaaggcaag taagtgtgtc cctgcattat atatatcgtc tgcatactcc ttgtattgag
11641 agttatccat tgaacctaaa gtttcatttt tgatcaactc tgcaaatcca ataatagcat
11701 taagaggtgt gcgtaattca tgtgtaacgc ttgcaaaaaa ctttagcttg tttgcatttt
11761 cttgctctaa tgcctctttt atctgctgta attcaatgtt agttttatgt tgcttggcca
11821 gcattttggt attagaaaag tgcaaataga acattattaa tattaaaatt attaataata
11881 agcctaaaaa tattaaaaat aggccatatg ctataacata agaattattg tagcttcgaa
11941 gaatcttcaa aaaaaacgac ggcttagcgc tattttcatg gaatataggg aaaatagaag
12001 ttaatgtatt tcctgtagta taaagtattt cttggttact taataattta tcaatttcat
12061 cgtttgtaag taactgttca taatcataat cttgagtgtt aaaattactg aatattattc
12121 tgccgttttg atcatacagt attaagctga aatcctttac tttgctcact gattgtaata
12181 attcagcacg cagttttact agttggttta tgtagccaaa atttttgtat ttaatgtgat
12241 gcttttctat tagtaaataa tggtattttt ttattatacc attttctaat acacttttta
12301 agtttacact tgaatcacgg taagaagaaa ggaaattact ccttagtaaa taattacaat
12361 atatcacact gaataaaatc ataaaaattg atgagaataa tgcgataact tttgtgtatt
12421 tgtttttccc agtgcagaaa acattaattt acctatctta tacatatcct tactcattgc
12481 gaaacaacgg cttttatgtt attaataata aaatttagca tataatattt tatgacaatt
12541 tctcctattt tgccagttta ttctcctatt aacataaatt tttcttatgg taaagatatt
12601 tacttgtatg atattgacgg taagcgctac gtagattttc actccggaat agccgttagt
12661 agcttaggtc acactaattc gcgattaatt agtgctctta aattgcaagg agaaaaatta
12721 tggcatatat caaataccta taatatacct gctgccaata attttgcaga aaaattaata
12781 aacaatagtt tcgctgacac tgtatttttc tcaaattctg gatcagaggc agtagaatgt
12841 gggcttaaga ttgctagggt ctatcaaaac ggaaaaggca ataaaaaccg tcacagaatt
12901 ttaacattcc atggtgcatt tcatggaaga accttcctaa cttgtgctgc aaacgataaa
12961 cagaaatttt ctgaattact gagtccctgt attgattggt gtgataatat cgagcccaat
13021 attgagagtg taaagaaagc aatttcaagt gatataggcg ttatattaat agaaccaata
13081 caagggcaag gtggtatcaa agtaatgaat gaagtcttta tgaaagaact aagaaagctg
13141 tgtgatgaaa atgacatatt gttatttttt gattgcgtgc aatgcggtgc tggtaggaca
13201 ggaaaattat ttgcatacga acatatagga gtggaacctg atatatgtgc tcttgcaaag
13261 ggaataggcg gcgggtttcc gctaggggct tgtcttgcga ctgaaaatgc tgctaaatac
13321 atggcagttg gtatgcacgg ttccactttt ggtggcaatc cgcttgcaac ctcagtaggt
13381 aatgctgtgt tcgaagaatt gctcagtcct ggctttttga aaaatgttga aattagaggt
13441 aagtatttaa aaaataaatt agaagactta gcaagtaaat ttccaataat agaagaagtg
13501 agagggaaag ggttaatgct tggaataaaa gtgaaagcag acaatcaaaa atttgcagaa
13561 gagttaagtc atcgtggttt actcactgtt ggagtaacat cagataacgt tgtgagaatt

13621 ttacctccac taattatcac tgaaaaagag attgataaag gaattgagat tctggctcaa
13681 tatttatctg agaaatcttc tgattagatg cttgatatta atatgaacga caatctggta
13741 tgcttaggaa ttattacctc tcctcacgga attaaaggag ctgttaaatt aaaaactttc
13801 accgaaaagc ccaaaaatat ctccttatat ggtgagctaa taagtggtga tgagaattat
13861 aaaatagact cagtatccat cataggtgat aatttagtaa tagctacaat aagtggagta
13921 aattcccgta atgaagcaga gcttttgagg aataagaagc tatatataga aagaagcaag
13981 ttaccagagc taaatgatga ggatgaattt tatcaaagtg accttgtaga tatggaagta
14041 aggctggaaa gtaatgaatt atatggcaat gtaaaatctg tgtataattt tggctcaggg
14101 gatatattag aaattttagt tatcagcaca aaaaaacgta ttatgctgtc tttcactaaa
14161 gagatatttc cacatataaa tataaagggg aggtatatag tattaaatat accagaattt
14221 attgactaat aaagactttg atatttatat aaagctattt ataattataa gatcaaattg
14281 aaaatgtaat tatggcagaa agagctaacg atatcagacc aggtcaggtg ttggaacata
14341 acggcggatt atttttggtt gtaggtatta tgcatactca acctggtaaa ggtggtgcat
14401 acatacaagc cgaaatgaaa aacatcaaaa ccggagcaaa acactatgaa agatttcgtt
14461 ctgatgcaac aatcagaagg gcaattttag atgaagagga atatgtttac cttttcactg
14521 aaggaaacat tgtaaatctt atgcatccaa gtaattacga gcaaatcact ataaatttgg
14581 acttattggg agaaaaaaaa atttatttac aggataacat gaaaattaaa gttgtagctt
14641 atcaagacaa aataatttct gcgcatgtgc ctgattatgt tacacttgct gtgaaagaaa
14701 cagagtctgt tattaaagga caaactgcta ctgcttctta taagcctgca attttggaaa
14761 acggaatgcg cgttaacgtg cctcagttta taaaagaaga ggataaaatt gtagtatata
14821 ctccagatga cagctattat gaaagagtga aagagtaaaa agaagaatgt ccatctcatc
14881 accacgaatt aatgtgatgc ttgattctgt gcgtagtgct tccaggcagc ttatgcgcga
14941 ttttaatgaa ttacaaattt ctaatgttaa atcagcagac tttatcaata aaacttattc
15001 aaaatccaag caaaccatat acgattgctt gcacgactac aaacgagact atgggtttat
15061 ttccgaagat gatactgatc aagaagtaaa agatgacact tatacctggt ttattatgcc
15121 tatagagggc agggagaact tttccagttg tatggtttat tttgcagtat cagtctgtct
15181 cattcataaa aataaagttg tggcggctgt aattgatgct ccggctctta gagaaacttt
15241 ttgggcagaa gaaaagaaag gtgcttttct tgaggacttt agatctcgtc atataaaaat
15301 gcgggtgaaa agccgtgaag gggtctaat agatgtaagt ggtaatttgt taaataaatt
15361 gctgcctggc aatagtagtg tacgctccat cggttcaaca attttaggct ttgcatacct
15421 tgcggcagga aggcacagag gaatagttta ctctagagtt aacaaatata aaaccttgct
15481 cggtagactt tttctacaag aaagcggtgg aaggctaacg gaagacaacg gattgattat
15541 tgctggcgat attaaattag gataaacaag ctacagagat agacaacttt tcgagaactt
15601 ttacaagaga ttctgtcaaa tgttctatca tttcatcggt atgataaggt gtaggggtaa
15661 tacggaaacg ctcagttcct cttggaactg tcgggtaatt tatatgttga acgtatattc
15721 catactcatc aaataataat tttgatgctt ttttggataa ctctgggtcg ccaattatta
15781 ttggaattat atgagtttct gttaaaatga agttaattcc tgcatttctc agtgagtttt
15841 ttactttttc aacgacttgc ttttgctttt ccctttcaat gttgcttgat tttaggtgtt
15901 caacgcttgc ctttgctgcc gctgctaaaa caggcgacat agcagtggta aaaataaatc
15961 ctggagcgga acttcttatt acatctacca agctctttga agacgcgata tacccaccca
16021 tcactccaaa agccttcgat agtgtacctt gaataacggt tattctatcc atcaggcctt
16081 ctctttctgc aattccacca ccatgtgaac catacatacc aactgcgtgc acctcatcca
16141 aataggtaat tgcattatgt tgatctgcca ggtcgcatat ttctttaagc ggagctacat
16201 caccatccat tgaatatacg gattcaagta ctatgatttt tggtgtcttt ttgtctatag
16261 acttaagtag ctgctctaga tgattaacat cattgtgttt aaatatatgt tttggccttt
16321 ttcctgattt tatgccttct atcatcgaag aatggttctt ctcgtctgaa aaaattacta
16381 cgcccggaat aacagacgat aaagtgctaa gtgttgtctg gttagcgagg tagccacaag
16441 caaaagttaa agcagcctct tttttatgca aacaagctaa ggatttttca agttcgacaa
16501 cctcttttgt tgtaccagat atatttcttg ttcctccagc accaacagat gaattttgga
16561 tagcggcaat tacactctca ttttgtgaca ttcccaaata gttattactg caccagacga
16621 ttacttccct atttctttca tagtccataa tgtgggggag cctacctggc aatgacgcaa
16681 agtgcgtaaa ttcacggtag cgcccctcat cttttatatc ttttatttta ttcaagaata
16741 tttcttcgta gtttaccaag tccttattta ccgataatta acataattat aataaattat
16801 taaatggtaa actacaatat ttattataca gatacggtag tagatgcttc cttcgtgttg
16861 aaaggtttgt caaccttgtt gttttcaact tggcttttag gtgtacagca gtatataact
16921 gcagcagcaa ccagacaaca agctccaact gctattccta ccgctaacat ttccaagtaa
16981 actgcaacag ccacccctga aataatacca gctattgcaa gtgcagaagc agctataaca
17041 ggcagcttac tactttttgt attttgtgga atagcattat ttttgcctga attattaggc
17101 aacccgccat tggtttgagt atcattcacc tgctcagatt tgttgttagc tactgaaggc
17161 actggttctg gtgttggcac tggattcggt gttggtgtta gattcggtgt tggcaccagc

17221 gtaggtgtta gaattggatc ttgtgttaga tccggtgtca gtcctggcgt tggcgccggc
17281 gtaggtgttg gaattggatt cggtgttggt gttaggttcg gcgttggcac cggtgtaggt
17341 gttggaattg gattcggttc ttgctgcctg gtggtagttt gagtaccatc ttcttgtcca
17401 ggatttcgt tagttgatgt aggtactgga tctcctagag gactattgcc atcatcactt
17461 gaaaaatcta ttaaatcttt ttctagtgca tcatcttggt gctctttaga cacgatttta
17521 tcatgctcac catcactcac aagactacgt gcttcttcag gcgtagttac tgctgcatta
17581 gcagaggctt ctgtaccttc aacatcacca ctcgcagaac tctgtggtga aagtggtggt
17641 gaaccttgtt gatcaccttt actttcagac tctaccggtt gaacaggagt ttgtttatcc
17701 tcaccatcaa ttgataacac ggtagcactt ttactttgaa gaacaagagc gtcctgtgta
17761 cttaaacttt caatatcatc cgtttgttct ggaactggag tctcagaaac tttttgttgc
17821 tcttgagtat tagaaggctg tgccttaagt tttgcacttt cttctggtac atgttcagca
17881 ggttgagcat gagcttgttt tttctcatca tcaatattaa caggagcttc gtgactactt
17941 gtagtagcgc taacatcagt tactgatgat acagcagcct tagcatcagt tccaatgctt
18001 ttgatctttt tttcaagaaa atctctagtt tttaagagtg tgtttctctg atcaatgcat
18061 ctttgcttct tagtaggacc tggattgata atgttcttct cctcctcctt cttcttttga
18121 atttcagcat ttactttgtc tatgatactc ttcaaaatag gttcatcttt tatattattc
18181 ctttttaagt aactctccat cgtcttagga atgtagatag tattcaacaa tttctgctct
18241 gtgctgtttt tatttctaga cattttctgc tttgtacttt ttttatctct agacatttta
18301 ccccccctat aatcattatc tatagcttct tctattgaag cctcttcatt gttcataata
18361 ttttctcgtc tgtccacatt ttcttgtggt tgttgactgc cagtactttc aagtcctgga
18421 ttttctgata gaatgtcact tgttataact ggaatttcta attcatctgc aacgttatta
18481 cttcttttc gtattataaa agttccagga gtttggcaca tttcatccca caaatcttga
18541 cgttcatgac aggaatattt ttgcacatca atagaaaatt gcagaatatt tctaatttgc
18601 attaaactga atattagatc aatattcttt tgacgtttat tgtcttttgg atctttagtg
18661 tattcatcat acactatgca agcagtctga actaatccat ttacaatacc tggaagacgg
18721 ctgttatatt cacgtaaagc ttcagattgc aataatttgt tttccttttc cagatcttct
18781 atgcgctttt ctatgctaga tttaaaaatc ttacactcct ctcgaaaaat tatgttaatt
18841 gcatcaattt ctgtgcataa gtaacatact tccttgacta aggagtaatc ttcttggcct
18901 attgagtagt gtgttgcatt tttcagtata ttctgtatga ccggcctaat cacttgctca
18961 ttatgtgata cgttttgatg gatttcttta gtgtaaaaaa gcacctcgac tataacgtct
19021 gtgtttgtgt attcaacatt agacaatgca atgaggattg attcgtgtat gttatcaaat
19081 tcgttatttt taaatttttg attgagatag ccgataacat cattgctgca tttagcacat
19141 ttttcacaca agtgctcgat aaatgcaata ttaccagcat ccaatgcagt tttcagaatt
19201 aaatcataaa tttcttttct gccacgagct cctatgtttt tcaggatttg ttgccttagt
19261 attgtaataa ctttatcatc tgtaaatgct tgtttatata catcgtaaat ggcatgatct
19321 gcactattag tgtatcgttt gcacaagtat tcaatgacag atatgtgttc ttgacttatc
19381 gcagtttcta acaaggtagc aaagctcttt tttaataact cttctgttga ggctttttta
19441 gttagcccat taatatatct ttcaaatgca tccagcacta tcgtgatgaa ttcttcattt
19501 ttgatcagag acgctgtttc aagcacacca tacaaaggat caggtttact tgtttttaac
19561 acctgtccaa aatctcttaa aacgtattcg acaaaaactg catttaataa ttcttgctct
19621 tttcttaatg ctgtagctga gaagaagtca atcttattag gttcgacaag ttgtatagct
19681 tgacctttta atcttccgta aactccatcg ttaccatgga atttttctat aaaagcttca
19741 atatattttt gattaccaac aaggagaggt aaaagactta agttttcttc agcaaaaaga
19801 aaatcctgat attgctctac tagcttgtca ttagctgcaa ttgccaatag taccaattct
19861 tttactgaat aatcatactt gaaaccctct tgatcaaatt tatattgtga tagtacattt
19921 gaatatatat gttgatggtt gcttctgttt tgtaactcca aattaagttg atttgaacag
19981 aaatctttga gattttcatt atccagcgta atagacctgc cttcataatc ttcaatattt
20041 tgtacaacct tgtctaattc ttcacaatta tcttcttgta tagcttgatt taagatattt
20101 ataatctcag caaaagttaa gtttttagga atgataccat atgaatcttc atcttttaac
20161 tctacagtaa acgacctatc acaaccatca ggaaagtttg caagtattgc attctctaaa
20221 cttagatctt caggggatgt actacatgga gtagctggta tctcttgact ttcgctatct
20281 aaaacttcaa actcttcttc aatacctgaa tcttcagaat ctggtttttc aaaaccatgc
20341 caacttttga ttaatctttc agaatttgat tggccctgtg aagttgtatc ttgtgataca
20401 aagtctctaa aattggaacc aaacatgttt tacctcctaa ctagcagcta agctaagtat
20461 agcattaaac agaaatcttg ccaagcaaat tattcagtct cttagcaaag ctgctcgcgt
20521 catccatctc ttcgccttcc acgatgcaag cttggtaaaa caataagtga accatgtcct
20581 ccaatgttgg gttttcacca ctttcagcat aagatttcat tatactcttt attacaggat
20641 gcttagtgtt aatttcaagc acctttggcg tgcggtaatt tagctgcttt tgttcacgca
20701 aaaaacgctc catgcgaaga tccatagcac cttcatcaac cgctaggcat acgggactat
20761 cagtcaattt tttggaaatt tttacgcttt ttactgaatc tccaagcact gtggtaaaat

20821 attgcagtat agtttcctca gtttttctc catttgattt attttcttca tctgtcttat
20881 cttcttctga agagaatttt tccaaatcaa catctgcacg ggttacagac ttgattttct
20941 gatctttgta ttcatgaatt acactagtcc aaaagtcatc aactggatca acaaatagga
21001 gcacttctag tcctttactg acgaatccct caagttgcgg actgtttttc actgaatcga
21061 ggctatttcc ggtgagataa tagatatgtt cctgctcagg cttcattctg cttatataat
21121 catcaatgct gatcaacttc tcatcaccgg tgctattaaa tctgcaaatt gagagcagcg
21181 cttctctctc atcagttggc atagcttcgc aaagaccttc ctttaaaact gcaccaaaat
21241 tggtccagaa ttttgtgtac tcctctaaat tctcttttgc ctttttaccg agctctgata
21301 tcgcgcgttt agtgagggat ttcctgattt gctcaacaac acgattattc tgcaacgttt
21361 ctctgctgat attaagaggt aagtccggtg aatcaacaat acctttcaag aaacgtaaat
21421 actgcggtat gatctgtaca ttatcttcag tgataaatac tttatttaca tataacttaa
21481 cagagcaacg tctatctgga tggaataaat caaaaggttt tatagaagga acataaagca
21541 aatttgtata ttctattgca ccttcatttt tattatgcaa tatcatccag ggctccccgc
21601 caacgtgggc aacactgcgg aaaaaatcgt tgtgctcctc ttgagtaacg tcattttttg
21661 gcttagtcca aattgcagcc ttactgttta acttttcact ttttccttct tcgtctataa
21721 attcaacagg aaaatttatg tgatcagagt aagtagtgac aatattttca acacgaaatt
21781 tatctaaaaa ctcgttttct tcaggatgca taatgagcgt aattttggtt ccgcgaggga
21841 cttgattatc tgacttgctg attgaatatt ccccatctcc tttggattgc cagatccaag
21901 actcctcttc tccagctttt ctcgattcta ctattacttc tgatgcaacc ataaagcttg
21961 agtaaaaacc aacaccaaac ttcccaatca gctccacagt ttggcttgaa tccttgctat
22021 tcttaatcgc atccaagaac ttttgcgtac cagagcttgc aattgtacca agattgtcta
22081 ttaaatcctg tctattcatt ccaatgccat tatcagtgat atatagctcg ttcttatctt
22141 tattggagct gatagtgatt tttaattcgt cactcaaatc tagcaaatta gggtttagtt
22201 gagattcata gcgcaactta tcacatgcat ctgatgcatt tgatattaat tcacgcagaa
22261 aaatgtcttt attggtgtaa agtgaatgaa tcactatatt cagtactttc cctacctcag
22321 cgtcaaattt taaattttca gtttcttgta cattatgcat tttgagctcc attatttgcc
22381 tactacccta ataatttaag tattgaatca cgatctttca aggtcacgta caccaaaatt
22441 aatgtgggtg tagttattat gacacccaat ttatgggatc tcttgcataa caaacttttg
22501 gcagcgcatt ggatgaaacg aaaaacttcc ttgacaccct tcgccagccc ccttatcatg
22561 aaactgaagc tatttattta tcttcgcaat ctctgcagtg gattaatgac aaaaaaactt
22621 aatgtatttg gcgtaaatca tgcttaattt tttgcactat gtgcacctca tgtctttatc
22681 aaatttctag ctacacaagc tgaaatacgc tatttaaaac atcaaaaaac gccaacttaa
22741 aaaatggata gtgaataatt agctacccta gggtttcttt gccttttttt ctgcttagta
22801 aatttcttaa cgtttataat ttagattagt tgcggcttaa aagcggctga gtcacggtta
22861 ttaagcgttt agaataaaaa aacgccatac ttgaaagtat aatgtaagta actagccaac
22921 catcgggctt tttttgcctt ttttttcgct tggtaaattt tttaatactt agattagttg
22981 caatttaaga gaccagaaat ggtcagtgct tggcactgga ataacgtcct cctttgttat
23041 cccagtgccc agacactggg atccaggaaa cttaattgca agtaatgcat tagatttggt
23101 gagtatgggt tttatgttat agaatgaaac acttttggtg aatttgtaaa gaaaactgga
23161 tcccagtgtt tgggcactgg gatgacagca gtcctacgtc ataccgccgc gaaccgtcat
23221 accgcgattc attcgcggta tctctagatc ccgctaacaa gcagcgggat gacgattgtc
23281 gtttagccat aaacattaag aaatttacca aacgaaaaaa aaggcaaaag aagccctggg
23341 gttattatcc gctttaaaat attggcgttt tttatgtttt aaacgcttga caagcgaaat
23401 tcagctgctt ttaattgcaa ctaacttacg ctgcaaatgt ttaagaagtt tactaagcag
23461 aaaaaaagac aaagaatccc cgagttagct agtcttttac tatctctgtc gagtattagc
23521 atttttgat gtcttgtaac gctttataag cgcgttcagc ttatttagat aaaaatctag
23581 atgtagatga agttttgtaa agacatacag tatctatata ctgcaaaaaa ttgaacataa
23641 gacgccgata cattaagtaa tccttacctt ttaatctgca gattggcgaa agcaaataca
23701 atagcttcac tatcatgata agtgggctgg cggagtttgt caaggaagtt tttttgtttc
23761 tattcccaat aaaagtttgt tatatggtta tgcaagaagt ctaatgaatc gcggcatgac
23821 gtcttagcta tagatgctgg tctgtgtgat ttcaagagaa gcttagtaga taagttgtgt
23881 tttaaaccag attcggtatt ttaaactgaa cattttcaat aataccacct ggcataactg
23941 aaatttttac actcaggttt acttttaggt agtccattaa ttcatcaact aatatgtcag
24001 gagcggaagc acctgcagtg attcctatct tttctacgcc ctggaaccag cttttattta
24061 cacatctgta attatcaatc aagtaggctc ttttttctct agcagtgcac agatctaata
24121 agcggtttga atttgagcta tttttacttc ctacaactaa taccatgtct acaatttcag
24181 ctagcttttt aacagcattt tgtctgtttt gcgttgcata gcatatgtcc tttaaatctg
24241 gacctgttat gcttggaaac ctgagcttca gggcaacaat aatttcatgg gtatcgtcga
24301 tgcttaacgt ggtttgtgta acataagaca aattatctgg atctttaacc tttaaatcat
24361 atacatcctg caaagtttgc actaaagtga taggattgtt tactcttccc ctaattccct

24421 taacttctgg atggttttca tgcccaatga gaattaattc tttaccactg ttttcatacc
24481 tttttgcttc tttatgcact ttgctaacta aaggacatgt tgcatcaatc acttgaatac
24541 cctttctttt tgcctcatct tctatatttt ttgatactcc gtgcgcgctg aagatcagta
24601 ttccttcatt atcttcaatt gcctcgatac tgcttacaaa aaccactcct tgtttcttaa
24661 agtcctctac tatatattta ttgtgaacaa tttcgtgtag cacataaact tggcgctcgt
24721 ttttatattt ttctaaagta atagctaata tgtccacagc tctttttacc ccggcacaaa
24781 aacctcgtgg ttcagctaag atgatttcca tttttgtcaa atcaagtaaa tatgcaagca
24841 ttacttaaaa ataagctccg ctcactgtgt ttcatagtac cattatttat attttatata
24901 ataattattt ttcgcatatt ctctttaaca tttgatcaac ctactacttc agaaaatttt
24961 agaaaagata atatagtaca taaacaacct gatattttag atagaaatgg agtggtaata
25021 gcaacaaatg tgcccacaac atcactatat atagatgcaa ccaaagtaaa gaatccggac
25081 agtatagcag cacaactgtg ttctactttg catgacctcg aatacaagaa cttatataga
25141 gtacttactt cagaaaagaa atttgcttgg ataaagcggc acttgactcc aaaagaatta
25201 ctagcgatca aaaacgctgg tgtaccagga gtaaattttt atgacgacat aaagcgtata
25261 tatcctcaca gtaatttatt ttcacacgtg cttggttaca ctgacataga tggcaatggt
25321 attgcaggag ttgaggcgta tataagtaaa aacaaacaac attctgttat ccaagtacct
25381 gatatacagt tgtatgaatg ttgcaacttg aaagcaattc ctgcacagca attccccaca
25441 caacagatcc agtgcgtgac actgggatcc agatataaaa atactgacaa attacgtgat
25501 gaacaacgga ttccagaaac atatgtcaaa aacaatgttt gtgatgaaat tgcgcataaa
25561 actggattcc agtgtcagtg cactggaatg acaccagacc aagcttctgc acaatcttct
25621 gtcatccgac agggatggtc acgctctgct gatcaatatg ttcaactatc cttagataca
25681 cgagtgcaaa gcatagtgca tgaagagcta actaaagctg taagcagata tcaggcactt
25741 ggcggagtag gaattgtttt aaatgtgaga aatagtgaag ttatctcgat ggtcagccta
25801 cctgatttta atcccaactt acagaataag gcagaagacg tacaaaagtt taatcgcgcc
25861 agtcttgggg tatatgagat ggggtcggta ttaaaatact ttacaatagc cgcagcgctt
25921 gatgcgaacg ctacaaaaac tagcgattta tatgacgtat caacaccaat caccattgga
25981 aagtataaaa ttcaggattt tcataaatct aaaattccaa aaattactgt gcaagatata
26041 tttgtaaaat catccaacat tggtgcagca aaaattgcag tcaaactagg tattgaaaaa
26101 caggtagaat actttaaagc tatgaagcta ttttctcctt tgaaaataga aataccagaa
26161 aaatccacac cgataatccc ggataaatgg agtgaaaaca ctttaataac agcatcttat
26221 ggttatggca tagctgtaac tcctatacat cttgcacaaa ctgcagcagc attaatcaac
26281 aatgggatat ttcataacgc aaccttgatg ttgaataaaa gaagtatagg agagcaaatt
26341 atctcaagaa gaacttccag ggaaatgaga aaattattac gtgcagcagt aacagatggc
26401 actggcagaa aagcaaaaat aaaggcatat tcaataggag gaaaaactgg atcggcggaa
26461 aaagttgtag atggtaaata tagcaaagat gcaaacatag catcatttat aggagtgcta
26521 actatgcttg acccaaggta catagtgcta attgctattg atgagcctca agggatgcac
26581 cataccgggg gagtaattgc tgcgcctata gtaagaaaca ttataaatag agtagcacct
26641 atactaaatg ttacacctga gatgtaaaat tataattgct ttactataat ttcaaattta
26701 tcattaccaa aatccttatt aatataccaa gaaccatcat ctttagttac aaattcaaga
26761 cctttaaggt ctttttttat tgtctttcca ttttgaccat caatagcgat attaatatcg
26821 aatgtaataa tgtctagatt tatcatagct tctttcccgc tgcacgaatt tagactgaac
26881 ttgcctaaca tgacatcatt aaaactcact tcccctgtat agacatcaca ctgaccgtcc
26941 acacaacacg tagtttcaat atagaaaaga tctgacatac aaaatacctt aaaaaaaaga
27001 actaaacata tatggccact acaccacacc gctaacaagt agtaaagcgt cgataaacct
27061 aagttacttt agctacgtag ccataataca gctagaaatg taaaattcaa cagttaattg
27121 tgtgattaat agagaagtct tatttagtac accttatagg aatggctgtt tagttttttt
27181 gttaatcttt tttaccatgc tgtcaatcat atgctttatc ccttgatata cagagtatgt
27241 actttcgtcg ctacgcatat aagcagaaca ggaaaataca ttatgttctt cgtcttctat
27301 tgataactct gtatcgcatt ttatgtgctc gccgccaagc ctttctatca actgttctct
27361 gtcatctcct atagttacct taaccttact ttcttcttta cctatcttgc tacttaaaat
27421 tgaaacaatg atggctggag atatgcatat cgctcctatt ggcttttttg taacaaaaaa
27481 ttctgaaact aatctttcaa attcaggcat tactgtcacc atgtctttac tttcagctaa
27541 gtcagataaa tttttcgcaa ccccgtatcc accaggtaca actagcatgt caaaattttc
27601 agctttggct tcttttaagt catatatttc gcctcttgcg attcttgctg cttctacaag
27661 tacattcctt ttttcttttg ttgcctcttt tgttctatga ttcataactt gtgtgatatt
27721 gatatcaggt gcaaagcatt taacatccac ttcctgctga tcaagaacaa gcaaacttaa
27781 aaccgcttct cttacctctg caccgtcaag gtgaccacac cctgataaga ccacagcagc
27841 ttttaatttt ttttcaccca taaagtacct tgatttcata taatctagat attttaatac
27901 aaattctata tttggaaagg ggtatgttat atcaattata atataattaa tgattagtat
27961 gtataattag gcatatttat aatcaacaaa gctgaatatg gaatctagca atagtatgtt

```
    28021 ttttgacact
//
LOCUS       JAQZAU010000048          22476 bp    dna     linear   ENV 17-MAR-2023
DEFINITION  MAG: Wolbachia pipientis isolate wEsol contig_32781, whole genome
            shotgun sequence.
ACCESSION   JAQZAU010000048 JAQZAU010000000
VERSION     JAQZAU010000048.1
DBLINK      BioProject: PRJNA929258
            BioSample: SAMN33142973
KEYWORDS    WGS; ENV; Metagenome Assembled Genome; MAG.
SOURCE      Wolbachia pipientis (insect metagenome)
  ORGANISM  Wolbachia pipientis
            Bacteria; Pseudomonadota; Alphaproteobacteria; Rickettsiales;
            Anaplasmataceae; Wolbachieae; Wolbachia.
REFERENCE   1  (bases 1 to 22476)
  AUTHORS   Fiutek,N., Couger,B.M., Roy,S., de la Torre,J.R. and Connor,E.F.
  TITLE     Genomic assessment of the contribution of the Wolbachia
            endosymbiont of Eurosta solidaginis to gall-induction
  JOURNAL   International Journal Molecular Sciences
REFERENCE   2  (bases 1 to 22476)
  AUTHORS   Fiutek,N., Couger,B.M., Roy,S., de la Torre,J.R. and Connor,E.F.
  TITLE     Direct Submission
  JOURNAL   Submitted (10-FEB-2023) Biology, San Francisco State University,
            1600 Holloway Ave, San Francisco, CA 94132, USA
COMMENT     ##Genome-Assembly-Data-START##
            Assembly Date          :: JUN-2019
            Assembly Method        :: FLYE v. 06-2019
            Genome Representation  :: Full
            Expected Final Version :: Yes
            Genome Coverage        :: 100.0x
            Sequencing Technology  :: Illumina HiSeq; PacBio Sequel
            ##Genome-Assembly-Data-END##
FEATURES             Location/Qualifiers
     source          1..22476
                     /organism="Wolbachia pipientis"
                     /mol_type="genomic DNA"
                     /submitter_seqid="contig_32781"
                     /isolate="wEsol"
                     /isolation_source="galls on Solidago altissima"
                     /host="Eurosta solidaginis MN-2018-EFC"
                     /db_xref="taxon:955"
                     /environmental_sample
                     /country="USA: Cowling Arboretum, Carleton College,
                     Northfield, MN"
                     /lat_lon="44.48 N 93.13 W"
                     /altitude="280 m"
                     /collection_date="2018-11"
                     /metagenome_source="insect metagenome"
                     /note="metagenomic"
     gene            complement(<1..113)
                     /locus_tag="wEsol_00420"
     CDS             complement(<1..113)
                     /locus_tag="wEsol_00420"
                     /codon_start=1
                     /transl_table=11
                     /product="hypothetical protein"
                     /translation="MSFQCLTLESSFPYNLIENVVTTFYASLLVNKQTFLD"
     gene            278..613
                     /locus_tag="wEsol_00421"
     CDS             278..613
```

```
                     /locus_tag="wEsol_00421"
                     /codon_start=1
                     /transl_table=11
                     /fullproduct="qcoverage 100 , hcoverage 100 , similarity
                     100 , identity 100 , evalue 1.62e-71 , alnlength 111 ,
                     Bacteria , Proteobacteria , WP_015589221.1 MULTISPECIES:
                     divalent-cation tolerance protein CutA [unclassified
                     Wolbachia]"
                     /cog="CutA COG1324 104 Uncharacterized protein involved
                     in tolerance to divalent cations"
                     /pfam="CutA1"
                     /product="divalent-cation
                     tolerance protein CutA"
                     /translation="MSSLVLIYTTFSNFEEAKTISEELLNEKLIVCVNIFPEVNSLYL
                     WEGKISNSCEVVAIMKSKNDQVDKIVEKIEAMHSYDQPAIAVMPIEKANKSFTNWANS
                     VIDVSSIGV"
                     /besthit="qcoverage 100 , hcoverage 100 , similarity 100
                     , identity 100 , evalue 1.62e-71 , alnlength 111 ,
                     Bacteria , Proteobacteria , WP_015589221.1 MULTISPECIES:
                     divalent-cation tolerance protein CutA [unclassified
                     Wolbachia]"
     gene            697..1272
                     /locus_tag="wEsol_00422"
     CDS             697..1272
                     /locus_tag="wEsol_00422"
                     /codon_start=1
                     /transl_table=11
                     /fullproduct="qcoverage 100 , hcoverage 100 , similarity
                     100 , identity 100 , evalue 5.16e-127 , alnlength 191 ,
                     Bacteria , Proteobacteria , WP_010962863.1 MULTISPECIES:
                     hypothetical protein [Wolbachia]"
                     /translation="MISLFYKLRLLNYILGSLKDIIVKLFLFTMYLYTFSIVLMFLSN
                     GFWYAVEFCSFKNAFYRLFQYCKWYYENREDLGYGISIRILVAFLTPHFLYKLLLGTG
                     WKSFLKEKIIQILNTRRKKGSEFSNSSKPSGSSYSDKNNYSNNSGNNERQAQDAKRKV
                     NMIKQLLIKDIEATIDRRLNYIFFGKRSKPH"
                     /product="hypothetical protein"
                     /besthit="qcoverage 100 , hcoverage 100 , similarity 100
                     , identity 100 , evalue 5.16e-127 , alnlength 191 ,
                     Bacteria , Proteobacteria , WP_010962863.1 MULTISPECIES:
                     hypothetical protein [Wolbachia]"
     gene            complement(1288..1575)
                     /locus_tag="wEsol_00423"
     CDS             complement(1288..1575)
                     /locus_tag="wEsol_00423"
                     /codon_start=1
                     /transl_table=11
                     /fullproduct="qcoverage 100 , hcoverage 100 , similarity
                     100 , identity 100 , evalue 1.01e-62 , alnlength 95 ,
                     Bacteria , Proteobacteria , WP_007549783.1 MULTISPECIES:
                     hypothetical protein [Wolbachia]"
                     /translation="MSNNFNFKEFFNHYETNSTSDDIQRYYLLWKSVIAQAMIDAASH
                     CKKTESLVEKRKAISWLSDFSQDFVHTCILADCDPVYVKNKIQPTLKSLTR"
                     /product="hypothetical protein"
                     /besthit="qcoverage 100 , hcoverage 100 , similarity 100
                     , identity 100 , evalue 1.01e-62 , alnlength 95 ,
                     Bacteria , Proteobacteria , WP_007549783.1 MULTISPECIES:
                     hypothetical protein [Wolbachia]"
     gene            complement(2165..4186)
                     /locus_tag="wEsol_00424"
     CDS             complement(2165..4186)
```

```
                     /locus_tag="wEsol_00424"
                     /codon_start=1
                     /transl_table=11
                     /fullproduct="qcoverage 100 , hcoverage 100 , similarity
                     100 , identity 100 , evalue 0.0 , alnlength 673 ,
                     Bacteria , Proteobacteria , WP_015589220.1 MULTISPECIES:
                     ATP-dependent DNA helicase RecG [unclassified Wolbachia]"
                     /cog="RecG COG1200 677 RecG-like helicase"
                     /pfam="Pfam-B_17158 ResIII DEAD SNF2_N SecA_DEAD
                     Helicase_C Helicase_C Pfam-B_5382"
                     /tigr="TIGR00643 recG 629 ATP-dependent DNA helicase
                     RecG"
                     /product="ATP-dependent DNA
                     helicase RecG"
                     /translation="MSNQIFLNSRLENIPKFHSAILPKLCGGDKVVDLLFYKPLSYVD
                     RSKLLSDARVGEFTTFIAKVYEHQSPTFRGRPYKIVVESESQYIFIVFFNYSVKYLYK
                     LFPIGANVIISGKLEKFAEHWQITHPDYVSLDINQFKKIACIEPVYQLCRGITNKRIG
                     NIISSNLKELPDLPEWIDDTLIKQKKWLSWRESIIKLHRPSSLVEAEVCRKRLAYDEL
                     FAYQLALKLARKNHVKERGREFIILSKYKEQVLNELSFQLTNDQTRAIDEISERQKSK
                     YRMISLLQGDVGSGKTVVALFAMINVVENNMQAALMAPTTILAEQHYNWIEEVLSCTD
                     IKVALLTGKTSRKERKIIMNELASGVLNIVIGTHALFQANVTFKNLGLAVIDEQQRFG
                     VMQRNRLVGKGENADILFVTATPIPRTLQQAMYGDVECSILKEKPKSRLPIKTVTMNI
                     SRVPDVIEKLKGAINRGEKAYWICPYIEENEETNIAAAEMRFQELQKTFLDKVGIIHG
                     KLTQEQKDQVMFSFKRNEFSLLVATTVIEVGIDVPDATIMIIENAEQFGLSQLHQLRG
                     RVGRGNKPSFCVLLYDNLSKSSSSKLKIMCESQDGFYIAERDMMLRGSGDILGTKQSG
                     CMEFKFADLYQDKELLNLAYNNAKGAMADFELLLDIFEYRSRLHFSKFQ"
                     /besthit="qcoverage 100 , hcoverage 100 , similarity 100
                     , identity 100 , evalue 0.0 , alnlength 673 , Bacteria ,
                     Proteobacteria , WP_015589220.1 MULTISPECIES:
                     ATP-dependent DNA helicase RecG [unclassified Wolbachia]"
     gene            complement(4236..4397)
                     /locus_tag="wEsol_00425"
     CDS             complement(4236..4397)
                     /locus_tag="wEsol_00425"
                     /codon_start=1
                     /transl_table=11
                     /fullproduct="qcoverage 100 , hcoverage 100 , similarity
                     98.1 , identity 98.1 , evalue 1.12e-27 , alnlength 53 ,
                     Bacteria , Proteobacteria , WP_208974573.1 hypothetical
                     protein [Wolbachia pipientis]"
                     /translation="MIANIVRGNLGLPMILKTTNSSFRYVLAAEIPRMNRGITLESCA
                     SYSAYLTIR"
                     /product="hypothetical protein"
                     /besthit="qcoverage 100 , hcoverage 100 , similarity 98.1
                     , identity 98.1 , evalue 1.12e-27 , alnlength 53 ,
                     Bacteria , Proteobacteria , WP_208974573.1 hypothetical
                     protein [Wolbachia pipientis]"
     gene            4483..5313
                     /locus_tag="wEsol_00426"
     CDS             4483..5313
                     /locus_tag="wEsol_00426"
                     /codon_start=1
                     /transl_table=11
                     /fullproduct="qcoverage 100 , hcoverage 100 , similarity
                     99.6 , identity 99.6 , evalue 1.07e-193 , alnlength 276 ,
                     Bacteria , Proteobacteria , WP_015589219.1 MULTISPECIES:
                     hypothetical protein [unclassified Wolbachia]"
                     /translation="MSSSISEEKENPVVTLRAQAEKFGFSKLRAGKDNAEANSGNTAF
                     LDFQRMDFVINGKSISRDLIAELYKEHDSLLPKSEGKNEDYRPFARKVFKEMFKYAKA
                     EVPSDSILEELVINCNQAGYEAGVHIEFQGALSQHSFIIERPEKVISIDCVSQNNVSV
```

```
                     KSDMTLPIFFQRSDGTFGDKVYDLSSSLEFTLKSQDDKNVTYTNGNLSLTVPERLKDY
                     NIGDKSLFDIIKEYFQKLCERLGFFKIKIEHGLGDPLEVSDHLENMEPPIRSNEHGLN
                     "
                     /product="hypothetical protein"
                     /besthit="qcoverage 100 , hcoverage 100 , similarity 99.6
                     , identity 99.6 , evalue 1.07e-193 , alnlength 276 ,
                     Bacteria , Proteobacteria , WP_015589219.1 MULTISPECIES:
                     hypothetical protein [unclassified Wolbachia]"
     gene            complement(5310..6194)
                     /locus_tag="wEsol_00427"
     CDS             complement(5310..6194)
                     /locus_tag="wEsol_00427"
                     /codon_start=1
                     /transl_table=11
                     /fullproduct="qcoverage 100 , hcoverage 100 , similarity
                     99.7 , identity 99.7 , evalue 1.48e-209 , alnlength 294 ,
                     Bacteria , Proteobacteria , WP_006280293.1 MULTISPECIES:
                     tRNA (adenosine(37)-N6)-dimethylallyltransferase MiaA
                     [Wolbachia]"
                     /cog="MiaA COG0324 308 tRNA
                     delta(2)-isopentenylpyrophosphate transferase"
                     /pfam="AAA_14 AAA_17 AAA_18 IPPT"
                     /tigr="TIGR00174 miaA 288 tRNA dimethylallyltransferase"
                     /product="tRNA
                     (adenosine(37)-N6)-dimethylallyltransferase MiaA"
                     /translation="MKNNIVIITGITASGKSELCDNLIEKYGNISIINCDSKQVYEEI
                     PIITAQPPKQEVFYKLYGYVSAKENYSVGLWLEDLEKEVNHALENAQIPIITGGSGLY
                     ISSLIKGLSSMPQISQKVRKNVSELRKNLSKEEFYKLALSKDPRIQGKVFMNDSHRLS
                     RALEVITETGKSIFVWQENRQPPLFNNFKIYTILPKREDVYRKINSRFITMIENGAID
                     EVKKLLSMNLAPHLPAMKAHGVPEIIKYLKGEITLDEVIQIAQTNTRHYAKRQYTWFK
                     KQFLNAEVIDCANKLKIF"
                     /besthit="qcoverage 100 , hcoverage 100 , similarity 99.7
                     , identity 99.7 , evalue 1.48e-209 , alnlength 294 ,
                     Bacteria , Proteobacteria , WP_006280293.1 MULTISPECIES:
                     tRNA (adenosine(37)-N6)-dimethylallyltransferase MiaA
                     [Wolbachia]"
     gene            6270..6740
                     /locus_tag="wEsol_00428"
     CDS             6270..6740
                     /locus_tag="wEsol_00428"
                     /codon_start=1
                     /transl_table=11
                     /fullproduct="qcoverage 100 , hcoverage 100 , similarity
                     100 , identity 100 , evalue 6.14e-102 , alnlength 156 ,
                     Bacteria , Proteobacteria , WP_015589217.1 MULTISPECIES:
                     hypothetical protein [unclassified Wolbachia]"
                     /translation="MAENNDNNSFTKRFTNRTPGGTGEGFSSKYSSQNTKERALGEKG
                     KISELAGKKTESKEAFSESGGIKSKSRTVNERSFADATKRSRSDLVYLVRGKDRGKSA
                     WHYVLVDKEKREMFLAKSRTGSIDVADYGEILYSGWGEDPPQAIIDKVNEEFGL"
                     /product="hypothetical protein"
                     /besthit="qcoverage 100 , hcoverage 100 , similarity 100
                     , identity 100 , evalue 6.14e-102 , alnlength 156 ,
                     Bacteria , Proteobacteria , WP_015589217.1 MULTISPECIES:
                     hypothetical protein [unclassified Wolbachia]"
     gene            6859..7446
                     /locus_tag="wEsol_00429"
     CDS             6859..7446
                     /locus_tag="wEsol_00429"
                     /codon_start=1
                     /transl_table=11
```

/fullproduct="qcoverage 100 , hcoverage 100 , similarity 100 , identity 100 , evalue 1.31e-131 , alnlength 195 , Bacteria , Proteobacteria , WP_141457207.1 MULTISPECIES: threonylcarbamoyl-AMP synthase [unclassified Wolbachia]"
/cog="SUA5 COG0009 211 Putative translation factor (SUA5)"
/pfam="Sua5_yciO_yrdC"
/tigr="TIGR00057 TIGR00057 202 Sua5/YciO/YrdC/YwlC family protein"
/product="
threonylcarbamoyl-AMP synthase"
/translation="MVSKIINAIQNNLLVCFPTETVYALACNALDNESIGKIYQIKKR
SQNKPLSIFVSDIYNLTKIAKVKERYIDLINHFSPGPITYILPLKNNNILPNEFFKDS
IGIRIPEHPIAISILNKLKVPIVATSINISGEKSVCKADDIPQSIKQHLSAVIEDDEL
VSGIESTIIDLTEDKIKVLREGAILLQTVNNAFSN"
/besthit="qcoverage 100 , hcoverage 100 , similarity 100 , identity 100 , evalue 1.31e-131 , alnlength 195 , Bacteria , Proteobacteria , WP_141457207.1 MULTISPECIES: threonylcarbamoyl-AMP synthase [unclassified Wolbachia]"

gene 7464..8285
/locus_tag="wEsol_00430"

CDS 7464..8285
/locus_tag="wEsol_00430"
/codon_start=1
/transl_table=11
/pfam="DNA_pol3_delta2 AAA_24 AAA_14 AAA"
/fullproduct="qcoverage 100 , hcoverage 100 , similarity 100 , identity 100 , evalue 4.02e-192 , alnlength 273 , Bacteria , Proteobacteria , WP_141457205.1 MULTISPECIES: DNA polymerase III subunit delta' [unclassified Wolbachia]"
/translation="MKKVIGHNQAKKKLMNNLSVQSWLICGKKGIGKATLAKSFSNWL
LIKSYNEVALDLHIVEGDTIGIEKVREMKNFLHLSPIQSEHKIAIIDSLEAMTNNAKN
AILKILEEPPKNSKIFIISHKPYDIQTTILCRCFQLNLLPLTYDETKQVVSSQCKFDD
QIFDEIITLFPGMPGMIINAISSDAYEAYKCFYKFLHDLKNPNMINKVINSEIELELA
SYIIQAFILESIKREVNDVEILLSQWKKIDELFVVAKQLHLDKKHVLANVVNIMT"
/product="DNA polymerase III
subunit delta'"
/besthit="qcoverage 100 , hcoverage 100 , similarity 100 , identity 100 , evalue 4.02e-192 , alnlength 273 , Bacteria , Proteobacteria , WP_141457205.1 MULTISPECIES: DNA polymerase III subunit delta' [unclassified Wolbachia]"

gene 8359..9153
/locus_tag="wEsol_00431"

CDS 8359..9153
/locus_tag="wEsol_00431"
/codon_start=1
/transl_table=11
/fullproduct="qcoverage 100 , hcoverage 100 , similarity 100 , identity 100 , evalue 4.20e-194 , alnlength 264 , Bacteria , Proteobacteria , WP_015589214.1 MULTISPECIES: hypothetical protein [unclassified Wolbachia]"
/translation="MTDNKLTVWQSIEVLTKAILNMQKELSSIKNIALDQDVTLEKGK
ELLSGIYEANFKLNKAINIATLPKIGYHMLNGEFVVRNHITEEEVRIPRDFHYLKVVK
SDHDNYKLTFCNFLGNEFFEYKKYDPQYSGVSDEYKFVDFGSVKKTHNLKFKEYVGHA
PKFFAAEGPIEPGSQNHVIDLFELVKSGKGRKVGTFADEFGYFDDQNKLHYYNYHKSV
ESNTYDPENFSVKMINIDVSKIDKFYLIAEQGDIIIHPIWENLDIF"
/product="hypothetical protein"
/besthit="qcoverage 100 , hcoverage 100 , similarity 100

, identity 100 , evalue 4.20e-194 , alnlength 264 , Bacteria , Proteobacteria , WP_015589214.1 MULTISPECIES: hypothetical protein [unclassified Wolbachia]"

```
gene            9222..9875
                /locus_tag="wEsol_00432"
CDS             9222..9875
                /locus_tag="wEsol_00432"
                /codon_start=1
                /transl_table=11
                /fullproduct="qcoverage 100 , hcoverage 100 , similarity
                100 , identity 100 , evalue 2.81e-146 , alnlength 217 ,
                Bacteria , Proteobacteria , WP_015589213.1 MULTISPECIES:
                type IV secretion system protein VirB8 [unclassified
                Wolbachia]"
                /cog="VirB8 COG3736 239 Type IV secretory pathway,
                component VirB8"
                /pfam="VirB8"
                /product="type IV secretion
                system protein VirB8"
                /translation="MEDKLLESVKNKSYFSKAVEWYCHRYLFCVAERSWMALIVSLLL
                VCLCLLTLNIYLLFPVKKDLNFVKYMNHTEDEFSAMHKLSFSKKEDEYTSTARYLISK
                YIEVYESIKTVEPKYQENFIRNNSIHKIHQGFQEKTNNEAVSSKRKITNINVITLSID
                RSTKDLVTFAGNATVVFTTEQNKEDTVEISFTLSNIKATLAGIIPFKFIVNGYKYRF"
                /besthit="qcoverage 100 , hcoverage 100 , similarity 100
                , identity 100 , evalue 2.81e-146 , alnlength 217 ,
                Bacteria , Proteobacteria , WP_015589213.1 MULTISPECIES:
                type IV secretion system protein VirB8 [unclassified
                Wolbachia]"
gene            complement(9872..11353)
                /locus_tag="wEsol_00433"
CDS             complement(9872..11353)
                /locus_tag="wEsol_00433"
                /codon_start=1
                /transl_table=11
                /fullproduct="qcoverage 100 , hcoverage 100 , similarity
                100 , identity 100 , evalue 0.0 , alnlength 493 ,
                Bacteria , Proteobacteria , AGK00129.1 Putative
                NADH-ubiquinone oxidoreductase subunit [Wolbachia
                endosymbiont of Drosophila simulans wHa]"
                /cog="HyfB COG0651 504 Formate hydrogenlyase subunit
                3/Multisubunit Na+/H+ antiporter, MnhD subunit"
                /pfam="Oxidored_q1"
                /tigr="TIGR01974 NDH_I_L 630 proton-translocating
                NADH-quinone oxidoreductase, chain L"
                /product="Putative NADH-ubiquinone
                oxidoreductase subunit"
                /translation="MYLSLPFSITENYLLITALIPLLSALVIFFTGKWPGVNNSITVT
                SSVLLFTYTCLCTLYWVYGDHSQFILMDFGNNLHISLKLESTGVIFSLLISFLWVLTS
                IYAICYMQNNYADSNHSSFLCFFSISISCAMFIAFSGDLLTTFVFYELLTISTYPLVT
                YNSTNESMIAGRYYFGVLFFSSLVLFFPAIGLLYNEFHTLDFVSGGIFKFDTSLITFI
                AVCFAMLIYGIGKAALMPMHFWLPKAMVAPTPVSALLHAVAVVKSGVFIIIKVILYTF
                GIGNMQRFAQQSWFAGGWLPYAAGFTIITASLIALKQKELKKLLAYSTISQLSYIILF
                ASIFSELSMRVAIFQLVCHAFAKITLFFVAGAIITRAGEKYIDKIYGIGRSMPISMTA
                FAIGALSMIGVPPAPTFWGKFLIFQAVFNSGNVILSVFVTLVLIVSTILNALYFLPII
                YNAFFLKPSQNYFIKKTPILLVLPPVITATCTLVIFFSYQVIF"
                /besthit="qcoverage 100 , hcoverage 100 , similarity 100
                , identity 100 , evalue 0.0 , alnlength 493 , Bacteria ,
                Proteobacteria , AGK00129.1 Putative NADH-ubiquinone
                oxidoreductase subunit [Wolbachia endosymbiont of
                Drosophila simulans wHa]"
```

```
gene            complement(11729..12085)
                /locus_tag="wEsol_00434"
CDS             complement(11729..12085)
                /locus_tag="wEsol_00434"
                /codon_start=1
                /transl_table=11
                /fullproduct="qcoverage 100 , hcoverage 100 , similarity
                100 , identity 100 , evalue 8.73e-77 , alnlength 118 ,
                Bacteria , Proteobacteria , WP_007549151.1 MULTISPECIES:
                holo-[acyl-carrier-protein] synthase [Wolbachia]"
                /cog="AcpS COG0736 127 Phosphopantetheinyl transferase
                (holo-ACP synthase)"
                /pfam="ACPS"
                /tigr="TIGR00516 acpS 125 holo-[acyl-carrier-protein]
                synthase"
                /product="holo-"
                /translation="MIHGIGTDIVYIPRVSRILQKYGEKFLNRIYTEKEIELSRKYNS
                QEMRARYFAKRFAAKEAFVKARGSGQGIIMKDIEIYNDVRGKPYLTVSKDFISKIHLS
                LSDDGDYATAFVVICV"
                /besthit="qcoverage 100 , hcoverage 100 , similarity 100
                , identity 100 , evalue 8.73e-77 , alnlength 118 ,
                Bacteria , Proteobacteria , WP_007549151.1 MULTISPECIES:
                holo-[acyl-carrier-protein] synthase [Wolbachia]"
gene            complement(12082..13350)
                /locus_tag="wEsol_00435"
CDS             complement(12082..13350)
                /locus_tag="wEsol_00435"
                /codon_start=1
                /transl_table=11
                /pfam="tRNA-synt_2b HGTP_anticodon"
                /fullproduct="qcoverage 100 , hcoverage 100 , similarity
                100 , identity 100 , evalue 2.48e-309 , alnlength 422 ,
                Bacteria , Proteobacteria , WP_015589211.1 MULTISPECIES:
                proline--tRNA ligase [unclassified Wolbachia]"
                /translation="MRLSKYYLPTLKEKPAHAKIISHQYSLRAGLIKQIASGIYSWLP
                LGLLVLKNIEDIIRDEMNKSGAIEVLMPCVQPANLWRESGRYDDYGKEMLRIRDRHEE
                DMLFGPTHEEVATDLIRDMVKSYKDLPLCLYQIQWKFRDEVRPRYGVMRGREFLMKDA
                YSFDVDYEGALNSYNLMYKTYIKIFKRMGLTPIGVRADTGPIGGNLSHEFHILANTGE
                STLYYDNKFSELLESEDVESLKSIYAVADDMHDPETCPISQEQLNVSKGIEIGHIFYF
                GDKYSKPMKASVTSQDGKNVNIHMGSYGIGVSRLVGAIIEAFHDDKGIIWPESVAPFR
                IGLINLQTKVTEAADKIYKALKSDEVLYDDTEGSVGVKFSRMDLIGLPWQIIVGKKAI
                DEKIVEVKNRATGEVKEMQIEEAINHFSTK"
                /product="proline--tRNA
                ligase"
                /besthit="qcoverage 100 , hcoverage 100 , similarity 100
                , identity 100 , evalue 2.48e-309 , alnlength 422 ,
                Bacteria , Proteobacteria , WP_015589211.1 MULTISPECIES:
                proline--tRNA ligase [unclassified Wolbachia]"
gene            complement(13666..15009)
                /locus_tag="wEsol_00436"
CDS             complement(13666..15009)
                /locus_tag="wEsol_00436"
                /codon_start=1
                /transl_table=11
                /pfam="DDE_Tnp_1"
                /fullproduct="qcoverage 100 , hcoverage 100 , similarity
                99.8 , identity 99.8 , evalue 0.0 , alnlength 447 ,
                Bacteria , Proteobacteria , AGJ99681.1 Transposase, IS4
                family [Wolbachia endosymbiont of Drosophila simulans
                wHa]"
```

/translation="MRELTMDRIACLSKDLNEFFNEKADEISIAVGFIKRKRKLNGSS
FIKAMVFGNIGVDDCSIETMCQLLNEDSIEITKQGLDFRFSEEAVEFMKKMYNEALCL
FKKSLQIDCKILQQFGSVKLLDSSYISLPISMEDMYKGYGSSYRDCENNTKSGIKLQL
VFDYLNQALDKLNFMEGIRSDQGYRDYLNGLSANDLLIFDLGYFVPSSFKQIDEAGAY
FVSRYKSDTNIYDIETNQKIELLEYLEGQLSSEKEVLLGKEVKIKVRIICQKLTEEQS
MARRRKANKLAKSHGYTSSQKNQKLLDWSIFITNVPESKINAEQILTVYRVRWQIELL
FKLYKSHINLDKLKGKTNPFRVLCELYAKLCAILVFHGMIGCVKLKKNTELSLTKAFI
ELKRRVRELFLILNNIVNNLKIFLKKLTIIWSKFSLKDRYRKTRVSTLSSLHSLT"
/product="Transposase, IS4 family"
/besthit="qcoverage 100 , hcoverage 100 , similarity 99.8
, identity 99.8 , evalue 0.0 , alnlength 447 , Bacteria ,
Proteobacteria , AGJ99681.1 Transposase, IS4 family
[Wolbachia endosymbiont of Drosophila simulans wHa]"

gene complement(15028..16155)
/locus_tag="wEsol_00437"

CDS complement(15028..16155)
/locus_tag="wEsol_00437"
/codon_start=1
/transl_table=11
/fullproduct="qcoverage 95.4666666666667 , hcoverage
86.8932038834951 , similarity 100 , identity 100 , evalue
3.94e-257 , alnlength 358 , Bacteria , Proteobacteria ,
WP_174517027.1 IS256 family transposase [Wolbachia
endosymbiont of Cardiocondyla obscurior]"
/cog="COG3328 COG3328 379 Transposase and inactivated
derivatives"
/pfam="Transposase_mut MULE DDE_Tnp_ISL3"
/product="IS256 family transposase"
/translation="MSQKVVNRTTGLVDYKELETNILSSIREGRPLTGRDGALTPFIK
RLLEASLEGEIENHLLAESEENNRRNGRNAKTLRTSAGSFELLTPRDREGSFEPQIVK
KRQTNLHPELETKILSTFASGMGYRDIASHVEEIYDHKISAAEISSITDKLLPVINEW
RSRPLQSVYPIVFMDGMFFKVKEDGHCVSKCMYNILGIDQNGRKEVLGFYLAESEGAN
FWLGVLNDLKERGVEDILIACVDGLKSFPTAINSVFPNAEVQLCVVHQIRNSLKYVSS
KDVKIFMSDLKKVYQASSREIAENYLLELEEKWGEKYPLVVKSWQNNWENLSGYFKYS
GPVRRLIYTTNPIEGLHRQIRKFTLRKSVSKVDRVKENNKV"
/besthit="qcoverage 95.4666666666667 , hcoverage
86.8932038834951 , similarity 100 , identity 100 , evalue
3.94e-257 , alnlength 358 , Bacteria , Proteobacteria ,
WP_174517027.1 IS256 family transposase [Wolbachia
endosymbiont of Cardiocondyla obscurior]"

gene 16307..17650
/locus_tag="wEsol_00438"

CDS 16307..17650
/locus_tag="wEsol_00438"
/codon_start=1
/transl_table=11
/pfam="DDE_Tnp_1"
/fullproduct="qcoverage 100 , hcoverage 100 , similarity
99.8 , identity 99.8 , evalue 0.0 , alnlength 447 ,
Bacteria , Proteobacteria , AGJ99681.1 Transposase, IS4
family [Wolbachia endosymbiont of Drosophila simulans
wHa]"
/translation="MRELTMDRIACLSKDLNEFFNEKADEISIAVGFIKRKRKLNGSS
FIKAMVFGNIGVDDCSIETMCQLLNEDSIEITKQGLDFRFSEEAVEFMKKMYNEALCL
FKKSLQIDCKILQQFGSVKLLDSSYISLPISMEDMYKGYGSSYRDCENNTKSGIKLQL
VFDYLNQALDKLNFMEGIRSDQGYRDYLNGLSANDLLIFDLGYFVPSSFKQIDEAGAY
FVSRYKSDTNIYDIETNQKIELLEYLEGQLSSEKEVLLGKEVKIKVRIICQKLTEEQS
MARRRKANKLAKSHGYTSSQKNQKLLDWSIFITNVPESKINAEQILTVYRVRWQIELL
FKLYKSHINLDKLKGKTNPFRVLCELYAKLCAILVFHGMIGCVKLKKNTELSLTKAFI
ELKRRVRELFLILNNIVNNLKIFLKKLTIIWSKFSLKDRYRKTRVSTLSSLHSLT"

```
                     /product="Transposase, IS4 family"
                     /besthit="qcoverage 100 , hcoverage 100 , similarity 99.8
                     , identity 99.8 , evalue 0.0 , alnlength 447 , Bacteria ,
                     Proteobacteria , AGJ99681.1 Transposase, IS4 family
                     [Wolbachia endosymbiont of Drosophila simulans wHa]"
     gene            complement(17642..18667)
                     /locus_tag="wEsol_00439"
     CDS             complement(17642..18667)
                     /locus_tag="wEsol_00439"
                     /codon_start=1
                     /transl_table=11
                     /product="hypothetical protein"
                     /pfam="CAP_N Pfam-B_11521"
                     /translation="MSTSNLKISIAALSLCVSAVAIYIHLPYVIAAVSGSGAIAAVPL
                     AIFAVSVVVALASVAYLIKLAVSRDDNKKEAGLNGQEFGHTASQEPQKSACKNCNNLD
                     SELIRKGLSVKQDSGSQTEVRSSEHVPNLTPQQPPLTLGTAIPQNNNAPPPPPPPPSS
                     NGTSSSKNASSPSDPDVKPDMSAVFNKITQQKPNLMSKEERDKKLKEQAAKKSSNKGG
                     GQQEKNEDLMADLSKALQKRRAGVNGKQGSDVQQPSHLGRAFSKVLAMIPPPRSRSSS
                     VSSDSSNVSDDENWETDPEPIEQASSGSQSLHRERSGSLDSGIGSGSEPTSPSHSGVL
                     NKPYASS"
                     /besthit="qcoverage 26.3929618768328 , hcoverage 100 ,
                     similarity 100 , identity 100 , evalue 1.74e-43 ,
                     alnlength 90 , Bacteria , Proteobacteria , WP_051062215.1
                     hypothetical protein [Wolbachia endosymbiont of
                     Drosophila simulans]"
     gene            19378..19758
                     /locus_tag="wEsol_00440"
     CDS             19378..19758
                     /locus_tag="wEsol_00440"
                     /codon_start=1
                     /transl_table=11
                     /pfam="RE_AlwI"
                     /fullproduct="qcoverage 100 , hcoverage 100 , similarity
                     100 , identity 100 , evalue 8.47e-81 , alnlength 126 ,
                     Bacteria , Proteobacteria , WP_010082467.1 hypothetical
                     protein [Wolbachia pipientis]"
                     /translation="MRKRKLSFIDIFILILRKSVKSLQVILNEFILYRKKDYTVTASA
                     FSQARKKMKHSAFSEINEGVVSLYYQDQKFKTCFGFRVLALDASKIILPTSVEIKNEF
                     GSRKIRNQKPKDLGDYASATFAVC"
                     /product="hypothetical protein"
                     /besthit="qcoverage 100 , hcoverage 100 , similarity 100
                     , identity 100 , evalue 8.47e-81 , alnlength 126 ,
                     Bacteria , Proteobacteria , WP_010082467.1 hypothetical
                     protein [Wolbachia pipientis]"
     gene            20058..20561
                     /locus_tag="wEsol_00441"
     CDS             20058..20561
                     /locus_tag="wEsol_00441"
                     /codon_start=1
                     /transl_table=11
                     /pfam="DDE_Tnp_1_6 DDE_Tnp_1"
                     /fullproduct="qcoverage 100 , hcoverage 100 , similarity
                     100 , identity 100 , evalue 2.98e-112 , alnlength 167 ,
                     Bacteria , Proteobacteria , WP_007549143.1 transposase
                     [Wolbachia pipientis]"
                     /translation="MVLPSGEIEVLATSLMDEQKFQTGGFKELYFLRWGVETFFAKLK
                     GRLSLENFTGKSVESVKQDFWSAIFISNLESVMTEDVEEALNMDLTDDKLKRSINKSV
                     SFNAIKNLAFDIFATESDTDCIMDQLSKLFLMNTLAVRKGRKVDRHKVPYLRSFNYQK
                     RVRKHIF"
                     /product="transposase"
```

/besthit="qcoverage 100 , hcoverage 100 , similarity 100 , identity 100 , evalue 2.98e-112 , alnlength 167 , Bacteria , Proteobacteria , WP_007549143.1 transposase [Wolbachia pipientis]"

gene 21002..21529
/locus_tag="wEsol_00442"

CDS 21002..21529
/locus_tag="wEsol_00442"
/codon_start=1
/transl_table=11
/fullproduct="qcoverage 100 , hcoverage 100 , similarity 100 , identity 100 , evalue 7.51e-119 , alnlength 175 , Bacteria , Proteobacteria , WP_015589210.1 MULTISPECIES: cytochrome c family protein [unclassified Wolbachia]"
/cog="COG3474 COG3474 135 Cytochrome c2"
/pfam="Pfam-B_5051 Cytochrom_C Cytochrome_CBB3"
/product="cytochrome c
family protein"
/translation="MELNKIAASILLSGLIIMIVSNVVDMLYSPEEYKIEHQTIVAAG
SNEFQQKIEQVALDIGALMQNASFEKGKSAAKKCIACHSFEKGGMNKVGPNLWNIVGN
KKAHLGNSFNYSKAVLDRGGKWGYEELFAFLKNPKAYIKGTRMAFAGISNPQEIADLV
SYLHQLSDSPVALPK"
/besthit="qcoverage 100 , hcoverage 100 , similarity 100 , identity 100 , evalue 7.51e-119 , alnlength 175 , Bacteria , Proteobacteria , WP_015589210.1 MULTISPECIES: cytochrome c family protein [unclassified Wolbachia]"

gene 21531..22292
/locus_tag="wEsol_00443"

CDS 21531..22292
/locus_tag="wEsol_00443"
/codon_start=1
/transl_table=11
/fullproduct="qcoverage 100 , hcoverage 100 , similarity 100 , identity 100 , evalue 1.46e-184 , alnlength 253 , Bacteria , Proteobacteria , WP_012673268.1 MULTISPECIES: alpha/beta hydrolase [Wolbachia]"
/cog="MhpC COG0596 282 Predicted hydrolases or acyltransferases (alpha/beta hydrolase superfamily)"
/pfam="Hydrolase_4 Abhydrolase_5 Abhydrolase_6 Peptidase_S9 Abhydrolase_1 Abhydrolase_2 DUF2920 BAAT_C Abhydrolase_1"
/product="alpha/beta
hydrolase"
/translation="MDYCKLFDGNNGHIAYRKLQGKKASVVFFGGFASNMDGTKATAI
YKFCQENDVALVLFDYFGHGHSSGNFIDYTISDWQKNCAKVMSELTSDKQIIIGSSMG
GWLMLLTALQFPEKIAALIGISSAPDFTEDLIFKQLSGKQKEELDSKGVIDFTSEHCT
YKITKNLIEDGRKNLLLNKKTIDINCPVRLLHSINDKDVPYQTSLNLAEKIKSTDVEV
HLIKSAEHNMSDNHSLEVLFKTIREFCNQTAFCDF"
/besthit="qcoverage 100 , hcoverage 100 , similarity 100 , identity 100 , evalue 1.46e-184 , alnlength 253 , Bacteria , Proteobacteria , WP_012673268.1 MULTISPECIES: alpha/beta hydrolase [Wolbachia]"

gene complement(22476..>22359)
/locus_tag="wEsol_00444"

CDS complement(22476..>22359)
/locus_tag="wEsol_00444"
/codon_start=1
/transl_table=11
/product="hypothetical protein"
/translation="ICKVPKCDGANRHTAAVSQPLTRSGMTVFQIVGKSKSV"

```
     gene            604..678
                     /locus_tag="wEsol_tRNA-03"
     tRNA            604..678
                     /locus_tag="wEsol_tRNA-03"
                     /gene="tRNA-Gln"
                     /intronpos="0..0"
                     /program="tRNAscan-SE"
                     /cove_score="61.8"
                     /evidence=predicted
                     /type="Gln"
                     /anticodon=TTG
                     /product="tRNA-Gln (TTG)"
ORIGIN
        1 ggatccagga aagtttgctt gtttacaagc aaactagcat agaaagtggt tacaacgttt
       61 tcgatgagat tatatggaaa actggattcc agtgtcaagc actggaatga catcacaggg
      121 gcactgggat gacaccttta cgtttgaggc aaactagcta taacacttaa catactacct
      181 ttgcaaacaa atgttcatac agtacgcgct ggcgttattt tctcttgatg aataaacctc
      241 ggtagagtat agtagcagtg cgtttatata gattctaatg agtagtttag ttttgattta
      301 tacaactttt tcaaattttg aagaagctaa aactatttct gaagaattgt taaacgagaa
      361 attaattgta tgtgtaaata tatttcccga agtgaattct ctgtatttat gggaaggtaa
      421 aattagtaat agttgtgaag tagtagcaat tatgaaaagc aaaaatgatc aagttgataa
      481 aattgtagaa aaaattgaag caatgcattc ttatgatcag ccagctattg ctgtgatgcc
      541 catagaaaaa gcaaataaat cttttactaa ttgggctaat agtgttattg atgtaagtag
      601 tattggggtg tagccaagtg gtaaggcagc ggtttttgat accgccacgc gaaggttcga
      661 atccttccac cccagccatg ttgaagtggg tgtgaaatga tatcactttt ctataaatta
      721 agattattga attatatatt aggtagtcta aaagatataa ttgtgaaact gtttctcttc
      781 acaatgtatc tttacacatt cagcattgtg ctcatgtttc tcagcaatgg tttttggtat
      841 gctgtagaat tttgctcttt caaaaatgcg ttttaccgtt tgtttcaata ttgcaagtgg
      901 tattatgaaa atagggaaga tctcggttac ggaatttcta ttcgaatatt agtggcgttc
      961 cttactccgc attttctcta taaattatta cttggcacag gctggaagtc ctttttaaag
     1021 gaaaagataa tacagatact caacacaaga agaaaaaagg gtagtgagtt cagtaacagt
     1081 agcaaaccaa gtggcagtag ttatagtgat aagaataatt acagcaataa cagtggtaac
     1141 aatgagcgtc aagctcaaga tgcaaagcga aaagtaaaca tgataaagca gctattaatc
     1201 aaagatatag aggcaactat agataggaga ttgaattata tttttttcgg taaaaggagt
     1261 aaacctcatt aagtatataa aaaggcttta tctagtaagg ctttttaagg ttggctgtat
     1321 tttatttttt acgtataccg gatcacaatc agccaatata catgtgtgca caaaatcttg
     1381 actaaagtct gataaccaag aaattgcctt acgtttttct accaagcttt ctgttttttt
     1441 gcagtgactt gctgcatcaa tcattgcctg cgctattact gatttccaca gcaaataata
     1501 cctttgtata tcatctgacg tactatttgt ttcatagtgg ttaaaaaatt ctttaaaatt
     1561 gaagttgttt gacataatga tacctctttg gattgaaatt tgaaatgatt tgagtttatg
     1621 tcgccacagc agattccgct aacaagcagc gggatgacgg ttgtcgttta gccataaaca
     1681 ttaagaaatt taccaaacga aaaaaaaggc aaaagaagcc ctggggttat tatccgcttt
     1741 aaaatattgg cgttttttat gttttaaacg cttgacaagc gagattcagc cgcttttaat
     1801 tgcaactaac ttacgctgca aatgtttaag aaatttacta agcagaaaaa aagacaaaga
     1861 atccccgagt tagctagtct ttttactatct ctgtcgagta ttggcatttt ttgatgtctt
     1921 gtaacgcttt ataagcgcgt tcagcttatt tagataaaaa tctagatgta gatgaagttt
     1981 tgtaaagaca tacagtatct atatactgca aaaaattgaa cataagacgc cgatacatta
     2041 agtaatcctt accttttaat ctgcagattg gcgaaagcaa atacaatagc ttcagtttca
     2101 tgataagggg gctggcgagg tttgtcaagg aagttttttt gtttctgcgg gctacctggt
     2161 ttttctactg aaattttgaa aaatgtaatc tactcctata ttcaaatata tcaagcagta
     2221 attcaaaatc agccattgcg cctttttgcgt tattatacgc gagattgagc agctctttgt
     2281 cctgatataa atcagcaaat ttaaattcca tacatcctga ttgttttgtg cctaaaatat
     2341 ctccactgcc tcttagcatc atatcccttt cagcaatgta aaatccatct tgtgactcac
     2401 acatgatctt taactttgaa gatgagcttt tacttaaatt atcgtataat aatacacaaa
     2461 aagatggctt gtttcctcgc cctactctgc ctcttaactg atgcaattgt gataacccaa
     2521 attgctctgc attttctata atcatgatgg ttgcatctgg cacatctata ccaacttcta
     2581 tcacagtggt tgcaacaagc agagaaaatt cattcctttt gaaggaaaac ataacttgat
     2641 ctttctgctc ctgagtcaat tttccgtgta ttatcccgac tttatcaaga aatgtttttt
     2701 gtaattcctg aaaacgcatc tctgctgcag caatattggt ctcttcgttt tcttctatat
     2761 atggacaaat ccaatatgct ttttcgcccc tgtttatagc accttttagc ttttcaataa
```

2821 catctggcac cctactaatg ttcatagtta cagtttttat tggcaatctg gattttggtt
2881 tttctttcaa aattgaacat tcaacgtcac catacatagc ttgctgcaaa gttcttggaa
2941 tgggagttgc agtaacgaaa agtatatcgg cattttctcc ttttcctacc aagcgatttc
3001 tctgcatcac tccaaatcgc tgttgctcgt ctatgactgc aagccctaaa tttttaaatg
3061 taacgttagc ttgaaatagt gcatgagtgc caatcactat atttaaaaca ccacttgcaa
3121 gttcgttcat gataatcttt ctttccttgc gcgaagtttt accagtaagc agcgcaactt
3181 ttatatcggt acaggataaa acttcctcaa tccaattata atgttgctcc gccaagatag
3241 tagtcggtgc cattagagct gcctgcatgt tgttttctac cacatttatc attgcaaaaa
3301 gtgcaactac ggttttgcca ctaccaacat caccttgcag caagcttatc atgcgatact
3361 tggatttttg tctctctgag atctcatcta ttgcacgagt ttgatcattt gttaattgaa
3421 acgacaattc atttaaaact tgctctttgt atttactcaa tattataaat tctcttcccc
3481 tttctttcac atgatttttt cttgcaagtt tcagcgctag ctgatacgca aacaattcat
3541 cataagcaag ccttttttcta caaacctcag cttccaccaa tgagcttggt ctgtgcaact
3601 ttatgatgct ttccctccaa cttaaccatt tttttgctt gattaatgta tcatctatcc
3661 actctggtaa atcaggcaat tcctttagat tagagcttat tatgttccca atgcgcttat
3721 tggtaatgcc acggcataat tggtaaactg gctctatgca agctattttt ttaaattggt
3781 tgatgtcaag cgacacgtaa tctggatgag taatttgcca atgttcagca aatttttcga
3841 gtttgccact gatgatcaca ttcgctccaa ttggaaatag tttatataaa tacttaactg
3901 agtaattaaa aaagactatg aatatatact gactctcact ttcaacgact attttatatg
3961 gtctacctct aaaagtgggc gactgatgct cataaacttt tgcaatgaaa gttgtaaatt
4021 ctccaactcg agcatcagat agtaacttgc ttctatccac ataactaagt ggcttataaa
4081 acagcagatc caccaccttg tccccaccac aaagtttagg caatattgcg gaatggaact
4141 taggtatatt ctccagcctg ctatttagaa aaatctgatt gctcattgag attcatacat
4201 gtgtaaaact gttatagagc ctttttaaaa tcttgtcacc ttatggtcag gtatgcacta
4261 tagctagcac agctctctaa cgttataccg cgattcattc gcggtatctc agccgctaac
4321 acgtaacgga atgacgaatt tgttgttttt aaaatcatcg gtaagcctaa gttacccccta
4381 actatatttg ctatcatagg gcttatagta ggtattttat taacttatta atcattttta
4441 tgtattatta tactatcagc agtctatatc aaggtattca taatgagtag ctctatatca
4501 gaagaaaagg aaaatccagt agttacatta agagctcaag cagaaaagtt tggttttagc
4561 aagttaagag ctggtaaaga caatgctgaa gcgaattctg gtaatacagc gttcttagat
4621 tttcagagaa tggattttgt tattaacggc aaaagtataa gtagagattt gattgccgag
4681 ctatacaaag aacatgatag tttactgcca aaaagtgaag gtaaaaatga agactaccgc
4741 ccatttgcaa gaaaagtttt taaggagatg ttcaaatacg ctaaggcaga agttccaagt
4801 gactctatct tggaagaatt agttatcaat tgtaatcaag caggatatga agctggtgta
4861 catatagagt ttcaaggtgc acttagccag cattcattca taatagaaag acctgaaaaa
4921 gtgataagta ttgactgtgt tagccaaaat aatgtgagtg ttaaatctga tatgacatta
4981 ccaatatttt tccaaaggag tgacggtaca ttcggtgaca aggtgtatga tctatccagc
5041 tcactagaat tcacgcttaa gtctcaagat gataaaaatg taacatacac aaatggtaac
5101 ttgtcactta ctgttcctga gagactgaag gactataata taggtgataa aagcttattt
5161 gacattataa aagaatactt tcagaaattg tgcgagaggc ttggattttt taaaataaaa
5221 atagagcatg gtttaggcga tccactagaa gtaagcgacc atctggaaaa tatggaaccc
5281 cctattcgta gtaatgagca cggcctgaat taaaatatct tgagcttgtt tgcacaatca
5341 atcacttcag catttagaaa ttgtttttta aaccaggtat actgacgttt cgcgtagtgt
5401 cttgtgtttg tttgagcaat ttgtatcact tcgtctaaag taatctcacc tttcaagtac
5461 tttataattt ctggcactcc gtgtgctttc atagctggca aatgtggggc taggttcatg
5521 ctaagtagct tttttacttc atcaattgct ccattttcaa tcattgtaat gaaacgagaa
5581 tttattttttc ggtatacatc ttcacgttta ggcagaattg tatatatctt aaaattattg
5641 aataaaggag gctgtctatt ctcttgccat acaaaaattg acttgccagt ttcagtgata
5701 acttcaagtg cccttgaaag gcggtgcgag tcattcatga acactttacc ttgaatcctt
5761 ggatctttgc ttagcgctaa tttgtaaaac tcctctttac ttaaattttt cctgagctca
5821 cttacgtttt ttctcacttt ctgacttatt tgtggcattg atgataagcc tttgattaaa
5881 ctgctgatgt aaagcccgct tcctccagtg atgataggta tttgagcatt ttctaatgcg
5941 tgattaactt ctttttctaa atcttctaac catagaccca cggaataatt ttcttttgct
6001 gaaacataac catatagttt ataaaatact tcttgctttg gtggttgagc agtaattatg
6061 ggaatttctt catataccctg ctttgaatca caatttatta tactaatatt tccatatttt
6121 tctatcaggt tatcgcataa ttctgattta cctgaggccg taattcctgt aataattact
6181 atattatttt tcatttatat taatttaatt aacatatgat aaagtgtagt aaaagtttgg
6241 attctacaat ctaaagtttg gagattttta tggcagaaaa caacgacaat aattcattta
6301 ctaagcgttt cacaaacagg actcctggcg gtacagggga aggtttttcc agtaagtatt
6361 cgtctcaaaa caccaaagaa cgggcattag gggaaaaagg aaaaatttct gaattagcag

6421 gcaaaaaaac cgaatccaag gaagcttttt ctgaaagtgg cggcataaaa agtaaaagca
6481 gaactgtcaa tgaaagatct tttgctgatg caacaaaaag aagtaggtca gatcttgtat
6541 acttagtccg tggtaaagat cgtggaaaat cagcgtggca ttatgtgtta gttgataaag
6601 agaaaagaga aatgtttcta gcaaaaagca gaactggctc tatagatgtt gcagactatg
6661 gagagatttt gtattcagga tggggagagg atccaccaca ggcaataatt gataaagtca
6721 atgaagagtt tgggttgtag caaatctgca taacaaatgg taagctgcgt tgcattacgt
6781 ttacaaaaac tagcagttac tatataagat acagtgcaac aaaactgatc tagttgttat
6841 gaataagttt ttagctatat ggtatctaaa ataataaatg caatacagaa caatttatta
6901 gtgtgttttc caacagaaac agtgtatgct cttgcttgta atgcactaga taatgaatct
6961 ataggaaaaa tatatcagat aaagaaacgc tctcaaaata aaccactgtc tatatttgtt
7021 agtgatattt acaatttgac aaaaatagca aaggtaaagg aaagatatat tgatttaata
7081 aaccactttt ctcctggacc aattacctat attttaccac ttaaaaacaa caacatattg
7141 ccaaacgaat tctttaaaga tagtataggt atcagaattc ctgagcatcc tattgcaatt
7201 tcaatactaa acaaactgaa agttccaata gttgcaacta gtataaatat ttcaggagaa
7261 aaaagtgtat gtaaggcaga cgatatacca caatccatta agcaacattt atctgcagtg
7321 attgaagatg acgaattagt ttctggtata gaatctacta ttattgactt aaccgaagat
7381 aagattaagg ttttaaggga aggtgcaatt ttattacaaa cagtaaacaa tgcattttca
7441 aactaagatc tataggagaa agaatgaaaa aagtaatagg tcacaaccag gccaaaaaaa
7501 aattaatgaa caacttatct gttcagtctt ggctgatctg tggtaaaaaa ggtataggaa
7561 aagcaacact tgcaaaatcc ttctccaatt ggttactcat aaaaagctat aatgaagtag
7621 cattggactt acacattgtt gaaggtgata ctatcggaat agaaaaagtc agagaaatga
7681 aaaactttct acacttaagt cccattcaat cagaacacaa aatagctata atagatagct
7741 tagaggcaat gactaataac gctaaaaacg caatattgaa aatattagaa gagccgccaa
7801 aaaattctaa gatatttata attagccata agccatatga tatacaaacc actatcttgt
7861 gtaggtgctt tcagctgaat ttactcccat taacttatga tgaaacaaag caagttgttt
7921 catctcaatg taaatttgat gatcaaatat ttgatgaaat cattacttta tttcctggaa
7981 tgccaggaat gataataaat gcaataagta gtgatgctta tgaagcatac aagtgctttt
8041 ataaatttct tcatgattta aagaatccca atatgattaa taaagtaatt aatagcgaaa
8101 tcgaactaga actagcatca tatataattc aggccttcat tttagaaagc ataaagagag
8161 aggtaaatga tgttgaaatt ctgctaagtc agtggaaaaa aatagatgaa ctttttgttg
8221 ttgctaagca actccactta gataaaaagc atgttttagc taatgtagtt aatatcatga
8281 cataagttta caaagtataa tacttttaac ataaattcat gataaaatcc tgttaattta
8341 atacttgaag tatataattt gactgataat aaattaactg tatggcaatc aatagaagtt
8401 ttgacgaaag ctatactgaa tatgcagaaa gaattgagtt caatcaagaa tatagcttta
8461 gaccaggacg tcaccttaga aaaaggaaag gagttattaa gtggcatcta tgaggcaaat
8521 tttaagctaa acaaggcaat caatatagcc actttgccaa aaattggcta tcatatgcta
8581 aatggtgagt ttgttgtacg taatcatatc acggaagagg aagtaaggat tcctagagat
8641 tttcattacc ttaaagtcgt aaaatccgat catgataatt ataaattaac gttttgtaat
8701 tttttaggta atgagttttt tgaatataaa aagtatgatc cacaatattc tggcgtttca
8761 gatgaatata agtttgtgga ttttggtagt gtaaaaaaga ctcataatct aaaatttaag
8821 gaatacgttg gtcatgcacc taaatttttt gctgcagaag ggcctattga gcctggatcc
8881 caaaaccatg taattgatct atttgagcta gtcaaaagtg gtaaaggtag aaaagtggga
8941 acctttgctg atgaatttgg ttattttgat gatcaaaata aattacatta ttataattat
9001 cacaaaagtg tagaaagtaa tacttatgat cctgaaaatt tcagtgtaaa aatgataaat
9061 atagatgtga gcaaaatcga taaattctac ctcattgcag aacaaggtga tataatcatt
9121 catcctattt gggaaaattt agacatattt taaagttttt tttagagtat ctaagcttat
9181 tgttattata atgatccgat taggtctggt agtattgtgt tatggaagat aaattgcttg
9241 aatcagtaaa aaacaaaagt tatttcagta aagcagtcga atggtactgc cataggtatc
9301 tgttttgtgt agcagaaaga tcttggatgg cattaatagt atcgcttctc ctagtatgct
9361 tatgtttatt aacattaaac atatacctgt tatttcctgt taaaaaagat ttaaattttg
9421 tgaagtatat gaaccacaca gaagatgagt tctctgcaat gcataagctt agtttcagta
9481 aaaaggaaga tgaatacact tccacagcga ggtacttaat aagtaaatac attgaagtat
9541 acgagtccat taagaccgtt gaaccgaagt accaagaaaa ttttataaga aacaattcta
9601 tacataaaat ccatcaaggc tttcaggaaa aaacgaataa tgaagctgtt tcatccaaaa
9661 gaaaaattac caacataaac gtaataacat tatctattga ccgatcgacc aaggacctag
9721 ttacatttgc tggaaatgct actgtagttt ttacaaccga gcaaaataag gaggatactg
9781 tggagattag cttcactttg tcgaatataa aagcaactct ggctggtata ataccattta
9841 aatttattgt taatggttat aaatacagat tttaaaatat tacttggtag ctaaaaaaaa
9901 taaccaaagt gcatgtagca gtaataactg gaggtaaaac tagaagaatt ggtgtttttt
9961 taataaagta attctgagaa ggtttaaga aaaaggcgtt gtaaattatc ggtaaaaagt

10021 acagagcgtt gagtatagta ctaacaatta acactagagt tacaaacaca gaaagtatta
10081 cattgccaga gttgaaaaca gcttgaaaaa tgagaaattt accccaaaaa gtcggagcag
10141 gcggcacccc tatcatagac aacgcaccta tagcaaatgc tgtcatgcta attggcatac
10201 tgcgtcctat accgtaaatc ttatctatat acttctcacc tgctcttgtt attattgctc
10261 ctgcaacgaa aaacaaagtg atttttgcaa atgcgtggca aactaattgg aaaattgcaa
10321 ccctcatgct gagctcacta aaaattgaag cgaataatat aatatacgac aattgagaga
10381 tggtagagta agcaagcaat ttttttaatt ctttttgctt taatgcaatc aatgatgcag
10441 taataatagt aaatccagca gcgtacggaa gccaccctcc agcaaaccaa ctttgctgcg
10501 caaaacgttg catgttaccg ataccgaaag tgtataatat aacttttatg atgatgaaaa
10561 ctccggattt cacaacagca acagcatgca gtagtgcgct tacaggagtt ggtgcaacca
10621 ttgcttttgg taaccaaaaa tgcattggca ttaatgcagc ttttcctatt ccgtaaatca
10681 gcatagcaaa acacactgca atgaaagtga tgagtgaagt atcaaatttg aatattccac
10741 cacttacaaa gtctaaagta tgaaattcat tatataaaag gcctattgca ggaaaaaaca
10801 ataccaaaga agagaaaaat agcacgccaa agtaatagcg ccctgcaatc attgattcat
10861 ttgttgagtt gtaagtaacc agagggtatg tactaatagt tagaagctca taaaagacaa
10921 atgtagtgag caaatctcca gaaaaagcaa taaacattgc acagctaatt gatattgaaa
10981 aaaagcatag aaaacttgag tgattgctat cagcatagtt attctgcata taacatattg
11041 catatatact ggtcagcacc cataaaaacg atattaataa actgaatatt actccggtag
11101 actctagctt taaactaatg tgcaaattat taccaaaatc catgaggata aattgagaat
11161 gatcaccata cacccaatat aaagtgcaca gacacgtata cgtaaacaga agtacagaag
11221 aagtgacagt aatactattg ttcactccgg gccatttacc agtaaaaaaa ataactaacg
11281 cacttaaaag tggaatcaac gcagtaatta ataaataatt ctcagtaatt gaaaatggta
11341 aggaaagata cattaagata tagtccatta aatttttgt ttaggtaaag ctagaattat
11401 actaaaatca tagaaaaact gctatgtttt tttagttgtt gcatatgcta tagctatact
11461 gccgcggtat ctctagatcc cgctaacacg tagcgggatg acgaatttgt cgttacgtca
11521 taccgcgatt cattcgcggt atctcatccg ctaactagta gcgggatgac gagcttatcg
11581 tcatgccacc gcgaaccgtc ataccgccgc ggtatctcag catagattcc gctaactagt
11641 agcggaatga cggtttttca aattgttgta agtcggttta gctatataga tagcataaag
11701 tcaactgcat aaattgaaac aatatctgct agacgcaaat tacaacaaat gcagtagcgt
11761 aatctccatc gtcactcaga gaaagatgaa ttttggagat aaaatccttg ctaacggtaa
11821 gatatggctt gcctcttaca tcattgtata tttctatatc tttcattata ataccctgac
11881 cactacctcg tgctttaaca aaagcctcct ttgccgcaaa acgcttagcg aaataccttg
11941 ctcgcatttc ttgactatta tactttctac ttagctctat ttctttctca gtgtagattc
12001 tattgagaaa tttttccccg tatttttgta atatcctcga tactcttggt atatatacta
12061 tgtcagtgcc gataccgtgt atcattttgt gctaaagtga tttattgctt cctcaatctg
12121 catttccttt acctctccag ttgctctatt ttttacttca actatctttt catctattgc
12181 ctttttttccg acaattattt gccacggcaa gcctatcaga tccattctag aaaattttac
12241 tcctacactt ccttccgtgt catcgtaaag cacttcatcg ctcttcaaag ctttgtatat
12301 tttatctgca gcctccgtta ctttcgtttg taaattgatt aaaccaattc tgaaaggagc
12361 caccgattct ggccagataa ttcctttatc atcatgaaaa gcttctatta ttgcaccgac
12421 aagccttgaa actccaatgc catacgaacc catatgtata ttgacatttt ttccatcttg
12481 agaagtaaca cttgccttca taggctttga gtatttgtcg ccaaaataaa aaatatgccc
12541 tatttcaata cctttactaa catttaactg ctcttgtgat atagggcaag tctcaggatc
12601 atgcatatca tctgcaactg cgtatatact cttcaaactt tcaacatctt cgctttctag
12661 caactcagaa aatttgttgt cgtaatataa agtgctttca ccagtgtttg ccaatatatg
12721 aaactcgtgg ctcaaatttc ctccaattgg ccccgtatca gctcgaactc caattggggt
12781 aagtcccatg cgtttgaaga tttttatgta agttttgtac atcaaattat atgaattcag
12841 tgcaccttcg taatctacat cgaagctata tgcatccttc atcaaaaatt ccctgcccct
12901 cataacgcca taacgtggtc ttacctcatc acgaaacttc cattgaattt ggtataaaca
12961 aagtggcaaa tctttgtaac tttttaccat atctctaatt agatcagttg ccacctcctc
13021 gtgcgttgga ccaaaaagca tgtcttcctc atgcctatcc cttatacgta acatttcctt
13081 accgtaatca tcgtaacgcc ctgattctcg ccaaagattt gctggttgga cacaaggcat
13141 caacacttca atagcaccgg atttgttcat ttcatctctg ataatgtctt caatattttt
13201 aagcactaag agaccaagtg gtaaccagga ataaatgcca gatgctatct gctttattaa
13261 acctgcacgt aaagaatatt gatgggagat aattttagca tgagcaggct tttcctttag
13321 agttggtaag taatacttgg ataaacgcat ataatagatc tgattgtatt aaacaatctt
13381 aatacaatca gatctatagg taaacttatt ttgaaaaaga agggaagtgt gtggtgcttt
13441 ccttaacgta ctaataccga tcccctctca aatttagtag ccgctccacc aaacatttta
13501 caacggtcct taacagaaga agctggtagc tttcctgctt cattagtcac tgttgttgca
13561 ggaagagctt caggtttcgt ttttaaatta acaggcttag gcgatatcag tggtttatct

13621 gcctttggcg cagaatgtga gggagtgtta aataaaccat acgcgtcaag ttaaggaatg
13681 tagagagctt aaagtagata ccctggtctt cctatatctg tctttcaaag aaaactttga
13741 ccagattata gtaagctttt ttagaaaaat ttttaaatta ttgacaatat tgtttaatat
13801 caaaaagagc tctctaaccc gccttttcaa ttcaataaat gcctttgtta aactgagctc
13861 cgtgttcttt tttagtttta cacagccaat cattccgtga aatacgagaa tagcacataa
13921 ttttgcgtat agctcgcaca atactctaaa tggatttgtc tttcctttta gtttatcaag
13981 attgatatga cttttatata gtttaaatag taattcgatt tgccatctta ctctataaac
14041 cgtcaatatt tgttcagcat taattttact ctctggaaca ttagttataa atattgacca
14101 atctaataac ttttgattct tttgagaaga tgtatatcca tgtgactttg ctaatttatt
14161 agcctttctt cttctagcca tagactgttc ttcagttagc ttttgacata taattcttac
14221 cttaattttt acttcctttc ctaataatac ttctttttct gaagataatt gaccttccaa
14281 atactccaat agctctattt tttgatttgt ttctatatca tatatattgg tatcagactt
14341 ataacgacta acaaaatatg cacctgcttc atcaatctgt ttaaaagaac taggcacaaa
14401 gtagcccaag tcaaatatta gcaaatcatt ggctgataaa ccgttcagat aatccctata
14461 accttgatcc gaccttattc cttccataaa atttaactta tctagcgctt gattcaggta
14521 atcaaagact aactgtagct ttattcctga tttggtatta ttctcacaat ctctatagct
14581 actgccatat cctttgtaca tatcttccat gctaatgggc aggctaatgt agctgctatc
14641 caataattta acgcttccaa attgctgtaa aattttgcaa tcaatctgta gacttttttt
14701 aaataaacac aaagcttcgt tatacatttt tttcatgaac tcaactgctt cttcactaaa
14761 cctaaaatct aaaccctgtt ttgtaatttc tatcgagtct tcatttagca attggcacat
14821 tgtttctatg ctgcaatcat caactcctat gttaccaaaa accatagctt ttatgaatga
14881 tgagccatta agttttctct ttctttttat aaaacctact gcaattgata tttcgtctgc
14941 tttttcatta aagaattcat tgaggtcttt tgataagcaa gctattctgt ccattgtaag
15001 ttctctcatt catatatcca agagaattta taccttatta ttttccttca ctctatcaac
15061 tttacttaca cttttcctta aagtgaattt tctaatctgt ctatgcagtc cctcaatagg
15121 attggtagta taaattagcc ttctaactgg gccagaatac ttgaaatagc cggataagtt
15181 ttcccaatta ttctgccatg attttacaac caatgggtac ttttctcccc atttttcttc
15241 caactcaagt agataatttt cagctatttc tctacttgaa gcctgatata cttttttttaa
15301 atcactcatg aaaatcttta catctttgct agatacatat ttcagagaat tgcgtatctg
15361 atgcaccaca catagctgta cctccgcatt aggaaacaca ctgtttatgg ctgtaggaaa
15421 gcttttcagc ccatcaacgc aggcaataag gatatcttct actcctcttt cttttagatc
15481 atttagtact cccaaccaga agttagctcc ttcactttca gccaaataaa agcccaagac
15541 ttcttttctg ccattttgat ctatgcccaa tatattgtac atgcatttac ttacgcaatg
15601 cccatcctct ttgactttaa aaaacatgcc atccatgaac actatcggat atacagattg
15661 cagcggacga ctgcgccatt cattgattac tggtagcaat ttgtcagtaa tacttgatat
15721 ctctgctgcc gatattttgt gatcataaat ttcctcaaca tgtgacgcta tatctctata
15781 acccatacca ctggcaaatg tgctcaaaat cttcgtttca agctctggat gtaagtttgt
15841 ttgccttttt ttgactattt gtggttcgaa acttccttct ctatctcttg gtgttaatag
15901 ctcaaatgag cctgcacttg tacgtaaagt ttttgcattc ctcccatttc ttcggttatt
15961 ttcttcgctt tcagccaaca agtggttttc tatttcaccc tctagactcg cctccagcaa
16021 ccttttttata aacggtgtta acgctccgtc ccttcctgtc agcggccttc cttctcgtat
16081 agacgacagg atatttgttt ctaattcttt ataatctacc aatccggtag ttctgtttac
16141 tactttctga ctcattgtca aacctccatt tttttatatc aatttattac ttttttttcg
16201 gtttgacaca gtttatttaa ccatacgcgt caagttaagg aaaagtgtaa gtaaagttga
16261 tagagtgaag gaaaataata aggtataaat tctcttggat atatgaatga gagaacttac
16321 aatggacaga atagcttgct tatcaaaaga cctcaatgaa ttctttaatg aaaaagcaga
16381 cgaaatatca attgcagtag gttttataaa aagaaagaga aaacttaatg gctcatcatt
16441 cataaaagct atggtttttg gtaacatagg agttgatgat tgcagcatag aaacaatgtg
16501 ccaattgcta aatgaagact cgatagaaat tacaaaacag ggtttagatt ttaggtttag
16561 tgaagaagca gttgagttca tgaaaaaaat gtataacgaa gctttgtgtt tatttaaaaa
16621 aagtctacag attgattgca aaattttaca gcaatttgga agcgttaaat tattggatag
16681 cagctacatt agcctgccca ttagcatgga agatatgtac aaaggatatg gcagtagcta
16741 tagagattgt gagaataata ccaaatcagg aataaagcta cagttagtct ttgattacct
16801 gaatcaagcg ctagataagt taaattttat ggaaggaata aggtcggatc aaggttatag
16861 ggattatctg aacggtttat cagccaatga tttgctaata tttgacttgg gctactttgt
16921 gcctagttct tttaaacaga ttgatgaagc aggtgcatat tttgttagtc gttataagtc
16981 tgataccaat atatatgata tagaaacaaa tcaaaaaata gagctattgg agtatttgga
17041 aggtcaatta tcttcagaaa aagaagtatt attaggaaag gaagtaaaaa ttaaggtaag
17101 aattatatgt caaaagctaa ctgaagaaca gtctatggct agaagaagaa aggctaataa
17161 attagcaaag tcacatggat atacatcttc tcaaaagaat caaaagttat tagattggtc

17221 aatatttata actaatgttc cagagagtaa aattaatgct gaacaaatat tgacggttta
17281 tagagtaaga tggcaaatcg aattactatt taaactatat aaaagtcata tcaatcttga
17341 taaactaaaa ggaaagacaa atccatttag agtattgtgc gagctatacg caaaattatg
17401 tgctattctc gtatttcacg gaatgattgg ctgtgtaaaa ctaaaaaaga acacggagct
17461 cagtttaaca aaggcattta ttgaattgaa aaggcgggtt agagagctct ttttgatatt
17521 aaacaatatt gtcaataatt taaaaatttt tctaaaaaag cttactataa tctggtcaaa
17581 gttttctttg aaagacagat ataggaagac cagggtatct actttaagct ctctacattc
17641 cttaacttga cgcgtatggt ttatttaaca ctcccgaatg tgacggagat gtgggttcag
17701 aaccagatcc tataccactg tcaagactcc cgcttctttc tctatggagc gattgggagc
17761 ctgaagatgc ctgctcaata ggttctgggt ctgtctccca attttcatca tcagatacat
17821 tactactatc acttgataca ctactactac gactccgtgg tggtgggatc atagctaaca
17881 ctttactgaa tgccctgccc aggtgacttg gctgttgtac gtcgctccct tgctttccat
17941 taacgcctgc ccttcgtttc tgaagtgctt tactcaaatc agccataaga tcttcatttt
18001 tttcttgttg cccacctcct ttattgcttg acttctttgc tgcctgttcc tttaacttct
18061 tatcacgctc ttccttgctc atgagatttg gtttttgttg tgttatttta ttaaatacag
18121 cgctcatgtc aggtttaacg tcaggatcac taggactact agcattcttt gatgaagaag
18181 ttccatttga acttggaggt ggaggtggtg gcggaggtgc attattattt tgaggaattg
18241 ctgtaccaag tgttagcgga ggttgttgag gagtaaggtt cggaacatgt tccgaacttc
18301 tcacttccgt ctgtgatcca ctatcttgct taacagacaa tccttttcta attagttcac
18361 tgtccagatt attacaattt ttacacgcag atttttgtgg ttcttgagat gcagtatggc
18421 caaattcctg tccattaaga cctgcttctt tcttattatc atctctagaa acggcaagtt
18481 taataagata tgctactgaa gccaatgcaa caaccacaga aacagcaaag attgctaaag
18541 gaactgctgc aatggcacca gaaccagaaa ctgctgcaat aacataaggg agatgtatat
18601 aaattgcaac ggcagataca cataatgata aagcagcaat cgaaatttta aggttagatg
18661 tagacatttt ttacctcatt aaccactaga atactaataa taactagatt atcaacagtg
18721 tcaataatta attatgaaaa ataaagtcaa aatttgaata gataaactat aagtaatata
18781 caaccggcaa tagctataca atagacttct tgcatagaaa aaaggcaaaa tagctccaag
18841 ttgtatccgt ttaggaaagc ggtaaaataa ggtagacaag gtagtataaa aagataacca
18901 tagagtaaaa gtgagtttac aacaaatagt gtcatgcaag tagctgatac tggaatctag
18961 tctttattat gcagccactt atttaaagtt aagttttctg gatccaagta gtcaaggcac
19021 tgggatgaca ggaggctact ttcatgacac cattcttttt cctggatccc agtgtctggg
19081 cactgggatg acatcatagg gacactggga ttacaccctg acaatcgtca tccagctact
19141 tgttagcggc taagagatac cgcgaatgaa tcgcggtatg acggttcgtg gcggcatgac
19201 gataacctcg tctcactgcc attaagttaa ggaaaaaaga tagattttat gggataatga
19261 tgtataccaa taagtttaaa ggtagtatga gtaaaaaagg cagtaataaa atttattcaa
19321 aatgaattgg caagtctaga atttatagaa tcccataaat catcttcaaa agattttatg
19381 agaaagagga agttatcttt tattgacata tttatcttaa ttttaagaaa aagtgtaaag
19441 tcgctgcaag taatactaaa cgaatttatt ttatatagga aaaaagacta taccgttact
19501 gctagtgctt ttagtcaagc aagaaaaaaa atgaaacaca gtgcattttc agaaattaac
19561 gaaggtgtgg tttccttgta ctaccaagac caaaaattta aaacttgttt tggctttaga
19621 gtgttagctc ttgatgcctc caaaatcatt ttaccaacaa gcgttgaaat aaaaaatgaa
19681 tttggctcaa gaaaaatacg taaccaaaaa ccaaaagatt taggtgatta tgcaagtgca
19741 acctttgcgg tttgttaaga tgtgctaaat aatgttgcca taaggtcagt gttagataga
19801 ggtaacactc atgaaattaa cttagcagat gaaatgctta aagttgtaaa tactaatgac
19861 ttactgattt gtatatttat cttataaatt tctttctagc cttacaaaaa gaaaaattgc
19921 ttatgtcatt cgatgtccca gatcatcttt taatgaagtg aatgctatgt ttgaagaaaa
19981 aagcccatct agtgttgagg ttaccatatg tgctcagcgt aagataaaac aagaaaatag
20041 tggaataacg cttcaaagtg gtactgccat ccggtgaaat tgaagtgtta gctacatcat
20101 taatggatga acaaaagttt caaactggag gatttaagga gttatatttt ttacgctggg
20161 gagttgagac atttttttgca aaactcaaag gaagactaag tctagaaaat tttacaggaa
20221 aaagtgttga gtctgttaaa caggattttt ggtcagctat ctttattagc aacctagaaa
20281 gtgttatgac agaagatgta gaagaagcgt tgaatatgga cttaaccgat gataagctta
20341 agaggagcat taacaaatct gtctcattta acgcgatcaa aaacttggct ttcgacattt
20401 ttgctacgga atcagatact gactgcatta tggatcaatt atcaaagcta tttttgatga
20461 acactttggc ggtaagaaag ggaaggaaag ttgatcgtca taaggtacct tatcttcgtt
20521 cgttcaatta ccaaaaaagg gttagaaaac acatattttg actagaggta gtattcatat
20581 cttctgataa taactgggta gcttattgcc atagctcctg taaaatgact tctaaacaaa
20641 ctatttctta agtaatttac acttcagagt ttatctgctt taaagtttac tctattctgt
20701 ttcagcattt gtcataaatc cttaattatt tctgactttt tagttcagtt tttaaccatt
20761 ctttccttaa cttaatggca gtgaacctcg tcatcccgct acttgttagc gggatctctt

```
    20821 gttagcggct gagataccgc gaatgaatcg cggtatgacg gttcgcggcg gtatggacgt
    20881 aagaaaacct ttcttgggtc agctatagag actctcatct ttagcgagct tcaaggtaat
    20941 tggttgctat ttgcgaaaat attcgcataa tttaagtaac attacttaat aaaaattaac
    21001 tatggagctt aataagattg cagcatcgat tttactttct gggctaatca ttatgatagt
    21061 tagtaatgta gttgatatgc tttacagtcc agaagaatat aagattgaac accaaacgat
    21121 agtagctgct ggcagcaatg agttccagca aaaaatcgag caagtggcgc ttgatattgg
    21181 agcgctcatg cagaatgcta gctttgagaa aggtaaatca gcagcaaaaa aatgtatagc
    21241 ctgccatagt tttgagaaag gtggaatgaa taaagtagga ccaaacttat ggaacatagt
    21301 tggaaataaa aaggcccatc ttggtaactc attcaattac tcaaaagcag tgcttgacag
    21361 aggtggaaaa tgggggtatg aggagctatt tgccttttta aagaacccaa aagcctatat
    21421 aaaaggtaca cgtatggcat ttgcaggtat ttccaatcca caagaaattg cagatctagt
    21481 tagttatttg catcaattga gtgatagccc ggttgctttg ccaaagtagt atggattatt
    21541 gtaaattgtt tgatggaaat aatggtcaca tagcatatcg aaagctacaa ggaaagaaag
    21601 cttctgtagt ttttttcggt ggatttgcat ccaatatgga tggaactaaa gcaactgcta
    21661 tttacaaatt ttgccaagaa aatgacgtag cacttgtgct atttgattat tttggtcacg
    21721 gtcattcaag tggtaatttt attgattaca cgataagtga ttggcaaaaa aattgtgcta
    21781 aagtaatgag tgaattaact agcgataaac aaataattat aggttcaagt atgggaggat
    21841 ggctgatgtt gctcactgcc cttcaatttc cagaaaaaat tgcagcacta attggtatat
    21901 cgtctgctcc cgatttttact gaagatttga tattcaagca attgtcaggc aagcaaaaag
    21961 aagaactaga ttctaaaggt gtaatagatt ttacttcaga gcattgtaca tacaaaataa
    22021 ctaaaaattt gatcgaagat ggcagaaaga accttctttt aaacaaaaag acaatagata
    22081 taaactgtcc tgtgcgcttg ctgcatagca ttaatgataa agatgttcct tatcaaactt
    22141 cattgaattt agctgaaaaa attaagtcaa ccgatgttga agtacactta ataaaatcag
    22201 cagagcacaa tatgtctgac aatcattcac tggaagtttt atttaagacc attagagaat
    22261 tttgtaacca gactgcattc tgtgattttt agcaaaacac gatttttcat ttgagctttc
    22321 ttcaacttaa aataaataca gaaaattgct gcctatagct aaactgactt agatttaccg
    22381 acaatttgaa aaaccgtcat cccgctacgt gttagcggct gagataccgc ggcggtatga
    22441 cggttcgcac catcacactt tggaacctta cagata
//
LOCUS       JAQZAU010000049           38835 bp    dna     linear   ENV 17-MAR-2023
DEFINITION  MAG: Wolbachia pipientis isolate wEsol contig_33092, whole genome
            shotgun sequence.
ACCESSION   JAQZAU010000049  JAQZAU010000000
VERSION     JAQZAU010000049.1
DBLINK      BioProject: PRJNA929258
            BioSample: SAMN33142973
KEYWORDS    WGS; ENV; Metagenome Assembled Genome; MAG.
SOURCE      Wolbachia pipientis (insect metagenome)
  ORGANISM  Wolbachia pipientis
            Bacteria; Pseudomonadota; Alphaproteobacteria; Rickettsiales;
            Anaplasmataceae; Wolbachieae; Wolbachia.
REFERENCE   1  (bases 1 to 38835)
  AUTHORS   Fiutek,N., Couger,B.M., Roy,S., de la Torre,J.R. and Connor,E.F.
  TITLE     Genomic assessment of the contribution of the Wolbachia
            endosymbiont of Eurosta solidaginis to gall-induction
  JOURNAL   International Journal Molecular Sciences
REFERENCE   2  (bases 1 to 38835)
  AUTHORS   Fiutek,N., Couger,B.M., Roy,S., de la Torre,J.R. and Connor,E.F.
  TITLE     Direct Submission
  JOURNAL   Submitted (10-FEB-2023) Biology, San Francisco State University,
            1600 Holloway Ave, San Francisco, CA 94132, USA
COMMENT     ##Genome-Assembly-Data-START##
            Assembly Date          :: JUN-2019
            Assembly Method        :: FLYE v. 06-2019
            Genome Representation  :: Full
            Expected Final Version :: Yes
            Genome Coverage        :: 100.0x
            Sequencing Technology  :: Illumina HiSeq; PacBio Sequel
            ##Genome-Assembly-Data-END##
FEATURES             Location/Qualifiers
```

```
source          1..38835
                /organism="Wolbachia pipientis"
                /mol_type="genomic DNA"
                /submitter_seqid="contig_33092"
                /isolate="wEsol"
                /isolation_source="galls on Solidago altissima"
                /host="Eurosta solidaginis MN-2018-EFC"
                /db_xref="taxon:955"
                /environmental_sample
                /country="USA: Cowling Arboretum, Carleton College,
                Northfield, MN"
                /lat_lon="44.48 N 93.13 W"
                /altitude="280 m"
                /collection_date="2018-11"
                /metagenome_source="insect metagenome"
                /note="metagenomic"
gene            complement(<..211)
                /locus_tag="wEsol_00445"
CDS             complement(<1..211)
                /locus_tag="wEsol_00445"
                /codon_start=1
                /transl_table=11
                /product="hypothetical protein"
                /pfam="Ribosomal_L1"
                /tigr="TIGR01169 rplA_bact 227 ribosomal protein L1"
                /translation="MNTYNGNPKQCLEKIVESASAKFNESVDVAVNLGVDSCKSEEQV
                RGTVVLPKGIGKNIKVAVFAQDKHLL"
                /cog="RplA COG0081 228 Ribosomal protein L1"
                /besthit="qcoverage 100 , hcoverage 32.258064516129 ,
                similarity 100 , identity 100 , evalue 2.17e-40 ,
                alnlength 70 , Bacteria , Proteobacteria , WP_209452367.1
                50S ribosomal protein L1 [Wolbachia endosymbiont of
                Ceratosolen solmsi]"
gene            complement(216..656)
                /locus_tag="wEsol_00446"
CDS             complement(216..656)
                /locus_tag="wEsol_00446"
                /codon_start=1
                /transl_table=11
                /fullproduct="qcoverage 100 , hcoverage 100 , similarity
                100 , identity 100 , evalue 6.05e-95 , alnlength 146 ,
                Bacteria , Proteobacteria , WP_015588765.1 MULTISPECIES:
                50S ribosomal protein L11 [unclassified Wolbachia]"
                /cog="RplK COG0080 141 Ribosomal protein L11"
                /pfam="Ribosomal_L11_N Ribosomal_L11"
                /tigr="TIGR01632 L11_bact 140 ribosomal protein L11"
                /product="50S ribosomal
                protein L11"
                /translation="MSVVVAKINLLMEAGKAVPGPKIASVLGPRGIPVPKFCEAFNKV
                TSAANANYKVGDLVTVRISIKDDRSHDFTVSGPPVAYLLKQEAKLSKGSGNPGKELVA
                KLPMSAIIKVAKCKMVDMKVDNEDSAVKMVVGTAKSMGIEVVEG"
                /besthit="qcoverage 100 , hcoverage 100 , similarity 100
                , identity 100 , evalue 6.05e-95 , alnlength 146 ,
                Bacteria , Proteobacteria , WP_015588765.1 MULTISPECIES:
                50S ribosomal protein L11 [unclassified Wolbachia]"
gene            complement(659..1495)
                /locus_tag="wEsol_00447"
CDS             complement(659..1495)
                /locus_tag="wEsol_00447"
                /codon_start=1
```

```
                     /transl_table=11
                     /pfam="NusG NusG KOW"
                     /fullproduct="qcoverage 100 , hcoverage 100 , similarity
                     100 , identity 99.6 , evalue 7.91e-206 , alnlength 278 ,
                     Bacteria , Proteobacteria , WP_015588764.1 MULTISPECIES:
                     transcription termination/antitermination protein NusG
                     [unclassified Wolbachia]"
                     /translation="MECEYKWYIIKVDYGYEQNIRELVSNAVYFKEVFIPYQTVCNSK
                     SDIKELCEVYVYMHLCDESKNILNQTPGFCSGESGDFEMISDDEISLKCKEFNRYKWY
                     ILRVASNCEEKVRQHILENSMRLGVNDYFKEVFIPYEELSEIELKSKKIATRRKCFPG
                     YVFLYVNLCDEVLNFINNIPKSLKVYGFLKNGNIPKVILDDEIHSMCNALYNAQETKK
                     LGYGYEKGEKVKINDGLFQNFTGKVNMVNDERKIINVEVSILGKPTIIELDLAQVEKI
                     ED"
                     /product="transcription
                     termination/antitermination protein NusG"
                     /besthit="qcoverage 100 , hcoverage 100 , similarity 100
                     , identity 99.6 , evalue 7.91e-206 , alnlength 278 ,
                     Bacteria , Proteobacteria , WP_015588764.1 MULTISPECIES:
                     transcription termination/antitermination protein NusG
                     [unclassified Wolbachia]"
     gene            complement(1791..2963)
                     /locus_tag="wEsol_00448"
     CDS             complement(1791..2963)
                     /locus_tag="wEsol_00448"
                     /codon_start=1
                     /transl_table=11
                     /fullproduct="qcoverage 100 , hcoverage 100 , similarity
                     100 , identity 99.7 , evalue 1.77e-279 , alnlength 390 ,
                     Bacteria , Proteobacteria , WP_141456826.1 elongation
                     factor Tu [Wolbachia endosymbiont of Carposina sasakii]"
                     /cog="TufB COG0050 394 GTPases - translation elongation
                     factors"
                     /pfam="GTP_EFTU MMR_HSR1 Miro GTP_EFTU_D2 GTP_EFTU_D3"
                     /tigr="TIGR00485 EF-Tu 394 translation elongation factor
                     Tu"
                     /product="elongation factor Tu"
                     /translation="MTAIVEAFGKPHVNVGTIGHVDHGKTTLTAAITKHYGNFVAYDQ
                     IDKAPEERKRGITIATAHVEYQTEKRHYAHVDCPGHADYVKNMIVGAAQMDAAILVVS
                     GVDGPMPQTREHILLAKQVGVGYIVVYINKADVADTDMIDLVEMEVRELLSKYGFPGD
                     EVPVVVGSALKALEDDSSEYGKKSIDKLMEKLDEYVAVPPRPVDLPFLLPIEDVFSIS
                     GRGTVVTGRIEKGEIKTGEEIEIIGLKATQKTICTGVEMFKKLLDKGSAGLNVGILLR
                     GTKREEVERGQVLAKPGTITPHRKFKAEVYILKKEEGGRHTPFFANYQPQFYLRTTDV
                     TGSIKLLDGKEMVMPGDNVSVEVELQVPIAMDKGLRFAIREGGRTVGSGVVSEILE"
                     /besthit="qcoverage 100 , hcoverage 100 , similarity 100
                     , identity 99.7 , evalue 1.77e-279 , alnlength 390 ,
                     Bacteria , Proteobacteria , WP_141456826.1 elongation
                     factor Tu [Wolbachia endosymbiont of Carposina sasakii]"
     gene            complement(2975..5050)
                     /locus_tag="wEsol_00449"
     CDS             complement(2975..5050)
                     /locus_tag="wEsol_00449"
                     /codon_start=1
                     /transl_table=11
                     /fullproduct="qcoverage 100 , hcoverage 100 , similarity
                     100 , identity 100 , evalue 0.0 , alnlength 691 ,
                     Bacteria , Proteobacteria , WP_141456828.1 MULTISPECIES:
                     elongation factor G [unclassified Wolbachia]"
                     /cog="FusA COG0480 697 Translation elongation factors
                     (GTPases)"
                     /pfam="GTP_EFTU MMR_HSR1 GTP_EFTU_D2 EFG_IV EFG_C"
```

/tigr="TIGR00484 EF-G 691 translation elongation factor
G"
/product="elongation factor
G"
/translation="MEIGISKYRNIGIMAHIDAGKTTTTERILFYTGKQNRIGEVHDG
AASMDWMEQEKERGITITSAATTCFWNDHRVNIIDTPGHVDFTIEVERSLRVLDGAVA
VFDGVAGVEPQSETVWRQADKYSVPRICFVNKMDRIGANFYRCVDMIKTKLGAAPLVI
HLPIGSEKDFKGIIDLISMKAIIWQEETLGAKFSYEDIPSDLLDKAQEYRNLLLDAAA
EMDDEAMNTYFESNDLPVDLLKKCVRNGTIKGKFVPVLCGSAFKNKGVQPLLDGVVDF
LPSPIDVDVIVGIDPKDSEKKIEVKPSEKEKFVALAFKVMTDKFVGSLTFIRIYSGKL
KSKSTVSNALKNETEGIGRMLLMHANNREDITEARAGDIVALVGLKKTTTGDTLCSVD
FPILLERMEFPEPVIEIAVEPKTTSDQEKLGIALNRLVAEDPSLRMSVNAESGQTILK
GMGELHLEIIVDRMKREFNVEANVGAPQVAYRETITKSVEIDYIHKKQSGGAGQFAKV
KIKFEPLEPGSGFQFESKIVGGAIPKEYIPGVENGLELIKEGGVISGFPLIDFKATLL
DGAFHEVDSSPLAFELAAKGAFKEMANKAGPKMLEPIMKVEIITPEEYMGDVMGDINS
RRGNVADMLDLGNNSKIITASVPLANMFGYINVLRSMSQGRAQYSMHFSCYEQVPQYV
VDELKLEYN"
/besthit="qcoverage 100 , hcoverage 100 , similarity 100
, identity 100 , evalue 0.0 , alnlength 691 , Bacteria ,
Proteobacteria , WP_141456828.1 MULTISPECIES: elongation
factor G [unclassified Wolbachia]"

gene complement(5072..5551)
/locus_tag="wEsol_00450"

CDS complement(5072..5551)
/locus_tag="wEsol_00450"
/codon_start=1
/transl_table=11
/fullproduct="qcoverage 100 , hcoverage 100 , similarity
100 , identity 100 , evalue 1.39e-104 , alnlength 159 ,
Bacteria , Proteobacteria , WP_007549313.1 MULTISPECIES:
30S ribosomal protein S7 [Wolbachia]"
/cog="RpsG COG0049 148 Ribosomal protein S7"
/pfam="Ribosomal_S7"
/tigr="TIGR01029 rpsG_bact 154 ribosomal protein S7"
/product="30S ribosomal
protein S7"
/translation="MARRNKAKKREISPDSRYGSVLLMRFINTIMKCGKKSTAEKIIY
DALSLAEKKIGEGGLSIFETAVGNVTPSIEVRSRRIGGATYQVPVEVRQDRAVSLALR
WIAKATSAARKKSGKTTVDCLQSEILDAYNKRGGAFKMCEEKYKMAEANKAFSHLRF"
/besthit="qcoverage 100 , hcoverage 100 , similarity 100
, identity 100 , evalue 1.39e-104 , alnlength 159 ,
Bacteria , Proteobacteria , WP_007549313.1 MULTISPECIES:
30S ribosomal protein S7 [Wolbachia]"

gene complement(5557..5931)
/locus_tag="wEsol_00451"

CDS complement(5557..5931)
/locus_tag="wEsol_00451"
/codon_start=1
/transl_table=11
/fullproduct="qcoverage 100 , hcoverage 100 , similarity
100 , identity 100 , evalue 8.87e-82 , alnlength 124 ,
Bacteria;Eukaryota , Proteobacteria;Arthropoda ,
WP_006279787.1 MULTISPECIES: 30S ribosomal protein S12
[Wolbachia]"
/cog="RpsL COG0048 129 Ribosomal protein S12"
/pfam="Ribosomal_S12"
/tigr="TIGR00981 rpsL_bact 124 ribosomal protein S12"
/product="30S ribosomal
protein S12"
/translation="MPTINQLIRKGRLGLTHKKKVPALGESNPQRRGVCTKVYTTTPR

KPNSALRKVARVKISGYGEVTAYIPGEGHNLQEHSVVLIRGGRVKDLPGVRYHIIRGA
LDLRGVQNRKKARSKYGVKKSG"
/besthit="qcoverage 100 , hcoverage 100 , similarity 100
, identity 100 , evalue 8.87e-82 , alnlength 124 ,
Bacteria;Eukaryota , Proteobacteria;Arthropoda ,
WP_006279787.1 MULTISPECIES: 30S ribosomal protein S12
[Wolbachia]"
gene 7046..7885
/locus_tag="wEsol_00452"
CDS 7046..7885
/locus_tag="wEsol_00452"
/codon_start=1
/transl_table=11
/fullproduct="qcoverage 100 , hcoverage 100 , similarity
100 , identity 100 , evalue 2.75e-202 , alnlength 279 ,
Bacteria , Proteobacteria , WP_010962305.1 MULTISPECIES:
peptide chain release factor N(5)-glutamine
methyltransferase [Wolbachia]"
/cog="HemK COG2890 280 Methylase of polypeptide chain
release factors"
/pfam="MTS Methyltransf_31 Methyltransf_26
Methyltransf_18 CMAS Eco57I"
/tigr="TIGR03534 RF_mod_PrmC 253 protein-(glutamine-N5)
methyltransferase, release factor-specific"
/product="peptide chain
release factor N(5)-glutamine methyltransferase"
/translation="MKTISTLIQEGSELLSSHKVESPHLDCEIIMQHVLGVERSFIIV
NHADQVPMEKELLFWKLTKKRAERYPISQIIGNREFWSKNFIVNQHVLDPRPDSETII
LAVLKYYPNKKQKLKIADFGTGTGCLLISVLSEYEYAVGVGFEKSLEAYKVAYQNTKK
HDLLSRAKIFPSSWTECRGSFDLIISNPPYIKRSKLKDLQAEVQKEPRIALDGGIDGL
SCYLSIFPILRKCLKKNGFAILEIGEDQSNIDKIIPSYELAFQEYVYDLAGMKRCIVV
KRT"
/besthit="qcoverage 100 , hcoverage 100 , similarity 100
, identity 100 , evalue 2.75e-202 , alnlength 279 ,
Bacteria , Proteobacteria , WP_010962305.1 MULTISPECIES:
peptide chain release factor N(5)-glutamine
methyltransferase [Wolbachia]"
gene complement(8050..8649)
/locus_tag="wEsol_00453"
CDS complement(8050..8649)
/locus_tag="wEsol_00453"
/codon_start=1
/transl_table=11
/product="hypothetical protein"
/pfam="Surface_Ag_2 OMP_b-brl"
/translation="MWHLVTQGELGNNSYRAELEGMYSSVKVDNIGLTSSQITVSYLK
ETGEDPNKETYLYSAAVSHDQIENISVMANVYHHWKSDRFSFSPYVGIGIGATRMTMF
EKPSIRPAGQLKAGFDYRINEDVNMHIGYRGFGAIGSDIKLTAKRLGQVVDDPNNDKK
KKLNPSSGSKVTEEINIGNQLFHTHGIEAGLTFHFASKA"
/besthit="qcoverage 96.4824120603015 , hcoverage
71.6417910447761 , similarity 100 , identity 100 , evalue
1.59e-134 , alnlength 192 , Bacteria , Proteobacteria ,
WP_209452369.1 P44/Msp2 family outer membrane protein
[Wolbachia endosymbiont of Ceratosolen solmsi]"
gene complement(8615..8833)
/locus_tag="wEsol_00454"
CDS complement(8615..8833)
/locus_tag="wEsol_00454"
/codon_start=1
/transl_table=11

```
                     /product="hypothetical protein"
                     /translation="MKQKGFTLVVGYYGQYLNNTSVLKTSTTGIKNLSINDRGAQNTE
                     GQSLSEYKGDYNPPFAANVAFGYTGRIG"
                     /besthit="qcoverage 95.8333333333333 , hcoverage
                     24.7311827956989 , similarity 92.8 , identity 89.9 ,
                     evalue 2.16e-34 , alnlength 69 , Bacteria ,
                     Proteobacteria , WP_151807687.1 P44/Msp2 family outer
                     membrane protein [Wolbachia endosymbiont of Nasonia
                     oneida]"
     gene            complement(8778..8900)
                     /locus_tag="wEsol_00455"
     CDS             complement(8778..8900)
                     /locus_tag="wEsol_00455"
                     /codon_start=1
                     /transl_table=11
                     /product="hypothetical protein"
                     /translation="MISKKTLAVTAFALLLSQQSFASETEGFYFGSRVLRSIFK"
                     /besthit="qcoverage 82.5 , hcoverage 11.7021276595745 ,
                     similarity 100 , identity 100 , evalue 1.85e-10 ,
                     alnlength 33 , Bacteria , Proteobacteria , WP_141456832.1
                     MULTISPECIES: P44/Msp2 family outer membrane protein
                     [unclassified Wolbachia]"
     gene            complement(9022..11019)
                     /locus_tag="wEsol_00456"
     CDS             complement(9022..11019)
                     /locus_tag="wEsol_00456"
                     /codon_start=1
                     /transl_table=11
                     /fullproduct="qcoverage 93.8345864661654 , hcoverage
                     93.5532233883059 , similarity 100 , identity 100 , evalue
                     0.0 , alnlength 624 , Bacteria , Proteobacteria ,
                     WP_077188467.1 MULTISPECIES: type IV secretory system
                     conjugative DNA transfer family protein [Wolbachia]"
                     /cog="VirD4 COG3505 596 Type IV secretory pathway, VirD4
                     components"
                     /pfam="T4SS-DNA_transf TrwB_AAD_bind AAA_10 TraG-D_C"
                     /product="type IV secretory
                     system conjugative DNA transfer family protein"
                     /translation="MSNGNHLRNILIGGVVAFSVLEFCFYLSGILFVLFADGPDAVDF
                     KAINPSLTPFPQALWPTIFDHIQYCWHHPELYSLELKITLIISSALPIIVLMIILWNL
                     RERIIEWRPFKKKESLHGDSQWASEKDIRKAGLRSKKGLLLGKDKRGYFISDGFQHAL
                     LFAPTGSGKGVGFVIPNLLFWTDSVIVHDIKLENYEITSGWRERQGQKVYVWNPAQPD
                     GISHCYNPLQWISEKPGQMVDDVQKIANLIMPEQDFWQNEARSLFVGVVLYLLAAPEK
                     VKSFGEVVRTMRSDDVVYNLAVALDTMGKILHPVAYMNIAAFLQKADKERSGVVSTMN
                     SSLELWANPLIDTATASSDFNILDFKKKKITVYVGLTPDNLTRLRPLMQVFYQQATEF
                     LCRKLPSDDEPYGVLFLMDEFPTLGKMEQFQTGIAYFRGYRVRLFLIVQDTEQLKGIY
                     EEAGMNSFLSNSTYRITFAANNIETANLISQLIGNKTVQQESLNKPKFLDLNPASRSL
                     HISEIQRALLLPQEVIMLPRDDQIILIESTYPIKSKKILYYSDTTFTRRLLKQTRVPT
                     QEPYDPNKVLSAAASKDKVNNEGNNVLEGPNSVDYPAETKTEDAVYDESDEFADEDID
                     DEDIDDKFEDDDEEDEDRSDDGFDDEEEENLKMRKLKNDEK"
                     /besthit="qcoverage 93.8345864661654 , hcoverage
                     93.5532233883059 , similarity 100 , identity 100 , evalue
                     0.0 , alnlength 624 , Bacteria , Proteobacteria ,
                     WP_077188467.1 MULTISPECIES: type IV secretory system
                     conjugative DNA transfer family protein [Wolbachia]"
     gene            complement(11050..12042)
                     /locus_tag="wEsol_00457"
     CDS             complement(11050..12042)
                     /locus_tag="wEsol_00457"
                     /codon_start=1
```

```
                     /transl_table=11
                     /fullproduct="qcoverage 100 , hcoverage 100 , similarity
                     100 , identity 100 , evalue 8.10e-232 , alnlength 330 ,
                     Bacteria , Proteobacteria , WP_141456834.1 MULTISPECIES:
                     P-type DNA transfer ATPase VirB11 [unclassified
                     Wolbachia]"
                     /cog="VirB11 COG0630 312 Type IV secretory pathway,
                     VirB11 components, and related ATPases involved in
                     archaeal flagella biosynthesis"
                     /pfam="T2SE"
                     /tigr="TIGR02788 VirB11 308 P-type DNA transfer ATPase
                     VirB11"
                     /product="P-type DNA
                     transfer ATPase VirB11"
                     /translation="MNYAALDTYLEPLQGIFQEEGVNEISINKPKEVWIENRGEVRCE
                     KLEIFDLNHLKSLGRLIAQATEQKLSEEAPLLSATLPNGYRIQIVFPPACEPDKVVMS
                     IRKPSSMQLALDDYEKMGAFSETVTEATDNPVDRHLDLLLRQKKIKEFLEYAVISKKN
                     IIISGGTSTGKTTFTNATLRAIPDEERIITVEDAREIVLNDHPNKVHLIASKGGQGRA
                     KVTTQDLIEACLRLRPDRIIVGELRGAEAFSFLRAINTGHPGSISTLHADSPTMALEQ
                     IKLMVMQANLGIPPDQIIPYIRNVIDIVIQLKRTGGGKRSISEILFTRSASENA"
                     /besthit="qcoverage 100 , hcoverage 100 , similarity 100
                     , identity 100 , evalue 8.10e-232 , alnlength 330 ,
                     Bacteria , Proteobacteria , WP_141456834.1 MULTISPECIES:
                     P-type DNA transfer ATPase VirB11 [unclassified
                     Wolbachia]"
     gene            complement(12044..13540)
                     /locus_tag="wEsol_00458"
     CDS             complement(12044..13540)
                     /locus_tag="wEsol_00458"
                     /codon_start=1
                     /transl_table=11
                     /pfam="Pfam-B_130 TrbI TrbI"
                     /fullproduct="qcoverage 100 , hcoverage 100 , similarity
                     100 , identity 100 , evalue 0.0 , alnlength 498 ,
                     Bacteria , Proteobacteria , WP_015588759.1 MULTISPECIES:
                     TrbI/VirB10 family protein [unclassified Wolbachia]"
                     /translation="MNKERRNNSEDESEIENKVVTVGSNQGHRALMVMILVLLVGVVY
                     YFYFSPSHKEDSEVIKKEETKQNVQEFKGKLEQVPDNVIVPERIITDSLPPLPPLPTP
                     QIVPEIKQIKKEEEKPKETPVSNIPILPKPNFPSSNVISNLPTSFPTIGGGGYPRDRR
                     SAQMLTISGSSGENKAADAILSDTSAQSSKATRVGKLGLMITQGKIIDAVLETAINSD
                     LQGMLRAMVSRDVYAETGDTVLIPKGSRLIGSYSFDSNVAKSRVNINWNRVILPHGID
                     VAISSLSTDELGRAGIAGIVDNKIVSALFSSVALAGVSISSAVIGQKASNLIDALTAM
                     DAVKSITATEIDISSLKGAIVERLEDAIKTEVGKVGDEKWKLGLRAIEKIRNAKSDQD
                     LLRIIKEEIVSVIKSSGASGVDAITSDNIGITLDDVRQLLRQTQKRSKSVYDEAIGKS
                     INDFSKDMRDIVGRYTDKKPTIYVDQGTALKVFVNQDIVFPPQAILNQ"
                     /product="TrbI/VirB10 family
                     protein"
                     /besthit="qcoverage 100 , hcoverage 100 , similarity 100
                     , identity 100 , evalue 0.0 , alnlength 498 , Bacteria ,
                     Proteobacteria , WP_015588759.1 MULTISPECIES: TrbI/VirB10
                     family protein [unclassified Wolbachia]"
     gene            complement(13557..14357)
                     /locus_tag="wEsol_00459"
     CDS             complement(13557..14357)
                     /locus_tag="wEsol_00459"
                     /codon_start=1
                     /transl_table=11
                     /fullproduct="qcoverage 100 , hcoverage 100 , similarity
                     100 , identity 100 , evalue 5.28e-189 , alnlength 266 ,
                     Bacteria , Proteobacteria , AGJ99506.1 Type IV secretion
```

```
                     system protein VirB9 [Wolbachia endosymbiont of
                     Drosophila simulans wHa]"
                     /cog="VirB9 COG3504 265 Type IV secretory pathway, VirB9
                     components"
                     /pfam="CagX"
                     /tigr="TIGR02781 VirB9 248 P-type conjugative transfer
                     protein VirB9"
                     /product="Type IV secretion system protein
                     VirB9"
                     /translation="MNMYRILLVLVLLVSSNLNASINYNEPISVDSRIKTFVYSPNEV
                     FTVVFSQGYYSYIEFAEGEKVKNIAVGDASSWKINPYDNKLLIMPFEVSSRTNMIITT
                     TKKRNYIFDLISRPNYDKYLDTNAKKVDHDYSAEKDISYVVRFYYPQEEGEFDVDLDE
                     VSLPTQMQYTTDKLEKIIQENDTKYNYTYIDEGGNADIVPIELFDDGYLTYLKFRNNN
                     KIPQIFVEGEDKPCKRLLFDDYVIIKGVHKKLFMRYEDDEVEIINRSL"
                     /besthit="qcoverage 100 , hcoverage 100 , similarity 100
                     , identity 100 , evalue 5.28e-189 , alnlength 266 ,
                     Bacteria , Proteobacteria , AGJ99506.1 Type IV secretion
                     system protein VirB9 [Wolbachia endosymbiont of
                     Drosophila simulans wHa]"
     gene            complement(14344..15024)
                     /locus_tag="wEsol_00460"
     CDS             complement(14344..15024)
                     /locus_tag="wEsol_00460"
                     /codon_start=1
                     /transl_table=11
                     /fullproduct="qcoverage 100 , hcoverage 100 , similarity
                     100 , identity 100 , evalue 2.22e-152 , alnlength 226 ,
                     Bacteria , Proteobacteria , WP_015588757.1 MULTISPECIES:
                     type IV secretion system protein [unclassified
                     Wolbachia]"
                     /cog="VirB8 COG3736 239 Type IV secretory pathway,
                     component VirB8"
                     /pfam="VirB8"
                     /product="type IV secretion
                     system protein"
                     /translation="MLRFFKREKSTESLDKDINWNSNRYSTVIAQRNILLLFALILLA
                     TISISILVIFKISTSSTIEPFVIEIDKKSGIVQLVDPVTVKQYSADEALNDYFISEYI
                     KAREVFDPYHYNYNYYTKVRLFSSSNVYNEFRNYIGSQNMDDLFNLYSDTKNEFKIRS
                     IQKLGNDALQVRFFVEFIRKDGSSTRKNKIVIMSYRYASLEMNDQQRYINPLGFQVIS
                     YRVDDEYV"
                     /besthit="qcoverage 100 , hcoverage 100 , similarity 100
                     , identity 100 , evalue 2.22e-152 , alnlength 226 ,
                     Bacteria , Proteobacteria , WP_015588757.1 MULTISPECIES:
                     type IV secretion system protein [unclassified
                     Wolbachia]"
     gene            complement(15097..16179)
                     /locus_tag="wEsol_00461"
     CDS             complement(15097..16179)
                     /locus_tag="wEsol_00461"
                     /codon_start=1
                     /transl_table=11
                     /pfam="Pfam-B_9688 DHBP_synthase GTP_cyclohydro2"
                     /fullproduct="qcoverage 100 , hcoverage 100 , similarity
                     100 , identity 100 , evalue 9.15e-260 , alnlength 360 ,
                     Bacteria , Proteobacteria , WP_015588756.1 MULTISPECIES:
                     GTP cyclohydrolase II [unclassified Wolbachia]"
                     /translation="MFIGNQSSNKVERAISEIRRGRPIVIYDESNYLLFAAAEALERD
                     LFNQYKLTSSNVYVTLTSSKVKYISQNKEHNSKRLLVNNFDELLYLINCSKEDCIKEL
                     QCSKTIDECAIALLKFSELLPYALVADMTFENNHEMRNWCEKNDVIALDTSFINNFQE
                     NQDVYEVCKTSLFLKQTQEVNIISYRTESGGREHHAIIIGNPDKDDEPLVRIHSSCYT
```

GDLLDSLSCDCRSQLHQAIQMIADFGSGIILYLMQDGRGIGLTNKLRAYSMQREHNLD
TVDANRILGFEDDERSFVVAAKMLKKLNINKIQLLTNNDRKLSELESSGIEVTKCLPL
IVERNKYNDSYMETKFGKLGHKLRVF"
/product="GTP cyclohydrolase
II"
/besthit="qcoverage 100 , hcoverage 100 , similarity 100
, identity 100 , evalue 9.15e-260 , alnlength 360 ,
Bacteria , Proteobacteria , WP_015588756.1 MULTISPECIES:
GTP cyclohydrolase II [unclassified Wolbachia]"
gene 16487..17761
/locus_tag="wEsol_00462"
CDS 16487..17761
/locus_tag="wEsol_00462"
/codon_start=1
/transl_table=11
/pfam="Metallophos Metallophos_2"
/fullproduct="qcoverage 100 , hcoverage 100 , similarity
100 , identity 100 , evalue 0.0 , alnlength 424 ,
Bacteria , Proteobacteria , WP_015588755.1 MULTISPECIES:
metallophosphoesterase [unclassified Wolbachia]"
/translation="MNIANIFIFLALSLITSFHFVHAQILYTWSQVIPENKLSIRAIT
DNDMCPIVYVDGEETEMLNRSSINNGDHNETVCELTVQTSAKNISIENLRVPILPKKV
NKIAFIGDTGCRINMLFQQECNSVDSWPLKKNLDSIALHKPDLIIHVGDYHYRQTKCR
NTKKCGDIYGYNKEVWYADWFEPAKDILSQSPFLFVRGNHESCDRAYEGWFRYLDSYP
FSSEKCGNFVSSWSLDAGPMKFFIFDSSSGEDIFTTQSTIDAFERQFDKLIQNSSDKP
MWFLTHKPLWRSPKKEFLTLKSHGNLTQIEAFGDKFPSNVTTIVSGHIHIAQILLMDN
VPDQIIVGNGGASLHAQDQESVYQNVEFDYLNGKNYLAHEVRNFFGFGFAILNLDDHE
FTFYNQHNKEMYSAKLTEDFKLKRIKTIVDSF"
/product="
metallophosphoesterase"
/besthit="qcoverage 100 , hcoverage 100 , similarity 100
, identity 100 , evalue 0.0 , alnlength 424 , Bacteria ,
Proteobacteria , WP_015588755.1 MULTISPECIES:
metallophosphoesterase [unclassified Wolbachia]"
gene complement(17789..19171)
/locus_tag="wEsol_00463"
CDS complement(17789..19171)
/locus_tag="wEsol_00463"
/codon_start=1
/transl_table=11
/fullproduct="qcoverage 100 , hcoverage 99.1379310344828
, similarity 100 , identity 100 , evalue 0.0 , alnlength
460 , Bacteria , Proteobacteria , EAL59173.1 chromosomal
replication initiator protein DnaA [Wolbachia
endosymbiont of Drosophila ananassae]"
/cog="DnaA COG0593 408 ATPase involved in DNA replication
initiation"
/pfam="DnaA_N Kinesin Bac_DnaA AAA_14 IstB_IS21
AFG1_ATPase AAA_25 AAA Bac_DnaA_C"
/tigr="TIGR00362 DnaA 437 chromosomal replication
initiator protein DnaA"
/product="chromosomal replication initiator
protein DnaA"
/translation="MNLTSPKVSTMFFDQIITVTDHNVTWEKIQNCLYNLYGEATYNS
WLSSLKFVSSRNGEVLLSVSTRFIKEWITVHYMKKILSLWQSEDKSIRSIDIQVIEER
NSNFNVILKNREESNHNLGSPLDPRFTFDNFVVGKPNELAFTAAKRVAESIDPILGSN
PLFLYGGVGLGKTHLMHAIAWHIVNSPSAKRKVVYLSAEKFMYQYITALRSKDIMLFK
EQFRSVDVLMVDDVQFISGKDSTQEEFFHTFNALIDQNKQLVISADRSPSDLDGVEER
IKSRLGWGLVADINETTFELRLGILQAKVEQMNMYVPKDVLEFLARNIKSNIRELEGA
LNKVTHTSLIGRSMTVESASETLIDLLRSNHRSVTIEEIQKKVAEFFNIKVADMQSNR

RLRSLARPRQIAMYFAKKFTQKSLPDIGRNFGGRDHATVIHAVKQVENFIKTDSKFAD
EINRLKKMFK"
/besthit="qcoverage 100 , hcoverage 99.1379310344828 ,
similarity 100 , identity 100 , evalue 0.0 , alnlength
460 , Bacteria , Proteobacteria , EAL59173.1 chromosomal
replication initiator protein DnaA [Wolbachia
endosymbiont of Drosophila ananassae]"
gene 19494..20231
/locus_tag="wEsol_00464"
CDS 19494..20231
/locus_tag="wEsol_00464"
/codon_start=1
/transl_table=11
/fullproduct="qcoverage 100 , hcoverage 100 , similarity
100 , identity 100 , evalue 1.64e-177 , alnlength 245 ,
Bacteria , Proteobacteria , WP_015589514.1 MULTISPECIES:
glutamine synthetase [unclassified Wolbachia]"
/cog="GlnA COG0174 443 Glutamine synthetase"
/pfam="Gln-synt_C"
/tigr="TIGR00653 GlnA 462 glutamine synthetase, type I"
/product="glutamine
synthetase"
/translation="MIILSNLSCYAVLGIELEFYIEGIEKEYLFLNSIKNKIASLEFS
CEKENSTHQYEIKSGCYTNSYNLIKHFELAKQSLTETAQKLGGNVSFKAKPYLDRAGS
ALNVHVNLVNSNNGNLFYINEQKYSDYLIHSIGGLCAMMKKHMLFFAPNDDSYLRFQY
PDIHTPTTISWGVNNRTAAIRIPYFGNDFKRCRLEHRVPGADCDLEKVLTAIIEGMVF
GIENKIAPPNRVYGIASDPQYEMERLI"
/besthit="qcoverage 100 , hcoverage 100 , similarity 100
, identity 100 , evalue 1.64e-177 , alnlength 245 ,
Bacteria , Proteobacteria , WP_015589514.1 MULTISPECIES:
glutamine synthetase [unclassified Wolbachia]"
gene complement(20235..20975)
/locus_tag="wEsol_00465"
CDS complement(20235..20975)
/locus_tag="wEsol_00465"
/codon_start=1
/transl_table=11
/pfam="Pfam-B_270"
/fullproduct="qcoverage 100 , hcoverage 100 , similarity
100 , identity 100 , evalue 7.02e-159 , alnlength 246 ,
Bacteria , Proteobacteria , WP_015589513.1 MULTISPECIES:
hypothetical protein [unclassified Wolbachia]"
/translation="MVAILNITKGKNKVIVSGLAGALSALIFLTAIHIAAKIAIIVSA
AAVTYLLLSLIHKIRTKKEQPEITGVSGEKVSEDEGYSSVDEGEEKDTAKEPEKTETE
LEEEIIRKRAQERMEKGSLGGPINAFGEPIHVTIDAENSNPVDYTKTMVVPEEIILSM
LNDKTLTQNNSSSIKNQMKPLPEHMDVPGNPLPGQVLSNLELESKSTQKLSALDLLLQ
CKNVDNDVKKMLKQPITQKGFNGLGFDR"
/product="hypothetical protein"
/besthit="qcoverage 100 , hcoverage 100 , similarity 100
, identity 100 , evalue 7.02e-159 , alnlength 246 ,
Bacteria , Proteobacteria , WP_015589513.1 MULTISPECIES:
hypothetical protein [unclassified Wolbachia]"
gene 21151..22053
/locus_tag="wEsol_00466"
CDS 21151..22053
/locus_tag="wEsol_00466"
/codon_start=1
/transl_table=11
/product="hypothetical protein"
/pfam="ACR_tran"

/tigr="TIGR00915 2A0602 1050 RND transporter, hydrophobe/amphiphile efflux-1 (HAE1) family"
/translation="MQSLLIERNRAVVLLLIVIFIFGSYVYVKMPRESNPDIQIPIIS
VFVGLPGISAQDSEKLLVLPIENELRSIEGVRELRAFATNDGAHMILEFGTEYDNKEV
LDNVRSKLSNIKSKLPVEAESPIINEINLSLFPILNVGLIGNLPERSLTEIARKLKKE
IESLPNVLKVEVAGMRKETVEVIIEPTVLTKYNIQSNEIFQAISSNNRLVGAGSLEND
TGKYSIKISGLLKDIEDIMNIPIRSQGDAVLRIKDIAKVYPRFEDHQGFARINGLPSV
VLEISKRNGKKHNRHSKSSKILNG"
/cog="AcrB COG0841 1009 Cation/multidrug efflux pump"
/besthit="qcoverage 95.3333333333333 , hcoverage 27.9296875 , similarity 100 , identity 100 , evalue 3.49e-183 , alnlength 286 , Bacteria , Proteobacteria , WP_174516802.1 efflux RND transporter permease subunit [Wolbachia endosymbiont of Cardiocondyla obscurior]"

gene 22046..24226
/locus_tag="wEsol_00467"

CDS 22046..24226
/locus_tag="wEsol_00467"
/codon_start=1
/transl_table=11
/product="hypothetical protein"
/pfam="ACR_tran"
/tigr="TIGR00915 2A0602 1050 RND transporter, hydrophobe/amphiphile efflux-1 (HAE1) family"
/translation="MDKAKDQLPENLKVVYLNDQSKNVRDVLDDLENGIIFAVLLILI
IMMLSMGTRIAILVALSIPGSFLIGIIALYFMGITLNIVVLFSLIMAVGMLVDDAIVI
SEYADRKMICGMGKVEAFHTSVRDMFYPVLSSTLTKLAVFFPLLLWPDTVGKFMQYIP
ITIILTLTGSLIMALVFIPTLGAIFGKPSVTSKEEIEKMNAIESGEIKNAGPIIRAYV
RMLEKVLDHPKKFVCATVFVLFSFSVLYFTFGPGVKFFPKVDSDNILISVKAKESLSA
KARDLILKEVEERILSIEKEIHVSYARSGAFSDNVIARIQLELMDWRFRRKAKHILND
IRSSVQDMKGIIINVQEEKPGPSADKPIQINLSGSASSLNLAAEKILEIMNQPSSGFI
NIQDSRSAPEIEWNMSVDKNKATSSEVSVATIGDFIKMVTNGVLIGKYRPNNTDEEID
IVLRFPGKDRNMKTIDNLFINTVNGPYPMSNIVKYAPEKKVNKLNRIDGLRTVTISAD
VDTGYLVDERVKFIQNSIAQDWDKGVKIDFKGDKEDQQKSGAFLLKAFILAITLMILV
LVTQFNNVYHTFIVMTAVFLSTTCVFFVFFLIQQVFVVVMCGVGIIALAGIIVNNNIL
LLDAFHHQIKVHKNDIKRCVINAAISRVRPILLTVATTVLGLIPMITRLNINFFTLQI
TYDAPSSQWWVDISTTIASGILAATVLTLFFTPALLVIQKHEKT"
/cog="AcrB COG0841 1009 Cation/multidrug efflux pump"
/besthit="qcoverage 100 , hcoverage 70.8984375 , similarity 100 , identity 100 , evalue 0.0 , alnlength 726 , Bacteria , Proteobacteria , WP_174516802.1 efflux RND transporter permease subunit [Wolbachia endosymbiont of Cardiocondyla obscurior]"

gene 24315..25877
/locus_tag="wEsol_00468"

CDS 24315..25877
/locus_tag="wEsol_00468"
/codon_start=1
/transl_table=11
/pfam="NusA_N S1 KH_5 KH_2 KH_4 KH_1 HHH_2"
/fullproduct="qcoverage 100 , hcoverage 100 , similarity 100 , identity 100 , evalue 0.0 , alnlength 520 , Bacteria , Proteobacteria , WP_015589511.1 MULTISPECIES: transcription termination factor NusA [unclassified Wolbachia]"
/translation="MIANRKSSVKQKSNKNNIVGSLDVIKTAGELSLQKGLDFDVVIK
ALESAIEAVAHQKYGSKSKIVVDIDRNTGKVTSYRKLKVIDDESNEKENTECESITLT
QAKLIKGDAKVGDTVSELLSLNTDLASARIAQQKIAQIIKDEESKKQYEEFKDKVGEI
RYGIVKQVEYSDLIIDINGTRAYLPLRNLTGGESFREGDKVKAYIQTVKRSDDGRQII
LSRTHEGFLEALLNQEIPEIADGLVTIKGIARDAGSRSKVAVFSPDKNIDPVGACVGV

KGDRIKTIIHELNGEKIDVINYSSDLGQFVIKAITPAEVSKVIIDENENCIELIVAED
QLSLAIGKKGQNVRLASELVGWKIEILSTQQESERRSKELSQCSALFAEALNLEEIMG
QLLVTEGFSSVEDISSASIKELASIEGFNEDIANELHNRANKYLKAENDRKIEELKSL
GMEDDVINLTLSIDNKIALSKHDIKTLEDIADLSSYEFCSILSSSSDSKEDLKDTVDS
IIIEARKKLGLI"
/product="transcription
termination factor NusA"
/besthit="qcoverage 100 , hcoverage 100 , similarity 100
, identity 100 , evalue 0.0 , alnlength 520 , Bacteria ,
Proteobacteria , WP_015589511.1 MULTISPECIES:
transcription termination factor NusA [unclassified
Wolbachia]"

gene 25887..28211
/locus_tag="wEsol_00469"

CDS 25887..28211
/locus_tag="wEsol_00469"
/codon_start=1
/transl_table=11
/fullproduct="qcoverage 100 , hcoverage 100 , similarity
100 , identity 100 , evalue 0.0 , alnlength 774 ,
Bacteria , Proteobacteria , WP_015589510.1 MULTISPECIES:
translation initiation factor IF-2 [unclassified
Wolbachia]"
/pfam="IF2_N SRPRB GTP_EFTU MMR_HSR1 PduV-EutP FeoB_N Ras
Arf Miro GTP_EFTU_D2 IF-2 GTP_EFTU_D2"
/tigr="TIGR00487 IF-2 587 translation initiation factor
IF-2"
/product="translation
initiation factor IF-2"
/translation="MNNEDISDKKLTLQGLSKLKLDLNLNSSASPSTGATVVKKRRKK
SHDAEEHNLFDESKLGSLTEKEQIFRINAVQNAALLKERNQREEKEKTAKEDSNKEID
EKNEADALSKEINKQVLSNTHLVEPKEDSIDHEDNDKKSFKVNKDIYSKHSKLVIAQA
IDEKTEQPPVFKQRFGIRGRKSKFTKGKNISREVIIPDKITIRELSIRMAEDSKSVLK
MFKEEVGESYRVDDLVDPDIACEIVEKFNHTAKRVSDTNREKDLFFIEGRESLPKKPK
PPIVTFMGHVDHGKTSLLDAFRESNVAERESGGITQHIGAYQIITKDKQKITFIDTPG
HEAFTAMRACGANITNIVVIVVAADDGIMKQTVEAINHAKAANVSIIVAINKIDKSEP
GDVEKVMSSLPQYDLIPEELGGDVMIVPVSAKKKTNLDKLEETILLIAELMKLEAIED
CRALGWVIESKIDKAKGISATLIVEEGTLKVGDILVVGTTYSKVRSMVNHLGQREKAA
LPSAPIEITGLNGVPNAGDKFVVVNSEKQAREIVEYRLELIKKKKEDLGDNNLDIFSR
NNSETEELSVVLKCDVTGSIEAISSSIDKLGKDQVKLNILHKAVGGITDSDVLLAEAS
SAVILAFNVKVDSKIRDLAKQKGIEIHTYNIIYELIDDMRMYLTKMLKPVTREVRVGS
ASVRQIFNVSKAGNIIGCYVSDGVIKKDSLIKVVRGGKLVHEGKLKAIRRFKDDVKEV
GVNFECGVSLEGNIDIKVGDILEAYQLVQEERVL"
/besthit="qcoverage 100 , hcoverage 100 , similarity 100
, identity 100 , evalue 0.0 , alnlength 774 , Bacteria ,
Proteobacteria , WP_015589510.1 MULTISPECIES: translation
initiation factor IF-2 [unclassified Wolbachia]"

gene 28221..28619
/locus_tag="wEsol_00470"

CDS 28221..28619
/locus_tag="wEsol_00470"
/codon_start=1
/transl_table=11
/fullproduct="qcoverage 100 , hcoverage 100 , similarity
100 , identity 100 , evalue 1.27e-87 , alnlength 132 ,
Bacteria , Proteobacteria , WP_015589509.1 MULTISPECIES:
ribosome-binding factor A [unclassified Wolbachia]"
/cog="RbfA COG0858 118 Ribosome-binding factor A"
/pfam="RBFA"
/product="ribosome-binding

```
                     factor A"
                     /translation="MKKEIRNLKIASVLHRAISRILMEGKVFNKNVAVSDVKLSKDLK
                     KADVYIVLSSLNIQVADYLDPEKRRTGDPSENNCDLNTVVNEMNQSAWSIRKSILCYV
                     DLRFIPELVFKPDLAFDNFVNVSKILHNHT"
                     /besthit="qcoverage 100 , hcoverage 100 , similarity 100
                     , identity 100 , evalue 1.27e-87 , alnlength 132 ,
                     Bacteria , Proteobacteria , WP_015589509.1 MULTISPECIES:
                     ribosome-binding factor A [unclassified Wolbachia]"
     gene            28623..29480
                     /locus_tag="wEsol_00471"
     CDS             28623..29480
                     /locus_tag="wEsol_00471"
                     /codon_start=1
                     /transl_table=11
                     /fullproduct="qcoverage 100 , hcoverage 100 , similarity
                     100 , identity 100 , evalue 2.72e-196 , alnlength 285 ,
                     Bacteria , Proteobacteria , WP_015589508.1 MULTISPECIES:
                     4-hydroxybenzoate octaprenyltransferase [unclassified
                     Wolbachia]"
                     /cog="UbiA COG0382 289 4-hydroxybenzoate
                     polyprenyltransferase and related prenyltransferases"
                     /pfam="UbiA"
                     /tigr="TIGR01474 ubiA_proteo 282 4-hydroxybenzoate
                     polyprenyl transferase"
                     /product="4-hydroxybenzoate
                     octaprenyltransferase"
                     /translation="MHFNRYFSLMRLHSLTGLWLVLFPSLSGILLASTSLSYQVLFLL
                     VLFTIGAFLMRPAGCIINDIFDRKIDAHVERTKYRPLASGALNIKQALALLSLLLSID
                     LVILLLTNKTTLILGIISMCMIATYPLLKRYVWWPQLFLGFTFNMGSLMGSAAITNQI
                     SIESLLFYIGCVFWTLSYDTIYAHQDKKDDEKLGMKSTALYFGDTTKSWLKRFYLISL
                     MTWLYAGILSSLNNIFYIALLAVGFIFHRQYKNFNPDDPSQCMSIFKNNSYVGLLLFF
                     GILLDRIIT"
                     /besthit="qcoverage 100 , hcoverage 100 , similarity 100
                     , identity 100 , evalue 2.72e-196 , alnlength 285 ,
                     Bacteria , Proteobacteria , WP_015589508.1 MULTISPECIES:
                     4-hydroxybenzoate octaprenyltransferase [unclassified
                     Wolbachia]"
     gene            complement(29799..29975)
                     /locus_tag="wEsol_00472"
     CDS             complement(29799..29975)
                     /locus_tag="wEsol_00472"
                     /codon_start=1
                     /transl_table=11
                     /fullproduct="qcoverage 100 , hcoverage 100 , similarity
                     100 , identity 100 , evalue 5.86e-32 , alnlength 58 ,
                     Bacteria , Proteobacteria , WP_179945710.1 MULTISPECIES:
                     hypothetical protein [Wolbachia]"
                     /translation="MKKKAKEALGLLSALKYWRFLCFKRLTSEIQPLLIATNLRCKCL
                     RNLLSRKKDKESPS"
                     /product="hypothetical protein"
                     /besthit="qcoverage 100 , hcoverage 100 , similarity 100
                     , identity 100 , evalue 5.86e-32 , alnlength 58 ,
                     Bacteria , Proteobacteria , WP_179945710.1 MULTISPECIES:
                     hypothetical protein [Wolbachia]"
     gene            complement(30266..31744)
                     /locus_tag="wEsol_00473"
     CDS             complement(30266..31744)
                     /locus_tag="wEsol_00473"
                     /codon_start=1
                     /transl_table=11
```

/fullproduct="qcoverage 100 , hcoverage 100 , similarity 100 , identity 100 , evalue 0.0 , alnlength 492 , Bacteria , Proteobacteria , WP_015589507.1 MULTISPECIES: UbiD family decarboxylase [unclassified Wolbachia]"
/cog="UbiD COG0043 477 3-polyprenyl-4-hydroxybenzoate decarboxylase and related decarboxylases"
/pfam="UbiD"
/tigr="TIGR00148 TIGR00148 438 UbiD family decarboxylase"
/product="UbiD family
decarboxylase"
/translation="MYNNLRDFIKALEEKKDLIRIKEEVSTVLEMTEIHRRVLSSKGP
AIIFENVITENGKNSIPVLVNLFGTIKRIALGLNINPDELRDLGKLLAFLQSPEPPKN
FKDAIKMFPLLKVVLSMRSKVVSKAPCQEVVLTGDEVDLSLLPIQTCWPNEPAPLITW
PIVVTKGPTKEKQDNFNLGIYRMQVIDKKTTLMRWLAQRGGATHHKRWKEKEQNMKFP
AAAVIGSDPATIIAAVTPVPETLSEYQFAGLLRKKPLELVNCRTIPLQVPAHAEIVLE
GYVSLDRYQDEGPYGDHTGYYNSVEQFPEFNITAITMRKNPIYLSTFTGKPPDEPSIL
GEALNEIFVPILINQFPEIVDFYLPPEGCSYRIAVVSIKKSYPGQAKRIVMGILSFLK
QFLYTKFIIVVDDDINIRDWKEVMWAMSTRMDPVRDTIMIENAPIDYLDFASPESGLG
GKMGFDATNKIPPETKREWGKKIEMSEEIVRKVTEKWEEYMD"
/besthit="qcoverage 100 , hcoverage 100 , similarity 100 , identity 100 , evalue 0.0 , alnlength 492 , Bacteria , Proteobacteria , WP_015589507.1 MULTISPECIES: UbiD family decarboxylase [unclassified Wolbachia]"

gene complement(31832..32911)
/locus_tag="wEsol_00474"

CDS complement(31832..32911)
/locus_tag="wEsol_00474"
/codon_start=1
/transl_table=11
/fullproduct="qcoverage 100 , hcoverage 100 , similarity 99.7 , identity 99.7 , evalue 9.88e-259 , alnlength 359 , Bacteria , Proteobacteria , WP_141456842.1 MULTISPECIES: Fic family protein [unclassified Wolbachia]"
/cog="COG3177 COG3177 348 Fic family protein"
/pfam="Fic Pfam-B_2040 Pfam-B_15630"
/product="Fic family
protein"
/translation="MPMPNYTITSKIANCLMRIEAAKEKVLHLPLTVSMLSSLRETAR
LYTTHYSTMIEGNRLEPKQIEEVLSGKSHFPKYRRDENEVKGYYAALTQVEQWAARTI
PITEKAIQTLHALVMASGKSKVKPTSYRDGQNVIRDSRTRAIIYMPPEAKDVPKLMSS
MIDWIRDSEQVPCPIIAGIAHYQFVTIHPYYDGNGRTARLLTTLILHLGGYDLKGLYS
LEEYYAKNLGAYYEAISVGPSHNYYMGRAEADITKWVEYFVEGMANSFENVLQCINEE
EYHTDQTDLIRKLDPKQRKALELFQEFSAITASQVGELFNFKPRTSAQLCKKWVEMGF
IKIVDFSNRGRKYKLSKQYEDLISN"
/besthit="qcoverage 100 , hcoverage 100 , similarity 99.7 , identity 99.7 , evalue 9.88e-259 , alnlength 359 , Bacteria , Proteobacteria , WP_141456842.1 MULTISPECIES: Fic family protein [unclassified Wolbachia]"

gene complement(32922..33074)
/locus_tag="wEsol_00475"

CDS complement(32922..33074)
/locus_tag="wEsol_00475"
/codon_start=1
/transl_table=11
/fullproduct="qcoverage 100 , hcoverage 100 , similarity 100 , identity 100 , evalue 6.23e-26 , alnlength 50 , Bacteria , Proteobacteria , WP_007548541.1 MULTISPECIES: hypothetical protein [Wolbachia]"
/translation="MSEPIIGREKEIAILQDKLLSQSAEFIAVYGRRRVNVVLSAFYQ
HNIEVM"

/product="hypothetical protein"
/besthit="qcoverage 100 , hcoverage 100 , similarity 100 , identity 100 , evalue 6.23e-26 , alnlength 50 , Bacteria , Proteobacteria , WP_007548541.1 MULTISPECIES: hypothetical protein [Wolbachia]"

gene 33406..34164
/locus_tag="wEsol_00476"

CDS 33406..34164
/locus_tag="wEsol_00476"
/codon_start=1
/transl_table=11
/fullproduct="qcoverage 100 , hcoverage 100 , similarity 100 , identity 100 , evalue 1.56e-174 , alnlength 252 , Bacteria , Proteobacteria , WP_015589505.1 MULTISPECIES: DsbA family protein [unclassified Wolbachia]"
/cog="DsbG COG1651 244 Protein-disulfide isomerase"
/pfam="Thioredoxin_4 Thioredoxin_2 DSBA"
/product="DsbA family
protein"
/translation="MSRISFLLILILAVASLPVINNWLSHRGQTLTDDQISTRLDDYI
SRNFDKIVKTLKEESIKSSYAARDNATKGKILQYKNEIFDSAYPYSGNENSSVIAVGF
FDYSCGYCKAMKNDIKQLINDGKIKYIFRDAPILGNDSLRAAKSALAVYFIDKEKYFD
FHYSALSHKGGFSDESILDIVKSIGIDEDDFNDSIKDNADKIEQMINNSRLLVRDLGV
GGTPFLIIGDSLFVGATDLNILRKKVDELSHKQG"
/besthit="qcoverage 100 , hcoverage 100 , similarity 100 , identity 100 , evalue 1.56e-174 , alnlength 252 , Bacteria , Proteobacteria , WP_015589505.1 MULTISPECIES: DsbA family protein [unclassified Wolbachia]"

gene 34385..35752
/locus_tag="wEsol_00477"

CDS 34385..35752
/locus_tag="wEsol_00477"
/codon_start=1
/transl_table=11
/pfam="OTU Pfam-B_1737 Pfam-B_2162 IncA"
/fullproduct="qcoverage 100 , hcoverage 100 , similarity 99.8 , identity 99.6 , evalue 1.28e-245 , alnlength 455 , Bacteria , Proteobacteria , WP_015589504.1 hypothetical protein [Wolbachia endosymbiont of Drosophila simulans]"
/translation="MKEPNYTREPIALFRQIKEKKKSSNLLHNLKSAYSKKLKIGAAL
KTGDCFFDSVAQGLNELKPGHRFTAKSLRKDCADYAENKKDSRWVFDEITKNIAEGYA
VPMIGSNGNVMKDNQNNIIPDNIASFDRYLKDIRSMATEVSLPIWGRPGIEGRMICDK
YEVELDIYSIEEDSQKVTVTKITAHDDDLEVFIQGVNLDQLKIDNSKKTVGVVNYMQH
FVPLLSGISEDIKEDQQVSSEAILGTGKRVPTSDQSEKEVQQEIINISTSATVYTDKE
IPFSHQLEQKTRQEGINASASATVYTDREIPTSNQSEQKVQQEIINTNTSAPVLQIYT
DKKIPSSHQQEQKTQQKEIQKKLINSYRKEAIYVIATVITAIAFTASAIAAIITQAIP
FACLAGTALVAACVCVYQLTQSHKDINELKSELGKGQEDNAHTFLEKVGLKMHDLINK
AISKV"
/product="hypothetical protein"
/besthit="qcoverage 100 , hcoverage 100 , similarity 99.8 , identity 99.6 , evalue 1.28e-245 , alnlength 455 , Bacteria , Proteobacteria , WP_015589504.1 hypothetical protein [Wolbachia endosymbiont of Drosophila simulans]"

gene 35806..36402
/locus_tag="wEsol_00478"

CDS 35806..36402
/locus_tag="wEsol_00478"
/codon_start=1
/transl_table=11
/fullproduct="qcoverage 100 , hcoverage 100 , similarity

```
                     100 , identity 100 , evalue 1.04e-143 , alnlength 198 ,
                     Bacteria , Proteobacteria , WP_141456846.1 MULTISPECIES:
                     TIGR02217 family protein [unclassified Wolbachia]"
                     /cog="COG5448 COG5448 184 Uncharacterized conserved
                     protein"
                     /pfam="DUF2460"
                     /tigr="TIGR02217 chp_TIGR02217 210 TIGR02217 family
                     protein"
                     /product="TIGR02217 family
                     protein"
                     /translation="MSFTEIRFPENISYGSAGGPEFSTDVVTTHNGCEQRNINWSHAR
                     TRYNIAYGVRSNEQLLELITFFHARKGKAIGFRFKDWSDFIAINQEIGIGDNKKTTFQ
                     LIKTYVSGEDKHIRMIKKPVHGTVKIYLNGKEESEYSVNYSTGEITFMKPPVKDAIIT
                     ASFEFDVPVRFDTDYLNASIDDYGSNSWNNIPLIEVKF"
                     /besthit="qcoverage 100 , hcoverage 100 , similarity 100
                     , identity 100 , evalue 1.04e-143 , alnlength 198 ,
                     Bacteria , Proteobacteria , WP_141456846.1 MULTISPECIES:
                     TIGR02217 family protein [unclassified Wolbachia]"
     gene            complement(36533..37321)
                     /locus_tag="wEsol_00479"
     CDS             complement(36533..37321)
                     /locus_tag="wEsol_00479"
                     /codon_start=1
                     /transl_table=11
                     /fullproduct="qcoverage 100 , hcoverage 100 , similarity
                     100 , identity 100 , evalue 6.62e-181 , alnlength 262 ,
                     Bacteria , Proteobacteria , WP_015589502.1 MULTISPECIES:
                     hypothetical protein [unclassified Wolbachia]"
                     /translation="MNTKIFEMLIRRTGLRRDSKAEIIQLNRNEKAFVDEDKKSIELA
                     EAIFEENNMFHFRKPGPAMDYILHKLINDRKLFEEMKDRGTSIIRLFSLLSMYHGRLN
                     CCVIRKGGCITECGILDYAGTYPELVPLEDFILIEPCSYLEYTVLDNDNIRNRLARDL
                     TEDKTKLDIFLNGILATKNSDLVLSCINKIEVLNEEDRYFVNRLQKNNMLDYILNKDD
                     TTLAKSTLKELLKLSILQEGESTKLESPQASSVNVSKAQQHCSI"
                     /product="hypothetical protein"
                     /besthit="qcoverage 100 , hcoverage 100 , similarity 100
                     , identity 100 , evalue 6.62e-181 , alnlength 262 ,
                     Bacteria , Proteobacteria , WP_015589502.1 MULTISPECIES:
                     hypothetical protein [unclassified Wolbachia]"
     gene            complement(37565..>38835)
                     /locus_tag="wEsol_00480"
     CDS             complement(37565..>38835)
                     /locus_tag="wEsol_00480"
                     /codon_start=1
                     /transl_table=11
                     /product="hypothetical protein"
                     /pfam="Transket_pyr"
                     /tigr="TIGR00239 2oxo_dh_E1 931 oxoglutarate
                     dehydrogenase (succinyl-transferring), E1 component"
                     /translation="SAAYTPKKADWFGGVWSKLRRAKLNDLSEYYTDSGVPPDELKKL
                     GVHINSNIPSSFNINNKVRKILDGRIDSINSGSNIDWATAESLAFASLLTEGIGVRLS
                     GQDSGRGTFSHRHSRLVDQVTEEAFIPLNNINEKQAHFEVIDSALSEYAVMGFEYGYS
                     LDSPYSLVLWEGQFGDFANGAQIMIDQFIASAETKWLRSSGLVLLLPHGYEGQGPEHS
                     SARIERFLQLCAEDNMQVVNCSTPANYFHVLRRQMHRDFRKPLVVFTPKSLLRHKRAV
                     SNLSDFEGKFLTVIPECRKGLVSSDKVRKVVICSGKVYYDIIEMLETQKINDIAVIRL
                     EQFYPFPADKLSNELEKYKNAEIIWCQEEPKNMGGWFFVNPLIEEVLSNLDIQAKRPK
                     CIARPAAASPACGYASVHAQQQEEILRQIA"
                     /cog="SucA COG0567 906 2-oxoglutarate dehydrogenase
                     complex, dehydrogenase (E1) component, and related
                     enzymes"
                     /besthit="qcoverage 100 , hcoverage 47.737556561086 ,
```

```
                     similarity 100 , identity 100 , evalue 3.64e-304 ,
                     alnlength 422 , Bacteria , Proteobacteria ,
                     WP_187470087.1 2-oxoglutarate dehydrogenase E1 component
                     [Wolbachia endosymbiont of Drosophila sechellia]"
     gene            complement(16212..16296)
                     /locus_tag="wEsol_tRNA-04"
     tRNA            complement(16212..16296)
                     /locus_tag="wEsol_tRNA-04"
                     /gene="tRNA-Leu"
                     /intronpos="0..0"
                     /program="tRNAscan-SE"
                     /cove_score="57.8"
                     /evidence=predicted
                     /type="Leu"
                     /anticodon=GAG
                     /product="tRNA-Leu (GAG)"
     gene            complement(1705..1777)
                     /locus_tag="wEsol_tRNA-05"
     tRNA            complement(1705..1777)
                     /locus_tag="wEsol_tRNA-05"
                     /gene="tRNA-Trp"
                     /intronpos="0..0"
                     /program="tRNAscan-SE"
                     /cove_score="72.6"
                     /evidence=predicted
                     /type="Trp"
                     /anticodon=CCA
                     /product="tRNA-Trp (CCA)"
ORIGIN
        1 ctaataaatg tttatcttga gcgaaaacag ctactttaat attcttcccg ataccttag
       61 gcaaaactac tgtaccacgc acctgctctt cagatttaca tgaatctacg cctagattga
      121 ccgcaacatc aactgattca ttaaattttg ctgaagcaga ctcaacaatc ttctccaaac
      181 actgcttagg attaccatta tatgtattca tttacctaac cttccacaac ttctataccc
      241 atagattttg cagtgcctac aaccattttc actgctgaat cttcattatc cactttcata
      301 tcaaccatct tgcactttgc cactttaatt atagcagaca taggtaattt agccactaat
      361 tctttaccag gattaccaga acctttactt aatttagctt cctgcttaag taaataagcc
      421 acaggaggac cgctgacagt aaaatcatga gagcgatcat cctttatgga aattcgcact
      481 gttactaaat cacctacttt ataattagcg ttagcagcgc tagtaacttt attaaaagct
      541 tcacaaaact taggaacagg tataccacgc ggaccaagta ctgaagcaat ttttggtcct
      601 ggaactgctt ttccagcttc cataagtaag ttaatcttag caactacaac actcataatt
      661 aatcctctat tttctctact tgagccagat caagctctat tattgtcggc ttacctaaaa
      721 tcgatacttc cacattaata atttttctct catcgtttac catatttacc ttaccagtaa
      781 aattttgaaa aagaccatca ttaattttca ctttttcacc tttttcataa ccataaccta
      841 acttctttgt ctcttgagca ttataaagtg cattacacat tgagtgaatc tcatcatcca
      901 aaattacctt tggaatatta ccatttttca aaaatccata aaccttaaga gattttggta
      961 tattattgat aaaattcaat acttcatcgc ataaattcac gtataaaaag acataaccag
     1021 gaaagcattt ccttcgtgta gcaattttct tagattttaa ctctatttca cttagctctt
     1081 catatggaat aaaaacttcc ttaaagtaat cattaacacc caatctcata gaattttcca
     1141 atatatgttg gcgcactttc tcttcacaat tggaagcaac tctcaagata taccatttat
     1201 atctattaaa ttctttacac tttaaactta tctcatcatc tgaaatcatc tcaaaatcac
     1261 cagattcgcc actacagaat ccaggcgttt gattcaaaat atttttgctt tcatcacaca
     1321 gatgcatata tacatacact tcacataact cttttatatc agactttgaa ttacataccg
     1381 tttgataagg aataaacact tccttaaaat aaacagcatt acttaccaat tcgcgtatgt
     1441 tttgctcata tccataatca actttaatga tataccattt atattcacat tccataaata
     1501 attccaaata aagccttaat tacgtaaaga gacataaaat ccacaaaaca gaagaaaatc
     1561 gaaaagcata atataacaat cattacaaca aatagagatg acaacacctc ctgtttcttt
     1621 acccaagcaa ttcttcgtat ttcttgcttt atatcacaga aaaaaccaca caaacttttt
     1681 aacatttcca ccacaacatt aatgcaggag cgataggaat tgaacctaca acctctggtt
     1741 ttggagacca gcgctctacc aattgagcta cactcctata actcaaactc tcactccaaa
     1801 atttcagaaa caacaccaga accaacagtt ctaccgcctt ctcttatcgc aaaacgcaat
```

1861 cccttatcca ttgctatcgg tacttgcaat tctacttcta cactcacatt atctcctggc
1921 attaccatct ccttcccatc tagcaatttt atgctcccag ttacatccgt tgtccttaaa
1981 taaaactgtg gctggtaatt cgcaaaaaat ggtgtatgcc ttcctccttc ctctttcttt
2041 aatatataaa cctccgcctt aaactttcta tgcggtgtta tcgtccccgg tttcgctaat
2101 acttgccctc tctccacttc ttctcttttt gttcctctta acaatattcc tacattgagt
2161 cctgcacttc ccttatccag caacttctta aacatctcaa cacctgtgca tatcgtcttt
2221 tgcgtcgctt tcagacctat tatctctatc tcttctcccg tctttatctc tcccttctct
2281 attcttcctg ttaccactgt tcctcgcccc gatatcgaaa atacatcttc gattggcaat
2341 aaaaatggta aatccacagg ccttggtgga accgctacat attcatctaa cttttccatc
2401 aatttgtcta ttgatttctt tccatattcg ctgctgtcat cctccagcgc cttgagcgct
2461 gaaccaacca ccacaggcac ttcatcccct ggaaatccgt atttactcag taattccctc
2521 acttccattt ctactaaatc tatcatatca gtatcagcaa catcagcttt atttatatac
2581 accacgatat atccaacacc tacctgcttc gccagcaata tatgctctct cgtttgtggc
2641 attggcccat caaccccсga cactaccaat atcgccgcat ccatctgtgc tgcacctact
2701 atcatgttct ttacatagtc agcgtgtcca ggacaatcaa cgtgtgcata atgccttttt
2761 tccgtctgat actcaacatg cgctgttgct atcgttatcc ccctcttcct ttcttctggc
2821 gctttatcta tctgatcgta cgctacaaaa tttccataat gcttcgttat cgccgctgtt
2881 aacgtcgtct tcccatgatc cacatgtcct attgttccca catttacatg cggctttcca
2941 aatgcttcca ctattgctgt cataattatt acccttaatt atactctaac tttaactcat
3001 caaccacata ttgtggcact tgctcataac aagaaaaatg catactatac tgcgcccttc
3061 cctgagacat agaacgcaaa acgttgatat aaccaaacat atttgcaaga ggtaccgaag
3121 cagtaattat tttactatta ttacctaaat cgagcatatc agcaacattt cctcttctac
3181 tatttatatc acccataaca tcacccatat attcttcagg agtaatgatt tcaactttca
3241 ttataggctc cagcattttt ggaccagctt tatttgccat ctctttaaaa gcacctttag
3301 cagcaagttc aaaagctaaa ggactagaat caacctcatg aaaagcacca tcaagaaggg
3361 tagccttaaa atcaatcaac ggaaatcccg aaattacacc accttctttt atcaactcca
3421 agccattttc taccccagga atatactctt ttggaatagc accacctaca attttacttt
3481 caaattgaaa cccagaacca ggctcaagag gttcaaattt tattttaacc ttagcaaact
3541 gaccagctcc accagattgt tttttatgaa tgtaatcaat ttcaacagat ttagtgatag
3601 tttcacgata tgcaacctga ggagcaccga cgttagcctc aacgttaaat tcgcgcttca
3661 ttctatcaac aataatctca agatggagct cacccatgcc tttcaatata gtctggccac
3721 tttccgcatt tactgacatt ctcaaagaag gatcttctgc aaccaaccta tttagagcaa
3781 tacctaattt ttcctgatct gaagtggttt taggttccac agcaatttca ataacaggct
3841 caggaaattc catacgctcc aataatatag gaaaatcaac agaacataaa gtatctccgg
3901 tggttgtttt ctttagccca actaaagcaa ctatatctcc agccctagcc tcagttatat
3961 cttctcggtt atttgcatgc ataagtaaca ttctgccaat cccttcagtt tcgtttttta
4021 acgcatttga aacggtagat ttagacttta atttaccaga ataaatacgg ataaacgtca
4081 agctgcctac aaatttatcc gtcatcactt taaatgcaag agcaacaaat ttctcttttt
4141 ctgaaggttt gacctcaatt ttcttttctg aatctttagg atcaattcca acaattacat
4201 caacatcaat aggagaaggt aaaaagtcaa ctacaccatc taaaagtggt tgtacgcctt
4261 tatttttaaa agctgatcca cataacacag gaacaaattt tcctttaatg gtcccatttc
4321 tcacgcattt tttcagtaaa tccactggca gatcattaga ctcaaagtaa gtattcatcg
4381 cttcatcatc catctcagct gcagcatcta ataaaagatt tcgatattct tgagccttat
4441 caagcaaatc agaaggaata tcttcataag aaaatttagc gcccaacgtt tcttcttgcc
4501 atataatggc tttcatagaa ataaggtcaa ttatacсttt gaaatctttc tcactcccta
4561 ttggtaaatg aataactaaa ggtgctgcac caagcttcgt ctttatcata tcaacacatc
4621 gataaaaatt tgcccctatt ctatccatct tattaacaaa gcagatacga ggaacgctat
4681 atttatcagc ttgacgccac actgtctcag attgaggttc aactccagca actccatcaa
4741 atacagcaac tgcaccatcc aaaaccctta aagatctctc aacttcaata gtgaaatcaa
4801 cgtgaccagg agtgtctatt atattgaccc tatgatcatt ccaaaaacat gttgttgcag
4861 cagatgtgat tgtaatacca cgctcttttt cctgctccat ccaatccata gaagctgcac
4921 catcgtgcac ttcaccaatt ctattttgct taccagtata aaataaaata cgctctgttg
4981 tggtagtctt accagcatct atgtgagcca ttatccctat atttctgtat ttagatattc
5041 caatttccat atcacaatta attagctaac attaaaaacg aagatgagaa aatgccttat
5101 tagcttcagc cattttatat ttttcttcgc acatcttaaa cgcaccaccg cgtttattat
5161 aagcatccag tatttcagat tgcaaacaat caacagtggt ttttccactc ttttttcgag
5221 cagcagaagt tgctttagca atccacctta acgctaaaga aaccgctcta tcttgtcgaa
5281 cttcaacagg tacttgataa gttgcaccac caatgcgtcg agaacgcact tctatagaag
5341 gagtaacatt tcctactgct gtctcaaaaa ttgataaccc accttcgcct atttctttt
5401 cagctaaaga caaagcgtca tagataatct tttctgcagt agattttta ccacatttca

5461 tgattgtatt aatgaaacgc atcagcaaaa cactgccgta acgcgaatca ggacttattt
5521 ctcttttttt tgctttattc cgacgagcca taaactttaa ccagatttct ttacaccata
5581 ttttgaacga gctttctttc gattttgcac accacgcaga tcaagggcac ctcttattat
5641 gtgataacgc acacctggca aatctttaac ccgcccacca cgtatcaaaa caacagagtg
5701 ttcttgtaaa ttatgacctt caccaggtat ataagccgtc acttcaccat acccactaat
5761 ttttactctg gctactttac ggagtgctga gttaggcttt ctaggagttg tagtatacac
5821 cttagtgcaa acacctctcc tttgagggtt gctttctccc aaagctggta ccttttttctt
5881 atgggtcaat cctaatctac ccttacgtat taattgattt atcgtaggca tatgttataa
5941 actcaagctc attaaaataa ctaagttttt agttataaaa taataaacct caatagtcaa
6001 tataaaaata aacattacta ctctaataat taagctcaac tgccttgaaa gccatcagta
6061 gacttcttgc actacctccc aagccacaac tgtcattcag tagaaacgaa aaaacttact
6121 tgacaccctt cgccagcccc attatcatgg tactgaaggt attcatttat cttcaatctg
6181 tgcagattaa acgacaacag tattacgtaa ttggcgtctt atttttaatt ttttgcacta
6241 tgcacacttt acgtcttcac aacatttctg ggtttttacc tatataagct gaaacgcgct
6301 tataaagcgt ttaagacagt atagtacgcc aatttgcagg attagagagt gacaactagc
6361 taatacggga tatcttttgc cttttttttct gcttagtaaa tttcttaaac atttaaacta
6421 aggtgagttg catttaaaag cagctaaatt gcagtgttta agacttaaaa aacgccaata
6481 ctgaaaatag acaatgacta gggcttcttt tgcctttttt tttgcttggt aaatttctta
6541 atatttatgg caacatgaac tgagcaggaa ggtgtcattc cagtgcttga cactggaatc
6601 cagttttcca tataatctca tcgaaaatgt tgtaaccact ttctgtgcta gtttgcttgc
6661 ttacaagcaa acttttctgg atctcagtgt ctgggcactg ggatgacagg ggagggaggc
6721 tacttggatg acaccattct tttcctggga tccaagtagt cagggcactg ggatgacagg
6781 agaaggtact aggatgacac cctgacctcg tcgtcccgct acgtgttagc ggaatctaga
6841 gataccgtga cggtatgacg taaagctcgc attagctata cttttatgct caccaactta
6901 gctggatcca agtagtcaag ctacttggat ctgcaaattg caatgttcgt acagctgcgt
6961 gcttgacact gggatttagc gcagctaccc taaactggaa gctcatcttg ctatttttgg
7021 ctgaaagaaa catattttta gaataatgaa aacaatcagc actttaattc aagaaggatc
7081 ggagttatta tcgtcacata aagttgaatc accacattta gattgtgaaa tcatcatgca
7141 gcacgtactt ggagtggaaa gatcatttat aattgtgaac catgctgatc aagtgccgat
7201 ggagaaagaa cttttatttt ggaaattaac aaaaaaaaga gcggaaagat atccaatatc
7261 gcaaataata ggtaatcgtg aattctggag taaaaatttt atagttaacc agcatgtttt
7321 agatccaagg ccagatagtg aaacaataat cctagcagtg ttaaaatatt acccaaacaa
7381 aaaacagaaa ctaaaaattg cagattttgg tacaggcaca ggttgtttat tgatatccgt
7441 acttagtgag tatgaatatg ctgttggcgt cggttttgaa aaaagcttgg aagcatataa
7501 agttgcctat cagaacacaa aaaaacatga tcttctcagc agagccaaaa tatttccaag
7561 ttcgtggaca gagtgcagag gttcattcga tcttataatc agtaatcctc catatattaa
7621 aagaagcaaa ttgaaagact tgcaagctga agtgcaaaag gaaccaagaa ttgcccttga
7681 tggtggtatt gatggtttga gctgttattt gagcattttt ccaatattga gaaagtgcct
7741 taaaaaaaat gggtttgcga tattggagat aggtgaagat caaagcaata ttgacaaaat
7801 aatcccctca tacgaattag cttttcagga atatgtatat gacttagcgg gaatgaagag
7861 atgcattgtt gtgaaaagaa catagctaat gtttttttgc cttcatccca tattatccta
7921 tatcatccca gtaccctcta cctagtcatc caagtagcgt gactacttgg atctatattt
7981 aactagatcc tatggtcaag ccataggacg acaggaacgg ttttatgcat tttttaaagg
8041 gcaacatgtt taagctttgc tggcaaaatg gaaagtaaga ccagcctcta taccgtgtgt
8101 gtgaaatagt tgattaccta tatttatttc ctcagttact ttgctacctg agctaggatt
8161 aagcttcttt tttttatcat tattagggtc gtctaccact tgtcctaacc tttttgctgt
8221 aagcttaata tcgctaccaa tagcaccaaa acctctatat ccgatatgca tatttacatc
8281 ttcgtttatg cgatagtcaa agccagcttt taattgacct gcgggtctta ttgacggttt
8341 ttcaaacatc gtcattcttg ttgcaccgat cccaatacca acgtaaggag aaaaagagaa
8401 acggtcactt ttccaatgat gataaacatt tgccattaca gatatgttct caatttggtc
8461 atgactaact gcagcactat agagataagt ttctttatta ggatcctcac cagtctcctt
8521 taggtatgaa acagttattt ggctacttgt taaaccaata ttatccactt ttacagaaga
8581 atacatccct tccaattcag ccctatagct gttgttaccc aattctccct gtgtaaccaa
8641 atgccacatt tgcagcaaaa ggtggattat aatctccttt atactcactt agtgactgac
8701 cttctgtgtt ctgagcaccc ctgtcattta tagacaaatt tttgatacct gtagtgcttg
8761 tttttagtac gcttgtgtta tttaaatatt gaccgtaata ccctactacc aaagtaaaac
8821 ccttctgttt cacttgcaaa agattgttgt gacaacaata aagcaaatgc tgtaaccgct
8881 aatgtttttt tactaatcat tactactccc ctagttaaaa tagctaatct aatactaatt
8941 aattaaaaat ggcttaatat gaaaaagcta attatttgca taacttgcac ttttctgagc
9001 taaaaatagg caaagttaac cttacttctc gtcatttttt aattttctca tcttcaaatt

9061 ctcctcttct tcgtcatcaa acccatcatc gctcctatcc tcatcttcct cgtcatcatc
9121 ttcaaactta tcgtcgatat cttcatcatc aatatcttcg tctgcaaatt catctgattc
9181 atcatatact gcatcttcgg ttttagtttc agcaggataa tcaactgaat taggtccctc
9241 aagaacatta ttcccctcgt tattaacctt atctttgctc gcagcggcag aaagaacttt
9301 gtttgggtca tatggttctt gtgtaggcac acgagtttgt ttgagcagcc ttcttgtgaa
9361 ggtggtatca ctgtagtaca aaattttctt tgatttaatt gggtaagtcg actctattaa
9421 aattatctga tcgtcacgtg gcaacataat aacttcttga ggcaatagca atgctctctg
9481 tatttcagag atatgtaatg accttgaagc aggatttaaa tctaaaaatt taggcttatt
9541 caacgattct tgttgtacag ttttgtttcc tataagttgc gatattaaat tagctgtttc
9601 aatattattg gctgcaaaag ttattctgta agtcgagttt gacaaaaagg agttcatccc
9661 tgcttcttcg tatattcctt tgagctgctc agtatcttga acaattaaga ataacctaac
9721 tcgataacca cgaaaatatg caatacctgt ttgaaattgc tccatttttc caagtgtagg
9781 aaactcatcc attaaaaaca atacgccata aggctcgtca tccgatggca attttctaca
9841 taaaaactca gttgcctgtt ggtaaaaaac ctgcattaaa ggcctgagcc tagtcaagtt
9901 gtcaggagtt aaaccaacat aaactgtaat tttcttcttt ttaaaatcta gaatattaaa
9961 atcacttgac gcagttgcag tatcaatcaa tgggtttgcc cacaattcaa gtgatgagtt
10021 catggttgat acaacacctg acctttcttt atcagctttt tgtaaaaaag ctgcaatatt
10081 catatatgct acagggtgta gtattttacc cattgtatca agagcaacag caagattata
10141 aaccacgtca tcactacgca tcgtacgcac aacttcacca aaagatttaa ctttctctgg
10201 tgcagcaagt aagtataata ccactccaac aaataagctt cttgcttcat tctgccaaaa
10261 gtcttgttca ggcatgatta ggttagctat tttctgcaca tcgtcaacca tctgcccagg
10321 tttttcacta atccattgta atggattgta acagtgactt attccatctg gctgcgctgg
10381 attccatacg tatacttttt gtccttgtcg ttctcgccaa ccacttgtta tttcatagtt
10441 ttctaatttt atgtcgtgta caattaccga gtcagtccaa aataataaat taggaattac
10501 aaagccaaca cccttaccag aacctgtagg tgcaaataac aatgcatgct gaaacccatc
10561 agaaatgaag tatcctcttt tatctttacc aagcaataat cccttttttgc ttcttaatcc
10621 tgcttttcgt atgtctttct ctgatgccca ctgcgaatct ccatgaagtg attccttctt
10681 tttaaatggt cgccactcaa ttatcctttc tcttaaattc cataagataa tcattaaaac
10741 aatgataggc agtgcagaag atataattaa tgtgattttg agctcaaggc tatataactc
10801 tggatgatgc caacaatatt gtatatgatc aaaaatcgtt ggccaaagag cttgagggaa
10861 aggcgtcaaa ctaggattaa ttgccttaaa atccacagca tccggaccat cagcaaacag
10921 aacaaacaat ataccagata gatagaagca aaactcaagt acgctaaaag ctactacacc
10981 tcctatgaga atattacgta ggtgatttcc attactcata aactactctt ccttaactta
11041 tgtataaatt taagcatttt cgctcgcaga tctagtaaat aatatctcag aaatgcttct
11101 tttgccccca ccggttcttt ttagctgaat aacaatatca atcacatttc taatgtatgg
11161 tataatttgg tctggaggaa tgccaaggtt tgcttgcata accatcagtt ttatttgctc
11221 aagggccatt gttggactat ccgcatgaag ggttgatatt gatccagggt gaccagtatt
11281 tatcgcccta agaaaactaa aagcttccgc tccacggagc tcaccaacta ttattctgtc
11341 tggccttaaa cgtaagcacg cttctatcaa atcttgagtg gttacttttg ctctgccctg
11401 ccctccttta gaggcaatta gatggacttt attgggatga tcgttcaaaa cgatttccct
11461 tgcatcctca acagtaataa ttctttcctc atctggaata gcacgcaaag tagcattagt
11521 aaaagtagtc ttaccagtgg aagttccacc actaattata atatttttct tacttattac
11581 agcgtattct aaaaactctt ttattttttt ttgccttaac agtaaatcca aatgacggtc
11641 aactggatta tcagttgctt ctgtaacagt ttcagaaaaa gcccccattt tttcgtaatc
11701 atcgagtgct aattgcatgc tagaaggctt acgaatcgac atgactactt tatcaggctc
11761 acatgctggc ggaaatacta tctgtatacg ataaccattt ggtaatgttg ccgaaagtaa
11821 cggcgcttct tcgctaagct tttgttctgt agcttgagca atcagtctgc caagagattt
11881 caaatggtta agatcgaata tttctaattt ttcacacctt acttcaccac gattttcaat
11941 ccatacttcc tttggcttat ttattgatat ttcatttacg ccttcttctt gaaatatgcc
12001 ctgtaatggc tccaaatatg tatcaagtgc agcatagttc atttcattga tttaatattg
12061 cctgtggagg aaatactata tcttggttaa caaatacttt caatgcagtg ccttgatcaa
12121 cataaatagt tggtttttta tctgtatatc tgcccactat atctcgcatg tctttagaaa
12181 aatcattgat tgatttcccg attgcttcat cataaacaga tttgcttctt ttctgagttt
12241 gcctcaacag ttgacgtaca tcgtctaaag ttataccgat attgtcacta gtaattgcat
12301 caactccact agctccagaa ctttttatca cacttactat ttcttccttt attattctca
12361 gtaaatcctg atcactttta gcattccgaa ttttttcaat agccctaaga cccaatttcc
12421 atttttcgtc tcctaccttc ccaacttcag tttttatagc atcctccaat ctctcaacaa
12481 tagccccttt gagagaagaa atatctattt cagttgcagt aatagacttc actgcatcca
12541 tggcagttag cgcatcgata agattagaag ccttttgccc tataacagcc gaactaattg
12601 aaacaccagc aagtgccact gaagagaata acgcacttac tattttatta tcaactattc

12661 ccgctatccc tgctctacca agctcgtcag tactaagtga tgagatggca acatctattc
12721 catgtggcag aatcactcta ttccagttta tatttacacg agatttagca acattcgagt
12781 caaatgaata actacctatc aatcttgagc ctttaggtat taaaactgta tcaccagttt
12841 ctgcataaac atctctactc accatagcac gcagcattcc ttgcagatca gaatttattg
12901 cagtttcaag aacagcatca ataattttac cttgagtaat cattaaacca agctttccta
12961 ctctggtagc tttgctcgac tgtgctgaag tgtcggataa aatagcatca gcagccttgt
13021 tctctccact actacctgaa attgttaaca tttgtgcact acgtctgtct ctaggataac
13081 ctccgccccc tattgtagga aacgaggtag gtaaattgct aataacatta ctagaaggaa
13141 aatttggctt tggcaaaata ggtatatttg acacaggggt ttcttttggt ttctcttctt
13201 ctttttaat ttgttttatt tctggtacga tttgtggtgt aggaagagga ggtaagggtg
13261 gtaaggaatc ggttattatt ctttctggaa ctattacatt atctggaact tgttccaact
13321 ttcctttaaa ttcttgaacg ttttgctttg tttcctcttt tttaataact tctgaatcct
13381 ctttatgaga aggactaaaa tagaaataat acactacacc aaccagaagc actaaaatca
13441 taaccatcaa tgccctatga ccttgattag agccaactgt tactaccttg ttctctattt
13501 ctgactcatc ttctgaattg ttacgccttt ctttattcat attgcatgta ccacagctaa
13561 agtgacctat ttataatttc aacttcatca tcttcataac gcataaatag ctttttatgt
13621 accccttta ttataacata atcatcaaat aacagccttt tacaaggttt atcctcccct
13681 tctacaaaaa tctgaggtat tttattatta tttctaaatt ttaaataggt taaataacca
13741 tcatcaaata actcaatcgg aactatatcc gcattaccac cttcatcaat atatgtataa
13801 ttatacttcg tatcattttc ttgtattatt ttttctagct tgtctgtagt atactgcatt
13861 tgagtaggta aagaaacctc atctaaatca acatcaaatt caccttcttc ttgaggataa
13921 taaaaacgta ctacgtaaga tatatccttt tcagcagaat aatcgtgatc cactttctta
13981 gcattagtgt ccaggtattt atcgtagttt ggccttgaga ttaaatcaaa aatgtaattt
14041 cttttttag ttgtcgtaat aatcatgtta gtgcggctac taacttcaaa tggcatgata
14101 agcaatttat tgtcataagg attaatttc caacttgatg catcaccaac agcgatattt
14161 ttaactttt ctccttccgc aaattcaata taagaatagt aaccctgact aaaaaccacc
14221 gtaaatactt cattagggct atacacaaaa gtctttattc tactatctac agaaataggt
14281 tcattataat taatggatgc gtttaaattg ctacttacaa gtaaaactaa aactaataat
14341 atcctataca tattcatcat ctactctata tgaaataact tgaaacccta atgggttaat
14401 atatctttgc tgatcattca tttcaagcga tgcatatcta tatgacataa taactatctt
14461 atttttctt gtggaacttc catctttccg tataaattcc acaaaaaacc tcacctgaag
14521 agcatcgtta cccaattttt gaatcgagcg aattttaaat tcatttttgg tatctgaata
14581 taaattaaaa agatcatcca tattctgcga tcctatataa tttctaaatt cattatacac
14641 attagatgag gaaaacaacc tcacctttgt ataatagtta taattataat gatatggatc
14701 aaaaacctcc cttgctttta tatactctga aataaaataa tcgttcagcg cttcatctgc
14761 agaatactgc tttactgtga caggatcaac caattgcact attccagatt tcttgtcaat
14821 ttctataaca aatggctcaa tagtgctact tgtgctaatt ttaaatatga ctaatatgct
14881 gatagagatc gttgctaata atattaacgc aaataaaagt agaatattcc tttgagcaat
14941 aactgtgctg tagcggtttg aattccaatt tatatcctta tctaacgatt cagtactttt
15001 ctctcgctta aaaaatctta gcattttaat attcttaaaa atcttaactt gtgttaataa
15061 acatagcaaa ttaacaaagt agttacaaca aaaaagctaa aaaactctta acttatggcc
15121 tagcttacca aactttgtct ccatatatga atcgttatat ttattacgtt ccacaataag
15181 tggtagacac tttgtaacct ctataccact actttccaat tctgacaatt tcctatcatt
15241 gtttgtaagt aattggattt tgttaatgtt caatttctta agcattttag ctgcaacaac
15301 aaaactcctt tcatcatctt caaaacccaa tattctattt gcatcaacag tatcaaggtt
15361 atgttctctt tgcatactgt atgctcttaa cttattagtt aaaccaatac ctcttccatc
15421 ctgcatcaaa tacaatataa taccacttcc aaagtcagct atcatttgaa ttgcttgatg
15481 taactgactt ctgcaatcgc atgataagct atctaacaag tcacccgtat agcacgaaga
15541 atgaattctc actaatggtt cgtcatcttt atctggattg ccaatgataa ttgcatgatg
15601 ttctcttcca ccactttcgg ttctataaga tatgatattt acttcttgag tctgttttaa
15661 aaataatgat gttttgcaca cttcatatac atcctgattt tcttgaaaat tatttatgaa
15721 tgacgtgtcc agtgcaataa cgtcattttt ctcgcaccaa tttcgcattt catggttatt
15781 ctcaaaagtc atatcagcca ctaacgcgta tggcaataat tctgagaact taagcaaggc
15841 aatagcacat tcatctattg tctttgagca ttgcaactct tttatgcaat cttcctttga
15901 acagtttatt aaatagagca gttcatcaaa attattcacc aacagacgtt tgctgttatg
15961 ttctttattc tgagatatgt attttacctt acttgaagtt aaagtaacat atacattact
16021 tgatgtaagc ttgtattgat taaataaatc tctttctaaa gcctcagcag cagcaaacaa
16081 taggtaatta ctttcatcat atattacaat tggccgaccg cgcctgattt cgctgatagc
16141 tctttctacc ttattgctac tctgatttcc tataaacatt tttacccata ttaaacagcg
16201 ataaaaactg gtgcggccga gaagacttga acttccaagg ccttatgagc cacagcgacc

16261 tcaacgctgc gtgtctacca attccaccac ggccgcacta tattctagtt tttatataat
16321 tattttaaat tacttaatca ttttagaatt tttcatctag agtgtcaatc tgatactaga
16381 ataatttaga aattgtttgt ataatttgat tatgaatttt gtgattataa attttaatca
16441 tttaagataa ccttactact aatttttaac ttaggttttt atacaaatga atatagctaa
16501 tatctttatt tttttagctc tctctcttat aactagcttt catttcgtcc atgcacaaat
16561 attatacaca tggtctcaag ttatcccaga aaataaacta agcatacgcg caattacgga
16621 caatgacatg tgtcccattg tttatgttga tggcgaggaa acagaaatgc tgaatcgcag
16681 ctcaattaac aatggtgatc ataacgaaac agtttgtgaa ctgacagtac aaacaagtgc
16741 caaaaacatt agtattgaga atctacgggt tcctatatta ccaaaaaagg ttaataaaat
16801 tgctttcatt ggtgacacgg gctgtagaat aaatatgtta tttcagcagg aatgtaattc
16861 ggtagatagc tggcctttaa aaaaaaattt agattcaatc gcccttcata aaccagattt
16921 aattatccat gttggtgatt atcattatag acaaacaaaa tgtagaaata caaaaaagtg
16981 tggcgatatt tatggataca ataaagaagt ttggtacgct gattggtttg agcctgcaaa
17041 ggatatttta tcacaatccc ctttcctttt tgttcgtgga aaccatgaga gttgtgatag
17101 agcttatgaa ggatggttca ggtatctaga ttcatacccc ttttcgtctg aaaaatgtgg
17161 aaacttcgtt tctagttggt ccttagatgc tggaccgatg aaatttttta tcttcgactc
17221 ttcatccggt gaggatattt ttacaaccca aagcacaatt gatgcttttg agaggcaatt
17281 tgataaattg atacaaaata gttctgacaa gcccatgtgg tttttaactc ataaaccgct
17341 ttggagatct ccaaaaaaag aatttttgac attaaaaagc catggtaatc ttacacaaat
17401 cgaagctttt ggagataaat ttccaagcaa tgttactacc atagtttctg gccacattca
17461 tatagcccag attttattaa tggataatgt tccagaccaa attatagttg gaaacggtgg
17521 tgcatcatta cacgctcaag atcaagaatc tgtttatcaa aatgtagaat ttgactattt
17581 aaatggtaaa aactacttag cacacgaggt tagaaacttt tttggctttg gctttgcaat
17641 actaaattta gatgatcacg aatttacctt ctataatcaa cacaataagg aaatgtattc
17701 tgcgaagcta acagaagatt ttaaacttaa acggattaaa actatagtcg attcatttta
17761 ggatttgtta ttagctattt taaaaacgct acttaaacat ttttttaat cgattgattt
17821 catcagcaaa ttttgagtcg gtctttataa aattttctac ttgttttact gcatgtataa
17881 cagtagcatg gtctctgcca ccaaagtttc tcccaatatc tggtaggctt ttttgcgtaa
17941 attttttggc aaaatacata gctatttgtc ttggccttgc aagactgcga agccttctat
18001 tggattgcat atctgcaacc tttatattga aaaattcagc tactttcttt tgtatttctt
18061 ctattgtgac tgacctatga tttgacctaa gaagatcgat tagggtttca ctagctgatt
18121 ctaccgtcat acttcttcca attaatgagg tatgggtaac cttatttaat gctccttcta
18181 attctcttat attagatttt atgttccttg ctaaaaactc taggacatct ttcggaacat
18241 acatattcat ctgctccact tttgcctgca atataccaag ccttaattca aaagttgttt
18301 cattaatatc tgccaccaac ccccaaccga gtcgtgattt tattctttct tccactccat
18361 caagatcact aggagaccta tcagctgata taaccaattg tttattttgg tctatcaatg
18421 cattgaaagt gtgaaaaaat tcttcttgtg tactatcttt accactgata aattgcacat
18481 catctaccat caatacatct actgatctaa attgctcttt aaataacata atatctttgc
18541 ttcgcagcgc tgtaatatat tggtacatga atttctctgc tgataaatac accacttttc
18601 tttttgctga tggggaattg actatgtgcc aagctatagc atgcattaga tgtgttttac
18661 caagtcccac tccaccatat agaaacaaag gattgctgcc taatattgga tctatagatt
18721 ctgccacacg ctttgccgcc gtaaatgcta attcatttgg ctttcccact acaaaattat
18781 caaaggtgaa ccttggatcc agcggcgaac caagattatg attactttcc tctctgtttt
18841 ttagtataac attaaaattt gaattcctct cctcaattac ttgaatatca atagaacgta
18901 tacttttatc ttcgctttgc cataatgata atattttttt catgtaatga actgtaatcc
18961 actcttttat aaaccttgtt gacacggata aaagaacttc cccatttctg ctactgacaa
19021 atttagtga actcagccag ctattatatg ttgcttctcc ataaagatta taaagacaat
19081 tttggatttt ttcccaagta acattgtgat ccgttactgt aataatctga tcaaaaaaca
19141 tagtagaaac cttagggcta gttaagttca tataccacac caagaaaaaa gcaataatta
19201 gaaaaatata agataatcaa tagcctactg caaaatgaaa agctaatact acttaataca
19261 tactaaaaat tatggtgatt ttttgatggg ttgtaaaggg tataaaaaca ttaaaattga
19321 tcatttttat ttcaaaaaaa tgaaaaattt gtaaatgtcg cttgtatcga ggattagaag
19381 catgcaaaaa cagtaataca ttatgtataa ataagatgtt aaatttactg ggaaataagt
19441 aaagtattca tataaatata gctaacctaa tatagattta ctgaagcaaa tttatgatta
19501 tattaagtaa tttgagttgt tatgctgtat taggtattga actagagttt tacattgaag
19561 gaatagaaaa agaatactta tttctgaata gcatcaaaaa taaaatagca tctcttgaat
19621 tttcttgcga gaaggagaat tctacacatc agtatgagat aaaaagtggt tgttatacga
19681 actcctacaa tttaattaag cattttgagt tagcaaaaca atcacttact gaaacagcac
19741 aaaaactagg tgggaatgtt tcttttaaag caaagcctta tttggataga gcaggcagtg
19801 cattaaatgt gcatgttaat ctcgtgaatt caaataacgg taacttattt tatatcaatg

19861 agcaaaagta cagtgactat ctgattcata gtattggtgg attatgtgca atgatgaaaa
19921 aacatatgtt gtttttgct ccaaatgatg attcatattt aagatttcag tatccggata
19981 ttcacactcc aactacgatt agttggggag taaataacag aactgctgcg ataagaattc
20041 cctattttgg caacgatttc aaaagatgcc gcctagagca ccgcgttccc ggagcagatt
20101 gtgaccttga aaaagtactc acagcaataa ttgaaggtat ggtttttggt atagagaaca
20161 aaattgctcc gccaaatagg gtctacggca ttgcatctga tcctcaatat gagatggaga
20221 ggttaatata acaactatct atcaaatcct aatccgttaa accccttttg ggtaattggc
20281 tgttttagca tttttttac atcattatct acatttttgc actgtaatag caagtctaaa
20341 gcagatagtt tctgcgtgct tttagattca agctctagat tgcttaacac ttgcccagga
20401 agtggattac caggcacatc catgtgctca ggaagaggtt tcatctgatt ctttatagag
20461 ctagagttat tttgtgtaag agttttatca tttagcatgc taagaataat ttcctcaggc
20521 actaccatag tttttgtata atcaacagga ttactatttt cagcatctat agtcacatga
20581 attggttcgc caaacgcatt tataggacca ccaagtgatc ctttctccat gcgttcttgt
20641 gctcttttcc ttattatttc ttcttctaac tcagtttcag ttttttctgg ttcttttgcg
20701 gtatcttttt cttcaccttc atcaacactg ctataccctt catcttctga aactttctct
20761 cctgaaacac cagtaatttc aggctgttct tttttagttc ttattttatg aatcagcgaa
20821 agtaataagt aagtaactgc agcagcagaa acgataatgg ctatctttgc ggcaatgtgg
20881 attgctgtta aaaatattaa agctgataaa gctccagcta atccagacac aataacttta
20941 tttttgcctt ttgttatatt tagaattgcc accataataa cctctcaaat actgttctag
21001 aattattata caggaatttt cttatacttg caattttgcg aaaattttaa ttatttgcat
21061 aaagttttaa agctacacaa ggattaatta atatgcaatg tatctcgact ataatattgt
21121 acaaacacac atattcagct aacattcaac atgcagagct tactcataga gcgaaacagg
21181 gcggtggtat tattacttat agtaattttt atcttcggtt catacgttta tgtaaaaatg
21241 ccaagggaaa gcaatcctga tatacagatt cctataatca gcgtgtttgt tggacttcct
21301 gggatttctg ctcaagatag tgaaaagcta ttagtgcttc ctatagaaaa tgaactaaga
21361 tccatcgaag gtgtaagaga attgagagct tttgcaacta atgatggtgc tcacatgata
21421 ctcgaatttg gaacagagta tgataataaa gaagtgctcg ataatgttcg ttcaaagctt
21481 tcaaacataa aatcgaagtt acctgttgaa gcggaatctc cgataatcaa tgaaataaac
21541 ttgagcttat ttccgatact caatgtcggc ttaattggta atttgccaga aagatcctta
21601 actgaaatag cacgcaagct aaagaaagaa atagaatctc tgccaaatgt tctcaaagtt
21661 gaagtggcgg gtatgcgtaa agaaacagtg gaagttataa ttgagcccac agttctaaca
21721 aaatataaca ttcaatcaaa cgaaatattc caagctatat caagcaataa tagactagtg
21781 ggagctggat cactggaaaa tgacaccggt aaatattcga ttaaaatatc aggattacta
21841 aaagatatag aagatattat gaatattcct attagatctc aaggcgatgc agttttgaga
21901 ataaaagata tagcaaaagt ataccctagg tttgaagatc accaaggggtt tgctcgcatt
21961 aatggattac ctagtgtcgt gctagaaatt tcaaaacgta acggaaaaaa acataataga
22021 cacagtaaat caagtaaaat acttaatgga taaggcaaaa gatcaattac ctgaaaattt
22081 aaaagtggtg tacttaaacg accagtcaaa aaacgtccgc gatgtgcttg atgacttgga
22141 aaacggcata atatttgctg tgttgttaat attgatcata atgatgcttt ctatgggaac
22201 aaggattgct attcttgtgg cactctcgat acccggttcc tttctcattg ggataatagc
22261 tctttacttt atgggtataa ccttaaatat cgttgtgctc ttcagtttaa ttatggctgt
22321 tggcatgctg gttgatgatg caattgtgat cagcgaatat gccgatagaa agatgatctg
22381 cggaatgggc aaagtagaag cctttcacac ttcagttcgt gatatgtttt atccggtctt
22441 atcatcaacg ttaactaaat tggcagtgtt ttttccttta ctactctggc ctgatactgt
22501 gggaaaattc atgcagtata taccaattac aataatttta actttaaccg gatcgctcat
22561 tatggctctg gtttttatac caacacttgg tgcaatcttt ggaaagccat cagtaacttc
22621 aaaggaagaa attgaaaaaa tgaatgctat cgaaagtggt gaaataaaaa atgctgggcc
22681 tataataaga gcttacgtac gaatgctaga gaaggttttg gatcatccta aaaaattcgt
22741 atgtgctact gtattcgttt tgttttcatt tagtgtgctg tactttactt ttggtcctgg
22801 agtaaagttt tttccgaaag tggattcaga taatatttta atcagtgtta aagcaaaaga
22861 aagcttatca gccaaagcac gagatctgat actcaaagaa gtggaagaac gcattttgag
22921 cattgaaaaa gaaattcatg tttcttatgc tagatcagga gcattttcag acaacgttat
22981 cgccagaata caactagaac ttatggattg gcgcttcagg cgcaaagcta agcacatact
23041 aaatgatata aggagcagtg tgcaagacat gaaaggaata ataattaatg ttcaagagga
23101 aaagccagga ccgtcggctg acaagccaat acaaatcaac ctcagcggga gtgcatccag
23161 tttgaattta gcagcagaga aaattctaga aattatgaac caaccttcat ccgggtttat
23221 aaatattcaa gatagcagat cagcacctga aatagagtgg aatatgagtg ttgataagaa
23281 taaggctaca agctctgaag taagtgttgc aaccattggc gattttataa agatggttac
23341 aaatggagtg ttgattggaa aatatagacc aaataacacg gatgaagaaa tcgatattgt
23401 actccgtttc cctggaaagg atcgcaacat gaagactata gacaatcttt ttatcaatac

23461 agttaatggc ccgtatccta tgagcaatat agtaaaatat gctcccgaaa aaaaggtaaa
23521 caaactaaac aggattgatg gattacgcac agtgacaatc tctgctgacg ttgatactgg
23581 ctatcttgtt gatgaaagag taaagttcat tcagaattct attgcccagg attgggacaa
23641 gggagtaaaa attgatttta aaggcgacaa agaagatcaa caaaaatcag gagcattttt
23701 gttaaaggct tttatattag cgattacact aatgatattg gtattagtga cacagtttaa
23761 taatgtatac catacattta tcgtaatgac agcagtgttt ttatccacaa catgtgtgtt
23821 ttttgttttc tttcttatcc aacaagtttt cgtagttgtt atgtgtggag tggggataat
23881 tgctttggca ggaattatag taaataataa catactattg cttgatgcct ttcatcacca
23941 aattaaggta cacaaaaatg acatcaagcg atgtgtaata aacgctgcaa tttctcgcgt
24001 taggccaata ttgcttaccg ttgcaaccac agttcttggt ttaataccga tgattaccag
24061 gttaaatatc aatttttta cgctacaaat cacatatgat gctccatcaa gccagtggtg
24121 ggttgacatt tcaacaacca tagcaagtgg aatattggct gcaacggttt taactttatt
24181 ctttactcct gcgttgcttg ttatacaaaa acatgaaaaa acttgactta aattagcaat
24241 catttaacat ttaagatagt gttctttgcc cgttggagct ataattataa ctaattacat
24301 tggatttatt tattatgatt gctaatcgta aaagcagtgt taagcagaaa agtaacaaga
24361 ataacatagt tggaagcctt gatgtaataa aaacagcagg agagctttca cttcaaaaag
24421 gtttagattt tgatgttgta ataaaggcac tagaaagtgc tatagaagca gtggctcatc
24481 aaaaatatgg tagcaaaagt aaaattgtag ttgatataga tagaaacaca ggcaaagtta
24541 cttcatatag aaaactaaaa gttattgatg atgaatcaaa tgaaaaagag aataccgagt
24601 gtgaatcaat tacgcttaca caagctaaat taataaaagg agatgcaaaa gttggagata
24661 ctgttagtga attactctct cttaacactg atcttgcttc agcaagaatt gcccagcaaa
24721 agattgctca aataattaaa gatgaagagt caaaaaagca atatgaggaa tttaaagata
24781 aggtgggaga aataaggtat ggtattgtta aacaagtaga atattcagat cttattatag
24841 acataaatgg cactagggca tatcttccat tgcgaaattt aactggtggt gaatcatttc
24901 gtgaaggtga taaggttaaa gcttacatac agactgttaa gcgctctgat gatggacgtc
24961 aaattattct ttctaggact catgaaggct ttttggaggc attattaaat caagaaatac
25021 cagaaattgc tgacggatta gtgacaatta aggtatagc tagagatgct ggttcaaggt
25081 ctaaggttgc cgttttctct cccgacaaga acattgatcc agtaggtgct tgtgttggag
25141 ttaagggaga tagaataaaa actattatac acgaattaaa tggtgaaaaa attgacgtta
25201 taaattactc ctcagatttg ggtcaatttg ttattaaagc cattactcct gcagaagtat
25261 cgaaggttat tattgatgaa aatgaaaatt gcatagagtt aatagttgct gaagatcaat
25321 tgagtttagc gataggaaaa aagggccaga atgtaagatt agcttcagag cttgttggat
25381 ggaaaattga gatattaagc actcagcaag aatcagaaag gcgaagtaaa gaacttagtc
25441 aatgttcagc tttatttgct gaagctttaa acttagaaga gattatgggt cagctattgg
25501 taacagaagg tttttcaagt gtagaagaca tatctagcgc ttcaattaaa gagcttgctt
25561 caatagaggg ctttaatgaa gatattgcaa acgaacttca caatagagca aataagtacc
25621 tgaaagcaga aaatgataga aaaatagaag agctaaaaag tttaggtatg gaggatgatg
25681 taatcaattt aactttatca atagacaata aaattgctct tagtaagcat gatattaaga
25741 ctttagagga catagcagat ttatcaagtt atgagttttg cagcatactc tcttcctcat
25801 ccgatagcaa agaagatcta aaagatacag tagattcaat aatcatagaa gcacgtaaaa
25861 agcttggatt aatataataa aataatatga ataatgaaga tatcagtgat aaaaaattaa
25921 cgctccaagg tttaagcaag cttaaattag acctcaattt aaactcttca gctagcccaa
25981 gtactggagc tacagtagta aaaaaaagaa gaaaaaaatc tcatgatgca gaagaacata
26041 atttatttga cgagagtaaa ttaggttctt taacagagaa agaacaaatt ttccgcatta
26101 atgctgtgca gaatgctgct ttgctgaaag aaagaaatca gagagaagaa aaagagaaaa
26161 cagcaaaaga agacagcaat aaagagattg acgagaaaaa tgaagcggac gctttatcta
26221 aggaaataaa taagcaagtt ttaagtaata cccacttagt tgaaccaaaa gaagatagta
26281 ttgaccatga ggacaacgat aaaaagtctt ttaaagtcaa caaagatata tattctaagc
26341 attctaaact ggtaattgca caggcaatag atgaaaaaac tgaacaaccc cctgtattta
26401 agcaaaggtt tggtataaga ggtagaaagt caaagtttac taagggtaaa aatatatcaa
26461 gagaagttat tataccagac aaaataacta tcagggagtt atctattcgc atggcagaag
26521 atagcaagag tgtgctaaaa atgttcaaag aagaagtcgg tgagagttat agagtagatg
26581 atctggtgga tccagatata gcatgtgaaa tagtggaaaa atttaatcat acggctaaac
26641 gagtgagcga taccaacaga gaaaaagatt tattttttat agaaggcaga gaaagcctgc
26701 ctaaaaaacc taaaccacca atcgtcactt ttatgggaca cgtcgatcat ggtaaaacct
26761 cattgctcga tgcatttcgt gaatctaatg ttgcagaaag agagtcaggt ggcataactc
26821 aacacatagg tgcttatcaa ataataacaa aagataagca aaaaattact ttcattgata
26881 caccaggaca tgaagcgttc actgcgatgc gtgcatgtgg tgccaatatt actaatatag
26941 ttgtaatagt agttgcagct gacgatggaa tcatgaaaca aacagttgaa gcaataaatc
27001 atgcaaaagc agcaaatgtt tctattatag ttgctattaa taaaattgat aaatctgagc

27061 ctggtgatgt ggagaaagta atgagtagct tacctcaata tgatctcatt cctgaagagc
27121 ttggtggtga tgttatgatt gtgccagtat cagcaaaaaa gaaaactaat ttagataaac
27181 tagaagagac gattttgtta atcgctgaat taatgaagct agaagcaata gaagattgtc
27241 gagcacttgg atgggtaata gaatccaaaa tagataaagc gaaaggaata tcagctacat
27301 tgatagttga ggaaggaacg ttaaaggttg gtgatatatt ggtagttggt acaacataca
27361 gtaaagtacg tagtatggtt aatcaccttg gtcaaagaga aaaagcagca ctaccctccg
27421 ctccaattga aattactggt ctaaatggtg taccaaatgc tggagataaa tttgttgttg
27481 taaattctga aaagcaggcg cgtgaaattg ttgaatacag gctagagtta atcaagaaaa
27541 agaaagaaga tttgggcgac aataatttag atatattcag tcgtaataac agcgaaacag
27601 aagaactatc tgtagtttta aagtgcgatg taactggttc tattgaagca atatcaagtt
27661 caattgataa actcggtaaa gaccaagtga aattaaatat cctacacaaa gcagtaggag
27721 ggataacaga ttcagatgtg ctgcttgcag aagcatcaag tgcagtaatt ttagctttta
27781 atgtaaaagt ggactcaaaa ataagggact tggcaaaaca aaaaggtata gaaatacata
27841 cttataacat aatatacgag ctcattgacg acatgagaat gtatttaact aaaatgttaa
27901 agcctgttac acgagaggtt cgtgttggtt ctgcatctgt aaggcaaata tttaatgtat
27961 ctaaagctgg taatatcatt ggatgttatg taagtgatgg agtcataaaa aaagattctt
28021 taattaaggt agtgcgtggt ggcaaattgg tgcatgaagg aaagttaaaa gcaatacgta
28081 gatttaaaga tgatgttaaa gaagtaggtg taaactttga atgcggagta tcactagagg
28141 gtaatatcga tattaaagtt ggagatattt tggaagctta tcaattagtg caagaagaaa
28201 gggtgttata gtagctggat atgaaaaagg aaattagaaa cctaaagata gcatcggtat
28261 tgcatagagc aatatcaaga attctaatgg aaggtaaggt gttcaataaa aatgtagcgg
28321 tatctgacgt aaaattaagt aaagatttga aaaaagcaga tgtatatatt gtgttatctt
28381 cattgaatat tcaagtagct gactacttag atccagaaaa aagacgcact ggagatcctt
28441 ctgagaataa ttgtgatctt aacaccgttg ttaatgaaat gaaccaatct gcatggtcaa
28501 tacgtaaatc tatactgtgt tatgttgatt tgcgatttat acctgaacta gttttcaagc
28561 ctgacttagc gttcgataat tttgttaatg taagcaaaat attacataat cacacttaat
28621 taatgcattt caatcgttac ttttcattaa tgaggttgca cagtttaacg ggtttgtggc
28681 ttgtgctatt tcccagtttg agcggtattc ttttagcctc aacttctcta tcgtatcagg
28741 ttcttttttct ccttgttttg tttactatag gtgcattttt aatgagaccc gcagggtgca
28801 taatcaatga tatcttcgat agaaaaatag atgcgcacgt tgaacgaaca aaatataggc
28861 cacttgcaag tggtgcatta aacataaagc aggctttagc tttactttca ctgctactgt
28921 ctattgacct ggtaatctta ttattaacta ataaaactac acttatactt ggaataatct
28981 caatgtgtat gatagctact taccctttat taaagcggta tgtttggtgg ccacaattgt
29041 tcttagggtt taccttcaat atgggatcgc tcatgggctc agcagcaata acaaaccaga
29101 tcagtataga atccttgctg ttttatatag gatgcgtctt ttggacactt agctatgaca
29161 ccatatacgc ccatcaagat aaaaaagatg atgaaaaact cgggatgaaa tctaccgcat
29221 tatattttgg tgatacaaca aaatcttggt tgaaaagatt ttatttaata tctcttatga
29281 cgtggctata tgctggtatt ttatcatcat taaataacat tttttatatt gctctacttg
29341 ccgtagggtt catatttcac cgccaataca aaaatttcaa tcctgacgat cccagtcaat
29401 gcatgtccat atttaaaaat aattcctacg tagggttgct tctctttttt ggcatccttt
29461 tagatagaat tatcacctag gtctcaagag ttttggctct aatgaagtca ataaactcag
29521 gtactcgcag aaacgaaaat tgcttgacac ccttcgccag ccctcttatc atgaaactga
29581 agctattgta tttgctttcg ccaatctgca gattaaaagg taaggattac ttaatgtatc
29641 ggcgtcttat gttcaatttt ttgcagtata tagatactgt atgtctttac aaaacttcat
29701 ctacatctag atttttatct aaataagctg aacgcgctta taaagcgtta caagacatca
29761 aaaaatgcca atactcgaca gagatagtaa aagactagct aactcgggga ttctttgtct
29821 tttttctgc ttagtaaatt tcttaaacat ttgcagcgta ggttagttgc aattaaaagc
29881 ggctgaatct cgcttgtcaa gcgtttaaaa cataaaaaac gccaatattt taaagcggat
29941 aataacccca gggcttcttt tgcctttttt ttcatttggt aaatttctta atatttacag
30001 ctaaacaaca accgtcatcc ctaagtatcc cgctgcttgt tagcggctga gataccgcga
30061 atgaatcgcg gtatgacggt tcgcggcagg atgacgataa gctcgtcatc ccactacttg
30121 ttagcggcta agagataccg cggcggtata gctataggat gacaaaaaaa gggcactgtg
30181 acgaacttaa atcacaatta tcagttaatg ctaattacgg cattataaaa aacgccaaac
30241 tttttctgc ttttatgcaa gaagtctagt ccatatattc ctcccacttt tccgtgactt
30301 ttcttactat ttcctcactc atctcaattt tctttcccca ctctcgtttg gtttcaggcg
30361 ggattttatt tgtcgcatca aatcccattt tacctccaag tccactttca ggggaagcaa
30421 agtctaaata atcaataggt gcattttcta tcatgattgt atcacgcaca gggtccatcc
30481 tcgttgacat tgcccacatc acttccttcc aatcacgtat atttatatca tcatcaacaa
30541 cgataataaa cttcgtgtat aaaaattgct ttagaaagga aagtatgccc ataacaattc
30601 tttttgcctg ccctggatag gatttttta tcgacactac cgctattcta tatgagcaac

30661 cttccggtgg cagataaaag tccactatct ctggaaattg attgataaga atgggcacaa
30721 aaatttcatt gagtgcttca ccaagaatcg atggttcatc aggtggcttg ccagtgaaag
30781 tgctaagata tatcgggttt ttacgcatag taattgcagt aatattaaat tcaggaaatt
30841 gctcaacaga attatagtag ccggtgtgat ctccgtatgg cccttcgtct tgatatctat
30901 ccaaactcac atagccttcc agaacgattt ccgcgtgagc tggtacttga agtggaatag
30961 tcctacaatt taccagctca agcggcttct ttcgcagcag tccagcaaat tggtattctg
31021 acaatgtttc tggcactgga gttactgcag caataatcgt tgcaggatca ctaccgatca
31081 cagcagcagc aggaaatttc atattttgtt ccttctcttt ccatcgtttg tggtgagttg
31141 cgccacctct ttgtgcaagc caacgcatta gtgttgtttt tttatctata acctgcatac
31201 gatatattcc aagattaaaa ttatcttgct tttctttcgt gggtcctttt gttaccacaa
31261 tcggccaggt gataagtggt gcaggttcat ttggccagca tgtttgaatg ggcagtagac
31321 ttaggtccac ttcatctccg gttagcacta cctcttgaca tggagccttg ctcacgactt
31381 tacttcgcat cgataacacg actttcaaca aaggaaacat ttttatagca tccttaaagt
31441 tttttggcgg ctcaggtgat tgcaaaaatg ctaaaagttt accaagatct cttagttcat
31501 caggatttat gttcaacccc aatgcaatcc tcttaatagt accaaataaa ttcaccaaaa
31561 ctggaatcga atttttgcca ttttctgtga taacattttc aaaaatgatc gctggccctt
31621 tgcttgacaa aactctgcga tgaatttctg tcatttcaag aactgttgaa acttcctcct
31681 taatcctaat cagatctttt ttttcttcta aggctttgat gaagtcgcgt aagttgttgt
31741 acatattcca gtttttaaaa ttttaagttt atatctagtt ttcaagcact ttattgaaat
31801 ttgacctatg cactgtctag cgagctttca gttaatttga gatcaaatct tcatattgct
31861 tactaagttt gtatttcctt cctctattgg aaaaatctac aatcttgata aaccccattt
31921 ccacccactt tttacaaagt tgcgcactag tacgaggctt aaaattaaat agttcgccaa
31981 cctgactagc tgtaatagcg gaaaactcct ggaagagctc aagagctttg cgttgtttag
32041 gatcaagttt acgtataaga tcagtctgat cagtgtggta ttcttcttca tttatacatt
32101 gtaaaacgtt ctcaaatgaa tttgccatac cttctacgaa atactccacc cacttagtga
32161 tatctgcttc agctcgtccc atataataat tgtgcgatgg acctacactg attgcctcat
32221 aataagctcc tagattctta gcataatatt cttccaatga ataaagccct tttaggtcat
32281 atccaccaag gtgtaaaatt aatgtagtca acaatcttgc agtacgacca ttaccgtcat
32341 agtaagggtg aattgttaca aattgataat gagcaatacc tgcaataatt gggcaaggca
32401 cttgctcact gtcacgaatc caatcaatca tactgctcat gagctttggt acatcctttg
32461 cttcaggggg catataaatt attgcacgtg tacggctatc acgaataacg ttttgaccat
32521 cacgataaga ggttggcttc actttagatt ttccactcgc cattactagg gcatgaagcg
32581 tttgaatggc tttctcagta atagggatcg ttctcgctgc ccattgttca acttgggtta
32641 aagctgcata atatccttta acctcatttt catctctcct atatttttgga aagtggcttt
32701 taccacttaa aacctcttca atctgtttag gttcaagtcg atttccttca atcatagtag
32761 aatagtgtgt ggtataaagc cgtgcagttt cacgaaggga cgagagcatc gatacagtga
32821 gtggcaaatg taacactttt tcttttgctg cttcaatcct cataaggcaa ttagcaattt
32881 tagaagtaat cgtataattt ggcatcggca taatatctgc attacattac ttctatatta
32941 tgctgataaa atgcagataa tacaacattt acacgacgcc gaccataaac agcaataaat
33001 tcggctgatt gagaaagaag tttgtcttgt agaatagcta tttctttttc tctgccaatt
33061 attggttcac tcatatgttt attccgcgga gtttgttgaa attatacaca gcggaataaa
33121 attatcaact ttgttttacc gccatgttta cagcgcggcg gtaaaagata gagcatctca
33181 taattggcta ttctgggggt agtttggtca cctacattat caagtcttct gatgaacttt
33241 gtgacaatat acctaatcat tgtacgtaaa gtttgtattc gaggcaaaac tgactataaa
33301 atacacttga tataaatgtt tttatatatt aacattagat gcttctttta atatatagat
33361 aaatgtgtac taaaagtttt atattgtagg ttctttaata agtatatgtc tagaatatcg
33421 tttttattga ttcttatact ggcagtagca agtttaccag taataaacaa ttggctttca
33481 caccgcggtc aaaccctaac cgatgaccaa ataagtacga gattagatga ttatatcagt
33541 agaaattttg ataagatcgt aaaaactctc aaagaggagt cgattaaaag tagttacgct
33601 gctcgagata acgcgactaa aggtaaaatt ttacagtaca aaaatgaaat attcgactcc
33661 gcttatcctt actcaggaaa tgaaaatagt agtgttatag ctgtaggctt ctttgactat
33721 tcttgtggat attgcaaagc tatgaagaat gatataaagc agttaattaa cgacggtaaa
33781 attaagtata tctttaggga tgctccaata cttggtaatg actctttaag ggcagcaaaa
33841 agcgctttag ctgtttactt tattgataaa gaaaaatatt tcgattttca ctattctgct
33901 ttaagtcaca aaggaggatt ttcagacgaa agcatattgg atatagtgaa aagcataggg
33961 attgatgagg acgattttaa tgattccata aaagataacg cggacaaaat tgagcaaatg
34021 ataaacaaca gtaggcttct agtaagagat ttgggagtag gtggtacacc tttttttaata
34081 attggggata gtctgtttgt aggagcaact gatttaaata tactacgcaa aaaagtagat
34141 gaattaagtc acaagcaagg ctagtttcaa tttgccataa aattttattt tttttgcttg
34201 acaactcaaa tgaacctgag tatagcaact gtgggtgtga taagtaacgg cacgtagaag

34261 tgacacagta gctcaagaaa agcaggtgat gcctatagtc aagcataacc tcagcctgca
34321 aagattgact aatccattaa aaagtgttaa actcaatata taattaccat tctggagtta
34381 caatatgaaa gaacctaatt atacaagaga accaattgct ctttttcgac aaatcaaaga
34441 aaagaaaaaa tcaagtaatt tattgcataa tttaaagagt gcttattcta aaaaactaaa
34501 gataggggct gctctaaaga caggggattg ctttttttgac tccgtggctc aagggttgaa
34561 tgagttaaaa cctggtcatc gttttaccgc aaaatcatta agaaaagatt gtgcagatta
34621 tgctgaaaac aaaaaggact cacgatgggt ttttgatgaa ataacaaaaa atattgctga
34681 aggatatgct gtacctatga taggttcaaa tggaaatgta atgaaagata accagaacaa
34741 tattatacca gacaatattg catcttttga tagatattta aaagatatta gaagtatggc
34801 tacagaagtt agcctaccta tatggggtcg cccagggatt gaagggagaa tgatatgtga
34861 taagtatgaa gtggagcttg atatttattc aatagaggaa gatagtcaaa aagttacagt
34921 aactaaaatt acagcacatg atgatgatct agaagttttt atacaaggtg tgaatcttga
34981 tcaattaaag atagataata gtaaaaaaac tgtaggagta gtgaattaca tgcaacattt
35041 tgtaccactc cttagtggaa tttctgaaga tataaaagaa gatcaacaag tttctagtga
35101 agcaattctt ggcacaggta aaagagttcc tacttctgat caatcagaga aagaagtaca
35161 acaagaaata attaatatca gtactagtgc gactgtttat acggataaag aaatcccttt
35221 ttctcatcaa ctagagcaaa aaacacgaca agaaggaatt aatgccagcg ctagtgcgac
35281 tgtttatacg gatagagaaa ttcctacttc taatcaatca gagcaaaaag tacaacaaga
35341 aataattaat accaacacta gtgcgccggt tttacaaatt tacacagata aaaaaatccc
35401 ttcctctcat cagcaagaac aaaaaacaca acaaaaagaa atacaaaaaa aattaatcaa
35461 tagttacagg aaagaggcta tttatgtaat agctactgta ataacagcta tagcattcac
35521 agcatctgct atagctgcta tcattactca agcaattcct tttgcatgtc ttgctggaac
35581 tgctttagtc gctgcatgtg tttgtgtata tcaattaacg cagagtcata aagacattaa
35641 cgaactgaaa agcgagttag gaaaaggtca agaagataat gcccacactt tcttagagaa
35701 ggtgggatta aaaatgcacg atcttattaa taaagcaatt tcaaaagttt aagatttttc
35761 ctactttatc tacatcaact caactttatt tctacaggag ggtatatgtc attcacagaa
35821 attagatttc cagaaaatat atcttatggt tccgctggag gaccggaatt ttccactgac
35881 gttgtaacaa ctcataacgg ttgtgaacag cgcaacatca attggtctca tgcacgcact
35941 agatacaaca tagcttacgg ggtcagatca aacgagcagc tactagaact aataacattt
36001 tttcatgcac gaaaaggtaa agcaataggg tttcgtttta aggattggtc ggattttata
36061 gccatcaacc aagagattgg tataggagat aacaaaaaaa cgacttttca gctaatcaaa
36121 acttatgtaa gtggggaaga taagcatata cgtatgatca aaaagccagt acatgggaca
36181 gtaaagattt atttaaatgg taaagaagag agtgaatatt cagtaaatta ttcaacggga
36241 gaaataacat tcatgaaacc gccagtaaaa gatgcaataa tcactgcgag cttcgaattt
36301 gatgtgcccg tgcgctttga tacagattac ttaaacgctt ctattgatga ctatggaagc
36361 aatagctgga ataacattcc actaatagag gtgaaatttt agctgtaagc gagcatagat
36421 ctacatcata gtagggctac tagtcaccat caattaaaaa agttaagcac cactgaggct
36481 ctatatttaa atgagcctta gtatatgcaa ttattttaaa ctttatcacc agctatatac
36541 tacaatgttg ctgagctttg ctaacattta cacttgaagc ttggggagat tctagcttag
36601 tgctttctcc ttcctgcaaa atagaaagct tcaacaattc ctttagagtt gattttgcaa
36661 gtgttgtatc atctttgttt aagatgtagt ctagcatgtt gtttttctgt aagcgattaa
36721 caaaatatct atcttcctca ttaagaacct ctatcttgtt aatacaagat aatactaaat
36781 ccgaattctt tgttgctaaa attccattca aaaagatatc taattttgtt ttgtcttcag
36841 tcaagtctct agctaaccta tttctgatgt tatcattatc aagcacagtg tactcgaggt
36901 acgagcaagg ctcaataagt ataaagtcct ctaaaggcac tagttctgga taggtgccag
36961 cgtaatcaag tatgccacac tcagtaatac accctccttt tcttataaca cagcaattga
37021 gacgaccatg atacattgat aacaaagaaa aaagacgtat tatacttgta cctctatctt
37081 tcatttcctc gaaaagcttc ctatcattta ttaatttgtg caaaatgtaa tccatagcag
37141 gacctggttt gcggaaatga aacatattat tctcttcaaa aatggcctca gctaattcga
37201 tagatttttt atcctcatct acgaaagctt tttcatttct atttaactgt atgatctcag
37261 ctttgctatc tctgcgtaga ccagttctcc ttattaacat ctcaaatatt tttgtattca
37321 tatatatcct atattagtta aagtaaatat taataatgtt tactttttaa ataaaaataa
37381 atgaaaattt taataaaaat tatttttttt aatagaataa aaatctttta tatttgcttg
37441 agtaccaaaa gggctttatt ggtgtaatgc gttttctatt gcgcctggct gcagcgcgtg
37501 atgcaaccgg attcaagtgt cagctacttt tatagcacca cttctgcttc aataactgta
37561 aatactaagc gatctgtctt aaaatctcct cttgttgctg agcatggaca ctagcataac
37621 cacatgcagg agatgcagca gcaggtctcg cgatacactt aggtcttttt gcttgaatat
37681 cgagattgga caacacttct tctatcaatg ggttgacaaa aaaccatcct cccatatttt
37741 ttggttcttc ttgacaccat ataatttcag cgtttttgta cttttcaagt tcattactta
37801 gtttatctgc aggaaacgga tagaattgtt ctaaacgtat cactgctata tcgtttattt

```
     37861 tttgtgtttc aagcatttca attatatcgt aataaacttt accgctgcat attacaactt
     37921 ttcgtacttt atcacttgaa actaaacctt ttctacattc tggaatcacc gtgaggaatt
     37981 ttccttcaaa atcggatagg ttagaaactg ctcttttatg acgcagtagt gatttaggtg
     38041 taaacactac taaaggcttg cgaaagtctc tatgcatttg tctgcgtaag acatggaagt
     38101 aattcgccgg agtggaacaa ttaactacct gcatattatc ctctgcgcag agttgcaaaa
     38161 atctctctat acgtgcggag ctatgctcag gtccctgtcc ttcataacca tgaggcagca
     38221 ataaaactag accgcttgat cgcaaccact ttgtttctgc agatgcgata aactggtcga
     38281 tcataatttg tgcaccattt gcaaaatcac caaattgccc ttcccagagc accagtgaat
     38341 acggggaatc aagactatat ccatattcaa agcccattac agcatactct gataaagcgc
     38401 tatctatgac ctcaaagtga gcttgctttt catttatatt gtttagcgga ataaacgctt
     38461 cttctgttac ctgatcaaca agccttgaat gacggtgcga gaaagttcca cgaccagagt
     38521 cttgtcctga taaacgcact cctattcctt ctgtgagcag tgatgcaaac gcaagacttt
     38581 cggcagttgc ccagtctata ttactaccgg aatttatgct gtctattctg ccatcaagta
     38641 tttttctaac cttattgtta atattaaagc tacttgggat gttgctattt atatgtacac
     38701 ctaatttttt caactcatct ggcggaacac cagaatccgt gtagtattcg ctcaaatcgt
     38761 tcaactttgc tcttcttaat tttgaccaca ctccaccgaa ccagtcagct ttttttggag
     38821 tataagccgc tgatt
//
LOCUS       JAQZAU010000050            3095 bp    dna     linear   ENV 17-MAR-2023
DEFINITION  MAG: Wolbachia pipientis isolate wEsol contig_33195, whole genome
            shotgun sequence.
ACCESSION   JAQZAU010000050  JAQZAU010000000
VERSION     JAQZAU010000050.1
DBLINK      BioProject: PRJNA929258
            BioSample: SAMN33142973
KEYWORDS    WGS; ENV; Metagenome Assembled Genome; MAG.
SOURCE      Wolbachia pipientis (insect metagenome)
  ORGANISM  Wolbachia pipientis
            Bacteria; Pseudomonadota; Alphaproteobacteria; Rickettsiales;
            Anaplasmataceae; Wolbachieae; Wolbachia.
REFERENCE   1  (bases 1 to 3095)
  AUTHORS   Fiutek,N., Couger,B.M., Roy,S., de la Torre,J.R. and Connor,E.F.
  TITLE     Genomic assessment of the contribution of the Wolbachia
            endosymbiont of Eurosta solidaginis to gall-induction
  JOURNAL   International Journal Molecular Sciences
REFERENCE   2  (bases 1 to 3095)
  AUTHORS   Fiutek,N., Couger,B.M., Roy,S., de la Torre,J.R. and Connor,E.F.
  TITLE     Direct Submission
  JOURNAL   Submitted (10-FEB-2023) Biology, San Francisco State University,
            1600 Holloway Ave, San Francisco, CA 94132, USA
COMMENT     ##Genome-Assembly-Data-START##
            Assembly Date          :: JUN-2019
            Assembly Method        :: FLYE v. 06-2019
            Genome Representation  :: Full
            Expected Final Version :: Yes
            Genome Coverage        :: 100.0x
            Sequencing Technology  :: Illumina HiSeq; PacBio Sequel
            ##Genome-Assembly-Data-END##
FEATURES             Location/Qualifiers
     source          1..3095
                     /organism="Wolbachia pipientis"
                     /mol_type="genomic DNA"
                     /submitter_seqid="contig_33195"
                     /isolate="wEsol"
                     /isolation_source="galls on Solidago altissima"
                     /host="Eurosta solidaginis MN-2018-EFC"
                     /db_xref="taxon:955"
                     /environmental_sample
                     /country="USA: Cowling Arboretum, Carleton College,
                     Northfield, MN"
```

```
                     /lat_lon="44.48 N 93.13 W"
                     /altitude="280 m"
                     /collection_date="2018-11"
                     /metagenome_source="insect metagenome"
                     /note="metagenomic"
     gene            300..614
                     /locus_tag="wEsol_00481"
     CDS             300..614
                     /locus_tag="wEsol_00481"
                     /codon_start=1
                     /transl_table=11
                     /product="hypothetical protein"
                     /pfam="PDDEXK_2"
                     /tigr="TIGR01784 T_den_put_tspse 273 conserved
                     hypothetical protein"
                     /translation="MALSKFLDPKLDLTFKRIFGTEKNKNILIHFLNDILGFTEINTI
                     QEVEFLSTIMDPEIASDKQSIVDVLCRDSSGARYVIEMQLTRDKGFEKRAQLYAAKAY
                     SR"
                     /besthit="qcoverage 100 , hcoverage 70.2702702702703 ,
                     similarity 100 , identity 99.0 , evalue 7.24e-65 ,
                     alnlength 104 , Bacteria , Proteobacteria ,
                     WP_174516951.1 Rpn family recombination-promoting
                     nuclease/putative transposase, partial [Wolbachia
                     endosymbiont of Cardiocondyla obscurior]"
     gene            825..1232
                     /locus_tag="wEsol_00482"
     CDS             825..1232
                     /locus_tag="wEsol_00482"
                     /codon_start=1
                     /transl_table=11
                     /product="hypothetical protein"
                     /pfam="PDDEXK_2 Pfam-B_9730 Pfam-B_12519 Pfam-B_10245"
                     /tigr="TIGR01784 T_den_put_tspse 273 conserved
                     hypothetical protein"
                     /translation="MDRWLFFFKYADETTEEDLRKIAEKAPIIKLAYDELDRFSWNEK
                     DLVAYEERIMDLRKEEAILEYRLDLATEKGKKIGKEEGKIEGKIEGKIEVAKAMLANN
                     VDVNTIVKFTGLSISEIEELSVNGYPYVIPPRR"
                     /besthit="qcoverage 100 , hcoverage 43.5483870967742 ,
                     similarity 100 , identity 99.3 , evalue 4.11e-72 ,
                     alnlength 135 , Bacteria , Proteobacteria ,
                     WP_197907250.1 Rpn family recombination-promoting
                     nuclease/putative transposase [Wolbachia endosymbiont of
                     Kradibia gibbosae]"
     gene            complement(1233..2273)
                     /locus_tag="wEsol_00483"
     CDS             complement(1233..2273)
                     /locus_tag="wEsol_00483"
                     /codon_start=1
                     /transl_table=11
                     /fullproduct="qcoverage 100 , hcoverage 100 , similarity
                     100 , identity 100 , evalue 3.11e-244 , alnlength 346 ,
                     Bacteria , Proteobacteria , WP_015588818.1 MULTISPECIES:
                     Mrp/NBP35 family ATP-binding protein [unclassified
                     Wolbachia]"
                     /cog="Mrp COG0489 265 ATPases involved in chromosome
                     partitioning"
                     /pfam="DUF59 Pfam-B_235 Pfam-B_2460 Pfam-B_3718 AAA_31
                     VirC1 MipZ Pfam-B_2271 Pfam-B_4013 CbiA ArsA_ATPase SRP54
                     Fer4_NifH ParA"
                     /product="Mrp/NBP35 family ATP-binding protein"
```

```
                     /translation="MINEKIVRENLKKVIEQKSGKDVIALGIISSIIIKGQDVGFVLE
                     VAGNTQANEELRRNCEQAVKAIPGVTKVTVVATCQKQTGQQKAKLHIEGVKNIIVVAS
                     GKGGVGKSTVALNLALSLAKLKHKVALVDADIYGPSIPKMLGAEKLKPEIQDSKAMPI
                     EKYGLHTISIGYFIDKDRAAIWRGPMITKALYSLLMGTKWSDIEYLIIDTPPGTGDVH
                     LSLMENFNLTGAIIVSTPQELSLIDARKIYDMFTKLSVPVIGIVENMSYFIQSGSKIY
                     IFGKDGAKKMSEELGIKLLGRVPLDPQICHASDCGNPLMLSEDLAGIYEGIAKDVIAK
                     VALQFEKPSFRY"
                     /besthit="qcoverage 100 , hcoverage 100 , similarity 100
                     , identity 100 , evalue 3.11e-244 , alnlength 346 ,
                     Bacteria , Proteobacteria , WP_015588818.1 MULTISPECIES:
                     Mrp/NBP35 family ATP-binding protein [unclassified
                     Wolbachia]"
     gene            complement(2281..2691)
                     /locus_tag="wEsol_00484"
     CDS             complement(2281..2691)
                     /locus_tag="wEsol_00484"
                     /codon_start=1
                     /transl_table=11
                     /fullproduct="qcoverage 100 , hcoverage 100 , similarity
                     99.3 , identity 99.3 , evalue 4.37e-81 , alnlength 136 ,
                     Bacteria , Proteobacteria , WP_174517022.1 MULTISPECIES:
                     hypothetical protein [unclassified Wolbachia]"
                     /pfam="PDDEXK_2 Pfam-B_5253"
                     /tigr="TIGR01784 T_den_put_tspse 273 conserved
                     hypothetical protein"
                     /product="hypothetical protein"
                     /translation="MKSTKLERSRMRLKVSRTVLKSSEEGRPSSLRQQYADETTEEDL
                     RKIAEKAPIIKLVYDELDRFSWNEKDLVAYEERIMDLLKEEGILAKKLDEGKIEGKIE
                     VAKNLLKADISIDIISQTTGLPQAEIKRLQEEIT"
                     /besthit="qcoverage 100 , hcoverage 100 , similarity 99.3
                     , identity 99.3 , evalue 4.37e-81 , alnlength 136 ,
                     Bacteria , Proteobacteria , WP_174517022.1 MULTISPECIES:
                     hypothetical protein [unclassified Wolbachia]"
     gene            complement(2688..2963)
                     /locus_tag="wEsol_00485"
     CDS             complement(2688..2963)
                     /locus_tag="wEsol_00485"
                     /codon_start=1
                     /transl_table=11
                     /pfam="HNH HNH_3"
                     /fullproduct="qcoverage 100 , hcoverage 100 , similarity
                     100 , identity 100 , evalue 2.74e-65 , alnlength 91 ,
                     Bacteria , Proteobacteria , WP_039963646.1 MULTISPECIES:
                     HNH endonuclease [unclassified Wolbachia]"
                     /translation="MLLNKVQKTRSPYDGDWVYWGKRLRKIPDKPLGVIKLLRLHQSK
                     CDNCRLWFKSDDTIKIHHKDQNRRNNMITNLSLLHGHCHDELHRRCA"
                     /product="HNH endonuclease"
                     /besthit="qcoverage 100 , hcoverage 100 , similarity 100
                     , identity 100 , evalue 2.74e-65 , alnlength 91 ,
                     Bacteria , Proteobacteria , WP_039963646.1 MULTISPECIES:
                     HNH endonuclease [unclassified Wolbachia]"
     gene            complement(2923..>3095)
                     /locus_tag="wEsol_00486"
     CDS             complement(2923..>3095)
                     /locus_tag="wEsol_00486"
                     /codon_start=1
                     /transl_table=11
                     /product="hypothetical protein"
                     /translation=""
ORIGIN
```

```
        1 tttatagaca aagacctata gctaacccaa gaaaggttgt aaccagactg cattctgtga
       61 tttttagcaa aacacgatct ttcatttgat ctttcttcaa tttaaagtaa atacagaaaa
      121 ttgctgccta tagctaaact gacttagatt taccgacaac cgtcatcccg ctacttgtta
      181 gcggctaaga gataccgcgg cggtatgacg gttcgcggcg gcatagctat cttcaaaaat
      241 atggttgaat aattgttaat aatagtgtat aattattaat atttttaagc actttggcta
      301 tggctttatc taagtttctc gaccctaagc tggatttaac ctttaaaaga atctttggca
      361 ctgaaaaaaa taagaatatt ctcatacatt ttttgaatga tattttaggg tttaccgaga
      421 taaatactat acaagaagtg gaattcctca gtactataat ggatcctgaa attgcctctg
      481 ataagcaaag tatagttgat gttctttgca gggactctag tggggctagg tatgtgattg
      541 aaatgcagct cactcgcgat aaaggcttcg aaaaacgagc tcaactatat gctgctaagg
      601 cttactcaag gtaagctgat aaatctggta agtacattga tttaaagaag gttttcttta
      661 ttgctatttc taattgtaat ttatttcctg ataagcttga ttatatatcc agccacacta
      721 taagagatga gaaaaccaat gagcatgact taaaagattt tcaatttata tttattgaac
      781 tacctaaatt tcctaagaat aaggaagaac agctagaaag catagtggat cgttggttat
      841 ttttcttcaa atatgcagat gaaactacgg aagaagactt aaggaaaata gcagaaaaag
      901 caccaataat aaagttagcc tacgatgaat tagacaggtt tagctggaat gaaaaagatt
      961 tagtagctta cgaagaaaga ataatggatc tacggaaaga agaagccatt cttgaataca
     1021 gacttgatct tgctactgaa aaaggcaaaa aaattggtaa agaggaaggc aaaattgaag
     1081 gcaaaattga agggaaaatc gaagtcgcaa aagcaatgct ggctaataat gttgatgtta
     1141 acactattgt caagtttact ggcctttcta ttagtgagat tgaagaatta agtgtgaacg
     1201 gttacccccta cgtcataccg ccgcggcgct aactagtagc ggaatgacgg tttttcaaat
     1261 tgtagagcca ctttagctat aacatcttta gcaatacctt cataaatccc tgctaaatct
     1321 tcacttaaca ttaaaggatt tccacaatca gaagcatggc atatttgtgg atctagagga
     1381 actctgccta gaagtttgat gcccagctcc tcagacattt tttttgcacc gtcttttcca
     1441 aatatgtata tttttgagcc gctttgaatg aaataactca tattttctac aatgccaata
     1501 accggtacgc tgagctttgt aaacatatca taaattttgc gagcatcgat taaagagagc
     1561 tcctgtggag ttgaaactat tatcgcccca gttaagttaa aattttccat aagacttaaa
     1621 tgcacatcgc cagttccagg tggtgtatca attatcaaat actctatatc ggaccatttt
     1681 gttcccatta gcagactata aagtgctttt gtaatcatag gtccgcgcca tattgctgca
     1741 cgatctttat cgataaaata gccaattgaa atagtatgca gtccatactt ttctataggc
     1801 attgctttac tatcctgtat ttctggcttt aatttttcag cgccaagcat tttaggaatt
     1861 gaaggaccat atatatctgc atcaaccagc gcaactttgt gttttaactt tgctaatgag
     1921 agagcaagat ttagcgcaac tgtggacttg cccacaccgc ctttaccaga ggccacaacg
     1981 attatatttt tcactccttc gatatgtaat ttagcttttt gttgcccagt ttgtttttga
     2041 caagtagcaa ccacggtcac ttttgtcacc cccggtatag ctttaacagc ttgctcacaa
     2101 tttcttctta attcttcatt tgcttgcgtg ttaccggcaa cttcaagcac aaaaccaaca
     2161 tcttgacctt taataataat tgaggatatt atgccaagag cgatcacatc tttaccgctt
     2221 ttttgctcta tgactttttt taagttctct cttacaattt tttcgttgat cattagcctt
     2281 ttaggtgatt tcttcctgaa gtcgtttaat ctcagcttga ggaaggccgg ttgtctgaga
     2341 gataatatca atagagatgt cagccttaag taggtttttt gctacttcaa tctttccttc
     2401 aatctttccc tcatcaagtt ttttagcgag gatgccttct tctttcagca gatccattat
     2461 tctttcttca tatgctacta aatctttttc attccagcta aacctgtcta attcatcgta
     2521 gactaacttt attattggtg ctttttctgc tattttcctt aagtcttctt ccgtagtttc
     2581 atctgcatat tgttgtctaa gtgaagaagg tcgcccctcc tcactcgact tcaaaaccgt
     2641 acgtgagact ttgagcctca tacggcttct ctctaatttg gtgcttttca tgcacacctc
     2701 ctgtgtaatt catcgtgaca atgtccatgt aacaaagata agtttgtgat catattgttt
     2761 cgtctattct ggtctttgtg atgtattttt attgtatcat cacttttaaa ccatagtcta
     2821 caattatcgc atttactttg gtgcaacctc aataatttta ttactcctag tggcttatct
     2881 ggtatttttc tcagacgttt accccaatat acccaatcac catcatatgg ggatctggtt
     2941 ttttgcactt tgtttaataa catgatcgct gtgtatagta agacgtacct tgttacttgt
     3001 cataaagcgc cagttttcgt tattatactt cttaaagtac tttcttttta tccagcattt
     3061 ccctttgttt gggtgcctaa agactgacct taccc
//
LOCUS       JAQZAU010000051           34015 bp    dna     linear   ENV 17-MAR-2023
DEFINITION  MAG: Wolbachia pipientis isolate wEsol contig_33339, whole genome
            shotgun sequence.
ACCESSION   JAQZAU010000051  JAQZAU010000000
VERSION     JAQZAU010000051.1
DBLINK      BioProject: PRJNA929258
            BioSample: SAMN33142973
```

```
KEYWORDS    WGS; ENV; Metagenome Assembled Genome; MAG.
SOURCE      Wolbachia pipientis (insect metagenome)
  ORGANISM  Wolbachia pipientis
            Bacteria; Pseudomonadota; Alphaproteobacteria; Rickettsiales;
            Anaplasmataceae; Wolbachieae; Wolbachia.
REFERENCE   1  (bases 1 to 34015)
  AUTHORS   Fiutek,N., Couger,B.M., Roy,S., de la Torre,J.R. and Connor,E.F.
  TITLE     Genomic assessment of the contribution of the Wolbachia
            endosymbiont of Eurosta solidaginis to gall-induction
  JOURNAL   International Journal Molecular Sciences
REFERENCE   2  (bases 1 to 34015)
  AUTHORS   Fiutek,N., Couger,B.M., Roy,S., de la Torre,J.R. and Connor,E.F.
  TITLE     Direct Submission
  JOURNAL   Submitted (10-FEB-2023) Biology, San Francisco State University,
            1600 Holloway Ave, San Francisco, CA 94132, USA
COMMENT     ##Genome-Assembly-Data-START##
            Assembly Date          :: JUN-2019
            Assembly Method        :: FLYE v. 06-2019
            Genome Representation  :: Full
            Expected Final Version :: Yes
            Genome Coverage        :: 100.0x
            Sequencing Technology  :: Illumina HiSeq; PacBio Sequel
            ##Genome-Assembly-Data-END##
FEATURES             Location/Qualifiers
     source          1..34015
                     /organism="Wolbachia pipientis"
                     /mol_type="genomic DNA"
                     /submitter_seqid="contig_33339"
                     /isolate="wEsol"
                     /isolation_source="galls on Solidago altissima"
                     /host="Eurosta solidaginis MN-2018-EFC"
                     /db_xref="taxon:955"
                     /environmental_sample
                     /country="USA: Cowling Arboretum, Carleton College,
                     Northfield, MN"
                     /lat_lon="44.48 N 93.13 W"
                     /altitude="280 m"
                     /collection_date="2018-11"
                     /metagenome_source="insect metagenome"
                     /note="metagenomic"
     gene            526..2079
                     /locus_tag="wEsol_00487"
     CDS             526..2079
                     /locus_tag="wEsol_00487"
                     /codon_start=1
                     /transl_table=11
                     /fullproduct="qcoverage 100 , hcoverage 100 , similarity
                     100 , identity 100 , evalue 0.0 , alnlength 517 ,
                     Bacteria , Proteobacteria , WP_015589241.1 MULTISPECIES:
                     UDP-N-acetylmuramoylalanine--D-glutamate ligase
                     [unclassified Wolbachia]"
                     /cog="MurD COG0771 448
                     UDP-N-acetylmuramoylalanine-D-glutamate ligase"
                     /pfam="ELFV_dehydrog 2-Hacid_dh_C ArgK Mur_ligase_M
                     Mur_ligase_C"
                     /product="
                     UDP-N-acetylmuramoylalanine--D-glutamate ligase"
                     /translation="MVRISYLAVQLNKYKNQNVAVFGLGKTGLSAINALTKSGARIYA
                     WDDHEEQIANTKMMYKKCNFIHPKEYNWHEISALVLSPGVPTEPHWIVKLARRFDCKI
                     KSDIELFLEAKTTNQKVIGVTGTNGKSTTTSLIGHILKSAGKKVAIGGNLGVPVLDLE
```

RDAEIYVIELSSFQLELMNEINVDISALLNITPDHIDRHGSMENYIATKLKLINGSEV
AVIGCDNEITADIFNKFTGDKIPISVTYSPMSFQRVTLESRKEKPLPTTQMTEGGARD
LISLAGNNLLDYSEQITGIDRNYLDPSVSYLDDKRGTGIQKISLKLENHSLSVSDVKI
NLISNAENIAATYAVCKVLGVDSNTIINGIKSFSGLRHRNELLGKIRNVFFVNDSKAT
NAKSSEKAILSYENINWIAGGRSKKGGIESLSKHFTRVRKAFLIGESTEAFANVMENK
VDYVKCCNLEDAFRLAFEEALNSAEEVTILLSPACASFDQWKNFEERGEAFCRMFENL
RYNHTCCLV"
/besthit="qcoverage 100 , hcoverage 100 , similarity 100
, identity 100 , evalue 0.0 , alnlength 517 , Bacteria ,
Proteobacteria , WP_015589241.1 MULTISPECIES:
UDP-N-acetylmuramoylalanine--D-glutamate ligase
[unclassified Wolbachia]"

```
gene            2086..2676
                /locus_tag="wEsol_00488"
CDS             2086..2676
                /locus_tag="wEsol_00488"
                /codon_start=1
                /transl_table=11
                /fullproduct="qcoverage 100 , hcoverage 100 , similarity
                100 , identity 100 , evalue 2.10e-133 , alnlength 196 ,
                Bacteria , Proteobacteria , WP_010082414.1 MULTISPECIES:
                COQ9 family protein [Wolbachia]"
                /cog="COG5590 COG5590 229 Uncharacterized conserved
                protein"
                /pfam="Pfam-B_17210 COQ9"
                /tigr="TIGR02396 diverge_rpsU 186 rpsU-divergently
                transcribed protein"
                /product="COQ9 family
                protein"
                /translation="MEQKLIIDKLIRVIPFEGISDETLLKVCTDLNLANSFCKFQNGI
                YSALEHIAEDLNSSMEAELRNSNLEDMKVRERVKLAIQIHLSNYAKLPNYRELLKNVL
                SFSVSPKNTYFSSKLLYRTISAIWYGIHDQSTDFNYYTKRAILAGVYLSTILFFINDY
                SEDFVDTLSFLDNRINNVMTFQKFKTRLKGIIGNFL"
                /besthit="qcoverage 100 , hcoverage 100 , similarity 100
                , identity 100 , evalue 2.10e-133 , alnlength 196 ,
                Bacteria , Proteobacteria , WP_010082414.1 MULTISPECIES:
                COQ9 family protein [Wolbachia]"
gene            2681..3013
                /locus_tag="wEsol_00489"
CDS             2681..3013
                /locus_tag="wEsol_00489"
                /codon_start=1
                /transl_table=11
                /fullproduct="qcoverage 100 , hcoverage 100 , similarity
                100 , identity 100 , evalue 1.06e-71 , alnlength 110 ,
                Bacteria , Proteobacteria , WP_006279274.1 MULTISPECIES:
                hypothetical protein [Wolbachia]"
                /translation="MIVGRKVLEHDPEKVCENKNIHADHKEHLVYREDNNGTTKYTSY
                EEKENKFIEEAHEYLIDSEFNKNFDLGQYRISNFSGNLTKIELELINEKYMQVQVNRE
                RKIILAIP"
                /product="hypothetical protein"
                /besthit="qcoverage 100 , hcoverage 100 , similarity 100
                , identity 100 , evalue 1.06e-71 , alnlength 110 ,
                Bacteria , Proteobacteria , WP_006279274.1 MULTISPECIES:
                hypothetical protein [Wolbachia]"
gene            complement(2971..3519)
                /locus_tag="wEsol_00490"
CDS             complement(2971..3519)
                /locus_tag="wEsol_00490"
                /codon_start=1
```

```
                     /transl_table=11
                     /fullproduct="qcoverage 100 , hcoverage 100 , similarity
                     100 , identity 100 , evalue 1.06e-126 , alnlength 182 ,
                     Bacteria , Proteobacteria , WP_141457227.1 MULTISPECIES:
                     16S rRNA (guanine(966)-N(2))-methyltransferase RsmD
                     [unclassified Wolbachia]"
                     /cog="COG0742 COG0742 187 N6-adenine-specific methylase"
                     /pfam="Cons_hypoth95 MethyltransfD12 Ubie_methyltran MTS
                     Methyltransf_31 Methyltransf_18 Pfam-B_47 Methyltransf_26
                     UPF0020 PrmA Methyltransf_15 Methyltransf_25
                     MethyltransfD12"
                     /tigr="TIGR00095 TIGR00095 194 RNA methyltransferase,
                     RsmD family"
                     /product="16S rRNA
                     (guanine(966)-N(2))-methyltransferase RsmD"
                     /translation="MLRIIAGKYRGRKITTGKHLAARPTMSIVREAIFNMLSSRKPIY
                     NLNVLDLFCGSGSFSFEALSRGAKHAFMVDSDYYNLQLPKKTAEDFGITDNVTLICCS
                     ANCLPQPISKCDIVFIDPPYNSNLVESTLNGLAHSGWLSDDALIILEIRKNEDFECNK
                     NFSIILERTYGIARIIFLSLLT"
                     /besthit="qcoverage 100 , hcoverage 100 , similarity 100
                     , identity 100 , evalue 1.06e-126 , alnlength 182 ,
                     Bacteria , Proteobacteria , WP_141457227.1 MULTISPECIES:
                     16S rRNA (guanine(966)-N(2))-methyltransferase RsmD
                     [unclassified Wolbachia]"
     gene            complement(3642..4304)
                     /locus_tag="wEsol_00491"
     CDS             complement(3642..4304)
                     /locus_tag="wEsol_00491"
                     /codon_start=1
                     /transl_table=11
                     /fullproduct="qcoverage 100 , hcoverage 100 , similarity
                     100 , identity 100 , evalue 3.39e-144 , alnlength 220 ,
                     Bacteria , Proteobacteria , CDR79215.1 hypothetical
                     protein WPAU_0843 [Wolbachia endosymbiont of Drosophila
                     simulans wAu]"
                     /translation="MQKEMRRGLKMKTNKNFKGLDYYKLQQDKDGNIKLNDYDDILNA
                     RRARVDLLRDNTSLDKDVNFAKLNKALSEKFVDNHSLLSEFDKEDKEIVQERIKVFSI
                     LESLEDLKSVKSEDLLEVSLLLSDLSFIERNVVLNTDQNSDLAGFKNLFIEKKASGEE
                     YSEEECRGFLDNIDKINTIIKLELESRIEGLNHDEKNDHDDLQDEIGQAINAAGKSEK
                     DN"
                     /product="hypothetical protein"
                     /besthit="qcoverage 100 , hcoverage 100 , similarity 100
                     , identity 100 , evalue 3.39e-144 , alnlength 220 ,
                     Bacteria , Proteobacteria , CDR79215.1 hypothetical
                     protein WPAU_0843 [Wolbachia endosymbiont of Drosophila
                     simulans wAu]"
     gene            complement(4264..7824)
                     /locus_tag="wEsol_00492"
     CDS             complement(4264..7824)
                     /locus_tag="wEsol_00492"
                     /codon_start=1
                     /transl_table=11
                     /pfam="TrbL"
                     /fullproduct="qcoverage 100 , hcoverage 100 , similarity
                     100 , identity 99.9 , evalue 0.0 , alnlength 1186 ,
                     Bacteria , Proteobacteria , WP_015589245.1 MULTISPECIES:
                     type IV secretion system protein [unclassified
                     Wolbachia]"
                     /translation="MQSRLGKCWFRVLLLAAYVLITGCSKNDMPFPRCISADYFGPKP
                     IAVSAHFSSDHDAFIPGDGEVVDPETGEINYGFHHNQVVEWEDTGLETNGDSLKVRVN
```

GAWTSWSNNNKKESKKGSYTLQSLEQLKYATKFEGKSGDNSLPDFHLVCNDYKPSAPQ
KISSKSNASCTVKCKCINEDDSANTVSRGAPCWFTNGHGAYLLFQRPGDDSPNKDLKS
MRNPSSPTVHLGYNSVAENGSGLFALDRDSTKLKDRNCDPVKLKKGWKIYVKILDRYY
LDNVGGYSFEFLSGVRQPSGVEFFDYIYNYLKCVLLINENCKEHFNSNDPAVAQAMFE
NIAEKSTSFHNFVLSLLVLFVMISSLLYLFGIIQETKHDMLIRMMKITLVIVLISPGS
FRFFYDHFLVLFVKGLEQLISIITNFAPNMNTGSIVQGDEAKLFSFMEDMFNKFFAYS
VWKKFAAFLHYQMWASLLMIPAIFIGIVLYFLLCLYAYIIFLSGFMGIAFLIAIMPLF
LISILFSPLKSLFEGWIKFCISFCLQSIMIFALLSLLASIIMNTFYRQLGFTVCYNKW
FEVKLCAPKWVFGGFCIVDKQYFGWTPGQIFVPYTLGEASPLNVDKNIEGIEKISREG
GTIKFTGGAGYIPLPPDYIEKGFRYIDYPFFNPIPGTKENPADHYIAESDMVVDNSCS
VKDLVRLTNSLSHASEKADILLLINNISKKTEIYCKQPGNESRCTNYKEIIEKVILQP
SLKSEIKSLVQDIIRQDGSCQSQNFYKVNRNYQENSDYQRVQDIQRGYLINAGEIFVL
FLLSFLMFSLREFVQQMGSSIAGGGFSVYNISSMYQASPLTGIMEEFKQQWQNFAGRG
LESLGSWATHLPDRLAGGAANLLGRVPRVGGVLKYAIDVPRKFVGATIEATKFATSPK
NDVDKLEEKIYRAFGVDKEDITHRRIGKYLDYYKGYVGSHLGYTIEDAMKFTWEHGLD
SIGVSRRTGEKDGYPNNLLFRAKSHRQDFLNKLHDDTIGRKREPEKDNPDQSNKVERE
LGTELVRDNHDQPLEEGDPLRLRGGGQPDKVVRELSTEQEDKPNQLQHDQPTEEDKPD
QSNEVTRNLGTEPKGSDEIDDFGSAFEGMEKRAEREGRRNQDKVYNLSPLFEMNTPEA
VEKRAEREEKRNREDSKRKLPEPMRKPDAKGDEKRIEDED"
/product="type IV secretion
system protein"
/besthit="qcoverage 100 , hcoverage 100 , similarity 100
, identity 99.9 , evalue 0.0 , alnlength 1186 , Bacteria
, Proteobacteria , WP_015589245.1 MULTISPECIES: type IV
secretion system protein [unclassified Wolbachia]"

gene complement(7914..10859)
/locus_tag="wEsol_00493"

CDS complement(7914..10859)
/locus_tag="wEsol_00493"
/codon_start=1
/transl_table=11
/pfam="TrbL"
/fullproduct="qcoverage 100 , hcoverage 100 , similarity
100 , identity 100 , evalue 0.0 , alnlength 981 ,
Bacteria , Proteobacteria , WP_174516844.1 MULTISPECIES:
type IV secretion system protein [unclassified
Wolbachia]"
/translation="MISKRSLLLILCLAITGCNMDCVEPGLQSKNTSVNVDVPVRKAD
KIHWVDSGQVISQGEKIKFTLDGSVNFCPFKEDKNPRKVLVPASFCADGSEPNYSKAA
DINSVPNNYGVNESKNELCGGKGFGSNRRYVDTGIKVNPGDKLSFSLVPRKITIDYDN
PEGKGINFDDNCYKAVENDRKETVYKKATVSEVHQGGEFFCDGKDGTKNKIQFLPLDK
TKAKESKVLVGNGYTPYDNKVHFNKGYIQDNEPWVYGSLLDLRRAKIGLNELCDGKNC
SLDERKKYSSYELNCYHQNICYTEKGIWNYGLGQAGERNCVSSIRYKKYDKGNKCDMY
SYLEKMEGNVKAGSFTEDDKSWAEALVAKIGDPDDKNIAKGIQCLPKKEGAKGDNMCL
QIGDNNKFEDFSLELNNDYPVNDEVTPGSSVMLAIADYGHYYYNRGGYHVKVTRSCEY
INGEKLYVYLGDEPPKDLHGVGTNDFKVVGVRERVNGKDEYTILDVISGEHFQNEESK
KIYFGINVSNVERDDITDKNGKYYENNKYTVTLFVKRKINDFISSNVNGVFKLIKEEV
IGDSVKDSYKGYARGLLQGVRALLTLYVIFTVVGYMLGTIQLSKFDFIVRMFKIAFIA
FAFSDRSWELFGTTLSRLFIDGSTYLVDSFSGYVGEGERKFAFLDLTAGVLFTAETWL
KFLSLMLSGPFGVIAFLAILCATYAFLKCIISATLKYVISTVLVAFLLSLTPLFIVFI
LFQKTKPLFDNWIKTLAHVSLEPVILFSFLSLLNQLMYSVLYNLTNFSACYQCLISVN
FLSYDLCLMKSILPLGYSPGTSVDVALSTGERAGGHFAALPIDLIQAFIYLIIAGAME
AFVSISETMVQTLFSSGYGVTGGVGTVAKSASQAMLSTVGLDDKTQSMIQSIKQKMSK
DRGKIEAKLPNTPKQPDKKEDSKGSSDKTTSDRPKTLKQSDQQKNFDETIRSDPSAKA
KNKEENGEE"
/product="type IV secretion
system protein"
/besthit="qcoverage 100 , hcoverage 100 , similarity 100
, identity 100 , evalue 0.0 , alnlength 981 , Bacteria ,
Proteobacteria , WP_174516844.1 MULTISPECIES: type IV

```
                     secretion system protein [unclassified Wolbachia]"
     gene            complement(10856..13243)
                     /locus_tag="wEsol_00494"
     CDS             complement(10856..13243)
                     /locus_tag="wEsol_00494"
                     /codon_start=1
                     /transl_table=11
                     /pfam="TrbL Pfam-B_6060"
                     /fullproduct="qcoverage 100 , hcoverage 100 , similarity
                     100 , identity 100 , evalue 0.0 , alnlength 795 ,
                     Bacteria , Proteobacteria , WP_174516845.1 MULTISPECIES:
                     type IV secretion system protein [unclassified
                     Wolbachia]"
                     /translation="MLKFLLIIPFFFLSGCGEHHCIKPENLSGLREKLDIVSTEQKWV
                     DSGVNISDRIRVTEIDIVPSKVNFCPKQYADFAIGPGRNDVALPFALKKGDTISFSVI
                     GSKVCKDKESETKIRYLKIDEKCSEQETEHFAHVLNQEKCQGGKDGICPNKYIIGDAQ
                     WLSGREYWFSKLDQDERKKEIENIINSIKQGENIDCSKLSESSASKIDTHILNSLCRR
                     ICKFSSGRRGEDCVYIEYNNIEIELFNALNSSGESVVAEIISNVLKGKQVKTDITFLK
                     VGMDNETLEHIPGGGLCTSCDHKMENDHQIPSLTFSLCSRNDKSCYNTNDKGGFNIRV
                     TKNHSLENSLYISVSDKFPEHKPGENQADISVDISKVHDMENLKGKLKDKTGTIYYGI
                     RDHGCDYKNEGQFSINLTTQEPPTKTFSAIYNFFDEKVKTAFFGSNYKDPNAIHSDTS
                     PVKSLYESFIKSSRTNTIRSTIISLLVLYIILYTLYYFFGLTHISIYEFLIICVKIGI
                     IAQLLQDNSWDFFYNNAFSIFINTPKQLIEIANFRGTTSNVFEFLDLPLNRFLSSHSV
                     LLIVSLIFSGPLGIVSFCLVIWGLITVSLSIFNALFSFITSIAIVALLLSLAPIFIIC
                     LLFAYTRQMFQNWVKNLARFAIHPVVLLIFISLISQVMDYIVYSVFDFEVCSTCILNI
                     NLKIFNPCIFYGYASKYTPNITAMMAFVILGHAMKALVEASSAISDSLFGAYVASEPG
                     RQYQQSLMGTVGLDERSVQRRERGDASQQSGTSRPQIPQGTQRSTPQIPQPGRQQ"
                     /product="type IV secretion
                     system protein"
                     /besthit="qcoverage 100 , hcoverage 100 , similarity 100
                     , identity 100 , evalue 0.0 , alnlength 795 , Bacteria ,
                     Proteobacteria , WP_174516845.1 MULTISPECIES: type IV
                     secretion system protein [unclassified Wolbachia]"
     gene            complement(13253..15817)
                     /locus_tag="wEsol_00495"
     CDS             complement(13253..15817)
                     /locus_tag="wEsol_00495"
                     /codon_start=1
                     /transl_table=11
                     /pfam="Pfam-B_18406 TrbL Pfam-B_18406 Pfam-B_18406"
                     /fullproduct="qcoverage 100 , hcoverage 100 , similarity
                     100 , identity 99.9 , evalue 0.0 , alnlength 854 ,
                     Bacteria , Proteobacteria , WP_015589248.1 MULTISPECIES:
                     type IV secretion system protein [unclassified
                     Wolbachia]"
                     /translation="MFKTKLSLLLIAIFLLNIDFLLSYPVLADGVSGTDKTEFVSRFN
                     STGSFSRASNPDCGAFKTAAALAGIAIAVGAIFTGIVLIVSSAGLFTALVIVGMIAAI
                     VGVWKAIGGLVVCEHSFVKHPVARDHDGKYKNFKLEDTGYSGCTNENYQTEEEYFSCL
                     QNKAREDVNKKDYDGKRAEKEALNSNVQESTESYYWPKNGVQYSEYIEVCHRNPLTFG
                     NLFNKVDFNSRGKPGYIDFDVREKDTGYVDGSWSSKVDGGLECRVLKAGQSEPIHGAT
                     FKAVRKTGRLCVELASVSMLGIEMTPWPQGVDMGCTELPPDPLAPMCEKSVMIFKNKD
                     GTGEEKVTIGNNDDYKTIIRSKEKGLNGQGGKIFVGYDNAGCFSSYVSEACYNQAGSK
                     SLAPIPVTSMIVQCIKESLDNLVAGKGAPNGESFISVAQKRLKNTVTAVLVLALILFS
                     IKAMSGGVQRPQEMYMLIIKFALVIYFTTGSTISHYYGELTRLSNGLSEIVLKASSES
                     KGICNYKAGTDYEYTRAGKRVSYSYLAPWDRLDCRILFYLGAPLDGIGGKIGTGGVAT
                     LAVLLGAAPVLLVAGSVIGIIFAGGQILVALVCIFMAVLMMMVILWLCYVFILSLVAL
                     SVIIILSPLFIPMVLFQHTKGYFEGWVKELITYSLYPVILFAFLSFMFIACDKIYFKN
                     LNFELDESYKNEQPDKSYEKKQAEISYSKKKQWFKLKDGECDKNETTLACMMQNYSFK
                     KSSILGLFDFTYMEFGSSLIGELLKLCLVLFLFYHFLNILPGMAAELAGNHRAALGSG
                     QTPGQMVGKALSAAKAAAGGVAGVAGAAAKKLSGGDKSKSRGSGSSSSESVNKSTE"
```

```
                     /product="type IV secretion
                     system protein"
                     /besthit="qcoverage 100 , hcoverage 100 , similarity 100
                     , identity 99.9 , evalue 0.0 , alnlength 854 , Bacteria ,
                     Proteobacteria , WP_015589248.1 MULTISPECIES: type IV
                     secretion system protein [unclassified Wolbachia]"
     gene            complement(15810..18215)
                     /locus_tag="wEsol_00496"
     CDS             complement(15810..18215)
                     /locus_tag="wEsol_00496"
                     /codon_start=1
                     /transl_table=11
                     /fullproduct="qcoverage 100 , hcoverage 100 , similarity
                     100 , identity 100 , evalue 0.0 , alnlength 801 ,
                     Bacteria , Proteobacteria , WP_010962886.1 MULTISPECIES:
                     VirB4 family type IV secretion/conjugal transfer ATPase
                     [Wolbachia]"
                     /cog="VirB4 COG3451 796 Type IV secretory pathway, VirB4
                     components"
                     /pfam="CagE_TrbE_VirB AAA_23 TrwB_AAD_bind AAA_10 AAA_22"
                     /tigr="TIGR00929 VirB4_CagE 792 type IV
                     secretion/conjugal transfer ATPase, VirB4 family"
                     /product="VirB4 family type
                     IV secretion/conjugal transfer ATPase"
                     /translation="MLRFRAIQSKNKSTLNREVHAAEFIPYSCYWNSTTLMTKENWLV
                     KVIKLSGFAFETADDEDLVIQNNIRNQMLRSISSPAFSLYFHTIRRKKNIFSDEFASQ
                     GLPNFFANHVNLKWREKHATRQSFINDLYITIIRRADTKGVEFLSHLLKKFGHVTSKH
                     AWESDIRATYEDLEETTNRVVTSLRNYSPKILGVKETPDGLFCEIMEFLSRIVNCGFV
                     TNTLFPLRTEISRYLPVHRLFFGRKMIQVVTHNESKYAGIISIKEYGNHTSAGMLDSF
                     LQLPYEFIITQSFQFINRQMAIGKMQIQQNRMIQSADKAISQIAEISQALDDAMSGKI
                     AFGQHHLTILCIEKSPKSLDNALSLVESELSNCGVYPIRERVNLEPAFWAQIPGNFDY
                     IVRKATISSLNLAGFASQHNYPTGNKFNNHWGDAVTVFDTTSGTPFFFNFHIRDVGHT
                     MIIGPTGAGKTVLMNFLCAQAMKFSPRIFFFDKDRGAEIFLRALSGIYTVIEPRTKTN
                     FNPLQLDDTSDNRTFLMEWIKSLISVYNDKFTSEDIARINDAIEGNFKLRKEDRFLRN
                     LVPFLGLAGPDTLAGAISMWHDNGSHAAIFDNKEDLLDFSRARVFGFEMASLLKDPVA
                     LGPVLIYLFHRISISLDGTPSIIVLDEAWALIDNPVFAPKIKDWLKVLRKLNAFVIFA
                     TQSVEDASKSTISDTLVQQTATQIFLPNLKATSVYRDVFMLTEREYILIKHTDPSTRF
                     FLVKQGVNAVVARIDLKGLDDIINVLSGRAESVLLLHDILNEVGDNPKVWLPIFYQKV
                     KNV"
                     /besthit="qcoverage 100 , hcoverage 100 , similarity 100
                     , identity 100 , evalue 0.0 , alnlength 801 , Bacteria ,
                     Proteobacteria , WP_010962886.1 MULTISPECIES: VirB4
                     family type IV secretion/conjugal transfer ATPase
                     [Wolbachia]"
     gene            complement(18225..18521)
                     /locus_tag="wEsol_00497"
     CDS             complement(18225..18521)
                     /locus_tag="wEsol_00497"
                     /codon_start=1
                     /transl_table=11
                     /fullproduct="qcoverage 100 , hcoverage 100 , similarity
                     100 , identity 100 , evalue 1.26e-62 , alnlength 98 ,
                     Bacteria , Proteobacteria , WP_007548615.1 MULTISPECIES:
                     type IV secretion system protein VirB3 [Wolbachia]"
                     /cog="VirB3 COG3702 105 Type IV secretory pathway, VirB3
                     components"
                     /pfam="VirB3"
                     /product="type IV secretion
                     system protein VirB3"
                     /translation="MSTGSIQTDQLFKGLTRPAMLFGVSYMFAILNVLICMLIFINSN
```

DLRVILLMLPGIHGLGYIASAKEPLFIELFMVKLGKCSKCLNRFYHGANSYDIA"
/besthit="qcoverage 100 , hcoverage 100 , similarity 100
, identity 100 , evalue 1.26e-62 , alnlength 98 ,
Bacteria , Proteobacteria , WP_007548615.1 MULTISPECIES:
type IV secretion system protein VirB3 [Wolbachia]"
gene 18630..20165
/locus_tag="wEsol_00498"
CDS 18630..20165
/locus_tag="wEsol_00498"
/codon_start=1
/transl_table=11
/fullproduct="qcoverage 100 , hcoverage 100 , similarity
99.8 , identity 99.8 , evalue 0.0 , alnlength 511 ,
Bacteria , Proteobacteria , WP_015589250.1 MULTISPECIES:
lysine--tRNA ligase [unclassified Wolbachia]"
/cog="LysS COG1384 521 Lysyl-tRNA synthetase (class I)"
/pfam="tRNA-synt_1f Pfam-B_854"
/tigr="TIGR00467 lysS_arch 515 lysine--tRNA ligase"
/product="lysine--tRNA
ligase"
/translation="MTSWPFQEAEKILQEFPNKKEIIFETGYGPSGLPHIGTFGEVFR
TTVIVNALKKIAPGIKTKIIAVSDDMDGLRKIPDNVPNQEMLREHLNKPLTMIPDPFG
THQSYGHHMNSLLCKFLDMFEFEYEFRSATECYKSGVYDEKLLLLLKNYDKVMDVMLP
SFREERQQTYSPFLPICPKTSQVLQVPVIETNTEKGTITYEDPNGEKIEVPVTKGKCK
LQWKPDWGMRWAAFGVNYEAHGKDLTPSAVLSSQICEILGEKPPLLFCYELFLDKERK
KISKSKGNGISIEEWLTYAPTESLALYIFQSPKKAKRLYFDVIPKSTDEYLEFVKRYH
ENKEKDINNPAWHIHQGNVPSIETSGINFSLLLNLAAACNAENKEILWGFISSYAPNV
TPENNKMLDRLSDFAVKYYHDFIKPTKSYKTPNEKEKAALLDLKDTLHSLSTTATAEE
IQSQVFSIGKKYDYTNLRDWFQLLYETLLGQKTGPRMGSFIKLYGIGNTISLIESATK
CIL"
/besthit="qcoverage 100 , hcoverage 100 , similarity 99.8
, identity 99.8 , evalue 0.0 , alnlength 511 , Bacteria ,
Proteobacteria , WP_015589250.1 MULTISPECIES:
lysine--tRNA ligase [unclassified Wolbachia]"
gene complement(21099..23732)
/locus_tag="wEsol_00499"
CDS complement(21099..23732)
/locus_tag="wEsol_00499"
/codon_start=1
/transl_table=11
/fullproduct="qcoverage 100 , hcoverage 100 , similarity
99.9 , identity 99.9 , evalue 0.0 , alnlength 877 ,
Bacteria , Proteobacteria , WP_141457239.1 MULTISPECIES:
alanine--tRNA ligase [unclassified Wolbachia]"
/cog="AlaS COG0013 879 Alanyl-tRNA synthetase"
/pfam="tRNA-synt_2c tRNA_SAD"
/tigr="TIGR00344 alaS 847 alanine--tRNA ligase"
/product="alanine--tRNA
ligase"
/translation="MKLNEIRERFIKFFVNNDHEQVSSSPLIPEHDSTLMFTNAGMVQ
FKNIFTGAQKTEMKRAVSSQKCLRAGGKHNDLENVGYTTRHHTFFEMLGNFSFGDYFK
ETAIEFAWEFITKELSLDKNRLSITVYHTDDEAYEIWRKISGFSSDKIIRITTDDNFW
SMGSTGPCGPCSEIFYDHGSPNLQEGDRIVEIWNLVFMEFNKDEEGNLHKLPKKCIDT
GMGLERIAAVMQNVHDNYDIDLFSALINKSQEYCGRTENKIAHKIVADHLRAAAFLIA
EGVLPGNEGRNYVLRRLIRRATRYIHLLGYNDSLLHRIFPVLIDSTSSAYMGAELVRA
KSLIETTLKSEEENFKDTLMKGISLLEKFTADLKPGDTLPGELAFKLYDTYGFPLDIT
LDILKEKKINFDQKGFDDAMGEQKERARAKWAGSGEKSVEQVWFDLIDKFGKTKFVGY
EFNEVNDAKILAIVSSKNEVIDSAKEGEKITIILDKTPFYGESGGQVGDTGSLIKSDR
SIIIVENTNKVNDLYLHRCIVKFGSICKGNTVAASIDKERRQTLRRNHSATHLLHFVL
RKILGDHVTQKGSLVAPDKLRFDFSHNTQVTQDQLFLVEDMVNSLIRENLSTSTKIQS

MNQAIDEGAMALFGEKYGNQVRVVKIGDSRELCGGTHVEHTGEIGLFKIVTESSVAFG
VRRIEALTGQEAINYVRDNEINLKKVAEFVKAPASEIINRLNILNQERKESEAKIKNL
YKKLISAESIKSTEINGINFVSHAFTNIPANIIREFVLQQQKPKTIIAFTATEKDKTV
LIIKVSKDLANKISAKELVSIAVEKNCGGNAELAQTGCDSNKIDDAITAICSKIAASK
HSTSYHHEPSYRRGANKQRDT"
/besthit="qcoverage 100 , hcoverage 100 , similarity 99.9
, identity 99.9 , evalue 0.0 , alnlength 877 , Bacteria ,
Proteobacteria , WP_141457239.1 MULTISPECIES:
alanine--tRNA ligase [unclassified Wolbachia]"

```
gene            23898..24341
                /locus_tag="wEsol_00500"
CDS             23898..24341
                /locus_tag="wEsol_00500"
                /codon_start=1
                /transl_table=11
                /fullproduct="qcoverage 100 , hcoverage 100 , similarity
                100 , identity 100 , evalue 5.38e-103 , alnlength 147 ,
                Bacteria , Proteobacteria , WP_015589252.1 MULTISPECIES:
                PAS domain-containing protein [unclassified Wolbachia]"
                /cog="COG5388 COG5388 209 Uncharacterized protein
                conserved in bacteria"
                /pfam="Pfam-B_17152 PAS_5"
                /product="PAS
                domain-containing protein"
                /translation="MEIYVGKEKRIANVVTQHWNDIKGSDRDWPERHEIDTAEIMESW
                QHCFIIEVKDQGYICENAGEKAVEFYGFENKTRIDNKYAIDAPFLRLYKIDAVIDKLD
                TVIESKCPINEEEESESVKMRQVLLPLGNKEGITHILGVITFKLL"
                /besthit="qcoverage 100 , hcoverage 100 , similarity 100
                , identity 100 , evalue 5.38e-103 , alnlength 147 ,
                Bacteria , Proteobacteria , WP_015589252.1 MULTISPECIES:
                PAS domain-containing protein [unclassified Wolbachia]"
gene            24440..24646
                /locus_tag="wEsol_00501"
CDS             24440..24646
                /locus_tag="wEsol_00501"
                /codon_start=1
                /transl_table=11
                /fullproduct="qcoverage 100 , hcoverage 100 , similarity
                100 , identity 100 , evalue 2.16e-36 , alnlength 68 ,
                Bacteria , Proteobacteria , WP_015589253.1 MULTISPECIES:
                50S ribosomal protein L35 [unclassified Wolbachia]"
                /cog="RpmI COG0291 65 Ribosomal protein L35"
                /pfam="Ribosomal_L35p"
                /tigr="TIGR00001 rpmI_bact 63 ribosomal protein L35"
                /product="50S ribosomal
                protein L35"
                /translation="MKKIKLKTKSSVKKRFHLTAKGKVISTQSGKRHGMVKRSKSNIR
                NQRGTTILGKSDSRIVKLYMPYGI"
                /besthit="qcoverage 100 , hcoverage 100 , similarity 100
                , identity 100 , evalue 2.16e-36 , alnlength 68 ,
                Bacteria , Proteobacteria , WP_015589253.1 MULTISPECIES:
                50S ribosomal protein L35 [unclassified Wolbachia]"
gene            24667..25032
                /locus_tag="wEsol_00502"
CDS             24667..25032
                /locus_tag="wEsol_00502"
                /codon_start=1
                /transl_table=11
                /fullproduct="qcoverage 100 , hcoverage 100 , similarity
                100 , identity 100 , evalue 6.56e-78 , alnlength 121 ,
```

```
                     Bacteria , Proteobacteria , WP_015589254.1 MULTISPECIES:
                     50S ribosomal protein L20 [unclassified Wolbachia]"
                     /cog="RplT COG0292 118 Ribosomal protein L20"
                     /pfam="Ribosomal_L20"
                     /tigr="TIGR01032 rplT_bact 114 ribosomal protein L20"
                     /product="50S ribosomal
                     protein L20"
                     /translation="MARVKRGVTTHARHKKILKLAKGYRGRAKSCYRIALQRVEKALQ
                     YAYRDRRNRKRDFRGLWIIRINAAVREHGLTYGRFMHGLTLAGIDLNRKILAEMAVNY
                     KDDFAKLVEAVSGKLAENS"
                     /besthit="qcoverage 100 , hcoverage 100 , similarity 100
                     , identity 100 , evalue 6.56e-78 , alnlength 121 ,
                     Bacteria , Proteobacteria , WP_015589254.1 MULTISPECIES:
                     50S ribosomal protein L20 [unclassified Wolbachia]"
     gene            25060..25944
                     /locus_tag="wEsol_00503"
     CDS             25060..25944
                     /locus_tag="wEsol_00503"
                     /codon_start=1
                     /transl_table=11
                     /fullproduct="qcoverage 100 , hcoverage 98.3277591973244
                     , similarity 100 , identity 100 , evalue 4.19e-208 ,
                     alnlength 294 , Bacteria , Proteobacteria ,
                     WP_015589255.1 MULTISPECIES: methionyl-tRNA
                     formyltransferase [unclassified Wolbachia]"
                     /cog="Fmt COG0223 307 Methionyl-tRNA formyltransferase"
                     /pfam="Formyl_trans_N Formyl_trans_C"
                     /tigr="TIGR00460 fmt 315 methionyl-tRNA
                     formyltransferase"
                     /product="methionyl-tRNA
                     formyltransferase"
                     /translation="MGSPEFAVSTLNLLLKSQNKIVAVYTKAPKPSGRGQKPTKSPVH
                     VIAEESNIEVCTPISLKFSAEQEKFRNFKPDVAVVAAYGLILPREILNIPKYGCINIH
                     PSLLPRWRGAAPIQHTILAGDQETGVSIMQLDEGLDSGPILKQEKFLIEKNDNYKTLH
                     DKLSELGSDLLLEVLNEIEKQLLLKQNDNDACYANKVEDYKIYASDACEVAYRKVKAF
                     YPKAFIKIENKRIRILDADFEALASEQGKIVNDNMHISLKGGTLIPKVVQMEGRNPCS
                     IEDFIRGLKSSMVKKFIE"
                     /besthit="qcoverage 100 , hcoverage 98.3277591973244 ,
                     similarity 100 , identity 100 , evalue 4.19e-208 ,
                     alnlength 294 , Bacteria , Proteobacteria ,
                     WP_015589255.1 MULTISPECIES: methionyl-tRNA
                     formyltransferase [unclassified Wolbachia]"
     gene            26025..27536
                     /locus_tag="wEsol_00504"
     CDS             26025..27536
                     /locus_tag="wEsol_00504"
                     /codon_start=1
                     /transl_table=11
                     /fullproduct="qcoverage 100 , hcoverage 100 , similarity
                     99.8 , identity 99.8 , evalue 0.0 , alnlength 503 ,
                     Bacteria , Proteobacteria , WP_015589256.1 MULTISPECIES:
                     bifunctional phosphoribosylaminoimidazolecarboxamide
                     formyltransferase/IMP cyclohydrolase [unclassified
                     Wolbachia]"
                     /cog="PurH COG0138 515 AICAR transformylase/IMP
                     cyclohydrolase PurH (only IMP cyclohydrolase domain in
                     Aful)"
                     /pfam="MGS AICARFT_IMPCHas"
                     /tigr="TIGR00355 purH 511
                     phosphoribosylaminoimidazolecarboxamide
```

```
                     formyltransferase/IMP cyclohydrolase"
                     /product="bifunctional
                     phosphoribosylaminoimidazolecarboxamide
                     formyltransferase/IMP cyclohydrolase"
                     /translation="MKIKRALISVYDKTNIIDLASFLMQQQIEILSTGNTYKALSDAG
                     IKTQEVSDYTQFPEILGGRVKTLHPKIHGGILYDREKHKKEVQDLEIEPIDLLITNLY
                     PFWETVSSSSNEEQIIEQIDIGGVALIRAAAKNFRFTSVISRIQDYEALKAEMIENNN
                     KTTLEYRKHLATKAFALTAHYDSNIHSWFLSQSKNNELPEFFALYGHKVQELRYGENP
                     HQKAAFYSNQFTEYPLEKLHGKELSYNNIVDIESALNITSEFEEPAAVIIKHNNPCGA
                     AIGNNALEAYEKALSCDEVSSFGGIVALNREIDLKLAEKLNEIFLEVVIAPSVNNEAL
                     KILQRKKNLRVIIHKSFQQNVKYQTKNVVGGFLVQENNDHTIKAEQVTECTATDKEKK
                     DLIFAWKICKHVKSNAIVIAKDGCAIGIGAGQTSRIDSVNIAVKKAGEKCKGAVLASD
                     AFFPFPDSIVESTKHGITAIIQPGGSLKDQDVIKAANENKIAMFFTGVRSFFH"
                     /besthit="qcoverage 100 , hcoverage 100 , similarity 99.8
                     , identity 99.8 , evalue 0.0 , alnlength 503 , Bacteria ,
                     Proteobacteria , WP_015589256.1 MULTISPECIES:
                     bifunctional phosphoribosylaminoimidazolecarboxamide
                     formyltransferase/IMP cyclohydrolase [unclassified
                     Wolbachia]"
     gene            28377..29897
                     /locus_tag="wEsol_00505"
     CDS             28377..29897
                     /locus_tag="wEsol_00505"
                     /codon_start=1
                     /transl_table=11
                     /fullproduct="qcoverage 100 , hcoverage 100 , similarity
                     100 , identity 100 , evalue 0.0 , alnlength 506 ,
                     Bacteria , Proteobacteria , WP_015589257.1 MULTISPECIES:
                     2,3-bisphosphoglycerate-independent phosphoglycerate
                     mutase [unclassified Wolbachia]"
                     /cog="GpmI COG0696 509 Phosphoglyceromutase"
                     /pfam="Metalloenzyme iPGM_N Phosphodiest Alk_phosphatase
                     Sulfatase"
                     /tigr="TIGR01307 pgm_bpd_ind 504
                     2,3-bisphosphoglycerate-independent phosphoglycerate
                     mutase"
                     /product="
                     2,3-bisphosphoglycerate-independent phosphoglycerate
                     mutase"
                     /translation="MNIKSVVLCILDGWGNGIENNKYNAISNANPPCWQYISSNYPKC
                     SLSACGTDVGLPGGQIGNSEVGHMNIGSGRVVMQSLQRINQEIGTIENNVNLQSFIND
                     LKSKNGICHLVGLVSDGGVHSHQKHISTLANKISQHGIKVVIHAFLDGRDTLPNSGKK
                     CIQEFIKSIKGNDIRIATVSGRYYAMDRDNRWERTIEAYEAIAFAKAPRHDNAVSLID
                     ENYQNNITDEFVRPAVIGDYQGIKPEDGVLLANFRADRMIQLASILLGKTDYIEVAKL
                     SSILSMMKYKEDLKIPHIFPPTSFADTLGQTIADNKLQQLRIAETEKYAHVTFFFNCG
                     KEEPFSGEERILIPSPKVQTYDLQPEMSAFELTEKLVEKIHSQEFALIVVNYANPDMV
                     GHTGNIKAAEKAVLAVDDCLAKVLSAVKKSSNTALIVTADHGNVECMFDEENNTPHTA
                     HTLNKVPFIVSCDNLKLRDGRLSDIAPTILQLLGIKKPNEMTGSSLISCITLCHSS"
                     /besthit="qcoverage 100 , hcoverage 100 , similarity 100
                     , identity 100 , evalue 0.0 , alnlength 506 , Bacteria ,
                     Proteobacteria , WP_015589257.1 MULTISPECIES:
                     2,3-bisphosphoglycerate-independent phosphoglycerate
                     mutase [unclassified Wolbachia]"
     gene            complement(29937..30917)
                     /locus_tag="wEsol_00506"
     CDS             complement(29937..30917)
                     /locus_tag="wEsol_00506"
                     /codon_start=1
                     /transl_table=11
                     /fullproduct="qcoverage 100 , hcoverage 100 , similarity
```

```
                     100 , identity 100 , evalue 2.13e-225 , alnlength 326 ,
                     Bacteria , Proteobacteria , WP_015589258.1 MULTISPECIES:
                     ACP S-malonyltransferase [unclassified Wolbachia]"
                     /cog="FabD COG0331 310 (acyl-carrier-protein)
                     S-malonyltransferase"
                     /pfam="Acyl_transf_1 Pfam-B_728"
                     /tigr="TIGR00128 fabD 290 malonyl CoA-acyl carrier
                     protein transacylase"
                     /product="ACP
                     S-malonyltransferase"
                     /translation="MIFAFPGQGSQFVGMGRSLYSEFSVARQVFNEVDSILDGKLSHL
                     IFNGPIEELTITENAQPAIMAVSIAMLRVMEHVFGKSLFTDHNVKYVCGHSVGEYTAL
                     CAAGALTLESAIKLLKVRSEAMHEASLKCKGGMVALLGAEINEVEDILKSVQIDGICE
                     IANDNGGGQVVISGTREALEMLPDLFKNSSVRKLIKLQVSGPFHSSLMKPADEKVLEF
                     LKGIKITRPIVPLISNVTAKEESDPKVIKTLLAKQVVSRVKWREMVLYMSSRGINKFV
                     EIGPNKVLSNLVKRIDQSISTKNIDSIGDIDSFFNESLVLTAKNLKVGLS"
                     /besthit="qcoverage 100 , hcoverage 100 , similarity 100
                     , identity 100 , evalue 2.13e-225 , alnlength 326 ,
                     Bacteria , Proteobacteria , WP_015589258.1 MULTISPECIES:
                     ACP S-malonyltransferase [unclassified Wolbachia]"
     gene            31046..31249
                     /locus_tag="wEsol_00507"
     CDS             31046..31249
                     /locus_tag="wEsol_00507"
                     /codon_start=1
                     /transl_table=11
                     /fullproduct="qcoverage 100 , hcoverage 100 , similarity
                     100 , identity 100 , evalue 3.19e-42 , alnlength 67 ,
                     Bacteria , Proteobacteria , WP_007548602.1 MULTISPECIES:
                     50S ribosomal protein L31 [Wolbachia]"
                     /cog="RpmE COG0254 75 Ribosomal protein L31"
                     /pfam="Ribosomal_L31 Pfam-B_2961"
                     /tigr="TIGR00105 L31 68 ribosomal protein L31"
                     /product="50S ribosomal
                     protein L31"
                     /translation="MNYHKIIIVMTDGQEFETRSTYGKEGDRVKLDRDPLTHPAWTGS
                     LTSGLASKTSKLAKFNDKYGSIF"
                     /besthit="qcoverage 100 , hcoverage 100 , similarity 100
                     , identity 100 , evalue 3.19e-42 , alnlength 67 ,
                     Bacteria , Proteobacteria , WP_007548602.1 MULTISPECIES:
                     50S ribosomal protein L31 [Wolbachia]"
     gene            31288..32082
                     /locus_tag="wEsol_00508"
     CDS             31288..32082
                     /locus_tag="wEsol_00508"
                     /codon_start=1
                     /transl_table=11
                     /fullproduct="qcoverage 100 , hcoverage 100 , similarity
                     100 , identity 100 , evalue 4.52e-189 , alnlength 264 ,
                     Bacteria , Proteobacteria , WP_141457245.1 MULTISPECIES:
                     NAD kinase [unclassified Wolbachia]"
                     /cog="nadF COG0061 281 NAD kinase"
                     /pfam="NAD_kinase Pfam-B_15665"
                     /product="NAD kinase"
                     /translation="MHKYKNIGYVASPSPKSQEVSKLLKKLNFINITEENKSEIDLLI
                     VVGGDGFMLRTLHNYVIENKNMHVYGVNTGNVGFLMNKCFSRSEDLIDHIEHATSTQL
                     TLLKMEATDTSGKRYHYIAVNEVYVFRKANQIVEMNITINDKLKVEKFRGDGVILSTP
                     TGSTAYNFSAGGPILPLNSNLLALTSINSYYPRHWNGALISNDTIVQIDINDTKNRPA
                     LVVSDYKEFHDISQIKIQKDHENTITLLFDKDYPLNERIFDRQFLY"
                     /besthit="qcoverage 100 , hcoverage 100 , similarity 100
```

```
                     , identity 100 , evalue 4.52e-189 , alnlength 264 ,
                     Bacteria , Proteobacteria , WP_141457245.1 MULTISPECIES:
                     NAD kinase [unclassified Wolbachia]"
     gene            32398..33861
                     /locus_tag="wEsol_00509"
     CDS             32398..33861
                     /locus_tag="wEsol_00509"
                     /codon_start=1
                     /transl_table=11
                     /fullproduct="qcoverage 100 , hcoverage 100 , similarity
                     100 , identity 100 , evalue 0.0 , alnlength 487 ,
                     Bacteria , Proteobacteria , WP_174516898.1 hypothetical
                     protein [Wolbachia endosymbiont of Cardiocondyla
                     obscurior]"
                     /translation="MHGSVEKSTVKLTSYEQTGDNSKITLKDPPSPEEAGNAISSVLN
                     VVPRVQGMSHEESDDEEVDPTYASVNRQKDGDGAPPILPKAPGLQDKEDKNLFGLSTE
                     VLPPSLEETKGETHDWVSQTASLGLGGLSGNGSEEHIYAAIDPNAKSSKKSLDSGIGG
                     QNSKDDLGASSNTLPSYKSKDDSEESLSLNSDIGSTTNKKTNRFLPRAKYAMQQKGFI
                     IFGASAIILSISAALLYLQDKAKFIAFFTNSPKYITVPVIALAALLAIGPIFFATRQF
                     IDTEEHSIQKKDADKILAEVLEHQPKDKAIKSVRLEYSNGTHSNFVLNAWEAKQGFTN
                     IDEKVITRTNKIKSVINDRPIFTALFTGVVAANIAFPLGLLATHGVNGVQKFYQNPLT
                     NNIGLSLLIGSGILALLIVGFGIHYYRKTNCTNLIYSQEKIDPESVNKKFIEEIKHER
                     TNVLGENHGKDAKRSSLTLEQVVVQSHNCKDVVYSVG"
                     /product="hypothetical protein"
                     /besthit="qcoverage 100 , hcoverage 100 , similarity 100
                     , identity 100 , evalue 0.0 , alnlength 487 , Bacteria ,
                     Proteobacteria , WP_174516898.1 hypothetical protein
                     [Wolbachia endosymbiont of Cardiocondyla obscurior]"
     gene            28272..28343
                     /locus_tag="wEsol_tRNA-06"
     tRNA            28272..28343
                     /locus_tag="wEsol_tRNA-06"
                     /gene="tRNA-Asn"
                     /intronpos="0..0"
                     /program="tRNAscan-SE"
                     /cove_score="77.3"
                     /evidence=predicted
                     /type="Asn"
                     /anticodon=GTT
                     /product="tRNA-Asn (GTT)"
ORIGIN
        1 ttaagaaatt taccaaatga aaaaaaaggc aaaagaaacc ccgtggtggt tggctattta
       61 ccatgtacta aaatatcggc attttttta ttctaaaacg cttgattaag agcgatttag
      121 ctgcttttaa cttgcaacta acctacaccg caagtgttta agaaatttac caagcaggaa
      181 aaaaggcaaa gaaaccctag ggtagctagt attcaaattc tcccttgtca atttgacgtt
      241 ttttgctgtc ttaaacgctt tgtaagcgcg tttcggctta tataggtaaa aacccagaga
      301 tttttaaaga catgcgatgc acgtagtgcg aaaaattaaa catgagacgc caaataccct
      361 aagtttttg tcattaacct gcacagattg cgaagataaa taaatagctt cagtttcatg
      421 ataagggggc tggcggagtt tgtcaaggaa gtttttttgtt tctattgaat gacagttgta
      481 acctacgaaa gaagtctgtt gtaatttgta cgatatcttt ataaaatggt tcgcataagt
      541 tatctagcag tgcaactaaa caaatacaaa aatcaaaacg tcgcggtttt tggtcttggt
      601 aaaactggtt tatccgccat taacgctcta acaaagagtg gtgcaagaat atatgcatgg
      661 gatgatcatg aagagcaaat agcaaataca aaaatgatgt ataaaaaatg taattttatt
      721 catcccaagg aatacaattg gcatgagatc agtgcattgg ttttaagccc tggagtgcca
      781 acagagccac attggatagt aaaacttgca aggagatttg attgcaaaat aaaatcagac
      841 attgagctat ttcttgaagc taaaactacg aaccagaagg taataggcgt cacaggaaca
      901 aatggtaaat caaccactac atcactaata gggcacatat taaaatctgc agggaaaaaa
      961 gtagctattg gtggaaattt aggcgttcct gttttggatc tagaaagaga tgcagaaatt
     1021 tatgtaatcg aactctcctc ttttcaattg gagttgatga atgaaattaa cgtggacatt
     1081 tcagcactgc tcaatattac accagatcac atagataggc atggaagtat ggagaactac
```

1141 atagcaacta aattaaaact gataaacggt agtgaggttg ccgtaatagg atgtgacaat
1201 gagattactg ctgatatatt caacaaattc actggagaca aaattccaat ctcagtaaca
1261 tattccccga tgtcattcca gcgcgtgacg ctggaatcca gaaaagaaaa accattgcca
1321 actactcaga tgacagaggg tggtgcaaga gatctaatat cattggcagg taacaatttg
1381 cttgattata gtgaacagat tactggtata gatcgaaatt atctggatcc cagtgtcagc
1441 tacttggatg acaaaagggg cactgggatc cagaagatct cgttaaaact agagaatcat
1501 agcttatcag taagcgacgt gaaaataaac ctaatatcca acgcagaaaa catagcagcc
1561 acatatgccg tatgtaaggt acttggagta gatagcaaca ctattatcaa tggaatcaag
1621 tccttttcag gactgaggca taggaatgaa ctgcttggca aaataagaaa tgtgtttttc
1681 gtgaacgata gcaaagcaac taatgcaaaa tcgagcgaaa aggcgatctt atcttatgaa
1741 aacataaatt ggatcgctgg tggaagaagc aaaaaaggtg gaatagaatc attaagcaag
1801 cattttacaa gggttagaaa agcttttctt attggagaat caaccgaagc ttttgcaaat
1861 gttatggaga ataaagtaga ttatgttaaa tgttgcaatc tagaagatgc attcagattg
1921 gcttttgaag aggccttaaa tagcgcagaa gaagtaacaa tattactttc tcccgcgtgt
1981 gcttcttttg atcaatggaa aaatttcgag gaacgtggtg aagcattttg tagaatgttt
2041 gaaaatctca ggtacaatca cacatgctgt ttagtatgat gttgcgtgga acaaaagtta
2101 ataatagata agctaattag ggttattcca tttgaaggga taagcgatga aaccctatta
2161 aaggtgtgca ctgaccttaa tctagctaat agcttttgca aatttcagaa tggaatatat
2221 agcgctttgg agcacatagc agaggactta aatagctcaa tggaagctga attaagaaat
2281 tctaatttag aagatatgaa agtgcgagag cgagtaaagt tagctattca aatacacctt
2341 tcaaactatg ctaagctacc gaattataga gaacttttaa aaaatgtttt atcattctcc
2401 gtatcaccaa aaaatacata tttttctagt aaacttttat acagaactat tagtgcgatt
2461 tggtatggca ttcatgacca atcaacagat tttaactact atacaaaaag agcaatatta
2521 gctggagtgt acttaagtac gatacttttt tttatcaatg attattcaga agattttgtg
2581 gatactttat cgtttcttga taaccgtatc aacaatgtca tgacatttca aaaattcaaa
2641 actcgcttaa aaggaatcat aggaaatttc ttataagcta ttgattgttg gtagaaaagt
2701 acttgagcac gatcctgaaa aggtttgtga aaataaaaat attcatgctg atcataaaga
2761 acatttagtt tatcgtgagg ataataatgg tacaacaaaa tacaccagtt acgaagaaaa
2821 agaaaataaa tttattgaag aagcacatga atatttaata gactctgaat ttaataaaaa
2881 ttttgatcta gggcagtaca gaataagtaa tttctccgga aacttgacga aaatagaact
2941 ggaactcatc aatgaaaagt atatgcaagt tcaagttaat agagaaagaa aaattattct
3001 cgctataccg taggtgcgtt ccaagattat actgaagttt ttattgcact caaaatcttc
3061 gtttttccta atctctagaa ttatcaatgc atcatcactt aaccagcctg agtgagctag
3121 tccattcaac gttgattcaa ctagattgct attgtaaggt gggtctataa aaactatgtc
3181 acattttgaa ataggttgtg gtaagcagtt agcactgcag caaattagcg taacattgtc
3241 cgtaattcca aaatcttctg ctgttttttt aggcaattgc aaattgtaat aatctgaatc
3301 taccataaat gcatgtttag cgccgcgaga aagcgcttca aatgaaaaag aaccactacc
3361 acagaataaa tcaagtacat tcaagttata aataggtttt cttgaggaaa gcatattaaa
3421 tattgcttct cggacaatac tcatagttgg ccgtgcagct aaatgcttac ctgtagttat
3481 ttttcttccg cgatatttgc ctgcaatgat gcgtagcata ttaaataagc tccaaaataa
3541 ttaattattt atactatatg attaatgtca atatggcttt agcagtttaa tagagagaag
3601 ctatcgattc gcttgtaaga aaaacacaaa cacaagactc atcagttatc tttttctgat
3661 ttgcctgctg cattaattgc ttgaccaatc tcatcttgta aatcatcatg atcatttttt
3721 tcatcatgat ttagcccttc aattctgctt tctaactcta acttaattat agtattgatt
3781 ttatctatat tatcaagaaa gcctctacat tcttcttcac tatactcttc accagatgct
3841 tttttttcaa taaataggtt tttaaatcct gctagatcag aattctgatc ggtatttagt
3901 acaacatttc tctctatgaa gctaagatct gacagcagta agctaacctc taacagatct
3961 tcacttttca cgcttttaag gtcttcaaga ctttctaaaa tactaaacac cttaatcctt
4021 tcttgtacaa tctctttgtc ttccttatca aattcagaca ataaactatg attatctaca
4081 aatttctcac tcaaagcttt attcaattta gcaaaattga catccttgtc aagtgatgtg
4141 ttatcccgca acaagtctac ccttgcccta cgcgcattta aaatgtcgtc gtagtcattt
4201 aactttatgt tgccatcttt gtcttgctgc aatttataat aatcaaggcc tttgaaattt
4261 ttattagtct tcatcttcaa tcctcttctc atctcctttt gcatctggtt tcctcattgg
4321 ttccggtaat ttccttttac tgtcttctcg atttcttttt tcctctcttt cagcacgttt
4381 ttctacagct tctggagtgt tcatttcaaa aagtggtgag aggttataaa ctttatcttg
4441 atttcttctg ccttctcttt cagcacgttt ttccatacct tcaaaagcag atccaaaatc
4501 atcaatttca tctgaacctt ttggttccgt gcctaaattt cgtgtcactt catttgattg
4561 atcaggctta tcttcttctg ttggctgatc atgctgtagt tgattaggct tgtcttcttg
4621 ttctgtgctt agttctcgta ctaccttatc tggctgacct ccccctcgta gtcgaagagg
4681 gtcgccttct tctagtggct gatcatggtt atctcttact agttccgtgc ctaactctcg

4741 ttctacctta tttgattgat caggattgtc tttttctggt tctctcttac ggcctatggt
4801 gtcatcatga agcttattca agaaatcttg cctatgcgat tttgctctaa aaagtaagtt
4861 atttggataa ccatcttttt ctcctgtacg acggctaact cctatagaat caaggccatg
4921 ctcccaagta aacttcatag catcttctat tgtatagccc aaatgtgatc ctacgtatcc
4981 tttataataa tctaaatatt taccgatcct tctgtgtgta atatcttcct tatcaactcc
5041 aaatgccctg tagatctttt cctcaagttt atcaacgtca tttttaggtg atgttgcaaa
5101 ttttgttgct tcaatagtag cacctacaaa tttacgtggc acatcaatag cgtatttcag
5161 cactcctcca actcttggta ctctccccag cagattagct gcacctccgg ctaatctatc
5221 cggtaaatga gtagcccaac tgcctaaaga ctcaagtcct ctaccagcaa aattttgcca
5281 ctgctgtttg aattcttcca ttatgccagt caaaggcgat gcctgataca tactagatat
5341 attgtaaacg ctgaatccac caccagcgat acttgagccc atttgttgta cgaactcgcg
5401 taaagagaac attaaaaatg aaagcaggaa taatacaaag atttcccctg cattgattaa
5461 ataacctctc tgtatgtctt gcaccctttg gtaatcacta ttttcttgat agtttctatt
5521 taccttataa aaattctgtg attggcagct cccatcttgt ctaattatat cttgcactaa
5581 agactttatt tcactttttta aacttggctg tagaattact ttttctatga tttccttgta
5641 attagtacac ctgctttcat ttcctggctg cttacaatag atttctgtct ttttacttat
5701 attattaatc aataataaga tatcagcttt ttctgatgca tgggataaac tatttgttaa
5761 acgcactaaa tcttttacac tacagctatt atctactacc atatcacttt ctgcaatata
5821 atggtctgct ggattctctt tagtaccagg aattgggtta aagaaaggat aatctatata
5881 acgaaaccct ttttcaatgt aatccggtgg aagagggata tacccagcac cgccagtaaa
5941 ttttattgta ccaccttctc tgcttatctt ttctatacct tctatgttct tatctacgtt
6001 cagcggagaa gcttctccaa gagtgtaagg tacgaaaatt tgccctgggg tccaaccaaa
6061 gtactgctta tcaacgatac aaaaaccacc aaacacccat tttggagcac ataactttac
6121 ctcgaaccat ttattgtagc atacagtaaa acctaactgc ctataaaagg tattcattat
6181 aatggaagcc agtaatgaca agagagcaaa tatcataatc gactgtaaac aaaagctgat
6241 gcagaatttt atccaacctt caaatagact tttcagcggt gaaaataaaa tagaaattag
6301 aaacagtggc ataatggcta taagaaaagc tatacccata aaaccagaga gaaatattat
6361 gtaagcatat aagcacagca agaagtatag cacaatccct atgaaaattg ccggtatcat
6421 aagaaggctt gcccacattt ggtaatgtaa aaacgctgca aactttttcc aaacagaata
6481 agcaaaaaac ttattgaaca tgtcctccat gaagctaaac aacttagctt catctccttg
6541 aacaatactt ccggtgttca tgttgggagc aaaattagta attatactta tcaattgttc
6601 aagaccttta acaaataaaa caagaaaatg atcatagaaa aatctaaaac ttccaggaga
6661 tataagcact attactagag tgattttcat cattctgatg agcatatcat gcttagtttc
6721 ttgtataatg ccaaaaagat aaagaagcga tgaaatcatt acaaatagaa caagcaagga
6781 aagaacaaaa ttgtgaaagc tcgtactttt ttcagctata ttttcgaaca tagcttgtgc
6841 aactgcagga tcattgctat taaaatgttc tttgcaattt tcgttaatca ataatacaca
6901 ttttagatag ttataaatat aatcaaaaaa ctcaacccca ctcggttgcc gcactccact
6961 taagaactca aaagagtagc cacctacatt atctaagtaa tatctatcta atatttttac
7021 atatattttc catccctttt ttaatttcac cgggtcacaa tttctgtcct ttaatttagt
7081 gctatctctg tctaaagcaa aaagaccgct cccattctct gccacggagt tgtaacctag
7141 atgtacagta ggagatgaag gattgcgcat agatttcaaa tctttgttag gactatcgtc
7201 accaggcctt tgaaacaaaa gataagcacc atgaccgttg gtaaaccaac atggagcacc
7261 acgtgatact gtatttgcgc tatcatcttc attaatgcac ttgcacttta cagtgcaact
7321 tgcattagac ttacttgaaa ttttctgtgg cgcactaggt ttgtaatcat tacaaactaa
7381 gtgaaagtca ggtagactat tatcacctga cttgccttca aatttagttg catattttaa
7441 ctgctccaag ctttgtaagg tataactgcc tttttagat tccttcttgt tattattact
7501 ccaagatgtc catgcaccat tcactcgcac ttttaaacta tctccattgg tttcgagccc
7561 ggtatcctcc cactccacca cttggttatg gtgaaaacca taattaattt caccagtttc
7621 aggatcaaca acttctccat ctcctggaat aaacgcatcg tggtcgcttg aaaaatgagc
7681 gcttactgca ataggtttag gaccaaaata gtcagcagat atacaccttg gaaaaggcat
7741 atcgttttta ctacaacctg tgatcagcac ataagctgca agaagtaata ccctaaacca
7801 acatttgcct aaacgtgatt gcatatctaa actttttcca aatacagaat aaatttctaa
7861 aactttactt aaaatatctt tttaattagt tactccataa tatcatttat atcttactcc
7921 tctccatttt cttccttgtt tttagcttta gcactaggat cacttcttat tgtttcatca
7981 aaattttttt gctgatctga ttgttttaat gttttaggtc tatcactcgt tgttttatca
8041 gaacttcctt tagaatcttc ttttttgtct ggttgctttg gtgtgttagg taatttagcc
8101 tcaatttttc cccgatcttt actcattttc tgcttgatac tctgtatcat actttgggtc
8161 ttatcatcaa ggccaacggt tgacagcata gcttgcgatg cgctcttagc aactgtacca
8221 acaccgccag taactccata accagagcta aatagtgttt gtaccatagt ttccgatata
8281 gaaacaaaag cttccattgc accggcaata atgaggtata taaacgcctg gattaggtct

8341 ataggtaatg ctgcaaagtg tccaccagcc ctttctccgg tactgagtgc aacatcaacg
8401 ctagtgccag gactgtatcc aagaggcagt attgatttca tcaagcacag atcatacgat
8461 aagaaattta cgctaattaa acattgatag catgcagaaa aatttgtgag gttatataaa
8521 actgaataca tcaactgatt taaaagagat aaaaatgaaa ataaaatgac tggttctaat
8581 gaaacatgag ccaacgtttt tatccaatta tcaaacaagg gtttagtctt ttgaaataga
8641 ataaatacga taaataaagg tgttaatgac agtaaaaacg ctactagaac agtagatatt
8701 acatatttca atgtagcact aataatgcat tttaaaaacg cataagtagc gcagagtatt
8761 gccaaaaatg caataacgcc aaaaggacca gaaagcatca acgataaaaa ctttagccaa
8821 gtttctgctg tgaataatac tcctgctgtc aagtctaaga atgcaaattt tctctcccct
8881 tctcctacat agccagaaaa actatcaact aaataagtgc taccatctat aaaaagtctg
8941 gataaagttg taccaaaaag ttcccagctt ctatcgctaa aggcaaaagc tataaatgct
9001 atcttgaaca ttcttacaat gaaatcaaat ttacttagct gtattgttcc aagcatatag
9061 ccaacgacag taaatataac gtatagagta agcaaagctc ttaccccttg aagaagacct
9121 cttgcatatc ctttgtatga atctttaacg ctgtccccaa ttacttcttc ttttataagt
9181 ttaaataccc cgtttacatt cgatgaaata aaatcattaa tttttctttt aacaaataaa
9241 gttactgtgt acttattatt ttcataatat ttgccatttt tatctgtaat gtcatctctc
9301 tctacgttgc ttacattgat gccgaaatat atcttcttag attcttcgtt ctgaaagtgt
9361 tctccgctta ttacatctaa tatggtgtac tcatcttttc cgtttactct ttctctcact
9421 cctaccactt taaaatcatt agttcccaca ccatgcaaat ctttaggtgg ctcatcacct
9481 aaatatacat ataatttttc gccattaata tattcacatg atctagtcac cttaacatgg
9541 taccctcctc tattatagta ataatgacca taatctgcga tagcgagcat cacactacta
9601 ccgggcgtta cctcgtcatt tactggataa tcattgttca attccagaga aaaatcctcg
9661 aatttattgt tatcgccaat ttgcaaacac atattatccc cttttgcacc ctctttcttt
9721 ggaagacatt gaatacсttt agcgatattt ttatcatcag gatcaccaat ttttgcaaca
9781 agagcctcag cccaagattt atcatcttca gtaaaactac ctgctttaac attcccttcc
9841 atttttttcta gataagaata catatcacat ttatttcctt tatcgtactt cttatacctt
9901 atagaggaga cacagttccg ttctcctgct tgacctaaac catagttcca tatacccttc
9961 tcagtatagc atatattttg atggtaacag ttaagctcat aggaggagta ctttttttctc
10021 tcatcaagac tacagttttt tccatcacat aactcgttca atcctatttt agcgcggcgt
10081 aaatcaagta gtgaaccata tacccatggt tcgttgtctt gaatataacc tttattaaaa
10141 tgcaccttat tatcatatgg ggtataccca ttgccaacta gcactttgct ttcctttgct
10201 tttgttttat caagaggtaa aaattgtatt ttatttttttg taccatcttt accatcacaa
10261 aaaaactctc ccccctgatg tacttcactc actgtagctt tcttatatac agtttcctta
10321 cggtcatttt ctacagcttt ataacaattg tcatcaaaat tgatgccttt cccttctgga
10381 ttgtcgtaat caattgttat ttttctggga actaagctga agcttaactt gtcaccagga
10441 tttactttaa ttccagtgtc cacataacgt ctattgctgc caaatccttt gccaccacat
10501 agttcgtttt tactttcatt aacaccataa ttgttaggta cactattaat atccgcagcc
10561 ttactataat ttggttctga gccatcagca caaaagctag ctggcacaag tactttcctt
10621 ggatttttgt cttctttaaa agggcaaaaa tttactgatc catcgagggt gaatttgatt
10681 ttctcaccct gactaattac ctgaccagaa tcaacccaat gaattttatc ggctttacga
10741 actggaacat ccacattaac gctggtgttt ttgctctgca acccaggctc gacacaatcc
10801 atattacaac cagtgattgc aagacacagt atcaataata gtgatctttt acttatcatt
10861 gctgtcttcc tggttgtggt atctgaggcg ttgacctttg agtcccttgt ggtatttgag
10921 gtcttgacgt acctgactgt tgagaggcat ctcctctttc tcttctttga acgcttcgtt
10981 catctaaccc tactgttccc attaagcttt gttgatactg tcttccaggt tcgcttgcta
11041 cataagcacc aaacaacgag tcagatattg ccgaagaagc ttcaactaaa gctttcatag
11101 cgtgtcccaa aataacaaaa gccatcatag cggtaatatt aggtgtatat ttggaagcat
11161 aaccataaaa aatacaaggg ttaaaaatct ttaggttaat attaagtata catgtagagc
11221 agacctcaaa atcaaatact gaatacacaa tataatccat aacctgactt atcagtgata
11281 taaaaattaa aaggaccact ggatgaattg caaatcttgc taaatttttta acccagtttt
11341 gaaacatctg cctagtatac gcaaataaaa gacaaataat aaaaataggt gctaaagaaa
11401 gtaataacgc aactattgct atagatgtaa taaaagaaaa taaggcatta aaaatagata
11461 agctcactgt gattaaaccc caaatcacca agcaaaaaga tacaatgccc aaagggccag
11521 aaaatataag agatactatt aacaatactg aatgcgatga caagaaccta ttgagcggca
11581 aatcaagaaa ctcaaaaaca tttgaagtag tgcctctaaa atttgctatt tctattagtt
11641 gttttggagt gttaataaat atagagaatg cgttgttata aaaaaagtcc cagctattat
11701 cctgcaatag ctgagcgatg attcctatct ttacgcatat aattaaaaac tcatatatag
11761 aaatatgagt taatccaaaa aagtaataaa gagtatataa gattatatat agtactaata
11821 gtgatattat agtagatcta atggtatttg ttctgcttga ttttataaaa ctttcgtaaa
11881 gagactttac aggactagta tcagagtgaa ttgcgttagg gtctttataa ttagagccga

11941 aaaatgcagt tttcactttt tcatcaaaga aattatatat tgcactaaaa gtttttgtag
12001 gtggctcttg agttgttagg ttaatgctaa actgaccttc atttttgtaa tcgcagccat
12061 ggtctcttat tccgtaatat atggtgcctg ttttgtcttt taattttcct ttcaagtttt
12121 ccatgtcatg aactttgctg atatctacag atatatccgc ttgattttca ccaggcttat
12181 gctcagggaa tttatcggaa acacttatat acaagctatt ttccagacta tggttcttcg
12241 ttactcttat gttgaacccg cctttgtcat ttgtattgta gcagctcttg tcatttctgc
12301 tacatagact aaaagttaaa cttggtattt gatggtcatt ctccatctta tgatcacaac
12361 tagtacataa accaccacct ggaatgtgtt caagagtttc attatccata ccaactttta
12421 gaaatgtaat gtcagttttt acctgttttc cctttaatac gttactaata atttcagcaa
12481 caacagattc accactagaa tttaaagcat tgaatagctc aatttctata ttattatatt
12541 caatatacac gcaatcctcg cctctacgac cagaagaaaa tttacatata cgtctacaga
12601 gtgaatttaa aatgtgcgta tctatcttac ttgcactgct ctctgaaagc ttgctgcaat
12661 ctatattctc tccttgtttt atagagttaa taatattttc aatttccttt tttctttcat
12721 cttgatctaa cttcgaaaac cagtactctc ttccactcaa ccattgcgca tcgcctatta
12781 tatatttatt aggacatatc ccatccttgc caccttggca tttctcttga tttaaaacat
12841 gtgcaaaatg ttccgtttct tgctcgctac acttttcgtc gattttcaaa tacctgattt
12901 ttgtctcact ctctttatct ttacacactt tacttccaat cacactaaag cttatcgtat
12961 cgcctttttt aagtgcaaat ggtaatgcaa cgtcatttct cccaggccca attgcaaaat
13021 ctgcatactg cttaggacaa aaattaacct tactaggcac tatatcaatc tctgttactc
13081 ttattctatc tgagatatta acaccagaat caacccattt ctgctctgtt gacactatat
13141 ctagtttttc acgcaaacct gataaatttt ctggcttaat acagtgatgt tccccgcagc
13201 ctgacagaaa aaaaaacggt attatcagca aaaattttag cataaatcca actcattcag
13261 tgcttttgtt aacactctcg gagctactag aaccagaacc tcgagattta ctcttgtctc
13321 caccactcaa cttcttagca gcagcaccag caactccagc aacaccccca gcagcagctt
13381 tggcagcaga taaagctttg cccaccattt gtccaggtgt ctgacctgag ccaagtgctg
13441 ctctatggtt accagcaagt tcagcagcca tgccaggaag aatgtttaaa aagtggtaaa
13501 agaggaataa tactaaacac aattttaata attctccgat gagagagctg ccaaactcca
13561 tgtatgtaaa gtcgaataga ccaagtatgc tgctcttttt aaagctataa ttttgcatca
13621 tacatgcaag agtagtttca tttttatcac attccccatc tttcaattta aaccactgtt
13681 ttttcttact atatgagatt tctgcttgtt tcttttcata tgacttgtcc ggttgttcat
13741 ttttatatga ctcgtccagt tcaaaattta aatttttaaa atagatttta tcacatgcta
13801 tgaacataaa ggacaaaaac gcaaaaagga taaccgggta taagctatag gtaatcaact
13861 ctttcaccca gccttcaaaa tatcctttgg tgtgttggaa taaaaccata ggaatgaaca
13921 aaggtgataa aatgatgatt acacttaaag caactagaga taaaataaat acatagcaca
13981 gccacaaaat aaccatcatc atcagaacgg ccataaatat acagacaaga gctactaaaa
14041 tttgtccacc agcaaaaata ataccgatta ccgaacctgc aactaataag acaggagcag
14101 caccaagcaa gactgctaac gttgcaacac ctccagtacc aattttgcca ccaattccat
14161 ctaaaggggc acctaaataa aataaaattc tacaatcaag cctatcccaa ggtgcaagat
14221 aactgtaaga taccctcttt cctgcacgag tatattcata atctgtacct gctttataat
14281 tacatatgcc tttactttca gatgatgctt ttagcactat ctctgacaaa ccatttgaga
14341 gacgagttaa ttctccataa taatgagaaa tggtactgcc tgttgtgaag taaatcacta
14401 aagcgaattt tatgatcaac atatacattt cttgaggcct ctgaactcca ccagacatgg
14461 cttttataga gaacagtatt aaagctaaaa ctaaaacagc agttacagtg tttttcagcc
14521 ttttctgtgc tacagatata aaactctcac catttggtgc acctttacct gcaactaaat
14581 tatctaatga ttccttaatg cattgcacta tcatagaagt tacgggaata ggagctaatg
14641 atttactccc tgcctggtta taacaagctt cagaaacata cgagctaaaa cagccagcat
14701 tatcataacc tacaaaaatt ttcccccctt gtccgttgag tccttttttct ttagatctga
14761 taatagtttt atagtcatca ttattaccaa tagtaacttt ttcctctcct gtaccatctt
14821 tatttttgaa tatcatcaca gatttttcac acataggagc aagtgggtca ggtggcaatt
14881 ccgtgcaccc catatcaacc ccttgcggcc aaggagtcat ttcgatacca agcatgctaa
14941 cactggctaa ctccacacat aatctgcccg tttttctcac tgctttgaat gtagctccat
15001 gtatgggttc gctctgtcct gctttgagaa ctctacattc cagaccacca tcaaccttcg
15061 aggaccatga cccatctacg tatccagtgt ctttctccct gacatcaaag tctatgtatc
15121 caggttttcc tctggaatta aagtctacct tattaaaaag attaccaaaa gttaaagggt
15181 ttcgatgaca tacttctata tattcactgt attgcacacc attttttggc cagtaataac
15241 tctctgtact ctcctgaacg ttagaattta aggcttcttt ttctgctctt ttcccgtcat
15301 aatctttttt gttcacgtct tcccgagctt tattttgcaa acaagaaaaa tattcctctt
15361 ctgtttggta attttcattg gtgcagccac tataacctgt gtcttctaat ttaaaatttt
15421 tgtattttcc atcatggtca cgtgcaactg ggtgtttaac aaaactatgt tcacaaacaa
15481 caagtcctcc tatagctttc cagactccaa caattgcagc tatcattcca acaataacaa

15541 gagcggtaaa caaaccagca gaagaaacga tcaaaacaat gccagtaaat attgcgccaa
15601 cagcaatcgc tattccagca agtgctgcag ctgttttaaa cgctccacaa tcaggattag
15661 aggcacgact aaaactgcca gtggaattaa atctgctaac aaattctgtc ttgtcagtac
15721 cacttactcc atctgcaagc actggatatg agagtaaaaa gtctatattt agtaaaaata
15781 tcgcaattaa tagcaatgac agcttagtct taaacatttt ttaccttctg ataaaatata
15841 ggcaaccata cttttgggtt gtccccaact tcatttaata tatcgtgtaa caatagaaca
15901 ctttctgcac gtccggataa tacgttgatt atatcatcca ggccttttaa gtctattcta
15961 gctactacag cattgactcc ttgtttcacc aaaaagaatc tagtactcgg atctgtgtgc
16021 ttaattaata tatattcacg ttcagttaac ataaaaacat cccgatagac actagtagct
16081 ttcagatttg gtaaaaaaat ctgtgtcgct gtttgttgta caagcgtatc gctaatagta
16141 cttttacttg catcctcaac actctgagta gcaaaaatca caaaagcatt tagttttctc
16201 agtaccttca accagtcttt tatcttgggt gcaaaaactg gattatctat taacgcccat
16261 gcctcatcaa gaacgataat agatggagtg ccatcaagag atatactaat cctatgaaat
16321 aaataaatca agaccggtcc aagagcaaca ggatctttga gcaagctagc catctcaaaa
16381 ccaaatactc ttgctcgtga aaaatctagc aaatcttctt tattgtcaaa tattgcagca
16441 tgagaaccat tatcatgcca catagatatt gccccagcta gagtatcagg acctgcaagc
16501 cctaaaaacg gtacgaggtt tctcaagaat ctgtcttctt ttcttaattt aaaatttcct
16561 tcaattgcat cattaattcg tgcaatatct tctgaagtaa atttatcatt atataccgaa
16621 attaaagact ttatccactc cattaaaaat gttctattat cagaggtatc atcaagttgt
16681 aaaggattaa aatttgtttt agttcttggt tctattacag tatagatacc actaagtgct
16741 cttaagaaaa tctctgcacc gcgatcttta tcaaaaaaga atatccttgg agaaaacttc
16801 atcgcttggg cacacaggaa attcattaaa acagttttac cagcaccagt tgggccaatt
16861 atcatagtgt gtccaacatc ccttatatga aagttgaaaa aaaatggagt accagatgtc
16921 gtgtcaaaaa ctgtaacggc atctccccag tggttattga atttattacc agtaggataa
16981 ttatgttgag atgcaaatcc agccaaatta agactactta ttgtagcttt tctcactata
17041 taatcgaaat taccaggaat ttgcgcccaa aatgctggtt caaggttaac tctctcacgt
17101 atagggtaaa caccgcaatt agaaagctct gattctacca acgacagagc gttatccaat
17161 gatttaggac tttttctat acataaaata gtcaggtgat gttgaccaaa cgcaatctta
17221 ccactcattg cgtcatcaag cgcttgagaa atttctgcta tttgagaaat agctttatct
17281 gcagactgta tcattcgatt ttgctgtatc tgcatttttc caatcgccat ttgcctgttt
17341 ataaattgaa aagattgcgt aattataaac tcgtaaggaa gttgtaaaaa cgaatcaagc
17401 attcctgcag aagtatgatt tccatattct ttgatactaa ttattccagc atatttactc
17461 tcattatgag tcacaacctg tatcatctta cggccaaaga ataacctatg tactggtaag
17521 tatcttgata tttcagttct tagcggaaaa agcgtatttg taacgaagcc acaatttacg
17581 attctagata gaaactccat tatttcgcag aacaaaccat ctggagtttc ttttactcca
17641 agaattttag gcgaataatt tctaaggctt gtcactacac gatttgtcgt ttcctctaaa
17701 tcttcatacg tggcacgtat atcactttcc caagcatgtt ttgaagttac atgtccgaat
17761 ttttttagta gatgtgacaa aaattctact ccttttgtgt ccgctctacg gataatcgta
17821 atgtataggt cattaataaa tgattgcctt gttgcgtgtt tttctctcca ttttagattt
17881 acgtgattag cgaaaaaatt cggcaaacct tgacttgcaa attcatcaga aaaaatattt
17941 ttcttacgtc tgatagtatg aaaatacaaa ctaaatgccg gagatgaaat gcttcttagc
18001 atctgattcc tgatgttgtt ctgtatgact aaatcctcgt catcggcagt ttcaaatgca
18061 aaaccactta attttataac cttaaccaac caattttctt ttgtcatcaa ggttgtgcta
18121 ttccaatagc aagaataagg aataaattca gcagcgtgaa cttccctgtt cagagtagat
18181 ttattctttg attgaatagc cctaaacctc agcatcacac aacgttaagc aatatcataa
18241 gaattggctc catggtaaaa acgatttaaa catttcgagc actttcccaa ttttaccata
18301 aataattcaa taaacaacgg ttcttttgca gaagctatat aaccaagtcc atgtatacct
18361 ggtaacatta agagaatcac tcttaaatca tttgaattaa tgaaaattag catgcaaatc
18421 aggacgttta atattgcaaa catataactc acaccaaaga gcattgcagg tcttgtaaga
18481 cctttaaata attgatctgt ttgtatacta cctgtggaca tatctttaac cttctaataa
18541 tagctctaac actaatacta tatcattata ttaaaaatcc attgaaacta gtagcacttt
18601 aagttaagat acattattta atcccgataa tgacaagttg gccatttcaa gaagcagaaa
18661 aaatattgca agagtttcct aataaaaaag aaataatatt tgaaactggt tatggacccct
18721 ctggtttacc ccacatagga acctttggag aggttttccg cactacagtt attgtaaatg
18781 cattaaaaaa aatagctcct ggtataaaaa ccaaaatcat tgcagtttca gatgatatgg
18841 atggtttgcg aaaaatacca gataatgtgc caaatcaaga gatgctaaga gagcacttaa
18901 ataaaccatt gactatgata cctgatccat ttggcactca tcagagttat ggtcatcata
18961 tgaattcatt gttgtgtaaa tttcttgata tgtttgaatt tgaatacgag ttcaggagtg
19021 caacagagtg ttataaatct ggcgtttacg atgaaaaact tttactctta ctgaaaaatt
19081 atgataaagt gatggacgta atgcttccat catttcggga agaaagacag cagacttata

19141 gtccattctt gccaatatgc ccaaaaactt ctcaagtttt acaagttcca gtaattgaaa
19201 caaatacaga gaaaggaaca atcacatatg aagacccaaa cggggaaaaa atagaagttc
19261 cagtgacaaa aggaaaatgt aaactgcaat ggaaacctga ttggggaatg aggtgggccg
19321 catttggagt aaattacgaa gctcacggaa aagatttaac tccatctgct gtgctttcaa
19381 gtcaaatatg tgaaatactt ggagaaaagc caccacttct gttttgctac gagcttttcc
19441 ttgataaaga aagaaagaaa atatcaaaat caaaaggaaa tggcatttca attgaagagt
19501 ggctaactta cgcaccaaca gagagcttag cattgtacat ttttcagagt cctaaaaaag
19561 ctaaacgttt atattttgat gtgataccaa aatcaactga tgaatattta gaatttgtaa
19621 agcgttatca tgagaacaaa gaaaaggata taaataatcc tgcttggcac attcaccagg
19681 gcaatgttcc cagtatagaa acttcaggca taaatttctc actactttta aacctagcag
19741 cagcttgtaa tgctgaaaac aaagagatcc tatggggttt tatatcctct tatgcaccaa
19801 acgttacacc agaaaataat aaaatgcttg atagactttc agacttcgca gtaaagtatt
19861 atcacgattt tatcaagcca acaaaatcat ataaaactcc aaatgaaaaa gaaaaagcag
19921 cgttactaga tttgaaggac actttacatt ctctatctac aactgctact gctgaagaaa
19981 ttcaatctca ggtattttct attggaaaaa aatatgatta caccaactta cgtgactggt
20041 ttcagctatt atatgaaacg ttacttggcc aaaaaaccgg tccaagaatg ggatctttca
20101 taaagcttta cggaataggt aatacgattt ctctcataga aagcgctact aaatgtatac
20161 tttagagttt acctcctttc atttgagctc tttagtttta ataagaatcc cttgacaaac
20221 tccgctagcc cccttatcat gaaactgaag ctattgtatt tgctttcgcc aatctgcaga
20281 ttaaaaggta aggattactt aatgtatcgg cgtcttatgt tcaatttttt gcagtatata
20341 gatactgtat gtctttacaa aacttcatct acatctagat ttttatctaa ataagctgaa
20401 cgcgcttata aagcgttaca agacatcaaa aaatgccaat actcgacaga gatagtaaaa
20461 gactagctaa ctcggggatt ctttgtcttt ttttctgctt agtaaatttc ttaaacattt
20521 gcagcgtaag ttagttgcaa ttaaaagcgg ctgaatctcg cttgtcaagc gtttaaaaca
20581 taaaaaacgc caatatttta aagcggataa taaccccagg gcttcttttg cctttttttt
20641 cacttggtaa atttcttaaa tatttgtggc taaacgacaa ccgtcatccc gctacttgtt
20701 agcggctgag ataccgcgaa tgaatcgcgg tatgacggtt cgcggcggca tgacgataag
20761 gtttatacct tgtcatccca gtgcttgaca ctgggatcca gctttcttta taaattcacc
20821 aaaagtgctt cattctataa cgcaaaaccc atactcacca aatccaatgc attacttgca
20881 attaagtttc ctggatccca gtgtctgggc actgggatga caccatcata aaggaatcag
20941 tgtcagctac tttcatgaca ccattctttt tcctggatcc aagtagtcga gctacttgga
21001 tgacagctag cctgacaacc gtcatcccgc tacctgttag cgggatctat gctaagagat
21061 accgcgaatg aatcgcggta tgacggtgta gacgatggtt aagtatcccg ctgcttgtta
21121 gcgccgcggc ggtatgacgg ttcgtggtgg tatgacgtag agtgcttaga agctgctatt
21181 ttgctacaaa tggctgtaat ggcatcatct attttattgc tatcacagcc tgtttgtgca
21241 agttcagcgt tcccaccaca atttttttca actgctattg atactagttc ttttgcacta
21301 attttattag ctaagtcttt acttacttta ataatcaaga ctgttttatc tttttctgtt
21361 gccgtgaaag ctattatcgt ttttggtttt tgttgctgca aaacaaactc tcttattata
21421 ttcgctggaa tattggtaaa agcatgactt acaaaattta ttccatttat ttcagtgctt
21481 tttatgctct ctgcgcttat aagtttttta tataaatttt ttattttagc ttcagattct
21541 ttgcgctctt gatttaaaat atttaatcgg tttattattt cacttgctgg tgcttttacg
21601 aattctgcaa ctttttttaa gttaatttca ttatcacgta cataattgat tgcttcctga
21661 ccagttaaag cttcaattct tctcactcca aaagcaacag aactttctgt tactatctta
21721 aataaaccaa tttctccagt atgttcaacg tgcgtgccac cacacagttc tcttgaatct
21781 ccaatcttca ctactcttac ttggttgcca tatttttcac cgaataacgc catagctcct
21841 tcgtctattg cctgattcat gctctgaatt tttgtagacg tagaaaggtt ttctctgatt
21901 agagagttta ccatatcttc tactaagaat agctgatcct gagtaacttg agtgttatga
21961 ctaaagtcaa atcgtaactt atccggtgca actagggaac ctttttgagt gacatggtca
22021 cccaagattt ttctcaatac aaagtgcaaa aggtgtgtag ctgaatgatt tcttcttaag
22081 gtttgccttc tttccttgtc aatgcttgct gcaactgtgt tacctttaca aattgaaccg
22141 aacttaacta tgcatctgtg caaatatagg tcattaacct tattggtatt ctcgactata
22201 ataatacttc tatcagattt gatcagactg ccagtatctc ccacctgtcc acctgactcg
22261 ccataaaaag gtgttttatc aagtataatg gttatctttt ctccctcctt tgcagaatca
22321 attacttcat ttttagagga aactatagct agtattttcg catcatttac ttcattgaac
22381 tcataaccaa caaattttgt tttgccaaat ttatcgatca aatcaaacca tacttgctca
22441 acagattttt caccagatcc agcccattta gcacgtgctc tctcttttttg ttctcccatt
22501 gcatcatcaa aacccttctg gtcaaaattt atttttttct cttttaaaat atcaagtgta
22561 atatccaaag gaaatccata agtatcatat agcttaaacg ctaattctcc aggcagagtg
22621 tcacctggtt ttaaatctgc agtgaatttt tccaatagac taatgccttt catcaaagtg
22681 tctttaaagt tttcttcctc tgattttaac gtcgtttcta ttaagctttt agctctgact

22741 aattcagctc ccatataagc cgaactcgtg ctatctatca acactggaaa aatacgatgg
22801 agtagggaat cattatatcc aagcaggtga atataacgtg tggctctcct aattaatctg
22861 cgtaatacgt aattcctgcc ctcatttcca ggaagcactc cttctgcgat gagaaatgca
22921 gccgcgcgaa gatgatctgc tacgatctta tgcgctattt tattttctgt tcttccacaa
22981 tactcttgag atttatttat caaagcagaa aataaatcaa tatcatagtt atcatggacg
23041 ttttgcataa cagccgctat tctttcaaga cccattccag tatcgatgca tttttttggc
23101 aatttgtgta aattaccttc ttcatctttg ttaaactcca tgaataccag attccagatt
23161 tcaacaattc tatcgccctc ttgtaaatta ggactcccat ggtcataaaa aatttcagaa
23221 catggaccgc acggaccagt gcttcccata ctccaaaagt tatcatccgt tgtaattctt
23281 atgattttat cacttgaaaa gccacttatc ttacgccaaa tctcgtacgc ctcatcatca
23341 gtatggtaga cggttatgga tagtctgttt ttatcaagag ataactcttt agtgataaat
23401 tcccacgcaa attctatcgc agtttcctta aagtaatcac cgaagctaaa atttccgagc
23461 atttcaaaaa atgtgtgatg ccgagttgta tagccaacat tttccagatc attgtgttta
23521 ccgcctgctc ttaggcactt ttgacttgag acagcacgtt tcatttcagt tttttgagca
23581 ccagtaaaaa tatttttaaa ctgcaccata ccagcatttg tgaacatgag cgttgagtca
23641 tgttctggaa tcaaaggaga agaagaaacc tgctcgtggt cattatttac aaaaaatttt
23701 ataaatcttt ctctgatttc gtttagcttc atcgttttta ttgaggttat ctataaataa
23761 taagttgtaa tggaaataaa atcaatacca ctctctagat ctcagaactt ctagtgacat
23821 gcaccttgtg gtaagaaaaa atcaatatat tcacgataaa tttacctcat atctgtgttt
23881 tatatgtgga aattggaatg gaaatttatg taggcaaaga aaaaagaata gcaaatgtgg
23941 tgactcaaca ctggaatgac ataaaaggat cagacagaga ttggccagaa aggcatgaaa
24001 tagacactgc agaaataatg gagtcatggc agcattgttt tatcattgaa gtcaaagatc
24061 aaggttatat ttgcgaaaat gcaggagaaa aggctgttga attctatggt tttgagaaca
24121 agacgcgtat tgataataag tatgcaattg atgcaccatt tttgcgacta tataaaatag
24181 atgcagtcat tgataaactt gatactgtaa tagagagtaa atgtccaatc aacgaagagg
24241 aagaaagtga aagtgttaaa atgaggcaag tattgttgcc acttgggaat aaagaaggta
24301 taacacatat attgggcgta attactttta aactccttta atatacaatt ttaatttgat
24361 tctttaataa aattatttat actactactt aattactatt aaagaactca atagtagtgc
24421 ttgtaaattt gtttagaata tgaagaaaat aaaattaaaa accaaatcct ctgtcaaaaa
24481 gcgctttcac cttacagcta agggtaaagt catttctact cagtcaggca aaaggcatgg
24541 catggtaaag agaagtaaat ctaatattcg taatcagcgc ggtacaacga ttcttggtaa
24601 atctgactcg cgtatagtta agctttatat gccttatggt atttaataaa atggaggtaa
24661 aataaaatgg ctcgggtaaa acgtggagtc actactcacg ctcgtcataa aaaaatattg
24721 aaactggcaa agggttatag agggcgcgca aaaagttgtt atagaattgc attacaaaga
24781 gttgaaaaag cactgcaata tgcttataga gacagaagaa atcgtaaacg tgattttcgt
24841 ggtttatgga taatacgtat taatgcagca gtaagagagc atgggcttac ttatggtagg
24901 tttatgcatg gtcttacact tgccggcatt gatttaaata gaaagattct tgctgagatg
24961 gccgttaatt ataaggatga ttttgccaaa ttagtagaag ctgtaagtgg taaattagca
25021 gagaattctt aaagcaaaga caccatgaga attattttca tggggtcacc agaattcgct
25081 gttagtacgc taaacttact attgaaatca cagaataaaa tagtagcagt atacaccaag
25141 gctccaaaac cttcagggcg tggacagaag ccaacgaaat ctccagtaca tgttatcgct
25201 gaagaaagta acatagaggt atgtactcct atctctctaa agttttcggc agagcaagaa
25261 aaatttagaa atttcaaacc agacgttgca gttgtagctg cgtatggatt gatacttcca
25321 agagaaattt tgaatattcc aaaatatggt tgtattaata ttcatccttc attactacca
25381 aggtggcgtg gtgcagcccc gatacagcac acaattttag caggagatca agaaaccggg
25441 gttagcatta tgcaactgga tgaaggatta gattccggcc ctattttaaa acaggaaaaa
25501 tttcttatcg aaaagaacga taattacaag acattgcatg ataaattgtc tgaattaggc
25561 agtgatttac tgctggaagt gctaaacgaa attgaaaaac agcttctctt aaaacagaac
25621 gataacgatg catgttacgc taacaaagtg gaagactata aaatttatgc aagtgatgcc
25681 tgtgaagttg cttatagaaa ggttaaagcg ttttacccaa aagcattcat taagatagaa
25741 aataaacgta tcaggatact tgatgctgac tttgaagctc tcgcttcaga acaaggtaag
25801 attgttaacg ataatatgca cataagttta aaaggtggca ctttaattcc taaagttgta
25861 caaatggaag ggagaaatcc ttgcagtatt gaagatttta ttcgtggctt gaaatcaagc
25921 atggtaaaaa aatttataga atagactgct ttaaattagt gtagaaatgt aatagctaaa
25981 tttccttttc ttagatataa tcttataaaa ttttctgtac aaaaatgaaa atcaaaagag
26041 ctttaatatc ggtatatgat aaaacgaata taattgatct tgcatcgttt ttaatgcagc
26101 aacaaataga aattctttca acaggaaata cttataaagc actatctgat gccggaataa
26161 aaacgcaaga agtatcagat tatacgcaat ttccagagat actgggtggt agagtaaaaa
26221 ctttacaccc taaaattcat ggaggaatac tttatgatcg agaaaagcac aaaaaggaag
26281 tgcaagatct agaaattgag ccaatagacc tgcttataac taacctatac ccatttgggg

26341 agacagtaag tagcagctca aatgaagagc aaattataga acaaattgat atcggcggag
26401 tggcattaat tagagctgca gcaaaaaact ttcgttttac ttcagttatt tctaggattc
26461 aagactatga agcactgaaa gctgagatga tagaaaataa caataaaaca acattggaat
26521 atagaaaaca cttagcaacc aaagcatttg ctctcactgc acactacgat tctaatattc
26581 acagttggtt tttatcccag agtaaaaata atgagttacc agagtttttt gctctatacg
26641 ggcataaagt acaagaactc aggtatggtg aaaatcccca tcaaaaagct gcattttata
26701 gtaatcaatt tacagaatat ccgttggaaa aactacatgg aaaagagttg agttataata
26761 atatagtaga tatagaatcc gcacttaaca taacttctga attcgaagaa cctgcagcag
26821 tgataatcaa gcataataac ccatgtggcg ctgctattgg taataatgct ttggaggcat
26881 atgaaaaagc tctatcgtgc gatgaagtaa gcagttttgg tggtatagtt gccttaaacc
26941 gggagataga tttaaagcta gcggaaaaat taaacgagat atttttagaa gtagtgatag
27001 caccatcggt aaacaatgag gcactaaaaa ttttacaaag aaagaaaaat ttaagagtga
27061 ttattcataa atcttttcaa caaaatgtga aataccaaac taaaaatgtt gttggtgggt
27121 ttttggtgca agaaaataat gaccacacaa taaaagcaga acaagtaaca gaatgcactg
27181 caacagacaa agaaaaaaaa gatcttattt ttgcctggaa aatatgtaag catgtgaaat
27241 ccaacgcaat agttatagca aaagatggtt gtgctattgg catcggcgca gggcaaacaa
27301 gcagaataga tagtgtgaac attgcagtga aaaaagcagg tgaaaaatgt aaaggtgcgg
27361 tgcttgcttc agatgcattt ttcccatttc cagatagcat agtagaaagc acaaaacatg
27421 gaattacagc tataattcag cccggtggct cgctaaaaga tcaagatgtg ataaaagctg
27481 caaatgaaaa taaaattgct atgtttttca ctggcgttcg cagtttttc cattaggctt
27541 tgtgtcttat cgtcataccg ccacaaaccg tcataccgcc gcggtatctc tcaacataga
27601 tcccgctagc gggatgacaa atataccgcc gcggcgctaa caagagatcc cgctaacaag
27661 cagcgggatg acagcagtcc tacgtcatcc caccgcgaac cgtcataccg ccgcggtatc
27721 tcagccgcta acaagagatc ccgctaacaa gcagcgggat gacgagctta tcgtgctgcc
27781 acgaaccgtc atgcctctgc gaaccgtcat accgcgattc attcgcggta tctcagccgc
27841 taacaagcag cgggatgacg gctgtcgctt agccacaaac gttaaaaaat ttaccaaata
27901 aaaaaaaagg caaagaagcc ccgtggtgcg agttttgacc ctctaatact ttaaattggc
27961 gctgtaataa tgtgctaacg cttaaaataa gcgcgatttg gctgaatgta gaaaaaatta
28021 aaaagacatg cagccgctat aattttatgt aatccgccaa tagatactcc aagtttttta
28081 ctgaattttt gttattgaac ccgcgcggat caaaaacaag ttttgagtca gaaatactct
28141 taatactaca ataagggggc tggcggagtt tgtcaagtaa gtttttttcgt ttctgtgacc
28201 agttggactt aaagtctaca caaaaaacgt ttacaaccaa cgttcaacat ggtaaattaa
28261 aaacggttta ttcctcggta gctcagtggt agagcagttg gctgttaacc aattggtcgc
28321 tggttcgaat ccggcccggg gagcgtttct ctgtgaaatt ctagtgttga ttttatatga
28381 acattaaatc agtggtttta tgtatactag atggctgggg aaatggaata gagaataata
28441 aatacaacgc tattagcaac gcaaatccac cttgttggca atacattagc tctaattatc
28501 caaaatgcag tttatccgcc tgtggaactg atgttgggtt acctggtggt cagataggta
28561 actcagaagt tggccacatg aatattggca gtggtagagt agtaatgcaa agccttcagc
28621 gcattaatca agagattgga acaatagaaa acaatgtaaa tctacaaagt tttattaatg
28681 atctaaaaag taagaatggt atatgccatc tagtgggatt ggtgtcagat ggcggtgttc
28741 attcgcatca aaagcatatt tcaactttag caaataaaat atcacagcat ggaatcaaag
28801 tggtgataca tgcattttta gatggcagag acacattgcc aaattcagga aaaaaatgca
28861 ttcaagaatt tataaagagt ataaagggaa atgacataag aattgctact gtttctgggc
28921 gttactatgc tatggaccgt gataacaggt gggagagaac aattgaagct tatgaggcta
28981 tcgcatttgc aaaggcacct cgtcatgata atgcagtatc gttgattgat gaaaattatc
29041 aaaataacat aactgatgag tttgtcaggc ctgcagtaat aggtgactat caaggtataa
29101 aaccagaaga tggagtgctg ttggctaact ttcgtgctga tcgaatgata caattagcaa
29161 gtattttgct aggcaaaaca gactatattg aggtagcaaa actctcttca attctaagca
29221 tgatgaaata taaagaagat ctcaaaattc ctcatatttt tcctcctact tctttcgctg
29281 atactctagg acaaacaata gcagacaata aattgcaaca attacgtatc gccgaaaccg
29341 agaaatacgc tcatgtgact ttctttttca attgtggaaa agaagaacct ttctctggtg
29401 aagaaagaat actcattccc tcaccaaaag ttcaaactta cgatttgcag cccgaaatgt
29461 cagccttcga gctcacagaa aagcttgtag aaaaaattca ctcccaagaa ttcgcactga
29521 tagttgtaaa ttacgctaat cctgatatgg tagggcatac aggtaatata aaagcagccg
29581 agaaagctgt gctagctgta gatgattgtc ttgcgaaagt gctgagtgct gttaaaaagt
29641 caagcaacac cgcattaatt gtcactgcag accacggtaa tgtggaatgt atgttcgatg
29701 aagaaaataa tacacctcac acagcacaca ctctaaataa agtgccattt attgtgtctt
29761 gcgataatct aaaactaaga gacggaaggt tatctgacat tgcccctact atttacagc
29821 tacttggaat taaaaaacca aacgaaatga caggcagttc gttgatttct tgcattaccc
29881 tctgtcattc cagctaggca gcgttatgca ccggaatgac atcaaataga tgtgttttaa

29941 cttaatccaa cttttaaatt tttcgctgtt agtactaatg attcattaaa gaagctgtca
30001 atatcaccaa tactatctat attttttgta ctgatagatt ggtcaattct tttgaccaaa
30061 ttagataaaa ctttattagg gccaatttca acaaatttgt taatgccacg gcttgacata
30121 tacaaaacca tttctctcca tttcactctg cttacaactt gcttggcgag taaagttttt
30181 ataactttgg gatcactctc ctctttagct gtaacatttg atatcaaagg aactatagga
30241 cgggttattt taataccctt caaaaactcc aaaacttttt catcagcagg tttcataaga
30301 gatgaatgaa aaggcccact aacctgtaat ttaattagtt tcctcacact cgagtttttg
30361 aataaatcgg gtaacatttc aagagcttct ctagtgccac ttataaccac ttgtccgcca
30421 ccgttatcgt ttgcaatttc acaaattccg tcaatttgaa ctgatttcaa tatatcttcc
30481 acttcattta tctctgctcc aagcaacgca accattccgc ctttacattt cagtgaagct
30541 tcatgcattg cttcgctgcg aactttcagc agcttgattg cagactcaag cgttaacgct
30601 cctgcagcac acagcgctgt atattcgcca actgaatgcc cacaaacata tttaacattg
30661 tgatcagtga aaagagattt gccaaacaca tgctccataa cacgtagcat tgcaattgat
30721 actgccatta tagctggctg agcgttttct gtaatggtta attcttcaat aggaccgttg
30781 aagattaaat gagacagctt tccatccaat atgctatcta cttcattaaa tacttgtctt
30841 gcaacagaaa actcactata taagctcctg cccattccta caaactgaga gccctgacca
30901 gggaaagcga aaatcatgat aattattact tattccttat taagtataac attttattta
30961 tttttgtca atattttaga ataatgattg acttagtgaa ttaagttcac tagaatgtta
31021 actgactaaa agtttataaa caacaatgaa ttatcataaa attattatag ttatgacaga
31081 cggtcaagag tttgaaactc gttccactta tggaaaggag ggagataggg taaaacttga
31141 tagagatcct cttactcacc ctgcatggac tggaagtttg acaagcgggt tagcaagtaa
31201 aactagcaaa ttagctaagt ttaacgataa atatggaagc attttctaat tctcctcctc
31261 tttaagtttt agagagttta ctgaagcatg cataaataca aaaatatagg ctatgttgct
31321 tctccatcac caaaatcgca ggaagtatct aaactattaa agaaacttaa ttttatcaat
31381 ataacagaag aaaataagtc cgaaatcgat ctattgatag ttgttggcgg tgatggcttt
31441 atgctacgca ccttgcataa ttacgttata gaaaataaaa acatgcatgt gtatggggta
31501 aataccggca atgttgggtt tttgatgaat aaatgcttta gccgcagtga agatttaatt
31561 gatcatatag aacatgcaac ttcaactcag ttaactttgc taaaaatgga agcaacagac
31621 acaagtggca agaggtatca ctacatagcg gtaaacgagg tatatgtttt tagaaaagca
31681 aaccaaatag tagaaatgaa tatcactatt aatgataagc taaaagtaga aaaatttaga
31741 ggggacggag taatattatc tactcccaca ggtagcactg catataactt ctctgccggt
31801 ggcccaatct tgccgctaaa ttcaaattta cttgcattaa cctctatcaa tagctattac
31861 ccaaggcatt ggaatggagc gttaatatca aacgatacaa tcgtacaaat tgacattaac
31921 gacacaaaaa accgtccagc acttgtagta tcagattaca aagaatttca tgatatatca
31981 cagataaaaa tacaaaaaga ccatgagaac acaatcactt tgcttttga caaagattac
32041 cctctgaatg aaagaatctt tgatagacaa tttctatact aataatgtat ctgttcagat
32101 acatggctaa tgtacaaata ttgaaacaaa aattccagtg cccctgtgat gtcattccag
32161 tgcttgacac tggaatccag cttttgatca aaaatgttgt atagtacaca atcaaatttt
32221 ctggatccca gtgtctgggc actgggatga cacccatttg ttcctatagt tgtcttttct
32281 cgtctacctt attttgccac tctgctaaac agatacatta tctgtaaatt ggtatttact
32341 acatcttaat aactatagcg taatcaatgc agcatagtaa ttaactttgg agtgaatatg
32401 catgggtcag tcgaaaaatc aacagtaaaa ttaacttctt acgaacaaac aggtgacaat
32461 agcaagataa cactaaaaga tccaccatcg cctgaagaag caggaaatgc tatctcaagt
32521 gtactgaatg tagtaccacg tgtgcaggga atgtcacatg aagaatcaga tgatgaggaa
32581 gtggatccta cttatgcatc tgtaaatcga caaaaagatg gtgatggagc accaccgatt
32641 ttgccaaaag ctccaggttt acaagacaaa gaagacaaaa atttgtttgg tcttagtact
32701 gaagtactac ctccttccct agaagaaaca aaaggtgaga ctcatgattg ggtatcacaa
32761 acagcaagtt taggtttagg aggattatct ggtaatggat cagaagaaca tatttatgca
32821 gctatagacc caaatgcaaa atcttcgaaa aagagtttag acagtggaat aggtggtcaa
32881 aactcaaaag atgatttagg tgcgtcttca aatactttac catcgtataa aagcaaagat
32941 gactctgaag agtctttgtc actaaattct gatataggta gcacaacaaa taaaaaaaca
33001 aacagattct tgccaagagc aaagtatgcc atgcagcaga agggttttat aatttttggt
33061 gcaagtgcta ttatactcag tatatctgca gctttattat atttacaaga taaagcaaaa
33121 tttattgcat tttttacaaa cagcccaaaa tatatcactg taccagttat cgcacttgct
33181 gcattgcttg caattggtcc aatatttttt gcaacaaggc aatttataga tactgaagaa
33241 cattccattc aaaaaaaaga tgcggataaa attttagctg aggtgttgga gcaccagcca
33301 aaagataaag ctataaaatc tgtaagactg gaatatagca atggcactca ttcaaatttt
33361 gtacttaatg cttgggaagc taaacaaggc ttcactaaca ttgacgaaaa agtaattact
33421 agaactaata aaataaaatc agtaattaac gacagaccaa tatttactgc attatttact
33481 ggtgtagttg ctgcaaatat agcgtttcct ctagggctac ttgcaacaca tggtgttaat

```
    33541 ggtgtacaaa aattttacca aaaccctttg actaataaca taggattatc attacttata
    33601 ggttctggta tacttgcatt actcattgtg ggttttggca tacattatta tagaaaaaca
    33661 aattgcacta atcttatata ttcgcaggaa aagattgacc ctgaaagcgt taacaagaaa
    33721 ttcattgaag aaataaaaca cgaaagaaca aatgttcttg gggaaaacca cggtaaagac
    33781 gctaaacgca gcagcttaac gcttgagcaa gttgtggttc aatctcataa ttgtaaggat
    33841 gttgtttata gtgtcggtta ataaaatgca tgatatagct agtgcagccc tacgtcatac
    33901 cgccgcagac cgtcataccg cgattcattc gcggtatctc tagatcccgc taacacgtag
    33961 cgggatgacg attgtcaggg tagcacctcc ctcccctgtc atccaagtag ctgac
//
LOCUS       JAQZAU010000052         6041 bp    dna     linear   ENV 17-MAR-2023
DEFINITION  MAG: Wolbachia pipientis isolate wEsol contig_33766, whole genome
            shotgun sequence.
ACCESSION   JAQZAU010000052 JAQZAU010000000
VERSION     JAQZAU010000052.1
DBLINK      BioProject: PRJNA929258
            BioSample: SAMN33142973
KEYWORDS    WGS; ENV; Metagenome Assembled Genome; MAG.
SOURCE      Wolbachia pipientis (insect metagenome)
  ORGANISM  Wolbachia pipientis
            Bacteria; Pseudomonadota; Alphaproteobacteria; Rickettsiales;
            Anaplasmataceae; Wolbachieae; Wolbachia.
REFERENCE   1  (bases 1 to 6041)
  AUTHORS   Fiutek,N., Couger,B.M., Roy,S., de la Torre,J.R. and Connor,E.F.
  TITLE     Genomic assessment of the contribution of the Wolbachia
            endosymbiont of Eurosta solidaginis to gall-induction
  JOURNAL   International Journal Molecular Sciences
REFERENCE   2  (bases 1 to 6041)
  AUTHORS   Fiutek,N., Couger,B.M., Roy,S., de la Torre,J.R. and Connor,E.F.
  TITLE     Direct Submission
  JOURNAL   Submitted (10-FEB-2023) Biology, San Francisco State University,
            1600 Holloway Ave, San Francisco, CA 94132, USA
COMMENT     ##Genome-Assembly-Data-START##
            Assembly Date          :: JUN-2019
            Assembly Method        :: FLYE v. 06-2019
            Genome Representation  :: Full
            Expected Final Version :: Yes
            Genome Coverage        :: 100.0x
            Sequencing Technology  :: Illumina HiSeq; PacBio Sequel
            ##Genome-Assembly-Data-END##
FEATURES             Location/Qualifiers
     source          1..6041
                     /organism="Wolbachia pipientis"
                     /mol_type="genomic DNA"
                     /submitter_seqid="contig_33766"
                     /isolate="wEsol"
                     /isolation_source="galls on Solidago altissima"
                     /host="Eurosta solidaginis MN-2018-EFC"
                     /db_xref="taxon:955"
                     /environmental_sample
                     /country="USA: Cowling Arboretum, Carleton College,
                     Northfield, MN"
                     /lat_lon="44.48 N 93.13 W"
                     /altitude="280 m"
                     /collection_date="2018-11"
                     /metagenome_source="insect metagenome"
                     /note="metagenomic"
     gene            287..2293
                     /locus_tag="wEsol_00510"
     CDS             287..2293
                     /locus_tag="wEsol_00510"
```

/codon_start=1
/transl_table=11
/product="hypothetical protein"
/pfam="Pfam-B_1913 SWIM Pfam-B_8147 DUF3670"
/translation="MTIYGRTWWGEKWLQCFNRIDYDNRLPRGRTCANTGRTFGIKIN
GHIVTAKVHSSGSYPYRGSKPQPYKVKIILNELSSSEQQTIRQIIETSPSILSKLINR
QLPTSLFDKLNDLGIKLFPSNWEEMNANCNCPDWAMPCKHIAAVIYLIAAEIDKNPFM
IFNIHNCNLSALIDDFGNGKLENAQNILKIDDIFKACSKIRIHNQAILNDIDLSTIPN
LFDCISSILTDNPLFFEKNFHNILQIAYKHWQKYPAGKLVYYPASKKELSEEELFIKR
WGNIENWQTFQLFIDDQHYLTQVSNGTTNVFMLSRNVLKDIARFLNDIPNSLLHRLNR
ELRFMHMISQFAHMLMEKSALIPQVLQNKRGEIIIRWIPALFNESVKEIHSKISSICP
PLLIKYQEIAMEPEEQVNVAISLILLGYIEDNFPASLDKHRDKYIFELFFTGRPYKFT
EFSNKEIPQAINLWLSRLYLADKPYKLYLMIKDHHEKFELEVQLSLDDEKSFIKLEKA
LSNQNVKLSILSDLALLSEYIPELERSIDSNSKLSFNLDDFAPLFLGIVPVLRAIGIT
VILPKSLQKIFKPQLSLSLSAKDKIKEDRESFLTLENLLKFDWKVAIGDKKLSVTEFK
KLLKDSRGFVRIVDQYVLLDDKEVEALLKKLDKLPDYLSQAELM"
/besthit="qcoverage 100 , hcoverage 56.8510638297872 ,
similarity 99.6 , identity 98.7 , evalue 0.0 , alnlength
668 , Bacteria , Proteobacteria , WP_015588903.1
MULTISPECIES: DEAD/DEAH box helicase [unclassified
Wolbachia]"

gene 2354..3814
/locus_tag="wEsol_00511"

CDS 2354..3814
/locus_tag="wEsol_00511"
/codon_start=1
/transl_table=11
/product="hypothetical protein"
/pfam="ResIII SNF2_N DEAD Helicase_C"
/translation="MFKKFNTYTPVTTPNNLIAQLRPYQERGFSWLVQNIESGFGSII
ADDMGLGKTIQVIAAILYCKNAGFLNEDKVLIVAPTSILSNWQREIERFAPELKLFIY
HGQNRELASDYDVALTSYGLARRDKKELNKIRWFLLLIDEAQNIKNPNTEQTKAIKAI
EATHRIAMSGTPVENRLLEYWSIFDFINKSYLGTPKQFKTYFATPIEKARDRTCLERF
MKITSPFMLRRLKSDKSIIQDLPDKIENNRYCSLTSEQTALYQKVVDTTMEKIEKSEG
IERKGLIFKLINALKQICNHPSQFGKKKRASIEQSGKMQMLEEILISIGEVAEKSLIF
TQYTEMGEIIARLLEERFESKVPFLHGGLSRKARDTMINDFKNLFQSNILIVSLKAGG
TGLNLTAANHVIHYDLWWNPAVEAQATDRAYRIGQERNVMVYRLLSTGTFEERIDEMI
QSKKELANLTISSGESWITEFNNDQLRDLVNIKNAL"
/cog="HepA COG0553 866 Superfamily II DNA/RNA helicases,
SNF2 family"
/besthit="qcoverage 100 , hcoverage 41.3617021276596 ,
similarity 99.2 , identity 97.7 , evalue 0.0 , alnlength
486 , Bacteria , Proteobacteria , WP_015588903.1
MULTISPECIES: DEAD/DEAH box helicase [unclassified
Wolbachia]"

gene 4592..>6041
/locus_tag="wEsol_00512"

CDS 4592..>6041
/locus_tag="wEsol_00512"
/codon_start=1
/transl_table=11
/product="hypothetical protein"
/pfam="Toprim_4 Toprim_3 Toprim_2 AAA_25"
/translation="MVQDFLTDVKQKNTLNDEYYDFNNTVEPLEQFDVEEIKRQLLLN
IRSCLSYLFPRGTFRGDKFYIGNVQGDKGKSLVVELSGSEAGLWHDFATKEGGDIIDL
WAAVHRKSTRIEFPEVMASIAEWFGHFKKLKTSGNPTAYWDYYDENGQAIVRVYRYDD
DSGKRYLPFDVKKSTFTAPEIRPLYNIPGILKSDKVILVEGEKCAEALIKQGITATTT
MSGANADVKKTDWSQLKGKHIIIWPDNDEAGAKYAKNVGNKLLEIGVASLAVLEIPRG
KPEKWDAADCVEEGIDVKEFLASTPLLTIDTPATDTSNMKSLERSTKQPLNILDWSAE
RFVGPVPEQKFLVEGLFPLGVTSIIAAMGDTGKGMLLLDLALKVASDMDQLCGFGPLV

TEHGSVVIFSAEDDASEIHRRLERLDPKCERLKHKDKLFIVPLPNVRGSLTILRNVRG
KIVEVSPEFESVMKQLEEIKDLKLIVFDPLASF"
/besthit="qcoverage 100 , hcoverage 66.4374140302613 ,
similarity 100 , identity 100 , evalue 0.0 , alnlength
483 , Bacteria , Proteobacteria , WP_174516735.1 AAA
family ATPase [Wolbachia endosymbiont of Cardiocondyla
obscurior]"

ORIGIN

```
        1 aaaaactact caataattgg gtaacttctg atgacatttt cactgtacat ttatcgcaca
       61 acattagtgc tacaatcaca ggtgtaaagg tattaaacaa cctttaaaaa gtttcacttt
      121 tcccttaatc aatttttcct ttgatataag tgtttattgc ttttgccctc tctgttgtta
      181 taattaacat tttgtagtag ttcaagatga gttttgcata ttcagagaaa atcatccata
      241 gaatcagcat ctagcttatc taggttaaaa tatatagttt tacaagatga ctatttatgg
      301 aagaacatgg tggggagaaa aatggcttca gtgcttcaat agaattgatt atgataaccg
      361 tcttccacgt ggcagaactt gtgctaatac tggtcgaact tttggtatta aaattaatgg
      421 tcacatagtt acagctaaag ttcatagctc aggatcttac ccatatcgtg gttcaaaacc
      481 tcaaccatat aaagtcaaaa ttatactcaa tgaattaagc tcatcagaac aacagaccat
      541 tcgtcaaatt attgaaactt caccttctat actatctaaa ttaataaata ggcaattacc
      601 aaccagcttg tttgataagc ttaatgatct tgggattaaa ttgtttcctt caaattggga
      661 agagatgaat gcaaattgca actgtcctga ttgggctatg ccttgtaaac acattgctgc
      721 tgtaatttat cttattgctg cagaaattga taaaaacccc ttcatgattt ttaatattca
      781 taattgtaat ttgtcagcac ttatcgatga ttttgggaat ggaaaattag aaaatgctca
      841 aaatatttta aagatagatg atatatttaa ggcatgttct aaaattagaa tacataacca
      901 agcaatttta aatgatattg atttatcaac tattcctaac ctttttgatt gtatcagtag
      961 tattttaaca gataatccac tttttttga aaaaaacttc cataatattt tacagatagc
     1021 ttataagcac tggcaaaagt atcctgcagg aaagttagtg tactatccag cttctaagaa
     1081 ggaactatca gaagaggagc tatttattaa aagatgggga aatattgaga attggcaaac
     1141 atttcaacta ttcattgatg atcagcatta ccttactcaa gtgagcaatg gtacaactaa
     1201 tgtatttatg ctaagtagaa atgtattgaa agatatagcg cgatttttaa acgacattcc
     1261 taattcgtta cttcacaggc ttaatcgtga acttcgcttc atgcatatga tttcacagtt
     1321 tgcacatatg ctgatggaaa agtcagcgct aataccacaa gtattacaaa acaaaagagg
     1381 agaaattata attaggtgga ttccggcgtt gtttaatgag tcagttaagg aaattcatag
     1441 caagatatca tctatttgtc cgcctctatt gattaaatat caagaaatcg ctatggaacc
     1501 agaggaacag gtaaatgttg ctatctctct catcctttta gggtatatag aagataactt
     1561 tcctgcatca ctggataagc atagagataa atacatcttt gaattattct ttactggaag
     1621 accatacaag tttactgagt ttagcaacaa ggaaataccg caagcaatta atctctggct
     1681 ttcacgtctt tatttagcag ataaacctta caaactctat ttaatgataa aagatcacca
     1741 tgaaaaattt gaacttgagg tacagttatc actggatgat gagaagtcat tcataaagct
     1801 ggaaaaagca ctttctaatc aaaacgtaaa gcttagtatt ttatctgatc ttgcactctt
     1861 atctgaatac atacctgaat tagaaagatc gattgatagc aatagcaagt tatcatttaa
     1921 tttagacgat tttgcacctt tattcctggg tattgtacca gtactaagag caataggtat
     1981 aacagttatc ttgccgaaat ctttgcaaaa aatcttcaaa ccacaattaa gtcttagttt
     2041 gtctgccaag gataaaataa aagaagatcg cgaaagtttt ttaacgctgg aaaatttatt
     2101 gaaatttgat tggaaagtag caataggaga taaaaaacta agcgttacag aatttaaaaa
     2161 attgctaaaa gattcccgag ggtttgtaag aatcgtggat caatacgtcc ttttggatga
     2221 caaagaggta gaagctttat taaaaaaact tgataaattg cctgattatt taagtcaagc
     2281 agagcttatg taagctgctc taacaggaga agtaaatgaa actaaagtag catttgacca
     2341 gcagcttaca agcttgttta aaaaatttaa tacttacaca cctgtaacta cgccaaataa
     2401 tctaatagca caactacgcc catatcaaga acgcggattt agttggcttg tacagaacat
     2461 agaaagtggg tttggtagca taattgctga tgatatgggt cttggtaaga ctatacaagt
     2521 tattgcagcc attctatact gtaaaaatgc aggattttta aatgaagata aagtcttgat
     2581 agtagcgcct acaagtatct taagcaattg gcaaagggag atagagcgtt ttgcaccaga
     2641 attaaagctt ttcatctatc acggacaaaa tcgagaattg gcaagtgatt atgatgtagc
     2701 tctaacatcc tatggtcttg cacgtcgtga taaaaaagaa cttaataaaa ttcgctggtt
     2761 tttgctcttg attgatgaag cacaaaatat aaaaaatcct aataccgaac aaaccaaggc
     2821 aattaaagct attgaggcaa cgcatagaat agcaatgagc ggtactcctg ttgagaatag
     2881 gttacttgag tattggagca tttttgattt tatcaataaa tcttatcttg gtacccctaa
     2941 gcagtttaag acctattttg caacaccaat tgaaaaagca agagatagaa cttgtctaga
     3001 aaggtttatg aaaatcacta gcccttttat gttacgtagg cttaagagtg ataaaagcat
     3061 tattcaggat ttaccagata aaattgagaa taatcgttat tgttccctca cttcagaaca
```

```
     3121 aacagctctt tatcaaaagg ttgttgatac aactatggag aagatagaaa agagtgaagg
     3181 aattgaacgt aagggactaa tctttaaact tatcaatgct ttaaagcaaa tttgtaacca
     3241 tccatcacag tttggcaaga aaaagcgtgc gagtattgaa caatccggta aaatgcagat
     3301 gcttgaggaa atattgatta gtattggtga agttgcagaa aaatcattga tatttactca
     3361 atacactgaa atgggtgaga ttatagctag gctacttgaa gaaaggtttg aatcaaaagt
     3421 gccattttta catggaggac tttctagaaa agcacgtgat acaatgatta atgactttaa
     3481 aaacttattc cagtccaaca ttcttatagt gtctttaaaa gctggtggaa cagggcttaa
     3541 tttaacagcg gcaaaccatg taatacatta cgatttatgg tggaatccag cagtggaagc
     3601 acaagcaaca gatcgggcct atcgtatcgg gcaagaacgt aatgttatgg tgtacaggct
     3661 actttcaaca ggtacatttg aagagcgtat tgatgagatg attcaaagca agaaggaatt
     3721 agcaaaccta actataagta gtggcgagag ttggattaca gaatttaaca acgatcaatt
     3781 aagagattta gttaatataa aaaatgcact ataatatagc tgagttattt aatgtaaaga
     3841 accagataac taccgctcaa tagttcaaaa tagagacaaa gatcaaaatt tacgtctcca
     3901 atttcatcat gtatagcgga gacaaaattt tgtgttttat gtctcattgg taggcaaaaa
     3961 acctgtaatt acgttttaaa gcgattttac cactctagat ccattgtacc tttttagcgc
     4021 ctgtaatgcc ctttaaaatt tgaataaaag gcatctttaa taattctatt catgaaccaa
     4081 aaagtaattg ccgttttcag aaacttttca tttttacaga tggcttgaaa agattatcat
     4141 taataggtag gccaaactcc tccaaagaaa aaagttttta attttttcag aaaaagtcat
     4201 aagaaacccg gtatatatat agtagagggt tttctattcg cttataattc caccttaaat
     4261 taaactatgc ctgcgtatat gagaaggttt agccaaaatt ccttacatat tagtagtatg
     4321 caatatatca acaaaataaa aactaagagg ttgacatagt gagtaaaatg tagtagagtt
     4381 atagtagagc atttttttctt aaaagaaaga agatactaaa gcctcacttt tcactataaa
     4441 cctaaactga cttcctttat ttcttaatat agccttgcca agagaacctc ctattgcagt
     4501 ttttacttca taagaagaga gtaagacccc aatcaaaaca gccttactca aaaacctttc
     4561 aaacgcttaa tcttaaaagg agtttcaatt tatggtacaa gattttttaa ctgatgtcaa
     4621 acaaaaaaat actttaaatg atgaatatta tgattttaac aatactgtag aaccattaga
     4681 gcaatttgat gtagaagaaa taaaaagaca actattatta aacataagat catgcctttc
     4741 ttatttattc ccaaggggaa cttttcgtgg tgataagttt tatataggca atgtacaagg
     4801 tgataaagga aagagtctag tagtagaatt aagtggcagt gaagctggcc tatggcacga
     4861 ttttgcaaca aaagaaggtg gtgatatcat cgatctttgg gcagcagtac atagaaaaag
     4921 tacgagaata gagtttcctg aagtaatggc ttctatagct gagtggtttg gtcattttaa
     4981 aaagctcaaa acttcaggta atccaactgc atattgggat tattatgatg aaaacggaca
     5041 agctattgtt agagtctatc gttatgatga tgatagtgga aagcgatatc ttcctttttga
     5101 tgtgaaaaaa tctactttta ctgcaccaga aatcagacct ttgtacaata ttccaggcat
     5161 tttaaaatct gacaaagtta ttctagttga aggggaaaaa tgtgcagaag cactgataaa
     5221 acaaggtatt acagctacaa caacaatgtc tggagcaaac gcagatgtta aaaagactga
     5281 ttggtcacaa ctgaagggca agcatattat tatttggcca gataatgatg aagcaggtgc
     5341 aaaatatgct aaaaatgttg gaaataagct tttagaaatt ggagttgcat cacttgctgt
     5401 gcttgaaatt cctcgaggta agcctgagaa atgggatgct gctgattgtg tagaggaagg
     5461 tatagatgtc aaagagtttt tagcttcaac tccgttactt actattgata ctccagctac
     5521 tgatacttca aatatgaaaa gtttagaaag atccacaaaa cagccactta acattttaga
     5581 ttggagtgca gagcgctttg taggtccggt accagagcaa aagtttcttg ttgaaggtct
     5641 atttccttta ggtgttactt caataatagc agcaatggga gatacaggca aaggtatgct
     5701 tcttcttgac ctggcactta aggttgcaag tgatatggat caactgtgcg gttttggtcc
     5761 tttggttact gaacatggat cagtagtgat tttttcagca gaagatgatg caagtgagat
     5821 acaccgccgc ttagaacgac ttgaccctaa gtgtgaaagg ttgaaacata aagataaatt
     5881 atttattgta ccactgccaa acgttagagg atcacttaca atactcagaa atgttcgtgg
     5941 taaaatcgtc gaagtttctc ctgaattcga atccgtaatg aaacagctcg aggaaattaa
     6001 agatctgaaa ttgatagtat ttgatccact tgcttcattt a
//
LOCUS       JAQZAU010000053         6975 bp    dna     linear   ENV 17-MAR-2023
DEFINITION  MAG: Wolbachia pipientis isolate wEsol contig_34043, whole genome
            shotgun sequence.
ACCESSION   JAQZAU010000053 JAQZAU010000000
VERSION     JAQZAU010000053.1
DBLINK      BioProject: PRJNA929258
            BioSample: SAMN33142973
KEYWORDS    WGS; ENV; Metagenome Assembled Genome; MAG.
SOURCE      Wolbachia pipientis (insect metagenome)
  ORGANISM  Wolbachia pipientis
```

```
            Bacteria; Pseudomonadota; Alphaproteobacteria; Rickettsiales;
            Anaplasmataceae; Wolbachieae; Wolbachia.
REFERENCE   1  (bases 1 to 6975)
  AUTHORS   Fiutek,N., Couger,B.M., Roy,S., de la Torre,J.R. and Connor,E.F.
  TITLE     Genomic assessment of the contribution of the Wolbachia
            endosymbiont of Eurosta solidaginis to gall-induction
  JOURNAL   International Journal Molecular Sciences
REFERENCE   2  (bases 1 to 6975)
  AUTHORS   Fiutek,N., Couger,B.M., Roy,S., de la Torre,J.R. and Connor,E.F.
  TITLE     Direct Submission
  JOURNAL   Submitted (10-FEB-2023) Biology, San Francisco State University,
            1600 Holloway Ave, San Francisco, CA 94132, USA
COMMENT     ##Genome-Assembly-Data-START##
            Assembly Date          :: JUN-2019
            Assembly Method        :: FLYE v. 06-2019
            Genome Representation  :: Full
            Expected Final Version :: Yes
            Genome Coverage        :: 100.0x
            Sequencing Technology  :: Illumina HiSeq; PacBio Sequel
            ##Genome-Assembly-Data-END##
FEATURES             Location/Qualifiers
     source          1..6975
                     /organism="Wolbachia pipientis"
                     /mol_type="genomic DNA"
                     /submitter_seqid="contig_34043"
                     /isolate="wEsol"
                     /isolation_source="galls on Solidago altissima"
                     /host="Eurosta solidaginis MN-2018-EFC"
                     /db_xref="taxon:955"
                     /environmental_sample
                     /country="USA: Cowling Arboretum, Carleton College,
                     Northfield, MN"
                     /lat_lon="44.48 N 93.13 W"
                     /altitude="280 m"
                     /collection_date="2018-11"
                     /metagenome_source="insect metagenome"
                     /note="metagenomic"
     gene            complement(<1..460)
                     /locus_tag="wEsol_00513"
     CDS             complement(<1..460)
                     /locus_tag="wEsol_00513"
                     /codon_start=1
                     /transl_table=11
                     /product="hypothetical protein"
                     /pfam="Aa_trans AA_permease_2 Trp_Tyr_perm AA_permease"
                     /tigr="TIGR00905 2A0302 473 transporter, basic amino
                     acid/polyamine antiporter (APA) family"
                     /translation="MSNKIGFWAIFALVISSQIGSGIFMLPISLAPYGMYSLISWIIS
                     GLGAISLALVFASLCAKFPETGGPHVYVKHAFGPAAAFFVGWTYWVISWVSTTAVIVV
                     GVGYLTPFFHEDIQNVHLFLEMLLLTIITLINFRGVATAGRVEFLLTVIKI"
                     /cog="PotE COG0531 466 Amino acid transporters"
                     /besthit="qcoverage 100 , hcoverage 36.0849056603774 ,
                     similarity 100 , identity 100 , evalue 1.23e-96 ,
                     alnlength 153 , Bacteria , Proteobacteria ,
                     WP_022626385.1 amino acid permease [Wolbachia
                     endosymbiont of Drosophila melanogaster]"
     gene            596..2041
                     /locus_tag="wEsol_00514"
     CDS             596..2041
                     /locus_tag="wEsol_00514"
```

                     /codon_start=1
                     /transl_table=11
                     /fullproduct="qcoverage 100 , hcoverage 100 , similarity
                     100 , identity 100 , evalue 0.0 , alnlength 481 ,
                     Bacteria , Proteobacteria , WP_197907256.1 hypothetical
                     protein [Wolbachia endosymbiont of Kradibia gibbosae]"
                     /translation="MNPRLKAFLVVCSIIAFLIALLFLVYYLNLLVNYILDPILVRPF
                     ERLYKNLIVDGLYAYLSTSTSSPALAFLATGFIVIAAFICLNKMLLPLQEKIINNYTS
                     FKNNNIDSNSMLFEVYKDPTIKLKDKILFFSIHLIYYLQLPFLYIASKVSALRGFLIF
                     NFGLLLLNYALLIPVALLELIKYPLVRLFSPLGLNVKQLSFLFNVSDVGTGSQSVHTS
                     SFEHSILQCAQELKQKFGAQPNVLNKEFEDYINNSDELTTEQKEQLKLYLNFNGSNEA
                     AWQESKTGLTLTQAANLVSTAAKNQKSDVNSLLLMNLKDGLGLCTLGMFDRIIYSLSC
                     LEAKNNFLVQINGAVNVLTPKFTERFLKEYCNYKKLKILVDKFEDFYSEDYADKNNIS
                     DEVKSNINQSIIEIREFVFKELYIKFYEEYGKSIQRGAIKQKLRELVTDDSIKEAVYY
                     SMYNIEIPAEPPTYLERVKAFFGGHARSSAA"
                     /product="hypothetical protein"
                     /besthit="qcoverage 100 , hcoverage 100 , similarity 100
                     , identity 100 , evalue 0.0 , alnlength 481 , Bacteria ,
                     Proteobacteria , WP_197907256.1 hypothetical protein
                     [Wolbachia endosymbiont of Kradibia gibbosae]"
     gene   2176..3174
                     /locus_tag="wEsol_00515"
     CDS   2176..3174
                     /locus_tag="wEsol_00515"
                     /codon_start=1
                     /transl_table=11
                     /fullproduct="qcoverage 99.3975903614458 , hcoverage
                     80.4878048780488 , similarity 98.8 , identity 98.5 ,
                     evalue 2.47e-223 , alnlength 330 , Bacteria ,
                     Proteobacteria , ERN55350.1 aspartate kinase [Wolbachia
                     pipientis wMelPop]"
                     /cog="LysC COG0527 447 Aspartokinases"
                     /pfam="AA_kinase"
                     /product="aspartate kinase"
                     /translation="MACKLLSLQKLYFSSEESYIVTMDKIIIKKFGGTSLTDLNRVAN
                     LIKNDIEKGCNVIVVVSAVAGFTDQMVFQARQISNLSYRQELSEYDVMLSAGEQISCG
                     LLAITLQSIGVNAKSWLAWQLPIATDDFYSESKIKTIKIERVKRSFAEGYTAAIIAGF
                     QGINDDRITTFGRGGSDISAVAFAVAFGVRTCEIFTDIDGIYTADPRIVPKARKLKFI
                     SYDEMLEMSSSGAKILHNRSVQLAMKHNIKVQVLSTFKEVEGTTVLHKRDVLERYLIT
                     GITYSTNEALVTFTNLANNLRTLRDIAGANVKIDMIHGSSFVISKFDIDFNGKVTE"
                     /besthit="qcoverage 99.3975903614458 , hcoverage
                     80.4878048780488 , similarity 98.8 , identity 98.5 ,
                     evalue 2.47e-223 , alnlength 330 , Bacteria ,
                     Proteobacteria , ERN55350.1 aspartate kinase [Wolbachia
                     pipientis wMelPop]"
     gene   3113..3409
                     /locus_tag="wEsol_00516"
     CDS   3113..3409
                     /locus_tag="wEsol_00516"
                     /codon_start=1
                     /transl_table=11
                     /product="hypothetical protein"
                     /pfam="ACT_7"
                     /tigr="TIGR00656 asp_kin_monofn 407 aspartate kinase,
                     monofunctional class"
                     /translation="MGQVLLSPNLILISMEKLLNKNENYAINDNVAKISIIGIGVMSN
                     TEVMHRTLKVLSEKKIEILAITTSEIKISIIVQKECAVALVKDLHTEYELDMQH"
                     /cog="LysC COG0527 447 Aspartokinases"
                     /besthit="qcoverage 85.7142857142857 , hcoverage 50 ,
                     similarity 100 , identity 100 , evalue 1.40e-46 ,

```
                     alnlength 84 , Bacteria , Proteobacteria , WP_006279315.1
                     ACT domain-containing protein, partial [Wolbachia
                     endosymbiont of Muscidifurax uniraptor]"
     gene            3501..3704
                     /locus_tag="wEsol_00517"
     CDS             3501..3704
                     /locus_tag="wEsol_00517"
                     /codon_start=1
                     /transl_table=11
                     /fullproduct="qcoverage 100 , hcoverage 100 , similarity
                     100 , identity 100 , evalue 8.38e-47 , alnlength 67 ,
                     Bacteria , Proteobacteria , WP_155969026.1 MULTISPECIES:
                     transposase [Wolbachia]"
                     /cog="COG3293 COG3293 124 Transposase and inactivated
                     derivatives"
                     /pfam="DUF4096"
                     /product="transposase"
                     /translation="MYPSDISDKKWEILEPHVAQGKIGRPRKHNIRTIINAIRYIMRG
                     GCQWRMLPKDFPPWKTVYDYFLR"
                     /besthit="qcoverage 100 , hcoverage 100 , similarity 100
                     , identity 100 , evalue 8.38e-47 , alnlength 67 ,
                     Bacteria , Proteobacteria , WP_155969026.1 MULTISPECIES:
                     transposase [Wolbachia]"
     gene            3738..3872
                     /locus_tag="wEsol_00518"
     CDS             3738..3872
                     /locus_tag="wEsol_00518"
                     /codon_start=1
                     /transl_table=11
                     /fullproduct="qcoverage 93.1818181818182 , hcoverage
                     97.6190476190476 , similarity 100 , identity 100 , evalue
                     2.12e-19 , alnlength 41 , Bacteria , Proteobacteria ,
                     ONI56075.1 hypothetical protein N500_0846 [Wolbachia
                     pipientis wUni]"
                     /translation="MLVKEVRKMFGKNETPSVGIIDSQSVKTTQKGDPEDMMLKKNKG
                     "
                     /product="hypothetical protein"
                     /besthit="qcoverage 93.1818181818182 , hcoverage
                     97.6190476190476 , similarity 100 , identity 100 , evalue
                     2.12e-19 , alnlength 41 , Bacteria , Proteobacteria ,
                     ONI56075.1 hypothetical protein N500_0846 [Wolbachia
                     pipientis wUni]"
     gene            4281..4493
                     /locus_tag="wEsol_00519"
     CDS             4281..4493
                     /locus_tag="wEsol_00519"
                     /codon_start=1
                     /transl_table=11
                     /pfam="RVT_1"
                     /fullproduct="qcoverage 100 , hcoverage 100 , similarity
                     100 , identity 100 , evalue 2.45e-45 , alnlength 70 ,
                     Bacteria , Proteobacteria , ONI57615.1 RNA-directed DNA
                     polymerase domain protein [Wolbachia pipientis wVitA]"
                     /translation="MHHAFDIWMKQNHPTVPFERYVDDAIVHCRTKRQAEFMRAAIEE
                     RLAQCKLKLQPDTNAIPKLHHYTALV"
                     /product="RNA-directed DNA polymerase domain
                     protein"
                     /besthit="qcoverage 100 , hcoverage 100 , similarity 100
                     , identity 100 , evalue 2.45e-45 , alnlength 70 ,
                     Bacteria , Proteobacteria , ONI57615.1 RNA-directed DNA
```

```
                     polymerase domain protein [Wolbachia pipientis wVitA]"
     gene            complement(4648..5100)
                     /locus_tag="wEsol_00520"
     CDS             complement(4648..5100)
                     /locus_tag="wEsol_00520"
                     /codon_start=1
                     /transl_table=11
                     /fullproduct="qcoverage 100 , hcoverage 100 , similarity
                     99.3 , identity 98.0 , evalue 6.49e-101 , alnlength 150 ,
                     Eukaryota , Arthropoda , GFR20813.1 DNA helicase II
                     [Trichonephila clavata]"
                     /cog="UvrD COG0210 655 Superfamily I DNA and RNA
                     helicases"
                     /pfam="UvrD_C UvrD_C_2"
                     /tigr="TIGR01073 pcrA 725 ATP-dependent DNA helicase
                     PcrA"
                     /product="DNA helicase II"
                     /translation="MLENEEVTGLARIENVKELISSLKNFDNATTFLEHISLVMEVDN
                     MNNDDTVYVMTLHAAKGLEFPCVFLPGWEEGLFPHQRSFDDKSGKALEEERRLAYVGI
                     TRAKERLIISCADRREINNQWQPMRTSRFIKELPRENVEVIKSNIAYC"
                     /besthit="qcoverage 100 , hcoverage 100 , similarity 99.3
                     , identity 98.0 , evalue 6.49e-101 , alnlength 150 ,
                     Eukaryota , Arthropoda , GFR20813.1 DNA helicase II
                     [Trichonephila clavata]"
     gene            complement(5102..6364)
                     /locus_tag="wEsol_00521"
     CDS             complement(5102..6364)
                     /locus_tag="wEsol_00521"
                     /codon_start=1
                     /transl_table=11
                     /product="hypothetical protein"
                     /pfam="UvrD-helicase Pfam-B_8467 UvrD_C"
                     /tigr="TIGR01073 pcrA 725 ATP-dependent DNA helicase
                     PcrA"
                     /translation="MVSRVLELTGTNIPWLGTFHAIAAKILRMHAEIVGLNPNFTIIG
                     VDDQLQVIKNIINEINPDYLSEKCKTIMNIIQQWKEKCLLPSEVEDIQSFRPVYVTAL
                     KVYHQYQERLKFLNSVDFGDLLLYNIQLFNQKTEVLSYYQNKFKYVMVDEYQDTNAIQ
                     YLLLKYLAKEHSNICCVGDDDQSIYSWRGAEVENILKFSDDFKNAKTVKLECNYRSTS
                     HILATASYVINHNKTRLEKKLWTTNIEGEKVNLIKLWDGKAEARFISEQILKLNKYRF
                     SDIAVLVRATFQTRVLEEYFIKYSIPYKIISGVKFYERQEVRDIIAYLRLVTNNNDDL
                     AFERIVNRPKRSIGATTLKKIYTTAQDNKISFFEAAKILVNSNQVTERIKLSLNDFLN
                     KIKAWEEIVSVKPLHEFVKIVANQSGIY"
                     /cog="UvrD COG0210 655 Superfamily I DNA and RNA
                     helicases"
                     /besthit="qcoverage 99.5238095238095 , hcoverage
                     65.5172413793103 , similarity 100 , identity 100 , evalue
                     2.56e-299 , alnlength 418 , Bacteria , Proteobacteria ,
                     WP_015589293.1 MULTISPECIES: UvrD-helicase
                     domain-containing protein [unclassified Wolbachia]"
     gene            complement(6378..6566)
                     /locus_tag="wEsol_00522"
     CDS             complement(6378..6566)
                     /locus_tag="wEsol_00522"
                     /codon_start=1
                     /transl_table=11
                     /product="hypothetical protein"
                     /pfam="UvrD-helicase AAA_30 AAA_19"
                     /tigr="TIGR01073 pcrA 725 ATP-dependent DNA helicase
                     PcrA"
                     /translation="MNDYLSLLNPEQQSAVTNIDGPVLILAGAGTGKTRTITSRIAHI
```

```
                     IRNGHAYSDEILAVTFHK"
                     /cog="UvrD COG0210 655 Superfamily I DNA and RNA
                     helicases"
                     /besthit="qcoverage 96.7741935483871 , hcoverage
                     53.0973451327434 , similarity 100 , identity 100 , evalue
                     1.20e-33 , alnlength 60 , Eukaryota , Arthropoda ,
                     GFQ94843.1 probable DNA helicase II homolog
                     [Trichonephila clavata]"
     gene            complement(6667..>6975)
                     /locus_tag="wEsol_00523"
     CDS             complement(6667..>6975)
                     /locus_tag="wEsol_00523"
                     /codon_start=1
                     /transl_table=11
                     /product="hypothetical protein"
                     /translation="NNTLASFSRSWREFKEKAYGFMYDIGTWLKETYENLTERPATHN
                     HYASKFPVKVEEREMEELNRTYKSFQTPPIHLTNSSLDQINLSLPTTSKLPNKGIYKT
                     "
                     /besthit="qcoverage 100 , hcoverage 74.4525547445255 ,
                     similarity 100 , identity 100 , evalue 1.45e-68 ,
                     alnlength 102 , Bacteria , Proteobacteria ,
                     WP_015589294.1 MULTISPECIES: hypothetical protein
                     [unclassified Wolbachia]"
ORIGIN
        1 aaatttttat caccgtcaat aaaaactcaa cacgtccagc agtagctact cctctaaaat
       61 ttattagtgt aataattgta agtaataaca tctctaaaaa cagatgtacg ttttgaatat
      121 cttcatggaa aaatggtgta agataaccaa cacccacaac tattacggct gttgtgctaa
      181 cccatgagat cacccaatac gtccaaccaa caaaaaaagc tgcagcaggg ccaaaagcat
      241 gctttacata gacgtgaggg ccacccgttt ccggaaattt tgcgcagagt gaggcaaaaa
      301 ctaaagcaag agatatagca ccaagccctg atattatcca gcttataagg ctatacatgc
      361 catatggagc aaggctaatt ggaagcataa aaattccaga gccaatttgg ctgctaatca
      421 ctaaggcaaa aatagcccaa aaacctattt tatttgacac aataattctt atctaaatag
      481 attaacaata taccctaaac tcagaaattt caattgatct tttaacataa aaacttgcat
      541 ttttttcaat ttattcctaa aatagattat attaacagat tattaagagg gggttatgaa
      601 tccgagatta aaagcatttt tggttgtttg tagtattatt gcatttttga ttgcactttt
      661 gtttctcgtg tattatttaa atcttcttgt taactatatc cttgacccga ttctagttcg
      721 tccctttgaa agattataca aaaacttaat tgttgatggt ctttatgcct atttatctac
      781 aagcacttct agccctgcac tagcattttt ggctacaggt tttattgtta ttgctgcgtt
      841 tatttgcctg aataaaatgc ttcttccctt gcaagagaag atcataaaca actataccte
      901 ctttaaaaac aacaatattg atagcaatag catgttgttt gaggtttata aagatccaac
      961 aataaaatta aaggacaaaa tcttattctt cagtatacac ttgatatatt acctacagct
     1021 tccatttttg tatattgcat caaaggtatc agctctacgt gggtttctta tatttaattt
     1081 tggtttatta ttgttaaatt atgcactgct aattccagtg gcacttcttg aattgataaa
     1141 gtatccatta gtgaggcttt tctcaccttt gggtttgaat gtaaagcagc taagcttttt
     1201 gtttaatgta agtgatgtgg gcactggcag tcagagcgtt cacacgagta gttttgaaca
     1261 tagtattctc caatgcgctc aagagttaaa acagaaattt ggagctcaac caaatgtact
     1321 caataaagag tttgaagact acataaataa ttcagatgag cttacaactg agcaaaaaga
     1381 gcaattaaaa ctttatctta actttaatgg ttccaatgaa gcagcttggc aagaatcaaa
     1441 aactggacta actctcacac aagctgcaaa cttagtttca actgctgcta agaatcaaaa
     1501 atcagatgtg aattcactat tactaatgaa tttaaaggat ggacttggtt tatgtactct
     1561 gggaatgttc gatcgtatta tttattcgct tagttgtctt gaagcgaaaa ataatttctt
     1621 ggttcaaata aatggagcag tgaacgtatt aacacctaaa tttactgaaa gatttttaaa
     1681 agaatattgc aattataaaa aattaaaaat tttggtagac aagtttgaag atttttactc
     1741 cgaagattat gcggataaaa ataatataag cgatgaggtg aagagcaaca ttaaccaatc
     1801 aataatagaa ataagagaat ttgtctttaa agagctttac ataaaatttt acgaagagta
     1861 tggaaaaagt attcaaagag gtgccataaa gcaaaaatta agggaattag ttacagatga
     1921 tagtataaag gaagcagtgt attattcaat gtataatata gaaatccctg cagaaccacc
     1981 tacttatctt gaaagagtaa aagcattttt tggaggtcat gcaaggtctt ctgctgctta
     2041 gtggtttagt tcgtgctata tagctaaact aacttagatt tactgacaat tttaaaacaa
     2101 caaattcgtc attccgctac tagttagcgc cgcggcggta tgacgtgttc tatagctttg
```

2161 tcttcccttt tctgaatggc ttgcaagctg ctatctctac aaaaactata tttttcttca
2221 gaagagagtt atattgtaac tatggacaaa ataatcataa aaaaatttgg tgggacttcg
2281 ctaactgatt taaaccgagt tgcaaatttg ataaaaaacg atattgagaa aggttgtaat
2341 gtaatcgttg ttgtatctgc tgttgcagga tttactgacc aaatggtttt tcaggctagg
2401 caaatctcaa atttaagtta tagacaagag ttatcagagt atgacgttat gctttcagca
2461 ggagaacaaa tctcttgcgg tctattagct atcactctcc aatcgatcgg agttaatgct
2521 aaatcgtggc ttgcctggca gttaccaatt gcaactgatg atttttattc tgagtctaaa
2581 ataaaaacaa taaagattga acgtgtgaaa agatcttttg ctgagggtta tactgctgcg
2641 atcattgctg gttttcaggg tataaatgat gatagaatca ctacttttgg aagaggaggc
2701 tctgatatat cagcagttgc cttcgcggta gcctttggtg ttagaacttg cgaaattttt
2761 actgatattg atggaatata tacagcagac ccgagaattg ttccaaaggc acgcaagctc
2821 aaatttatct cttacgatga aatgttggaa atgtcgtcat ctggtgctaa aatactgcat
2881 aatcgttcag tacaacttgc aatgaaacat aacattaaag tgcaagtgct atccactttt
2941 aaggaagtag aaggcaccac agtgctacac aaaagggatg tactagagag atacttaatt
3001 actggaataa cttatagcac taatgaagct cttgtaactt tcactaacct tgcaaataac
3061 ttgcgtactt taagagatat agcaggagca aacgttaaaa ttgatatgat acatgggtca
3121 agttttgtta tctccaaatt tgatattgat ttcaatggaa aagttactga ataagaatga
3181 aaattatgct ataaacgata atgtagctaa aatttcaata attggtattg gtgttatgtc
3241 taacactgaa gtgatgcacc gcacactcaa ggttttaagc gaaaaaaaga tagaaatact
3301 tgctatcaca acatctgaaa tcaaaatcag tataattgtt caaaaagaat gcgctgtagc
3361 tttagtcaaa gatttacata ctgagtatga gcttgatatg cagcactagg ataggcttga
3421 cgtccagttg tctaagagta tgtcttgaaa aggaatgagg aaaaaaatat gatagtgtaa
3481 gtaaaaaata ggagggaaaa ttgtatccaa gtgatataag tgataagaaa tgggaaattt
3541 tggagccaca tgttgcacaa ggaaagatag ggaggccaag aaagcacaat atcagaacaa
3601 ttattaatgc aataaggtat ataatgagag gtggttgtca atggagaatg ctgccgaaag
3661 attttcctcc atggaagacg gtgtatgact attttctcag gtgacgtagt aatggcaaat
3721 tggaaaaaat acataatatg ctagtaaaag aggtaagaaa aatgtttggt aaaaatgaaa
3781 caccttcagt aggaataatt gacagtcaat cagtaaaaac cactcaaaaa ggggatccag
3841 aggatatgat gctgaaaaaa aataaagggt agaaaacgtc atattgttgt agatacagtg
3901 ggcttaatta ttgcggctga tgttcatagt gcaagtgttc aagatcgtga tggtgctcta
3961 aatcttttca ctcaagctaa atacaaggct ccaactttac gtaagttttt gctgatcaag
4021 gatacatagc aaaaccgctg tttattggag acaggatacg attaccaaga aagctgttga
4081 tgttacagga tttttcgaca atctggacca caatttagca ctgcaggcta tcaagaagca
4141 cacagactgc aaatgggtca tactgtatgt tgaaaggtgg atgaaatccc ctattcagca
4201 agcagatggc agtaaggtag ttagggaaaa aaggagttcc gcaaggaggt tcaataagcc
4261 caatcatttc aaacatattt atgcaccatg catttgatat atggatgaaa caaaatcacc
4321 caacagtacc atttgaaaga tatgtggatg atgcgatagt gcactgcaga actaaaagac
4381 aggcagaatt tatgcgagca gcaattgaag aaagattggc acaatgtaag ctaaaattgc
4441 aacctgatac caatgccatt ccaaaactcc atcattatac tgccttagta taaccttctc
4501 acttctattg tctatttgtt tgttatgtaa tagaaattgg tatgagattg aagaattaag
4561 tgtgaacggt tacagaattt ttgccaagag aaacatatag ttaaagtaat tcagatttgt
4621 aagatatgtt tatctgcaat tatagcttca acagtaggca atgttgcttt taattacctc
4681 aacattttct cttggcaatt ctttaatgaa ccgagaagtg cgcatcggtt gccactggtt
4741 gttaatttcc ctcctatctg cacatgaaat gattaacctt tctttggctc tggttatgcc
4801 aacatatgcg agtctccttt cttcttctaa agctttacca cttttgtcat caaaagatct
4861 ttgatgagga aataatccct cttcccaacc aggtagaaac acgcacggga attcaagtcc
4921 tttggctgca tgaagagtca taacatatac ggtgtcatcg ttattcatat tatccacttc
4981 catcaccaga cttatatgct ccaagaaagt tgtggcatta tcgaaatttt ttaaagatga
5041 gatgagctct ttaacatttt ctattcgtgc taagcctgtt acctcctcat tttcaagcat
5101 ttcaatatat ccctgattgg tttgctacga ttttaacgaa ttcatgcagt ggttttacgc
5161 ttactatttc ttcccaagct ttaattttat ttaaaaaatc gttgagtgag agtttaattc
5221 tttcggttac ctgattacta ttaactaata tttttgctgc ctcaaaaaaa gaaattttgt
5281 tatcctgagc agtcgtgtat attttcttta gagttgtagc tcctatgctt ctttttggcc
5341 gatttactat cctttcgaaa gctaagtcat cgttattgtt tgtaacaagt cttaaatatg
5401 caattatatc cctgacttcc tgacgttcgt agaattttac gccacttata atcttgtagg
5461 gaattgaata ttttataaaa tactcttcga gaactctagt ttgaaaagtg gcccttacca
5521 gcactgcaat gtcactaaat ctgtatttat tgagtttcaa tatctgttcg cttatgaacc
5581 ttgcttcagc tttcccatcc cataatttta ttagatttac cttttcccct tcaatgtttg
5641 ttgtccacaa tttttttct aagcgagtct tattgtgatt gataacatat gacgcagttg
5701 caagtatgtg ggatgttgac ctgtagttac actctagttt aacggttttt gcatttttaa

```
     5761 aatcatcaga aaacttcaaa atattttcaa cttctgcacc acgccaacta tatatcgact
     5821 ggtcatcatc tcctacacag caaatatttg agtgctcttt tgcaaggtat tttagcaaaa
     5881 gatattgtat cgcatttgta tcttgatatt catctaccat gacatattta aacttgtttt
     5941 ggtagtagga cagaacttcg gtcttttgat taaagagttg tatgttatat agcaataagt
     6001 caccaaaatc gacagagtta aggaatttca acctttcctg atactggtga tagaccttga
     6061 gtgcagttac atatactggc ctaaatgatt gaatatcttc cacttcagat ggcaataagc
     6121 acttctcttt ccattgctga ataatattca taatggtctt acatttttct gataggtaat
     6181 caggatttat ttcattgata atatttttta ttacctgtaa ttggtcatcc acaccaatga
     6241 ttgtaaaatt ggggtttaag cccacaattt ctgcgtgcat gcgcaaaatt tttgctgcaa
     6301 ttgcatggaa agtgccaagc catggtatgt ttgtgcccgt tagctcaagt acccttgata
     6361 ccatctcatt tgctgcttta tttgtgaaat gtaactgcta atatctcatc agaataagcg
     6421 tgaccgtttc taattatgtg tgctattctt gaagtaatcg ttcttgtttt tcccgtcccg
     6481 gctcctgcca gtatcaaaac tggtccatct atgttagtta cagctgattg ttgttctgga
     6541 tttagcagtg agagataatc gttcatcgta gccttataga ttataacttc gtaaagttaa
     6601 acctatgatt gaaatattgt aaaggtttta ttgaatagaa cagttaaaga atgcttcact
     6661 caagtgttac gttttgtata tacctttgtt aggcagcttg gatgtggtgg gtaacgataa
     6721 attgatctga tctaggctgc tgtttgttag gtggataggt ggagtttgga atgacttata
     6781 tgtacggttt agctcctcca tttctctttc ttcaacttta acagggaact ttgaggcata
     6841 gtggttatgc gttgcaggtc gttcagtgag attttcatat gtttctttca accaagttcc
     6901 gatatcatac ataaatccat acgctttttc tttaaattct ctccatgatc tactgaaact
     6961 tgctagtgtg ttatt
//
LOCUS       JAQZAU010000054           61158 bp    dna     linear   ENV 17-MAR-2023
DEFINITION  MAG: Wolbachia pipientis isolate wEsol contig_34079, whole genome
            shotgun sequence.
ACCESSION   JAQZAU010000054  JAQZAU010000000
VERSION     JAQZAU010000054.1
DBLINK      BioProject: PRJNA929258
            BioSample: SAMN33142973
KEYWORDS    WGS; ENV; Metagenome Assembled Genome; MAG.
SOURCE      Wolbachia pipientis (insect metagenome)
  ORGANISM  Wolbachia pipientis
            Bacteria; Pseudomonadota; Alphaproteobacteria; Rickettsiales;
            Anaplasmataceae; Wolbachieae; Wolbachia.
REFERENCE   1  (bases 1 to 61158)
  AUTHORS   Fiutek,N., Couger,B.M., Roy,S., de la Torre,J.R. and Connor,E.F.
  TITLE     Genomic assessment of the contribution of the Wolbachia
            endosymbiont of Eurosta solidaginis to gall-induction
  JOURNAL   International Journal Molecular Sciences
REFERENCE   2  (bases 1 to 61158)
  AUTHORS   Fiutek,N., Couger,B.M., Roy,S., de la Torre,J.R. and Connor,E.F.
  TITLE     Direct Submission
  JOURNAL   Submitted (10-FEB-2023) Biology, San Francisco State University,
            1600 Holloway Ave, San Francisco, CA 94132, USA
COMMENT     ##Genome-Assembly-Data-START##
            Assembly Date          :: JUN-2019
            Assembly Method        :: FLYE v. 06-2019
            Genome Representation  :: Full
            Expected Final Version :: Yes
            Genome Coverage        :: 100.0x
            Sequencing Technology  :: Illumina HiSeq; PacBio Sequel
            ##Genome-Assembly-Data-END##
FEATURES             Location/Qualifiers
     source          1..61158
                     /organism="Wolbachia pipientis"
                     /mol_type="genomic DNA"
                     /submitter_seqid="contig_34079"
                     /isolate="wEsol"
                     /isolation_source="galls on Solidago altissima"
                     /host="Eurosta solidaginis MN-2018-EFC"
```

```
                     /db_xref="taxon:955"
                     /environmental_sample
                     /country="USA: Cowling Arboretum, Carleton College,
                     Northfield, MN"
                     /lat_lon="44.48 N 93.13 W"
                     /altitude="280 m"
                     /collection_date="2018-11"
                     /metagenome_source="insect metagenome"
                     /note="metagenomic"
     gene            complement(<1..629)
                     /locus_tag="wEsol_00524"
     CDS             complement(<1..629)
                     /locus_tag="wEsol_00524"
                     /codon_start=1
                     /transl_table=11
                     /product="hypothetical protein"
                     /pfam="ParBc Pfam-B_14045 HTH_Crp_2 KorB"
                     /tigr="TIGR00180 parB_part 186 ParB/RepB/Spo0J family
                     partition protein"
                     /translation="MKDDRRLGRGLAGLIGDNYDNKEDRQEHLPISLLHPSKFQPRKH
                     FDEESLRELANSIKKSGIIQPIVVRKDSNDDGYEIIAGERRWRASKIANLDSAPVIIK
                     DLSDKECLEVSIIENIQRQDINPIEEGEAYRKLIDEFFYTHEELASAIGKSRSHITNM
                     IRMLLLPDRVKTMINEKKLSMGHARALINVENAESIAEKIVSQGLSVRQ"
                     /cog="Spo0J COG1475 240 Stage 0 sporulation protein J
                     (antagonist of Soj) containing ParB-like nuclease domain"
                     /besthit="qcoverage 100 , hcoverage 79.467680608365 ,
                     similarity 100 , identity 100 , evalue 4.13e-143 ,
                     alnlength 209 , Eukaryota , Arthropoda , XP_044573786.1
                     probable chromosome-partitioning protein ParB [Drosophila
                     ananassae]"
     gene            complement(626..1468)
                     /locus_tag="wEsol_00525"
     CDS             complement(626..1468)
                     /locus_tag="wEsol_00525"
                     /codon_start=1
                     /transl_table=11
                     /fullproduct="qcoverage 100 , hcoverage 100 , similarity
                     100 , identity 100 , evalue 1.14e-190 , alnlength 280 ,
                     Bacteria , Proteobacteria , WP_006280226.1 MULTISPECIES:
                     AAA family ATPase [Wolbachia]"
                     /cog="Soj COG1192 259 ATPases involved in chromosome
                     partitioning"
                     /pfam="Pfam-B_3718 Pfam-B_2460 AAA_31 MipZ YhjQ
                     Pfam-B_4013 CbiA ArsA_ATPase Fer4_NifH"
                     /product="AAA family ATPase"
                     /translation="MSKIIAIVNQKGGVGKTTTSINLSTAFAAVGKSTLLVDLDPQGN
                     ASTGLGISYRSREEKNIYKILLSSENKLIESAIFNIKEIPNLSLISSVVDLSAAEIEL
                     SQLERGKFVLKSALEKIRDNYEYIIIDCPPSLGLLTINALTAANSIIVPLQCEFFALE
                     GLSHLVKTVELIKRNNLNPFLAIEGIVLTMYDRRNKLSEQIKNDICQYLNDKVYKTII
                     PLYETVIPRNVRLSEAPSHGKPAIVYDLKCPGAQAYISLAREILKKHASSCKEKKLVS
                     EGVV"
                     /besthit="qcoverage 100 , hcoverage 100 , similarity 100
                     , identity 100 , evalue 1.14e-190 , alnlength 280 ,
                     Bacteria , Proteobacteria , WP_006280226.1 MULTISPECIES:
                     AAA family ATPase [Wolbachia]"
     gene            complement(1559..4042)
                     /locus_tag="wEsol_00526"
     CDS             complement(1559..4042)
                     /locus_tag="wEsol_00526"
                     /codon_start=1
```

```
                     /transl_table=11
                     /pfam="PAS_8 PAS_9 Pfam-B_10272 HisKA HATPase_c
                     Response_reg"
                     /fullproduct="qcoverage 100 , hcoverage 100 , similarity
                     100 , identity 100 , evalue 0.0 , alnlength 827 ,
                     Bacteria , Proteobacteria , WP_174516849.1 response
                     regulator [Wolbachia endosymbiont of Cardiocondyla
                     obscurior]"
                     /translation="MSKRYIDNKKENRVDFILRNYGMSIVVMAVIMLLPPVAISILYF
                     FDIYSQHINIEINLLVSILTTLFMMYNVKRYRYLLNTIEFQNAIFANALNHNTEFYLI
                     LHKDEGVIYADARFYERFKDHIIDDDITLGKILEIGDVSEADKKVLYHALKNNSSVQV
                     CISLNKKNRVSNFLLLFEPIPDNPQIAINSNKILNLSLVPIARPDGYFVLKATQINKE
                     QIYEELIEKHSIGTYIANAKGVILSVNKRFLDIFELKKLEKGSSISDFISQSKYNNTI
                     TDNEILFFTISGIPFKAYMSTAIFCDKYNHSYIYGFITPTESNLVDYQLHPCFANSSI
                     AIAQCDINGNFVKKNTALIKLAGSDNNSIFTLILDSYHVKIREYFSSNRINNASFDVQ
                     LNGGNNIKIYFNKFLHNKMIFILCYFIDNTEHKNLEIKLEHYQKMQAIGQLAGGIAHD
                     FNNILTGIIGFCDLLLLQHSAGDPSFGDIIQIQQNAKRGSNLVRQLLAFSRRQTLQPK
                     IIDVNNTIANLYEMIKRLIGENIKFNIYYGRDLGAVRADQGQLEQVILNLAVNASAAM
                     EKGGELTIRTFNQKIDSLNSTPQDMFSPDKEAIEHGNYVVIEVIDTGCGMTSDTTEKI
                     FDPFFSTKDITSGTGLGLSTVYGIIKQTEGYIYVASKVNHGTKFSIFLPMVYISDEND
                     REEDSEETEKPVVSEIKGNGIILLIEDEDSVREFISKALKRKGFDVIEASIGSEALEI
                     ISKKSQHIDLIITDVIMPEVSGPEIVKEALIHRPNINVIFISGYAEDAFLKSDDINIE
                     DFHFLPKPFTLNELGNKVQSVLHKAKKTV"
                     /product="response regulator"
                     /besthit="qcoverage 100 , hcoverage 100 , similarity 100
                     , identity 100 , evalue 0.0 , alnlength 827 , Bacteria ,
                     Proteobacteria , WP_174516849.1 response regulator
                     [Wolbachia endosymbiont of Cardiocondyla obscurior]"
     gene            4203..5027
                     /locus_tag="wEsol_00527"
     CDS             4203..5027
                     /locus_tag="wEsol_00527"
                     /codon_start=1
                     /transl_table=11
                     /fullproduct="qcoverage 100 , hcoverage 100 , similarity
                     100 , identity 100 , evalue 2.87e-206 , alnlength 274 ,
                     Bacteria , Proteobacteria , WP_015589449.1 MULTISPECIES:
                     oxygen-dependent coproporphyrinogen oxidase [unclassified
                     Wolbachia]"
                     /cog="HemF COG0408 303 Coproporphyrinogen III oxidase"
                     /pfam="Coprogen_oxidas"
                     /product="oxygen-dependent
                     coproporphyrinogen oxidase"
                     /translation="MEKQRTQAFKWFCALRDKIIESFLLIEKQSSAEPKIEKRKWDRP
                     GGGGGESTIIFGNVFEKVGVNVSKVHGKFADSAINEIPGASESNGEFWASGISLVSHM
                     QSPLIPAAHMNTRLIYTSKQWFGGGMDFTPIYRNEEDCKYIHESIKMTCDRFDTGYYP
                     KFKEQCDNYFFLQHRKEPRGIGGIFYDNLSSGNWENDFEFTKAVGEAFLEIYSHVIRK
                     HMQKSWTKEQRENQLIKRGRYVEFNLLYDRGTRFGLMTDGNPDAIMMSMPPLVKWL"
                     /besthit="qcoverage 100 , hcoverage 100 , similarity 100
                     , identity 100 , evalue 2.87e-206 , alnlength 274 ,
                     Bacteria , Proteobacteria , WP_015589449.1 MULTISPECIES:
                     oxygen-dependent coproporphyrinogen oxidase [unclassified
                     Wolbachia]"
     gene            5076..6320
                     /locus_tag="wEsol_00528"
     CDS             5076..6320
                     /locus_tag="wEsol_00528"
                     /codon_start=1
                     /transl_table=11
                     /pfam="Ank_2 CagA"
```

/fullproduct="qcoverage 100 , hcoverage 100 , similarity 100 , identity 100 , evalue 1.11e-253 , alnlength 414 , Bacteria , Proteobacteria , WP_015589448.1 MULTISPECIES: hypothetical protein [unclassified Wolbachia]"
/translation="MIVSFYIGGIIMGIIDNFLIKSVKNNDIKGVKLTLILYDALNSF
RKFFSKTMSLKNTGSFDINNNQALNQALGHATDHKIMELLINRGADIHSIDENGKNVI
IRVIESLAESKNLKLDSEKNLPAEIILHPTVSFLLGKGAEYYSTDDLNVEAIQQKPPR
TSEYEPITMYAEVNNNVIPPQKPSRTFEYKPKSTNYDALPSKEPIYAEVYDAKVSNSL
GENGYLSVSEESIYAEIYDSHKKSTENLSLEDSGYLSISEEPIYCTIDDLSEQKKPAS
LKQKEPEQSSEPIYARVDLSKKRAERKAKDIKANSGTCHGLVQDKVNDWQVKLNELSA
NKSISQQQTNDQKIKTKAGFSVEEIRQKYERRDSGYDSGYDSDTKVEQQLELAPKSEI
GSVSIEHGVIGGCRYKITDISL"
/product="hypothetical protein"
/besthit="qcoverage 100 , hcoverage 100 , similarity 100 , identity 100 , evalue 1.11e-253 , alnlength 414 , Bacteria , Proteobacteria , WP_015589448.1 MULTISPECIES: hypothetical protein [unclassified Wolbachia]"

gene 6489..6890
/locus_tag="wEsol_00529"

CDS 6489..6890
/locus_tag="wEsol_00529"
/codon_start=1
/transl_table=11
/product="16S rRNA (uracil(1498)-N(3))-methyltransferase"
/pfam="Methyltrans_RNA"
/tigr="TIGR00046 TIGR00046 240 RNA methyltransferase, RsmE family"
/translation="MGKIRLYVEEALSQGVSLALNPQQSHYICNVMRLKKYDNLSLFN
GKDGEWLGEVVNISRKLTKITLKECAKQQQYEENLYLYCAMVKSAALNNIVRQATEMG
ITCIQFISTERTVVKNINLSRAKLQAIEAAE"
/cog="COG1385 COG1385 246 Uncharacterized protein conserved in bacteria"
/besthit="qcoverage 100 , hcoverage 58.3333333333333 , similarity 100 , identity 100 , evalue 2.92e-87 , alnlength 133 , Bacteria , Proteobacteria , WP_015589447.1 MULTISPECIES: 16S rRNA (uracil(1498)-N(3))-methyltransferase [unclassified Wolbachia]"

gene 6900..7175
/locus_tag="wEsol_00530"

CDS 6900..7175
/locus_tag="wEsol_00530"
/codon_start=1
/transl_table=11
/product="16S rRNA (uracil(1498)-N(3))-methyltransferase"
/pfam="Methyltrans_RNA"
/tigr="TIGR00046 TIGR00046 240 RNA methyltransferase, RsmE family"
/translation="MSIPEILPPINFCELPDSQSKNFVLCDETGKADKVLKGKKNVAI
IVGPEGGFSSYELDLADKFCQKLSLGKRILRVDTAVVAALTFTNWCS"
/cog="COG1385 COG1385 246 Uncharacterized protein conserved in bacteria"
/besthit="qcoverage 100 , hcoverage 79.1304347826087 , similarity 100 , identity 98.9 , evalue 1.05e-58 , alnlength 91 , Bacteria , Proteobacteria , WP_146038223.1 RsmE family RNA methyltransferase, partial [Wolbachia sp. wMel_KL]"

gene 7658..7858
/locus_tag="wEsol_00531"

CDS 7658..7858

```
                     /locus_tag="wEsol_00531"
                     /codon_start=1
                     /transl_table=11
                     /fullproduct="qcoverage 100 , hcoverage 100 , similarity
                     100 , identity 100 , evalue 1.34e-27 , alnlength 66 ,
                     Bacteria , Proteobacteria , WP_006279819.1 MULTISPECIES:
                     30S ribosomal protein S21 [Wolbachia]"
                     /cog="RpsU COG0828 67 Ribosomal protein S21"
                     /pfam="Ribosomal_S21"
                     /tigr="TIGR00030 S21p 58 ribosomal protein S21"
                     /product="30S ribosomal
                     protein S21"
                     /translation="MIEVSVHYGDVDRALPVLKKTIQKEGRGLKMKKQYHEKKSEKRA
                     KKKAEAKKKRYQQECRRQRYGW"
                     /besthit="qcoverage 100 , hcoverage 100 , similarity 100
                     , identity 100 , evalue 1.34e-27 , alnlength 66 ,
                     Bacteria , Proteobacteria , WP_006279819.1 MULTISPECIES:
                     30S ribosomal protein S21 [Wolbachia]"
     gene            7896..9047
                     /locus_tag="wEsol_00532"
     CDS             7896..9047
                     /locus_tag="wEsol_00532"
                     /codon_start=1
                     /transl_table=11
                     /fullproduct="qcoverage 100 , hcoverage 100 , similarity
                     100 , identity 100 , evalue 8.51e-263 , alnlength 383 ,
                     Bacteria , Proteobacteria , WP_015589445.1 MULTISPECIES:
                     ADP-forming succinate--CoA ligase subunit beta
                     [unclassified Wolbachia]"
                     /cog="SucC COG0045 387 Succinyl-CoA synthetase, beta
                     subunit"
                     /pfam="ATP-grasp_2 ATP-grasp_5 GARS_A Ligase_CoA"
                     /tigr="TIGR01016 sucCoAbeta 386 succinate-CoA ligase,
                     beta subunit"
                     /product="ADP-forming
                     succinate--CoA ligase subunit beta"
                     /translation="MNIHEYQAKEVLHKFNVPVPKGFVATSAEEVKTQVSQLKSDVFV
                     VKAQIHAGGRGKAGGVKLAKSVEEAQQFVKDMIGMTLVTHQTGPSGQQVRKVYVEEGS
                     SIKKEYYLSLVIDPKLSRPIFIFSSEGGMDIEEVAKNSPAKIVKFDIDSATSFDSSKL
                     SSSFDLSPEQIEKISNIAKKIYDAFIATDASQIEINPLVETNSGDFIALDAKINFDDN
                     ALYRHPEIMELRDYDEEVKEEIEASKYGLSYIKMDGSIGCMVNGAGLAMATMDIIKYY
                     GAEPANFLDVGGGASKETVTEAFKIILSDSNVKGILVNIFGGIMRCDIIASGIVAAAK
                     EMSIKVPLVVRLSGTNFEEGKKILEESGLNIIAADELGDAAQKIVKEVK"
                     /besthit="qcoverage 100 , hcoverage 100 , similarity 100
                     , identity 100 , evalue 8.51e-263 , alnlength 383 ,
                     Bacteria , Proteobacteria , WP_015589445.1 MULTISPECIES:
                     ADP-forming succinate--CoA ligase subunit beta
                     [unclassified Wolbachia]"
     gene            9051..9926
                     /locus_tag="wEsol_00533"
     CDS             9051..9926
                     /locus_tag="wEsol_00533"
                     /codon_start=1
                     /transl_table=11
                     /fullproduct="qcoverage 100 , hcoverage 100 , similarity
                     100 , identity 100 , evalue 2.96e-203 , alnlength 291 ,
                     Bacteria , Proteobacteria , WP_015589444.1 MULTISPECIES:
                     succinate--CoA ligase subunit alpha [unclassified
                     Wolbachia]"
                     /cog="SucD COG0074 293 Succinyl-CoA synthetase, alpha
```

subunit"
/pfam="CoA_binding Succ_CoA_lig Ligase_CoA"
/tigr="TIGR01019 sucCoAalpha 286 succinate-CoA ligase, alpha subunit"
/product="succinate--CoA ligase subunit alpha"
/translation="MSVLVNKDTRLICQGFTGAQGTFHSEQAIDYGTKMVGGVTPGKG
GSTHLNLPVFNTVAEAKEKTGANATVIYVPAKFAAAAILEAIDAKIELIICITEGIPI
LDMVKVKRALIGSKSRLIGPNCPGIITPEECKIGIMPGHIHKRGHIGIMSRSGTLTYE
AVAQTTAVGLGQSTCIGIGGDPIHGMTFVDCMELFLKDDDTHGIVVIGEIGGNEEEDV
SHFVKTEKTKKPIVGFVAGQTAPPGRRMGHAGAIISSSGGSAGAKLEVMRSAGIAIAE
TPAVIGKKVLEVMHH"
/besthit="qcoverage 100 , hcoverage 100 , similarity 100 , identity 100 , evalue 2.96e-203 , alnlength 291 , Bacteria , Proteobacteria , WP_015589444.1 MULTISPECIES: succinate--CoA ligase subunit alpha [unclassified Wolbachia]"

gene complement(9935..10726)
/locus_tag="wEsol_00534"

CDS complement(9935..10726)
/locus_tag="wEsol_00534"
/codon_start=1
/transl_table=11
/fullproduct="qcoverage 100 , hcoverage 100 , similarity 100 , identity 100 , evalue 5.09e-190 , alnlength 263 , Bacteria , Proteobacteria , AOV87829.1 diaminopimelate epimerase [Wolbachia endosymbiont of Drosophila incompta]"
/cog="DapF COG0253 272 Diaminopimelate epimerase"
/pfam="DAP_epimerase DAP_epimerase"
/tigr="TIGR00652 DapF 270 diaminopimelate epimerase"
/product="diaminopimelate epimerase"
/translation="MMSGQNSENFIKMHGTGNNFVIIDSRSTNNLDWNHRQIADQSSC
DQVIIITMSNAADCFMHIYNADGSRAEMCGNAARCVGYLIMSEKGAEYSIIELTSKRI
LECFKVGDKSIKVNMGKPLLKWHEIPLSTECDTLHLPIELEMLKDPVAVNIGNPHIVF
FVDNISEIPLRNLGPKLENHALFPRKINVSIAQVEKSGEIALRVWERGTGITASCGSA
ACAALVASTLRGYLVAQQTSVNLPGGKLLIEWANNVFMTGDIGFL"
/besthit="qcoverage 100 , hcoverage 100 , similarity 100 , identity 100 , evalue 5.09e-190 , alnlength 263 , Bacteria , Proteobacteria , AOV87829.1 diaminopimelate epimerase [Wolbachia endosymbiont of Drosophila incompta]"

gene complement(10704..11291)
/locus_tag="wEsol_00535"

CDS complement(10704..11291)
/locus_tag="wEsol_00535"
/codon_start=1
/transl_table=11
/fullproduct="qcoverage 100 , hcoverage 100 , similarity 100 , identity 100 , evalue 9.30e-141 , alnlength 195 , Bacteria , Proteobacteria , WP_006279823.1 MULTISPECIES: nitroreductase family protein [Wolbachia]"
/cog="NfnB COG0778 207 Nitroreductase"
/pfam="Nitroreductase"
/tigr="TIGR02476 BluB 205 5,6-dimethylbenzimidazole synthase"
/product="nitroreductase family protein"
/translation="MNYLCTIKLKMMSKQDLLSLMKARHSGRSYDPTRAISQEDMDIL
MEAVRLTPSCFGDEPWRYVICNKQNNQSAWEKLLNCLDESNQKWAKDAQVLIISLSAK

NFRKQGKGENLWANHDTGAANYGLMLQAAVMNLMAHQMGGFDRDKIVKEFNIPNDFNV
MSVIAVGYEEEGAETSKKKRRPIEEIFFYDEWPKF"
/besthit="qcoverage 100 , hcoverage 100 , similarity 100
, identity 100 , evalue 9.30e-141 , alnlength 195 ,
Bacteria , Proteobacteria , WP_006279823.1 MULTISPECIES:
nitroreductase family protein [Wolbachia]"

```
gene            11377..11904
                /locus_tag="wEsol_00536"
CDS             11377..11904
                /locus_tag="wEsol_00536"
                /codon_start=1
                /transl_table=11
                /fullproduct="qcoverage 100 , hcoverage 100 , similarity
                100 , identity 100 , evalue 6.38e-111 , alnlength 175 ,
                Bacteria , Proteobacteria , WP_015589442.1 MULTISPECIES:
                biotin transporter BioY [Wolbachia]"
                /cog="BioY COG1268 184 Uncharacterized conserved protein"
                /pfam="BioY"
                /product="biotin transporter
                BioY"
                /translation="MFITQSSSRSTLAEILSCVLLLFLMAQISIPLQPVPITLQTLGV
                MLIGLKFNRRTAFYSVLTYLSLGAAGLPVLANFSGGYHALLGPTGGYLIGCLAAVMVM
                SKVNELLNSKYKSFVCNSLSCLAGTVVIFICGVSWLAVYLGLGQAIMVGVLPFILPGL
                IKIFLLVAALQYLKK"
                /besthit="qcoverage 100 , hcoverage 100 , similarity 100
                , identity 100 , evalue 6.38e-111 , alnlength 175 ,
                Bacteria , Proteobacteria , WP_015589442.1 MULTISPECIES:
                biotin transporter BioY [Wolbachia]"
gene            11928..12290
                /locus_tag="wEsol_00537"
CDS             11928..12290
                /locus_tag="wEsol_00537"
                /codon_start=1
                /transl_table=11
                /fullproduct="qcoverage 100 , hcoverage 100 , similarity
                100 , identity 100 , evalue 5.85e-85 , alnlength 120 ,
                Bacteria , Proteobacteria , WP_227738570.1 DUF1284
                domain-containing protein [Wolbachia endosymbiont of
                Drosophila simulans]"
                /cog="COG3543 COG3543 135 Uncharacterized conserved
                protein"
                /pfam="DUF1284"
                /product="DUF1284 domain-containing
                protein"
                /translation="MCTLAFRGYGYSQGFVENYKRIASKVISTQIEVVGNLDNICSAC
                PNQTKQGKCTTQAKVLELDRRHMEILGIKIGETLTWNEAVKKIREKMSLKKFEYACEG
                CNWQPYGICKNALLNLQR"
                /besthit="qcoverage 100 , hcoverage 100 , similarity 100
                , identity 100 , evalue 5.85e-85 , alnlength 120 ,
                Bacteria , Proteobacteria , WP_227738570.1 DUF1284
                domain-containing protein [Wolbachia endosymbiont of
                Drosophila simulans]"
gene            12459..13334
                /locus_tag="wEsol_00538"
CDS             12459..13334
                /locus_tag="wEsol_00538"
                /codon_start=1
                /transl_table=11
                /pfam="Pfam-B_3485 Pfam-B_1969 Pfam-B_333 Pfam-B_10017
                Pfam-B_3875 TPR_16 TPR_18 Pfam-B_121 YfiO Tcf25 TPR_11
```

TPR_12 Pfam-B_1822 Pfam-B_1799 Pfam-B_392 Apc3 TPR_8
TPR_2 TPR_1 TPR_7 TPR_6 TPR_14 TPR_11 Pfam-B_333
Pfam-B_8853 TPR_11 TPR_12 TPR_1 TPR_8 TPR_2"
/fullproduct="qcoverage 100 , hcoverage 100 , similarity
100 , identity 100 , evalue 5.11e-204 , alnlength 291 ,
Bacteria , Proteobacteria , WP_174516848.1 MULTISPECIES:
tetratricopeptide repeat protein [unclassified
Wolbachia]"
/translation="MLRIAQRNKVYWLHAAANATTLGPMFICMLICVVILWRSSVTYA
SENLEIQKAFDSVVKHIKADKKYKDFDVIERKSDKFNIKISQNSGKSFDIYSTLKKAK
DSFESGDNEAAISLLNQIIAKFPYHKNALIGLGNIYYANKEYKKAVEIYTRLLKEHPS
NPYILKNFLTIISQYDPDLALSEMLKLYDIHKNYAPLLANLGLIYMKKEDYVKGKEYM
ITAISLDENNIFYTYNLAVILDKLSDFQNATIFYLKLLNMATTSKDVSEKIPLYKVTA
RLKFIKLHSTHPKIS"
/product="tetratricopeptide
repeat protein"
/besthit="qcoverage 100 , hcoverage 100 , similarity 100
, identity 100 , evalue 5.11e-204 , alnlength 291 ,
Bacteria , Proteobacteria , WP_174516848.1 MULTISPECIES:
tetratricopeptide repeat protein [unclassified
Wolbachia]"

gene 14300..17008
/locus_tag="wEsol_00539"

CDS 14300..17008
/locus_tag="wEsol_00539"
/codon_start=1
/transl_table=11
/fullproduct="qcoverage 100 , hcoverage 100 , similarity
99.8 , identity 99.8 , evalue 0.0 , alnlength 902 ,
Bacteria , Proteobacteria , WP_141456691.1 MULTISPECIES:
DNA gyrase subunit A [unclassified Wolbachia]"
/cog="GyrA COG0188 804 Type IIA topoisomerase (DNA
gyrase/topo II, topoisomerase IV), A subunit"
/pfam="DNA_topoisoIV DNA_gyraseA_C DNA_gyraseA_C
DNA_gyraseA_C DNA_gyraseA_C DNA_gyraseA_C DNA_gyraseA_C"
/tigr="TIGR01063 gyrA 800 DNA gyrase, A subunit"
/product="DNA gyrase subunit
A"
/translation="MQDNIVPISIVKELEDSYLSYAMSVIISRAIPDVRDGFKPVHRR
ILYAMSRAGFDAGKPYKKAARIVGDVMGKYHPHGDAAIYDSLVRMAQDFSLLLPLVDG
QGNFGSIDGDPPASMRYTEARLQKVSHFLLNDIDEDTVNFRPNYDGNETEPVVLPAEF
PNLLVNGASGVAVGMATNIPSHNLGEIIDACILYVNNPEVTLDELLEVMPGPDFPTGG
TILGRSGIRSAFATGRGSIVVQGKTHIEDLPQDRQAIVIDEIPYQVNKVKLIEKIGEL
VKEKRIDGITEIRDESDKSGIRVVIDLKRNAEASFILNQILGLTPLRSSFSVNTLVLN
NNRPALMSLKEIIEAFIDFRKEVLIRRTEFRLRKTREKAHIYIGLYIAVLSIDEVIKI
IRGAKDPAEASRELLNKEWKTSNEINTIIELISDSASFLKDGVYRLTELQIKAILDMK
LQRLTGLEKDKLEAELSSMINLIKEYIAFLGSGEKLMKEIKNNLQEIKNRFAVPRKTS
IEESDVDIEAEDLIPQEDMVITVTMNGYIKRVKLSHYRTQRRGGKGKLGQGLKEEDVI
TELFVGNTHTSFLFFSNIGRVYRLKVYKLPLAEPTARGRALVNIFPLSDGETITNIMP
LPSENDENQNIVFATAYGNIRRNSLADFHYIPSNGKIAIKLDEGDKLVSVKVCNEIDH
VLLSTTLGKSIRFVVSDVRQFKSRNSDGVRGIKLVKNDSVISMTILNGIGVTTETKEL
YLKVPLAKRLGTAINNSIDSKLEKTLNDLGIDNELFLKLAVNEEFILTITENGFGKRT
SAYEYRVTNRGGVGITNILTTSRNGNVVASFPVEQGDNIMLITDKGKLIRISVNEIRI
AGRSTQGVTLFKTESREKVVSVAKIEDPDSTEDSIFEVENSISSQP"
/besthit="qcoverage 100 , hcoverage 100 , similarity 99.8
, identity 99.8 , evalue 0.0 , alnlength 902 , Bacteria ,
Proteobacteria , WP_141456691.1 MULTISPECIES: DNA gyrase
subunit A [unclassified Wolbachia]"

gene 17095..17694
/locus_tag="wEsol_00540"

```
CDS             17095..17694
                /locus_tag="wEsol_00540"
                /codon_start=1
                /transl_table=11
                /fullproduct="qcoverage 100 , hcoverage 100 , similarity
                100 , identity 100 , evalue 2.27e-143 , alnlength 199 ,
                Bacteria , Proteobacteria , WP_015589439.1 MULTISPECIES:
                ubiquinol-cytochrome c reductase iron-sulfur subunit
                [unclassified Wolbachia]"
                /cog="QcrA COG0723 177 Rieske Fe-S protein"
                /pfam="UCR_Fe-S_N Rieske"
                /tigr="TIGR01416 Rieske_proteo 176 ubiquinol-cytochrome c
                reductase, iron-sulfur subunit"
                /product="
                ubiquinol-cytochrome c reductase iron-sulfur subunit"
                /translation="MDKNLTKNKKPLANKEDKKTPPVKDLTKNKSRRDFITLTTCAMA
                GIGAASGLWPLVKSMNPSAEVLATSTVEVNLSGIQEGHGVKVKWQGKPVFIRRRTKQE
                IEAARAVNVKSLRDPQLDEQRVYEGKDEWLIMLGICTHLGCVPVDHATRDGNGWFCPC
                HGSYYDTSGRVIGGPAPKNMAVPDYFFPSENVVVIGKKA"
                /besthit="qcoverage 100 , hcoverage 100 , similarity 100
                , identity 100 , evalue 2.27e-143 , alnlength 199 ,
                Bacteria , Proteobacteria , WP_015589439.1 MULTISPECIES:
                ubiquinol-cytochrome c reductase iron-sulfur subunit
                [unclassified Wolbachia]"
gene            complement(17704..20046)
                /locus_tag="wEsol_00541"
CDS             complement(17704..20046)
                /locus_tag="wEsol_00541"
                /codon_start=1
                /transl_table=11
                /pfam="Pfam-B_4868 ResIII DEAD AAA_19 Pfam-B_14354
                Pfam-B_4868 Pfam-B_19902 Pfam-B_4868 Helicase_C"
                /fullproduct="qcoverage 100 , hcoverage 100 , similarity
                99.9 , identity 99.9 , evalue 0.0 , alnlength 780 ,
                Bacteria , Proteobacteria , WP_015589438.1 MULTISPECIES:
                primosomal protein N' [unclassified Wolbachia]"
                /translation="MLNSITMAKTVDVLLSLPIDQLFSYAIEENTEILLGDYVVVPFG
                KKRLIGIVWKYSDKSNRELKFIEQKIDLPNIRTKLIAFAEWVAQYNLIPIGMLAKVIM
                GGVLKVNHIDKLVCVEQKQETSEISCQLSPEQQIASDKIISNLNEYSVTLLDGETGSG
                KTEVYLSVIAQLIKNYTAVPNAAQTLPSQCPETQLYEHCDLGLPRGCYPSSLTLGSSF
                SYNLITNVYFNLKPIPNLQSKFLDSSVKHWNDTFILKAKPTVTFSIPSLPRTSMTPEY
                VEMTSGEGNTQILILLPEIVLTSQLVNRVRGQISKNLVEWHSGLTPKTRRNNWLNIAN
                GNAQIIIGARSALFLPYKNLKLIIVDEEHDSSFKQEQGIIYNARDMAIILAKFENIPI
                ILSSATPLLETIHHVKNGNYNHVKLTKRFGGAELPLIKVVDMRNNKQWISSELFESIK
                QTIEKKQQVMLFLNRRGYAQLAICKKCGYKISCLNCAVWLTYHKKKNALLCHHCSYQL
                KLPEKCSNCQSEQPLSLYGIGIERLLEEMVKLIPNAKTAMISSDQKSVSNIIDLVLKE
                EVNIIIGTQIIAKGHNFPKLTLVGVINADLSLENSDLRAAEKTYQLLHQVAGRSGRFN
                EKGMVIVQTNNPESSIIKALLHQKRDSFYEIELKSRREAKMPPFSRLIALIICGKNQI
                ATQKAANEIVKSLLCYPSSLTTCIQEKNVGVDKKEFEILGPSPAAINFLNNKYRHRVL
                LKIHNKHSLSIQKKLKYWIKNCNLNSSIAVIIDVDPVSFF"
                /product="primosomal protein
                N'"
                /besthit="qcoverage 100 , hcoverage 100 , similarity 99.9
                , identity 99.9 , evalue 0.0 , alnlength 780 , Bacteria ,
                Proteobacteria , WP_015589438.1 MULTISPECIES: primosomal
                protein N' [unclassified Wolbachia]"
gene            20191..21975
                /locus_tag="wEsol_00542"
CDS             20191..21975
                /locus_tag="wEsol_00542"
```

```
                     /codon_start=1
                     /transl_table=11
                     /pfam="Pfam-B_150"
                     /fullproduct="qcoverage 100 , hcoverage 100 , similarity
                     100 , identity 100 , evalue 0.0 , alnlength 594 ,
                     Bacteria , Proteobacteria , WP_141456689.1 MULTISPECIES:
                     hypothetical protein [unclassified Wolbachia]"
                     /translation="MVNDKITLNFEIEKGEPIDRYYFSAHIKLTDESKNLLREKLGST
                     IQFDNLDDHFNLNHENFYIYYNKYSEHNGRSELHVRDDNGKYLTSNLPLNYYGFSMEL
                     SPDGASIADYGFYPLYAIYNPNHYNKAKEKVFDDHGELSPIASNVLDNFLSNTNSLTT
                     QDILQNVFSQLQKKADEEAERLAREREAERQREEELNEKNEELKELVEKDEIKIFKVV
                     KGNDLGNGHSAVVLQLDGEEGDKIPLDSSKYYIAEYRHHPEWEGDEYLNFVSPDSDML
                     VNYENLFFVLNQSSYSDFESGFYSENRTDPHNYAKYSKEPYLSGDQLSEKLSNELEED
                     SNQIGTEFTIDDEQVDEEVVVESEAKSGEKGTVGEDNAKNTIQEEESNYIDQMQADDS
                     SSYKPALQSNRDDYADTKKALQEKLHGDEIHKQSNEKAQKDIEVKKVTVETDSSSNKT
                     EKQTKPTEDEQETVMASVIENMDKNSDIEAETSNSVANSQHPLKIKTGSPLEVGKYKV
                     ELQVTYDDVKNFYDTVRGKYHAGDGYNYEQVMVLYNEIVRPAHNHHYELFTLDEAHVA
                     NDHLFIKGQDFGTLNDFNEMFYKSDELM"
                     /product="hypothetical protein"
                     /besthit="qcoverage 100 , hcoverage 100 , similarity 100
                     , identity 100 , evalue 0.0 , alnlength 594 , Bacteria ,
                     Proteobacteria , WP_141456689.1 MULTISPECIES:
                     hypothetical protein [unclassified Wolbachia]"
     gene            complement(22048..22902)
                     /locus_tag="wEsol_00543"
     CDS             complement(22048..22902)
                     /locus_tag="wEsol_00543"
                     /codon_start=1
                     /transl_table=11
                     /fullproduct="qcoverage 100 , hcoverage 100 , similarity
                     100 , identity 100 , evalue 7.29e-205 , alnlength 284 ,
                     Bacteria , Proteobacteria , WP_015589436.1 MULTISPECIES:
                     FAD-dependent thymidylate synthase [Wolbachia]"
                     /cog="THY1 COG1351 273 Predicted alternative thymidylate
                     synthase"
                     /pfam="Thy1"
                     /tigr="TIGR02170 thyX 211 thymidylate synthase,
                     flavin-dependent"
                     /product="FAD-dependent
                     thymidylate synthase"
                     /translation="MNEATKRTIVKEIDAILYEEHKVLDHGFIRVVDYMGSDSAIVQA
                     ARVSYGKGTKQISQDEALIKYLMRHHHTTPFEMCEIKFHVKLPIFVARQWIRHRTANV
                     NEYSARYSILDNEFYTPKPEQVAKQSDNNKQGSGEAFDPDTSKEIIDSLTNDSNLVYS
                     HYEKFIEQGLAREIARTNLMLNYYTQFYWKIDLHNLLHFLKLRADKHAQYEIRVYAEV
                     MLDIIKKWVPLAYNAFVEYCLESACISRTGLEIIRKLIKGENVTREESNIGKREWGEL
                     MSILDKQS"
                     /besthit="qcoverage 100 , hcoverage 100 , similarity 100
                     , identity 100 , evalue 7.29e-205 , alnlength 284 ,
                     Bacteria , Proteobacteria , WP_015589436.1 MULTISPECIES:
                     FAD-dependent thymidylate synthase [Wolbachia]"
     gene            complement(22912..24189)
                     /locus_tag="wEsol_00544"
     CDS             complement(22912..24189)
                     /locus_tag="wEsol_00544"
                     /codon_start=1
                     /transl_table=11
                     /fullproduct="qcoverage 100 , hcoverage 100 , similarity
                     100 , identity 100 , evalue 1.86e-301 , alnlength 425 ,
                     Bacteria , Proteobacteria , WP_006279833.1 MULTISPECIES:
                     UDP-N-acetylglucosamine 1-carboxyvinyltransferase
```

```
                     [Wolbachia]"
                     /cog="MurA COG0766 421 UDP-N-acetylglucosamine
                     enolpyruvyl transferase"
                     /pfam="EPSP_synthase"
                     /tigr="TIGR01072 murA 416 UDP-N-acetylglucosamine
                     1-carboxyvinyltransferase"
                     /product="
                     UDP-N-acetylglucosamine 1-carboxyvinyltransferase"
                     /translation="MHKILIRNNYKPLVGKIKINGSKNAVLPIMAASLLSSSPVILHN
                     VPDLIDVHLMSKLLESLGAEVNFMHNKNYKANHTLKIDCSNINNHVMPYKTASKLRTS
                     FLILGPMLSRFGKARTAFPGGCNIGKRPVDMHIKALEEMGAKIEIDGYNIIATVKGKL
                     QGKEITFEKISVGATENVIMAATFAEGVTTINNAATEPEVLDLIDFLKKMGADIEIDN
                     TKVIITGVEALNGCVHKIIPDRIEAGTYALAAIITGGKLELEGINLSDIRCITNELET
                     IGAMVELYDGGIVISRKNGSIKSANVATDPYPNFPSDMQPQLMSAMCIADGISVIEEN
                     IFENRFTHADELRKLGANISIEKSKATISGIKSLSGANLYATDLRSTAALVLASLVAG
                     GETIINNSHHLWRGYEAMHEKLNSCGADISISS"
                     /besthit="qcoverage 100 , hcoverage 100 , similarity 100
                     , identity 100 , evalue 1.86e-301 , alnlength 425 ,
                     Bacteria , Proteobacteria , WP_006279833.1 MULTISPECIES:
                     UDP-N-acetylglucosamine 1-carboxyvinyltransferase
                     [Wolbachia]"
     gene            complement(24345..25181)
                     /locus_tag="wEsol_00545"
     CDS             complement(24345..25181)
                     /locus_tag="wEsol_00545"
                     /codon_start=1
                     /transl_table=11
                     /fullproduct="qcoverage 100 , hcoverage 100 , similarity
                     100 , identity 100 , evalue 5.28e-195 , alnlength 278 ,
                     Bacteria , Proteobacteria , WP_010963091.1 MULTISPECIES:
                     CPBP family intramembrane metalloprotease [Wolbachia]"
                     /cog="COG1266 COG1266 226 Predicted metal-dependent
                     membrane protease"
                     /pfam="Abi"
                     /product="CPBP family
                     intramembrane metalloprotease"
                     /translation="MGVCMNLTTINCTLFVVITYFLTCIIAWCLPFKDITLLTTFVPA
                     SVAILLTIYNKQKVRDLFKLSSPKNCLLGFILILVSMLISNGLLSIYCGFSFFRESFT
                     LDRVKDLSAVLSPLVMLFVLLSTFVMWIIMSLGEEIGWRGYLLKNLKSKISNFYIRAI
                     IVGIIWSVWHIPTYVVAGSALWKDGFTFPTICAYTLYICAMSIMFTWLFEKDNSIWPV
                     TIAHATNNLVYSVLSILMPSPPLSTFDAVIRLTLGAAGYFIVAIGVIWFDKARERHLS
                     NS"
                     /besthit="qcoverage 100 , hcoverage 100 , similarity 100
                     , identity 100 , evalue 5.28e-195 , alnlength 278 ,
                     Bacteria , Proteobacteria , WP_010963091.1 MULTISPECIES:
                     CPBP family intramembrane metalloprotease [Wolbachia]"
     gene            complement(25421..26692)
                     /locus_tag="wEsol_00546"
     CDS             complement(25421..26692)
                     /locus_tag="wEsol_00546"
                     /codon_start=1
                     /transl_table=11
                     /fullproduct="qcoverage 100 , hcoverage 100 , similarity
                     100 , identity 100 , evalue 8.96e-308 , alnlength 423 ,
                     Bacteria , Proteobacteria , WP_015589434.1 MULTISPECIES:
                     beta-ketoacyl-ACP synthase II [unclassified Wolbachia]"
                     /cog="FabB COG0304 412 3-oxoacyl-(acyl-carrier-protein)
                     synthase"
                     /pfam="ketoacyl-synt Thiolase_N Ketoacyl-synt_C"
                     /tigr="TIGR03150 fabF 407
```

beta-ketoacyl-acyl-carrier-protein synthase II"
/product="beta-ketoacyl-ACP
synthase II"
/translation="MSRRVVVTGVGLITPLAADVDNTWLRLIKGESGIKAINTDRFDS
SDLACKIAGQVPLQSDNIEHHFNPLDYISEKDSKRTDRFIHYGIAAAIQAVDDSLILE
SQNINRERVGVTIGSGIGGLPSIQENVITMQEKGPRRVSPFFVPASLINLISGHISIK
YEFTGPNDSAVTACATGAHAIINSARTIKLGEADVMIAGGAESALCRVGIAGFASMKA
LSTKFNDKPKEASRPWDAERDGFVMGEGAGILVLEEYEHAKKRGAKIYAELNGYGLTG
DAHHITAPHPEGRGAFKAMQLALHSAQINPNQVGYINAHGTSTPLGDKIEVIAMKKLF
GDYAYKIPVSSTKSSTGHLLGAAGSVEAIFSILALNNGIIPPTLNLHKPSEGCDLNFV
PFKAQEHKIQYALSNSFGFGGTNASLIFGQV"
/besthit="qcoverage 100 , hcoverage 100 , similarity 100
, identity 100 , evalue 8.96e-308 , alnlength 423 ,
Bacteria , Proteobacteria , WP_015589434.1 MULTISPECIES:
beta-ketoacyl-ACP synthase II [unclassified Wolbachia]"
gene complement(26706..26978)
/locus_tag="wEsol_00547"
CDS complement(26706..26978)
/locus_tag="wEsol_00547"
/codon_start=1
/transl_table=11
/fullproduct="qcoverage 100 , hcoverage 100 , similarity
100 , identity 100 , evalue 8.01e-51 , alnlength 90 ,
Bacteria , Proteobacteria , WP_007549723.1 MULTISPECIES:
acyl carrier protein [Wolbachia]"
/cog="AcpP COG0236 80 Acyl carrier protein"
/pfam="Pfam-B_5835 Pfam-B_10478 PP-binding Pfam-B_17829
Ribosomal_L50"
/tigr="TIGR00517 acyl_carrier 77 acyl carrier protein"
/product="acyl carrier
protein"
/translation="MVRNVNSELAKSTREDIEEKVKKIILEHISKDVEKFNSSSKLSE
HGTDSLDAVEIIMAAEEEFGIEIPDEDAQKMETMEQIVEYINNKKG"
/besthit="qcoverage 100 , hcoverage 100 , similarity 100
, identity 100 , evalue 8.01e-51 , alnlength 90 ,
Bacteria , Proteobacteria , WP_007549723.1 MULTISPECIES:
acyl carrier protein [Wolbachia]"
gene 27112..27903
/locus_tag="wEsol_00548"
CDS 27112..27903
/locus_tag="wEsol_00548"
/codon_start=1
/transl_table=11
/fullproduct="qcoverage 100 , hcoverage 100 , similarity
100 , identity 100 , evalue 2.91e-180 , alnlength 263 ,
Bacteria , Proteobacteria , WP_141456688.1 MULTISPECIES:
polyprenyl synthetase family protein [unclassified
Wolbachia]"
/cog="IspA COG0142 322 Geranylgeranyl pyrophosphate
synthase"
/pfam="polyprenyl_synt"
/tigr="TIGR02748 GerC3_HepT 319 heptaprenyl diphosphate
synthase component II"
/product="polyprenyl
synthetase family protein"
/translation="MLDETTKNLLIVEVNKLLPENSENKLISAMRYILLAPAKHIRSF
LVIASSQVFNAEAKKVISVAAAIEFVHAYSLIHDDLPCMDNSDTRRSQLSCHKKFDEA
TAVLAGDALLTLAFEVLSSLNEKRCEIIKVLSQAIGIRGMVGGQALDIDTDFNKIKEI
HLMKTAKLFAASCEIGAIIGGATDEQRRALYNYGINLGLIFQAKDDIEDYEQDKTNNL
MSVLGKSEVEDYIDGLFKQGLDNLSALSGDTNYLYDLLNQVKKDG"

```
                     /besthit="qcoverage 100 , hcoverage 100 , similarity 100
                     , identity 100 , evalue 2.91e-180 , alnlength 263 ,
                     Bacteria , Proteobacteria , WP_141456688.1 MULTISPECIES:
                     polyprenyl synthetase family protein [unclassified
                     Wolbachia]"
     gene            27896..28837
                     /locus_tag="wEsol_00549"
     CDS             27896..28837
                     /locus_tag="wEsol_00549"
                     /codon_start=1
                     /transl_table=11
                     /pfam="FKBP_C FKBP_C"
                     /fullproduct="qcoverage 100 , hcoverage 100 , similarity
                     100 , identity 100 , evalue 1.48e-214 , alnlength 313 ,
                     Bacteria , Proteobacteria , WP_015589432.1 MULTISPECIES:
                     FKBP-type peptidyl-prolyl cis-trans isomerase
                     [unclassified Wolbachia]"
                     /translation="MVRKIILQMLISVVMILTLTSAFVFTIVYINKGKPEKKDKLYEI
                     NATHGGLIQTIAYYLVKPILESALDRYIEKHGLTEYLEEMTQQKEEDSINFYEITEGS
                     GSKAFCGLEVLLQIYKISNNNSATLPSKVSDVTLKIGQDDLKEVSLGAIGMKEGGERV
                     VTIANENKMNFNSYYVKLIEVKDKYPDSVNNLMVFNDLINKTGKQVRCGDEISVKYSV
                     KEHNGEYIVKDQTVQFRVGDKKIPLAIELGVVGMRAGNKRTILSPPDLLTITDDMLIK
                     DIDYDEKNISIIDLSLDVKQEVAAHHSTVEKQPKEYS"
                     /product="FKBP-type
                     peptidyl-prolyl cis-trans isomerase"
                     /besthit="qcoverage 100 , hcoverage 100 , similarity 100
                     , identity 100 , evalue 1.48e-214 , alnlength 313 ,
                     Bacteria , Proteobacteria , WP_015589432.1 MULTISPECIES:
                     FKBP-type peptidyl-prolyl cis-trans isomerase
                     [unclassified Wolbachia]"
     gene            complement(28906..30396)
                     /locus_tag="wEsol_00550"
     CDS             complement(28906..30396)
                     /locus_tag="wEsol_00550"
                     /codon_start=1
                     /transl_table=11
                     /fullproduct="qcoverage 100 , hcoverage 100 , similarity
                     100 , identity 100 , evalue 0.0 , alnlength 496 ,
                     Bacteria , Proteobacteria , WP_006280200.1 MULTISPECIES:
                     ATP-dependent protease ATPase subunit HslU [Wolbachia]"
                     /cog="HslU COG1220 444 ATP-dependent protease HslVU
                     (ClpYQ), ATPase subunit"
                     /pfam="MCM AAA_16 Zeta_toxin Sigma54_activat AAA_2 AAA_5
                     Mg_chelatase AAA AAA_17 AAA_33 AAA_18 AAA_22 AAA_2 AAA
                     ClpB_D2-small"
                     /tigr="TIGR00390 hslU 441 ATP-dependent protease HslVU,
                     ATPase subunit"
                     /product="ATP-dependent
                     protease ATPase subunit HslU"
                     /translation="MSSKQKTLCTNFSNQPITLSEGRSCSSDTYDEKDNLYKDTKSGF
                     DSNDSDVQVNDSTQVLLDDLPPQKIVKELDRFIIGQDDAKRAVAIALRNRWRRNQVPF
                     PLRDEIIPKNILMIGHTGVGKTEIARRLAKLAGAPFIKIEATKFTEIGYVGRDVDSII
                     RDLVDAAIVLVKEKARKALAKKALILAEKTIVNSMVGENATEESKKIFRERLRNKEFE
                     DGEVSINVRESKSMLPTFDIPGMPGGQVGVMNVTEIMGKMFNGSKKTKTITVKVKEAR
                     EILINEESERLMDEDKIIKEAIDLVSNDGIVFLDEIDKIAARTEVKGEVNREGVQRDL
                     LPLLEGTTVTTKYGHVKTDYILFIASGAFHQSKPSDLLPELQGRLPIRVELKALTQED
                     LIRILKEPESSLLKQYIALMKTENVTLEFTDDGIKTIAEIAFTVNRQVENIGARRLHT
                     VMEKLLDEISFIASEKNSEKFIIDSKYVKDKLESISKQLDLSKFIL"
                     /besthit="qcoverage 100 , hcoverage 100 , similarity 100
                     , identity 100 , evalue 0.0 , alnlength 496 , Bacteria ,
```

```
                     Proteobacteria , WP_006280200.1 MULTISPECIES:
                     ATP-dependent protease ATPase subunit HslU [Wolbachia]"
     gene            complement(30398..30952)
                     /locus_tag="wEsol_00551"
     CDS             complement(30398..30952)
                     /locus_tag="wEsol_00551"
                     /codon_start=1
                     /transl_table=11
                     /fullproduct="qcoverage 100 , hcoverage 100 , similarity
                     100 , identity 100 , evalue 8.00e-123 , alnlength 184 ,
                     Bacteria , Proteobacteria , WP_006280198.1 MULTISPECIES:
                     ATP-dependent protease subunit HslV [Wolbachia]"
                     /cog="HslV COG5405 178 ATP-dependent protease HslVU
                     (ClpYQ), peptidase subunit"
                     /pfam="Proteasome Pfam-B_18807"
                     /tigr="TIGR03692 ATP_dep_HslV 171 ATP-dependent protease
                     HslVU, peptidase subunit"
                     /product="ATP-dependent
                     protease subunit HslV"
                     /translation="MIQHDNNKMYGTTILSIRKDKSVVVIGDGQVSLGHTVIKSGAKK
                     VRRLSGDSVIAGFAGATADAFTLFERLESKLDKHPGQLMRACVELAKDWRMDKYLRKL
                     EAMMIVADKSISLVITGTGDVLEPEDGVAAIGSGGNFALSAARALIDIKGISIEEIAK
                     KAMKIAGDICVYTNHNVVIEKIEE"
                     /besthit="qcoverage 100 , hcoverage 100 , similarity 100
                     , identity 100 , evalue 8.00e-123 , alnlength 184 ,
                     Bacteria , Proteobacteria , WP_006280198.1 MULTISPECIES:
                     ATP-dependent protease subunit HslV [Wolbachia]"
     gene            31068..32228
                     /locus_tag="wEsol_00552"
     CDS             31068..32228
                     /locus_tag="wEsol_00552"
                     /codon_start=1
                     /transl_table=11
                     /fullproduct="qcoverage 100 , hcoverage 100 , similarity
                     100 , identity 100 , evalue 9.13e-280 , alnlength 386 ,
                     Bacteria , Proteobacteria , WP_015589431.1 MULTISPECIES:
                     glutamate--cysteine ligase [unclassified Wolbachia]"
                     /pfam="GshA"
                     /tigr="TIGR02049 gshA_ferroox 403 glutamate--cysteine
                     ligase"
                     /product="
                     glutamate--cysteine ligase"
                     /translation="MQNIIHPDLEKSINNWFEAKFNGLTSPFYSSIDLRNSGYKIVPV
                     DANLFPAGFNNLSEESRTMAARLIKSYFKRHQYKKVLIIPENYTRNKMYIENVFVIEK
                     ILQLAGFETRIGLLYNEVYNLIEQYETVVKENSLLKTTSGFVPDVIILNRDMTSHIPD
                     TLKDVKQDIVPSPLYGWHSRQKFKYFEIYQELASEFCGEFKIDPWLISVLTESCDGVD
                     FDDDLSLGAVATKVDQILSLVQKKYEEYEIKTQPYVFIKASNGTYGMGIITATSGEEI
                     LNLNKKKRHKMKKIKEGIAINSVIIQEGVPTIDMFKSSSAEPLIYYIGDTPTCYLYRC
                     NSRKDVYSSLNATDCEFHDVSQVVEGKILSLWSIVSKLAVLALAVEIKSFHP"
                     /besthit="qcoverage 100 , hcoverage 100 , similarity 100
                     , identity 100 , evalue 9.13e-280 , alnlength 386 ,
                     Bacteria , Proteobacteria , WP_015589431.1 MULTISPECIES:
                     glutamate--cysteine ligase [unclassified Wolbachia]"
     gene            complement(32232..33029)
                     /locus_tag="wEsol_00553"
     CDS             complement(32232..33029)
                     /locus_tag="wEsol_00553"
                     /codon_start=1
                     /transl_table=11
                     /pfam="DUF3278"
```

```
                     /fullproduct="qcoverage 100 , hcoverage 100 , similarity
                     100 , identity 100 , evalue 2.60e-184 , alnlength 265 ,
                     Bacteria , Proteobacteria , WP_006280195.1 MULTISPECIES:
                     hypothetical protein [Wolbachia]"
                     /translation="MPNKLKKKSNILAQRVHDIYDHDFRKVICITTGLTALLCKISLQ
                     FCCEFIDNNRSNIFLIRNVARLVKFPTSILYIAHSAFTLQDLIKDYRNPESKKELAKI
                     VGKSANLASSTIEALMMLKIIHFCTGSNTDIISYINLTSTILFLFISEPISYYYTCKE
                     YLDNKDPEKSAECKKDLIISTLTLSLGLLNFILRRIETPTIPINLGNGIIYNFNLSVT
                     VSIIYSAVFLAVQLDKLFQQPVNDDPSTELDDVHNINGHSNDSTVEV"
                     /product="hypothetical protein"
                     /besthit="qcoverage 100 , hcoverage 100 , similarity 100
                     , identity 100 , evalue 2.60e-184 , alnlength 265 ,
                     Bacteria , Proteobacteria , WP_006280195.1 MULTISPECIES:
                     hypothetical protein [Wolbachia]"
     gene            complement(33100..34047)
                     /locus_tag="wEsol_00554"
     CDS             complement(33100..34047)
                     /locus_tag="wEsol_00554"
                     /codon_start=1
                     /transl_table=11
                     /fullproduct="qcoverage 100 , hcoverage 100 , similarity
                     100 , identity 100 , evalue 3.43e-230 , alnlength 315 ,
                     Bacteria , Proteobacteria , WP_010082099.1 MULTISPECIES:
                     ferrochelatase [Wolbachia]"
                     /cog="HemH COG0276 320 Protoheme ferro-lyase
                     (ferrochelatase)"
                     /pfam="Ferrochelatase"
                     /tigr="TIGR00109 hemH 322 ferrochelatase"
                     /product="ferrochelatase"
                     /translation="MKKAVILFNLGGPDSLNAVRPFLFNLFYDRRIINLPNPFRFLLA
                     KFISAKRENTARKIYEEIGGKSPILENTKMQANASELKLNENRNHVHKVFICMRYWRP
                     FADEVIESVKQFDPDEVILLPLYPQYSTTTTLSSIENWQKNAKRYGLKCNTKMIHRYY
                     DNQDFIEAHTNLIAKYYKLARKIGKPRVLFSAHSLPLSIIKKGDPYASQVERSVELIV
                     EKLAINNLDWSICYQSKIGPVKWLEPSTESELLRAKADGVPVVLSPISFVSEHSETLV
                     ELDIEYKAIIKDGYYFRVPTLSTDPLFIKCLADLCINLP"
                     /besthit="qcoverage 100 , hcoverage 100 , similarity 100
                     , identity 100 , evalue 3.43e-230 , alnlength 315 ,
                     Bacteria , Proteobacteria , WP_010082099.1 MULTISPECIES:
                     ferrochelatase [Wolbachia]"
     gene            complement(34048..34869)
                     /locus_tag="wEsol_00555"
     CDS             complement(34048..34869)
                     /locus_tag="wEsol_00555"
                     /codon_start=1
                     /transl_table=11
                     /fullproduct="qcoverage 100 , hcoverage 100 , similarity
                     100 , identity 100 , evalue 6.56e-182 , alnlength 273 ,
                     Bacteria , Proteobacteria , WP_015589429.1 MULTISPECIES:
                     hypothetical protein [unclassified Wolbachia]"
                     /translation="MGECCKNNTTKQAEPTTKKQAKSTTKKLVSNFKSSLFCGTKGFL
                     TFNLAFLSFLEYSLHHNTELKKGAAFAMKRNTIPFLEMAIIVPFVCFALPAMLGSMSL
                     MQCALLCASLMIATCFIVRTFHLREKLMEKITNHSRKEIDAEFKKDTIKFPIFDQNRK
                     HTEYNSPNSGRLLDETSSTSLFVKVLLFPLKVLLNVLQSCIMLSLAAVELLETVPSLF
                     VDTFFDWNFTSTKSNLKRSGHLLYASVRNLVPITKFDECVANFIGTPEVTCCSSA"
                     /product="hypothetical protein"
                     /besthit="qcoverage 100 , hcoverage 100 , similarity 100
                     , identity 100 , evalue 6.56e-182 , alnlength 273 ,
                     Bacteria , Proteobacteria , WP_015589429.1 MULTISPECIES:
                     hypothetical protein [unclassified Wolbachia]"
     gene            34983..35402
```

```
                     /locus_tag="wEsol_00556"
     CDS             34983..35402
                     /locus_tag="wEsol_00556"
                     /codon_start=1
                     /transl_table=11
                     /fullproduct="qcoverage 100 , hcoverage 100 , similarity
                     100 , identity 100 , evalue 5.71e-92 , alnlength 139 ,
                     Bacteria , Proteobacteria , WP_015589428.1 MULTISPECIES:
                     nucleoside-diphosphate kinase [Wolbachia]"
                     /cog="Ndk COG0105 135 Nucleoside diphosphate kinase"
                     /pfam="NDK"
                     /product="
                     nucleoside-diphosphate kinase"
                     /translation="MAIEKTLSILKPDAVKNNITGNINSYIEQSGLKITAQKMMLLTK
                     KQAELFYEIHKDRPFFGELVEFMTSGSVVVQVLVGENAVSKYRQIMGATDPKQADKGT
                     IRGDFADDISENRVHGSDSLENARKEIAFFFAECELV"
                     /besthit="qcoverage 100 , hcoverage 100 , similarity 100
                     , identity 100 , evalue 5.71e-92 , alnlength 139 ,
                     Bacteria , Proteobacteria , WP_015589428.1 MULTISPECIES:
                     nucleoside-diphosphate kinase [Wolbachia]"
     gene            complement(35714..35890)
                     /locus_tag="wEsol_00557"
     CDS             complement(35714..35890)
                     /locus_tag="wEsol_00557"
                     /codon_start=1
                     /transl_table=11
                     /fullproduct="qcoverage 100 , hcoverage 100 , similarity
                     100 , identity 100 , evalue 4.12e-32 , alnlength 58 ,
                     Bacteria , Proteobacteria , WP_182309489.1 hypothetical
                     protein [Wolbachia pipientis]"
                     /translation="MKKKAKETLGLLSALKYWRFLCFKRLTSEIQPLLIATNLRCKCL
                     RNLLSRKKDKESPS"
                     /product="hypothetical protein"
                     /besthit="qcoverage 100 , hcoverage 100 , similarity 100
                     , identity 100 , evalue 4.12e-32 , alnlength 58 ,
                     Bacteria , Proteobacteria , WP_182309489.1 hypothetical
                     protein [Wolbachia pipientis]"
     gene            36383..36988
                     /locus_tag="wEsol_00558"
     CDS             36383..36988
                     /locus_tag="wEsol_00558"
                     /codon_start=1
                     /transl_table=11
                     /fullproduct="qcoverage 100 , hcoverage 100 , similarity
                     100 , identity 100 , evalue 3.33e-137 , alnlength 201 ,
                     Bacteria , Proteobacteria , WP_010963080.1 MULTISPECIES:
                     recombination mediator RecR [Wolbachia]"
                     /cog="RecR COG0353 198 Recombinational DNA repair protein
                     (RecF pathway)"
                     /pfam="RecR Toprim_4 Toprim"
                     /tigr="TIGR00615 recR 196 recombination protein RecR"
                     /product="recombination
                     mediator RecR"
                     /translation="MNVNIKNLVHAFSKLPSLGPSSSRRLVIHLLQNKEKVMLPLASL
                     IKELADLIIECEVCGNLDTKSPCSICTNPKRDTKLLCVVEELGDLWAFEKGNIYSGLY
                     HVLGGRLSAINGIGPKELNLDTILKRVTESKIEEIIIAINPTLEGQVTAQYIIELLKN
                     LDVKISRLACGIPMGGEIDYLDEGTLRIALTSRQEYELNIK"
                     /besthit="qcoverage 100 , hcoverage 100 , similarity 100
                     , identity 100 , evalue 3.33e-137 , alnlength 201 ,
                     Bacteria , Proteobacteria , WP_010963080.1 MULTISPECIES:
```

recombination mediator RecR [Wolbachia]"
gene   36992..38212
                /locus_tag="wEsol_00559"
CDS   36992..38212
                /locus_tag="wEsol_00559"
                /codon_start=1
                /transl_table=11
                /pfam="KAP_NTPase AAA_22"
                /fullproduct="qcoverage 100 , hcoverage 100 , similarity
                100 , identity 100 , evalue 4.75e-283 , alnlength 406 ,
                Bacteria , Proteobacteria , WP_015589427.1 MULTISPECIES:
                hypothetical protein [Wolbachia]"
                /translation="MCLKKLISLITFKKKEIAPPLKVVAWENDRLNYKQFSGKFNNII
                KTIDQSFTIISLEGYDGWGKTFFLKEWVKELKQQNEIAAYYSAWDINALDQPLPSFLN
                FLFEDLFVSYKVKRSVIQQFKNINQELFSLNTLGKLISKSPLAMLSVFLDAAKEADKK
                DIGFVLSELSLLQRRKESTRDFKTQLADVVNKIRKDKNIYIMVDNLDVCRPKFVVDFL
                ESIKYMLDVEGLVFIIPVSKDKSNVQRAISTILGPNFDLKSFTDLSLHLPKQPIEKFT
                KELFENIKLPKKSKNLIVDSFIFYAESLSLSLKTIEYCVKKIKLCLLNHTREELPDPN
                LFSFLVILQSINIDIYEELGFSYQRALEKIENEYKSLVLNGQEEWEKLKTSLETAFAE
                RESKINKAIKDILF"
                /product="hypothetical protein"
                /besthit="qcoverage 100 , hcoverage 100 , similarity 100
                , identity 100 , evalue 4.75e-283 , alnlength 406 ,
                Bacteria , Proteobacteria , WP_015589427.1 MULTISPECIES:
                hypothetical protein [Wolbachia]"
gene   complement(38258..39082)
                /locus_tag="wEsol_00560"
CDS   complement(38258..39082)
                /locus_tag="wEsol_00560"
                /codon_start=1
                /transl_table=11
                /fullproduct="qcoverage 100 , hcoverage 100 , similarity
                100 , identity 100 , evalue 1.71e-173 , alnlength 274 ,
                Bacteria , Proteobacteria , WP_015589426.1 MULTISPECIES:
                hypothetical protein [unclassified Wolbachia]"
                /translation="MNTTVEKNNPKIFPQNEGKINSKGMVAPPVPPKSSQAKFAGIKA
                IFERNVPHSQANDSNETVSSNLGRTFGPTMQKLMEDNIHSTPNKQRRNSGDSGISDIS
                SSTSDDEDRTIKDKMMENVKQDNHQKSFDLHKVQATDELETVKFLKTLSKKEREKLGF
                SPKQQNNQKVETSKKTYNPLDWNPIYQGKKLFSLGNMSSNSSSCSEKSNISDVSDKSN
                NSNIVRCNITRSNSKEDLRKPDASHNDKSAGKKLSNVNVSELIRKFEQGNDIVKGR"
                /product="hypothetical protein"
                /besthit="qcoverage 100 , hcoverage 100 , similarity 100
                , identity 100 , evalue 1.71e-173 , alnlength 274 ,
                Bacteria , Proteobacteria , WP_015589426.1 MULTISPECIES:
                hypothetical protein [unclassified Wolbachia]"
gene   complement(39152..40480)
                /locus_tag="wEsol_00561"
CDS   complement(39152..40480)
                /locus_tag="wEsol_00561"
                /codon_start=1
                /transl_table=11
                /fullproduct="qcoverage 100 , hcoverage 100 , similarity
                100 , identity 100 , evalue 3.26e-306 , alnlength 442 ,
                Bacteria , Proteobacteria , WP_141456686.1 MULTISPECIES:
                pyruvate dehydrogenase complex dihydrolipoamide
                acetyltransferase [unclassified Wolbachia]"
                /cog="AceF COG0508 404 Pyruvate/2-oxoglutarate
                dehydrogenase complex, dihydrolipoamide acyltransferase
                (E2) component, and related enzymes"
                /pfam="Biotin_lipoyl Biotin_lipoyl_2 E3_binding

```
                     2-oxoacid_dh"
                     /tigr="TIGR01349 PDHac_trf_mito 437 pyruvate
                     dehydrogenase complex dihydrolipoamide acetyltransferase"
                     /product="pyruvate
                     dehydrogenase complex dihydrolipoamide acetyltransferase"
                     /translation="MPIEILMPALSPTMSKTGGKIVKWHKKEQDKVEVGDVIAEIETD
                     KAIMEFESVDEGVVAKILVTEGTSGVPVNQPIALMLEEGEDENALNNYTSTSINSAVK
                     KEVTKCAVDNQKSEHQDLNGKPISHSSVSFQRVTLESNLSMSSQCLTLGSRKEEDTKT
                     TEGRIKISPLAKKIAQNEGVNVQQLKGTGPYGRIIKADVLEFLGSGIHTESPEIVEVS
                     NIRQVIAQRLTESKQNVPHFYLTVDCQVDKLISLKNEINSADENNKVTINDLIIKAVA
                     FSMKKFPDINSSWIDNKILKYSNVDISIAVALEDGLITPIVKNADKKGILSISKEVKD
                     LVSRARSGKLKPEEFQGGGFTISNLGMFGIKAFSAIINPPQSCIMAVGASKKQPIVMN
                     EKIETAEIMTVTLSVDHRAVDGALGAKFLNAFKHYIENPLVMLIETAVIY"
                     /besthit="qcoverage 100 , hcoverage 100 , similarity 100
                     , identity 100 , evalue 3.26e-306 , alnlength 442 ,
                     Bacteria , Proteobacteria , WP_141456686.1 MULTISPECIES:
                     pyruvate dehydrogenase complex dihydrolipoamide
                     acetyltransferase [unclassified Wolbachia]"
     gene            40551..41042
                     /locus_tag="wEsol_00562"
     CDS             40551..41042
                     /locus_tag="wEsol_00562"
                     /codon_start=1
                     /transl_table=11
                     /fullproduct="qcoverage 100 , hcoverage 100 , similarity
                     100 , identity 100 , evalue 8.31e-117 , alnlength 163 ,
                     Bacteria , Proteobacteria , WP_015589424.1 MULTISPECIES:
                     hypothetical protein [unclassified Wolbachia]"
                     /cog="COG3807 COG3807 171 Uncharacterized protein
                     conserved in bacteria"
                     /pfam="Pfam-B_9058 SH3_4 SH3_4 SH3_3"
                     /product="hypothetical protein"
                     /translation="MMNKSVTLSLTLSQCVTLGSWFSTIILLFLLFSHSLFANNFVST
                     KSNKINMRTGPGFHYPVKWIYTCKNLPLKVIEEFESWKKVCDIDEDCGWIKGNLLSDK
                     RYAIVKEDTYGYQKQSVDSKITIKIDKFVVMKIEKCNEEWCFLSTPKRKAWVQKKHIY
                     GVD"
                     /besthit="qcoverage 100 , hcoverage 100 , similarity 100
                     , identity 100 , evalue 8.31e-117 , alnlength 163 ,
                     Bacteria , Proteobacteria , WP_015589424.1 MULTISPECIES:
                     hypothetical protein [unclassified Wolbachia]"
     gene            complement(41097..41534)
                     /locus_tag="wEsol_00563"
     CDS             complement(41097..41534)
                     /locus_tag="wEsol_00563"
                     /codon_start=1
                     /transl_table=11
                     /fullproduct="qcoverage 100 , hcoverage 100 , similarity
                     99.3 , identity 99.3 , evalue 1.27e-91 , alnlength 145 ,
                     Bacteria , Proteobacteria , WP_015589423.1 hypothetical
                     protein [Wolbachia endosymbiont of Drosophila simulans]"
                     /translation="MSLKNKISELIEQQFSIKYHDNYLAYAIHKWAASEISNSIAFCI
                     IGCGNCKIGYQDAANSFNITKEEIEQGNIASIESKIKSEAEKSGSEFSELISRVDQSL
                     REQSEKSIVQILDSSAEEMLRSFCDSPVQSPEHEEVSAILCFT"
                     /product="hypothetical protein"
                     /besthit="qcoverage 100 , hcoverage 100 , similarity 99.3
                     , identity 99.3 , evalue 1.27e-91 , alnlength 145 ,
                     Bacteria , Proteobacteria , WP_015589423.1 hypothetical
                     protein [Wolbachia endosymbiont of Drosophila simulans]"
     gene            complement(41656..44463)
                     /locus_tag="wEsol_00564"
```

```
CDS             complement(41656..44463)
                /locus_tag="wEsol_00564"
                /codon_start=1
                /transl_table=11
                /pfam="Pfam-B_6118"
                /fullproduct="qcoverage 99.2513368983957 , hcoverage 100
                , similarity 99.8 , identity 99.8 , evalue 0.0 ,
                alnlength 928 , Bacteria , Proteobacteria ,
                WP_174516895.1 hypothetical protein [Wolbachia
                endosymbiont of Cardiocondyla obscurior]"
                /translation="MSGYRYFMRSVRLNFLLIFFLILGLSYQGYADCPTFVESNTWLE
                AIVGGDWVKEIKVQAIKDQGVDGYFNPKIRVCAYDGQSYCYDLQSGESCRQIYGTQGT
                GAGVSAAAFIDWEGDKTKAGGEIARGLKWEWADGVTDEEKRRFANSPKICACSQKGAC
                MSGISSWGSRVFSGENIFRPGEMEKVCDTCYQTAVKCAPVPLAPSPPPFCEQLAMSPP
                QVRIVPITNEDNDYFDPRVGVIISGLEERKELGFPHVVSNNENIPNHSYTVTDKDTHY
                YFKTYRKKGQLCAEYLGTQSINEQNPQFVRCFPAPPAPEPEIVEIVNKNTLKVEMKMS
                ESTCAKAHGTYSNGSCTFNVNTDLNHPINIGPLSLKVVKPAIVAKTTDSNIDNIIEGI
                LESNPQQFKVLKEYGYVPDVQIKCSKGGSFYSFNDFEKKLKDKSIQCEFNNSGQPEIE
                VKYKGNSNSKMLCLSGWQPEPEEFVLKRENKIIPLKLMGTRYTKYNAVYSKESNQFYY
                LPSKENKRADLLNRDQSELDKIIFNKQGYVFFPDENEQKKRECSYCVKRNDEIDDTFK
                SSKGLKVVYKLTDVEHREKCNQGDDGCICFTGTCSRSTRYVDKENTQPFYTKISQNTN
                GVVTQQEAPIKANRTEVFYADKLCRFDLEGLKEKLKEIIEKQLEKEKQEPEKKSEKSH
                VFGSGDYDVDGSQITSSRVEIEVWGCGEAGHIKDTPYTYTPNSTESRVGMPGDYIKAE
                LEIDLDYPIININIKVEQGGGNREKEYTDKDGGPIFIRKCRLGKQECKYLITMGGGGIYK
                IYEYKDYTEPIIDDSVKLKEKTTTTGLRVTEDNKIIYIENGEIKHETVKCSKGFGSSK
                PGAGGCIDKSKRIYGKGSAGQVKVTPIIKGFDINIINDAIEKMIGNRYDPGEVRDFSR
                SEFDTSLTKMIEEELKKELLR"
                /product="hypothetical protein"
                /besthit="qcoverage 99.2513368983957 , hcoverage 100 ,
                similarity 99.8 , identity 99.8 , evalue 0.0 , alnlength
                928 , Bacteria , Proteobacteria , WP_174516895.1
                hypothetical protein [Wolbachia endosymbiont of
                Cardiocondyla obscurior]"
gene            complement(44423..46777)
                /locus_tag="wEsol_00565"
CDS             complement(44423..46777)
                /locus_tag="wEsol_00565"
                /codon_start=1
                /transl_table=11
                /fullproduct="qcoverage 100 , hcoverage 100 , similarity
                100 , identity 100 , evalue 0.0 , alnlength 784 ,
                Bacteria , Proteobacteria , WP_141456684.1 MULTISPECIES:
                type VI secretion protein [unclassified Wolbachia]"
                /cog="VirB4 COG3451 796 Type IV secretory pathway, VirB4
                components"
                /pfam="CagE_TrbE_VirB TrwB_AAD_bind AAA_10"
                /tigr="TIGR00929 VirB4_CagE 792 type IV
                secretion/conjugal transfer ATPase, VirB4 family"
                /product="type VI secretion
                protein"
                /translation="MQLKVPTVNSKLKKKNNLALLESIDLDFVPYACHYDEETILTKQ
                GELLKIIKLEDYSSVDNYSDLRTEIRKSISKNINSLCFTVWIHTVRKRNKLSLKWNKT
                EDFSDNLHSTWFNKLTDSKLQYINELYIVILFSDFGKHTNNSFFFNRIKNKHKLSLQE
                SHQELQKITDVIQNDLGPFGAKKLSLRFSEGKIYSEMIEFLHYIVTLTHKDYPIHERD
                LSQYIRNFKIAFGFNTFQTIFENQQKFGSIFSIKEYREISLGNIDRCLQLESEFIITE
                IMIFTSNNKAIKEFKKQINMLQISEDNTLLKSSGIEEIIELEKTSSIDFCQQKIIFTI
                FADDRNKLAESISDLSSIMSLVGFMMFRTDLHMESHFWAQLPGNFAFITQPKNILTKY
                ACSFAMLHDFTSGTLKGRRWKEAVMIFFSKKGNPYFFNFHGKRNNGHTTILGAPNSGR
                TSLINFLLSESRKFNPRIVILDNTGKSIIFTKAVSGKYYIIDPKYKDKSLKFNPLNIE
                DSASNRNMLVELIRRMVADTSLVDVEEKIRKIVDSIFAIPKESRSISQISEVLLLLGG
```

```
                     KISRWCDDGDFAYLFQDGNESDIDWETKIISLNTANLTKQKECMSVILYYFLYSFEAK
                     CDGSPAILVLDEAWEISNIFPTEKEFDNWMQRMTELNVVVILSTENLNLAFASKFTQY
                     LDKHVDTRILMPNVNANRLYMKAFSLSKEELNIILQTPTQEGLFLIKQYKGLVTLNLD
                     LKNMQEIHVLSANKETIKYMYEAIKEKGEEVNEWLPVFYEKCKA"
                     /besthit="qcoverage 100 , hcoverage 100 , similarity 100
                     , identity 100 , evalue 0.0 , alnlength 784 , Bacteria ,
                     Proteobacteria , WP_141456684.1 MULTISPECIES: type VI
                     secretion protein [unclassified Wolbachia]"
     gene            complement(46822..47196)
                     /locus_tag="wEsol_00566"
     CDS             complement(46822..47196)
                     /locus_tag="wEsol_00566"
                     /codon_start=1
                     /transl_table=11
                     /pfam="MMPL"
                     /fullproduct="qcoverage 100 , hcoverage 100 , similarity
                     100 , identity 100 , evalue 1.03e-71 , alnlength 124 ,
                     Bacteria , Proteobacteria , WP_022626145.1 MULTISPECIES:
                     hypothetical protein [Wolbachia]"
                     /translation="MRIQLNKVLVFIALLSFIFASYSSEASSASDELKGIQETVKNFI
                     SSFSVKDTVENMSPSTIIGLCIAAVILAVVFRGLIFFMIMFGVLVLMFGSTEKATDYL
                     KEKFNFAKDIKLQSDSEKKDKN"
                     /product="hypothetical protein"
                     /besthit="qcoverage 100 , hcoverage 100 , similarity 100
                     , identity 100 , evalue 1.03e-71 , alnlength 124 ,
                     Bacteria , Proteobacteria , WP_022626145.1 MULTISPECIES:
                     hypothetical protein [Wolbachia]"
     gene            complement(47291..48514)
                     /locus_tag="wEsol_00567"
     CDS             complement(47291..48514)
                     /locus_tag="wEsol_00567"
                     /codon_start=1
                     /transl_table=11
                     /pfam="Pfam-B_1673 DUF3534"
                     /fullproduct="qcoverage 100 , hcoverage 100 , similarity
                     100 , identity 100 , evalue 2.62e-230 , alnlength 407 ,
                     Bacteria , Proteobacteria , WP_015589419.1 MULTISPECIES:
                     hypothetical protein [unclassified Wolbachia]"
                     /translation="MLSSKKDINQQLHDTIYKKVTKENHKYGSPQKGDGTIKKCIELF
                     KEGASPEVLTELEKSLREQLEKSPEKQEEYQKHYKYYIENFLPALKDEVIKSEELAVP
                     TVFNNEREQSQRPQTIRGKIVKTILDITNRSSSVSSIPNAVEENIEDQMQLKHIEEQN
                     NTQETPLNVAEKKSGDNKILSNSDVAEEVFEKYSKVVKEIEEKKTSKLSSGNDSDSGI
                     DSPRTSKESLKSPVSSRRSSLTLVTSTSSEESLNSTLSSVEGDNGLQPEGGEKTELLE
                     SNLESPKENETQVQPEQTVKKEPKMQENENSDGQANPDQVKKDILLKNQPNNRNLHVA
                     LVAGCALSTIGCIVAGAMTSGLIGAGLLAMVAVLAIAAVAELHSNFLSSKLTSINVSP
                     LVDDKSLQHVHSGKN"
                     /product="hypothetical protein"
                     /besthit="qcoverage 100 , hcoverage 100 , similarity 100
                     , identity 100 , evalue 2.62e-230 , alnlength 407 ,
                     Bacteria , Proteobacteria , WP_015589419.1 MULTISPECIES:
                     hypothetical protein [unclassified Wolbachia]"
     gene            complement(48637..49665)
                     /locus_tag="wEsol_00568"
     CDS             complement(48637..49665)
                     /locus_tag="wEsol_00568"
                     /codon_start=1
                     /transl_table=11
                     /fullproduct="qcoverage 100 , hcoverage 100 , similarity
                     100 , identity 100 , evalue 6.00e-240 , alnlength 342 ,
                     Bacteria , Proteobacteria , WP_015589418.1 MULTISPECIES:
```

```
                     nitronate monooxygenase [unclassified Wolbachia]"
                     /cog="COG2070 COG2070 336 Dioxygenases related to
                     2-nitropropane dioxygenase"
                     /pfam="NMO ThiG His_biosynth FMN_dh DHO_dh IMPDH"
                     /tigr="TIGR03151 enACPred_II 307 putative
                     enoyl-[acyl-carrier-protein] reductase II"
                     /product="nitronate
                     monooxygenase"
                     /translation="MLSSLWKKGTDFLGSEFAIMGGAMSWVSERNLVSAISNAGGFGV
                     IACGAMFPDLLEKEIIETQKLTNKPFGVNLITMHPKLSELIDVCIETKVSHIVLAGGL
                     PTKPNIEKIKGAGIKVMCFAPALSLAKRLVKMGVDALIIEGMEAGGHIGPVSTSVLAQ
                     EILPHFKNEQIPVFVAGGIGRGEVIVNYLEMGASGCQIGTLFVCTNESIAHKNFKEVF
                     IKSAARNAIPSVQISADFPVIPVRAIANKASDDFMKRQKEVIDEYQKGQISKEDGQLE
                     IEKFWAGALRRAVIDGDVETGSLMAGQSVGMVDREKPVKEVVNMLVQQAKNHINSKSV
                     EVESPESK"
                     /besthit="qcoverage 100 , hcoverage 100 , similarity 100
                     , identity 100 , evalue 6.00e-240 , alnlength 342 ,
                     Bacteria , Proteobacteria , WP_015589418.1 MULTISPECIES:
                     nitronate monooxygenase [unclassified Wolbachia]"
     gene            complement(49671..50192)
                     /locus_tag="wEsol_00569"
     CDS             complement(49671..50192)
                     /locus_tag="wEsol_00569"
                     /codon_start=1
                     /transl_table=11
                     /fullproduct="qcoverage 100 , hcoverage 100 , similarity
                     100 , identity 100 , evalue 5.30e-118 , alnlength 173 ,
                     Bacteria , Proteobacteria , WP_015589417.1 MULTISPECIES:
                     cytochrome b [unclassified Wolbachia]"
                     /cog="CybB COG3038 181 Cytochrome B561"
                     /pfam="Cytochrom_B_N Ni_hydr_CYTB Cytochrom_B561 DUF4405"
                     /product="cytochrome b"
                     /translation="MEDNKYSLGLRVIHWLMAVFIIGMLCSGLYMKSLPLSNEIKFNI
                     YAIHKACGITILGLIIVRIFFRVFTYVPPLPANFSRFVINISKAVHFGLYSLMVLMPL
                     SGYVMSSASGKQIKYFFHIPLLINQNKDLANTANELHSMLAYFMVLFIILHILGALKH
                     TFIDKQNIFKRMI"
                     /besthit="qcoverage 100 , hcoverage 100 , similarity 100
                     , identity 100 , evalue 5.30e-118 , alnlength 173 ,
                     Bacteria , Proteobacteria , WP_015589417.1 MULTISPECIES:
                     cytochrome b [unclassified Wolbachia]"
     gene            complement(50607..50783)
                     /locus_tag="wEsol_00570"
     CDS             complement(50607..50783)
                     /locus_tag="wEsol_00570"
                     /codon_start=1
                     /transl_table=11
                     /fullproduct="qcoverage 100 , hcoverage 100 , similarity
                     100 , identity 100 , evalue 3.40e-31 , alnlength 58 ,
                     Bacteria , Proteobacteria , WP_215401836.1 hypothetical
                     protein [Wolbachia endosymbiont of Drosophila simulans]"
                     /translation="MKKKAKEALRLLSALKYWRFLCFKRLTSEIQPLLIATNLRCKCL
                     RNLLSRKKDKESPS"
                     /product="hypothetical protein"
                     /besthit="qcoverage 100 , hcoverage 100 , similarity 100
                     , identity 100 , evalue 3.40e-31 , alnlength 58 ,
                     Bacteria , Proteobacteria , WP_215401836.1 hypothetical
                     protein [Wolbachia endosymbiont of Drosophila simulans]"
     gene            51492..52688
                     /locus_tag="wEsol_00571"
     CDS             51492..52688
```

```
                     /locus_tag="wEsol_00571"
                     /codon_start=1
                     /transl_table=11
                     /fullproduct="qcoverage 100 , hcoverage 100 , similarity
                     100 , identity 100 , evalue 5.73e-280 , alnlength 398 ,
                     Bacteria , Proteobacteria , WP_015589416.1 MULTISPECIES:
                     phosphoglycerate kinase [unclassified Wolbachia]"
                     /cog="Pgk COG0126 395 3-phosphoglycerate kinase"
                     /pfam="PGK"
                     /product="phosphoglycerate
                     kinase"
                     /translation="MSIPSIENCDLHNKAVLLRVDFNVPIKDGEIRDVTRILRALPTI
                     QYLVNASAKIIIISHFGRPKARDNNLSLKNVIDTLSQLLNKKVKFIDDCFGEKVQRAV
                     SVMDAGDIILLENLRFYKEEEQSDSNFAKQLASLADIYVNDAFSCSHRAHASISRITE
                     FLPSYAGFCLQDELKYLEKAVSFKAKPITAIVGGAKISTKIKVLMKLTEKVDYLVLGG
                     AIANNFLSFSKVNIGKSFFQNGVDDLLHNIVETANKNNCKIVVPEDVLVAVNSDYSTS
                     ISRRTESILDGDIILDIGPQTLSTISSIIASSKTLLWNGPIGVFEHSAFASGTIGVMK
                     IVSDLTHKGKLTSIIGGGDSLSAISAAGLTDKDFTYVSTGGGAFLDWLSGDEMPGVAA
                     LQKRLD"
                     /besthit="qcoverage 100 , hcoverage 100 , similarity 100
                     , identity 100 , evalue 5.73e-280 , alnlength 398 ,
                     Bacteria , Proteobacteria , WP_015589416.1 MULTISPECIES:
                     phosphoglycerate kinase [unclassified Wolbachia]"
     gene            52738..53025
                     /locus_tag="wEsol_00572"
     CDS             52738..53025
                     /locus_tag="wEsol_00572"
                     /codon_start=1
                     /transl_table=11
                     /fullproduct="qcoverage 100 , hcoverage 100 , similarity
                     100 , identity 100 , evalue 6.10e-64 , alnlength 95 ,
                     Bacteria;Eukaryota , Proteobacteria;Arthropoda ,
                     WP_012673373.1 MULTISPECIES: hypothetical protein
                     [Wolbachia]"
                     /translation="MSKTVKLILCLIIPIIGFIFYIKDHYFHTGYVEENVEVNFEPFF
                     SSQPATVSELTDFDRGVLKVCGDWGAHPDKEDFKILLSCPQHQEVVKGIYK"
                     /product="hypothetical protein"
                     /besthit="qcoverage 100 , hcoverage 100 , similarity 100
                     , identity 100 , evalue 6.10e-64 , alnlength 95 ,
                     Bacteria;Eukaryota , Proteobacteria;Arthropoda ,
                     WP_012673373.1 MULTISPECIES: hypothetical protein
                     [Wolbachia]"
     gene            53022..53366
                     /locus_tag="wEsol_00573"
     CDS             53022..53366
                     /locus_tag="wEsol_00573"
                     /codon_start=1
                     /transl_table=11
                     /pfam="XendoU EndoU_bacteria"
                     /fullproduct="qcoverage 100 , hcoverage 100 , similarity
                     100 , identity 100 , evalue 5.31e-85 , alnlength 114 ,
                     Bacteria , Proteobacteria , WP_227738569.1 EndoU
                     domain-containing protein [Wolbachia endosymbiont of
                     Drosophila simulans]"
                     /translation="MITPDADLELLKDELANIWFTNSESEKETIGFGHIFCGEPNDKL
                     GGMHFVGRYVEAQEDKWAGAIWNDKSLCNKSDIKPPVYTFGMKYLGKDGEVKVKCPNG
                     YAYNLHADNFCN"
                     /product="EndoU domain-containing protein"
                     /besthit="qcoverage 100 , hcoverage 100 , similarity 100
                     , identity 100 , evalue 5.31e-85 , alnlength 114 ,
```

```
                     Bacteria , Proteobacteria , WP_227738569.1 EndoU
                     domain-containing protein [Wolbachia endosymbiont of
                     Drosophila simulans]"
     gene            53323..53532
                     /locus_tag="wEsol_00574"
     CDS             53323..53532
                     /locus_tag="wEsol_00574"
                     /codon_start=1
                     /transl_table=11
                     /fullproduct="qcoverage 100 , hcoverage 93.2432432432432
                     , similarity 100 , identity 100 , evalue 1.85e-45 ,
                     alnlength 69 , Bacteria , Proteobacteria , KDB19218.1
                     hypothetical protein wGmm_0106 [Wolbachia endosymbiont of
                     Glossina morsitans morsitans]"
                     /translation="MDTHITFMPIISATKVFKELGKDGMCLYEMEDDDYQSVFVRKND
                     AILTFYPDLTPKCDDKSTYCSCGKS"
                     /product="hypothetical protein"
                     /besthit="qcoverage 100 , hcoverage 93.2432432432432 ,
                     similarity 100 , identity 100 , evalue 1.85e-45 ,
                     alnlength 69 , Bacteria , Proteobacteria , KDB19218.1
                     hypothetical protein wGmm_0106 [Wolbachia endosymbiont of
                     Glossina morsitans morsitans]"
     gene            53630..53980
                     /locus_tag="wEsol_00575"
     CDS             53630..53980
                     /locus_tag="wEsol_00575"
                     /codon_start=1
                     /transl_table=11
                     /fullproduct="qcoverage 100 , hcoverage 100 , similarity
                     100 , identity 100 , evalue 5.09e-66 , alnlength 116 ,
                     Bacteria , Proteobacteria , WP_007549043.1 MULTISPECIES:
                     diacylglycerol kinase [Wolbachia]"
                     /cog="DgkA COG0818 123 Diacylglycerol kinase"
                     /pfam="DAGK_prokar"
                     /product="diacylglycerol
                     kinase"
                     /translation="MKKGIIRLIKAIQYSCEGIKSAFISEIAFRQELLLFIVCVSILF
                     VLDVSNLERAVMISSLFLVLIVEIVNTAFETTIERISSEQHILSKKVKDLGSAAVFLS
                     LINFLITWMIILIG"
                     /besthit="qcoverage 100 , hcoverage 100 , similarity 100
                     , identity 100 , evalue 5.09e-66 , alnlength 116 ,
                     Bacteria , Proteobacteria , WP_007549043.1 MULTISPECIES:
                     diacylglycerol kinase [Wolbachia]"
     gene            complement(53992..54804)
                     /locus_tag="wEsol_00576"
     CDS             complement(53992..54804)
                     /locus_tag="wEsol_00576"
                     /codon_start=1
                     /transl_table=11
                     /fullproduct="qcoverage 100 , hcoverage 100 , similarity
                     100 , identity 100 , evalue 1.84e-191 , alnlength 270 ,
                     Bacteria , Proteobacteria , AGK00383.1 Pseudouridine
                     synthase [Wolbachia endosymbiont of Drosophila simulans
                     wHa]"
                     /cog="RluA COG0564 289 Pseudouridylate synthases, 23S
                     RNA-specific"
                     /pfam="PseudoU_synth_2 Pfam-B_415"
                     /tigr="TIGR00005 rluA_subfam 298 pseudouridine synthase,
                     RluA family"
                     /product="Pseudouridine synthase"
```

/translation="MKLLGIPIINNDHIVKPGEEYIVHLVQPDISTSIEPNYDIKLDI
VYEDEDIIVLSKQSGLTVHPGAGTNNDTLLNAVIAHLNVRPGIVHRLDKDTSGLMVIA
KNEAAHSFLSELLSNRKIKREYLAVVWGTLSSQQGTIKTNIAPKRGNKEMMCVTKITG
KLAITHYLVQKVIGQVSLVKCTLETGRTHQIRVHMSHIGHSIVGDQVYGKNSSKSTKY
AKNSSFIRNFNRQALHAYTLGLYHPKSKGYMEFVSDLPQDIKTLIGEFDNIS"
/besthit="qcoverage 100 , hcoverage 100 , similarity 100
, identity 100 , evalue 1.84e-191 , alnlength 270 ,
Bacteria , Proteobacteria , AGK00383.1 Pseudouridine
synthase [Wolbachia endosymbiont of Drosophila simulans
wHa]"
gene complement(54927..56630)
/locus_tag="wEsol_00577"
CDS complement(54927..56630)
/locus_tag="wEsol_00577"
/codon_start=1
/transl_table=11
/fullproduct="qcoverage 98.7654320987654 , hcoverage
99.1150442477876 , similarity 100 , identity 99.8 ,
evalue 0.0 , alnlength 560 , Bacteria , Proteobacteria ,
WP_141456661.1 MULTISPECIES: hypothetical protein
[unclassified Wolbachia]"
/translation="MLWLQTLKDPFKDYFDFLGFRKLSDQPSRILSSIRKYSGTISIA
STIATLIAFTAFSIQPVTVSIAGFAFTPAPLAFIPLIVLLFTAVINFINTQKIKENEI
DKKVGNYQVDEQYSQIVQEAEKIEIVTNLDKKGEKLSIVLPISKTQREILEDIEAENR
NRILLFTGSYVFGTIIVVSAISQSINAPIILIALLVAILLCIISTLSNLISNSVDHEN
HTIRKHSSLGLLFPYWSGKGMIVSVVENKLGKRENELISLMKKLLSPLCNSFDSNLKK
ACDLLDNSLLKPANINIKETLDHLKKIREDIKKDLDWLHAEISEVLNNTKELTKKAHS
LDIEGTFSRLENAIKIAEDKIERFEPSRFVGWLQSTTQSNNNEFLSMLKKLKEDTEKE
KTELKKQLNVVRNELEEIKKKQAQPSTPDLEANNGNNTNKLLVLENKVKELRDTVRKL
ELEAEQNKLQQKKEKLEQKKSEQEKTDEWKEKINGKPSDFLYYLLESIKLEEKDNKEK
KVEATLRDFDNKVTIYWKDGSQTICHIKKQGDLQIESSATMFTDPNVQAAWEMARGKS
L"
/product="hypothetical protein"
/besthit="qcoverage 98.7654320987654 , hcoverage
99.1150442477876 , similarity 100 , identity 99.8 ,
evalue 0.0 , alnlength 560 , Bacteria , Proteobacteria ,
WP_141456661.1 MULTISPECIES: hypothetical protein
[unclassified Wolbachia]"
gene complement(56805..58949)
/locus_tag="wEsol_00578"
CDS complement(56805..58949)
/locus_tag="wEsol_00578"
/codon_start=1
/transl_table=11
/fullproduct="qcoverage 99.0196078431373 , hcoverage
96.9821673525377 , similarity 98.6 , identity 97.6 ,
evalue 0.0 , alnlength 707 , Bacteria , Proteobacteria ,
EAL59121.1 ComEC/Rec2 family protein [Wolbachia
endosymbiont of Drosophila ananassae]"
/cog="ComEC COG0658 453 Predicted membrane metal-binding
protein"
/pfam="DUF4131 Competence"
/product="ComEC/Rec2 family protein"
/translation="MSLTSYRAAPSRVVAYLVIIYTNNEKIKIKFHHMQISTHLLTFL
DIIVSYIRNNLRNEQYNLILWFPVFQCVGILTYFSLSFEPSCISIFLLLLPTLILIAI
LYKRYAILCIALIAVLIGFTASKLRTALVDTQILDKERYVKDIVATVKDINDRGSYKQ
FLLSVEKSPTISKSSPVIPAPPFVIPVLDTGIQKKKIWIPASRAGMTPDRALDNIRIS
VRTKVEKGIKIGDQVKLSAKLFPLKIAPSEYAYDFARIAYYQKISATGFATSKIALHK
KAEARKFQEYIESFRQYIYENLQQNIKKPHADIISALLIGKKDGIDQKTMDAIRDSGI
AHLFAISGLHLSFVAGLFFVVFRNLFAISETLTLKYNTKKISAFLTILPTTFYLLITG

MQISAQRAYIMVILVLVAIMIERKYRGLIAIAFAASVILSVEPEAILKPGFQMSFSAV
LALVASYQINANKLFKIKIMKYFVSIMISSVIASLATVPYTIYNFNYFSISGIITNLV
AIPIVTLIIIPLGIIYVLLIPFGIEWIIAPLVERPIDSILYITNAIASLQYLVIPIRT
FPASSIIIITFGLLWLCLWERNWRFLGFFFIVLGICFSAAYKTPDILINADNVAAKES
DNLLYSLTRKSRNFVIKTWAKQNGQNQILNHTKYNNSNKRLQCEDYDCIYNKGNNKSV
LLAYKKEDISENCDKVDLTIQLSEFRLFSLQR"
/besthit="qcoverage 99.0196078431373 , hcoverage
96.9821673525377 , similarity 98.6 , identity 97.6 ,
evalue 0.0 , alnlength 707 , Bacteria , Proteobacteria ,
EAL59121.1 ComEC/Rec2 family protein [Wolbachia
endosymbiont of Drosophila ananassae]"
gene complement(58993..59643)
/locus_tag="wEsol_00579"
CDS complement(58993..59643)
/locus_tag="wEsol_00579"
/codon_start=1
/transl_table=11
/fullproduct="qcoverage 100 , hcoverage 100 , similarity
97.7 , identity 95.4 , evalue 1.35e-148 , alnlength 216 ,
Bacteria , Proteobacteria , Q73G09.1 RecName:
Full=Pyridoxine/pyridoxamine 5'-phosphate oxidase;
AltName: Full=PNP/PMP oxidase; Short=PNPOx; AltName:
Full=Pyridoxal 5'-phosphate synthase [Wolbachia
endosymbiont of Drosophila melanogaster]"
/cog="PdxH COG0259 214 Pyridoxamine-phosphate oxidase"
/pfam="Pyridox_oxidase Pyridox_oxase_2 PNPOx_C"
/tigr="TIGR00558 pdxH 217 pyridoxamine 5'-phosphate
oxidase"
/product="RecName: Full=Pyridoxine/pyridoxamine
5'-phosphate oxidase; AltName: Full=PNP/PMP oxidase;
Short=PNPOx; AltName: Full=Pyridoxal 5'-phosphate
synthase"
/translation="MAVALIFATRARIKILFFDMIFSLEKDPFDLFSKWYKAVLNSPC
EQPTAMTLATCSKDCIPSARVVLLKEYSKEGFVFFTNVNSKKGKELTENPKAALVFHW
IEFSRQVRIEGEVKLLNDERTDKYFSSRARDSQISAWCSKQSSILKDWQDFEQAIELK
NKEFYDQKVPRPDFWVGFCVIPKVIEFWQEGEYRKHIRFRYTLVEGSDWKVEQLYP"
/besthit="qcoverage 100 , hcoverage 100 , similarity 97.7
, identity 95.4 , evalue 1.35e-148 , alnlength 216 ,
Bacteria , Proteobacteria , Q73G09.1 RecName:
Full=Pyridoxine/pyridoxamine 5'-phosphate oxidase;
AltName: Full=PNP/PMP oxidase; Short=PNPOx; AltName:
Full=Pyridoxal 5'-phosphate synthase [Wolbachia
endosymbiont of Drosophila melanogaster]"
gene complement(59603..60652)
/locus_tag="wEsol_00580"
CDS complement(59603..60652)
/locus_tag="wEsol_00580"
/codon_start=1
/transl_table=11
/fullproduct="qcoverage 100 , hcoverage 100 , similarity
99.7 , identity 99.7 , evalue 7.46e-233 , alnlength 349 ,
Bacteria , Proteobacteria , CDR79518.1
formamidopyrimidine-DNA
glycosylase,Formamidopyrimidine-DNA
glycosylase,formamidopyrimidine/5-formyluracil/
5-hydroxymethyluracil DNA
glycosylase,formamidopyrimidine-DNA
glycosylase,Formamidopyrimidine-DNA glycosylase
N-terminal domain [Wolbachia endosymbiont of Drosophila
simulans wAu]"

```
                     /cog="Nei COG0266 273 Formamidopyrimidine-DNA
                     glycosylase"
                     /pfam="Fapy_DNA_glyco H2TH zf-FPG_IleRS"
                     /tigr="TIGR00577 fpg 276 formamidopyrimidine-DNA
                     glycosylase"
                     /product="formamidopyrimidine-DNA
                     glycosylase,Formamidopyrimidine-DNA
                     glycosylase,formamidopyrimidine/5-formyluracil/
                     5-hydroxymethyluracil DNA
                     glycosylase,formamidopyrimidine-DNA
                     glycosylase,Formamidopyrimidine-DNA glycosylase
                     N-terminal domain"
                     /translation="MIMNNTKKNKEKEEEKKRLAKALKQNILKRKKQQQSRQRTLSKL
                     VCGEEFLGESTAEYSNVFENDKITIRNLSFEGGIMPELPEVEVISNFLLDKIKNKQIS
                     NVIVNNWNLRAPITKNIDDMLKGKVIRNIKRRGKYTIWNTDGSVAVIIHLGMSGKLIY
                     ADHDQMRNKHDHVVFLFSDNTSIIFNDPRRFGLVIVLNKEQETDFFSDFGIEPLTDEF
                     SGDYLQELLKNKKVNIKSALMDNKSIVGVGNIYASESLFRACISPLRSAKNLTYRECE
                     KLAAEIKNTLSDAIAAGGSTLKDYAQPSGSAGYFQNNFYVYGKVQKPCKICNNIITLI
                     RQNGRSTYFCNACQN"
                     /besthit="qcoverage 100 , hcoverage 100 , similarity 99.7
                     , identity 99.7 , evalue 7.46e-233 , alnlength 349 ,
                     Bacteria , Proteobacteria , CDR79518.1
                     formamidopyrimidine-DNA
                     glycosylase,Formamidopyrimidine-DNA
                     glycosylase,formamidopyrimidine/5-formyluracil/
                     5-hydroxymethyluracil DNA
                     glycosylase,formamidopyrimidine-DNA
                     glycosylase,Formamidopyrimidine-DNA glycosylase
                     N-terminal domain [Wolbachia endosymbiont of Drosophila
                     simulans wAu]"
     gene            complement(60792..>61158)
                     /locus_tag="wEsol_00581"
     CDS             complement(60792..>61158)
                     /locus_tag="wEsol_00581"
                     /codon_start=1
                     /transl_table=11
                     /product="hypothetical protein"
                     /pfam="Carboxyl_trans"
                     /tigr="TIGR01117 mmdA 512 methylmalonyl-CoA decarboxylase
                     alpha subunit"
                     /translation="YAEATVPKISLITRKAYGGAYIVMNSKHLKGDINYAWPTAEIAV
                     MGPESAVEIIFRHEKDQQTLIKEYKEKFANPFFAASYGYIDDIIVPSKTRYHFCKALE
                     LLKNKKVERIWKKHDNLPL"
                     /cog="COG4799 COG4799 526 Acetyl-CoA carboxylase,
                     carboxyltransferase component (subunits alpha and beta)"
                     /besthit="qcoverage 100 , hcoverage 44.8148148148148 ,
                     similarity 100 , identity 100 , evalue 1.73e-80 ,
                     alnlength 121 , Bacteria , Proteobacteria , EEH12500.1
                     propionyl-CoA carboxylase, beta subunit, partial
                     [Wolbachia endosymbiont of Muscidifurax uniraptor]"
     gene            6389..6460
                     /locus_tag="wEsol_tRNA-07"
     tRNA            6389..6460
                     /locus_tag="wEsol_tRNA-07"
                     /gene="tRNA-Glu"
                     /intronpos="0..0"
                     /program="tRNAscan-SE"
                     /cove_score="62.3"
                     /evidence=predicted
                     /type="Glu"
```

```
                     /anticodon=TTC
                     /product="tRNA-Glu (TTC)"
     gene            7385..7458
                     /locus_tag="wEsol_tRNA-08"
     tRNA            7385..7458
                     /locus_tag="wEsol_tRNA-08"
                     /gene="tRNA-Pro"
                     /intronpos="0..0"
                     /program="tRNAscan-SE"
                     /cove_score="75.8"
                     /evidence=predicted
                     /type="Pro"
                     /anticodon=TGG
                     /product="tRNA-Pro (TGG)"
     gene            14175..14247
                     /locus_tag="wEsol_tRNA-09"
     tRNA            14175..14247
                     /locus_tag="wEsol_tRNA-09"
                     /gene="tRNA-Val"
                     /intronpos="0..0"
                     /program="tRNAscan-SE"
                     /cove_score="69.8"
                     /evidence=predicted
                     /type="Val"
                     /anticodon=TAC
                     /product="tRNA-Val (TAC)"
     gene            58913..58989
                     /locus_tag="wEsol_tRNA-10"
     tRNA            58913..58989
                     /locus_tag="wEsol_tRNA-10"
                     /gene="tRNA-Met"
                     /intronpos="0..0"
                     /program="tRNAscan-SE"
                     /cove_score="75.9"
                     /evidence=predicted
                     /type="Met"
                     /anticodon=CAT
                     /product="tRNA-Met (CAT)"
     gene            60666..60738
                     /locus_tag="wEsol_tRNA-11"
     tRNA            60666..60738
                     /locus_tag="wEsol_tRNA-11"
                     /gene="tRNA-Thr"
                     /intronpos="0..0"
                     /program="tRNAscan-SE"
                     /cove_score="71.6"
                     /evidence=predicted
                     /type="Thr"
                     /anticodon=GGT
                     /product="tRNA-Thr (GGT)"
ORIGIN
        1 gtttgtctaa cgcttaagcc ctgagaaact attttttctg caatactttc cgcattttca
       61 acattaatca acgctcttgc atgacccata gacaattttt tctcattgat cattgttttc
      121 accctatcag ggagcaacaa catccgaatc atattggtta tgtggctgcg gcttttgcct
      181 atagctgaag ctaattcctc atgtgtatag aaaaactcat ctatcagttt tctgtaagct
      241 tcaccttcct ctattggatt aatatcttgc ctttgtatgt tttcaataat ggatacttcc
      301 aagcattcct tgtcgctcag atcttttatg ataaccggtg cactatcaag atttgcaatc
      361 ttgcttgctc gccaacgacg ttctccagct attatttcat aaccatcatc atttgaatct
      421 ttgcgtacca cgataggctg tataatgcca cttttcttta tcgaatttgc aagttctctt
      481 aatgactcct cgtcaaaatg tttcctcggt tgaaatttgc ttgggtgcag taatgaaata
```

541 ggcaaatgct cttgtcgatc ttctttatta tcataattat cgcctatgag gccggcaaga
601 cctctgccga ggcgtctatc atccttcata ctacgccctc gcttactagt ttcttctcct
661 tacaactgct cgcgtgcttt tttaaaattt cccttgccaa acttatatat gcttgtgcac
721 caggacactt aagatcatac acaatagcgg gttttccatg agaaggcgct tctgataacc
781 gtacgttacg tggaataaca gtctcataca atgggatgat agttttgtat actttatcat
841 ttaaatactg gcaaatatcg tttttaatct gttcactgag tttgttgcgc ctgtcataca
901 ttgttaacac tatcccttct attgccaaaa aagggtttag gttatttctt tttatcagct
961 ctacagtttt aactaaatgg cttaatccct ccaaagcaaa aaattcacac tgaagaggga
1021 caataataga attagcagca gtcaaagcat ttatggttag aagcccaagt gatggagggc
1081 aatcgatgat tatatactca taattatcgc gtatcttctc taatgcgctt tttagcacaa
1141 attttcctcg ctcaagttgc gataattcaa tttctgcagc tgataaatca actactgacg
1201 aaattagtga taaatttgga atttccttta tattgaaaat tgccgattct atcaatttat
1261 tttcgctgct tagtagtatt ttgtatatat tttttcttc cctactacga taagaaatcc
1321 cgagcccggt gctagcattt ccttgaggat caagatctac caataaagtg ctttttccca
1381 cagcagcaaa ggctgtcgat aaatttatac tagttgtggt tttgccaact ccaccctttt
1441 gatttactat tgcaataatc ttgctcacgc cttttatctt atgtataaaa taattctatt
1501 gtatatatgc aagaaacttt tgcattagaa aattttttg ttgttaacca cactaaaatt
1561 agactgtctt ttttgctttg tgtaacacac tttgaacctt gtttcctaat tcattcagag
1621 tgaatggttt tggcaaaaag tgaaaatctt ctatattgat gtcatcactc tttaaaaaag
1681 catcttctgc atacccagaa ataaaaataa cgttgatatt tggcctatgg atcaatgctt
1741 ctttaactat ttctgggccg ctcacctctg gcatgattac atcagtgatt ataagatcta
1801 tgtgttgact tttttactg attatttcta gcgcctcgct gcctatgctt gcttctatta
1861 catcaaatcc ttttctttg agtgctttag agatgaattc ccttactgaa tcttcatctt
1921 cgattaataa aattatacca ttacctttaa tttcacttac taccggtttt tctgtttctt
1981 cactatcttc ctctctatca ttttcatctg atatgtaaac catgggcaaa aatatgctaa
2041 atttagttcc atgatttact ttgctagcaa catagatgta tccttcagtt tgtttaataa
2101 tgccatatac agtagagagg ccaagacctg taccagaggt aatatctttg gtagaaaaaa
2161 atggatcaaa gatcttttca gttgtatcac tcgtcattcc acatccagta tcaattactt
2221 caatcacaac ataatttcca tgttcaatcg cttccttgtc tggagaaaac atatcctgag
2281 gtgtagaatt taatgagtca atcttttgat taaaggttcg tatagttaat tctccgccct
2341 tttccatagc agcactagca ttgactgcta agttaagtat aacttgctct aattgccctt
2401 gatcagccct aacagcaccc aagtctctac cataataaat attaaatttt atattttcac
2461 ctattaatct ttttatcatt tcataaagat tagctatagt attatttaca tcaataattt
2521 ttggctgcaa ggtctgtctt cttgagaaag caagcaattg tcttactaaa tttgatccac
2581 gctttgcatt ttgctgtatc tgtattatat ctccaaaaga tgggtcacca gctgagtgtt
2641 ggagtaaaag caaatcgcaa aatcctatta tcccagtcaa tatattgttg aaatcatgcg
2701 caataccacc tgctaactgc cctatagctt gcatcttttg ataatgctca agctttatct
2761 ctaggttttt gtgttcagta ttgtcaataa aataacaaag tatgaagatc atttattat
2821 gaagaaactt attgaaatat atttttatgt tattaccgcc gttgagctgt acatcaaagg
2881 acgcattatt tattctatta ctcgaaaaat attcacgtat tttcacatga taactatcca
2941 atatcaatgt aaagattgaa ttattatctg accctgcaag ctttattagt gccgtatttt
3001 tctttacaaa gttgccgttt atgtcacatt gtgcaatagc aattgatgaa tttgcaaaac
3061 aagggtgtaa ttgataatca acaagatttg attcggttgg tgttataaag ccatatatat
3121 agctatggtt gtatttatca caaaatatag cagtgctcat ataagccttg aatggaatac
3181 cacttatagt aaaaaacaaa atttcattat cggttattgt attgttatac ttagattgag
3241 atataaaatc actgattgag ctaccttttt ccagtttttt cagttcaaat atgtctaaaa
3301 atcttttatt tacagataaa atcactcctt tggcatttgc aatgtaagtt cctatactgt
3361 gtttttctat taactcttca tatatctgct ccttgtttat ctgtgttgct tttaacacaa
3421 aatacccatc tggccttgct attggcacta atgataaatt taaaatctta ttactattta
3481 tagcaatttg gggattatct ggtattggct caaagagcaa aagaaagtta gatactctat
3541 tcttcttgtt taaggaaatg catacctgta cagaagagtt attttttagt gcatgataga
3601 gtactttttt gtctgcttct gaaacatctc ctatttcaag aatcttgcct aacgtaatgt
3661 catcatctat tatatgatct ttaaaccttt cgtagaacct tgcatcagca taaataacac
3721 cctcatcttt atgtaaaata aggtaaaatt ctgtattatg atttagtgca tttgcaaata
3781 ttgcattttg aaattctata gtattaagta ggtatctgta gcgttttaca ttatacatca
3841 taaagagagt tgttagaatg ctaactaata aattaatctc tatattaata tgctgactat
3901 atatatcaaa aaagtagagt atcgatattg ctactggagg taagagcata ataactgcca
3961 tcacaacaat tgacattcca taatttctta gtatgaagtc aaccctgttc tctttttttgt
4021 tatctatgta tcttttactc attattgtta cttggcattg ttgtacatac gaaaatgttt
4081 tattatattg atttaattat tatagattta ttaacgtctt tgatagataa ttatcatcca

4141 aatctaatta agttccttaa atatcagata taacaatgtt aataagctag cgagagggta
4201 ttatggaaaa acaaagaaca caagcattta aatggttctg tgcactaaga gataagatca
4261 tagaatcttt cttattaatt gaaaaacaat catccgcaga gccaaagatc gaaaagagaa
4321 agtgggatcg cccaggtggt ggaggtggtg aatctacaat tatttttggt aatgtttttg
4381 aaaaagttgg agtaaacgtt tcaaaagtac acggaaaatt tgcagattca gccatcaatg
4441 aaattcctgg tgcaagtgaa agtaacggag agttctgggc aagcggtata tctttagtgt
4501 ctcatatgca atcgcccctt atccctgcag cgcatatgaa cacaaggctg atatatactt
4561 caaaacaatg gtttggtgga ggaatggatt ttacgccaat atatagaaat gaagaagatt
4621 gcaagtatat tcatgaatca atcaaaatga catgtgatag atttgacacc ggatattatc
4681 caaaatttaa agagcaatgt gacaattatt ttttcttaca acacagaaaa gagccacgtg
4741 gcattggtgg catttttctat gataatctga gttctggcaa ctgggaaaac gattttgagt
4801 tcacaaaagc agtaggtgaa gcctttttgg aaatttattc gcacgtcata cgtaaacata
4861 tgcaaaaatc ttggacaaaa gaacaacgag aaaatcaatt aataaaacgt ggcagatatg
4921 tagaattcaa tctgttatac gatcgtggca caaggtttgg tttaatgacg gacggcaatc
4981 cagatgcaat tatgatgtca atgccaccac ttgttaagtg gttatagaga gctgctatta
5041 gtagaaaata aatcatcata gtttaaaatt gcaccatgat agtgagcttt tatatagggg
5101 gtataatcat gggcataata gataattttt taattaaatc tgtcaaaaac aatgacataa
5161 aaggtgttaa actgactcta attctctatg atgcattaaa tagttttaga aaattctttt
5221 caaagactat gtctctcaag aatacaggat catttgatat aaataataat caagctttaa
5281 atcaggcttt aggacatgct acagatcaca aaataatgga attgctgatt aatcgtggag
5341 ctgatatcca ctcaatagat gagaatggaa agaatgttat aattcgtgtt attgagagtt
5401 tagcagaatc aaaaaatcta aaattggata gtgagaaaaa cttaccagca gagataattc
5461 ttcaccccac agtttccttt ttactaggaa aaggcgcaga gtattattct acagatgatt
5521 tgaatgttga agctatacaa caaaaacctc ctagaacctc tgaatatgag ccaataacta
5581 tgtacgctga ggtgaacaac aatgtaatac cacctcaaaa accttctaga acttttgagt
5641 ataagccaaa gagcactaat tatgatgctt tgccttcaaa agagccaatt tacgctgagg
5701 tgtatgatgc taaggttagt aactcattag gggagaatgg ttatttgagt gtatcagaag
5761 aatctattta tgctgaaata tatgattcac ataaaaaatc cactgaaaat ttatcattgg
5821 aagatagtgg ttatttgagt atatcagaag aacctattta ttgcactata gatgatcttt
5881 cagaacaaaa aaagcctgct agcctaaaac agaaagagcc agaacagtcg tcagaaccta
5941 tttatgctag ggtagatttg tccaagaaaa gagcagaaag aaaagcaaaa gacataaaag
6001 ctaattctgg aacttgccat gggttagtgc aagataaagt gaatgattgg caagtgaagc
6061 taaatgagct aagtgctaat aagtcgataa gtcagcaaca gactaatgat caaaaaatta
6121 agaccaaggc tggctttagt gtagaagaaa taaggcagaa atatgagcgt agagattctg
6181 gctacgattc tggctatgat tccgatacaa aagttgaaca acaattagaa ttagctccta
6241 aatctgagat aggttcagtg agtattgagc atggagtaat aggaggctgt cgatataaga
6301 ttactgatat atccctctaa ctatgtgatt caaaataatt gaattgactt tatatatgta
6361 atcgatataa cctagttaga ttcaagttga ctctttcgtc tagtggttag gacaccaccc
6421 tttcacggtg gtaacacggg ttcaaatccc gtaagagtca ctcaaataga atataattaa
6481 agttttaaat gggaaaaatt aggctttatg ttgaagaagc tttatcacaa ggcgtgagtt
6541 tagcacttaa tccacaacaa agtcactata tttgcaatgt aatgcggctt aagaagtatg
6601 ataatctctc tctttttaat ggaaaagatg gagaatggtt aggagaagta gttaatatat
6661 cgcgtaaatt gacaaaaatt acactcaaag aatgtgccaa acaacagcaa tatgaggaaa
6721 atttatacct gtattgtgcc atggtaaaaa gtgctgcttt gaacaacata gtaagacagg
6781 caactgaaat gggaataact tgcattcaat ttatttcaac ggaacgtaca gtagtaaaaa
6841 acattaacct aagtagagca aaattacaag caatcgaagc agcggaataa tccggtagga
6901 tgagtatacc agagattttg cctcctatta acttttgcga attacctgat tcccagagta
6961 aaaattttgt tttatgtgat gaaacaggta aagctgataa agtgctgaaa ggtaagaaaa
7021 atgttgctat tattgttggc cctgaaggtg gtttttcatc ttatgaactt gatcttgccg
7081 ataagttttg tcaaaaattg agtctaggaa aaagaatttt aagagtcgat accgctgtgg
7141 ttgctgcact aactttcacc aattggtgta gctagatttt taaatatggc taagataaag
7201 aaatctggtt cagtgttgcg cgcacaaaaa ccctgtagac tttctacgaa gcagtctgtt
7261 gttaaagagg agctaataaa aaacatattc taagataata ttaaatctca tattatattt
7321 tttctaaagt aaacaaatga atcttgcatt ttgcctgagt tattgatact ctgagtaaag
7381 aagtcggggc gtagcgcagc ctggtagcgc atttggtttg ggaccaaagg gtcgggagtt
7441 caaatctctc cgccccgatg ttagtataat tgtaagaacg ctgtaacata agagtaatct
7501 ccatcagtaa gcaagtagct gacactggaa tgacattatt caccatgtaa attatggcat
7561 attacatttt aacgctcaag tttataaaat aattgacaat gttaattttc taatttataa
7621 atagattttg aaatgattac cgaaggaggt atagagtttg atagaagtat cagtccatta
7681 tggtgatgta gacagagctc ttccagtatt gaaaaaaaca attcaaaagg aaggaagagg

7741 gttgaaaatg aaaaaacaat atcatgaaaa aaaatcagaa aaaagagcta aaaagaaagc
7801 tgaagctaaa aaaaagaggt accaacaaga gtgcagaagg cagcgttatg gttggtagtt
7861 tgtactcagg ttaatagttt atcattggtt attttatgaa tattcacgaa tatcaagcga
7921 aagaagtttt gcataaattt aatgtcccag tgccaaaagg ttttgttgct acatctgcgg
7981 aagaagtaaa gactcaagta agtcaattaa aatctgacgt gtttgtagtt aaagctcaaa
8041 ttcatgcagg tggtaggggt aaagccggtg gcgtaaagct agcaaagtca gttgaagaag
8101 ctcaacagtt tgtaaaggac atgattggta tgactttagt tactcatcaa acagggccaa
8161 gcgggcagca agtaagaaaa gtgtacgttg aagaaggctc aagcattaag aaagagtatt
8221 atttaagcct ggtaatcgac ccgaagctca gtaggccaat attcatattt tcctcagaag
8281 gtggaatgga cattgaagaa gtggcgaaaa attctcctgc aaaaattgtg aaatttgata
8341 ttgattctgc tacaagtttt gatagcagca agttaagcag cagttttgat ttgagtccag
8401 aacaaataga aaagatatcg aacattgcaa aaaaaatata tgatgcgttt attgcaactg
8461 acgcgagcca aattgaaatt aatccactgg ttgaaacaaa ttctggagat tttattgcgc
8521 tcgatgcgaa aattaatttc gatgacaatg ctttatatcg tcacccagaa attatggaac
8581 ttcgtgatta tgatgaagaa gtgaaagaag agatagaggc ttcaaaatat gggctcagtt
8641 acatcaaaat ggatggcagt attggttgca tggtaaacgg tgcaggtctt gctatggcaa
8701 caatggatat aataaaatac tacggagcag agcctgctaa ctttttggat gttggtggtg
8761 gagcgagtaa agaaactgta actgaagcat ttaaaatcat attgtctgat agcaacgtaa
8821 aaggaattct ggttaacata tttggtggta taatgcgttg cgatattatc gcaagtggaa
8881 ttgttgcggc tgcaaaagaa atgagcatta aagttcctct agtggttaga ttatcaggta
8941 ctaattttga agaaggaaaa aaaatcttag aagagtcagg attaaatatt attgctgcag
9001 atgaactggg tgatgctgca caaaagatag taaaagaggt aaaataaagc atgtccgttt
9061 tagtaaacaa agatacaaga ttaatatgcc agggttttac tggtgcacaa ggcacatttc
9121 attcagagca agcaattgat tacggaacga aaatggtggg aggtgtaact cctggaaagg
9181 gtggtagcac tcaccttaat ttaccggttt ttaatactgt agcagaagcc aaagaaaaaa
9241 ccggtgcaaa tgctacagtt atatatgtac ctgctaaatt tgctgctgct gcaatacttg
9301 aagcaataga tgcaaaaata gaattgataa tttgtattac agagggcatt cctatacttg
9361 atatggtgaa agttaagcgt gcgcttatcg gctcaaaaag tcgattgatt ggtcccaact
9421 gtccgggaat tattacacct gaagaatgca agataggaat tatgcctggc catatccata
9481 agcgtggaca cataggaatt atgtctcgct ccggaacttt aacttacgag gcagtagcac
9541 aaacaaccgc tgttggtctt ggtcaatcca cgtgtatcgg aattgggggg gatccaattc
9601 atggtatgac atttgtcgac tgtatggagc tctttttaaa agatgacgat actcatggta
9661 ttgtagttat tggtgaaata ggtggaaacg aagaagaaga tgtatcacat tttgtgaaaa
9721 cagaaaaaac taaaaagcca atcgttggtt ttgtagcagg ccaaacggca cctccaggaa
9781 gacgtatggg acacgctgga gcaatcatct cctccagcgg tggaagtgct ggtgcaaaac
9841 tagaagttat gcggagtgcc gggattgcaa ttgctgaaac ccctgcagta attggtaaga
9901 aggtattgga agtaatgcat cactagaatt agggtcacaa aaaccctata tcaccggtca
9961 tgaatacgtt atttgcccat tcgattaata acttgcctcc tggtaaattt actgaagtct
10021 gttgggcaac cagatatcca cgtagtgtgg atgcaacaag tgctgcacag gctgcactac
10081 cgcatgatgc agtaatgcct gtacctcttt cccaaactct taaagctatt tctccagact
10141 tttcaacttg tgcaatacta acattaattt tccgaggaaa taatgcgtga ttttctagct
10201 ttggtcctaa atttcgcaat ggtatttcac ttatgttatc aacaaaaaaa actatgtgag
10261 gattgccaat atttactgca acaggatctt ttagcatttc aagctctata ggtaagtgaa
10321 gagtgtcaca ctcagtagac aagggaattt catgccattt tagcaaaggt ttacccatgt
10381 taacctttat ggatttatca cccactttaa aacactctaa aatgcgtttg cttgttagct
10441 caataatgga gtattcagca cctttttctg acattatcaa atatccaaca cagcgtgctg
10501 cgtttccgca catttcagct cgactgccat cagcattata gatatgcata aaacagtctg
10561 cagcattaga cattgttata attatcactt gatcacaact gctttgatca gcaatttgtc
10621 tatggttcca gtctaaattg tttgttgaac gtgagtctat gataacgaaa ttgttaccag
10681 tgccgtgcat tttgataaaa ttttcagaat tttggccact catcataaaa aaatatttct
10741 tctattggtc ttcttttctt cttacttgtt tcagcaccct cttcttcgta accaaccgct
10801 atcactgaca ttacgttaaa gtcattgggt atattgaact ctttcactat tttatctcta
10861 tcaaatccac ccatttgatg ggccattaag ttcataactg cagcttgtaa cataagtcca
10921 taatttgctg caccagtatc atgattagcc caaagatttt ctcctttacc ttgtttacgg
10981 aagtttttag cacttaaaga tatgattaac acttgtgcat cttttgccca tttttgatta
11041 gattcatcaa ggcaatttag cagcttttcc catgcgcttt gattgttctg tttgttgcat
11101 attacgtatc tccagggttc atcaccaaaa catgagggag tcagccttac agcttccatt
11161 aaaatatcca tgtcttcctg agatattgct cttgtaggat cgtatgaacg tccgctgtgt
11221 cttgctttca tcaacgataa tagatcttgt ttactcatca tttttaattt aatagtgcat
11281 aaataattca ttgtagcaca atataagcaa aatttaaatt gcaaaacctc tcatacacat

11341 atattacatt taataagtta tttttattgg gaaggtatgt ttataacaca atctagcagc
11401 agatcaacac tggctgaaat attgtcttgc gttttacttt tattcttgat ggctcaaata
11461 agcataccat tgcagcctgt gcctatcaca ttgcaaactt taggagtaat gcttattggg
11521 cttaaattta accgcagaac agcattctat tctgtgctta catatctatc acttggtgca
11581 gcaggattgc ctgttcttgc gaatttttct ggtggttatc acgctcttct tggaccaacc
11641 ggtggatatt taattggctg tttagctgct gttatggtaa tgagcaaagt aaatgaatta
11701 ctgaactcta agtataaatc atttgtatgt aattctttaa gttgtctggc tggtacagtt
11761 gtaatcttta tttgtggtgt tagttggctt gctgtttact tgggtctggg acaagcaata
11821 atggtgggtg ttttaccatt tatccttcct ggtttgataa aaatttttct acttgtagca
11881 gctttgcaat atttgaaaaa atgataaaat ttcgccctca tcatttcatg tgcaccctcg
11941 cgtttcgggg atatgggtat tctcagggtt ttgtagaaaa ttataaaagg atagcaagca
12001 aagtaattag tactcaaatc gaagtagttg gtaaccttga taatatctgt agtgcttgtc
12061 caaatcagac taaacaaggt aaatgcacca cacaagctaa agttttagag ctagatagaa
12121 ggcatatgga aattttagga attaaaattg gcgagacttt aacttggaat gaggcagtaa
12181 aaaagattag ggagaaaatg tctttaaaga aattcgagta cgcatgtgag ggtgcaact
12241 ggcagccata tggaatatgt aaaaatgccc ttttaaatct acaaaggtag ttgacaaaat
12301 gttaagataa attgaagcat ttttgtggaa ataaagcgaa atatggaaga caaaagataa
12361 atgcattctg aaaagctaca cagcatgttc gtcccgctaa ttaataatat ctttattatt
12421 gtatgagata ggtaatttag catttatgaa tttgtaatgt gttacgcata gctcaaagaa
12481 ataaggttta ctggttacac gctgcagcca atgccacaac actaggacct atgtttatct
12541 gcatgttaat ttgcgttgtg attttatgga ggtcttctgt tacctatgct agtgaaaatt
12601 tggagataca aaaggccttc gatagtgttg tcaagcatat aaaagctgat aaaaaatata
12661 aagattttga tgttattgag agaaaaagtg acaaatttaa tatcaaaatt tcgcagaatt
12721 ctggcaagag ttttgacata tactctactt taaaaaaagc aaaagattcc tttgaatcag
12781 gagataatga agcagctatt tctctcctta atcagattat cgcaaagttt ccttatcata
12841 aaaatgcttt aattggattg ggaaatattt attacgctaa taaagaatac aaaaaagctg
12901 tagagattta cacaagactg ttaaaagaac accctagtaa cccttacata ttaaaaaatt
12961 ttctgacgat aatctcacaa tatgatcctg atctggcgtt gagtgaaatg ttaaaattgt
13021 atgatataca taaaaattat gctcctttat tagcaaattt aggtctgatc tatatgaaaa
13081 aggaggatta tgtaaaaggt aaagagtata tgataactgc tatttccctc gatgaaaaca
13141 atattttta tacttataat ttagctgtta ttctagataa actttcagat tttcaaaatg
13201 ctacaatatt ctacttaaag ttgttaaaca tggctacaac ttcgaaagat gtgagtgaaa
13261 aaatacctt atacaaagtg acagcaagac taaaatttat aaaacttcac agcacgcacc
13321 caaagatttc ataaaagctt atagcttacc taaagctaat ttatcgtatc tgttcaaggg
13381 agtggctaat gtgcaaatat tgaagcaaag tgtgtgacac acagctgtac gaacatttgt
13441 ttgcaaaggt aatttgcaca acaaatgcat aactgtacga acattgtgat tttggcccat
13501 caggagggtg tcatcccagt gcttgacact gggatggctt tgttgcatcg ctacttatga
13561 aaggctaact atagttagga ttataagcaa cctattgaaa atcttgtttt tttgcaatca
13621 atctgatcaa atttaagaat agtaatttgt tatttatatt aataaattta attctattga
13681 aaatagctaa agcattgaaa ttcttgaatt ttagccggat tggtaaatgg tagtgaaatt
13741 tcttttactc aaatttaggt attcattgac cgttcttgtt ataaatacca gaataataag
13801 ctactgatat tccctatctt ttttatcttt aatccatcat agctagcgat gcaacaaagc
13861 ccactgggat ccaggaattt tattaagttg gcgagcataa aaatagctgt tttatgtaaa
13921 aatacaacgt tttaatgatt atgaaaaagc tggattccag tgtcaagcac tggaatgaca
13981 ccattctttt ttctggatcc cagtgtcggc tacttggatg acaccattta ttgtaaggac
14041 ttacctcaca tttactctat gattatcttt ttagatcacc ttgtctacct atttgccac
14101 tctgctgaac ggatacaatt tattgctatt tttgtttaca ataataatat tgagctatag
14161 aattagatct aaaggggcaa ttagctcagc tggtagagca tctcgtttac accgaggagg
14221 tcggcagttc aagtctgtca ttgcccattt gcttaactga atgttagtat tattaattac
14281 ttcaattaaa gtatagaaaa tgcaggataa tatagtacca atctcaatag taaaagagct
14341 ggaagattct tatctttcct acgcaatgag cgttatcata agccgggcta tacctgacgt
14401 gcgggatgga tttaaacctg ttcacaggcg catattatat gcaatgtcaa gagctggatt
14461 cgatgccggt aagccatata aaaaggcagc tcgtatagtt ggggatgtaa tggggaaata
14521 tcacccacat ggtgatgcag ctatttacga ttccttggtt agaatggccc aagatttctc
14581 tcttctttta ccacttgttg atgggcaagg taactttggt tcgatagatg gagatccacc
14641 agcttcaatg cgatacacag aagcaagact tcaaaaagtg tcgcactttt tactgaatga
14701 tattgatgaa gatacagtta attttaggcc aaactacgat ggaaatgaaa cagaaccagt
14761 tgtgctgcct gcagagtttc cgaatctatt ggtaaacggt gcaagtggtg ttgcagttgg
14821 tatggcaacc aatattcctt ctcataatct tggagaaata atagatgctt gtatattata
14881 tgtaaataat cctgaagtta ctttggatga gttacttgaa gtgatgccag ggccggattt

14941 tccaactggg ggaacaattc ttggaaggtc tggaataaga tcagcatttg caactggtcg
15001 tggatcaatt gttgtgcaag gcaagactca tatagaagat ctgccacaag ataggcaagc
15061 aatagtgatt gatgaaatac cttaccaggt aaataaagta aaattaattg agaagatagg
15121 tgagcttgta aaagaaaaaa gaattgatgg tataacagaa attagggatg agtccgataa
15181 gtctggtatt agagtagtaa ttgacctcaa aagaaatgct gaagcaagtt ttatacttaa
15241 tcaaatattg gggctaactc cactaagaag tagttttagt gttaacactt tagtgctcaa
15301 taacaacagg cctgctttga tgtcattgaa agaaatcata gaggccttta ttgattttag
15361 aaaagaagtg ttaatcagga gaacagaatt tcgtctaaga aaaacgagag aaaaagctca
15421 tatatatata gggctctaca ttgcggtcct cagcatagat gaagtgataa aaatcatccg
15481 cggtgcaaaa gatcctgcgg aagcaagtag agaactttta aataaagaat ggaaaacttc
15541 gaatgaaatc aacactatta tcgagttgat ttcagatagc gcaagttttt tgaaagatgg
15601 agtgtataga ttaactgagc tacaaattaa agctatcctt gatatgaaac tgcagcgttt
15661 aacaggcctt gaaaaagaca aattagaagc tgagctaagt tcaatgatca acctgataaa
15721 agaatatatc gcttttcttg gctcaggaga gaagttgatg aaagaaataa aaacaatct
15781 acaagaaata aagaacagat ttgctgtgcc gcgaaaaact tcaatagaag agtcagacgt
15841 ggacattgaa gctgaagatc taattccaca agaggatatg gtgataaccg taactatgaa
15901 tggttatatc aagcgtgtaa aactttctca ctacagaact cagcgtcgtg gtggaaaagg
15961 aaagctagga cagggattaa aagaagagga tgtaatcaca gaattatttg ttggaaatac
16021 ccatactagc tttttattct tttctaacat tggtcgagtt tatagattaa aagtttataa
16081 gttacctctt gcagagccaa ccgcacgtgg aagagcactt gttaacatat ttccgcttag
16141 cgatggtgaa actataacta atatcatgcc attgccaagt gaaaatgacg aaaatcaaaa
16201 catagttttt gctactgcct atggaaacat aaggcgtaac tccttagcgg actttcacta
16261 tattccaagc aatggaaaaa tagcaataaa gctcgatgaa ggagacaaat tagtatcggt
16321 taaggtatgt aatgaaattg accatgtttt actttcaaca acgcttggaa aaagtatcag
16381 atttgttgta agtgatgtgc gtcaatttaa gagtcgcaat tcagatggcg taagaggtat
16441 caaacttgta aagaatgaca gcgtgatatc catgaccata ttaaatggga taggtgtaac
16501 aacagaaaca aaagaacttt acttaaaagt tccacttgca aaaagactgg ggactgcaat
16561 taataactcc attgattcta aattggaaaa gactttgaat gatttaggaa tagataacga
16621 attattctta aaacttgcag tgaatgagga gtttatatta actattactg aaaatggctt
16681 tggtaaaaga acttctgcgt atgaatatag agtaactaac aggggtggtg ttggtatcac
16741 caatattctt actactagca gaaacggtaa tgttgttgct agttttccag ttgagcaggg
16801 tgataatata atgcttatta cagataaagg aaagttaatt cgcatttcgg tcaatgagat
16861 tagaattgca ggacgcagca ctcaaggagt cactctgttt aaaacagaaa gtagagaaaa
16921 agtggtgtca gtagcgaaaa ttgaagatcc tgattccact gaagatagta tttttgaagt
16981 tgaaaactct atctcttctc aaccgtagta caggttaaac aaaaagcgca agtttttgtt
17041 gattaatacg cagtatttaa attaaaatat atacttatgg ctaagtaaag atcaatggac
17101 aaaaatttaa ctaaaaataa aaaaccactg gcaaacaaag aggataaaaa aacacctcct
17161 gttaaagacc ttactaaaaa taagagtagg agagatttta taacattaac tacatgcgct
17221 atggcaggta taggagctgc aagcggactt tggccactgg ttaaatctat gaatccttct
17281 gctgaagttc tagcaacttc cacagtggag gtgaatttgt ctggaattca ggaaggacac
17341 ggagtaaaag taaagtggca aggtaaacca gtattcattc gtagacgcac aaagcaagaa
17401 attgaggctg caagagctgt gaatgtgaag agcttgaggg atcctcaatt agatgagcag
17461 agagtatacg aaggaaaaga cgaatggtta attatgcttg gaatatgtac acatcttggg
17521 tgtgtaccgg ttgaccacgc tacaagagat ggtaatggtt ggttctgtcc ttgccacggt
17581 tcatattatg atacatcagg ccgagtgatt ggggggtcctg caccgaaaaa tatggctgta
17641 cctgattatt tttttcccag tgagaatgtt gtggtaattg gcaagaaggc ttaacggaac
17701 tcgttaaaaa aaactcacag gatcaacatc tattatcact gcaatactcg aattcaagtt
17761 acaattttta atccaatatt tcagcttttt ttgtatagat aaactgtgtt tattgtgtat
17821 ttttaataat accctatgcc gatacttatt atttaaaaaa tttattgctg ctggtgatgg
17881 gccaagaatt tcaaattcct ttttgtctac acctacattt ttttcctgga tacaagtagt
17941 taagctactt ggataacaaa gaagagattt tactatttca ttcgctgctt tttgtgttgc
18001 aatctgattc ttaccacaaa ttataagcgc tattaatctg ctaaatggtg gcattttggc
18061 ttcgcgtctt gacttaagtt cgatttcata aaatgagtct ctttttttgat gcagcaatgc
18121 ttttattatt gaactttcag gattattagt ttgcactatt accattcctt tttcattaaa
18181 ccttcctgat ctgcctgcaa cttggtgcaa taattgatac gtcttttccg ctgctcttaa
18241 atcagaattt tcgaggctca aatctgcatt tattactcca accaaagtta atttgggaaa
18301 attgtgcccc ttagcaatta tctgtgtgcc gattataatg ttcacctctt cctttaacac
18361 taaatcaatg atgttactaa ccgacttctg atcgctgctt atcatcgcag tttttgcgtt
18421 tggtattaat ttcaccattt cttcaagtaa cctttcaatt cctatgccgt agagggacaa
18481 tggctgttca ctttggcaat tagagcattt ttctggaagt ttcaattgat aagagcagtg

18541 gtggcacaaa agagcatttt ttttcttgtg atatgtaagc caaacagcac aatttaagca
18601 ggaaatttta tatccacact ttttacaaat tgccagttgt gcataccctc tgcggtttag
18661 aaaaagcata acctgctgtt ttttctctat ggtttgtttg atgctttcaa aaagctcaga
18721 ggagatccat tgcttgttgt tcctcatatc tactacttta atgagtggca attctgcacc
18781 accaaatcgc ttagttagct ttacatgatt gtaattcccg tttttaacat gatgaatcgt
18841 ttcaagcagt ggagtagctg atgataaaat aatcggaata ttttcaaatt tggctaagat
18901 tatcgccata tctcgagcat tatatataat tccctgttct tgtttaaaag atgagtcgtg
18961 ctcttcatca acgataatca attttaggtt tttataaggc aaaaaaagcg ctgaccgtgc
19021 accaataatt atctgcgcat ttccatttgc tatattgagc caattattcc ggcgagtttt
19081 tggagtgagc cctgagtgcc attcaactaa gtttttttgat atctgaccgc gaacacgatt
19141 taccaattgt gaagttaaaa caatttcagg taggaggatt aaaatttgtg tattaccttc
19201 tcctgatgtc atttcaacat attctggtgt catactagta cgtggcaaag acggtatgct
19261 aaatgttaca gtgggttttg ccttgagtat aaaggtgtca ttccagtgct tgacactgga
19321 atccaggaat tttgattgta aattaggtat tggttttaag ttaaaataaa catttgtaat
19381 gaggttatat gaaaaactgg atcccagtgt caggctactc ggataacacc ctcttggtag
19441 acccaaatca caatgttcgt acagttgtgt ctctgggcac tgggatggta aagtctgagc
19501 agcatttgga acggcagtgt aattcttaat caattgtgca atcacagaga gataaacttc
19561 ggtttttcca gatcctgtct caccgtcaag caaagtcact gaatactcgt tcaaattgct
19621 tattatttta tcactagcta tctgctgttc agggcttaat tggcaactta tttcacttgt
19681 ttcctgcttt tgttcaacac aaaccaattt atctatgtga tttactttta acactcctcc
19741 cattatcact ttggcaagca taccaatagg tattaaattg tattgtgcaa cccactctgc
19801 aaatgcaatc aattttgttc taatatttgg taaatcgatt ttttgctcaa taaattttaa
19861 ttctcggtta cttttatcac tgtatttcca aactattccg attaagcgtt ttttgccaaa
19921 tggcaccact acgtaatctc caagtaaaat ctcagtattt tcctcaattg catatgaaaa
19981 taactgatca attggaagtg atagtaatac gtcaactgtt tttgccatag tgattgagtt
20041 taacattgtt ttaaagagct tgtaacttta ttaagcctgg caatatagat gaatcggtac
20101 agagaaaatc ttaacgttta acacattttt catagtacag accgtatgat tgataaataa
20161 aatgatttat agaatttgtg gaggctacca atggtcaacg acaagattac tttgaacttt
20221 gaaatagaaa aaggcgaacc tatagatcga tactactttt ctgcacatat aaagcttaca
20281 gatgaaagca aaaatctttt gcgggagaag cttggcagta caattcaatt cgataatcta
20341 gatgatcact tcaatcttaa ccatgagaat ttttatatct actacaacaa atatagtgag
20401 cacaatggta gaagtgagct acacgttagg gatgataacg gtaaatattt aacctccaat
20461 cttccactta attactatgg attctcaatg gaactatcac ctgacggagc gtcaattgca
20521 gattatggtt tttatcctct gtatgcaatc tataacccaa atcactataa taaagcaaag
20581 gaaaaagtat ttgatgatca cggtgaattg tcaccaattg catcaaatgt gttagataat
20641 tttttaagca acacaaatag cttaaccaca caagatatat tacaaaatgt attctcacaa
20701 ctgcagaaga aagctgatga agaagcagaa agattggcaa gagaaagaga agcagaaaga
20761 caaagggagg aagagttaaa tgaaaagaac gaggagttaa aagaacttgt agaaaaggat
20821 gagattaaaa tattcaaagt ggtaaaaggt aatgatttag gtaatgggca ttcagcagta
20881 gttttacaac ttgatgggga agaaggtgac aagatacctc tcgactctag taaatactac
20941 atagcagagt atagacacca cccagaatgg gaaggcgatg aatatttaaa ttttgttagc
21001 cctgatagtg atatgctggt taattatgaa aacttatttt ttgtgctcaa tcaaagctcc
21061 tactcagact ttgaaagtgg gttttattca gaaaacagaa ccgatcccca taattatgct
21121 aaatattcaa aggaaccata tttgagtggt gaccagctat ctgaaaaatt atctaatgaa
21181 ttggaggaag atagcaatca gataggtact gaatttacaa ttgatgatga gcaagtagac
21241 gaagaagttg tggtagagag tgaagccaag tctggggaga aaggaacggt tggtgaggat
21301 aacgcaaaaa atactataca ggaagaagaa tctaactata tagatcaaat gcaagcggat
21361 gatagcagca gttataaacc agctttgcaa agcaatcgtg atgattatgc tgatacgaaa
21421 aaagctctgc aggaaaaatt gcatggagat gaaatacaca agcaaagtaa tgagaaagct
21481 caaaaaagata tagaagtaaa aaaagtaact gtggaaactg acagtagcag taataagact
21541 gaaaaacaaa caaaaccaac agaagatgaa caagaaactg ttatggcaag tgtgatagaa
21601 aatatggata aaaattctga tattgaagca gaaacttcta acagtgttgc aaattcacaa
21661 catcccttaa aaataaagac aggaagtcct cttgaagtgg gaaagtataa ggtggagctg
21721 caagtgacat atgatgatgt aaagaatttt tatgataccg tacgtggtaa atatcatgct
21781 ggcgatggat ataactacga acaagtaatg gttttatata atgaaattgt taggcccgct
21841 cacaatcatc attacgagct gtttactctt gatgaagcac atgttgctaa tgatcacctg
21901 tttatcaagg gtcaggattt tggaactcta aatgatttta atgagatgtt ctataaaagt
21961 gatgagttaa tgtgatgtaa aaagcaagat tcaatctggt gaacgagagt taactgacag
22021 aggagattga cacgtatgcc tttatcttta agattgttta tcaagtatag acatcagctc
22081 gccccattct cttttaccaa tattactttc ttctctagta acgttttccc ctttaattaa

22141 tttgcgaatt atttctagac cagttcttga aatacaggct gattctaggc aatattcaac
22201 aaaggcattg taggccagtg gaacccattt ttttattata tccagcataa cttctgcata
22261 aactctgatt tcatattggg catgcttatc agctctaagc ttcaaaaaat gaaggagatt
22321 atgcagatct attttccaat aaaattgcgt gtagtaatta agcattaggt tagttctggc
22381 aatttctctt gcaagcccct gctcaataaa tttttcataa tgagaatata ctaaattaga
22441 gtcatttgtt agagaatcta ttatttcctt tgaggtatct ggatcaaaag cttccccgct
22501 accttgttta ttattgtcag attgttttgc aacttgttct ggtttaggtg tataaaattc
22561 attatcaagt attgaatacc ttgctgaata ttcattcaca tttgcagttc tatgccttat
22621 ccattgcctt gcaacaaaaa ttggaagttt cacgtgaaac ttaatttcac acatttcaaa
22681 tggagttgta tgatgatgtc tcattaaata ctttataagt gcttcatcct gacttatctg
22741 ttttgttccc ttgccataag aaacacgagc agcttgaact atagcgctat cagagcccat
22801 ataatccact actcgaataa atccatggtc taatacctta tgttcttcat ataagattgc
22861 atctatttct tttactatag ttcgtttggt tgcttcattc ataaaatatc ctcaagacga
22921 aatggagata tcagctccac atgaattgag tttttcatgc attgcttcat atcctctcca
22981 taaatgatgt gaattgttta ttatagtttc tccaccggct accaaagaag caagcaccaa
23041 agctgctgtt gatcttaagt cagtagcata tagattagct ccagataagc tttttattcc
23101 acttatagta gctttgcttt tctcgatgct gatattagca cctaactttc tcaattcatc
23161 tgcatgtgta aatctgtttt caaaaatgtt ctcttcaatt actgatatcc catcagcgat
23221 gcacattgca gacatcagtt gtggttgcat atcactggga aaattgggat atggatctgt
23281 cgcaacgtta gcagacttaa tagagccatt ttttctggaa ataacaatgc ctccatcata
23341 cagttcaacc atagctccta tagtctccaa ctcatttgta atacacctta tatcagatag
23401 atttattcct tccaactcca acttaccacc ggttattata gcagctagtg cataggtgcc
23461 tgcttctatg cgatctggta ttattttatg aacacatcca tttagtgctt caactcctgt
23521 tattatgacc tttgtattat caatttcaat atcagcaccc attttcttca gaaaatctat
23581 taaatcaaga acttccggct cagttgcagc attgtttatt gttgtcactc cttctgcaaa
23641 tgttgctgcc attattacat tttctgttgc gccaacactt attttctcaa atgtgatttc
23701 ttttccttgt agctttcctt tcactgttgc aattatatta tagccgtcaa tttcaatttt
23761 agctcccatt tcttctaatg ctttaatatg catatcaacg ggacgctttc caatgttgca
23821 tccaccagga aatgctgttc tcgctttgcc aaatctgcta agcattggac ccaatattag
23881 aaaagaagtt cttagcttac ttgcggtttt atatggcatc acgtgattgt tgatattgct
23941 gcagtcaatt ttcaaagtat ggttcgcttt ataattttta ttgtgcataa agttcacttc
24001 tgctccaaga ctttcaagca acttagacat tagatgcaca tcaattaaat caggtacatt
24061 atgcaaaatt accggggagc tacttaatag acttgctgcc attattggta aaacagcatt
24121 ttttgagcca ttaatcttga tttttccgac caaaggtttg tagttatttc ttattaatat
24181 cttgtgcatt taattagata taaagtataa ttcattagtt tattgaagtc ttactaagta
24241 taatagattg accgtaaaag gcaaaatttg ctttttcttc ctcatcatcc cagtgtcatg
24301 ctacctgcat gacacctgat gcgaattgcc cccgaaattt tatactatga attactcaaa
24361 tgacgctctc gagctttatc aaaccaaata acaccaattg caacaatgaa ataaccagct
24421 gctcctaatg taaggcgaat tactgcatcg aaagttgaca gaggaggaga cggcattaga
24481 atgctcaaaa cactatacac aagattattt gttgcatgag caatagttac tggccaaatc
24541 gagttgtctt tttcaaacaa ccaggtaaac attatagaca ttgcacaaat ataaagggta
24601 taagcacaaa tagtcgggaa tgtaaagcca tccttccaca aagctgagcc agcaaccaca
24661 taagttggta tgtgccatac tgaccaaata ataccgacaa taattgctct gatataaaaa
24721 tttgatattt tgcttttaag atttttcaaa agatagccac gccaaccgat ctcttcacca
24781 agagacataa tgatccacat cacaaaggtt gataataaaa caaataacat cactaatgga
24841 ctcaaaactg cactcaaatc ctttacacgg tctaacgtaa aagactctcg gaaaaatgaa
24901 aaaccgcagt aaattgacaa aagaccattg gaaattagca tacttaccag aataagtata
24961 aaccctaata gacagttttt tggcgaacta agtttaaaaa gatcccttac cttctgttta
25021 ttataaatgg ttagcaaaat tgcaactgaa gccggaacaa atgttgttaa taatgttatg
25081 tctttgaaag gtaagcacca agctataatg cacgtcaaaa aatatgtaat tacaacaaat
25141 aatgtacaat taatagtggt taaattcatg catacgccca tcaattcctt ttctgcatta
25201 ttgatcttta ttcataaatt gtcaataaaa tacttcttca gggatgaata gaatattttt
25261 tctttttgaa ttgctattga tagtttaatt tttatatgta ttccatttca caaccgtttg
25321 tcatcccagt gtcacgtgct gggatgacag taggagagct gcttgcatga caagaggagg
25381 gtactggaat gacaagagga agacactggg gtgacatcaa ctaaacttgt ccaaatatga
25441 gcgatgcatt agtgccacca aaaccaaacg aattagaaag tgcatattga attttatgct
25501 cctgagcttt aaacggtaca aaatttaaat cacatccttc tgaaggcttg tgtaaattta
25561 aggttggtgg aataatccca ttatttaatg caagaatact gaatattgcc tcaacgcttc
25621 ctgcagcacc aagcaagtgc cctgtagagg atttagttga agaaacgggt atttataaag
25681 cgtaatcgcc gaacaacttt ttcattgcta tcacttcgat tttatctcca agtggtgttg

25741 aagttccatg tgcattgata taccctactt gattgggatt aatttgtgca ctatgaagag
25801 caagttgcat tgctttaaat gcgcctcttc cttctggatg cggtgcagtg atgtggtgtg
25861 cgtctcctgt gagtccatat ccattcagtt cagcatatat ttttgctccc cttttttttg
25921 catgttcgta ttcttccaac accaatatac ctgctccttc acccataaca aaaccatcgc
25981 gttctgcatc ccatggtctg gaagcttctt tgggtttatc attgaattta gttgataacg
26041 ctttcataga tgcaaaacct gcaattccaa ctctacacag tgcactttct gctccacctg
26101 caatcatcac atccgcttca ccaagtttta tagttcttgc tgagtttatg attgcatgcg
26161 cacctgttgc acatgcggtt actgctgaat cgtttggacc tgtaaattcg tatttaatag
26221 aaatatggcc agatatcaaa tttattagac ttgcagggac aaaaaatgga ctgacacgtc
26281 tgggcccttt ttcctgcatg gtaataacat tttcctgaat tgacggaaga ccacctatac
26341 cagagccaat agtcacacca acacgttccc gattgatatt ttggctttcc aaaattaatg
26401 aatcatctac tgcctgaata gctgctgcaa taccgtaatg gataaaacga tctgtccttt
26461 ttgagtcttt ctcagaaata taatctagtg gattgaaatg gtgctcaatg ttgtctgatt
26521 gcaacggcac ctgccctgca attttgcaag caagatcaga agagtcaaat ctatccgtat
26581 tgattgcttt tatgccggat tcacctttta ttagccttaa ccaagtgttg tcaacatctg
26641 ctgctagtgg agtgattaaa ccaacaccag tgactactac tcttctgctc atttaacact
26701 aactattagc ctttttttatt attaatgtac tcaactattt gctccatagt ttccattttt
26761 tgtgcgtctt catctggaat ttctatcccg aactcttctt ctgccgccat gatgatttca
26821 actgcatcta aactgtccgt accatgctcc gaaagctttg aagagctgtt gaatttctct
26881 acatccttac taatgtgctc tagtataatc tttttcactt tttcttctat gtcttctcta
26941 gtgctctttg ctagttcgct attcacattt cttaccaaaa aaataaccta ttctttgatg
27001 ttataaaaaa tttttatgtt tgcaatatct tttataactg gtaaactaca gcaaagtttt
27061 ctactgccct agctggctag cgttacgtgc taaacaatta acaattcaaa aatgctagac
27121 gaaacaacaa aaaatttgct tattgtagaa gtaaacaaac tcttacccga aaatagcgag
27181 aataagctta tatcagctat gcgttatata ctccttgctc ctgcaaaaca tatacgctcc
27241 tttttagtca tagcttcatc gcaagtgttt aacgcggaag ctaaaaaagt aatatcagtt
27301 gctgcagcaa ttgaatttgt tcatgcttac tctctaattc acgatgattt accgtgcatg
27361 gacaacagtg atactcgcag aagccagcta agctgccata aaaaatttga tgaagcaaca
27421 gcagtgcttg ccggagatgc actacttact ctggcttttg aggtattatc ttcgttaaat
27481 gagaaacgct gtgagatcat aaaagtgctc tctcaagcaa taggaattag gggaatggtg
27541 ggaggacagg ctttagatat tgacacagat tttaacaaaa taaaagaaat tcatttgatg
27601 aaaaccgcaa aactatttgc agcttcatgt gaaataggtg ctataatagg gggtgctaca
27661 gatgaacaac ggagagcgtt atataactac gggataaacc tagggcttat ctttcaagct
27721 aaggatgata ttgaagacta cgaacaagat aaaacaaata acctaatgtc tgtgcttggt
27781 aaaagtgaag tagaggacta tatagacggc ctctttaaac aaggcttgga taatttgagt
27841 gcgctttcag gggatactaa ttatttatat gatttattga atcaagtaaa gaaagatggt
27901 tagaaaaatt atattacaaa tgcttatttc cgtagtaatg attctcactt taacttctgc
27961 ttttgtgttt actattgtct atataaacaa aggtaaaccg gaaaaaaagg acaagcttta
28021 tgagataaat gccacgcatg gtggtttgat acagacgata gcatattacc tagtgaagcc
28081 aatacttgaa tcagcacttg accgctatat tgaaaagcat gggctaacag aatatttaga
28141 agaaatgacg cagcaaaaag aagaggattc gataaatttt tatgaaatta ctgaaggcag
28201 tggcagcaag gctttttgtg gcctggaagt cctattgcaa atatataaaa tttctaataa
28261 taactcagca acactcccta gcaaagtttc tgatgttact ttgaaaatag gtcaagatga
28321 cctaaaagaa gtgagtttag gggcaatagg tatgaaagaa ggcggagagc gtgtagttac
28381 tattgccaat gagaacaaaa tgaattttaa ttcttattac gtcaaactga tcgaagtaaa
28441 ggataaatat cccgactcag taaataatct gatggttttt aatgacttaa ttaacaagac
28501 tggaaaacaa gtaaggtgcg gtgatgagat atcggttaaa tatagcgtaa aggagcataa
28561 tggggaatat atagtcaaag atcaaacagt gcaatttagg gttggcgata aaaaaatacc
28621 acttgctata gagcttggag ttgtgggaat gagagctggt aataaaagaa ctattctttc
28681 tccgcctgac cttttgacta ttactgacga tatgttaata aaagacatag attatgatga
28741 aaaaaatatc tcaataattg acttaagctt agatgttaag caagaggtcg cagcacacca
28801 ttctactgtt gaaaaacagc ctaaagaata tagctgaaat tttaggtcta ctggctaatc
28861 ttctgaacac tctctatact acttaaagta ggcttttcgt ttttcctaaa gtataaactt
28921 agataaatca agctgcttcg aaattgactc aagtttatct tttacatact tgctgtctat
28981 aatgaatttt tcgctatttt tctcagaagc aatgaaactt atttcatcta aaagcttctc
29041 catgacagtg tgaagccttc ttgcgcctat attttccact tgcctattaa ctgtaaacgc
29101 tatttcagct atggttttta taccatcatc agtgaactca agtgtcacat tttctgtttt
29161 cattaaagct atgtactgct ttaacaaact agattctggt tcctttaata ttcttattaa
29221 atcctcttga gtaagtgcct taagttctac cctgatcggt aatctgccct gcaattccgg
29281 taagaggtca gatggtttag actgatgaaa agcaccagat gcaataaata atatatagtc

29341 tgtttttaca tggccatact tagttgtaac agttgttcct tcaagtaatg gtaacagatc
29401 gcgttgcact ccttctctgt ttacttcacc ttttacttct gtacgcgctg caattttgtc
29461 tatttcatcc aaaaatacta taccgtcatt actaacaaga tcaatggctt ctttgattat
29521 cttatcttca tccattagcc tttcactttc ttcattaatt aatatttcac gcgcttcttt
29581 taccttcacc gtaatagttt ttgttttttt gctcccgtta aacatcttgc ccattatttc
29641 tgttacattc atcacaccaa cttgcccacc tggcatgcct ggtatatcga aagtaggtaa
29701 catactcttg ctctctctga cgttaataga aacttctccg tcctcaaact ctttattcct
29761 caacctttct ctaaaaattt ttttactctc ttcggttgca ttttcgccta ccatagagtt
29821 tactattgtt ttctcagcta aaattaaagc ctttttagct aaggctttac gggctttctc
29881 tttaactaaa actattgctg catcaactaa atcgcgtatt atcgaatcaa cgtcacgtcc
29941 aacgtaccct atttcagtaa acttcgttgc ctcaattttt atgaacggtg caccggcaag
30001 ttttgctaag cgacgagcta tttcagtttt accaacccct gtatgaccga tcatcaatat
30061 attcttaggt atgatttcat cacgcaatgg aaatggaacc tgattgcgac gccaacgatt
30121 tctgagcgca atagcaacag cacgctttgc atcatcctgt ccgattatga atctgtccaa
30181 ttctttaact atcttctgtg gtggcaggtc atctaacaaa acttgagtgc tgtcattaac
30241 ttgaacatcg ctgtcatttg aatcaaaacc actcttagta tctttataga ggttatcttt
30301 ttcatcgtag gtgtcactac tacaagaccg cccttctgaa agggttatgg gctgattgga
30361 aaaattggtg cataaagttt tttgcttgga agacatatca ctcctctatc ttttcaataa
30421 ctacattatg atttgtataa acacatatat cgccagctat cttcatagcc tttttcgcaa
30481 tttcctctat tgatattcct ttaatgtcaa ttaaagctct tgctgcagat agagcaaaat
30541 ttcccccaga gccaatagct gcgactccat cttcaggttc aagaacatca cctgttcccg
30601 taattactaa tgaaatagat ttatctgcaa cgatcatcat agcttctaat ttcctcagat
30661 atttatccat tctccagtct tttgcaagtt caacacatgc tctcattaat tgccctgggt
30721 gcttgtcaag tttagattct aatctttcaa aaagagtaaa tgcgtcggct gttgccccgg
30781 caaatccagc aattacggaa tcaccagaaa gacgcctaac tttttttgct ccagatttta
30841 taacagtgtg gcccagtgaa acttgtccgt cacctattac tactacactt ttatcttttc
30901 taattgataa tatagtagtg ccatacattt tattgttatc gtgttgaatc atttttctat
30961 cactttaaat aagaatctca ttatacttta atctttagtc aaatttacta aatatgttca
31021 actatagctt ataatataat accttcctta taacatttaa agtgcagatg caaaacataa
31081 ttcacccaga tttggaaaaa agtataaata attggttcga agcaaaattt aatggcctta
31141 catcgccatt ttatagctct atagatctta gaaactctgg ctataaaatc gttccagtag
31201 acgctaattt atttcctgcg gggttcaata acttaagtga agaatcaaga acgatggcag
31261 caagattgat aaaaagctat tttaaaagac atcaatacaa aaaggttctt ataataccgg
31321 aaaattatac acgcaataaa atgtatatag aaaacgtatt tgttatagag aaaatactac
31381 aactcgcagg ttttgagact agaattggcc ttttatataa tgaagtatac aatttaatag
31441 aacagtatga aactgttgtg aaagaaaact ctctgctaaa gacaacttca ggatttgtgc
31501 ctgatgttat tatactgaac cgtgatatga cgagccacat tccagacacg ctgaaagacg
31561 taaagcaaga tatagtacca agcccactat acggttggca tagcaggcag aaatttaagt
31621 attttgaaat ctatcaagaa ttagcatctg agttttgtgg cgaatttaaa atagacccat
31681 ggctaatttc tgtgcttaca gagagctgtg atggagttga ttttgatgat gatttgtcac
31741 taggagcagt agcaactaaa gttgaccaaa tattatctct agtgcaaaaa aaatatgagg
31801 aatatgaaat aaaaacacaa ccttacgttt ttatcaaagc aagcaatggc acatatggga
31861 tgggcattat aacagcaaca agtggagagg aaatattaaa cctcaataaa aaaaagcgtc
31921 ataaaatgaa aaagataaaa gaaggaatag caatcaatag cgttattata caagaaggtg
31981 taccaaccat tgacatgttt aaaagcagtt ctgcagaacc gctaatatac tatataggag
32041 atactccaac atgttactta taccgatgta atagcagaaa agatgtatat tctagcttga
32101 atgccactga ctgtgagttt catgatgtta gccaggttgt agaaggtaaa attctatcac
32161 tttggagtat tgttagcaaa ttagcagtac tagcgttggc agtagaaata aaatcttttc
32221 atccctaatt cttatacctc tacagtgctg tcattagaat gaccgtttat attatgaaca
32281 tcatcaagtt cagtggaagg atcatcattg acaggttgtt gaaacaattt gtcaagctga
32341 actgcaagaa atactgcact atatattatg ctgactgtta cgcttaagtt gaaattatag
32401 attataccat tacctaagtt tattggtata gttggagtct ctatccttct taatatgaag
32461 tttaataaac ctaacgacaa agttaatgtg ctaataataa ggtccttctt acactcagca
32521 gacttttcag gatccttatt atccagatat tccttgcatg tataataata gcttataggt
32581 tctgaaatga acaaaaatag gatcgttgag gttaagttaa tataacttat tatgtctgta
32641 ttgcttccag tgcaaaaatg gatgattttc aacatcatta gcgcttctat ggtgctgctt
32701 gcaagattag ccgatttacc cacaattttt gccagctctt ttttactctc agggtttctg
32761 taatccttaa ttagatcctg tagagtaaat gcagaatggg caatgtatag aatagatgtc
32821 ggaaacttta ctagtcgagc aacattccgt ataagaaaga tattggatct attattgtca
32881 atgaactcgc agcaaaattg cagtgaaatc ttacacaata gggctgttaa acctgttgtg

32941 atgcagatta ccttcctgaa atcatgatca taaatatcat gcaccctttg tgcgagtatg
33001 ttagatttct tttttagctt gtttggcatg aaatagttaa taacttcctg ttattctatc
33061 acataaaatc ctaaaagaaa agccctatga atcaggactt taagggagat ttatgcacaa
33121 atcggctaag cattttataa acaagggatc ggtactgaga gttggcacac gaaagtaata
33181 tccatccttg ataattgctt tatattctat atcaagttca actagagttt ctgaatgctc
33241 agaaacaaaa gatataggtg atagaactac aggtacacca tcagcttttg cgcgtaatag
33301 ctcactttca gtgcttggct ctagccattt tacaggacca atcttactct gataacatat
33361 tgaccaatct aagttattga tagctaactt ttctactatt aactctacgc ttctttctac
33421 ctgtgaagcg taagggtcgc cctttttaat gatactaaga ggtaagctat gggctgaaaa
33481 taggactctt ggtttaccga tttttctagc caatttatag tacttagcta tcagattagt
33541 gtgagcttca ataaaatctt ggttatcata ataacggtga atcatttttg tattgcattt
33601 taggccatac cgtttagcat ttttttgcca attctcgatg gatgacagag ttgtggtagt
33661 tgaatattgt ggatacagtg gcagcagaat tacttcgtca ggatcaaact gttttacact
33721 ttcaataact tcgtcagcaa atggacgcca atagcgcatg cagatgaata ccttgtgtac
33781 atggtttcta ttttcgttta attttagttc tgaagcatta gcttgcattt ttgtattctc
33841 tagaattggc gatttacctc caatttcctc gtatattttt cgtgcagtat tttctcgttt
33901 cgcagatata aactttgcta aaagaaaccg aaagggattc ggtagattga ttattctcct
33961 gtcataaaaa aggttgaata aaaaaggacg gaccgcattt agtgaatcag gcccgccaag
34021 attaaataag ataactgcct ttttcaccta cgcactacta caacaagtta cttctggggt
34081 gcctataaaa ttagctacac actcatcaaa tttagttata ggcactaaat ttcttacact
34141 ggcatacaat aagtggcctg atctcttcag gttactcttg gtagatgtga agttccaatc
34201 gaaaaatgta tcgacaaaaa gacttggtac cgtttcaaga agttcaacag cagctaaaga
34261 tagcattata caactttgca ggacattcag aagaaccttg aggggaaaaa gtaaaacttt
34321 aacgaaaaga gaggtgctac ttgtttcatc cagtagtcgc cctgagttag gcgaattata
34381 ctcggtgtgt tttctatttt gatcaaagat aggaaatttt atagtatctt tcttaaattc
34441 cgcgtctatt tcttttctac tatggttagt gattttttcc attaacttct cacgcagatg
34501 aaaagttctg acaataaaac atgtggcaat cattaaggaa gcacataaaa gtgcgcattg
34561 catgaggctc atactgccta gcatagctgg cagtgcaaaa caaacaaatg gcactatgat
34621 agccatttca agaaatggaa tagtgtttct tttcattgca aaagcagcac cttttttttaa
34681 ttctgtatta tgatgtaagc tatattccaa aaagcttaaa aaggccaaat taaaagttaa
34741 aaatcctttt gttccacaaa aaagtgatga cttaaagtta cttactaatt ttttagtagt
34801 agatttagct tgttttttag tggtaggctc agcttgttta gtagtgttat ttttacaaca
34861 ttcacccata caatacctcc ttgatggttt attcttaact ataaataaaa aactactttt
34921 atcaaatcaa aatttgcaat aaaatgaatc cataaatata caatcttata taagggaata
34981 atatggcaat tgagaagaca ctttcgatat taaaacctga tgcagtaaaa aataatatta
35041 caggcaatat aaattcctac atcgaacaat ctgggctgaa aatcacagca caaaaaatga
35101 tgttactaac aaaaaaacaa gcagagctgt tttatgaaat tcataaagat aggccttttt
35161 ttggggaatt agtagagttt atgacctctg gttctgtggt agttcaagtt ttagttggtg
35221 aaaatgcagt aagtaaatac agacaaatca tgggagctac cgatccaaag caggcagata
35281 aaggcacaat caggggtgat tttgctgatg atattagtga aaatagagtt cacggctcag
35341 atagtctaga aaatgctcgt aaggagatag ctttcttttt tgctgaatgt gagttggtgt
35401 agtcaactca gattggcgtc atccaagtag ctgacaacat agaaacgaaa aaacttgctt
35461 gacaccctcc gccagccccc ttatcatgaa actgaagcta ttgtatttgc tttcgccaat
35521 ctgcagatta aaaggtaagg attacttaat gtatcggcgt cttatgttca atttttttgca
35581 gtatatagat actgtatgtc tttacaaaac ttcatctaca tctaggtttt tatctaaata
35641 agctgaacgc gcttataaag cgttacaaga catcaaaaaa tgccaatact cgacagagat
35701 agtaaaagac tagctaactc ggggattctt tgtctttttt tctgcttagt aaatttctta
35761 aacatttgca gcgtaagtta gttgcaatta aaagcggctg aatctcgctt gtcaagcgtt
35821 taaaacataa aaaacgccaa tattttaaag cggataataa ccccagggtt tcttttgcct
35881 tttttttcat ttggtaaatt tcttaatatt tatagctaaa caacaaccgt catcccgcta
35941 cgtgttagcg ggatctatgc tttgagatac cgcgaatgaa tcgcggtatg acggtgtaga
36001 tgatggttaa gtaattcgtc atcccgctgc ttgttagcgg ccgagatacc gcgaatgaat
36061 cgcggtatga cggttcgcgg cagtatgacg tagagctcac gttagctata cttttatgct
36121 tacaaactta ataaaattcc tggatcccag tgtctgggca ctgggatgac aaaggagagc
36181 tactcggatg ataccttttt ctacttagtt tgggttatgc aagaagtcta ttataatcag
36241 gctttaagtg tgcggaagta attttttgcg ttagagtaaa aaattttttc tatagctaat
36301 caaagtggat caagagaaac ttaccacttt ctttacaaag ggtttgatgt taccatttga
36361 ctgcctattc tgaatttatt gaatgaacgt taacataaag aatctagttc atgctttctc
36421 taaattgcca agtttagggc catcatcatc acgcaggtta gttatacatt tactccaaaa
36481 caaagaaaaa gttatgctac cacttgcatc tttgattaaa gagctagcag atcttataat

36541 agagtgtgaa gtttgcggaa atctagatac taaatcacct tgttctattt gtactaaccc
36601 aaaacgtgac actaagttac tgtgtgtagt agaagaattg ggtgatttat gggcgtttga
36661 aaaaggaaat atatattcag gtttatacca tgttttgggg ggcagattgt ctgcgataaa
36721 tggcataggt ccaaaagaac tcaaccttga tactattctg aaaagagtga cagagtctaa
36781 aattgaagag ataattatcg caattaatcc gacattagag ggtcaagtta ctgcacaata
36841 tataattgaa ttgctaaaaa atttggatgt gaaaatatca cgtcttgctt gcggcatacc
36901 aatgggcgga gaaattgact atcttgatga aggaacgctg aggatagcac ttacttcaag
36961 acaagaatat gagttaaata taaaataaga catgtgctta aaaaaactta tatcgctaat
37021 aacattcaaa aagaaagaaa tagcgccacc acttaaagtg gtggcttggg aaaatgaccg
37081 tctaaactat aagcaatttt caggtaaatt taacaatata attaaaacaa ttgatcagtc
37141 ttttacaata atatctttgg aaggatatga tggatggggc aagacgtttt ttttgaaaga
37201 atgggtaaaa gagttaaaac agcaaaatga aattgcagct tattatagtg catgggatat
37261 caatgcattg gaccaaccac tgccttcttt tttaaatttc ctatttgaag atttatttgt
37321 atcgtataaa gtaaaaagaa gtgttataca acagttcaag aacataaacc aagagctatt
37381 ttctctaaac acactaggaa agctgatcag taaatctcca cttgctatgc tttcagtatt
37441 tcttgatgct gcaaaagagg ctgataaaaa agacataggt tttgtcttga gtgaattaag
37501 tcttctccaa agaagaaaag agagtactag agattttaaa acacagttag cggatgtggt
37561 taataaaatc agaaaagaca aaaacattta tataatggtg gataatcttg atgtgtgccg
37621 tcctaaattc gttgtcgatt ttttggaatc tataaagtat atgcttgatg tggaggggct
37681 ggttttcatt attcctgtaa gtaaagataa gagtaatgta caaagagcaa tcagcacaat
37741 acttggacca aattttgacc taaaatcttt tactgatctc tctttacatt tgccaaaaca
37801 accgatagaa aaatttacaa aagaactatt tgaaaatatt aaattaccga aaaagtcaaa
37861 aaatttgatt gtagatagtt ttatatttta tgcagaaagc ctgtcactat cgttaaaaac
37921 aatagaatac tgcgtaaaaa aaataaagct gtgtcttctg aatcacacaa gagaagaatt
37981 acctgatccg aatttgtttt catttttggt aatactgcaa tcgataaata ttgatatata
38041 tgaggaatta ggtttttcat atcaaagagc attggaaaaa attgaaaatg aatataaatc
38101 tttagttcta aatggtcaag aagaatggga aaaacttaaa acctctctag aaactgcttt
38161 tgcagagagg gagtcaaaaa ttaacaaagc tataaaggat atactatttt gaaaagatac
38221 cctaccttca catatacaaa gatagggtaa catttgatta acgccctttt actatgtcat
38281 taccttgttc aaactttctg attaattcgc ttacattgac attagataat tttttacctg
38341 cagatttgtc attatgtgaa gcatcaggtt tacgaaggtc ttccttactg ttactcctag
38401 taatattaca tctaacaatg tttgaattat tagacttatc gcttacatct gaaatattgc
38461 ttttttcact acatgaagac gaattactcg acatattgcc taagctaaag agctttttac
38521 cttgatagat tggattccaa tcaagtggat tataagtttt tttgctagtc tcaacctttt
38581 ggttattttg ctgtttagga gaaaaaccaa gtttttctct ttctttcttg cttagcgttt
38641 tgagaaattt tacggtttct aattcatcag ttgcttgtac tttatgtaaa tcaaagcttt
38701 tttgatggtt atcctgctta acattttcca tcattttatc ttttattgtt ctatcctcat
38761 catcagaagt gctgctacta atatcactga taccactatc ccctgaattg cggcgttgtt
38821 tgttaggagt gctatgaatg ttgtcttcca tgagtttttg cattgttggg ccaaatgttc
38881 ttccaagatt gctactcact gtttcattac tatcatttgc ttgagaatga ggtacatttc
38941 gctcaaaaat tgcttttatt ccagcaaatt tagcttggga agatttcgga ggaacaggtg
39001 gggcaaccat gccttttgag ttaatctttc cttcattttg aggaaaaatt tttggattat
39061 tttttttctac tgtagtattc attttttttac ctctaaacct attaactaat aaaagtataa
39121 acttagaggt attaattttg taataacaat gctaatagat taccgcagtt tcaataagca
39181 tcaccagagg attctctata taatgcttaa aggcatttaa aaactttgct ccgagtgccc
39241 catcaactgc tctatggtca acagagagcg ttactgtcat tatttctgct gtttctattt
39301 tctcattcat aacaattggt tgttttttag atgcaccaac tgccataatg caagattgcg
39361 gcggattgat tatagcactg aaggctttta taccaaacat ccctaagttg gagatagtaa
39421 accctcctcc ttgaaactct tcaggtttca attttccaga tcttgctctg cttactaaat
39481 ctttcacttc ttttgatata gataaaatac ccttttttatc agcatttttt actataggag
39541 taattagccc atcttcaagt gccacagcga ttgagatatc tacattcgag tatttaagta
39601 ttttattatc tatccacgaa gaatttatat caggaaattt tttcatgcta aaagccacag
39661 ctttgataat taagtcattg attgttactt tattgttttc atctgctgag ttaatctcgt
39721 tttttaacga tattaactta tcaacctggc agtctacagt taaataaaaa tgtggaacat
39781 tctgtttgga ttcagtcagg cgctgtgcta tcacttggcg tatgttgctt acttcaacta
39841 tctcaggact ttcagtatgt ataccactgc ctaaaaactc caacacatca gccttaatta
39901 tgcgaccgta tggtcctgta cctttttaatt gctgcacatt aacccccttca ttttgagcta
39961 ttttcttagc taatgggctt atttttattc taccttctgt tgtcttagta tcttcttctt
40021 ttctagatcc cagtgtcaag cactgggatg acattgagag gttggattcc agcgtcacgc
40081 gctggaatga caccgaagaa tgacttattg gctttccgtt taaatcttgg tgttctgatt

40141 tttgattatc aacagcacat tttgtcactt ccttttttaac tgcactattg atggaagttg
40201 aggtatagtt gttaagtgca ttttcatcct ccccttcttc cagcattaaa gctattggtt
40261 gatttacagg cacgccactt gttccttctg ttactaaaat ttttgctaca actccttcat
40321 ctacagattc aaactccatt atggctttat cagtctctat ctcagcaatt acgtcgccta
40381 cttcaacttt gtcttgttct tttttatgcc actttacaat ttttcctcca gttttgctca
40441 ttgtcggtga aagagcaggc attaatattt ctataggcat tgattttgca tttaaagatc
40501 tattcaattt ttaatttact ctttatctta tgtcaaccta atcgtgaatt atgatgaata
40561 agtctgttac actatccctc acgttatccc agtgcgtgac actgggatct tggttcagta
40621 ctattatcct attgtttttta ttatttagcc atagcttgtt tgctaacaac tttgtttcaa
40681 ccaaatcaaa taagatcaac atgagaacgg ggccagggtt tcactatcct gtaaaatgga
40741 tatacacttg caaaaatctg cccctgaaag tgatagaaga gtttgagagt tggaaaaagg
40801 tctgcgatat agatgaagac tgcggatgga taaaaggcaa tcttcttagt gataagcgct
40861 acgcgattgt aaaagaagat acttatggat atcaaaaaca gagtgtagac agcaagatta
40921 ctataaaaat agacaaattt gttgtaatga aaatagaaaa gtgtaacgaa gagtggtgtt
40981 ttctttctac cccaaaacgc aaagcatggg tacaaaaaaa acatatatat ggggttgatt
41041 aggttgacaa aagatctgaa ttaacactag tttctaggcc ttctgtagca tgctcttcat
41101 gtgaagcaga ggatggcact gacttcttca tgctctggag actgtacagg gctatcacag
41161 aaactcctta acatctcctc agcggatgaa tctaaaattt gtacaataga cttttcactt
41221 tgttctctta gcgattgatc cactctagaa atcaactcag aaaactcgct acctgatttt
41281 tcagcttcac tttttatttt actttctatg cttgcaatat taccttgttc aatttcttct
41341 tttgttatgt tgaacgaatt tgcagcatct tgatatccaa tcttgcaatt tccacaacca
41401 attatgcaaa aagcaatgga attagatatt tcgcttgcag cccacttatg tatagcgtaa
41461 gccaaataat tgtcatggta tttaatgcta aattgttgtt ctattagctc agaaatttta
41521 ttctttagag acataaagta ccctccttac attcgttata ccttttttgtt aagatacagc
41581 aattacacca tttactacat gctttacttt aattaattat taaaacgaga gcataatttt
41641 cagagttata tctttttacc ttaacaattc tttttgagt tcctcttcaa tcatcttagt
41701 taaactcgtg tcaaactcac tcctcgaaaa atccctaacc tccccgggat catatcgatt
41761 tcctatcatt ttttcaatag catcatttat tatgtttatg tcaaatccct ttataatagg
41821 cgttaccttc acctgccctg cagagccttt accataaatt cttttgcttt tatcaatgca
41881 accacctgca ccaggtttac tgcttccaaa acccttgcta cactttactg tttcatgctt
41941 gatttcacca ttttctatat atattatttt attatcttca gtaacccgca atcctgttgt
42001 tgtggttttt tcttttaatt ttacactatc atcaattatt ggttcggtat aatccttata
42061 ctcatatatt ttatagatgc ctccgcctcc catagtgatt aaatacttgc actcttgttt
42121 acctaaacga cactttctta taaaaatagg acccccatcc ttatctgtat attccttttc
42181 cctgttacct cctccctgtt caactttaat gttaattata ggataatcaa gatcaatttc
42241 caattcagct ttaatataat ctcctggcat tcctactcta ctttcagtgg aattaggtgt
42301 gtaagtgtaa ggtgtatctt ttatgtgacc tgcttcacca catccccaca cttctatttc
42361 aacacggcta gatgttattt gtgagccatc cacatcataa tctccacttc caaaaacatg
42421 ggacttttcg ctcttttttt ccggttcttg tttctctttt tccagttgtt tctcgattat
42481 ctcttttaac ttctctttga gaccttctaa atcgaaccta cacaatttat cagcgtaaaa
42541 gacctccgtt ctgtttgctt taattggtgc ttcttgttgt gtgacaacgc catttgtatt
42601 ttgtgaaatc tttgtataaa aaggttgagt gttttctttg tctacatatc gtgttgaacg
42661 gctgcatgtt ccagtaaagc agatacaacc atcatcacct tgattacatt tttctctatg
42721 ctctacatcc gtcaatttgt atactacttt cagtccttta ctagatttaa atgtatcgtc
42781 tatctcatca tttcttttta cacagtaact gcattctctc ttcttctgct cattttcatc
42841 agggaagaaa acataccctt gtttattgaa tattatttta tctaactcac tctgatctct
42901 gtttaagaga tcggctcttt tattttcctt gcttggaaga taatagaact gatttgattc
42961 cttggagtaa acagcattgt atttagtata tcttgttccc attaatttta gtggtattat
43021 tttattttct cttttaagaa caaactcctc tggttctggc tgccatccag aaagacaaag
43081 cattttactg ttagaatttc ctttatactt tacttctatt tccggctgcc cagagttatt
43141 aaattcacat tgaatagatt tgtcttttaa ctttttctca aaatcattaa aactataaaa
43201 acttccgcct ttagaacatt taatttgcac atcaggaaca tagccatatt ctttcagaac
43261 tttaaattgt tgcggattac tttctaatat accttctata atattatcaa tattactatc
43321 agttgttttt gctactattg ctggttttac tacttttaaa gatagggggc caatgtttat
43381 aggatggttg aggtcagtat taacattaaa agtacaggaa ccattactat aggttccatg
43441 tgctttagca caagtgctct cactcatctt catttctact ttcaatgtat ttttatttac
43501 tatctccact atctcaggtt ctggagcagg tggtgcagga aaacagcgga cgaactgtgg
43561 attttgttca tttatacttt gagttccaag gtattcagca cagagctggc cctttttttct
43621 ataggttttg aagtaatagt gagtatcctt atctgtaaca gtatatgagt gatttggaat
43681 gttctcatta tttgaaacaa cgtgtggaaa acctaactct tttctttcct ccagtccgct

43741 aattatcacc ccaactcttg gatcgaagta atcattatct tcatttgtta taggaacaat
43801 tctaacttgt ggaggtgaca tcgcaagttg ttcacagaat ggaggggggc taggtgcaag
43861 tggcacagga gcacatttta ctgccgtttg gtagcaggta tcacagactt tttccatctc
43921 cccaggacgg aaaatattct cacccgaaaa tactctagaa ccccatgaag aaattcctga
43981 catacaagcc cctttttgac tgcaagcaca tattttagga gagttagcga atcttctttt
44041 ttcctcatca gtaacaccgt ctgcccattc ccatttaaga ccccgagcaa tttctcctcc
44101 agcttttgtt ttatctcctt cccagtcgat aaaagctgca gcagaaactc cagctcctgt
44161 accttgcgtt ccatatatct ggcggcagga ttccccactt tgtaagtcat aacaataact
44221 ttgtccatca taagcacata ctctaatttt gggattaaag taaccatcaa ctccttgatc
44281 ttttattgct tgcaccttaa tttcctttac ccaatctcca ccaactattg cttccaacca
44341 agtattactt tctacaaatg ttggacaatc agcatatcct tgatacgaaa gaccaagtat
44401 caaaaaaaat atcaataaga aattaagcct tacacttctc ataaaatacc ggtaaccact
44461 cattaacttc ttctcctttt tcctttattg cctcgtacat atactttatc gtctctttgt
44521 tagctgaaag tacatgtatt tcttgcatat tctttaaatc taaatttaaa gttactaatc
44581 ctttatactg ttttatcaaa aataaacctt cctgcgttgg cgtctgcaga attatattca
44641 gctcctcttt tgataaagaa aatgctttca tgtataatct atttgcattg acgttcggca
44701 tcaagattct tgtatcaaca tgtttatcta aatattgagt aaatttgcta gcaaatgcga
44761 gatttaaatt ttcagtactt aggattacta caacatttag ttccgtcatt ctttgcatcc
44821 agttatcaaa ctctttctct gtaggaaaaa tattacttat ctcccatgct tcatccaaaa
44881 caagtattgc aggcgagcca tcacattttg cctcaaaaga atataaaaaa tagtaaagta
44941 tcacggacat acattccttt tgttttgtaa ggtttgcggt gttgagagat ataatttttg
45001 tttcccagtc aatatctgat tcattaccat cttgaaataa gtaagcaaag tcaccatcgt
45061 cacaccatct acttatctta cccccaagga gcaagagcac ctcagaaatt tgtgaaatag
45121 aacgtgattc cttgggtata gcaaatatag aatccacaat ttttcttatc ttttcttcta
45181 cgtccaccaa gctcgtatcg gctaccattc ttctgatcag ttcaaccaac atattgcgat
45241 tggaagcact atcttcaatg tttagtggat taaattttag gcttttgtcc ttatattttg
45301 gatcgattat gtaatattta ccactcactg ctttagtaaa aattattgat tttccagtgt
45361 tatccaatat gacaattctt gggttaaact ttcttgattc tgataataga aaattaatga
45421 gtgaagttct gcctgaattt ggagctccga gtatcgtagt atggccatta tttctttttc
45481 catgaaaatt aaagaagtaa ggatttcctt tttttgagaa aaaaatcatt accgcttctt
45541 tccaccttct tccttttaat gtacctgacg taaaatcgtg cagcatagca aagctgcaag
45601 cgtattttgt taagatattt ttcggttgcg taataaaagc aaaattgcct ggaagttgtg
45661 cccaaaaatg actttccata tgtaaatcag ttctaaacat catgaagcca actaatgaca
45721 ttatagaaga taaatcacta atactttcag ctaacttatt cctatcatct gcaaatattg
45781 taaaaataat tttctgttgg cagaagtcta tactagaggt cttttcaagc tctattattt
45841 cttcaattcc tgagctttta agtaaggtat tatcctcact aatttgcagc atgttgattt
45901 gtttttttaaa ttcttttatt gctttgttat tactagtaaa tatcattatt tctgtaatga
45961 taaattcaga ttcaagctgc aagcatctat ctatattacc taaagagatt tcccggtatt
46021 cttttatgct gaaaatagag ccaaatttct gttgattttc aaatattgtt tgaaacgtat
46081 tgaaaccaaa agctatttta aaatttctaa tatattgcga caggtccctt tcatgtattg
46141 gataatcttt gtgcgttaat gtaacaatat agtggagaaa ttctatcatt tcagaatata
46201 ttttaccttc actaaacctt agactcagtt ttttagctcc aaaaggtcct aaatcatttt
46261 gaattacgtc agttatcttt tgcaactcct gatggctttc ttgtaaagac aacttatgct
46321 tattctttat tctattgaaa aaaaacgaat tattagtatg cttgccaaaa tcgctaaaca
46381 atattacaat gtatagttca tttatatact gtaatttact atcagttagt ttattaaacc
46441 acgttgaatg taggttgtca gagaagtctt cagttttatt ccatttcaag ctcagtttat
46501 tgcgctttct aacggtatga atccaaacgg taaaacacag gctattaata ttttttgata
46561 tgctttttct aatttcagtt ctgaggtcgc tgtagttatc aacagaagaa taatcttcta
46621 acttgattat ttttaacaat tctccctgtt ttgttagtat ggtttcttca tcgtaatgac
46681 aagcataagg aacaaaatct aaatcaatgc tttctagtaa tgctaagttg ttcttttttt
46741 taagcttaga gttaactgtt ggtactttta actgcatatg cgtattcaat aaagataatt
46801 aaaaaagata aaaatactat cttaattttt atcttttttt tcgctatcgg attgtaattt
46861 tatatctttt gcaaagttaa atttttcttt taagtagtct gttgcctttt ctgtactacc
46921 gaacatcagg accagtacac caaacattat cataaaaaag attagacctc tgaacactac
46981 tgcaagtatc actgcagcta tacacaaccc tatgattgtt gaaggactca tattttctac
47041 agtatcttta actgaaaatg aagaaataaa attttttacc gtttcttgta taccctttaa
47101 ttcatcactc gccgaacttg cttcgctaga atatgatgca aaaatgaaac ttaataaggc
47161 tataaaaacc aaaactttat tcaattgaat gcgcatcaac taaacacctt ttaaacattc
47221 ctcaaatata acaactttta tatcaatagg caagaactga attcacttat ccactacaag
47281 taacacatat ttaattttta ccgctgtgaa catgctgtaa gctcttatca tcaacaagag

47341 gactcacatt aatggatgtt aatttacttg ataggaagtt tgaatgcaat tcagctactg
47401 ctgctatagc aagcactgca accatagcaa gtagtcctgc tcctatcaat cctgatgtca
47461 ttgctcctgc aacaatgcac cctattgtgg agagagcaca acctgctaca agtgcaacgt
47521 gaagatttct gttattgggt tggtttttaa gtaggatatc tttcttaact tgatctggat
47581 ttgcttgacc atctgagttt tcattttctt gcatttttgg ctcttttttt acagtctgct
47641 ctggctgcac ttgagtctcg ttttcttttg gggattccaa atttgattct aataactcag
47701 tttttttctcc accctctggt tgtaagccat tatctccttc aacagagctc aatgttgagt
47761 ttaaactttc ctcagatgaa gtgcttgtta ctaaagttaa actgctcctt ctgctactta
47821 ctggtgattt caaactttct ttactcgttc taggtgagtc tattccacta tctgagtcat
47881 tgccactact gagtttagaa gttttcttct cctctatttc tttcacaacc ttgctatatt
47941 tttcaaacac ttcctctgca acatctgaat tactaagtat tttattatca ccactttttt
48001 tctcagctac attcaagggt gtttcttgtg tgttgttttg ctcttcaata tgttttagct
48061 gcatttgatc ttcaatattt tcttcaacag catttggtat tgagcttaca ctgctgctcc
48121 tgttagtaat atccaaaatt gtttttacta tttttcctct tattgtttgt ggtctttgtg
48181 attgttctct ttcattgtta aatactgtag gtacagctag ttcttctgat tttattactt
48241 catctttcaa tgcaggaaga aagttttcga tataatactt ataatgtttt tgatattcct
48301 cttgttttttc tggtgatttt tctaattgtt ctctcagcga tttttctaat tcagttaata
48361 cctcaggact agctccttcc ttgaacaact ctatgcattt tttgatagtt ccatctccct
48421 tttgtgggct tccatattta tgattttctt ttgttacctt tttgtatata gtgtcgtgca
48481 attgttggtt aatatctttt ttagaactta gcatagactc ctccatacat aacaaaatgt
48541 atagatacct ctttagcgtt aattttacat taaaactggg caattatgcc atattataaa
48601 taattgcggc aacttgctta ttggaaggtc gcttaactat ttgctttctg ggctttctac
48661 ttctacagat ttgctattaa tatgattctt tgcttgttga actaacatat tcactacttc
48721 ttttacaggc ttttccctat caaccatgcc aacactttgg ccagccatta gagatcctgt
48781 ttcaacatct ccatcaatta ctgctcttct taaagctcca gcccagaatt tttctatttc
48841 aagctgccca tcttcttttg agatctggcc cttttggtat tcatcaataa cttctttttg
48901 acgcttcatg aagtcatcac ttgctttgtt ggctatagct cttacaggaa ttacaggaaa
48961 atcagcactt atctgtacag aaggtattgc attacgtgcg gccgatttaa taaacacttc
49021 cttaaaattt ttgtgggcaa ttgactcatt agtgcagaca aataatgtgc ctatttgaca
49081 gccacttgct cccatttcta agtaattaac tatcacttcg cctctgccta ttccacctgc
49141 aacaaacact ggtatttgtt catttttaaa atgaggcaat atctcttgtg caagaacaga
49201 agtgctcact ggacctatat gcccacctgc ttccattcct tctattatta atgcatctac
49261 tcccattttt actaaccttt tcgcaaggct taatgctggt gcaaagcaca taactttaat
49321 gcctgcaccc ttgatttttt ctatattagg ctttgttggc agtccaccgg caagcactat
49381 atggctcact ttcgtttcaa tacacacatc tatcaactca ctcaactttg ggtgcatagt
49441 aattaggttt acaccaaatg gcttgttagt taatttttgt gtctctatga tttctttttc
49501 cagtaagtct gggaacattg caccacatgc aatcacacca aaaccaccag cgtttgagat
49561 tgctgagacc aagtttctct ctgaaaccca gctcatagca ccacccatta tagcaaactc
49621 actgccaaga aaatctgttc ctttttttcca gaggctactt aacatataac ctatatcata
49681 cgtttaaaaa tgttttgctt atctataaat gtgtgcttca aagcgccaag tatatgtaag
49741 attataaaga gtaccataaa atatgcaagc atcgaatgta gctcgttagc cgtgttagct
49801 agatctttgt tttgattaat caacaaaggg atatggaaaa aatatttgat ctgtttgcca
49861 gaagcagaag acatgacata gccagatagt ggcattagca ccattaaaga atacaaacca
49921 aagtgcactg ctttacttat attgattaca aaccgagaaa aatttgctgg aagtggtgga
49981 acataagtaa agacgcgaaa aaatatgcgt actatgatta atccaagaat agtgataccg
50041 caagccttat gaatagcgta tatgttgaac ttaatttcat tgctaagtgg taagcttttc
50101 atataaagtc cagagcaaag cataccgata ataaaaacag ccatcaacca atggataact
50161 cttaaaccta ggctatattt attgtcttcc attttgatta ctctaaagta gcatttatat
50221 aatagaaaaa ctatttttca atcaaaatta ttaaagcaat ttagatttac caacaatgta
50281 gctatacaaa ttcgacccag ccggaagtca agggcaccta aatcaagaaa cggattaaac
50341 gaaaaactta cttgacaccc ttcgccagcg cccttatcat gaaactgaag ctattgtatt
50401 tgctttcgcc aatctgcaga ttaaaaggta aggattactt aatgtatcgg cgtcttatgt
50461 tcaatttttt gcagtatata gatactgtat gtctttacaa aacttcatct acatctagat
50521 ttttatctaa ataagctgaa cgcgcttata aagcgttaca agacatcaaa aaatgccaat
50581 actcgacaga gatagtaaaa gactagctaa ctcggggatt ctttgtcttt ttttctgctt
50641 agtaaatttc ttaaacattt gcagcgtagg ttagttgcaa ttaaaagcgg ctgaatctcg
50701 cttgtcaagc gtttaaaaca taaaaaacgc caatatttta aagcggataa taacctcagg
50761 gcttcttttg ccttttttttt catttagtaa atttcttaaa tatttatagc taaacgacaa
50821 ccgtcatccc gctacgtgtt agcgggatct atgttaaggt atagtagggg atctatgtta
50881 agagataccg cgaatgaatc gcggtatgac ggtgtaatga tggttaagta attcgtcatc

50941 ccgctgcttg ttagcgggat ctcttgttag cgccgcggca gaatgacggt tcgtagcggt
51001 atgacggtta agcaatttgt catcccacta cgtgttagcg ggatctcttg ttagcgctgc
51061 ggcggtatga cggttcgtgg cggtatgacg gttaagcaat ttgtcatccc actacgtgtt
51121 agcagctaag agataccgcg aatgaatcgc ggtatgacgg tctgcggcgg tatgacggtt
51181 cgtggctgta tgacggttcg cgcggtggca tgacctcgtc atcccgctac gtgttagcgg
51241 gatctatgct ttgagatacc gcgaatgaat cgcggtatga cggtgtagat gatggttaag
51301 taattcgtca tcccgctacg tgttagcggg atctcttgtt agcgccgcag cggtatgacg
51361 gttcgtggcg gcatgacgat aaggttccag tgtcaagcgt ttggcatgca aatttcctta
51421 aacgttcgta agcttaaaac tttactttag atcaaactgc tgtatagtga ctttatagct
51481 ttagttttac aatgagtata cctagtatag aaaattgtga tcttcacaat aaagccgtct
51541 tactcagagt tgacttcaat gttcctataa aagatggaga aattcgtgac gtcactcgta
51601 ttttgagagc attgcctact attcagtatt tagtgaatgc gagtgcaaag attattatta
51661 tatcacattt tggacgccca aaggccagag acaataatct gtcgctaaaa aatgtaattg
51721 acactttatc gcagttacta aataaaaaag tgaaattcat tgacgattgc tttggtgaaa
51781 aagtacaaag agcagtaagt gttatggatg caggagatat aatattacta gagaatctaa
51841 gattttataa agaggaagag caaagtgact caaattttgc caaacaacta gcatctctag
51901 cggatatata tgtaaatgat gcattttctt gctctcacag agctcacgct tctatttcac
51961 gcattacaga atttttacct tcttatgcgg gattttgctt gcaagatgag ctaaagtatc
52021 ttgaaaaagc tgtatcattt aaagctaaac ctattactgc gatagttggg ggagctaaaa
52081 tatcaactaa aataaaagtg cttatgaagc taacagaaaa ggttgattac ctagttctag
52141 gtggtgcaat agccaataat tttttgtcat ttagcaaagt aaatataggg aaatctttct
52201 ttcaaaatgg tgttgacgac cttctacata atattgttga aacagcaaat aaaaacaatt
52261 gcaaaatagt tgtgccagaa gacgttctgg ttgcagtaaa ctctgattac agcactagta
52321 tttcaagaag aactgagtcc attttggacg gtgatataat tttggatatc ggaccacaaa
52381 ctttgagtac aataagcagt ataatagcaa gcagtaagac tctgctgtgg aatggaccta
52441 ttggtgtttt tgaacattca gcttttgcaa gtggtacaat aggggtaatg aaaatcgtaa
52501 gtgacttaac gcacaaagga aagttaacca gcataatagg gggaggcgat agtctatctg
52561 caataagcgc tgcaggcctt accgataagg attttacata tgtttccact ggtggaggag
52621 catttttaga ttggttaagt ggcgatgaaa tgccgggagt tgctgctctg caaaaacgat
52681 tagattaaaa ctttttaaa ttatttctta taggctagct tttttgtgaa cgtgattatg
52741 tcgaagacag ttaaactaat tttgtgttta ataataccaa taataggatt tattttttat
52801 attaaggatc attattttca caccggctat gtagaagaaa atgttgaagt caattttgaa
52861 ccatttttca gttcacaacc agccaccgtt tcagaattaa ctgacttcga tagaggagtg
52921 ttgaaagttt gtggagattg gggagcacat ccagataaag aagatttcaa aattttactt
52981 agttgtccgc agcatcaaga agtagtgaaa ggaatatata agtgattacg ccagatgcgg
53041 atttggagtt acttaaagat gaactggcta acatttggtt tacaaatagt gaaagtgaaa
53101 aagaaactat agggtttgga catattttt gtggtgagcc aaacgataaa ctaggtggta
53161 tgcattttgt aggtagatat gttgaagctc aagaagataa atgggcaggt gcaatttgga
53221 atgataagtc cctatgcaat aaatcagata ttaagccgcc agtttataca tttggaatga
53281 aatatttagg taaggatggg gaagtgaaag ttaaatgccc aaatggatac gcatataacc
53341 ttcatgccga taatttctgc aactaaggtg tttaaggaat taggaaaaga tggaatgtgt
53401 ctatatgaaa tggaagatga cgattatcaa tcagtgtttg taagaaagaa tgatgctata
53461 ctcacgttct atcctgactt aacaccaaaa tgtgatgata agagtactta ttgtagttgc
53521 ggtaaatctt aatttcaaaa ccaatagggt aaacaaatga taaaaatgct atagggtgta
53581 gcaaatccaa atgagttaaa attttgatta aagggttttg tgaataataa tgaagaaggg
53641 cattattcgt ctaataaaag caattcaata ctcttgtgaa ggaataaaat cagcttttat
53701 atcagaaatt gcgttcagac aggagttact gctttttata gtatgtgtct caattttgtt
53761 tgttttggat gtaagtaatc tagaacgtgc agtaatgata agcagcctat tcctggtgtt
53821 aattgtggaa attgttaaca ctgcttttga aacgacaatt gagcgtattt ccagtgaaca
53881 acacatactt tcaaaaaagg tgaaagatct aggtagtgca gcagtttttc tttcattaat
53941 taacttcctg ataacatgga tgataatatt gataggttaa tacgaatgtt attaagatat
54001 gttgtcaaac tcaccaatta aagtttttat atcttgcggt aaatcagaga caaattccat
54061 gtatccctta cttttggggt gatataagcc cagtgtataa gcatgtagtg cttgtctatt
54121 gaaattacga ataaaactag agtttttagc atattttgtg cttttactac tattttttcc
54181 ataaacttga tcaccaacta tggaatgtcc tatgtggctc atgtggactc gaatttggtg
54241 tgttctgcct gtctctaaag tgcatttaac gaggcttact tgtcctataa ctttttgcac
54301 taaatagtga gtgattgcta atttgcctgt gatttttgtt acacacatca tttctttatt
54361 accgcgtttg ggagcaatgt tagtttttat cgtgccttgc tgagaagata atgttcccca
54421 aactactgct aagtattccc gctttatttt gcgattcgat aacaattcag acaaaaaact
54481 atgggctgct tcattttttg caattaccat tagcccacta gtatctttat cgagtctatg

54541 aacaattcct ggtcttacat taaggtgagc gataactgcg ttcaaaagcg tatcattgtt
54601 tgtcccagca cctgggtgca ctgttaatcc actttgttta ctcagaacta taatatcctc
54661 atcttcatag acgatatcaa gttttatgtc ataattaggc tcaattgatg tggatatgtc
54721 aggttgaacg agatgtacta tatactcttc acctggtttt actatgtgat cattattaat
54781 tatcggtatg ccaagtaatt tcacctgctc attttgtatc aatctttgag ctttactgcg
54841 tgatatgttg catttttcag ctatataggt gtctaacctt aatttctctt tatcattatt
54901 aacacttaag gttattattg agtgatctat agtgatttac cccgcgccat ttcccatgct
54961 gcttgaacat ttgggtctgt aaacattgtt gcactagact caatttgcag atcaccttgt
55021 tttttaatat ggcaaattgt ttgagagcca tctttccagt agattgtaac tttattatca
55081 aaatctcgta atgttgcctc aacctttttt tctttattat ctttttcttc cagcttaata
55141 gattctaata aataatatag aaagtcactt ggtttgccgt tgattttttc tttccattca
55201 tctgtttttt cttgttcaga ttttttttgc tctaactttt ccttcttttg ctgcagctta
55261 ttttgttctg cttccagctc taactttcta actgtgtcac gtagttcttt gactttattt
55321 tctaatacta ataatttatt agtattattt ccattgttag cttctaaatc aggcgtactt
55381 ggttgagctt gcttcttttt tatttcttct aattcattcc ttactacgtt taattgcttc
55441 ttcaattccg ttttttcctt ttctgtatcc tcttttaact tttttagcat gctaaggaac
55501 tcgttattat tagactgcgt agttgattga agccagccta caaagcggct tggttcaaat
55561 cgttcaattt tatcttcagc aattttaata gcattctcta acctagaaaa agtaccttca
55621 atatctagtg aatgtgcttt ttttgtcaat tctttagtgt tatttaaaac ttctgaaatt
55681 tctgcatgta gccaatctaa atcttttttg atgtcttctc tgattttttt taggtgatca
55741 agcgtttctt taatgttaat atttgcaggc tttaaaagac tattatcaag taaatcgcaa
55801 gcttttttaa ggttactgtc aaatgagttg caaagcggac taagtagttt tttcattaga
55861 gatattagtt cattttccct tttaccaagc ttattttcta ctactgatac aatcataccc
55921 tttccagacc agtatggaaa tagaagacct aaagaactgt gttttctaat agtatgattt
55981 tcatgatcca cgctattact tattagattg ctaagtgttg atatgatgca taaaagaata
56041 gcgactaata aagctattaa tattattgga gcattaatgg attgtgatat tgctgaaact
56101 actataatag taccaaatac atatgatcca gtaaacaaga gaatcctatt tctattttct
56161 gcttctatat cttctagtat ttctctctgc gtcttagaaa ttggtaaaac tattgaaagc
56221 ttttcccccct ttttatctaa atttgttaca atttcaattt tttcggcttc ttgaacaatc
56281 tggctgtact gttcatcaac ttgatagttt cctacttttt tatctatctc attctctttt
56341 attttttgtg tatttataaa gtttataaca gcagtaaata agagaactat aagaggaata
56401 aaggcaagag gtgctggcgt gaacgcaaac ccagcaatac ttactgtaac aggctgaata
56461 gaaaaggcag taaatgctat aagagtagca atggtacttg ctatcgatat agtaccagaa
56521 tactttctta tagagctcaa aattctactg ggctggtcag aaagttttct aaatcctaaa
56581 aaatcaaaat agtctttgaa aggatctttg agtgtctgta accataacat ttttctagtt
56641 taaacttatt taggtataca ttaaccaaac cttaaaatca agtttcttaa tttttcaaca
56701 tgtgccaagg cctattggag cgcactgtat taatctttac gtaacgattt gttaaccaaa
56761 cagaatgtgt gccatacgtt tctagatcag catgtttaat aattttagcg ttgcaagctg
56821 aatagtcgaa actcacttaa ttgaattgtt aaatcaactt tatcacaatt ttctgaaata
56881 tcttcttttt tataagcaag tagcactgat ttattatttc ctttattata tatacaatca
56941 taatcttcac actgtagcct tttatttgaa ttgttatatt ttgtatgatt caaaatttgg
57001 ttctgtccgt tttgctttgc ccacgttttg ataacaaaat ttctgctttt tctagtgaga
57061 gaatatagta agttatcact ctcctttgca gcaacattat cagcatttat taggatgtcg
57121 ggggttttat atgcagcgct aaagcaaata cctagtacaa taaaaaaaaa tcccaaaaaa
57181 cgccaatttc tttcccacaa gcacagccac aataacccaa atgttattat aataattgat
57241 gaggcaggaa aagtacgaat gggaataacc aaatactgaa gactagcgat agcgtttgtt
57301 atatacaaaa tgctgtcaat tgggcgctct acaagtggcg ctataatcca ttcaataccg
57361 aaaggaatca acaaaacata gattattcca agtggaataa taattaatgt aactatcggt
57421 atagcaacta aatttgtgat aatgccacta attgaaaaat aattaaaatt atatatcgta
57481 tacggaacag ttgctaaact tgctattact gagctgatca ttatcgacac aaaatatttc
57541 attattttta ttttgaataa tttgttggca ttgatttgat aactggcaac cagtgccaaa
57601 acagcagaaa atgacatctg aaagccaggc tttaaaattg cttctggttc tactgagagt
57661 atcactgaag cagcaaacgc aattgctatt aaccctcgat attttctctc tatcattatt
57721 gcaacgagta ctaaaatcac catgatgtag gcacgctgag cagaaatttg cataccggta
57781 atcaacaaat aaaacgtggt tggcaagata gtaaggaatg cagatatttt cttggtgtta
57841 tacttaagag tcaaagtttc agatattgcg aataaattac gaaacactac aaaaaacaga
57901 ccagcaacga atgataaatg caaaccagat atggcaaaca aatgtgctat acctgaatct
57961 cgtatcgcgt ccatagtttt ttgatctatg ccatcttttt taccgatcag taatgcagag
58021 attatatccg catgtggttt cttgatattt tgctgcaggt tttcataaat gtattgacgg
58081 aaagattcta tatattcttg aaatttcctt gcttcagcct ttttgtgcag ggctattttg

58141 cttgttgcaa agcctgttgc acttattttc tggtaatatg ctattcttgc aaaatcgtat
58201 gcatactccg agggcgcaat ctttagaggg aagagttttg ctgataattt tacttgatcg
58261 cctattttaa tgcctttttc cactttggtt ctaactgata ttctgatgtt atctagagct
58321 ctatccggtg tcattccagc gcgtgacgct ggaatccata tctttttttt ctggatccca
58381 gtatcaagta ctgggatgac aaaagggggc gctgggatga caggagagga ttttgagata
58441 gtaggggatt tttcaacaga aagcagaaat tgtttatatg agcccctgtc gttaatatcc
58501 ttcactgtgg caacgatatc ttttacatat ctctctttat caagaatttg agtatcaact
58561 aaagctgttc ttaatttact agctgtaaat cctatgagca ctgcaattaa agcgatacac
58621 aatatcgcgt accttttata caatattgca atcaaaatta gtgtgggtaa aagcaaaaga
58681 aaaatagata tgcagcttgg ttcaaaactt agggaaaaat aggttagaat tcctacacac
58741 tgaaaaacag gaaaccacag tatcaagtta tattgttcat tgcgcagatt gttacgtata
58801 taactaacta ttatgtcaag aaaagttaat aagtgtgtcg atatttgcat gtggtgaaat
58861 tttattttta ttttttcatt atttgtatat ataataacta aataagcaac gacgcgggat
58921 ggagcagctc ggtagctcgt caggctcata acctgaaggt cgtaggttca aatcctactc
58981 ccgcaaccat ttttaaggat atagttgttc cactttccaa tcgcttccct caacaagagt
59041 gtatctaaat ctaatatgct tcctatattc accttcttgc caaaattcaa ttacttttgg
59101 gattacgcaa aatcccaccc aaaaatcagg acgagggacc ttttgatcat aaaattcttt
59161 attcttcaat tcaatagctt gctcaaaatc ttgccaatct ttcagaatgc tcgattgttt
59221 tgagcaccat gcgctaattt gactatcacg tgctcgagaa gagaagtatt tgtcagttct
59281 ttcatcgttt agaagtttaa cctctccttc aattcttacc tgcctagaaa attctatcca
59341 gtgaaacact aacgcagctt tggggttctc agtcaattct ttcccttttt tactgtttac
59401 gttagtgaaa aacacaaaac cttctttgct atattccttt agtaatacca ctcttgcaga
59461 tggaatacag tctttgctac aagttgctag cgtcattgca gttggttgtt cacacggaga
59521 attaagtact gctttgtacc actttgaaaa caaatcaaat ggatcttttt caagtgaaaa
59581 tatcatatca aaaaataaaa tcttaattct ggcacgcgtt gcaaaaataa gtgctacggc
59641 cattttgtcg tataagtgtt atgatattat tgcaaatctt gcaaggtttt tgaactttac
59701 catatacgta aaagttattt tgaaagtatc cagcagatcc agatggctgt gcataatctt
59761 tcagtgttga accgcctgca gcaattgcat cactcagagt atttttttatt tcagcagcaa
59821 gtttttcgca ctctctatag gtcaagtttt ttgctgacct gagtggtgat atacaggctc
59881 taaacaagct ctcagaagca tatatgttgc ctacgccaac tattgattta ttgtccatta
59941 acgctgattt gatattcact tttttgtttt tcagcaactc ctgtaaatag tctccactga
60001 attcatccgt gaggggctct attccaaaat cgctaaaaaa atccgtttct tgttctttat
60061 ttaaaacgat aactaatcca aatcttcttg ggtcattaaa aattattgaa gtattgtcag
60121 aaaataaaaa taccacgtga tcatgcttat tccgcatttg atcgtggtca gcatatataa
60181 gctttccgct catgccaaga tgtatgatta cagccacact accatctgta ttccagatgg
60241 tatacttacc tctacgcttg atgttgcgta tgactttgcc ctttagcata tcatcaatat
60301 tttttgttat tggggcacgt aaattccaat tattcactat aacattactg atttgcttgt
60361 ttttaatttt atcaagcaaa aaattagaga ttacttccac ttccggtagt tctggcatga
60421 tacctccttc aaaacttaag tttctgatgg taattttgtc gttctcaaat acattcgagt
60481 attctgcggt gctttctcct aaaaattcct caccacaaac gagttttgaa agggttctct
60541 gtctactctg ttgctgcttt tttcttttca aaatattttg ctttaaagct ttcgctaatc
60601 ttttttttctc ctcttctttt tccttattct tttttgtgtt attcattatc ataatatata
60661 aacttgccgt tgtagctcag gtggtagagt acgtcattgg taatgacgag gtcccaagtt
60721 cgagtcttgg taacggcaca ctgattatac ttataatatt gcataacttg taagcaaggc
60781 ttagaagaaa gttacagagg taagttgtca tgctttttcc atatcctttc taccttcttg
60841 tttttgagta gctctaatgc tttacaaaag tgataccttg ttttacttgg cactataatg
60901 tcatcaatgt atccatatga tgcagcaaaa aatgggttag caaatttttc tttatattct
60961 ttaattaacg tttgctgatc tttttcgtgt cgaaatataa tctcaactgc actttcaggg
61021 cccatcacag ctatttcagc agttggccag gcataattta tatcaccttt tagatgtttc
61081 gaattcataa caatgtatgc accaccatac gcttttctag tgataaggct aattttcggc
61141 acggtcgctt cagcgtaa
//
LOCUS   JAQZAU010000055   6859 bp   dna   linear   ENV 17-MAR-2023
DEFINITION MAG: Wolbachia pipientis isolate wEsol contig_34592, whole genome
            shotgun sequence.
ACCESSION   JAQZAU010000055 JAQZAU010000000
VERSION   JAQZAU010000055.1
DBLINK   BioProject: PRJNA929258
            BioSample: SAMN33142973
KEYWORDS   WGS; ENV; Metagenome Assembled Genome; MAG.

```
SOURCE      Wolbachia pipientis (insect metagenome)
  ORGANISM  Wolbachia pipientis
            Bacteria; Pseudomonadota; Alphaproteobacteria; Rickettsiales;
            Anaplasmataceae; Wolbachieae; Wolbachia.
REFERENCE   1  (bases 1 to 6859)
  AUTHORS   Fiutek,N., Couger,B.M., Roy,S., de la Torre,J.R. and Connor,E.F.
  TITLE     Genomic assessment of the contribution of the Wolbachia
            endosymbiont of Eurosta solidaginis to gall-induction
  JOURNAL   International Journal Molecular Sciences
REFERENCE   2  (bases 1 to 6859)
  AUTHORS   Fiutek,N., Couger,B.M., Roy,S., de la Torre,J.R. and Connor,E.F.
  TITLE     Direct Submission
  JOURNAL   Submitted (10-FEB-2023) Biology, San Francisco State University,
            1600 Holloway Ave, San Francisco, CA 94132, USA
COMMENT     ##Genome-Assembly-Data-START##
            Assembly Date          :: JUN-2019
            Assembly Method        :: FLYE v. 06-2019
            Genome Representation  :: Full
            Expected Final Version :: Yes
            Genome Coverage        :: 100.0x
            Sequencing Technology  :: Illumina HiSeq; PacBio Sequel
            ##Genome-Assembly-Data-END##
FEATURES             Location/Qualifiers
     source          1..6859
                     /organism="Wolbachia pipientis"
                     /mol_type="genomic DNA"
                     /submitter_seqid="contig_34592"
                     /isolate="wEsol"
                     /isolation_source="galls on Solidago altissima"
                     /host="Eurosta solidaginis MN-2018-EFC"
                     /db_xref="taxon:955"
                     /environmental_sample
                     /country="USA: Cowling Arboretum, Carleton College,
                     Northfield, MN"
                     /lat_lon="44.48 N 93.13 W"
                     /altitude="280 m"
                     /collection_date="2018-11"
                     /metagenome_source="insect metagenome"
                     /note="metagenomic"
     gene            complement(5..1375)
                     /locus_tag="wEsol_00582"
     CDS             complement(5..1375)
                     /locus_tag="wEsol_00582"
                     /codon_start=1
                     /transl_table=11
                     /fullproduct="qcoverage 100 , hcoverage 100 , similarity
                     99.8 , identity 99.6 , evalue 3.37e-290 , alnlength 456 ,
                     Bacteria , Proteobacteria , WP_015589086.1 MULTISPECIES:
                     magnesium transporter [unclassified Wolbachia]"
                     /cog="MgtE COG2239 451 Mg/Co/Ni transporter MgtE
                     (contains CBS domain)"
                     /pfam="MgtE_N CBS MgtE"
                     /tigr="TIGR00400 mgtE 450 magnesium transporter"
                     /product="magnesium
                     transporter"
                     /translation="MNIKISSHYGLDKKAIDDLIESLDNEELENVCSIVKTIDSVQLA
                     YFLSTSISDHREKLVNILDQHLLSDALVHVVPDLQIEIIETLGIENTAKLLMLLDVED
                     IVTIVKDLDRKSVENILYYLPSKTQKSVEELLSYPEESAGRLIHKDMVIAPYYWTINQ
                     LTEFVCNYKKIPEKFHQIFIINSKLEPIGSVNLNRVISHSGDTIIKEIMDHDIKIIKT
                     GVDQEEVARIFKDYSLLSAPVVNKNGKIIGVILIEDVIKVVQQETEEDVLKISGVSSK
```

```
                     ADINAPIHKTIIKRLPWLLFNLLAAAMCSIVVGFFDDVIKSFIVLPIIMPIIASMSGN
                     AGSQTVTLTIRAIATKYLTEQNAKRILMKEFLIGLINGVILSTISLVVLAIRFHNFKV
                     EMIFVVSMIMMSIIATFIGTFIPIMLHRLKSDPAVSSSILTSATTDILSAFIFLGLAT
                     IFLLNS"
                     /besthit="qcoverage 100 , hcoverage 100 , similarity 99.8
                     , identity 99.6 , evalue 3.37e-290 , alnlength 456 ,
                     Bacteria , Proteobacteria , WP_015589086.1 MULTISPECIES:
                     magnesium transporter [unclassified Wolbachia]"
     gene            1623..2633
                     /locus_tag="wEsol_00583"
     CDS             1623..2633
                     /locus_tag="wEsol_00583"
                     /codon_start=1
                     /transl_table=11
                     /fullproduct="qcoverage 100 , hcoverage 100 , similarity
                     100 , identity 100 , evalue 3.09e-239 , alnlength 336 ,
                     Bacteria , Proteobacteria , AGJ99932.1 Peptide chain
                     release factor 2 [Wolbachia endosymbiont of Drosophila
                     simulans wHa]"
                     /cog="PrfB COG1186 239 Protein chain release factor B"
                     /pfam="Pfam-B_1081 PCRF RF-1"
                     /tigr="TIGR00020 prfB 365 peptide chain release factor 2"
                     /product="Peptide chain release factor 2"
                     /translation="MRLEELDSQASNDDLWQDNQKAQEVLKERSKIKNDVESFLKLES
                     DYNDAISLMKSAIDENDEEFFSEVENELAKLEKLIKYKETESLFTGEADNNNCFLEIH
                     SGAGGTESNDWAEMLMRMYIRWAEIYHNFKVEVVEKLEGESVGIKSAMIKIVGEKAYG
                     WAKSESGIHRLVRISPFDANGKRHTSFASVGVTPVIEDSIDIAVDEKDLKIDTYRASG
                     AGGQHVNKTESAVRITHIPTGVVVQCQNGRSQHRNKDEALKLLKGRLYQIELEKKEQK
                     MAEEYGKKCDIGWGNQIRSYVMHPYQMVKDLRTGHEVGNINSVFDGNIDCFIVSVLTK
                     QN"
                     /besthit="qcoverage 100 , hcoverage 100 , similarity 100
                     , identity 100 , evalue 3.09e-239 , alnlength 336 ,
                     Bacteria , Proteobacteria , AGJ99932.1 Peptide chain
                     release factor 2 [Wolbachia endosymbiont of Drosophila
                     simulans wHa]"
     gene            complement(2813..2977)
                     /locus_tag="wEsol_00584"
     CDS             complement(2813..2977)
                     /locus_tag="wEsol_00584"
                     /codon_start=1
                     /transl_table=11
                     /fullproduct="qcoverage 100 , hcoverage 100 , similarity
                     100 , identity 100 , evalue 2.14e-30 , alnlength 54 ,
                     Bacteria , Proteobacteria , WP_010082072.1 MULTISPECIES:
                     IS3 family transposase [Wolbachia]"
                     /cog="Tra5 COG2801 232 Transposase and inactivated
                     derivatives"
                     /pfam="rve_3 rve_2"
                     /product="IS3 family
                     transposase"
                     /translation="MSNKGCCYDNSVAESFFSSLKREILIDTSQHSAQQTRTAIFEYI
                     EIFYNKQRHY"
                     /besthit="qcoverage 100 , hcoverage 100 , similarity 100
                     , identity 100 , evalue 2.14e-30 , alnlength 54 ,
                     Bacteria , Proteobacteria , WP_010082072.1 MULTISPECIES:
                     IS3 family transposase [Wolbachia]"
     gene            complement(2970..3131)
                     /locus_tag="wEsol_00585"
     CDS             complement(2970..3131)
                     /locus_tag="wEsol_00585"
```

```
                     /codon_start=1
                     /transl_table=11
                     /fullproduct="qcoverage 100 , hcoverage 100 , similarity
                     100 , identity 100 , evalue 5.54e-28 , alnlength 53 ,
                     Bacteria , Proteobacteria , WP_010082071.1 MULTISPECIES:
                     hypothetical protein [Wolbachia]"
                     /cog="Tra5 COG2801 232 Transposase and inactivated
                     derivatives"
                     /pfam="rve"
                     /product="hypothetical protein"
                     /translation="MSSAINKQLVMNSLLMAINRRKPVKNLLLHSDQGSQYTAQGYQY
                     LLAVKNIDE"
                     /besthit="qcoverage 100 , hcoverage 100 , similarity 100
                     , identity 100 , evalue 5.54e-28 , alnlength 53 ,
                     Bacteria , Proteobacteria , WP_010082071.1 MULTISPECIES:
                     hypothetical protein [Wolbachia]"
     gene            complement(3135..3332)
                     /locus_tag="wEsol_00586"
     CDS             complement(3135..3332)
                     /locus_tag="wEsol_00586"
                     /codon_start=1
                     /transl_table=11
                     /fullproduct="qcoverage 100 , hcoverage 100 , similarity
                     100 , identity 100 , evalue 1.88e-40 , alnlength 65 ,
                     Bacteria , Proteobacteria , WP_010082070.1 hypothetical
                     protein [Wolbachia endosymbiont of Drosophila incompta]"
                     /translation="MQKNDIRAILRRKFKIKKQQTDNRAVAPNILDQNFIVDQPNKVW
                     DYLHQNQGGMAIFGSNNSHVW"
                     /product="hypothetical protein"
                     /besthit="qcoverage 100 , hcoverage 100 , similarity 100
                     , identity 100 , evalue 1.88e-40 , alnlength 65 ,
                     Bacteria , Proteobacteria , WP_010082070.1 hypothetical
                     protein [Wolbachia endosymbiont of Drosophila incompta]"
     gene            complement(3554..3847)
                     /locus_tag="wEsol_00587"
     CDS             complement(3554..3847)
                     /locus_tag="wEsol_00587"
                     /codon_start=1
                     /transl_table=11
                     /fullproduct="qcoverage 100 , hcoverage 100 , similarity
                     99.0 , identity 99.0 , evalue 6.55e-60 , alnlength 97 ,
                     Bacteria , Proteobacteria , WP_155968835.1 transposase
                     [Wolbachia pipientis]"
                     /cog="COG2963 COG2963 116 Transposase and inactivated
                     derivatives"
                     /pfam="HTH_Tnp_1 HTH_23 HTH_28"
                     /product="transposase"
                     /translation="MVREYTAEFKLEAVKLTNEQRKVGQPVAKVARDLGIRDSVLGKW
                     MKKYNEKKSAANAFPDVAPYDKERFDLQSKSNKGKRHLKKSPGQSKRVKYFFI"
                     /besthit="qcoverage 100 , hcoverage 100 , similarity 99.0
                     , identity 99.0 , evalue 6.55e-60 , alnlength 97 ,
                     Bacteria , Proteobacteria , WP_155968835.1 transposase
                     [Wolbachia pipientis]"
     gene            complement(3926..4684)
                     /locus_tag="wEsol_00588"
     CDS             complement(3926..4684)
                     /locus_tag="wEsol_00588"
                     /codon_start=1
                     /transl_table=11
                     /fullproduct="qcoverage 100 , hcoverage 100 , similarity
```

```
                     100 , identity 100 , evalue 7.50e-182 , alnlength 252 ,
                     Bacteria , Proteobacteria , WP_006280935.1 MULTISPECIES:
                     phosphate ABC transporter ATP-binding protein PstB
                     [Wolbachia]"
                     /cog="PstB COG1117 253 ABC-type phosphate transport
                     system, ATPase component"
                     /pfam="AAA_23 DUF258 SMC_N AAA_29 AAA_22 AAA_21 ABC_tran
                     AAA_21 Pfam-B_16450 Pfam-B_15070"
                     /tigr="TIGR00972 3a0107s01c2 248 phosphate ABC
                     transporter, ATP-binding protein"
                     /product="phosphate ABC
                     transporter ATP-binding protein PstB"
                     /translation="MSNTRASVKDLNLWYGSKQVLFDINLNIYKRKVTTFIGPSGCGK
                     STFLRCFNRMNDYVPGCKTVGELDIDGLGDIYSRDMDVVLLRAKVGMVFQKPNPFPKS
                     IYDNVACGPKLHGMAKDKQKLNEMVENSLTKVGLWEELKDRLQDSALNLSGGQQQRLC
                     IARAIAVKPTILLMDEPCSALDPMATNAIENLIQELKLRFTIIMVTHSMKQAKKLSDS
                     VIFFHNGRIVESGSTQEIFENAQSPLTKEYILDH"
                     /besthit="qcoverage 100 , hcoverage 100 , similarity 100
                     , identity 100 , evalue 7.50e-182 , alnlength 252 ,
                     Bacteria , Proteobacteria , WP_006280935.1 MULTISPECIES:
                     phosphate ABC transporter ATP-binding protein PstB
                     [Wolbachia]"
     gene            complement(4681..5466)
                     /locus_tag="wEsol_00589"
     CDS             complement(4681..5466)
                     /locus_tag="wEsol_00589"
                     /codon_start=1
                     /transl_table=11
                     /fullproduct="qcoverage 100 , hcoverage 100 , similarity
                     99.6 , identity 99.6 , evalue 4.31e-181 , alnlength 261 ,
                     Bacteria , Proteobacteria , WP_010962542.1 MULTISPECIES:
                     4-hydroxy-tetrahydrodipicolinate reductase [Wolbachia]"
                     /cog="DapB COG0289 266 Dihydrodipicolinate reductase"
                     /pfam="DapB_N DapB_C"
                     /tigr="TIGR00036 dapB 270 dihydrodipicolinate reductase"
                     /product="
                     4-hydroxy-tetrahydrodipicolinate reductase"
                     /translation="MKIRVGVIGCLGRMGKKILNELITNTKVEIAGAVARSGSKYIDS
                     DIGPIIANLGIKVTSSISGIFESSDVVIDFTTKECMLDCLKAAVKFKTPLVSGTTGIE
                     GVDLKEYAAEVPILWSANMSVGVNVLLKLVKKAAELLGNEYDVEIWEMHHNLKKDSPS
                     GTAIELGKTIANASKVDFQSNQCLHSGSNIRKKGGIGFAVSRGGGVIGDHSVMFVNSD
                     ERIELNHKAIDRTTFARGAVQAAVWLYENKREIPGLYSMQDVI"
                     /besthit="qcoverage 100 , hcoverage 100 , similarity 99.6
                     , identity 99.6 , evalue 4.31e-181 , alnlength 261 ,
                     Bacteria , Proteobacteria , WP_010962542.1 MULTISPECIES:
                     4-hydroxy-tetrahydrodipicolinate reductase [Wolbachia]"
     gene            complement(5873..>6859)
                     /locus_tag="wEsol_00590"
     CDS             complement(5873..>6859)
                     /locus_tag="wEsol_00590"
                     /codon_start=1
                     /transl_table=11
                     /fullproduct="qcoverage 100 , hcoverage 95.3488372093023
                     , similarity 100 , identity 100 , evalue 5.59e-235 ,
                     alnlength 328 , Bacteria , Proteobacteria ,
                     WP_174516781.1 Fic family protein [Wolbachia endosymbiont
                     of Cardiocondyla obscurior]"
                     /cog="COG3177 COG3177 348 Fic family protein"
                     /pfam="Fic DUF977"
                     /product="Fic family protein"
```

/translation="VAEIDEFKGAWQLFGNLAPERLQMLKKIATIESIGSSTRIEGAR
LSDREVEQLLSKLDTRSFRSLDEREIAGYAYVCEEVFQNFNKMPFTENTIKQFHIWLL
QFSYKDQRHMGEYKKFPNHVEAFDESGKSLGIVFETASPFETATQMQELIYWTSQQLE
VKSLHPLLIIGIFIVTFLAIHPFQDGNGRLSRILTTFLLLKLGYIYVPYSSLESNKES
YYLALRRTQQSLKTSSNDFNPWLIFFLRSLQKQKFYLEQKVSHEKVLNLYLPPLSAEI
LALLSKHGRLTIKDLENITKANCNTLKKHVSNLVKSNHITQHGKGRATWYVI"
/besthit="qcoverage 100 , hcoverage 95.3488372093023 ,
similarity 100 , identity 100 , evalue 5.59e-235 ,
alnlength 328 , Bacteria , Proteobacteria ,
WP_174516781.1 Fic family protein [Wolbachia endosymbiont
of Cardiocondyla obscurior]"

ORIGIN

```
        1 agtattagct atttaataaa aagatcgtag ctaaaccaag aaatataaaa gctgagagaa
       61 tatcagttgt tgccgatgtt aggattgaag aagaaacggc agggtcagac tttaagcgat
      121 gaagcattat aggaatgaaa gttccgatga acgttgcaat aattgacatc ataatcatgg
      181 agaccacaaa aatcatctcc actttgaagt tgtgaaacct tattgctaac actactaatg
      241 aaatagtaga taagatcacc ccgtttataa gacctattaa aaattctttc atcagtattc
      301 tttttgcatt ttgctcagtt agatattttg ttgcgattgc ccggatggtt agcgttactg
      361 tttgggatcc tgcatttccg ctcattgatg caattatcgg catgattatt ggcagcacta
      421 taaaactttt tattacatca tcgaaaaagc caactactat ggaacacatt gctgcagcta
      481 agaggttaaa cagtaaccaa ggtagccttt taattatagt tttatgtata ggggcattta
      541 tatcggcttt agaagataca ccgcttattt tgagtacatc ctcttctgtt tcttgttgaa
      601 caacttttat cacatcttca attaagatca caccaataat tttaccattc ttatttacta
      661 ccggagccga taatagagag taatctttaa atattcttgc tacttcctct tggtctactc
      721 cagttttaat gatttttatg tcatgatcca ttatctcttt tattattgtg tctcctgagt
      781 gagatattac cctatttaaa ttaacactgc ctataggctc taattttgag ttgataatga
      841 atatctggtg aaattttttct ggtatttttt tatagttgca cacaaattct gtcagttgat
      901 ttattgtcca ataatatgga gctataacca tatctttatg tattaacctt cctgcacttt
      961 cttctggata tgatagcaac tcctctaccg atttctgagt tttgctgggc aaatagtaaa
     1021 gtatattttc tacagacttt ctatctaaat ctttcactat ggttactata tcttctacat
     1081 caaggagcat tagtaacttt gctgtatttt ctattcccaa tgtttctatg atctctattt
     1141 gtaaatctgg cactacatgc actagggcat cacttaacaa atgttgatcg aggatattaa
     1201 ctaatttctc cctgtgatca ctgattgaag tagataaaaa ataagctaac tgaacgctat
     1261 ctatcgtttt tacaatacta caaacattct ctagctcctc gttatcaagt gactctatta
     1321 agtcatcaat agcctttttg tctaaaccat aatgagaact tattttaata ttcattactt
     1381 taccttattt tttaataaat atacgcgctt gatttcctta aacattaaag tataatgaaa
     1441 ttatttaaat taactatcat atggttaaat ttctattttg tttactgtta ttgttattga
     1501 tagtaaatat attaaaatct aaagtataat gaaaacttat ccagaaattc ttgaacactt
     1561 tcaaagctta aataaaagta tttctcttat caggaggtgt ctttgacata gaaaaattaa
     1621 agttgcgcct tgaagagctt gactcccaag cgtcgaatga tgacctatgg caagataacc
     1681 aaaaagcaca agaagtttta aaagaacgtt ctaaaattaa gaatgacgta gagtcgtttt
     1741 taaagctgga aagcgattac aacgatgcca tcagcttaat gaagtctgct attgatgaaa
     1801 atgatgaaga atttttctct gaagttgaaa atgaattagc taagctagag aaattaatca
     1861 agtataaaga aacagagtcc ttatttactg gtgaagcaga taataataac tgctttttag
     1921 aaatccactc aggagctggc ggaaccgaga gcaatgattg ggctgaaatg ctgatgcgca
     1981 tgtatataag gtgggcagaa atttatcaca actttaaagt tgaagttgta gaaaaattag
     2041 aaggagagtc agttggtata aagtctgcaa tgataaaaat cgttggagaa aaagcttatg
     2101 ggtgggcaaa aagtgaaagt ggaattcaca ggctggttag aatatcgccg tttgatgcaa
     2161 atggtaaacg tcataccagt tttgcaagtg taggagtaac tccagtgata gaagattcaa
     2221 ttgatattgc tgtggatgaa aaggatctaa agatcgacac ctaccgagct tctggagcag
     2281 gcggtcagca tgtgaacaaa actgaaagtg cggtacgcat tacgcatatt ccaactggtg
     2341 ttgtagttca atgccaaaat ggtcgttctc aacacagaaa taaagatgag gcattgaaat
     2401 tacttaaagg acgtttatac caaattgaac tggaaaaaaa agaacagaaa atggctgaag
     2461 aatatggtaa aaaatgcgat ataggctggg gtaatcagat cagatcgtac gttatgcatc
     2521 catatcaaat ggtgaaggat ttaagaaccg gacatgaagt gggcaatata aattctgttt
     2581 ttgatggtaa tatagattgt ttcatagtta gtgtgcttac taagcaaaat taggtagggt
     2641 tgactttctt gccataatat atgataatta aaatattgtt aaagtaggag taaagttatg
     2701 gcgattaaat atgatcagtg gaaaagtgac cttacccaga aaaagtggag agaaagtgtg
     2761 gaatgttttt tacaaaaaga atgatgcatc aaattgttct ggaatgcaat agttaatagt
     2821 gacgttgttt gttataaaaa atttctatgt attcaaatat tgcagttcta gtttgttgag
```

2881 cagagtgttg tgaagtatca ataagtattt ctcttttcaa tgagctaaag aagctttctg
2941 caacagaatt gtcgtaacaa cagcctttat tactcatcta tattctttac agctaagaga
3001 tattgatacc cttgcgcagt atattgtgaa ccttggtcac tgtgcagcag tagattttta
3061 acaggtttac gcctattaat ggccattaat aaagagttca taaccaattg tttatttatt
3121 gcactactca ttaactacca tacgtgagaa ttattgctgc caaatatagc catccctcct
3181 tggttttgat gtaagtaatc ccatacttta tttggttgat caacaataaa gttttgatct
3241 agtatattgg gagctacagc tctattatct gtttgttgtt ttttaatttt aaactttctt
3301 ctcagtatag ccctgatgtc atttttctgc ataatacttt gcaccgtttt caaattgcaa
3361 cttttaccca aagccttcaa ttcagcatga attttaggag ctccatatct acatttagaa
3421 gcttgatata ttttttgaat atctgctaat aactctttat ttgctgattc tctactgctt
3481 atttttcttg tggtccactt atagtagcca ctagcagaaa atcctacata attcctgtac
3541 tttatagcag ttactatata aaaaaatatt ttactctttt tgactggcca gggctttttt
3601 ttaaatgtct ctttcccttg ttactcttgc tttgtaaatc aaacctctct ttgtcataag
3661 gtgctacatc tggaaatgca tttgccgctg actttttttc gttatatttc ttcatccatt
3721 ttcctaatac actatccctt attcctagat ctcttgctac ttttgcaact ggttgaccta
3781 cctttctttg ttcatttgtc agcttaacag cttccaattt gaattctgcc gtatattctc
3841 ttaccatttc taatcctcca aaattttatt ttacccaaaa aaactctttt ctttctctcc
3901 actttttctg ggtaaggtca gaaatttaat gatccaggat atactccttt gttaagggag
3961 attgagcatt ttcgaatatt tcttgagtgc ttccagactc aacaattctg ccattatgaa
4021 agaaaattac actatcagat aacttttttag cttgcttcat tgaatgagtc accataatta
4081 tagtaaatct taatttcagc tcttgtataa gattttcgat tgcattggtt gccattggat
4141 caagagcaga gcacggttca tccattaata aaatagttgg cttcactgca attgcgcgag
4201 cgatgcatag cctctgttgt tggccaccgg ataaattgag tgcgctatct tgcaatctgt
4261 ccttcaattc ttcccataaa ccaaccttgg ttaaactatt ttccaccatc tcatttaatt
4321 tttgcttatc cttcgccata ccatgcaatt ttggcccaca agcaacatta tcatatattg
4381 attttggaaa aggatttggc ttttgaaata ccataccaac ttttgctcta agtagtacaa
4441 catccatatc gcgtgaatat atatcaccca gcccatcgat atctaattca ccaacagttt
4501 tgcaacctgg tacatagtca ttcatacgat taaaacaacg caaaaatgtt gatttaccgc
4561 acccggatgg tccgataaaa gtagtaactt ttcttttata aatattcaaa tttatatcga
4621 ataaaacttg cttagagccg taccagagat ttaaatcttt aacagaggca cgtgtattgc
4681 tcatatcaca tcctgcattg aatagagccc cggaatttct cttttatttt cgtatagcca
4741 tactgctgct tgaacagctc ctctagcaaa tgttgtgcga tcaattgctt tgtggtttag
4801 ttctatccgt tcatcagaat taacaaacat cacgctgtga tctcctatta ctccacctcc
4861 acgagatact gcaaaaccta ttcccccctt tttccttatg tttgaaccac tatgtaaaca
4921 ttgattggac tgaaaatcca cttttgaagc gttagcaatt gttttgccaa gttctatagc
4981 tgttccagat ggtgaatcct tttttaagtt atgatgcatt tcccaaatct caacgtcata
5041 ttcattacct aaaagctcag cagctttttt taccaatttc agcaacacat taaccccaac
5101 actcatattt gctgaccata atattggaac ttcagcagca tattctttca aatcgacacc
5161 ttctattcca gttgtgccac taaccagcgg tgttttaaat tttacagctg ctttaaggca
5221 gtctaacata cattctttag ttgtaaaatc tattacaacg tcagatgact caaatatgcc
5281 actaatagaa cttgtaactt taattcctag attggctata attggtccaa tatctgaatc
5341 tatgtactta ctacctgaac gggcaacagc acctgctatt tccactttgg tatttgtaat
5401 taattcattg agtatttttt tgcccattct gcctaagcag cctattactc caactctgat
5461 tttcataaag ctttttacgt ttattaagaa tgttaggcct ttttttaagc tctgtcactt
5521 ctgatctata ttctatagct gtacgaacac tgcaatttca gatcccagtg ccttgaccac
5581 aactattgca atatggtaca tagtgaacga tgtcattcca gtctggaatc tagcctttct
5641 gcaatctcat cgaaaacgtt gtaaccactt tctatgctag tttgcttgct tacaagcaaa
5701 cttttctgga tcccagtgtc tgggcactgg gatgacagga gaaggcactg ggatgacaag
5761 aatagggcac tgggatctag tgggctcaaa ctacaatgtt tgtacagctg tgatctatat
5821 tctatagctg aataatttaa caattcaaaa tgaatacata tcctattgag ctctatatta
5881 cataccaggt tgctctgcct tttccatgct gagtaatgtg attactcttt acgagattag
5941 atacgtgctt ctttaaggta ttgcagtttg ctttagtaat attttccaaa tccttaattg
6001 taagacgtcc atgtttgctg agtagtgcta gtatttcagc agaaagtggt ggtaaataga
6061 gatttaacac tttttcatgt gacacctttt gttcaagata aaatttttgc ttttgtagtg
6121 accttaaaaa gaatataagc catggattaa aatcattgct gctcgtcttc aaggattgtt
6181 gagtacgacg caaagctaag tagtaactct ccttattact ttcaagggaa ctgtaaggta
6241 catagatata ccctaacttt agtaacaaga aagttgtgag aatgcgtgat aatctgccat
6301 ttccatcctg gaagggatga atagctaaaa aagtaactat aaaaatgcca ataattaata
6361 gagggtgtaa cgatttcacc tctaattgct ggctagtcca ataaataagt tcttgcattt
6421 gtgttgctgt ctcaaatgga gaggctgttt caaaaactat gcccaagctt tttccagatt

```
     6481 catcaaacgc ttcaacatga ttggggaatt ttttatattc tcccatgtgc ctttgatctt
     6541 tatatgaaaa ttgtaaaagc cagatgtgaa actgttttat agtgttttct gtaaatggca
     6601 tcttgttaaa gttttgaaat acttcttcac acacatacgc atatccggct atttctcgtt
     6661 catctagaga acgaaatgag cgtgtatcaa gtttggatag taattgctca acttcacggt
     6721 ctgataatct tgccccttca atacgagtag aagaaccaat actttcaatt gttgcaattt
     6781 tcttgagcat ttgtaaacgt tctggagcta aattaccaaa caattgccat gctcctttaa
     6841 attcatcaat ttcagcaac
//
LOCUS       JAQZAU010000056            8361 bp    dna     linear   ENV 17-MAR-2023
DEFINITION  MAG: Wolbachia pipientis isolate wEsol contig_34920, whole genome
            shotgun sequence.
ACCESSION   JAQZAU010000056  JAQZAU010000000
VERSION     JAQZAU010000056.1
DBLINK      BioProject: PRJNA929258
            BioSample: SAMN33142973
KEYWORDS    WGS; ENV; Metagenome Assembled Genome; MAG.
SOURCE      Wolbachia pipientis (insect metagenome)
  ORGANISM  Wolbachia pipientis
            Bacteria; Pseudomonadota; Alphaproteobacteria; Rickettsiales;
            Anaplasmataceae; Wolbachieae; Wolbachia.
REFERENCE   1  (bases 1 to 8361)
  AUTHORS   Fiutek,N., Couger,B.M., Roy,S., de la Torre,J.R. and Connor,E.F.
  TITLE     Genomic assessment of the contribution of the Wolbachia
            endosymbiont of Eurosta solidaginis to gall-induction
  JOURNAL   International Journal Molecular Sciences
REFERENCE   2  (bases 1 to 8361)
  AUTHORS   Fiutek,N., Couger,B.M., Roy,S., de la Torre,J.R. and Connor,E.F.
  TITLE     Direct Submission
  JOURNAL   Submitted (10-FEB-2023) Biology, San Francisco State University,
            1600 Holloway Ave, San Francisco, CA 94132, USA
COMMENT     ##Genome-Assembly-Data-START##
            Assembly Date          :: JUN-2019
            Assembly Method        :: FLYE v. 06-2019
            Genome Representation  :: Full
            Expected Final Version :: Yes
            Genome Coverage        :: 100.0x
            Sequencing Technology  :: Illumina HiSeq; PacBio Sequel
            ##Genome-Assembly-Data-END##
FEATURES             Location/Qualifiers
     source          1..8361
                     /organism="Wolbachia pipientis"
                     /mol_type="genomic DNA"
                     /submitter_seqid="contig_34920"
                     /isolate="wEsol"
                     /isolation_source="galls on Solidago altissima"
                     /host="Eurosta solidaginis MN-2018-EFC"
                     /db_xref="taxon:955"
                     /environmental_sample
                     /country="USA: Cowling Arboretum, Carleton College,
                     Northfield, MN"
                     /lat_lon="44.48 N 93.13 W"
                     /altitude="280 m"
                     /collection_date="2018-11"
                     /metagenome_source="insect metagenome"
                     /note="metagenomic"
     gene            20..274
                     /locus_tag="wEsol_00591"
     CDS             20..274
                     /locus_tag="wEsol_00591"
                     /codon_start=1
```

```
                     /transl_table=11
                     /pfam="PAAR_motif DUF4150 PAAR_motif"
                     /fullproduct="qcoverage 100 , hcoverage 100 , similarity
                     100 , identity 100 , evalue 9.03e-34 , alnlength 84 ,
                     Bacteria , Proteobacteria , WP_197907399.1 MULTISPECIES:
                     PAAR domain-containing protein [unclassified Wolbachia]"
                     /translation="MGKAIVCVGDYCSGIPAHVCMSGSNDVFVNGRSVCRKGDILTMG
                     ETLTQGSNSVFVNDIGITRTGDLASCGFHVMSISKNVFAS"
                     /product="PAAR
                     domain-containing protein"
                     /besthit="qcoverage 100 , hcoverage 100 , similarity 100
                     , identity 100 , evalue 9.03e-34 , alnlength 84 ,
                     Bacteria , Proteobacteria , WP_197907399.1 MULTISPECIES:
                     PAAR domain-containing protein [unclassified Wolbachia]"
     gene            277..612
                     /locus_tag="wEsol_00592"
     CDS             277..612
                     /locus_tag="wEsol_00592"
                     /codon_start=1
                     /transl_table=11
                     /fullproduct="qcoverage 100 , hcoverage 100 , similarity
                     100 , identity 100 , evalue 1.09e-69 , alnlength 111 ,
                     Bacteria , Proteobacteria , WP_197907398.1 MULTISPECIES:
                     GPW/gp25 family protein [unclassified Wolbachia]"
                     /cog="COG3628 COG3628 116 Phage baseplate assembly
                     protein W"
                     /pfam="DUF2634 GPW_gp25 Pfam-B_2001"
                     /product="GPW/gp25 family
                     protein"
                     /translation="MKGMSKETGKELEGIEHLKQSIIDILTTPIGSRVMRRDYGSRLL
                     ELVDKPINRDLTLEIYAAVAEALQKLEQRFKLEKVKITEVKEGKVNISLDGIYLPNGK
                     NIHFGGIVV"
                     /besthit="qcoverage 100 , hcoverage 100 , similarity 100
                     , identity 100 , evalue 1.09e-69 , alnlength 111 ,
                     Bacteria , Proteobacteria , WP_197907398.1 MULTISPECIES:
                     GPW/gp25 family protein [unclassified Wolbachia]"
     gene            615..1337
                     /locus_tag="wEsol_00593"
     CDS             615..1337
                     /locus_tag="wEsol_00593"
                     /codon_start=1
                     /transl_table=11
                     /pfam="Baseplate_J"
                     /fullproduct="qcoverage 109.583333333333 , hcoverage 100
                     , similarity 91.3 , identity 91.3 , evalue 2.80e-156 ,
                     alnlength 263 , Bacteria , Proteobacteria ,
                     WP_209452514.1 baseplate J/gp47 family protein [Wolbachia
                     endosymbiont of Ceratosolen solmsi]"
                     /translation="MEQPNIIEPLNFNDPAIKVLEVAAWRELLLRERINEAVKSNLLK
                     FAGGEDLDRLAEFYGVEREKEEDDERFRKRIKARIVGWSTGGNYRFQALSADRRVKDA
                     LVESKVPGSVEISILSTELSTNGIASEELLDIVGKQVTRDDIRVLTDTITVVGCNIIE
                     INIHSRISIKRPDIIETVKKKFIEKFETTKRLGWKVTRSWIIANLFVEGVENVELIEP
                     REDVVVLGNECAALRSLNVELN"
                     /product="baseplate J/gp47 family protein"
                     /besthit="qcoverage 109.583333333333 , hcoverage 100 ,
                     similarity 91.3 , identity 91.3 , evalue 2.80e-156 ,
                     alnlength 263 , Bacteria , Proteobacteria ,
                     WP_209452514.1 baseplate J/gp47 family protein [Wolbachia
                     endosymbiont of Ceratosolen solmsi]"
     gene            1337..2488
```

```
                     /locus_tag="wEsol_00594"
     CDS             1337..2488
                     /locus_tag="wEsol_00594"
                     /codon_start=1
                     /transl_table=11
                     /pfam="Pfam-B_16372 Tail_P2_I"
                     /fullproduct="qcoverage 100 , hcoverage 100 , similarity
                     100 , identity 100 , evalue 6.01e-288 , alnlength 383 ,
                     Bacteria , Proteobacteria , WP_209452515.1 phage tail
                     protein [Wolbachia endosymbiont of Ceratosolen solmsi]"
                     /translation="MLLPPNATKQEKALVDAVDYKVDPGCIRGFKFSPGEKVLPWIIE
                     EYGLGEILHWVTDKRKAIKEGIKFQRLRGTPESLKIALKWANIEDITIIEEPPGKHFF
                     ELQVGIRDVPFVDAVVELAKLSLPARSRLMRLFNDHYNIGRFILDESFFGSLLSDYSG
                     VKIEKDGPVLSFGRVNFFRSSGPVIRIIENYLRDHYERALSNDIYRLDVAILGETEPH
                     TKNYNGIYERNHVWYNFKALYPLPQSLLPAIKFAKAQIVLSDSWNLGEINACFPVTGI
                     EEEGDKFLLGSSKLSEQLWNLKYKPILERFSVTHHYKVEDFTNQKVIRFGLAEHNVHF
                     ESDLNSEQKDSIHEPENYILVFYPGVLTWHEHRHLNRSWEEKQVICLMS"
                     /product="phage tail protein"
                     /besthit="qcoverage 100 , hcoverage 100 , similarity 100
                     , identity 100 , evalue 6.01e-288 , alnlength 383 ,
                     Bacteria , Proteobacteria , WP_209452515.1 phage tail
                     protein [Wolbachia endosymbiont of Ceratosolen solmsi]"
     gene            2662..3144
                     /locus_tag="wEsol_00595"
     CDS             2662..3144
                     /locus_tag="wEsol_00595"
                     /codon_start=1
                     /transl_table=11
                     /product="hypothetical protein"
                     /pfam="Ank_4 Ank_2 Pfam-B_14646 Ank Ank_3 Ank_4 Ank_5
                     Pfam-B_10896 Ank_2 Ank Ank_3 Ank_4 Ank_2 Ank_5 Ank Ank_3
                     Ank_4"
                     /translation="MWISQEERDDFNELFQELLAGKLSKKHINRKNENGETMLHRAAK
                     MSTRKKVNWLIWEGADVDARDNEGYTPLHLAALGMRLENVKELIEARADINATEEDGN
                     TALHLACMVGGKKIVEELIKAGAEINRVSISGFSPMYYASDEEISEVLKKNGGKIVNK
                     "
                     /cog="Arp COG0666 235 FOG: Ankyrin repeat"
                     /besthit="qcoverage 100 , hcoverage 79.6019900497513 ,
                     similarity 100 , identity 99.4 , evalue 3.48e-49 ,
                     alnlength 160 , Bacteria , Proteobacteria ,
                     WP_174515614.1 ankyrin repeat domain-containing protein
                     [Wolbachia endosymbiont of Cardiocondyla obscurior]"
     gene            3154..3267
                     /locus_tag="wEsol_00596"
     CDS             3154..3267
                     /locus_tag="wEsol_00596"
                     /codon_start=1
                     /transl_table=11
                     /product="hypothetical protein"
                     /translation="MEKISKVSEKQVVNGGKKLNDREKLTTAGGRFNRLSI"
                     /besthit="qcoverage 100 , hcoverage 18.407960199005 ,
                     similarity 100 , identity 100 , evalue 1.55e-13 ,
                     alnlength 37 , Bacteria , Proteobacteria , WP_174515614.1
                     ankyrin repeat domain-containing protein [Wolbachia
                     endosymbiont of Cardiocondyla obscurior]"
     gene            3444..4025
                     /locus_tag="wEsol_00597"
     CDS             3444..4025
                     /locus_tag="wEsol_00597"
                     /codon_start=1
```

```
                     /transl_table=11
                     /fullproduct="qcoverage 100 , hcoverage 100 , similarity
                     100 , identity 100 , evalue 1.78e-103 , alnlength 193 ,
                     Bacteria , Proteobacteria , WP_197907395.1 ankyrin repeat
                     domain-containing protein [Wolbachia endosymbiont of
                     Kradibia gibbosae]"
                     /cog="Arp COG0666 235 FOG: Ankyrin repeat"
                     /pfam="Ank_4 Pfam-B_10896 Ank_2 Ank Ank_3 Ank_4 Ank_5
                     Ank_4 Ank_3 Ank Ank_2 Ank_5 Ank Ank_4 Ank_3 Pfam-B_19574"
                     /product="ankyrin repeat domain-containing
                     protein"
                     /translation="MVKFSKKEREEFNKSWKEVLDNPIENINKKDTKGRTILHYAVGM
                     LDPKKVRLLIEKGADLDAADAGKYRPLHLAVMGQRLENIKELIKAGVEVNVVERSSKF
                     AALHLACMVSEVKIVEELVKAGANVEQKDKYGKTPMDYARNNKEIKEVLENVKMANKQ
                     REFIERIRVVSESTTAGVIAAKEELETVDEKVL"
                     /besthit="qcoverage 100 , hcoverage 100 , similarity 100
                     , identity 100 , evalue 1.78e-103 , alnlength 193 ,
                     Bacteria , Proteobacteria , WP_197907395.1 ankyrin repeat
                     domain-containing protein [Wolbachia endosymbiont of
                     Kradibia gibbosae]"
     gene            4068..4499
                     /locus_tag="wEsol_00598"
     CDS             4068..4499
                     /locus_tag="wEsol_00598"
                     /codon_start=1
                     /transl_table=11
                     /pfam="DUF2924"
                     /fullproduct="qcoverage 100 , hcoverage 100 , similarity
                     100 , identity 100 , evalue 3.67e-90 , alnlength 143 ,
                     Bacteria , Proteobacteria , WP_197907394.1 MULTISPECIES:
                     DUF2924 domain-containing protein [unclassified
                     Wolbachia]"
                     /translation="MEKKVEKEVKCLEKKPLAELKKIWKKVFEEEAPRHSKKYLIPRL
                     AYRLQEKAYGEISRKGAKRLEYLADRLEKGKRISSDKLPVEGTELILERGEEVHVVMV
                     TNTGLIYREEFYTSLSAVAGKIMGMSYNGPLLFGMRDKKVS"
                     /product="DUF2924
                     domain-containing protein"
                     /besthit="qcoverage 100 , hcoverage 100 , similarity 100
                     , identity 100 , evalue 3.67e-90 , alnlength 143 ,
                     Bacteria , Proteobacteria , WP_197907394.1 MULTISPECIES:
                     DUF2924 domain-containing protein [unclassified
                     Wolbachia]"
     gene            4510..6033
                     /locus_tag="wEsol_00599"
     CDS             4510..6033
                     /locus_tag="wEsol_00599"
                     /codon_start=1
                     /transl_table=11
                     /pfam="Resolvase Recombinase Zn_ribbon_recom Pfam-B_11632
                     Pfam-B_1335 Bot1p Pfam-B_16452"
                     /fullproduct="qcoverage 100 , hcoverage 100 , similarity
                     100 , identity 100 , evalue 0.0 , alnlength 507 ,
                     Bacteria , Proteobacteria , WP_209452274.1 recombinase
                     family protein [Wolbachia endosymbiont of Ceratosolen
                     solmsi]"
                     /translation="MLKEVRCAIYTRKSNEDGLEQKFNSLDAQRVACEKYIKSREGWV
                     ALAKRYDDGGYSGSNLNRPAIKELFEDVKAGEVDCVVVYTLDRLSRETKDCIEVTSFF
                     RRHRISFVAVTQIFDNNTPMGKFVQTVLSGAAQLEREMIVERVKNKIATSKEEGLWMG
                     GNPPLGYDVKDKELIINEKEAKVIKHIFERYMELKSMAELARELNREGYRTKKFEAKS
                     GKVYGGEIFKKATVRRIITNPIYMGKTRHYEKQYKGKHEAIIEEEKWKKAQELIKDQP
```

```
                     YRKAKYEEALLKGIIKCKSCDVNMTLTYSKKENKRYRYYVCNNHLRGKDCESGNRNVI
                     AGEVEKEVMKRAERLYENWERGEEKWENLSFGKQKEVVKKLVKGVMVKEDGIEVHSES
                     EKTFIAMNLKKKGNKCTVVEPEGKTNNALLKAVVRAHLWKRQLEERKYASLKELSVKI
                     NIGTRRIQQILRLNYLAPKIKEDIVNGRQPSNLRLVDLREIPTLWSEQMEKFYGLVS"
                     /product="recombinase family protein"
                     /besthit="qcoverage 100 , hcoverage 100 , similarity 100
                     , identity 100 , evalue 0.0 , alnlength 507 , Bacteria ,
                     Proteobacteria , WP_209452274.1 recombinase family
                     protein [Wolbachia endosymbiont of Ceratosolen solmsi]"
     gene            6065..6802
                     /locus_tag="wEsol_00600"
     CDS             6065..6802
                     /locus_tag="wEsol_00600"
                     /codon_start=1
                     /transl_table=11
                     /fullproduct="qcoverage 100 , hcoverage 100 , similarity
                     100 , identity 100 , evalue 8.48e-155 , alnlength 245 ,
                     Bacteria , Proteobacteria , WP_206759920.1 hypothetical
                     protein [Wolbachia endosymbiont of Kradibia gibbosae]"
                     /translation="MKTPKKIWKSIAKKFKNSTAAKANVSSSADKDILEEGKKLSEEV
                     LNKEVTIKQQVVTGKTESVSLIQDTASIIGNIETPADAYENSNKSSLEEFIEHKPCEN
                     SNESNTIAIEHIATSIEKLDNTGICEKNETAVTETDIQEQNASNSLSSAACFDNGSSK
                     ENTADKKEKPSMQQRQAILAGVVGAALLVSSIIAYLLKMYAIAVIGGIVGLACVSYAL
                     YSVLKPNTKLEKVEEQSITAHPPLNPT"
                     /product="hypothetical protein"
                     /besthit="qcoverage 100 , hcoverage 100 , similarity 100
                     , identity 100 , evalue 8.48e-155 , alnlength 245 ,
                     Bacteria , Proteobacteria , WP_206759920.1 hypothetical
                     protein [Wolbachia endosymbiont of Kradibia gibbosae]"
     gene            complement(6924..>8361)
                     /locus_tag="wEsol_00601"
     CDS             complement(6924..>8361)
                     /locus_tag="wEsol_00601"
                     /codon_start=1
                     /transl_table=11
                     /product="hypothetical protein"
                     /pfam="HrpB7 Baculo_PEP_C Pfam-B_8957 Pfam-B_13482
                     Pfam-B_15144"
                     /translation="EQGNTQLIDLFSQSQKKESVPLSSEQNFEESDDEFYDAEESILK
                     GASEDESEVNTQQATVEESSNGQSGDQKSPEPESTGSQQIEKNVLSSTKESNTINNVG
                     TSMEEPVSNDFSEGKMPTNTQQVGSSLGQEEPENMRNQLEKLQKELNEKTQALESTKT
                     QLAEKEEKLKGAQVQLETKEAELQNELTEKDVQLKNAQAQLEQEKTKLQDALKKKEEE
                     LESKIGKVTKLNGTIEKLEAQLKEQKGELDSTRQDLDSKTSEVTKLITKIEELERTKV
                     QLAEKEKALGIVQKSLADEDVKVTQLTSETNKLNATIGTLNTQLTKQKEALGSANQEV
                     TRLKNTIEELEGIKNQLTEKEQELNSTKQDLVKKASEVTQLSSENIQLQTQNLNAKNK
                     KLLAVSAQSRKQVTYASVSFVLSGAFAVGASLTIPYLAICITFAVAASIFLAVGCYCS
                     YKANTALSNVEVKNGIDSAAVEVLNSPS"
                     /besthit="qcoverage 100 , hcoverage 44.2592592592593 ,
                     similarity 86.8 , identity 86.6 , evalue 1.06e-179 ,
                     alnlength 478 , Bacteria , Proteobacteria ,
                     WP_197907392.1 ankyrin repeat domain-containing protein
                     [Wolbachia endosymbiont of Kradibia gibbosae]"
ORIGIN
        1 aataaatcta aactagtaga tgggtaaagc aatcgtttgt gtaggagatt attgcagtgg
       61 aataccagca catgtttgta tgagtggtag taacgatgtt tttgtaaacg gtagatctgt
      121 gtgtcgaaaa ggagatatct taactatggg agagacacta acgcaaggat caaacagcgt
      181 atttgttaat gatattggca taacaagaac cggtgacttg gcatcgtgtg gttttcatgt
      241 aatgagtatt agcaaaaatg tatttgcgag ctaacaatga aagggatgag caaagaaaca
      301 ggtaaagaat tagaaggaat agaacatctg aaacaatcaa taattgatat actgaccaca
      361 ccgataggat cacgtgttat gcgaagggat tatgggtcaa gactacttga attagtagat
```

421 aagccaatta atagggattt aactttggaa atctatgcag cagtagcaga agctctacaa
481 aagttggagc aaagatttaa gttagaaaaa gtaaaaatta cagaagtgaa agaaggaaag
541 gtcaatattt cattagatgg tatttatcta ccaaatggaa aaaatattca ttttggtggg
601 attgtggtat agagatggag cagcctaaca ttattgagcc actgaacttt aatgatccag
661 caataaaggt tttagaagta gcagcatggc gagaactttt gctgagagaa agaataaacg
721 aagcggtaaa aagtaattta cttaagttcg caggaggaga agacttagat cgtttagctg
781 agttttacgg agttgagaga gaaaaagaag aagatgacga acgatttaga aaaagaatca
841 aggcaagaat agttggctgg agcactggag gaaattatag gtttcaagca ctctcagcag
901 atagaagagt aaaggatgcg ctagttgaat caaaagtgcc aggaagtgta gagatctcga
961 ttttatccac ggagttatcc acaaatggca tagcgtctga agaactactt gatattgtag
1021 gaaagcaagt caccagggat gatataaggg tattaacaga tacgataaca gtagttggtt
1081 gcaatattat tgaaataaat atccacagca gaattagtat taaaagacca gatattattg
1141 aaacggtgaa gaagaaattt atagaaaaat ttgaaacaac gaaaagattg ggatggaagg
1201 tgacaagatc atggattata gcgaatttgt tcgtagaagg agtagaaaac gtagaattaa
1261 tagagccaag agaggatgtt gtggtattgg ggaatgagtg tgctgcctta agaagtttaa
1321 atgttgagtt gaattaatgc tattaccacc aaatgcaaca aaacaagaaa aagcgctggt
1381 tgatgcagta gattacaaag tagatcctgg ttgtatcaga gggtttaaat ttagcccagg
1441 ggaaaaagta ttaccatgga taattgaaga gtacgggctg ggagaaatcc tgcattgggt
1501 aacagacaaa agaaaagcga taaaagaagg gataaaattt caaagactgc gtggcacacc
1561 agaatcactt aaaatagcat taaaatgggc aaatatagaa gatattacga tcattgaaga
1621 gccacccggt aaacactttt ttgagttgca ggtagggata agagatgtac cgttcgtaga
1681 tgcagtagta gaactagcaa aactatcatt gccagcaaga tcgaggctaa tgaggctttt
1741 caatgaccac tataatattg gaagatttat tttagatgaa agctttttgg gcagtttgtt
1801 atcggattat tcaggggtaa aaatagaaaa agatggacca gtattatcat ttggaagagt
1861 aaatttttc aggtctagtg gtccagttat taggattata gaaaactatc tacgcgatca
1921 ttatgaacga gctttaagca atgacatata tcgcttggat gtagcaatcc ttggagaaac
1981 cgagcctcat actaagaact ataacggtat ttatgaaaga aatcatgtgt ggtataactt
2041 caaagcgcta tatccgctac cacagagctt actaccggca attaagtttg ctaaagcgca
2101 gatagtatta tcagatagct ggaacttagg agaaataaac gcatgttttc cggttactgg
2161 tatagaagag gaaggggata aatttttatt gggaagtagc aaactatctg aacaactgtg
2221 gaatttaaaa tacaagccaa ttttagaaag gtttagcgtt actcaccact acaaggtaga
2281 agatttacc aaccaaaaag ttataagatt tggtttagca gaacacaatg ttcattttga
2341 aagcgattta aattcagagc aaaaggattc aatacatgaa ccggaaaatt acattttagt
2401 gttttaccca ggtgtactaa cgtggcacga acatcgacat ttgaacagaa gttgggaaga
2461 aaaacaggtc atatgtttaa tgagttaaat atatatactt atatcttcat acactacatt
2521 aacattcggt aaaaatgtgt gtttttgaa aaaaagaagt tgcttttcca tgccaaaaca
2581 gctataaatt aggtaggtgt aggtaaacaa aaaagattta ccaaacgcta gaagttagga
2641 agttttatag tgaggtacat catgtggatt agtcaagaag aaagggatga ttttaatgaa
2701 ttatttcaag agttgcttgc aggcaaattg tctaaaaagc atataaatag aaaaaatgaa
2761 aacggtgaaa caatgttgca ccgagcagca aaaatgtcga cgagaaaaaa agtaaactgg
2821 ctgatatggg aaggagcaga tgtagatgca agagataacg aaggttatac accactgcac
2881 ttagcagcgc ttggaatgcg tctggaaaac gtaaaagaat taatagaagc cagagcagat
2941 ataaacgcaa ctgaagaaga tggaaatacg gcactacatc tagcgtgtat ggtaggtgga
3001 aaaaagatag tagaagagtt aataaaagca ggggcagaaa taaaccgggt aagtatatca
3061 ggatttagcc caatgtatta cgcaagtgat gaagagataa gcgaggtgtt aaagaaaaat
3121 ggaggaaaaa tagtgaataa atagagggag ttaatggaaa aaataagtaa ggtatcggaa
3181 aagcaggtgg taaatggtgg aaagaagcta aatgatagag aaaagctgac aacagcagga
3241 ggtagattta atagattaag tatatagatt tatatcttaa tataatatgc ggaaaaaatg
3301 tgtatatttt gtaaaaaaaa acttgctttt tatgccaaaa cagccctaag tgaaaaagta
3361 gctagtaaac aaacaagatt tgctaagcgc taaagtgagc caaattggag ccactttata
3421 ttgtattaat agctgaggaa cttatggtaa aatttagtaa gaaagaaaga gaagaattta
3481 ataagtcgtg gaaagaagta ttagataacc caatagaaaa tattaataaa aaagacacaa
3541 aaggaaggac gatattacat tatgcagtag ggatgctaga tccaaaaaaa gtgaggttat
3601 taatcgaaaa aggagcagat ttagatgcag cagatgccgg gaaatataga ccactacacc
3661 tagcagtgat ggggcaacgt ctagaaaata taaaagagct gataaaggcg ggagtagagg
3721 taaacgtagt ggaacgtagt agcaaatttg ccgcgttaca cctggcatgc atggtcagtg
3781 aggtaaaaat agtagaagaa ctagtaaaag cgggagcaaa tgtagagcaa aaggataaat
3841 atggcaaaac cccaatggat tatgcgagaa ataataaaga gataaaagaa gtgttagaga
3901 acgtaaaaat ggcaaataaa cagagagagt ttatagaaag aataagggtt gtatcggaaa
3961 gtacgacagc aggtgtgata gcggcaaagg aagagctaga aactgtggat gagaaagtgc

4021 tatagcgttt aaaaattcag aagggctgtg tttgaaaagg tgatcatatg gaaaaaaaag
4081 tagaaaaaga ggtaaaatgc ttagagaaaa aaccactagc agaactgaaa aaaatatgga
4141 agaaggtatt tgaggaagaa gcacctagac actcaaagaa atacctgata ccgaggttag
4201 catataggtt acaggagaaa gcgtatggag aaatatcaag aaaagggca aaaagactag
4261 aatatctagc agatcgacta gagaagggaa aaagaataag tagtgacaaa ctgccagtag
4321 agggaacaga gttaatatta gagagaggag aagaagtcca cgttgtaatg gtaacaaata
4381 caggtttgat ctaccgagaa gagttttata cgtcattatc agcggtagcc ggaaaaataa
4441 tgggaatgag ctacaacggt cctttattat ttgggatgcg tgataaaaag gtaagttgag
4501 ggggagaaaa tgctaaaaga ggtaaggtgt gcgatatata cgagaaaatc aaatgaagac
4561 gggctagagc agaagtttaa tagtcttgat gcacagcgag tagcatgtga aaaatatata
4621 aaaagccgag aagggtgggt agcattagcc aaaaggtatg atgatggagg ctattcagga
4681 agtaatttaa atagaccagc gataaaggaa ttatttgaag atgtaaaagc cggagaggta
4741 gactgcgtgg tagtatacac gttggacagg ttatcaaggg aaacgaagga ttgcatagaa
4801 gtaacgtcat tttttagaag gcaccggata agttttgtag cagtgacgca aatatttgat
4861 aacaacacac caatgggaaa atttgtgcaa acagtgttat caggagcagc acaactagaa
4921 agagaaatga tagtagagag agtaaaaaat aaaatagcaa catcgaaaga ggaaggatta
4981 tggatgggag gaaatccacc actcggctat gatgtaaaag ataaggaatt aataataaat
5041 gagaaagaag caaaggtaat aaagcacata tttgaaaggt atatggagct gaaatcaatg
5101 gcagaattgg caagagagtt aaacagagaa ggatatcgaa cgaagaagtt tgaagcaaaa
5161 tcgggaaaag tatatggagg agagatattt aagaaagcaa cggtaaggag aataataaca
5221 aaccctatat atatgggaaa aacaagacat tacgagaaac aatataaagg aaagcatgaa
5281 gcgataatag aagaagaaaa atggaagaag gcacaggaat taataaagga tcagccgtat
5341 agaaaagcaa aatatgaaga ggcgctacta aagggaataa taaagtgcaa gagctgtgat
5401 gtaaacatga cgctgacata ctcaaaaaaa gaaaataaaa ggtaccgata ttacgtgtgc
5461 aataatcatt tgaggggaaa ggattgtgaa tcaggaaacc gaaatgtaat agccggagaa
5521 gtagagaaag aagtgatgaa gagagctgaa cgcttatatg aaaattggga aagaggggaa
5581 gaaaaatggg aaaatttaag ttttggaaaa cagaaagaag tggtgaagaa attagtaaag
5641 ggagtaatgg taaaagaaga tggaatagag gtgcattctg agtcagagaa gacatttata
5701 gcaatgaatt taaagaagaa aggaaacaaa tgcacagtag tagaaccaga agggaaaaca
5761 aacaatgcgc tactgaaagc agtggtaaga gcccatctgt ggaaacgtca actagaggaa
5821 agaaaatatg caagtctgaa ggagctgagc gttaaaatta atataggtac aaggcgcata
5881 caacaaattt taaggttaaa ttatttagct ccaaagatca aagaagacat agtaaatggg
5941 agacaaccaa gtaatttgcg gttagttgat ttaagggaaa taccgacgct gtggagtgag
6001 cagatggaaa aattttatgg attagtgtcg taacgtttat aaaagcaaca agaggaaaaa
6061 aactatgaaa acaccgaaaa aaatatggaa aagtattgct aaaaaattca aaaatagcac
6121 tgccgcaaaa gcaaacgttt ctagcagtgc agacaaagat attttagaag aaggcaaaaa
6181 actttcagaa gaagttctta ataaggaagt aaccataaag cagcaggttg taacaggcaa
6241 aactgaaagt gtgagtctga tccaggatac tgcttctata atcggtaata tcgagacacc
6301 ggctgacgca tatgaaaata gtaataaaag tagtcttgaa gaatttatag aacataaacc
6361 atgcgaaaat agtaatgaaa gtaatacaat agctatagag cacatcgcta cttcaataga
6421 aaagcttgat aatactggaa tatgcgaaaa aaatgaaact gctgtaacag aaactgacat
6481 acaagagcaa aatgcttcca acagcctttc ttctgctgca tgttttgata atgggtctag
6541 caaagaaaat actgcagaca aaaaagaaaa accatcaatg caacaaaggc aagcaatcct
6601 ggctggtgtt gttggtgcgg cattactagt cagtagtatc atagcttatt tattgaaaat
6661 gtacgccata gcggtgattg ggggaatagt tggattagct tgtgtgagtt atgcgttgta
6721 tagtgtgcta aagccgaata caaagcttga gaaagtagag gaacaatcga ttactgctca
6781 tcctcctttg aatccaacat aaaaggccat gtccaaaaaa ccataaatgt caagaaagtt
6841 tttaaataag ataacttatt tagaagaatc tctaaattat taacttaata cctaaacaat
6901 tattctattc ctgctatcct ttctcaactt gggctattta aaacttccac agccgcagaa
6961 tcaataccat ttttaacttc aacattacta agcgctgtat ttgccttata cgaacagtaa
7021 catcctactg caagaaaaat cgatgcagct acagcaaatg taatacatat cgctaaataa
7081 ggtattgtta aacttgcacc aacagcaaat gccccagata atacaaaaga aacagaggca
7141 taggttactt gctttctact ctgagcgctt actgctaaga gtttttttgtt tttagcattc
7201 agattttgag tttgaagttg gatattttct gatgaaagtt gagttacttc actagctttt
7261 ttaaccaagt cttgctttgt actattcagc tcttgctctt tttctgtgag ttgatttttt
7321 atgccttcaa gttcttctat agtattttta agtcgagtta cttcttgatt tgcactacct
7381 agtgcttcct tctgctttgt aagttgggtg tttagtgtcc ctatagtagc gttaagtttg
7441 tttgtttcac tagtaagttg agttacttta acatcttcgt cagccaagct tttctgtacg
7501 atacccaacg ctttctcttt ctctgcgagt tgaacttttg ttctctcaag ttcctcaatc
7561 tttgttataa gcttagttac ttcgctagtt ttgctatcca agtcttgcct tgtactatcc

```
     7621 agctctccct tctgctcttt gagctgagct tctaacttct ctatagtacc attaagcttg
     7681 gttacttttc caattttgct ttccagctct tcttctttct tcttgagtgc atcttgcagt
     7741 tttgttttct cttgctctaa ttgagcttgt gcattcttta gctgcacatc tttctctgtg
     7801 agttcatttt gtagttctgc ttcctttgtt tctaattgaa cttgtgcacc cttcagcttt
     7861 tcctctttct ctgcaagctg agttttcgtg ctttcaagtg cctgagtttt ttcattcaat
     7921 tctttttgta gcttttcaag ctgatttctc atattctcag gttcctcttg tcctaatgag
     7981 cttccgacct gctgtgtgtt tgtaggcatt ttaccctcac tgaaatcatt gcttacaggt
     8041 tcctccattg aagtccctac attatttatt gtattacttt cttttgttga tgaaagaaca
     8101 tttttttcta tctgttggct gccagtactt tcaggttctg gacttttttg atcgcctgat
     8161 tgaccgttac tactttcctc aactgttgct tgttgtgtat tgacttcaga ctcatcttca
     8221 ctagcacctt tcagtatact ttcttctgca tcatagaatt catcatcact ctcttcaaaa
     8281 ttttgttcac tactaagtgg aacagactct tttttttgtg attgactaaa tagatctata
     8341 agctgagtat taccttgctc a
//
LOCUS       JAQZAU010000057          23382 bp    dna     linear   ENV 17-MAR-2023
DEFINITION  MAG: Wolbachia pipientis isolate wEsol contig_35600, whole genome
            shotgun sequence.
ACCESSION   JAQZAU010000057 JAQZAU010000000
VERSION     JAQZAU010000057.1
DBLINK      BioProject: PRJNA929258
            BioSample: SAMN33142973
KEYWORDS    WGS; ENV; Metagenome Assembled Genome; MAG.
SOURCE      Wolbachia pipientis (insect metagenome)
  ORGANISM  Wolbachia pipientis
            Bacteria; Pseudomonadota; Alphaproteobacteria; Rickettsiales;
            Anaplasmataceae; Wolbachieae; Wolbachia.
REFERENCE   1  (bases 1 to 23382)
  AUTHORS   Fiutek,N., Couger,B.M., Roy,S., de la Torre,J.R. and Connor,E.F.
  TITLE     Genomic assessment of the contribution of the Wolbachia
            endosymbiont of Eurosta solidaginis to gall-induction
  JOURNAL   International Journal Molecular Sciences
REFERENCE   2  (bases 1 to 23382)
  AUTHORS   Fiutek,N., Couger,B.M., Roy,S., de la Torre,J.R. and Connor,E.F.
  TITLE     Direct Submission
  JOURNAL   Submitted (10-FEB-2023) Biology, San Francisco State University,
            1600 Holloway Ave, San Francisco, CA 94132, USA
COMMENT     ##Genome-Assembly-Data-START##
            Assembly Date          :: JUN-2019
            Assembly Method        :: FLYE v. 06-2019
            Genome Representation  :: Full
            Expected Final Version :: Yes
            Genome Coverage        :: 100.0x
            Sequencing Technology  :: Illumina HiSeq; PacBio Sequel
            ##Genome-Assembly-Data-END##
FEATURES             Location/Qualifiers
     source          1..23382
                     /organism="Wolbachia pipientis"
                     /mol_type="genomic DNA"
                     /submitter_seqid="contig_35600"
                     /isolate="wEsol"
                     /isolation_source="galls on Solidago altissima"
                     /host="Eurosta solidaginis MN-2018-EFC"
                     /db_xref="taxon:955"
                     /environmental_sample
                     /country="USA: Cowling Arboretum, Carleton College,
                     Northfield, MN"
                     /lat_lon="44.48 N 93.13 W"
                     /altitude="280 m"
                     /collection_date="2018-11"
                     /metagenome_source="insect metagenome"
```

```
                     /note="metagenomic"
     gene            <1..1003
                     /locus_tag="wEsol_00602"
     CDS             <1..1003
                     /locus_tag="wEsol_00602"
                     /codon_start=1
                     /transl_table=11
                     /fullproduct="qcoverage 100 , hcoverage 96.8023255813954
                     , similarity 99.7 , identity 99.7 , evalue 6.22e-199 ,
                     alnlength 333 , Bacteria , Proteobacteria ,
                     WP_015589478.1 MULTISPECIES: hypothetical protein
                     [unclassified Wolbachia]"
                     /translation="DYPELRKAISDIIESIAQSSQEGKKELFTTLSEQVKGMNLKDLG
                     LAINDQGLQKEVNKELFIKLIEEALKDLTSENVNSKIIEEALKSVNQQAISNVINNNK
                     GPLDNLVNYFKSASANEKIGIASAGMVLGVAFSPLIVLATLIALVAIGARYVGIGIGK
                     AGEKIKEGAIDAGKAVKSLSKNLGDKLPEVGPKKDNFSKLYNSLAQEITGYNMSEEGE
                     KIKNAQSKAKVIGMLQNEGQRSAIQELVKNGTIQNFSEDDMKKLREKGTSTYESDKEA
                     LEELMEKFQSKIMAIGEGKIEEVEKIVDEKVAAMKPSSHLGISSAARVSEGGITGPS"
                     /product="hypothetical protein"
                     /besthit="qcoverage 100 , hcoverage 96.8023255813954 ,
                     similarity 99.7 , identity 99.7 , evalue 6.22e-199 ,
                     alnlength 333 , Bacteria , Proteobacteria ,
                     WP_015589478.1 MULTISPECIES: hypothetical protein
                     [unclassified Wolbachia]"
     gene            complement(1048..1599)
                     /locus_tag="wEsol_00603"
     CDS             complement(1048..1599)
                     /locus_tag="wEsol_00603"
                     /codon_start=1
                     /transl_table=11
                     /fullproduct="qcoverage 100 , hcoverage 100 , similarity
                     100 , identity 100 , evalue 1.06e-131 , alnlength 183 ,
                     Bacteria , Proteobacteria , WP_015589477.1 MULTISPECIES:
                     5-formyltetrahydrofolate cyclo-ligase [unclassified
                     Wolbachia]"
                     /pfam="5-FTHF_cyc-lig"
                     /tigr="TIGR02727 MTHFS_bact 182 5-formyltetrahydrofolate
                     cyclo-ligase"
                     /product="
                     5-formyltetrahydrofolate cyclo-ligase"
                     /translation="MFKDIKQHKKEIREQYRAIRKDIDESYSSYAANSLINLFNQNLS
                     YVKGKTIAAYIPMDGEINVVPLMHSLLDLDYKVAIPNKNKLLKFEEWNKTNEDVIPDT
                     IITPVIAFDDHFNRLGFGGGWYDTMIEKLRPLGKIFIGVAYEKQYCKDLPVEKHDQKL
                     DIIITEMCVRCGSGLLKKVDKKE"
                     /besthit="qcoverage 100 , hcoverage 100 , similarity 100
                     , identity 100 , evalue 1.06e-131 , alnlength 183 ,
                     Bacteria , Proteobacteria , WP_015589477.1 MULTISPECIES:
                     5-formyltetrahydrofolate cyclo-ligase [unclassified
                     Wolbachia]"
     gene            complement(1583..2203)
                     /locus_tag="wEsol_00604"
     CDS             complement(1583..2203)
                     /locus_tag="wEsol_00604"
                     /codon_start=1
                     /transl_table=11
                     /pfam="NAD_binding_1"
                     /fullproduct="qcoverage 100 , hcoverage 100 , similarity
                     100 , identity 100 , evalue 1.36e-143 , alnlength 206 ,
                     Bacteria , Proteobacteria , WP_015589476.1 MULTISPECIES:
                     hypothetical protein [unclassified Wolbachia]"
```

```
                     /translation="MKNIMLIGGGVGNAVLFSIGKACLENNNKVLYFAGYKKLSDVFK
                     RALIERASNAVVWACEEGLIETSRDQDKSFHGNIVDAIVSYQQGRLGINLNVIDKIIT
                     IGSDKMMKAVNEARKTILKPYLKSSHIAISSVNSPMQCMMKEICAQCVQQHINKETGE
                     RSFVYSCSNQDQDMEFIDFDFLSERLKQNSLQEKLTAKWIDHVQRY"
                     /product="hypothetical protein"
                     /besthit="qcoverage 100 , hcoverage 100 , similarity 100
                     , identity 100 , evalue 1.36e-143 , alnlength 206 ,
                     Bacteria , Proteobacteria , WP_015589476.1 MULTISPECIES:
                     hypothetical protein [unclassified Wolbachia]"
     gene            complement(2307..3548)
                     /locus_tag="wEsol_00605"
     CDS             complement(2307..3548)
                     /locus_tag="wEsol_00605"
                     /codon_start=1
                     /transl_table=11
                     /pfam="SurA_N_3 SurA_N SurA_N_2 YbbR"
                     /fullproduct="qcoverage 100 , hcoverage 100 , similarity
                     99.3 , identity 97.6 , evalue 1.80e-270 , alnlength 413 ,
                     Eukaryota , Arthropoda , GFQ68659.1 parvulin-like
                     peptidyl-prolyl isomerase, SurA,peptidyl-prolyl cis-trans
                     isomerase SurA [Trichonephila clavata]"
                     /translation="MVFISIVTEITIKKLASIDSVFIIELVINLTYMHKILILLLIVL
                     PLRLLAIEIEIVADVNGEPISNLDIEKRINFINSLFGTQSVDQKGAKPQILRELIDEI
                     IIINEAQRLNIKLSNEELDNAIMLFLTQSFKLKANEVDQYIEKHNIDLGILRKQIKCQ
                     LLWSKIIEVRIVPFINISDKEVNDVKRQTEKPDYLITFQEFIIPDQKDKDVYGIAEDL
                     VKKLRNSNNPESPIKMRKATVNLSQLKGKLKNVLEGLETSDIAGPFSFSEGYSVIKVI
                     DKVQLNHALLESTLKLKQIVVEGSESLLDNLKEQKVNCLNFDKLADNFKLPNAKEFEI
                     KMRDLNPDLQILFSKTSVNEIVELRENGTAKLMMLCDIKSNVAGIEAIKQKMYQQKIM
                     IQSNLLLDDMRKNAAISYRYS"
                     /product="parvulin-like peptidyl-prolyl
                     isomerase, SurA,peptidyl-prolyl cis-trans isomerase SurA"
                     /besthit="qcoverage 100 , hcoverage 100 , similarity 99.3
                     , identity 97.6 , evalue 1.80e-270 , alnlength 413 ,
                     Eukaryota , Arthropoda , GFQ68659.1 parvulin-like
                     peptidyl-prolyl isomerase, SurA,peptidyl-prolyl cis-trans
                     isomerase SurA [Trichonephila clavata]"
     gene            complement(4109..4624)
                     /locus_tag="wEsol_00606"
     CDS             complement(4109..4624)
                     /locus_tag="wEsol_00606"
                     /codon_start=1
                     /transl_table=11
                     /fullproduct="qcoverage 100 , hcoverage 100 , similarity
                     100 , identity 100 , evalue 2.64e-117 , alnlength 171 ,
                     Bacteria , Proteobacteria , WP_012673395.1 MULTISPECIES:
                     inorganic diphosphatase [Wolbachia]"
                     /cog="Ppa COG0221 171 Inorganic pyrophosphatase"
                     /pfam="Pyrophosphatase"
                     /product="inorganic
                     diphosphatase"
                     /translation="MDLSKITAGPNAVNVVIEISANAEPVKYEFNKEFGLLQVDRFLS
                     TSMTYPCNYGFIPNTCAGDGDPVDVLVLTQFPLASSVLISVRPIGALLTKDEKGEDEK
                     ILAVPVSSVDSYYDNIKDYSDLSKSLLDKITHFFSHYKDLEKGKTVAVGEWVGVEEAK
                     QIVASSIKSGN"
                     /besthit="qcoverage 100 , hcoverage 100 , similarity 100
                     , identity 100 , evalue 2.64e-117 , alnlength 171 ,
                     Bacteria , Proteobacteria , WP_012673395.1 MULTISPECIES:
                     inorganic diphosphatase [Wolbachia]"
     gene            complement(4687..5910)
                     /locus_tag="wEsol_00607"
```

```
CDS             complement(4687..5910)
                /locus_tag="wEsol_00607"
                /codon_start=1
                /transl_table=11
                /fullproduct="qcoverage 100 , hcoverage 100 , similarity
                100 , identity 100 , evalue 2.48e-290 , alnlength 407 ,
                Bacteria , Proteobacteria , WP_015589474.1 MULTISPECIES:
                hypothetical protein [unclassified Wolbachia]"
                /translation="MIKEIEELIKIVGKGNLLETLFIFLGSFQHADLGRFMDHRNHFG
                LSEFIRKKEEPLDKYFNLKFSLQGLFLIYAQAYQILSENNNLNTKDVIDKIYEVIKGS
                ELNEYINEVVDGRKLDLLNILANPKREPIFKTEEDFENKVLIGLRKLLSEGNTLNKYD
                REFALNKCVIGCYYSIHNIKVDKDIALLFSIGSYYKELSPSNKTVRREVLEICENQLI
                ENYIVHLASKLHMIGLSSRDKYTIDHENVDRQEKALKMDGLTCPINTKNTLEQLLKNQ
                NSDEFEQLVMSPENIYYSVLPEENEYRPNLITSKKKEKDYKNTVCKLILGMFILSVGL
                SVADYFLMEQKLLNSIKNVLPQDNLTNLIIGAVLTIVIVYALFQLLKPPQEPPLSTVN
                KMMNENLGPLHAKQA"
                /product="hypothetical protein"
                /besthit="qcoverage 100 , hcoverage 100 , similarity 100
                , identity 100 , evalue 2.48e-290 , alnlength 407 ,
                Bacteria , Proteobacteria , WP_015589474.1 MULTISPECIES:
                hypothetical protein [unclassified Wolbachia]"
gene            complement(6709..6807)
                /locus_tag="wEsol_00608"
CDS             complement(6709..6807)
                /locus_tag="wEsol_00608"
                /codon_start=1
                /transl_table=11
                /fullproduct="qcoverage 100 , hcoverage 103.225806451613
                , similarity 90.6 , identity 90.6 , evalue 1.51e-10 ,
                alnlength 32 , Bacteria , Proteobacteria , AAS14905.1
                hypothetical protein WD_1260 [Wolbachia endosymbiont of
                Drosophila melanogaster]"
                /translation="MLLTKSFNAECKHTIDFLHNHITLGIETKKLA"
                /product="hypothetical protein"
                /besthit="qcoverage 100 , hcoverage 103.225806451613 ,
                similarity 90.6 , identity 90.6 , evalue 1.51e-10 ,
                alnlength 32 , Bacteria , Proteobacteria , AAS14905.1
                hypothetical protein WD_1260 [Wolbachia endosymbiont of
                Drosophila melanogaster]"
gene            complement(6798..7901)
                /locus_tag="wEsol_00609"
CDS             complement(6798..7901)
                /locus_tag="wEsol_00609"
                /codon_start=1
                /transl_table=11
                /fullproduct="qcoverage 100 , hcoverage 100 , similarity
                100 , identity 100 , evalue 1.32e-267 , alnlength 367 ,
                Bacteria , Proteobacteria , WP_015589473.1 MULTISPECIES:
                tRNA 2-thiouridine(34) synthase MnmA [unclassified
                Wolbachia]"
                /cog="TrmU COG0482 356 Predicted
                tRNA(5-methylaminomethyl-2-thiouridylate)
                methyltransferase, contains the PP-loop ATPase domain"
                /pfam="Asn_synthase Pfam-B_78 NAD_synthase ThiI
                tRNA_Me_trans ExsB Pfam-B_18759"
                /tigr="TIGR00420 trmU 351 tRNA
                (5-methylaminomethyl-2-thiouridylate)-methyltransferase"
                /product="tRNA
                2-thiouridine(34) synthase MnmA"
                /translation="MLKEFEIEPLLKDKAPHQTKAVVAMSGGVDSSVAAALLYNFGYQ
```

VIGVTLQLYGTDGARKGACCAGQDIYDAKRVAESVGFPHYILNYEEIFKKEVIEDFAS
TYMRGETPIPCVRCNQTVKFRDLLQVTKNLGADVLVTGHYVRRLEKNGEVKLCRSIDK
SKDQSYFLFATTQEQLKLLRFPLGGFYKSDIRKLAKYFGLQISEKPDSQDICFVSESY
SKTIAKLAPQSVQKGKIVDVNGKVLGEHSGIVNFTVGQRKGLGIAHNEPLYVIKINTE
NNEVIVGPINVLMQKKILIKELNWLEQPKEGMEVTVKLRSSHAGSLATIHSTDEKNKA
CVILNDDYFGISPGQACVAYKDEQVIGGGWICS"
/besthit="qcoverage 100 , hcoverage 100 , similarity 100
, identity 100 , evalue 1.32e-267 , alnlength 367 ,
Bacteria , Proteobacteria , WP_015589473.1 MULTISPECIES:
tRNA 2-thiouridine(34) synthase MnmA [unclassified
Wolbachia]"
gene complement(8104..9180)
/locus_tag="wEsol_00610"
CDS complement(8104..9180)
/locus_tag="wEsol_00610"
/codon_start=1
/transl_table=11
/fullproduct="qcoverage 100 , hcoverage 100 , similarity
100 , identity 100 , evalue 3.01e-248 , alnlength 358 ,
Bacteria , Proteobacteria , WP_141456703.1 MULTISPECIES:
rod shape-determining protein [unclassified Wolbachia]"
/cog="MreB COG1077 342 Actin-like ATPase involved in cell
morphogenesis"
/pfam="MreB_Mbl Actin HSP70 Pfam-B_12247 Pfam-B_178 DDR
EutA Competence_A FtsA"
/tigr="TIGR00904 mreB 334 cell shape determining protein,
MreB/Mrl family"
/product="rod
shape-determining protein"
/translation="MNFVRKLTEKFCSLFTFKGLFASDIAIDLGTANTLVYQKNQGIV
LDEPSVVARIKEKGSYVPYAFGKKAKMMLGKTPGEIEAIRPLKDGVIADFKSAEEMLK
YFIRSANTKFTVNKPNIIICVPSGSTPVERRAIQDAAESAGANEVFLIEEPMAAAIGA
GLPVTEPEGSMIVDIGGGTTEVAIISLGGIVYSRSARVGGDIMDEAIKSYIRENHKLL
IGETTAEKIKKSIGSANLPGENNKEGMIIKGRDLVSGMPKEMLLSEYQVAESLIEPVH
QIISAIRTALESTPPELSSDIVDKGIILSGGGGLLRNLGKVISETTKLPVRVADDPLC
CVALGSGKVLENMDYFGHVLFKQD"
/besthit="qcoverage 100 , hcoverage 100 , similarity 100
, identity 100 , evalue 3.01e-248 , alnlength 358 ,
Bacteria , Proteobacteria , WP_141456703.1 MULTISPECIES:
rod shape-determining protein [unclassified Wolbachia]"
gene complement(9204..10625)
/locus_tag="wEsol_00611"
CDS complement(9204..10625)
/locus_tag="wEsol_00611"
/codon_start=1
/transl_table=11
/fullproduct="qcoverage 100 , hcoverage 100 , similarity
100 , identity 100 , evalue 0.0 , alnlength 473 ,
Bacteria , Proteobacteria , WP_015589471.1 MULTISPECIES:
Thermostable carboxypeptidase 1 [unclassified Wolbachia]"
/cog="COG2317 COG2317 497 Zn-dependent carboxypeptidase"
/pfam="Peptidase_M32"
/product="Thermostable
carboxypeptidase 1"
/translation="MKSYKFLEEVLYRVKNIENTLKVLNQSQLNAEDKVEQMSLLEEI
RHEIISHDVIKESLADALDNKKSADAWQLKLIERIHKSNSAVPIDLVKSLSKAKVECQ
SLWRLNNSEASNLEKLKERFTDLITLIREVASIKSQQLKCSKYDSLLADYDSDITEKN
IREVFPKVGKFFSGNVDKIIEKQKKDKVTNIQKVATQKQIELGSSCLQQMGVALHEVR
TSYYYPIDYDESDFCYGLFSLLRHSGYAIYQKCLAQNSISSPITRHVMYETQGLFMER
MIGTSREFIEFIQPHIKEKFAIKGKTNEKVSSVENLYLIFNEINLSSFLKNADEFSLL

```
                     AHIMLRTRLEQDIINGTLEVKDLHDAWLEGMKHYKIPVKAENELNTYFQDEYWASGVM
                     GYFPIKIIALIAAVQIFSCVKKNHYESLSAIIKGDFSLLISWLSQNIYSAKCGLELLK
                     KVTGKGLDVECCISYLSGKYNLS"
                     /besthit="qcoverage 100 , hcoverage 100 , similarity 100
                     , identity 100 , evalue 0.0 , alnlength 473 , Bacteria ,
                     Proteobacteria , WP_015589471.1 MULTISPECIES:
                     Thermostable carboxypeptidase 1 [unclassified Wolbachia]"
     gene            10901..11380
                     /locus_tag="wEsol_00612"
     CDS             10901..11380
                     /locus_tag="wEsol_00612"
                     /codon_start=1
                     /transl_table=11
                     /fullproduct="qcoverage 100 , hcoverage 100 , similarity
                     100 , identity 100 , evalue 6.12e-108 , alnlength 159 ,
                     Bacteria , Proteobacteria , WP_010081889.1 MULTISPECIES:
                     OmpA family protein [Wolbachia]"
                     /cog="OmpA COG2885 190 Outer membrane protein and related
                     peptidoglycan-associated (lipo)proteins"
                     /pfam="OmpA"
                     /product="OmpA family
                     protein"
                     /translation="MWSRLVIMCCFCLLLTGVSSCPKKGANTTNKINSVVKQIGDKRV
                     FFGYDESSIAEVSADALLDVMEVLQDNPDAKVTLTGHTDNRGSHEYNLALGARRADAA
                     KKFMVSCTPYIENRIKTASKGETEPLVNVKDDSRNSKYEKEHAKNRRVEFSFSGIKK"
                     /besthit="qcoverage 100 , hcoverage 100 , similarity 100
                     , identity 100 , evalue 6.12e-108 , alnlength 159 ,
                     Bacteria , Proteobacteria , WP_010081889.1 MULTISPECIES:
                     OmpA family protein [Wolbachia]"
     gene            11558..12853
                     /locus_tag="wEsol_00613"
     CDS             11558..12853
                     /locus_tag="wEsol_00613"
                     /codon_start=1
                     /transl_table=11
                     /pfam="Asn_synthase ATP_bind_3 PAPS_reduct"
                     /fullproduct="qcoverage 100 , hcoverage 100 , similarity
                     100 , identity 100 , evalue 2.26e-314 , alnlength 431 ,
                     Bacteria , Proteobacteria , WP_015589470.1 MULTISPECIES:
                     tRNA lysidine(34) synthetase TilS [unclassified
                     Wolbachia]"
                     /translation="MELELLFQNVVNSFAFYNNQVAVAVSGGVDSIILLHLMNNWAKK
                     NKLPLPIALTVNHGLRSESQKEADFVVSYAKELGAKESFILNWEKQNIKGNIQLQARK
                     ARYKLLAEWCKNNNVKYLLVAHHKDDQAETFLLRLERGSGVDGLSSMDYKSFLNGIYI
                     FRPLLNFSRSEIEKYAKLHRLKWIEDRSNYDLKYRRTLYRNLLKASDNQEILTERICL
                     TALHMKRAAKALMHYTRLAFNDCVNVHDLGYIEIKLSEFYQLPEEIALRLLLYSIMAI
                     ASKHYKPRYNSLIVIFNKILQKDSNVNCTLSGCKIRKHGENILIIRESSKIQEITLNL
                     PLNEPTQWDNRFSCTILGNQECSVIIAPLKKTQKVPEFLKDYNCCPEVFYSLPVVLKD
                     GKVLAYPDVNYNGKNTNDDKVQCIINSTIKQNLVSLISI"
                     /product="tRNA lysidine(34)
                     synthetase TilS"
                     /besthit="qcoverage 100 , hcoverage 100 , similarity 100
                     , identity 100 , evalue 2.26e-314 , alnlength 431 ,
                     Bacteria , Proteobacteria , WP_015589470.1 MULTISPECIES:
                     tRNA lysidine(34) synthetase TilS [unclassified
                     Wolbachia]"
     gene            12866..14704
                     /locus_tag="wEsol_00614"
     CDS             12866..14704
                     /locus_tag="wEsol_00614"
```

```
                     /codon_start=1
                     /transl_table=11
                     /fullproduct="qcoverage 100 , hcoverage 100 , similarity
                     100 , identity 100 , evalue 0.0 , alnlength 612 ,
                     Bacteria;Eukaryota , Proteobacteria;Arthropoda ,
                     WP_012673393.1 MULTISPECIES: ATP-dependent zinc
                     metalloprotease FtsH [Wolbachia]"
                     /cog="HflB COG0465 596 ATP-dependent Zn proteases"
                     /pfam="FtsH_ext RuvB_N TIP49 IstB_IS21 AAA_5 AAA_22 AAA
                     AAA_17 Pfam-B_15939 Peptidase_M41"
                     /tigr="TIGR01241 FtsH_fam 496 ATP-dependent
                     metallopeptidase HflB"
                     /product="ATP-dependent zinc
                     metalloprotease FtsH"
                     /translation="MKKFLEGLLIWLVIIVLISVAYIQFSGNVGKSKTIIPFSEFLTR
                     LEDNDVENIVIKNQSIEGKFRDGSSFNSSGVIYSDLIKNLHDRKVRFSFSTGDSAMNV
                     IGGLLISWVPTFIFIGFLLFFLKQTQAGGNRTISFGKSRARLMTSGKKVTFDDVAGID
                     EAKEELVEIVDFLKQRQKFQILGGKIPKGCLLIGSPGTGKTLLARAIAGEANVPFFSI
                     SGSDFVEMFVGVGASRVRDMFDQGKKNAPCIIFIDEIDAVGRHRGIGLGGGNDEREQT
                     LNQLLVEMDGFESNEGVIIIAATNRPDVLDPALLRPGRFDRQITISLPDINGREKILN
                     THIKKISIAPDVNVKTVARGTPGFSGADLANLVNESALIAARRNKKIVTMDDFEYARD
                     KVMMGVERRSLIMTEEEKRLTAYHEAGHAMIAVNMPASDPIHKATIIPRGRALGLVMR
                     LPETDRVSLTREKMLADITVAMGGRVAEELIFGYDKVTSGASSDIKQASDLSRAMVTK
                     WGMSDKIGPIYHNREQTIHGSEIISEDTLKLIDEEVKKVVSSCYEKAKDILTKHKKGL
                     DLIAENLLEFETLTGDEIKDILNGKKIVREENEKNEKIKKSSLYES"
                     /besthit="qcoverage 100 , hcoverage 100 , similarity 100
                     , identity 100 , evalue 0.0 , alnlength 612 ,
                     Bacteria;Eukaryota , Proteobacteria;Arthropoda ,
                     WP_012673393.1 MULTISPECIES: ATP-dependent zinc
                     metalloprotease FtsH [Wolbachia]"
     gene            14859..15527
                     /locus_tag="wEsol_00615"
     CDS             14859..15527
                     /locus_tag="wEsol_00615"
                     /codon_start=1
                     /transl_table=11
                     /fullproduct="qcoverage 100 , hcoverage 100 , similarity
                     100 , identity 100 , evalue 1.56e-150 , alnlength 222 ,
                     Bacteria , Proteobacteria , WP_007548786.1 MULTISPECIES:
                     hypothetical protein [Wolbachia]"
                     /translation="MIDIASIGVSTVIDQNYRRKAQLCLSGLFVTVSIATVTWFIFPA
                     SQNVLAPFLLPAMFLLFTLSMGYYMFIFSCEEKSLVKNVLGMVEPAESVKRVHCQENI
                     KSLLAEVRSTTKDRFRNVLVNLCFEDRSKAHKLALYVKEIDNIVSSIKNKDLLDHFDQ
                     FFTITEVSNKKNRSELMNLARAYKMISEEDPDKIQDFLATQTKSYSYEIMLPEIDNFL
                     SQAR"
                     /product="hypothetical protein"
                     /besthit="qcoverage 100 , hcoverage 100 , similarity 100
                     , identity 100 , evalue 1.56e-150 , alnlength 222 ,
                     Bacteria , Proteobacteria , WP_007548786.1 MULTISPECIES:
                     hypothetical protein [Wolbachia]"
     gene            complement(15520..16158)
                     /locus_tag="wEsol_00616"
     CDS             complement(15520..16158)
                     /locus_tag="wEsol_00616"
                     /codon_start=1
                     /transl_table=11
                     /fullproduct="qcoverage 100 , hcoverage 100 , similarity
                     100 , identity 100 , evalue 3.87e-146 , alnlength 212 ,
                     Bacteria , Proteobacteria , ACN95910.1 thymidylate kinase
                     [Wolbachia sp. wRi]"
```

/cog="Tmk COG0125 208 Thymidylate kinase"
/pfam="AAA_18 AAA_28 AAA_17 Pfam-B_2168 Thymidylate_kin"
/tigr="TIGR00041 DTMP_kinase 196 thymidylate kinase"
/product="thymidylate kinase"
/translation="MYTVCIFFFSIIIIKLMFITFEGIDGSGKTTQSKLLANHFKQIH
GENNVVLTREPGGTNFAEKIRGVLLTNNIDPISELLLLISMRREHMKKLILPALAEGK
IVICDRFIDSTIAYQGYGLSVDLGLIRDLHKLVEIKCPDITFILDIDVKVGLNRAKDK
NKYEEMDVNFYNKVRKGFQEIAIKEPVRCSVITGIETKNDNHVHNEIIDKIT"
/besthit="qcoverage 100 , hcoverage 100 , similarity 100
, identity 100 , evalue 3.87e-146 , alnlength 212 ,
Bacteria , Proteobacteria , ACN95910.1 thymidylate kinase
[Wolbachia sp. wRi]"
gene 16219..16791
/locus_tag="wEsol_00617"
CDS 16219..16791
/locus_tag="wEsol_00617"
/codon_start=1
/transl_table=11
/pfam="AhpC-TSA Redoxin Thioredoxin_8"
/fullproduct="qcoverage 100 , hcoverage 100 , similarity
100 , identity 100 , evalue 1.18e-136 , alnlength 190 ,
Bacteria , Proteobacteria , WP_015589468.1 MULTISPECIES:
thioredoxin family protein [unclassified Wolbachia]"
/translation="MVALNTPKVDFSFTAKDFNLLGVDNKYYTLSDCCGKNGLIVMFI
CNHCPYVQSIISNLVSDVDQLKKDYQVNTIAIMPNDVNEYPEDSFENMINFAKENKFT
FPYLIDSTQKIAKEYGAVCTPDFFGFNSNLNLCYRGRFNNAKKEKVQSYEVGSSDLFQ
AMKFIAETGNSPIDQKSSIGCSIKWSNSTG"
/product="thioredoxin family
protein"
/besthit="qcoverage 100 , hcoverage 100 , similarity 100
, identity 100 , evalue 1.18e-136 , alnlength 190 ,
Bacteria , Proteobacteria , WP_015589468.1 MULTISPECIES:
thioredoxin family protein [unclassified Wolbachia]"
gene 16797..18515
/locus_tag="wEsol_00618"
CDS 16797..18515
/locus_tag="wEsol_00618"
/codon_start=1
/transl_table=11
/pfam="Na_H_Exchanger Asp-Al_Ex TrkA_N"
/fullproduct="qcoverage 100 , hcoverage 100 , similarity
100 , identity 100 , evalue 0.0 , alnlength 572 ,
Bacteria , Proteobacteria , WP_141456699.1 MULTISPECIES:
monovalent cation:proton antiporter-2 (CPA2) family
protein [unclassified Wolbachia]"
/translation="MHSGSQHLFDIIILLSAAVFIVIAFWKMNISPVLGYFVAGAVIG
SHGFNLIHSAEAMDNLAEFGVVFLLFIIGLELTFERLIAMRIHVFGFGSLQVIVTMVA
IWCIALAFGVNTNIATVIGGGLALSSTAIVLQVLQEKGSQASQVGRLSIAVLLMQDFA
VVPLIVLVPLLTGNSEHSLISSLAGSLVQAAIALVLIFITGRLLLRPLFSVIAKMESN
EIFISTTLLIVLGAAFITEQFHLSLALGAFVAGLLVAETEYRHSVEHAVLPFKDLFLG
LFFMTVGMSINTELLLNKLPLITLLSIILIVLKTSIIYILCRFFGFKSAPAIQAGLLL
SQGGEFAFILFRLADELNVLPSEIAQVLMMVTTVTMAFTPLLSGLGDWIANSFSTEKT
ILDDEAVETDTQDLYNHVIVAGFGRVGYMVTKMLTAEHLSYVVVDIQSKIVKEGKSDS
FPIYLGDVTRCEILKSVGIERAQALVISIKNEVTIKKVVSLVAANFPHVNIVIRLPDL
SNVEVYRDLGASKIIPETSEIGLQLGGAALSLSGISESGVMSLKSRFRKGNYSMLKDL
GSDKDE"
/product="monovalent
cation:proton antiporter-2 (CPA2) family protein"
/besthit="qcoverage 100 , hcoverage 100 , similarity 100
, identity 100 , evalue 0.0 , alnlength 572 , Bacteria ,

```
                     Proteobacteria , WP_141456699.1 MULTISPECIES: monovalent
                     cation:proton antiporter-2 (CPA2) family protein
                     [unclassified Wolbachia]"
     gene            complement(18546..19055)
                     /locus_tag="wEsol_00619"
     CDS             complement(18546..19055)
                     /locus_tag="wEsol_00619"
                     /codon_start=1
                     /transl_table=11
                     /fullproduct="qcoverage 100 , hcoverage 88.0208333333333
                     , similarity 100 , identity 100 , evalue 7.83e-121 ,
                     alnlength 169 , Bacteria , Proteobacteria ,
                     WP_206759902.1 MULTISPECIES: SURF1 family protein
                     [unclassified Wolbachia]"
                     /cog="COG3346 COG3346 252 Uncharacterized conserved
                     protein"
                     /pfam="SURF1"
                     /product="SURF1 family
                     protein"
                     /translation="MSLPVVHLLPNNDLEKFNYRHVKIDGILSDIELYVFAGQYGYHV
                     LSPMLLTTGNYMLVNKGIVKEKKEERAKIEKVAAGGVLYCNSSKSKNWFIKNDTTSNT
                     WFTLSTEEISNELGIKLEKCILWPNNFGSKVAIQPMKHLEYAITWFALSLIWLIMCVI
                     YYRQNLNKA"
                     /besthit="qcoverage 100 , hcoverage 88.0208333333333 ,
                     similarity 100 , identity 100 , evalue 7.83e-121 ,
                     alnlength 169 , Bacteria , Proteobacteria ,
                     WP_206759902.1 MULTISPECIES: SURF1 family protein
                     [unclassified Wolbachia]"
     gene            complement(19165..19641)
                     /locus_tag="wEsol_00620"
     CDS             complement(19165..19641)
                     /locus_tag="wEsol_00620"
                     /codon_start=1
                     /transl_table=11
                     /fullproduct="qcoverage 100 , hcoverage 100 , similarity
                     100 , identity 99.4 , evalue 2.49e-102 , alnlength 158 ,
                     Bacteria , Proteobacteria , WP_007549093.1 MULTISPECIES:
                     bacterioferritin [Wolbachia]"
                     /cog="Bfr COG2193 157 Bacterioferritin (cytochrome b1)"
                     /pfam="Ferritin"
                     /tigr="TIGR00754 bfr 157 bacterioferritin"
                     /product="bacterioferritin"
                     /translation="MNEEIVKHLNKLLTNELTSVRQYLLHFAILKNNGINRLAEKVKN
                     ELNEELEHANKLAERILLFKGVPNFQDTNEISKHDGEFTKDTIRKILEANLKLEGKGI
                     KDIKETISIAEKEKDFVSVMLLEEMLKNEEEHFHWIEKQIDLIELMGVENYLRTQI"
                     /besthit="qcoverage 100 , hcoverage 100 , similarity 100
                     , identity 99.4 , evalue 2.49e-102 , alnlength 158 ,
                     Bacteria , Proteobacteria , WP_007549093.1 MULTISPECIES:
                     bacterioferritin [Wolbachia]"
     gene            complement(19701..20057)
                     /locus_tag="wEsol_00621"
     CDS             complement(19701..20057)
                     /locus_tag="wEsol_00621"
                     /codon_start=1
                     /transl_table=11
                     /product="hypothetical protein"
                     /pfam="RDD MscS_TM Pfam-B_1921"
                     /translation="MCVNCSYFTYFISSKAQATPGQQLLNIYTINLDNSKIDLNLAFD
                     RSTSQFFLPLLNSVAVILTEFLQDQEVLVNVLSALKVMIVLLTLCWYLVACFSKKKQT
                     YHDMLFNTVVIKGTIK"
```

```
                     /cog="COG1714 COG1714 172 Predicted membrane
                     protein/domain"
                     /besthit="qcoverage 100 , hcoverage 73.2919254658385 ,
                     similarity 100 , identity 100 , evalue 2.99e-75 ,
                     alnlength 118 , Bacteria , Proteobacteria ,
                     WP_015589465.1 MULTISPECIES: RDD family protein
                     [unclassified Wolbachia]"
     gene            complement(20186..20824)
                     /locus_tag="wEsol_00622"
     CDS             complement(20186..20824)
                     /locus_tag="wEsol_00622"
                     /codon_start=1
                     /transl_table=11
                     /fullproduct="qcoverage 100 , hcoverage 100 , similarity
                     100 , identity 100 , evalue 7.26e-151 , alnlength 212 ,
                     Bacteria , Proteobacteria , WP_015589464.1 MULTISPECIES:
                     HAD-IA family hydrolase [unclassified Wolbachia]"
                     /cog="Gph COG0546 220 Predicted phosphatases"
                     /pfam="Pfam-B_6632 Hydrolase HAD_2 HAD Put_Phosphatase
                     PNK3P Hydrolase_like"
                     /tigr="TIGR01449 PGP_bact 213 phosphoglycolate
                     phosphatase, bacterial"
                     /product="HAD-IA family
                     hydrolase"
                     /translation="MKNSPLAVVFDWDNTLVDTQDNIFNAIKHTINSMGYSNKAADRN
                     SHESRKSYMVNLFGDQWKKANQIYQQYLDNALLQNIALNQGVEEMLQTLKSHNVYLAI
                     VSNKKNTNLREEVTYFKLDSYFERVVGSCDTAEDKPSATPLLFALEESTLPINRENVF
                     FVGDSITDVLCAQNANCLPIIYGQSISGYEDLLCFQHFDKLTDFVIKYLEDR"
                     /besthit="qcoverage 100 , hcoverage 100 , similarity 100
                     , identity 100 , evalue 7.26e-151 , alnlength 212 ,
                     Bacteria , Proteobacteria , WP_015589464.1 MULTISPECIES:
                     HAD-IA family hydrolase [unclassified Wolbachia]"
     gene            20976..21809
                     /locus_tag="wEsol_00623"
     CDS             20976..21809
                     /locus_tag="wEsol_00623"
                     /codon_start=1
                     /transl_table=11
                     /pfam="Pfam-B_16420"
                     /fullproduct="qcoverage 100 , hcoverage 100 , similarity
                     100 , identity 100 , evalue 1.77e-172 , alnlength 277 ,
                     Bacteria , Proteobacteria , WP_015589463.1 MULTISPECIES:
                     hypothetical protein [unclassified Wolbachia]"
                     /translation="MAQTSQESVEKKFKDVFERSPGAFPEGTSLEEKVNYIAKGTKAL
                     KAVKSKELENKMKNVAELNQKLESQEGRADEKARQDFERIAELDQQYKGKGIFTSEKV
                     AELKNDKKFQEAANTLRETTTEGKSVEQSGQKKTSEASVQTDTQPSEHIVSPSVSKSS
                     VKEKLNTEPDKKDTTPQPQENQKEEGFLSLLKRFVKKILEKLLGEDEKSVENTHDMRE
                     DAQRDTSNNEEQQKSKQLDEILKDKSVSEGLKQATKLENPAITPVNTPKVNEVNTGRE
                     R"
                     /product="hypothetical protein"
                     /besthit="qcoverage 100 , hcoverage 100 , similarity 100
                     , identity 100 , evalue 1.77e-172 , alnlength 277 ,
                     Bacteria , Proteobacteria , WP_015589463.1 MULTISPECIES:
                     hypothetical protein [unclassified Wolbachia]"
     gene            22697..23197
                     /locus_tag="wEsol_00624"
     CDS             22697..23197
                     /locus_tag="wEsol_00624"
                     /codon_start=1
                     /transl_table=11
```

```
                     /fullproduct="qcoverage 100 , hcoverage 100 , similarity
                     100 , identity 100 , evalue 1.73e-115 , alnlength 166 ,
                     Bacteria , Proteobacteria , WP_222933351.1 phospholipase
                     D family protein [Wolbachia endosymbiont of Drosophila
                     sechellia]"
                     /cog="Cls COG1502 438
                     Phosphatidylserine/phosphatidylglycerophosphate/
                     cardiolipin synthases and related enzymes"
                     /pfam="PLDc_2 Regulator_TrmB Pfam-B_9369 DUF1669"
                     /tigr="TIGR04265 bac_cardiolipin 483 cardiolipin
                     synthase"
                     /product="phospholipase D family protein"
                     /translation="MCFSLYYYAGFIFSPCPKATVCFSPGEDCAVPIINVIDQSKKSI
                     LVQEYTFTLGTVAKSLINAKERGVDVKVILDKSQLYSKYSVINELFSGGVPIWIDDKP
                     KIAHNKIMIVDNQKVITGSFNLSKTAKKGNAENLLIITDYPELIQQYVKNWEARMSQS
                     YKYAPN"
                     /besthit="qcoverage 100 , hcoverage 100 , similarity 100
                     , identity 100 , evalue 1.73e-115 , alnlength 166 ,
                     Bacteria , Proteobacteria , WP_222933351.1 phospholipase
                     D family protein [Wolbachia endosymbiont of Drosophila
                     sechellia]"
     gene            23307..>23382
                     /locus_tag="wEsol_00625"
     CDS             23307..>23382
                     /locus_tag="wEsol_00625"
                     /codon_start=1
                     /transl_table=11
                     /product="hypothetical protein"
                     /translation="MDYINDAAGSYTAIHDFFQNNGVGD"
                     /besthit="qcoverage 100 , hcoverage 40.3225806451613 ,
                     similarity 100 , identity 100 , evalue 6.87e-09 ,
                     alnlength 25 , Bacteria , Proteobacteria , EAL58815.1
                     conserved hypothetical protein [Wolbachia endosymbiont of
                     Drosophila ananassae]"
     gene            complement(3629..3719)
                     /locus_tag="wEsol_tRNA-12"
     tRNA            complement(3629..3719)
                     /locus_tag="wEsol_tRNA-12"
                     /gene="tRNA-Ser"
                     /intronpos="0..0"
                     /program="tRNAscan-SE"
                     /cove_score="73.8"
                     /evidence=predicted
                     /type="Ser"
                     /anticodon=GCT
                     /product="tRNA-Ser (GCT)"
ORIGIN
        1 tgactatcca gaactaagaa aagctatttc agatataata gaaagtattg cacaatcctc
       61 acaagagggg aaaaaagagt tatttacaac cctatcagaa caagttaaag ggatgaacct
      121 caaagattta ggtttagcaa ttaatgacca aggcttgcaa aaggaggtaa ataaagaatt
      181 atttataaaa ctcattgaag aagcacttaa agatttaact tctgaaaatg ttaactcaaa
      241 aataatagaa gaggcgttaa aatctgttaa tcagcaagca ataagtaatg ttataaataa
      301 taataaaggt ccactggaca atctagtcaa ttattttaaa tcagcctctg caaatgaaaa
      361 aataggaata gcttctgctg gaatggtatt aggagtagca ttttctccgc ttattgtgct
      421 tgctacatta atagcgcttg tagctattgg agcacgttat gtaggaatag gtataggaaa
      481 ggcaggggaa aaaataaaag aaggtgcgat tgatgcagga aaagcagtga aaagtttatc
      541 taaaaattta ggggataaat tgcctgaggt tgggccaaaa aaagataact ttagtaaatt
      601 atacaacagt ttggctcaag agattacagg ctataatatg tccgaagaag gtgaaaaaat
      661 aaaaaacgcc cagagtaaag caaaggtcat aggcatgctt caaaatgaag ggcagcgatc
      721 tgctatacaa gagttagtaa agaatggaac tattcaaaat ttttcagaag atgacatgaa
```

781 aaagcttaga gaaaaaggta ctagtactta tgaaagtgat aaggaagcac ttgaggaact
841 tatggagaaa tttcaatcta aaattatggc cattggtgag ggtaaaatag aggaggtaga
901 aaaaattgtt gatgagaaag ttgcagcaat gaagccatct tctcacttag gtatttcaag
961 tgctgcacga gttagtgaag gaggaatcac aggaccaagt tgatgcattt ttttacgcaa
1021 aaattactat cagaaagtgg ggttacgcta ctccttttta tccacttttt tgagcaatcc
1081 acttccacat ctaacacaca tctcagtgat tataatatcc aatttttgat catgtttttc
1141 tacaggtaaa tctttacaat attgtttctc ataggctaca cctataaata tttttccaag
1201 tggccgcagt ttttctatca ttgtatcata ccaaccaccg ccaaaaccca atctattaaa
1261 atgatcatca aaagcaataa caggagtaat gattgtgtcg ggaattacat cttcatttgt
1321 tttgttccat tcctcaaatt ttagtaactt atttttatta ggaattgcta ctttataatc
1381 taaatcgagt aaactatgca tcaaaggtac aacatttatt tccccgtcca ttggaatgta
1441 agctgctatt gttttgcctt taacgtagct taagttctga ttaaagagat taataaggga
1501 atttgctgca taactggaat aactttcatc aatatctttt cttatagctc tatattgctc
1561 tcttatttcc tttttgtgct gtttaatatc tttgaacatg atctatccat tttgcagtaa
1621 gtttttcttg taaactattt tgcttcaagc gctcacttaa aaagtcaaag tcaataaact
1681 ccatatcctg gtcttgatta ctgcagctat aaacaaaact tctttctcct gtttccttat
1741 ttatatgttg ctgaacacat tgagcacaga tttctttcat catacactgc ataggagaat
1801 taactgacga tattgctata tgacttgact tcagatatgg ctttaaaatt gtttttctag
1861 cttcattcac agctttcatc attttatcag aaccaatagt gataatttta tctataacgt
1921 tcaaattaat acctaatctt ccttgttgat aagagactat tgcatcaact atattaccat
1981 gaaaggattt atcttgatct ctgcttgttt ctatcaatcc ttcttcacat gcccaaacta
2041 ctgcatttga tgcacgctct atcagtgctc gtttaaatac atcacttaac ttcttatagc
2101 cagcgaagta caaaaccttg ttattatttt caagacatgc cttccctatc gaaaataaca
2161 ctgcatttcc aactccacca ccaattagca ttatattctt catgtgctat tttaactaca
2221 tttgttcaaa ttttttgaaa ttttatagaa caaagtaatt tatatagttc tatacacaaa
2281 gacagagaca aggacactct ctgacttcaa ctataccgat aactgatagc tgcattttta
2341 cgcatatcat ctaataacaa gttgctttgt atcataatct tttgttggta cattttctgt
2401 ttgattgctt ctatacccgc tacattactc ttgatatcac acaacatcat caactttgca
2461 gtgccatttt ctctcaattc tactatttca ttcacacttg ttttactaaa taaaatctgt
2521 aaatcaggat ttaaatctcg catttttatt tcaaattctt ttgcgtttgg cagcttaaaa
2581 ttatctgcta atttatcaaa atttaaacaa ttaacttttt gctccttgag attatctaat
2641 aaactttctg aaccttcaac cacaatctgt tttaatttta aagtgctttc cagcagtgca
2701 tgattaagtt gtactttatc tattactttt ataacagagt aaccttcact aaaactgaat
2761 ggacctgcta tgtcgctggt ttctaatcct tctaaaacgt tcttgagttt tccttttagc
2821 tgacttaaat taactgttgc tttacgcatc tttattggag attctgggtt attactgttg
2881 cgtaattttt ttactaaatc ttcagctata ccataaacgt ccttgtcttt ttgatcagga
2941 attataaact cttggaacgt gataagataa tccggctttt ctgtttgcct ttttacatca
3001 tttacttctt tatcgcttat attaataaat ggcacaattc ttacttcaat aattttgctc
3061 cacagtaact gacattttat ttgttttctt aaaataccaa gatctatatt atgcttctct
3121 atgtattgat caacttcatt agccttaagt ttaaaacttt gggttaaaaa taacatgata
3181 gcattatcta actcctcgtt gctcaatttt atattcagcc tctgcgcttc attgataatg
3241 ataatttcgt ctattagctc cctaagaatt tgaggctttg ctcctttttg atcaacactt
3301 tgagtgccaa acaatgaatt tataaagttg atgcgttttt cgatatctaa atttgaaatt
3361 ggctcaccat ttacatctgc aacaatttca atctcgattg caagcaacct aagtggcaac
3421 actattaaca gtaaaattag tattttatgc atataagtaa ggttaataac caattctata
3481 ataaatactg agtcaatgct agccaacttt ttaatagtga tctcagtgac aatggaaata
3541 aaaaccattg caacaaaatc aggtacgctg gtaaataggc taaaaacatg ctaatatagc
3601 gaatttagac cgtgctcaaa acagtatacg gaaggagagg gattcgaacc ctcgatacga
3661 cttttgggtc gtataacggt ttagcaaacc gccgccttca gccgctcggc caccccttcca
3721 aatttagaac ctaaagatta tataatacca agacctctac cacctgtcta atgcttttaa
3781 ttgcaatatt tgtatctgtt cagatgcatg gctaatgctg aaacaaaaat tccagtgctc
3841 cctttcttgt catcccagtg cgtgacactg ggatccagga ttttgattag cactaagttg
3901 gtgagcatat aagtgttatg ttaaaatata acgtttttac ggaaagtctg gatcccagtg
3961 tcacgcactg ggatgacatc gtttactatg tgccatattg caatgttcgt acggttgtgc
4021 gcggtataga ttccgctaac gagtagcgga atgacgaatt tgttattttt caaatcgtag
4081 agccacttta gctataaatc aacaaaaact aatttccgct tttaatagag cttgcaacaa
4141 tttgttttgc ttcttctaca ccaacccatt ctccaactgc taccgttttt ccctttttcta
4201 gatctttata atgagaaaag aaatgagtaa ttttatctag taggctttta gataagtcag
4261 aatagtcctt tatattgtca taatagctat caacactgga aacaggcaca gctaatattt
4321 tctcatcttc tcctttttca tctttagtga gtaatgcgcc tattggacgc accgatatta

4381 aaacactaga tgctaaggga aattgagtta gcaccaaaac atccacagga tcaccatcac
4441 ctgcgcaggt atttggtata aacccataat tgcaaggata agtcattgag gtagacaaaa
4501 atctgtcaac ttgtaacaac ccaaactctt tattaaattc atactttaca ggctcagcat
4561 tcgcacttat ttcaattacc acattcaccg catttggtcc cgctgttatt ttacttaaat
4621 ccataaatta cctcttaact taagattata caatatgtac ttttaagttg ctacatttat
4681 gtagttctaa gcttgttttg catggagcgg tcctaaattt tcattcatca tcttattgac
4741 tgttgaaagt ggtggttcct gtggtggttt caataattga aataaggcat atactattac
4801 aatagtaagc accgcaccaa ttatcaggtt agtaaggttg tcttgtggca aaacattttt
4861 tattgagttg agcaattttt gctccataag gaaataatca gcaacagata acccaacaga
4921 caatataaac atacctaaaa taagtttaca gactgtgttt ttatagtctt tttctttttt
4981 cttgctggtg ataagatttg gcctatattc attctcttct ggcaacactg agtagtatat
5041 attctcaggg ctcattacta attgttcaaa ttcatcacta ttttggtttt ttaacagttg
5101 ctctagggtg tttttttgtat ttataggaca tgttaatcca tccattttca atgctttctc
5161 ttgccgatcc acattctcat ggtcgattgt atatttatct ctacttgata atccgatcat
5221 gtgtagtttg ctagctaagt gcactatata gttctctatt aactgattct cacatatctc
5281 tagtacttct cttcttactg ttttgttaga tggtgaaagt tctttatagt aactacctat
5341 ggaaaacaac aatgcaatat ctttatctac ttttatatta tgaatagagt agtaacagcc
5401 aataacgcac ttatttaatg caaattctct gtcatatttg tttaatgtat ttccttcact
5461 tagtaacttt ctaagaccaa tgagtacttt attttcaaaa tcttcttctg ttttaaatat
5521 tggttctctc tttggatttg ctaagatgtt aagtaaatct aattttcttc catctactac
5581 ttcatttata tattcattta actcagatcc tttaataact tcatatattt tatctatgac
5641 atctttagta ttcaggttat tgttttcact tagaatttga taagcttgag cataaattaa
5701 aaacagccct tgtagactaa attttaagtt aaaatattta tctaatggtt cctctttttt
5761 tctaatgaat tcagacaaac caaaatgatt tctgtgatcc ataaaacgac ctaagtcagc
5821 atgttgaaag ctacctaaaa aaataaataa cgtctctaat aaattaccct taccaacaat
5881 ttttattagt tcttcaattt cttttatcat tgctgaaacc tataatgttc tactttatag
5941 tataagggat acatggcaaa atagcaataa atgttattag acttcttgca taacctaaac
6001 taagtagaaa aaggtatagc tataccgccg cggcactaac aagagatccc gctaacacgc
6061 agcgggatga cgagacttat ctttatgccg ccgcgaaccg tcataccgcg attcattcgc
6121 ggtatctctc aacatagatc ccgctaacac gtagcgggat gacgaattac ttaaccatca
6181 ttacaccgtc ataccgcgat tcattcgcgg tatctcagct agtcccgcta acaagcagcg
6241 ggatgacggt tgtcgtttag ctacaaacat taagaaattt accaaatgaa aaaaaaggca
6301 aaagaagccc cgtggttggc taattactta cattatactt tcaagtatgg cgttttttta
6361 ttctaaacac ttaataaccg cgatttagct gcttttaagc cgcaactaat ctaaattata
6421 aacgttaaga aatttactaa acagaaaaaa aggcaaaaga aaccctaggg tagctagtta
6481 ttcactatcc attttttaag ttggcgtttt ttaatgtttt aaatgcttta taagcgtatt
6541 tcagcttgtg taggtaaaaa cccagaaatt tgataaagac atagagtgca catagtgcaa
6601 aaaattaaac atgagacgcc agatacgtga gtttttttgt catttaatct gtacagagaa
6661 gataaataaa tagcttcagt ttcatgataa gggggctggc ggagtttgtc aagcaagttt
6721 ttttgtttct attcccaatg ttatatggtt atgcaagaag tctattgtat gtttacactc
6781 tgcattaaag cttttagtta agagcatatc catccaccgc cgataacttg ctcatcttta
6841 tacgctacac aggcttgacc tggactgatg ccaaagtaat cgtcgtttaa aatgacacag
6901 gctttatttt tttcgtcagt tgaatgtatt gttgctaaac ttcccgcatg tgatgatctc
6961 agcttcacag ttacttccat accttccttt ggttgttcta accaatttaa ctccttgatc
7021 aatatttttt tttgcattaa tacgttgatc ggacctacta taacctcatt attttctgta
7081 ttaattttta tcacataaag aggttcattg tgtgcgatac ccaagccctt tctttgcccc
7141 actgtaaaat ttactatgcc actgtgctcg cctagcactt ttccattaac atccactatt
7201 ttacctttct gtacagattg tggagctaat ttagctattg ttttactata gctttcggaa
7261 acaaagcata tatcttgact gtccggcttt tcagaaattt gtaagccaaa atactttgct
7321 aattttctta tatcactttt atagaaccca cctagtggaa atcgcaaaag cttcaattgc
7381 tcttgagttg tagcaaacaa aaaataactt tgatctttac ttttatcaat gcttctacac
7441 aactttacct caccattttt ttctaacctt ctcacgtaat gtcctgttac gagcacatcc
7501 gcaccaagat tttttgtaac ttgcaataga tcacgaaatt ttaccgtttg gttacatctc
7561 acgcatggta tgggagtttc cccacgcata taggtactcg caaaatcctc tattacttcc
7621 tttttgaata tttcctcata gtttaaaata tagtgaggaa agccaacact ttcagccaca
7681 cgcttagcgt cataaatatc ttgtccagcg cagcatgcgc cctttcttgc accatcggtg
7741 ccatagagtt gaagagttac acctatcacc tggtacccaa agttatatag cagtgctgca
7801 gcaacggagc tatcaactcc cccggacatt gcaacaacag ccttggtctg gtgcggagct
7861 ttatctttta gcagaggttc aatttcaaat tcttttagca taatcgcctt aatttataaa
7921 cacaaacttg gtgtcatgca agtagctgac actggaatct agtttctatt acacaatttc

7981 atcgaaacag catacttgtt tatagtcaat ttttctggat cccagtgttg gctacttgga
8041 tgacatcttt atgcttgagg taaaatttgc tataaaattc gatgaactac ctttaaaaat
8101 aatttaatcc tgcttaaata aaacatggcc aaaataatcc atgttttcaa gcacttttcc
8161 gctacccaga gcaacacaac aaagtgggtc atctgcaaca cgaactggta gttttgttgt
8221 ttcactgata actttgccca agttacgcaa taatccgccg ccaccggata aaattattcc
8281 tttatcgact atatcagaag aaagttcagg tggagtgctc tccagtgcag tcctaatagc
8341 agaaattatc tgatgtacag gctctattaa actctctgca acttggtatt ctgataaaag
8401 catttctttt ggcatgccac tcactaaatc cctgccttta attatcattc cctctttgtt
8461 attttcacct ggcaaattgg ctgaacctat gcttttctta attttctcag cggttgtttc
8521 accgattaat aatttatgat tttcacgaat atatgatttt attgcttcat ccataatatc
8581 gccacctact ctggcagaac gtgaataaac aattccacct aaagaaataa ttgcaacttc
8641 agttgtaccg cctcctatat caacgatcat agaaccctca ggttcagtaa ccgggagccc
8701 agctccgatt gctgcagcca ttggttcttc aattaaaaat acttcatttg caccagcact
8761 ttctgctgca tcttgtatag cacgcctttc aactggcgtg gatccagatg gaacgcatat
8821 gataatatta ggtttattaa cagtgaattt tgtgtttgca ctgcgtatga aatattttag
8881 catttcttcc gcacttttaa aatcagcaat gactccatct tttaagggcc ttatcgcctc
8941 tatttctcca ggcgtttttc caagcatcat tttagctttt ttaccaaaag cataaggaac
9001 gtagcttcct ttttctttta ttcttgctac aactgaaggc tcatcaagca ctattccctg
9061 atttttctga taaactaaag tgtttgcagt gccaagatct atagcaatat cgctagcaaa
9121 taaacctttg aaagtgaaaa gactacaaaa cttttcggtt aattttcgaa caaaattcat
9181 acgcaactca agaaaatcat ttattaagac aaattatact ttccagataa gtagctaatg
9241 cagcactcaa cgtctaaacc cttacctgtt acttttttta gcagttccag gccacacttt
9301 gcactgtata tattttgaga taaccaactg ataagtaaac taaaatctcc ttttattata
9361 gcacttaatg attcgtaatg attcttttta acgcaagaga aaatctgcac agcagcaatt
9421 aaagcaataa tttttatagg aaagtagccc ataacaccgc ttgcccaata ttcatcttga
9481 aaataagtgt ttagctcatt ttcagctttt actggaattt tatagtgctt catacсttcc
9541 agccacgcat catgcaggtc tttgacttcc aatgtaccat ttattatatc ttgttctaac
9601 ctagttctca acataatatg agctaacaga ctaaattcat ctgcattttt taaaaaagaa
9661 gaaaggttta tttcattgaa aatcaagtac aaattttcaa cactgctaac tttctcgtta
9721 gttttgcctt ttatagcaaa tttctctttt atgtgtggtt gaatgaactc aataaattct
9781 ctggatgttc caatcatcct ttccatgaat aacccttgag tttcatacat aacatgtctc
9841 gtaattgggc tacttataga attttgcgct aaacattttt gataaattgc ataaccacta
9901 tgccgtaaaa gagaaaataa accataacaa aagtcagatt catcataatc tatagggtag
9961 taataagaag tacgaacctc atgcagtgcg acacccattt gctgtaaaca tgacgagcca
10021 agttcaatct gcttctgagt agcaactttt tgtatattag taaccttatc ctttttctgc
10081 ttttcaatta ttttatctac atttccacta aagaatttgc ctaccttggg gaatacttcc
10141 cttatattct tttctgtgat atcagagtca tagtcagcaa gcagtgagtc gtattttgag
10201 cattttaatt gctgcgattt tatagaagct acttcgcgaa tgagtgtgat taaatcagta
10261 aaacgttctt ttagtttttc caagttactg gcttcagaat tgtttagcct ccataaactt
10321 tggcattcaa ctttagcttt ggacaaagat tttactaaat caataggaac agcactgttg
10381 ctcttatgta ttctctctat taactttagc tgccaagcat ctgcactctt tttgttgtca
10441 agagcatctg ctaatgattc ctttattacg tcatgagaga taatttcatg tctgatttct
10501 tctagaagac tcatttgttc aactttgtcc tctgcattca attggctttg gtttagtact
10561 tttagcgtat tttcgatatt ctttacccta tataatactt cctctaaaaa cttataagat
10621 ttcatatttg ttcattattt tagaaaacat ctgtttaaca tcgtgataga ttaataataa
10681 agttaaaagt atatttgctt aaagtacaat aaaaaattgt acttttttatt cttttattgg
10741 atttttctac tcagagtcca gcatattata ggaaaattaa ttgtaaggtt gtagaaaaag
10801 tatttagtat tttatttctc ttgactatat tacaatatgg agagatatta gctactgaaa
10861 ttaaagttac gcactattta aaatttatgg aggtttagta atgtggagta gactggttat
10921 aatgtgctgt ttttgtttat tacttactgg tgtaagttct tgcccaaaaa aaggagcaaa
10981 tacaacaaat aaaataaatt ctgttgttaa gcagataggt gataaaagag ttttctttgg
11041 ttatgatgaa tctagtattg ctgaagtaag tgcagatgca ttacttgacg taatggaggt
11101 gttacaagat aaccctgacg cgaaggttac tttaactggt cacactgaca accgtggttc
11161 tcatgaatat aatcttgcgc taggcgctag aagggcagat gcagctaaaa aatttatggt
11221 tagttgtaca ccctacatag aaaacagaat aaaaactgct tctaaaggtg aaactgagcc
11281 tttggttaat gtaaaagatg attctagaaa ttctaaatat gaaaaagagc atgctaaaaa
11341 tcgtagagtg gaattttcat tttctggaat aaagaaatag tttcttgcat taaaacctgg
11401 atcccagtgt caagcactgg gatgacataa agaaagggca ctggggtaga caaaaaagga
11461 caaacttgtc atatttcgtt gcttgttagc gggatctata tataccacaa ataagtcgct
11521 gtatgacgta gagactatct ggccatcaag aggcataatg gaattagagt tattatttca

11581 aaatgtggtt aatagttttg ctttctataa taatcaagtt gcagttgcag tatcaggtgg
11641 tgtagatagt ataatcttac tgcacttaat gaataactgg gcaaaaaaaa acaagcttcc
11701 acttcctata gcattaacag taaatcatgg gttacgttca gagtctcaaa aagaagctga
11761 ttttgttgta agttatgcaa aggaacttgg agcaaaggaa tcgttcatat taaattggga
11821 gaagcaaaat attaaaggca atattcagtt acaggcacga aaagcacggt ataagttact
11881 agcagagtgg tgtaaaaata ataatgttaa atatttactt gttgctcatc acaaagatga
11941 tcaagcagaa acgttcttgt taagattaga gcgaggtagt ggcgtagatg gattatcatc
12001 aatggactac aagtctttcc taaatggtat ttatatattt aggccattgt taaattttag
12061 tcgtagtgaa atagaaaagt acgctaagct tcatcggtta aaatggatcg aagatagaag
12121 caactatgac ttaaaataca ggcgaacttt ataccgtaac ttacttaaag caagtgataa
12181 tcaagagatt ttaacagagc gaatatgcct cacagccctt catatgaaaa gagctgcaaa
12241 agcgttgatg cactacacac gccttgcgtt taatgactgc gttaatgtgc atgatcttgg
12301 ttatattgaa attaaactaa gtgagtttta tcaattgcca gaggaaatag ccttgaggct
12361 tcttctttac tccataatgg caattgccag taaacattat aaaccaaggt acaacagtct
12421 tatcgtaata tttaataaaa tattgcaaaa ggatagtaat gttaattgta cactttctgg
12481 gtgtaaaata agaaaacatg gagaaaatat cttgataatt agagaatcgt caaagataca
12541 agaaattacc ttaaacctac ctttaaatga acctactcaa tgggataaca gatttagctg
12601 cacaatactc ggaaatcaag agtgttcagt tatcatcgct ccattaaaaa aaacacaaaa
12661 agttcctgaa tttctgaagg attacaactg ctgccctgaa gttttctatt ctttgcctgt
12721 agtgctaaaa gatggaaagg tgcttgctta tcctgatgta aattataacg gaaaaaatac
12781 caatgacgat aaggttcaat gcattattaa tagcacgata aaacaaaatt tggtaagctt
12841 gattagtatt tagttagaaa tagaaatgaa aaaatttta gaaggcttat tgatctggtt
12901 agtaattatt gttcttattt cagttgctta tattcagttc agcggaaatg taggtaaaag
12961 taaaacaatt ataccttttt cggaattttt aactagacta gaagacaatg atgtagaaaa
13021 tattgtcata aaaaaccaga gcattgaagg caagttcagg gacggttcaa gtttcaattc
13081 aagcggtgtc atatatagcg acctaataaa aaatttacat gatagaaaag tgagattctc
13141 cttttcaact ggagattctg ccatgaacgt aattggtgga ttacttatct catgggtccc
13201 aacatttatc tttattggct tcttgctatt ctttcttaaa caaacacaag cgggggggcaa
13261 cagaactata agctttggca agtcaagggc tagactcatg actagtggaa aaaaagtaac
13321 atttgatgat gttgccggaa ttgatgaagc aaaagaagag ctagtagaga tcgttgattt
13381 tcttaaacaa aggcaaaaat ttcaaatatt aggtggaaaa ataccaaaag ggtgcctttt
13441 gattggctct cctggaactg gtaaaacttt acttgctcgt gcaattgcag gagaagctaa
13501 tgtgccattc tttagtattt ctggatctga ttttgttgaa atgtttgtgg gtgttggtgc
13561 aagtcgtgtt cgtgatatgt ttgatcaagg caagaaaaat gctccttgta taatttttat
13621 agatgaaata gatgcggtgg gtagacatcg tggcattggt cttggtggcg gcaatgatga
13681 aagagaacaa acattaaatc agttattagt tgagatggat ggctttgagt ctaatgaagg
13741 tgtaataata attgctgcga ctaaccgtcc ggatgtctta gatccagcac tgcttagacc
13801 tggtcgtttt gaccgacaga ttactatttc tttacccgat ataaatgggc gtgaaaagat
13861 attaaatact catataaaga aaatatcaat agcaccagat gtaaacgtga aaacagttgc
13921 aagaggaaca ccaggttttt caggggctga tctagcaaat ttggtgaatg aatctgcact
13981 tatcgctgca agaagaaaca agaagattgt taccatggat gatttcgaat atgcacgtga
14041 taaagtgatg atgggcgtag aaaggcgatc tctcattatg acagaagagg aaaagagact
14101 gactgcatac catgaagctg gtcatgcgat gattgctgtt aatatgcctg cttctgatcc
14161 tatacacaag gcaacaatta ttccacgcgg tagggcactc ggtttagtta tgagactacc
14221 agaaacagat agagtgtccc tcacaagaga aaagatgcta gcagatataa ctgttgcaat
14281 gggtggaaga gtggcagaag agctaatttt tggctacgat aaagtcacaa gcggtgcatc
14341 ttcggatata aaacaagcat ctgatttatc acgcgctatg gtaacaaaat ggggaatgag
14401 tgacaaaata ggcccgatct atcacaatcg cgaacaaacc atacatggtt ctgagataat
14461 ttctgaagat acgttaaaac ttatagatga agaagtgaag aaagttgtat cttcttgcta
14521 tgaaaaagca aaagacattt tgaccaagca taagaaaggc ttagatctca ttgccgaaaa
14581 tctactggag tttgaaactt taacaggaga tgaaataaag gacatattaa atggaaaaaa
14641 aattgttaga gaggaaaatg agaagaatga gaaaataaaa aatcctctc tctatgagag
14701 ttgacctcat cttaaaaatg ctaatcagct aacgctgcaa taaaacattc agaattgtaa
14761 ctgtcaatat aaattgtatt aaaatattca taatattgca ttttcatcac aataatgcta
14821 tcattcagtt agtattaatg tttaatgagg tgtgagtaat gattgatata gcttctattg
14881 gtgtaagtac agttattgat cagaattata gaagaaaagc acaattatgt ttatcaggat
14941 tatttgtaac tgtgtcaata gcaactgtta cctggtttat tttccctgcg tcacaaaatg
15001 tattagctcc gtttttactt ccggctatgt tcttgctctt cactttatcg atgggttatt
15061 acatgttcat atttagttgt gaagaaaaat ctttggtaaa gaatgttctt ggtatggttg
15121 aaccggctga atcggtaaag agagtgcatt gccaagaaaa tattaagtcc ttattagcag

15181 aagtgagaag tactacgaaa gataggtttc gtaacgtgct tgttaatctt tgttttgaag
15241 atcgtagtaa agcacacaaa cttgcattat atgtgaaaga aattgacaat attgtgtctt
15301 caataaaaaa taaagacctc ttagaccatt ttgatcaatt ttttactatt actgaagtaa
15361 gcaataaaaa aaatagaagt gaattaatga acctcgcaag ggcatataag atgataagtg
15421 aagaagatcc agacaaaata caggattttt tagctactca aacaaaaagt tattcatatg
15481 aaataatgtt acctgaaatt gataattttc tatcacaagc taggtgattt tatcaataat
15541 ttcattgtgt acgtgattat catttttttgt ttcaattcca gtgataacac tgcacctaac
15601 gggctctttt atggctattt cctgaaatcc ttttctgacc ttattgtaaa aattaacatc
15661 catttcttca tatttattct tgtcttttgc tctatttaac ccaactttaa catcaatatc
15721 tagaataaat gtaatatctg gacatttaat ttctactaac ttatgtaagt cccttattag
15781 ccctaaatca acactaagcc catatccttg gtatgcaata gttgaatcaa taaaccgatc
15841 acaaatcact atttttcctt ctgcaagagc tggtaatatt aattttttca tatgttcgcg
15901 cctcatcgaa ataagtagca agagttcaga aataggatca atattattcg ttaacaacac
15961 tcctcttatc ttttctgcaa agttagtgcc acctggttct cgagtcaata ccacattatt
16021 ttcgccgtga atttgcttaa aatgatttgc aagtagctta gattgtgttg ttttaccaga
16081 gccgtctatt ccttcgaaag ttatgaacat aagttttata attataatac taaaaaagaa
16141 tatgcaaaca gtatacatag aaaccgtttg acatttaaat aataagaccg taaattacat
16201 gtggtataat aatagtctat ggttgctctg aatactccta aagtcgattt tagttttact
16261 gcaaaagatt ttaatctttt gggagtagac aataaatact atacattaag tgactgttgt
16321 ggaaaaaatg gtcttattgt aatgtttata tgcaatcact gtccttacgt tcagtcaatt
16381 attagtaatc tggtaagcga tgttgatcaa ttgaaaaaag attatcaggt aaacaccatt
16441 gcaataatgc caaatgatgt aaatgaatat ccagaagatt cctttgaaaa tatgattaat
16501 tttgccaagg aaaataagtt tacatttcca tatttaattg atagtacaca gaaaatagct
16561 aaagaatacg gggctgtttg cactcctgac tttttcggct ttaattccaa tttaaacctt
16621 tgctatcgtg gacgttttaa caacgcaaaa aaagaaaagg ttcaaagcta tgaagtagga
16681 agtagtgact tatttcaagc tatgaaattt attgcagaaa ctggtaattc tccaattgac
16741 caaaaatcaa gtattggctg ctcaattaaa tggtctaatt ctacaggtta aatatcatgc
16801 acagtggctc tcagcatttg tttgatatta taattttgct ttctgctgct gtgtttatag
16861 tcatagcatt ttggaaaatg aatattagtc cagtgcttgg ttacttcgtt gcaggtgcag
16921 ttattggttc tcacggattt aatctgatac actcagctga agcaatggat aatcttgcag
16981 aatttggtgt agtttttcta ttattcatta taggccttga attgacattt gaacgcctga
17041 tcgctatgcg tattcatgta tttgggttcg gttctcttca agttatagtt accatggtag
17101 caatatggtg catcgctctt gctttcggag tgaatacgaa tatagcaaca gttattggtg
17161 gtgggcttgc gctatcctca acagcaatag tgttgcaggt tctgcaagaa aaaggttctc
17221 aagcaagtca ggttggtaga ttgtcgatag cagtgctatt aatgcaagat tttgcagtgg
17281 taccattaat agtattggta cctttactta ctggcaattc tgagcacagc ctgataagct
17341 cattggcagg ttcattggtg caagcagcta ttgcattagt gctgatattt ataactggta
17401 gattattgct tagaccttta ttttcggtta ttgctaaaat ggaaagtaat gagatcttta
17461 tatcaacaac gcttttaata gtattaggag cagcatttat tactgagcaa ttccatttat
17521 cgttagcatt aggtgcattt gttgctggat tgttagttgc agaaacagaa tatagacact
17581 cagtagaaca cgcagtattg ccatttaaag atttgtttct tggcttattt ttcatgactg
17641 ttggtatgtc catcaatacc gaacttttac tcaataagtt accactaatt actttattat
17701 cgattatcct tatcgtttta aaaacatcta ttatatatat attgtgtaga ttttttgggt
17761 ttaagagtgc acctgctata caagctgggt tactgctttc acaaggcggt gaatttgcgt
17821 ttattttatt tcgcttagca gatgagctaa atgtgctacc aagtgaaatc gctcaagtac
17881 ttatgatggt aactacagta accatggctt tcactcctct tttatcaggg cttggagatt
17941 ggatagcaaa ctcatttagc actgagaaaa caatattaga tgatgaagct gttgaaacag
18001 atacacaaga tctttataac catgtgatag ttgctggatt tggtagagtg ggatatatgg
18061 taacaaaaat gcttacggca gagcatttaa gttacgttgt tgtagatatt caatcgaaaa
18121 tagtcaaaga aggaaaaagt gatagttttc ctatatatct tggagatgtt acaagatgtg
18181 aaattttaaa atcggtagga atagaaagag ctcaagctct cgttatttca ataaagaatg
18241 aagttactat aaaaaaagtt gtttctttag ttgcagcaaa ttttccgcat gtaaatattg
18301 taatacgctt accagatctg agtaatgtgg aggtttatag agatctagga gctagtaaaa
18361 ttattcctga aacatctgag ataggattgc agctaggtgg agctgcactg agccttagtg
18421 gtattagcga aagtggagtt atgtctttaa aaagtagatt cagaaaaggc aattacagta
18481 tgttaaaaga ccttggtagt gataaagatg aataactact tctctaattt tatgtacaga
18541 aagcttcaag ctttattgag gttttgccta tagtaaatta cgcacataat caaccaaatt
18601 aaggaaagtg caaaccaagt gattgcatat tccaaatgct tcattggctg tatagctact
18661 ttgctgccaa aattgttcgg ccacaatata cacttctcta gcttaatacc taactcattg
18721 gaaatttctt ctgtactcag ggtaaaccat gtattcgaag tagtatcgtt cttgataaac

18781 caatttttgc ttttactgct attgcaatat aaaactccac ctgcagctac tttttcaatt
18841 tttgctcttt cctctttttt ttctttaact attcctttgt tcactagcat ataatttcca
18901 gtagtaagta gcataggaga taacacatga tatccatatt gccctgcaaa aacatatagt
18961 tctatgtcac ttagaatccc atcgatcttg acatgcctat agttaaattt ttcaaggtcg
19021 ttattgggca atagatgaac aactggaaga ctcatatttt tgataatatt attcttccag
19081 ttcaatctga atacttgcca taatcctaat aaaaaaagga gcaagcaagg tacaattaaa
19141 ataaatacta tttttttaa cactctatat ttgtgttctt aaatagtttt caacacccat
19201 tagttcaata aggtcaatct gtttctcaat ccaatggaag tgctcttctt catttttaa
19261 catctcttct aataacatta cactaacaaa atctttttct ttctcagcga tagaaatcgt
19321 ttctttgata tccttaatac cttttccctc taatttcaaa ttagcttcta agattttccg
19381 tattgtgtct tttgtaaact ctccatcatg ctttgatatt tcgtttgtat cctgaaaatt
19441 tggaactcct ttaaataaca aaattctttc tgccaacttg tttgcatgtt caagttcttc
19501 gttaagctca ttttttacct tttctgcaag tctattaatt ccattatttt tgagaatcgc
19561 aaaatgcaaa agatactggc gtacagaagt cagctcattg gtcaataatt tattcaaatg
19621 ttttactatc tcttcattca taatcttccc ttagttcaat tcatcagcat aatttatatg
19681 ataacgatag ttatagcaac tcatttaata gttcctttga taacaaccgt attaaatagc
19741 atgtcgtggt atgtctgttt ctttttagaa aaacaagcaa ctagatacca gcagagagtg
19801 agcagcacta tcattacttt caatgcactc aagacattca ctaatacttc ctgatcttgc
19861 aaaaattcag taaggataac tgctacactg tttaacaaag gaagaaaaaa ctgggaagtg
19921 cttctgtcaa acgctaaatt caagtctatt ttagaattat ctaaattgat agtatatata
19981 ttcagtaact gttggccggg agttgcttgg gcctttgaag atataaaata tgtgaagtaa
20041 ctacaattta cacacatgta tgatagatgt aagatcaacg gagaatcctc taatagcagt
20101 ataattaata aagttggaat taataaaatt gctacatcta ttcaatacgc gcaaaagcgc
20161 ctttttatgc tggcaatctc tgtcattacc tatcttctaa atactttatt acaaaatctg
20221 taagtttatc aaaatgttga aaacataaca aatcttcata accgcttatt gattgaccgt
20281 atataatagg taaacaattg gcattttgtg cacacagaac atctgtgatg ctatcaccaa
20341 cgaaaaacac attttctcta tttataggca acgtactctc ttctagtgca aatagcagtg
20401 gggttgcaga tggtttatct tccgcggtat cgcatgaccc aaccactctt tcaaagtaag
20461 aatccagttt aaaataggta acttcttcac gtaaattagt atttttctta ttacttacta
20521 ttgctagata aacattgtgg cttttcagtg tctgcaacat ttcctctact ccttgattca
20581 gagcaatgtt ttgtaatagc gcattatcta agtattgttg atatatctga ttcgcttttt
20641 tccactgatc gccaaataaa ttgaccatat agctctttct cgactcatgg gaatttctat
20701 cagcagcttt attactatac cccattgagt ttatggtatg cttaatagca ttaaaaatat
20761 tatcttgagt gtcaactaag gtattatccc aatcgaatac tactgctaat gggctgtttt
20821 tcatttctat attgttatat attttaagtt aataaataaa tagcatttta atgaaaaaga
20881 tataaatagg acgctctacc acaagttatg ttaactgttt tttatataat tagctaatat
20941 actggttata tgatatgttt taataggtaa taggtatggc ccagacatct caagaaagtg
21001 ttgaaaaaaa gtttaaagac gtctttgaac gttctccagg tgcttttcca gaaggtactt
21061 cattagaaga gaaggttaat tatatcgcaa aaggaacaaa ggctctaaaa gctgtgaaaa
21121 gtaaggagct tgagaacaaa atgaaaaacg ttgcagaatt gaatcagaaa cttgaaagtc
21181 aagaaggacg tgcagatgaa aaagcacgtc aagattttga aagaattgct gagcttgacc
21241 aacaatataa agggaagggc atcttcacaa gtgaaaaagt tgcagaatta aaaaatgaca
21301 aaaaatttca agaagcagct aacactctta gagaaactac aacagaaggt aaaagtgttg
21361 aacagagtgg acaaaaaaag acttctgagg cttctgtaca aacagataca cagccatcag
21421 agcacatcgt tagtccctca gtgagcaaga gttctgtaaa agagaagctc aacactgagc
21481 cggacaaaaa agataccacg cctcaaccac aagaaaatca aaaagaagaa gggtttttga
21541 gtcttctgaa gcgctttgtt aagaaaatat tagaaaaact tttgggtgag gatgaaaaga
21601 gtgtggagaa tactcatgac atgagagaag atgctcaaag agatacgtca aataacgaag
21661 agcaacaaaa gagtaagcag cttgatgaaa ttttaaagga caaaagtgta agcgagggtt
21721 tgaagcaggc aacaaagctt gagaacccgg caataacgcc tgttaatact cctaaagtga
21781 atgaagtaaa cacgggtcgt gaaaggtgat ccgctttcaa gcttcattat accgccacga
21841 accgtcatac cgccgcggta tctcatccgc taacaagtag cgggatgacg attgtcaggg
21901 tgtcatccca gtgtccctat gatgtcatcc cagtgccctg actacttgga tctagcaagc
21961 tttgcttgca aataagtcta atacgcgtcg cgttttatgc caaaacacaa ttggagttac
22021 atgcaaaata gatcccagtg tcagctactc ggatgacaaa aaaaggagca ctgggatgac
22081 accatttgtt gtatttaaca aagttcgtac ttagcttcat gttagctata aacattaaga
22141 aatttaccaa gcgaaaaaaa aggcaaaaga agccccagct aatatctatt ttagaacatt
22201 ggcgtttttt aagtcttaaa cactgcaatt tagctgcttt taaatgcaac tcaccttagt
22261 ttaaatgttt aagaaattta ctaagcagaa aaaaaggcaa aagaaacccc gtgttagcta
22321 gttgtcactc cctaatcctg caaattggcg tactatactg tcttaaacga cttataagcg

```
    22381 cgtttcagct tgtgtaggta aaaacctaga aatattgtga agacataagg tgcacatagt
    22441 gcaaaaaatt aaaaataaga cgccaactac gttgttttct tgctgtttaa tctgcacaga
    22501 tgaagataac tgaatacctt cagtttcatg ataagggggc tggcggagtt tgtcaagtaa
    22561 gtttttcgt ttctaacggt tgttgccaaa aactatgcaa gaagtctatt gacaggtgat
    22621 taaattataa tgtagcctat cgccgtatat ccgggggtgg gttgttgtga ggctttttca
    22681 ctttttattc ttgttaatgt gtttttctct gtattactat gcgggtttta tattttcgcc
    22741 ctgcccaaaa gctacggttt gcttttcacc tggagaagat tgtgctgtac cgataatcaa
    22801 cgtaatagat cagtctaaaa aatctatctt agttcaagaa tatacattta ctcttggaac
    22861 agttgcaaaa tctttgatta atgctaaaga acgcggtgtt gatgttaaag tcatcttaga
    22921 taaatcgcaa ctctactcaa aatatagtgt tataaatgaa ttattttcag gtggagtacc
    22981 aatttggatc gatgataagc caaagattgc tcataataag ataatgattg ttgacaatca
    23041 aaaagtcatc acagggtcat ttaaccttag taagacagct aaaaaaggaa atgctgaaaa
    23101 tttattaatt attacagatt atcctgaatt gattcagcag tatgtgaaaa actgggaggc
    23161 acgaatgtca caatcttaca aatacgctcc caactaatcg agagcgcttg ctcaaaatca
    23221 gttttaaaaa cagcaaggga gagctttttt gattgattgg cataatgatt atgatataat
    23281 gaactaaaaa tttaaggaat aaagtaatgg attatataaa tgatgctgcc ggtagctaca
    23341 ctgctattca tgattttttt caaaacaacg gtgttggaga tt
//
LOCUS       JAQZAU010000058           19355 bp    dna     linear   ENV 17-MAR-2023
DEFINITION  MAG: Wolbachia pipientis isolate wEsol contig_36250, whole genome
            shotgun sequence.
ACCESSION   JAQZAU010000058 JAQZAU010000000
VERSION     JAQZAU010000058.1
DBLINK      BioProject: PRJNA929258
            BioSample: SAMN33142973
KEYWORDS    WGS; ENV; Metagenome Assembled Genome; MAG.
SOURCE      Wolbachia pipientis (insect metagenome)
  ORGANISM  Wolbachia pipientis
            Bacteria; Pseudomonadota; Alphaproteobacteria; Rickettsiales;
            Anaplasmataceae; Wolbachieae; Wolbachia.
REFERENCE   1  (bases 1 to 19355)
  AUTHORS   Fiutek,N., Couger,B.M., Roy,S., de la Torre,J.R. and Connor,E.F.
  TITLE     Genomic assessment of the contribution of the Wolbachia
            endosymbiont of Eurosta solidaginis to gall-induction
  JOURNAL   International Journal Molecular Sciences
REFERENCE   2  (bases 1 to 19355)
  AUTHORS   Fiutek,N., Couger,B.M., Roy,S., de la Torre,J.R. and Connor,E.F.
  TITLE     Direct Submission
  JOURNAL   Submitted (10-FEB-2023) Biology, San Francisco State University,
            1600 Holloway Ave, San Francisco, CA 94132, USA
COMMENT     ##Genome-Assembly-Data-START##
            Assembly Date          :: JUN-2019
            Assembly Method        :: FLYE v. 06-2019
            Genome Representation  :: Full
            Expected Final Version :: Yes
            Genome Coverage        :: 100.0x
            Sequencing Technology  :: Illumina HiSeq; PacBio Sequel
            ##Genome-Assembly-Data-END##
FEATURES             Location/Qualifiers
     source          1..19355
                     /organism="Wolbachia pipientis"
                     /mol_type="genomic DNA"
                     /submitter_seqid="contig_36250"
                     /isolate="wEsol"
                     /isolation_source="galls on Solidago altissima"
                     /host="Eurosta solidaginis MN-2018-EFC"
                     /db_xref="taxon:955"
                     /environmental_sample
                     /country="USA: Cowling Arboretum, Carleton College,
                     Northfield, MN"
```

```
                     /lat_lon="44.48 N 93.13 W"
                     /altitude="280 m"
                     /collection_date="2018-11"
                     /metagenome_source="insect metagenome"
                     /note="metagenomic"
     gene            complement(<1..873)
                     /locus_tag="wEsol_00626"
     CDS             complement(<1..873)
                     /locus_tag="wEsol_00626"
                     /codon_start=1
                     /transl_table=11
                     /fullproduct="qcoverage 100 , hcoverage 86.865671641791 ,
                     similarity 100 , identity 100 , evalue 5.01e-201 ,
                     alnlength 291 , Bacteria , Proteobacteria ,
                     WP_015589155.1 MULTISPECIES: tRNA
                     (adenosine(37)-N6)-threonylcarbamoyltransferase complex
                     transferase subunit TsaD [unclassified Wolbachia]"
                     /cog="QRI7 COG0533 342 Metal-dependent proteases with
                     possible chaperone activity"
                     /pfam="Peptidase_M22"
                     /tigr="TIGR03723 bact_gcp 314 putative glycoprotease GCP"
                     /product="tRNA
                     (adenosine(37)-N6)-threonylcarbamoyltransferase complex
                     transferase subunit TsaD"
                     /translation="MKTILAVETSCDETAVAIVNSDKQVLAHKILSQVEHKKRGGVIP
                     EIASRAHMKHLSGLIKSAVEKSNLNFCDLNAIAATSGPGLIGGLIVGTMMAKAIAHVA
                     QKPFIAVNHLEAHALVIRLLHEVKFPFLVLLISGGHCQFLIAQDVGKYIKLGETLDDS
                     LGEAFDKVAKMLGLSYPGGPLIEKLAKKGNGTRFKLPRAMIKRSGCNFSFSGIKTAVK
                     NLVQELKMSEQDVCDVCASFQECISDILLDRVSNAIIMAESLNIKINDFVITGGVAAN
                     NFLREKLKQHINLNI"
                     /besthit="qcoverage 100 , hcoverage 86.865671641791 ,
                     similarity 100 , identity 100 , evalue 5.01e-201 ,
                     alnlength 291 , Bacteria , Proteobacteria ,
                     WP_015589155.1 MULTISPECIES: tRNA
                     (adenosine(37)-N6)-threonylcarbamoyltransferase complex
                     transferase subunit TsaD [unclassified Wolbachia]"
     gene            complement(1313..2050)
                     /locus_tag="wEsol_00627"
     CDS             complement(1313..2050)
                     /locus_tag="wEsol_00627"
                     /codon_start=1
                     /transl_table=11
                     /fullproduct="qcoverage 100 , hcoverage 100 , similarity
                     100 , identity 100 , evalue 5.42e-167 , alnlength 245 ,
                     Bacteria , Proteobacteria , WP_010081987.1 MULTISPECIES:
                     hypothetical protein [Wolbachia]"
                     /translation="MLIRNLIKQATQVGSSENAIRYVEYLYQFEKFRNTLNLTLSLIK
                     QNRLSFEIYQEGLVDMEEGVCKTIRSSSVNRYVIVLRKSNPYIIVHELSHMVENELNL
                     SLEQEFLHKVYQDIEQNLKQANILVQKIIGQVIFKEIQAYQTAKSRASELFARYFELF
                     AWAQEVYPKDKEYLIRTQDLNKVFLATGQWKKSYLDSQMMERIDKEIKEYSNKTATKD
                     VTKVQSSWTNKFSPGGKKISSIFGDDD"
                     /product="hypothetical protein"
                     /besthit="qcoverage 100 , hcoverage 100 , similarity 100
                     , identity 100 , evalue 5.42e-167 , alnlength 245 ,
                     Bacteria , Proteobacteria , WP_010081987.1 MULTISPECIES:
                     hypothetical protein [Wolbachia]"
     gene            complement(2964..4100)
                     /locus_tag="wEsol_00628"
     CDS             complement(2964..4100)
                     /locus_tag="wEsol_00628"
```

```
                     /codon_start=1
                     /transl_table=11
                     /fullproduct="qcoverage 100 , hcoverage 100 , similarity
                     100 , identity 100 , evalue 3.79e-268 , alnlength 378 ,
                     Bacteria , Proteobacteria , WP_151808057.1 MULTISPECIES:
                     cysteine desulfurase [unclassified Wolbachia]"
                     /cog="NifS COG1104 386 Cysteine sulfinate
                     desulfinase/cysteine desulfurase and related enzymes"
                     /pfam="Aminotran_5 Aminotran_1_2 Beta_elim_lyase"
                     /tigr="TIGR03402 FeS_nifS 379 cysteine desulfurase NifS"
                     /product="cysteine
                     desulfurase"
                     /translation="MSPFSLENSGCVYADYNATAPISENVKKSIFEVLLKQTLNPSSL
                     HKRGQEARKILQDARDNVRGAIGVSSDKEIVFTSGATEANNLVMRGIAGYLHVISAIE
                     HPSILNSACNPYIIPVNQEGIVDFLELEKILSELKGNKAIVSVMMANNETGVIQPIEE
                     IAEIAHKFGAICHTDTAQSVGKIKVNMEDLGVDLLTLSAHKFGGVAGSGVLIFNKELA
                     IEPIIIGGGQEKGFRGGTENIVAIAGLSAALQNIPDLLSKMDEVKELRDQLECELLNL
                     VSDIRIFGKNSKRLPNTSFIYMPGVKSDVQLMHFDLNYIAVSNGSACSSGKVEPSHVL
                     LAMGATKEQTECSIRISIGPETKPQDIKKIVDCWYNIYKQNSLV"
                     /besthit="qcoverage 100 , hcoverage 100 , similarity 100
                     , identity 100 , evalue 3.79e-268 , alnlength 378 ,
                     Bacteria , Proteobacteria , WP_151808057.1 MULTISPECIES:
                     cysteine desulfurase [unclassified Wolbachia]"
     gene            4256..4954
                     /locus_tag="wEsol_00629"
     CDS             4256..4954
                     /locus_tag="wEsol_00629"
                     /codon_start=1
                     /transl_table=11
                     /fullproduct="qcoverage 100 , hcoverage 100 , similarity
                     100 , identity 99.6 , evalue 1.11e-164 , alnlength 232 ,
                     Bacteria , Proteobacteria , WP_151808056.1 MULTISPECIES:
                     alpha/beta hydrolase [Wolbachia]"
                     /cog="COG2945 COG2945 210 Predicted hydrolase of the
                     alpha/beta superfamily"
                     /pfam="Abhydrolase_5 Abhydrolase_6 Peptidase_S15
                     Peptidase_S9 Abhydrolase_1"
                     /product="alpha/beta
                     hydrolase"
                     /translation="MIEVFLNNATRKIEGEYHQSKDANAPVVLVLHHHPQYGGNMDSK
                     IVHSTYTSFIDNNFSALKINFRGVGKSTGTFDKGIGELTDAAVAIDWLQEHNPSNVPI
                     WIAGFSFGAWVAMQLTMRRPEIVGFIALSLPVTKYDFSFLSPCPVSGLIIQSSNDTIS
                     EESDVTELAKRLINSVRSDHMKYHIIDDTNHFLKDKEEEVTQIVDNYIKLRLNSATIS
                     FQKVKKEVRVKEYA"
                     /besthit="qcoverage 100 , hcoverage 100 , similarity 100
                     , identity 99.6 , evalue 1.11e-164 , alnlength 232 ,
                     Bacteria , Proteobacteria , WP_151808056.1 MULTISPECIES:
                     alpha/beta hydrolase [Wolbachia]"
     gene            5007..5171
                     /locus_tag="wEsol_00630"
     CDS             5007..5171
                     /locus_tag="wEsol_00630"
                     /codon_start=1
                     /transl_table=11
                     /pfam="LPAM_1 Pfam-B_7772"
                     /fullproduct="qcoverage 100 , hcoverage 100 , similarity
                     100 , identity 100 , evalue 7.44e-31 , alnlength 54 ,
                     Bacteria , Proteobacteria , WP_015589158.1 MULTISPECIES:
                     lipoprotein [Wolbachia]"
                     /translation="MFKKLLFFVLAVFMLSGCSLSKQRKLKSPCIKSNESISCELYSV
```

```
                     NDHWLDKYKA"
                     /product="lipoprotein"
                     /besthit="qcoverage 100 , hcoverage 100 , similarity 100
                     , identity 100 , evalue 7.44e-31 , alnlength 54 ,
                     Bacteria , Proteobacteria , WP_015589158.1 MULTISPECIES:
                     lipoprotein [Wolbachia]"
     gene            complement(5193..5867)
                     /locus_tag="wEsol_00631"
     CDS             complement(5193..5867)
                     /locus_tag="wEsol_00631"
                     /codon_start=1
                     /transl_table=11
                     /fullproduct="qcoverage 100 , hcoverage 100 , similarity
                     100 , identity 100 , evalue 9.03e-151 , alnlength 224 ,
                     Bacteria , Proteobacteria , WP_015589159.1 MULTISPECIES:
                     ABC transporter ATP-binding protein [unclassified
                     Wolbachia]"
                     /cog="SalX COG1136 226 ABC-type antimicrobial peptide
                     transport system, ATPase component"
                     /pfam="DUF258 AAA_15 AAA_29 Pfam-B_16450 AAA_22 AAA_21
                     ABC_tran Pfam-B_3333 SMC_N"
                     /tigr="TIGR02211 LolD_lipo_ex 221 lipoprotein releasing
                     system, ATP-binding protein"
                     /product="ABC transporter
                     ATP-binding protein"
                     /translation="MGGNVALELTSVDKSFKDNSAVVKDINLSVTRGQVVALIGNSGA
                     GKTTILQIAGLLDKPTSGIVTIDGINCTQASNKHKTHVRRNFLSFVYQFHYLLQELSV
                     LENIMLPQLIAGKSKAEAKKNSQAMLEKFGLENKASSMVSEVSGGERQRVAIARSIVN
                     SPKLLLADEPTGNLDPTNSFKVFLLLHSYVKENNSSMLIVTHNHLLAEKADCIFQLRD
                     RSLVKL"
                     /besthit="qcoverage 100 , hcoverage 100 , similarity 100
                     , identity 100 , evalue 9.03e-151 , alnlength 224 ,
                     Bacteria , Proteobacteria , WP_015589159.1 MULTISPECIES:
                     ABC transporter ATP-binding protein [unclassified
                     Wolbachia]"
     gene            complement(5907..6368)
                     /locus_tag="wEsol_00632"
     CDS             complement(5907..6368)
                     /locus_tag="wEsol_00632"
                     /codon_start=1
                     /transl_table=11
                     /fullproduct="qcoverage 100 , hcoverage 100 , similarity
                     100 , identity 100 , evalue 8.07e-92 , alnlength 153 ,
                     Bacteria , Proteobacteria , WP_141456915.1 MULTISPECIES:
                     heme biosynthesis protein HemY [Wolbachia]"
                     /cog="sufA COG0316 110 Fe-S cluster assembly scaffold
                     protein"
                     /pfam="Fe-S_biosyn Pfam-B_8392 Pfam-B_458 Fe-S_biosyn"
                     /product="heme biosynthesis
                     protein HemY"
                     /translation="MSTNYNINLTDNALKKIHSLAEQEGDKSSILRVAVSGGGCSGFK
                     YNFLMDQMNKNLSLDDEDDDYDDEFDDEDEDDDYEESEDYRSHSSFSKKGKDIVINDE
                     NGNPVLMVDNCSAKFLNNSVIDYTEDLSGSGFQIKNAFAKSQCGCGNSFSV"
                     /besthit="qcoverage 100 , hcoverage 100 , similarity 100
                     , identity 100 , evalue 8.07e-92 , alnlength 153 ,
                     Bacteria , Proteobacteria , WP_141456915.1 MULTISPECIES:
                     heme biosynthesis protein HemY [Wolbachia]"
     gene            6416..7615
                     /locus_tag="wEsol_00633"
     CDS             6416..7615
```

```
                     /locus_tag="wEsol_00633"
                     /codon_start=1
                     /transl_table=11
                     /fullproduct="qcoverage 100 , hcoverage 100 , similarity
                     100 , identity 100 , evalue 7.35e-288 , alnlength 399 ,
                     Bacteria , Proteobacteria , WP_065106726.1 MULTISPECIES:
                     deoxyguanosinetriphosphate triphosphohydrolase
                     [Wolbachia]"
                     /cog="Dgt COG0232 412 dGTP triphosphohydrolase"
                     /pfam="Pfam-B_1409 HDOD HD HD_assoc"
                     /tigr="TIGR01353 dGTP_triPase 389 putative dGTPase"
                     /product="
                     deoxyguanosinetriphosphate triphosphohydrolase"
                     /translation="MSNNNFLLNYACFPSKTKGRYFKEPEDENRSCFQRDRDRIIHSN
                     AFRKLEYKTQVFINYEHDYYRTRLTHSLEVAQIARSIARRLGLNEDITECIALAHDLG
                     HPPFGHAGEDALKKSVQDLNLDNEKYEFDHNVQAIRILTYLEQKHADFDGMNLSWEVI
                     EGVAKHNGPLLGQNAESSTSNQLLLEYNKKYDLKLEEFSSIEAQVASIADDIAYSVHD
                     LDDALRANLVTIEDLLNVPLIEKMFKDIRSRYSELPQSKLIHESLSGTIGTMISDVVS
                     QTERNIEDHKIKSVEDVRSLNKMLVTFSPEVANATKEMKRFNMEKIYRSYKLSRTMNK
                     AKRIIQELFQCFYENPGLLPTEWSKLACESQRSVIICDYISGMTDRFAIHEHRRIFDT
                     SYEMTSF"
                     /besthit="qcoverage 100 , hcoverage 100 , similarity 100
                     , identity 100 , evalue 7.35e-288 , alnlength 399 ,
                     Bacteria , Proteobacteria , WP_065106726.1 MULTISPECIES:
                     deoxyguanosinetriphosphate triphosphohydrolase
                     [Wolbachia]"
     gene            7615..8697
                     /locus_tag="wEsol_00634"
     CDS             7615..8697
                     /locus_tag="wEsol_00634"
                     /codon_start=1
                     /transl_table=11
                     /fullproduct="qcoverage 100 , hcoverage 100 , similarity
                     100 , identity 100 , evalue 1.30e-259 , alnlength 360 ,
                     Bacteria , Proteobacteria , WP_015589162.1 MULTISPECIES:
                     bifunctional diaminohydroxyphosphoribosylaminopyrimidine
                     deaminase/5-amino-6-(5-phosphoribosylamino)uracil
                     reductase RibD [unclassified Wolbachia]"
                     /pfam="dCMP_cyt_deam_1 RibD_C"
                     /tigr="TIGR00326 eubact_ribD 343 riboflavin biosynthesis
                     protein RibD"
                     /product="bifunctional
                     diaminohydroxyphosphoribosylaminopyrimidine
                     deaminase/5-amino-6-(5-phosphoribosylamino)uracil
                     reductase RibD"
                     /translation="MTDDHFMSIALRLAEKNLGNVAPNPAVGCVIVKDGTIVSEGYTG
                     IGGRPHAEVVALQNAKDSTHGATIYITLEPCCHHGVTGPCTAKIIKASIKRVVIATID
                     PDSRVSGRGMKALKEAGIEVEQGIMQKEAKELNVGFFTTKELHRPFIACKVATTLDGK
                     IATFTGDSKWITSENTRNWVHELRAKYDAIMIGSNTLINDDPLLTCRLPGLGSRSPIR
                     LIIDSQGRLKEEHNIAKTADKVITWVITNKEVEKKIKNINYLVVNSSNAGKVCLKDMA
                     LKLVSEIGITRLLVEGGGVLITELLKCNLIDRLIICRSGKILGNDATPFVGDLGIQFI
                     NNCYQFKKTKIIEFSEDIVEVWDRSS"
                     /besthit="qcoverage 100 , hcoverage 100 , similarity 100
                     , identity 100 , evalue 1.30e-259 , alnlength 360 ,
                     Bacteria , Proteobacteria , WP_015589162.1 MULTISPECIES:
                     bifunctional diaminohydroxyphosphoribosylaminopyrimidine
                     deaminase/5-amino-6-(5-phosphoribosylamino)uracil
                     reductase RibD [unclassified Wolbachia]"
     gene            8810..9190
                     /locus_tag="wEsol_00635"
```

```
CDS             8810..9190
                /locus_tag="wEsol_00635"
                /codon_start=1
                /transl_table=11
                /pfam="DUF972 Pfam-B_6733 CASP_C"
                /fullproduct="qcoverage 100 , hcoverage 100 , similarity
                100 , identity 100 , evalue 6.99e-55 , alnlength 126 ,
                Bacteria , Proteobacteria , ONI56251.1 transposase
                [Wolbachia pipientis wUni]"
                /translation="MSDLLASLLKLCESFKIEIEQLKAEIKRLEIENENFRSENKALR
                IENAELKERLGLNSQNSSIPSSKELYKLKKKKKKSDRKIGAQIGHEGKYRPKMEADEV
                VKIEFLRVRRRNCDIERAIHSSKS"
                /product="transposase"
                /besthit="qcoverage 100 , hcoverage 100 , similarity 100
                , identity 100 , evalue 6.99e-55 , alnlength 126 ,
                Bacteria , Proteobacteria , ONI56251.1 transposase
                [Wolbachia pipientis wUni]"
gene            9202..9672
                /locus_tag="wEsol_00636"
CDS             9202..9672
                /locus_tag="wEsol_00636"
                /codon_start=1
                /transl_table=11
                /pfam="DDE_Tnp_IS66"
                /fullproduct="qcoverage 100 , hcoverage 100 , similarity
                99.4 , identity 99.4 , evalue 1.15e-106 , alnlength 156 ,
                Bacteria , Proteobacteria , CDR79089.1
                transposase,Transposase IS66 family [Wolbachia
                endosymbiont of Drosophila simulans wAu]"
                /translation="MLWNTNGRCCKCGKRKSSRLPEGVTADTFGPKVKSTIAALSGFY
                KNSKREVANIIKNIFNLDISVGSVSNSEARVAEKCQEAYEQIEQEVSKSEVLHIDETS
                HYNKGKLGWCWMFASNTASFIKLTESRGMKVLQNSKFCNRNSLRQVRSIQLLCR"
                /product="transposase,Transposase IS66 family"
                /besthit="qcoverage 100 , hcoverage 100 , similarity 99.4
                , identity 99.4 , evalue 1.15e-106 , alnlength 156 ,
                Bacteria , Proteobacteria , CDR79089.1
                transposase,Transposase IS66 family [Wolbachia
                endosymbiont of Drosophila simulans wAu]"
gene            complement(10292..10972)
                /locus_tag="wEsol_00637"
CDS             complement(10292..10972)
                /locus_tag="wEsol_00637"
                /codon_start=1
                /transl_table=11
                /fullproduct="qcoverage 100 , hcoverage 100 , similarity
                100 , identity 100 , evalue 2.81e-155 , alnlength 226 ,
                Bacteria , Proteobacteria , WP_015589163.1 MULTISPECIES:
                ribulose-phosphate 3-epimerase [unclassified Wolbachia]"
                /cog="Rpe COG0036 220 Pentose-5-phosphate-3-epimerase"
                /pfam="Ribul_P_3_epim NanE OMPdecase QRPTase_C
                His_biosynth PcrB"
                /tigr="TIGR01163 rpe 211 ribulose-phosphate 3-epimerase"
                /product="ribulose-phosphate
                3-epimerase"
                /translation="MSIKIAPSILSADFAKLGEEVRKISDLGVDYIHIDVMDGNFVPN
                ITIGPSVVSAIRKYSNLPFDVHLMVKSPGNHIESFINAGADIITVHAEAEIHLERLIR
                KIKSYKNVNDAKKPIQAGVSIVPSTSPSVLEYIIHELDIVLIMTVNPGFGGQEFIHSQ
                LNKISTIKKMIQERNLKTQISVDGGINFSNAADIIKAGADILVAGSAIFKAEDIKKAV
                NDLKNPSL"
                /besthit="qcoverage 100 , hcoverage 100 , similarity 100
```

```
                , identity 100 , evalue 2.81e-155 , alnlength 226 ,
                Bacteria , Proteobacteria , WP_015589163.1 MULTISPECIES:
                ribulose-phosphate 3-epimerase [unclassified Wolbachia]"
gene            complement(11075..11503)
                /locus_tag="wEsol_00638"
CDS             complement(11075..11503)
                /locus_tag="wEsol_00638"
                /codon_start=1
                /transl_table=11
                /pfam="zinc_ribbon_5 zinc_ribbon_4"
                /fullproduct="qcoverage 100 , hcoverage 100 , similarity
                100 , identity 100 , evalue 1.63e-97 , alnlength 142 ,
                Bacteria , Proteobacteria , WP_010962781.1 MULTISPECIES:
                zinc-ribbon domain-containing protein [Wolbachia]"
                /translation="MKIQCNSCTKTYLVSPKQIGASGRKVKCTNCNHIWHEYLEEVSN
                KSHSANIQEKKVSGRNFLQNLAFTTLAFATATGLCVVIANGIFPREMNKAYKTISLYK
                DSISYKLGYKKEQTESPNVKKLAVNKFYQDYLFLSNLRSS"
                /product="zinc-ribbon
                domain-containing protein"
                /besthit="qcoverage 100 , hcoverage 100 , similarity 100
                , identity 100 , evalue 1.63e-97 , alnlength 142 ,
                Bacteria , Proteobacteria , WP_010962781.1 MULTISPECIES:
                zinc-ribbon domain-containing protein [Wolbachia]"
gene            11577..12407
                /locus_tag="wEsol_00639"
CDS             11577..12407
                /locus_tag="wEsol_00639"
                /codon_start=1
                /transl_table=11
                /fullproduct="qcoverage 100 , hcoverage 100 , similarity
                99.6 , identity 99.6 , evalue 3.09e-177 , alnlength 276 ,
                Bacteria , Proteobacteria , WP_015589164.1 MULTISPECIES:
                2,3,4,5-tetrahydropyridine-2,6-dicarboxylate
                N-succinyltransferase [unclassified Wolbachia]"
                /cog="DapD COG2171 271 Tetrahydrodipicolinate
                N-succinyltransferase"
                /pfam="Pfam-B_10572 Pfam-B_54 Hexapep Hexapep"
                /tigr="TIGR00965 dapD 271
                2,3,4,5-tetrahydropyridine-2,6-dicarboxylate
                N-succinyltransferase"
                /product="
                2,3,4,5-tetrahydropyridine-2,6-dicarboxylate
                N-succinyltransferase"
                /translation="MEGLQLKKTQSEIEDIWKNREKFNDCNLKKTARIAIKEVIELLD
                SGKIRVAEKLSSGEWVVHKWIKQAILLHFLTEENKIIDNTNCWFDKIGNKFSEWNEEK
                FRQLKIRAVPGCFVRRSAYIGTNVVLMPSFINVGAYVDSGTMIDTWSTIGSCAQIGKN
                CHISGGVGIGGVLEPIQASPVIIEDNCFIGARSEVAEGVVVREGSVLGIGVFIGASTK
                IIDRETSKVFCGEVPPYSVVVPGSIPSKNNISTYCAVIVKKVDEKTRSKISINEILRD
                "
                /besthit="qcoverage 100 , hcoverage 100 , similarity 99.6
                , identity 99.6 , evalue 3.09e-177 , alnlength 276 ,
                Bacteria , Proteobacteria , WP_015589164.1 MULTISPECIES:
                2,3,4,5-tetrahydropyridine-2,6-dicarboxylate
                N-succinyltransferase [unclassified Wolbachia]"
gene            complement(12414..12698)
                /locus_tag="wEsol_00640"
CDS             complement(12414..12698)
                /locus_tag="wEsol_00640"
                /codon_start=1
                /transl_table=11
```

```
                     /pfam="DUF2610"
                     /fullproduct="qcoverage 100 , hcoverage 100 , similarity
                     98.9 , identity 98.9 , evalue 1.29e-60 , alnlength 94 ,
                     Bacteria , Proteobacteria , WP_010962783.1 MULTISPECIES:
                     DUF2610 domain-containing protein [Wolbachia]"
                     /translation="MAESIKKFTVQCDFKGQSLPFAIYIGNPKSDAHPIHHQDSWLVK
                     ERGGNIPNKVKESLQKLYKLSQENGVSFSELCAYAITVVSNNDKKSDDKE"
                     /product="DUF2610
                     domain-containing protein"
                     /besthit="qcoverage 100 , hcoverage 100 , similarity 98.9
                     , identity 98.9 , evalue 1.29e-60 , alnlength 94 ,
                     Bacteria , Proteobacteria , WP_010962783.1 MULTISPECIES:
                     DUF2610 domain-containing protein [Wolbachia]"
     gene            complement(12699..13070)
                     /locus_tag="wEsol_00641"
     CDS             complement(12699..13070)
                     /locus_tag="wEsol_00641"
                     /codon_start=1
                     /transl_table=11
                     /fullproduct="qcoverage 100 , hcoverage 100 , similarity
                     100 , identity 100 , evalue 1.22e-74 , alnlength 123 ,
                     Bacteria , Proteobacteria , WP_041573529.1 MULTISPECIES:
                     hypothetical protein [unclassified Wolbachia]"
                     /translation="MDDKNTDYVVLIAQDDSIEYKFEEAVNFIENKVINADGLTLAEV
                     FQVLVQMLDGDLALVKKVLAYANIMAKHGMKVAKESQLSRADVRRALEGRESVVNRQD
                     FIKKPPLPPAIKSTKKKSRGL"
                     /product="hypothetical protein"
                     /besthit="qcoverage 100 , hcoverage 100 , similarity 100
                     , identity 100 , evalue 1.22e-74 , alnlength 123 ,
                     Bacteria , Proteobacteria , WP_041573529.1 MULTISPECIES:
                     hypothetical protein [unclassified Wolbachia]"
     gene            13172..14221
                     /locus_tag="wEsol_00642"
     CDS             13172..14221
                     /locus_tag="wEsol_00642"
                     /codon_start=1
                     /transl_table=11
                     /fullproduct="qcoverage 100 , hcoverage 100 , similarity
                     100 , identity 100 , evalue 6.89e-254 , alnlength 349 ,
                     Bacteria , Proteobacteria , WP_015589166.1 MULTISPECIES:
                     SAM-dependent methyltransferase [unclassified Wolbachia]"
                     /cog="COG1565 COG1565 370 Uncharacterized conserved
                     protein"
                     /pfam="Methyltransf_28"
                     /product="SAM-dependent
                     methyltransferase"
                     /translation="MLTYIHELIDKSQGSISISDFMNAVLYHEKYGYYTSKLPLGKDG
                     DFTTAPEISQLFGEVIAVWIMHTWEKLGKPSKFSLVELGPGKGTLIHDIIRVTKKYSS
                     FFNSMLIHLVEISPTLRKIQKEKLKSLDVNWHKNIDNLPEQPTIFLANEFFDALPVDQ
                     FVYRDGQWYENMVTKQDDGSLLVSCQCVTLESRKKESWIPVSATQMTNGKFFNGAVVE
                     ICSVGVEILKKLEKKIYNNKGAALIVDYGYVYPAYKSTLQSIKQHKYANFLENVGNSD
                     ITALVNFQALRDSLKHVDCEILTQREFLYLFGIKERTQALMKSASDEQKNRIFSEFLR
                     LTENMGTLFKAMLLI"
                     /besthit="qcoverage 100 , hcoverage 100 , similarity 100
                     , identity 100 , evalue 6.89e-254 , alnlength 349 ,
                     Bacteria , Proteobacteria , WP_015589166.1 MULTISPECIES:
                     SAM-dependent methyltransferase [unclassified Wolbachia]"
     gene            complement(14462..15913)
                     /locus_tag="wEsol_00643"
     CDS             complement(14462..15913)
```

/locus_tag="wEsol_00643"
/codon_start=1
/transl_table=11
/pfam="Terminase_6"
/fullproduct="qcoverage 100 , hcoverage 100 , similarity 99.6 , identity 99.2 , evalue 0.0 , alnlength 483 , Bacteria , Proteobacteria , WP_141457085.1 phage terminase large subunit [Wolbachia endosymbiont of Carposina sasakii]"
/translation="MNFLKFIELCFQTVVPGCEYNDYQYIKVIADRLEAASAGEVKRI
IFNMPPRSMKSMCISVAWPAWILGNQPTARIIVASYSQRLSEKHSLDTRCVMQSSWYR
ALFPEIELSKDQNTKYKFQTVQRGYRIATSVGGTLTGEGGDFIIVDDPLSSAQALSET
LRKRAINWFDQTLVTRLNDRKKGVIVLVMHRLHQEDLTGHLLSKPKNIWHHICLPMIS
ENKQVIYSIKKPMPPVPVIRVAQHCATRRLYNKPASPVRILYSREESQLLYPLDGGKE
EIEMIKVELGSYAFAAQYQQNPLPLSSGIIKPEWLKRYKKFPDNLSHITQSWDTAVST
SDMSNFSVCTTWAKIDNKFYLLDVYRAKLEYPKLKEQVLSLAARWSPHAILIEAKTSG
QQLAQELKANSHLPIIEIVPHDGKLARFHRIAPMIESGRIILPHQAVWLNDFEYEILM
FPETRHDDQVDSTVQYLQWVRNSTSRVAAIRAL"
/product="phage terminase large subunit"
/besthit="qcoverage 100 , hcoverage 100 , similarity 99.6 , identity 99.2 , evalue 0.0 , alnlength 483 , Bacteria , Proteobacteria , WP_141457085.1 phage terminase large subunit [Wolbachia endosymbiont of Carposina sasakii]"

gene complement(16896..18470)
/locus_tag="wEsol_00644"

CDS complement(16896..18470)
/locus_tag="wEsol_00644"
/codon_start=1
/transl_table=11
/fullproduct="qcoverage 100 , hcoverage 100 , similarity 99.8 , identity 99.8 , evalue 0.0 , alnlength 524 , Bacteria , Proteobacteria , WP_015589167.1 MULTISPECIES: penicillin-binding protein [unclassified Wolbachia]"
/cog="FtsI COG0768 599 Cell division protein FtsI/penicillin-binding protein 2"
/pfam="PBP_dimer PBP_dimer Transpeptidase"
/tigr="TIGR03423 pbp2_mrdA 593 penicillin-binding protein 2"
/product="penicillin-binding protein"
/translation="MWTKNKVFNRRAFILGGIQLTISAVFSCRLYNLQIRNRQKYEAL
SNSNRIRVATIMPKRGKILDRNSIELAVNKISYVVLFDGSGKEVDLQTLSEVESKIAK
SSEKITALYKRYYPFGSMCSHVIGYTKRQQGISEVGISGIEYTYDHILKGKSGKSEQE
INSKKRVIKELSSIPQQDGQDVQLTIEINLQEKTAEVFKDHQGSAVVIDVNNGEILAL
YNSPSYDNNLFASRLSNETWENLNAPSLPLVNRALSYQIPPGSIFKVIVALAGLKDGI
ITSEEKFSCKGYMKIGERKFRCLKSKVHGYVSLNEAMALSCNTYFYNIGKKISVDSLV
EMARKFGIGSGPLIGTFKEEAPGLLPDRDWRTRKLYSQWYLGDTINLVIGQGYMLTTP
LQLAVLAARIATGKEVIPHIEMSKTIQDFPDIDVDCEHLSIVRKAMFDVVNSKTGTYK
KGLSGIQIAGKTGTPEINSKGESHKLFIAYGPYHNPRYAISVFIEHGKAPRQDVAIAN
EIFQYMLETMSIKLLA"
/besthit="qcoverage 100 , hcoverage 100 , similarity 99.8 , identity 99.8 , evalue 0.0 , alnlength 524 , Bacteria , Proteobacteria , WP_015589167.1 MULTISPECIES: penicillin-binding protein [unclassified Wolbachia]"

gene complement(18425..19264)
/locus_tag="wEsol_00645"

CDS complement(18425..19264)
/locus_tag="wEsol_00645"
/codon_start=1
/transl_table=11

```
                     /fullproduct="qcoverage 100 , hcoverage 100 , similarity
                     100 , identity 100 , evalue 5.32e-200 , alnlength 279 ,
                     Bacteria , Proteobacteria , WP_015589168.1 MULTISPECIES:
                     polysaccharide deacetylase family protein [unclassified
                     Wolbachia]"
                     /cog="CDA1 COG0726 267 Predicted xylanase/chitin
                     deacetylase"
                     /pfam="Polysacc_deac_1"
                     /product="polysaccharide
                     deacetylase family protein"
                     /translation="MFIRIITFFLLYSSTAFCSDCNFNLPYCGLSCNLDLSYRNLSNI
                     KKLLNNDDKFVALTFDDGPSNNRINDIINVLESYEAKATFFVLGERINEKTSEIVKKI
                     HESGHELGNHSWSHRKLTSLSSEEQLQELEKTNTAIKNAIKQDVKWFRPPYGCYDDNL
                     IENTDQLNMYSILWTVDSLDWQGDKPETLVERVLSNVHNGAVILFHDHNNRSNTIEAL
                     PHIIKILKKSDYKFVTLSEWEKRVCNTVKARSVTIKEGKKVCFEGMLCGQKTKSLTAE
                     HSY"
                     /besthit="qcoverage 100 , hcoverage 100 , similarity 100
                     , identity 100 , evalue 5.32e-200 , alnlength 279 ,
                     Bacteria , Proteobacteria , WP_015589168.1 MULTISPECIES:
                     polysaccharide deacetylase family protein [unclassified
                     Wolbachia]"
ORIGIN
        1 tatgtttaag tttatgtgtt gttttaattt ttctctcaag aaattatttg ctgcaactcc
       61 gccggtaatc acaaagtcat tgattttgat atttaaagat tcggccataa taatcgcatt
      121 actaaccctg tccagcagta tgtcactaat acactcttga aacgaagcac atacatcaca
      181 cacatcttgc tcgctcattt taagttcttg tactaagttt tttactgctg ttttaatccc
      241 agaaaacgaa aagttacatc cagaacgttt tatcattgcc cttggcagtt tgaatcttgt
      301 gccattgcct tttttagcca acttttcaat taatgggcct cctgggtagc ttaaacccag
      361 catcttagca actttatcaa atgcttctcc tagtgaatca tcgagcgttt ctccaagttt
      421 aatgtattta cctacatcct gtgcaattaa aaattgacag tgaccacctg atattagtag
      481 gactaaaaat ggaaatttta cctcatgcag taacctaata accaatgcat gcgcttctaa
      541 gtgattcact gcaatgaacg gtttttgtgc tacatgcgca attgctttag ccatcattgt
      601 accaactatt aatccaccta tgagtcctgg tcctgatgtt gctgcaattg cattgaggtc
      661 acaaaagtta aggttagatt tttccacggc gctttttatt agaccactta aatgcttcat
      721 atgagcacgt gaagctattt cagggatcac cccaccgcgt tttttgtgtt ctacttgaga
      781 aagaatcttg tgagcaagga cttgcttgtc actatttaca atcgctactg cagtttcgtc
      841 gcagcttgtt tcaacagcta aaatggtttt catgtatatt tagttactgt attaccaaaa
      901 ctatactttg aaacgctata gaagttaact gcaaagttaa cttctatagc taaaagattt
      961 accgacaacc gtcattccgc tacgtgttag ctgatgagat accgcgcaca actgtacgaa
     1021 cattcattct taaaggtaaa ctgcacagca aatggtgtca ttccagtgct tgacactgga
     1081 atccagcctt gccgtaatct catcaagaac gttgtgtttt aacataagat cagccacttt
     1141 tatgctcacc aacttagtgc ccaatccaaa ttcctggatt ccagtgtcaa gcactggaat
     1201 gacaccatct actactcaaa ttacctgcaa attgcaatgt tcgtacacca aaagaatgaa
     1261 tcgcggtatg acggttcgcg gcggcatgac gtaggaaaac ctgcactata agctaatcat
     1321 catctccaaa tatagagcta atttttttgc ccccaggaga aaatttatta gtccaactgc
     1381 tttgcacttt ggttacgtct ttagtggcag ttttattgct gtattccttg atttccttat
     1441 ctattctctc catcatttgt gaatcaaggt agctcttttt ccattggcca gtagcaagga
     1501 acactttgtt taagtcttga gtgcggatta agtattcttt atctttaggg taaacctcct
     1561 gcgcccaagc aaatagctca aaatagcgtg caaataattc agatgcacgt gattttgcag
     1621 tttgataagc ctgtatctcc ttaaatataa cttggccgat aattttctgc acaagaatat
     1681 ttgcttgctt taggttctgc tcaatatcct ggtaaacttt atggagaaat tcctgttcca
     1741 aacttaagtt cagttcattt tcaaccatgt gtgaaagttc atgcactata atgtatgggt
     1801 ttgacttcct cagtacaata acatatctat ttacactgga agacctgatt gtcttacaca
     1861 ctccctcttc catatccact aatccctctt ggtatatttc aaatgagagc ctgttttgct
     1921 ttattagtga taaagtgagg tttaatgtgt ttctaaactt ctcaaattga tagagatatt
     1981 cgacataccg tattgcattt tcactgcttc ctacttgagt tgcttgtttt attaaattcc
     2041 tgattagcat accttattat taaaggaatt ctttttatac tcaagaattg attggctaca
     2101 aagtgttttt aggaagcctg attgcatagc taaagtaatt catgtttact gataaccgtc
     2161 ataccgctgc ttgtcagcgg gatctatggt gagataccgt gaatgaatcc atagctgtac
     2221 gaacattcat ttttgaaggt aaattgcaca gcaaatggtg tcattccagt gctcttttt
```

2281 tgtcatccca gtctgggatc tattttgcat gtaactccaa ttgtgttttg gcataaaacg
2341 cgacacgtat tagacttatt tgcaagcaaa gcttgctaga tccaagtagt cagggcactg
2401 ggatgacatt acaggggcac tgaaatgaca ccattttcgg tctcttaaat tgctttagcc
2461 ataaatattt aagaaattta ctaaataaaa aaaaggcaaa agaagccccg tggtggttgg
2521 ctatttacta tgtactaaaa tatcggcgtt tttttattc taaaacgctt gattaagagc
2581 gatttagctg cttttaactt gcaactaacc tacaccgcaa gtgtttaaga aatttactaa
2641 gcaggaaaaa aggcaaagaa accccgtagt agctagcatt caaattctcc cttgtcaatt
2701 tgacgttttt tgctgtctta aacgctttgt aagcgcgttt cggcttatat aggtaaaaac
2761 ctaaaaattt ttaaagacat gcgatgcacg tagtgcgaaa aattaaacat gagacgccaa
2821 ataccctaag tttttgtca ttaacctgca cagattgcga agataaataa atagcttcac
2881 tgttatgata agggggctgg cgaaacttgt aaaggaattt tttacatcta atttatagct
2941 ccctgcatga tatcacgtat tatctatacc aaggagttct gcttgtagat attataccaa
3001 caatccacta ttttttttat gtcttgtggt ttagtttctg gacctatact gattctaatt
3061 gaacactctg tttgctcttt tgttgccccc attgcaagca aaacatggga aggctcaact
3121 tttccagaag aacatgcgga gccattacta actgcaatat aattcaagtc aaaatgcatg
3181 agctgcacat cactctttac tcctggcata taaataaaac ttgtgtttgg cagcctctta
3241 gagtttttac cgaagattct tatgtcactg acaagattta ataattcaca ctctaattga
3301 tcgcgtagct cctttacttc atccattttt gatagaagat ctggaatatt ttgcagtgca
3361 gcagaaaggc ctgcaatcgc aacaatattt tccgtaccac cacgaaatcc cttctcttgt
3421 ccaccaccta ttataatagg ttctatcgca agttctttat tgaatattaa aactccactg
3481 cctgctacac cgccaaattt atgggcggat aaagtgagta aatccactcc taaatcttcc
3541 atattaactt taattttccc aacactttga gcagtgtcag tgtggcaaat tgctccaaat
3601 ttgtgggcta tttcagctat ttcctcaata ggttgaataa ctccggtttc gttatttgcc
3661 atcataactg aaactattgc tttgtttcct ttaagttcgc ttagaatttt ttctagctct
3721 aaaaagtcaa caatgccctc ctgattaacg ggtattatgt atggattaca tgcagaatta
3781 agaattgaag gatgctctat agctgaaatt acatgcaggt agcctgctat tcctctcata
3841 acaaggttat tcgcttcggt tgcaccagac gtgaaaacta tttctttatc acttgagacg
3901 ccaatagcac cacgtacgtt atctcttgca tcctgaagaa tcttcctcgc ttcttgtcct
3961 cttttatgta gtgatgatgg attgagcgtt tgttttaaca agacctcaaa tatacttttt
4021 tttacatttt cactaattgg agcagttgca ttgtaatcag catatacgca accactattt
4081 tctaaagaga atggactcat ctgtcactta aataagttta ccatcaacta tagccataac
4141 tatgtacgtt agctttaaca cgataaaaaa tttattgcaa aaacagcgaa attaatacat
4201 aattaataac tcacgtcatt aatacctgtc aagcgggttc ttattagggg ttaccatgat
4261 tgaagttttt ttgaataatg caacaagaaa gatagaaggt gaataccacc aaagcaaaga
4321 tgccaacgcg ccggttgtgc tggttttaca tcatcatcct caatatggtg gtaatatgga
4381 tagtaaaatt gtacatagta catatacgtc ttttatcgat aacaattttt ctgcattgaa
4441 gattaacttt cgtggtgtgg gaaaatctac cggaactttt gataagggta taggagaatt
4501 aactgacgct gcggtagcca ttgattggct tcaggaacat aatcctagca atgttccaat
4561 ttggatcgct ggtttttctt ttggagcatg ggtggctatg cagctaacaa tgcgccgccc
4621 tgagatagtg ggttttattg ctctttctct tccggtaact aagtacgatt tttcttttct
4681 ttctccctgt ccagtttctg ggcttataat acaaagcagt aatgatacaa tctcagaaga
4741 aagcgatgta acagaattag caaaaaggtt gataaattca gtaagaagtg atcacatgaa
4801 ataccatata atagatgata ctaatcactt tctaaaggat aaagaagagg aagtgactca
4861 aatcgtggac aattatataa aactgcgctt gaatagtgca actatttctt ttcaaaaggt
4921 caaaaaagag gtaagggtaa aagaatatgc ctaatcttta caaacaagag aaatgagtgt
4981 agtaatataa atgaaaaggt tttgttatgt ttaaaaagct attatttttt gttttagctg
5041 tgtttatgtt atcggggtgt tccctaagta aacagcgcaa gttaaaaagt ccgtgtataa
5101 aaagcaatga aagcatttca tgcgagttgt actctgttaa tgatcattgg ttagataagt
5161 ataaggcata gaaattgtgc tatttttcct attcacaact tcactaatga tcgatctctc
5221 aattggaaaa tgcagtctgc cttttctgca aggagatgat tgtgtgttac tataagcata
5281 gagctgttat tttcctttac gtatgaatgt agcagtaaaa acaccttaaa agaatttgtt
5341 ggatctaaat ttcctgttgg ctcatctgca agtaaaagct ttggagaatt tacaatgctt
5401 cttgcaattg caactctctg cctttcccca cccgaaactt cagataccat gctacttgct
5461 ttgttttcca gaccaaattt ttccaacatt gcttgcgaat ttttttttgc ttcagctttg
5521 ctttttcctg caatgagttg aggaagcata atattttcca acaccgataa ctcttgtaac
5581 aaatagtgaa attgataaac aaaactaaga aaatttcttc ttacatgagt tttatgctta
5641 ttactggctt gtgtgcaatt tattccatct atcgtaacta tacctgaagt tggcttgtcc
5701 aacaagcctg cgatttgcaa tatagttgtt ttccctgcac ctgaattgcc aatcagcgct
5761 actacttgcc ctcttgtaac acttaaattg atatccttta caacagcaga attgtccttg
5821 aagcttttat ctacagaagt tagttctagt gctacattac cacccatgtt taaaagatcc

5881 agaaggcatc taagttttag taataattaa accgaaaaac tgttaccaca accacattga
5941 gacttagcaa aggcattctt tatttgaaaa ccagaaccac tgagatcctc agtataatct
6001 ataactgagt tatttaaaaa ttttgctgaa caattatcaa ccattaatac agggtttcca
6061 ttttcatcat taattactat atctttacct tttttgctaa aactggagtg gcttctatag
6121 tcctcacttt cttcatagtc atcatcttcg tcttcgtcat caaactcatc atcgtaatca
6181 tcgtcttcat catccaaaga tagattttta ttcatttgat ccataagaaa attgtatttg
6241 aagccagaac atccaccacc tgaaactgca acccgtaaaa tggaactctt atccccttcc
6301 tgctctgcaa gagagtggat ttttttagt gcattatcag ttaagttgat gttgtaattt
6361 gttgacatag taaccaccgt ttattattga taacttatta tagtagaaat tttacatgtc
6421 aaacaataat tttctattaa attatgcatg ttttccaagc aaaacaaaag ggagatactt
6481 caaagagcca gaagatgaga atcgcagctg ctttcagcgt gatagggacc gtatcattca
6541 ctctaatgcg tttagaaagt tggagtacaa aacacaagtt tttatcaatt atgagcacga
6601 ctactatcgc actcggctta ctcatagcct tgaagttgca caaattgcaa ggtccattgc
6661 gcgtagactt ggcttaaatg aggatatcac tgaatgcata gcgctcgcac atgatcttgg
6721 tcatcctcca tttggtcatg caggtgaaga tgctctaaag aaatcagttc aagatttgaa
6781 tcttgataat gagaagtatg agtttgatca taatgttcaa gctataagga ttttaactta
6841 tcttgaacaa aaacatgctg attttgatgg tatgaatcta agttgggagg tgattgaagg
6901 agttgcaaaa cataacggtc ccttgcttgg tcaaaacgcg gagtcttcta caagtaatca
6961 gctgttatta gaatacaaca aaaaatatga tctaaaactt gaagaatttt caagcattga
7021 agcgcaagtt gcttcaattg ccgatgatat tgcttacagt gttcacgatc ttgatgatgc
7081 actcagggcg aatttagtaa ccatcgaaga tttgctgaat gttcctttaa ttgaaaaaat
7141 gtttaaagac ataaggagca gatattcaga attgcctcag agcaaactca tacatgaatc
7201 actgagtgga actataggaa ctatgataag tgatgttgtt tctcagactg aaagaaatat
7261 tgaagatcac aaaataaaaa gcgtagaaga cgtaagaagt ctaaataaaa tgctagtcac
7321 attttcacca gaagttgcga atgctacaaa agaaatgaaa agattcaaca tggagaaaat
7381 atacagaagc tataaactga gtagaacgat gaacaaagca aaacgcataa tacaggaact
7441 ctttcaatgt ttttatgaaa acccaggatt acttcccaca gagtggagca aactcgcttg
7501 tgaatctcag cgttcagtaa taatatgtga ctatatctca ggtatgacag atagatttgc
7561 tatacacgag cacagaagaa tttttgatac ctcatacgaa atgacttctt tctaatgact
7621 gatgatcatt tcatgtcaat tgcattaagg cttgcagaaa aaaatcttgg aaatgttgca
7681 ccaaatcctg ctgtcgggtg tgttattgta aaggatggta caattgttag tgagggatac
7741 acagggattg gtgggcgtcc gcatgcggag gtagtcgctt tgcaaaacgc taaagattca
7801 actcatggcg caactatata tatcactctt gagccatgtt gtcatcacgg agtcacgggg
7861 ccttgcactg caaaaatcat aaaagctagc ataaaaagag tagtaattgc aactattgac
7921 ccagatagta gagtttcagg cagaggcatg aaagctctga aagaagcagg aattgaagtt
7981 gagcaaggta ttatgcaaaa agaggcaaaa gaactgaatg tcggtttttt caccactaaa
8041 gaattacata gaccatttat agcttgcaaa gtcgcaacaa ctcttgacgg aaaaatcgca
8101 acatttacag gcgatagcaa atggataaca agtgaaaata cgagaaattg ggtgcatgag
8161 cttagagcaa aatatgatgc aattatgatt ggcagcaata ctcttattaa tgatgatcca
8221 ctcttaactt gcagattacc aggacttgga agtagatcgc caataaggct aattatagat
8281 agccaaggga gattaaagga agagcataac attgcaaaga ctgcagacaa agtaataact
8341 tgggtcatca caaataaaga agtagagaaa aaaataaaaa acattaacta cttagtagtt
8401 aattcaagca acgcaggtaa ggtttgccta aaggacatgg cattaaaact tgtttcagaa
8461 attggtataa caagattatt agttgaaggt ggaggagtgt taatcacaga actattaaag
8521 tgtaatttaa tcgacaggtt gataatatgc cgcagtggta aaattttagg caatgatgcc
8581 actccttttg taggagattt agggattcaa ttcattaaca actgttatca gtttaaaaaa
8641 acaaagataa tagagtttag tgaggatata gttgaggttt gggataggtc gtcataatcc
8701 ttacatactc aaacagtaaa aaaaaggtat ccgttcagcg gagcggtaaa ataaaaagta
8761 gacaaggagt gctaaaaaca taatatcaca agtagtatgt gtggtaaata tgtctgatct
8821 actagctagt cttctcaagc tctgtgaaag ctttaaaata gagatagagc aactaaaagc
8881 agagataaag aggttagaga tagaaaacga aaattttaga tctgagaata aggcgctaag
8941 aatcgaaaat gctgaattga aagaaagact aggtctgaat tcacaaaatt catcgatacc
9001 gagctcaaaa gagttataca agctaaagaa gaaaaagaaa aaaagtgaca ggaaaatagg
9061 ggcacagatt gggcatgaag gaaagtatcg ccctaaaatg gaagcagatg aagtggtgaa
9121 aatagagttc ttgcgagtgc ggaggagaaa ttgcgatatc gaaagagcca tacattcatc
9181 aaaaagttga tttgccagct tatgttgtgg aataccaatg gccgctgttg caagtgtgga
9241 aaaagaaaaa gtagcaggct accagaaggt gttactgcgg atacatttgg tccaaaagtt
9301 aagtcaacaa ttgcggcgct gagcggattt tacaagaact cgaaaagaga agtagcaaat
9361 attataaaaa atattttcaa cctggacatc agtgttggta gcgtgtcaaa tagcgaggct
9421 agagttgcag aaaaatgcca agaagcatat gagcaaatcg agcaagaagt aagtaaaagt

9481 gaagttctgc atatcgatga aacgagccat tataacaaag gtaaacttgg ttggtgctgg
9541 atgtttgcca gtaatacagc aagttttata aaattgacag agtcaagagg aatgaaggtt
9601 ttacaaaata gtaagttctg caatcgcaat agcttacgac aggtacgcag catacaacta
9661 ctttgccgat aagaacaggc aaatctgttg ggcacattta tcaagagatt ttgaaagatt
9721 agcacatagt tgaagttaaa gttctgggct gttacttaag aaacgtagcc actgaattat
9781 ttgcactaaa aaaggccttg ctaaaaaatg agatagatgt ttttagattt accagacgtg
9841 ccagaaaatt gcgaaaacgt acaaggtatt acttgaagcc gtttacctga agcaattgga
9901 gcttctcgag ttgcaaaaaa tattttgaaa tctgaaagaa taatgtggaa ttttttagat
9961 gacccagaaa acattccgct gacaaataac catgctgaac ggcaaatacg gcattatgtc
10021 gtttatcgca aaaattcata ttttacccaa tcgaagtggg gaacttgaac ggataatttc
10081 attatacttg acatggaaac aaatgggctt tccaaaacct cttatctatc atctctcaca
10141 ccgcttagct gaacggatac aacccaagaa aggtttttct acgtcatacc gccgcggtat
10201 ctcagcatag attccgctaa cgagtagcgg aatgacgaat ttgttgtttt tcaaattgta
10261 ggtaaaccta agccacttta gctatatagg gtcacaacga tggattttta agatcgttta
10321 cggctttctt aatgtcttca gccttgaata tcgccgatcc cgcaactaaa atatctgcac
10381 ctgcttttat tatatcagct gcgttagaaa agttaattcc accatctact gaaatttgtg
10441 ttttaagatt acgctcctgt atcatttttt ttatagtaga tatcttgttc aactgtgaat
10501 gaataaattc ctgccctcca aagccagggt tgactgtcat aatcagcaca atatctagct
10561 catgtattat atattcaagc acacttgggg aagttgaagg aacaattgag actccagctt
10621 gaatcggttt ttttgcatcg tttacatttt tgtatgactt tatctttctt atcaacctct
10681 caaggtgtat ctctgcttct gcatgtacag tgataatatc agcaccagca tttataaagc
10741 tttcaatgtg gttaccagga gatttgacca ttaaatgcac atcaaaagga agattgctat
10801 atttacgtat tgcagagaca acgctaggac caattgtaat atttggaacg aaattcccat
10861 ccataacgtc tatgtgtatg taatctacac ctaaatcgct aatttttctt acttcttctc
10921 ctaattttgc aaagtctgct gaaagtatag aaggtgcaat tttaatactc atgagctgcc
10981 tttttttacta aaaaataact tattttttaaa ctctgtcacc ctaaacacca tctaagtagc
11041 tagagaagct ggataccgat atcggtatgc ctatttaact agatcttaaa ttagataaaa
11101 aaaggtagtc ttgataaaac ttatttacag caagtttttt tacatttgga ctttctgttt
11161 gttctttttt gtaccctaat ttatagctta ttgaatcctt gtataaactg atcgttttgt
11221 atgctttgtt catctctcta ggaaagatgc cattagcaat tacgacgcat aaccctgtag
11281 ctgttgcaaa agctagagtg gtaaatgcta gattttgtaa aaaattcctt ccactaactt
11341 ttttttcctg tatattagca gagtgcgatt tgtttgacac ttcttctaga tattcatgcc
11401 atatgtgatt gcagttcgta cattttacct ttcttccaga tgcacctatt tgcttaggag
11461 atactaagta agttttagta caactattac attgtatttt catagcactt acatataagt
11521 agtaacaagc ccaaatgata cgagctacaa atttaatatc aatatatata taacaaatgg
11581 aaggtttgca actaaaaaaa acacagagtg agatagaaga tatttggaaa aatagagaaa
11641 aatttaatga ctgtaatcta aagaaaacag caagaatagc aattaaagag gtaattgagc
11701 tccttgatag tggcaaaatt agagtagcag aaaagctatc aagtggagaa tgggtagtac
11761 ataagtggat aaagcaggca atattattac attttctcac cgaagaaaat aaaataatag
11821 ataatactaa ttgctggttt gacaagatcg gtaacaaatt tagtgaatgg aatgaggaga
11881 aatttcgcca gttaaaaatt agagcagttc ctgggtgttt tgttcgccga tctgcttata
11941 taggtacaaa tgttgttcta atgccaagtt ttatcaacgt tggtgcatat gttgattcag
12001 gaacaatgat agatacctgg tcaacgattg gtagctgtgc gcaaatagga aaaaactgtc
12061 atatttccgg tggagtggga ataggtggag ttcttgagcc tattcaagct tcacctgtca
12121 ttatagaaga taattgcttt attggagcac gtagcgaagt ggccgagggt gtggtagtga
12181 gagaaggatc agtccttggc ataggtgtgt ttattggggc atcaacaaag attattgata
12241 gagaaactag caaggtattt tgcggcgaag tgccacctta ttctgtagtg gtaccagggt
12301 ctattccatc taaaaataat atttcaacct attgtgcagt catagtaaaa aaagtggatg
12361 aaaagacgag atcgaaaatc tctataaatg aaatattgag ggattaaact atcttactcc
12421 ttgtcgtcac ttttttttatc attattacta accacagtaa tggcatacgc gcataactct
12481 gagaaagaga ctccattttc ttgagatagt ttatataatt tttgtagact ttcttttact
12541 ttattaggaa tattccctcc acgttctttt acaagccagg aatcttgatg atgaattgga
12601 tgagcatcac tttttggatt tcctatgtat attgcaaaag gtaaactttg tcctttgaag
12661 tcacattgta cagtaaattt ttttatagat tcagccatct aaagacctct acttttcttt
12721 ttggtcgatt ttatagcagg tggaagaggt ggtttcttta taaaatcttg tctattgaca
12781 acactttctc taccttccaa agctcggcga acatcagctc tgcttaattg cgactctttt
12841 gctaccttca ttccatgctt tgccataata ttagcatatg ctagtacttt ttttaccaat
12901 gctagatcac catctagcat ttgaactagc acttgaaaca cttccgctaa agttagccca
12961 tcagcattta taaccttgtt ttctataaaa ttgacagctt cttcaaattt gtattcaata
13021 ctatcatctt gagctataag gacaacataa tcggtgtttt tgtcgtccat aactgaagca

13081 ctattactca caaacttaag tgtatactat atatataaat actctactat tttatattat
13141 ggcacgctat agttaaacat ttattaataa catgctcact tatatacacg aattaattga
13201 caaaagtcaa ggatcaatat ccatcagtga tttcatgaat gccgttttgt accatgaaaa
13261 atacggctat tatacaagta aattaccgct tggtaaggat ggtgatttta ctaccgcacc
13321 tgagatcagc caattatttg gtgaagtaat tgcagtttgg ataatgcata catgggaaaa
13381 attaggaaag ccatcaaaat tttctctagt tgaacttggg ccaggcaaag gaacactcat
13441 tcacgatata ataagagtca ctaaaaaata cagcagcttt tttaattcaa tgttgatcca
13501 cttagttgaa ataagcccta ctttacggaa gatacaaaag gaaaaattaa aagcttaga
13561 tgttaattgg cacaaaaata ttgacaacct accagaacaa ccaaccattt ttttagcaaa
13621 tgagttcttt gacgctcttc cggtagatca gtttgtttac cgcgatgggc agtggtatga
13681 aaatatggtg acaaaacaag atgatggcag tctcttggtg tcatgccagt gcgtgacact
13741 ggaatccaga aaaaaagaat catggattcc agtgtcagct actcagatga caaatggaaa
13801 attctttaat ggtgcagtgg tggaaatatg ttcagttggg gttgaaatat taaaaaaact
13861 tgagaagaag atatataata ataaaggagc tgctttgatt gtagattacg gttatgtata
13921 ccccgcatat aagagcactt tgcaatcgat aaaacaacat aagtatgcta attttcttga
13981 gaatgttggt aatagtgata ttaccgcact tgtgaacttt caagcgttaa gagattcatt
14041 aaagcatgta gattgtgaga ttttaactca aagggaattt ttatatcttt ttggcataaa
14101 agaaagaacc caggctttaa tgaaaagcgc aagtgatgaa caaaagaata ggatctttag
14161 tgaattccta aggttgactg aaaatatggg cactcttttt aaagcaatgc tattgatata
14221 gctaacccaa gaaaggtttc cctacgtcat accgccgcga tatctcagca tagattccgc
14281 taacgagtag cggaatgacg agcttattgt catgccgccg cgaaccgtca taccgccacg
14341 aactgtcata ccaccacgaa ccgtcatacc gcgattcatt cgcggtatct ctagatcccg
14401 ctaacaagca gcgggatgac ggttgtctcc tagctgtcat cccagtactg gtagactcaa
14461 atcacaatgc tcgtatagct gcaaccctgg aggtactgtt tctcacccac tggagatatt
14521 gcacggtact atctacttga tcatcgtggc gcgtttctgg aaacattaaa atttcatact
14581 caaaatcatt tagccatact gcttgatgcg gcaaaataat cctgccagac tctatcatcg
14641 gagcaatccg gtggaatcga gcgagcttac catcatgtgg cactatttcg ataatgggca
14701 aatgactgtt tgccttcagc tcttgtgcta attgctgacc acttgttttg gcttcgatca
14761 aaatcgcgtg tggtgaccat cttgcagcta gtgataaaac ttgttcttta agctttggat
14821 actcaagctt tgcgcgatat acatcaagta aatagaattt attatctatt tttgcccagg
14881 tggtgcagac actaaagttg ctcatgtcgc ttgttgaaac cgcagtatcc caactttgtg
14941 ttatatgcga gagattatca ggaaattttt tatagcgttt caaccactct ggtttaatta
15001 taccacttga aagcggcaga gggttttgtt gatattgagc agcaaaagcg taactcccaa
15061 gttcaacctt tatcatctca atttcttctt ttcctccatc taggggatat agcaactgac
15121 tttcttctct tgaatataat atccttacag gtgacgctgg tttattatat aatcgcctgg
15181 tcgcacagtg ctgagctact cggatgacag gaacgggagg cattggcttt ttgattgaat
15241 aaataacctg cttattttca gaaatcattg gcaaacaaat atgatgccat atgtttttg
15301 gcttggagag aaggtgtcca gttaagtcct cctggtgtag cctgtgcatt acaagaacga
15361 tgactccttt ttttctatcg ttcagcctgg ttactaaagt ctgatcaaac cagtttatgg
15421 cacgctttct aagcgtttca ctcaaagctt gagcagaact gagcggatcg tccacaatga
15481 taaaatcacc accttcacca gttagtgttc caccaactga tgttgcgatt ctgtatcctc
15541 tctgcactgt ttgaaattta tattttgtat tctggtcttt ggatagttct atctctggaa
15601 atagcgctct ataccaacta gactgcatca cgcacctggt gtcgagcgag tgcttttcgc
15661 tgagccgctg agaataactt gcaactatta ttctcgcagt tggttggttt cccagtatcc
15721 atgcgggcca cgcaacgctt atgcacatgg acttcatcga gcgcggaggc atattgaata
15781 ttattcgttt cacttcacca gcgctcgctg cttccagcct atctgctatg actttatat
15841 actgataatc gttatactca caccccggca ctaccgtttg aaagcacagt tcaatgaatt
15901 ttagaaagtt catgatataa tgttgaaacc aacgcgtgac gctgggatga caaaaaaagg
15961 agcactgaag gctataggtg agacgtcgcg gtatctctag atcccgctaa caagcagcgg
16021 gatgatgaat tactatgccg ccgcgaaccg tcataccgcc gcggtatctc agccgctaac
16081 aagtagcgga atgacaacaa tcctacgtca taccgccgcg gcgctaacaa gagatcccgc
16141 taacaagcag cgggatgacg aattacttaa ccatcatcta caccgtcata ccgccgcggt
16201 atctcagccg ctaacaagca gcgggatgac gaggtcatgc caccgcgcga accgtcatac
16261 cgccgcggta tctctagatc ccgctaacac gtaggggaat gacgaggtca tgccgccgca
16321 aaccgtcata ccgcgattca ttcgcggtat ctctagatcc cgctaacaag tagcgggatg
16381 acgattgtcg tttagccata aacattaaga aatttaccaa ataaaaaaaa ggcaaaagaa
16441 gccctggggt tattatccgc tttaaaatat tggcgttttt tatgttttaa acgcttgaca
16501 agcgaaattc agctgctttt aattgcaact aacctacgct gcaaatgttt aagaaattta
16561 ctaagcagaa aaaaagacaa agaatccccg agttagctag tcttttacta tctctgtcga
16621 gtattggcat tttttgatgt cttgtaacgc tttataagcg cgttcagctt atttagataa

```
    16681 aaatctagat gtagatgaag ttttgtaaag acatacagta tctatatact gcaaaaaatt
    16741 gaacataaga cgccgataca ttaagtaatc cttacctttt aatctgcaga ttggcgaaag
    16801 caaatacaat agcttcacta tcatgataag agcgctggcg aagggtgtca agtaagtttt
    16861 ttcgtttcta ttcccaataa aagtttgtta tatggttatg caagaagttt aatactcata
    16921 gtttcaagca tatactgaaa tatctcatta gcaattgcaa catcttggcg tggggctttg
    16981 ccgtgttcta taaacacaga gattgcatag cgcgggttat ggtaagggcc ataggcgata
    17041 aataacttat gactttcgcc cttagagttt atttctggtg taccggtttt gccagcaatt
    17101 tgtataccgc ttagcccttt cttataggtt ccagttttag aattcaccac atcaaacata
    17161 gcttttcgaa ctatgctaag atgctcacaa tccacatcaa tatcgggaaa atcttgtatc
    17221 gttttactca tctcaatgtg gggaattacc tcttttcctg ttgcaatcct cgctgcaaga
    17281 actgcaagct gtagtggtgt tgtaagcata tacccttgtc ctataactaa gttgatagtg
    17341 tcacctaaat accactgcga atatagcttt cgtgtacgcc aatctctatc tggcaacaac
    17401 cctggagctt cttctttaaa cgttccaatc agtggcccac ttccaatacc aaattttctg
    17461 gccatttcta ctagagagtc tacacttatt tttttcccta tattataaaa gtaagtgttg
    17521 catgataatg ccattgcctc atttaaagat acatacccat ggactttgct tttcagacaa
    17581 cgaaatttcc gctcacctat tttcatatag cccttgcacg agaatttctc ttctgatgtt
    17641 attatcccgt cttttaaacc cgcaagcgca actattactt taaatattga accaggtgga
    17701 atttgatatg ataacgcacg attcacaagc ggtaatgaag gagcattcaa gtttttcccaa
    17761 gtctcatttg atagcctgct agcaaaaaga ttattatcgt aagaaggtga attatataat
    17821 gctaaaattt ctccattatt tacatcaatt accactgcgg agccttggtg atctttaaat
    17881 acctctgcgg ttttctcttg cagattaatc tcaattgtca gttgtacatc ttgtccgtct
    17941 tgttgtggta tgcttgataa ttcttttatg acgcgttttt tagaatttat ttcctgctca
    18001 gattttcctg acttgccttt caatatatga tcatatgtat attcaatacc gctgattcct
    18061 acttcgctta tgccttgctg tctttttgta tatccgatta catgagaaca cattgaacca
    18121 aacgggtaat aacgtttata aagagcagtt attttttctg acgattttgc tattttagac
    18181 tcgacttctg ataatgtctg caaatcaact tccttaccag aaccatcaaa caaaacaaca
    18241 tacgaaattt tgtttaccgc aagttcaatg ctattcctat ctaaaatttt gccccgctta
    18301 ggcataatag tagcaactct tattctatta ctattagata gtgcttcgta cttttgtctg
    18361 tttcgtattt gtaaattata taacctgcaa ctaaaaaccg cagaaatggt aagctgaata
    18421 ccacctaata tgaatgctct gcggttaaag actttgtttt ttgtccacat aacattcctt
    18481 caaaacacac tttttcccc tcttttattg ttacacttct ggcttttacc gtattacaaa
    18541 ccctttttc ccactcactt aaggtcacaa acttataatc tgattttttt agtattttaa
    18601 taatatgagg taaagcttca attgtgtttg acctgttatt gtgatcatga aacagtataa
    18661 ctgctccatt atgtacatta cttaaaactc tttcaactaa agtttctggc ttatcacctt
    18721 gccaatctag cgaatcaact gtccataata ttgaatacat atttaattga tcagtatttt
    18781 caatcagatt atcgtcataa catccatatg gcggacgaaa ccattttaca tcttgtttta
    18841 ttgcattttt aattgctgta tttgtctttt ccaattcttg caattgttcc tcacttgaaa
    18901 gtgatgtcaa cttcctatgc gaccaagagt gattgcctaa ctcatgacct gattcatgaa
    18961 ttttctttac tatttcagac gtttttttcat ttatacgttc accaagcaca aaaaatgttg
    19021 cttttgcttc atagctttcc agaacattaa taatatcgtt tattctattg ttggaaggtc
    19081 catcatcaaa cgtaagcgca acaaacttat cgtcgttatt caataatttt tttatattac
    19141 ttaaatttct atatgataga tctagattac aactgagccc acagtatggt aaattaaaat
    19201 tacaatcact acaaaaagcc gtacttgaat atagtaagaa aaaagtgatt atccttataa
    19261 acatttgtaa tcagcaaagt gatagaatac tacacttttt tgctaagtta agccatcttt
    19321 aattgctgta gcctttactt catcgcttct ttaat
//
LOCUS       JAQZAU010000059            9449 bp    dna     linear   ENV 17-MAR-2023
DEFINITION  MAG: Wolbachia pipientis isolate wEsol contig_36397, whole genome
            shotgun sequence.
ACCESSION   JAQZAU010000059  JAQZAU010000000
VERSION     JAQZAU010000059.1
DBLINK      BioProject: PRJNA929258
            BioSample: SAMN33142973
KEYWORDS    WGS; ENV; Metagenome Assembled Genome; MAG.
SOURCE      Wolbachia pipientis (insect metagenome)
  ORGANISM  Wolbachia pipientis
            Bacteria; Pseudomonadota; Alphaproteobacteria; Rickettsiales;
            Anaplasmataceae; Wolbachieae; Wolbachia.
REFERENCE   1  (bases 1 to 9449)
  AUTHORS   Fiutek,N., Couger,B.M., Roy,S., de la Torre,J.R. and Connor,E.F.
```

```
  TITLE     Genomic assessment of the contribution of the Wolbachia
            endosymbiont of Eurosta solidaginis to gall-induction
  JOURNAL   International Journal Molecular Sciences
REFERENCE   2  (bases 1 to 9449)
  AUTHORS   Fiutek,N., Couger,B.M., Roy,S., de la Torre,J.R. and Connor,E.F.
  TITLE     Direct Submission
  JOURNAL   Submitted (10-FEB-2023) Biology, San Francisco State University,
            1600 Holloway Ave, San Francisco, CA 94132, USA
COMMENT     ##Genome-Assembly-Data-START##
            Assembly Date          :: JUN-2019
            Assembly Method        :: FLYE v. 06-2019
            Genome Representation  :: Full
            Expected Final Version :: Yes
            Genome Coverage        :: 100.0x
            Sequencing Technology  :: Illumina HiSeq; PacBio Sequel
            ##Genome-Assembly-Data-END##
FEATURES             Location/Qualifiers
     source          1..9449
                     /organism="Wolbachia pipientis"
                     /mol_type="genomic DNA"
                     /submitter_seqid="contig_36397"
                     /isolate="wEsol"
                     /isolation_source="galls on Solidago altissima"
                     /host="Eurosta solidaginis MN-2018-EFC"
                     /db_xref="taxon:955"
                     /environmental_sample
                     /country="USA: Cowling Arboretum, Carleton College,
                     Northfield, MN"
                     /lat_lon="44.48 N 93.13 W"
                     /altitude="280 m"
                     /collection_date="2018-11"
                     /metagenome_source="insect metagenome"
                     /note="metagenomic"
     gene            <1..774
                     /locus_tag="wEsol_00646"
     CDS             <1..774
                     /locus_tag="wEsol_00646"
                     /codon_start=1
                     /transl_table=11
                     /pfam="Ribosomal_S6"
                     /fullproduct="qcoverage 100 , hcoverage 100 , similarity
                     100 , identity 99.6 , evalue 1.14e-174 , alnlength 257 ,
                     Bacteria , Proteobacteria , WP_065094495.1 MULTISPECIES:
                     30S ribosomal protein S6 [unclassified Wolbachia]"
                     /translation="VNLYEFTFITQQGLLQQEVEEMVQELAVSLKNIKADVMFQQIRD
                     ILKKGNDKLTTQELEVRAENIKEGLVAYSDFLEDLTKILWVELEEDLSNLKEVKSKID
                     KELKDDLKDLGITQDFTKFLGGSTKSAFIHNAVNALKRNISEHLIKIFQDILKDFRIN
                     GPTQSSKALEMLLKNIEASGLIKYEHWGLLDFAYHKNKMKSGHYCIMCISSTASILDE
                     FMRRMKLNENVIRHFPVQVDKIFEGKSHMMNKQIEEQSA"
                     /product="30S ribosomal
                     protein S6"
                     /besthit="qcoverage 100 , hcoverage 100 , similarity 100
                     , identity 99.6 , evalue 1.14e-174 , alnlength 257 ,
                     Bacteria , Proteobacteria , WP_065094495.1 MULTISPECIES:
                     30S ribosomal protein S6 [unclassified Wolbachia]"
     gene            774..1049
                     /locus_tag="wEsol_00647"
     CDS             774..1049
                     /locus_tag="wEsol_00647"
                     /codon_start=1
```

```
                     /transl_table=11
                     /fullproduct="qcoverage 100 , hcoverage 100 , similarity
                     100 , identity 100 , evalue 2.76e-56 , alnlength 91 ,
                     Bacteria , Proteobacteria , WP_007548890.1 MULTISPECIES:
                     30S ribosomal protein S18 [Wolbachia]"
                     /cog="RpsR COG0238 75 Ribosomal protein S18"
                     /pfam="Ribosomal_S18"
                     /tigr="TIGR00165 S18 70 ribosomal protein S18"
                     /product="30S ribosomal
                     protein S18"
                     /translation="MMKRRNSFNNSYVSVNNRTGFRRSKVCPLAASKDEDIDYKNIDL
                     LSKFTSDYGRILPRRLTGVCAKKQRKLRLAIIRARFLALIPYCTKKV"
                     /besthit="qcoverage 100 , hcoverage 100 , similarity 100
                     , identity 100 , evalue 2.76e-56 , alnlength 91 ,
                     Bacteria , Proteobacteria , WP_007548890.1 MULTISPECIES:
                     30S ribosomal protein S18 [Wolbachia]"
     gene            1057..1611
                     /locus_tag="wEsol_00648"
     CDS             1057..1611
                     /locus_tag="wEsol_00648"
                     /codon_start=1
                     /transl_table=11
                     /fullproduct="qcoverage 100 , hcoverage 100 , similarity
                     100 , identity 100 , evalue 3.92e-114 , alnlength 184 ,
                     Bacteria , Proteobacteria , WP_141457004.1 MULTISPECIES:
                     50S ribosomal protein L9 [unclassified Wolbachia]"
                     /cog="RplI COG0359 148 Ribosomal protein L9"
                     /pfam="Ribosomal_L9_N Ribosomal_L9_C"
                     /tigr="TIGR00158 L9 148 ribosomal protein L9"
                     /product="50S ribosomal
                     protein L9"
                     /translation="MLIIILKENIRTLGKLGEVVKVKPGYARNFLFPQRKAVKATKENL
                     TKLEEQRLLLEEENIKRLNVAKELALSLHDKFVVLIKQASEDGKIFGSVTTREIAKIL
                     LQEGHVIDHRSLSFGGVSIKNLGEYQVNVELHSEVVVPITIYVVKSETDANELRQVKL
                     QNQKSEQQEAEQDANKEAADGDDS"
                     /besthit="qcoverage 100 , hcoverage 100 , similarity 100
                     , identity 100 , evalue 3.92e-114 , alnlength 184 ,
                     Bacteria , Proteobacteria , WP_141457004.1 MULTISPECIES:
                     50S ribosomal protein L9 [unclassified Wolbachia]"
     gene            1875..3140
                     /locus_tag="wEsol_00649"
     CDS             1875..3140
                     /locus_tag="wEsol_00649"
                     /codon_start=1
                     /transl_table=11
                     /fullproduct="qcoverage 100 , hcoverage 100 , similarity
                     100 , identity 100 , evalue 4.55e-300 , alnlength 421 ,
                     Bacteria , Proteobacteria , EAL58989.1 adenylosuccinate
                     lyase [Wolbachia endosymbiont of Drosophila ananassae]"
                     /cog="PurB COG0015 438 Adenylosuccinate lyase"
                     /pfam="Lyase_1 ADSL_C"
                     /tigr="TIGR00928 purB 436 adenylosuccinate lyase"
                     /product="adenylosuccinate lyase"
                     /translation="MSSIWEENNKFNIWLKIEKLACEAQAKLGVIPNNIAENLSGAIE
                     FDVECINEIESIVKHDVIAFLTYIAKKAGVDVRYLHYGMTSSDVLDTCLAVQLKESCD
                     ILLENLKNLLAALKKKAEDYKDIVCVGRSHGMHAEPTTLGIKFARFYAEFKRNYQRLI
                     SAQKEISICKISGAVGNFANVDPFVEEYVAKEIGLIPEIISSQVIPRDRHAMFFSVLG
                     VIASSIENVAVEIRHLQRTEIGEISEHFSTGQKGSSAMPHKCNPILSENLTGLSRLIR
                     SYVFPALENVALWHERDISHSSVERCIAPDACITMDFALVRLTDLIDKLVINKENIEK
                     NLNSSKGLVFSQRVLLELVNSGLAREEAYKIVQSNAMKVKQNNSDFLAELKQDKPLLK
```

```
                     VINSEKLESLFDLKYYTKHIDRVYNKVFN"
                     /besthit="qcoverage 100 , hcoverage 100 , similarity 100
                     , identity 100 , evalue 4.55e-300 , alnlength 421 ,
                     Bacteria , Proteobacteria , EAL58989.1 adenylosuccinate
                     lyase [Wolbachia endosymbiont of Drosophila ananassae]"
     gene            complement(3383..4663)
                     /locus_tag="wEsol_00650"
     CDS             complement(3383..4663)
                     /locus_tag="wEsol_00650"
                     /codon_start=1
                     /transl_table=11
                     /pfam="Fe-ADH_2 Fe-ADH DHQ_synthase DHQ_synthase"
                     /fullproduct="qcoverage 100 , hcoverage 100 , similarity
                     100 , identity 100 , evalue 1.61e-307 , alnlength 426 ,
                     Bacteria , Proteobacteria , WP_141457000.1 MULTISPECIES:
                     iron-containing alcohol dehydrogenase [unclassified
                     Wolbachia]"
                     /translation="MHYLKQILHQTEQTFLREVITDYYLADDIYEICTRHGNDVFLVA
                     DKNTAKLLNKNVLNKIPNLIIPAPPVIPARDTGIHKLFSWIPVSATCMTPSRAASLET
                     VNLVRNKAKDSDLIVAFGSGTVNDICKYASYLEKKDYISFPTAASMNGYTSANASILV
                     NGYKKSFKAHLPRAVYIDIDILANAPLRLTLSGFADFICRSTVQADWLLSHLLLGTEY
                     NELPFTLVRDLEQILLREYTALTKRSRKVVLLLMEVLLISGLGMVISKGSYSASQGEH
                     MIAHAMEMVTRDHSLLHGEKIAVTTITMANLQEKILSIQNPIIKPTTLDVEHITQCFD
                     NIEFVRTFEQKQIMQQKIQEIICKEWHNISSLIKQNLLPAKHLQKIFEDLSIPHLPEH
                     LSWNKEQYCKVVDLAFTTRDRFTFLDLANCIEES"
                     /product="iron-containing
                     alcohol dehydrogenase"
                     /besthit="qcoverage 100 , hcoverage 100 , similarity 100
                     , identity 100 , evalue 1.61e-307 , alnlength 426 ,
                     Bacteria , Proteobacteria , WP_141457000.1 MULTISPECIES:
                     iron-containing alcohol dehydrogenase [unclassified
                     Wolbachia]"
     gene            4706..5902
                     /locus_tag="wEsol_00651"
     CDS             4706..5902
                     /locus_tag="wEsol_00651"
                     /codon_start=1
                     /transl_table=11
                     /fullproduct="qcoverage 100 , hcoverage 100 , similarity
                     100 , identity 100 , evalue 1.43e-289 , alnlength 398 ,
                     Bacteria , Proteobacteria , WP_015589204.1 MULTISPECIES:
                     succinyl-diaminopimelate desuccinylase [unclassified
                     Wolbachia]"
                     /cog="ArgE COG0624 409 Acetylornithine
                     deacetylase/Succinyl-diaminopimelate desuccinylase and
                     related deacylases"
                     /pfam="Peptidase_M28 Peptidase_M20 M20_dimer"
                     /tigr="TIGR01246 dapE_proteo 370 succinyl-diaminopimelate
                     desuccinylase"
                     /product="
                     succinyl-diaminopimelate desuccinylase"
                     /translation="MKIDPVELTRKLISFESITPEDSGAIEYIATIFKKSGFDCEILE
                     FGDKVKNLYAKYINGVPNLCFAGHVDVVPPGQLKDWAFGPFNPEVRDGMLYGRGAADM
                     KSGVAAFIAAMVNLIAEKFQFNGSISALITSAEESMEEYGTKAVLEWMKNKQKKIDFC
                     VVGEPTSSEKLGDTIKIGRRGSVTFELICHGKQGHVAYPDLADNPIYKVISILSKVKN
                     TTFDHGNKYFQPSHCEVTTIDVGNNTSNLIPGSATTRFNIRYNNEQTPGGLYKLIDEI
                     CSSVTSDYKLSVHSSRDVFLSTPDRNTDIMLDAISKVTNIDAILSTSGGTSDAAFIKD
                     VCPVIEFGIINKTAHQINECVSVNDIHKLTAIYKEFIENYFNPTNKILNQVNVVRNIS
                     SGPLLA"
                     /besthit="qcoverage 100 , hcoverage 100 , similarity 100
```

, identity 100 , evalue 1.43e-289 , alnlength 398 , Bacteria , Proteobacteria , WP_015589204.1 MULTISPECIES: succinyl-diaminopimelate desuccinylase [unclassified Wolbachia]"

```
gene            complement(6020..6658)
                /locus_tag="wEsol_00652"
CDS             complement(6020..6658)
                /locus_tag="wEsol_00652"
                /codon_start=1
                /transl_table=11
                /fullproduct="qcoverage 100 , hcoverage 100 , similarity
                100 , identity 100 , evalue 2.43e-149 , alnlength 212 ,
                Bacteria , Proteobacteria , WP_006279797.1 MULTISPECIES:
                endonuclease III [Wolbachia]"
                /cog="Nth COG0177 211 Predicted EndoIII-related
                endonuclease"
                /pfam="HhH-GPD HHH Pfam-B_5204 EndIII_4Fe-2S"
                /tigr="TIGR01083 nth 192 endonuclease III"
                /product="endonuclease III"
                /translation="MDSKKVELIFEKFQQSNPAPKIELNYTNHFTLLVAIVLSARTTD
                VSVNKITRELFNIADTPEKMLSFGQSELKKCISSIGLYNSKAKNIIGLSKILVERYNS
                KVPTNFDDLVSLPGVGRKSANVFLNSGLGIPTLAVDTHVFRVSNRIGLVKEKDVFKTE
                QSLLNVVPKKYLLYAHHWLVLHGRYVCKAQKPSCETCIIHDLCEFECKRYKV"
                /besthit="qcoverage 100 , hcoverage 100 , similarity 100
                , identity 100 , evalue 2.43e-149 , alnlength 212 ,
                Bacteria , Proteobacteria , WP_006279797.1 MULTISPECIES:
                endonuclease III [Wolbachia]"
gene            complement(6686..8122)
                /locus_tag="wEsol_00653"
CDS             complement(6686..8122)
                /locus_tag="wEsol_00653"
                /codon_start=1
                /transl_table=11
                /fullproduct="qcoverage 100 , hcoverage 100 , similarity
                100 , identity 100 , evalue 0.0 , alnlength 478 ,
                Bacteria , Proteobacteria , WP_141456998.1 MULTISPECIES:
                isocitrate dehydrogenase [unclassified Wolbachia]"
                /cog="LeuB COG0473 348 Isocitrate/isopropylmalate
                dehydrogenase"
                /pfam="Iso_dh"
                /tigr="TIGR02924 ICDH_alpha 473 isocitrate dehydrogenase"
                /product="isocitrate
                dehydrogenase"
                /translation="MSTLITVAYGDGIGPEIMEAVLSILREAEAEISIDIIEIGERVY
                SKEWSHGISPSGWESIERTKILLKSPTTTPQGKGHKSLNVALRKNLGLYANIRPCISY
                HPVIENKFDKFDIVVIRENEEDVYTGIEHRLTGDSYQCTKIITRSGSEKICRYAFEYA
                KKHNRKKVTCLTKDNIMKMTDGTFHAAFDRIAKEYPDIKAEHYIVDIGMARVATEPEN
                FDVIVTENLYGDILSDIVAQTSGSVGLAGSSNIGNEYAMFEAVHGSAPDIAGKNIANP
                SGLLNAAVHMLIYIGQVGTAKLIYDAWLKTLEDGIHTADLYKEKRSKQKVGTKEFAEA
                VIDNLGKKPAELIIGSGSDSKINLDSKINKVQDNYEQDYKVKKLVGSDITLAWDKSSN
                VDQIVGLFESSNLQMIAIYSKGLAIWPGNSKPSSDQITCRFIANNEKQAITNSDVNNL
                LIKLEEHNFDVVRMDKLYLYDGKEGFFV"
                /besthit="qcoverage 100 , hcoverage 100 , similarity 100
                , identity 100 , evalue 0.0 , alnlength 478 , Bacteria ,
                Proteobacteria , WP_141456998.1 MULTISPECIES: isocitrate
                dehydrogenase [unclassified Wolbachia]"
gene            8268..8804
                /locus_tag="wEsol_00654"
CDS             8268..8804
                /locus_tag="wEsol_00654"
```

```
                     /codon_start=1
                     /transl_table=11
                     /fullproduct="qcoverage 100 , hcoverage 100 , similarity
                     100 , identity 100 , evalue 1.07e-99 , alnlength 178 ,
                     Bacteria , Proteobacteria , WP_006279799.1 MULTISPECIES:
                     RDD family protein [Wolbachia]"
                     /cog="COG1714 COG1714 172 Predicted membrane
                     protein/domain"
                     /pfam="RDD"
                     /product="RDD family
                     protein"
                     /translation="MNTEINVKVNYVGFTRRIVAEILDLIIFSPHFIFGFIYGVFIVS
                     LQDQDKDSFQELAQLATEIPFIVSTVVATLVLTISKILMVTKLGGTPGKLLCGMHIKD
                     ANTFTNVTLMQATIRCISKGSIWIIFRFLLHLHYHGYTFLYLLIVLILFLFAVFDQRK
                     QTFYDKIAKTVVIDYKSS"
                     /besthit="qcoverage 100 , hcoverage 100 , similarity 100
                     , identity 100 , evalue 1.07e-99 , alnlength 178 ,
                     Bacteria , Proteobacteria , WP_006279799.1 MULTISPECIES:
                     RDD family protein [Wolbachia]"
     gene            8827..9303
                     /locus_tag="wEsol_00655"
     CDS             8827..9303
                     /locus_tag="wEsol_00655"
                     /codon_start=1
                     /transl_table=11
                     /fullproduct="qcoverage 100 , hcoverage 100 , similarity
                     100 , identity 100 , evalue 3.54e-102 , alnlength 158 ,
                     Bacteria , Proteobacteria , WP_006279800.1 MULTISPECIES:
                     RDD family protein [Wolbachia]"
                     /cog="COG1714 COG1714 172 Predicted membrane
                     protein/domain"
                     /pfam="RDD"
                     /product="RDD family
                     protein"
                     /translation="MDKKVNYSTMTRRTLAFIVDCILFIAIPFGLMAVGFSLTHAIQG
                     IMGAIIVITTIVFSLFLMIPCIFHIFMLVKFGGTPGHLLFSMRVKDKDTFESITTMQA
                     MKRTIALFIIYCISLCNPILFLAILILVLVCAVGDEHKQAFHDKIANTVVIDYKPS"
                     /besthit="qcoverage 100 , hcoverage 100 , similarity 100
                     , identity 100 , evalue 3.54e-102 , alnlength 158 ,
                     Bacteria , Proteobacteria , WP_006279800.1 MULTISPECIES:
                     RDD family protein [Wolbachia]"
     gene            3140..3212
                     /locus_tag="wEsol_tRNA-13"
     tRNA            3140..3212
                     /locus_tag="wEsol_tRNA-13"
                     /gene="tRNA-Met"
                     /intronpos="0..0"
                     /program="tRNAscan-SE"
                     /cove_score="74.9"
                     /evidence=predicted
                     /type="Met"
                     /anticodon=CAT
                     /product="tRNA-Met (CAT)"
ORIGIN
        1 gtgaatcttt atgaatttac ttttataaca caacaaggat tattacagca agaagtagag
       61 gaaatggtcc aagagctagc tgtttcattg aaaaatatta aagcagatgt tatgttccag
      121 caaatcaggg atattctgaa aaaaggaaat gacaagctta caacacaaga gttagaagtg
      181 cgtgctgaaa acattaaaga aggtctagtt gcttactctg atttcttgga agatcttaca
      241 aaaattttat gggttgaatt ggaagaagat ctttcaaatt tgaaagaagt gaagtcaaaa
      301 atcgataaag agttgaaaga tgacctaaaa gatttaggaa taacgcaaga ttttacaaaa
```

361 tttctaggag gcagtactaa aagtgcgttt atccataacg cagtaaatgc tttgaagaga
421 aatatttcag aacatctaat aaagattttt caagatattt tgaaagattt tagaataaac
481 ggtccaactc aatcgagcaa agcgttggaa atgcttttga aaaacattga agcatcagga
541 ttaatcaagt atgaacattg gggtcttttg gattttgcat atcacaaaaa taaaatgaag
601 agcggtcatt attgtataat gtgcataagt tctactgcaa gcattctgga tgaatttatg
661 cgtagaatga aacttaatga gaacgttatt cgccactttc ctgtgcaggt tgataaaatt
721 tttgaaggta aatctcatat gatgaataaa caaattgagg agcaaagcgc ataatgatga
781 aaagacgaaa tagttttaat aattcttacg tatctgtaaa taataggact ggctttaggc
841 gttctaaagt ttgccctctt gctgcgtcta aagatgaaga catagattat aagaatatag
901 atttattatc caagtttacc tctgactatg gtaggatatt acctagaaga ttaacaggcg
961 tatgtgcaaa aaaacaaagg aagttacgct tagcgatcat aagggcacgt tttttagctc
1021 ttattcctta ctgtactaaa aaagtttagg taatttatgt tgataatttt aaaggaaaat
1081 ataaggactt taggtaagct tggtgaagtt gtcaaagtta aaccaggtta tgcacgtaat
1141 tttcttttc cacaaagaaa agcggtgaaa gctaccaagg aaaatttaac aaagctagag
1201 gagcaacgtt tattactgga agaagagaat ataaaaagat taaatgtagc aaaagagctt
1261 gcattgtcac tacatgataa gtttgtggta ttgataaagc aagcttcaga ggatggtaaa
1321 atttttggtt ctgtaacaac acgtgaaatt gcgaaaattt tactacaaga aggacatgtg
1381 atagaccacc gtagtttatc ttttggtgga gtaagcatca aaaacttggg tgaataccaa
1441 gtaaacgtag aattacatag tgaagtagta gtaccaatta ctatatacgt cgtgaaatcc
1501 gaaacagatg caaatgagct aaggcaagta aaattgcaaa atcagaagtc tgagcaacaa
1561 gaggcagagc aagatgcgaa taaagaagct gctgatggtg atgatagctg atcaaagatg
1621 atcccttgtt atagccgaaa agaaatgtct gccatggcta agctggtgtc atcccagtgc
1681 ttccctcatg tcatcccagt gcgtgacact gggatccagt ttatttcatc atggacatta
1741 ttttatgctt ctagaatccg ttgtccattg aataatttgc aaatactttt atacctggat
1801 tccagtgtca cgcactggaa tgacaccatt attggaataa atcaaaaatg attccccgct
1861 atagcagaaa agaaatgtct tccatttggg aggaaaataa taaatttaat atatggctta
1921 aaatagaaaa attagcgtgt gaagctcaag caaagttagg agtgattccg aataatattg
1981 ctgagaacct ctctggtgct attgaatttg atgttgaatg tatcaacgaa attgaatcca
2041 ttgtaaaaca tgatgttata gcttttctga catatattgc taaaaaagcg ggagttgatg
2101 ttcgttatct tcattatgga atgacaagtt ctgatgtttt ggatacatgc cttgcggtgc
2161 aactgaaaga gtcctgtgat attttgctcg agaatctaaa aaatttactt gcagcattga
2221 aaaaaaaagc tgaggactat aaagatattg tttgtgttgg acgcagtcac ggaatgcatg
2281 cagaaccaac aactcttgga ataaaatttg ccagatttta tgctgaattt aaacgcaatt
2341 atcagagatt aattagcgcg caaaaagaga tctcaatttg taaaatatca ggtgcagtag
2401 gcaattttgc aaatgttgat ccgtttgttg aagaatatgt agcgaaagaa ataggactca
2461 tacctgaaat catatcgtct caagtcattc ctcgtgatag acatgctatg ttcttttcag
2521 ttttgggagt gattgcaagc tcaatagaaa atgttgctgt tgaaattcgt catttgcaaa
2581 gaactgaaat cggcgaaatt tctgagcatt tttccacggg gcagaaggga agctctgcta
2641 tgccacacaa atgtaatcct attttaagtg aaaatctgac tggactctca cgtttaatac
2701 gcagttatgt ttttcctgcg ttagaaaatg ttgcattgtg gcacgaacgt gatatatcac
2761 actcgtctgt ggaaagatgc attgctcctg atgcttgtat aacaatggat tttgctttag
2821 tgcgattaac agatttgata gataaattgg tgatcaataa ggaaaacatt gaaaagaact
2881 taaattcttc aaaaggttta gttttctcac agcgtgtatt gcttgagtta gtaaatagtg
2941 gtttggcgag ggaagaggcg tataaaattg ttcagagcaa tgcaatgaaa gtgaaacaaa
3001 acaacagtga ttttctggcc gaattgaaac aagataaacc cttacttaag gttattaact
3061 cggaaaaact tgaatctttg tttgatttga agtattatac gaaacatata gatcgtgtgt
3121 ataataaggt ttttaattag gctgagtagc tcagttggta gagcaggagg atcataatct
3181 cttggtcggg ggttcaagtc cctcctcagc cactcgaact aaactatgtt tagcaaagca
3241 gtgctactgg tgaaacccag tgtcagttac ttggaatgag ttgtatatcc actcaggtga
3301 ggagacaatg gataagagat tgtgaaaagg gtttagcttt tggcctatga tctctttaaa
3361 tgacagaatt tcactacaaa acttaacttt cttctatgca gttagctaag tcaagaaagg
3421 tgaatctatc tcttgttgta aacgcaagat cgaccacttt acaatattgt tctttattcc
3481 aactgaggtg ctctggtaag tgtggaattg atagatcttc aaatattttt tgcaggtgtt
3541 ttgcaggtag taaattttgc ttaattaaac tggaaatatt atgccattct ttacaaataa
3601 tctcttggat tttttgctgc ataatttgct tctgttcaaa agttcttaca aattcgatat
3661 tatcaaagca ctgagttata tgttctacat ctaaagtggt cggtttgata atcgggtttt
3721 gtattgataa tatcttttcc tgcaaattag ccatggtaat cgtcgtaaca gcaatttttt
3781 cgccatgtag taaggaatga tctcttgtta ccatttccat tgcgtgagct atcatatgct
3841 ctccttggct tgcagaatag ctgccttttg atattaccat tccaagtcct gaaattagta
3901 gaacttccat aagtaatagg accacttttc tacttctttt agtaagcgcc gtatactctc

3961 ttagcaaaat ttgctccaaa tcgcgaacaa gtgtaaaagg taattcattg tattctgtgc
4021 caagcaacaa atgagatagt agccagtcag cctgaactgt tgaacgacaa atgaaatctg
4081 caaatccgct taatgtgaga cgcagtggtg cattggcaag tatgtctata tctatatata
4141 ctgctcttgg aagatgtgct ttgaacgatt ttttatatcc atttactaat attgaagcat
4201 ttgcagaggt atatccattc atagaagcgg ctgttgggaa cgatatataa tctttttttct
4261 cgaggtagct tgcatatttg cagatatcgt taacagtgcc gctaccaaat gcaactatta
4321 agtcactgtc ttttgctttg tttctaacta gatttacagt ttcaagagag gcagctctgc
4381 ttggtgtcat gcaagtagct gacactggga tccaggaaaa caacttatgg attccagtgt
4441 cacgcgctgg aatgacaggt ggagctggaa taattaagtt aggaatttta ttaagtacgt
4501 ttttgtttaa aagttttgct gtatttttat ctgcaactag aaagacgtca tttccgtgtc
4561 gtgtgcatat ttcatagata tcatcagcaa ggtagtaatc tgttattact tcccttagaa
4621 aagtttgttc ggtttgatgt aatatttgtt ttaaatagtg catagagtgt ttacaatata
4681 gcagtataag tattttaaga ttgacatgaa aattgaccct gtagagctaa ctaggaaatt
4741 gatctctttt gagagtataa caccagaaga cagcggggca atagagtata tagcaacaat
4801 cttcaagaag agcggctttg attgtgagat tttagaattt ggtgataaag ttaaaaatct
4861 ttatgcgaaa tatataaatg gagtaccaaa tttgtgcttt gctgggcacg ttgatgttgt
4921 accaccgggt cagttaaaag attgggcatt tggtccattt aatccagaag tgagggatgg
4981 aatgctatat ggaagagggg ccgctgatat gaaaagcgga gtagctgcat ttattgctgc
5041 tatggtaaat ttgattgcag agaagtttca atttaatggt tcgataagtg cattgattac
5101 cagtgctgaa gaaagcatgg aagaatatgg aacaaaggca gttttggaat ggatgaaaaa
5161 taagcagaaa aagatagatt tttgtgtggt tggcgaacca accagtagtg agaaattagg
5221 tgataccata aaaataggca gaagaggttc tgtaacattt gaattaattt gccatggaaa
5281 acaaggcac gttgcctacc cagacctagc agacaatcca atatataagg taatatcgat
5341 attgagtaag gtaaaaaata ctacttttga tcatggtaat aaatattttc agccttcaca
5401 ttgcgaagtt actactatcg atgttggaaa taatactagc aatctaatac ctggctcagc
5461 aactacacgt tttaacattc gatataacaa tgaacaaaca ccgggtggct tatataagct
5521 gattgatgaa atatgctcca gtgtaactag cgattataaa ctttctgtgc atagcagcag
5581 ggacgttttc ctctctactc ccgatagaaa tactgatatt atgcttgatg caataagtaa
5641 ggttaccaac attgatgcta tacttagcac aagtggtggc acatctgacg ctgcgtttat
5701 caaagatgtt tgtccagtga ttgaatttgg tataatcaac aaaaccgcac atcagataaa
5761 cgaatgcgta tcagtaaacg atatacacaa gttaacagct atatataagg aatttataga
5821 aaattatttc aatcccacta ataaaatatt gaatcaggtc aacgtggtta ggaatatatc
5881 tagtggtcca ttgttagctt agatgtaatc agatctcttg cataactgtt gtctgaataa
5941 caactggtgt cattccagca aaatgtgtca agtaagtttt ttcgtttcta tagctacttg
6001 gatgacaaaa gaagaaatac tagactttat accttttgca ctcaaattca cataaatcat
6061 gaattatgca cgtttcacac gaaggtttct gtgctttgca aacatatcta ccatgcaaaa
6121 ctagccaatg atgagcataa agcaggtatt ttttgggaac cacatttaaa agagattgct
6181 ctgttttaaa cacatctttt tctttcacaa gcccaattct attgctcact ctaaaaacgt
6241 gagtatcaac tgccaatgtt ggaatgccaa gccccgaatt taagaacaca ttagcgctct
6301 ttctccccac tccaggcaaa gacaccaaat catcaaaatt agtaggtact ttgctgttgt
6361 atcgttctac caatatttta ctaagcccga ttatattttt tgctttggaa ttatataaac
6421 caatactgct tatgcatttt ttcagctcac tctgcccaaa gctcagcatt ttttctggcg
6481 tatcagcgat attaaatagt tctcttgtaa ttttattaac gcttacatca gttgtccgcg
6541 ctgataaaac tatcgcaacc agtaacgtaa aatgattggt ataatttagc tctatttttg
6601 gcgcaggatt tgattgttga aacttttcaa atattagttc tacttttttt gagtccatct
6661 catttaccac acctctttta aatatttaaa caaagaaccc ctctttccca tcatacaaat
6721 acaatttgtc cattctcacc acgtcaaaat tgtgttcttc gagtttgatt agcaagttat
6781 ttacatcact atttgtaata gcttgctttt cattatttgc gataaaccta caggttattt
6841 ggtcacttga aggttttgag tttcctggcc aaatcgcaag tccttttgag tatattgcta
6901 tcatttgcag attacttgat tcaaataatc ctactatttg atcaacatta ctggatttgt
6961 cccaagcgag agttatatca ctacccacta gctttttaac tttgtaatct tgttcgtaat
7021 tgtcttgtac tttatttatt ttactatcca aatttatttt actatccgaa ccactgccaa
7081 ttataagctc agcaggtttc tttcctaaat tatctataac agcttctgca aattctttgg
7141 taccaacttt ctgtttactt cttttctcct tatataaatc ggcagtgtgt attccatcct
7201 ccaaagtctt gagccatgca tcgtagatta gttttgcagt acccacttga cctatgtaaa
7261 ttagcatatg cactgcagca tttaaaagac cagaaggatt ggctatgttt tttcctgcaa
7321 tatcaggggc agaaccgtgc actgcttcaa acattgcgta ttcattcccg atgttgctac
7381 ttccggcaag ccccacagat ccagaggttt gtgcgactat atctgataat atgtcgccat
7441 ataagttctc ggttactata acatcaaaat tctccggttc tgttgcaaca cgcgccattc
7501 caatatcaac tatataatgt tctgccttta tatctgggta ttcctttgca atgcgatcaa

```
     7561 acgcggcatg gaaagttccg tcagtcattt tcatgatgtt atctttagtc aggcaagtta
     7621 cttttttct gttgtgtttt tttgcatatt caaaagcgta cctgcatatc ttctctgagc
     7681 cagatctagt aataatttta gtgcattggt aagagtcacc agtgagccta tgctctattc
     7741 cggtgtaaac gtcctcttca ttttcacgta tcactacgat atcaaactta tcgaatttat
     7801 tttctataac aggatgatat gaaatgcatg gtctgatatt tgcatacaac cctagatttt
     7861 ttctgagtgc aacgtttaag cttttgtgcc ctttaccttg aggagttgtt gtcggagatt
     7921 ttaataatat ttttgtcctt tcaattgatt cccaaccact tggagaaatt ccgtgagacc
     7981 attctttact gtaaacacgc tcccctattt caataatatc tattgagatt tccgcttctg
     8041 cttcgcgcaa tatcgacagc accgcttcca tgatttctgg tccgataccg tcgccatatg
     8101 caactgtgat tagtgttgac ataacttccc tagttataaa tgatttaata ttaatttaaa
     8161 taatagagtg gcacaagtag ctaatattac ccaagttagt atatttacgg ttatttaata
     8221 acgtattttt atatcatatg atgatattat gaaatatagg tggggttatg aatactgaaa
     8281 taaatgttaa agtaaattat gtaggattta caaggcgtat tgtagcagaa atacttgacc
     8341 tcatcatatt ttcccctcat tttatcttcg gttttatcta tggtgtattc atcgtatcat
     8401 tacaagatca agataaagac tcttttcagg aacttgcaca actagcaaca gaaataccat
     8461 ttatagtttc tacagtggtc gcaacattag tactaacaat atccaaaata ttaatggtaa
     8521 caaaacttgg tggtacccca ggaaaactat tgtgtggtat gcatattaaa gatgctaata
     8581 catttacaaa tgttaccctt atgcaagcaa caataagatg tatttctaag ggaagcatct
     8641 ggattatttt tcgctttttg cttcatctgc attatcatgg ttatactttt ctatatcttt
     8701 taattgtgct aattttattt ttgtttgcag tatttgatca acgcaaacag actttttacg
     8761 ataaaattgc aaaaacagta gtgatagact ataaatctag ttgatatttt tctttatagg
     8821 tagtacatgg ataaaaaagt aaactactca acgatgacaa gacgtactct agcatttata
     8881 gttgattgta ttttatttat cgcaatacca tttggtttaa tggcggtggg tttctcactt
     8941 acacacgcaa tacaagggat catgggtgca ataattgtaa tcaccacgat tgtattttct
     9001 ttatttttaa tgataccatg tatatttcat atattcatgc tagtaaaatt tggcggcact
     9061 ccaggacact tgttatttag catgcgtgta aaagacaaag atacatttga gtcaatcact
     9121 acaatgcaag caatgaaaag gactatagct ctttttataa tttattgcat atcactttgt
     9181 aatcccattt tattcttagc tatactaatc ttagttttag tatgtgcagt aggtgatgaa
     9241 cataaacaag cttttcacga caaaattgca aacacggtag tgatagatta taaacctagt
     9301 tgatattttc ctttagctgt ctgccaccaa gggtgtcatt ccagtgtcac gcgctaactt
     9361 aaggtaaatt gccaacttaa ctataaatat attttggtaa atccaaatta ttttagctat
     9421 aagagcgttt tacttttgc tcagcatat
//
LOCUS       JAQZAU010000060         5710 bp    dna     linear   ENV 17-MAR-2023
DEFINITION  MAG: Wolbachia pipientis isolate wEsol contig_36686, whole genome
            shotgun sequence.
ACCESSION   JAQZAU010000060  JAQZAU010000000
VERSION     JAQZAU010000060.1
DBLINK      BioProject: PRJNA929258
            BioSample: SAMN33142973
KEYWORDS    WGS; ENV; Metagenome Assembled Genome; MAG.
SOURCE      Wolbachia pipientis (insect metagenome)
  ORGANISM  Wolbachia pipientis
            Bacteria; Pseudomonadota; Alphaproteobacteria; Rickettsiales;
            Anaplasmataceae; Wolbachieae; Wolbachia.
REFERENCE   1  (bases 1 to 5710)
  AUTHORS   Fiutek,N., Couger,B.M., Roy,S., de la Torre,J.R. and Connor,E.F.
  TITLE     Genomic assessment of the contribution of the Wolbachia
            endosymbiont of Eurosta solidaginis to gall-induction
  JOURNAL   International Journal Molecular Sciences
REFERENCE   2  (bases 1 to 5710)
  AUTHORS   Fiutek,N., Couger,B.M., Roy,S., de la Torre,J.R. and Connor,E.F.
  TITLE     Direct Submission
  JOURNAL   Submitted (10-FEB-2023) Biology, San Francisco State University,
            1600 Holloway Ave, San Francisco, CA 94132, USA
COMMENT     ##Genome-Assembly-Data-START##
            Assembly Date          :: JUN-2019
            Assembly Method        :: FLYE v. 06-2019
            Genome Representation  :: Full
            Expected Final Version :: Yes
```

```
            Genome Coverage        :: 100.0x
            Sequencing Technology  :: Illumina HiSeq; PacBio Sequel
            ##Genome-Assembly-Data-END##
FEATURES             Location/Qualifiers
     source          1..5710
                     /organism="Wolbachia pipientis"
                     /mol_type="genomic DNA"
                     /submitter_seqid="contig_36686"
                     /isolate="wEsol"
                     /isolation_source="galls on Solidago altissima"
                     /host="Eurosta solidaginis MN-2018-EFC"
                     /db_xref="taxon:955"
                     /environmental_sample
                     /country="USA: Cowling Arboretum, Carleton College,
                     Northfield, MN"
                     /lat_lon="44.48 N 93.13 W"
                     /altitude="280 m"
                     /collection_date="2018-11"
                     /metagenome_source="insect metagenome"
                     /note="metagenomic"
     gene            complement(<1..986)
                     /locus_tag="wEsol_00656"
     CDS             complement(<1..986)
                     /locus_tag="wEsol_00656"
                     /codon_start=1
                     /transl_table=11
                     /fullproduct="qcoverage 100 , hcoverage 84.7545219638243
                     , similarity 100 , identity 99.7 , evalue 6.76e-219 ,
                     alnlength 328 , Bacteria , Proteobacteria ,
                     WP_006279533.1 MULTISPECIES: multidrug effflux MFS
                     transporter [Wolbachia]"
                     /pfam="MFS_1 MFS_2 Pfam-B_3877 MFS_3 Sugar_tr TRI12
                     MFS_2"
                     /tigr="TIGR00710 efflux_Bcr_CflA 385 drug resistance
                     transporter, Bcr/CflA subfamily"
                     /product="multidrug effflux
                     MFS transporter"
                     /translation="MILSVAIVDMATDLYSVALPSIANYFKVEGSVVQLTISLNLVGF
                     AVSGLIYGPLSDHYGRRPMMLIGMTIFTLASVMCYIADSIALLILIRFIQGTGAGVAG
                     VVGYSAIRDMYSGSEYSRVISKLNMVVALSPGIAPVVGSYIISHGYSWKFLFFIISLA
                     AIAILIFIYFKLQETLTVNKNETSITNITTNICKQYISIFRNYRFLGFSAIHGLTFMW
                     LWAYIANYPFIFESMGIEVQYFGYLISIIVIFYIIGTLINRRYVPKVGVSKMLIIGLV
                     LPIISDSLLVYFYFADKLNILILQTVWIPANIGLALIISNNVTSALETIKGI"
                     /besthit="qcoverage 100 , hcoverage 84.7545219638243 ,
                     similarity 100 , identity 99.7 , evalue 6.76e-219 ,
                     alnlength 328 , Bacteria , Proteobacteria ,
                     WP_006279533.1 MULTISPECIES: multidrug effflux MFS
                     transporter [Wolbachia]"
     gene            complement(1172..2038)
                     /locus_tag="wEsol_00657"
     CDS             complement(1172..2038)
                     /locus_tag="wEsol_00657"
                     /codon_start=1
                     /transl_table=11
                     /fullproduct="qcoverage 100 , hcoverage 100 , similarity
                     100 , identity 100 , evalue 5.82e-213 , alnlength 288 ,
                     Bacteria , Proteobacteria , WP_015589080.1 MULTISPECIES:
                     hypothetical protein [unclassified Wolbachia]"
                     /translation="MSKEMVNALKAEVKKLKYADFKANTWDEVISGEGTYKDLNRTHG
                     LAINGQKITNEFINNLYSNHKNLILRDENGKGNYRPFLRQIFTEMFKHVGATIPNNSI
```

```
                     IEELINNYYQGGYTHFLPNDMNQALGSFHLSTQGNCMTREYIMDCREPNCIKLSFSMK
                     YTKMLNYDDCTYIETPGFCSSAEFKLKCKDENVTYEDGKVLLTIPKELEDYKAGDDGL
                     FDKLTYIINKLIEYFKKLCEKLGFEFDTRIEYDPERNIKIEHNLGEPLKVVSEANASS
                     VEDLKLKVNNQR"
                     /product="hypothetical protein"
                     /besthit="qcoverage 100 , hcoverage 100 , similarity 100
                     , identity 100 , evalue 5.82e-213 , alnlength 288 ,
                     Bacteria , Proteobacteria , WP_015589080.1 MULTISPECIES:
                     hypothetical protein [unclassified Wolbachia]"
     gene            complement(2096..4177)
                     /locus_tag="wEsol_00658"
     CDS             complement(2096..4177)
                     /locus_tag="wEsol_00658"
                     /codon_start=1
                     /transl_table=11
                     /product="hypothetical protein"
                     /pfam="GTA_TIM Pfam-B_29 Phage-tail_3 Pfam-B_29"
                     /translation="MVLNFAQLTKIRDEQGNYPAVIELVKLAKRIKLNLGKEVTVTYA
                     ADWSEYHSYDGWYNMDELWSSEYIDVVGIDAYFPLTDGPEPPFGYSAEDVMGGWSSGV
                     GYDYFYDYSKSESEKVKYNDSRYAWKNIEKWWSETHINPGGSKTKWQPKMKKIWFTEY
                     GFPSMNGCTNEPNVFVDKGSIESKYPRYSNGEVSFLSQKIAIEGTLKKWQSSEMVEKM
                     FLWAWDARPFPYFPNLCDMWTDCHNWQTGHWIQGKLSQLNISDVLSDLLQKTGLKSDQ
                     FDASNVKGLLSGYVINDQQSVRSIIKMLQSCYFFDVVEQDSKLKFVQKGRGVTTGIPI
                     GEMVFSNNSKLANISQLDLNNKVNVVYFNRNFGYPIDVKYAELPKQGAAITVEIPLIM
                     EEGEAQNIAEVLLYSSWQERNIYNFKLPIKYAWLVPSDVITILDGEKKHTVRIIKTKF
                     ESMSIQAIGVGYDHSIYKLSFPSTRSLMLKEYPPSHISKTIVEMIDLPDIKGNSASFT
                     LISEEKNWKGATLFISYNDKDYKPIASTNKQSTYGYVMESTDEGLIVVLRFGILSSSY
                     NHYLSDGVIPALDYISPGSQCLGTGMTEEGFTSTNVSTINVSNLALVGKEVIKFQSAE
                     LIDKNKYKLSNLIRGQEGTKEYQHTTGEKFVLLDDSIISFEVQPGKRLFLKAVTYGDS
                     LDNTEAKVLSN"
                     /besthit="qcoverage 99.4227994227994 , hcoverage
                     62.1841155234657 , similarity 99.9 , identity 99.9 ,
                     evalue 0.0 , alnlength 689 , Bacteria , Proteobacteria ,
                     WP_174516821.1 glycoside hydrolase TIM-barrel-like
                     domain-containing protein [Wolbachia endosymbiont of
                     Cardiocondyla obscurior]"
     gene            complement(4137..5423)
                     /locus_tag="wEsol_00659"
     CDS             complement(4137..5423)
                     /locus_tag="wEsol_00659"
                     /codon_start=1
                     /transl_table=11
                     /product="hypothetical protein"
                     /pfam="Pfam-B_29 Pfam-B_29 GTA_TIM"
                     /translation="MSTIILSSIFGQAGSIFGPIGRIIGSELGALLGAQLDGAMFGLE
                     AEQKVTHGARLKNLQVQTSTYGRTIPIIYGTARVAGNIIWAQPIKEEAITTKSKIGRG
                     MNVTYNYYATLAIAICKGKVEKLNRIWADTTSLSFDEIDYTFYSGREDQNPDPFMSSI
                     EGEGNVPAYRGISYIVIKNFPLADYNNHVPVFTFEVQTAVKSNEFSVAKNIRNINIIP
                     GSGEFVYDTKIQKKIAQEKISSNQYIPYGLAQRVNHNNHTKKSDAMLSLDQLKEDLPN
                     VELVSVVVNWFVNNLNIKDCKIYPAVEFQDDSAILPDDWQVGGVTRDNAQLISKDASG
                     NPRYGGTVSDAALTRYIEELHSRGYKVMLYPMPLLDTKNKEWRGRLSGAPEDISDFFK
                     NQYNKFIEHYASIAKQTKVEGFIIGSELCATYQNQR"
                     /besthit="qcoverage 97.8971962616822 , hcoverage
                     37.8158844765343 , similarity 100 , identity 100 , evalue
                     6.45e-295 , alnlength 419 , Bacteria , Proteobacteria ,
                     WP_141456988.1 glycoside hydrolase TIM-barrel-like
                     domain-containing protein [Wolbachia endosymbiont of
                     Carposina sasakii]"
ORIGIN
        1 cctatacctt tgatcgtttc taaagcagaa gttacgttat tgcttattat taatgcaagt
```

61 ccgatattgg ctggaatcca aacagtttga agaattaata tatttaactt gtccgcgaaa
121 taaaaatata ccaataaact atcggatatt atcggtaaca ccaaacctat tattagcatt
181 ttgctaaccc ctacttttgg tacatacctt ctattaatta aagttccaat tatgtaaaat
241 atgactatga tcgatatgag gtagccaaaa tactgtactt caatgcccat cgattcaaat
301 atgaaagggt agttagcaat gtatgcccaa agccacatga aagttaatcc atgaatggct
361 gaaaacccaa ggaaacgata atttctaaat attgatatat attgcttgca gatattagta
421 gttatgttag ttatactggt ttcattttta tttacagtga gtgtttcttg taacttaaag
481 tagataaaaa taagtatagc aattgctgca agtgatataa taaaaaacaa aaatttccaa
541 ctatacccat gtgaaattat ataactgccc accactggag ctattccagg tgagagtgcc
601 acaaccatat ttaattttga aatgactctc gagtattcac tacccgagta catatccctt
661 attgctgaat acccaacaac acctgcaaca ccagcccctg ttccttgtat gaagcgaatt
721 agtatcaaga gcgcaatact gtcggctata taacacatga cacttgccaa agtaaaaatt
781 gtcataccaa tcagcatcat tggacgccta ccataatggt ccgataatgg accataaatt
841 aagccagata ctgcaaatcc aactagattc aagctaattg taagctgcac tacactgcct
901 tctactttaa aataattggc aatgcttggt agtgcaactg agtataggtc agttgccata
961 tcgacaattg ctacagacaa tatcataata agagagatga tattttgcgg aagagctgtt
1021 ttcacttaat gatttactgg attaaatata cttaattata atgtattttt ttcattagta
1081 taagtacaat gttacatagt aaacgatgtc attcgagtag cggacactga aatcttaagc
1141 taaaatcatg aaaagcctga atttcaatac cttatctttg attattcact ttaagcttta
1201 agtcttcaac agaagatgca tttgcctcac ttactacctt tagtggttca ccaagattat
1261 gctctatttt tatattacgc tcaggatcgt actctattct tgtatcaaac tcaaagccca
1321 atttctcaca aagtttttttg aaatactcaa taagcttatt tataatataa gtaagcttat
1381 caaacaaacc gtcatcacca gctttatagt cctctaactc tttaggaatg gtaagcaata
1441 ccttaccgtc ttcatacgtc acattttcat ccttacattt cagcttaaac tccgctgaac
1501 tgcaaaaacc tggtgtttcg atatatgtac aatcatcata gttaagcatt tttgtatatt
1561 tcatgctaaa actaagcttt atacaattgg gttcacgaca atccataata tactctcttg
1621 tcatacagtt gccttgcgtt gacaaatgaa aagatccaag agcttgattc atatcattag
1681 gaagaaaatg agtatatccg ccctgataat agttatttat taactcctca ataatagagt
1741 tattgggaat tgttgcacca acgtgtttaa acatttctgt aaaaatttgt ctgagaaatg
1801 gacgataatt cccttttccg ttttcatccc gtagaattag atttttatgg tttgaataca
1861 gattgttaat aaattcgtta gttatttttt gtccgttaat agcaaggccg tgtgttctat
1921 taagatcctt atatgttccc tcaccagaaa ttacttcgtc ccaagtgtta gctttgaaat
1981 cagcatattt gagcttcttc acttcagctt tcaacgcatt gaccatttcc tttgacatgt
2041 tatatctcta ccattaatac ctgtatatta attttagcac ttactttatt aaatttcaat
2101 tactcaagac cttcgcttcc gtattatcca atgaatcacc ataagtcact gctttaagga
2161 aaagtcgttt tcctggttgc acttcaaaag atattattga atcatcaagt agaacaaatt
2221 tttcacctgt ggtgtgctga tactctttgg tgccttcttg ccctctaatt aggttgctaa
2281 gcttatattt atttttatct attagttcag cactctggaa ctttatcacc tcttttccaa
2341 ccagtgctaa attgcttaca tttatagtac taacgttagt gcttgtaaaa ccctcttccg
2401 tcatcccagt gcccagacac tgggatccag gagaaatata gtcaagcgct gggatgacac
2461 catcacttaa ataatggtta tatgaagaac tcaatatacc aaaacgtagc actactataa
2521 gcccctcgtc ggtagattcc attacatatc cgtaagtaga ctgcttgttt gtgcttgcaa
2581 taggcttata atccttatcg ttgtacgaaa taaagagtgt tgctcccttc caattctttt
2641 cctcactaat taaagtaaag cttgcactat tacctttaat atccggtaaa tctatcattt
2701 ctacgatggt tttgctgata tgagagggag ggtattcctt cagcataagt gaccttgttg
2761 aaggaaaaga aagcttatat atagagtgat cataaccaac gccaattgct tgaatggaca
2821 tgctttcgaa ttttgttttt ataattctca ccgtatgctt tttctcaccg tctaaaatcg
2881 ttattacatc acttggcaca agccacgcat attttatcgg cagcttgaaa ttgtatatat
2941 ttctctcttg ccacgaagaa taaagtaaaa cctcagctat attttgcgcc tctccctcct
3001 ccataatgag cggtatttca actgttatag cagcgccttg ctttggcaac tcagcatatt
3061 taacatcaat tggatagcca aaattgcgat taaagtaaac gacgttaact ttattgttta
3121 aatctaattg actgatatta gcaagttttg aattgttact gaaaaccatt tcaccaattg
3181 gtattccagt cgtaactccc ctgccctttt ggacaaattt cagtttagag tcctgttcaa
3241 ccacatcaaa aaaatagcaa ctttgcagca ttttaataat tgaacgtacc gattgctggt
3301 cgtttatcac atacccagac aataaccctt tgacgttgct tgcatcgaac tgatcgcttt
3361 ttaaacctgt tttttgtaac agatccgata aaacatcgga aatattaagc tgtgaaagct
3421 ttccctgaat ccaatgtcct gtctgccaat tatggcagtc agtccacata tcacacaaat
3481 tggggaaata aggaaatggc cttgcatccc atgcccagag aaacattttt tctaccattt
3541 ctgaactttg ccactttttt agcgttcctt caattgcaat tttttgcgaa agaaagctca
3601 cctctccgtt tgaatatcgt ggatatttgc tctctatgct gcctttgtca acaaacacat

```
     3661 ttggctcatt agtgcagccg ttcatactgg gaaatccata ttcagtaaac catatttttt
     3721 tcatttttgg ttgccatttt gttttactac caccggggtt tatgtgagtt tcactccacc
     3781 atttctctat atttttccac gcatacctgc tgtcgttata ctttactttt tcagactcac
     3841 tctttgagta atcatagaaa taatcatacc ccactccact gctccaacca cccattacat
     3901 cttccgcaga atagccaaaa ggaggctctg gaccatcagt cagtggaaaa taagcatcta
     3961 tgccaacaac gtcgatgtac tctgaagacc acagttcatc catattatac caaccatcat
     4021 atgaatggta ttcgctccaa tctgcagcgt aggtaactgt tacttctttt ccaagattaa
     4081 gttttattcg ctttgcaagc ttgaccagct ctataacggc agggtaattc ccttgttcat
     4141 ctctgatttt ggtaagttgc gcaaagttca gaaccaatga taaacccttc cactttagtt
     4201 tgtttggcaa tgcttgcata atgctctata aatttgttgt actgattctt aaaaaaatca
     4261 cttatatctt caggagcccc actcaatctt cctcgccact ctttgttctt tgtatcaagt
     4321 aagggcattg gatagagcat caccttataa cctctgctgt gcagctcttc tatatatcta
     4381 gttaacgctg catcactaac tgtgccacca taccttgggt ttccactcgc atcttttgaa
     4441 attagctgag cattatctct ggttacaccc cctacttgcc aatcatcagg taatatcgcg
     4501 gaatcatctt gaaattcaac tgcagggtat attttacaat ctttaatgtt cagattattc
     4561 acaaaccaat tgactaccac cgacaccaac tcaacatttg gcaaatcttc ttttaattga
     4621 tccaaagaaa gcattgcatc gctcttttta gtgtggttgt tgtgatttac cctttgcgcc
     4681 agcccatatg ggatatattg gttgctgctt attttctctt gcgcgatttt cttctgtatt
     4741 tttgtatcat atacaaactc acctgaacct ggtatgatat tgatattccg aatatttttt
     4801 gctactgaaa attcatttga ctttactgca gtttgcactt caaacgtaaa taccggaaca
     4861 tgattattat agtctgctaa aggaaaattc ttgatgacta tgtaagatat tcccctataa
     4921 gccggcacat tcccctctcc ttcaattgat gacataaatg gatcaggatt ttggtcttct
     4981 ctgccgctgt aaaaagtata atctatttca tcaaagctaa gtgatgttgt atctgcccag
     5041 attctgttta atttttctac tttccccttg cagatcgcaa ttgcaagcgt tgcatagtaa
     5101 ttatatgtaa cgttcatacc tcttccaatt ttacttttgg tggttatcgc ctcttctttt
     5161 attggctgtg cccaaataat attccctgca acacgagcag tgccatagat aattgggatc
     5221 gttctgccgt aggttgaggt ttgaacttgc agatttttta gccttgcccc atgcgttact
     5281 ttttgctcgg cttcaaggcc gaacattgcc ccatctagct gtgcaccaag aagagcacca
     5341 agttctgagc cgataatccg gccaattggc ccaaaaatgc tgcctgcttg accaaaaatt
     5401 gatgataaaa ttattgtaga catggtaatc ctccttttttg tttctatagt taatgtaggc
     5461 tctacgtcat gccgccgcgc cgctaacaag cagcgggatg acgagcttat cgttatgccg
     5521 ccgcgaaccg tcataccgtc acggtatctc tagatcctgc taacacgtag cagggtactt
     5581 aaccgtcata ccgcgattca ttcgcggtat ctcttagccg ctaacaagca gcggaatgac
     5641 gaatttgtcg ttttacagcc acttggatga caatttgcta ctaaatagta ggaaattcaa
     5701 taatttacaa
//
LOCUS       JAQZAU010000061         2713 bp    dna     linear   ENV 17-MAR-2023
DEFINITION  MAG: Wolbachia pipientis isolate wEsol contig_36894, whole genome
            shotgun sequence.
ACCESSION   JAQZAU010000061 JAQZAU010000000
VERSION     JAQZAU010000061.1
DBLINK      BioProject: PRJNA929258
            BioSample: SAMN33142973
KEYWORDS    WGS; ENV; Metagenome Assembled Genome; MAG.
SOURCE      Wolbachia pipientis (insect metagenome)
  ORGANISM  Wolbachia pipientis
            Bacteria; Pseudomonadota; Alphaproteobacteria; Rickettsiales;
            Anaplasmataceae; Wolbachieae; Wolbachia.
REFERENCE   1  (bases 1 to 2713)
  AUTHORS   Fiutek,N., Couger,B.M., Roy,S., de la Torre,J.R. and Connor,E.F.
  TITLE     Genomic assessment of the contribution of the Wolbachia
            endosymbiont of Eurosta solidaginis to gall-induction
  JOURNAL   International Journal Molecular Sciences
REFERENCE   2  (bases 1 to 2713)
  AUTHORS   Fiutek,N., Couger,B.M., Roy,S., de la Torre,J.R. and Connor,E.F.
  TITLE     Direct Submission
  JOURNAL   Submitted (10-FEB-2023) Biology, San Francisco State University,
            1600 Holloway Ave, San Francisco, CA 94132, USA
COMMENT     ##Genome-Assembly-Data-START##
            Assembly Date          :: JUN-2019
```

```
            Assembly Method        :: FLYE v. 06-2019
            Genome Representation  :: Full
            Expected Final Version :: Yes
            Genome Coverage        :: 100.0x
            Sequencing Technology  :: Illumina HiSeq; PacBio Sequel
            ##Genome-Assembly-Data-END##
FEATURES             Location/Qualifiers
     source          1..2713
                     /organism="Wolbachia pipientis"
                     /mol_type="genomic DNA"
                     /submitter_seqid="contig_36894"
                     /isolate="wEsol"
                     /isolation_source="galls on Solidago altissima"
                     /host="Eurosta solidaginis MN-2018-EFC"
                     /db_xref="taxon:955"
                     /environmental_sample
                     /country="USA: Cowling Arboretum, Carleton College,
                     Northfield, MN"
                     /lat_lon="44.48 N 93.13 W"
                     /altitude="280 m"
                     /collection_date="2018-11"
                     /metagenome_source="insect metagenome"
                     /note="metagenomic"
     gene            <1..373
                     /locus_tag="wEsol_00660"
     CDS             <1..373
                     /locus_tag="wEsol_00660"
                     /codon_start=1
                     /transl_table=11
                     /product="hypothetical protein"
                     /pfam="DUF1626"
                     /translation="ICGDWQYQPEKYSKESGAQIDLVFDREDGCVMLCEIKYSDKPYT
                     VTKDVEQLKRKEAIYRERTESKKQIFWVLIAANGVSENQYLKDTIHHVITLEDLFKVI
                     LNFNMYTNLRDFIKALEEKKI"
                     /besthit="qcoverage 81.3008130081301 , hcoverage
                     31.1526479750779 , similarity 99.0 , identity 96.0 ,
                     evalue 1.74e-59 , alnlength 100 , Bacteria ,
                     Proteobacteria , MBV2146633.1 winged helix-turn-helix
                     domain-containing protein [Wolbachia endosymbiont of
                     Pissodes strobi]"
     gene            409..810
                     /locus_tag="wEsol_00661"
     CDS             409..810
                     /locus_tag="wEsol_00661"
                     /codon_start=1
                     /transl_table=11
                     /fullproduct="qcoverage 95.4887218045113 , hcoverage
                     81.4102564102564 , similarity 98.4 , identity 95.3 ,
                     evalue 1.78e-79 , alnlength 127 , Bacteria ,
                     Proteobacteria , EAL58093.1
                     3-octaprenyl-4-hydroxybenzoate carboxy-lyase [Wolbachia
                     endosymbiont of Drosophila ananassae]"
                     /cog="UbiD COG0043 477 3-polyprenyl-4-hydroxybenzoate
                     decarboxylase and related decarboxylases"
                     /pfam="UbiD"
                     /tigr="TIGR03701 mena_SCO4490 433 menaquinone
                     biosynthesis decarboxylase, SCO4490 family"
                     /product="3-octaprenyl-4-hydroxybenzoate
                     carboxy-lyase"
                     /translation="MTEIHRRILSNKGPAIIFENVVTENGKNSIPVLVNLFGTIKRIA
```

```
                     LGLNINPGELRDLGKLLAFLRSPEPRKNFKDAVKMFPLLKVVLSMRSKVVSKAPCQEV
                     VLTGDEVDLSLLPIQTCWPNEPAPLSPDQLW"
                     /besthit="qcoverage 95.4887218045113 , hcoverage
                     81.4102564102564 , similarity 98.4 , identity 95.3 ,
                     evalue 1.78e-79 , alnlength 127 , Bacteria ,
                     Proteobacteria , EAL58093.1
                     3-octaprenyl-4-hydroxybenzoate carboxy-lyase [Wolbachia
                     endosymbiont of Drosophila ananassae]"
     gene            896..1198
                     /locus_tag="wEsol_00662"
     CDS             896..1198
                     /locus_tag="wEsol_00662"
                     /codon_start=1
                     /transl_table=11
                     /product="hypothetical protein"
                     /pfam="UbiD"
                     /tigr="TIGR00148 TIGR00148 438 UbiD family decarboxylase"
                     /translation="MRWLAHRGGAGHHKRWKEKEQNMKFPAAAVIGSDPATIIAAVTP
                     VPETLSEYQFAGLLRKKPLELVNCKTIPLQVPAHAEIVLEGCVSLDRYQDEGPYGR"
                     /cog="UbiD COG0043 477 3-polyprenyl-4-hydroxybenzoate
                     decarboxylase and related decarboxylases"
                     /besthit="qcoverage 99 , hcoverage 32.3529411764706 ,
                     similarity 99.0 , identity 99.0 , evalue 2.98e-63 ,
                     alnlength 99 , Bacteria , Proteobacteria , WP_121721681.1
                     UbiD family decarboxylase, partial [Wolbachia
                     endosymbiont of Drosophila ananassae]"
     gene            1176..1523
                     /locus_tag="wEsol_00663"
     CDS             1176..1523
                     /locus_tag="wEsol_00663"
                     /codon_start=1
                     /transl_table=11
                     /product="hypothetical protein"
                     /pfam="UbiD"
                     /tigr="TIGR00148 TIGR00148 438 UbiD family decarboxylase"
                     /translation="MKGHTVGDHTGYYNSVEQFPEFNITAITMRKNPIYLSTFTGKPP
                     DEPSILGEALNEIFVPILINQFPEIVDFYLPPEGCSYRIAVISIKKVLSRAGKKNCYG
                     HTFLSKAIFIHEV"
                     /cog="UbiD COG0043 477 3-polyprenyl-4-hydroxybenzoate
                     decarboxylase and related decarboxylases"
                     /besthit="qcoverage 73.0434782608696 , hcoverage
                     51.5337423312883 , similarity 100 , identity 98.8 ,
                     evalue 8.43e-54 , alnlength 84 , Bacteria ,
                     Proteobacteria , EAL58158.1
                     3-octaprenyl-4-hydroxybenzoate carboxy-lyase, partial
                     [Wolbachia endosymbiont of Drosophila ananassae]"
     gene            1571..1813
                     /locus_tag="wEsol_00664"
     CDS             1571..1813
                     /locus_tag="wEsol_00664"
                     /codon_start=1
                     /transl_table=11
                     /product="hypothetical protein"
                     /translation="MWAISTRMVPVRDTITIENAPIDYLDFASPESGIGGKMGFDATN
                     KIPPETKREWGEKIKMSEEIVRKVTEKWEEYGLTGD"
                     /cog="UbiD COG0043 477 3-polyprenyl-4-hydroxybenzoate
                     decarboxylase and related decarboxylases"
                     /besthit="qcoverage 100 , hcoverage 17.2043010752688 ,
                     similarity 98.8 , identity 97.5 , evalue 7.76e-47 ,
                     alnlength 80 , Eukaryota , Arthropoda , CAD7414331.1
```

```
                     unnamed protein product [Timema poppensis]"
     gene            1887..2387
                     /locus_tag="wEsol_00665"
     CDS             1887..2387
                     /locus_tag="wEsol_00665"
                     /codon_start=1
                     /transl_table=11
                     /fullproduct="qcoverage 100 , hcoverage 100 , similarity
                     99.4 , identity 99.4 , evalue 1.00e-114 , alnlength 166 ,
                     Eukaryota , Arthropoda , GFR09019.1 phospholipase D
                     [Trichonephila clavata]"
                     /cog="Cls COG1502 438
                     Phosphatidylserine/phosphatidylglycerophosphate/
                     cardiolipin synthases and related enzymes"
                     /pfam="PLDc_2 Regulator_TrmB Pfam-B_9369 DUF1669"
                     /product="phospholipase D"
                     /translation="MCFSLYYYAGFIFSPCPKATVCFSPGEDCAVPIISVIDQSKKSI
                     LVQEYTFTLGTVAKSLINAKERGVDVKVILDKSQLYSKYSVINELFSGGVPIWIDDKP
                     KIAHNKIMIVDNQKVITGSFNLSKTAKKGNAENLLIITDYPELIQQYVKNWEVRMSQS
                     YKYAPN"
                     /besthit="qcoverage 100 , hcoverage 100 , similarity 99.4
                     , identity 99.4 , evalue 1.00e-114 , alnlength 166 ,
                     Eukaryota , Arthropoda , GFR09019.1 phospholipase D
                     [Trichonephila clavata]"
     gene            2497..>2713
                     /locus_tag="wEsol_00666"
     CDS             2497..>2713
                     /locus_tag="wEsol_00666"
                     /codon_start=1
                     /transl_table=11
                     /product="hypothetical protein"
                     /translation="MDYINDADGSYTAIHDFFQNNGVGDYKVQCSYGGVPYATDEYVH
                     SGDKFWSNNFIDSHFGREIKKSFPTCFP"
                     /besthit="qcoverage 88.8888888888889 , hcoverage
                     35.5555555555556 , similarity 100 , identity 100 , evalue
                     4.99e-42 , alnlength 64 , Bacteria , Proteobacteria ,
                     MBV2146636.1 hypothetical protein [Wolbachia endosymbiont
                     of Pissodes strobi]"
ORIGIN
        1 tatatgtgga gattggcaat atcaaccaga aaaatacagt aaagagtctg gtgctcaaat
       61 agatctagtt ttcgatcgag aagatggatg tgtaatgctc tgtgagataa aatatagtga
      121 taaaccttat acagtaacaa aggatgtcga acaattaaaa agaaaagaag caatttatag
      181 agaaagaacg gaatcaaaga agcaaatatt ctgggtgtta attgctgcaa atggagtttc
      241 tgagaaccaa tatctaaaag atacgataca tcatgtgatt acactagaag acttgtttaa
      301 agttatttta aattttaata tgtacaccaa cttacgcgac ttcattaaag ccttagaaga
      361 aaaaaagatc tgattaggat taaggaggaa gtttcaacag ttcttgaaat gacagaaatt
      421 caccgtagga ttttgtcaaa taaaggacca gctatcatct ttgaaaatgt tgtcacggaa
      481 aacggtaaaa attcgattcc agttttggtg aatttatttg gtactattaa gaggattgca
      541 ttggggttga acataaaccc tggtgaacta agagatcttg gtaaactttt agcatttttg
      601 cgatcacctg agccacgaaa aaacttcaaa gatgccgtga aaatgtttcc tttactaaaa
      661 gttgtattgt cgatgcgaag taaagttgtg agcaaggctc catgtcaaga agtggtgcta
      721 actggggatg aggtggatct tagtttgcta cctattcaaa catgctggcc aaatgaacct
      781 gcaccactat cacctgacca attgtggtga caaaaggccc aatggaagac aagcaagata
      841 atttcaatct tggaatatat cgtatgcagg ttacagataa aaaaaaacga ctctaatgcg
      901 ctggcttgca catcgtggtg gtgcaggtca ccacaaacga tggaaagaga aggaacaaaa
      961 tatgaaattt cctgctgctg ctgtgatcgg tagtgatcct gcaacaatta ttgctgcggt
     1021 aactccagtg ccagaaacat tgtcagaata ccaatttgct gggttgctgc gcaaaaaacc
     1081 gcttgagctt gtaaattgta agactattcc gcttcaagtg ccagctcatg cagagattgt
     1141 tctggaaggc tgtgtaagtt tggatagata tcaagatgaa gggccatacg gtcggtgatc
     1201 acaccggcta ctataattct gttgagcaat ttccagaatt taatattact gcaattacta
```

```
     1261 tgcgcaaaaa tccaatttat ctcagcactt tcactggcaa gccacctgat gaaccatcta
     1321 ttcttggtga agcgctcaac gaaatctttg tgcccattct tatcaatcaa tttccagaga
     1381 tagtggattt ttatctacca ccagaaggtt gttcatatag aatagcggtg atatcgataa
     1441 aaaaagtttt atccagggca ggcaaaaaga attgttatgg gcatactttc ctttctaaag
     1501 caatttttat acacgaagtt taattatcgt tgttgatgat gatataaatg tacgtgattg
     1561 gaaggaagtg atgtgggcaa tatcaacgag aatggtccct gtgcgtgaca caatcacaat
     1621 agagaacgca cctattgatt atttagattt tgcttcccct gaaagtggaa ttggtggtaa
     1681 aatgggattt gatgcaacaa ataaaatccc gcctgaaacc aagcgagaat ggggagagaa
     1741 aattaaaatg agtgaggaaa tagtaagaaa agtcacggag aagtgggaag aatatggatt
     1801 gacaggtgat taaattatga tgtagcctat cgccgtatat ccgggggtgg gttgttgtga
     1861 ggctttttca ctttttattc ttgttaatgt gtttttctct gtattactat gcgggtttta
     1921 tattttcgcc ctgcccaaaa gctacggttt gcttttcacc tggagaagat tgtgctgtac
     1981 cgataatcag cgtaatagat cagtctaaaa aatctatctt agttcaagaa tatacattta
     2041 ctcttggaac agttgcaaaa tctttgatta atgctaaaga acgcggtgtt gatgttaaag
     2101 tcatcttaga taaatcgcaa ctctactcaa aatatagtgt tataaatgaa ttattttcag
     2161 gtggagtacc aatttggatc gatgataagc caaagattgc tcataataag ataatgattg
     2221 ttgacaatca aaaagtcatc acagggtcat ttaaccttag taagacagct aaaaaaggaa
     2281 atgctgagaa tttattaatt attacagatt atcctgaatt gattcagcag tatgtgaaaa
     2341 actgggaggt acgaatgtca caatcttata aatacgctcc caactaatcg agagcgcttg
     2401 cttaaaatca gttctaaaaa cagcaagaga gagctttttt gattgattga cataatgatt
     2461 atgatataat gaactaaaag tttaaggaat aaagtaatgg attatataaa tgatgctgac
     2521 ggtagctaca ctgccattca tgattttttt caaaacaacg gtgttggaga ttacaaagtt
     2581 caatgcagtt atggtggggt gccgtatgct actgatgaat acgtgcattc tggcgataaa
     2641 ttttggagta ataatttcat agactcacat tttggcaggg aaataaaaaa gtcttttccg
     2701 acttgttttc cag
//
LOCUS       JAQZAU010000062         1424 bp    dna     linear   ENV 17-MAR-2023
DEFINITION  MAG: Wolbachia pipientis isolate wEsol contig_37018, whole genome
            shotgun sequence.
ACCESSION   JAQZAU010000062 JAQZAU010000000
VERSION     JAQZAU010000062.1
DBLINK      BioProject: PRJNA929258
            BioSample: SAMN33142973
KEYWORDS    WGS; ENV; Metagenome Assembled Genome; MAG.
SOURCE      Wolbachia pipientis (insect metagenome)
  ORGANISM  Wolbachia pipientis
            Bacteria; Pseudomonadota; Alphaproteobacteria; Rickettsiales;
            Anaplasmataceae; Wolbachieae; Wolbachia.
REFERENCE   1  (bases 1 to 1424)
  AUTHORS   Fiutek,N., Couger,B.M., Roy,S., de la Torre,J.R. and Connor,E.F.
  TITLE     Genomic assessment of the contribution of the Wolbachia
            endosymbiont of Eurosta solidaginis to gall-induction
  JOURNAL   International Journal Molecular Sciences
REFERENCE   2  (bases 1 to 1424)
  AUTHORS   Fiutek,N., Couger,B.M., Roy,S., de la Torre,J.R. and Connor,E.F.
  TITLE     Direct Submission
  JOURNAL   Submitted (10-FEB-2023) Biology, San Francisco State University,
            1600 Holloway Ave, San Francisco, CA 94132, USA
COMMENT     ##Genome-Assembly-Data-START##
            Assembly Date          :: JUN-2019
            Assembly Method        :: FLYE v. 06-2019
            Genome Representation  :: Full
            Expected Final Version :: Yes
            Genome Coverage        :: 100.0x
            Sequencing Technology  :: Illumina HiSeq; PacBio Sequel
            ##Genome-Assembly-Data-END##
FEATURES             Location/Qualifiers
     source          1..1424
                     /organism="Wolbachia pipientis"
                     /mol_type="genomic DNA"
```

```
                     /submitter_seqid="contig_37018"
                     /isolate="wEsol"
                     /isolation_source="galls on Solidago altissima"
                     /host="Eurosta solidaginis MN-2018-EFC"
                     /db_xref="taxon:955"
                     /environmental_sample
                     /country="USA: Cowling Arboretum, Carleton College,
                     Northfield, MN"
                     /lat_lon="44.48 N 93.13 W"
                     /altitude="280 m"
                     /collection_date="2018-11"
                     /metagenome_source="insect metagenome"
                     /note="metagenomic"
     gene            complement(153..494)
                     /locus_tag="wEsol_00667"
     CDS             complement(153..494)
                     /locus_tag="wEsol_00667"
                     /codon_start=1
                     /transl_table=11
                     /fullproduct="qcoverage 100 , hcoverage 100 , similarity
                     100 , identity 100 , evalue 1.53e-79 , alnlength 113 ,
                     Bacteria , Proteobacteria , AAS13810.1 transposase, IS5
                     family, OrfB [Wolbachia endosymbiont of Drosophila
                     melanogaster]"
                     /cog="COG3293 COG3293 124 Transposase and inactivated
                     derivatives"
                     /pfam="DDE_Tnp_1_2 DDE_Tnp_1"
                     /product="transposase, IS5 family, OrfB"
                     /translation="MRTKYTKVKKMWADMGYQGRNLKNHIKEEYDIDIEIVKRPPCRF
                     WVHKDTPPELLPTREQGFKVQPRRWVVERTFAWVNRNRRLSKEYDLLTTSTENFIYLA
                     MSRVMLKREYA"
                     /besthit="qcoverage 100 , hcoverage 100 , similarity 100
                     , identity 100 , evalue 1.53e-79 , alnlength 113 ,
                     Bacteria , Proteobacteria , AAS13810.1 transposase, IS5
                     family, OrfB [Wolbachia endosymbiont of Drosophila
                     melanogaster]"
     gene            complement(605..985)
                     /locus_tag="wEsol_00668"
     CDS             complement(605..985)
                     /locus_tag="wEsol_00668"
                     /codon_start=1
                     /transl_table=11
                     /fullproduct="qcoverage 100 , hcoverage 100 , similarity
                     100 , identity 100 , evalue 1.16e-87 , alnlength 126 ,
                     Bacteria , Proteobacteria , AOV87106.1 transposase
                     [Wolbachia endosymbiont of Drosophila incompta]"
                     /cog="COG3293 COG3293 124 Transposase and inactivated
                     derivatives"
                     /pfam="DUF4096"
                     /product="transposase"
                     /translation="MRKKYPTDLSEREWARIEKHFRVSYKKGGRPPKYSKREILEAIF
                     YVLRTGCQWRYLPNDFPLWKTVYEQFRQWKKQGIFEKMNYEITKYSRRKIGRNEQPSA
                     CIVDSQSVKTTEKGGSKAMMEVKK"
                     /besthit="qcoverage 100 , hcoverage 100 , similarity 100
                     , identity 100 , evalue 1.16e-87 , alnlength 126 ,
                     Bacteria , Proteobacteria , AOV87106.1 transposase
                     [Wolbachia endosymbiont of Drosophila incompta]"
     gene            1180..>1424
                     /locus_tag="wEsol_00669"
     CDS             1180..>1424
```

```
                     /locus_tag="wEsol_00669"
                     /codon_start=1
                     /transl_table=11
                     /fullproduct="qcoverage 100 , hcoverage 91.0112359550562
                     , similarity 98.8 , identity 98.8 , evalue 1.08e-50 ,
                     alnlength 81 , Bacteria , Proteobacteria , WP_213863653.1
                     PD-(D/E)XK nuclease family transposase, partial
                     [Wolbachia endosymbiont of Ceratitis capitata]"
                     /pfam="PDDEXK_2"
                     /tigr="TIGR01784 T_den_put_tspse 273 conserved
                     hypothetical protein"
                     /product="PD-(D/E)XK nuclease family
                     transposase, partial"
                     /translation="MALSKFLDPRNDLCFKKIFGTEKNKNILTHFLNDILGFTEINAI
                     QEVEFLSTIMDPEVASDKQSIVDVLCKDSHGNRYIAE"
                     /besthit="qcoverage 100 , hcoverage 91.0112359550562 ,
                     similarity 98.8 , identity 98.8 , evalue 1.08e-50 ,
                     alnlength 81 , Bacteria , Proteobacteria , WP_213863653.1
                     PD-(D/E)XK nuclease family transposase, partial
                     [Wolbachia endosymbiont of Ceratitis capitata]"
ORIGIN
        1 aacacgatct ttcatttgag ctttcttcaa tttaaaataa atacagaaaa ttgctgccta
       61 tagctaaact gacttacgac aatttgaaaa actgtcatcc cgctacttgt tagcgggatc
      121 tatgctaaga ggttgtccgg aaactagtaa attcaagcat attccctctt taacataacc
      181 ctactcatag ctaggtatat gaaattctca gtggatgttg tgagtaaatc atactccttc
      241 gatagccttc tattcctatt aacccaagca aaagtccttt ctacaaccca tcttcttggc
      301 tgtactttaa acccttgttc tcttgttggt agtagctcag gtggcgtatc tttgtgcacc
      361 caaaatctac atggaggcct tttaacaatt tcaatatcta tgtcatattc ttcctttatg
      421 tgattcttta aatttcttcc ttggtatccc atgtcagccc acattttttt aactttagta
      481 tattttgttc tcatattgtt taatgctatt ttaataccat ctctatcatt ttcgttagca
      541 gcgcctacgt aacaacctag tataaaaccc tgagtgtctg taattatatg ccttttttcta
      601 ccctttactt ttttacttcc atcatagcct ttgatccccc cttttctgta gtctttacag
      661 attgactatc tactatacag gcactcggct gctcattcct tcctattttt cttctactat
      721 attttgtaat ttcataattc attttctcaa aaattccctg cttcttccat tgcctgaact
      781 gctcatacac agtcttccat agcggaaaat catttggtaa ataccgccat tgacaccctg
      841 tacgcaatac atagaaaatt gcttctaata tttctctttt gctatacttt ggcggccttc
      901 ctccctttctt gtatgatact ctgaagtgtt tttctattct tgcccattcc ctttcgctta
      961 gatctgttgg atactttttt ctcatcttta ccccgtacta aaatttcaga tattatagcc
     1021 ttttacttat ttccggacaa cctctaagat accgcgaatg aatcgcggta tgacggttcg
     1081 tggcggttat gacgtgttag ctatagctat cttcaaaaat atggttgaat aattgttaat
     1141 aatagtgtat aattattaat atttttaagc actttggcta tggctctttc aaaatttctc
     1201 gatcctcgca atgatttgtg tttcaaaaaa atctttggaa cggaaaaaaa taagaatatc
     1261 ctgacccact ttttgaacga tattttaggg tttactgaga taaatgcaat acaagaagtt
     1321 gagtttctta gcactattat ggatcccgaa gttgcctctg ataaacaaag tattgttgat
     1381 gttctctgca aggattctca cggaaataga tatatagcag aaat
//
LOCUS       JAQZAU010000063          10423 bp    dna     linear   ENV 17-MAR-2023
DEFINITION  MAG: Wolbachia pipientis isolate wEsol contig_37429, whole genome
            shotgun sequence.
ACCESSION   JAQZAU010000063  JAQZAU010000000
VERSION     JAQZAU010000063.1
DBLINK      BioProject: PRJNA929258
            BioSample: SAMN33142973
KEYWORDS    WGS; ENV; Metagenome Assembled Genome; MAG.
SOURCE      Wolbachia pipientis (insect metagenome)
  ORGANISM  Wolbachia pipientis
            Bacteria; Pseudomonadota; Alphaproteobacteria; Rickettsiales;
            Anaplasmataceae; Wolbachieae; Wolbachia.
REFERENCE   1  (bases 1 to 10423)
  AUTHORS   Fiutek,N., Couger,B.M., Roy,S., de la Torre,J.R. and Connor,E.F.
```

```
  TITLE     Genomic assessment of the contribution of the Wolbachia
            endosymbiont of Eurosta solidaginis to gall-induction
  JOURNAL   International Journal Molecular Sciences
REFERENCE   2  (bases 1 to 10423)
  AUTHORS   Fiutek,N., Couger,B.M., Roy,S., de la Torre,J.R. and Connor,E.F.
  TITLE     Direct Submission
  JOURNAL   Submitted (10-FEB-2023) Biology, San Francisco State University,
            1600 Holloway Ave, San Francisco, CA 94132, USA
COMMENT     ##Genome-Assembly-Data-START##
            Assembly Date          :: JUN-2019
            Assembly Method        :: FLYE v. 06-2019
            Genome Representation  :: Full
            Expected Final Version :: Yes
            Genome Coverage        :: 100.0x
            Sequencing Technology  :: Illumina HiSeq; PacBio Sequel
            ##Genome-Assembly-Data-END##
FEATURES             Location/Qualifiers
     source          1..10423
                     /organism="Wolbachia pipientis"
                     /mol_type="genomic DNA"
                     /submitter_seqid="contig_37429"
                     /isolate="wEsol"
                     /isolation_source="galls on Solidago altissima"
                     /host="Eurosta solidaginis MN-2018-EFC"
                     /db_xref="taxon:955"
                     /environmental_sample
                     /country="USA: Cowling Arboretum, Carleton College,
                     Northfield, MN"
                     /lat_lon="44.48 N 93.13 W"
                     /altitude="280 m"
                     /collection_date="2018-11"
                     /metagenome_source="insect metagenome"
                     /note="metagenomic"
     gene            complement(298..474)
                     /locus_tag="wEsol_00670"
     CDS             complement(298..474)
                     /locus_tag="wEsol_00670"
                     /codon_start=1
                     /transl_table=11
                     /fullproduct="qcoverage 100 , hcoverage 100 , similarity
                     100 , identity 100 , evalue 5.86e-32 , alnlength 58 ,
                     Bacteria , Proteobacteria , WP_179945710.1 MULTISPECIES:
                     hypothetical protein [Wolbachia]"
                     /translation="MKKKAKEALGLLSALKYWRFLCFKRLTSEIQPLLIATNLRCKCL
                     RNLLSRKKDKESPS"
                     /product="hypothetical protein"
                     /besthit="qcoverage 100 , hcoverage 100 , similarity 100
                     , identity 100 , evalue 5.86e-32 , alnlength 58 ,
                     Bacteria , Proteobacteria , WP_179945710.1 MULTISPECIES:
                     hypothetical protein [Wolbachia]"
     gene            803..2146
                     /locus_tag="wEsol_00671"
     CDS             803..2146
                     /locus_tag="wEsol_00671"
                     /codon_start=1
                     /transl_table=11
                     /fullproduct="qcoverage 100 , hcoverage 100 , similarity
                     100 , identity 100 , evalue 1.63e-313 , alnlength 447 ,
                     Bacteria , Proteobacteria , WP_141457221.1 MULTISPECIES:
                     DNA repair protein RadA [unclassified Wolbachia]"
```

/cog="Sms COG1066 456 Predicted ATP-dependent serine
protease"
/pfam="Pfam-B_5795 RecA AAA_25 DnaB_C KaiC AAA_16 GvpD
AAA_24 AAA_22 AAA DnaB_C KaiC ChlI Lon_C"
/tigr="TIGR00416 sms 454 DNA repair protein RadA"
/product="DNA repair protein
RadA"
/translation="MKTVYVCQSCGHSTGRWVGKCIACDNWDTIVEEIAVKTGKRSIS
APILIKTLSGSEIITPSRFLTGIEELDRVFGGGIVQGASILIGGEPGIGKSTLLLRIA
ANVVQLSSFECLYVSGEESLEQVSLRAKRLKINEPKIKLLSTVSLTDVVATIKENKSI
KFLVIDSIQTMYDSRITSAPGTVTQVRTCAHELTILAKQYGVSLLIVGHITKDGQIAG
PKTLEHMVDTVLYFEGENSNQYRILRTIKNRFGPANEIGVFEMSEAGLVPVDNPSSLF
LMAHDREVVGSAVFAGIEGSRPILMEVQALIAGTNMATPRRAVVGWDINRLAMIIAVL
NARCKMFLHDKEVYLNIAGGLKIQEPSADLAVAASLISSVVNLPLPTSSIICGEVALS
GEIRNVSHADLRLKEAQKLGFKKAIMPKNGNYSSHDIEIIKLGHVRELREVFNYN"
/besthit="qcoverage 100 , hcoverage 100 , similarity 100
, identity 100 , evalue 1.63e-313 , alnlength 447 ,
Bacteria , Proteobacteria , WP_141457221.1 MULTISPECIES:
DNA repair protein RadA [unclassified Wolbachia]"
gene complement(2133..3044)
/locus_tag="wEsol_00672"
CDS complement(2133..3044)
/locus_tag="wEsol_00672"
/codon_start=1
/transl_table=11
/fullproduct="qcoverage 100 , hcoverage 100 , similarity
100 , identity 100 , evalue 8.88e-211 , alnlength 303 ,
Bacteria , Proteobacteria , WP_015589234.1 MULTISPECIES:
signal recognition particle-docking protein FtsY
[unclassified Wolbachia]"
/cog="FtsY COG0552 340 Signal recognition particle
GTPase"
/pfam="SRP54_N Pfam-B_5968 Pfam-B_12884 AAA_30 Zeta_toxin
AAA_22 ArgK SRP54 AAA_14 AAA_19 cobW MobB CbiA CmcI
IstB_IS21"
/tigr="TIGR00064 ftsY 279 signal recognition
particle-docking protein FtsY"
/product="signal recognition
particle-docking protein FtsY"
/translation="MSLFSNLYKGLLKTSSRFSDGVKSIFSGKKKLDQSLLDELEELL
ISMDIGHKTSKLIIDRLTSIKFDKEVEHNIISQQLMNEIEAILSPVVQPLILNKKPHI
IMVCGVNGNGKTTTIGKLAYKYKEMGKSVMLVACDTFRAAASEQLNIWAERSDCLIVT
GEYGSDSASVAYRAVSQAIKDNVDVVLIDTAGRLQNNVNLMEELSKIYRTIKKLDDTA
PHDVILVLDATTGQNAYSQLEAFSKMVHVTGLIVTKLDGTAKGGVVIGLAEAYKVKLH
AIGIGENIEDLREFTSKEFAEALFNCN"
/besthit="qcoverage 100 , hcoverage 100 , similarity 100
, identity 100 , evalue 8.88e-211 , alnlength 303 ,
Bacteria , Proteobacteria , WP_015589234.1 MULTISPECIES:
signal recognition particle-docking protein FtsY
[unclassified Wolbachia]"
gene 3248..3631
/locus_tag="wEsol_00673"
CDS 3248..3631
/locus_tag="wEsol_00673"
/codon_start=1
/transl_table=11
/fullproduct="qcoverage 100 , hcoverage 100 , similarity
100 , identity 100 , evalue 9.86e-85 , alnlength 127 ,
Bacteria;Eukaryota , Proteobacteria;Arthropoda ,
WP_007548853.1 MULTISPECIES: Fe-S cluster assembly

```
                     scaffold IscU [Wolbachia]"
                     /cog="IscU COG0822 150 NifU homolog involved in Fe-S
                     cluster formation"
                     /pfam="NifU_N Pfam-B_829"
                     /tigr="TIGR01999 iscU 124 FeS cluster assembly scaffold
                     IscU"
                     /product="Fe-S cluster
                     assembly scaffold IscU"
                     /translation="MSYNEKILDHYENPRNVGSLDKNDPNVGTGLVGAPSCGDVMKLQ
                     IKVNDKGVIEDAKFKTFGCGSAIASSSLLTEMIKGRTISDVTQIKNTQIVEELSLPPV
                     KIHCSVLAEDAIKAAIHDYQSKQKN"
                     /besthit="qcoverage 100 , hcoverage 100 , similarity 100
                     , identity 100 , evalue 9.86e-85 , alnlength 127 ,
                     Bacteria;Eukaryota , Proteobacteria;Arthropoda ,
                     WP_007548853.1 MULTISPECIES: Fe-S cluster assembly
                     scaffold IscU [Wolbachia]"
     gene            3637..4050
                     /locus_tag="wEsol_00674"
     CDS             3637..4050
                     /locus_tag="wEsol_00674"
                     /codon_start=1
                     /transl_table=11
                     /fullproduct="qcoverage 100 , hcoverage 100 , similarity
                     100 , identity 100 , evalue 4.21e-93 , alnlength 137 ,
                     Bacteria , Proteobacteria , WP_174516985.1 iron-sulfur
                     cluster assembly accessory protein [Wolbachia
                     endosymbiont of Cardiocondyla obscurior]"
                     /cog="sufA COG0316 110 Fe-S cluster assembly scaffold
                     protein"
                     /pfam="Fe-S_biosyn"
                     /tigr="TIGR00049 TIGR00049 106 iron-sulfur cluster
                     assembly accessory protein"
                     /product="iron-sulfur cluster assembly
                     accessory protein"
                     /translation="MSEYQEVGGGETVKKNRKLITITAKAVERIRYLLDQKKHTNLEK
                     PEEAIGIRVLIKQKGCSGLKYDIEYAYDTRPLESIIEENCSDDQKVKVLIDPKSVMFI
                     LGSEMDYVEEKFSSGFVFKNPNEKGKCGCGESFHV"
                     /besthit="qcoverage 100 , hcoverage 100 , similarity 100
                     , identity 100 , evalue 4.21e-93 , alnlength 137 ,
                     Bacteria , Proteobacteria , WP_174516985.1 iron-sulfur
                     cluster assembly accessory protein [Wolbachia
                     endosymbiont of Cardiocondyla obscurior]"
     gene            4043..4501
                     /locus_tag="wEsol_00675"
     CDS             4043..4501
                     /locus_tag="wEsol_00675"
                     /codon_start=1
                     /transl_table=11
                     /fullproduct="qcoverage 100 , hcoverage 100 , similarity
                     100 , identity 100 , evalue 6.20e-100 , alnlength 152 ,
                     Bacteria , Proteobacteria , WP_015589237.1 MULTISPECIES:
                     hypothetical protein [unclassified Wolbachia]"
                     /pfam="DnaJ HSCB_C"
                     /tigr="TIGR00714 hscB 156 Fe-S protein assembly
                     co-chaperone HscB"
                     /product="hypothetical protein"
                     /translation="MSSSYFTLFEIEPTFNISFDELEKKYIELSKTDINERQSQNMEN
                     INRAYQILKSPLKRAEHLLDFFDVKSQKDHLNSEILSESMEIREYLLDCDDLQFASRM
                     IDEKIKDCVKNLINTFAIKNFDGAATQVLRLKYLYKSFEEVKKNATNSNF"
                     /besthit="qcoverage 100 , hcoverage 100 , similarity 100
```

                     , identity 100 , evalue 6.20e-100 , alnlength 152 ,
                     Bacteria , Proteobacteria , WP_015589237.1 MULTISPECIES:
                     hypothetical protein [unclassified Wolbachia]"
     gene   4479..6227
                     /locus_tag="wEsol_00676"
     CDS   4479..6227
                     /locus_tag="wEsol_00676"
                     /codon_start=1
                     /transl_table=11
                     /fullproduct="qcoverage 100 , hcoverage 100 , similarity
                     100 , identity 100 , evalue 0.0 , alnlength 582 ,
                     Bacteria , Proteobacteria , WP_141457223.1 MULTISPECIES:
                     Fe-S protein assembly chaperone HscA [unclassified
                     Wolbachia]"
                     /cog="DnaK COG0443 579 Molecular chaperone"
                     /pfam="HSP70 MreB_Mbl Pfam-B_178 Pfam-B_4180"
                     /tigr="TIGR01991 HscA 600 Fe-S protein assembly chaperone
                     HscA"
                     /product="Fe-S protein
                     assembly chaperone HscA"
                     /translation="MQPIQISEPNSSEVIFGIDLGTTNSLIAMVNKAGNVEIFEDEQG
                     RELLPSALSYENNTLKTGYDVGQNAIYSIKRLMGKSVEELNKEGVNFEIDHESEKVIR
                     IKCAEEKYLTPVEISAEILKALCERVKKSKGLEVKKAVITVPAYFDDSARNATKYAAK
                     LAGIEVLRLVNEPTAAALSYSIEKNNSSGIYAVYDLGGGTFDISILKLHQGVFQVLAV
                     GGDTKLGGDDFDHLLSSIVIDKYREKTAKQLNSLLIESRSVKEYLSKNTSGIFEFSIN
                     GKPFECEITREEFEQAISSLVNKTINIVTRTISDIDLKIDNIKGVILVGGATRTPLVQ
                     NSLVKLFGNKVLNDVDPDKAVAIGAALQAHDLTSSSKDRNILLDVLPLSLGIETMGGI
                     VEKIIPRNTPLPVSETKEFTTYFDGQTAMKIHVCQGEREMIEDNKSLAQFELKGIPPL
                     PAGSARVEIEFTVNVDGILTVTAREKTTGIEQTVEVNSSFGLSEADVQNMVNQSINSF
                     DEDMKARSLAGAKINGNKLIHLVENNNNLSTDQKLKNLLQNAKNALQGNDLNEINNTI
                     AELENSSLELCELTNR"
                     /besthit="qcoverage 100 , hcoverage 100 , similarity 100
                     , identity 100 , evalue 0.0 , alnlength 582 , Bacteria ,
                     Proteobacteria , WP_141457223.1 MULTISPECIES: Fe-S
                     protein assembly chaperone HscA [unclassified Wolbachia]"
     gene   complement(6235..6981)
                     /locus_tag="wEsol_00677"
     CDS   complement(6235..6981)
                     /locus_tag="wEsol_00677"
                     /codon_start=1
                     /transl_table=11
                     /fullproduct="qcoverage 100 , hcoverage 100 , similarity
                     100 , identity 100 , evalue 6.91e-176 , alnlength 248 ,
                     Bacteria , Proteobacteria , WP_010962911.1 MULTISPECIES:
                     signal peptidase I [Wolbachia]"
                     /cog="LepB COG0681 166 Signal peptidase I"
                     /pfam="Pfam-B_817 Peptidase_S24 Peptidase_S26
                     Peptidase_S26"
                     /tigr="TIGR02227 sigpep_I_bact 162 signal peptidase I"
                     /product="signal peptidase
                     I"
                     /translation="MEELSKNWVIRTGRFLSSLFFLLLIALSIRSFLFEPFHIPSGSM
                     KSTLLEGDYIFTSKYSYGYSKHSFPFSPNIFSGRIFYTPPKRGDIVVFKPTRNDSIRF
                     VKRVIGTPGDKVQMIEGELYLNDQKVERRQIESFFDYESNRNIPRYIETLLSGKEHEI
                     LVDDISNKLSYNTPVYYVPNDQFFVMGDNRNNSLDSRFPEVGFVPMENIIGRVSIVGL
                     SFKLGKVDWLPFNFRLPVALRLNRVLHKVV"
                     /besthit="qcoverage 100 , hcoverage 100 , similarity 100
                     , identity 100 , evalue 6.91e-176 , alnlength 248 ,
                     Bacteria , Proteobacteria , WP_010962911.1 MULTISPECIES:
                     signal peptidase I [Wolbachia]"

```
gene            complement(7923..8810)
                /locus_tag="wEsol_00678"
CDS             complement(7923..8810)
                /locus_tag="wEsol_00678"
                /codon_start=1
                /transl_table=11
                /fullproduct="qcoverage 100 , hcoverage 100 , similarity
                100 , identity 100 , evalue 3.76e-208 , alnlength 295 ,
                Bacteria , Proteobacteria , WP_015589239.1 MULTISPECIES:
                aspartate carbamoyltransferase catalytic subunit
                [Wolbachia]"
                /cog="PyrB COG0540 316 Aspartate carbamoyltransferase,
                catalytic chain"
                /pfam="OTCace_N OTCace"
                /tigr="TIGR00670 asp_carb_tr 304 aspartate
                carbamoyltransferase"
                /product="aspartate
                carbamoyltransferase catalytic subunit"
                /translation="MNKRRNLLNISDLTVDDVENITKLANQYLKKEVANSHILENKTV
                INLFFEDSTRTLASFEIAAKSLGANVVTLPIRSSSINKGEDLKDMIKTLNAMNPDYII
                IRHKSSGIINTLAKYVNCSLINAGDGSSEHPTQALADYLVISNHKKQIKDLKVVICGD
                ILHSRVARSNIRLLKMFGAEINLVAPPTLICKHFPEVDSVHYSLIEGIKDADVIMLLR
                LQKERMNNSSSEKEYFHLYGLDLQKLSYAKPDAIVMHPGPINRGIEISSDVADCVILQ
                QVEFGLAIRKAVLHYYRPC"
                /besthit="qcoverage 100 , hcoverage 100 , similarity 100
                , identity 100 , evalue 3.76e-208 , alnlength 295 ,
                Bacteria , Proteobacteria , WP_015589239.1 MULTISPECIES:
                aspartate carbamoyltransferase catalytic subunit
                [Wolbachia]"
gene            complement(9031..10056)
                /locus_tag="wEsol_00679"
CDS             complement(9031..10056)
                /locus_tag="wEsol_00679"
                /codon_start=1
                /transl_table=11
                /fullproduct="qcoverage 100 , hcoverage 100 , similarity
                100 , identity 100 , evalue 1.12e-239 , alnlength 341 ,
                Bacteria , Proteobacteria , WP_010082338.1 MULTISPECIES:
                Fe(3+) ABC transporter substrate-binding protein
                [Wolbachia]"
                /cog="AfuA COG1840 299 ABC-type Fe3+ transport system,
                periplasmic component"
                /pfam="SBP_bac_11 Phosphonate-bd SBP_bac_1 SBP_bac_8
                SBP_bac_6"
                /tigr="TIGR03261 phnS2 334 putative
                2-aminoethylphosphonate ABC transporter, periplasmic
                2-aminoethylphosphonate-binding protein"
                /product="Fe(3+) ABC
                transporter substrate-binding protein"
                /translation="MKKAFIFTSLIAIILIVITYLYRNDGTNDSQVVNVYSSRKEELV
                RTLFDEFTKNTGIKVRYIIDDYSQLLSRMENGGEADLFLTADAINLILAKKRGLLSQV
                DSEVLKSVIPARFRDSEDYWFGLTKRARILVYNKESVDPKDLSTYEDLANEKWKGKIL
                VRSSTSPYNRSLIAFMIANNGFEKTKEWVSGIVSNMARKPSGGDTDQIYAVAAGEGDV
                AIVNSYYFARILSSENKKNVADKLGAFFPNRNDHGVMVNISGAAVTKNAKNRENAIVL
                LEFLVSKQAQELYAKKNQEYPIIEGVETSDILKSWGNYSQSDLPLSELEKHLFEAVMI
                ADECKWK"
                /besthit="qcoverage 100 , hcoverage 100 , similarity 100
                , identity 100 , evalue 1.12e-239 , alnlength 341 ,
                Bacteria , Proteobacteria , WP_010082338.1 MULTISPECIES:
                Fe(3+) ABC transporter substrate-binding protein
```

[Wolbachia]"

ORIGIN

```
        1 tacataacca tataacattg ggaatagaaa caaaaaactt gcttgacacc cttcgccagc
       61 cccctttatca taaaactgaa gctattgtat ttgctttcgc cgatctgcag attaaaaggt
      121 aaggattact taatgtatcg gcgtcttatg ttcaattttt tgcagtatat agatactgta
      181 tgtctttaca aaacttcatc tacatctaga tttttatcta aataagctga acgcgcttat
      241 aaagcgttac aagacatcaa aaaatgccaa tactcgacag agatagtaaa agactagcta
      301 actcggggat tctttgtctt tttttctgct tagtaaattt cttaaacatt tgcagcgtaa
      361 gttagttgca attaaaagcg gctgaatctc gcttgtcaag cgtttaaaac ataaaaaacg
      421 ccaatatttt aaagcggata ataaccccag ggcttctttt gccttttttt tcatttggta
      481 aatttcttaa tgtttatggc taaacgacaa tcgtcatccc gctacgtgtt agcggctaag
      541 agataccgcg gcggtatgac ggtttgcggc ggcatgacct cgtcattccc ctacgtgtta
      601 gcgggatcta gagataccgt gacggtatga cggttcgcgc ggtggcatga cctcgtcatc
      661 ccgctacttg ttagcgggat ctagagatac cgcgaatgaa tcgcggtatg acatagggaa
      721 ccctttcttg ggttagctat agatttactt cgcatattca gctaattttt attacaatta
      781 caacaaaata attttggctt tgatgaaaac tgtatatgta tgtcaatcct gtggtcacag
      841 cactggtagg tgggtaggga agtgcatcgc atgtgacaat tgggatacaa ttgttgagga
      901 aatagcagta aaaacgggca aaagaagtat atctgcacca attttaataa agacgttatc
      961 gggtagcgaa attatcactc caagtcgttt tttaacagga atagaagaat tagatagagt
     1021 ttttggtggg ggtatcgttc aaggtgctag cattttaatt ggtggcgaac ctggtattgg
     1081 aaaatccacc cttttgctta gaattgcagc taatgttgtg caactttcct cttttgaatg
     1141 tctttatgta tccggcgagg agtctttaga gcaggtgagt ctcagagcaa agcgtcttaa
     1201 gataaatgaa cctaaaatta agcttttatc tacagtgtct ttaactgatg tagtagcaac
     1261 aataaaggaa aacaaaagta ttaaattctt agtaattgac tctatacaaa caatgtatga
     1321 tagcagaatt acatcggcac caggaactgt aactcaggtt cgtacttgcg cacatgaatt
     1381 aactattctt gcgaaacaat atggtgtttc tcttttaata gttggtcata taactaagga
     1441 tggacaaata gctggaccca aaactctgga gcatatggtg gatacggtat tatattttga
     1501 aggtgaaaat agcaatcaat atcgcatttt gcgtactatt aaaaatagat ttggccctgc
     1561 aaatgaaatt ggtgtttttg agatgtctga agcaggactt gtgcctgttg ataatccatc
     1621 atcattgttt ctgatggccc atgacagaga agtagttggc agcgccgtat ttgcaggaat
     1681 tgaaggttca agaccaattt taatggaagt gcaagcatta atagcaggga ctaatatggc
     1741 aaccccaaga agggcagtag ttgggtggga tattaatcga ttggctatga tcattgcggt
     1801 gctaaatgct cgttgcaaaa tgtttttgca tgataaagag gtatacctaa acattgcagg
     1861 gggactcaaa atacaggaac catcggctga tcttgctgtt gctgcttccc ttatttcaag
     1921 tgtagtaaat ttaccgctgc caacttcttc aataatctgc ggtgaagttg cactttcagg
     1981 agaaattagg aatgtctcgc atgctgatct cagactaaaa gaagcacaaa agttaggatt
     2041 caaaaaagca attatgccta aaaatggcaa ttattcttct catgatattg agataattaa
     2101 gcttggtcat gtaagggaat taagagaagt ctttaattac aattaaataa tgcttcagca
     2161 aattctttac tagtaaactc ccttaagtcc tctatatttt cgccaattcc tatagcatgt
     2221 aactttacct tataagcttc tgcaagtcca attactactc cgcccttagc ggtgccatct
     2281 agctttgtta cgattagccc ggtaacatgg accatcttac taaatgcctc taattggcta
     2341 taagcatttt ggccggtagt tgcgtcaaga actaaaataa cgtcatgagg ggcagtatca
     2401 tccaatttct ttatcgttct atatattttt gataactcct ccataaggtt cacattattt
     2461 tgcagccttc ctgctgtgtc aattagaaca acatcaacgt tatcttttat agcttgactt
     2521 acagctctat atgccacact tgcagagtcg ctaccgtact ctccagtaac aatcaagcaa
     2581 tcagaacgtt ctgcccaaat gtttaactgc tcactagcag cagctctaaa tgtgtcacac
     2641 gcaacaagca taacggattt tcccatctcc ttatatttat atgcaagctt acctatagtc
     2701 gtggttttac cattaccgtt tactccgcat accattatta tatgtggttt tttatttaaa
     2761 attaacggct gtacaactgg gctcaatata gcttctattt cgttcattaa ctgctgcgag
     2821 ataatattat gttcaacttc cttgtcaaac ttgatgcttg tgagcctatc gataatcaac
     2881 ttagaagttt tatgaccaat gtccatgcta attagcaatt cctctaattc atctaaaagc
     2941 gactgatcta attttttttt gccagaaaaa atacttttca ccccgtcgct aaagcgagaa
     3001 gaagttttta acaagccttt ataaaggtta ctaaacaaac tcaagggaat actccttttt
     3061 aaattataaa cttaatgaca acatagagac gctgattgta aagaaataca cgtattatgt
     3121 gtatttgtaa agttaaacaa tcgttttacg ggaattttga aagtaaaaat cacgtaaaac
     3181 gattgttttc ctttagtttt taacctaaat atgttataat gtaaaatagc agtttatagg
     3241 taattttatg agttataatg agaaaatttt agatcattat gaaaatccaa ggaatgttgg
     3301 ctctctggat aaaaatgatc caaatgttgg tactggttta gttggtgcac catcgtgcgg
     3361 tgatgtaatg aaattgcaga taaaggtcaa tgacaaaggt gttattgaag atgcaaaatt
     3421 taaaactttt ggttgtggtt ctgccattgc ttcaagttcc ttgttaacag agatgataaa
```

3481 gggaagaacc atcagtgacg ttacacagat aaagaatact caaatagtgg aagagttgtc
3541 tttacctcca gtgaagatac actgttcggt acttgctgaa gatgctataa aggctgctat
3601 tcatgattat caaagtaagc aaaagaatta agaagcatga gtgaatatca agaagtaggc
3661 ggaggtgaaa ctgtaaaaaa gaatagaaaa cttatcacta taactgcgaa agcagtagaa
3721 aggataaggt atttattgga ccaaaagaag catactaacc ttgagaaacc ggaagaagca
3781 ataggaataa gagtgctaat taagcaaaaa ggatgttctg gtttaaaata tgatattgaa
3841 tatgcgtatg atactcgtcc tttagagtcg ataattgagg aaaactgcag tgatgaccaa
3901 aaagttaaag tgttgatcga tccaaaatcc gtcatgttta ttcttggctc tgaaatggat
3961 tatgtagaag aaaaattttc atcgggtttt gttttcaaaa atcctaatga gaaaggaaaa
4021 tgtggttgtg gagagagttt tcatgtctag cagctatttt acattgtttg aaattgaacc
4081 aactttcaat atcagctttg atgagttaga gaaaaaatat attgagctaa gcaaaactga
4141 tataaatgaa agacagtccc aaaacatgga aaatattaac agagcttatc agatactaaa
4201 gtcaccgcta aaacgtgccg agcatttgct agattttttt gatgtgaaaa gccaaaagga
4261 ccacttaaat tctgaaatat taagtgaatc aatggaaata agagaatatt tgctggactg
4321 cgatgattta caatttgcaa gcaggatgat tgatgaaaaa ataaaagatt gcgttaaaaa
4381 cctaattaac acttttgcta taaagaattt tgatggagct gctacacaag tcttaagatt
4441 gaaatattta tataagtcat ttgaggaggt gaaaaagaat gcaaccaatt caaatttctg
4501 aaccaaattc aagtgaagta atttttggta tagatcttgg tactaccaat tctttgattg
4561 ctatggtaaa taaagctggt aacgtggaga tatttgaaga tgaacaagga agagaactgc
4621 tgccttctgc tctttcatat gaaaataata cattaaaaac tggctacgat gtcggtcaaa
4681 atgcaatcta ctcaataaag cgtttaatgg gtaaaagtgt tgaagaatta aataaagaag
4741 gcgtcaattt tgaaatagat catgaaagcg aaaaagttat tagaataaaa tgcgcagagg
4801 aaaagtatct tactcctgtt gagatttctg ctgagatatt aaaagcttta tgtgagaggg
4861 taaaaaaagtc taagggactg gaagtaaaaa aagcagtgat tactgtgcca gcttactttg
4921 acgattcagc acgtaacgca actaaatatg cggcaaaatt agctggaata gaagttcttc
4981 gcctcgttaa cgagccaacc gctgcagcgc tttcttactc cattgaaaag aacaatagca
5041 gtggaatata tgcagtgtat gaccttggtg gcggaacgtt cgatatttcg atattgaaat
5101 tgcatcaagg agtgtttcaa gttcttgcag ttggtggtga caccaaactc ggcggtgatg
5161 attttgatca tttgctgagt tcaattgtga ttgataagta tagagaaaag acggccaagc
5221 aactaaattc cttacttatc gaatcacgct ctgtgaagga atatctgagc aaaaacacct
5281 caggcatttt tgaattcagc attaacggca agccatttga atgtgaaatt accagagaag
5341 aattcgagca ggcaatcagt tctttggtca ataagactat caatatagtc actcgtacta
5401 taagcgacat agatcttaaa attgataata taaaaggagt aattttagtt ggtggtgcaa
5461 ctagaacacc attggttcaa aattcactag tcaaactctt tggaaataaa gtgctgaatg
5521 acgtggatcc agataaagca gttgccattg gggcagctct gcaagctcat gatttaactt
5581 cgagctcaaa agataggaat attctcctgg atgttctacc tttatcactt ggcatagaaa
5641 ctatgggagg catagttgaa aaaatcatac caagaaatac gccactacca gtttcagaaa
5701 caaaagagtt tacaacttat tttgatgggc aaacagcaat gaaaattcac gtttgtcagg
5761 gagaacgtga aatgatagag gataacaaat ctctagcgca gtttgaacta aaaggtatac
5821 cgccactacc tgcaggttct gcaagagttg aaatagaatt tacagttaac gttgacggaa
5881 tcttgactgt tactgcaaga gaaaaaacta ctggaatcga gcagacagtt gaagtaaatt
5941 caagttttgg cttgagtgaa gctgatgttc aaaatatggt taaccagtca ataaacagct
6001 ttgatgaaga tatgaaggct cgttcccttg caggcgctaa aattaatgga aataaactca
6061 tacacttggt tgaaaacaat aataaccttt ccactgatca aaagttgaaa aacctattac
6121 aaaatgcaaa aaacgcttta caaggcaatg acttgaatga aattaacaac actatcgccg
6181 agttagaaaa ttcttcttta gagttgtgtg aattaacaaa taggtagaaa aagcttacac
6241 aaccttgtgc aataccctat ttaatctcaa agcaacaggt aacctgaaat taaatggtaa
6301 ccaatcgacc ttccccagtt taaacgataa accaactata cttacacgcc caatgatatt
6361 ttccattggt acaaaaccaa cttcaggaaa cctgctatcc aaagaattat ttctattgtc
6421 tcccataacg aaaaattgat cgttaggtac ataataaacc ggagtgttat atgatagctt
6481 attagaaatg tcatctacca aaatctcatg ttccttgcca cttaaaagcg tttctatata
6541 tcttggtatg ttacgatttg actcataatc aaaaaaactt tcaatttgcc ttcgctctac
6601 tttctgatca tttaggtata attctccctc tatcatttgc actttgtcac ctggtgttcc
6661 tattactcgc ttaacaaacc tgatgctgtc atttcttgtg ggtttgaaaa ctactatgtc
6721 gccacgcttt ggaggggtat aaaaaatcct gccgctaaaa atgtttggag aaaatggaaa
6781 tgagtgttta ctgtaaccgt atgaatattt actagtaaaa atgtaatccc cttcaagtag
6841 ggtgcttttc attgaaccag aaggtatatg aaatggctcg aataaaaaac tacgtattga
6901 cagcgcaatc aacagtaaaa aaaacaatga agataaaaat cttcccgttc ttatcaccca
6961 gtttttgctt agttcttcca actttgtttt tcttatagat taagcttatt ataatacata
7021 aattaaaaaa tggtaatatg aaattttact tgtgaatgtt attcctctat tcaaaacgat

```
     7081 tttgaaaagg cggttcattt ctttcaacgg tgtcatccaa gtagcgtgac acataactgt
     7141 acgaacattg caatttgcag gtggcagatg aaagtagctg atgcacatct gtacgaacat
     7201 ttgcttttgt taaggtaata tggtgttatg aaagtagctg acactggaat ggctttgttg
     7261 catcgctact tatgaaaggc taactatagt taggattata agcaacttat tgaaaatctt
     7321 gtttttttgc aatcaatctg atcaaattta agaatagtaa tttgttattt atattaataa
     7381 atttaattct attgaaaata gctaaagcat tgaaattctt gaattttagc cggattggta
     7441 aatggtagtg aaatttcttt tactcaaatt taggtattca ttgaccgttc ttgttataaa
     7501 taccagaata ataagctaat gatattccct atctttttta tctttaatcc atcatagcta
     7561 gcgatgcaac aaagcccact ggaatccagg ttgtaaccgt tcacggattg tgagaatgaa
     7621 taaagaacta agcagtcgta actttggaa gggattttgg ccattgagac gtgatgtcaa
     7681 aaatatcgac aacattcttt ccacaaatct ttctccatgt tccgaccagg tgaaaaagct
     7741 gtttttacga tagccgtcta atttgccgtt ctgcaaggtt gttgattgac ttcaggcttt
     7801 cgtaaaaact tccacaccat atcctcggcc ttaacatctt aatggccatt ctcaaaccat
     7861 ttacaagcct tgtcatttca tgcccccccc tttttgcatt agttgtgtag catcttttaa
     7921 tattagcaag gcctataata gtgcaacact gccttacgta ttgccaatcc aaattctact
     7981 tgctgtaaaa taacacaatc tgctacatcg ctgctaatct caatccctct atttattggc
     8041 cctgggtgca taacaatcgc atctggtttt gcgtatgata gcttttgtaa atcaagtcca
     8101 tacaaatgaa aatattcttt ctctgaagaa ctattattca tgcgctcttt ctgcaatctc
     8161 aaaagcataa ttacgtcagc atcctttata ccttcaatta atgaataatg gactgaatct
     8221 acttcaggaa aatgcttaca aatcaaagtt ggtggtgcga ccaagtttat ctctgctcca
     8281 aacattttta gcaatcttat atttgatctt gcaactctac tgtgcagaat atctccacat
     8341 atcacaactt tgaggtcctt tatttgcttt ttgtgattgc tgattacaag atagtctgca
     8401 agagcttgag ttggatgttc actacttcca tcgcctgcat tgattagcga gcagttaaca
     8461 tactttgcca gtgtattaat gataccacta cttttatgcc tgattattat gtaatcagga
     8521 ttcattgcat ttaatgtttt gatcatgtct tttagatctt ctcccttgtt aatagaagaa
     8581 gatcttattg gcaaagttac aacatttgct ccaaggcttt ttgctgctat ttcaaaagac
     8641 gcaagtgtac gcgttgaatc ttcaaagaat aaatttatta ctgtcttatt ttccaagata
     8701 tgactatttg caacttcttt tttaaggtac tggttggcca acttagttat attttctaca
     8761 tcatcgactg taaggtctga gatgtttaac aaattccttc tcttgttcat aatttccttt
     8821 taacatgcaa ttgttatcca actacttgaa ccaattctac tttcttatta tcgtttttca
     8881 atagacctct tgtgcagcaa ttgttttggt ggaagaacgg aaaaagatag taaagtcaaa
     8941 attaagttag aaggaagaaa aagttcagca agttaagagt aaggtacatt agcataaatc
     9001 tttcatccca atgtttagtg gcactggaga ttacttccac ttacactcat ccgctatcat
     9061 aacagcctca aaaagatgct tctcaagttc acttagaggc aaatcacttt gtgaataatt
     9121 tccccaagat tttagtatat cggaagtttc aacgccttca ataataggat attcttggtt
     9181 ttttttagcg tataactctt gagcctgctt gcttactaaa aattccaata agactatagc
     9241 attttccctg ttttttgcgt tttttgttac tgctgcacca ctgatgttta ccattacacc
     9301 gtgatcgttg cgattaggaa aaaaagcccc tagcttgtct gcaacattct ttttattttc
     9361 tgatgaaaga attctcgcaa agtagtagct gtttactatt gcaacatctc cctcacccgc
     9421 tgctacggcg taaatttgat cagtatcacc accacttggt tttcttgcca tattgctcac
     9481 aattccgctt acccattctt tcgtcttttc aaaaccatta tttgcaatca taaaagcaat
     9541 taacgatctg ttatatggac ttgtagaaga acgcactaat atttttcctt tccatttttc
     9601 atttgctaaa tcttcataag tacttaagtc cttagggtct actgactctt tattgtaaac
     9661 caatatccta gctctttttg taagaccaaa ccaataatct tcactgtctc taaatcttgc
     9721 aggtataaca ctttttaaaa cctctgaatc tacttgagat aaaagtcctc tttttttttgc
     9781 tagaattaag ttgattgcat cagcagtcaa aaataagtca gcttcaccac cgttttccat
     9841 tcgtgaaagt agctgagaat aatcatcaat gatgtaacgt actttaatac ctgtattttt
     9901 cgtgaattca tcaaacaaag tacgtactaa ttcttctttg cgcgatgaat aaacatttac
     9961 cacttgtgag tcattagttc cgtcgtttct gtataaataa gtaataacta tcaatataat
    10021 tgctattaag gatgtgaata taaaggcttt tttcatattg gataaaaaat aacttctttc
    10081 agttaaagtt accaagatgg tacaacttta gcaagtgtaa agttcgttag ataaatcata
    10141 aaaacttcct tgacaagctt cgccagcccc cttattataa cactgaagct atttatttat
    10201 cttcgcaatc tctgcagtgg attaatgaca aaaaacttaa tgtatttggc gtaaatcatg
    10261 cttaattttt tgcattatgt gcaccttatg tctttataaa atttctaggt ttttacctaa
    10321 tataagctga aatgcgctat ttaaaacatt aaaaaacgcc aacttaaaaa atggatagtg
    10381 aataactagc taccctaggg tttcttttgc cttttttttct gtt
//
LOCUS       JAQZAU010000064           8664 bp    dna     linear   ENV 17-MAR-2023
DEFINITION  MAG: Wolbachia pipientis isolate wEsol contig_37626, whole genome
            shotgun sequence.
```

```
ACCESSION   JAQZAU010000064 JAQZAU010000000
VERSION     JAQZAU010000064.1
DBLINK      BioProject: PRJNA929258
            BioSample: SAMN33142973
KEYWORDS    WGS; ENV; Metagenome Assembled Genome; MAG.
SOURCE      Wolbachia pipientis (insect metagenome)
  ORGANISM  Wolbachia pipientis
            Bacteria; Pseudomonadota; Alphaproteobacteria; Rickettsiales;
            Anaplasmataceae; Wolbachieae; Wolbachia.
REFERENCE   1  (bases 1 to 8664)
  AUTHORS   Fiutek,N., Couger,B.M., Roy,S., de la Torre,J.R. and Connor,E.F.
  TITLE     Genomic assessment of the contribution of the Wolbachia
            endosymbiont of Eurosta solidaginis to gall-induction
  JOURNAL   International Journal Molecular Sciences
REFERENCE   2  (bases 1 to 8664)
  AUTHORS   Fiutek,N., Couger,B.M., Roy,S., de la Torre,J.R. and Connor,E.F.
  TITLE     Direct Submission
  JOURNAL   Submitted (10-FEB-2023) Biology, San Francisco State University,
            1600 Holloway Ave, San Francisco, CA 94132, USA
COMMENT     ##Genome-Assembly-Data-START##
            Assembly Date          :: JUN-2019
            Assembly Method        :: FLYE v. 06-2019
            Genome Representation  :: Full
            Expected Final Version :: Yes
            Genome Coverage        :: 100.0x
            Sequencing Technology  :: Illumina HiSeq; PacBio Sequel
            ##Genome-Assembly-Data-END##
FEATURES             Location/Qualifiers
     source          1..8664
                     /organism="Wolbachia pipientis"
                     /mol_type="genomic DNA"
                     /submitter_seqid="contig_37626"
                     /isolate="wEsol"
                     /isolation_source="galls on Solidago altissima"
                     /host="Eurosta solidaginis MN-2018-EFC"
                     /db_xref="taxon:955"
                     /environmental_sample
                     /country="USA: Cowling Arboretum, Carleton College,
                     Northfield, MN"
                     /lat_lon="44.48 N 93.13 W"
                     /altitude="280 m"
                     /collection_date="2018-11"
                     /metagenome_source="insect metagenome"
                     /note="metagenomic"
     gene            <1..2953
                     /locus_tag="wEsol_00680"
     CDS             <1..2953
                     /locus_tag="wEsol_00680"
                     /codon_start=1
                     /transl_table=11
                     /product="hypothetical protein"
                     /pfam="Pfam-B_2381 NB-ARC TPR_12 Pfam-B_7784 TPR_10
                     Pfam-B_19818 Pfam-B_2142 TPR_12 TPR_10 TPR_11 TPR_2 TPR_8
                     TPR_1 Pfam-B_1978 TPR_7 TPR_14 Pfam-B_7784 TPR_10 TPR_11
                     TPR_1 TPR_8 TPR_2 TPR_7 Pfam-B_17261 Pfam-B_422 TPR_12
                     Pfam-B_2643 TPR_12 TPR_10 TPR_1 TPR_2 TPR_7 TPR_12
                     Pfam-B_19818 TPR_10 TPR_11 TPR_2 TPR_7 TPR_8 TPR_14
                     TPR_10 TPR_8"
                     /translation="ENKQSEVLSNRTFKKRDGTSTGDHGRKYEVKLLMLYLLHGLSSK
                     IDFWLASNIEGFGAFDDIFFEYKDQKNKSRIILVQAKHCNTHRSSKNITFEDLFIDGN
```

DRKQQKEDFGLNKYFESHPEIKQNPECKNSDITLVIHTNVNVNLKKGTEKQGSVETLE
KYLEESKLTNDNTLNIYEGQKGTCYKIKNHTELKKYFSCGSDDEIEDFLLRIRFVVDQ
PNHSKLDDIIKKEIGNIYKISGEEDIDTVFVRVYDKVGKWWAQQGSKVSYLTRKDDYF
REVIEHLYSLKPREDLFERWNIPPKIASGNFIEREYLNEEIVNHLKTKNEPVILTALY
GLGGVGKTQSVLDFIQKHGKEYKAICWFNAKDEDQLLSEYVNLGRKLNIIHDEDNISI
AQRAYYVKNWLEDSKREGWLLIYDNVSNYEVISKLRPTKGGKIIVTSRNTEWPQGSIG
VDVFTPPESELYVKKILGQVSESDRPNIGILAEKLGRLPLALAQACAYIKENKMTISR
YLEIYAERKLYLLSHKTLPKDSNHEPVFITWDITMDAIRNESKLASKLLVACAYLDND
IPKDLLRIFPEIVENNSEEKPFEEVLGILLRYSMLVSNEQSSSVSIHCLVQDVIRLKS
KESGKIKKDIKTVFQLLKESFPDDSDKLEDYAKKRQLLPHLEAFLSHIDNWLAKNPSE
KQTIEKDYLVYLLIWMNDGYHDLGNPRRQKELLEQALSIFKKYYGPYNFQVAIALANL
GIAYGDLGNHRRQKELLKQALPILEKHYGSDHFQVAIILTNLSNAYGGLGNPQKAKEL
LERALAIQEEHYGPDHSQVAVILTNLGIIYGNLGNYEEKKELLERALAIQEKHYEPDH
SQVASTLVGLGTSYRALGDSQKAKELLGRALAIQEKHYGSDHFQVAITLTNLSNAHGD
LGNLQKAKELLERALPIFEKHYGPDHLEVAKLLANLSITYGDLGNPQKAKELLEQASF
ILEKHHVHDYF"
/besthit="qcoverage 75.5849440488301 , hcoverage
83.5770528683915 , similarity 100 , identity 100 , evalue
0.0 , alnlength 743 , Bacteria , Proteobacteria ,
WP_174516902.1 tetratricopeptide repeat protein
[Wolbachia endosymbiont of Cardiocondyla obscurior]"

gene 2934..3116
/locus_tag="wEsol_00681"

CDS 2934..3116
/locus_tag="wEsol_00681"
/codon_start=1
/transl_table=11
/pfam="TPR_12"
/fullproduct="qcoverage 96.6666666666667 , hcoverage 100
, similarity 100 , identity 100 , evalue 8.01e-35 ,
alnlength 58 , Bacteria , Proteobacteria , WP_141457140.1
MULTISPECIES: tetratricopeptide repeat protein
[unclassified Wolbachia]"
/translation="MSMITSKVVELWEILGPAYGALGNHKKQKELFARASSIFKKHYG
PDHPEVAKLLAELDDI"
/product="tetratricopeptide
repeat protein"
/besthit="qcoverage 96.6666666666667 , hcoverage 100 ,
similarity 100 , identity 100 , evalue 8.01e-35 ,
alnlength 58 , Bacteria , Proteobacteria , WP_141457140.1
MULTISPECIES: tetratricopeptide repeat protein
[unclassified Wolbachia]"

gene complement(3155..4327)
/locus_tag="wEsol_00682"

CDS complement(3155..4327)
/locus_tag="wEsol_00682"
/codon_start=1
/transl_table=11
/fullproduct="qcoverage 100 , hcoverage 100 , similarity
100 , identity 100 , evalue 2.54e-263 , alnlength 390 ,
Bacteria , Proteobacteria , WP_015589060.1 MULTISPECIES:
2-oxoglutarate dehydrogenase complex
dihydrolipoyllysine-residue succinyltransferase
[unclassified Wolbachia]"
/cog="AceF COG0508 404 Pyruvate/2-oxoglutarate
dehydrogenase complex, dihydrolipoamide acyltransferase
(E2) component, and related enzymes"
/pfam="Biotin_lipoyl Biotin_lipoyl_2 E3_binding
2-oxoacid_dh"
/tigr="TIGR01347 sucB 404 dihydrolipoyllysine-residue

```
                     succinyltransferase, E2 component of oxoglutarate
                     dehydrogenase (succinyl-transferring) complex"
                     /product="2-oxoglutarate
                     dehydrogenase complex dihydrolipoyllysine-residue
                     succinyltransferase"
                     /translation="MSKIIEIRAPKTLGGESVTEGIVKIKKGIGEAVKVDDLIFEIET
                     DKTALELTAEASGQITEFLVKEDDVISPDQLLAKLSVGEVKEEARKEDKSESAAKKDA
                     PSARKIMEENAISAESVKGTGMGGRITKADVIGHMNKAEQPAIKQYELPKSVVSGEQR
                     EERVKMSKIRQVIAARLKASQNTAAILTTFNEIDMKNVMDLRAKYKDAFEKKYGIKLG
                     FMSFFIKAAVQALKEIAEINAEISGDEIIYKHYYDVGVAVGTDKGLVVPVIRGADQMS
                     FAEIELTLVALGKKAREGKLQVSEMEGATFTISNGGVYGSLLSTPIINPPQSGILGMH
                     SIQNRPVAMGNSIEIRPMMYIALSYDHRIVDGKGAVTFLVKIKNYIEDPNRLVLEV"
                     /besthit="qcoverage 100 , hcoverage 100 , similarity 100
                     , identity 100 , evalue 2.54e-263 , alnlength 390 ,
                     Bacteria , Proteobacteria , WP_015589060.1 MULTISPECIES:
                     2-oxoglutarate dehydrogenase complex
                     dihydrolipoyllysine-residue succinyltransferase
                     [unclassified Wolbachia]"
     gene            5364..6242
                     /locus_tag="wEsol_00683"
     CDS             5364..6242
                     /locus_tag="wEsol_00683"
                     /codon_start=1
                     /transl_table=11
                     /fullproduct="qcoverage 100 , hcoverage 100 , similarity
                     100 , identity 100 , evalue 2.74e-204 , alnlength 292 ,
                     Bacteria , Proteobacteria , WP_015589059.1 MULTISPECIES:
                     hydroxymethylbilane synthase [unclassified Wolbachia]"
                     /cog="HemC COG0181 307 Porphobilinogen deaminase"
                     /pfam="Porphobil_deam Porphobil_deamC"
                     /tigr="TIGR00212 hemC 293 porphobilinogen deaminase"
                     /product="
                     hydroxymethylbilane synthase"
                     /translation="MPVKIGTRGSKLAVAQALEAKQKLLDSFPNLSIEIVKIKTSGDK
                     YANANLAEIGGKGLFIKEIETELLENNIDMAVHSLKDMPAFFSGGLTIPCVLERLSSC
                     DAFISHKHNSLESLPQQATIATSSIRRKVQLLNVRPDLNIVPLRGNVTTRLQNQSFDG
                     IILAEAGLIRLEKHHLITEVLPPKVMLSAVGQGAVCIQCRRNDVKIINLLEKINNNMS
                     FIGVKSERSFMKTVNGSCFTPLAALAEYVSENMLHLRCMLASGKNIYFTERTSFIEDA
                     EKMGMDAGLELKSKCL"
                     /besthit="qcoverage 100 , hcoverage 100 , similarity 100
                     , identity 100 , evalue 2.74e-204 , alnlength 292 ,
                     Bacteria , Proteobacteria , WP_015589059.1 MULTISPECIES:
                     hydroxymethylbilane synthase [unclassified Wolbachia]"
     gene            6233..7120
                     /locus_tag="wEsol_00684"
     CDS             6233..7120
                     /locus_tag="wEsol_00684"
                     /codon_start=1
                     /transl_table=11
                     /fullproduct="qcoverage 100 , hcoverage 100 , similarity
                     100 , identity 100 , evalue 1.17e-211 , alnlength 295 ,
                     Bacteria , Proteobacteria , WP_141457145.1 MULTISPECIES:
                     UDP-N-acetylmuramate dehydrogenase [unclassified
                     Wolbachia]"
                     /cog="MurB COG0812 291 UDP-N-acetylmuramate
                     dehydrogenase"
                     /pfam="FAD_binding_4 MurB_C"
                     /tigr="TIGR00179 murB 290
                     UDP-N-acetylenolpyruvoylglucosamine reductase"
                     /product="
```

```
                     UDP-N-acetylmuramate dehydrogenase"
                     /translation="MLISLPKVRGIYRYDVLMSKATWLNVGGRADVLFKPCDIEDLTY
                     LIKNTELPVSVIGATSNIIVRDSGIRGITVKLGKEFAYIKSKGNNSIVAGGAVLLSNL
                     AHFAGNQQISGLEFLVGIPGTVGGGIEMNAGAYGSDIASVVQSIKAVNLEDGNLYEFS
                     SEEMGYFYRGHSLKGNWIFVEAEFKGINSEYELILQRLKEVIERKNKSQPIRGKTAGC
                     IFKNPKNYRAWELIDKSGCLGLNIGGARISKKHCNFLLNYDNATASDLENLGNKVKDA
                     VKDKFNVELEWEIRVLGSY"
                     /besthit="qcoverage 100 , hcoverage 100 , similarity 100
                     , identity 100 , evalue 1.17e-211 , alnlength 295 ,
                     Bacteria , Proteobacteria , WP_141457145.1 MULTISPECIES:
                     UDP-N-acetylmuramate dehydrogenase [unclassified
                     Wolbachia]"
     gene            complement(7523..8455)
                     /locus_tag="wEsol_00685"
     CDS             complement(7523..8455)
                     /locus_tag="wEsol_00685"
                     /codon_start=1
                     /transl_table=11
                     /fullproduct="qcoverage 100 , hcoverage 100 , similarity
                     100 , identity 100 , evalue 3.60e-193 , alnlength 310 ,
                     Bacteria , Proteobacteria , WP_015589057.1 MULTISPECIES:
                     Rpn family recombination-promoting nuclease/putative
                     transposase [unclassified Wolbachia]"
                     /pfam="PDDEXK_2 Pfam-B_9730 Pfam-B_12519 Pfam-B_10245"
                     /tigr="TIGR01784 T_den_put_tspse 273 conserved
                     hypothetical protein"
                     /product="Rpn family
                     recombination-promoting nuclease/putative transposase"
                     /translation="MALSKFLDPKLDLTFKKIFGTEKNKNILIHFLNDILGFTEINTI
                     QEVEFLSTIMDPEIASDKQSIVDVLCRDSSGARYVIEMQLTRDKGFEKRAQLYAAKAY
                     SRQADKSGKYIDLKKVFFIAISNCNLFPDKLDYISSHTIRDEKTNEHDLKDFQFVFIE
                     LPKFPKNKEEQLESIVDRWLFFFKYADETTEEDLRKIAEKAPIIKLAYDELDRFSWNE
                     KDLVAYEERIMDLRKEEAILEYRLDLATEKGIQIGEERGIKIGKEEGKIEGKIEGKIE
                     VAKAMLANNVDVNTIVKFTGLSISEIEELSVNGY"
                     /besthit="qcoverage 100 , hcoverage 100 , similarity 100
                     , identity 100 , evalue 3.60e-193 , alnlength 310 ,
                     Bacteria , Proteobacteria , WP_015589057.1 MULTISPECIES:
                     Rpn family recombination-promoting nuclease/putative
                     transposase [unclassified Wolbachia]"
ORIGIN
        1 agaaaacaaa cagtctgaag tacttagtaa tcgtacattt aagaagagag atggcacatc
       61 aacaggtgat catggtcgca agtatgaagt aaaattatta atgctgtatc tacttcatgg
      121 gttaagtagt aaaattgact tttggttagc atcaaacata gaaggttttg gagcctttga
      181 tgatattttt tttgagtata aagatcagaa gaacaaatcg agaattatct tggttcaagc
      241 taagcactgc aatacacatc gctcttcgaa gaatattact tttgaagatt tgtttattga
      301 tggaaatgat agaaagcaac agaaagaaga tttcgggtta aacaaatatt ttgaatctca
      361 tcctgagatt aagcaaaacc ctgagtgtaa gaacagtgat attacgcttg tcattcatac
      421 taatgtcaat gtgaacctaa aaaaaggtac agagaagcaa ggaagcgtag aaacattaga
      481 gaagtatctg gaagagtcaa agttaacaaa tgataacact ttaaatattt atgaaggtca
      541 aaaaggaacg tgctacaaaa taaaaaatca tactgaactg aaaaaatact tcagttgtgg
      601 tagtgacgat gaaatagaag atttttttact tagaataagg tttgtagttg atcaacctaa
      661 tcattcaaaa ttagatgata ttattaagaa agaaataggg aatatataca aaatatcagg
      721 ggaagaagat attgatacag tttttgtccg tgtatatgat aaggtaggga aatggtgggc
      781 acaacagggt agcaaagttt cttatctaac aagaaaagac gattatttta gggaagttat
      841 agaacatctt tatagtctca aacctagaga agatctattt gaacggtgga acattccacc
      901 taaaatagct agtggaaatt tcattgaacg agaatatctc aatgaagaaa tagtaaatca
      961 tctaaagaca aaaaatgagc cagttatatt aactgctttg tacgggttag gtggagtagg
     1021 taaaacacag tcagtgctag atttattca aaaacatggg aaagaataca aggcaatatg
     1081 ttggtttaat gcaaaagatg aagatcaact actcagtgaa tatgttaatt taggaagaaa
     1141 attaaatatt attcacgatg aagacaacat ttctattgca cagcgagctt attatgtgaa
```

1201 aaactggcta gaagattcta aacgtgaagg ctggttgttg atttatgata atgtatcaaa
1261 ttatgaagtc atttctaagc tcagacctac aaaaggcggc aaaataatag ttacgtctcg
1321 taatactgaa tggccacaag ggagtatagg tgtagacgtt tttactcccc cagaatcaga
1381 attatatgtt aagaaaattc taggccaggt ttcagaaagc gataggccaa atataggtat
1441 attggcagaa aagttaggta gattaccttt ggcattagct caggcttgcg cttacatcaa
1501 agaaaacaaa atgactatct cacgttactt agaaatttat gcagaaagga agctttattt
1561 attatctcat aaaacactac ctaaggattc taaccatgag cctgtgttta ttacttggga
1621 tattacaatg gacgcaattc gtaatgagtc taaactagca agcaagttgt tagtagcttg
1681 tgcatattta gacaatgata ttcctaaaga tttgttaaga atatttcctg agattgtaga
1741 gaacaattct gaagaaaaac cttttgaaga agtattaggt atattattga gatattcgat
1801 gctagtgtcc aatgagcaga gtagtagtgt ttctattcac tgtttagtac aagatgtaat
1861 taggctaaag tcaaaggaaa gtggaaaaat taaaaaagat ataaaaaccg tttttcaatt
1921 attaaaagaa agcttccctg acgacagtga taaattagaa gattatgcta agaagcgaca
1981 attattgccg cacttagaag cgtttttatc acatatagat aattggctag caaaaaatcc
2041 ttcagaaaaa caaacaatag aaaaggatta tcttgtgtat ttgttaatat ggatgaacga
2101 tggataccat gacttgggta atcccagaag acaaaaggag ctgcttgaac aggctttatc
2161 tatctttaag aaatattatg ggccttataa ttttcaagtt gctatagcgc tggcaaatct
2221 aggtatcgct tatggggatc tgggtaatca taggagacaa aaggagttac ttaaacaggc
2281 tttacctatt cttgagaaac attatggctc tgaccatttc caggttgcta ttatactgac
2341 aaacctaagc aacgcttatg gggtctgggg taaccctcag aaagcaaaag agttacttga
2401 acgggctcta gcaattcaag aggaacatta tgggcctgat cattcccaag ttgctgttat
2461 attgacaaac ctcggtatca tttatgggaa tttaggtaac tatgaagaaa aaaaggagtt
2521 gcttgaacgg gctttagcaa ttcaagagaa acattatgag cctgatcatt cccaagttgc
2581 tagcacatta gtaggtctag gtacctctta tagagcttta ggtgattctc agaaagcaaa
2641 agagttactt ggacgggctt tagcaattca agagaaacat tatggctccg atcatttcca
2701 agttgctatc acattgacaa acctaagtaa cgctcatgga gatttaggca atcttcagaa
2761 agcaaaagag ttgcttgaac gggctttacc tatttttgag aaacattacg gtcctgatca
2821 tcttgaagtt gctaaactat tggcaaacct aagtattacc tatggggatt taggtaaccc
2881 tcagaaagca aaggagttgc ttgagcaggc ctcatttatt cttgagaagc atcatgtcca
2941 tgattacttc taaagttgtt gaactatggg aaattctagg ccccgcttat ggtgctctag
3001 gtaaccataa gaaacaaaag gagttgtttg cacgggcttc atccatcttt aagaaacatt
3061 atggccctga tcatccggaa gttgctaaac tgctggcaga actagatgac atttaggttt
3121 ttagcaatac tggattaaca acaattcatt tgagctacac ttccaaaact aatctatttg
3181 gatcttctat ataatttttt attttaacga ggaaagtaac tgctcctttg ccgtcaacta
3241 ttctgtgatc gtaagagagg gcaatgtaca tcataggtct aatctcaatt gagttgccca
3301 tggcaactgg cctgttttgt attgaatgca ttccaagtat tccagattgc ggagggttta
3361 ttatcggagt agaaaggagc gaaccatata ctccgccatt tgaaatggta aatgttgcac
3421 cttccatttc tgatacttgt aatttacctt ctcgtgcttt tttgccaaga gcaactaaag
3481 tcaactcaat ttctgcaaat gacatctgat cggcaccccg aataactggt acaacaagac
3541 ctttatcggt gccaacagca acacctacgt catagtaatg tttatatatg atttcatcgc
3601 ctgagatttc agcgttaatt tcagcaattt ctttcaatgc ttgcaccgct gcctttataa
3661 aaaacgacat aaaaccaagt tttattccat attttttttc aaaggcgtcc ttatattttg
3721 ctcttagatc catgacattc ttcatgtcaa tttcattgaa cgtggtcagt attgcagcag
3781 tattttgcga tgctttcaaa cgagcggcaa ttacttgtct tattttgctc atttttactc
3841 gttcctctct ttgctctcca cttactacac tttttggcaa ttcatattgt tttattgcag
3901 gttgttcagc tttgttcata tggcctatca catctgcttt tgttattctg cctcccatgc
3961 cagttccttt gacgctttct gcactgattg cattttcttc cataatttta cgagctgatg
4021 gagcatcttt tttagcagca ctttcgcttt tatcttcttt tctcgcttcc tcttttactt
4081 ctcccacgga aagcttcgct aacaattgat cagggcttat tacatcatct tcctttacaa
4141 gaaattcagt tatttgcccc gaagcttctg cagttaattc gagtgctgtt ttgtcagttt
4201 caatttcaaa gatcaagtca tctactttta ctgcttctcc aataccettc tttatttta
4261 ctataccttc tgtaactgat tcaccaccaa gagtttttgg tgctctgatt tctataattt
4321 tgctcataaa acaacttccg tataaaactt tctatagatt tatacatgaa aagagacatg
4381 taataaacta ataaattcat tgaggctctt atcatttaga agtggaccac aactcccaaa
4441 tgtcattcca gcgcagccat aaaaattcct tgacaaactc cgccagcccc cttatcatga
4501 aactgaagct atttatttat cttctctgta cagattaaat gacaaaaaaa ctcacgtatc
4561 tggcgtctca tgtttaattt tttgcactat gtgcactcta tgtctttatc aaatttctgg
4621 gtttttacct acacaagctg aaatatgctt ataaagcatt taaaacataa aaaaacgcca
4681 acttaaaaaa tggatagtga ataactagct accctagggt ttcttttgcc ttttttctg
4741 tttagtaaat ttcttaatgt ttataattta gattagttgc ggtttaaaag cagctgaatc

4801 gcggttatta agtgtttaga ataaaaaaac gccatacttg aaagtataat gtaagtaatt
4861 agccaaccac ggggcttctt ttgccttttt tttcgtttgg taaatttctt aatatttatg
4921 gctaaacgac aatcgtcatc ccgctacttg ttagcgggat ctagagatac cgcgaatgaa
4981 tcgcggtatg acggtatgcg gcggcgtgac gataagctcg tcatcccgct gcttgttagc
5041 gggatctctt gttagcggct gagataccgc gattcattcg cggtatgacg gttcgcggcg
5101 gtatgacgat aaggtttata ccttgtcatg caagtactag aatccagtaa gcttatttat
5161 gcatgtaaaa ggctattatt ttgtaaagtc aaagttgctt ttggttttaa gttaaagtac
5221 aataaatgca aaggctcata taaactggat ccaagtactt gaatgacatc agagagaggg
5281 taaccgaggt tcatgcgata cacgctacca aagtgtttaa ataattaatt aggttaaggt
5341 ttggtattca aacagtcgta cacatgccag tcaaaatagg taccagagga agcaagcttg
5401 cagttgccca ggctttggaa gcaaaacaaa agttgctgga ttcttttcct aacttatcta
5461 ttgaaattgt taaaataaaa acttctggag ataagtatgc aaacgcaaat cttgctgaaa
5521 taggaggcaa aggattattc attaaagaaa ttgagactga attgctcgaa aataatatag
5581 acatggcagt tcattcgcta aaagatatgc ctgcgttttt ctcagggggt ttaacaattc
5641 cttgtgtttt agaaaggcta agttcatgcg atgcgtttat ttctcataaa cacaatagcc
5701 ttgagtcttt gccacagcag gctactattg ccacttcttc aataagaaga aaagttcagt
5761 tattaaatgt tagaccagat ttaaatatag taccgctacg tggaaatgta acaaccagat
5821 tgcaaaatca aagttttgat ggaataattc tagctgaagc aggactcata agattagaaa
5881 aacatcactt gattacagag gtattgccac caaaagttat gttaagtgca gtggggcagg
5941 gggcggtttg tattcaatgt agaaggaatg atgtaaaaat tatcaatctt ttagagaaaa
6001 ttaataataa tatgtctttt ataggagtga aatcagagcg cagttttatg aagacagtga
6061 atggttcatg ttttacacca cttgcagctt tggctgaata tgtgagcgaa aatatgctac
6121 accttcgttg tatgttagca agtggaaaaa atatatactt cactgagcgc acttcattta
6181 tagaagatgc ggagaaaatg ggtatggatg caggattaga gttgaaatca aaatgcttat
6241 aagcttacct aaagtacgtg gaatctatcg ttatgatgtc ttaatgtcta aagcgacgtg
6301 gttaaatgtt ggtggacgag ctgatgtatt atttaaacca tgtgatattg aagatttaac
6361 atacttgatt aaaaatactg aactgccagt tagcgttatt ggtgcaacat ccaacataat
6421 agtgcgagat agtggcattc gaggaataac agtaaaatta ggtaaggagt ttgcatatat
6481 taaaagtaag ggtaacaact ctattgtagc tggggagct gtgctgctca gcaaccttgc
6541 tcactttgcg ggaaatcagc agattagtgg gttggagttt ctagttggaa ttccaggaac
6601 agttggtggc ggaatagaaa tgaatgcagg tgcatatggt agtgatatcg caagtgttgt
6661 acaatccata aaagcagtga atctagagga tggaaaccta tatgagttct ccagcgaaga
6721 aatggggtat ttttaccgtg gacatagcct aaaaggaaat tggatttttg ttgaagctga
6781 gtttaaagga ataaactcag agtatgagct tatactgcaa aggttaaagg aagttattga
6841 gaggaaaaat aaaagccagc caataagagg aaaaactgct gggtgcatat tcaaaaatcc
6901 aaaaaactac cgagcatggg agttaattga taaatctggc tgcctagggt taaatattgg
6961 tggagctaga atctctaaga aacattgtaa cttcttgctc aattacgata atgcaactgc
7021 atctgacttg gaaaaccttg gcaacaaagt aaaagatgca gtaaaggata aatttaatgt
7081 tgaacttgag tgggaaataa gggttttggg tagctattag atagttttta tagcttatgt
7141 atattcgttc agtagaacgg caaataaggt aaacaagtaa tctaaaaaaa taatcataga
7201 gaaaccgtga ggtaatgtgg ttaatcttgt agaactttgc aagaatctag agagaaaaat
7261 agcaaagtta taccaattct cattattagt gaatcaaata agcgatattg tagagttatt
7321 taattgtgga gtgagaaaga aaggcaatag tatggctttt tttccattgt tatctatccg
7381 tttattgtgc agtgggaatt ggtattagct gaacggacac tattgttata ctatatctta
7441 tatacaattg tgcaatatgg acaatggcaa caaatttact agcgaaattc agtgatttat
7501 gattgataat taaagttctt aattagtaac cgttcacact taattcttca atctcactaa
7561 tagaaaggcc agtaaatttg acaatagtgt tgacatcaac attgttagcc agcattgctt
7621 ttgcgacttc gatttttcct tcgatttttc cttcaatttt cccttcctct ttaccgattt
7681 tgatgcctct ttcttcgcca atttggatgc ctttttcagt agcaaggtca agtctgtatt
7741 caagaatggc ttcttctttc cgtagatcca ttattctttc ttcataagct actaaatctt
7801 tttcattcca gctaaacctg tctaattcat cgtaggctaa ctttattatt ggtgcttttt
7861 ctgctatttt ccttaagtct tcttccgtag tttcatctgc atatttgaag aaaaataacc
7921 aacgatccac tatgctttct agctgttctt ccttattctt aggaaattta ggtagttcaa
7981 taaatacaaa ttgaaaatct tttaagtcat gctcattggt tttctcatct cttatagtgt
8041 ggctggatat ataatcaagc ttatcaggaa ataaattaca attagagata gcaataaaga
8101 aaaccttctt taaatcaatg tacttaccag atttatcagc ttgccttgag taagccttag
8161 cagcatatag ttgagctcgt tttcgaagc ctttatcgcg agtaagctgc atttcaatca
8221 catacctggc cccactagaa tccctgcaaa gaacatcaac tatactttgt ttatcagatg
8281 caatttcagg atccattata gtactgagga attccacttc ttgtatagta tttatctcag
8341 taaaccctaa aatatcattc aaaaaatgta tgagaatatt cttatttttt tcagtgccaa

```
     8401 atatttttt gaaggtcaaa tccagcttag ggtcgagaaa cttagataaa gccatagcca
     8461 aagtgcttaa aaatattaat aattatacac tattattaac aattattcaa ccatattttt
     8521 gaagatagct atgccgccgc gaaccgtcat accgccgcgg tatctctaga tcccgctaac
     8581 aagcagcggg atgacgaatt acttaaccgt cataccaccg tgaaccgtca taccgccgcg
     8641 gtatctcggc atagattccg ctaa
//
LOCUS       JAQZAU010000065         7719 bp    dna     linear   ENV 17-MAR-2023
DEFINITION  MAG: Wolbachia pipientis isolate wEsol contig_38164, whole genome
            shotgun sequence.
ACCESSION   JAQZAU010000065 JAQZAU010000000
VERSION     JAQZAU010000065.1
DBLINK      BioProject: PRJNA929258
            BioSample: SAMN33142973
KEYWORDS    WGS; ENV; Metagenome Assembled Genome; MAG.
SOURCE      Wolbachia pipientis (insect metagenome)
  ORGANISM  Wolbachia pipientis
            Bacteria; Pseudomonadota; Alphaproteobacteria; Rickettsiales;
            Anaplasmataceae; Wolbachieae; Wolbachia.
REFERENCE   1  (bases 1 to 7719)
  AUTHORS   Fiutek,N., Couger,B.M., Roy,S., de la Torre,J.R. and Connor,E.F.
  TITLE     Genomic assessment of the contribution of the Wolbachia
            endosymbiont of Eurosta solidaginis to gall-induction
  JOURNAL   International Journal Molecular Sciences
REFERENCE   2  (bases 1 to 7719)
  AUTHORS   Fiutek,N., Couger,B.M., Roy,S., de la Torre,J.R. and Connor,E.F.
  TITLE     Direct Submission
  JOURNAL   Submitted (10-FEB-2023) Biology, San Francisco State University,
            1600 Holloway Ave, San Francisco, CA 94132, USA
COMMENT     ##Genome-Assembly-Data-START##
            Assembly Date          :: JUN-2019
            Assembly Method        :: FLYE v. 06-2019
            Genome Representation  :: Full
            Expected Final Version :: Yes
            Genome Coverage        :: 100.0x
            Sequencing Technology  :: Illumina HiSeq; PacBio Sequel
            ##Genome-Assembly-Data-END##
FEATURES             Location/Qualifiers
     source          1..7719
                     /organism="Wolbachia pipientis"
                     /mol_type="genomic DNA"
                     /submitter_seqid="contig_38164"
                     /isolate="wEsol"
                     /isolation_source="galls on Solidago altissima"
                     /host="Eurosta solidaginis MN-2018-EFC"
                     /db_xref="taxon:955"
                     /environmental_sample
                     /country="USA: Cowling Arboretum, Carleton College,
                     Northfield, MN"
                     /lat_lon="44.48 N 93.13 W"
                     /altitude="280 m"
                     /collection_date="2018-11"
                     /metagenome_source="insect metagenome"
                     /note="metagenomic"
     gene            complement(<1..123)
                     /locus_tag="wEsol_00686"
     CDS             complement(<1..123)
                     /locus_tag="wEsol_00686"
                     /codon_start=1
                     /transl_table=11
                     /product="hypothetical protein"
```

```
                     /translation="MKDKPVRQKKNLDKGLKRSVGKPACCVSKGGSSSNTASLPD"
                     /besthit="qcoverage 100 , hcoverage 77.3584905660377 ,
                     similarity 100 , identity 100 , evalue 2.37e-19 ,
                     alnlength 41 , Bacteria , Proteobacteria , WP_211907584.1
                     hypothetical protein [Wolbachia endosymbiont of
                     Spodoptera picta]"
     gene            complement(120..1667)
                     /locus_tag="wEsol_00687"
     CDS             complement(120..1667)
                     /locus_tag="wEsol_00687"
                     /codon_start=1
                     /transl_table=11
                     /pfam="RVT_N RVT_1 GIIM"
                     /fullproduct="qcoverage 100 , hcoverage 100 , similarity
                     100 , identity 100 , evalue 0.0 , alnlength 515 ,
                     Bacteria , Proteobacteria , WP_155968541.1 MULTISPECIES:
                     group II intron reverse transcriptase/maturase
                     [Wolbachia]"
                     /translation="MITSKPVSASTESFEAWKQLPWKKCQKVIVRLQRRIVKAVQEGR
                     WGKVKALQHLLTRSFSGKALAVKRVTENQGKTTAGVDRQLWSTCNAKFQGIKLLKQRG
                     YKPSPLKRIYISKSNGKRRPLGIPTIKDRAMQALYLLALEPVAETISDRHSYGFRPKR
                     SCADATVACHLLLASRNQLQWILEGDIKGCFDNINHEWLMKHIPMEKKILHRWLKAGF
                     LESKTLYPTTAGTPQGSIISPILANFTLDGLEQLLESRFGKLGSKRRGKIRSGVNVIR
                     YADDFIISGFTHEVLENEVKPLVSSFLHERGLILSEEKTKITSITTGFDFLGCNVRRY
                     NKKLIIKPSKESIKRLLNKARTLIKANIANTQAIVIKSLNSLLRGWGNYYHHVCAKKA
                     FRKIDNEIWHSLWKWAKKRHPRKGLRWIKNRYFKVMGQRQWVFAAPICKNKPKEIRYL
                     RLLKLIDIPIRRHVKIRADANPLDLKWKKYFDERVKRTRMLASSFSREGSLLLVSPLK
                     LMYSEDL"
                     /product="group II intron
                     reverse transcriptase/maturase"
                     /besthit="qcoverage 100 , hcoverage 100 , similarity 100
                     , identity 100 , evalue 0.0 , alnlength 515 , Bacteria ,
                     Proteobacteria , WP_155968541.1 MULTISPECIES: group II
                     intron reverse transcriptase/maturase [Wolbachia]"
     gene            complement(1676..1942)
                     /locus_tag="wEsol_00688"
     CDS             complement(1676..1942)
                     /locus_tag="wEsol_00688"
                     /codon_start=1
                     /transl_table=11
                     /fullproduct="qcoverage 100 , hcoverage 100 , similarity
                     100 , identity 100 , evalue 2.05e-61 , alnlength 88 ,
                     Bacteria , Proteobacteria , WP_026097253.1 hypothetical
                     protein [Wolbachia endosymbiont of Ceratitis capitata]"
                     /translation="MYANNNSTGIEKPPNPLLYDWRNRVSLYFRLHGKQTARNADGSA
                     GKGEWRKRMPFCNEMDRICNINSRENEQTSIWSLVTRNFVNHLR"
                     /product="hypothetical protein"
                     /besthit="qcoverage 100 , hcoverage 100 , similarity 100
                     , identity 100 , evalue 2.05e-61 , alnlength 88 ,
                     Bacteria , Proteobacteria , WP_026097253.1 hypothetical
                     protein [Wolbachia endosymbiont of Ceratitis capitata]"
     gene            complement(2104..2304)
                     /locus_tag="wEsol_00689"
     CDS             complement(2104..2304)
                     /locus_tag="wEsol_00689"
                     /codon_start=1
                     /transl_table=11
                     /fullproduct="qcoverage 100 , hcoverage 100 , similarity
                     100 , identity 100 , evalue 9.65e-39 , alnlength 66 ,
                     Bacteria , Proteobacteria , CAQ55081.1 hypothetical
```

                     protein WP0973 [Wolbachia endosymbiont of Culex
                     quinquefasciatus Pel]"
                     /translation="MVRPESLRSYCLKGEEFIQKETERILKLIKPDVLPSESTHQCES
                     GNRLVPSDADNEPPLEKEDAQS"
                     /product="hypothetical protein"
                     /besthit="qcoverage 100 , hcoverage 100 , similarity 100
                     , identity 100 , evalue 9.65e-39 , alnlength 66 ,
                     Bacteria , Proteobacteria , CAQ55081.1 hypothetical
                     protein WP0973 [Wolbachia endosymbiont of Culex
                     quinquefasciatus Pel]"
     gene   2461..2568
                     /locus_tag="wEsol_00690"
     CDS   2461..2568
                     /locus_tag="wEsol_00690"
                     /codon_start=1
                     /transl_table=11
                     /product="hypothetical protein"
                     /translation="MRMPLFFSVPKIRLNDISFLGSRNLEKVIRNQPGE"
     gene   complement(2641..3888)
                     /locus_tag="wEsol_00691"
     CDS   complement(2641..3888)
                     /locus_tag="wEsol_00691"
                     /codon_start=1
                     /transl_table=11
                     /pfam="Pox_D5 RVT_1 GIIM"
                     /fullproduct="qcoverage 100 , hcoverage 100 , similarity
                     100 , identity 100 , evalue 7.62e-314 , alnlength 415 ,
                     Bacteria , Proteobacteria , WP_155968744.1 group II
                     intron reverse transcriptase/maturase [Wolbachia
                     pipientis]"
                     /translation="MNKTKSFDIPKQLIWKAYKQVSKNRGAAGVDEVSITKFEENLKD
                     NLYKLWNRMSSGSYFPESVKAVAIPKDTGGQRTLCVPSVFDRIGQTAATMYLEPLVEP
                     EFHEDSYGYRPNKSALDAVSTACKRCWRSDWTIDLDISGFFDNLDHVLALQTIKKHTD
                     CKWVILYVERWMKAPIQLADGSKVVRNKGVPQGGSVSPIISNIFMHHAFDMWMRQNYP
                     TIPFERYVDDAMVHCKTQKQAKFIKDKIEERLAEFKLKLHPEKTQIVYCKDDNRRDEF
                     PIQSFDFLGYTFRPRLAKNNKIGNYFVSFLPAISNKAKKKINQTIRSWKIRRQTYTTL
                     EKISKKINPIVRGWYQYYGRFYKSEMYPSLRNVEWHLVGWVRAKYKKLRNHRRLAKQF
                     LAKVRKRSPNIFYHWTLGLGSKG"
                     /product="group II intron reverse
                     transcriptase/maturase"
                     /besthit="qcoverage 100 , hcoverage 100 , similarity 100
                     , identity 100 , evalue 7.62e-314 , alnlength 415 ,
                     Bacteria , Proteobacteria , WP_155968744.1 group II
                     intron reverse transcriptase/maturase [Wolbachia
                     pipientis]"
     gene   4553..6370
                     /locus_tag="wEsol_00692"
     CDS   4553..6370
                     /locus_tag="wEsol_00692"
                     /codon_start=1
                     /transl_table=11
                     /fullproduct="qcoverage 100 , hcoverage 100 , similarity
                     100 , identity 100 , evalue 0.0 , alnlength 605 ,
                     Bacteria , Proteobacteria , WP_213863493.1 DNA mismatch
                     repair endonuclease MutL [Wolbachia endosymbiont of
                     Ceratitis capitata]"
                     /cog="MutL COG0323 638 DNA mismatch repair enzyme
                     (predicted ATPase)"
                     /pfam="HATPase_c HATPase_c_3 DNA_mis_repair MutL_C"
                     /product="DNA mismatch repair endonuclease

```
                     MutL"
                     /translation="MAIILLDTKTINRIAAGEVIERPASVVKELVENAIDAGSSEIEI
                     KIESGGRNLITVTDDGNGIEKDDLELAFMRHATSKLSDSELIEIKHLGFRGEALPSIA
                     AVSRMKLSSKASGAKESWSIRYEGGEKIREITPCSLLQGTYIEVRDLFFATPNRLKFL
                     KTERAETQSIVDIVNNLAMINYSIGFTLTSGNKKLLKYVKQTSLFNRLCEIEEEFQSN
                     SLEVKEEEEGIKLKGHICKPNVNRGNSTQIYTFVNGRPIKDNLLVGAIRYAYHDFIPN
                     NRYPFAVLHLEVPYDQVDVNVHPNKSEVRFQNKRLIYEIVRRGIIKTLSTRFAAGDQG
                     IEEELIFNDSKSQEQVDSQEKKDQKEFYEKRPSLLENRLMKEFNAPDERRQSLPETFK
                     YGESPPQKGTMVLERKQIDLIEDYPLGYSRCQVYNTYIIAEAKGKLIIVDQHAAHERL
                     IYECLKQKSSIKRQKLLLPEIVEIKNQAGMEMVEMYKDKLFEMGFGIEIESEDKVRVK
                     EIPAILGTINVKEMVMNIVDRLMEIGDTLPIEEKVNKILATIACHGSIRAGREMKLEE
                     MNELMRQMEETPYAGQCNHGRPTYIEMKLSDIEKLFERR"
                     /besthit="qcoverage 100 , hcoverage 100 , similarity 100
                     , identity 100 , evalue 0.0 , alnlength 605 , Bacteria ,
                     Proteobacteria , WP_213863493.1 DNA mismatch repair
                     endonuclease MutL [Wolbachia endosymbiont of Ceratitis
                     capitata]"
     gene            6438..7376
                     /locus_tag="wEsol_00693"
     CDS             6438..7376
                     /locus_tag="wEsol_00693"
                     /codon_start=1
                     /transl_table=11
                     /pfam="HTH_31 HTH_19 HTH_3 HTH_26 HTH_31 HTH_19 HTH_3
                     HTH_26"
                     /fullproduct="qcoverage 100 , hcoverage 100 , similarity
                     100 , identity 100 , evalue 3.30e-206 , alnlength 312 ,
                     Bacteria , Proteobacteria , WP_213863494.1
                     helix-turn-helix domain-containing protein [Wolbachia
                     endosymbiont of Ceratitis capitata]"
                     /translation="MKKENKCSNFLDYKVIGQEVRNRRLAKGYTQKDLAKKIDTTYQV
                     ILQYEKGTRRISIKKLYELAEALSTTARDLACVQEVSNEGRYEGEEVLNLVRRHREIK
                     DQELRETFYLLTKFIRISEEESGKAVKIEVAKGLVKEGVSAHVISQTTSLSIDEYDND
                     EKKISIPYKVGQRIKEWRLRRGYTQEDLASKVGIINQRIYEYEQGRAGVSLEMLDEIA
                     KVLLISITDLLPETTENENSEVELSRLIEEYKKIKSQELRNVLIKSLFESIQVCKEKV
                     KRVEKMKITKNLVKEGISINIILKTVDISLDEIQQI"
                     /product="helix-turn-helix
                     domain-containing protein"
                     /besthit="qcoverage 100 , hcoverage 100 , similarity 100
                     , identity 100 , evalue 3.30e-206 , alnlength 312 ,
                     Bacteria , Proteobacteria , WP_213863494.1
                     helix-turn-helix domain-containing protein [Wolbachia
                     endosymbiont of Ceratitis capitata]"
     gene            7547..>7719
                     /locus_tag="wEsol_00694"
     CDS             7547..>7719
                     /locus_tag="wEsol_00694"
                     /codon_start=1
                     /transl_table=11
                     /product="hypothetical protein"
                     /translation="MNNKENSKDRREEIEFRALESNGKALLDREVIETFLSAVHDREE
                     ARIIARKLIDRGC"
                     /besthit="qcoverage 94.7368421052632 , hcoverage
                     45.7627118644068 , similarity 100 , identity 100 , evalue
                     1.87e-25 , alnlength 54 , Bacteria , Proteobacteria ,
                     ERN55805.1 DNA repair protein RadC, partial [Wolbachia
                     pipientis wMelPop]"
ORIGIN
        1 gtcaggtaag gaagcagtat tactactact ccctccctta gaaacgcagc atgcaggttt
       61 ccccacacta cgcttgagcc ccttatctaa gttcttcttt tgtcgaaccg gcttgtcttt
```

121 cataaatcct cagaatacat caactttagt ggtgacacta atagtagaga accttctctt
181 gaaaaggagc ttgccaacat tcttgttcgt tttactctct catcaaaata ctttttccat
241 tttaagtcaa ggggatttgc atccgctctg attttaacat gtcgtctgat aggtatatca
301 atcagcttaa gcagtcttaa ataccgtatc tcttttggtt tgttcttgca tatgggtgca
361 gcaaaaaccc attggcgttg acccattact ttgaagtaac gattctttat ccaacgtaat
421 cctttacgag gatgtctttt ctttgcccat ttccataaac tatgccaaat ttcattgtcg
481 atctttctaa acgctttttt agcacacaca tggtgatagt aatttcccca tcctctcagt
541 agagaattga gtgacttaat tactatagcc tgagtgttcg ctatatttgc ttttatcaat
601 gtacgtgctt tattcagaag tcttttaata ctttctttcg aaggtttaat aattaacttc
661 ttattatatc tacgtacatt acaaccaaga aagtcaaacc ctgttgtgat agatgtaatc
721 tttgtcttct cttctgatag aattaaacct cgctcatgaa gaaaagatga tactaaaggc
781 ttaacttcat tttccagtac ttcatgtgtg aaacctgaaa taataaagtc gtctgcgtac
841 ctgattacat tcactccact tctgattttt cctcttcttt tgctacctaa tttaccaaag
901 cgactttcta ataactgttc aagaccatct aaggtaaagt tagctagtat tggagaaatt
961 atacttcctt gcggagtgcc agcagttgtg ggatacagag ttttcgattc taggaaacca
1021 gcttttaacc atctatgaag aatttcttc tccataggaa tatgcttcat gagccattca
1081 tggttaatgt tatcaaaaca acctttgata tcaccttcga gtatccattg tagttgatta
1141 cgacttgcca gtaataagtg gcaagccaca gtagcgtctg cgcaggatct tttaggtcta
1201 aagccgtaag agtgacgatc actgattgtt tcagctaccg gttccaacgc gagcagatac
1261 aatgcttgca tcgctctatc ttttatcgtg ggtataccaa gaggccttct tttgccatta
1321 gatttactga tataaattcg tttgagcgga gaaggtttat atcctctttg ttttagcagc
1381 tttattcctt gaaatttagc attgcaagtt gaccatagtt gacgatctac acctgctgtg
1441 gtttttcctt ggttttcagt tactctctta acagccaaag cttttccgct aaaagagcgt
1501 gtgagaagat gttgtaaagc ttttacctta ccccatcttc cttcttggac agccttaaca
1561 atacgtcttt gtagcctcac aataactttc tggcatttct tccatggcaa ttgcttccat
1621 gcttcaaagc tttcggtaga tgcacttaca ggtttacttg taatcatctg ctttcctacc
1681 ttaagtggtt aacaaagttt cttgtcacga gggaccaaat ggaagtctgc tcgttttcac
1741 gagaattaat gttacaaatt ctatccattt cgttacaaaa tggcattcgc tttctccatt
1801 ctcctttacc tgcacttcca tcagcattcc ttgcggtttg cttgccatgt aggcgaaaat
1861 acaggcttac cctgtttctc cagtcataca acagtgggtt aggtggcttc tctataccgg
1921 tggaattgtt atttgcgtac atccagagat gaaaaatgta tcttatccac attacctttt
1981 ggtcagagct tatcagcatc tttagctctt tcatcgtatc ggtacttata aagcttcact
2041 tacgttcacc ataccactaa gcctagccct ccaaccacat gatgcttgca gtttatgcct
2101 ctctcacgat tgagcatctt ctttttcaag aggaggttca ttgtccgcat cgcttggcac
2161 caaccggttg cccgattcgc actgatgcgt agactcagat ggcaaaacat ctggtttaat
2221 aagttttaga atcctttcag tttctttctg aatgaattcc tctcctttta aacaatatga
2281 ccgaagactt tcaggtcgca ccatctcgca aatgtattga gcacctgtag aatctctaca
2341 gagaacatca acaatgcttt gtttatcaga agcaatttca gggtcttgaa tagtacttaa
2401 aaactctata tcttttattc atttttacca gtaaagccaa gaatatcatt gaggaagtga
2461 atgaggatgc ctttattttt ttcagtacca aagatacgct tgaacgatat atcatttttg
2521 ggatcaagaa acttagaaaa agtcataaga aatcaacctg gagagtagac cggcggagct
2581 tccccaccag tctctcgcag aactgtacgt aaacctctca atttatacag ctcccgttat
2641 tcagcctttt gatcccaatc ctagtgtcca gtgatagaaa atatttggag acctctttct
2701 cacttttgct agaaattgct ttgctagtct tctatgattt cgaagtttct tatatttggc
2761 tctgacccat cctactaggt gccactctac atttctgaga gatggataca tctctgattt
2821 ataaaacctg ccatagtact gataccagcc tcggactata ggatttattt tctttgatat
2881 tttctctagt gttgtatatg tttgcctacg tattttccat gaccttatgg tttggttgat
2941 cttcttcttg gctttgttgc taattgcagg tagaaatgag acaaaatagt ttcctatttt
3001 attgttcttt gccagtctag gtctgaacgt gtaacccaga aagtcaaagc tctgtattgg
3061 aaactcatct ctcctattgt catccttaca gtacactatt tgtgtctttt caggatgtaa
3121 ttttagctta aactcagcca atctttcttc gatcttgtct tttatgaatt ttgcctgttt
3181 ctgggtttta cagtgcacca ttgcatcatc tacatatctc tcaaacggta ttgttgggta
3241 gttttgcctc atccacatat caaatgcatg atgcataaat atgttagaaa tgattgggct
3301 tacagaacct ccttgtggaa ctcccttatt cctaactacc ttactgccat ctgctagttg
3361 aatcggggct ttcatccacc tttccacgta cagtatgacc cacttgcaat ctgtatgctt
3421 cttgatagtt tgcagtgcta acacatgatc cagattgtcg aaaaatccag aaatatcaag
3481 atctatcgtc caatcacttc tccaacatct cttacacgct gtacttaccg catccagtgc
3541 agacttgttt ggtctataac catatgaatc ttcgtgaaat tccggctcta ctaacggttc
3601 gagatacata gtagctgctg tttgccctat cctgtcgaat actgaaggaa cacataaagt
3661 tctttgtcct cctgtatctt ttggtatcgc aacagctttt actgattctg gaaaataact

3721 tccggatgac atccgattcc acagtttgta tagattatct tttagatttt cttcaaactt
3781 tgttatcgag acctcatcta cacctgccgc acctctgttt ttggatactt gtttataagc
3841 tttccaaatc agttgctttg gtatgtcaaa agattttgtt ttattcatta actcttccct
3901 ttcggttgat aaataattaa aactaaataa ctcagcccct tcgctccatt tccattacaa
3961 aaacttcttc actactacaa gctaatccgc ccctgttttt cgcatcagta ctctcttcct
4021 tagagttcag ctccttggac ttctccctta tcatcgaaac gacaggttcc cgtagttcca
4081 cacaatagcc tgaaataggt tcatgccacc tatatgccgg atgccaccta cccagtagac
4141 aagttcccga taggcttatc ccaagttact ttttctcccc tggttttgac atcatcgcat
4201 tacctttcga cgtgtaatca gtggttcact tgcgttcatc tctctatttc acacatgacg
4261 cataattacg ccttttccat aacgctcact accttggctc tttaccaaag cagcttatgg
4321 ttgtttgaag cctgctcttg taaaccggct ccgaggggtc caccctcatc tattgtgcag
4381 ctttcgcact tagtttgctc tttcctgaac attccttgta tctctacggc acacaaaaag
4441 cattaataat tatacataat tctaatgatc ttcatgaaag ggcttagaat agaatttaat
4501 agacaatctg attttaattg atattaaact gatcagactg agagttgaaa atatggcaat
4561 aattctttta gacacaaaaa ctataaatcg tatagcagcg ggagaggtaa tagaaaggcc
4621 agcgagtgta gtaaaggaat tagtagaaaa tgcaatagat gctggaagtt cagagataga
4681 gatcaaaata gaaagtggtg gacgtaacct tataactgtg acagatgatg gaaatggaat
4741 agaaaaggac gacttagaac tagcatttat gaggcatgcc acttcaaaat taagcgatag
4801 tgagttaata gaaatcaagc accttgggtt taggggagaa gctttacctt caattgcagc
4861 agtaagcaga atgaaattat cgtctaaggc aagtggagca aaggaatcat ggtctataag
4921 gtatgaggga ggagaaaaaa taagagagat taccccttgt tctttgttgc aaggtacata
4981 tattgaagtt cgtgacttat tttttgctac accaaataga ctaaagtttc taaaaaccga
5041 aagggcagaa acacaaagta ttgttgatat tgtgaataac ttagcgatga ttaattatag
5101 tattgggttt actctcactt ctggtaataa aaagctctta aaatatgtta agcaaacttc
5161 gctgttcaac agattatgtg aaatagaaga agaatttcaa agcaattcac tggaagttaa
5221 agaggaagaa gaaggcatca aacttaaggg acacatctgt aaacctaatg tcaatcgtgg
5281 caactcaact cagatttata cgtttgttaa tggaaggcca ataaaagaca atctacttgt
5341 tggtgcaatt agatatgcgt atcacgattt tattccaaac aataggtatc cttttgcagt
5401 gttgcattta gaagtaccat atgaccaagt agatgtaaat gtgcatccaa ataaatcgga
5461 agtaagattt cagaataaaa ggctaatata tgaaatagtg agaagaggga taataaaaac
5521 actatcaacg agatttgcag cgggtgatca aggtattgaa gaggagctaa tttttaatga
5581 tagtaaaagc caagagcagg ttgatagtca agagaaaaaa gatcaaaaag agttttatga
5641 aaagagacca agtcttttag aaaatcgtct aatgaaagaa ttcaatgcac cagatgaaag
5701 aaggcaaagc ttaccagaaa cgtttaaata tggagaatct ccaccccaaa agggaacgat
5761 ggttttagaa aggaagcaaa ttgatttaat agaggattat cctctgggat attcacgctg
5821 tcaggtctac aatacttaca ttattgctga ggctaaaggc aaattaatta tagtagacca
5881 gcacgcagct catgagaggt tgatctatga gtgcttaaaa caaaaatcaa gcataaaaag
5941 gcaaaagctt cttcttcctg aaatagttga gattaaaaac caagcaggaa tggagatggt
6001 tgaaatgtat aaagataagc ttttcgaaat gggttttggt attgaaatag aatcagaaga
6061 taaagtaagg gtgaaagaaa tacctgcaat tttaggaaca ataaatgtga aagagatggt
6121 gatgaatata gttgatagat taatggagat aggagatacg ctacctatag aagaaaaagt
6181 gaacaagata ctagctacca ttgcgtgcca tgggtcaatt agagctggaa gggaaatgaa
6241 gttggaggag atgaatgagt tgatgagaca aatggaggaa acaccgtatg ctgggcaatg
6301 caatcacgga agaccaacgt atatagaaat gaaactaagt gatatagaaa aattgtttga
6361 aaggagatga gttcagtttg tcaaaaaggt tcccaattaa gtatacgtaa cggctcttct
6421 gattatgagt ataaaatgtg aaaaaagaga ataaatgctc taatttttta gattacaaag
6481 taataggaca ggaagtaaga aatcgtaggt tagcaaaggg atatactcaa aaagatttag
6541 caaaaaaaat cgatacaaca tatcaggtaa tactgcaata tgaaaaagga acacgcagaa
6601 tttcaattaa gaagttatat gaattagcag aagcattatc aacaactgct agagatctag
6661 cttgcgtaca agaagtatca aatgagggaa ggtatgaggg agaagaggta ttaaatctag
6721 taagaagaca tagagagatt aaggatcaag aattacgtga aacgttttat ttattaacta
6781 aattcatccg tattagtgag gaagaaagtg gaaaggcggt aaaaatagaa gtagcaaagg
6841 gtttggttaa ggaaggagtt tctgctcatg ttatctctca aacgaccagt ttatctattg
6901 atgaatatga taatgatgag aaaaaaattt ccattccgta taaagtaggt caaagaataa
6961 aagaatggag gttgagaaga ggatacactc aagaagattt agcaagtaaa gtgggtataa
7021 taaatcaaag aatatatgaa tatgaacaag gacgagctgg tgtttcactt gaaatgttag
7081 atgaaatagc aaaggtacta ttaattagta ttacagacct acttccagaa acaacagaaa
7141 atgagaatag tgaagtggaa ctatcaaggt taatagaaga atacaaaaag attaaaagcc
7201 aagaactacg taatgtacta ataaaatctc tgtttgaaag catacaagtt tgcaaagaga
7261 aagtgaagag agtagaaaag atgaaaatta caaagaattt agttaaggaa ggaatttcta

```
     7321 tcaatattat tttaaaaaca gtagacatct ctttggacga aattcaacaa atttaaaata
     7381 aaaaatcatt atctattaat atctttactt tttgatgaaa aaataagtaa aaaagattga
     7441 aaaattgatt tgacttcact caaaagctat ggccttatgc caatgctgac aaaaaaacag
     7501 cagtaaatat ataaaaacat tttgttatta gtgaagggta atttttatga ataataagga
     7561 aaacagtaaa gatcgtagag aagaaataga attcagagca ttagaaagca acggtaaagc
     7621 actacttgat cgtgaagtaa tagaaacgtt tctaagtgca gtgcatgaca gggaagaagc
     7681 tcgaattatt gctagaaagc taatagatag aggttgtcc
//
LOCUS       JAQZAU010000066          42782 bp    dna     linear   ENV 17-MAR-2023
DEFINITION  MAG: Wolbachia pipientis isolate wEsol contig_38312, whole genome
            shotgun sequence.
ACCESSION   JAQZAU010000066 JAQZAU010000000
VERSION     JAQZAU010000066.1
DBLINK      BioProject: PRJNA929258
            BioSample: SAMN33142973
KEYWORDS    WGS; ENV; Metagenome Assembled Genome; MAG.
SOURCE      Wolbachia pipientis (insect metagenome)
  ORGANISM  Wolbachia pipientis
            Bacteria; Pseudomonadota; Alphaproteobacteria; Rickettsiales;
            Anaplasmataceae; Wolbachieae; Wolbachia.
REFERENCE   1  (bases 1 to 42782)
  AUTHORS   Fiutek,N., Couger,B.M., Roy,S., de la Torre,J.R. and Connor,E.F.
  TITLE     Genomic assessment of the contribution of the Wolbachia
            endosymbiont of Eurosta solidaginis to gall-induction
  JOURNAL   International Journal Molecular Sciences
REFERENCE   2  (bases 1 to 42782)
  AUTHORS   Fiutek,N., Couger,B.M., Roy,S., de la Torre,J.R. and Connor,E.F.
  TITLE     Direct Submission
  JOURNAL   Submitted (10-FEB-2023) Biology, San Francisco State University,
            1600 Holloway Ave, San Francisco, CA 94132, USA
COMMENT     ##Genome-Assembly-Data-START##
            Assembly Date          :: JUN-2019
            Assembly Method        :: FLYE v. 06-2019
            Genome Representation  :: Full
            Expected Final Version :: Yes
            Genome Coverage        :: 100.0x
            Sequencing Technology  :: Illumina HiSeq; PacBio Sequel
            ##Genome-Assembly-Data-END##
FEATURES             Location/Qualifiers
     source          1..42782
                     /organism="Wolbachia pipientis"
                     /mol_type="genomic DNA"
                     /submitter_seqid="contig_38312"
                     /isolate="wEsol"
                     /isolation_source="galls on Solidago altissima"
                     /host="Eurosta solidaginis MN-2018-EFC"
                     /db_xref="taxon:955"
                     /environmental_sample
                     /country="USA: Cowling Arboretum, Carleton College,
                     Northfield, MN"
                     /lat_lon="44.48 N 93.13 W"
                     /altitude="280 m"
                     /collection_date="2018-11"
                     /metagenome_source="insect metagenome"
                     /note="metagenomic"
     gene            complement(<1..143)
                     /locus_tag="wEsol_00695"
     CDS             complement(<1..143)
                     /locus_tag="wEsol_00695"
                     /codon_start=1
```

```
                     /transl_table=11
                     /product="hypothetical protein"
                     /translation="MRKVIKYISIIGIACLVLLFFISNVETRVKTQEEQLFLAVEDGN
                     AQE"
                     /besthit="qcoverage 100 , hcoverage 79.6610169491525 ,
                     similarity 100 , identity 100 , evalue 1.28e-21 ,
                     alnlength 47 , Bacteria , Proteobacteria , WP_223823054.1
                     ankyrin repeat domain-containing protein [Wolbachia
                     pipientis]"
     gene            complement(570..914)
                     /locus_tag="wEsol_00696"
     CDS             complement(570..914)
                     /locus_tag="wEsol_00696"
                     /codon_start=1
                     /transl_table=11
                     /fullproduct="qcoverage 100 , hcoverage 100 , similarity
                     100 , identity 100 , evalue 3.48e-72 , alnlength 114 ,
                     Bacteria , Proteobacteria , WP_015588865.1 MULTISPECIES:
                     F0F1 ATP synthase subunit epsilon [unclassified
                     Wolbachia]"
                     /cog="AtpC COG0355 135 F0F1-type ATP synthase, epsilon
                     subunit (mitochondrial delta subunit)"
                     /pfam="ATP-synt_DE_N Ub-RnfH"
                     /tigr="TIGR01216 ATP_synt_epsi 130 ATP synthase F1,
                     epsilon subunit"
                     /product="F0F1 ATP synthase
                     subunit epsilon"
                     /translation="MNTFKVQFFSPDDQISFSGVVSLSVTGLEGELMILAHHAPYLIY
                     LLPGMITVKMSNQIEKKVVIDSGVLEVANNNCSIITSQIQVFDRAIHDEKSFKNKRIS
                     IYLSYLDEKFLS"
                     /besthit="qcoverage 100 , hcoverage 100 , similarity 100
                     , identity 100 , evalue 3.48e-72 , alnlength 114 ,
                     Bacteria , Proteobacteria , WP_015588865.1 MULTISPECIES:
                     F0F1 ATP synthase subunit epsilon [unclassified
                     Wolbachia]"
     gene            complement(927..2378)
                     /locus_tag="wEsol_00697"
     CDS             complement(927..2378)
                     /locus_tag="wEsol_00697"
                     /codon_start=1
                     /transl_table=11
                     /fullproduct="qcoverage 100 , hcoverage 100 , similarity
                     100 , identity 99.8 , evalue 0.0 , alnlength 483 ,
                     Bacteria , Proteobacteria , CDR78583.1 ATP synthase F0F1
                     subunit beta,ATP synthase subunit beta,F0F1 ATP synthase
                     subunit beta,F0F1-type ATP synthase, beta subunit,ATP
                     synthase F1, beta subunit,ATP synthase alpha/beta family,
                     nucleotide-binding domain [Wolbachia endosymbiont of
                     Drosophila simulans wAu]"
                     /cog="AtpD COG0055 468 F0F1-type ATP synthase, beta
                     subunit"
                     /pfam="ATP-synt_ab_N ATP-synt_ab ATP-synt_ab_C"
                     /tigr="TIGR01039 atpD 462 ATP synthase F1, beta subunit"
                     /product="ATP synthase F0F1 subunit beta,ATP
                     synthase subunit beta,F0F1 ATP synthase subunit
                     beta,F0F1-type ATP synthase, beta subunit,ATP synthase
                     F1, beta subunit,ATP synthase alpha/beta family,
                     nucleotide-binding domain"
                     /translation="MLGDKNKEMNIGRAIKVTQAVVDIKFEGELPKIFNALKSKLKYK
                     DKELVLEVSQHIGDNIVRCIAMDSTDGMSRGDEFVDTGAPISVPIGRSTLGRIFNVVG
```

ELIDECGPLKGKYNLEPIHRAPPSFTEQRIQEEVLVTGIKVIDLLAPYLKGGKIGLFG
GAGVGKTVLIMELINNIAKAHKGFSVFAGVGERTREGNDLYHEMITSNVININEHEKS
QAVLVYGQMNEPPGARARIALTALTMAEYFRDRENQDVLFFVDNIFRFTQAGSEISAL
LGRIPSAVGYQPTLATDMGAMQERIASTTSGSITSVQAIYVPADDLTDPAPATTFSHL
DATTVLSRQIAEMGIYPAVDPLDSTSQSLSAEIIGEEHYNVASEVKRILQTYKSLQDI
IAILGMDELSDEDKIIVDRARKIQKFLSQPFHVAEIFTGMPGKFVSLSDTVSSFKGIV
EGKYDHLPEAAFYMVGNIDEAIKKAELIQAEAK"
/besthit="qcoverage 100 , hcoverage 100 , similarity 100
, identity 99.8 , evalue 0.0 , alnlength 483 , Bacteria ,
Proteobacteria , CDR78583.1 ATP synthase F0F1 subunit
beta,ATP synthase subunit beta,F0F1 ATP synthase subunit
beta,F0F1-type ATP synthase, beta subunit,ATP synthase
F1, beta subunit,ATP synthase alpha/beta family,
nucleotide-binding domain [Wolbachia endosymbiont of
Drosophila simulans wAu]"

gene complement(2456..3556)
/locus_tag="wEsol_00698"

CDS complement(2456..3556)
/locus_tag="wEsol_00698"
/codon_start=1
/transl_table=11
/fullproduct="qcoverage 100 , hcoverage 100 , similarity
99.7 , identity 99.7 , evalue 5.49e-239 , alnlength 366 ,
Bacteria , Proteobacteria , AGJ99643.1 Phosphate ABC
transporter, permease protein, putative [Wolbachia
endosymbiont of Drosophila simulans wHa]"
/cog="PstC COG0573 310 ABC-type phosphate transport
system, permease component"
/pfam="BPD_transp_1"
/tigr="TIGR02138 phosphate_pstC 296 phosphate ABC
transporter, permease protein PstC"
/product="Phosphate ABC transporter, permease
protein, putative"
/translation="MHGRMNSVIVIPVLLILLFCFSRFQEVNKVKSYLYLSCVWMLTW
LLVFYNNCFVTFISIALLFFMLAFIFKNKRNKIIKLSLFVALAISFFITLFIMLSIFI
QSISFFNKVAISEFLFCLKWGHNVVTINEEKIGCFGIAPLLVGTLLITIIAMLVVVPL
GLFSAIYISEYASEKVRYIVNTTLQVLSAIPTVVYGYFAVVFLSFFIKQVANFFGLSI
HSESALVAGLSIGIMILPFIISLLEDAIRSVPKSLRYGFMALGATPAETIWHITIPYA
MPTILSAILLSISRVIGETMIVLMAVGINANLTFNPLNSVTTITVQIATLLTGDQDFN
SVQTLAAYALSLVLFIITWLLNAFALFVMKRN"
/besthit="qcoverage 100 , hcoverage 100 , similarity 99.7
, identity 99.7 , evalue 5.49e-239 , alnlength 366 ,
Bacteria , Proteobacteria , AGJ99643.1 Phosphate ABC
transporter, permease protein, putative [Wolbachia
endosymbiont of Drosophila simulans wHa]"

gene 3992..4129
/locus_tag="wEsol_00699"

CDS 3992..4129
/locus_tag="wEsol_00699"
/codon_start=1
/transl_table=11
/pfam="Ribonuclease_P"
/fullproduct="qcoverage 100 , hcoverage 100 , similarity
100 , identity 100 , evalue 4.19e-22 , alnlength 45 ,
Bacteria , Proteobacteria , WP_006279896.1 MULTISPECIES:
hypothetical protein [Wolbachia]"
/translation="MLAKISILNISNIGHYYIILTHRNIMQASYKNLQKDLTICLKKI
K"
/product="hypothetical protein"
/besthit="qcoverage 100 , hcoverage 100 , similarity 100

```
                     , identity 100 , evalue 4.19e-22 , alnlength 45 ,
                     Bacteria , Proteobacteria , WP_006279896.1 MULTISPECIES:
                     hypothetical protein [Wolbachia]"
     gene            4216..4479
                     /locus_tag="wEsol_00700"
     CDS             4216..4479
                     /locus_tag="wEsol_00700"
                     /codon_start=1
                     /transl_table=11
                     /fullproduct="qcoverage 100 , hcoverage 100 , similarity
                     100 , identity 100 , evalue 2.51e-57 , alnlength 87 ,
                     Bacteria , Proteobacteria , WP_141456820.1 MULTISPECIES:
                     hypothetical protein [unclassified Wolbachia]"
                     /translation="MVEHIKEVNNTGFGQMYQFLFLPKGTGTDWRSAFRQQEELFKKV
                     GGNVILPEISNSPSKGNQLGEVGTMLNNVENDQEHIRKKRYHS"
                     /product="hypothetical protein"
                     /besthit="qcoverage 100 , hcoverage 100 , similarity 100
                     , identity 100 , evalue 2.51e-57 , alnlength 87 ,
                     Bacteria , Proteobacteria , WP_141456820.1 MULTISPECIES:
                     hypothetical protein [unclassified Wolbachia]"
     gene            7839..8915
                     /locus_tag="wEsol_00701"
     CDS             7839..8915
                     /locus_tag="wEsol_00701"
                     /codon_start=1
                     /transl_table=11
                     /pfam="TPR_11 TPR_12 TPR_2 TPR_1 TPR_8 Apc3 TPR_7
                     Ubie_methyltran Methyltransf_32 CheR Methyltransf_23
                     Methyltransf_16 MTS NodS Methyltransf_31 MetW
                     Methyltransf_18 Methyltransf_26 Methyltransf_25
                     Methyltransf_12 Methyltransf_11"
                     /fullproduct="qcoverage 100 , hcoverage 100 , similarity
                     100 , identity 100 , evalue 3.61e-265 , alnlength 358 ,
                     Bacteria , Proteobacteria , WP_015588862.1 MULTISPECIES:
                     methyltransferase domain-containing protein [unclassified
                     Wolbachia]"
                     /translation="MKSNLSFIKNVALNCMSKLIGVLNIKKVKHFIVNKYNTLHKEMT
                     VLLKKSKNLLNTNIEIGLYHFYKGSISDAKLRFWLISIFYPHLPIVWYNIGRCHFAVG
                     NTNKAYNYLTRALKLDNDHEEASYYIKKMTNSAPITELPKNLIRQYFDYTGEYFVEHW
                     LIAKQYRGHELVHMIITKIFNNSTSELNILDLGCGTGICGHFLKINSIGNHIIGIDIS
                     SRMLNIARGCFIKGKPVYNELIHMEMKEFLKQEKNQQYDVIIFAEVLHYLHDFQEELE
                     LAKRSTSKKGVIVCLIRRKEGEGIDFVNKGDYFRHSESYVQHVAKEINMQISYMSYCK
                     IYGSQVDGILFALQHQQENPKNLT"
                     /product="methyltransferase
                     domain-containing protein"
                     /besthit="qcoverage 100 , hcoverage 100 , similarity 100
                     , identity 100 , evalue 3.61e-265 , alnlength 358 ,
                     Bacteria , Proteobacteria , WP_015588862.1 MULTISPECIES:
                     methyltransferase domain-containing protein [unclassified
                     Wolbachia]"
     gene            8974..10758
                     /locus_tag="wEsol_00702"
     CDS             8974..10758
                     /locus_tag="wEsol_00702"
                     /codon_start=1
                     /transl_table=11
                     /pfam="Ribonuc_red_lgN Ribonuc_red_lgC Ribonuc_red_lgC"
                     /fullproduct="qcoverage 100 , hcoverage 100 , similarity
                     100 , identity 100 , evalue 0.0 , alnlength 594 ,
                     Bacteria , Proteobacteria , WP_006280151.1 MULTISPECIES:
```

ribonucleoside-diphosphate reductase subunit alpha
[Wolbachia]"
/translation="MNNITINYAKDSKLTDFGKAVLSDRYLIENESYQDLFARIANYY
SDNKEHAQRLYDYMSNLWFMPSTPILSNGGTKRGLPISCFLNETEDSLQGIVDLWNEN
VWLAARGGGIGSYWGNLRSIGESVKGSGKTSGIVPFIVVQNALTLAISQGSLRRGSSA
VYLPVSHPEIEEFLDLRKPTGGDPNRKALNIHHAVIVTDKFMQAVENDQEWNLISPHN
NKVISTVKARDIWIKILTARVETGEPYIIFLDATNNNKPESYKKLNLDIKMSNLCSEI
TLTTGYDHLNKSRTAVCCLSSVNLEYYEEWKDNKLFIEDIMRFLDNVLEDFINKAPNE
MQRAKYSAARERSIGLGVMGFHSFLQSKMVPFESVTAQQWNKKIFKYLREQADIVSKK
LAEEKGACSDAKEVDLMERFTHKLAIAPTASISIIAGNTSPGIEPYAANVFIQKTLTG
SFVVRNKFLQKLLAEKNQDNDKIWSSISTNEGSVQHLDFLSEHEKLTFKTAYELDQRW
IIEHASDRTSYICQSQSVNLFLPANVHKRYLHKIHMLAWKKGLKSLYYCRSQSMQRAD
KVSHDIFKKSEILQQKTDIDYDECLSCQ"
/product="
ribonucleoside-diphosphate reductase subunit alpha"
/besthit="qcoverage 100 , hcoverage 100 , similarity 100
, identity 100 , evalue 0.0 , alnlength 594 , Bacteria ,
Proteobacteria , WP_006280151.1 MULTISPECIES:
ribonucleoside-diphosphate reductase subunit alpha
[Wolbachia]"
gene 11726..13288
/locus_tag="wEsol_00703"
CDS 11726..13288
/locus_tag="wEsol_00703"
/codon_start=1
/transl_table=11
/pfam="GATase Peptidase_C26 GATase_3 NAD_synthase
Pfam-B_78 Asn_synthase tRNA_Me_trans PAPS_reduct
GMP_synt_C"
/fullproduct="qcoverage 100 , hcoverage 100 , similarity
100 , identity 100 , evalue 0.0 , alnlength 520 ,
Bacteria , Proteobacteria , WP_141456818.1 MULTISPECIES:
glutamine-hydrolyzing GMP synthase [unclassified
Wolbachia]"
/translation="MSAIAIIDFGSQFTQLIARQVRGMGVYCEIFPSNISFETISKFN
GFILSGGPQSVNDDCSETSRVVHEIIKLNEATSVPILGICYGQQLICHYFGAKVKESF
KQEFGRTKIKILKESPIVKDTWDVNSEVDVLMNHADSVDTIPQGFTVIASGVINQTIA
MIVNEQRKIYCTQFHPEVRPTTNGSKLLSNFLDIANCERDWTMKSFIEEQKEKIKNVV
GEKKVIAAVSGGVDSSVAAALTYKAIGKQLNCIFIDTGLLRKNQTIAMLKEIPINYVD
KSNLFLSRLKGITDPEEKRKIIGNTFIEVFEEEAKKIGDVDFLMQGTIYSDVVESGHA
SDNTSTIKSHHNVGGLPEKMNLKLVEPLRYLFKDEVRLLGKEIGLSDEIIFQHPFPGP
GLAVRIISEVDKEKVQILQEVDEIYINTMKNYDLYDKIWQAFAVLLPIKTVGVMGDGR
TYGYVCALRAVTSSDGMTADAFPFENKDQHLLVFWDFLQNVGSIIVNNVPGVNRVVYD
ITSKPPATIEWE"
/product="
glutamine-hydrolyzing GMP synthase"
/besthit="qcoverage 100 , hcoverage 100 , similarity 100
, identity 100 , evalue 0.0 , alnlength 520 , Bacteria ,
Proteobacteria , WP_141456818.1 MULTISPECIES:
glutamine-hydrolyzing GMP synthase [unclassified
Wolbachia]"
gene complement(13321..14028)
/locus_tag="wEsol_00704"
CDS complement(13321..14028)
/locus_tag="wEsol_00704"
/codon_start=1
/transl_table=11
/fullproduct="qcoverage 100 , hcoverage 100 , similarity
100 , identity 100 , evalue 2.73e-146 , alnlength 235 ,
Bacteria , Proteobacteria , WP_038199715.1 MULTISPECIES:

```
                     membrane protein [Wolbachia]"
                     /cog="TerC COG0861 254 Membrane protein TerC, possibly
                     involved in tellurium resistance"
                     /pfam="TerC"
                     /tigr="TIGR03716 R_switched_YkoY 220 integral membrane
                     protein, YkoY family"
                     /product="membrane protein"
                     /translation="MLTDAWTLLTLTLLETILSVDNLIFISLAIDKVPNALRERVRLM
                     GLGLALLMRFVILFFTSSILSMQKPIFHTASARDLLMIAGGLFLIVKSSMELRDDIFV
                     CKKNKKKANVKSKFFLVVLQIILIDLVFSVDSILTAIALTYNMVIIATAFTFSMLAML
                     FLSSYTAQLIKSNPGLKIIAILFILLVGVYLILHGFHIELPKGYLYSSFMFALLVEII
                     SKIKKINRHTAIHSRYL"
                     /besthit="qcoverage 100 , hcoverage 100 , similarity 100
                     , identity 100 , evalue 2.73e-146 , alnlength 235 ,
                     Bacteria , Proteobacteria , WP_038199715.1 MULTISPECIES:
                     membrane protein [Wolbachia]"
     gene            14289..14402
                     /locus_tag="wEsol_00705"
     CDS             14289..14402
                     /locus_tag="wEsol_00705"
                     /codon_start=1
                     /transl_table=11
                     /fullproduct="qcoverage 100 , hcoverage 100 , similarity
                     100 , identity 100 , evalue 7.01e-20 , alnlength 37 ,
                     Bacteria , Proteobacteria , AAS13939.1 hypothetical
                     protein WD_0193 [Wolbachia endosymbiont of Drosophila
                     melanogaster]"
                     /translation="MYIKGSPSGKVFAGYQAFIPSPLPPKFEWDNDLVNSL"
                     /product="hypothetical protein"
                     /besthit="qcoverage 100 , hcoverage 100 , similarity 100
                     , identity 100 , evalue 7.01e-20 , alnlength 37 ,
                     Bacteria , Proteobacteria , AAS13939.1 hypothetical
                     protein WD_0193 [Wolbachia endosymbiont of Drosophila
                     melanogaster]"
     gene            14426..14608
                     /locus_tag="wEsol_00706"
     CDS             14426..14608
                     /locus_tag="wEsol_00706"
                     /codon_start=1
                     /transl_table=11
                     /fullproduct="qcoverage 100 , hcoverage 100 , similarity
                     100 , identity 100 , evalue 2.84e-33 , alnlength 60 ,
                     Bacteria , Proteobacteria , WP_006279522.1 MULTISPECIES:
                     hypothetical protein [Wolbachia]"
                     /translation="MVENLGVVFTTAQRAIVKLEDLGIVSQTSQGKRDRVYCATDILN
                     ILEEPTKITENFDSTL"
                     /product="hypothetical protein"
                     /besthit="qcoverage 100 , hcoverage 100 , similarity 100
                     , identity 100 , evalue 2.84e-33 , alnlength 60 ,
                     Bacteria , Proteobacteria , WP_006279522.1 MULTISPECIES:
                     hypothetical protein [Wolbachia]"
     gene            14794..15612
                     /locus_tag="wEsol_00707"
     CDS             14794..15612
                     /locus_tag="wEsol_00707"
                     /codon_start=1
                     /transl_table=11
                     /pfam="Ank_2 Ank_5 Ank Pfam-B_3279"
                     /fullproduct="qcoverage 100 , hcoverage 100 , similarity
                     100 , identity 100 , evalue 1.47e-173 , alnlength 272 ,
```

```
                     Bacteria , Proteobacteria , WP_141456816.1 MULTISPECIES:
                     ankyrin repeat domain-containing protein [unclassified
                     Wolbachia]"
                     /translation="MINNLDSIILGIKDGEKAIEDLRVVLKKRKEEVITTQTFNYALR
                     CPKTTKEAIVHEILLHFIQNPGEQSLEQVIQCLDRAIQSGIYAKNNPIRNLDEEFRTH
                     INFKDEEGNTLLHHAVIGNKTEEIITLLVTYSANPLIQNADNKIPLDLAQGETKEVLI
                     KSMKKQANTKKESAMVGSLVPGVIIGGFLGVVLGAGVCVAVSLSGGMILGVMIASSLV
                     ASIAIGLAMYFLSQDYEQAKAIEKTISTVSSEISVDDTTAANKNNKEGPQLTYS"
                     /product="ankyrin repeat
                     domain-containing protein"
                     /besthit="qcoverage 100 , hcoverage 100 , similarity 100
                     , identity 100 , evalue 1.47e-173 , alnlength 272 ,
                     Bacteria , Proteobacteria , WP_141456816.1 MULTISPECIES:
                     ankyrin repeat domain-containing protein [unclassified
                     Wolbachia]"
     gene            16037..18601
                     /locus_tag="wEsol_00708"
     CDS             16037..18601
                     /locus_tag="wEsol_00708"
                     /codon_start=1
                     /transl_table=11
                     /fullproduct="qcoverage 100 , hcoverage 100 , similarity
                     100 , identity 99.9 , evalue 0.0 , alnlength 854 ,
                     Bacteria , Proteobacteria , AGJ99635.1 DNA mismatch
                     repair protein MutS [Wolbachia endosymbiont of Drosophila
                     simulans wHa]"
                     /cog="MutS COG0249 843 Mismatch repair ATPase (MutS
                     family)"
                     /pfam="MutS_I MutS_II MutS_III MutS_IV MutS_V"
                     /product="DNA mismatch repair protein MutS"
                     /translation="MCNIIYNNVYQYNILKNIMSFVKEKNTPVMEQYLNLKAQYKDHL
                     LFYRLGDFYELFFDDAIKAAKLLNIVLTKRGNSCGQEIPMCGVPAHSSESYLHKLIDL
                     GFKVAICDQLETADEAKKRGYKSIVKRDVVRVVTPGTIIEDSLLEDKSNNYLASIVEQ
                     NDEYAISWLELSTGKFFHTLTSLKALDSDLLRISPRELLISEKFTEDEKIRSILKNYK
                     ISITQHAQSFFEYSKSHRTLCEFYKIRELGSIGNFSKVEIMACGALLEYVRVTQRGSI
                     PRLEFPKTYKQQNFMLIDASARRNLELFSTQFGEKKGSLISVIDHTVTASGGRLLKQM
                     LASPLACSKAINLRLSTAQFFVNNHEPRRKIREILSNIPDIERSLSRLILGRGSPKDM
                     NLLKIGLGKTLELSEFLSTLHNYCLSEKSEINSQMSFQCLTGEKEPVSTTQVISSDES
                     ELSTIHKSLGNHKDLFELLNSAILDNNLSSVKEGGFIYPKYNSELSELSYILNNSNKL
                     VTKLRESYRDLTGIAALKILHNNILGYYVEVSANHKITSDIFIHRQSLANSMRYTTNE
                     LKELENKILTARDAAIGLEMKIFSELCSEVAKESEKIALAANALAKLDIRTAFAELAV
                     QNNYVKPIIDDSKEFNICSGRHPVVEVNDKFIANSINLAGIHLITGPNMAGKSTFLRQ
                     NALIAILAHMGSFVPAESVHIGVIDKIFSRVGATDNITAGYSTFMVEMIETATIVNQA
                     TDRSLVILDEIGRGTGVYDGLSIAQAVIEHIHNVNKCRAIFATHYHELTKVSKYLKNV
                     KCFCVKIREWNGEVIFLHEVIEGIADESYGIHVAKLAGFPDSVLNRASEVFEELKA"
                     /besthit="qcoverage 100 , hcoverage 100 , similarity 100
                     , identity 99.9 , evalue 0.0 , alnlength 854 , Bacteria ,
                     Proteobacteria , AGJ99635.1 DNA mismatch repair protein
                     MutS [Wolbachia endosymbiont of Drosophila simulans wHa]"
     gene            complement(18923..19099)
                     /locus_tag="wEsol_00709"
     CDS             complement(18923..19099)
                     /locus_tag="wEsol_00709"
                     /codon_start=1
                     /transl_table=11
                     /fullproduct="qcoverage 100 , hcoverage 100 , similarity
                     100 , identity 100 , evalue 1.44e-32 , alnlength 58 ,
                     Bacteria , Proteobacteria , RLT60659.1 hypothetical
                     protein WANA31_0944 [Wolbachia endosymbiont of Drosophila
                     ananassae]"
```

```
                     /translation="MKKKAKEALGLLSALKYWRFLCFKRLTSEIQPLLIATNLRCKCL
                     RNLPSRKKDKESPS"
                     /product="hypothetical protein"
                     /besthit="qcoverage 100 , hcoverage 100 , similarity 100
                     , identity 100 , evalue 1.44e-32 , alnlength 58 ,
                     Bacteria , Proteobacteria , RLT60659.1 hypothetical
                     protein WANA31_0944 [Wolbachia endosymbiont of Drosophila
                     ananassae]"
     gene            complement(19591..19989)
                     /locus_tag="wEsol_00710"
     CDS             complement(19591..19989)
                     /locus_tag="wEsol_00710"
                     /codon_start=1
                     /transl_table=11
                     /fullproduct="qcoverage 100 , hcoverage 100 , similarity
                     100 , identity 100 , evalue 6.82e-92 , alnlength 132 ,
                     Bacteria , Proteobacteria , WP_007548550.1 MULTISPECIES:
                     hypothetical protein [Wolbachia]"
                     /translation="MVDFIDLFQGSKDFTMCMHNGSGDAIYNRCISAVRMDPPCMHGE
                     SIQGLFESSFEQGVVTVRVNLDLETLNVVVYKSPPTSDKLDAFSLAYQIDRYHIDENT
                     IVFDEKVNTIKFDISNPLKHKFITFEIFKR"
                     /product="hypothetical protein"
                     /besthit="qcoverage 100 , hcoverage 100 , similarity 100
                     , identity 100 , evalue 6.82e-92 , alnlength 132 ,
                     Bacteria , Proteobacteria , WP_007548550.1 MULTISPECIES:
                     hypothetical protein [Wolbachia]"
     gene            20063..21301
                     /locus_tag="wEsol_00711"
     CDS             20063..21301
                     /locus_tag="wEsol_00711"
                     /codon_start=1
                     /transl_table=11
                     /fullproduct="qcoverage 98.7864077669903 , hcoverage
                     99.5110024449878 , similarity 98.8 , identity 97.5 ,
                     evalue 1.49e-286 , alnlength 407 , Bacteria ,
                     Proteobacteria , ERN55977.1 ribonuclease D [Wolbachia
                     pipientis wMelPop]"
                     /cog="Rnd COG0349 361 Ribonuclease D"
                     /pfam="DNA_pol_A_exo1"
                     /tigr="TIGR01388 rnd 363 ribonuclease D"
                     /product="ribonuclease D"
                     /translation="MTLIAKFNISSKVEPLNKKHMLINTTSELEDICEGLIAKKPKFI
                     AVDTEFIRNNLIYYPKLSLIQISYGEKSFIVDALVPEIDLSFIKKIMLNQGITKVFHS
                     CRQDIESLLTVFKCIPTPIFDTQVAAMFCHYYHDFIGYSKVVEQYQGIALDKIKAKNS
                     DWLRRPLSEDQLDYAVNDVVHLYDLYQILCNKLEENNRMGWFQEEMESIVDINKYLHS
                     PKDAWKRIKFNYEVNPRLILTVKAVSEWQETLAQRYNMNRNKIINNAVIADFIEKNVE
                     HVDEILDDLKRNTKNIKDADLLEFVNIFNENERNWMQQNNTLSSNYDKSVLDILSIIL
                     DSKCKESNISRKLVSSKDELTGSISRQTDKLFKGWRYDFFGRSVESFLNTSSKFEISA
                     VKSANNITKIQSNLVENNCC"
                     /besthit="qcoverage 98.7864077669903 , hcoverage
                     99.5110024449878 , similarity 98.8 , identity 97.5 ,
                     evalue 1.49e-286 , alnlength 407 , Bacteria ,
                     Proteobacteria , ERN55977.1 ribonuclease D [Wolbachia
                     pipientis wMelPop]"
     gene            21613..22200
                     /locus_tag="wEsol_00712"
     CDS             21613..22200
                     /locus_tag="wEsol_00712"
                     /codon_start=1
                     /transl_table=11
```

/fullproduct="qcoverage 100 , hcoverage 100 , similarity 100 , identity 100 , evalue 3.09e-130 , alnlength 195 , Bacteria;Eukaryota , Proteobacteria;Arthropoda , WP_006280338.1 MULTISPECIES: dephospho-CoA kinase [Wolbachia]"
/cog="CoaE COG0237 201 Dephospho-CoA kinase"
/pfam="CoaE AAA_17 AAA_18"
/tigr="TIGR00152 TIGR00152 188 dephospho-CoA kinase"
/product="dephospho-CoA
kinase"
/translation="MIISLTGGIGVGKSFVANCFQEFGAVVFDADSVVHQLYKVDKSI
ISYAEKNFPGVVVNGEIDRTVLSKYFLAYDENWKQFQSLVHSAVLRELEFFIAKEKKI
DRKLLVLDVPLLLETKFYLYCDLIVFVHADSVVQAQRLNERNIDKEKLNLISDVQLSI
EEKRKMSDFIIDTSVSKEYVFSQVKDIVDSLNLNT"
/besthit="qcoverage 100 , hcoverage 100 , similarity 100 , identity 100 , evalue 3.09e-130 , alnlength 195 , Bacteria;Eukaryota , Proteobacteria;Arthropoda , WP_006280338.1 MULTISPECIES: dephospho-CoA kinase [Wolbachia]"

gene 22864..24288
/locus_tag="wEsol_00713"

CDS 22864..24288
/locus_tag="wEsol_00713"
/codon_start=1
/transl_table=11
/pfam="NMO"
/fullproduct="qcoverage 100 , hcoverage 100 , similarity 99.2 , identity 98.1 , evalue 0.0 , alnlength 474 , Bacteria , Proteobacteria , RLT60449.1 nitronate monooxygenase family protein [Wolbachia endosymbiont of Drosophila ananassae]"
/translation="MKDCEEVLKNKIKKIVISGKEVWPIIEGGKGIAVSDGRSSGAFA
AADAVGTFSGANAKLIDDNGKLVPLIYKGKTRNEKHSELIEYSIEAGISQARIANEIS
KGHGRIHMNVLWEMGAAERVLHGILEGAKGLIHGITCGAGMPYRLGEIAAKYQVYYYP
IISSVRAFKALWKRAYQRISSYLLGGVVYEDPWLAGGHNGLSNSEDPELPQAPFERVA
ELRSFMNEIGLSETPIVMAGGVWHLKDWEHWFNNLQIGPIAFQFGTRPLLTKESPISA
EWKKKLLTLEEGDVFLNKFSPTGFYSSAVKNNFIRELQERNSRQIKFSENASEEFNNE
FAMGSRGRKIYLTAKDKELANTWTKAGYTEAMKTPDSTVIFVTPDKFKQIREDQINCM
GCLSHCLFSNWKDHGDHSTGRKPDPRSFCIQKTLQNVIHDGDIENELMFSGHNVYKFK
QDPFYENGYVPAVKELVERILTGY"
/product="nitronate monooxygenase family
protein"
/besthit="qcoverage 100 , hcoverage 100 , similarity 99.2 , identity 98.1 , evalue 0.0 , alnlength 474 , Bacteria , Proteobacteria , RLT60449.1 nitronate monooxygenase family protein [Wolbachia endosymbiont of Drosophila ananassae]"

gene complement(24380..24544)
/locus_tag="wEsol_00714"

CDS complement(24380..24544)
/locus_tag="wEsol_00714"
/codon_start=1
/transl_table=11
/fullproduct="qcoverage 100 , hcoverage 100 , similarity 100 , identity 100 , evalue 5.23e-31 , alnlength 54 , Bacteria , Proteobacteria , WP_080717614.1 MULTISPECIES: IS3 family transposase [Wolbachia]"
/cog="Tra5 COG2801 232 Transposase and inactivated derivatives"
/pfam="rve_3 rve rve_2"

/product="IS3 family
transposase"
/translation="MSHKGCCYDNSVVESFFSSLKREILIDTSQHSAQQTRTAIFEYI
EIFYNKQRHY"
/besthit="qcoverage 100 , hcoverage 100 , similarity 100
, identity 100 , evalue 5.23e-31 , alnlength 54 ,
Bacteria , Proteobacteria , WP_080717614.1 MULTISPECIES:
IS3 family transposase [Wolbachia]"
gene complement(24534..24698)
/locus_tag="wEsol_00715"
CDS complement(24534..24698)
/locus_tag="wEsol_00715"
/codon_start=1
/transl_table=11
/fullproduct="qcoverage 100 , hcoverage 100 , similarity
100 , identity 98.1 , evalue 4.17e-28 , alnlength 54 ,
Bacteria , Proteobacteria , WP_167482801.1 hypothetical
protein [Wolbachia endosymbiont of Carposina sasakii]"
/cog="Tra5 COG2801 232 Transposase and inactivated
derivatives"
/pfam="rve"
/product="hypothetical protein"
/translation="MSSAINKQLVMDSLLMDVNKRKPAKNLLLHSDQGSQYTLQGYQY
LLSIKNIDES"
/besthit="qcoverage 100 , hcoverage 100 , similarity 100
, identity 98.1 , evalue 4.17e-28 , alnlength 54 ,
Bacteria , Proteobacteria , WP_167482801.1 hypothetical
protein [Wolbachia endosymbiont of Carposina sasakii]"
gene complement(24702..24899)
/locus_tag="wEsol_00716"
CDS complement(24702..24899)
/locus_tag="wEsol_00716"
/codon_start=1
/transl_table=11
/fullproduct="qcoverage 100 , hcoverage 100 , similarity
100 , identity 100 , evalue 1.88e-40 , alnlength 65 ,
Bacteria , Proteobacteria , WP_010082070.1 hypothetical
protein [Wolbachia endosymbiont of Drosophila incompta]"
/translation="MQKNDIRAILRRKFKIKKQQTDNRAVAPNILDQNFIVDQPNKVW
DYLHQNQGGMAIFGSNNSHVW"
/product="hypothetical protein"
/besthit="qcoverage 100 , hcoverage 100 , similarity 100
, identity 100 , evalue 1.88e-40 , alnlength 65 ,
Bacteria , Proteobacteria , WP_010082070.1 hypothetical
protein [Wolbachia endosymbiont of Drosophila incompta]"
gene complement(25121..25414)
/locus_tag="wEsol_00717"
CDS complement(25121..25414)
/locus_tag="wEsol_00717"
/codon_start=1
/transl_table=11
/fullproduct="qcoverage 100 , hcoverage 100 , similarity
99.0 , identity 99.0 , evalue 6.55e-60 , alnlength 97 ,
Bacteria , Proteobacteria , WP_155968835.1 transposase
[Wolbachia pipientis]"
/cog="COG2963 COG2963 116 Transposase and inactivated
derivatives"
/pfam="HTH_Tnp_1 HTH_23 HTH_28"
/product="transposase"
/translation="MVREYTAEFKLEAVKLTNEQRKVGQPVAKVARDLGIRDSVLGKW

```
                     MKKYNEKKSAANAFPDVAPYDKERFDLQSKSNKGKRHLKKSPGQSKRVKYFFI"
                     /besthit="qcoverage 100 , hcoverage 100 , similarity 99.0
                     , identity 99.0 , evalue 6.55e-60 , alnlength 97 ,
                     Bacteria , Proteobacteria , WP_155968835.1 transposase
                     [Wolbachia pipientis]"
     gene            complement(25585..26757)
                     /locus_tag="wEsol_00718"
     CDS             complement(25585..26757)
                     /locus_tag="wEsol_00718"
                     /codon_start=1
                     /transl_table=11
                     /pfam="Porin_4"
                     /fullproduct="qcoverage 100 , hcoverage 100 , similarity
                     99.7 , identity 99.7 , evalue 4.74e-284 , alnlength 390 ,
                     Bacteria , Proteobacteria , WP_141456814.1 MULTISPECIES:
                     porin [unclassified Wolbachia]"
                     /translation="MKLKYYLVALLSSLFLQNSASSKTILDKEIFYAELDGKIDLRFG
                     YAFNRDSFNAKPKDKTSSYSYLRFLYLQQVYPNTQMGVNVKAGVSGIANLKALDIEKL
                     DMEEWYFIIKNQELGSIEYGKRSLVSQSMLINTSKIYAAAGGVNGHWTNYANLRGDHK
                     KDGDPGYDQDKVFWVKPNIYSDYNELELGLKQSSINYISPEIYNFQLGFSYVPGKNNL
                     QYSNLIAAGLSYKNSLSDDIGFTTALTGEFARENLTDCSDGTSKNYECRNQLLHWNFG
                     LKLKLFDLDCIFSYGNGGKSGEKRNPETNNTYYMNAGIAYRSDSYKSSLTYFSSGRDI
                     AGEGTNELTSYALSLENSLFLGTSYYFDIVKFSTKEPKIENNNSGYVFLAGLKLSF"
                     /product="porin"
                     /besthit="qcoverage 100 , hcoverage 100 , similarity 99.7
                     , identity 99.7 , evalue 4.74e-284 , alnlength 390 ,
                     Bacteria , Proteobacteria , WP_141456814.1 MULTISPECIES:
                     porin [unclassified Wolbachia]"
     gene            complement(26820..27182)
                     /locus_tag="wEsol_00719"
     CDS             complement(26820..27182)
                     /locus_tag="wEsol_00719"
                     /codon_start=1
                     /transl_table=11
                     /product="hypothetical protein"
                     /translation="MSPLFLELYVILNFAFQHQINAAIGMIGLNSSCSETNDALKVVI
                     EHFYKNCRSDDFLGYIDENKVLFVLINCDVKSTSKIINRIHSAINKQLLMQKLPSVSI
                     IYGNIAQKQAAKSTSGAF"
                     /besthit="qcoverage 95.8333333333333 , hcoverage
                     46.7479674796748 , similarity 100 , identity 99.1 ,
                     evalue 4.08e-71 , alnlength 115 , Bacteria ,
                     Proteobacteria , ONI56753.1 hypothetical protein
                     N500_0192 [Wolbachia pipientis wUni]"
     gene            complement(27221..27637)
                     /locus_tag="wEsol_00720"
     CDS             complement(27221..27637)
                     /locus_tag="wEsol_00720"
                     /codon_start=1
                     /transl_table=11
                     /fullproduct="qcoverage 100 , hcoverage 88.4615384615385
                     , similarity 97.1 , identity 96.4 , evalue 1.05e-86 ,
                     alnlength 138 , Bacteria , Proteobacteria , EAL59419.1
                     conserved hypothetical protein [Wolbachia endosymbiont of
                     Drosophila ananassae]"
                     /translation="MDDFITARRKDDAVVSVCQDNKKKNVLILGLNQAARNLLKYEEG
                     NLLNKPLINILSARAADDMKNYLEYTENGHDLLDILPKVIDFSLTDAKGEDIRTKVKV
                     FRTAQFASNKINYELLIRDISLSHKLGIFRDEISNG"
                     /product="conserved hypothetical protein"
                     /besthit="qcoverage 100 , hcoverage 88.4615384615385 ,
                     similarity 97.1 , identity 96.4 , evalue 1.05e-86 ,
```

```
                     alnlength 138 , Bacteria , Proteobacteria , EAL59419.1
                     conserved hypothetical protein [Wolbachia endosymbiont of
                     Drosophila ananassae]"
     gene            28218..28658
                     /locus_tag="wEsol_00721"
     CDS             28218..28658
                     /locus_tag="wEsol_00721"
                     /codon_start=1
                     /transl_table=11
                     /product="hypothetical protein"
                     /pfam="adh_short_C2"
                     /translation="MQCIYRAILFTALAQRAEKMMSGGGSLLTLSYYGAEKVMPNYNV
                     MGLCKAALEASVKYIACDLGPQNIRVNAISAGPIRTLASSGISDFHFISEWNRNNSPL
                     RRNVTIEDVGKAALYLLSDLSSGTTGEILHVDSGYNVVGMKVVD"
                     /cog="FabI COG0623 259 Enoyl-"
                     /besthit="qcoverage 93.8356164383562 , hcoverage
                     52.6923076923077 , similarity 100 , identity 100 , evalue
                     1.13e-90 , alnlength 137 , Eukaryota , Arthropoda ,
                     GFY65360.1 enoyl-[acyl-carrier-protein] reductase 1
                     [Trichonephila inaurata madagascariensis]"
     gene            complement(28655..30163)
                     /locus_tag="wEsol_00722"
     CDS             complement(28655..30163)
                     /locus_tag="wEsol_00722"
                     /codon_start=1
                     /transl_table=11
                     /fullproduct="qcoverage 100 , hcoverage 100 , similarity
                     100 , identity 99.8 , evalue 0.0 , alnlength 502 ,
                     Bacteria , Proteobacteria , WP_015588851.1 MULTISPECIES:
                     protein translocase subunit SecD [unclassified
                     Wolbachia]"
                     /cog="SecD COG0342 506 Preprotein translocase subunit
                     SecD"
                     /pfam="Sec_GG SecD_SecF MMPL"
                     /tigr="TIGR01129 secD 397 protein-export membrane protein
                     SecD"
                     /product="protein translocase subunit SecD"
                     /translation="MYNRLIVKSFSVLCICLFALYITLPNFFDNKLFISKKRINLGLD
                     LKGGASLLLNIDLDFYFKEKLSMLANEIKETLLTKNIESNVQNSIITLNNIDDYKKAS
                     VLINAINPNLELNRKDSSILISYKPHYKNSLISEVAAESINNVQRRLDKLGTKEVSVQ
                     KQGQNKILVQVPGVEDTKQIKSLLGKTAKLAFHLANTNIAKVQDIDHETTVMLKDSLG
                     NSYPIFRKTEIGGDSLVNASVRFGHLGKPTVHFKFDSIASKRFAKITKENVGKPFAIV
                     LDNTVLTVPTIREPILNGEGEISGNFTEKQASELAILLKSGALPAPLKIIEEKNIGPS
                     LGEESIKAGEMAATISIIAVALFIIITYGKLGVLASVALFSNVILILSILTLLEATLT
                     LPGIAGIALTVGMAVDANVLIFERIREEIKSGKRVERAIEEGFKNAIKTILDSNITTL
                     IAAGIMFIIGSGAIRGFSVTLSIGILCSMFSAITVTKLLIELCMNPKELVLC"
                     /besthit="qcoverage 100 , hcoverage 100 , similarity 100
                     , identity 99.8 , evalue 0.0 , alnlength 502 , Bacteria ,
                     Proteobacteria , WP_015588851.1 MULTISPECIES: protein
                     translocase subunit SecD [unclassified Wolbachia]"
     gene            complement(30668..30820)
                     /locus_tag="wEsol_00723"
     CDS             complement(30668..30820)
                     /locus_tag="wEsol_00723"
                     /codon_start=1
                     /transl_table=11
                     /fullproduct="qcoverage 100 , hcoverage 100 , similarity
                     100 , identity 100 , evalue 6.45e-28 , alnlength 50 ,
                     Bacteria , Proteobacteria , WP_007548649.1 transposase
                     [Wolbachia endosymbiont of Drosophila ananassae]"
```

```
                     /translation="MQENGIKTKLRRRFKNKKQQISNRVVAPNTLDQNFTTDQPNKVW
                     DYLHKN"
                     /product="transposase"
                     /besthit="qcoverage 100 , hcoverage 100 , similarity 100
                     , identity 100 , evalue 6.45e-28 , alnlength 50 ,
                     Bacteria , Proteobacteria , WP_007548649.1 transposase
                     [Wolbachia endosymbiont of Drosophila ananassae]"
     gene            complement(31044..31175)
                     /locus_tag="wEsol_00724"
     CDS             complement(31044..31175)
                     /locus_tag="wEsol_00724"
                     /codon_start=1
                     /transl_table=11
                     /fullproduct="qcoverage 100 , hcoverage 100 , similarity
                     100 , identity 100 , evalue 1.06e-21 , alnlength 43 ,
                     Bacteria , Proteobacteria , ONI56748.1 hypothetical
                     protein N500_0187 [Wolbachia pipientis wUni]"
                     /translation="MEKCLEHTHFQAKAPYDKERFDLKKELIRERDILKKALGKEKE"
                     /product="hypothetical protein"
                     /besthit="qcoverage 100 , hcoverage 100 , similarity 100
                     , identity 100 , evalue 1.06e-21 , alnlength 43 ,
                     Bacteria , Proteobacteria , ONI56748.1 hypothetical
                     protein N500_0187 [Wolbachia pipientis wUni]"
     gene            complement(31175..31309)
                     /locus_tag="wEsol_00725"
     CDS             complement(31175..31309)
                     /locus_tag="wEsol_00725"
                     /codon_start=1
                     /transl_table=11
                     /fullproduct="qcoverage 100 , hcoverage 100 , similarity
                     100 , identity 100 , evalue 5.41e-20 , alnlength 44 ,
                     Bacteria , Proteobacteria , WP_007548354.1 MULTISPECIES:
                     IS3 family transposase [Wolbachia]"
                     /cog="COG2963 COG2963 116 Transposase and inactivated
                     derivatives"
                     /pfam="HTH_Tnp_1"
                     /product="IS3 family
                     transposase"
                     /translation="MTNRREYTAEFKLEAVKLVKETGQASTKIAKDLGIDGSMLSKKI
                     "
                     /besthit="qcoverage 100 , hcoverage 100 , similarity 100
                     , identity 100 , evalue 5.41e-20 , alnlength 44 ,
                     Bacteria , Proteobacteria , WP_007548354.1 MULTISPECIES:
                     IS3 family transposase [Wolbachia]"
     gene            complement(31373..32857)
                     /locus_tag="wEsol_00726"
     CDS             complement(31373..32857)
                     /locus_tag="wEsol_00726"
                     /codon_start=1
                     /transl_table=11
                     /fullproduct="qcoverage 100 , hcoverage 99.3963782696177
                     , similarity 100 , identity 100 , evalue 0.0 , alnlength
                     494 , Bacteria , Proteobacteria , WP_065095043.1
                     MULTISPECIES: IMP dehydrogenase [unclassified Wolbachia]"
                     /pfam="IMPDH CBS CBS NanE B12-binding His_biosynth PcrB
                     FMN_dh Glu_synthase DeoC NMO Ribul_P_3_epim His_biosynth
                     Dus PcrB"
                     /tigr="TIGR01302 IMP_dehydrog 450
                     inosine-5'-monophosphate dehydrogenase"
                     /product="IMP dehydrogenase"
```

```
                     /translation="MEACYSFDDILLLPAYSNILPCDADTKTYLTNSIELNIPLISSA
                     MDTVTESGFAIAIAQHGGIGCIHKNLSIDEQVLEVRRVKKYESWIVYNPITISPDKTV
                     AEAISLMREHNYSGIPVVDQRKLVGILTNRDVRFIEDQNMNVKVSEVMTKDKLVTVRE
                     QGVNSASAMKLLHENRIEKLLVVDENSCCIGLITVKDIEKYNRYPNSCKDSKGRLRVA
                     AAIGTGKKDGIDRCEALIGEEVDVVVVDTAHGHSENVINTIREIKKMYPNTQLIGGNI
                     ATKEAAEALIDAGVDAVKVGIGPGSICTTRIVTGVGVPQFSAIKNVAEACRARNVRLI
                     ADGGVKYSGDVAKAIAAGADSVMIGSIFAGTDESPGEIIMYKGRAYKGYRGMGSISAM
                     KRGSASRYFQDKDSKLKLVPQGVEGRVPFKGPASGVIHQLIGGLQAAMGYTGNRNIEE
                     MKKNCKFVTITASGLRESHAHDIVITQEAPNYAYQTSNLSTDSE"
                     /besthit="qcoverage 100 , hcoverage 99.3963782696177 ,
                     similarity 100 , identity 100 , evalue 0.0 , alnlength
                     494 , Bacteria , Proteobacteria , WP_065095043.1
                     MULTISPECIES: IMP dehydrogenase [unclassified Wolbachia]"
     gene            complement(32983..33636)
                     /locus_tag="wEsol_00727"
     CDS             complement(32983..33636)
                     /locus_tag="wEsol_00727"
                     /codon_start=1
                     /transl_table=11
                     /fullproduct="qcoverage 100 , hcoverage 80.3703703703704
                     , similarity 100 , identity 99.5 , evalue 1.69e-148 ,
                     alnlength 217 , Bacteria , Proteobacteria ,
                     WP_015588849.1 MULTISPECIES: 16S rRNA
                     (adenine(1518)-N(6)/adenine(1519)-N(6))-
                     dimethyltransferase RsmA [unclassified Wolbachia]"
                     /cog="KsgA COG0030 259 Dimethyladenosine transferase
                     (rRNA methylation)"
                     /pfam="RrnaAD"
                     /tigr="TIGR00755 ksgA 256 dimethyladenosine transferase"
                     /product="16S rRNA
                     (adenine(1518)-N(6)/adenine(1519)-N(6))-
                     dimethyltransferase RsmA"
                     /translation="MHNPKSLLSIEKDRDLVKHHDQLLNEHQGKYKIIEADALHVIEE
                     ELIERPVKVIANLPYNISVVLFLKWLNNIKFFTNLTLMFQKEVADRITARPNSKDYGS
                     LSVLSQLLCDIKKEFDIEPKEFFPRPKIHSSVITVNPLPIPKFAVNLETLTRLTRAVF
                     SQRRKMLRNSLQNITNHAETVLENAKLSGNQRPENLTIEQFCLLANNVECLFCNKMY"
                     /besthit="qcoverage 100 , hcoverage 80.3703703703704 ,
                     similarity 100 , identity 99.5 , evalue 1.69e-148 ,
                     alnlength 217 , Bacteria , Proteobacteria ,
                     WP_015588849.1 MULTISPECIES: 16S rRNA
                     (adenine(1518)-N(6)/adenine(1519)-N(6))-
                     dimethyltransferase RsmA [unclassified Wolbachia]"
     gene            34000..34698
                     /locus_tag="wEsol_00728"
     CDS             34000..34698
                     /locus_tag="wEsol_00728"
                     /codon_start=1
                     /transl_table=11
                     /fullproduct="qcoverage 100 , hcoverage 95.8677685950413
                     , similarity 100 , identity 100 , evalue 9.59e-166 ,
                     alnlength 232 , Bacteria , Proteobacteria ,
                     WP_015588848.1 MULTISPECIES: triosephosphate isomerase
                     [Wolbachia]"
                     /cog="TpiA COG0149 251 Triosephosphate isomerase"
                     /pfam="TIM"
                     /tigr="TIGR00419 tim 228 triose-phosphate isomerase"
                     /product="triosephosphate
                     isomerase"
                     /translation="MNGMRSSFVDFIGKLNNKSNEITSKLVICPPFTSFPSSIELNNN
                     INIGAQNCHHKKFGSYTGEISAEMLKELGCTYVILGHSERANEKDSEIKLKSEIAIES
```

GLHPIICVGENSEDYKNEKTKEVIEYQCKNRLPTHGEYTVAYEPIWAIGTGHVPNNDA
IAKVIEVIKLCTSKKHIIYGGSVSSENIENLLSISNLSGVLIGSASLDFDHFYKIIQQ
VEKKFSLINSKISN"
/besthit="qcoverage 100 , hcoverage 95.8677685950413 ,
similarity 100 , identity 100 , evalue 9.59e-166 ,
alnlength 232 , Bacteria , Proteobacteria ,
WP_015588848.1 MULTISPECIES: triosephosphate isomerase
[Wolbachia]"
gene complement(34922..36010)
/locus_tag="wEsol_00729"
CDS complement(34922..36010)
/locus_tag="wEsol_00729"
/codon_start=1
/transl_table=11
/fullproduct="qcoverage 100 , hcoverage 100 , similarity
100 , identity 100 , evalue 6.36e-252 , alnlength 362 ,
Bacteria , Proteobacteria , WP_015588847.1 MULTISPECIES:
DNA-processing protein DprA [unclassified Wolbachia]"
/cog="Smf COG0758 350 Predicted Rossmann fold
nucleotide-binding protein involved in DNA uptake"
/pfam="DNA_processg_A Pfam-B_17296"
/product="DNA-processing
protein DprA"
/translation="MKINKLNNKELEVWLSLARTIGPTKFFSILRTHGSLDEVLKYLN
RVANNKVYGIQDAREEINNAERIGAKIIPACDPDYPDLLRNISSCPPVITALGDISLL
SREIIAIIGGRNSSMNGRNFANKLALDLSEAGFVIVSGLAKGIDTAANSVIYKNHPTI
AVTASGIDVVYPKENFDLYKKITGNGGLVITELPFATKPKPQYFPQRNRIISGLSLGV
AVIEASKRSGSLITADFALNQGREVFAVSGFPLDSRCSGSNYLIKNGAKLIESADDII
ESIRFSLPPQQKKLFDVEHYAVNQKQEKLQQAKSVIVDHINSVPVDIDELILASGLST
NIALMALLELELENRIERSPGNKISLIF"
/besthit="qcoverage 100 , hcoverage 100 , similarity 100
, identity 100 , evalue 6.36e-252 , alnlength 362 ,
Bacteria , Proteobacteria , WP_015588847.1 MULTISPECIES:
DNA-processing protein DprA [unclassified Wolbachia]"
gene 36072..36446
/locus_tag="wEsol_00730"
CDS 36072..36446
/locus_tag="wEsol_00730"
/codon_start=1
/transl_table=11
/pfam="Fer4_11 Fer4_6 Fer4_10 Fer4_2 Fer4_4 Fer4_7 Fer4_9
Fer4_10 Fer4 Fer4_6 Fer4_2 Fer4_15 Fer4_8 Fer4_4 Fer4_17
Fer4_16 DUF3470"
/fullproduct="qcoverage 100 , hcoverage 100 , similarity
100 , identity 100 , evalue 4.06e-87 , alnlength 124 ,
Bacteria , Proteobacteria , WP_141456813.1 MULTISPECIES:
ferredoxin family protein [unclassified Wolbachia]"
/translation="MTHFVTDKCIKCKYTDCVEVCPVDCFYEGKNMLVINPDECIDCG
VCIPECPVDAIVTDDSIKDILELDEELLNSEQKIFKSFYNINVEYSQKWPNITAKKQP
LYTAEEYKEKKDKTTYFEENLE"
/product="ferredoxin family
protein"
/besthit="qcoverage 100 , hcoverage 100 , similarity 100
, identity 100 , evalue 4.06e-87 , alnlength 124 ,
Bacteria , Proteobacteria , WP_141456813.1 MULTISPECIES:
ferredoxin family protein [unclassified Wolbachia]"
gene 36640..37092
/locus_tag="wEsol_00731"
CDS 36640..37092
/locus_tag="wEsol_00731"

```
                     /codon_start=1
                     /transl_table=11
                     /product="hypothetical protein"
                     /translation="MSINKRETIKKIEGLLFQANGNLEKTCDLLSTVKLTEDDIKILL
                     EEMIKEYYSLFKKISWSCGITAGGAQDANASSFNEAYKLGSKELALAIMSDFKKSYDQ
                     ELFKKYKKEEMSYPEGEGVQFIRELRVLSRILTISLIRKLDEVYSRRN"
                     /besthit="qcoverage 97.3333333333333 , hcoverage
                     55.9386973180077 , similarity 100 , identity 100 , evalue
                     3.56e-93 , alnlength 146 , Bacteria , Proteobacteria ,
                     WP_015588845.1 MULTISPECIES: hypothetical protein
                     [unclassified Wolbachia]"
     gene            37067..37417
                     /locus_tag="wEsol_00732"
     CDS             37067..37417
                     /locus_tag="wEsol_00732"
                     /codon_start=1
                     /transl_table=11
                     /product="hypothetical protein"
                     /translation="MKFTPEEIERLKEYILVSNYDTSKSLSDIDKYMHALSALIRNPS
                     LDTKKIFELNNRMIIHTTALIDLVTSGVVKKEEAKEQSAEDLEKLVCEQYKGAANTPV
                     PLPSTLFEVDKKSA"
                     /besthit="qcoverage 99.1379310344828 , hcoverage
                     44.0613026819923 , similarity 100 , identity 100 , evalue
                     2.25e-70 , alnlength 115 , Bacteria , Proteobacteria ,
                     WP_015588845.1 MULTISPECIES: hypothetical protein
                     [unclassified Wolbachia]"
     gene            37513..38460
                     /locus_tag="wEsol_00733"
     CDS             37513..38460
                     /locus_tag="wEsol_00733"
                     /codon_start=1
                     /transl_table=11
                     /fullproduct="qcoverage 100 , hcoverage 100 , similarity
                     100 , identity 100 , evalue 5.46e-227 , alnlength 315 ,
                     Bacteria , Proteobacteria , AGJ99620.1
                     D-alanine--D-alanine ligase [Wolbachia endosymbiont of
                     Drosophila simulans wHa]"
                     /cog="DdlA COG1181 317 D-alanine-D-alanine ligase and
                     related ATP-grasp enzymes"
                     /pfam="Dala_Dala_lig_N ATP-grasp_4 RimK CPSase_L_D2
                     Dala_Dala_lig_C"
                     /tigr="TIGR01205 D_ala_D_alaTIGR 318 D-alanine--D-alanine
                     ligase"
                     /product="D-alanine--D-alanine ligase"
                     /translation="MVPTIAILSGGFSCEREISLMSGKAVKKALDSLSYNAIEIDVDS
                     NIAEKLRKINSGLAFIALHGPYGEDGCIQGLLEILGIKYTHSGVMASAVAMNKVMSKH
                     IFRSLSIDTPKGYVISREDLLKNNIKIDYPYVLKPINEGSSIGVYIIFSHEDYLELKD
                     NSSTIMEKMIVEEYIPGIELHTAVLLDEAIGTIEVRPKNKFYDYEAKYTDGFAEHIFP
                     AEIPNNIYKMTLEHALKVHQFLGCKTISRSDFRYNPKNNTLKMLEINTHPGFTELSLV
                     PEIAKLAKGINFNELVKIIIEDSLQHKNIRDLSHVEQYY"
                     /besthit="qcoverage 100 , hcoverage 100 , similarity 100
                     , identity 100 , evalue 5.46e-227 , alnlength 315 ,
                     Bacteria , Proteobacteria , AGJ99620.1
                     D-alanine--D-alanine ligase [Wolbachia endosymbiont of
                     Drosophila simulans wHa]"
     gene            38441..39199
                     /locus_tag="wEsol_00734"
     CDS             38441..39199
                     /locus_tag="wEsol_00734"
                     /codon_start=1
```

```
                     /transl_table=11
                     /fullproduct="qcoverage 100 , hcoverage 100 , similarity
                     100 , identity 100 , evalue 3.42e-178 , alnlength 252 ,
                     Bacteria , Proteobacteria , WP_174516959.1 FtsQ-type
                     POTRA domain-containing protein [Wolbachia endosymbiont
                     of Cardiocondyla obscurior]"
                     /cog="FtsQ COG1589 269 Cell division septal protein"
                     /pfam="POTRA_1 FtsQ"
                     /product="FtsQ-type POTRA
                     domain-containing protein"
                     /translation="MLSSITRSQRSFLRKCALVIITALFLKLILYSSLDKIINRFNYY
                     FTWYNDCLSSLLLSSGFSIDEVVVSGNKFTNKKDILSLTDRTQPILYISLSKLAGNIQ
                     SVSRWIKHVRVHRILPNTLHINIDEHKPFALWKDNNKTSVIDFEGKVIVDDYLVDDLV
                     VIKGQNSLSNLEFVRDVLESKTQLRDHISSFAYIGNRRWNIILDNDSTVKLPEDNPYS
                     AWDYLNHLHNTTDFTFSDWSIIDMRITDKIFVKR"
                     /besthit="qcoverage 100 , hcoverage 100 , similarity 100
                     , identity 100 , evalue 3.42e-178 , alnlength 252 ,
                     Bacteria , Proteobacteria , WP_174516959.1 FtsQ-type
                     POTRA domain-containing protein [Wolbachia endosymbiont
                     of Cardiocondyla obscurior]"
     gene            39888..40994
                     /locus_tag="wEsol_00735"
     CDS             39888..40994
                     /locus_tag="wEsol_00735"
                     /codon_start=1
                     /transl_table=11
                     /fullproduct="qcoverage 100 , hcoverage 100 , similarity
                     100 , identity 100 , evalue 2.43e-259 , alnlength 368 ,
                     Bacteria , Proteobacteria , WP_010082605.1 MULTISPECIES:
                     D-alanyl-D-alanine carboxypeptidase [Wolbachia]"
                     /cog="DacC COG1686 389 D-alanyl-D-alanine
                     carboxypeptidase"
                     /pfam="Peptidase_S11 Peptidase_S13 Beta-lactamase2
                     PBP5_C"
                     /product="D-alanyl-D-alanine
                     carboxypeptidase"
                     /translation="MLSRLVILLLVFILPFSSYSYQFRTKAKQAVVLDLASDSFIFDH
                     NSDEKMAPSSMSKLMTLYIAFDYLKAGIIHMEDKFRVSRKAWERRGSSMFLKEGQSVT
                     VRELLEGITIVSGNDACITLAEGIAGSEENFVAEMNEVAQNLNLNDSHFVNSSGWPDE
                     DHFMNAKDLVMLAKRIFTDFPEYYDLFSEQYLTYNEIVQKNKNLLLFHDIGVDGLKTG
                     YTNAGGYGIVASAKRNDRRIFAVVNGLNTEKERIEEAKRLIQYSLNHFNTKKIFVKDS
                     VVEEVNVLYGKDRKVPITVANDVTITYNRKLHDQIKVRIEYKDMIPAPIKKGQEVGKV
                     FVEIPGIEQQTTPLYAANDVQELNFVEKFFRMLF"
                     /besthit="qcoverage 100 , hcoverage 100 , similarity 100
                     , identity 100 , evalue 2.43e-259 , alnlength 368 ,
                     Bacteria , Proteobacteria , WP_010082605.1 MULTISPECIES:
                     D-alanyl-D-alanine carboxypeptidase [Wolbachia]"
     gene            41094..41408
                     /locus_tag="wEsol_00736"
     CDS             41094..41408
                     /locus_tag="wEsol_00736"
                     /codon_start=1
                     /transl_table=11
                     /fullproduct="qcoverage 100 , hcoverage 100 , similarity
                     100 , identity 100 , evalue 8.97e-59 , alnlength 104 ,
                     Bacteria , Proteobacteria , WP_010082606.1 MULTISPECIES:
                     hypothetical protein [Wolbachia]"
                     /cog="EmrE COG2076 106 Membrane transporters of cations
                     and cationic drugs"
                     /pfam="Multi_Drug_Res"
```

```
                     /product="hypothetical protein"
                     /translation="MCWLYLLLSSILEILWAVTLKFSNSFTKVVPSIATLVIMIISIY
                     FLSLAACFLPIRVCYAVSSGICTIGITIIGATTFTENINLSQVLCIVLIVIGSIGLKL
                     SI"
                     /besthit="qcoverage 100 , hcoverage 100 , similarity 100
                     , identity 100 , evalue 8.97e-59 , alnlength 104 ,
                     Bacteria , Proteobacteria , WP_010082606.1 MULTISPECIES:
                     hypothetical protein [Wolbachia]"
     gene            41425..41769
                     /locus_tag="wEsol_00737"
     CDS             41425..41769
                     /locus_tag="wEsol_00737"
                     /codon_start=1
                     /transl_table=11
                     /fullproduct="qcoverage 100 , hcoverage 100 , similarity
                     100 , identity 100 , evalue 1.17e-70 , alnlength 114 ,
                     Bacteria , Proteobacteria , WP_006279756.1 MULTISPECIES:
                     multidrug efflux SMR transporter [Wolbachia]"
                     /cog="EmrE COG2076 106 Membrane transporters of cations
                     and cationic drugs"
                     /pfam="Multi_Drug_Res"
                     /product="multidrug efflux
                     SMR transporter"
                     /translation="MNWIYLLLSSLIEVFWVITLKHSYGFTHLVPSIISILSMALSTY
                     LLSLATRSIPIGVCYAIWTGVGAIGASILGVYLFDEPVNLFEVICFILVTFGIIGLKL
                     FDTAKQTVDKME"
                     /besthit="qcoverage 100 , hcoverage 100 , similarity 100
                     , identity 100 , evalue 1.17e-70 , alnlength 114 ,
                     Bacteria , Proteobacteria , WP_006279756.1 MULTISPECIES:
                     multidrug efflux SMR transporter [Wolbachia]"
     gene            42313..42597
                     /locus_tag="wEsol_00738"
     CDS             42313..42597
                     /locus_tag="wEsol_00738"
                     /codon_start=1
                     /transl_table=11
                     /fullproduct="qcoverage 100 , hcoverage 100 , similarity
                     100 , identity 100 , evalue 1.51e-59 , alnlength 94 ,
                     Bacteria , Proteobacteria , AAS13858.1 hypothetical
                     protein WD_0102 [Wolbachia endosymbiont of Drosophila
                     melanogaster]"
                     /translation="MNLSWRGYRLIAADGSGMRLPSSGEIVSEFEPNGTTGTIGNLFV
                     DLCTSLICSARLAAWNIGEQTLAAEQLPEVITQMRLLNQEKLLFIYDRLQ"
                     /product="hypothetical protein"
                     /besthit="qcoverage 100 , hcoverage 100 , similarity 100
                     , identity 100 , evalue 1.51e-59 , alnlength 94 ,
                     Bacteria , Proteobacteria , AAS13858.1 hypothetical
                     protein WD_0102 [Wolbachia endosymbiont of Drosophila
                     melanogaster]"
     gene            33873..33945
                     /locus_tag="wEsol_tRNA-14"
     tRNA            33873..33945
                     /locus_tag="wEsol_tRNA-14"
                     /gene="tRNA-Thr"
                     /intronpos="0..0"
                     /program="tRNAscan-SE"
                     /cove_score="73.6"
                     /evidence=predicted
                     /type="Thr"
                     /anticodon=TGT
```

```
                     /product="tRNA-Thr (TGT)"
     gene            complement(4729..7504)
                     /locus_tag="wEsol_rRNA-1"
     rRNA            complement(4729..7504)
                     /locus_tag="wEsol_rRNA-1"
                     /product="23S ribosomal RNA"
     gene            complement(4566..4670)
                     /locus_tag="wEsol_rRNA-2"
     rRNA            complement(4566..4670)
                     /locus_tag="wEsol_rRNA-2"
                     /product="5S ribosomal RNA"
ORIGIN
        1 acttcttgtg cattaccatc ttctacagct aggaataatt gttcttcctg tgtttttacg
       61 cgtgtttcca cattagatat aaaaaataac aatactaaac atgcaatacc aataatgcta
      121 atatatttta taacttttct cataagatat tttacaatgc ttctaataat attagatatc
      181 atctataaaa ttaaaaaatc catgaaaggg gatgaaaata tgtaaaaaag tattattgat
      241 taattaaaaa cagattttga tttgataata ctaataaaat cgatttatct taaatttacg
      301 ttgattttta cctgttattt tttgtagttt atccaacgta aattccaggt aaaacaaagt
      361 cgttatctcc aaccgttaca accaactctc cttctttaat ttttatttga ttaggtgtat
      421 ggtcccatac catcacactc tgggatcata cacctaaagt actccgacac tgggatgaca
      481 ccccggggtt tctgggatga caaaaaagag ccccctgagc aaaaatctta tagccacttg
      541 aatgataatt gactttttac ctaaaataac taagaaagaa acttctcatc aagataactt
      601 aaatatatac taattctctt atttttaaac gatttctcat catgaattgc acgatcaaaa
      661 acctgaattt gacttgttat aatgctacaa ttattatttg caacttctaa tacgccacta
      721 tcaattacaa ccttcttttc tatttggtta ctcattttaa cagtaatcat accaggcaat
      781 aaataaatta agtaaggagc atggtgagct aaaatcataa gctccccttc gagcccagtt
      841 actgaaagag aaaccactcc actgaatgaa atttgatcat caggagagaa aaattgcact
      901 ttaaaagtat tcataatctt taacttttat ttagcttcag cttgtattaa ttcagccttt
      961 tttattgctt catctatatt ccccaccata taaaaagcag cctctggtaa gtgatcatat
     1021 ttgccttcaa caatcccttt aaaactggaa acagtatcag aaagtgaaac aaatttacca
     1081 ggcatgccag taaatatttc tgcaacgtga aaaggttgag aaagaaattt ctgaatctta
     1141 cgagccctat caacaataat tttatcttca tcagatagct catccatacc aagtattgcg
     1201 ataatatctt gcagtgattt ataagtttgc aatatacgtt tcacctcaga agctacatta
     1261 taatgttctt caccaatgat ttcagcagat aaagactgag aagttgaatc aagtggatca
     1321 acagcagggt atattcccat ttcagctatt tgccttgaca acactgtggt tgcatcaagg
     1381 tgagagaatg tagttgctgg ggctggatca gttaaatcgt ccgcaggaac atatatagct
     1441 tgcacagaag taatagagcc agaagttgtt gaagctattc tttcttgcat tgcacccata
     1501 tcagttgcaa gggttggctg ataaccaaca gctgacggta ttcttccaag taaagcagaa
     1561 atttcagaac cagcttgtgt aaatctaaag atattatcca caaaaaatag aacatcttgg
     1621 ttttcacggt cacgaaaata ctctgccata gtaagtgctg ttaaagcaat tctagccctt
     1681 gctccaggag gctcattcat ctgaccataa accaaaacag cttgagattt ttcatgctca
     1741 tttatattta ttacatttga agtgatcatc tcgtgataaa gatcgttacc ttcacgcgtt
     1801 ctctccccta ccccggcaaa cacagaaaat cctttatgag cttttgctat attattaatt
     1861 aattccatta ttaggactgt tttaccaaca ccggctccac caaataagcc aatttttcct
     1921 cctttaagat aaggtgcaag aagatctata acttttattc ccgtaactaa aacttcttcc
     1981 tgtattctct gttcagtaaa acttggaggt gctctgtgta taggctctaa gttatacttt
     2041 cccttcagtg gaccacactc atctataagc tctccaacaa cattaaaaat ccttcctaaa
     2101 gttgaacgcc caattggcac cgatattggt gcacctgtat caacaaattc atcccccctt
     2161 gacatgccat ctgtgctatc catagcaata caacgaacta tattgtcacc tatatgctgc
     2221 gaaacttcta aaaccagctc cttatcctta tattttagtt tgctttttaa agcattaaat
     2281 attttaggca attcaccttc aaattttata tcaacaactg cttgagttac cttaatcgct
     2341 ctacctatat tcatctcttt atttttatca cctaacacta taattccata aaagaacttc
     2401 taaagaaaat aaattttata ttattaatag aaattttgca acattaaaac gcttactagt
     2461 tacgcttcat tacaaacaat gcaaatgcat ttaatagcca agtaataata aacaatacta
     2521 aactaagcgc ataagcagca agagtttgta cactattgaa atcctgatct ccggtaagta
     2581 atgtagcgat ctgcacggta atggtagtaa ctgaattaag agggttaaaa gtcaaatttg
     2641 cgttgattcc cacagccatt agcacaatca ttgtttcacc tatcactctt gaaattgaca
     2701 ataaaattgc acttaaaatt gtaggcattg cataaggtat tgttatatgc catatagttt
     2761 ccgctggagt tgcaccaagt gccatgaagc cataacgcaa gctttttgga acagatctta
     2821 tggcatcttc gagtaaagaa ataataaaag gaagaatcat tatcccaatc gataaaccgg
```

2881 caactaaggc actttctgag tgtatactta aaccaaaaaa atttgctacc tgctttataa
2941 agaaagacaa aaacacaacc gcgaaatatc catatacaac cgtaggaata gcagacaaaa
3001 cttgcaaagt tgtattaaca atataacgca ctttctcact cgcatattca ctaatatata
3061 ttgcagaaaa taaaccaagc ggaacgacaa ctaacattgc tataatagtt ataagtaatg
3121 taccaactaa aagcggcgct ataccaaaac atcctatctt ctcttcattg atagtgacta
3181 cattgtggcc ccatttcaag caaaacaaga attctgaaat agctacttta ttaaaaaaac
3241 taatggattg aataaaaata gacagcatta taaataaagt gataaaaaat gatatggcca
3301 aagccacaaa taacgaaagt tttattatct tatttctttt atttttaaag ataaaagcaa
3361 gcatgaaaaa aagtaacgct atagaaataa aagttacaaa acaattatta tagaatacta
3421 acaaccaagt tagcatccat acacaactaa gatatagata agatttaacc ttatttactt
3481 cttgaaacct actaaaacaa aataaaagga ttagtaagac aggtatgact atcaccgaat
3541 tcattcttcc atgcatcaaa ttttattcta cgaaaatgtc atacaagttc accattattt
3601 gacaataaag taaaacaatt taaaataaat aattatatta atataaaaac aatgaagaga
3661 acatttcaac caaaaaattt gataagaaag cgcagacacg gatttcgttc acgtatggca
3721 acaagagctg gaagaaaaat ccttaatagg cgccgttcat tagggtgtaa caaattatgc
3781 gcatagtaag tataaaaaaa aagatttttc ttctgctttc aaaaataagt tagcactcaa
3841 cagtcttttt tatcgtgggc tttatatatc actgtacgct ataaaagaaa gagaacctga
3901 aaagtacatt catactatta gagtaggtct ggctatcagt aaaaaaaccg gaaaagcagc
3961 aaaaagaaat aaaataaaaa ggcaactacg aatgcttgct aaaattagca ttttaaatat
4021 aagtaatata gggcactact acataatact aactcataga aacattatgc aagcaagtta
4081 taaaaattta caaaaagatc taactatttg cttaaaaaag ataaaataaa aaaaaatctc
4141 aattaaggta tttacaacta tattttttat gataatatag gtaaaatttt taaaaatagt
4201 taatgggtgt gatgaatggt agagcatata aaagaggtaa ataacacagg ctttggtcag
4261 atgtatcaat tcttatttct tcctaaagga acaggaacag attggaggtc agcatttcgc
4321 caacaagaag aattatttaa aaaagtggga ggtaatgtaa tattaccaga aatcagtaat
4381 tctccttcaa aagggaatca attaggggaa gtaggaacaa tgctaaataa tgtagaaaat
4441 gaccaagaac atataagaaa aaaaagatat catagttaaa gtaaaaactt aaagaatagt
4501 aacaaaacaa gaaagttaaa atataagtat acctactgtc taattaacaa agaaattttt
4561 ataaacttgg cgacgaccta ctctcccttt tcaagtacca tcagcgctaa aaagtttcac
4621 ttccgagttc gagatgagat cgggtggttc atttttgcta tagccaccaa gtcaataaaa
4681 atttctaact taacaatttt aacttaatat ttaacactgc atacacacat atagaaagca
4741 aataaatcaa tcaggctatt agtactagtt agcttcacat gttaccatgc ttccacacct
4801 agcctatcaa cgtggtagtc ttccacggcc ttaattggga aatctttttg aagagggttt
4861 cttgcttata tgctttcagc aattatccca tctatacata gccacccagc gatgctattg
4921 gcataacagc tggtacacca gaggtatatc catctcggtc ctctcgtact agagtcagac
4981 cttctcaaat ttccttcacc cacggcagat agaaaccgaa ctgtctcacg acgttctaaa
5041 cccaactcac gtatcacttt aatcggcgaa cagccgaacc cttgggacct tcttcagccc
5101 caggatgtga tgagtcgaca tcgaggtgcc aaacggtgtc gtcgctatga acgctcgaac
5161 accatcagcc tgttatcccc ggcgtacctt ttatccgttg agcgatgacc cttccataca
5221 gaatcaccgg atcactatga ccgactttcg tctctgcttg gcttgtcagc ctcgcagtca
5281 ggcaagctta tgccattata ctatcaagct gatttccgac cagctctagc ttaccttcgc
5341 acgcctccgt tactttttag gaggcgaccg ccccagtcaa actacccacc atacaatgtc
5401 ctagttccag ataatgaaac atagttagat atcaaaagtg taaagggtgg tatctcaagg
5461 ttgactccat tatagctagc gccataactt caaagtctcc cacctatcct gcacatcaca
5521 cttttaatag caatgtaaag ctatagtaaa ggtgcacggg gtctcttcgt ctaaccgcgg
5581 gtaccccgca tctgcacggg gaattcaatt tcgctgaagt gatgttggag acagtggaga
5641 aatcgttacg ccattcgtgc gggtcggaac ttacccgaca aggaatttcg ctaccttagg
5701 accgtcagtg ttacggccgc cgtttactgg ggcttcaatt cggagcttgc acccttccta
5761 ttaaccttcc agcaccgggc aggcgtcaga ccctatactt ccacttacgt gtttgcagag
5821 tcctgtgttt ttagtaaaca gtcgctactc cctattttgt gccacctact catagttgcc
5881 taaaagcagg ttacccttct tccgaagtta caggtataat ttgccgagtt ccttcaacat
5941 cattctttca acaccttagt atactctact catccaccag tgtcggtttg cggtacggcc
6001 tcataaatat aagtgctatt tcctggagct tcttttaagc atagatcaat ccaataagat
6061 ctatacaaat acgaaacccg tcacacttaa gaggtttagg aatattaacc taattgccat
6121 cgactactcc tttacggact cgccttagga accgactaac cctacgcaga ttaacttaac
6181 gtaggaaccc ttagattttt ggtgagagtg tttttcacac tcttttacgc tacttatgtc
6241 agcattctca cttccgatat ctccagcagt tttcacaaac caccttcaca gacttacgga
6301 acgctccgct accgcgccta ttgatcgaaa tcaataagca ctcacatctt cggtatacag
6361 ctttagcccc ggtacatttt cggcgcagaa aaacttattt agacaagtga gctgttacgc
6421 tttctttaaa ggatggctgc ttccaagcca acctcctagc tgtaatggtt tttctacttc

6481 cttccccact tagctgtaat tttgggacct tagatagtga tctgggttgt ttccctcttc
6541 accacggact tagcacccgt agtgtgcctg ctatataatt aattgttggt attcggagtt
6601 tagttagatt tggtaagacg gtgaatcccc ctagtctatc cagtgctcta cccccaacaa
6661 catacataca acgctctacc taaatagatt tcgcggagaa ccagctattt ccaagtttga
6721 ttggcctttc accсctaatc acaactcatc caataatgtt gcaacattaa ctggttcgat
6781 cctccagtgt gttttaccac accttcaatc tggtcatgac tagatcactt ggtttcgggt
6841 ctaatccata aaactaaaac gccctattca gacttgcttt cgctacgcct acacctaacg
6901 gcttaagctt gcttcataga ttaactcgct gacccattat gcaaaaggta cgctgtcact
6961 ctaaatatca ataaattgat atagagctcc aactgtttgt aagcacttga tttcagattc
7021 tatttcactc ccctcccggg gttcttttca cctttccctc acggtacttg ttcactatcg
7081 gtcgttaagg agtatttagg cttggaggat ggtcccccca tattcaaaca ggatttcacg
7141 tgccccgctc tactcaagga tttaaaaact ttctacttat acaggactat caccttctat
7201 ggttactatt tccagagtat tctaattctt attcttaaat cactagcctt ttccgctttc
7261 gctcgtcact actaacagaa tctcggttga tttcttttcc tttggctact tagatatttc
7321 agttcaccaa gtttgcttta catacttagt acgtaataac tagctaaact agttgggttt
7381 ccccattcgg aaatctgcgg atcaaaattt gttgacaatt ccccacagct tatcgcagcc
7441 tgccacgtcc ttcatcgcct cttaacgcca aggcatccat caagtgctct taataattta
7501 tttatccgac tatatgcaga atatgcagag ttaaatatta ttaaaattga attgagatta
7561 aattaatctc taccataaaa cttgtcaaac atctaaaaac taaattaatc ttttatatgt
7621 tataacctaa taaataatat gtcaacgcct ttttgcacaa aacatttact tatattatca
7681 acaacaatat actagcaatc ctaaacagta taatctgaga aatatgtggt aaacttatat
7741 atacagtaaa aattaataaa aaagatattt tgtaagataa aatgtgttaa aatcaattgc
7801 ctaaaaatga taataaacat tcggagtatt tctaacgtat gaaaagtaat ctctctttta
7861 taaaaaacgt tgctttaaat tgtatgtcta agctcattgg cgtattaaat attaagaaag
7921 taaaacactt cattgtgaat aaatataata ctcttcataa agaaatgaca gtgcttctta
7981 aaaaatctaa gaacctgtta aacaccaaca ttgaaatcgg tttatatcac ttttataaag
8041 gtagcatatc agatgcaaag ttacgatttt ggttaattag catattttac ccccatttac
8101 ctatagtctg gtataacatc ggaaggtgtc atttgcggt agggaatact aacaaagctt
8161 ataattattt aacaagggca ctaaaattag ataatgacca cgaggaggcg tcttactata
8221 taaaaaaaat gacaaactca gcgcctatta cagaattgcc aaaaaacctc ataagacaat
8281 attttgatta cacaggtgaa tattttgttg agcattggtt gattgccaaa caatatagag
8341 ggcatgaact tgtacacatg ataattacaa aaatcttcaa caactccact tctgagctca
8401 atatactcga ccttggttgt ggaactggaa tatgtggtca tttcttgaaa ataaatagta
8461 ttggaaacca tataatagga attgacattt caagtagaat gctaaatatc gcaagaggat
8521 gctttataaa aggtaagcct gtttacaatg aattaataca tatggaaatg aaagagtttc
8581 ttaaacaaga gaaaaaccag cagtatgatg taattatctt tgctgaagtg ctacattatc
8641 tacatgactt tcaagaagaa ttggaattag caaaaagatc tacgagcaaa aaagggggtta
8701 ttgtatgttt aataagaaga aaggaaggtg aaggtattga ctttgtaaat aaaggagact
8761 attttcgtca ctcagaaagt tatgttcaac atgttgcaaa agaaataaat atgcaaataa
8821 gttatatgag ttactgtaaa atatatggca gtcaggttga cggtatcttg tttgcattac
8881 agcatcaaca agaaaatccg aaaaacctaa cttaaaaatc gctcaaaatg acttttaagt
8941 atctatatat aataaaagaa taaaaggaac tttatgaata acattaccat taattacgca
9001 aaggacagca aattaaccga ctttggaaaa gcagttctat cagacaggta tttaatagaa
9061 aatgaaagtt atcaagacct ctttgcacgt attgctaatt actattctga taataaagaa
9121 catgcgcagc gtctttatga ctacatgagc aacttgtggt tcatgccttc aacaccaata
9181 ctcagcaatg gtggcactaa gagagggtta cctatctctt gctttcttaa tgaaaccgaa
9241 gacagcttgc agggaatagt tgatctatgg aatgaaaatg tttggcttgc tgcacgtgga
9301 ggaggcatag gtagttattg gggaaattta cgctcaatag gcgaaagcgt aaagggtagc
9361 ggcaaaacat caggaattgt tccatttatt gtagtgcaaa atgctctaac gcttgcaatt
9421 agtcagggat ccctaaggcg aggaagttca gcagtctacc ttcctgtatc tcatccggag
9481 atagaagagt ttctagattt acgcaagcca acaggcggtg acccaaatcg caaagcgtta
9541 aatatacatc atgctgtaat agtaacagat aaatttatgc aggctgttga gaatgatcaa
9601 gaatggaatt taataagccc tcacaacaat aaggttattt caactgtaaa agcgcgggat
9661 atatggatca aaatattaac agcaagagtt gaaactggag aaccttacat tattttcctt
9721 gatgcaacaa ataacaataa gccagaatct tacaaaaagc tcaacttaga catcaagatg
9781 tcaaatctat gcagtgaaat aactttaacc acaggttatg atcacctgaa taagtcacgc
9841 actgctgtgt gttgtctatc atccgtaaac cttgagtact acgaagaatg gaaagacaat
9901 aaactcttca tagaagatat aatgcgcttt cttgataatg tattggaaga tttcataaat
9961 aaagcaccga atgaaatgca gcgagcaaaa tattctgcag ccagagaacg cagtattggt
10021 cttggtgtga tgggctttca ctcattttta caaagcaaaa tggttccttt tgaatcagta

10081 acagcacaac aatggaataa aaaaatattt aagtatttac gcgagcaagc agatatagtt
10141 tctaaaaaat tagcagaaga aaagggagca tgttctgatg ccaaagaagt tgatctaatg
10201 gaaaggttta cacacaaact cgctattgct cctactgctt cgatctccat tattgcaggt
10261 aatacctctc ctggaataga accatatgca gcaaacgtat tcatacaaaa gacacttaca
10321 ggctcatttg tagtgcgaaa taaattcttg caaaaactat tagcagaaaa aaatcaagac
10381 aatgataaaa tatggtcttc aatttcaaca aacgagggtt ccgttcagca tttagatttt
10441 cttagtgagc atgaaaaatt gacatttaaa acagcgtacg agcttgacca aagatggatt
10501 atagaacatg caagtgatag aacttcgtat atttgtcaat ctcaatcagt aaatctgttc
10561 ttacctgcca acgtacataa acgttacttg cacaaaatac acatgcttgc ttggaaaaaa
10621 ggattgaaga gcttatatta ctgcagatca caatcaatgc agagagccga taaggtctcg
10681 catgacatat tcaaaaaaag tgaaatattg caacaaaaaa cagatattga ctacgatgaa
10741 tgtctgtcat gccaatagcg gccttactca aaaaagtaga gagaaagtta agcaaatggg
10801 taaatagact tcttgcataa ccatataacg cacttttatt gggaatagaa acgaaaaaac
10861 tttcttgaca aactccgcca gccctcttat cataaaattg aagctattta tttatcttct
10921 ctgtacagat taaatgacaa aaaaactcac gtatctggcg tctcatgttt aattttttgc
10981 actatgtgca ccttatgtct ttatcaaact tctacctaca taagctgaaa cgcgcttata
11041 aagcgtttaa aacataaaaa aacgccaact taaaaaatgg atagtgaata actagctacc
11101 ctagggtttc ttttgccttt ttttctgttt agtaaatttc ttaaacattt atagctaagg
11161 ttagttgcat ttaaaagcag ctgagtcgcg gttattaagc gtttagaata aaaaaacgcc
11221 atacttgaaa gtataatgta agtaattagc caactacggg gcttcttttg cctttttttt
11281 atttggtaaa tttcttaaac atttatggct aaacgacaat cgtcatcccg ctacttgtta
11341 gcgggatcta ttgccgagat accgcgaatg aatcgcggta tgacggttcg cggtggcatg
11401 acgataaagt ttatacctcg tcatcccgct gcttgttagc gggatctctt gttagcggct
11461 gagataccgc gaatgaatcg cggtatgacg gttcgtggca gcatgaagat aatcccgcta
11521 cttgttagcg gctgagatac tgcggcggta tgacgtagat tgctgtcatt ccgctactag
11581 ttagcgccgc agcggtatgg tgtaggaaaa ttagctatat agctaacttg taccacgcgt
11641 taaagctttt aaacaggtct tggtttactt actccattat tttctttatt atcagatatc
11701 atttaaaata atatggagga gcaaattgtc agcaattgcc attattgatt ttggttcaca
11761 gtttacacag cttatcgcaa gacaagtaag aggaatgggc gtttattgtg agatatttcc
11821 aagcaacatc agttttgaaa caatatcaaa attcaatggg tttattctct ctggaggacc
11881 acaatctgta aatgatgatt gttctgaaac aagtagagta gtacatgaaa ttataaaact
11941 taatgaggca acaagcgttc ctatacttgg aatatgctat ggacagcaac tcatttgtca
12001 ttattttgga gcaaaagtaa aagagagttt caaacaggag tttggtagaa ctaaaatcaa
12061 gatactaaaa gaatccccca ttgtaaagga tacctgggat gttaattctg aagtggatgt
12121 attaatgaac catgcggaca gtgttgatac tataccacag ggatttactg ttatcgcatc
12181 aggtgtgata aatcaaacaa ttgcaatgat tgttaacgaa cagcgaaaga tttactgtac
12241 tcaattccat cctgaagtga ggcccacaac aaatggcagt aaattgctct ctaacttctt
12301 ggatattgca aattgcgaaa gagactggac aatgaagtcg tttattgagg agcaaaagga
12361 aaaaatcaaa aatgtagtag gagagaaaaa agtaatcgct gcagtaagtg gtggggttga
12421 ttcaagtgtt gcagcggctc tcacatataa agctatagga aaacaattaa actgtatttt
12481 tatcgatact gggttgttac gcaagaacca gaccattgct atgttgaaag agattccgat
12541 aaactacgtt gataaatcaa atttattttt gagtagattg aagggaataa ctgatccaga
12601 agaaaaacga aaaattatcg gtaacacttt tattgaagtg tttgaagaag aagcaaaaaa
12661 aataggtgat gtggattttt tgatgcaagg taccatctac tctgatgtag ttgagtcagg
12721 gcatgcctca gacaacacta gtacaattaa gtctcaccat aatgttggtg ggttgccaga
12781 aaagatgaat ctgaagttag tggagccttt acgttacctc tttaaagatg aggtaaggct
12841 acttggaaag gaaattgggc tttcagacga gataatattt caacatccat ttcctggacc
12901 cggacttgca gtgaggatta taagtgaagt cgataaagaa aaggtgcaaa tattgcaaga
12961 agtagatgaa atatatatta atacgatgaa aaattacgat ctatatgata agatatggca
13021 agcttttgct gtattattac caataaaaac tgtaggcgtt atgggagatg gtcgtacgta
13081 tggatatgtt tgtgctttaa gggctgtaac atcatctgat ggtatgacag ctgatgcatt
13141 tccatttgaa aataaggatc aacacttgct agtattttgg gattttttac agaatgttgg
13201 cagtataatt gttaataatg ttcccggagt aaatagagtt gtttatgata tcacttctaa
13261 accacccgca actattgagt gggagtaaat caaaaagatc ccgctgcttg ttagctggat
13321 ctagagatac cgcgaatgaa tcgcggtatg acggttaatt ttctttattt tgcttataat
13381 ttccacaagc agtgcaaaca taaatgaaga atacaaatat ccttttggta attctatatg
13441 aaatccatga agtatgaggt acacaccgac aagtaaaata aataaaatgg cgattatttt
13501 taaccctggg ttagatttga ttaactgggc ggtataactt gataaaaaca gcattgctag
13561 catggaaaat gtaaatgctg tagcaattat taccatgtta taagttagtg ctatagcagt
13621 taatatcgaa tcaaccgaaa aaactaaatc tattaatata atttgtagca caactaaaaa

13681 aaattttgat ttaacgtttg ctttcttttt atttttctta catacaaaga tgtcatcccg
13741 taactccata gagctcttaa caataaggaa taatcccccct gcgatcataa gtaaatccct
13801 tgctgatgca gtgtgaaata taggtttttg cattgacaat atagatgatg taaaaaatag
13861 tattacaaaa cgcattaata gtgctaatcc aaggcccata aggcgcactc tttctctcag
13921 cgcatttggt accttatcta ttgctagaga aataaagatt aaattgtcta cactaagtat
13981 agtttcaagt agtgtaagtg tcagtaaagt ccaagcatca gttagcattt ttgtttcaaa
14041 ttcggttctt cttactctac aatagatgtg taaaaattaa gtgacttcac tcagaagttt
14101 tatagcagtt ctccagaaaa acaggatata taaagttcag cagcttcttc tattttcgtt
14161 tttgagcaaa atcaataata agagagtttc ttattttcat gcacgcaaat aagataaagc
14221 cttattttca tttttgactt tatttgaaaa taatatatac tataaaaata gtgtgctagg
14281 ttgaatgaat gtatattaaa ggatcaccat caggcaaagt attcgctggt tatcaggcat
14341 ttataccaag ccccttgcca ccaaaatttg aatgggacaa tgatttagtt aatagtcttt
14401 aaacccttac tttactataa ggaaagtggt tgaaaactta ggtgtcgtat ttacaacagc
14461 ccaaagagca atcgtaaagc ttgaagattt aggcatcgtt tcacaaactt ctcaaggaaa
14521 gagagatcgg gtttattgtg caactgatat tttgaatatt ctagaagaac caactaagat
14581 tacagagaac ttcgatagca cattgtagct attgtgatga ataaattcag caaacagcaa
14641 gcttaaagag tctgatccaa tggacatgct gaacaaaaag taaggagctc ctttggaggg
14701 attatattaa cttattaatt aattgcttga cttttagcag gatttattat attatttatt
14761 aatgagcaat gcaatcaaac caaggaggat agcatgataa ataatttgga ttcaataatt
14821 cttggaataa aagatggtga aaaggcgatt gaagatttaa gggttgttct taaaaaaaga
14881 aaagaagaag ttattactac acagacattt aattacgctt tacgatgccc aaaaaactaca
14941 aaggaagcga tagtacacga aattctgctt cattttatac aaaatcctgg cgagcaatca
15001 ctagaacaag tgatacaatg cttagataga gcaatacaat ctgggatata tgcaaaaaac
15061 aacccaataa gaaatctaga tgaagaattt cgtactcata taaactttaa agatgaagag
15121 ggaaatacat tactacatca tgcagttata ggtaataaaa ctgaagaaat aatcacccctt
15181 cttgttacat atagtgcaaa tcctttgata caaaacgcgg ataataaaat tcctttagat
15241 ttagctcaag gagaaacaaa agaagtgctt attaaaagta tgaaaaaaca agctaatacg
15301 aaaaaagaaa gcgctatggt aggcagtttg gtgcctggcg taataattgg tggttttctt
15361 ggtgttgtac ttggagctgg tgtgtgtgtt gcagtaagtc tttctggtgg tatgatacta
15421 ggtgtaatga tagcatcttc tcttgttgct agtatagcta ttggactggc catgtatttt
15481 ttgagtcaag actatgaaca ggctaaagcg atagaaaaaa ctattagcac tgttagctct
15541 gaaatatctg ttgatgatac aactgccgct aataaaaata ataaagaagg gccacaactt
15601 acatatagct gacccaagca acacgtcata ccgcgattca ttcgcggtat agattccgct
15661 aactagtagc ggaatgacga atgtgtcgtt tttcaaattg tcgtaagtca ctttagctat
15721 aatacacaca aggagggtgt catcccagcg cccctatgat gtcatcccag tgcttgacac
15781 tgggatccag aaaagtttgc ttgtaaacta gcatggaaag tagctgatct tacactaaat
15841 gaatgttttt gatgaggttg catagaagct ggattccagc gtcacgcgct ggaatgacaa
15901 ctgcgttttt tgtttttttg tcatcaaata ctcctactct ttatgtcatc caagtgtcct
15961 gactacttgg atccaagaaa tttaattgtt ttaaattttt ccctctatac acctatttaa
16021 aatttatgtt atccacatgt gcaatataat ctataataac gtttatcaat acaatatatt
16081 aaaaaatatt atgagcttcg taaaagaaaa aaacacccct gtgatggagc agtatttgaa
16141 cctgaaagct caatacaaag atcatctgct attttatagg ctaggagatt tttatgaatt
16201 gtttttcgat gatgctatta aagctgcgaa attgctgaat atagtgctca ctaagagagg
16261 taattcgtgt gggcaagaaa taccaatgtg tggagtgcca gcacacagta gtgaatctta
16321 cctacacaag ctaatagatt taggattcaa agtagcaatc tgtgaccaat tagaaactgc
16381 tgatgaagca aaaaagaggg gctataaatc catagtaaaa cgtgatgtag tgcgagttgt
16441 aactccaggt acaattatag aagattcact actggaggat aaaagcaata attatctcgc
16501 gtccatagtt gaacaaaatg acgaatatgc tattagttgg cttgaattat cgacgggaaa
16561 attttttcac actttaacga gtttgaaagc tctagatagt gatttattgc gtatatcacc
16621 aagagaatta ttaatttctg aaaaattcac tgaggatgaa aaaattagat cgattttaaa
16681 aaattataaa atatcaatta cgcaacacgc acagagtttt tttgagtata gtaaatctca
16741 cagaacgtta tgcgagtttt ataaaatcag ggaacttggg agtataggaa atttcagcaa
16801 agtagaaatc atggcgtgtg gtgcactgct tgaatatgtc agagtaacgc aaaggggctc
16861 cattccaagg cttgaattcc caaaaaccta taagcaacaa aatttcatgc ttattgatgc
16921 ttcagcaagg agaaatcttg agttgttttc aactcaattt ggtgaaaaga aaggttcact
16981 aatttcagtt attgatcata cagttacagc ttctggtgga cgcctgctca aacaaatgct
17041 cgcttcaccc cttgcttgct ccaaggcaat caatttgagg ctcagcaccg ctcaattttt
17101 tgtaaataat catgagccac gcagaaaaat acgagagata ttatctaaca ttccagatat
17161 tgagaggtct ttatcgcgct taatactagg gcgtggttca ccaaaggata tgaacctatt
17221 gaaaataggc ctaggaaaga ctttagagtt gtctgagttt ctgtctacct tgcataatta

17281 ttgtttaagt gaaaaatcag aaattaactc tcagatgtca ttccagtgct tgaccggaga
17341 aaaggaacca gtgtctacta ctcaggtgat aagtagtgac gagagtgaac tcagcacaat
17401 acataagagc cttggtaatc ataaagacct atttgaactg ctaaatagtg ctatacttga
17461 taataatctc agttccgtga aagagggagg gtttatctac ccaaaataca actcagaatt
17521 atctgagtta tcttatatat tgaacaacag taacaagttg gtaactaagc tccgtgaatc
17581 ttatcgcgac ctaactggca ttgccgcact caaaatattg cacaacaata tactcggtta
17641 ttacgttgaa gtatcagcaa atcacaaaat aacttcggat atatttattc ataggcaaag
17701 cttagcaaat agcatgcgct acactactaa tgaattgaaa gaactagaaa ataaaattct
17761 tacggcacgt gatgctgcaa tcggcttaga aatgaaaatt tttagtgaac tgtgtagtga
17821 agttgctaaa gagtctgaaa aaattgctct tgctgcaaat gctttggcaa aacttgatat
17881 tagaaccgcg tttgcagagc ttgcagtgca aaataattac gtaaaaccca ttattgatga
17941 cagcaaagag ttcaatatct gtagcggaag acatccagtg gttgaagtta acgataaatt
18001 cattgcaaat agcatcaatt tagctggtat acatctaatt actggtccta atatggctgg
18061 aaaaagcact ttcttgaggc aaaacgctct cattgcaatt ttagctcaca tggggtcatt
18121 tgtgccagca gagagtgtac atattggagt gattgacaag atattcagca gggttggcgc
18181 aacggataat ataacagctg gttattctac cttcatggta gagatgatcg aaacagcaac
18241 gatagtaaat caggcaacgg accgttcctt ggtgatactt gatgaaattg gtaggggcac
18301 aggggtgtat gatggattat ctattgcaca ggcggtgatt gaacatattc acaatgtaaa
18361 taagtgccgc gccatttttg caactcatta tcatgaatta actaaagtaa gcaaatactt
18421 gaagaacgtg aaatgttttt gtgtaaaaat aagagagtgg aacggagagg ttatctttct
18481 acatgaagta attgaaggca ttgcagatga gtcatatgga atacatgtag caaaacttgc
18541 tggttttcct gattctgttt taaatagagc aagtgaggta tttgaggagc tgaaggcttg
18601 aatcattatt ataagtgaag aattacctat acaatcgtgg tagtagctat agaaacgaaa
18661 aacttacttg acacccttcg ccagcgccct tatcatgata gtgaagctat tgtatttgct
18721 ttcgccaatc tgcagattaa aaggtaagga ttacttaatg tatcggcgtc ttatgttcaa
18781 ttttttgcag tatatagata ctgtatgtct ttacaaaact tcatctacat ctagattttt
18841 atctaaataa gctgaacgcg cttataaagc gttacaagac atcaaaaaat gccaatactc
18901 gacagagata gtaaaagact agctaactcg gggattcttt gtcttttttt ctgcttggta
18961 aatttcttaa acatttgcag cgtaagttag ttgcaattaa aagcggctga atctcgcttg
19021 tcaagcgttt aaaacataaa aaacgccaat attttaaagc ggataataac cccagggctt
19081 cttttgcctt ttttttcatt tggtaaattt cttaatgttt gtggctaaca tgaacaaaca
19141 tttgagagta acttccgcta gtagggtgtc atcccagtgt ctgggcactg ggatgacaaa
19201 aaaaggaggc tactcggatg acagctagcc tgacaaccgt catcccgctg cttgttagcg
19261 ggatctcttg ttagcgccgc ggcggtatga cgtagaattt tacactcacc aattcagtgt
19321 ccaatctgga ttccagactg gaatgacacc cttttggatg gaaaccagtg tcaggacact
19381 gggatgacaa gaaagaggct acttggatga tacctatagc cctgtcattc cagcgcgtga
19441 cgctggaatc tagttcttat tatatatcca gagatttact atattagtaa tatcaaacta
19501 gatcaattct tgctacttca atttttcttt cagttaattc ctgcctggca cgttaagtct
19561 agccttttat aaatgaacca gaattataat ttatctttta aaaatctcaa atgtaataaa
19621 tttatgtttt aaaggatttg atatatcaaa ttttattgta tttactttct catcaaaaac
19681 tatagtattc tcatctatgt gatatctgtc tatttgatat gctagtgaaa atgcgtcaag
19741 cttatcagaa gttggaggag atttatatac cactacattt aaagtttcta agtctaagtt
19801 cactctaaca gtcacaacac cttgttcaaa cgaactttca aacaaacctt gaatagattc
19861 accatgcata catggcgggt ccattcttac agcagatata catcgattat atattgcatc
19921 ccctgaaccg ttgtgcatgc acatcgtaaa atctttactt ccttgaaaaa ggtctataaa
19981 atctaccata acacttcacc actacataac aaacacttta taatattata agtaactagc
20041 aagctccatt actaatttcg tagtgactct aattgcaaaa tttaatataa gtagtaaagt
20101 agaaccactg aataaaaaac atatgttaat taatacaacg tcggagctgg aggacatatg
20161 tgagggggttg atagcaaaaa agccaaaatt tatagcagtt gacacggagt tcattagaaa
20221 taatttaatc tactacccaa aattatcgtt aattcaaatt tcttacggag agaagagttt
20281 tattgtagac gcattagtgc cagaaattga tttatcattc attaagaaaa taatgctaaa
20341 tcaggggata accaaagtgt ttcatagctg ccggcaggat atagaatcct tactcactgt
20401 atttaaatgt attcccactc ccatttttga tacccaagtt gctgctatgt tttgtcatta
20461 ttatcatgac tttattggtt actcaaaagt agtggagcaa tatcaaggaa tagcactgga
20521 taaaattaaa gctaaaaatt cagattggtt aaggcgtccg ttgtccgaag atcagttaga
20581 ttatgcagta aacgacgtgg tacacctata tgacctatac caaatattgt gcaataaact
20641 tgaagaaaat aataggatgg gttggtttca agaagagatg gaatcaatag ttgatataaa
20701 taagtattta catagcccaa aagatgcatg gaagaggatt aaatttaatt atgaagtaaa
20761 tccaagattg atactaactg ttaaagcagt tagtgagtgg caagaaacct tagcacagcg
20821 ctataatatg aaccgtaata aaataatcaa taatgctgta atagctgatt ttattgaaaa

20881 aaatgtagag catgttgatg agattttaga tgatctcaag aggaatacaa aaaatataaa
20941 agatgcggat ttactagagt ttgtgaatat ttttaatgaa aatgagagaa attggatgca
21001 gcaaaacaac accttatcga gtaattatga taaatctgta cttgatatac tctcaattat
21061 tttagatagt aaatgtaaag aaagtaacat atcaaggaaa ttagtttctt caaaagatga
21121 gttaaccggg tcaatatcta gacagacaga taaattattc aagggatgga gatatgattt
21181 ttttggcagg tcagttgaat catttttgaa cacaagctca aaatttgaga tttcagcagt
21241 gaaatccgca aataatataa ccaaaattca gagtaattta gtggagaata actgttgcta
21301 aagtaactta gatttatcga caatcgtcat cccgctacgt gttagtggct aagagatacc
21361 gcggcggtat gacggttaag tagggtgtca tcccagtgcc ttgactactt cgatccagga
21421 aaattgattg taaacgagca aacattttcg ataaaattac caaaaagcta gatcccagac
21481 tgggatgaca ccacctacaa attgcaatgt tcgtatagtt atgtgcttga cactgggatc
21541 tagaaaatct agttaatata tatagagaaa ccttagctat agtttcaaaa gaagtcgaat
21601 gtattttgtt atatgattat aagtctaaca ggcggaattg gggtaggaaa gagctttgta
21661 gctaattgct ttcaagagtt tggtgctgtt gtatttgatg ctgattctgt tgtacaccaa
21721 ctttataaag tggataaaag cataataagt tatgcagaga aaaattttcc tggagtggta
21781 gtaaatggtg aaatagatag aacagtactg tctaaatatt tcttagccta cgatgaaaat
21841 tggaaacaat ttcaatcttt ggttcactct gctgtgctgc gtgaattaga atttttttatt
21901 gccaaggaaa aaaagatcga tagaaaactt ttagttttag atgtgccact tctattagaa
21961 acaaaatttt atttatattg cgaccttatt gtttttgttc atgcagatag cgttgtgcaa
22021 gctcaaagac ttaacgaacg caacatagat aaagaaaagc ttaatctaat ctccgatgtt
22081 cagttatcta ttgaggagaa aaggaagatg agtgacttta ttatcgatac cagtgtaagc
22141 aaagagtatg tttttttctca agtaaaagat atagtggatt cattaaacct aaacacgtag
22201 ctctacgtca taccgccgcg gtatctctag atcccgctaa caagcagcgg gatgacggtt
22261 gtcaggatcc cagcagcctc cctcccctgt catccaagta gccccctatg gtgtcatccc
22321 agtgcccaga cactgggatc caggaaaatt gattgtgcac tatagcaaca tttttgatca
22381 aaaactggat tccagtgggc tttgttgcat cgctacttat gaaaggctaa ctatagttag
22441 gattataagc aaactattga aaatcttgtt tttttgcaat caatctgatc aaatttaaga
22501 atagtaattt gttatttata ttaataaatt taattctatt gaaaatagct aaagcattga
22561 aattcttgaa ttttagccgg attggtaaat ggtagtgaaa tttcttttac tcaaatttag
22621 gtattcattg accgttcttg ttataaatac cagaataata agctactgat attccctatc
22681 ttttttatct ttaatccatc atagctagcg atgcaacaaa gccattccag tgtcaagcac
22741 tggaatgaca cccttccgat gggaactgct ctcaaactac aacagcatga gtagctatag
22801 aagctttaat gatacaagaa aaaataaaca gctatcattt ttttagtaat taagtaaaat
22861 agagtgaaag attgcgagga agttttgaaa aataagataa aaaagatagt aatttcagga
22921 aaggaagtgt ggccaatcat cgagggcggc aaaggcattg cagttagtga tggaagatca
22981 agcggagcat ttgctgcagc agatgctgtg ggtacatttt ctggtgcaaa tgctaagctt
23041 attgatgata atggcaagtt agtgccacta atttataaag gcaaaacaag aaatgaaaag
23101 catagtgaat tgattgagta tagtattgaa gctggaatta gtcaggcaag aatagcaaat
23161 gaaatatcaa aaggtcatgg aagaatacac atgaatgtac tttgggagat gggagcagca
23221 gaacgcgtgc ttcatggcat attagaagga gcgaaaggtt tgattcatgg tattacctgc
23281 ggtgctggta tgccttatag gcttggagaa attgcagcta aatatcaggt ttattattac
23341 cctattatct catcggtgcg tgcttttaaa gcattatgga agcgtgctta ccaaagaata
23401 tcttcctatt tgcttggtgg agtagtgtat gaagatccat ggcttgctgg agggcacaat
23461 ggactcagta acagtgaaga tccagagcta ccacaggctc catttgaaag agttgcagag
23521 cttagatctt tcatgaacga gatcggtctt tcagaaacac caattgttat ggcaggagga
23581 gtgtggcatt tgaaagattg ggaacattgg tttaacaatc tgcaaattgg accaatagct
23641 tttcagtttg gcactcgtcc acttttgacg aaagaaagcc caatttctgc agagtggaaa
23701 aagaagctac tcactttaga agaaggtgat gtatttttaa ataaattcag tccaacaggg
23761 ttttactcat ctgcagtaaa aaataacttc atacgtgagt tgcaggaacg gaattcacgt
23821 caaataaagt tttcagaaaa tgctagcgaa gaatttaata atgaatttgc aatgggtagc
23881 agaggtagaa agatctacct tactgcaaaa gacaaagaac tggcaaatac atggactaaa
23941 gcagggtaca cagaagcaat gaaaacccca gattcaactg ttatttttgt gacgccagat
24001 aaatttaaac aaataagaga agatcaaatc aattgtatgg gatgtttgag ccattgtttg
24061 tttagcaatt ggaaagatca tggtgaccat tcaacaggac gaaagccaga tccgcgtagt
24121 ttttgcatac aaaaaacact gcaaaacgtt atacacgatg gtgatattga gaacgaactc
24181 atgttttccg ggcacaatgt gtacaaattt aagcaagatc cattttatga gaatggctac
24241 gtaccggcag taaaagaact ggtggaaagg atattgactg ggtattgagt cagtgacctt
24301 acccagaaaa agtggagaga aagtgtggaa tgttttttac aaaaagaatg atgcatcaaa
24361 ttgttctgga atgcaatagt taatagtgac gttgtttgtt ataaaaaatt tctatgtatt
24421 caaatattgc agttctagtt tgttgagcag agtgttgtga agtatcaata agtatttctc

24481 ttttcaatga gctaaagaag ctttccacaa cagaattgtc gtaacaacaa cctttatgac
24541 tcatctatgt tttttatgga taagagatat tgataacctt gcaaagtata ttgtgaacct
24601 tgatcactat gtaatagtag atttttggca ggtttacgct tgttaacgtc cattaataaa
24661 gagtccataa ccaattgttt atttattgca ctactcatta actaccatac gtgagaatta
24721 ttgctgccaa atatagccat ccctccttgg ttttgatgta agtaatccca tactttattt
24781 ggttgatcaa caataaagtt ttgatctagt atattgggag ctacagctct attatctgtt
24841 tgttgttttt taattttaaa ctttcttctc agtatagccc tgatgtcatt tttctgcata
24901 atactttgca ccgttttcaa attgcaactt ttacccaaag ccttcaattc agcatgaatt
24961 ttaggagctc catatctaca tttagaagct tgatatattt tttgaatatc tgctaataac
25021 tctttatttg ctgattctct actgcttatt tttcttgtgg tccacttata gtagccacta
25081 gcagaaaatc ctacataatt cctgtacttt atagcagtta ctatataaaa aaatatttta
25141 ctctttttga ctggccaggg cttttttttta aatgtctctt tcccttgtta ctcttgcttt
25201 gtaaatcaaa cctctctttg tcataaggtg ctacatctgg aaatgcattt gccgctgact
25261 ttttttcgtt atatttcttc atccattttc ctaatacact atcccttatt cctagatctc
25321 ttgctacttt tgcaactggt tgacctacct ttctttgttc atttgtcagc ttaacagctt
25381 ccaatttgaa ttctgccgta tattctctta ccatttctaa tcctccaaaa ttttatttta
25441 cccaaaaaaa ctcttttctt tctctccact ttttctgggt aaggtcagtt tactggcctc
25501 tccatgagtg agattgaaaa attaagtgga aatctgtgaa cggttacctt ggatgacaga
25561 aaagagtgtt ggaaggaggc aatattaaaa acttaatttc agtccagcca agaacacata
25621 accagagttg ttattttcta ttttaggctc ttttgtacta aactttacaa tatcgaaata
25681 gtatgaagta cctaaaaaaa gagaattttc aagacttaaa gcatatgatg ttaactcgtt
25741 tgtaccttcg cctgcaatat ccctaccact actaaaatat gttaagctcg atttgtaaga
25801 atcagaacgg taagcgatac ctgcattcat ataatacgta ttatttgttt caggattacg
25861 cttctcacca gatttaccac cattaccata cgaaaaaatg caatcaagat cgaacaattt
25921 tagcttcaaa ccaaaattcc agtgtagtaa ttgattgcgg cattcataat ttttagaagt
25981 cccatctgag caatcagtta aattttctct tgcaaattca ccagttaagg cagtagtaaa
26041 acctatatca tctgacaagc tgtttttata ggaaaggcca gcagcaatta aattgctgta
26101 ctgtaagtta ttttttcctg gaacataact gaatccgagt tgaaagttat agatttcagg
26161 ggagatgtag ttaattgatg attgttttaa ccctagttcg agttcattat aatcgctata
26221 aatattgggc ttgacccaga acaccttgtc ttgatcatat ccagggtcac catccttttt
26281 atgatcaccg cgcaaatttg catagtttgt ccaatgacca tttactcctc cagcagctgc
26341 ataaattttt gaagtgttaa ttaacatgct ctgactaact aaacttcttt taccatattc
26401 aattgatcca agttcctggt tttttatgat aaaataccat tcctccatgt ctaacttttc
26461 tatgtctaac gcttttaggt ttgcaatacc agaaacccct gccttaacat taacccccat
26521 ttgagtattt ggataaactt gctgtaaata aagaaagcgc aagtatgaat aacttgaagt
26581 tttatcctta ggcttagcat tgaaagaatc tctgttgaag gcgtagccga atctcaaatc
26641 tatttttccg tctagttctg cataaaatat ttccttatcc aaaattgttt tagagctagc
26701 actattctgc aaaaacaatg atgaaagcag tgctaccagg tagtacttta acttcataaa
26761 taaccttaaa tctttataaa aatttaagtt taaactcaat ataaataaaa aacaactttt
26821 cagaaggcac cgctggtaga ttttgctgcc tgtttttgag ctatattccc ataaattatt
26881 gatacacttg gtaatttttg cattaataac tgcttattaa tggcagaatg tatacgatta
26941 attattttag atgtactttt tacatcgcag ttaatcaaaa caaaaagcac tttattctca
27001 tcaatatacc ctagaaaatc atcactacgg caatttttat aaaaatgttc tatgacaact
27061 ttcagtgcat catttgtctc actacagcta ctatttaagc caatcattcc tatagctgca
27121 ttaatttgat gttgaaaggc gaaattcaat ataacgtata actctagaaa tagtggactc
27181 attgcccggt atatcgaata agtcatggtt tttatatttc ttacccatta gatatttcat
27241 ctctaaagat tcctagttta tgggacaaac tgatatcacg tattaatagc tcataattta
27301 ttttattact ggcaaattgt gcagtgcgga aaacttttac ttttgtcctg atatcttctc
27361 ctttagcatc agttaaggaa aaatctatca ctttaggtaa tatatcaagt aaatcgtgcc
27421 cattttcggt atactctaaa taattcttca tgtcgtcagc tgctcttgca cttaaaatat
27481 tgattaacgg cttattaagt aaattccctt cctcgtattt taacaaattc cttgcggctt
27541 gattgagccc taaaattaac acgttttttt tcttattatc ctgacaaaca gataccacag
27601 catcatcttt tcttcttgca gttatgaaat catccatact ttcattctat acagcgtatt
27661 tataagtata ataagattat attaagattt acttacttat tgatatgctt gattaggtat
27721 agacaaaatg tcatacggga tggctttgtt gtagaagaat caagattact gtttatagac
27781 ttcaagttac tgcaaaaaat attttaaaac atagtgatat cttgacttta cctagttaat
27841 attccaaatt ataaaaaaac agatagttct atgacgacaa gtctattaca aggcaaaaag
27901 ggattaataa ccggaataat aaataagaga tcgatagcat gcggtatagc aaaaagctct
27961 ctcagagcac ggagcagaac ttgcaatcac ctaccagaac gaaataataa aagaaaaact
28021 attaccaata gcagatgaac taaatgtgga gctaatatta cactgcgacg tttcaaatga

28081 aaaaatcgta gataacgctt tcgaaataat agagaaaaaa tggggtactc ttgacttttt
28141 ggtgcatgca atagcattct ccgataaaaa tgagttagat ggtaaatatg tcaatacttc
28201 actgaataac ttcctcaatg caatgcatat atcgtgctat tcttttcact gctttagcac
28261 aaagagctga aaaaatgatg tcaggtggtg gtagtttgct tactttatct tattatggcg
28321 ctgaaaaagt tatgccaaat tataacgtta tgggtttatg taaagcagca cttgaagcaa
28381 gtgtaaaata tatagcatgt gatctcgggc cacaaaatat cagagtaaat gcaatttccg
28441 ctggtccaat cagaacttta gcatcttctg gaataagcga ttttcacttc atatcagaat
28501 ggaatagaaa taattctccg ctcagacgca atgttacaat agaggatgtt ggcaaggcag
28561 cgctatactt attaagcgat ttaagtagtg gcactactgg agaaattttg cacgttgatt
28621 caggatataa cgttgtggga atgaaggtag ttgactaaca aagcactagt tccttcgggt
28681 tcatacacaa ctctatcagg agttttgtga ctgtgattgc agaaaacatc gaacataaaa
28741 ttcctattga taaagtgaca gaaaagcctc taattgctcc actgccaata atgaacatta
28801 ttcctgcagc aattaatgta gtgatatttg aatccagtat tgtctttata gcgtttttaa
28861 acccttcttc aatagcacgt tccactctct tgccagattt tatttcctca cgaattcgct
28921 caaatattaa aacatttgca tccaccgcca taccgacagt gagtgcaata ccggcaattc
28981 caggtaaagt tagagttgct tcaagcagag tgagaattga taatataaga attacattag
29041 aaaaaagcgc aacagaggcc aatacaccta atttaccgta agtgatgatt ataaataaag
29101 ccacggctat aatagagatt gttgctgcca tttcccctgc ctttattgat tcttctccaa
29161 gacttgggcc aatgtttttt tcttcaatta ttttaagtgg tgctggcagt gctccagatt
29221 ttaaaagtat tgcaagttcg cttgcttgtt tttcagtaaa attaccgcta atttctccct
29281 ctccatttaa aattggctca cgtattgtag gtactgttaa gactgtgtta tccagaacaa
29341 ttgcaaaagg ttttcccaca ttttctttag tgatttttgc gaatctttta ctcgctatgc
29401 tgtcaaattt aaaatgcact gttggtttac ctaagtgacc aaatctaact gatgcgttaa
29461 ccagcgaatc accacctatt tcagttttgc ggaatattgg ataagaatta cctaaagaat
29521 ccttgagcat gacagtggtt tcatggtcta tatcctgcac cttggctatg ttagtgtttg
29581 ccaaatgaaa agctagctta gcagttttgc caagcagaga ttttatctgc ttggtatctt
29641 ctactccagg cacctgcact aatattttat tctgcccttg tttttgaacg ctgacttcct
29701 ttgtgccaag tttgtccaaa cggcgctgga cattgtttat tgattctgca gctacttcac
29761 tgatcaaaga atttttataa tggggtttat acgaaatgag aatcgaagaa tcttttctat
29821 ttagctctaa atttggattt atcgcattaa tcaacacaga tgctttttta taatcatcaa
29881 tattatttaa agttataatg ctattttgta cgttagattc aatatttttt gttagcaatg
29941 tctcttttat ttcgttagct agcatgctta gcttttcctt aaaatagaag tctaagtcta
30001 tgtttaaaag caaagatgcc cctcctttca ggtcaaggcc taagtttatt ctttttttcg
30061 aaataaaaag cttattatca aagaaatttg gcagtgttat gtataaagca aataaacaga
30121 tgcataatac tgaaaaagat ttgactataa gtctgttata cataagttgt atgtttaaaa
30181 ttgtaataat tgattgtaag ttaaaaatag atttttgtaa aattctctat ttgaccttac
30241 ccagaaaaag tagagagaaa gttaagacgt tttatcatg agaataaatt gtgcaggaat
30301 gcaatagtta atagtagaat gtctacgtta tttgttataa aagatttcta tgtattcaaa
30361 tatggcagtt ctggtttgtt gagcgagaag tatcaataag cagctccctt ttaagtgaac
30421 ttttctgcaa cagagttatc gtaacaacag cccttatggc tcatgctgga aatgatgttt
30481 tttgtattca atagaaattg gtaattttga gaggtatact gtgacccttg atcgctatgt
30541 aacagcagat ttttatcggg tttgcgttta tcaacggcca tcaataaaga atctataact
30601 aattatcatg ttactcatta accaccatgc gtgaatatag gtcaattatt gccgccaaca
30661 accttcttta gtttttatgt aggtaatccc aaactttatt tggctgatca gtagtaaaat
30721 tttgatccaa tgtgttcgga gccacaactc ggttgcttat ttgctgtttt ttatttttga
30781 accttcgtct aagcttggtt ttaatgccat tttcttgcat aacactttgt actgttttaa
30841 tttttgccta aagcttttaa ttcagcgtga attttagggg ctccatatct gcatttagaa
30901 tgcttgatgt attttttgaa tatctgctag aagcccttct tttgctaatt ctttactgct
30961 tttttcctta gcaaccaata ccaccagtac aaaaccctac atagttcctg tgttttatag
31021 caatttctat ataaaagaat actttactct ttttctttgc ccagggcttt ttttaagatg
31081 tctctttctc ttattagctc tttttttaga tcaaatcttt ctttgtcata aggagccttt
31141 gcctggaaat gcgtatgctc cagacatttt tccatcatat tttcttactt aacatactgc
31201 catctatacc tagatctttt gctatttttg tacttgcttg acctgtttct ttaaccagct
31261 taacagcttc taatttgaat tctgctgtat attctcttct atttgtcata ttttgaaaaa
31321 acgttttatt ttactccaaa aacgtcttaa ctttctctct actttttctg gactactccg
31381 aatcggttga caaattggac gtttgataag cataatttgg agcttcttgt gtaatgacta
31441 tatcatgagc gtgactctct cttaatcctg atgcagtaat agtaacaaat ttacaatttt
31501 ttttcatctc ttctatattc ctattaccag tataccccat tgcagcttgc aatccgccaa
31561 tcaattgatg gattactcct gaagctggac ctttgaatgg aactcttcct tccactcctt
31621 gtggaactaa tttgagtttt gaatctttat cttgaaaata acggctagct gaacctcgtt

31681 tcattgcact aatagatccc attcctcgat agcctttata tgctctgccc ttatacataa
31741 taatctcacc tgggctttcg tcagtgccag caaaaatcga accaatcatc acagagtcag
31801 caccagctgc aatagctttt gcaacatctc ctgagtattt taccccacca tcagcaatta
31861 gtctgacgtt tcttgctcta cacgcctctg caacattctt gattgcagag aattgtggca
31921 caccaacacc tgtaactatt ctggttgtac agattgatcc tggtcctatt ccaaccttca
31981 ctgcatcaac acccgcatca atcaacgctt cagcagcctc ctttgttgca atgtttccgc
32041 caattaattg cgtattagga tacatctttt ttatttccct aatggtattg ataacgtttt
32101 cggaatgacc gtgagcagta tccacaacaa ctacatcaac ttcttctccg atcaaagctt
32161 cacatctgtc tataccatct tttttaccag tgccaattgc agcggcaact ctgagacgcc
32221 ctttactgtc tttacatgaa tttgggtatc tattgtattt ttcaatgtct ttaaccgtaa
32281 ttaaacctat acaacaagaa ttttcatcta cgaccaaaag cttttctatt ctattttcat
32341 gcaacaattt cattgctgag gcactattca ctccctgctc ccgcactgtc actaacttat
32401 cttttgtcat cacttcggaa acttttacat tcatgttctg gtcttcaata aatctcacat
32461 ctcggttagt taaaattcca actaacttgc gttgatcgac tacaggaatg ccagaataat
32521 tgtgctctct cattaatgaa attgcttccg caactgtttt atccggtgaa atcgtaattg
32581 ggttatacac gatccaactt tcatattttt tcacccttct tacttctaaa acttgttcat
32641 ctattgataa attcttatgt atgcaaccta tccctccatg ttgggcaata gctattgcaa
32701 agcccgattc agtaacagta tccattgcag aggatatgag agggatattt agttctatac
32761 tatttgttaa ataagttttt gtatctgcat cgcaaggcaa tatattagaa taggctggca
32821 aaagaagtat atcgtcaaac gaataacaag cttccatttt tttcataggc tttatttaaa
32881 gcttatacgc caattaatta aattgcaata ggcaaaatta gctaggagcc tttaattgta
32941 tagctctaac tgtttttagg agatagaaaa gctatatagt tgctaataca ttttattgca
33001 aaataagcat tctacattat ttgccagcaa acaaaactgc tcaatagtta agttttctgg
33061 acgctggttt ccgctcaatt tagcgttttc aaggacagtt tcagcgtggt tcgttatatt
33121 ttgcaagcta tttctcagca tctttcttct ttgagaaaaa acagcacgtg ttaatcttgt
33181 taaggtttcc aaatttactg caaattttgg gataggcaaa ggatttactg taattactga
33241 agaatgtatt tttggtcttg gaaaaaactc tttaggttca atatcaaatt cctttttat
33301 atcacatagt aactggctta gcactgataa ggaaccataa tctttagaat tgggtcttgc
33361 cgtaatgcga tctgctacct ctttttgaaa cattaatgtc aagttcgtaa aaaattttat
33421 attatttaac cactttaaaa ataacactac cgagatattg taaggcaagt tagcaataac
33481 tttaactgga cgttctatca gctcttcttc tataacgtgc agtgcatctg cttctataat
33541 tttatacttt ccttgatgct catttagcag ttgatcatgg tgttttacta aatctctatc
33601 tttttctata gaaagtagag actttggatt atgcaccaat atttctcttg ttaatgcacc
33661 atatccagga ccaatttcaa tgacattaaa attctccaga ctagcagcta aagcgactat
33721 tttttgtta tctcactcga taaaataaaa ttttgcccta gactcttttt tggcttcagt
33781 aaaaattttt tcatattatt ttcattatac agtattttaa aaattagttg actaaaattc
33841 attgataatt gtatattatt aatgtgttta acgccggctt agctcaattg gtagagcaac
33901 tgacttgtaa tcagtaggtt gtcagttcaa gtccgacagc cggcactctc actcatataa
33961 gtaaagttga tgtccttttt aatagtagca aattggaaaa tgaatggaat gcgttcttca
34021 tttgttgact ttataggcaa acttaacaac aagagcaacg aaattacctc taagctagtg
34081 atttgccctc cttttacatc atttccaagc agtatagagt tgaacaataa tattaatata
34141 ggagcacaga attgccatca taaaaagttc ggttcttaca caggtgaaat tagtgcagaa
34201 atgttaaaag aactagggtg tacttacgta atacttgggc attctgaaag ggctaatgaa
34261 aaagatagtg aaataaaact taagtcggaa atagcaatag aatcaggctt acacccaatt
34321 atctgtgtag gtgaaaattc agaagattat aagaatgaaa aaacaaaaga agtaatagaa
34381 tatcaatgta aaaaccgctt gccaacacac ggtgaatata ccgtagcata tgagccaata
34441 tgggcaatag gcacaggtca tgtgccaaat aatgatgcaa ttgctaaggt gatagaggta
34501 ataaaattgt gtactagtaa aaaacacatt atatatggtg gttcagtcag ttcagaaaat
34561 atagaaaatt tgctcagtat ctcaaattta tcaggagttt taattggcag tgcaagctta
34621 gatttcgatc acttttataa aattatacaa caagttgaaa aaaagttttc tcttatcaat
34681 tctaaaatca gcaactaaga atcctataac aaatgacatc atttcagtgt caagcatata
34741 tagctaaccc aagaaaggtt tccctacgtc ataccgcgat tcattcgcgg tatctctaga
34801 tcccgctaac acgtagcggg atgacgaatt tgttgttttt caaattgtag gtaaacctaa
34861 gccactttag ctatatctgt acgaacatct agtaaaacga cctagctata gctaaagtaa
34921 tctagaaaat taacgatatt ttattccccg gggaacgctc tattctgttt tccaactcta
34981 gttccaaaag agccattaga gctatgttgg tagaaagtcc gcttgctaat ataagttcat
35041 ctatatcaac aggtacagag ttaatatgat caactataac agattttgcc tgttgtaatt
35101 tttcttgttt ttggttaaca gcatagtgct caacatcaaa tagttttttt tgctgcggag
35161 gtagactaaa cctaatactc tctattatgt catcagcgga ttcgataagc ttagcaccgt
35221 ttttaattaa ataattacta ccactacagc gtgaatctaa agggaaacca gaaactgcaa

35281 acacctctct cccttgattt aaagcaaaat ctgctgttat tagagagcca gaacgtttcg
35341 atgcttcaat cactgcaaca cctagcgata ggccagatat aattcgattc ctctgaggaa
35401 aatactgagg ctttggttta gtagcaaatg gaagctcagt aattactaac ccaccgttcc
35461 cagtgatttt tttgtataga tcaaaattct cttttggata caccacatca attccgcttg
35521 ctgtaacagc aatagtggga tgatttttgt atattacact gtttgctgca gtatcgattc
35581 cttttgcaag tccagaaacg ataacaaaac cagcttcgct taaatcaagt gccaacttat
35641 tggcaaaatt tcttccattc attgaagagt tacgcccacc gattattgca attatctcac
35701 ggcttaataa tgatatatca ccaagcgcag ttattacagg aggacaacta gagatatttc
35761 ttaaaagatc agggtaatct gggtcacatg cgggtataat tttagctcca attctttctg
35821 cattattgat ttcttctcgt gcatcttgaa tgccatacac cttattatta gccaccctat
35881 tgagatattt tagcacctca tctagcgatc catgtgttct tagtatacta aaaaactttg
35941 ttggtcctat agttctagcc aagcttaacc atacttctaa ttctttgttg ttcaatttat
36001 ttatcttcat aaaaggcttg aatttcttat caatttagtg tacccaagat aatgcaaata
36061 tacagtgttt tatgacgcat tttgttacag ataaatgcat aaaatgtaaa tatacggact
36121 gtgtggaagt atgtcccgtt gactgcttct atgaaggtaa aacatgctc gtgattaacc
36181 cagatgaatg tattgattgc ggagtgtgca tacctgagtg tccagtagat gcaattgtaa
36241 ctgatgattc cataaaagat attttagaac tagatgaaga gttactgaac agtgaacaaa
36301 aaatcttcaa atcattttac aacataaacg tagaatactc acaaaaatgg ccaaatatca
36361 cagctaagaa gcaacctctc tatacagcag aagagtataa ggaaaaaaag gataaaacaa
36421 cttattttga ggaaaattta gaataatact gctgaaggaa aagaaacttt ttgtatttca
36481 gggttattgt ttttggttgc tgaaaatagt tgcgttatct aagttgttat gctgataaat
36541 tagtatcgat taatttaagg attaaggaaa aaggcttatt gacttttgaa atatttgatt
36601 ataaaactag ctgttagcat taaatcttgg gggtaagtta tgagtataaa taaaagagaa
36661 accattaaaa agattgaagg gttacttttt caagcgaacg gtaatcttga aaaaacatgt
36721 gatctattat caacagtaaa gttaaccgaa gatgatatta aaatattgct ggaagagatg
36781 ataaaagagt attatagctt gtttaaaaag atttcttgga gttgtggaat tactgcaggc
36841 ggtgcacaag atgccaacgc aagcagcttc aacgaagctt ataagttagg ttcaaaagag
36901 ttagcattgg caataatgtc agattttaag aagtcttatg atcaagaatt attcaagaaa
36961 tataaaaagg aagaaatgtc ttatcctgaa ggagaaggag tacaatttat tagagaatta
37021 agagttctaa gtagaatact tactatatct ttaataagga aacttgatga agtttactcc
37081 agaagaaatt gagcgtttga aggaatatat attagttagc aactatgata caagcaaaag
37141 tctttctgat atagacaaat atatgcatgc actatctgcc ttaattagaa acccctcact
37201 tgatacaaaa aagatatttg agctaaataa tcgaatgata atacacacta cagccttaat
37261 tgatttagta actagtggag tagtgaaaaa agaagaagca aaagaacaat ctgctgagga
37321 tttagaaaag cttgtctgtg aacagtataa aggtgcagca aacacgccag taccgttgcc
37381 gagcacgcta tttgaagtgg ataaaaaaag tgcttaaagg aaagtaagaa ggtcttatag
37441 ttagccaaag taggtattta ataagcttaa tgtaaccatt atatatgcta tatagtaaaa
37501 catagaatgc ttatggttcc aactatagca attttaagtg gtgggttttc ttgtgagaga
37561 gaaatatcgc ttatgagcgg aaaagcagta aagaaggcgc tcgatagcct ttcatataat
37621 gcaatagaaa tagatgttga cagtaacatt gctgaaaagc ttagaaaaat taattcgggc
37681 cttgctttta tcgctctgca tggaccttat ggtgaagatg gctgcattca aggtttattg
37741 gagattttag gtataaaata tacacactca ggagttatgg cttctgctgt tgctatgaat
37801 aaagtgatgt caaagcatat atttcggtcc cttagtatcg acactccaaa aggttatgta
37861 attagccgag aagatctact aaaaaataat attaaaatcg attatccgta cgtcttaaaa
37921 ccaattaatg aaggttcaag tattggagtg tacataattt tctcgcacga ggattactta
37981 gaactgaaag ataatagttc tactataatg gaaaagatga tcgtagaaga atacataccg
38041 ggcatagagt tacatactgc tgtattgctg gatgaagcaa ttggcactat agaagtacga
38101 ccaaaaaata aattctatga ttatgaagca aagtatacag atggatttgc agaacatata
38161 tttcccgctg aaattcctaa caatatatat aaaatgacct tggaacacgc actgaaagtt
38221 catcaatttt taggatgcaa aactatttcc cgctcagatt tccgttataa tcccaaaaat
38281 aacactttaa aaatgcttga gattaataca catcctgggt ttactgagtt atcgttagtg
38341 ccagaaattg caaaattggc aaaaggaatc aattttaatg aattagttaa aattattatt
38401 gaagatagtt tgcagcacaa aaatattagg gatttaagtc atgttgagca gtattactag
38461 aagccaaagg agttttttgc gtaagtgtgc cttggttatt atcacagcac tttttcttaa
38521 gctaatactt tacagctcac ttgataaaat aataaatcga tttaattact actttacttg
38581 gtataatgac tgcctatcta gtttattact cagtagtgga ttttcaatcg acgaagtagt
38641 cgtgagcggc aataaattta caaacaaaaa ggatattcta agtttgacag atagaacgca
38701 acctatcctg tatatatcac tttcaaaact tgccggtaac atacaatccg taagcaggtg
38761 gataaaacac gtaagagttc acagaatttt gcccaatact ctacacatta atatagatga
38821 gcataaaccg tttgctctct ggaaagataa taacaaaacc tcggtaattg atttgaagg

38881 taaagtgatc gtagatgact atctagtaga tgatcttgtt gtaattaaag gacaaaactc
38941 gttatcaaac ctagaatttg ttagagatgt gttggagagt aaaactcaat taagagacca
39001 catttcttct tttgcttata tagggaatag aagatggaat atcatacttg ataacgattc
39061 cacagtaaaa ctgcctgaag ataaccctta tagtgcatgg gactatttaa accacttaca
39121 caatacaact gattttactt tcagcgattg gagtattatt gatatgcgta ttactgataa
39181 aatttttgtg aagaggtgat ctgctgtcat ccaagtagcc cctttcctg tcatcccagt
39241 gcccagacac tggaatcccg ccgcgaaccg tcataccgcg attcattcgc ggtatctcag
39301 ccgctaacaa gagatcccgc taacaagcag cgggatgacg attgtcgttt agccataaat
39361 attaagaaat ttaccaaatg aaaaaaaggc aaaagaagcc ccgtggttgg ctaattactt
39421 acattatact ttcaagtatg gcgttttttt attctaaacg tttaataacc gcgattcagt
39481 tgcttttaag ccgcaactaa tctaaattat aaacattaag aaatttacta aacagaaaaa
39541 aaggcaaaag aaaccctagg gtagctagtt attcactatc cattttttaa gttggcgttt
39601 ttttatgttt taaatgcttt ataagcgcat tttagcttat attaggtaaa aacctagaat
39661 tttataaaga cataaggtgc acatagtgca aaaaattaaa catgaaacgc cagatacgtg
39721 agtttttttg tcatttaatc tgtacagaga agataaataa atagcttcag tttcatgata
39781 agggggctgg cgaggtttgt caaggaattt ttatgcaaga gatctattga aatgcccctg
39841 taaaattata ctattaatta gtacactata aatttaaaat tatagatatg ttaagcagat
39901 tggtaatttt gctattagtt tttatacttc ctttttcttc atactcatac cagtttagaa
39961 ccaaagcaaa gcaagcagta gtcttagatt tagcttcaga ctcattcatt tttgaccata
40021 attctgatga aaaaatggcc ccatcttcaa tgagcaagct aatgacttta tatatagcct
40081 tcgattatct aaaagctgga ataatacaca tggaggataa atttcgagta agtagaaaag
40141 cgtgggaaag gagaggctct tccatgtttt tgaaagaagg tcaatccgtt acggtgaggg
40201 aattacttga aggaattaca atagtctcag gcaatgatgc ctgcataaca ctagccgagg
40261 gcattgcagg atcagaagag aattttgtgg ctgaaatgaa tgaagttgca caaaatttga
40321 atctgaatga cagtcatttt gtcaactcaa gcgggtggcc agatgaagat cattttatga
40381 acgcaaaaga cttggtaatg ctggcaaaaa ggattttcac cgattttcct gaatattatg
40441 atttattttc cgaacaatat ttgacatata atgaaattgt acaaaagaat aaaaatcttc
40501 tactttttca cgatattggg gttgatggct taaaaaccgg ttatacaaat gctggtggtt
40561 acggcattgt agcatctgca aaacgaaatg ataggagaat ttttgctgtc gtaaatggtt
40621 taaacactga gaaagagcga atagaagaag caaaaagatt gatacaatac tccttaaatc
40681 attttaacac taagaaaata ttcgttaaag acagcgtagt tgaggaagta aatgttctct
40741 atggaaaaga tagaaaagtg cctattacag ttgcaaacga tgttaccata acttacaacc
40801 gcaaattgca tgaccaaatt aaggtgcgta ttgaatataa agatatgata cctgcaccca
40861 ttaaaaaagg tcaagaagtg ggcaaagttt ttgtagaaat accaggtatt gagcaacaaa
40921 ctacaccact ttatgcagca aatgacgtgc aggaattgaa tttcgtagaa aagtttttta
40981 ggatgttgtt ttaagtttga tattaaagca attccgaaag agagatctat tgcactttag
41041 tgtagtttta actaacaatt tctgttacaa tacaagattg aaatgttacg aaaatgtgct
41101 ggttgtattt gttgctgtct agtatcctag agatactgtg ggctgtaacg ctaaagttta
41161 gtaacagttt tactaaagtt gtgccttcga ttgcaacatt ggtaattatg atcattagca
41221 tttacttttt atctcttgca gcatgctttc tacctattag ggtatgctat gcagtttcct
41281 ctggaatatg cacaattgga ataactataa ttggtgcaac cactttcaca gagaatatta
41341 atttatctca agttttatgt atcgttttaa ttgtaatagg aagcataggg ctgaagctct
41401 ctatttaaac ctaaaaattt atgcatgaat tggatatatt tactcttatc gtcactaatt
41461 gaagtgttct gggtcataac acttaagcat agttatggat ttactcatct cgtaccatca
41521 ataattagca tactctccat ggctcttagt acatatttac tttcgctcgc tacacgttct
41581 atacctatcg gagtatgcta tgcaatttgg actggagtgg gcgctatagg agcttcgata
41641 cttggagttt acctatttga tgaaccagtt aatttattcg aagtaatatg ctttatttta
41701 gttacattcg ggattatagg attaaaattg tttgatactg caaaacaaac tgttgataaa
41761 atggaataaa agatcccact gggatgaaag aagcagatac tccatattgt catgcaaatt
41821 ggaatccaga ctttttttcta tatagcatgg tgaagacggg aaaccgtaac cgttcacaca
41881 atggcgtcaa cttaaggaaa aatatcagcg atagtgtata aaatttctct ctaaattttt
41941 tatcattaaa ttatccaaag agtgcaaggg acagcacttt tgtttctgtt ttatttcaac
42001 aaagaagtct aaaatgtgtc ctttttaggt gaaatatgca aaaaggaaaa aaccttattc
42061 tacaaattaa aaatataata tactcgaaaa cttttcagaa aatccattgt gttgagtttt
42121 acacgaacaa gaaaacttcc tttttcgaca gtattttcta tgattttaaa gttagttaaa
42181 aaaagtttgg gaatagaatg tgaacttatg gagccattga catgtaccct caaagcaagc
42241 tttttctaaa gcaaggtata agatttgcca tacaggcttt caagaattta agcatccaaa
42301 cagcctatca ggatgaacct gagttggaga ggctatagac ttattgcagc agatggttct
42361 ggtatgagat tacccagctc tggggaaatt gtatccgagt ttgaaccaaa tggaacaaca
42421 ggcaccattg gcaatttgtt tgttgatttg tgtacttcgc taatttgtag tgctcgtctt

```
    42481 gcagcttgga atataggtga acaaacgttg gcggcagagc aattacccga agttatcact
    42541 caaatgcgtt tattaaatca agagaaatta ttatttatct atgatcgtct tcagtaaaat
    42601 tcattcagca gcattatgat ttggaagtag attttatttt tcgcttgcaa aaaagaaatt
    42661 atagtaagct atgggaacgc tggcgaatta gattttgatt ttatattaga aaatgaaaag
    42721 ctaaaaaata aaatgaaagg acagaaagta agagttatag tgctgacatt agccaactga
    42781 ga
//
LOCUS       JAQZAU010000067          35791 bp    dna     linear   ENV 17-MAR-2023
DEFINITION  MAG: Wolbachia pipientis isolate wEsol contig_39139, whole genome
            shotgun sequence.
ACCESSION   JAQZAU010000067 JAQZAU010000000
VERSION     JAQZAU010000067.1
DBLINK      BioProject: PRJNA929258
            BioSample: SAMN33142973
KEYWORDS    WGS; ENV; Metagenome Assembled Genome; MAG.
SOURCE      Wolbachia pipientis (insect metagenome)
  ORGANISM  Wolbachia pipientis
            Bacteria; Pseudomonadota; Alphaproteobacteria; Rickettsiales;
            Anaplasmataceae; Wolbachieae; Wolbachia.
REFERENCE   1  (bases 1 to 35791)
  AUTHORS   Fiutek,N., Couger,B.M., Roy,S., de la Torre,J.R. and Connor,E.F.
  TITLE     Genomic assessment of the contribution of the Wolbachia
            endosymbiont of Eurosta solidaginis to gall-induction
  JOURNAL   International Journal Molecular Sciences
REFERENCE   2  (bases 1 to 35791)
  AUTHORS   Fiutek,N., Couger,B.M., Roy,S., de la Torre,J.R. and Connor,E.F.
  TITLE     Direct Submission
  JOURNAL   Submitted (10-FEB-2023) Biology, San Francisco State University,
            1600 Holloway Ave, San Francisco, CA 94132, USA
COMMENT     ##Genome-Assembly-Data-START##
            Assembly Date          :: JUN-2019
            Assembly Method        :: FLYE v. 06-2019
            Genome Representation  :: Full
            Expected Final Version :: Yes
            Genome Coverage        :: 100.0x
            Sequencing Technology  :: Illumina HiSeq; PacBio Sequel
            ##Genome-Assembly-Data-END##
FEATURES             Location/Qualifiers
     source          1..35791
                     /organism="Wolbachia pipientis"
                     /mol_type="genomic DNA"
                     /submitter_seqid="contig_39139"
                     /isolate="wEsol"
                     /isolation_source="galls on Solidago altissima"
                     /host="Eurosta solidaginis MN-2018-EFC"
                     /db_xref="taxon:955"
                     /environmental_sample
                     /country="USA: Cowling Arboretum, Carleton College,
                     Northfield, MN"
                     /lat_lon="44.48 N 93.13 W"
                     /altitude="280 m"
                     /collection_date="2018-11"
                     /metagenome_source="insect metagenome"
                     /note="metagenomic"
     gene            236..970
                     /locus_tag="wEsol_00739"
     CDS             236..970
                     /locus_tag="wEsol_00739"
                     /codon_start=1
                     /transl_table=11
```

/fullproduct="qcoverage 100 , hcoverage 100 , similarity 100 , identity 100 , evalue 2.13e-168 , alnlength 244 , Bacteria , Proteobacteria , WP_015589136.1 MULTISPECIES: 3-oxoacyl-[acyl-carrier-protein] reductase [Wolbachia]"
/cog="FabG COG1028 251 Dehydrogenases with different specificities (related to short-chain alcohol dehydrogenases)"
/pfam="Shikimate_DH Pfam-B_3397 adh_short KR NAD_binding_10 Epimerase Saccharop_dh adh_short_C2"
/tigr="TIGR01830 3oxo_ACP_reduc 239 3-oxoacyl-[acyl-carrier-protein] reductase"
/product="3-oxoacyl-"
/translation="MLKLESRKFLITGASGGIGQAIVKIMHKAGATLCISGTKKEVLE
EVAKQYEKNMHVLPCDLSNAEEVNQLINRASELMEGFDGLVCNAGITRDSLLLRMTDE
AWQKVIDINLSSTFKLSREACRKLIKNNWGRIINISSIIGLTGNAGQANYAASKAGII
AMSKSIAKEVASRNITVNCIAPGFIDTKMTEVLNEGQKGKILDNIPMKRMGTGEEIAA
GVLFLASDEAKYITGHVLNINGGLFM"
/besthit="qcoverage 100 , hcoverage 100 , similarity 100 , identity 100 , evalue 2.13e-168 , alnlength 244 , Bacteria , Proteobacteria , WP_015589136.1 MULTISPECIES: 3-oxoacyl-[acyl-carrier-protein] reductase [Wolbachia]"

gene 1066..1401
/locus_tag="wEsol_00740"

CDS 1066..1401
/locus_tag="wEsol_00740"
/codon_start=1
/transl_table=11
/pfam="TrbC"
/fullproduct="qcoverage 100 , hcoverage 100 , similarity 100 , identity 100 , evalue 4.12e-74 , alnlength 111 , Bacteria , Proteobacteria , WP_007550269.1 MULTISPECIES: TrbC/VirB2 family protein [Wolbachia]"
/translation="MSSIIGFFGVLCVVLLFSFSGYADNPGVDMDDTTKVICNIIGYV
WGIGGPLMTVVIIGAALLAIFGRMPWPALFALGMFCGVFFGAKTIITKIMPNVSDEAK
TMLEKCGQK"
/product="TrbC/VirB2 family protein"
/besthit="qcoverage 100 , hcoverage 100 , similarity 100 , identity 100 , evalue 4.12e-74 , alnlength 111 , Bacteria , Proteobacteria , WP_007550269.1 MULTISPECIES: TrbC/VirB2 family protein [Wolbachia]"

gene complement(1429..2709)
/locus_tag="wEsol_00741"

CDS complement(1429..2709)
/locus_tag="wEsol_00741"
/codon_start=1
/transl_table=11
/fullproduct="qcoverage 100 , hcoverage 100 , similarity 100 , identity 100 , evalue 1.62e-291 , alnlength 426 , Bacteria , Proteobacteria , WP_012673175.1 MULTISPECIES: M48 family metalloprotease [Wolbachia]"
/cog="COG4783 COG4783 484 Putative Zn-dependent protease, contains TPR repeats"
/pfam="Peptidase_M48 Pfam-B_1978 Pfam-B_1799 Apc3 Pfam-B_333 TPR_11 TPR_12 NARP1 Pfam-B_1822 TPR_16 TPR_11 TPR_2 TPR_12 TPR_1 Pfam-B_392 Pfam-B_324 TPR_11"
/product="M48 family metalloprotease"
/translation="MFRVAKFLTLLFFFAYCNNAYSINIIRDSEVEAIVKELAQPLFS
AADIDHEQIKVFIINDNSINAFVINNNSIFIHLGLLQYSAKPYVLLGILAHEIAHISA

GHILQMSSAMGYFQSIAMISYMVGLVSSIIINPQVAGAILLSGVAFSSRLFFNYSQEQ
ESVADSYALRYLDESGYDNSGMKEIFDYFKSIEHENTEEYFRTHPLSEKRIFAVQNYK
VKNNVKPIFADKLLKFERVVAKLDSFFAPIHVLSNKYEDNSEYVNAIVCYRQGKIEEA
IAKVNSLIQESHSDPYLYELKAEMLYKAGNLSEAIKMYEESLRYLSEKNSYLVKLALS
HTLLLHGDAKKAIFYLEQILNVEPNNAFVWKYLSIAYKCEADTAMHYFALTKKACIEG
DLKQFMKYAELAVKTLPKDSPYLLQVEDMKRFNG"
/besthit="qcoverage 100 , hcoverage 100 , similarity 100
, identity 100 , evalue 1.62e-291 , alnlength 426 ,
Bacteria , Proteobacteria , WP_012673175.1 MULTISPECIES:
M48 family metalloprotease [Wolbachia]"

gene complement(2781..3425)
/locus_tag="wEsol_00742"

CDS complement(2781..3425)
/locus_tag="wEsol_00742"
/codon_start=1
/transl_table=11
/fullproduct="qcoverage 100 , hcoverage 100 , similarity
100 , identity 100 , evalue 2.33e-148 , alnlength 214 ,
Bacteria , Proteobacteria , WP_007549343.1 MULTISPECIES:
3,4-dihydroxy-2-butanone-4-phosphate synthase
[Wolbachia]"
/cog="RibB COG0108 203 3,4-dihydroxy-2-butanone
4-phosphate synthase"
/pfam="DHBP_synthase Pfam-B_7664"
/tigr="TIGR00506 ribB 204
3,4-dihydroxy-2-butanone-4-phosphate synthase"
/product="
3,4-dihydroxy-2-butanone-4-phosphate synthase"
/translation="MVQATYASMSLPGISSVEDVLEDARSGKLFILVDDENRENEGDL
IVLAEKIKPEHMAFMVRYGTGVVCLAMTKFHMKRLGLNFMEKKNIGENHTAFTTSIDA
RYGITTGVSAEDRTKTIHAAINKDGTQDDIITPGHVFPVIAHKGGVAQRAGHTEASVE
MAKLVGFDHSAVICELVNDDGSMMRLPQLLKFAEQHKIKLTTIDKLISYVKNLN"
/besthit="qcoverage 100 , hcoverage 100 , similarity 100
, identity 100 , evalue 2.33e-148 , alnlength 214 ,
Bacteria , Proteobacteria , WP_007549343.1 MULTISPECIES:
3,4-dihydroxy-2-butanone-4-phosphate synthase
[Wolbachia]"

gene complement(3432..3926)
/locus_tag="wEsol_00743"

CDS complement(3432..3926)
/locus_tag="wEsol_00743"
/codon_start=1
/transl_table=11
/fullproduct="qcoverage 100 , hcoverage 100 , similarity
100 , identity 100 , evalue 6.03e-106 , alnlength 164 ,
Bacteria , Proteobacteria , WP_007549342.1 MULTISPECIES:
transcription elongation factor GreA [Wolbachia]"
/cog="COG0782 COG0782 151 Uncharacterized conserved
protein, YhbC family"
/pfam="GreA_GreB_N Pfam-B_5216 GreA_GreB"
/tigr="TIGR01462 greA 151 transcription elongation factor
GreA"
/product="transcription
elongation factor GreA"
/translation="MASSIINKFPMTREGFENMQAELEKLKEEKPSVIQAISDARDQG
DLSENAEYHAARERLGFIEGRIMEIESKLSHAEVIEVKNLSGDSVMFGATVTLSMLSD
NNGEVEYVYKVVGEYEADVSKQLISTSSPLGSALIGKKVGEYVEVTVPNGEKLYKIVK
IEFK"
/besthit="qcoverage 100 , hcoverage 100 , similarity 100
, identity 100 , evalue 6.03e-106 , alnlength 164 ,

```
                     Bacteria , Proteobacteria , WP_007549342.1 MULTISPECIES:
                     transcription elongation factor GreA [Wolbachia]"
     gene            complement(3936..5477)
                     /locus_tag="wEsol_00744"
     CDS             complement(3936..5477)
                     /locus_tag="wEsol_00744"
                     /codon_start=1
                     /transl_table=11
                     /fullproduct="qcoverage 100 , hcoverage 100 , similarity
                     100 , identity 100 , evalue 0.0 , alnlength 513 ,
                     Bacteria;Eukaryota , Proteobacteria;Arthropoda ,
                     WP_012673176.1 MULTISPECIES: F0F1 ATP synthase subunit
                     alpha [Wolbachia]"
                     /cog="AtpA COG0056 504 F0F1-type ATP synthase, alpha
                     subunit"
                     /pfam="ATP-synt_ab_N HAS-barrel ATP-synt_ab
                     ATP-synt_ab_C"
                     /tigr="TIGR00962 atpA 503 ATP synthase F1, alpha subunit"
                     /product="F0F1 ATP synthase
                     subunit alpha"
                     /translation="MKNSMNVSEIASIIREKVETFDNPIKRENIGEVISVTDGIALVY
                     GLEKAKFGEKVFFASGVEGIVLDLDHDTAGIVVLGNDRDVKEGDVVKCSGDVVQVPVG
                     HELLGRVVNALGHPMDDGGEIRAKNRMDIESKAPGIIDRKSVHEPLQTGIKIIDLLIP
                     IGRGQRELIIGDRQIGKTTIALDTIINQKKINDEVNENQKVYCVYVAIGQKISTVAKV
                     VNKLKESGALEYTTVVVASASDCAPMQFLAPYAGCTIGEFFRDNGMHCLIIYDDLSKH
                     AVAYRQMSLLLRRPPGREAYPGDIFYVHSRLLERAAKMSDKKGQGSLTALPIVETQAG
                     DVSAYVPTNVISITDGQIFLESELFYKGFRPAVNIGLSVSRVGSAAQLKSVKKVAGSI
                     KLSLAQYRELEDFAKFGSDLDASVQLSLNKGKYLVELLKQKQHLPMSIEEQVVLMYIF
                     SNLYNQLSKIQISYINKFEHDLINYFHTVHPGVLKKLSNDMSDDIKGDIFNIVSNFVT
                     QFNCV"
                     /besthit="qcoverage 100 , hcoverage 100 , similarity 100
                     , identity 100 , evalue 0.0 , alnlength 513 ,
                     Bacteria;Eukaryota , Proteobacteria;Arthropoda ,
                     WP_012673176.1 MULTISPECIES: F0F1 ATP synthase subunit
                     alpha [Wolbachia]"
     gene            complement(5474..6034)
                     /locus_tag="wEsol_00745"
     CDS             complement(5474..6034)
                     /locus_tag="wEsol_00745"
                     /codon_start=1
                     /transl_table=11
                     /fullproduct="qcoverage 100 , hcoverage 100 , similarity
                     100 , identity 100 , evalue 8.57e-119 , alnlength 186 ,
                     Bacteria , Proteobacteria , WP_010962742.1 MULTISPECIES:
                     ATP synthase F1 subunit delta [Wolbachia]"
                     /cog="AtpH COG0712 178 F0F1-type ATP synthase, delta
                     subunit (mitochondrial oligomycin sensitivity protein)"
                     /pfam="OSCP Pfam-B_17059"
                     /tigr="TIGR01145 ATP_synt_delta 172 ATP synthase F1,
                     delta subunit"
                     /product="ATP synthase F1
                     subunit delta"
                     /translation="MKKTQYNNLVSSYARVLFHVSGSRLGIIRKEVEFLLAFFKDQRD
                     VFVYLSHPMISFAHKKEVMLSINEHLSENLVKFIMVIFANKRSSLLILILEKFLSLAR
                     ENENEFEITIKSAETLKESDIKIITESLSFLGKIIKVSNVVDPSILGGFVVRYGFNLI
                     DASLKSYLDRLVDLSKMEILKVRNFV"
                     /besthit="qcoverage 100 , hcoverage 100 , similarity 100
                     , identity 100 , evalue 8.57e-119 , alnlength 186 ,
                     Bacteria , Proteobacteria , WP_010962742.1 MULTISPECIES:
                     ATP synthase F1 subunit delta [Wolbachia]"
```

```
gene            complement(6076..6426)
                /locus_tag="wEsol_00746"
CDS             complement(6076..6426)
                /locus_tag="wEsol_00746"
                /codon_start=1
                /transl_table=11
                /fullproduct="qcoverage 100 , hcoverage 100 , similarity
                100 , identity 100 , evalue 4.36e-76 , alnlength 116 ,
                Bacteria , Proteobacteria , WP_012673177.1 MULTISPECIES:
                YraN family protein [Wolbachia]"
                /cog="COG0792 COG0792 114 Predicted endonuclease
                distantly related to archaeal Holliday junction
                resolvase"
                /pfam="NERD UPF0102 Pfam-B_2097"
                /tigr="TIGR00252 TIGR00252 123 TIGR00252 family protein"
                /product="YraN family
                protein"
                /translation="MVSRLRYFVGYFGELLVLVYLKLKWYNVIKRRYRCKFGEIDLIV
                SKKKELIFIEVKTSLLGKEIPISHLQCQSIINSSKYFLSKNLSFLDYSVRYDLYFLSL
                KRRPVYIKNAWIEE"
                /besthit="qcoverage 100 , hcoverage 100 , similarity 100
                , identity 100 , evalue 4.36e-76 , alnlength 116 ,
                Bacteria , Proteobacteria , WP_012673177.1 MULTISPECIES:
                YraN family protein [Wolbachia]"
gene            complement(6450..6878)
                /locus_tag="wEsol_00747"
CDS             complement(6450..6878)
                /locus_tag="wEsol_00747"
                /codon_start=1
                /transl_table=11
                /fullproduct="qcoverage 100 , hcoverage 100 , similarity
                100 , identity 100 , evalue 8.70e-93 , alnlength 142 ,
                Bacteria , Proteobacteria , WP_007550873.1 MULTISPECIES:
                50S ribosomal protein L17 [Wolbachia]"
                /cog="RplQ COG0203 116 Ribosomal protein L17"
                /pfam="Ribosomal_L17"
                /tigr="TIGR00059 L17 112 ribosomal protein L17"
                /product="50S ribosomal
                protein L17"
                /translation="MKHGIKKRKLSRCTEHRLSTLKNLSISLINHEQIVTTLPKAKEL
                RPYVEKFITIAKNKNTLHGRRLLLSRLHNSKLAVDKLLNVLASRYQDRKGGYSRIIKF
                STRKGDCASMAVIELVDRDIAARGKVYSKNKEGGKVVTQS"
                /besthit="qcoverage 100 , hcoverage 100 , similarity 100
                , identity 100 , evalue 8.70e-93 , alnlength 142 ,
                Bacteria , Proteobacteria , WP_007550873.1 MULTISPECIES:
                50S ribosomal protein L17 [Wolbachia]"
gene            complement(6886..7953)
                /locus_tag="wEsol_00748"
CDS             complement(6886..7953)
                /locus_tag="wEsol_00748"
                /codon_start=1
                /transl_table=11
                /fullproduct="qcoverage 100 , hcoverage 100 , similarity
                100 , identity 99.7 , evalue 2.49e-250 , alnlength 355 ,
                Bacteria , Proteobacteria , WP_012673178.1 MULTISPECIES:
                DNA-directed RNA polymerase subunit alpha [Wolbachia]"
                /cog="RpoA COG0202 317 DNA-directed RNA polymerase, alpha
                subunit/40 kD subunit"
                /pfam="RNA_pol_L RNA_pol_A_bac RNA_pol_A_CTD
                Pfam-B_14457"
```

```
                     /tigr="TIGR02027 rpoA 298 DNA-directed RNA polymerase,
                     alpha subunit"
                     /product="DNA-directed RNA
                     polymerase subunit alpha"
                     /translation="MYYNNDVSLCSNLDKLIKPSSVKVISDDSSEKSDIVLEPLESGF
                     ALTLGNALRRVMLSSLKGSAVYGIKIEGVTHEFTSIQGVREDVTDIVLNMGMLRCKLN
                     GASNKCLNLNAKGPCQVLAGMIETDDQCSIVNKDLLICTLGQNVELNMTIYVASGKGY
                     LPVTKYKENELLKLMSEQDLIGFIPVNALYSPVNRVSYRVENSRVGQVTDKDKLILSI
                     ETDGTISPSQAVDSAARILQEQLQPFISSDVSYKKSQVSSSSGAKDLGYDPILLRKVD
                     EMELSVRSHNCLKNENITYIGDLVQKTEGEMLRTANFGRKSLNEIKAVLTNFGLSLGM
                     NVLNWPPKDIDELAKQHTDED"
                     /besthit="qcoverage 100 , hcoverage 100 , similarity 100
                     , identity 99.7 , evalue 2.49e-250 , alnlength 355 ,
                     Bacteria , Proteobacteria , WP_012673178.1 MULTISPECIES:
                     DNA-directed RNA polymerase subunit alpha [Wolbachia]"
     gene            complement(7975..8361)
                     /locus_tag="wEsol_00749"
     CDS             complement(7975..8361)
                     /locus_tag="wEsol_00749"
                     /codon_start=1
                     /transl_table=11
                     /fullproduct="qcoverage 100 , hcoverage 100 , similarity
                     100 , identity 100 , evalue 2.41e-81 , alnlength 128 ,
                     Bacteria , Proteobacteria , WP_015589137.1 MULTISPECIES:
                     30S ribosomal protein S11 [Wolbachia]"
                     /cog="RpsK COG0100 129 Ribosomal protein S11"
                     /pfam="Ribosomal_S11"
                     /tigr="TIGR03632 bact_S11 117 30S ribosomal protein S11"
                     /product="30S ribosomal
                     protein S11"
                     /translation="MKKVKTVSKSKKEFITGVVHIRATFNNTFVNVTDVHGNTLCQTS
                     VGACGFSGSRKSTPYAAGKAAESAAKNAMERFGMKVVSVIIRGPGFGTEAAVKALQSC
                     GLTVTSIADKTAIPHNGCRLRKKRRV"
                     /besthit="qcoverage 100 , hcoverage 100 , similarity 100
                     , identity 100 , evalue 2.41e-81 , alnlength 128 ,
                     Bacteria , Proteobacteria , WP_015589137.1 MULTISPECIES:
                     30S ribosomal protein S11 [Wolbachia]"
     gene            complement(8386..8754)
                     /locus_tag="wEsol_00750"
     CDS             complement(8386..8754)
                     /locus_tag="wEsol_00750"
                     /codon_start=1
                     /transl_table=11
                     /fullproduct="qcoverage 100 , hcoverage 100 , similarity
                     100 , identity 100 , evalue 2.56e-80 , alnlength 122 ,
                     Bacteria , Proteobacteria , WP_010962745.1 MULTISPECIES:
                     30S ribosomal protein S13 [Wolbachia]"
                     /cog="RpsM COG0099 121 Ribosomal protein S13"
                     /pfam="Ribosomal_S13 FbpA"
                     /tigr="TIGR03631 bact_S13 113 30S ribosomal protein S13"
                     /product="30S ribosomal
                     protein S13"
                     /translation="MARIAGINIPVRKCVPFALTYIYGIGIATANVICHACEVDKRKR
                     VSELRDEDIEKISGFIRQNYVIEGELRKEVAMNIKSLVEMGCYRGVRHRKGLPVRGQR
                     THTNAKTRKGRSRLPIAGKK"
                     /besthit="qcoverage 100 , hcoverage 100 , similarity 100
                     , identity 100 , evalue 2.56e-80 , alnlength 122 ,
                     Bacteria , Proteobacteria , WP_010962745.1 MULTISPECIES:
                     30S ribosomal protein S13 [Wolbachia]"
     gene            complement(8814..9455)
```

```
                     /locus_tag="wEsol_00751"
     CDS             complement(8814..9455)
                     /locus_tag="wEsol_00751"
                     /codon_start=1
                     /transl_table=11
                     /fullproduct="qcoverage 100 , hcoverage 100 , similarity
                     100 , identity 100 , evalue 5.93e-146 , alnlength 213 ,
                     Bacteria , Proteobacteria , WP_007550178.1 MULTISPECIES:
                     nucleoside monophosphate kinase [Wolbachia]"
                     /cog="Adk COG0563 178 Adenylate kinase and related
                     kinases"
                     /pfam="Pfam-B_5968 AAA_17 AAA_18 ADK Spt4"
                     /tigr="TIGR01351 adk 212 adenylate kinase"
                     /product="nucleoside
                     monophosphate kinase"
                     /translation="MIITIFGPPGSGKGTQSSLLIAKYNLKLISVGDLLRNIISSSSE
                     LGKKIKGTVESGNLIQDEIICGLLRDQLALVDDNCLLDGFPRNLNQAHFLTQVLQEKY
                     NRDVDIVVELQLDDNIAIDRLKNRLACLDCKSIYSVSSFKSTTCAKCKSTRLEKRIDD
                     ADMSAINKRISEYHLQMKGLREYYKGKLLTIDANLSVDEVTQEIESKISCNLV"
                     /besthit="qcoverage 100 , hcoverage 100 , similarity 100
                     , identity 100 , evalue 5.93e-146 , alnlength 213 ,
                     Bacteria , Proteobacteria , WP_007550178.1 MULTISPECIES:
                     nucleoside monophosphate kinase [Wolbachia]"
     gene            complement(9452..10780)
                     /locus_tag="wEsol_00752"
     CDS             complement(9452..10780)
                     /locus_tag="wEsol_00752"
                     /codon_start=1
                     /transl_table=11
                     /fullproduct="qcoverage 100 , hcoverage 100 , similarity
                     99.8 , identity 99.8 , evalue 7.84e-298 , alnlength 442 ,
                     Bacteria , Proteobacteria , WP_010962747.1 MULTISPECIES:
                     preprotein translocase subunit SecY [Wolbachia]"
                     /cog="SecY COG0201 436 Preprotein translocase subunit
                     SecY"
                     /pfam="Pfam-B_13180 SecY"
                     /tigr="TIGR00967 3a0501s007 414 preprotein translocase,
                     SecY subunit"
                     /product="preprotein
                     translocase subunit SecY"
                     /translation="MGNKSAFNSLDLTLLYKGDLLKRIFFTLIALICYRLGTYVPIPG
                     INLDVINDIFPKDGSGVFGVFNLFSGGALARMTILALNVMPYIMASIIIQLLSSAIKG
                     MKEVKNDGESGRRKMNSYIRYLTIVICVFQSIPILVGLEGMNREGVLVVIEPGVMFRT
                     IGVFSLLGGTMLLIWLGERISASGIGNGISLIIFTGIISELHSALSSLLTLNKNGSVS
                     LFIILFVLVLFFLLLLLVIFVESSYRKVIVQHPKKQFKRLHNDDFTYIPLKINLSGVV
                     PTIFANAILLTPISIANFYKGHAFSDFILNYFMANKVVYIVAYLVLIVFFNFFYTNFI
                     FNPEENADFLKKNGGFIPGRRPGKHTSDYLQDIVFKLTFIGSAYLVVICTVPEVMRYY
                     YDIPFIFGGTSLLIIVNVITDTIMQIQSYIFSNRYDNWIKKYESKTRKVR"
                     /besthit="qcoverage 100 , hcoverage 100 , similarity 99.8
                     , identity 99.8 , evalue 7.84e-298 , alnlength 442 ,
                     Bacteria , Proteobacteria , WP_010962747.1 MULTISPECIES:
                     preprotein translocase subunit SecY [Wolbachia]"
     gene            complement(10784..11254)
                     /locus_tag="wEsol_00753"
     CDS             complement(10784..11254)
                     /locus_tag="wEsol_00753"
                     /codon_start=1
                     /transl_table=11
                     /fullproduct="qcoverage 100 , hcoverage 100 , similarity
                     100 , identity 100 , evalue 1.45e-100 , alnlength 156 ,
```

```
                     Bacteria , Proteobacteria , WP_141456930.1 MULTISPECIES:
                     50S ribosomal protein L15 [unclassified Wolbachia]"
                     /cog="RplO COG0200 152 Ribosomal protein L15"
                     /pfam="Ribosomal_L18e"
                     /tigr="TIGR01071 rplO_bact 144 ribosomal protein L15"
                     /product="50S ribosomal
                     protein L15"
                     /translation="MNNAVKLNSIFTKLSKKKKPKLLGRGIGCGKGKTSGRGHKGQKA
                     RSGVSINGFEGGQQSIYTRLPKRGFKPIRRSIYSIINVGDIQRLMEAKKIVKDSVIDK
                     ERLYKLGFIKSIKDKIKLLNKGKLSEKFVFHVDFASEAAKKSVASVCGSVEILS"
                     /besthit="qcoverage 100 , hcoverage 100 , similarity 100
                     , identity 100 , evalue 1.45e-100 , alnlength 156 ,
                     Bacteria , Proteobacteria , WP_141456930.1 MULTISPECIES:
                     50S ribosomal protein L15 [unclassified Wolbachia]"
     gene            complement(11258..11770)
                     /locus_tag="wEsol_00754"
     CDS             complement(11258..11770)
                     /locus_tag="wEsol_00754"
                     /codon_start=1
                     /transl_table=11
                     /fullproduct="qcoverage 100 , hcoverage 100 , similarity
                     100 , identity 100 , evalue 1.52e-111 , alnlength 170 ,
                     Bacteria , Proteobacteria , WP_010962748.1 MULTISPECIES:
                     30S ribosomal protein S5 [Wolbachia]"
                     /cog="RpsE COG0098 181 Ribosomal protein S5"
                     /pfam="Ribosomal_S5 Ribosomal_S5_C"
                     /tigr="TIGR01021 rpsE_bact 156 ribosomal protein S5"
                     /product="30S ribosomal
                     protein S5"
                     /translation="MAIKNLQNNNDLSELLVSVRRVTTVTKGGRRFSFSILVVVGDEK
                     GRVGCGIGKHAEVAEARVKAVNAAKKSMIRVYLREGRTLHHDIKAKFCSGEIVLRTAR
                     AGTGIIAGGAIRSVFEVLGIKDVVAKSTRSNNPHNVICAVFKAFDSMLSPRQVASKRG
                     KKISEIVGNR"
                     /besthit="qcoverage 100 , hcoverage 100 , similarity 100
                     , identity 100 , evalue 1.52e-111 , alnlength 170 ,
                     Bacteria , Proteobacteria , WP_010962748.1 MULTISPECIES:
                     30S ribosomal protein S5 [Wolbachia]"
     gene            complement(11783..12154)
                     /locus_tag="wEsol_00755"
     CDS             complement(11783..12154)
                     /locus_tag="wEsol_00755"
                     /codon_start=1
                     /transl_table=11
                     /fullproduct="qcoverage 100 , hcoverage 100 , similarity
                     100 , identity 100 , evalue 1.26e-76 , alnlength 123 ,
                     Bacteria , Proteobacteria , WP_006279867.1 MULTISPECIES:
                     50S ribosomal protein L18 [Wolbachia]"
                     /cog="RplR COG0256 125 Ribosomal protein L18"
                     /pfam="Ribosomal_L18p"
                     /tigr="TIGR00060 L18_bact 114 ribosomal protein L18"
                     /product="50S ribosomal
                     protein L18"
                     /translation="MKRSYNFLSNSEKRKLRNRAKLDKSAERLRISIFKSNRHFYVQL
                     INDVKGITLTSASTLDAKIKDVCKGKVNAETIKQVSSLMVERLSGMKLEQQLTFDRGA
                     YKYTGLVSQFAEALRSSGFEF"
                     /besthit="qcoverage 100 , hcoverage 100 , similarity 100
                     , identity 100 , evalue 1.26e-76 , alnlength 123 ,
                     Bacteria , Proteobacteria , WP_006279867.1 MULTISPECIES:
                     50S ribosomal protein L18 [Wolbachia]"
     gene            complement(12160..12705)
```

```
                     /locus_tag="wEsol_00756"
     CDS             complement(12160..12705)
                     /locus_tag="wEsol_00756"
                     /codon_start=1
                     /transl_table=11
                     /fullproduct="qcoverage 100 , hcoverage 100 , similarity
                     100 , identity 100 , evalue 1.90e-124 , alnlength 181 ,
                     Bacteria , Proteobacteria , WP_007551152.1 MULTISPECIES:
                     50S ribosomal protein L6 [Wolbachia]"
                     /cog="RplF COG0097 178 Ribosomal protein L6P/L9E"
                     /pfam="Ribosomal_L6 Ribosomal_L6"
                     /tigr="TIGR03654 L6_bact 175 ribosomal protein L6"
                     /product="50S ribosomal
                     protein L6"
                     /translation="MSRIGAAPINIPAGVSVEYNNGSMLIKSAKAEKEVSLIRDVVCQ
                     IIDNQLLLSVDQDKDNYDKIKPMWGTYRSNINNIINGMVDGFSVNLEINGVGYKAECD
                     GKYLTLYLGYSHNVKYKVPKDVEIKCIKPTHLVVSGMDKQKVYMVASDICKIRKYDPY
                     KGKGVVIKGKFMLRKVVSKKK"
                     /besthit="qcoverage 100 , hcoverage 100 , similarity 100
                     , identity 100 , evalue 1.90e-124 , alnlength 181 ,
                     Bacteria , Proteobacteria , WP_007551152.1 MULTISPECIES:
                     50S ribosomal protein L6 [Wolbachia]"
     gene            complement(12722..13117)
                     /locus_tag="wEsol_00757"
     CDS             complement(12722..13117)
                     /locus_tag="wEsol_00757"
                     /codon_start=1
                     /transl_table=11
                     /fullproduct="qcoverage 100 , hcoverage 100 , similarity
                     100 , identity 100 , evalue 1.18e-87 , alnlength 131 ,
                     Bacteria , Proteobacteria , WP_007551150.1 MULTISPECIES:
                     30S ribosomal protein S8 [Wolbachia]"
                     /cog="RpsH COG0096 132 Ribosomal protein S8"
                     /pfam="Ribosomal_S8"
                     /product="30S ribosomal
                     protein S8"
                     /translation="MALSDSIGDFLTRIRNAQLAMHRATRVLFSKMNSSILEILKEEG
                     YILDYEKQVVDNLPSFIVKLKYYEKSPVISDIVRVSKPGCRRYSKYKDISKAYNGLGI
                     FIISTPKGVMTDYNAHKLKVGGEVLCRVF"
                     /besthit="qcoverage 100 , hcoverage 100 , similarity 100
                     , identity 100 , evalue 1.18e-87 , alnlength 131 ,
                     Bacteria , Proteobacteria , WP_007551150.1 MULTISPECIES:
                     30S ribosomal protein S8 [Wolbachia]"
     gene            complement(13139..13447)
                     /locus_tag="wEsol_00758"
     CDS             complement(13139..13447)
                     /locus_tag="wEsol_00758"
                     /codon_start=1
                     /transl_table=11
                     /pfam="SpoIIIAH Ribosomal_S14"
                     /fullproduct="qcoverage 100 , hcoverage 100 , similarity
                     100 , identity 100 , evalue 2.50e-64 , alnlength 102 ,
                     Bacteria , Proteobacteria , WP_006279345.1 MULTISPECIES:
                     30S ribosomal protein S14 [Wolbachia]"
                     /translation="MAKKSMIERNLRRIKLCDQYKEKREKLKSIINNKNLSIVERFTA
                     QNKLIKKLPRNSSKTKIRNRCALTGRPRGVYRKFGLCRIVLRDLCSFGQIPGVTKSSW
                     "
                     /product="30S ribosomal
                     protein S14"
                     /besthit="qcoverage 100 , hcoverage 100 , similarity 100
```

, identity 100 , evalue 2.50e-64 , alnlength 102 ,
Bacteria , Proteobacteria , WP_006279345.1 MULTISPECIES:
30S ribosomal protein S14 [Wolbachia]"
gene complement(13464..13997)
/locus_tag="wEsol_00759"
CDS complement(13464..13997)
/locus_tag="wEsol_00759"
/codon_start=1
/transl_table=11
/fullproduct="qcoverage 100 , hcoverage 100 , similarity
100 , identity 100 , evalue 2.23e-121 , alnlength 177 ,
Bacteria;Eukaryota , Proteobacteria;Arthropoda ,
WP_010962750.1 MULTISPECIES: 50S ribosomal protein L5
[Wolbachia]"
/cog="RplE COG0094 180 Ribosomal protein L5"
/pfam="Ribosomal_L5 Ribosomal_L5_C"
/product="50S ribosomal
protein L5"
/translation="MFKELYKDSIVKSLKDKFNYGNIMQVPKLVKVCINMGVGDAATD
SKAINEPLDSLYLIAGQKPLSTFAKKSISGFKIRKGATVGCKVTLRRDKMYEFLERLI
YIALPREKDFRGFSVKQFDGNGNFSFGIKEHISFLEIDYDKISKIRGMDINIITSAVS
DKEAKELLLALKFPFFD"
/besthit="qcoverage 100 , hcoverage 100 , similarity 100
, identity 100 , evalue 2.23e-121 , alnlength 177 ,
Bacteria;Eukaryota , Proteobacteria;Arthropoda ,
WP_010962750.1 MULTISPECIES: 50S ribosomal protein L5
[Wolbachia]"
gene complement(14004..14321)
/locus_tag="wEsol_00760"
CDS complement(14004..14321)
/locus_tag="wEsol_00760"
/codon_start=1
/transl_table=11
/fullproduct="qcoverage 100 , hcoverage 100 , similarity
100 , identity 100 , evalue 1.08e-64 , alnlength 105 ,
Bacteria;Eukaryota , Proteobacteria;Arthropoda ,
WP_006279347.1 MULTISPECIES: 50S ribosomal protein L24
[Wolbachia]"
/cog="RplX COG0198 104 Ribosomal protein L24"
/pfam="KOW Pfam-B_10078"
/tigr="TIGR01079 rplX_bact 104 ribosomal protein L24"
/product="50S ribosomal
protein L24"
/translation="MSAKIKSGDDVIVLTGKDKGKIGKVIKVIARDAKKKVIVSGVNV
HKRHTKPKAGSSGGILNKELAIDISNVATLDPKYKTPTRVGFKVIDGRKVRFAKVSGE
VID"
/besthit="qcoverage 100 , hcoverage 100 , similarity 100
, identity 100 , evalue 1.08e-64 , alnlength 105 ,
Bacteria;Eukaryota , Proteobacteria;Arthropoda ,
WP_006279347.1 MULTISPECIES: 50S ribosomal protein L24
[Wolbachia]"
gene complement(14321..14680)
/locus_tag="wEsol_00761"
CDS complement(14321..14680)
/locus_tag="wEsol_00761"
/codon_start=1
/transl_table=11
/fullproduct="qcoverage 100 , hcoverage 100 , similarity
100 , identity 100 , evalue 1.06e-73 , alnlength 119 ,
Bacteria;Eukaryota , Proteobacteria;Arthropoda ,

```
                     WP_006279348.1 MULTISPECIES: 50S ribosomal protein L14
                     [Wolbachia]"
                     /cog="RplN COG0093 122 Ribosomal protein L14"
                     /pfam="Ribosomal_L14"
                     /tigr="TIGR01067 rplN_bact 122 ribosomal protein L14"
                     /product="50S ribosomal
                     protein L14"
                     /translation="MIQKNTLLEVADNSGARAVLCIGLLGGRKSASIGDTVIVSTKSI
                     TPRGKVEKGKVYRAVVVRVKSPIRKSDGSVIRFSSNAVVLINDQGEPLGTRVFGPVKK
                     LSSGSFMKIMSLAVEVL"
                     /besthit="qcoverage 100 , hcoverage 100 , similarity 100
                     , identity 100 , evalue 1.06e-73 , alnlength 119 ,
                     Bacteria;Eukaryota , Proteobacteria;Arthropoda ,
                     WP_006279348.1 MULTISPECIES: 50S ribosomal protein L14
                     [Wolbachia]"
     gene            complement(14694..14930)
                     /locus_tag="wEsol_00762"
     CDS             complement(14694..14930)
                     /locus_tag="wEsol_00762"
                     /codon_start=1
                     /transl_table=11
                     /fullproduct="qcoverage 100 , hcoverage 100 , similarity
                     100 , identity 100 , evalue 1.62e-49 , alnlength 78 ,
                     Bacteria , Proteobacteria , WP_007549925.1 MULTISPECIES:
                     30S ribosomal protein S17 [Wolbachia]"
                     /cog="RpsQ COG0186 87 Ribosomal protein S17"
                     /pfam="Pfam-B_14863 Ribosomal_S17"
                     /tigr="TIGR03635 S17_bact 72 30S ribosomal protein S17"
                     /product="30S ribosomal
                     protein S17"
                     /translation="MPKKVFCGTVTKAKCDKTVKVSVLQVYKDELYKKVIKKYKKYTA
                     HDENNSCKEGDKVLIQEHKPISTTKKWVIVNSSH"
                     /besthit="qcoverage 100 , hcoverage 100 , similarity 100
                     , identity 100 , evalue 1.62e-49 , alnlength 78 ,
                     Bacteria , Proteobacteria , WP_007549925.1 MULTISPECIES:
                     30S ribosomal protein S17 [Wolbachia]"
     gene            complement(14923..15126)
                     /locus_tag="wEsol_00763"
     CDS             complement(14923..15126)
                     /locus_tag="wEsol_00763"
                     /codon_start=1
                     /transl_table=11
                     /fullproduct="qcoverage 100 , hcoverage 100 , similarity
                     100 , identity 100 , evalue 4.07e-36 , alnlength 67 ,
                     Bacteria , Proteobacteria , WP_006279350.1 MULTISPECIES:
                     50S ribosomal protein L29 [Wolbachia]"
                     /cog="RpmC COG0255 69 Ribosomal protein L29"
                     /pfam="Ribosomal_L29"
                     /tigr="TIGR00012 L29 56 ribosomal protein L29"
                     /product="50S ribosomal
                     protein L29"
                     /translation="MDIADIESRSSQELHEILVNLRKEFVNLVFQKKLGQCNNISRFS
                     LIRKSIARILTTLNKRRREEKNA"
                     /besthit="qcoverage 100 , hcoverage 100 , similarity 100
                     , identity 100 , evalue 4.07e-36 , alnlength 67 ,
                     Bacteria , Proteobacteria , WP_006279350.1 MULTISPECIES:
                     50S ribosomal protein L29 [Wolbachia]"
     gene            complement(15138..15551)
                     /locus_tag="wEsol_00764"
     CDS             complement(15138..15551)
```

```
                     /locus_tag="wEsol_00764"
                     /codon_start=1
                     /transl_table=11
                     /fullproduct="qcoverage 100 , hcoverage 100 , similarity
                     100 , identity 100 , evalue 1.71e-92 , alnlength 137 ,
                     Bacteria;Eukaryota , Proteobacteria;Arthropoda ,
                     WP_010962752.1 MULTISPECIES: 50S ribosomal protein L16
                     [Wolbachia]"
                     /cog="RplP COG0197 146 Ribosomal protein L16/L10E"
                     /pfam="Ribosomal_L16"
                     /tigr="TIGR01164 rplP_bact 126 ribosomal protein L16"
                     /product="50S ribosomal
                     protein L16"
                     /translation="MFIPKKSKYKKVFKGRIKGNTKGGSTLSFGDYGLKAMEAGRIQS
                     KHIETARRVISRTLKRSGKVWIRIFPDTPVSKKPADVRMGKGKGSVEFWVFKAKPGRM
                     LFEISSDVPMHLARLALEKATAKLPMKCKFVSNHN"
                     /besthit="qcoverage 100 , hcoverage 100 , similarity 100
                     , identity 100 , evalue 1.71e-92 , alnlength 137 ,
                     Bacteria;Eukaryota , Proteobacteria;Arthropoda ,
                     WP_010962752.1 MULTISPECIES: 50S ribosomal protein L16
                     [Wolbachia]"
     gene            complement(15566..16180)
                     /locus_tag="wEsol_00765"
     CDS             complement(15566..16180)
                     /locus_tag="wEsol_00765"
                     /codon_start=1
                     /transl_table=11
                     /fullproduct="qcoverage 100 , hcoverage 100 , similarity
                     100 , identity 100 , evalue 5.31e-140 , alnlength 204 ,
                     Bacteria;Eukaryota , Proteobacteria;Arthropoda ,
                     WP_010962753.1 MULTISPECIES: 30S ribosomal protein S3
                     [Wolbachia]"
                     /cog="RpsC COG0092 233 Ribosomal protein S3"
                     /pfam="KH_2 Ribosomal_S3_C"
                     /tigr="TIGR01009 rpsC_bact 212 ribosomal protein S3"
                     /product="30S ribosomal
                     protein S3"
                     /translation="MGNVNPKGFRLKIINTWSSIWYAEKGYKQGLHQDLSIRSYINES
                     FKHAGVSKVIIERTIDLVSVIIHSSRPGVIIGKKGSDIEKIKQKIAEKVKNNVEVNVV
                     GVKRSEIDAVLISNSIAQQLEKRVSFRRAMKKAIQSCLRMGARGIKVSCSGRLGGAEI
                     ARTEWYKEGRLPLHTLRANIDYAFAEAKTIYGIIGVKVWVYIGN"
                     /besthit="qcoverage 100 , hcoverage 100 , similarity 100
                     , identity 100 , evalue 5.31e-140 , alnlength 204 ,
                     Bacteria;Eukaryota , Proteobacteria;Arthropoda ,
                     WP_010962753.1 MULTISPECIES: 30S ribosomal protein S3
                     [Wolbachia]"
     gene            complement(16220..16567)
                     /locus_tag="wEsol_00766"
     CDS             complement(16220..16567)
                     /locus_tag="wEsol_00766"
                     /codon_start=1
                     /transl_table=11
                     /fullproduct="qcoverage 100 , hcoverage 100 , similarity
                     100 , identity 100 , evalue 1.53e-71 , alnlength 115 ,
                     Bacteria;Eukaryota , Proteobacteria;Arthropoda ,
                     WP_007549821.1 MULTISPECIES: 50S ribosomal protein L22
                     [Wolbachia]"
                     /cog="RplV COG0091 120 Ribosomal protein L22"
                     /pfam="Ribosomal_L22"
                     /tigr="TIGR01044 rplV_bact 103 ribosomal protein L22"
```

```
                     /product="50S ribosomal
                     protein L22"
                     /translation="MKNRDVIVKAGSRVLKSTPRKLNLVAGLVRNKKVSFATVQLRFC
                     EKKAAGLIRKVLNSAIANAQNYGLDIDNLYIKEILIGKSLTLRRVCPKAMGRANRVSK
                     RYSNITVKLGEII"
                     /besthit="qcoverage 100 , hcoverage 100 , similarity 100
                     , identity 100 , evalue 1.53e-71 , alnlength 115 ,
                     Bacteria;Eukaryota , Proteobacteria;Arthropoda ,
                     WP_007549821.1 MULTISPECIES: 50S ribosomal protein L22
                     [Wolbachia]"
     gene            complement(16573..16857)
                     /locus_tag="wEsol_00767"
     CDS             complement(16573..16857)
                     /locus_tag="wEsol_00767"
                     /codon_start=1
                     /transl_table=11
                     /fullproduct="qcoverage 100 , hcoverage 100 , similarity
                     100 , identity 100 , evalue 3.99e-64 , alnlength 94 ,
                     Bacteria , Proteobacteria , WP_006279181.1 MULTISPECIES:
                     30S ribosomal protein S19 [Wolbachia]"
                     /cog="RpsS COG0185 93 Ribosomal protein S19"
                     /pfam="Ribosomal_S19"
                     /tigr="TIGR01050 rpsS_bact 92 ribosomal protein S19"
                     /product="30S ribosomal
                     protein S19"
                     /translation="MSRSAWKPPFCHPSILKSVNNALNKGFVNMAIKVHSRASVILPN
                     CLGLKFAVYNGKDYIPVNVNDQNMIGHKFGEFSPTRKFTGHSGDKKATRR"
                     /besthit="qcoverage 100 , hcoverage 100 , similarity 100
                     , identity 100 , evalue 3.99e-64 , alnlength 94 ,
                     Bacteria , Proteobacteria , WP_006279181.1 MULTISPECIES:
                     30S ribosomal protein S19 [Wolbachia]"
     gene            complement(16861..17685)
                     /locus_tag="wEsol_00768"
     CDS             complement(16861..17685)
                     /locus_tag="wEsol_00768"
                     /codon_start=1
                     /transl_table=11
                     /fullproduct="qcoverage 100 , hcoverage 100 , similarity
                     100 , identity 100 , evalue 9.52e-196 , alnlength 274 ,
                     Bacteria , Proteobacteria , WP_015589143.1 MULTISPECIES:
                     50S ribosomal protein L2 [Wolbachia]"
                     /cog="RplB COG0090 275 Ribosomal protein L2"
                     /pfam="Ribosomal_L2 Ribosomal_L2_C"
                     /tigr="TIGR01171 rplB_bact 275 ribosomal protein L2"
                     /product="50S ribosomal
                     protein L2"
                     /translation="MGMKFFNPVTPSSRGTVLVSKVGLSKDEPEKSLTSGKKSSGGRN
                     NHGRITTRHRGGGHKKKYRVIDFKRNRSGQGIVEKIEYDPNRSGFLALISYKEDDIKS
                     YILAPQGMKPGDVVTAGNDADILPGNCLLLKHIPVGSFVHNVELKPGNGAAIARAAGC
                     YAQIVGRDGQYVLLRLRSGQIRLILSSCKATIGVVSNPDHKNRKLGKAGRSRWLGIRP
                     TVRGVAMNPVDHPHGGGEGKTSGGRHPVTPWGVATKGKKTRRRNKSSDKYIKQLKG"
                     /besthit="qcoverage 100 , hcoverage 100 , similarity 100
                     , identity 100 , evalue 9.52e-196 , alnlength 274 ,
                     Bacteria , Proteobacteria , WP_015589143.1 MULTISPECIES:
                     50S ribosomal protein L2 [Wolbachia]"
     gene            complement(17687..17974)
                     /locus_tag="wEsol_00769"
     CDS             complement(17687..17974)
                     /locus_tag="wEsol_00769"
                     /codon_start=1
```

```
                     /transl_table=11
                     /fullproduct="qcoverage 100 , hcoverage 100 , similarity
                     100 , identity 100 , evalue 1.90e-58 , alnlength 95 ,
                     Bacteria , Proteobacteria , WP_015589144.1 MULTISPECIES:
                     50S ribosomal protein L23 [Wolbachia]"
                     /cog="RplW COG0089 94 Ribosomal protein L23"
                     /pfam="Ribosomal_L23"
                     /product="50S ribosomal
                     protein L23"
                     /translation="MIKYNNIIKSPIITEKASFLREKFNKYSLYVFVNVNKRQIKLAI
                     ESLFDVKISSINVVRIKPKYRRFRGVIGCEKQKKKVYFSLMDGQKLDIMSV"
                     /besthit="qcoverage 100 , hcoverage 100 , similarity 100
                     , identity 100 , evalue 1.90e-58 , alnlength 95 ,
                     Bacteria , Proteobacteria , WP_015589144.1 MULTISPECIES:
                     50S ribosomal protein L23 [Wolbachia]"
     gene            complement(17971..18585)
                     /locus_tag="wEsol_00770"
     CDS             complement(17971..18585)
                     /locus_tag="wEsol_00770"
                     /codon_start=1
                     /transl_table=11
                     /fullproduct="qcoverage 100 , hcoverage 100 , similarity
                     100 , identity 100 , evalue 2.35e-143 , alnlength 204 ,
                     Bacteria , Proteobacteria , WP_015589145.1 MULTISPECIES:
                     50S ribosomal protein L4 [unclassified Wolbachia]"
                     /cog="RplD COG0088 214 Ribosomal protein L4"
                     /pfam="Ribosomal_L4"
                     /tigr="TIGR03953 rplD_bact 188 50S ribosomal protein L4"
                     /product="50S ribosomal
                     protein L4"
                     /translation="MECNLVNLSNDNVGTAQLNPLIFSAKQKLSILHDIVRWQLAKRR
                     AGTHKTKGISDVSGTTAKPYDQKRTGRARQGSLRSPQFRGGGIIFGPVVRSHTYSLNK
                     KVRKFGLKIALSLKYLNNQVIILDNLNIDVKKTSEMCKCIKNFKFSSFLIVGDYGDDL
                     LRAAKNLHYVDLIKPIGLNVFDILNHECVMLTKDTLKHLEGRLL"
                     /besthit="qcoverage 100 , hcoverage 100 , similarity 100
                     , identity 100 , evalue 2.35e-143 , alnlength 204 ,
                     Bacteria , Proteobacteria , WP_015589145.1 MULTISPECIES:
                     50S ribosomal protein L4 [unclassified Wolbachia]"
     gene            complement(18588..19310)
                     /locus_tag="wEsol_00771"
     CDS             complement(18588..19310)
                     /locus_tag="wEsol_00771"
                     /codon_start=1
                     /transl_table=11
                     /fullproduct="qcoverage 100 , hcoverage 100 , similarity
                     100 , identity 100 , evalue 4.68e-170 , alnlength 240 ,
                     Bacteria , Proteobacteria , WP_006279218.1 MULTISPECIES:
                     50S ribosomal protein L3 [Wolbachia]"
                     /cog="RplC COG0087 218 Ribosomal protein L3"
                     /pfam="Ribosomal_L3"
                     /tigr="TIGR03625 L3_bact 202 50S ribosomal protein L3"
                     /product="50S ribosomal
                     protein L3"
                     /translation="MKRINSLRRIGLLMMNIGHTAMYSDNSRMAVTLLHLSETYIIDI
                     KGQDKCGYNSVILGTGDFKNIAKPQLEYLKKKGINNKCKLYESRLNDLSGIECGKKVG
                     INHFVVGQYLDITGYSIGKGFAGVMKRHNFSGLRASHGVSIAHRSQGSTGQCQDPGRV
                     FKGKKMAGHLGNSRITAQNMKILSIDHENSIIAVKGNNVPGFKNSYVFVRDAVKKSLH
                     KDVPFPVGLLLDVNDDASNLVS"
                     /besthit="qcoverage 100 , hcoverage 100 , similarity 100
                     , identity 100 , evalue 4.68e-170 , alnlength 240 ,
```

```
                     Bacteria , Proteobacteria , WP_006279218.1 MULTISPECIES:
                     50S ribosomal protein L3 [Wolbachia]"
     gene            complement(19320..19640)
                     /locus_tag="wEsol_00772"
     CDS             complement(19320..19640)
                     /locus_tag="wEsol_00772"
                     /codon_start=1
                     /transl_table=11
                     /fullproduct="qcoverage 100 , hcoverage 100 , similarity
                     100 , identity 100 , evalue 7.02e-66 , alnlength 106 ,
                     Bacteria;Eukaryota , Proteobacteria;Arthropoda ,
                     WP_012673184.1 MULTISPECIES: 30S ribosomal protein S10
                     [Wolbachia]"
                     /cog="RpsJ COG0051 104 Ribosomal protein S10"
                     /pfam="Ribosomal_S10"
                     /tigr="TIGR01049 rpsJ_bact 99 ribosomal protein S10"
                     /product="30S ribosomal
                     protein S10"
                     /translation="MKQDIYINIKAFDCSLLEECVRKFVDELKRSSAKLSGPIALPRK
                     DSKFIVNRSPHVDKKSREQFEMRTSRRLIVLHDLTPTMMQMLTGLSFSAGVEVDLKVK
                     EVKV"
                     /besthit="qcoverage 100 , hcoverage 100 , similarity 100
                     , identity 100 , evalue 7.02e-66 , alnlength 106 ,
                     Bacteria;Eukaryota , Proteobacteria;Arthropoda ,
                     WP_012673184.1 MULTISPECIES: 30S ribosomal protein S10
                     [Wolbachia]"
     gene            complement(19655..20827)
                     /locus_tag="wEsol_00773"
     CDS             complement(19655..20827)
                     /locus_tag="wEsol_00773"
                     /codon_start=1
                     /transl_table=11
                     /fullproduct="qcoverage 100 , hcoverage 100 , similarity
                     100 , identity 99.7 , evalue 1.77e-279 , alnlength 390 ,
                     Bacteria , Proteobacteria , WP_141456826.1 elongation
                     factor Tu [Wolbachia endosymbiont of Carposina sasakii]"
                     /cog="TufB COG0050 394 GTPases - translation elongation
                     factors"
                     /pfam="GTP_EFTU MMR_HSR1 Miro GTP_EFTU_D2 GTP_EFTU_D3"
                     /tigr="TIGR00485 EF-Tu 394 translation elongation factor
                     Tu"
                     /product="elongation factor Tu"
                     /translation="MTAIVEAFGKPHVNVGTIGHVDHGKTTLTAAITKHYGNFVAYDQ
                     IDKAPEERKRGITIATAHVEYQTEKRHYAHVDCPGHADYVKNMIVGAAQMDAAILVVS
                     GVDGPMPQTREHILLAKQVGVGYIVVYINKADVADTDMIDLVEMEVRELLSKYGFPGD
                     EVPVVVGSALKALEDDSSEYGKKSIDKLMEKLDEYVAVPPRPVDLPFLLPIEDVFSIS
                     GRGTVVTGRIEKGEIKTGEEIEIIGLKATQKTICTGVEMFKKLLDKGSAGLNVGILLR
                     GTKREEVERGQVLAKPGTITPHRKFKAEVYILKKEEGGRHTPFFANYQPQFYLRTTDV
                     TGSIKLLDGKEMVMPGDNVSVEVELQVPIAMDKGLRFAIREGGRTVGSGVVSEILE"
                     /besthit="qcoverage 100 , hcoverage 100 , similarity 100
                     , identity 99.7 , evalue 1.77e-279 , alnlength 390 ,
                     Bacteria , Proteobacteria , WP_141456826.1 elongation
                     factor Tu [Wolbachia endosymbiont of Carposina sasakii]"
     gene            complement(21178..22269)
                     /locus_tag="wEsol_00774"
     CDS             complement(21178..22269)
                     /locus_tag="wEsol_00774"
                     /codon_start=1
                     /transl_table=11
                     /fullproduct="qcoverage 100 , hcoverage 100 , similarity
```

```
                     100 , identity 100 , evalue 6.50e-266 , alnlength 363 ,
                     Bacteria , Proteobacteria , WP_015589146.1 MULTISPECIES:
                     glutamine-hydrolyzing carbamoyl-phosphate synthase small
                     subunit [unclassified Wolbachia]"
                     /cog="CarA COG0505 368 Carbamoylphosphate synthase small
                     subunit"
                     /pfam="CPSase_sm_chain GATase Peptidase_C26"
                     /tigr="TIGR01368 CPSaseIIsmall 361 carbamoyl-phosphate
                     synthase, small subunit"
                     /product="
                     glutamine-hydrolyzing carbamoyl-phosphate synthase small
                     subunit"
                     /translation="MQDAVLILQDGKCFWGKSVGKKGKCIGEVCFTTGMTGYQHTITD
                     PSFADQIITFTFPHIGNVGINHKDNEGKKIFASGVVMRELSSMSHPSSYISLNDWLEK
                     NNVIGISGVDTRALTRHLREHGSQNGMICSSSKAHALDKLKEYKSVNGIEITNKVSLH
                     SNFKSDLNAKYRVVIVDFGVKISIVSRLIELGCTVELIRPSTGFAQKVLSMNPDGIVL
                     SNGPGDPQEIGESVVSEIDIIIKSKIPIFGICMGHQLLAITLGAKTIKMDIGHRGSNH
                     PVYDVISGKVEITSQNHGFVVDSASLLSNVEITHISLFDNSVEGIMMKDYPVFSVQYH
                     PEEAPGTHDSHYLFKRFIDNIVLYKMKSA"
                     /besthit="qcoverage 100 , hcoverage 100 , similarity 100
                     , identity 100 , evalue 6.50e-266 , alnlength 363 ,
                     Bacteria , Proteobacteria , WP_015589146.1 MULTISPECIES:
                     glutamine-hydrolyzing carbamoyl-phosphate synthase small
                     subunit [unclassified Wolbachia]"
     gene            22475..22969
                     /locus_tag="wEsol_00775"
     CDS             22475..22969
                     /locus_tag="wEsol_00775"
                     /codon_start=1
                     /transl_table=11
                     /pfam="CopD"
                     /fullproduct="qcoverage 100 , hcoverage 100 , similarity
                     100 , identity 100 , evalue 4.38e-99 , alnlength 164 ,
                     Bacteria , Proteobacteria , WP_015589147.1 MULTISPECIES:
                     hypothetical protein [unclassified Wolbachia]"
                     /translation="MNTGTNQSKKINEKWLAGGIGALLFVMMLPSILLLAKIAIGLIM
                     VSVAAIAIKVALKTISNALEDKQADQSQATQQTTQSKIINLAIGLTSIFVGGLLLFII
                     AQTGLIAACAAITALALHEYVKNQEIGKEINDKFDKVADDTINFIVSSVEKLIPTKPP
                     GITA"
                     /product="hypothetical protein"
                     /besthit="qcoverage 100 , hcoverage 100 , similarity 100
                     , identity 100 , evalue 4.38e-99 , alnlength 164 ,
                     Bacteria , Proteobacteria , WP_015589147.1 MULTISPECIES:
                     hypothetical protein [unclassified Wolbachia]"
     gene            complement(23390..24757)
                     /locus_tag="wEsol_00776"
     CDS             complement(23390..24757)
                     /locus_tag="wEsol_00776"
                     /codon_start=1
                     /transl_table=11
                     /fullproduct="qcoverage 100 , hcoverage 100 , similarity
                     98.9 , identity 98.5 , evalue 0.0 , alnlength 455 ,
                     Bacteria , Proteobacteria , WP_155968318.1 MULTISPECIES:
                     hypothetical protein [Wolbachia]"
                     /translation="MFSSIVNLLNGFFKNSSTKESEEKLTLLSPQANLQFDETQVQSV
                     LSDFEELEFKDNKCFDKIADDYEKKGESALFNYVILNKRKEIIDILDRNLPLLEEQEE
                     FRKKYGQYVLQIDKGMSYGKELQDGLRSFEDRAEKVLSDFERLNFDFKDNKCEDSSDS
                     VNYNNGWPADPFDKIANYYREDDIFLFNYVVLSKRKEIMGILDKKLTSKDQEELKENF
                     KKRLGRYLIRIGEGMSDGQEFLKTGSAIAFFDGCYVPCPYANRLLNHYDHLNINITKP
                     EQSLETSRLFTEKTKRMEEEKSEVKRAKIYSNGERIVTIEKNEKQQRNYSFTKFAICD
```

IEISWGAKDESGKDLNCTVVLNINLGQVSIKKSTIDGKDVEPKKILELAKQNEAVLIK
GKALHEVLAARFIKQTPCEEKKDMQSHENIVVPGSGAPRSINEEITVEHPFGKAANKD
NHREM"
/product="hypothetical protein"
/besthit="qcoverage 100 , hcoverage 100 , similarity 98.9
, identity 98.5 , evalue 0.0 , alnlength 455 , Bacteria ,
Proteobacteria , WP_155968318.1 MULTISPECIES:
hypothetical protein [Wolbachia]"
gene complement(24839..25756)
/locus_tag="wEsol_00777"
CDS complement(24839..25756)
/locus_tag="wEsol_00777"
/codon_start=1
/transl_table=11
/fullproduct="qcoverage 100 , hcoverage 100 , similarity
100 , identity 100 , evalue 2.97e-210 , alnlength 305 ,
Bacteria , Proteobacteria , WP_041573528.1 MULTISPECIES:
class II fructose-bisphosphatase [unclassified
Wolbachia]"
/cog="GlpX COG1494 332
Fructose-1,6-bisphosphatase/sedoheptulose
1,7-bisphosphatase and related proteins"
/pfam="FBPase_glpX"
/tigr="TIGR00330 glpX 334 fructose-1,6-bisphosphatase,
class II"
/product="class II
fructose-bisphosphatase"
/translation="MEDLAYKLVKVTEAAALAAYKLAGFGDEKKADQVAVDAMRTVLN
SMEINGTIVIGEGERDEAPMLYIGEKVGTGNGPEIDIAVDPLEGTTICAHYKPGAMSV
LAATKKGNFLHAPDVYMEKIAVGKNLPEGVVSLKNSIEKNLDNLAKAKKCKVNDLVVT
ILNRERHNELIVKIRKLGAKIKLIDDGDIVAVVSLVNGNHDMYIGIGGAPEGVLAAAA
LSSIGGQMEGRLIFDTDQLKERAKNLNIDDPEKVYAVKDMARGESVFIATGVTSGELV
DGVDNICSTSSLIIILPNNLIKLQTMQNLC"
/besthit="qcoverage 100 , hcoverage 100 , similarity 100
, identity 100 , evalue 2.97e-210 , alnlength 305 ,
Bacteria , Proteobacteria , WP_041573528.1 MULTISPECIES:
class II fructose-bisphosphatase [unclassified
Wolbachia]"
gene 25883..26593
/locus_tag="wEsol_00778"
CDS 25883..26593
/locus_tag="wEsol_00778"
/codon_start=1
/transl_table=11
/product="hypothetical protein"
/pfam="Ank_2 Ank_5"
/translation="MAYSDDDVRNFYLDIDRITGRDNTVTSLEELKKRLRKEVNFQDE
CEGGNSLGNLLLKHATENNNPRVKDFLLQNGCEPLQQEQITAAINKEEEAPNVEENRA
DSNEVDSSNFINPGMTAEVLYEQLIQGKETNLAIFDSFANAVRESNITGDRGIFLDVV
KLFTDLDAVIHLTDTASRTILGIATITKQADVVKVLLDSGKFYEEEKFNALCLAITQD
NAQEAELILGYVSPANRK"
/besthit="qcoverage 100 , hcoverage 53.0337078651685 ,
similarity 100 , identity 100 , evalue 5.58e-158 ,
alnlength 236 , Bacteria , Proteobacteria ,
WP_015589149.1 MULTISPECIES: hypothetical protein
[unclassified Wolbachia]"
gene 26603..27220
/locus_tag="wEsol_00779"
CDS 26603..27220
/locus_tag="wEsol_00779"

```
                     /codon_start=1
                     /transl_table=11
                     /pfam="Pfam-B_450"
                     /fullproduct="qcoverage 100 , hcoverage 94.9074074074074
                     , similarity 100 , identity 99.5 , evalue 1.54e-110 ,
                     alnlength 205 , Bacteria , Proteobacteria ,
                     WP_197907367.1 hypothetical protein [Wolbachia
                     endosymbiont of Kradibia gibbosae]"
                     /translation="MAIQVGKTEIVNAFLNRGKLNEENRAEPSSSNGNIGSRPVATVP
                     PVSTNGDNRNHDNEIPVVTETNQTTTPNSDNVNGQSNHNNKNKTPVIPEAKQTATPNN
                     DNKANGFVDAQFSVPNEKEAKYKKSKENFYTSLTKDVVGVVITGLFITAAVMVPFVTG
                     AVICGVIAALVAIATGLHVKNSTLPSYREMEENKVEHVQKTAKTL"
                     /product="hypothetical protein"
                     /besthit="qcoverage 100 , hcoverage 94.9074074074074 ,
                     similarity 100 , identity 99.5 , evalue 1.54e-110 ,
                     alnlength 205 , Bacteria , Proteobacteria ,
                     WP_197907367.1 hypothetical protein [Wolbachia
                     endosymbiont of Kradibia gibbosae]"
     gene            complement(27273..30047)
                     /locus_tag="wEsol_00780"
     CDS             complement(27273..30047)
                     /locus_tag="wEsol_00780"
                     /codon_start=1
                     /transl_table=11
                     /fullproduct="qcoverage 99.6753246753247 , hcoverage 100
                     , similarity 100 , identity 99.9 , evalue 0.0 , alnlength
                     921 , Bacteria , Proteobacteria , QDH18700.1 pyruvate,
                     phosphate dikinase [Wolbachia endosymbiont of Carposina
                     sasakii]"
                     /cog="PpsA COG0574 740 Phosphoenolpyruvate
                     synthase/pyruvate phosphate dikinase"
                     /pfam="PPDK_N Pfam-B_16844 PEP-utilizers PEP-utilizers_C"
                     /tigr="TIGR01828 pyru_phos_dikin 865 pyruvate, phosphate
                     dikinase"
                     /product="pyruvate, phosphate dikinase"
                     /translation="MYNVFISENPEKMMGEKLIHYFSQGKCEGNAEMKNLLGGKGANL
                     AEMCNVGIPVPPGFTISTSACKVYYQDNRSSVIQVSDYSDPEKNVVTHWNDICSGIKN
                     YMTMLENDIGCKFGDLNNPLLISIRSGSVSSMPGMLDTILNVGLNDETVVGLAKKSGE
                     RFAYDSYCRFIMMYSNVVLQLDHHLFQDVIDNEQQKSGAKSLADLDVDVLKRIVNDFK
                     KIVYEKTEKHFPQNVEEQLLNSVNAVFASWKNDRAVSYRKIHNIPENLGTAVNVQAMV
                     FGNLNDNSATGVIFTRNPSTGEKKLFGEFLVNAQGEDVVSGVYTPMPIDGEQKNTMEK
                     LLPSVYLELCAVCEKLERHYKDMQDIEFTVQDGKLWILQTRSGKRTAEAAIRIIVDMV
                     NEGTITKEEGILRIDPKTFDNLLHPVLDVKSDQKVIGKGLPASPGVASGYVVFSASDA
                     EKAAEQGKKVILVRSETSPEDINGMNAASGIVTARGGMTSHAAVVTRGMGKPCICSVS
                     GLYIDKDGTFFSVGDTKVNKGEPITINGGTGEVMLGILPTISPELSQEFKTIINWIDE
                     IKTVKVRANADTPKDAKIAKEFGAEGIGLCRTEHMFFASDRIEFIQKLIMADDENERA
                     NALIKLEEMQKSDFKEIFSIMEGREVTIRLLDPPLHEFLPNNQSTIEKIAKSLSKSVE
                     SVKNKIAQLSEKNPMLGHRGCRLAISHPEIYSMQIRAILSAASELKKEKKIEVEPEIM
                     VPFIMNEKEFVLICELVKKESSVISARIKKQIPASRAGMTSDKAYSIGTMIELPRAAL
                     IADKLAKHAEFFSFGTNDLTQTTMGLSRDDSVNFLDSYKESNIFENDPFEVLDIEGVG
                     ELIKMAIERGKKTRKEIKLGICGEHGADPKSIEFLIESGVDYVSCSPYRVPVAKLVAA
                     QFSIKSKCVG"
                     /besthit="qcoverage 99.6753246753247 , hcoverage 100 ,
                     similarity 100 , identity 99.9 , evalue 0.0 , alnlength
                     921 , Bacteria , Proteobacteria , QDH18700.1 pyruvate,
                     phosphate dikinase [Wolbachia endosymbiont of Carposina
                     sasakii]"
     gene            30219..31070
                     /locus_tag="wEsol_00781"
     CDS             30219..31070
```

```
                     /locus_tag="wEsol_00781"
                     /codon_start=1
                     /transl_table=11
                     /fullproduct="qcoverage 100 , hcoverage 100 , similarity
                     100 , identity 100 , evalue 3.17e-194 , alnlength 283 ,
                     Bacteria , Proteobacteria , WP_015589151.1 MULTISPECIES:
                     Rpn family recombination-promoting nuclease/putative
                     transposase [unclassified Wolbachia]"
                     /pfam="PDDEXK_2"
                     /tigr="TIGR01784 T_den_put_tspse 273 conserved
                     hypothetical protein"
                     /product="Rpn family
                     recombination-promoting nuclease/putative transposase"
                     /translation="MALSKFLDPKNDVAFRRIFGTEKNKDILIHFLNDILGFTGKDEI
                     KEIEFLSTIQDAEIASKKQSIVDVLCRDENGVQVIVEMQVAKTKGFEKRAQYYAAKAY
                     SRQADKGDQYQGLKEIIFIAIADCILFPNKSEYKSDHVMLDKDSYEHDLKDFYFTFIE
                     LPKFPKTKEDQLESIVEKWIYYFKYADETSEEELEKIIGSDVIIKKAYEELNRFNWSE
                     KEFIAYEQEIKRIRDERAVLEQKLDDAKKEGKIEVAKTMLANNVDVTTIVKFTGLSMS
                     EIKELQN"
                     /besthit="qcoverage 100 , hcoverage 100 , similarity 100
                     , identity 100 , evalue 3.17e-194 , alnlength 283 ,
                     Bacteria , Proteobacteria , WP_015589151.1 MULTISPECIES:
                     Rpn family recombination-promoting nuclease/putative
                     transposase [unclassified Wolbachia]"
     gene            complement(31177..32532)
                     /locus_tag="wEsol_00782"
     CDS             complement(31177..32532)
                     /locus_tag="wEsol_00782"
                     /codon_start=1
                     /transl_table=11
                     /fullproduct="qcoverage 100 , hcoverage 100 , similarity
                     100 , identity 100 , evalue 0.0 , alnlength 451 ,
                     Bacteria , Proteobacteria , WP_227738550.1
                     phosphomannomutase/phosphoglucomutase [Wolbachia
                     endosymbiont of Drosophila simulans]"
                     /cog="{ManB} COG1109 464 Phosphomannomutase"
                     /pfam="PGM_PMM_I PGM_PMM_II PGM_PMM_III PGM_PMM_IV"
                     /tigr="TIGR03990 Arch_GlmM 444 phosphoglucosamine mutase"
                     /product="WP_227738550.1
                     phosphomannomutase/phosphoglucomutase"
                     /translation="MDNTIIRKYDIRGIVGKDLQINDGYEIGRKFGQTVASVCVGYDS
                     RIDSPGIERELIRGLTLSGANVIRVGLCSSPMLYAATQITQADLGIIITASHNPSEYN
                     GFKFFSNKKVFSDQEMKEIISSPIKNSTRIGSLININIYSEYISILKSAVKNNTTQKL
                     KIAWDCGNSPASGIIRYIEKILPGHTHIVTNNSIDGAFPLHDPDPIEEKNLAQLIDIV
                     KEYGCDLGIALDGDSDRVRLIDNKGNVVSNDHLFIIFAREVLEEYPESKVIANVKMSM
                     KVHDFVSKLGGQVITCATGHSLVKKKMVEEEAKFAGELSGHFFFSELSFDDGLYSAVK
                     AVDILLKKNQSLSQVIEDLPKLYITHEVKIVVKDEKKFQIIESIKKTLEQQNIVFSDL
                     DGVKVTDNKGWWLLRVSNTQNCITARCEGETLEGFELTKKVLFHYIDEARSLNCSYSQ
                     K"
                     /besthit="qcoverage 100 , hcoverage 100 , similarity 100
                     , identity 100 , evalue 0.0 , alnlength 451 , Bacteria ,
                     Proteobacteria , WP_227738550.1
                     phosphomannomutase/phosphoglucomutase [Wolbachia
                     endosymbiont of Drosophila simulans]"
     gene            32660..33868
                     /locus_tag="wEsol_00783"
     CDS             32660..33868
                     /locus_tag="wEsol_00783"
                     /codon_start=1
                     /transl_table=11
```

```
                     /product="hypothetical protein"
                     /pfam="Pfam-B_13991 AAA_13 Mod_r"
                     /translation="MPQENENVFIAESQSLDVDEQQKKDILDVIESCSSFQETIKQLE
                     EKNLIWYFIGPLRKDEDITGQINLKWEKEFQKFEQSINETHREFEASLPNQNFLAYST
                     KDDDFKIRFENEEKPIEISNILRASSEGKVYNISLGKDSQITATKKGSERHYDFTGSS
                     SCEMTINWQAKDSEGNSIDCSMTVKLDSGGIQSVIGEPKFGGITSSEEVLKLVGQNKE
                     VFINRKTLYQAFTDIGQDMDSLEPTQDLSSQLPTPSSSGYSSPSTPSSRRSSISTSSE
                     FSDEDEIFEDDMSGDEEEYEQEGLKDKVDEIINEIEELKEGKETFAQHFAAQAKQLED
                     LRLERDSLRDKVNEQQRAIFERGQRIKEQEEELQDKNDVIKNLNNQLKDKEGETNEAN
                     KELQRKVKLI"
                     /besthit="qcoverage 89.5522388059701 , hcoverage
                     68.5714285714286 , similarity 100 , identity 100 , evalue
                     8.27e-238 , alnlength 360 , Bacteria , Proteobacteria ,
                     WP_041573599.1 hypothetical protein, partial [Wolbachia
                     endosymbiont of Drosophila simulans]"
     gene            33895..34731
                     /locus_tag="wEsol_00784"
     CDS             33895..34731
                     /locus_tag="wEsol_00784"
                     /codon_start=1
                     /transl_table=11
                     /product="hypothetical protein"
                     /pfam="Herpes_BLRF2"
                     /tigr="TIGR02168 SMC_prok_B 1181 chromosome segregation
                     protein SMC"
                     /translation="MENDLKKIVEGLKDDHKKQLAEQLMETKKENNTLAETASQLSEE
                     LNELQREKEDLEREIRQLEPGRSLADELSEENTKGEIEKLQYQLKEKEGMLTCTQEEL
                     ASVKESLEQLQNILQQEKGQATQTEVEYESRGTSMQQQDQGIQAVNNNSSRTASGLRQ
                     ENKKLTRDKLTISEQLKQKIREIDELKKNLKQLQEENKQLKQEVTKNAGVDASSSTGQ
                     KEAKEAEVIPQNIMDWPVPIPDDKPEEQKTPLEPIKVNTELSDPKSIPAEGPEKSPPL
                     SK"
                     /besthit="qcoverage 100 , hcoverage 60.5664488017429 ,
                     similarity 97.5 , identity 96.8 , evalue 5.00e-112 ,
                     alnlength 278 , Eukaryota , Arthropoda , GFY79758.1
                     uncharacterized protein TNIN_54101, partial
                     [Trichonephila inaurata madagascariensis]"
     gene            35253..35504
                     /locus_tag="wEsol_00785"
     CDS             35253..35504
                     /locus_tag="wEsol_00785"
                     /codon_start=1
                     /transl_table=11
                     /pfam="DEDD_Tnp_IS110"
                     /fullproduct="qcoverage 100 , hcoverage 100 , similarity
                     100 , identity 100 , evalue 9.78e-51 , alnlength 83 ,
                     Bacteria , Proteobacteria , WP_051062211.1 MULTISPECIES:
                     hypothetical protein [unclassified Wolbachia]"
                     /translation="MCKELRSFGLPAICVDARHMAAALSARINKNDKNDARGIAQMMR
                     SVSKISCQIKIALGSRRQLMCSKQQVIGTIRGLLKIHGR"
                     /product="hypothetical protein"
                     /besthit="qcoverage 100 , hcoverage 100 , similarity 100
                     , identity 100 , evalue 9.78e-51 , alnlength 83 ,
                     Bacteria , Proteobacteria , WP_051062211.1 MULTISPECIES:
                     hypothetical protein [unclassified Wolbachia]"
     gene            35494..>35791
                     /locus_tag="wEsol_00786"
     CDS             35494..>35791
                     /locus_tag="wEsol_00786"
                     /codon_start=1
                     /transl_table=11
```

```
                     /pfam="Transposase_20"
                     /fullproduct="qcoverage 100 , hcoverage 81.1475409836066
                     , similarity 100 , identity 100 , evalue 8.11e-62 ,
                     alnlength 99 , Bacteria , Proteobacteria , WP_174516888.1
                     transposase, partial [Wolbachia endosymbiont of
                     Cardiocondyla obscurior]"
                     /translation="MVGKGSKFETFALRIQEAINNLDEISRSSIEALVCSLEAIEKSL
                     RKLDKMLSEQGEKDDDCKLLTTVPGVGIIVAMTYKAAIDNPHRFEKSDTVGAYIG"
                     /product="transposase, partial"
                     /besthit="qcoverage 100 , hcoverage 81.1475409836066 ,
                     similarity 100 , identity 100 , evalue 8.11e-62 ,
                     alnlength 99 , Bacteria , Proteobacteria , WP_174516888.1
                     transposase, partial [Wolbachia endosymbiont of
                     Cardiocondyla obscurior]"
     gene            complement(20981..21063)
                     /locus_tag="wEsol_tRNA-15"
     tRNA            complement(20981..21063)
                     /locus_tag="wEsol_tRNA-15"
                     /gene="tRNA-Tyr"
                     /intronpos="0..0"
                     /program="tRNAscan-SE"
                     /cove_score="69.2"
                     /evidence=predicted
                     /type="Tyr"
                     /anticodon=GTA
                     /product="tRNA-Tyr (GTA)"
     gene            complement(20906..20976)
                     /locus_tag="wEsol_tRNA-16"
     tRNA            complement(20906..20976)
                     /locus_tag="wEsol_tRNA-16"
                     /gene="tRNA-Gly"
                     /intronpos="0..0"
                     /program="tRNAscan-SE"
                     /cove_score="76.5"
                     /evidence=predicted
                     /type="Gly"
                     /anticodon=TCC
                     /product="tRNA-Gly (TCC)"
ORIGIN
        1 taacacacaa aaacaaagaa tctataattt aagcattcca tttagactgt agagcaccag
       61 aataaactat ttagtaacaa ttcctgcatc aactatgttt ttaaaatttt ttgacgttta
      121 actttattat aactttttct ttattttgtg aatatctgag ttacttatat tatgtaagaa
      181 atctattgcc aactagtcaa tattctgtta ggaattaaga taatataaag ttattatgct
      241 taaattggaa agcagaaagt tcctgattac tggcgcatca ggcggaatag ggcaagcaat
      301 tgtaaaaatt atgcacaaag ccggagcaac tttatgtatt tctggcacaa aaaaagaagt
      361 gctcgaagaa gttgctaaac aatatgaaaa aaacatgcat gtactccctt gtgatttatc
      421 aaatgctgag gaagtaaatc aactaataaa tagagcaagt gagctgatgg aaggctttga
      481 cgggctcgta tgcaatgcgg gtattacgcg agacagttta ttattgagaa tgacagatga
      541 agcatggcag aaagtaattg atattaactt gagctctaca tttaagctga gtagagaagc
      601 atgcagaaaa ctaattaaga ataattgggg aagaattata aatatttcct caataatagg
      661 gctgacagga aacgccgggc aagcaaatta tgcggcgtct aaagctggaa taatagctat
      721 gagcaaatcc atagcaaaag aagttgcaag tcgtaatata acagtaaatt gtattgctcc
      781 tgggtttata gatactaaaa tgactgaagt tttaaatgaa ggacaaaagg gaaaaatatt
      841 agataatatt ccaatgaaaa gaatgggaac aggggaagaa atagcagcag ggtcttgtt
      901 tttagcaagt gatgaagcaa aatatataac agggcatgtg cttaacatta atggtgggtt
      961 atttatgtag caacacttat gtatggacaa attataattt ttactttaaa ataaaaatat
     1021 gtgtaggttt aggtttgcgt taatttatta tttagaggca aaaaaatgag ctctataata
     1081 ggattttttg gtgttttgtg tgtagtattg cttttctctt tctcaggtta tgcagataac
     1141 cctggtgttg atatggatga cacaactaaa gtaatatgca atattattgg ttatgtttgg
     1201 gggataggcg gtccacttat gacggtagta ataattggtg cagctttgct tgcaatattt
```

1261 ggcaggatgc cgtggccagc cctctttgct cttggcatgt tctgtggtgt atttttttggc
1321 gctaaaacta tcataacgaa aattatgccc aacgtaagcg atgaagcaaa aactatgttg
1381 gaaaagtgtg gacaaaaata atccaaaaag tgcagaaatg cttttttgtt atccattaaa
1441 ccgcttcata tcttcaactt gcaacaaata agggctatct tttggtagag ttttaacagc
1501 taactcagcg tatttcataa attgctttaa atcaccttca atacaagcct tttttgtcaa
1561 ggcaaaataa tgcattgccg tatcagcttc acatttatat gcaatgctta aatatttcca
1621 gacaaaagcg ttatttggtt ctacattcaa aatctgctcc aggtaaaaaa tcgccttttt
1681 tgcatcaccg tgtaataata aagtatgaga taatgcaagt ttcactaaat aactgttttt
1741 ctcagataaa tacctaagcg attcctcata cattttttatt gcttcactta aatttccggc
1801 cttgtatagc atttctgctt ttaattcata taggtatgga tcactgtgtg attcttgaat
1861 caatgagtta actttagcaa tagcctcttc tatctttcct tgcctatagc aaactatagc
1921 atttacatac tcagaattat cttcatattt attagataac acatgaatag gagcaaagaa
1981 agagtctagc tttgcaacca cacgctcaaa ctttagtaac ttatccgcaa aaattggttt
2041 tacgttgttt ttaaccttat aattttgtac agcaaatatg cgtttctcac taagtgggtg
2101 agtgcggaaa tattcctcgg tgttctcatg ctcaatactt ttaaaataat caaaaatctc
2161 tttcatacct gaattatcat agccagattc atcaagatac cttaaagcat agctatctgc
2221 tacactttct tgctcttgag aatagttaaa aaatagcctt gaactaaacg ctacaccact
2281 aagcaaaatt gcaccagcaa cctgagggtt aatgataata ctagaaacta atcctaccat
2341 ataactaatc attgctatcg attgaaaata acccatagca ctactcattt gcaatatatg
2401 accagcagat atgtgagcaa tctcatgtgc taatatacca agcaggacat aaggtttagc
2461 cgaatattgt aaaagtccta aatgaatgaa aatactgtta ttgttaatta caaaagcatt
2521 aatcgaattg tcattaatta taaaaacttt tatttgctca tgatcaatat ccgcagcaga
2581 aaataaaggt tgcgctagct ctttcactat tgcttccact tcactatctc taataatgtt
2641 aatagagtaa gcattattac aatatgcaaa aaagaacaat aaggtcaaaa acttagcaac
2701 tctaaacatg aaaattctgt gcttgtataa tttatagaaa tcctatattt taacacttaa
2761 attatacaaa tttataaacg ctagtttaaa tttttaacgt aactgataag tttgtcgatg
2821 gtagttaact taattttatg ttgttcagca aatttaagta actgaggtaa gcgcatcata
2881 gagccatcat cattcactaa ttcacaaata actgctgaat gatcaaaccc tactaacttc
2941 gccatttcaa cactcgcttc agtatgacca gcacgttgtg ccaccccacc tttatgtgca
3001 attacaggaa aaacatgacc tggagttatg atgtcatcct gagtaccatc cttgttgata
3061 gcagcatgta tagttttcgt tctatcctct gctgaaacac cggttgtaat gccataacgt
3121 gcatcaattg atgtagtaaa tgcggtatga ttttcaccaa tatttttctt ttccatgaaa
3181 ttaaggccca accttttcat gtgaaattta gtcattgcta agcaaacaac ccctgtaccg
3241 tacctaacca taaaagccat atgttccggc tttattttct cagctaagac aatcaaatcg
3301 ccttcatttt ctctattttc atcatcaact aaaatgaata atttaccgga acgagcatcc
3361 tctaatacat cttccacaga agaaatacct ggtagactca ttgaagcata agtggcctgt
3421 accataataa tttacttaaa ttcaatctta actattttat acaatttctc tccatttggc
3481 actgtcactt ccacatattc accgactttt ttgccgatta aggcactccc aagtggtgaa
3541 ctagtagata ttaactgctt tgaaacatca gcttcatatt cgcctacaac cttataaaca
3601 tattccactt caccgttatt atcacttaac atgcttaatg tcacagttgc accaaacatt
3661 actgaatcac cagacaaatt ttttacttct attacttccg catgtgagag cttactttct
3721 atctccatta tacggccttc aataaaacct aacctttccc gtgctgcatg atactctgcg
3781 ttttcagaca aatcaccttg atcacgagca tcagaaatag cctgtatcac agaaggtttt
3841 tcttccttta atttttcaag ttcggcttgc atattctcaa aaccttccct cgtcatagga
3901 aatttattaa taatggatga agccataatc accacctaaa cgcaattaaa ttgagtaacg
3961 aaattactca caatgttaaa aatatcacct tttatatcat cactcatgtc atttgacaac
4021 tttttttaaaa ccccagggtg tacagtatga aaataattaa taagatcatg ttcaaattta
4081 ttaatgtaac ttatttgtat tttacttaac tgattatata ggttagaaaa aatatacatc
4141 agcactactt gctcttctat tgacataggt aaatgttgtt tttgctttaa taactcgaca
4201 agatatttac ccttattcaa ggataattga acactagcat caagatcaga accaaatttt
4261 gcaaaatctt ctaattctct atactgagct aagcttagct ttatagaacc agcaactttc
4321 ttcacagact ttagttgtgc agcagaacca acccgtgaaa ctgacaaacc tatatttact
4381 gcaggtcgaa atcctttata aaataattca gactcaagaa agatctgccc atcggtaatt
4441 gaaataacat ttgttggcac atatgcagat acatcaccag cttgagtttc gacaattggc
4501 aaagcagtta aagaaccctg cccttttttta tcagacattt tagcagctct ttcaagcaag
4561 cgagaatgta catagaatat atctccaggg taagcttcac gaccaggagg acgtctgagc
4621 aataaagaca tctgcctata tgccacagca tgcttagata aatcatcata tataatcaaa
4681 caatgcattc cattgtcacg gaaaaattcc ccaatagtgc aaccagcata aggtgctaaa
4741 aattgcatag gcgcgcagtc agatgcacta gccacaacta cagttgtata ctctaatgct
4801 ccgctttctt tcagcttatt taccactttt gctactgttg aaattttttg cccaatagca

4861 acataaacac agtaaacttt ttgattttcg tttacctcat cgttaatctt cttctgattg
4921 ataatagtat caagcgcaat agtagtttta ccaatttgtc tatcaccaat aattaattca
4981 cgctgccctc tacctattgg aattagcaaa tctataattt taattcctgt ttgcagtggc
5041 tcatgcacag acttacgatc aataatgcct ggcgctttag attctatatc cattctgttt
5101 ttggctctaa tttccccacc gtcgtccata ggatggccca acgcatttac aactctccct
5161 aacaattcat gccctacagg cacctgcaca acatcaccac tacattttac aacgtcccct
5221 tctttcacat cacggtcatt accaagcaca actattccag ccgtatcatg atctaaatcg
5281 agaactattc cttctacacc acttgcaaaa aatacctttt caccgaattt tgctttttcc
5341 agcccataaa ccaatgcgat accatctgtt actgaaatta cttcacctat attttcccgc
5401 tttataggat tatcaaacgt ctcaaccttt tctcttatta tacttgctat ctcagaaaca
5461 ttcatactgt tcttcataca aaattcctta cttttaatat ttccatttta cttaagtcaa
5521 ctaacctatc caagtaactt tttagcgaag catcgatcaa attaaaacca tacctaacta
5581 cgaagccacc tagtatagaa gggtcaacca cattgcttac ttttattatt ttaccaagaa
5641 agctcaaaga ttcagtaatt atttttatat cagattcctt taaagtttct gctgacttta
5701 tagtaatttc gaattcattt tcattttctc ttgcaagact taagaatttt tctaatataa
5761 ggatcaataa actagagcgt ttatttgcaa atataaccat aataaatttt actaaattct
5821 cactcaagtg ctcatttata gaaagcatca cttctttttt gtgtgcaaaa gaaatcatag
5881 gatgagatag gtacacaaaa acatcacgtt gatccttaaa aaaagccaac aaaaattcta
5941 cttctttcct tataatacct aacctgcttc ctgagacatg aaatagcact ctagcgtaag
6001 atgaaactaa attattgtat tgggtctttt tcatattaaa tccagtaaat ctaaattaat
6061 tcagctacaa atctttcatt cttcaatcca agcatttttt atataaacag gtcttctctt
6121 taaggaaaga aaatataaat catatctgac tgaataatct aaaaaactaa ggtttttact
6181 taagaaatac ttagaagaat ttataataga ctgacattga agatgagata tcggtatttc
6241 ttttccaagt aaacttgttt taacttctat aaaaatcagc tccttttttt tagatacaat
6301 taagtcaatt tcaccgaact tacaacggta acggcgtttt ataacattat accatttcag
6361 ttttaaatat actaaaacta ataactcgcc aaagtaacct acaaagtagc gtaacctact
6421 taccacaaga agaacaatta tgataacaat taactttgtg taactacttt gcctccttct
6481 ttatttttac tataaacctt acctcttgct gcaatatccc tatcaaccag ttctatcact
6541 gccattgaag cgcaatcacc ctttcgagtg ctaaatttta ttattctaga atacccacct
6601 ttacgatctt gataacggct ggctaagacg tttaataact tatcaactgc taacttacta
6661 ttatgaaggc gtgaaagcaa aagtcttcta ccatgtaaag tattcttatt cttagcaatt
6721 gtaataaact tttcgacata tggacgaagt tccttagctt ttggtaaagt agttacaatc
6781 tgctcatggt taattaatga gatagataga ttctttaacg tcgataacct atgttcagta
6841 caacgagata gtttacgttt ttttattcca tgtttcatat atcacctaat cttcatcagt
6901 atgttgttta gccaactcat ctatatcctt aggtggccag tttagcacat tcatacccaa
6961 agacaagcca aaattagtca aaactgcctt aatctcattc aaagactttc tgccaaaatt
7021 agcagttctt aacatttcac cttctgtctt ttgcacaaga tcacctatat aagtgatgtt
7081 ttcgtttttc agacagttat gagacctgac agataattcc atttcatcta ccttacgcaa
7141 taagataggg tcataaccca aatctttagc accactagac gaagaaacct gcgatttttt
7201 ataacttaca tcagaactaa taaagggctg aagttgctcc tgtaatattc ttgcagcaga
7261 gtcaacagct tgactcggag aaattgtacc atcagtttca attgacaata tcagcttatc
7321 cttatcagtc acttggccaa cacgactgtt ctctaccctg tacgaaaccc tgttgacagg
7381 gctatacaaa gcattaactg gaataaaacc aattaaatcc tgttcactca taagcttcaa
7441 aagttcattt tctttatatt tagttacagg aagataacct tttccgctag ccacatatat
7501 ggtcatgtta agctccacat tttgccccag cgtacatatt aacaaatctt tattaacaat
7561 agagcattga tcatcagttt ctatcatccc agctaatacc tggcaaggcc ctttagcgtt
7621 taaatttaag catttattag aagcaccatt taatttacac ctcaacatac ccatatttaa
7681 tactatatca gttacgtctt ctctcacccc ttgaattgaa gtgaactcat gagttacacc
7741 ttcaattttt attccataaa cagcactacc tttaagagaa gataacatta cacgtcttaa
7801 tgcattacct aatgttaaag caaaaccact ttccaacggt tctagcacta tatcactttt
7861 ttcacttgaa tcatctgata ttaccttaac tgaactaggt ttaatcaatt tatctaaatt
7921 gctacagaga gaaacatcat tattataata cataaaacaa ccttttcaaa tatcctatac
7981 tcttcttttt tttcttaacc tgcacccatt atgaggaatt gcggttttat ctgcaattga
8041 agtcacagtc aacccacagc tctgaagtgc tttaaccgca gcttcggtac caaagccagg
8101 accgcgaatt attacagaga caaccttcat accaaatctc tccattgcgt tcttcgcagc
8161 agactccgca gccttacctg cagcataagg tgtggatttt ctcgaacctg aaaaaccaca
8221 tgcacccaca gaagtttgac atagcgtatt accatgaaca tcagttacat ttacaaaagt
8281 attattaaaa gttgcacgaa tatggacaac accagtaata aactccttct tactcttact
8341 aaccgttttg actttttca ttaaaactca caataataat aaattttatt ttttcccagc
8401 aataggcaac cgagatctac ctttacgagt cttagcatta gtatgagtcc tttgccctct

8461 cacaggtaaa ccttttctat gtctcacccc tctatagcat cccatttcca ctaaagattt
8521 tatgttcata gccacttctt ttctgagctc accttctata acataatttt gtctaatgaa
8581 accgctgatc ttttctatat cttcatctcg taattctgaa acacgtttgc gcttatcaac
8641 ttcgcaagcg tgacaaatta cattcgcagt agcaatacct ataccatata tataagttaa
8701 tgcaaaagga acacatttcc tcactggaat atttatgcct gcaatacgtg ccactggtac
8761 ctcgatactt tattaataac aactcttaat ttataccata aaacaactca gagtcaaacc
8821 aaattacaag aaattttact ttcaatctct tgcgttacct catcaacact caaattagcg
8881 tcaattgtta ataatttgcc tttataatac tcgcgtaaac ctttcatttg aaggtgatat
8941 tcacttattc tcttattaat tgcagacata tcagcatcat caattctttt ctctaacctc
9001 gtacttttac atttagcaca agtagtactc ttaaaagaag atacactata tatgctttta
9061 cagtccaaac acgcgagacg atttttaac ctatcgattg caatattatc gtcaagctgc
9121 agctcaacta caatgtcaac atccctatta tatttttctt gcaaaacttg agtcaaaaaa
9181 tgagcctggt ttaaatttct cggaaaacca tctaataagc aattatcatc cactaatgca
9241 agctggtcac gtaataatcc acatataatt tcatcttgaa ttaaattacc agattccacg
9301 gtacccttta tttttttacc taactcacta ctgctagata taatattcct taataaatcc
9361 cctactgaaa ttaattttaa attatatttt gctattaaca agcttgactg agtaccttta
9421 ccagaacctg gaggaccaaa aatcgtaatg atcatcttac tttcctcgtt ttagactcat
9481 atttctttat ccagttgtcg tacctatttg aaaaaatata agattgtatt tgcataatag
9541 tatcagtaat aacgttcact ataatcagta agctcgtccc accaaaaata aatggtatat
9601 cataataata tctcataact tcaggtacag tacatatgac taccaaatac gcagagccaa
9661 tgaatgtcaa cttaaaaact atatcttgaa ggtaatcaga agtatgtttc ccaggccttc
9721 taccaggtat aaaaccacca ttttttttaa gaaaatctgc gttttcctct ggattaaata
9781 taaaattggt atagaaaaaa ttaaaaaata ctatcagcac caaataggct acaatatata
9841 ctactttatt tgccataaag taattcaaaa taaaatcaga aaaagcgtgc cccttataga
9901 aatttgcaat tgaaataggt gtcaataaaa ttgcattagc aaaaatggtt ggtactacac
9961 cagataaatt aatcttcaat ggaatataag taaaatcgtc attatgtaat cttttaaatt
10021 gctttttagg atgttgaaca ataacctttc tataagaaga ctctacaaaa atgactaaga
10081 gtagcagcaa aaaaaacaat accaaaacaa aaaggataat aaataacgat acgctaccgt
10141 ttttatttaa cgttaacaaa gatgacaaag cgctgtgtaa ctctgatatt atacccgtga
10201 aaatgattaa tgagatacca ttgcctatac cgctagcgct gattcgttca ccaagccata
10261 ttaaaagcat agttccacct aaaaggctga aaacacctat tgtacgaaac ataacaccag
10321 gttcgattac aaccaatacc ccttccctat tcatcccttc caatccaacc aaaattggaa
10381 ttgattgaaa tacacaaatt actatggtca gatagcgtat ataagaattc atctttctac
10441 ggcctgactc tccatcattc ttaacttcct tcattccttt aatcgcagaa gaaagtaact
10501 gaataatgat agaagccatt atatatggca taacgttcaa tgctagaatt gtcattctag
10561 ccaatgcacc accagaaaat aaattaaata ctccaaaaac gcctgagcca tctttgggaa
10621 atatatcatt aattacatca agattaattc caggaatagg cacataagta cccaagcgat
10681 agcaaattaa agctattagc gtaaaaaata tacgctttaa taaatcgcct ttatataaca
10741 acgtaagatc caagctatta aatgctgatt tattgcccat gacttacgat agtatttcca
10801 cactaccaca aactgaagct acagattttt ttgcagcttc tgacgcaaaa tcaacatgaa
10861 acacaaattt ttcgctcaac ttacctttat taagaagttt aattttatcc ttaatagact
10921 ttataaaacc taatttatac agcctctcct tatctataac agagtctttc actatttttt
10981 tagcttccat taagcgctgt atatcgccaa cattaattat agagtatatg ctcctacgta
11041 taggcttaaa acctctttta ggcaaacgag tatatataga ctgttgtcca ccctcaaaac
11101 catttataga aactccactt ctagccttct gccctttatg ccctctaccg gatgttttac
11161 ctttaccaca accgatacct ctacccagca acttaggctt ctttttctta gataatttag
11221 taaatataga gtttaatttt acagcattat tcatacttta cctgtttcca actatctcgc
11281 taatcttttt gcctcttttg cttgctacct gacgaggaga taacatacta tcaaaagcct
11341 taaacaccgc acatataacg ttatgaggat tatttgatct agtggattta gctaccacat
11401 cctttatacc taacacctca aaaactgatc taatcgctcc accagcaata atacctgttc
11461 cggctcttgc ggttcttaaa actatctcac cagaacaaaa tttagcttta atatcatgat
11521 gcaaagttct accttcacgc aagtacactc taatcattga tttctttgca gcatttacag
11581 ctttgactct tgcttcagca acttctgcgt gcttacctat tccacaccct accctaccct
11641 tctcatcgcc aacaacaacc aaaattgaaa atgaaaatct tctaccacct ttggtaaccg
11701 ttgtcactct tcgtaccgaa actaaaagct ctgataaatc attattattt tgtaaattct
11761 ttatagccat attcaaaacc ttctaaaatt caaatccaga acttcttaaa gcctcagcaa
11821 attgagaaac taatcctgtg tatttatacg ctccacgatc aaacgtaagc tgctgctcta
11881 atttcatgcc agacagacgc tcaaccatta aagaagaaac ctgttttata gtttcagcgt
11941 tgactttccc tttacataca tccttaattt tagcatccaa agtagaagct gaagtgagag
12001 ttattccttt tacatcatta attaactgga cataaaaatg cctattagat ttaaatatgg

12061 atatacgtaa acgttcagcg ctcttgtcaa gcttcgctct attacgtaac ttcctttttt
12121 cagaattact taaaaaatta tacgatcttt tcatttttat tacttctttt tacttacaac
12181 cttacgcagc ataaatttgc cttttattac aacaccctta cctttataag gatcatattt
12241 tctaattttg catatatcag acgccaccat atagaccttc tgcttatcca taccactaac
12301 tactaagtga gttggcttta tgcacttaat ctcaacatct ttaggcactt tatatttaac
12361 attatggcta taaccaagat ataaagtcaa atacttacca tcacactctg ccttatatcc
12421 cacaccgtta atctcaagat taacagaaaa accatcaacc atgccgttaa tgatattatt
12481 aatattactc ctataagtgc cccacatagg ctttatttta tcataattat ctttatcttg
12541 atcaacagaa agcaatagct gattatcaat aatctgacac acaacatccc tgatcaagct
12601 aacttccttt tcagccttag cgctctttat taacatacta ccattattat attcaactga
12661 aacaccagca ggaatattga taggagcagc acctatacga gacatgaatt tctcctatta
12721 tttaaaacac acgacacaaa acttctccac caacctttaa cttatgtgca ttataatcag
12781 tcatcactcc tttgggtgtc gatataataa aaataccaag accattatat gctttagaaa
12841 tgtccttata cttagaataa cgacgacaac caggctttga caccctaact atgtcactaa
12901 ttacaggtga cttctcataa tacttcaatt ttacaatgaa agaaggtaaa ttatctacaa
12961 cttgcttttc ataatcaagg atataccctt cctcttttaa aatctccaat atagaagaat
13021 tcatcttaga aaatagtacc cttgttgccc tatgcattgc caattgagca ttacgtatcc
13081 ttgttaaaaa atcaccaata ctatcagata acgccacaat atactcctta tattatattt
13141 accaactaga ctttgtaact cctggaattt gcccaaaaga acataaatca cgcaaaacaa
13201 ttctacataa accaaatttt ctatatactc ctcttggtct cccagttaaa gcacacctat
13261 ttctaatttt ggttttagaa gaatttctag gcaatttttt aattaattta ttttgagctg
13321 taaacctttc tacaatagac aaattcttat tatttattat agatttcaat ttttctcttt
13381 tttctttata ttgatcgcac aactttattc tgcgaagatt tctttctatc atagattttt
13441 ttgccatata tttctcctct caattaatca aaaaaaggaa atttaagagc cagtaataat
13501 tcctttgctt ccttatcact aactgcactt gttataatat taatatccat acctctaatt
13561 ttacttattt tatcatagtc tatttctaaa aacgagatat gttcctttat accaaaggaa
13621 aaattaccat taccgtcaaa ttgcttcaca ctaaatcctc taaaatcttt ttcccttggc
13681 aaagcaatat atatcaatct ttctaaaaac tcatacattt tatctctacg caaagttact
13741 ttgcaaccta ctgtagcacc ttttctgatc ttgaagccag aaatagattt ttttgcaaaa
13801 gttgataaag gcttctgccc agcaatcaaa tataggctat caagtggttc atttattgct
13861 ttactatctg tagcagcatc tccaacgccc atattgatac acaccttgac aagtttaggc
13921 acttgcatta tattgccgta attaaactta tccttcaagg actttactat gctatcttta
13981 tacaattctt taaacatatc aacctaatct atcacttctc cagaaacttt tgcaaaacgc
14041 acttttctac cgtctataac cttaaacccc actctagttg gagttttata tttagggtcc
14101 aatgttgcaa cgttggatat atcaatagct aactctttat ttaatatacc accactacta
14161 ccagcttttg gtttagtatg tctcttatgc acattcactc cagaaacaat tactttcttt
14221 ttagcatcac gtgctataac tttgattacc ttaccaattt ttcccttatc tttaccagtt
14281 aaaactataa cgtcgtcacc actttttatt ttagcactca ttataaaacc tcaacagcta
14341 atgacattat cttcataaaa gaaccagatg ataacttttt taccggacca aacacccgag
14401 tgccaagtgg ttcaccttga tcattaatca aaactacagc attgctagaa aaacgaatta
14461 cagaaccatc agattttcta atagggcttt tcaccctcac cacaactgct ctatatactt
14521 ttcccttctc aactttccct cttggagtaa tagatttagt agatacaata actgtgtcac
14581 ctatagatgc agattttcta ccacccaata aaccaataca aagcactgca cgcgcaccag
14641 aattatcagc tacttccaac aatgtatttt tttgaatcat ataaatatcc tattcaatgt
14701 gagctattaa caataaccca ttttttagta gtagaaatag gcttgtgttc ttgtattaaa
14761 accttgtccc cttctttaca actattattc tcatcatgtg ctgtatactt cttgtatttt
14821 tttataacct ttttatacag ctcatcctta tacacttgta ataccgaaac ctttacagtc
14881 ttatcacact tagccttagt tacagtacca caaaaaacct ttttaggcat tcttttcctc
14941 tcttcttctt ttatttaatg tagttaaaat acgagctatg ctctttctta ttaagctaaa
15001 acgcgaaata ttgttgcact ggcccagctt cttttgaaaa accaaattaa caaactcttt
15061 tctcaaattc acaagaattt catgcaattc ttgcgaggac ctcgattcaa tgtcagctat
15121 atccattgca actccattta attatgatta gatacaaatt tacacttcat aggaagctta
15181 gcagttgcct tttcaagcgc caatcttgct aaatgcatgg gaacatcact gctaatttca
15241 aacaacattc taccgggctt agctttaaat acccaaaact caacactacc tttcccttta
15301 cccatacgca catccgccgg ctttttacta acaggggtat caggaaaaat tcttatccac
15361 actttaccag agcgttttaa tgttctagat attacacgcc ttgcagtttc aatatgctta
15421 gattgaatcc tacctgcttc catagccttt agaccataat ctccaaaaga taatgtgcta
15481 ccacctttcg tgttaccctt aattcgtcct ttaaatactt ttttatattt actttttta
15541 ggaataaaca tttcaatata cctaattagt taccaatata aacccagacc ttaactccta
15601 tgatgccata tatagttttt gcttcagcaa aagcataatc tatattagca cgtaaagtat

15661 gcaaaggcaa acggccttct ttataccact cagtacgagc tatctcagct ccgccaaggc
15721 gcccagaaca acttacttta atacctctag cacccatcct caaacaactt tgaatagctt
15781 ttttcatcgc tcttctaaat gaaacccttt tttctaactg ctgtgcaatg ctgtttgata
15841 ttaaaactgc atctatttca gacctttaa cccctactac attcacttcc acattatttt
15901 ttacttttc agctatcttt tgctttatct tctcaatatc cgagcctttc ttacctatta
15961 tcactccagg tctagaagaa tgtattatta cagataccaa atcgatcgta cgctctataa
16021 ttactttaga aacaccagca tgcttaaaag attcatttat ataactgcga atagataaat
16081 cttgatgcaa cccttgttta taacccttct cagcatacca aatagatgac caagtattaa
16141 ttatttttaa cctaaatcct ttaggattaa cgtttcccat atttttgcac tttccttatt
16201 aattttctat ttttatgaat catataattt cccccaattt tacagttata ttactataac
16261 gtttactaac tctattagct ctacccatag cttttggaca taccctacgc aaagtaagag
16321 acttacctat taaaatttct tttatatata aattatcgat atccaatcca taattttgag
16381 cattagcaat cgcagaattt aataccttcc ttataagacc tgcagctttc ttttcacaaa
16441 atcttaattg cacagtggca aaagaaactt ttttattacg tactaaacca gcaaccaaat
16501 tcaatttacg aggagttgat tttaaaaccc tagaaccagc tttaactatt acatctctgt
16561 ttttcatatc aattaccttc ttgtcgcctt tttatcacca ctatgcccag taaatttacg
16621 agtgggtgaa aactcaccaa acttatgacc tatcatattc tgatcattaa cattcacagg
16681 aatgtaatcc ttaccattat aaacagcaaa ctttaaacct aaacaattag gaagaattac
16741 agaagccctg gaatgaactt ttatagccat gttaacaaac cccttgttta aagcattatt
16801 caccgacttt aatatagaag gatgacaaaa aggaggcttc catgcagatc tactcataaa
16861 ctaacctttc aattgtttta tatacttatc actagattta tttctccttc gagttttctt
16921 tcctttttgtt gcaacacccc aaggagtaac aggatgacga ccaccagagg tttttccttc
16981 cccgcctcca tgaggatggt caaccggatt cattgcaact ccacgtacag taggtctaat
17041 cccaagccac ctacttctcc cagctttacc caactttcta ttcttatggt cagggttaga
17101 tactacacca atagtagcct tgcaagaaga taaaatcaac ctaatttgac cagacctgag
17161 tcgtaataaa acatattggc catcacgacc aacgatttgt gcataacaac cagcagctct
17221 agcaatcgca gcaccattac ctggcttcag ctcaacatta tgaacgaaag aaccaacagg
17281 tatatgcttg agtagtaaac aattccctgg caaaatatca gcatcattcc cagctgtaac
17341 aacatcacct ggcttcatac cttgaggagc taatatataa gacttaatat catcttcctt
17401 atatgatatt aacgccaaaa acccacttct atttgggtca tactctattt tttcaactat
17461 accttgacca cttctattac gcttaaaatc tatcactcta tacttctttt tgtgaccacc
17521 accccctatga cgagttgtaa ttctaccatg attatttctt ccaccactgg acttttttacc
17581 ggatgtaaga gacttttctg gctcatcttt tgataaacca actttactta ctaacacagt
17641 cccacgagaa gacggagtaa caggattaaa aaatttcata cccatactaa acgctcatta
17701 tatctaattt ttgaccatcc atcaaagaaa aataaacttt ctttttctgt tttcacaac
17761 caatcacacc cctaaaacgc ctatatttag gcttaattct aacaacattt atagaagaaa
17821 ttttaacatc aaatagagac tctattgcca actttatttg acgcttattt acatttacaa
17881 aaacatacaa agaatattta ttaaacttct ccctcaaaaa agaggccttt tctgtaatta
17941 taggagattt tattatatta ttatatttga tcataacaat ctaccttcaa gatgctttaa
18001 agtatccttt gtcaacatca cgcattcgtg attcaatata tcaaaaacat ttaacccaat
18061 aggtttgatt aagtctacat agtgcaagtt tttagccgca cgcaacaaat catctccata
18121 atcaccaact atcaaaaagg aagaaaattt aaaattttta atacatttac acatttcaga
18181 cgttttcttc acatcaatat ttaaattatc cagaataatt acttgattat ttaaatactt
18241 tagagataaa gcaattttta aaccaaattt gcgtaccttc ttattaagag aataagtatg
18301 actcctcaca acagggccga aaataatccc accacctcta aattgaggag atcgcaagct
18361 cccttgtctt gccctaccgg tacgtttttg atcataaggt ttagctgttg taccagagac
18421 atcactaata ccttttgttt tatgagtacc agctcttctc ttcgctaatt gccatctcac
18481 tatatcatgc aaaatactca atttttgctt agcagaaaat atcaaaggat tgagctgagc
18541 agtacctaca ttatcattag atagattaac taaattacat tccataacta acttaccaaa
18601 ttactagcat catcatttac atctaacagt aaacctaccg gaaaaggaac atctttatgt
18661 aaagattttt taactgcatc tcttacaaaa acataggaat ttttaaaccc aggaacatta
18721 ttacccttca cagcaattat actattttca tgatcaatgg ataatatctt catattttgt
18781 gcagttatcc tgctgttacc taaatgacca gccattttct ttcctttgaa tactctacca
18841 ggatcctgac attggccagt ggaaccttgt gatctgtgag caatagaaac accatgagat
18901 gccctaagcc cactgaaatt atgccgcttc atcacaccag caaatccttt acctatagaa
18961 taacccgtaa tatcaagata ttgaccaacc acaaaatgat tgattcccac ttttttacca
19021 cattctattc ccgacaggtc atttaatcta ctttcgtata acttacattt attatttata
19081 ccctttttct ttaaatattc caactgaggt tttgctatat ttttaaaatc tcctgtgcct
19141 aaaatgactg aattatagcc acatttatct tgtcctttta tatcaataat ataagtttcg
19201 ctgagatgca ataaagttac agccatgcga ctattgtcag aatacatagc agtatgacca

19261 atattcatca ttagtaaacc aattctcctc agcgaattta ttctcttcat ttccttctcc
19321 taaaccttaa cttccttgac ttttaaatcc acttctacgc cagcagaaaa agataaacct
19381 gtaagcatct gcatcatagt aggagtaaga tcatgtaaaa caatcaaacg cctagaagtt
19441 ctcatttcaa actgttcacg agatttttta tcaacgtgag gagatctatt aacaataaat
19501 ttagaatctt tccttggcaa cgcaatcgga ccagataatt ttgcactgga ccgctttaat
19561 tcatcaacaa attttcgaac acactcctcc aataaagaac aatcgaaagc cttaatgtta
19621 atatatatat cttgcttcat cttagttact acactcactc caaaatttca gaaacaacac
19681 cagaaccaac agttctaccg ccttctctta tcgcaaaacg caatccctta tccattgcta
19741 tcggtacttg caattctact tctacactca cattatctcc tggcattacc atctccttcc
19801 catctagcaa ttttatgctc ccagttacat ccgttgtcct taaataaaac tgtggctggt
19861 aattcgcaaa aaatggtgta tgccttcctc cttcctcttt ctttaatata taaacctccg
19921 ccttaaactt tctatgcggt gttatcgtcc ccggtttcgc taatacttgc cctctctcca
19981 cttcttctct ttttgttcct cttaacaata ttcctacatt gagtcctgca cttcccttat
20041 ccagcaactt cttaaacatc tcaacacctg tgcatatcgt cttttgcgtc gctttcagac
20101 ctattatctc tatctcttct cccgtcttta tctctccctt ctctattctt cctgttacca
20161 ctgttcctcg ccccgatatc gaaaatacat cttcgattgg caataaaaat ggtaaatcca
20221 caggccttgg tggaaccgct acatattcat ctaacttttc catcaatttg tctattgatt
20281 tctttccata ttcgctgctg tcatcctcca gcgccttgag cgctgaacca accaccacag
20341 gcacttcatc ccctggaaat ccgtatttac tcagtaattc cctcacttcc atttctacta
20401 aatctatcat atcagtatca gcaacatcag ctttatttat atacaccacg atatatccaa
20461 cacctacctg cttcgccagc aatatatgct ctctcgtttg tggcattggc ccatcaaccc
20521 ccgacactac caatatcgcc gcatccatct gtgctgcacc tactatcatg ttctttacat
20581 agtcagcgtg tccaggacaa tcaacgtgtg cataatgcct tttttccgtc tgatactcaa
20641 catgcgctgt tgctatcgtt atccccctct tcctttcttc tggcgcttta tctatctgat
20701 cgtacgctac aaaatttcca taatgcttcg ttatcgccgc tgttaacgtc gtcttcccat
20761 gatccacatg tcctattgtt cccacattta catgcggctt tccaaatgct tccactattg
20821 ctgtcataat ttaaactttt ctacctaaaa tacaaataca tcaataaata atatgttgat
20881 tttataaaaa tacaacaaaa aaaagagcgg atagtgggaa tcgaacccac gtcactagct
20941 tggaaggcta gagctctacc attgagctat acccgcacaa tggaggaggt aggattcgaa
21001 cctacgtacg ctcacgcgga cagatttaca gtctgttacc tttaaccact cggtcactcc
21061 tccgcaaaaa tttgttaaga gaatactatt ttagcatctt aacttaagct cttattgtat
21121 ttaatactaa acgcctaaaa ttaaaaaatc aagcaataaa acctaccctg attaaaatca
21181 cgcagatttc attttataca acacaatatt gtcaataaaa cgtttaaaca aataatgcga
21241 atcatgcgta cctggcgctt cttctggatg gtattgcaca gaaaagactg ggtaatcctt
21301 catcattatt ccctctacac tattatcaaa tagagaaatg tgagtaattt caacattact
21361 tagaagagaa gctgaatcaa caacgaaacc atgattttgg ctagtgatct caacttttcc
21421 actaattaca tcataaactg gatggttact ccctcggtga cctatatcca tcttgatggt
21481 ctttgccccc aaagtaatcg caagtaattg atggcccatg catatgccaa aaattggtat
21541 tttagatttt ataataatgt ctatttctga aactacactt tctcctattt cttgcggatc
21601 accaggacca tttgaaagca ctatcccatc tggattcata ctcaacactt tttgagcaaa
21661 acctgtgctt ggtctgatta attcaactgt acaaccaagt tctattaagc gtgaaactat
21721 actgattttt acgccaaaat caacgattac aaccctatat tttgcattaa ggtcactttt
21781 aaaattacta tgcaagctga ctttattggt tatttctatc ccatttacag atttgtattc
21841 cttcaatttg tccaacgcat gcgccttact cgatgagcat atcattccat tttgagatcc
21901 atgttctctc aaatgtctcg ttaaagctct agtatcaact cctgatatcc caatcacgtt
21961 atttttctct aaccaatcat ttaaactgat atatgaagaa gggtgggaca tagaagaaag
22021 ttcacgcata accacaccac ttgcaaaaat tttctttcct tcattatcct tatggtttat
22081 tccaacgtta ccaatatgag gaaaagtgaa cgttataatt tgatcggcaa aagaaggatc
22141 agttatagta tgctgataac cggtcatacc agtggtaaaa cagacctcac ctatgcattt
22201 gcctttttta cctactgatt ttccccaaaa gcacttgcca tcttgtaaaa ttaaaactgc
22261 gtcctgcact tcattatcta tttaactgat tcaaaacagt atacaagaat ttcaaataat
22321 acgtaactct ttctaaaatt ccaaatttaa ccacgaagca gcaaaaaata acatgaaaaa
22381 cttttaatac aatctttcat ataaaatata tagttgacta ccttcatatt tcgtattata
22441 tataggtaaa tcataagtaa agtggagggt aataatgaac actggtacaa atcaatcaaa
22501 aaaaataaat gagaagtggt tagctggagg aattggcgcg ctattatttg taatgatgct
22561 accgtcgatt cttcttttag caaaaatagc tatagggcta attatggttt cagttgcagc
22621 aatagcgata aaagttgcct taaaaacaat atcgaatgct ttagaggata aacaggcaga
22681 tcaaagccaa gcgacgcaac aaacaacaca aagtaaaatt ataaacctgg ctattggatt
22741 aacatctata ttcgtaggag gattattact attcattata gcacaaactg gcttaattgc
22801 agcatgtgca gctataactg ctttagctct acacgaatat gttaaaaatc aagaaatagg

22861 aaaagagata aatgataaat tcgataaggt agctgacgat acaatcaatt ttattgttag
22921 cagtgttgaa aaacttattc caacaaagcc gccaggcata acagcctaaa ctgtctccgt
22981 tcagggcata agtgttacaa gtagcattat ataaagcgat taatggcatc ctcctgataa
23041 tagaataatt gtatctgttc aggggcatcg caaatattga agcaaaagtg gtcagtgctt
23101 actatacgaa tattgtgatt tgaatccact agggaggatg tcatcccagt gcccagacac
23161 tgggatccag gaattttgat tagagattaa attggtgagt ataaaagtag ctaatcctac
23221 gttaaaatac aacgtttta atgggactgc gtaaaagggc tggattccag tgtcaagcac
23281 tggaatgaca cctttgtgtt tgaggcaaaa cctgctataa catttcttcg caaacaaacg
23341 ttcgtacagt tatgtatcag ctactttcat ggcaccattt gttgtaaact cacatttctc
23401 tatgattatc tttatttgcc gctttcccga acggatgctc tacagttatt tcctcattaa
23461 tagagcgagg tgcaccggaa ccaggtacaa cgatattctc gtgcgattgc atatccttct
23521 tttcctcaca tggtgtttgc ttaatgaaac gtgctgctaa aacttcatgt aaagctttac
23581 cttttataag cacagcctcg ttttgcttag ctaactctaa aattttcttt ggttcaacat
23641 ctttaccgtc aatagttgat tttttaatac taacttgacc taaatttata tttagaacaa
23701 cagtacagtt aaggtctttt ccagattcat cttttgcacc ccaactaatt tcgatgtcac
23761 atattgcaaa ttttgtaaag gagtagttcc tctgttgttt ctcattcttc tcaattgtta
23821 ctattctttc accattgctg tatatttttg ctctttttac ctcgcttttc tcttcttcca
23881 ttcttttagt tttttcagtg aaaagacgac tggtttctaa actttgttca ggtttagtaa
23941 tgttgatatt aagatggtca taatgattaa gaagacggtt tgcatatgga catggcacat
24001 aacacccatc aaagaaagca attgcactac cagtttttag aaattcttgt ccatcgctca
24061 ttccttcacc aattcgaata agatatctac ccaacctctt tttaaaattt tcttttaact
24121 cctcctgatc tttagaagta agcttcttat ccaatatacc cattatttct tttcgcttac
24181 tcaataccac ataattgaaa aggaaaatat catcctctcg ataataattt gctatcttgt
24241 caaatggatc tgccggccaa ccgttattat aatttacaga atcagaagaa tcttcacact
24301 tattatcctt aaaatcaaaa ttcaatcttt caaaatctga taatactttt tctgccctat
24361 cttcaaaaga ccgaagtcca tcttgaagtt cttttccata actcattcct ttgtcaattt
24421 gaagaacata ttgaccatat ttctttctaa actcctcctg ttcttcgagg agaggaagat
24481 tcctatctaa tatatcgatt atttctttcc gcttattcaa tattacatag ttaaaaaggg
24541 cactctcccc cttcttttca taatcgtctg ctattttgtc aaaacactta ttatccttaa
24601 actccaattc ctcaaaatct gagagcacac tttgtacctg agtctcatcg aattgaagat
24661 ttgcttgagg agatagaaga gtcaactttt cttcactttc cttggtactc gaatttttga
24721 aaaaaccatt caatagattt acaatactag aaaacatttc acacctctat tgttataagt
24781 aaacttaatt atatcttaat gctaaatgtt gtaaagctaa aattagaaac taaaaaagct
24841 aacataagtt ctgcattgtt tgcagcttta tcaggttgtt aggtagaatt attaaagaac
24901 tagttgaaca tatgttatca actccatcca caagttctcc gcttgttact ccagttgcaa
24961 tgaacactga ttcacctctt gccatatctt ttacggcgta gactttttct gggtcatcaa
25021 tatttaaatt ttttgctctt tcttttaact gatctgtgtc aaatatcaat cttccctcca
25081 tctgcccacc gattgaactc agcgctgcag ccgcaagcac cccttctggt gcccctccta
25141 ttccgatata catatcatga ttgccattta ctagtgaaac tacggccaca atatcaccat
25201 catctattag tttaattttt gctcctaact tcctgatttt tactattaat tcattgtgcc
25261 tttcacgatt aagtatagtt accacgagat catttacttt gcattttttt gccttggcta
25321 aattgtctag attcttttca atgctatttt ttagtgagac tacaccctct ggaagatttt
25381 ttcctaccgc tattttttcc atataaacat caggtgcatg taaaaaatta cctttttttcg
25441 ttgcagcaag aacagacatt gcccctggtt tgtaatgagc acaaatcgta gtgccctcaa
25501 gtgggtcaac ggcaatgtcg atttcaggac catttccagt gccaaccttc tcgccaatat
25561 atagcatcgg tgcttcatct ctctcacctt caccaattac gatagtacca tttatttcca
25621 ttgaatttaa tactgtacgc attgcatcaa ctgcaacctg atcagccttt ttttcatcgc
25681 cgaagcctgc tagtttatat gcagcaagcg ccgcagcttc ggtcacttta actaatttat
25741 aggctaaatc ttccatcatt tgctttaaga actatattca atatgtatca tgatatatca
25801 tgctgcttga aactgcaata tgtcttgact gattattagg gagttgatat aatataagca
25861 ttatagtagt gggattatag ttatggcata tagtgatgat gatgttagaa atttctatct
25921 agatatagat agaattacag gacgtgataa cacagttact agtttggaag aactaaaaaa
25981 gaggttaagg aaagaagtta attttcaaga tgaatgtgaa ggaggcaatt cattagggaa
26041 cctacttcta aaacatgcaa ccgaaaataa caacccacgc gttaaggatt ttctcctcca
26101 aaatgggtgt gaacccccttc aacaagagca aataaccgca gcaataaaca aggaagaaga
26161 ggcacctaat gtggaagaga atagagcaga cagtaatgaa gtagattcta gtaacttcat
26221 aaaccctggt atgactgctg aagtacttta cgagcaacta atacaaggga aagaaacaaa
26281 tctggcaatt tttgactcgt ttgcaaatgc tgtaagagag tccaatatta ccggcgatcg
26341 tggcatattt ctagatgttg taaaattgtt tacagattta gatgctgtaa tccatcttac
26401 agatactgct tccagaacta tattgggcat agctacaata acaaaacaag ctgacgttgt

26461 taaggtactt ttggatagtg gcaaatttta tgaagaagaa aaatttaacg ctctgtgtct
26521 tgctattact caggataatg cccaagaagc tgagctaatt ttaggttacg tgagtcctgc
26581 aaacaggaaa taggctttac aagtggccat acaggtaggt aaaactgaaa ttgttaatgc
26641 gttcttgaat agaggtaagc ttaatgaaga aaatagagca gaacccagca gcagtaatgg
26701 aaacattggt agtagacctg tagcaacagt gcctccagtt agtactaatg gtgataatcg
26761 caatcacgac aatgaaatac ctgtagtaac agagactaac caaactacca cgccaaatag
26821 tgataatgtt aatggccaat caaatcataa taacaaaaat aaaacgcctg taataccaga
26881 ggctaagcaa actgccacac caaataatga taataaagct aacggctttg tagatgctca
26941 attttctgtg ccaaatgaaa aagaagctaa atataaaaaa agcaaggaaa atttttacac
27001 ctcattaaca aaagatgttg ttggagttgt tattacagga ttattcatta ctgctgctgt
27061 gatggttcca tttgtaactg gtgcagtgat ttgtggtgtt atagctgctt tggttgcaat
27121 agctaccggg ttgcatgtaa aaaattctac attaccaagc tatagagaaa tggaagaaaa
27181 taaagttgaa cacgtccaaa aaactgcaaa aacgttgtga ttcaatttcg ctagggagga
27241 tgttatgcca gtgcgtgaca ctggcatcta atttacccaa cacacttaga ttttatgcta
27301 aactgtgctg ccactaactt tgcaaccggt actctatagg gtgagcatga aacataatcc
27361 accccctgatt cgatgagaaa ctctatagat tttggatctg ctccatgctc tccacatata
27421 cccagtttta tttcttttcg ggtttttttg cctctttcaa tggccatctt gattaactcc
27481 cctaccccctt cgatgtccag cacttcaaat gggtcgtttt cgaatatgtt gctttcctta
27541 taagaatcga ggaaattaac tgaatcatct cttgaaagtc ccatggttgt ttgcgttaaa
27601 tcattagtgc caaaactaaa gaactctgca tgttttgcta acttatcagc aatcagtgct
27661 gctcgtggta gctctatcat tgttccaatt gagtacgcct tgtctgatgt cattccagcg
27721 cgtgacgctg gaatctgctt cttgatccta gctgagatga cagaggattc tttcttcact
27781 aactcgcata tcaaaacaaa ctctttctca ttcatgataa aggggaccat gatttcaggc
27841 tccacttcta tcttcttttc tttctttaat tcacttgcag cactaagtat tgccctaatc
27901 tgcatgctat atatttcagg atgagaaatg gcaagtctac aacctcgatg gccaagcatt
27961 gggttctttt ctgataactg tgctatttta ttttttactg attcaactga cttactaagt
28021 gatttggcga ttttttctat agtagactga ttattgggta aaaattcatg caaaggtgga
28081 tcaagcaacc gtatagtgac ctccctgccc tccataatag aaaatatttc tttgaaatca
28141 gacttttgca tttcttccag tttaatgagc gcatttgccc tttcattttc atcgtcagcc
28201 attatcaact tttgaatgaa ttcgattcta tcactagcga aaaacatatg ttctgtgcga
28261 cataagccta taccttctgc accgaattct tttgcaattt ttgcgtcttt tggagtatca
28321 gcgttcgctc tcactttgac cgttttgatt tcatctatcc agttgattat cgttttgaat
28381 tcttgcgata attcaggtga aattgtagga agaatgccaa gcataacctc ccctgttcct
28441 ccgttgatgg taattggttc acctttattt actttgtat cccccacaga aaagaaagtt
28501 ccatctttat cgatataaag tccacttaca ctgcaaatgc atggcttacc cattccacgg
28561 gttacaacag cagcgtgcga ggtcatccct ccccgtgctg ttactatgcc acttgcagca
28621 ttcattccat taatatcttc aggactcgtt tctgacctta ctaaaatcac tttttttaccc
28681 tgctctgcag ctttttcagc atcacttgca ctaaacacta catatccaga agcaacccct
28741 ggagaagccg gcagtccctt ccctattact ttttggtcac tcttaacgtc aagaactgga
28801 tgcaataaat tgtcaaaagt tttggatca attctcaata ttccttcttc ttttgtaatc
28861 gttccttcgt ttaccatatc aactattatg cgaatagcag cttcagccgt gcgcttgcca
28921 gacctagtct gcaaaatcca taatttaccg tcctgtacag taaattcgat atcctgcatg
28981 tctttataat gcctttcaag tttttcacat accgcgcata attccaggta gacacttggc
29041 agcaacttct ccatggtgtt tttttgctct ccgtcaattg gcataggagt ataaacacca
29101 gaaaccacat cctcaccctg agcattaacc aaaaactcac caaaaagctt tttttcccca
29161 gttgaaggat ttcgtgtaaa tatcacacca gttgcagaat tatcatttaa attaccaaaa
29221 accattgctt gcacgttgac cgctgttcca aggttttcag gaatattatg tatttttcta
29281 taggaaacag ccctatcatt tttccaagaa gcaaatactg cattaactga gtttaacaat
29341 tgctcttcaa cgttctgcgg gaaatgtttc tcagtttttt catatactat ctttttaaaa
29401 tcgttaacaa ttctctttaa aacatcaaca tcaagatcag ctaagctttt tgctccactc
29461 ttttgctgct cattatcaat aacatcttga aataggtgat gatcaagctg tagtacaaca
29521 ttggagtaca tcatgatgaa acggcagtag ctatcgtaag caaaacgttc gccacttttt
29581 tttgcaagcc caacaacggt ttcatcattt agaccaacat ttaaaatagt atcaagcatg
29641 cccggcattg aactaacact accagagcgt atggaaatta ataagggatt atttaaatcc
29701 ccaaatttac aaccgatgtc attttcgagc atcgtcatgt agtttttaat tccactacat
29761 atgtcattcc agtgcgtgac cacatttttt tctggatccg agtaatcaga tacttggatg
29821 acagaagatc tattgtcttg ataatagact ttgcaagcag aggtggaaat tgtgaaacca
29881 ggtggaacag gaatgccaac attgcacatt tctgctaaat ttgccccctt tcctcccagc
29941 agatttttca tttctgcatt gccttcgcat ttaccctggc taaagtaatg tattaacttt
30001 tcccccatca ttttctccgg attttcactg ataaacacat tgtacatata gttccaataa

30061 acctgtgaga acttgaaacc ttaggataat aaattttaca aaattttgca acaacgttcc
30121 ctctggcaac atcagtaaag ttaacaaaaa agatgacaaa ttattgaatt attatcaata
30181 agaatatata atataaagat acatttaaaa atttttttat ggctctttct aagtttctcg
30241 accccaagaa tgatgttgcg tttcggcgta tttttggtac tgaaaaaaat aaagacatcc
30301 tcattcactt tctcaatgat attttggggt tcactggcaa agatgaaata aaagaaatag
30361 aattcctcag cactattcaa gatgctgaaa ttgcctctaa aaaacaaagt atcgttgatg
30421 ttctttgcag ggatgaaaat ggggtgcaag taatagtcga aatgcaagtt gccaaaacta
30481 aaggctttga aaaacgcgct caatattacg ctgctaaagc ttattcaagg caagctgata
30541 aaggtgatca atatcagggt cttaaagaaa tcatttttat agctattgct gattgtattc
30601 tctttcctaa taaatctgag tataagtcag atcatgtaat gttagacaaa gatagctatg
30661 aacatgattt aaaagatttt tattttacat ttattgagtt accaaaattt cctaaaacta
30721 aagaagacca attggagagt atagttgaaa aatggatcta ttattttaaa tatgcagatg
30781 aaactagcga agaagaattg gaaaagataa taggtagtga tgtaataatt aaaaaggcat
30841 atgaagaact aaatagattc aactggtcag agaaagaatt tattgcctac gaacaggaaa
30901 taaaacgtat tcgtgatgaa cgggctgttc ttgaacaaaa acttgatgat gctaaaaagg
30961 aaggaaaaat tgaagttgca aaaacaatgc tagctaataa tgttgatgtc accactattg
31021 ttaagtttac tggcctctcc atgagtgaga tcaaagaatt gcagaattaa gtggacccgc
31081 aaacggttac acgaaataat gctgcagcct gaccagatga taagtacatt tagcctattt
31141 atctttatac tttcatgtca attttataaa tcaaatttat ttctgtgagt agctacaatt
31201 taaagacctt gcttcatcaa tgtaatggaa caaaaccttt ttagtaagtt caaaaccctc
31261 taaagtctct ccttcgcatc ttgctgtaat gcagttttgc gtatttgata ccctaagaag
31321 ccaccaacct ttattatcgg ttaccttaac accatcaaga tctgaaaata caatattttg
31381 ctgctccagt gttttcttta ttgactcaat tatttgaaac tttttctcat ctttcactac
31441 aatcttcact tcatgagtga tatataactt cggtaaatcc tcaatcacct gagataggct
31501 ttggtttttc ttaagcaaaa tgtcaactgc tttaacagca gaatatagcc catcatcaaa
31561 acttagctca gaaaagaaga agtgcccgct taattcacca gcaaactttg cctcttcctc
31621 taccatcttc ttcttaacta gtgaatgtcc agtagcacag gtgataactt gccctcctaa
31681 cttgctaaca aaatcatgca ctttcatact catttttacg ttagcaataa ctttgctttc
31741 tggatattcc tccaacactt cacgtgcaaa aattataaat aaatggtcat tggaaacaac
31801 gttgccttta ttatcaatta agcgcaccct atcgctatcg ccatctagcg caatgccaag
31861 atcacatcca tattccttca caatgtcaat taattgagca agattttttt cctctatggg
31921 atctggatca tgcagtggaa atgccccatc tatagagtta ttggttacga tgtgcgtatg
31981 accaggcaag atcttttcaa tataccttat aattccactt gctggactat ttccacaatc
32041 ccaggctatt tttaatttct gtgtagtatt attcttcact gcacttttca atatgctaat
32101 gtactcacta tatatattta tgttaattaa gcttccaatt cttgtgctgt ttttaattgg
32161 actgcttata atttccttca tttcttgatc tgagaagact tttttattac taaaaaattt
32221 aaagccatta tattcgctgg ggttatgaga ggcagttatt atgataccaa gatccgcctg
32281 tgtgatttgt gttgcagcgt aaagcatagg tgaagagcat agtccaacgc gtataacatt
32341 tgcaccggat aaagttaagc ccctaattaa ttctctttct atacctggtg aatctattct
32401 gctgtcgtag ccgacacaaa cactagctac ggtttggcca aactttctac ctatctcgta
32461 cccatcatta atctgcaaat ctttgcctac tatacctcta atatcgtatt ttcttataat
32521 ggtattgtcc atactgtact tcttgaagtg actactatat aacattttgg ccaaatgctc
32581 aagtttattt atccaacaat tagttgactc tttaaagaaa tctgttaata ttatagtata
32641 ttttttaaaa tgtaaaatta tgccacagga aaacgaaaat gtatttatag ctgaaagtca
32701 aagtttagat gtagatgaac agcagaaaaa ggatatttta gatgtgattg aatcttgtag
32761 cagctttcaa gagactataa aacaattgga ggaaaaaaat ttaatctggt attttattgg
32821 ccctttacgc aaagatgagg acattacagg acagataaat ttaaagtggg aaaaggagtt
32881 ccaaaaattt gagcaatcaa taaatgaaac acatagagaa tttgaagcat ctttaccaaa
32941 tcaaaatttc ttagcgtatt caacaaaaga tgatgacttc aaaattagat ttgaaaatga
33001 agaaaaacca atagaaatta gcaatatttt aagagctagc agtgaaggta aggtttataa
33061 catatcttta gggaaagata gtcaaataac tgcaaccaaa aaaggcagcg aaaggcacta
33121 tgattttact ggttcaagct catgtgaaat gaccattaat tggcaggcta aggattctga
33181 aggtaacagc atagactgta gcatgaccgt aaaattggat tctggtggta tacagagtgt
33241 tattggtgag ccaaaatttg gaggaataac gagttcagaa gaagtattaa agttagttgg
33301 acaaaataaa gaagttttta tcaacagaaa aactctgtat caagctttta ctgatatagg
33361 tcaagatatg gacagtctcg agcccacaca agatctatca tcgcaattgc ctacaccaag
33421 cagcagtggg tattcctctc ctagcacacc ttcttcacgt agaagttcta tatccaccag
33481 cagtgaattt agtgatgaag atgaaatatt tgaggatgat atgtctggtg atgaagaaga
33541 gtatgagcaa gaaggattaa aagataaagt agatgaaatt ataaacgaaa ttgaggaatt
33601 aaaggaagga aaagaaacat ttgctcaaca ttttgcagcg caggcaaagc agcttgagga

```
    33661 tctaagatta gaacgtgact ccttgagaga taaagtcaat gaacaacagc gagcgatatt
    33721 tgaaagaggt caaagaataa aggaacaaga ggaagaacta caggataaaa atgatgtaat
    33781 aaagaactta aataatcagt tgaaagataa ggaaggtgaa acaaatgagg caaataaaga
    33841 attacagaga aaagtgaagc taatttaact gtactaaagg aaaagaatgc aaatttggag
    33901 aatgacctga agaaaatagt ggaaggttta aaggatgatc ataaaaagca gttggcagaa
    33961 cagttaatgg aaacaaaaaa ggaaaacaat acattggctg aaacggcgag ccaattgtct
    34021 gaagaattaa atgaattaca gagagaaaag gaagacttag agagggaaat tcgacaactt
    34081 gaacctggaa gatcacttgc agatgagcta agcgaagaaa atacgaaggg ggaaatagaa
    34141 aagcttcaat atcaacttaa ggaaaaggaa gggatgttaa cgtgtacaca ggaagagctg
    34201 gcatctgtca aagagagtct agagcaactg cagaacatat tacagcagga aaaaggtcag
    34261 gcaactcaaa ctgaagttga atatgaaagt agaggaacaa gcatgcagca gcaagaccaa
    34321 ggtatacaag ctgtaaacaa caactcaagc agaacagcta gtgggttaag acaagaaaac
    34381 aaaaaattga ctagagacaa actaactata tctgaacaat taaaacaaaa aataagagag
    34441 attgacgagt taaagaaaaa tttaaagcaa ctgcaagaag aaaataaaca actcaaacaa
    34501 gaagttacta aaaatgcagg agttgatgca tcatcttcaa caggtcaaaa agaagctaaa
    34561 gaagcagaag ttataccgca aaacattatg gattggcctg ttccaattcc agatgataaa
    34621 ccggaagagc agaaaactcc attagagcca atcaaggtaa atacagaatt atcagatcca
    34681 aaatcaatcc ctgctgaggg tccggaaaaa tctcctcctc tttcaaaatg atgagttttg
    34741 cttctatagc tgacccaagc aacacgtcat cccgcgattt attcgcgtta tctcagccgc
    34801 taacacgtag cgggatactt aaccgtcata ccgtgattta ttcacagtat ctcttagcca
    34861 ctaacaagca gcgggatgac agcaatccta cgtcatgccg ccgcggtacc tctagatccc
    34921 gctaactagt agcggaatga cgaatttttg tttttcaaat tatagctaaa catactgtaa
    34981 ttgctattga atgatggaga gacatactcc attattttag cacgaagtaa caaattcaat
    35041 gaattggttc ttcaaaaata atttaaactt ttacagtagg tcatgaaata ttatagcgat
    35101 ctcattaaaa gaaactttta taagtatcgt tgatgagaaa ggaaaagttg ttaaggaaga
    35161 agttgttgca agcgaaagta aggcaatagc agattagcca aggcaaaaaa tacgaaccca
    35221 taggaataga aagtggatag ttgtcaatat caatgtgcaa agagttaagg agttttggat
    35281 taccagcaat ttgtgtagat gcaaggcata tggcagcagc gttgtctgca agaatcaata
    35341 agaatgataa aaacgatgcg aggggtatag cgcagatgat gagaagtgtt agtaaaatca
    35401 gttgtcaaat taaaatagcg cttggaagca gaagacagct aatgtgtagc aaacagcagg
    35461 ttattggaac aataagaggg ttgttgaaga tacatggtcg gtaaaggatc aaaatttgaa
    35521 actttcgctt taagaataca agaggcaata aataatttgg atgaaatcag taggagttca
    35581 attgaagcgt tagtttgtag tttggaagca atagaaaagt cattaagaaa gcttgacaaa
    35641 atgctttcag aacaaggtga aaaagacgat gattgtaaat tgttaactac tgttccagga
    35701 gtggggatta tagtagcaat gacgtataaa gctgcaattg ataacccaca taggtttgag
    35761 aaatctgata cagttggagc atatataggg t
//
LOCUS       JAQZAU010000068           60654 bp    dna     linear   ENV 17-MAR-2023
DEFINITION  MAG: Wolbachia pipientis isolate wEsol contig_39992, whole genome
            shotgun sequence.
ACCESSION   JAQZAU010000068  JAQZAU010000000
VERSION     JAQZAU010000068.1
DBLINK      BioProject: PRJNA929258
            BioSample: SAMN33142973
KEYWORDS    WGS; ENV; Metagenome Assembled Genome; MAG.
SOURCE      Wolbachia pipientis (insect metagenome)
  ORGANISM  Wolbachia pipientis
            Bacteria; Pseudomonadota; Alphaproteobacteria; Rickettsiales;
            Anaplasmataceae; Wolbachieae; Wolbachia.
REFERENCE   1  (bases 1 to 60654)
  AUTHORS   Fiutek,N., Couger,B.M., Roy,S., de la Torre,J.R. and Connor,E.F.
  TITLE     Genomic assessment of the contribution of the Wolbachia
            endosymbiont of Eurosta solidaginis to gall-induction
  JOURNAL   International Journal Molecular Sciences
REFERENCE   2  (bases 1 to 60654)
  AUTHORS   Fiutek,N., Couger,B.M., Roy,S., de la Torre,J.R. and Connor,E.F.
  TITLE     Direct Submission
  JOURNAL   Submitted (10-FEB-2023) Biology, San Francisco State University,
            1600 Holloway Ave, San Francisco, CA 94132, USA
COMMENT     ##Genome-Assembly-Data-START##
```

```
          Assembly Date          :: JUN-2019
          Assembly Method        :: FLYE v. 06-2019
          Genome Representation  :: Full
          Expected Final Version :: Yes
          Genome Coverage        :: 100.0x
          Sequencing Technology  :: Illumina HiSeq; PacBio Sequel
          ##Genome-Assembly-Data-END##
FEATURES             Location/Qualifiers
     source          1..60654
                     /organism="Wolbachia pipientis"
                     /mol_type="genomic DNA"
                     /submitter_seqid="contig_39992"
                     /isolate="wEsol"
                     /isolation_source="galls on Solidago altissima"
                     /host="Eurosta solidaginis MN-2018-EFC"
                     /db_xref="taxon:955"
                     /environmental_sample
                     /country="USA: Cowling Arboretum, Carleton College,
                     Northfield, MN"
                     /lat_lon="44.48 N 93.13 W"
                     /altitude="280 m"
                     /collection_date="2018-11"
                     /metagenome_source="insect metagenome"
                     /note="metagenomic"
     gene            <1..1034
                     /locus_tag="wEsol_00787"
     CDS             <1..1034
                     /locus_tag="wEsol_00787"
                     /codon_start=1
                     /transl_table=11
                     /fullproduct="qcoverage 100 , hcoverage 85.9649122807018
                     , similarity 100 , identity 100 , evalue 9.89e-244 ,
                     alnlength 343 , Bacteria , Proteobacteria ,
                     WP_209452298.1 phage major capsid protein [Wolbachia
                     endosymbiont of Ceratosolen solmsi]"
                     /cog="COG4653 COG4653 422 Predicted phage phi-C31 gp36
                     major capsid-like protein"
                     /pfam="Phage_capsid"
                     /tigr="TIGR01554 major_cap_HK97 385 phage major capsid
                     protein, HK97 family"
                     /product="phage major capsid protein"
                     /translation="ERLDLIETAAQRPEVNTDFSTSDKYFSDYIRKGMESGLSHKTLS
                     GDDSDIGGYLVTPHIVKRINKRVTDSSPMRQICSSQRISTETLDYIIEDFDRAGAGWS
                     SETVDDENGGNKSKYDFAKDTDTPKIQKISVTTYELYAQPQISQKLLDDAFVDVESWL
                     VEKIAETFSKEESEAFIKGEGTFQPKGILAYENGNSYNKIEQVKTEKLDSDSIMMLYY
                     SLDEYYSKNASFLINRSTLKDIRLLKSQTGQYLWQPSLSLEAPDTLMGIPVYQSADMP
                     PAPNNQLPVIAMADFKQAYKIVDNRGMRILRDSYTNKPYVRFFVTKRVGGEVVNTSAI
                     KLLKVASKY"
                     /besthit="qcoverage 100 , hcoverage 85.9649122807018 ,
                     similarity 100 , identity 100 , evalue 9.89e-244 ,
                     alnlength 343 , Bacteria , Proteobacteria ,
                     WP_209452298.1 phage major capsid protein [Wolbachia
                     endosymbiont of Ceratosolen solmsi]"
     gene            complement(1425..2066)
                     /locus_tag="wEsol_00788"
     CDS             complement(1425..2066)
                     /locus_tag="wEsol_00788"
                     /codon_start=1
                     /transl_table=11
                     /fullproduct="qcoverage 100 , hcoverage 100 , similarity
```

```
                     100 , identity 100 , evalue 2.07e-146 , alnlength 213 ,
                     Bacteria , Proteobacteria , WP_010962606.1 MULTISPECIES:
                     ATP-binding cassette domain-containing protein
                     [Wolbachia]"
                     /cog="GlnQ COG1126 240 ABC-type polar amino acid
                     transport system, ATPase component"
                     /pfam="DUF258 AAA_23 AAA_22 AAA_29 AAA_16 AAA_17
                     Pfam-B_16450 AAA_18 AAA_21 AAA_10 ABC_tran SMC_N
                     Pfam-B_3333 AAA_21 Pfam-B_3856 Pfam-B_15070"
                     /product="ATP-binding
                     cassette domain-containing protein"
                     /translation="MIKMHNVCKLFNNVPVLNNINLIIEQSIVGLAGPSGSGKSTLLR
                     CIQKLEVLDSGSIECNDSTGFVFQDFQLFSHMTVWENLTYAPKLKNKTQDQSDSLLES
                     LGIASKKNDYPSNLSGGQKQRVALARSLMMNPRILLCDEPTSGLDVATIVDVVELLQS
                     VRDMGITMIIASHNLDFLTKISDRIILLKNGKIIVDINPKEIDEPTNYLKNNY"
                     /besthit="qcoverage 100 , hcoverage 100 , similarity 100
                     , identity 100 , evalue 2.07e-146 , alnlength 213 ,
                     Bacteria , Proteobacteria , WP_010962606.1 MULTISPECIES:
                     ATP-binding cassette domain-containing protein
                     [Wolbachia]"
     gene            complement(2050..2697)
                     /locus_tag="wEsol_00789"
     CDS             complement(2050..2697)
                     /locus_tag="wEsol_00789"
                     /codon_start=1
                     /transl_table=11
                     /fullproduct="qcoverage 100 , hcoverage 100 , similarity
                     100 , identity 100 , evalue 1.50e-140 , alnlength 215 ,
                     Bacteria , Proteobacteria , WP_007548901.1 MULTISPECIES:
                     amino acid ABC transporter permease [Wolbachia]"
                     /cog="HisM COG0765 222 ABC-type amino acid transport
                     system, permease component"
                     /pfam="BPD_transp_1"
                     /tigr="TIGR03003 ectoine_ehuD 212 ectoine/hydroxyectoine
                     ABC transporter, permease protein EhuD"
                     /product="amino acid ABC
                     transporter permease"
                     /translation="MQELISNFLFVGTGLAFTLKLLIGGLLLGMSLGGLFSVLRYNKI
                     CIPLINGIISVIRGTPLILQLSVVYFMTPTIAGIKLNIFSAGIIAFGLNSCAYIAEIL
                     RAGIETISKNQFEAAKTLQIPSFYMWRDIILPQVIKNILPAMINEIIALLKETALIST
                     IGGVDIMRSAQMLAAEQFTYFIPLCLAGAYYYALVLLIEYIGKKIEQKGIYDKNA"
                     /besthit="qcoverage 100 , hcoverage 100 , similarity 100
                     , identity 100 , evalue 1.50e-140 , alnlength 215 ,
                     Bacteria , Proteobacteria , WP_007548901.1 MULTISPECIES:
                     amino acid ABC transporter permease [Wolbachia]"
     gene            complement(2697..3431)
                     /locus_tag="wEsol_00790"
     CDS             complement(2697..3431)
                     /locus_tag="wEsol_00790"
                     /codon_start=1
                     /transl_table=11
                     /fullproduct="qcoverage 100 , hcoverage 100 , similarity
                     99.6 , identity 99.6 , evalue 2.59e-169 , alnlength 244 ,
                     Bacteria , Proteobacteria , WP_015588976.1 MULTISPECIES:
                     ABC transporter substrate-binding protein [unclassified
                     Wolbachia]"
                     /cog="HisJ COG0834 275 ABC-type amino acid
                     transport/signal transduction systems, periplasmic
                     component/domain"
                     /pfam="SBP_bac_3"
```

```
                     /tigr="TIGR01096 3A0103s03R 250
                     lysine-arginine-ornithine-binding periplasmic protein"
                     /product="ABC transporter
                     substrate-binding protein"
                     /translation="MKKYILNAIIVISILLTGCKENKHDNIIRFAISADYPPFEYYEN
                     GKFTGFDIELAQLVAKELGKEAIFKDMQFSTIFAALQSNSVDAAISTIGSTEEREKNF
                     DFSNSYYTGEFGILYHKNKPIAGINNLTRKKIAVQLGTVMEIWLKEHVPTTAQIIAID
                     NNNQAVETLKSGHVDGVLIDEFQGKVFSKKNPGLSCSATAKLDGGYVIAVNKGSALKD
                     KINEALKSLESKGVIQKIAEKWLGDN"
                     /besthit="qcoverage 100 , hcoverage 100 , similarity 99.6
                     , identity 99.6 , evalue 2.59e-169 , alnlength 244 ,
                     Bacteria , Proteobacteria , WP_015588976.1 MULTISPECIES:
                     ABC transporter substrate-binding protein [unclassified
                     Wolbachia]"
     gene            3557..4012
                     /locus_tag="wEsol_00791"
     CDS             3557..4012
                     /locus_tag="wEsol_00791"
                     /codon_start=1
                     /transl_table=11
```
[truncated: 2,853,046 more chars]
