# Supplementary material for: Genomic Assessment of the Contribution of the Wolbachia Endosymbiont of Eurosta solidaginis to Gall Induction
Source: Int J Mol Sci. 2023 Jun 1;24(11):9613. doi: 10.3390/ijms24119613 (PMC10253641; doi:10.3390/ijms24119613)
Supplement: Supplementary file 1 [file ijms-24-09613-s001.zip › Supplemental Table S5 - Cytokinin and Adenine Salvage Enzymes.pdf]

Supplemental Table S5. KEGG EC and KO numbers for potential cytokinin biosynthesis and adenine salvage enzymes

Enzyme Function	KEGG EC Number	KEGG KO Number	Enzyme	Abbreviation
**cytokinin biosynthesis**	EC: 2.5.1.27, EC:2.5.1.112	K10760	adenylate dimethylallyltransferase	IPT
***t*RNA modifying**	EC: 2.5.1.75	K00791	tRNA dimethylallyltranferase	TRIT1, miaA
	EC: 2.8.4.3	K06168	tRNA (N6-isopentenyl adenosine(37)-C2)-methylthiotransferase	CDK5RAP1, miaB
	EC:1.14.99.69	K06169	tRNA 2-(methylsulfanyl)-N6-isopentenyladenosine37 hydroxylase	miaE
**Adenine Salvage**	EC: 3.1.3.5	K01081, K19970, K03787, K11751, K08693	5'- nucleotidase	NT5E, ushA, surE, yfkN
	EC: 3.2.2.1	K01239	purine nucleosidase	
	EC: 3.2.2.-	K22522	cytokinin riboside 5'-monophosphate phosphoribohydrolase	LOG
	EC:2.4.2.1	K03783, K03784, K09913	purine-nucleoside phosphorylase	punA
	EC:2.7.1.20	K00856	adenosine kinase	ADK
	EC:2.4.2.7	K00759	adenine phosphoribosyltransferase	APRT
	EC:3.2.2.4	K01241, K06966	AMP nucleosidase	amn
**formation of O- and N-glucosides of cytokinins**	EC:2.4.1.203	K13492	zeatin O-glucosyltransferase	ZOG1
	EC:2.4.1.215	K13495	cis-zeatin O-glucosyltransferase	CISZOG
	EC:2.4.1.-	K13496, K23452	UDP-glucosyltransferase 73C & 85A	UGT73C UGT85A
	EC:2.4.2.40	K13494	zeatin O-xylosyltransferase	ZOX1
	EC:2.4.1.-	K13493	cytokinin-N-glucosyltransferase	UGT76C1_2
**inactivate CKs**	EC:1.5.99.12	K00279-7	cytokinin dehydrogenase/oxidase	CKX
