# Supplementary material for: Genomic Assessment of the Contribution of the Wolbachia Endosymbiont of Eurosta solidaginis to Gall Induction
Source: Int J Mol Sci. 2023 Jun 1;24(11):9613. doi: 10.3390/ijms24119613 (PMC10253641; doi:10.3390/ijms24119613)
Supplement: Supplementary file 1 [file ijms-24-09613-s001.zip › Supplemental Table S6 - IAA Synthesis Enzymes.pdf]

Supplemental Table S6. KEGG EC and KO numbers for IAA synthesis enzymes

KEGG EC Number	KEGG KO Number	Enzyme	Abbreviation
EC:1.13.12.3	K00466	tryptophan 2-monoxygenase	iaaH
EC: 3.5.1.-, EC:3.5.1.4	K21801, K01426	indoleacetamide hydrolase , amidase	iaaM, amiE
EC:2.6.1.27, EC:2.6.1.99, EC: 1.4.3.2	K14265, K00838, K16903, K03334	tryptophan aminotransferase, aromatic amino acid amino transferase, L-tryptophan pyruvate aminotransferase, L-amino acid oxidase	Tam1, ARO8, TAA1, IL4I1
EC:1.14.13.168	K11816	indole-3-pyruvate monooxygenase	YUCCA
EC:4.1.1.74	K04103	indolepyruvate decarboxylase	idpC
EC:1.2.1.3, EC:1.2.3.7	K00128, K14085, K00149, K11817, K22417	aldehyde dehydrogenase (NAD+), indole-3-acetaldehyde oxidase	ALDH, AA01_2, AA04
EC:1.14.14.156	K11812, K11813	tryptophan N-monooxygenase	CYP79B1_2, CYP79B3
EC:4.8.1.3	K11868	indoleacetaloxime dehydratase	CYP71A13
EC:3.5.5.1	K10501	nitrilase	
EC4.1.1.28, EC:4.1.1.105	K01593	aromatic L-amino acid / L-tryptophan decarboxylase	DDC, TDC
EC:1.4.3.4, EC:1.4.3.22	K00274, K11182	monoamine oxidase, diamine oxidase	MAO, AOC1
EC:4.2.1.84	K10721, K20807	nitrile hydratase subunit alpha, subunit beta	nthA, nthB
EC:1.14.14.45	K11818	aromatic aldoxime N-monooxygenase	CYP83B1
EC:4.4.1.-	K11819	S-alkyl-thiohydroximate lyase	SUR1
EC:2.4.1.195	K11820	N-hydroxythioamide S-beta-glucosyltransferase	UTGZ4B1
EC:2.8.2.24	K11821	aromatic desulfoglucosinolate sulfotransferase	ST5A, SOT16
EC:3.2.1.147	K10237	myrosinase	
