# Supplementary material for: Genomic Assessment of the Contribution of the Wolbachia Endosymbiont of Eurosta solidaginis to Gall Induction
Source: Int J Mol Sci. 2023 Jun 1;24(11):9613. doi: 10.3390/ijms24119613 (PMC10253641; doi:10.3390/ijms24119613)
Supplement: Supplementary file 1 [file ijms-24-09613-s001.zip › Supplemental Table S7 - MEP Enzymes.pdf]

Supplemental Table S7. KEGG EC and KO number for MEP pathway for DMAPP and HMDBP synthesis enzymes

**KEGG EC Number**	**KEGG KO Number**	**Enzyme**	**Abbreviation**
EC:2.2.1.7	K01662	1-deoxy-D-xylulose-5-phosphate synthase	dxs
EC: 1.1.1.267	K00099	1-deoxy-D-xylulose-5-phosphate reductoisomerase	dxr
EC:2.7.7.60	K00991, K12506	2-C-methyl-D-erythritol 4-phosphate cytidylyltransferase<br>2-C-methyl-D-erythritol 4-phosphate cytidylyltransferase /<br>2-C-methyl-D-erythritol 2,4-cyclodiphosphate synthase	ispD, ispDF
EC:2.7.1.148	K00919	4-diphosphocytidyl-2-C-methyl-D-erythritol kinase	ispE
EC:4.6.1.12, EC: 2.7.7.60	K01770, K12506	2-C-methyl-D-erythritol 2,4-cyclodiphosphate synthase<br>2-C-methyl-D-erythritol 4-phosphate cytidylyltransferase /<br>2-C-methyl-D-erythritol 2,4-cyclodiphosphate synthase	ispF, ispDF
EC:1.17.7.1, EC: 1.17.7.3	K03526	(E)-4-hydroxy-3-methylbut-2-enyl-diphosphate synthase	ispG, gcpE
EC:1.17.7.4	K03527	4-hydroxy-3-methylbut-2-en-1-yl diphosphate reductase	ispH, lytB
