# Supplementary material for: Genomic Assessment of the Contribution of the Wolbachia Endosymbiont of Eurosta solidaginis to Gall Induction
Source: Int J Mol Sci. 2023 Jun 1;24(11):9613. doi: 10.3390/ijms24119613 (PMC10253641; doi:10.3390/ijms24119613)
Supplement: Supplementary file 1 [file ijms-24-09613-s001.zip › Supplemental Table S8 - SAM Enzymes.pdf]

Supplemental Table S8. KEGG EC and KO numbers enzymes for S-adenosyl L-methionine synthesis

KEGG EC Number	KEGG KO Number	Enzyme	Abbreviation
EC: 2.7.2.4, EC: 1.1.1.3	K00928, K12524, K12525, K12526	aspartate kinase,<br>bifunctional aspartokinase / homoserine dehydrogenase 1,<br>bifunctional aspartokinase / homoserine dehydrogenase 2,<br>bifunctional diaminopimelate decarboxylase / aspartate kinase	lysC, metL, lysAC
EC:1.2.1.11	K00133	aspartate-semialdehyde dehydrogenase	asd
EC: 2.3.1.31, EC: 2.3.1.46	K00641, K00651	homoserine O-acetyltransferase/O-succinyltransferase,<br>homoserine O-succinyltransferase/O-acetyltransferase	metX, metA
EC: 2.5.1.48	K01739	cystathionine gamma-synthase	metB
EC: 2.5.1.-	K10764	O-succinylhomoserine sulfhydrylase	metZ
EC: 2.5.1.49	K01770, K12506	O-acetylhomoserine (thiol)-lyase	metY
EC: 2.8.1.16	K23975, K23976	L-aspartate semialdehyde sulfurtransferase,<br>L-aspartate semialdehyde sulfurtransferase ferredoxin	
EC: 2.6.1.7, EC: 4.4.1.13	K00816,01760,K14155	kynurenine---oxoglutarate transaminase / cysteine-S-conjugate beta-lyase / glutamine---phenylpyruvate transaminase,<br>cysteine-S-conjugate beta-lyase	CCBL, metC, patB
EC: 2.1.1.5	K00544	betaine-homocysteine S-methyltransferase	BHMT
EC: 2.1.1.10	K00547	homocysteine S-methyltransferase	mmuM, BHMT2
EC: 2.1.1.13, EC: 1.5.1.54	K00548, K24042	5-methyltetrahydrofolate--homocysteine methyltransferase,<br>methionine synthase / methylenetetrahydrofolate reductase (NADH)	metH, yitJ
EC: 2.1.1.14	K00549	5-methyltetrahydropteroyltriglutamate--homocysteine methyltransferase	metE
EC: 2.5.1.6	K00789	S-adenosylmethionine synthetase	metK
